# Genome-Wide Identification and Characterization of Long Noncoding RNAs Involved in Chinese Wheat Mosaic Virus Infection of *Nicotiana benthamiana*

**DOI:** 10.3390/biology10030232

**Published:** 2021-03-17

**Authors:** Weiran Zheng, Haichao Hu, Qisen Lu, Peng Jin, Linna Cai, Cailin Hu, Jian Yang, Liangying Dai, Jianping Chen

**Affiliations:** 1 College of Plant Protection, Hunan Agricultural University, Changsha 410128, China; rancki@163.com (W.Z.); Huhdfs@163.com (H.H.); hnnydxluqisen@163.com (Q.L.); PengJ0310@163.com (P.J.); cln7471lcy@163.com (L.C.); cailinhu1952@163.com (C.H.); 2 State Key Laboratory for Quality and Safety of Agro-Products, Institute of Plant Virology, Ningbo University, Ningbo 315211, China; yangjian@nbu.edu.cn

**Keywords:** long non-coding RNA, Chinese wheat mosaic virus, microRNA target, RNA-sequencing

## Abstract

**Simple Summary:**

Recent studies have shown that a large number of long noncoding RNAs (lncRNAs) can regulate various biological processes in animals and plants. However, the roles of long non-coding RNAs (lncRNAs) in the interaction between plants and viruses is unclear, particularly for the Chinese wheat mosaic virus (CWMV) interaction. In this study, we used a deep RNA sequencing strategy to profile lncRNAs involved in the response to CWMV infection in *Nicotiana benthamiana* and analyzed differentially expressed lncRNAs that responded to CWMV infection, using a bioinformatics method. We identified 1175 new lncRNAs in *N. benthamiana* infected with CWMV, with 65 lncRNAs showing differential expression. These lncRNAs were mainly enriched in plant hormone signal transduction and other pathways according to GO and KEGG pathway enrichment analyses. In addition, differential expression of XLOC_006393 after CWMV infection may be the precursor of NbmiR168c, which can respond to CWMV infection by modulating the expression of its target gene *NbAGO1*. We believe that our study makes a significant contribution to the literature because these results provide a valuable resource for studying lncRNAs involved in CWMV infection and improving the understanding of the molecular mechanism of CWMV infection.

**Abstract:**

Recent studies have shown that a large number of long noncoding RNAs (lncRNAs) can regulate various biological processes in animals and plants. Although lncRNAs have been identified in many plants, they have not been reported in the model plant *Nicotiana benthamiana*. Particularly, the role of lncRNAs in plant virus infection remains unknown. In this study, we identified lncRNAs in *N. benthamiana* response to Chinese wheat mosaic virus (CWMV) infection by RNA sequencing. A total of 1175 lncRNAs, including 65 differentially expressed lncRNAs, were identified during CWMV infection. We then analyzed the functions of some of these differentially expressed lncRNAs. Interestingly, one differentially expressed lncRNA, XLOC_006393, was found to participate in CWMV infection as a precursor to microRNAs in *N. benthamiana*. These results suggest that lncRNAs play an important role in the regulatory network of *N. benthamiana* in response to CWMV infection.

## 1. Introduction

With the application and development of high-throughput gene sequencing technology and large-scale transcriptome research, genomic information on an increasing number of species has been obtained. Research has shown that more than 90% of the eukaryotic genome can be transcribed into RNA but only approximately 2% of transcribed RNAs can encode proteins [1,2]. As a result, such non-coding RNAs were previously regarded as “DNA junk,” “noise,” or experimental artifact [3,4]. According to their genomic origin and mechanisms of action, non-coding RNAs can be classified as non-coding RNAs (ncRNAs) and regulatory ncRNAs. Housekeeping ncRNAs play a major role in ribosomal and cellular activities and include small nuclear RNA, small nucleolar RNA, transfer RNAs, and ribosomal RNAs (rRNAs). Regulatory ncRNAs can be divided into short and long non-coding RNAs based on the transcript size [1,5,6].

Long noncoding RNAs (lncRNA), which typically have lengths of over 200 nucleotides, form a subgroup of ncRNAs that has been widely identified in many species [7,8,9,10,11,12]. LncRNAs can be classified into different types according to the length of the transcript, relative position in the protein-coding genes, and other features; these types include long intergenic ncRNAs, antisense transcripts ncRNAs, and overlapping lncRNAs [3,13]. Unlike mRNAs, most lncRNAs are located mainly in the nucleus and sub-nuclear compartment chromatin, with the remaining lncRNAs found in the cytosol [3,14]. Similar to mRNAs, lncRNAs are transcribed mainly by RNA polymerase II, and some lncRNAs have similar structures as mRNAs, including the presence of a 5′-cap and 3′-poly(A) tail. In contrast, the length of lncRNA transcripts and number of exons are less than those of mRNAs. Additionally, compared with those in mRNAs, lncRNAs show lower expression levels and a higher natural mutation rate [3,14,15,16].

Recent evidence shows that lncRNAs can participate in many biological processes by regulating the expression of genes involved in growth and development, responses to abiotic and biotic stresses, and chromosome modification [3,17,18,19,20]. Although the mechanism of lncRNAs has been widely studied, few studies have investigated lncRNA functions in plants. 

For instance, 3857 lncRNAs associated with flowering in cucumber were identified, of which 1601 lncRNAs were differentially expressed at the flowering stages [21]. Moreover, lncRNA expression levels were up- or down-regulated in response to drought, cold, salinity, and other responses [1,3,22]. Compared with those of the control group, the expression levels of approximately 1800 lncRNAs changed significantly when *Arabidopsis* was subjected to drought, cold, or other abiotic stresses [22]. These results show that lncRNAs play important roles in plant biotic stress and abiotic stress responses.

Plants have evolved a set of effective defense mechanisms for inhibiting pathogen infection [1,3,16,23,24,25]. Recent research has shown that lncRNAs are an important part of these plant defense mechanisms in the response to pathogens. In wheat infected with powdery mildew, numerous lncRNAs have been identified and characterized, among which four wheat lncRNAs showed significant alterations during infection, indicating that they exert important functions [24,25]. A recent study revealed that lncRNAs may be useful for defending plants against viral infection. LncRNAs in tomato were identified and characterized by high-throughput sequencing methods after infection with tomato yellow leaf curl virus (TYLCV), which identified 2056 lncRNAs including 1767 long intergenic non-coding RNA and 289 long non-coding natural antisense transcripts. In addition, overexpression of lncRNA (SILNR1) can reduce the accumulation of TYLCV in tomato [26]. These results suggest that lncRNAs play a critical role in host immune regulatory networks.

Chinese wheat mosaic virus (CWMV) was isolated and identified from wheat mosaic disease in China and is a member of the genus *Furovirus*, family *Virgaviridae* [27,28,29]. CWMV is one of the most important wheat viruses in China and is mainly transmitted by the fungus *Polymyxa graminis* in soil. In wheat infected by CWMV, the young leaves show light chlorotic streaking and older leaves exhibit bright chlorotic streaking [28,29,30]. The CWMV genome information consists of two bipartite single-strand positive RNA1 and RNA2. CWMV RNA1 encodes three proteins involved in viral replication and movement. CWMV RNA2 is predicted to encode four proteins: The major capsid protein (CP, 19 kDa), two minor CP-related proteins (N-CP, 23 kDa; CP-RT, 84 kDa), and cysteine-rich RNA silencing suppressor [28,29,30]. Full-length cDNA clones of CWMV, which were infectious both wheat and *Nicotiana benthamiana*, have been constructed [31,32,33].

Increasing studies have shown that lncRNAs have key functions in response to drought, nitrogen, phosphate, and Cadmium stresses [1,3,22]. However, their role in the interaction between plants and viruses has not been explored, particularly in the CWMV interaction. In this study, we used a deep RNA sequencing strategy to profile lncRNAs involved in the response to CWMV infection in *N. benthamiana* and analyzed differentially expressed lncRNAs (DElncRNAs) that responded to CWMV infection using a bioinformatics method. These results provide a valuable resource for studying lncRNAs involved in CWMV infection and improving the understanding of the molecular mechanism of CWMV infection. 

## 2. Material and Methods

### 2.1. Plant Growth and Virus Infection Conditions

The model plant *Nicotiana benthamiana* was used in the experiment. *Nicotiana benthamiana* plants were grown in a greenhouse at 25 ± 2 °C and 70% relative humidity with a 16-h light/8-h dark cycle. CWMV full infection clones were constructed in our laboratory [31,32,33]. *Nicotiana benthamiana* plants at the four-leaf stage were injected with the CWMV infection clone. To confirm the success of this infection, we measured the accumulation of CWMV by quantitative reverse transcription (RT)-PCR.

### 2.2. RNA Extraction, Library Construction, and RNA-Sequencing

Leaf samples were collected at 7 days post-inoculation and rapidly frozen in liquid nitrogen. CWMV-infected and untreated samples were used to perform RNA-seq, with three independent biological replicates evaluated for each treatment. Total RNA was extracted from the leaves using an RNAprep Pure Plant Kit (Tiangen, Beijing, China). Because some lncRNAs do not contain a polyA tail, the rRNA of total RNA was removed using a Ribo-Zero rRNA Removal Kit (Illumina, San Diego, CA, USA) according to the manufacturer’s instructions. cDNA library construction and RNA sequencing were performed by Nuoan (Hangzhou, China) on an Illumina HiSeq 4000 system.

Total RNA was isolated from different tissues of *N. benthamiana* plants using TRIzol reagent (Invitrogen, Carlsbad, CA, USA) according to the manufacturer’s instructions; each sample included three independent biological replicates. Purified total RNA (1 μg) was reverse-transcribed into cDNA using a First Stand cDNA Synthesis Kit (TOYOBO, Osaka, Japan) according to the manufacturer’s instructions. The relative expression levels of these lncRNAs were analyzed by quantitative RT-PCR (qRT-PCR) using the AceQ RT-qPCR SYBR Green Master Mix Kit (Vazyme, Nanjing, China). Each q RT-PCR sample contained 10 μL of 2× SYBR PCR master mix, 1 μL each of forward and reverse primer (10 μM), 0.5 μL of cDNA, and ddH2O to a volume of 20 μL. All reactions were performed in triplicate on a 7900 Real-Time PCR System (Applied Biosystems, Foster City, CA, USA). The quantitative PCR program was as follows: 95°C for 10 min, followed by 40 cycles of 95 °C for 15 s, 60 °C for 20 s, and 72 °C for 30 s. For each treatment, three biological and three technical replicates were evaluated, and the gene for ubiquitin-conjugating enzyme was used as an internal reference in this experiment. Relative expression levels of all lncRNAs were calculated using the 2^−△△Ct^ method [32,33]. All primers used for qRT-PCR are listed in Supplementary Table S1.

### 2.3. Transcript Assembly, Mapping, and Identification of LncRNAs

To obtain clean reads, we filtered the raw sequences to remove the adapter, low-quality reads, and contaminating sequences. Clean reads were mapped to the *N. benthamiana* genome using HISAT2 software, and the transcripts were assembled using String Tie. To obtain information on the location relationships of these transcripts, they were compared with known mRNAs and lncRNAs using Cufflinks software and then assembled using Cuffmerge. Fragments per kilobase million is an important index for measuring gene expression levels. We considered transcripts as lncRNAs based on the following criteria: sequence length >200 bp, expression levels of fragments per kilobase per million >0.5, number of exons > 2, and coverage > 1. The newly assembled transcripts were compared with the *N. benthamiana* reference genome annotations, and annotated transcripts and other ncRNA transcripts were removed using Cuffcompare software. To future evaluate the potential lncRNAs, we used three predictive software packages (coding potential calculator, txCdsPredict, and Coding-Non-Coding Index) to predict their transcript-coding ability. Those with significant coding ability were removed.

### 2.4. Analysis of Differentially Expressed Genes and Prediction of Target Genes

Sting Tie software was used to calculate lncRNAs and coding genes expression levels. Differentially expressed of LncRNAs (with a *p* < 0.05 and |fold-change| > 2) were considered by edge R. Based on the regulatory patterns of lncRNAs, we predicted their potential target genes. Recently, the regulatory patterns of lncRNAs were divided into cis- and trans-acting groups. Coding genes in the 100-kb region upstream or downstream of lncRNAs were selected as *cis*-target genes. Trans-target genes of lncRNAs were predicted based on their expression levels, and the correlation between coding genes and RNA expression levels is an important basis for determining trans-regulators. To further understand the function of the lncRNAs and their corresponding target genes, the putative target genes of DElncRNAs were subjected to Gene Ontology (GO) term and Kyoto Encyclopedia of Genes and Genomes (KEGG) pathway enrichment analysis. 

### 2.5. Construction of LncRNA, MiRNA, and MRNA Regulatory Network

To determine whether lncRNAs act as potential microRNA (miRNA) precursors, DElncRNAs were aligned to the miRBase database using the Blast tool, and lncRNAs and miRNA precursors showing more than 90% homology were selected for further study. The target genes of potential miRNAs were predicted using the psRNATarget online tool (http://plantgrn.noble.org/psRNATarget/, accessed on May 2020). Regulatory networks of lncRNAs, miRNAs, and miRNA target genes were constructed using Cytoscape 3.7.1 software. 

A co-expression network was also constructed to describe the relationships between the lncRNAs and target genes using the STRING database (http://string-db.org/, accessed on July 2020). A target gene list was extracted from the database to construct the co-expression network. Because the relationship between lncRNAs and target proteins was not clear in *N. benthamiana*, we selected reference protein sequences by Blastx according to the target gene sequences, and the network was constructed based on known interactions of the reference species (*Arabidopsis thaliana*). Data on the relationship pairs between lncRNAs and target genes were imported into Cytoscape 3.7.1. Co-expression networks were obtained and adjusted using Cytoscape software.

### 2.6. Vector Construction and CWMV Infection

The tobacco rattle virus (TRV)-mediated gene silencing system was used to silence the lncRNAs [26]. The silence fragment was designed using the SGN VIGS web server (http://vigs.solgenomics.net/, accessed on August 2020). An approximately 300-bp fragment of XLOC_006393 was cloned and inserted into the TRV-RNA2 vector by double enzyme digestion (*BamHI* and *SmaI*). The TRV-VIGS constructs (containing TRV1 and TRV2: XLOC_006393) were transformed into *Agrobacterium tumefaciens* strain GV3101. The transformed *A. tumefaciens* strain GV3101 was used to infiltrate the leaves of *N. benthamiana* seedlings at the four-leaf stage, according to the method described by Yang et al. [32]. Empty TRV2 (TRV:00) and TRV1 were used as negative controls. In VIGS plants, the expression levels of XLOC_006393 were silenced, and negative control plants were subjected to CWMV infection, respectively. The CWMV inoculation method is as described in 2.1. 

To further analyze the potential target gene of *NbmiR168c*, we cloned the *NbAGO1* open reading frame, which contains the *NbmiR168c* binding site. The *NbAGO1* open reading frame containing the restriction site was inserted into pCAMBIA1300-sGFP vector by double enzyme digestion to obtain the *NbAGO1*:GFP fusion expression vector. *NbmiR168c* precursor sequences were obtained by PCR, PCR products and pCAMBIA1300 vector were digested with *SacⅠ* and *XbaⅠ*, and they were ligated using T4 DNA ligase to generate the *NbmiR168c* vector. All plasmids were transformed into *A. tumefaciens* strain GV3101 by electroporation. For *agrobacterium*-mediated transient expression in *N. benthamiana*, *Agrobacterium* cells were cultured overnight in YEP broth media containing 50 μg/mL kanamycin, 50 μg/mL gentamicin, and 50 μg/mL rifampicin, at 28 °C. The cultivated cells were collected at 5000× g for 5 min, then re-suspended in infiltration buffer (1 M KCl, 100 mM MES, and 10 mM Acetosyringone (As)). All Agrobacterium cells were then incubated at room temperature 2 h with OD values ranging from 0.6 to 1.0. Mixed Agrobacterium cells were used to co-transform *N. benthamiana* at the four-leaf stage. The fluorescence intensity of GFP was observed under UV light after 48 h.

### 2.7. Protein Extraction and Western Blot Assays 

Approximately 1 g of tissue samples were ground in liquid nitrogen and then added to 1 mL protein extraction buffer (extraction buffer containing 100 mM Tris-HCl, pH 8.8, 60% SDS, and 2% β-mercaptoethanol) and protease inhibitor cocktail tablets (1 tablet per 50 mL protein extraction buffer; Roche, Basel, Switzerland). The supernatants of all samples were collected by centrifugation at 18,000× g for 20 min at 4 °C. The supernatant was incubated in boiling water for 5 min and then separated by 12.5% SDS-PAGE. Total protein was separated by electrophoresis and transferred to nitrocellulose membranes (Life Technologies, Carlsbad, CA, USA). Antibodies against CWMV CP were used to detect CWMV accumulation following the different treatments. The membranes were incubated with anti-CP polyclonal antibodies and a secondary antibody (anti-rabbit goat IgG conjugated with alkaline phosphatase) at room temperature for 1 h, and the membranes were washed with TBST. The CWMV CP signals were visualized using a Molecular Image ChemiDoc Touch (Bio-Rad, Hercules, CA, USA).

## 3. Results 

### 3.1. Genome-wide Identification of LncRNAs in N. benthamiana

To systematically identify lncRNAs in *N. benthamiana*, we performed high-throughput strand-specific RNA-seq at 7 days post-inoculation (dpi) of the plant with CWMV and of the wild-type plants using the Illumina sequencing platform. More than 630,783,694 reads were generated by Illumina sequencing and 626,749,541 clean reads were obtained after removing low-quality reads, including those containing sequencing adaptors and sequencing primers and nucleotides with q quality scores lower than 20 (Table 1).

**Figure 1:**
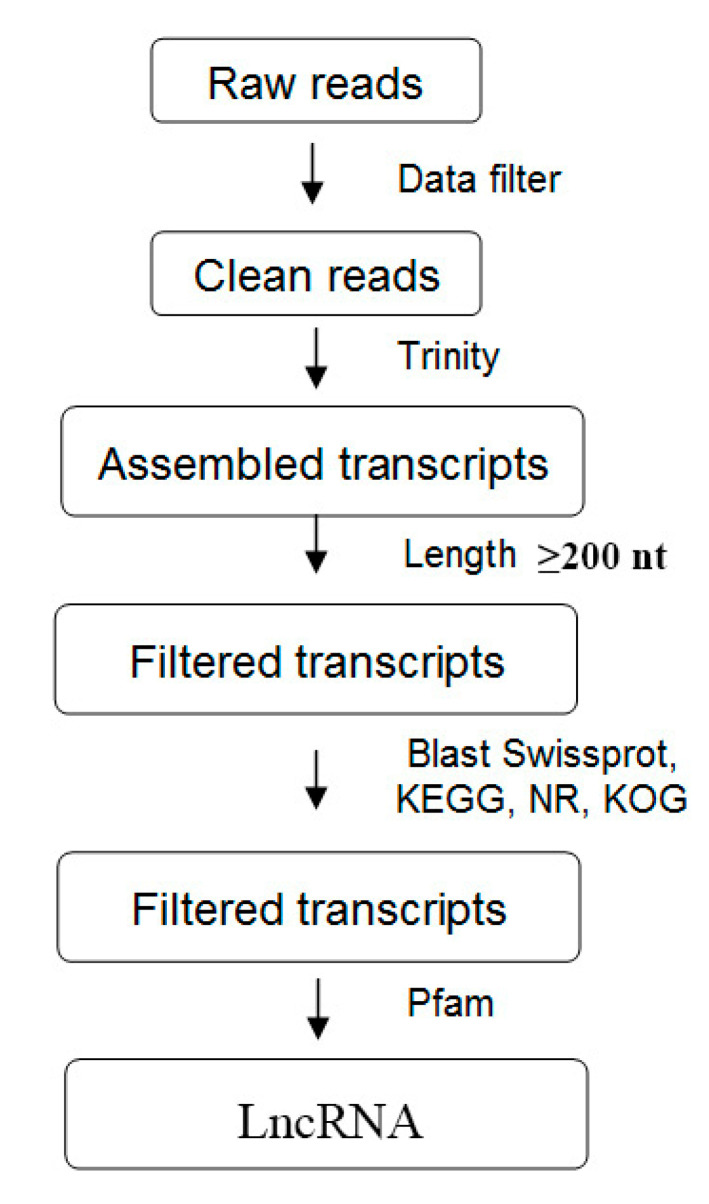
General flowchart of the pipeline used to systematically identify long non-coding RNAs (lncRNAs) in *Nicotiana benthamiana* infected with Chinese wheat mosaic virus.

**Figure 2:**
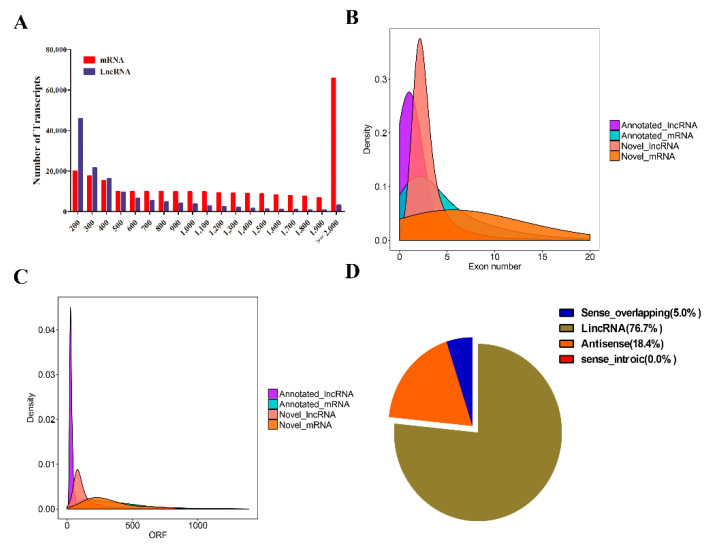
Characteristics of mRNA and lncRNA in *Nicotiana benthamiana*. (**A**–**C**) Num ber of different lengths of lncRNAs and mRNAs in these transcripts, Exon numbers and open reading frame length density characteristics in the identified lncRNAs and mRNAs; (**D**) different types of lncRNAs were identified in the leaf of *N. benthamiana*, including intergenic lncRNAs, antisense lncRNAs (lncRNAs with exonic overlap with a known transcript), sense_overlapping lncRNAs (lncRNAs with generic exonic overlap with a known transcript), and sense_introic lncRNAs (lncRNAs with intronic and exon regions of a known transcript).

The integration of the lncRNA computational identification methods and processes were shown in Figure 1 [7]. First, we constructed a *N. benthamiana* lncRNA pipeline based on high-throughput strand-specific RNA-seq using the six whole transcriptome ssRNA-seq datasets (Table 1). All clean reads were assembled *de novo* using Trinity, which generated 196,798 transcripts with a mean length of 1139 bp. A total of 65,144 unigenes with a mean length of 839 bp was produced. The size distributions of these transcripts and unigenes are shown in Figure 2A. For annotation, the 196,798 transcripts were searched using BLASTX against six protein databases (non-redundant (NR), GO, Swiss-Prot, KEGG, Pfam, and KOG) with a cutoff E-value of 10^–5^ to exclude transcripts that may encode proteins. We annotated 135,068, 222,956, 170,648, 92,179, 188,840, and 116,847 transcripts in the Swiss-Prot, NR, Pfam, KEGG, KOG, and GO protein databases, respectively (Table S2). We next evaluated the coding potential of the remaining transcripts and identified novel lncRNAs. To eliminate protein-coding transcripts, HMMER was used to scan each transcript unit in all three reading frames to exclude those encoding any known protein domains cataloged in the Pfam protein family database [11,34]. In addition, considering the characteristics of the lncRNA sequences (≥200 nt) and their differences from other classes of RNA (mRNA, transfer RNA, rRNA, small nuclear RNA, small nucleolar RNA, pre-miRNA, and pseudogenes), we classified the transcripts into different subtypes using Rfam software [35]. Finally, the coding potential of each transcript was analyzed using coding potential calculator software [36,37,38]. Only transcripts with a score ≤−1 in these calculations were retained, giving 1175 lncRNA candidates (Table S3, Table S4 and Text S1). The exon number of novel lncRNAs mainly ranged from 2 to 4, and ORF lengths of novel lncRNA mainly ranged from 150 to 250 bp, respectively. These results were displayed in Figure 2B and Figure 2C. In addition, as shown in Figure 2D, our analysis of these newly identified lncRNA types shows that approximately 76.7% of the novel lncRNA is mainly long intergenic ncRNAs.

### 3.2. Characterization and Expression Analysis of N. benthamiana LncRNAs in Response to CWMV Infection

**Figure 3:**
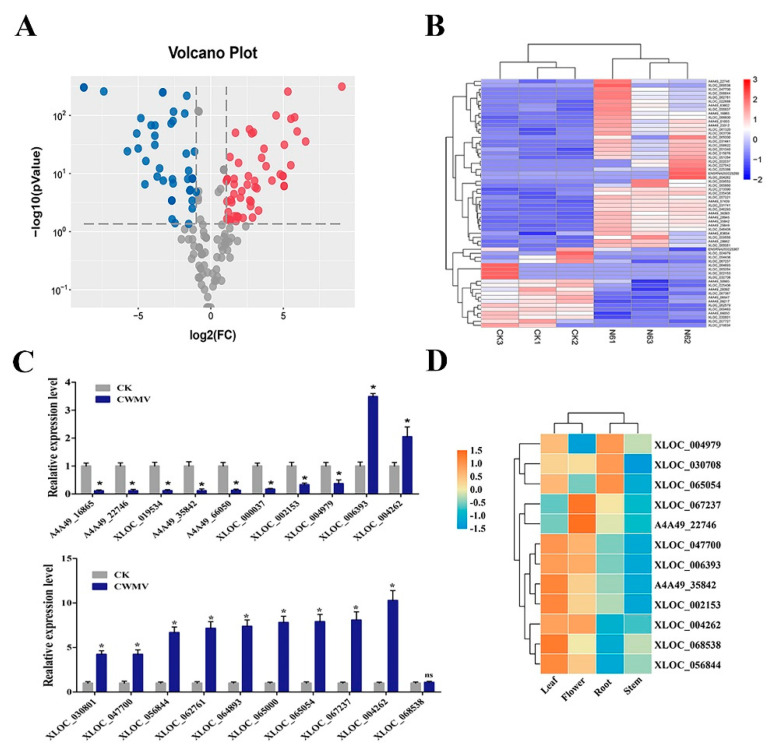
Identified differentially expressed lncRNA responses to Chinese wheat mosaic virus in *Nicotiana benthamiana* and verification of the expression level of differentially expressed lncRNAs (DElncRNAs) in different tissues by qRT-PCR. (**A**) Volcano plots were created to distinguish DElncRNAs, Vertical lines indicate 2-fold up- or down-regulation, and horizontal lines represents *p* = 0.05. Red and blue dots represent up- and down-regulated lncRNAs, respectively. (**B**) Heatmap was constructed based on expression profile data of DElncRNAs between CK and CWMV infection samples (log_2_ |fold-change| > 2 and false discovery rate < 0.05), blue and red represent down- and upregulated lncRNAs in different samples, respectively; (**C**) partial DElncRNAs were verified by qRT-PCR. (**D**) Expression levels of partial DElncRNAs in different tissues, including root, stem, leaf, and flower, were analyzed by qRT-PCR. Heatmaps were constructed based on the qRT-PCR results, and red and blue colors represent higher and lower expression of lncRNAs in different tissues.

Global inspection of expression normalized to reads per kilobase of transcript per million mapped reads for all lncRNAs was performed using RSEM software [39]. To identify differentially expressed *N. benthamiana* lncRNAs between the control check (CK)and CWMV samples, those with a greater than 2 fold-change and false discovery rate < 0.05 were considered as differentially expressed. A total of 65 lncRNAs were differentially expressed between the CK and CWMV samples, including 42 and 23 up- and down-regulated lncRNAs, respectively (Figure 3A and Figure 3B). These significant lncRNAs from the six samples were used as features for hierarchical clustering [40], which clustered samples into two groups. These results indicate the good repeatability of the lncRNAs in the three biological repetitions and that they could be used for subsequent analyses.

### 3.3. Identification of N. benthamiana LncRNAs by qRT-PCR

Twenty of the DElncRNAs identified by RNA-seq were validated by measuring their expression levels by RT-PCR. As shown in Figure 3C, the expression levels of XLOC_006393, XLOC_030708, XLOC_030801, XLOC_047700, XLOC_056844, XLOC_062761, XLOC_064893, XLOC_065000, XLOC_065054, XLOC_067237, and XLOC_004262 were significantly increased after CWMV infection. However, the expression of A4A49_16865, A4A49_22746, A4A49_35842, XLOC_000037, XLOC_019534, A4A49_66050, XLOC_002153, and XLOC_004979 were slightly downregulated after CWMV infection. The results are consistent with the RNA-seq expression pattern, indicating that these DElncRNAs play a major role in the response to CWMV infection in *N. benthamiana*.

### 3.4. Tissue Expression Profile of N. benthamiana LncRNAs

To understand the expression patterns of DElncRNAs in different tissues, XLOC_004979, XLOC_030708, XLOC_065054, XLOC_067237, XLOC_047700, XLOC_006393, A4A9_22746, A4A9_35842, XLOC_002153, XLOC_004262, XLOC_068538, and XLOC_056844 levels were examined in the roots, stems, leaves, and flowers by qRT-PCR. As shown in Figure 3D, the expression levels of most lncRNAs were upregulated in the leaves and flowers and downregulated in the roots and stems. In the leaves, only XLOC_067237 and A4A9_22746 were slightly downregulated, whereas other lncRNAs were significantly up-regulated. Notably, XLOC_067237 and A4A9_22746 were significantly upregulated in the flowers, showing much higher expression levels than those in other tissues. However, almost all lncRNAs were down-regulated in the roots and stems except for XLOC_004979, XLOC_030708, and XLOC_065054. These results demonstrate that the evaluated lncRNAs are important in the plant growth and developmental stages.

### 3.5. GO and KEGG Enrichment Analysis of LncRNAs Target Genes (LTGs)

**Figure 4:**
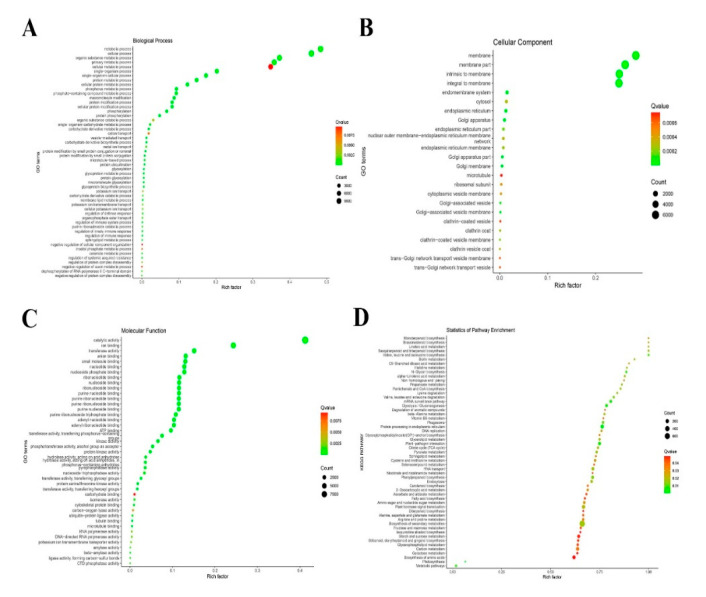
GO and KEGG pathway enrichment analysis of differentially expressed lncRNAs (DElncRNAs) in response to Chinese wheat mosaic virus in *Nicotiana benthamiana*. To analyze DElncRNAs function, we predicted potential target genes of these DElncRNAs and evaluated them by GO and KEGG pathway analysis. These potential target genes were individually enriched into biological processes (**A**), cellular components (**B**), molecular function (**C**), and KEGG pathway (**D**). The Rich factor is the ratio of differentially expressed gene numbers annotated in this pathway term to all gene numbers annotated in this pathway term. The Q-value is a corrected *p*-value ranging from 0 to 1, with lower values indicating greater intensiveness. The circle represented the number of enriched genes, with a larger number indicating more enriched genes.

LncRNAs perform biological functions by regulating their *cis-* and *trans-*target genes. A total of 13,211 target genes regulated in *cis*- and *trans*- were predicted for 63 DElncRNAs, of which 299 were *cis*-regulatory and 12,912 were *trans*-regulatory. To further understand the mechanism of these lncRNAs, all predicted target genes were annotated by GO and KEGG enrichment analyses. Enrichment analysis of the LTGs showed that 119 GO terms were enriched in cellular components, molecular functions, and biological processes (Figure 4A). Fifty-three GO terms were enriched in biological processes, of which metabolic process (GO:0008152), cellular process (GO:0009987), primary metabolic process (GO:0044238), and cellular metabolic process (GO:0044267) were the most abundant GO terms. At the molecular function level, the GO terms were significantly enriched in catalytic activity (GO:0003824), ion binding (GO:0043167), and transferase activity (GO:0016740). Among these GO terms, the most abundant GO terms in the cellular component category were membrane (GO:0016020), membrane part (GO:0044425), and intrinsic to membrane (GO:0031224) (Figure 4A–C). To better understand these lncRNAs, all LTGs were examined by KEGG pathway analysis using KOBAS software. The results showed that these LTGs were significantly enriched in 55 different pathways (Figure 4D), of which the most abundant LTGs were mainly enriched in four pathways: Biosynthesis of secondary metabolites, plant hormone signal transduction, carbon metabolism, and plant pathogen interaction following CWMV infection.

### 3.6. Construction lncRNA-miRNA-mRNA Regulatory Network during CWMV Infection

Previous studies revealed that lncRNAs can regulate gene expression through multiple RNA-mediated gene regulation mechanisms, with lncRNAs acting as miRNA precursors in important regulatory mechanisms. To determine whether lncRNAs are precursors of miRNAs, all lncRNA sequences were aligned to the miRBase database to screen for miRNA precursors using Blastn. Eight expressed lncRNAs were identified as three completely known miRNA precursors (Figure S1), among which only the lncRNA XLOC_006393 was significantly differentially expressed in CWMV infection. Sequence alignment analysis revealed that XLOC_006393 can act as a miRNA precursor of *NbmiR168c*. In addition, we analyzed the secondary structures of lncRNA transcripts using the RNAfold web server. The results suggested that XLOC_006393 contained a stable stem-loop structure for miRNA precursors. To better understand the function of DElncRNAs, we constructed a DElncRNAs-miRNA-mRNA regulatory network using Cytoscape. The expression regulatory network revealed that lncRNAs can indirectly regulate downstream mRNA expression by regulating the expression levels of one or several miRNAs. In addition, functional annotation of these target genes showed that some were involved in RNA-mediated gene silencing and abscisic acid-mediated signal transduction. In summary, lncRNAs act as major regulators of miRNAs and participate in many biological processes.

### 3.7. Silencing XLOC_006393 Inhibits Accumulation of CWMV in N. benthamiana

**Figure 5:**
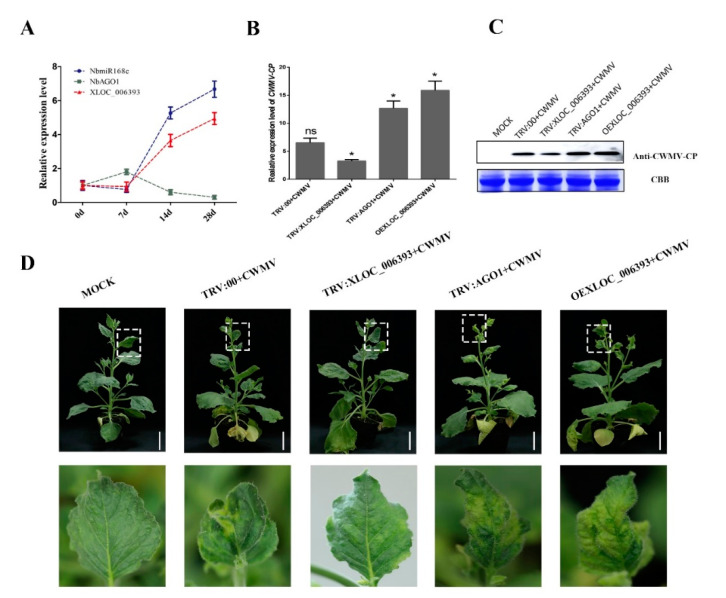
Validation of XLOC_006393 by virus-induced gene silencing. (**A**) The expression levels of XLOC_006393, *NbmiR168c*, and *NbAGO1* were analyzed at different time points (0, 7, 14, and 28 days) after CWMV infection; (**B**,**C**) accumulation of CWMV CP was detected in different plant materials, including TRV:NbmiR168c, TRV:NbAGO1, TRV:XLOC_006393, and TRV:00 by qRT-PCR and Western blotting (WB), of which coomassie brilliant blue (CBB) was used to calculate the protein content of different samples—full WB images in Figures S2 and S3. * and ns showed significant and no significant differences between the two samples, respectively; (**D**) mild green mosaic lesions formed successively in the leaves of different plant materials after 30 days of CWMV infection. Bottom photograph is an enlargement of the boxed part of (D). These plant materials contained Mock, TRV:00, TRV:XLOC_006393, TRV:NbAGO1, and OEXLOC_006393.

To investigate the potential biological functions of the lncRNA XLOC_006393 in *N. benthamiana* respond to CWMV infection. We constructed the lncRNA XLOC_006393 silent plant using the VIGS method. Compared with that of the negative control, the expression level of XLOC_006393 was reduced by more than 50% in VIGS plants. We selected three VIGS plants (#2, #4, and #7) and negative plants for CWMV infection. At 21 days post-infection, we observed more severe mosaic symptoms in #2, #4, and #7 plants than in those of TRV: 00 plants. In addition, total genomic RNA from CWMV-infected *N. benthamiana* plants was extracted to detect virus accumulation by qRT-PCR. Accumulation of CWMV coat protein in the TRV: XLOC_006393 plants was significantly decreased compared to that in the control group (Figure 5A). Western blotting also showed lesser CWMV CP protein accumulated in the leaves of the TRV: XLOC_006393 plants than that in the control leaves (Figure 5C). These results suggest that XLOC_006393 plays a major role in the response to CWMV infection in *N. benthamiana*. 

The prediction results showed XLOC_006393 can be used as an *NbmiR168c* precursor. To further analyze the function of XLOC_006393, we predicted the potential target gene of *NbmiR168c*. The results showed that multiple *NbAGO1* genes can bind and cleave NbmiR168c. We analyzed the expression levels of *NbmiR168c*, *NbAGO1*, and XLOC_006393 after CWMV infection by qRT-PCR. *NbmiR168c* and XLOC_006393 expression levels were significantly upregulated at different time points after CWMV infection (Figure 5A). However, *NbAGO1* expression was downregulated at these time points after CWMV infection and negatively correlated with *NbmiR168c* and XLOC_006393. To verify these results, we constructed 35S:XLOC_006393 overexpression transgenic plants and TRV:*NbAGO1* plants. We found that TRV:*NbAGO1* and 35S:XLOC_006393 plants showed more severe mosaic symptoms compared to those by TRV:XLOC_006393 plants after CWMV infection (Figure 5D). In addition, CWMV CP accumulation in the leaves of TRV:*NbAGO1* and 35S:XLOC_006393 plants was significantly higher than that in the control and TRV:XLOC_006393 (Figure 5C). These results suggest that XLOC_006393 responds to CWMV infection by acting as an *NbmiR168c* precursor, which can regulate *NbAGO1* expression.

### 3.8. Expression Regulation of NbAGO1 by NbmiR168c through Post-Transcriptional Process In Vivo

**Figure 6:**
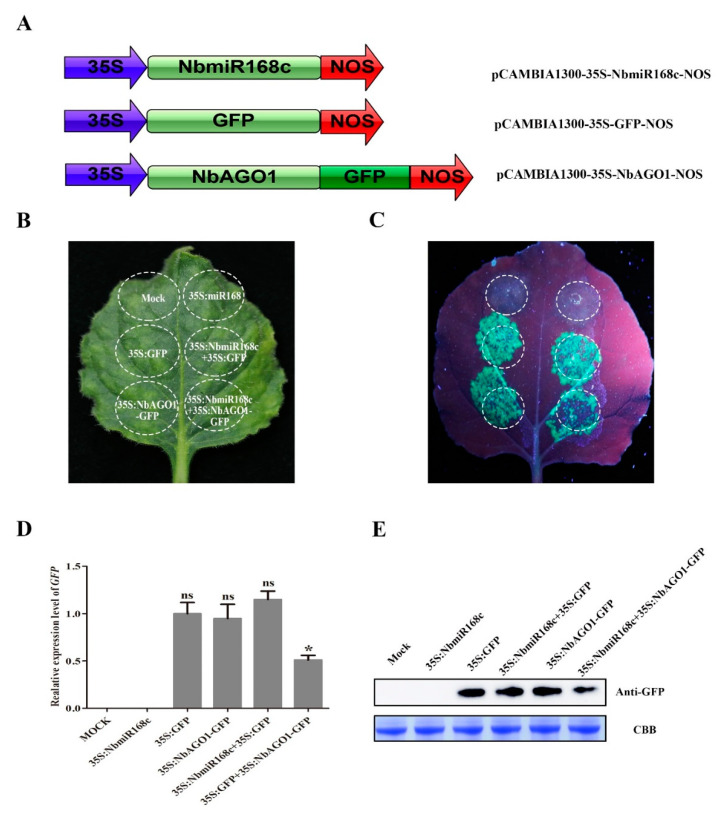
*NbmiR168c* regulates *NbAGO1* expression by a post-transcriptional process in vivo. (**A**) Construction of NbAGO1-GFP fusion expression vector and NbmiR168c expression vector; (**B**,**C**) GFP fluorescence intensity of infiltrated sites of the leaf with different vectors; (**D**,**E**) accumulation of GFP was detected in different *Nicotiana benthamiana* leaves after 48 h of transient infection with different vectors by qRT-PCR and Western blotting—full WB images in Figures S4 and S5. * and ns showed significant and no significant differences between the two samples, respectively.

To further verify the regulatory mechanism of *NbmiR168c* and its potential target gene, *NbAGO1*, the *NbAGO1*-encoding sequence was fused upstream of GFP in the plant expression vector, generating pCAMIBI1300-35S-*NbAGO1*-GFP as a reporter. The *NbmiR168c* precursor sequences were inserted into the plant expression vector pCAMIBI1300 rather than the GFP gene, and the resulting vector pCAMIBI1300-35S-*NbmiR168c* was used as an effector (Figure 6A). As shown in Figure 6B,C, GFP fluorescence was markedly attenuated after *NbAGO1*-GFP and *NbmiR168c* co-injection. However, the leaves were infiltrated with equally mixed GV3101 cells containing *NbmiR168c* and *NbAGO1*-GFP. We also analyzed the expression of GFP at the transcript and protein levels after co-injection. The expression level of GFP was significantly decreased when *NbmiR168c* and *NbAGO1*-GFP were co-injected. In addition, GFP protein accumulation levels were much lower than those after *NbmiR168c* and *NbAGO1*-GFP were co-injected. These results suggest that *NbmiR168c* can recognize *NbAGO1* and regulate its expression at the transcriptional level.

## 4. Discussion

In this study, lncRNAs that responded to CWMV infection in *N. benthamiana* were identified and studied by high-throughput sequencing techniques. Recently, 1175 putative lncRNAs were identified in *N. benthamiana* inoculated with CWMV at the whole transcriptome level. Based on bioinformatics analysis, 65 DElncRNAs were found to respond to CWMV infection; among them, 42 and 23 lncRNAs were up- and down-regulated, respectively. To gain insight into the function of these lncRNAs, GO terms and KEGG pathway enrichment analyses were applied to target genes of the DElncRNAs. Some targets accompanied by lncRNAs were enriched in plant hormone signal transduction and plant pathogen interactions. These metabolic pathways are considered as important for the plant defense against pathogens. These results suggest that lncRNAs play an important role in CWMV infection of *N. benthamiana*. 

In a recent study, many lncRNAs related to plant responses to pathogen infection were identified [16,23,24,25]. In tomatoes, 688 DElncRNAs were identified between *P. infestans*-resistant and -susceptible tomatoes, among which the high expression level of lncRNA1637, which acts as an antisense transcript of SIGRX22 that can induce SIGRX21 and SIGRX22 expression and ROS accumulation and disease symptoms, were reduced compared to those in the wild-type, thus enhancing tomato resistance to *P. infestans* [41]. Additionally, 20 lncRNA responses to *Fusarium oxysporum* infection were identified in *A. thaliana*, and a large number of *cis*-regulatory elements in response to pathogen infection were found in these lncRNA promoters, which responded to *F. oxysporum* [16]. The lncRNA SILNR1 in tomato can complement the non-coding region of the TYLCV genome and interacts with intergenic region-derived virus-derived RNAs to regulate disease development after TYLCV infection [16,26]. Our study revealed 65 DElncRNAs in *N. benthamiana* during CWMV infection. These lncRNAs, which were upregulated after CWMV infection, were detected in VIGS plants showing mosaic symptoms during CWMV infection. Silencing of XLOC_006393 slightly reduced the mosaic symptoms and accumulation of CWMV compared to those in wild-type plants. These findings suggest that XLOC_006393 positively regulates *N. benthamiana* in response to CWMV infection.

Numerous studies have indicated that lncRNAs can function as miRNA precursors or endogenous target mimics [5,41,42,43,44]. Some lncRNAs can act as miRNA sponges, which contain miRNA binding sites and have been shown to regulate miRNA and target gene expression [43,45]. This recently discovered regulatory mechanism of miRNA functions in plants. LncRNAs can act as miRNA precursors and were first discovered in humans and mice. The first imprinted ncRNA (H19) can produce a 23-nucleotide miRNA involved in regulating vertebrate development at the post-transcriptional level [44]. In plants, ptc-miR1448 and ptc-miR482a can be processed from TCONS_0006173, which may play a major role in the response to stress in *P. tomentosa*. Moreover, TCONS_00035853 and TCONS_00021861, which can induce expression following low-nitrogen treatment in *P. tomentosa*, act as pto-miR1448 and pto-miR168b precursors [5]. In this study, we identified eight lncRNAs that can act as miR168c, miR4995, and miR1664 precursors. Virus-specific siRNAs were produced by RNase-III ribonuclease and Dicer-like proteins, and then loaded into the RNA-induced-silencing complex (RISC) to mediate virus accumulation in the host. In this process, AGO proteins play an important role as the central component of the RNA-induced-silencing complex [46]. Interestingly, as a feedback mechanism, the mRNAs of the AGO protein family were cleaved and translationally inhibited by miRNA. MiR168 is one of the most abundant and highly conserved miRNAs in plants and can directly regulate the expression of AGO1, the most important miRNA effector in the miRNA pathway [46,47]. Studies have shown that the expression levels of *ARGONAUTE1* (AGO1) were induced, but were not accompanied by increased AGO1 protein accumulation, and led to specific induction of miR168 accumulation in Cymbidium ringspot virus-infected plants [47]. Similarly, studies have shown that miR168 and AGO1 are upregulated in rice during ringspot virus infection [48]. XLOC_006393, which was significantly upregulated after CWMV infection, can act as an *NbmiR168c* precursor. In addition, silencing of XLOC_006393 reduced the accumulation of CWMV in *N. benthamiana*. These results show that lncRNAs, which respond to CWMV infection, can function as a new category of miRNA precursors and regulate miRNA and target gene expression to cope with CWMV infection.

## 5. Conclusions

Increasing studies have shown that lncRNAs play a major role in plant growth and development and in the response to biotic and abiotic stresses. However, the roles of lncRNAs in plant viral infections are relatively unknown. In this study, 1175 new lncRNAs were identified in *N. benthamiana* infected with CWMV. Of these, 65 lncRNAs, including 42 lncRNAs were upregulated and 23 lncRNAs were downregulated, showing differential expression after CWMV infection. These lncRNAs were mainly enriched in plant hormone signal transduction and other pathways according to GO and KEGG pathway enrichment analyses. In addition, differential expression of XLOC_006393 after CWMV infection may be the precursor of *NbmiR168c*, which can respond to CWMV infection by modulating the expression of its target gene *NbAGO1*.

## Figures and Tables

**Table 1:** Summary of the RNA-seq data.

Sample Name	Raw Reads	Clean Reads	Clean Reads Rate (%)	Q20%	Q30%	GC%	Mapped Reads	Mapped Reads Rate (%)
CK1	98,235,824	96,516,697	98.25	97.06	93.87	43.95	59,010,308	61.14
CK2	104,512,954	103,132,920	98.68	97.14	94.04	44.85	61,372,953	59.51
CK3	107,653,544	106,146,914	98.60	97.22	94.24	43.74	63,872,643	60.17
N61	101,064,306	99,414,122	98.37	97.16	94.08	43.95	49,184,563	49.47
N62	95,583,802	93,815,501	98.15	97.26	94.27	43.78	47,780,234	50.93
N63	95,751,460	94,688,618	98.89	96.25	92.2	43.79	52,078,740	55.43

## Data Availability

RNA-seq data of *N. benthamiana* infected or uninfected with CWMV have been submited to the NIH.

## Supplementary Materials

The following are available online at https://www.mdpi.com/2079-7737/10/3/232/s1, Table S1: List of primers used for this study; Table S2: Number of transcripts annotated in different databases; Table S3: Characteristics of genes and transcripts in *N.benthamiana*; Table S4: All of Novel lncRNA annotation information in this study; Text S1: All of Novel lncRNA sequences were identifted in this study; Figure S1: The interaction network among lncRNAs, miRNAs and miRNA target protein-coding genes, Figure S2: Full Western blotting image for Figure 5C—Anti-CWMV CP; Figure S3: Full Western blotting image for Figure 5C—CBB; Figure S4: Full Western blotting image for Figure 6E—CBB; Figure S5: Full Western blotting image for Figure 6E—Anti-GFP.
