# Supplementary material for: Genome-Wide Identification and Characterization of Long Noncoding RNAs Involved in Chinese Wheat Mosaic Virus Infection of Nicotiana benthamiana
Source: Biology (Basel). 2021 Mar 17;10(3):232. doi: 10.3390/biology10030232 (PMC8002735; doi:10.3390/biology10030232)
Supplement: Supplementary file 1 [file biology-10-00232-s001.zip › biology-1095616-supplementary.pdf]

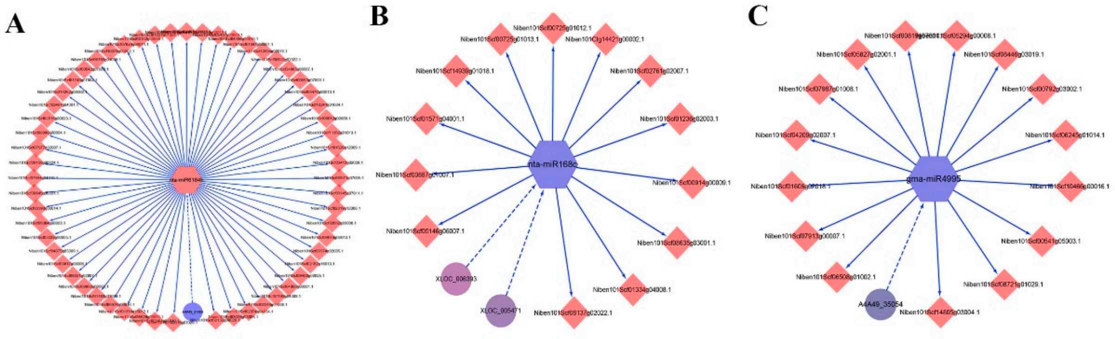

Figure S1. The interaction network among lncRNAs, miRNAs and miRNA target protein-coding genes.

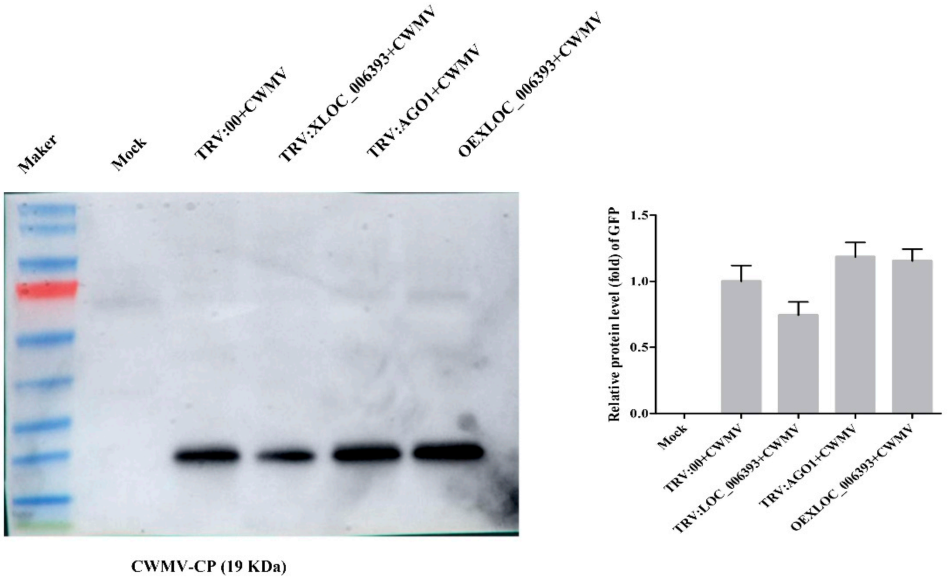

Figure S2. Full Western blotting image for Figure 5C—Anti-CWMV CP.

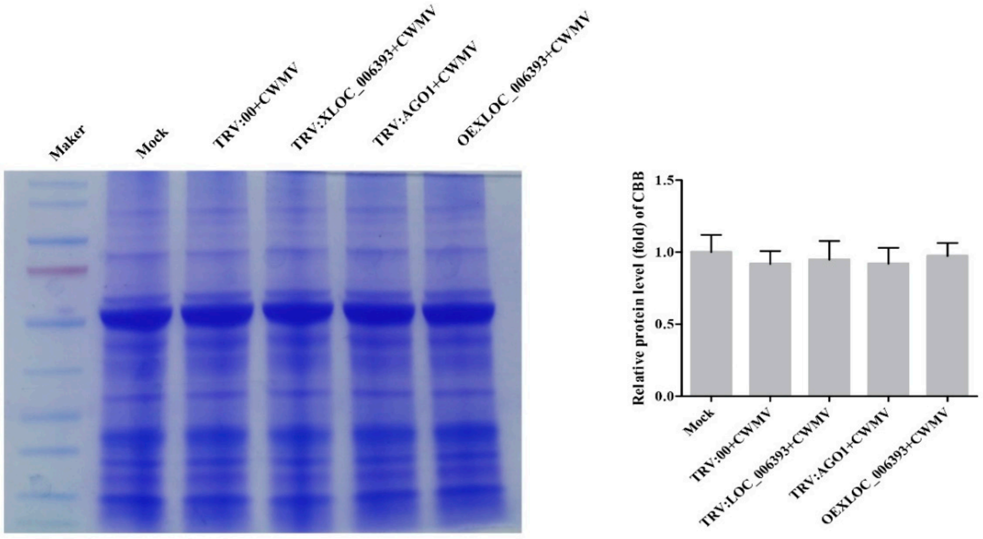

Figure S3. Full Western blotting image for Figure 5C—CBB.

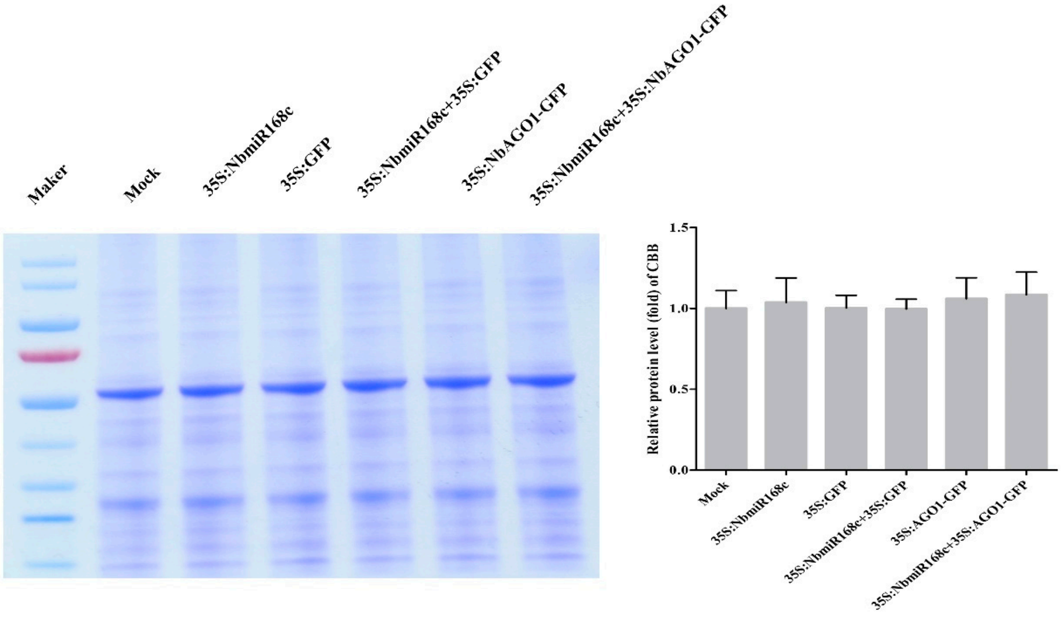

Figure S4. Full Western blotting image for Figure 6E—CBB.

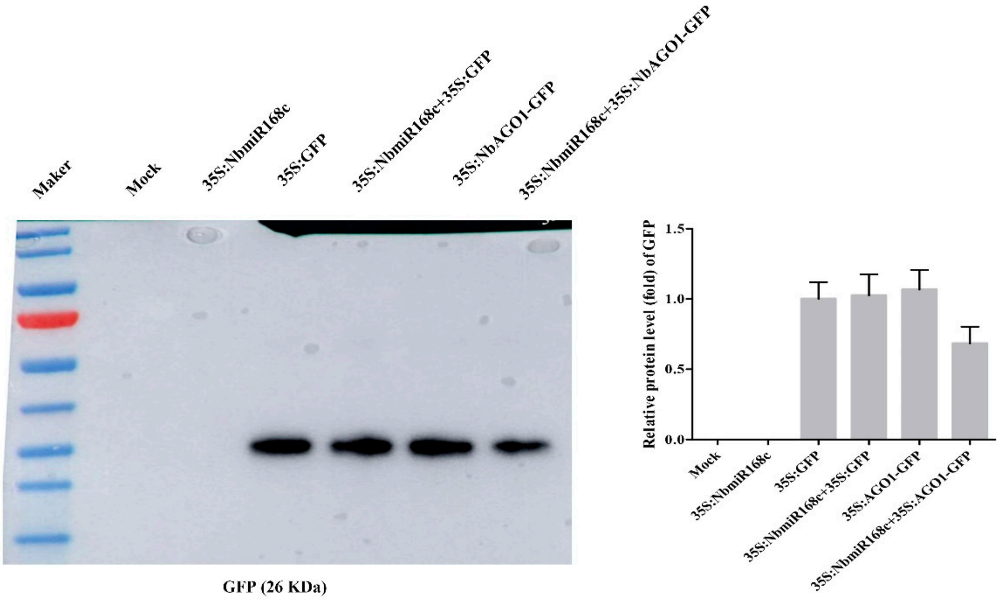

Figure S5. Full Western blotting image for Figure 6E—Anti-GFP.

Table S1. Primers used in this study.

Purpose	Primer Name	Sense Primer Sequence(5'–3')	Antisense Primer sequence(5'–3')
Used as qPCR	A4A49_35842	ATCACGAACCCGATTCTG	GCGTTGGAGGAAGTTTACA
	XLOC_068538	ACACTCAACACTCTGTGCTCA	TGAGTTGTCTGTGCTTAGCG
	XLOC_004979	TCATCCACTTCATTGACCG	ATTCTGCTCTGTCCTTGGC
	XLOC_067237	AGATAACGCAGGTGTCCTAAG	CACGGTTCGTATTCGTATTG
	A4A49_22746	CACCAACTCCGTGAATACC	CAGCAAGTCCTACAAGAGGTT
	XLOC_004262	TGACCAAGGCGTTGACTT	TCGTGTGCTCCCTACTTTG
	XLOC_047700	TCCTTGACAATGGAGATGC	TGTTGAACTGGTTTCGCC
	XLOC_064893	TTCTGTCTCAGTCACCGTGT	CCTGAAACTACTTGTGCCAA
	A4A49_66050	AACCTGGACAAACACAGTTG	CAGAAGACAGAGTGGATGAGG

	XLOC_056844	AGGAGCAGGAGTTGGCTTA	TTCCCACAAATCTACACTGG
	XLOC_062761	CCATTATCACTTCGGAGACG	CGGCACAAGATGAGATTTC
	A4A49_16865	ACTCTCTTGTCCATTCCGTC	TAGACAATGAGGACGGAATG
	XLOC_065054	AGGCGTTGACTTCTTGAAAC	GCTCGTGTGCTCCCTACTTT
	XLOC_002153	CCCACCTTCTTCCGAATA	TCCAGAGGATGAAAGTAGCAG
	XLOC_030708	TGTGAAGAGAGAGGACTTTGAG	CCCATTAGATGACCAAGTGTC
	XLOC_065000	GAGACATCCTGAAGCCCTAA	GCTCAAGAAATCGCTGAATG
	XLOC_019534	CGTGTGAATCTGTGATGGTG	TCTGCCAAACCCAAACGA
	XLOC_006393	ACGCTCACAGGAAACAAGC	CATCAACTGAATCGGAGACTG
	XLOC_030801	ACATCTTTGAGTTTCCCTGC	CATAGGTGAGAAGGAAGCCA
	XLOC_000037	AAACTCACCTCTGGAAGAAGG	TTGACATTCACGGGAAGC
	NbUBC	TGGAGGTACATTTAAGCTGACAC	TCACAGAGCAAAGACTGGATTG
	NbMIR168c	TGGAAGGGGCATGC	GTGCAGGGTCCGAGGT
	NbMIR168c-steam-loop	GTCGTATCCAGTGCAGGGTCCGAGGTATTCGCACTGGATACGAC CTCCTCT	
	NbAGO1	CGTCCTACACAGATGGGATTA	TTTACACCTCTCAGTGCCTTC
For cloning of silent genes	TRV:XLOC_006393	TTGGAGTTATACGCTCA	CTCGCCGGCGACAATGAC
	TRV:NbAGO1	TATTTTCGAGAAACATATG	AGCCTCAACTTGTGCCAAC

Table S2. Number of transcripts annotated in different databases.

	Transcript Number	Swiss-prot Database	NR-Database	Pfam-Database	KEGG-Database	KOG-Database	GO-Database
Transcript number	332628	135068	222956	170648	92179	188840	116847
Annotation rate (%)	100	40.61	67.03	51.30	27.71	56.77	35.13

Table S3. Characteristics of genes and transcripts in N.benthamiana.

	All	Median GC%	Mean GC%	Min Length	Median Length	Mean Length	Max Length	Total Assembled Bases
Gene	65144	39.60	39.54	201	547	839	20418	54714923
Transcript	196798	39.70	39.62	201	876	1139	20148	224179653

Text S1. All of novel lncRNA sequences that were identified in this study.

>TCONS_00000040

AAATCAAGCAATTCATGATTAGCTAAGTAAACTCACCTCTGGAAGAAGGAACCAGCTTTCCGTCTTTTTCATA
AAAGTCACGTATTGCCACAAAGGGCTTCCCGTGAATGTCAAGAATCCCGATACTCCTCTTGTCTGACAGCTTA
CAAATAGTTCGACAATTATTCTCATTGGAATCAAGAAAATTCCCATTAGGCAATGAACCATCTGCTTCTTGTTT
TTGAGTAGGTACGTTGTCTGCTGACAATTGTACTTCAGCTACAAAATCCAATGTGGGTTGTTCAACTGTTGAG
AGTAAAAAAGACTCAATAACATGCCTTATAAACTGCTTCTCTTGAATGTTCAATATCTCAGTACCAAGCTGCT
GGGCTACAGTGGTTCGAACACTGTACTCTGTTGCTGTTTCTATGTCAGCTGTTTTAAGGATGTCCAA

>TCONS_00000056

AGCGCACACTCGATTTCGACACCGAGAAGCAAAAAAGCAATGGAACCTTCACTTCATCTTCAAGTTTTCTTGA
ATTTCGAGACAGACGCATAGCATATCCAATTCCTCACGTCACTGAATCAACAAAACCAACTATTTATACGAA
ATGGAGTTAGGGCTGGTCACCCTGCTAAGAGCTGCATGGATTGCTGCAACTTTGCCAATTGTTATTGCTTTGAT

TCCTTCCTCAAAGCTTAGGTCTTTTCAACAGTTCTTGTTAGGGTTTGCTAAGCGTGGCAAGATTGTGCAGTCTT
CCTCAAAT
>TCONS_00000148
GAACTCAGCGGTATAGGCTTTCAGATCTCAGATTCTCTGCAATCTCAGTTTCCCATCGATCTCCATTCTCAAGA
AGCTTCTCCTTGTCGTAAAGGAGTTCCTCATGAGTTTCAGTTGCAAATTCAAAGTTAGGCCCCTCAACAATGG
CATCAACAGGACAGGCTTCTTGGCAGAATCCACAGTAGATGCACTTTGTCATATCAATATCATA
>TCONS_00000359
ATCTAGACAGCAAGCTGAGGTGATCGCATTGTCTAAGCTGACATGCTTTGTGCTGCTTCATGAGCATAATAAT
CTGCTCCAGCCCAAATCAATCTGGAATGCAGATAAAGAAAGAGATAAGTGCCCACTTGTGGAGCACATATTG
CACTTGGATTCTATAGAAGTGAACATTTCCCAAGGTATAACATTGCCAGATGCCCCCAAATTTGGGAAAGTGT
TTGGAGGTCAGTTTCTTGGGCAGGTAAAATTGAAATGTTC
>TCONS_00000391
GAAACCAATTAAGGCCTCCATAATCAGTTAGCTGGCATGTCATGAATTTCGCTGCATTTACCTCTGCTCAACT
AACCACCTTTTCCTCAACTCTCTCCTTTTTCTCTTGACTTTTGTGGACTAAGTTTGAGCTAAATTTGATCTATTCA
TGTATAAATTCATAGAATAACATCACTTGGATTGATAAGAAAGATAAGTTGTGGATTCTTTTAATTTTAGGGA
AGAATGGAAAGTTATACGGTGAAAGTTGAAGAAAGTCGACCGGCAGTTGATGGAAAGCCATCTGCAGGGCT
AGTTTATAGGTGCATTTATGCTAAAGATGGTCTCATGGAAATGCCTTTTAGTTTTGAGTCACCTTGGGATTTCTT
CAGCTCTTTTTCACACAATGTACCTCATAATTGAGAGTTTCAAGAGTTTACTGGAATTGATTTTAAGGAGAAG
ATGTATCGTTTCATGCTT
>TCONS_00000426
CTGGTAAAAGATTTCAACTGGTTCTGCATGCACGTTGGTTTCCTCTGTTGTTGCTGAGAGATCCATGGGTTCTT
CACCCTGGTATGAACTATAGTTAGCAGTACCAAGGAAACTCTGCTCTTTGTCTACCAAACTTAAGCCTTGCAG
GGCAGCATCTTTAGTTTTTGTACTTCCTTCATAAACATCTGATTTACCCCCTTCATCAGCAACAAGTTCTGGAG
CAGCATCTTCACCTCCCTGTTCATCACGGATCTCATTAACATCGGGTTCCAACGGCAAATTCTCATGCTGGCTT
GGTGGAAAGACAAAGTGACCAGACATAAACATTGCCTGGGAAGTCTCAGCTACATACTCTGCACTACTATTA
TTATCATCATTCGGATCAACCTGCTTTGGACCAGGGGCAGCAGCATATGCTGATTGTGTAAGTGACACAACTT
CCCACTCATTTCCACGAGGAGCAGTTTCCTCCCCTTCCTCGTTGCTAGCCATCTTTCGGAAATTGGAACAAA
>TCONS_00000794
AGCTATACATACTTTTATTGATAGTAAAGCAGTTTACAGAGGAAAAGGGAAAAAATTACTAGAAGCACAGGT
GCCAAGCATGCAAGGATTTGCACACCAGTTTTCTTATCTAACGTGAAAGAACCAGACGCCATCCTCATCCTTG
TCATAATGCCTTTAGAAATAGAATATGCAGCAAAAAGCTAACAATTCTCCGAAATGCAAGTCCAGCAGTTAT
GTCACCGGTTTGAGAAGGGAACTTTCAAGGAGATGGATAGACTGAGATTCTTGCCTCAGGCAGTAGCATCAG
GCTGCTTTTCTGGCA
>TCONS_00000825
CGGAAGGACATTCTAGTTCTAGTAAACATATGTTTAGTTACAAGCTGTTCAAATTTTGCAATTGGAACAGAGC
GATACAGAAACAGAGTGTCAGCAGTTAGCCACAACAGTTGCTCTTCTGCTCAATTGGTTGTCCTTTGATTTGA
ACAGTGCTACCTGACTTCTTGGCTCCAGCTGGTTGATTACCCATTTTTTTCTTGATCTCTCCCGCCATTGTTAAG
AAAGCCTGTTCCACGTTAATGGAGTCTTTTGCACTCGTCTCAAGGAAAGGGATACCTAGCTCATCTGCCAAAG
CCTTTCCCGTCTGTGTGTCCACAACCTTGTTTTCTACCAAATCGCATTTGTTTCCCACCAGAAGCTTGCAAACA
CTCTCATTTGCATATCTGTCAATTTCATTCAGCCATTGCTTGACATTGTTGAAGCTCTCCATCTCAGTTACATCG
TAAACAATAATGATCCCATGTGCTCCCCGATAATAACTGCTTGTTATAGTGCGGAACCGCTCCTGGCCTGCAG
TATCCCAAATTTGCAGCTTGATTGTCTTTCCATCCAGCTCCACCGTTCTAATTTTGAAATCAACCCCGATGGTG

CTAATGTAACTGTCCACGTACGAATCATCGGCGAATCTGAGAAGAAGACACGATTTGCCGACAGAAGAATC
GCCGATTAATAACAGCTTGAACAAGTAATCATATTCGTTGCTCATGTTTTCTTATTCCTTTTTTGGCTAATGGAG
AAAAAAGGTTTTTTCTTATGCGAGGATTTCT
>TCONS_00000972
GAGAGAGAGAGAGAGAGAGAGCAGCTGCCGGTTACTTTCCCCGTCTCTGGGGAACTCTCCAAAAATCAATCT
TCAGTTTGTTGATACCAAATCCCAGCATCAATTGGAAATCAGAGTCTCCGAAGAGATCTTGGATGTGATTGAG
GAGGGTTTTGGTTTTTTTAAATCCTCTAGATTCTTACCTTGTTTAAAATCCGTGCAAAAAGATTTTCTTTTGATG
CCATTTTGATGGCGAAACGACCCGAATCATCGGTTGGATACTCTGTCACTATACCACCTCCTCAGCAAGGCAC
TGCCCAGTCACCAATTTTTAGTCCTCCCCCATTCCCAACAACAACAACAGGTCCCATATTTCCTCCACCAATT
GTACAACCGAACTAGATTCCTTCTCCGTCCATCAAGACCCCCAATTTACCATCACCGGCTAATGGAGTTAGAA
CTGGAAGCCCT
>TCONS_00000973
GAGAGAGAGAGAGAGAGAGAGCAGCTGCCGGTTACTTTCCCCGTCTCTGGGGAACTCTCCAAAAATCAATCT
TCAGTTTGTTGATACCAAATCCCAGCATCAATTGGAAATCAGATAGATTTATCATCCCGGAGAAAAGACCTC
ATCTGGAGTCTCCGAAGAGATCTTGGATGTGATTGAGGAGGGTTTTGGTTTTTTTAAATCCTCTAGATTCTTAC
CTTGTTTAAAATCCGTGCAAAAAGATTTTCTTTTGATGCCATTTTGATGGCGAAACGACCCGAATCATCGGTTG
GATACTCTGTCACTATACCACCTCCTCAGCAAGGCACTGCCCAGTCACCAATTTTTAGTCCTCCCCCATTCCCA
ACAACAACAACAGGTCCCATATTTCCTCCACCAATTGTACAACCGAACTAGATTCCTTCTCCGTCCATCAAGA
CCCCCAATTTACCATCACCGGCTAATGGAGTTAGAACTGGAAGCCCT
>TCONS_00001055
GAGTGTAAAAGTGAGAATTCATGTATGCCCTGCTCTGCTGTCCATGGACTATTATTGTCATGGATTTATTTTTA
GTTTGTCTATGTACTTTGCTTCCACAATATCTTCTCCAGAAATGAAGTCAAGGAAATCACTGAAGCCTCCTTCT
TTGTAGGACAAGATATGTTGTTCATCGACAAGTGCTGGTGCAGGTCTAACTTTCTTGCCTAAAGCTAGACTGA
GAAGGCTGGCAATGGAAAGCCGCTTTTTCTCTGAGTTAACTGTTGCTCGGTGGAGAACACCTTTGTATCTACC
ATTGCTCAATACTTCCATGTGATCTCCCAGCTGAACAATGAGGGCTCCTTCTATGACTGGAACTGAATGCCAC
TTTCCATCATGATCCATGATTTGCAAGCCCTGATGATTTTGAAGAATGATAGATAGCAAAGCATAATCTGTGT
GTGGTGGCAAGCCTAGTGTAAGATCTGGCTCAGGACATGCTGGATAGCAATTAACGGCCATGACCTGGGAGC
CTTCTGCAATATCTTCATTACATGATTGCAGGTGACATGACACGAAAGATGTACCTGGAAGAAACCTAGTTCC
TTGCAAGCCAGGTGTGCTTCCTCGATTATCCGAGGCCGTTGAAGAGGGTGTCGCAGAAAGGATAGATCTACA
ATTGGCAGGCATGTGTTAGACCGGGTTCCATCGGGGCGTAAGGATGGCGGAAGAATGTAACGTTTGGGAAC
>TCONS_00001056
GAGTGTAAAAGTGAGAATTCATGTATGCCCTGCTCTGCTGTCCATGGACTATTATTGTCATGGATTTATTTTTA
GTTTGTCTATGTACTTTGCTTCCACAATATCTTCTCCAGAAATGAAGTCAAGGAAATCACTGAAGCCTCCTTCT
TTGTAGGACAAGATATGTTGTTCATCGACAAGTGCTGGTGCAGGTCTAACTTTCTTGCCTAAAGCTAGACTGA
GAAGGCTGGCAATGGAAAGCCGCTTTTTCTCTGAGTTAACTGTTGCTCGGTGGAGAACACCTTTGTATCTACC
ATTGCTCAATACTTCCATGTGATCTCCCAGCTGAACAATGAGGGCTCCTTCTATGACTGGAACTGAATGCCAC
TTTCCATCATGATCCATGATTTGCAAGCCCTGATGATTTTGAAGAATGATAGATAGCAAAGCATAATCTGTGT
GTGGTGGCAAGCCTAGTGTAAGATCTGGCTCAGGACATGCTGGATAGCAATTAACGGCCATGACCTGGGAGC
CTTCTGCAATATCTTCATAGAGAAAATATATCTATCAGAAATCATTTCAGATAAGGGTGCAAAACTTACTTGT
AACATGTGGGATTTGATGGCCACAAATCAATCCAATTTGAGATTGGATTAGCATAGTGCTTGAGGAAGTCTCT
CCAAAAGGATACTTTGTCTTTGACATCATTTAGGCTGGTATTATACCTTACGGGATCATGAACATTTGAAGAC
AAGAGATGCATTTTCTTTTCATTTGGTAAATCGAAGAACTCAGTCGCAGCTTCTAAGGCATCTTTCATGACTGA

CAATGGGATTCCATGGTTGACTACCTGCAGAGTCATTTATTGCATGCATGCTGTTATTTTACGTCCAGGTGACA
TGACACGAAAGATGTACCTGGAAGAAACCTAGTTCCTTGCAAGCCAGGTGTGCTTCCTCGATTATCCGAGGC
CGTTGAAGAGGGTGTCGCAGAAAGGATAGATCTACAATTGGCAGGCATGTGTTAGACCGGGTTCCATCGGGG
CGTAAGGATGGCGGAAGAATGTAACGTTTGGGAAC
>TCONS_00001063
CTAGAAGAAACACACCTTTCTGCAAAGATGATCCAATGTCAGCAACCAGCTGAAATTTCCAGCTTCATTACA
TAACATGTAACGAGCACGGCTGATGTCTGACCCTTCGTTCTCCATTCAGTTTTGCAAAAAGGAATCGTGATA
AGACTAGATATTATGCCTCGTCAAAATTTTCTGACCCACTACTGGATGCTTTCAGGGAATAAATGGGCATCCT
GGCCAGCTTCTATTTGTGATATCCGTGTTTCTTATGTTACCATGAATAAACACCTTCGGTTCCTTTACCTACATG
ATGCAAACAGGTTAAAGTAACAAGGATGCTGCCCAGAACATTACAACTATCATTTGACATCGTCTAAGATAT
CCTTACCAGTGATATTGGAGGAAAACCAATTCGTACACAAAGCCGAGAAAAACCATCCACGCTCATCAGAC
ATTTGAATCCTTTGATGTAAACATTCTCATTCAGAAGATATGTGAAATTTCTCATCTGAAAGTCTTCCAGATAA
ATATTTTCTTCTTTAGAGTACCTCCATGGATAATTATCTTCACAAAATCGGGATATTCCATTCATCGCTGAAAA
AGTGTCAATATGAACCCAGAGTTCATCCGAGTTCTTTGTTATACCACCTATCCTATGCAGAGGTTTTAGAGCAT
AACCACTAGGATAGTTTTCGTATGATGCCATGAAAAAAACTGCCGTGCAGGCTAGACTGCCAAGCATTAATC
CCAGCATAGCAATGTACAGAAACTTCCAGAAACTCTTTTTCCTGTTATTATACCTGCATAAAGTTGAGACTTG
AG
>TCONS_00001087
TGATCATAGTCATATCCTCCAACATCCTCATCATAATGTACATCCTCGCCAAATGCTTCTTCGTAAGTCTCATC
TACTTCCGGATCAATTTTGCTGCTCTTTACCATGTTATTATCCATTGAATCATCCACATTTCGACTTTTCTTTGGG
TTCTCAAGTTTTGCAGACTCATCAAAAAGCTTCTCTAGCACATCATAGTTGATCTTAGAGCTCAGCCTCTTTCT
AGTTAACATTTGCTTCGTTGCTTCTGCAGCAGTTTGAGCAGGACCAGAATTTTTCAGTTCTGCAGCCCTTTTCTG
TTGTTTTTCCTTTCTTGATTTTGCAACAGCAGCAGCAGCAGCAGCCGCAAGCTCCTGTGCAGCTTGCACATCCT
CCGAACAGTTAGCTAAATTGAACTCCTTATCGTTCTTTGCTGCTAATTCAGCCGCTTGCTTAGCCGCTTGTTCCT
CAAGATATTCTCGGTTCATCTTTACCCAAATGATCTCCTTAAAACGCCTCTCCTCCTCATTGTGAAGATAACCA
TCAACCTCTGCATCATCGATATCGGAAAAGTTCTCTGATTCGTCATGATCATCAGGACCCATACCATCCAAGC
CATGTAATGTATCAGGAGTCGCATTATCATCAGTTTTAGCCACTGATAGTGCCCCTATTATCTCAGACCCTGAT
ATCTGCAAATATTTACTTCCAGTCACACTCAGGGTGCTCCCTCCCTTTTCAGGCTCCAGATACTCGCCATCATT
GTTCTCCACCTGATTGGACATAGGAATATTTGGATCCTCAGCATTTTCTTCAGCAGCTTCTTTTGCCATTAATCT
TTCCCTCTCAGCGCGTTGGAAGGCTGGAGGTTCAGAACCTCCATCAAGCCCACCTGAAAGTTTTACAAAATCA
GTATAACAACTTTCACAAAGACCATGGGCAAAGGGCAGCTCAACGCTTTTCTTGTGTTCACATAGCACTTCTG
TGATCCCAGATCCTTTCGACGCCGAATAAAGTTGCACGGTCAGCCTCTCTTCTTTCTCAAGCTCCTCAGCCCTT
GTATTGAACTCATCAATCGTTAAGCTTCCAGATTCTGTGTTTTCGAATTCAATCAAACGCTTTGTCAATGTTGC
TTCACAAATGCGCACGACTTTGATAATTTCTGATTTAGTACAACTCAGTCCATATGAAAGAGCAGAGATGTAC
AGTGCAGCTCCACATACCCCACTCGGCTTTCTCCCTGTCTGCATCCAATCTCGCTTCATGCTGGCCACAATGTG
AAGTGCAGTTCTAGAAATATTTGGCTTCCTTCCCCCAAATAAACGATCAGTGAATCGATGAATGAAAAGGCT
AGGATCCACAGGTTTTTGAACAATTGGGTGATTTTCAAGGCTTAGAAGCTTGCAAAGCTGTAAGAACACAGC
CCCTAAAATATACACATTTATCCTCAAGTACTCAGAAAAGTCAATGAGTAGGAATGGCTTTTTGTTCTCCCTG
CATGCAATGTAAAGACACGCAGCCTCAACTTGCTCTTTCCTACGCCCCCTTGTGAAATTCCTCTCAACTGCTAT
CGTATAGAAGGCCAAAGCTGGACGAGCTATTGAATCACCACCATCAATTCCTAATGCATAAGCCATACCCTC
TATATCACTATATGCTTCGTTAAGTGTTCTTTCACGTGAAGCTGAATAATCACTTTTGACCGTCCTCACAAAGT
TCCCTGACAATTGGCTCTGTCCAGCAGCATTCTTAACAAAAGTGGGCTCACTTGAAAAATTATCTTCATCAAG

AACTCTTCCACAGAAGGAACAACATATTTACCATCCACATTGTTGGATTGCCTGTTCTCTGCACAATAACCA
CACCAAACCATGCTCGAAGTAACCTGAATCAAC
>TCONS_00001088
ATCCAGTAACTTTCGACCACATAAATTGTACCCACAGAAAAATGATACAACCTGCATCCAATCTCGCTTCATG
CTGGCCACAATGTGAAGTGCAGTTCTAGAAATATTTGGCTTCCTTCCCCCAAATAAACACATTTATCCTCAAG
TACTCAGAAAAGTCAATGAGTAGGAATGGCTTTTTGTTCTCCCTGCATGCAATGTAAAGACACGCAGCCTCAA
CTTGCTCTTTCCTACGCCCCCTTGTGAAATTCCTCTCAACTGCTATCGTATAGAAGGCCAAAGCTGGACGAGCT
ATTGAATCACCACCATCAATTCCTAATGCATAAGCCATACCCTCTATATCACTATATGCTTCGTTAAGTGTTCT
TTCACGTGAAGCTGAATAATCACTTTTGACCGTCCTCACAAAGTTCCCTGACAATTGGCTCTGTCCAGCAGCA
TTCTTAACAAAAGTGGGCTCACTTGAAAAATTATCTTCATCAAGAACTCTTCCACAGAAGGAACAACATATTT
TACCATCCACATTGTTGGATTGCCTGTTCTCTGCACAATAACCACACCAAACCATGCTCGAAGTAACCTGAAT
CAAC
>TCONS_00001306
CTTTTCTTACATCGGTGTTGTCAGGTCAGCTTGACTAATCCACCAGGCTCTTGTCCAGCCAACAAACACAAGTT
GCCAAGGTAATCTTGATCATCAAGGCTGGAGCTGGAGTAACACAAAATGTTGTAGTTGAGATTCGAACCTGG
GATCTCCAGTGAGCTGGATTATGACCTTGCTACTAAGGCCCGTCTAGCAGCGGCATTGATGACTCTGCCAATG
TAGCACCATCTAATAACCAGTTTGCTAATTGCCTTTTGGTTATAGATTATAAAGGAGTGAAATTGTTTTATTAG
TAGCCGAGCACAGCTGTCAGTTTATTCCATTTGTAAAATGTGAAGGCTTAAGGTGTTCACTCTCTACTGAAAG
GTTGGCTTGAGAATCACTAGTATTACGAGGAAATACCAGATGAGTAAAAGAACTTGCATAAGTTTTTCATATC
CAATAAGGTTTACATCAGGGATAGATCTCATGGTAGTCTTGGCGAGAGCTAAATTCTTCTATCCTAGTATTTGC
TTCTTATAAAGACAAGATATCCATTTGACTTTGCTGCAGGATTTTGCAGAAAAGATGATCATTCTCAAACAAT
TTTGGTGGATTAACTGCCAAGGGTTCGTCAGCTCCTCTTTGGAGCATTTCTCCACTGAGACATGTTCAGTGAAA
AATGTTCTAGTTGGGAGTCATCTGTTGATTTATGAACAGATCCTTTTAGTTGCAACTTGAAGTTGGAAAGTGAG
CTTATGCAGAAACTGACTGCATGTTGAAGTTTATGTTCGGCTGGATGAAA
>TCONS_00001315
CTATCATTGGTCCTCATTTTGTTATTTTCTTATCCTGATCTGTCAATTTGGTAAAGTTTACAATACATATATCTAC
AAAACAAATTCCTTTTGCAGAAGGATTACAAAAAGGATATATTGCAAATTAAATGTGAGCACTTTCTGGTCA
GCTCAGGAGAGTATTTTCCGCCATTTTGCAAGATAATCTTTTCCATCTCCTTCCGCTCATCTATAGGAATTCTG
GTGACACAAATTGTCAACCCAGAAAAAGGAAGGACTTTGAATGACTCCTGAGGAACAAGACGGTGTTCTTTC
CAGCATTGCTGAAGCCAATCAATAGTGACAATTGGTTTCTTTAGTTTGTTTACAGCCCACTTGTACTTTGCAGC
CAAAACATTCTTCACAATAACAAAGCTCACATCCAATGAGGCTTTAGTATGCAGGACCCCTCCCATAGCACT
CACCAACTTTCCAAGTTCAACCTTTTCATCCATCTCAAAACCAGATGCCAGGATCTTGACCCCATCCATTGCA
AGGCAACAAGTAAACCCCTGCTGGGGTAGTGGCCTCTGTTCTTTGGCACAAGAAAGCACGCACTGAGGACCT
ATCAAGTTGCAGCCCTTAGCTTGAAGATCCCCAAACTTCTCATGATGCGGAGAAGAAATGACGTGGTAATCA
GTGCAAGAATTTCGGGAGGGGTCGCAACAGAGAAAGACTAGAGCTCCGTTGTGCTTGAGCGCGTCGTGAAG
CGCGTCGAATTCCTCCGGCGGGACGAGGTTCCGCGACATGAACACACTCGCTCCATTGAACACCTTCGTCGTC
ATCATCTTCTTCTCTGCC
>TCONS_00001349
CATATTCAACGAATAATCACTTTCTCGCTCTAACTCGCGCATCATCTCTGTCGTCAACCCAAACCATTCATCAA
TCCAGGCAAAGTAATTACGATGACTTTCCAGGAAAAGAGCCCTTTCCCCCGCCATCAAAGCTTGCTCCAATTT
TCCCCCAAAGCCCCAGTATGGAACATCTATTGTCACCAGTTTGTATGCTGTCATAACGGGACTACATTGGTCC

TGAAAATACCAAGTAAGTCTCAAGTTAATAGAACTCTTCTTAATATAGGTCGGCGAGTAGAAGTCGAAATGC
ATATTATTGAAATCTTCAAACCCTGAAGC
>TCONS_00001509
GGAAGCATCATTTGAACATCCAGATCAGAGAGGACCTTTTTTGAATATCTTAATTCGTATAAGTATTTAGTTGT
CTGACAAAGCTAGAGAAGTTGTTGTGTTTGAAGAATCTGGGGAGTAAACTAGTGGAGAAGGCATGAGGATCC
CAAACAACAAAGCTTTGACCACCTCTGTTCCAAGAAACAACATGATCTGTGCTTGAATCATCCACCATTTCAT
ATGTCTTTGTCAGGAATGGTGGTGGACCGATGTCGTGTAGCCCCTCCTTTGGCTGTGGTGGTGTCATTACCGCC
GGCGGTGGCTCACCGCCGCCACCTCCGGAACTTGATCCGGG
>TCONS_00001543
AAAACATACAAGAGATCATGAAGCGAAATTTACAATGCCAAAGCACAACCATGTCAACGGACATTTTCCAT
ATTATCTAGATTCGCACATACCATTTAGACATGTAATATCGGATAGAATACCAGGTGAATCAGAACAACACA
TAACAGACGTAATCAAAGTGTATATCAAACCAGCACAAAAGAAGGCATGAAACTGTAGCCAACACACAAAT
TCTCCTAATAATTCTTCAAGCTGCAATATCTTTTAATCTTCTTTGCCCCCCTTCTAGATCTTGTCAAGCTTCTCTG
CCTTCACAACAGGGTTGGCCTTTGCTATTGCATCTTGGGCAGCAGAGCGGTAATCAAACTGGCAGTCATGTTT
ATCCGAGTAACGGTGTGCTCCACAATACAGGTGACCGCAGCGACACTTGAATCCAGTGAAGCCAACCCTTTT
TTTACAAGTGCCGCAGCGGTTAGGACCCTCCTTAGCCTTTACTTCAGAAACAACACTGGAGCTTGAGGTTGGA
GATGATGGCAAAGCTACAGCCTTTGCTTCCACAGAATCAGATTGCACATCGACTGAGCCAACAACAATCGTT
TCCCCCTTCTCACTGGCGGTTGATCCATTGACAAGGTTTTCAATCGATGATGCAGCTAACTTAGCTTGTTCTTG
TTTCAATACCGTGTCCCTGTAACACTTAGAGCACATGTTCATGTTTGCAGCACTTCCAAAGAAGCCACAGTTG
TTAACGCACAAAATAGGGCCTTCAGGAGGGGGTTGGCATCCCGTCTCATCATGCTCCATATTGTCAACACTCC
CAATTCTTGAGGTAATCCAACAGATCCACAAACACAGCAAAAAGAATTGTAATACTAAATATTAATACAAAT
CATAAAATAAAATACAAAAAGAGATAAGAACAAAAGGGGTTTTAGAAATTAACCACGTAAGAGATTAAGAT
CGCCGCAAGAGAGAGGA
>TCONS_00001544
AAAACATACAAGAGATCATGAAGCGAAATTTACAATGCCAAAGCACAACCATGTCAACGGACATTTTCCAT
ATTATCTAGATTCGCACATACCATTTAGACATGTAATATCGGATAGAATACCAGGTGAATCAGAACAACACA
TAACAGACGTAATCAAAGTGTATATCAAACCAGCACAAAAGAAGGCATGAAACTGTAGCCAACACACAAAT
TCTCCTAATAATTCTTCAAGCTGCAATATCTTTTAATCTTCTTTGCCCCCCTTCTAGATCTTGTCAAGCTTCTCTG
CCTTCACAACAGGGTTGGCCTTTGCTATTGCATCTTGGGCAGCAGAGCGGTAATCAAACTGGCAGTCATGTTT
ATCCGAGTAACGGTGTGCTCCACAATACAGGTGACCGCAGCGACACTTGAATCCAGTGAAGCCAACCCTTTT
TTTACAAGTGCCGCAGCGGTTAGGACCCTCCTTAGCCTTTACTTCAGAAACAACACTGGAGCTTGAGGTTGGA
GATGATGGCAAAGCTACAGCCTTTGCTTCCACAGAATCAGATTGCACATCGACTGAGCCAACAACAATCGTT
TCCCCCTTCTCACTGGCGGTTGATCCATTGACAAGGTTTTCAATCGATGATGCAGCTAACTTAGCTTGTTCTTG
TTTCAATACCGTGTCCCTGTAACACTTAGAGCACATGTTCATGTTTGCAGCACTTCCAAAGAAGCCACAGTTG
TTAACGCACAAAATAGGGCCTTCAGGAGGGGGTTGGCATCCCGTCTCATCATGCTCCATATTGTCAACACTCC
ACGTAAGAGATTAAGATCGCCGCAAGAGAGAGGA
>TCONS_00001587
ACTACAGATTTATCAATGAAGGTTGAACGAGTAGGTAAGGGCTTTGAAGCCATTGTCTTTCTTCGTTTTAACTC
TCTAAACGACGAAGTGCATGAAAACAGAATCTCCCGAGTAAGCGGAACCTCTGCCGGAAAACTGAAACAGC
AATCTCCACAGCAAACTCGAACTCGGCTGATTTAAACTGAGACCTCGATGACAGAGGTGGCTTGAAATGGCA
AAAAAAGTAAACTCCA
>TCONS_00001596

CTTTGACATTAGAGCAACCAACTGAACCCACTTGAGATTGAGAAGAAAAGCAGTCGCAAATAGAACACTGC
AGTGTATATAACCCCTCTTTGTTTACGGAACAGATTAGAAACTCGAAGATTTGCACCAAGAAAACTATCAAA
AAAGGAAAACATAGTCCCACTGGCCTGTAAATCAACATTAAAATATCTTCAGCACCATCCTCATCATTTATTT
CAATTGAAAACGGAATAAATAAGCCTATAACCAAAGACGAGCCAAAAGGCTACAACAAACACGATCATCAT
TCATTCTAACATGAAGGATCTGCATTACAGTTGTTTCTTGGAATAAGTTACATGAAAATGTTATGCCTGATAA
GATTTTAAAATGATAATACTATCAACTTCAATTTTTATGATTCATGAAAACAGAGAACGACTTCGATTGCGCT
CCAGCCGAGCCTCCCTCTCCTTTTTCTTGCATTCCTTGTAAACATCAAAATGTTCTTGACATTTGCTCTTGTCTG
AGTTAAACTGTTCCAAACATTTGAGAGAAGCAGTGTACTGAGGGTAGCAATCGGAATCAGAAATTCTTGCTG
CACTTGGATATGCCGGTGTTTGGATTTTCGAACTCGCCATTTTGCCTT
>TCONS_00001712
GCAGTAGGTTGCATTTCAAGTGTGCCCAATTCGTTGTTCACAACCTCAGCTACCTTTCCATTGTCACTGGAGCC
TTTGGAGAAAGATGCAACTTTCTCTGAAGTAGTGTTACAATTAATAACCACTGATGGAGGAACAACTAAAGA
AGCATTAGTGAACAACTCTCTGTGACAATCTTTCAGCCCAATTTTCTTCACACTTGAAGTTGCATTGTCACCTT
TAGTGCTCTCAGTTGCCTCCCCCAAGAGATTTGTTTCCTTGACTTATTAGAAGATCTCTTTTTGAAGCCAACGT
CTCCATCAACCTTCC
>TCONS_00001764
AAGTAGCATTAAAAGACCAAGCAGAAACTCATTTGAATTCTTGCTTATCCCATTTCATTCAAACTATGGAAAC
CAATTGTATAGTAGTGCTAACATTGTATTGTCTGGAGTATTCTCAGAAGCAACAGAATGCAAATACAACTCAT
TGAACTTCAGCAACTTTCTTCACCTAAAAATAAAGTCATTTCTTCATTTACTTCTCGGATGAGTTCGGGCCCCT
CTTCTTCCCGAAACCAACAACTGCTGTGACGAATCTGCGGTTGTATTGCATCCTCTTGTGAGCACGGCCGCGG
GGCTTCTTCTTCTTGTCCTGTTTGGCAACCTTTGGAGTTTGCCCCCTCACTTTACCAGCACGAGCAAGTGATCC
GTGAACTTTCCCCATAGTGATATCTGAGTCGCCGTTTGCCGGTGAAGTTAGGAGCGTCTGGAGCTGCTCAGGT
CAGGACTCTTAG
>TCONS_00001807
GAGTCACGTCAACAACGGCAAGACAGAGAAACATAATAACCATGAGTAGAAAGCAATACGCAATAGCAAA
AACTTCTAGTACTTTTGAGGTGGCTTTTGGGTTGGAAGCAAACCAAATCGCTTCTTCAGAAGCACTCTTTGCCT
TGAATATTTATCATCGGGGGAAAAGCGGGCTGGGTGAGCGGATTCTGTGGCCAAACCCAGTGGTGACTCTTT
CTTAGTGGTGTAAACTTTGTCACCATTCTCATTGATGTAAAACTGGAGATACATCCTCGCTAAATTTTCACCTG
AGCTGCTTCGCCGGTTTATGGAGCTAA
>TCONS_00001836
TAGAAAGGTAGACATCCTTTCTGACTTAGGCAGAGTAACTTAAATGCCGTAGAAATAGTTAAATGAGATGAA
GGCATAGGTTATATACTACAGTGGCCTGCAAAAATGTGTATGAAAGTTGATAGATGAGATAGTATTTTAACTT
GTAAGGCGAAGGCATAGGTTATATACTACAATGGCTACTTGTACAGTTCTTCTCAACGTATGAACTTGTGAAG
TCAAAAAAAAA
>TCONS_00001884
CTTTTTTCCCTCTTCCGATTAGTCTATCTTTTTAGAAATTTGTTCCAATTGAGTTGTTATGTGGACCTACGCAATA
TAGTTGAAACACTACTAATCCAATGTTGTTGATGTCATTTATCGACTATAGGCTTCTCAGTCAAAATAACTTCT
TTATAACACTCTAGACACAACATCCTCCATCAAGCATCTCAAGCTTAATCGTATTTACATCAACCGGGCGAAA
AAAGCGGCTTGGTGAACCAGTAGAGACTGCCTGCAATGCTGCCCAAAGTAAACCACCCTTCCTCTGGTCGGG
ACCTTGTTCACGGCTCAGTGCACTCGACATATTTTCTGATTGAAAAGGAACGGGACTGAATACAATTCCCTGC
AATTACTAACAGAACCACAGTTAATACGGACCATGAC
>TCONS_00001946

CATGACAAATAACAGAGCAATACAATCTTTGAAAAACAAATGGTTTCAGCAAGTCTTAGGAGGGAGGGGGT
GAGGTCTGCATTTCAACTACCTTCTTCCACGATTCTCTGCTAGATATCTCGTCCCACCACCTTCCCACGTTCTTT
ATTGAAGTGAAGAACTCTGCTCTGTCTGTCCCGTTCACCAAATATTGGATGTTTGGAAGGTGAGAGAGATCGG
CCAATGTGAACTCATCTCCGGCCAAGTACTGACTCTCACCGAGCCTCTTTTCATACACATCAAGCACTTTTTTG
AGCTTCTCTTCGTTCTGTCTGATCAGGTTCTCGTCTTGTTTGATCTTCATTCGCGGTGCAAAAGCCAACTGGAAT
ACGAGAATTGAACTTGGCGGATTGAAGCTTTGTCCTTCTGCTTCTATCCATTGATCTATAGATGCTTTCTCTAA
CGGGTTTGTTCCGTACAGCCCCTTGTTCCCTTGATGTCCATATTTGTCACATATGTACCTATTTATAGATCTGGA
TTCTGAATTTTGAGATAGTCAGGTTTCTTATGTTCCCCTTTGGCCATATTAACAGGGAGAAGCTGAAAGTGGA
CATCTTTTTCAAGAAGACAAGCTAGAACTCTTGAGACTGCAGTTGACAAAGTCGGTCCATATACTTTCACTGG
AGTAGCCA
>TCONS_00001969
GTGGTCCAGCCTATTCTTGAAATAAACAACTCGCACACATCCCTTTCCAAAAAAAATCAATACACCAAGCAC
TACACTTAGATTTATTGGATTTGTTGCTAAAATATCGGTATTAAACCCGAAACTCCCGGCAGATGGCCAGTGG
CCCAAAGAAACGAAAGAATCGGTTACGTTTTTCATATGATCTCCTCTTATAGATAGACTAAAAAATCGAACA
GAGTTCTTTTTGTAGCACTTCGCCCCTCTTTTTATTTATTCTTTT
>TCONS_00001978
ATGATAAAAGAATCAAGAAAAAGGATTAAAAAAGGACTTAACTTTTTAATCCTTTTTTTTTCCCTTTTAAACA
TGAGGCTTGCTTCTCTTGTGATTAGCTTTTCCATCTGATCTTAAAACACCACTTGAATCTCCTTCAGATGAAGC
ATCAACTGATTTTGTGAAATTACAAGAAAGATCAGCATAAAGATCAACAACTCTTCTGATATTATTGTTAAGC
TCTCTGATTAAACCAACATTTCTGCTCAAATTATCAGGAATCTTGGACTCATGATTCTGATTAATCTCATTGAT
TAGTAACCTATTTTGATCCAATATGTTCTGCACTTGTACAAAACTCTTCTGAAATGTCTGAAAAACTTTTCCAT
CTACTTGAGTACCATTACCTAGGCCTGAAAATGTATCTCCCTCCATAATTCCTTTTGTTTTTTTTTGGTCCTTAGT
TGGTGAAGAACAATCCTCTTTTCAATTCAAAACAGGTTCTTGAAAATCTGAGAACATTATAATTAAGGAAGA
GAGGCAGTAGACATAGATTGGAAAAGCCGAAATGATCGGCGATTGAATC
>TCONS_00001979
ACTGCATATCAATTCTAACTGTATAATGTTTTTTCTCGTAGGAAGGATCAGTTGGACACAATGGTCGAATACA
ACAGAGTGAAGGTCAAGTGTTTGATTGAATTTACTAGAGGGTTTTTGCGGGGAATTAACCGCAAATACTAGTT
TATCTCATTATTGTTGATAAAATGGCCTAGTCCTTCACATCTACCGCGCATTGTGCTCCAAAAGGTACTAAGA
GAGGTTAGCCTATACCACAATATCTCAATTGGGTTATATGATGTTAGCTCTAGCAGTTCTAGTTAGTAATATAT
TACTTGAATTTCATGCACAA
>TCONS_00002101
CGAGTTCAGTAAATCGAGGCTCTATGTTGTCCTGGATGAAGAGAGTGTTCACAACTTCTTTCCGACTCATGTAT
TGCCGTAGACACTGCTCTATCCGATTTTGCACCATTTGTATATCTTCACAAGAGACTTTTCTTCCATCTCCGCTC
GACATTTTTGAGGCTCCAACACTTGGAACTATTGCAATAAAGCTCTATGCCAATATTTCAGTAGAAGTGTACA
CCCAGCTTATGCGTACCATCAATGAGTATATTGTAAAAGGTGGTCACTTACACCTATTGTCCAGC
>TCONS_00002140
ATTTGCTGTTGCAGAATGAAAATATTCGAATGAAGTGCCGAATAAAGCGGAGTGGATACAGAGAATTGCAAC
TAGGTCGGGATTGAGGTGTAGTTGAATGATTGATTGATCATTGTCCAATTGTTTCTTCAAATTATTTCACAATG
ATGAAAAGGTTAGGATCAATCCGGAGCTATGTCAATCACAGCATACATCCTTCTTTCAACAATGTACAGTCA
AGTAATGATCATATAATCTTCTTATCATCAGAGGAAGAAAAAATTCACAGGCAACATACTTTCCATGGCTGA
AAGAAGGTTCCTCTTGGAAATAGCTGTTTCTTCTAGTATAGAAGTCACTGTGGAGGAAAACTGAGTGCTGATC
TTTCCATTTGATTCCATGGCACTCCTGAAGATATGAAGGTAAAAAAGGCCATTGAGTATAGCATTAACATCGG

CAGAAATCTTTGCAACAAGCTGCACTAGGAATCAGGAAAAATTGAGTGATGAAAGGGTGTAAATGAAGTAA
CACCAACTGAAGGAAGGATCTTTACTTACATCCATAAAGGAATAAGAATGCTTTGATACTTCTGAATTCACTT
CGCTAAAAGCTAGCTGAGAATTTTCATTAAGTTCTTGGGCCGAAACAGCTAAATTCTGAATATCAGAGCTATA
CTTGTCTTTCAGCTTCTGGAGCTGAGTCTTTAACTCATCTGTAGCCTGCCGTTAAATTCAAGAAAGATTGGTAC
TGTTTGCAGGTTGCATATAATGAAAGTACCATATGTCAAAGATTTTTTACTGTACCCTCCTTTTAGTAGCTAAG
AAGGATTGTGTATCTTCTAATATTACATTCAATTGCTGCTCTTGTTTGGTTACAGAAACTGCAGTGTTTCTCTTT
AAACTTTCAAGTTGCTGAGTTAATTTGTTGCGGAAATTTTGCACGAGAATTTTGTTTCTCTCCTCCCTATTGTTG
CTTTTCTCTATTTTATAAGAAACATATGTTAGAAGAAGAAAGATGCTAGCCAGATAAAGATCATAAGAAATA
GTAAGACGAACCAATTTTGGCGAACAAGGTGGATATCTCTGATTCTGCATTCTCTACTTCAGCTCGAAGCTCA
AGTGCTTTGTCAGTCATTGCCTTTTCTA
>TCONS_00002156
CTGCACTATCCGTTTGAGGACATTCTGGTAAATTGACGCTCCCCGCGCACGTCTCCCGGTGTTCGAATAAATA
TTGTTTCCCTGGAAAATCAAATGGTAGTTTCCACTTTCCACCACCGTCCCCTTCCTCAGTTTTCAATTGCTTTCG
CCGATGAATTACGAGCAGAGACTAATCGCCGCATCGAAGTTCGTCTACGCCGGCGACTCAGCCGACGATTCT
CTGCCGGTCAACACTGTTGACTTGGGAGTTAAAGCGACTCTTAAACCTCACCAAATTGAAGGAGTCTCATGGC
TTATTCGAAGATACCGTCTAGGCGTCAACGTCATTCTCGGTTCCATGGAGTTATGCAATAATTGACGAAGCAC
AAAGGCTCAAAAATCCATCTAGTGTATGTCTGGAATTCTTCTTTTACATAAAATGCAACTGAGATATCACACC
TGATCTTGGTATGAGATAGAATATTGACTACTTATGTAGTCACGATGTGTGCTCATGGTCTGCTGAAGTTAATT
CCGTATACTACCTTTAAATTTGTATTAACTAAATGCCTCTTTGAGGGTAAATAGGTGATATCTCTTCAATATCC
TTATTACTTCGAAAATAAAGTTCTTCCAAATTTCCTTGAAATGC
>TCONS_00002232
TATATATATATTTCACTAGAGGGATAAGAAAACTTTGTTATTGTGCAAGATATAATCATAGAGCAAATATTAT
GTGTAGCCATAATACATTTATATTTGTCCAAGTTCAGTGACTTTTAAGACAATTGTTAGTTATCTTCTCACCCG
CACATGTACTCCTACGTATCTCCGATACTTAAGAATTATACGATGACTAATCCAACTTCTCGACCTGCTCAGG
TACCCATACCCTTTTCCATGTAAAACAGTCTGGAAATATCTAACAAAAGAGCAAATAGTCAACTTATCATACC
TGTTGCTTCCTGCAGGGCCTTTTGCAGTGCTTCCAGTTCAATTAATCTGTCTTCCACTTCCTGAGTTTTGGTCAT
AGAAAACCGCAGCTTTCCCACTTCGACTTGCAGATGGTCAAAAAACTCTTTGTTCAATC
>TCONS_00002321
CCATCACATTAAATCAACTGTCATCTGCACTTGGCTGCTCTTGTTCTAATTTCTCTACCGCATTCTGAGGTTCT
TGATTAATAGGGGAATATTCAAAGAAGAATCCACAGGACATGGCGAAATAGGTTATGTCCAAACACCTCTTT
CTCGTTTGTTGAGGAAAGATGGAGAAAATAGCATGGCTGCTCTTGTCCTACTTGAAGCTAGCCCAGTGATGCT
CGCGCCATGGCATTTTTTGAGTGCCCGCGCTCTGGCAAAAGGGAATACTGCAGCATTTAGTGCAGTTCATGG
>TCONS_00002405
GGGAATCTTGATCTCCCTATACAAATTAGGAATTTCAATTAGCTTAACCCTAACCCGAAAAATAAGATCCCTA
ATTTTGTTGGATTTGGATACAGAGGGAGAAATAGAGACATTCAAATGTGAAATTAGTCCATCGCAAGGAGAC
ATTCAAATTTGAAGATGATTTTTCCGATCGTTTGTTCTCATCTCTTTCGCATCGCTTCTGAAGACGACGACATCT
ACGCCAGGTACGAATAGTGAGATTGTGGCACGTTCCCGACCGCGACAAACCAGACAACACCGTCTCCCTTGA
ATTAATTATTCAAGACGAAAAGGTGCATTCTTTTTGAAAAAAACTTATTTTTT
>TCONS_00002454
TCAATAAAGCTGACCGCCATTGCCATTACGCCATCACTCCAACTTGATCACTCGGGAACTTGTGATTCCAAAT
GCTTCCTCCATTTCTTCACTTATAGATCTTCTTCTACTTCCCATTTGCTAAATTTCTTCTTACTTTTCAAGATTCTA
CTAGGAATTTTCATTTTCCTTTTGGTGTTTGGTTACTTCTATGATCTGAGCTTCTCTCACACTCTTGTCTTGACCC

TTTAATCCTGCAATATCCATTATCATCTGTGTCCAGAAGTGTAATCAACTGATATGGATACTTGCTGTATGGCT
TTGAAATCAACTGCCCATTTGAGGAGAGCGAGCAAAGTTGGCTTTGAAAATGGTGACAAGGAGTTTTTGGGG
GAGCAGATTAGAGGGAGTTTAAACAACAATAATCTCAGGGTTAATAATTTGTCGAAAAGTTTGAAACTTGAG
AAGAAGGAAAGCAAGATCAAACCTGGGGTTGCTTTCTCTGTTATCACTACAGAAAATGACAAAGAGACTTTG
GTAAAAATCATTTCTTTTTATTCTTTGGGACATG
>TCONS_00002543
GCAAACACCAATTCAAAGGGGAGAACATGCTTCATTCTGAACGGAAATTTATCCAAAGAAGAAAAGGAAGT
GGCTATGTTTACCATCGAGGAGCTGAAAGATACAAAAATCTTCATGTTTGGCCTTAGTTCCATATGAAGCATA
TAAAAATCATCTTCTACATGTTTCAGCAGGTTGAGAGGGCTGAGCCATTGAAAGTAGCTTGTATCGGTGTCCT
AGATAGAATGCTGTCAGTATCCACATGAAGCAGACAGGTAGGGCATATAGAGAAACAGCTGATGCCTTGCC
ATTGAGAGTGCTGAAGAGTAGCTTGTACATTCCAGCTGCTGTAGCATCTCCAAGTCTTTGAACAATCACATCT
ATGCATACCTTTGCTTTATACTTCTCATCCTGTGAGACAACAGTAAAAAGAAGCTCCCTTCCAGGCCTGGTAA
CAACCTACTTATCTTTTTTTCATTGCAAAACCTGTTAGTTCCCTGTTAAACTAAAAAGGTAGAATACCTCATTA
CCTCCCTGTACTTGGCACGGTTTATTTAGCGCACACGGCACACCTAAACTTCACATATTTCCAGTTACCC
>TCONS_00002564
TGAGTTGCAACAAGTGCCTCTTCCTGTGTTACCCACCTACCTAAAACATCTGTATAATAGAAGCCAGAGTTAG
GATCATAGTGACATCCATTACTTTGATTGTAATAATAGCCTGATGAGCTGTCATACTCCCACTCTTCAGATACA
GCTGATCCCTTGGCAGTCGCTTGACCATTATCATGAGTAACCAATGCCTGTATGTTGGTACTTCTGGCCTCTTT
AACTCGAGCCATATCCTTCTGATAGCTGCATTGGGCTTTTGCTTCAATTTGCTCCAGTGCACGGGCTGCTTCTTT
TAAATCTTTATCTTTTGCAGCTTTCTCTTGTCGCATCGTGTTGAGCCTATTGGCAACATTTTCCTTGTGACGTGTC
CCAAGGTCATGATTCCTAATGCTCGTAGGATTATTTGATATGAAGATTTTGCAGAAATCGCACCATTTATTTCC
TTGGCTAACCCAAAACTCAGTCATGGCTGCAACGGATATACAAATGGATTGAATGCCAAGAGAACGGGAAG
GAACGGAGATTCCCAGGCTCAAATTTGTTTCCCTATTCAAAATCAACGGCCTTTTCTTCTAAACCCTACTCTTG
AACAAAAGCGGTAA
>TCONS_00002565
TGAGTTGCAACAAGTGCCTCTTCCTGTGTTACCCACCTACCTAAAACATCTGTATAATAGAAGCCAGAGTTAG
GATCATAGTGACATCCATTACTTTGATTGTAATAATAGCCTGATGAGCTGTCATACTCCCACTCTTCAGATACA
GCTGATCCCTTGGCAGTCGCTTGACCATTATCATGAGTAACCAATGCCTGTATGTTGGTACTTCTGGCCTCTTT
AACTCGAGCCATATCCTTCTGATAGCTGCATTGGGCTTTTGCTTCAATTTGCTCCAGTGCACGGGCTGCTTCTTT
TAAATCTTTATCTTTTGCAGCTTTCTCTTGTCGCATCGTGTTGAGCCTATTGGCAACATTTTCCTTGTGACGTGTC
CCAAGGTCATGATTCCTAATGCTCGTAGGATTATTTGATATGAAGATTTTGCAGAAATCGCACCATTTATTTCC
TTGGCTAACCCAAAACTCAGTCATGGCTGCAACGGATATACAAATGGATTGAATGCCAAGAGAACG
>TCONS_00002572
TTTCACTCCTCTCATTTTGTACTAACGGTGGTTTCGTCACAGGTTTAAGATCTGCTGCAAAGCATCCCATATCTT
CAGACATTCTTCACCTACAGAGTAGGATATAGACAGCTCACTGACTGTTGAAGACTCCGCCTAGTCACCAGC
AATTGTTCTCCAGATTTTGGTTGATTATATACTTTCATACCATTAGCAAGAAGCAAAAGTGGCAT
>TCONS_00002577
ATTAGGCCTTGGACTGATTAAGGAAAGAGACCAACTTTTTCAGCACGACCATAGCATTTTCACTCGTAGGAGC
AAACAAATGAAACACATGTTCTTCATCCTTAATCTCCACAACCTCTACTGTCCCTTGCCACCCACTCTTCTCTA
GTAACTCCTTATAGTACAATCCCCTGTATTTCAACGGATCTTTTCCAGCAACATAAACAAGTACTTTATCACAC
CCTAAGCTAGACAATTTCGGATCCTTTTCCGGGTTCATCAACGGGTCATCTAATCCGGAGCTATTTGGATGCG
CAAAAGCCCAGAGCTTCTCAATGTAATTTTTGCAATCCAAGTTTTCAGCCTCGCCGTCAATTAAATCAATCCC

CCAGAAATACGGACAGGCTAAAAAAATCCCATCAAGTTTGACGCCATCCAACTTTTCCAACCCGACCCGAAT
TGCTATATTATGAGCAATATTAGCTCCAGCGCTATCTCCACCAAAATAAACGTGATTAAAATCAGCGTGGTCT
TTTAGCCATGGCTCATGACCTTCACCTTTAGTATGTGAAGCGACCCATTTGAGAGCTACCCAGGAATCTTCAT
AAGCTATGGGTAATGGGTATTCAGGAGCTAACCTGTAGTTAACAGAAACGATTATAACATTGGCTTTAGTTGC
TACCAAATTAAGATGCTTGTGGTATGAAGGTGAAGAAGCAGATTCAATCACAAAGCCGCCGCCATGAAAGT
AGACGAAAAGGGGAATTTTTTGAACCGGGTCGAGATTTTTGGGCAGGATCCAATTCAGCTGGGACAACGCCT
TCGCCCATCAACCTCTCGATTCGGCCATCTTTGTAAACTCTGATCAATGGGAAAAGGTCATGTACTATTTCCGC
CATTGCTGAAAGCTAAGTTGC
>TCONS_00002625
AATGCAAACCACGAATCAAGAACTCAGCCAAGGGAACACTTTGGGAGATGTCGGCAGGCGTGCTGTGAGTA
CTTCAACTCCAGTCTCTGTGACCAGGAGAGTGTGTTCAAACTGAGCACTTCGTTTCCCATCTGCTGTCACAGC
AGTCCATCCATCAGGCCACATGCGATCACGCCAAACACCAGCATTAATCATAGGTTCAATTGTAAAAGTTTG
GCCAGCTTTCATAACGCCAACTGCTTTGTTTCTTGAATAATGTGGAATGTTTGGTGCGCAATGGAAAAGTTCTC
CAATGCCATGACCACAATATGACTTCACCACAGATAAGCCAGACATTGAAGCATGTCGATTGATGACCTCCC
CAATTTCCCGAAACCGTACCCCAGGCTTAACAATTGATATTGCTTTCTCCAAACACTCATATGTGCACTGGAC
TAACCGTTGAGATGCTTCATCAACATTACCAACAAAGAAGGTTTCATTCAGATCACCATGAACCCCTTTGTAA
TATACAGTAACATCAACATTTACAATATCACCATCCTCTAATTTCCTTGCATCTGGAATCCCATGGCAGATAA
CTTCATTGACTGACGTGCAGCATGACTTGGGGAAAAAGTGATAATTCAACGGAGATGGATATGCACCAGCGG
CAACAGTAGCCTCATGCACCACCGCATCAATTTCATCAGTTGTCACGCCAGGCCGAATAATACGAGCCGCTG
CATCCAGAACTTCCCTTGCTATTCGGCATGTTTCTCTCATCCTCGCAATCAGCTCTGGAGTTTTGATCTCAACG
ACATGCTGTAAATCACTATTGGGTTCAATTTTTGGGATACCATCATTTGCCCAATCAGGTTGATCGATATGATC
AGGTACAAGACGCTTTTTTGATATCGGATAGGGCCTTAGCGTCCTGAAAGAAGGCGGCACCATAGGTTTTTAG
ACAACAAACATTAGCAGCAACTTTGTAGGAACATAAGAGAAAA
>TCONS_00002626
AATGCAAACCACGAATCAAGAACTCAGCCAAGGGAACACTTTGGGAGATGTCGGCAGGCGTGCTGTGAGTA
CTTCAACTCCAGTCTCTGTGACCAGGAGAGTGTGTTCAAACTGAGCACTTCGTTTCCCATCTGCTGTCACAGC
AGTCCATCCATCAGGCCACATGCGATCACGCCAAACACCAGCATTAATCATAGGTTCAATTGTAAAAGTTTG
GCCAGCTTTCATAACGCCAACTGCTTTGTTTCTTGAATAATGTGGAATGTTTGGTGCGCAATGGAAAAGTTCTC
CAATGCCATGACCACAATATGACTTCACCACAGATAAGCCAGACATTGAAGCATGTCGATTGATGACCTCCC
CAATTTCCCGAAACCGTACCCCAGGCTTAACAATTGATATTGCTTTCTCCAAACACTCATATGTGCACTGGAC
TAACCGTTGAGATGCTTCATCAACATTACCAACAAAGAAGGTTTCATTCAGATCACCATGAACCCCTTTGTAA
TATACAGTAACATCAACATTTACAATATCACCATCCTCTAATTTCCTTGCATCTGGAATCCCATGGCAGATAA
CTTCATTGACTGACCTACGTGCAGCATGACTTGGGGAAAAAGTGATAATTCAACGGAGATGGATATGCACCA
GCGGCAACAGTAGCCTCATGCACCACCGCATCAATTTCATCAGTTGTCACGCCAGGCCGAATAATACGAGCC
GCTGCATCCAGAACTTCCCTTGCTATTCGGCATGTTTCTCTCATCCTCGCAATCAGCTCTGGAGTTTTGATCTCA
ACGACATGCTGTAAATCACTATTGGGTTCAATTTTTGGGATACCATCATTTGCCCAATCAGGTTGATCGATATG
ATCAGGTACAAGACGCTTTTTTGATATCGGATAGGGCCTTAGCGTCCTGAAAGAAGGCGGCACCATAGGTTTT
TAGACAACAAACATTAGCAGCAACTTTGTAGGAACATAAGAGAAAA
>TCONS_00002765
CATCACAGCCGACACAATCCAAAAAGCGTCCTGGACGTGAGTGTCCGCAACATTGGACAGTTGATGCAATAG
CCACGATTCTGTTTTAAGACAACTGAGTTAAATGCACGAGCATATGTTTATAGTTCTATTGGAAAGAAGTGGA

GTTCAGGAAGGCAAAGGTTGTGGTATGAGTTCAATGACCCACTTAAGACCAAAGCAGAAATTATGGATAATG
TTCCATCAG
>TCONS_00002769
CGCCCACCTTCTTCCGAATATCTTCATTCCTATTCTTGTCTATCCTAGTGTGCCCGCACATCCACCTCAGCATCT
GCATCTCTGCTACTTTCATCCTCTGGATATGGGAGTTCTTAACCGGCCAACACTCTGCCCCATACAACATGGC
CGGTCTAACCACAACTCTATAAAACTTACCTTTGAGTATCAGTGGCACTTTCTTGTCACACAAGACTCCAGAT
GCAAGCCTCCATTTCAT
>TCONS_00002934
TAGGTGCAATTATTCCACCTTTGACATAATAAGTGGAGCACATAGCTAGAATTATCTCTGCAGCACCCTTCCA
ATGTCTTACGGACCATTGCATGATCCAGCATCATTCTTCTCATAGAATATGCCAAAGTTAGCGTAACAGCCAA
TGGCAGACCTTCTGGAATTGCAACCACTATGATGGTTACTGCTGCAGCAATCATACGAACCAAAGAATTCAT
GATATCACCTGCTTTGGTTTTGCTTCCTATGAACTCCCGACGACTATTGTCATATT
>TCONS_00002935
TAGGTGCAATTATTCCACCTTTGACATAATAAGTGGAGCACATAGCTAGAATTATCTCTGCAGCACCCTTCCA
ATGTATAGCTTACGGACCATTGCATGATCCAGCATCATTCTTCTCATAGAATATGCCAAAGTTAGCGTAACAG
CCAATGGCAGACCTTCTGGAATTGCAACCACTATGATGGTTACTGCTGCAGCAATCATACGAACCAAAGAAT
TCATGATATCACCTGCTTTGGTTTTGCTTCCTATGAACTCCCGACGACTATTGTCATATT
>TCONS_00002988
GGCTGCTTCAACGAATTTAACGTCTTATTTATTGGTGTCCGTCAACAGGTCTCGTCATATTCAGCCCTAGCTCG
TGGAGCTTTAATCCACAGGGACCAAAAGGGAGGGACCTCGAAACATCTTTCTTTGCCGTTTTAGTCCTCAACC
TTTTTGAAATCTTAACCCCAGATTCCCTGTCAATACTGTTTCTTGAATTCTTTCCCTCCTCTTCAAAAAGATTCC
AATTTTTCCTGTTACTTTAGATCCCTAATTCCTTCACTCAGATTAACACCCACCATATCTTTTATTTATTTATTTA
TTTATTTTTGTTCGCATTCGGCTGCTGTAATTGAACCAAGCAGTCACTTGTGAAGTTTCTGCAAAATTAACGTTT
AGGGTCTTTTTTGAGTTTCTAAGTTGGAGAGGGGAAAATAAATAAGGAAGTTGAATGTGTATGCAAGAAATT
GAAGAGTAAATTTTGAAGACCTAGAACTTGCTGTTGCGTCCGATATGAAGCAGTTGTGGGACGCAAAACTGA
CTGTTCATGCTGTGAAAACTGAAGTTTGATATTGGTGTTCCACTGCTCTGAGGTTTGAGGAAATTTTGTTCCGG
AAACTGTTTGAATTCCTTTCATCAGTGGACCGGTACTAGGTAACTCTGTGCACCAAGGTTTGGGCAAATGGGG
AAGAAGTCACCTAACATTTTTGCCTCCACTGGATTTGAGCC
>TCONS_00003083
GTACCTCTCCATCGTCGCCAATATATCAGATGAAAACAAATACCCTGTTCCATCCAAGGAAGAGTAAACAGC
TCGAGGAGCTATGGAGTAGAGGAGGATAAGGGCATCATTGGTATTTCCTCCAGTGAATATGTTCTCTATACAC
CATGAAAGGGCGTAGGTGCTCTCCTCGCTCTCATCAACGGCCACCAAGATCTTCCGCGCCTTTGCTGCCACGT
CAGCCATCCCAATATTTGACCGCACAAAGATGATTGATTGAAGAAATTTTGAGTGTCTTTGGACTTTTAGCTG
AAAATGGAAAGTGAGGGAATCAATTGAAGATATTTATAGCG
>TCONS_00003400
ATTCTTTTTGCTTCAATTGCCAACCTCACTTGCGTCTTCAGACCAGCATATGAGAAGTTGCAATCCTTGTGTTGT
TTCATAGGGACTTTAAATTTGACAGATTTGGCATTTCCTTCTCGAGCTAGCTCCTCCACAGCGGGGCCCCCACT
CCTCCTCAAATCAAGACCAAGCCATTTTGCTGTTTTGTCATATGCCTCGCCAATTGCATCATCAACTGTTGTCC
CAAGTTGTATATAATGGCCAAGATCACGTGCAAGGATTAGAAGGTTATGCCCTCCTGAGACAAGTAGGGCCA
TGAAAGGAAACTGCAACTCTCTTTCCACTAACCTGGCAACCAGAGTATGAGCTTCCATGTGATGGACACCGA
CAAGAGGTAAATTATGAGAACCAGCAACTTTTCTTGCTTTCTGCACACCAACTCGCAGACAAAGGCTTAAAC
CAGGACCAATGGTGACAGCAACTGCAGTTAGATCCTTTTCAGTCAGATTAGCTTTATCTAGCGCTTCTTGTACC

ACCTGATCAATCACCAGTGCATGTGCTTCCTCTGCCATCTTAGGAGCAACTCCTCCATATCGAGCAAGCAGTT
CTGCCTGAGAAGAGATAACTTGGCTGAGAATTTCACCATTGCTATTTACCACAGCGGCAGCAGTATCATCAC
AGCTAGTTTCAATGCCCAAAACAACTAAATTCTCATTTTCTC
>TCONS_00003401
TCTTCATCGGAAGCTGAAGCAAGAGAGATTTCAGCATTGCTGCATACAAGATTCTTTTTGCTTCAATTGCCAA
CCTCACTTGCGTCTTCAGACCAGCATATGAGAAGTTGCAATCCTTGTGTTGTTTCATAGGGACTTTAAATTTGA
CAGATTTGGCATTTCCTTCTCGAGCTAGCTCCTCCACAGCGGGGCCCCCACTCCTCCTCAAATCAAGACCAAG
CCATTTTGCTGTTTTGTCATATGCCTCGCCAATTGCATCATCAACTGTTGTCCCAAGTTGTATATAATGGCCAA
GATCACGTGCAAGGATTAGAAGGTTATGCCCTCCTGAGACAAGTAGGGCCATGAAAGGAAACTGCAACTCTC
TTTCCACTAACCTCGCAGACAAAGGCTTAAACCAGGACCAATGGTGACAGCAACTGCAGTTAGATCCTTTTC
AGTCAGATTAGCTTTATCTAGCGCTTCTTGTACCACCTGATCAATCACCAGTGCATGTGCTTCCTCTGCCATCT
TAGGAGCAACTCCTCCATATCGAGCAAGCAGTTCTGCCTGAGAAGAGATAACTTGGCTGAGAATTTCACCAT
TGCTATTTACCACAGCGGCAGCAGTATCATCACAGCTAGTTTCAATGCCCAAAACAACTAAATTCTCATTTTC
TC
>TCONS_00003505
CAGCCTGGATATGCATGACTAGCAAGTACACAAAATAACAAGGTTTCTCTCCAAAAGCAAGGTAATCCAAG
CCCCGGAAAACTTTCCAGGCTTATTTCATTATATATTGCATTCCTTCAGTTTCCTATGACTACCAGCTGTGTTAG
CAGGTTGCAGAAGCTATTAATAGATGAAGTACCCGACCACTGCAAAAATAACAATACTGCTAAATGAAAAG
TTTAATAAGGTGTCAAGCAGCCTCAAGTCTTTCTGTGTTGTCAAGCGAATAGACATCCATAGCAAGATGAGA
ACGATGGCTGGTCATAACTGGCGGAAATGAAGCTTCAATACTTGCCTTATCTGCCAAGACTCTTTCTTCAGGG
TATACAAAAATGCTCAACTAGCACCTGAAAGCTCCATCCATCTAGTGAACAGACCTCATGCTACTCCAAACG
TTTGAAGCCTGTCACAGTGAGGAGTTGGGTACTTCCTGTTCCATTGTCGCCTTTGGAGTTCTATATGACAGTAA
CGACCAAGCATATTTTTGTAATCCCTGATAACATGGTCTTCAATGTTTTCTCGAGCAGGACTATCAAGAGGAT
ATTTCTGATCAAATTCAGGCGATTTAACAAAATATTCTACTCCATGTTCTTCTGTAATCTTCCTAAACTGATATT
GGTAGTTCTTCTGCAAAGAATACTCAGGCTCCGAGAAAGGAAGATAAGCAAGTAAGATGATAAGCAAAAAT
GGTAATAACTGAAGGAGGAGAAGCAAGTTAGGCCCAGTGGAACCTAAATCCTCTCTCTGTGGAGCAGCATTT
GTTCTTGTCCTGTAAACATGAGATGTCCTAAACATATCAGAACGACCAAAAAATGCCCTGAAAATCTCGTCA
GGATCAAAATCATCATCATAATAGCTGTGCCCAGTTCTCCTCCTGCGTCTAACATTATGCTGCTGATTATACTC
AAATTCCTCAGCCAAACCAGTCTGGTCATACTGCCTCCTGGAATCATCATCACTCAAACACTTAAACGCCTTA
GAGACTTTCTTAAACGCCTCTTCAGAGCCCGGAGCCTTATTTTTGTCAGGATGAACTTTCAATGACAGTTTTCT
GTATGCTTTCCTGATCTCCTCAACTGAACTACCCTTCTCTAGACCAAGAATAGCGTAATAGTCCTTCTTAGTCT
TAATTTGCCTAATCAACTGCACGTGTTCTTCTGTATAGTTCCTCTCCCCGTCAGATACATCATCAGACTTGACA
CGACTCGTGACATTATTCACATTTTCAACATTTCTAACCTCACTTGAGGACCCAGCAGCTGACGACTCCAGAT
TTTCACAAGCAGCCAAAAGATCTTCTACAGATAAATTACTGTTAAGGCGCCGTGCAATTCTAATAAACTTGAG
CGCTCTCTGCTTATTGCCAGATGCAATTGCATCCTTGGCAATACCAATGCATTTTAAAGCCTCATCCTTGTTAC
TATCCATCTTATCGTTCAAAT
>TCONS_00003598
TAATCTATAACCAAAAGGCAATTAGCAAACTGGTTATTAGATGGTGCTACATTGGCAGAGTCATCAATGCCG
CTGCTAGACGGGCCTTAGTAGCAAGGTCATAATCCAGCTCACTGGAGATCCCAGGTTCGAATCTCAACTACA
ACATTTTGTGTTACTCCAGCTCCAGCCTTGATGATCAAGATTACCTTGGCAACTTGTGTTTGTTGGCTGGACAA
GAGCCTGGTGGATTAGTCAAGCTGACCTGACAACACCGATGTAAGAAAAGAG
>TCONS_00003615

GGGATGCTGGTGCATATTCGCCAATCTTTTGATTCTGTTGACCTTTGTGCATGTATAAATCAGACTTGGAGAAG
AGAGGTTGTGCATATGCACTGGCTTTTCGACTTGTATGCATCTGTGGTCAACCATGTTTTCTGGAAATTGTTAG
GTTTCCTTGTTTGGTGAGGATTCTGGGGAGATCTGTAAGAGGAGGCTTGTAGTATGTC
>TCONS_00003750
TGACCAAGCCCAAATCAAACCCGTTCAGTTGGAACACTCGGGTCCCGGAATTTCACCAAAACCCTAAACCCT
AGAATCTGGAATCCAAACCAATTTCTATTCCACAATTACACCAAAACCCTAAACCCAGGTATGCAACTCGCA
AATCGGCTTGTCTATTTACTCGGAAGCTCAAATCGGAAACCCGATGAATTTAGGGGTTTCACGCAATTCACAG
CATCAATCTCAAGCCTTAAAGTAGCATGGCGCAAGGACTCAAAGCTAGACCAAGCAATCGAAAACGACAAA
AAATGGAAGCATTGTGCAAGAGTTGTTAAAGAAGTGCTAAACGAACCGGGTCAGGCATCTGTAGTTCAATGT
CGATAGCTGCATTAGAAGGGAGATTTGAGCATCAAGGATGAAGAATTCATAATAAATTCGAGTAATATGGAT
TATGCTATTCTGTGGTCGACCTGCAATTATAACGTGCCTTGTAAACGAGGTGTCAGCCTCGCATAAACTCAGT
TCATAATGAGAGATGATGACTACCAAACGCCAATCCTGGAAGTGGAGCTAATCTGATGTGATAAAATCATTG
ATCATACCAAATTAGCAAAATTGCTGAAGATTGCAGTTTCTCAATAATAATTGCACTTGTGGACTGCTCCATA
GGGCATGACAGGGGAGCACATGCTAATTATCTAGCCAATTGGTGATTGAACGGATGAAAGAGGTGGACAAT
ACGATGACCGGACAATAGTTATGTTTCGTGGAGCAAGGGACGTGGTTGGAAACTTATGGGCCTAATGATAAC
TGCAGGGGATGTTTAATGTGATGTAAATTTTCAGTATAAGCATCATGCAGACCGAGAACAGGCTACAGATGC
TAATTTCTTTGTAAGATAAGTCTGGTGCAATGGAGTTAATCATGATCAGTATATATTCATCTTCCTTTCCTGAA
ATTCCGAGCACCTTCCCTTATGCCAAAAAAAAAATAGTAAGGGACAGGGAAATGGTATAGTTTGTACTTTGT
ACTAATCTTTGACACTAGTGTGACTTTGTTCCCTCTAGTTTGGTTAATATTCGGATCTCCCTCTATTTGGGATTG
AGGCGTATTCGTTTTTGTTTGTTGTAGTTATTGAGATCTCCGTCTAAACTTTTCCTTTGTGAGGATAATATGCAC
TGGTTGGATTTCCTGGCATCATCATAGCTACCAGAAACTATCTCATATCTTTGCTTCCAAGTTTCTTTAAAGTA
GAGTAATATTTTGTGATCTCAAATTGTTAGAGGAGTTCCAATTTGTAATTTAACTTCGTAAAGATATTGTTTAG
GGCTGGACTTAGCTGCAGTTACCTCTTGGTGTTGGAAGTATATTATGCTTATAGGAACTCCTTGATCCTGCTAT
ACATCATTTCCTTTTTCTTTTCCAAATTTTTGTCCATAATTTTTTGCAACAACTGGCCAAGCTTATATATTAAGG
GGTTTACCGATGGATTGCAGCACACATTTGTACCTCAGCAATGTTGACCTATGTTGGGTCTGTGCAGGAAATA
AACAGTCATGTTTGTATAATCTACGTTTTGTATCACAAGGCTTAAAATAAGTGTGAGATTAATTCTCGCTCTCC
>TCONS_00003850
ATGAACACTGCTATATCTAGACATTACTGAAGGCACATTTTACATCAGACGACCAGTATGTAACAACCTTTGC
TAAAACATAAGGCACGATAAAGTATAATCACACGATATACATATAGAAATGAACATAATACGAAGAGATAA
AGAACAAAACATCATTCTGTAACCTTCACGCCGGTTAGGTAGACTTGGCACTGGATCACACTGCCGCCGATA
TAGAAACCAGGCATAACCATCAGACCAACCTTAGGCTTCTCCTCATTTTCATCTACGATAAAATTCTTCAGAA
CACTTTCCATATAAACCTCTGAGAACTCACTTCCCTTCTTGACTTGAAATACTCTAACCGGAGGATCAAATGA
ATATGCCAACCTATGCAGAAGCCAAATTGACTTGGCTAGTTTCAGAAAAGCCTGGTAGAAAGCTGTTCTTGG
GTGCCCGCCACCCATAACGTAGTTTCGCTGATCCAAGTTCCCAAAGAATGAAGCCTCCATCTTTTGATGAACC
ACCACTATATATTTGCTCCGGCAGAAGTTCCCAAAAAGGGAATCTGGACTTTGGCCTACAGCATCCAGTGGA
TCCATTTCACGTAATGCAAGATACTGGTGGAAAAAACTATCCTTGGTGACAGCTGAATTCTCATGTTTCACAG
AGAAGAACTCGTCCTGAAATCCGGTGAACATTCTTTGGCAGATATGTGACTCAAACGCATACTTCTTGTGAGC
TCTCTTTGCATAAGCTATATCTGGCTCTATGGAGTTTGCTGCAGCATCAAGATCCCAACCAGCAACTTTCATCA
TGTTGATCAATGGCTTAGAGAAGTCGTGAATGGCCCTGTAAGCAGCTTCCACAGCTGACCTGAAAAGCTCAG
GTGTTAGGTCCATGACGAAAAGCCCGTTCTCGTCAACTGCATTTTCTGATTCTTTGGCTGACAAGCCCCTGAGC
TTAAGGTTTTTCTCCAATTTGACCCGCTTCTGATTGGCCTCTTGTATCTGATGCTGCAGCTGACCAATCTCAGA
ATCTTTATTCTGAATCTCAGATTGAAACTTCTTCACCATAACTTCATATGTTTTCAACAAACTCTGCTGTTCTTG

GATCTCTGCAGCCAAGCGAGAGTCCTGAGGTGAAACACACACAGGTTTGGGATTGTGCTCCCTGTAGAAGTG
CTTGAGTTCAGAGAGAGTCTTCAGCTCCGAAATTACAAGTTTATCGGCAGCTTGGATTTTATCCGGGTCGTAT
GGAGTATGAGCAGATTGGAGTTGAATGTAGGCAGACTTCAGGGAAGAGATATTTGTGAAAATCCTAGATACC
ATAGCTTCAACTGCTTCAGGATTTTGGTTTGCGGCTTCCTCCATAGGTTGTGGATGGACTTTTTGGCTGTTGCTC
TCGCGGAGTTGTGTATCTTTTGCACCACTGGGTTGCATAGCTACTTGTAATGCGATAAGATTAATCAAAGCCC
AAACCCTAAAAGATTGTGGTGATTAACCTGTAAAACCCAAACCCTGTCAGAGATGCGTGAAATAATCAAAA
ACAGGCAAATAAGAATTTCCAAACATCTAACAGTTGCAGTGTCCAGAAAAATCTAAGAAAACTACAGAGCG
ATGACTGAAGGTGTCCAACATACATAATAACCTTAACACTGATCGGAGATCCAGCTGACGAGTTACCGGAAT
TGAAGAGCAGAGTTAAAGCTGTGGTGGTGGCGTTGGAGTTTTCCGACAATATCTCAAACCTTGGGAAGCATC
TTTGTTTGTCTGTTCTTTTTAATCTTTCGGTGGTAGCACGCCTGCTGGATTTGGTGCTTTC
>TCONS_00003971
CGGGTCTTGACAGCTGATTCAGTTTAATCATAAAGTATCTTATAGCTCACTACAATATCACAGACACTCAGTA
ACGATACTTGGTAGAGTAATCTTTAGGTGCAATAACTAGACCACGACCCTTTCTCGCGCCACGGTACACTGTC
TCAACAATATCAATGAACTCTTGTTTATCTTTGAGAGCCCAGTTGATCTTGTTATTGTTTCCTGTGCCAAGATC
AATCATGATGTGCTTGTTCCTGAAGAAGAACATGATGGTGGATGGATCGTACAACTCATACATTGTGTTAAAA
TCAGGGACCTCCGTGATGTCCACCAGGTATATGACAGCAAAATTCTTTAATGTATCTGCAACTGAAGCCAGC
ACCTCGTCCATCTGCATGCAAGTTTCGTCCCAGTCATGGCCGAAGCGGATAATGACGAGGCGTTCCTCTTCTG
CGAGAATGGCCTGATCCACCGCCCATCCCGAGTGAAGGTGAGGCAGCAAGTACGACATCTTTCTCTGATCGC
TAGGATAGCAAGGGAAAAGTAGCTCAA
>TCONS_00004098
GACTTCACAAGTTCATACGTTGAGAAGAACTGTACAAGTAGCCATTGTAGTATATAACCTATGCCTTCGCCTT
ACAAGTTAAAATACTATCTCATCTATCAACTTTCATACACATTTTTGCAGGCCACTGTAGTATATAACCTATGC
CTTCATCTCATTTAACTATTTCTACGGCATTTAAGTTACTCTGCCTAAGTCAGAAAGGATGTCTACCT
>TCONS_00004160
AGGTTGACTGTAGAATTTTTTGGTGGTCATGGTCCGTATTAACTGTGGTTCTGTTAGTAATTGCAGGGAATTGT
ATTCAGTCCCGTTCCTTTTCAATCAGAAAATATGTCGAGTGCACTGAGCCGTGAACAAGGTCCCGACCAGAG
GAAGGGTGGTTTACTTTGGGCAGCATTGCAGGCAGTCTCTACTGGTTCACCAAGCCGCTTTTTTCGCCCGGTTG
ATGTAAATACGATTAAGCTTGAGATGCTTGATGGAGGATGTTGTGTCTAGAGTGTTATAAAGAAGTTATTTTG
ACTGAGAAGCCTATAGTCGATAAATGACATCAACAACATTGGATTAGTAGTGTTTCAACTATATTGCGTAGGT
CCACATAACAACTCAATTGGAACAAATTTCTAAAAAGATAGACTAATCGGAAGAGGGAAAAAAGGGATTAC
TGTTAATTGCTACCACTAGTTTGAGCTGCCTTTTGTATATGACTTCATACGATCCTTTTTGAAATGGAAGTTGCT
TTACAAAGGGTACTCACATGGATAATACTGCTCAATATGGGTATGCTTTGACTTCTACACAAATAAATAGAAA
TTAACTCTAGTTGTTTAAAAAAAAA
>TCONS_00004234
ATTATATGTAAGAACCATAGCATTTCGCGATTCATTGGTAAATCTATTTTGATTCTCTATTAACCAATAATGTG
GAACTATTAACATGGTTAAAACAAACTGTTTGAAGTCTAGACGCAGCATGGTACTCTTTCTACCACTATGTTA
ATATAGAGGTGGTTTCAAAATAAATATTTTATCGATATAGGATACTCATATTGATAAAATGATTTGAACCGC
>TCONS_00004235
ATTATATGTAAGAACCATAGCATTTCGCGATTCATTGGTAAATCTATTTTGATTCTCTATTAACCAATAATGTG
GAACTATTAACATGGTTAAAACAAACTGTTTGAAGTCTAGACGCAGCATGGTACTCTTTCTACCACTATGTTA
ATATAGAGGTGGTTTCAAAATAAATATTTTATCGATATAGGATACTCATATTGATAAAATGATTTGAACCGCG
>TCONS_00004271

CTTGCTTGCGGTTAAGCTTTCAATTCGGAAGAGGACATGCGAGTTCACCACATGTCACAGAACCAGTGGTCA
AATCCAGATTGTTCTAATCATTGGATCAACCGCCACAAACGAAACGTCGTACATCGTACAGCTTATCTAAGTC
GGTTATGTTTTCCTCCTAGGATTCTGTCGCCGCCACGCAAAAAGTTCTTATCTTAGTCGGTTATCATCTTATATA
TAGGCTTTATATTCCCTCAACTACAACACCAACTATTGAGATTCTACAATCTCAGCTGTCGCGTTGGCTGCTCT
GATAAGATGGTCTCTCCTGCCTTCTTTGCCTCTAGAAAGTTTTTGGCTCAAAAAGCTGGAATGCAGACAAATT
AAAATTCTTTGCGTGGTTTGCTGGATTTTCCATATTGTAAGATGTCAATCAAGGTTATGTATTCAGAAGCGTGG
AGAACATCCGTAGGAATTTGCTCATGTTGAATGAAGATGTAATAGGTATAATGGGGACATAAAAGGCCTAAT
TTCAGGGGGGTCAAGTCCCTTTTAAATTACTCTCTGTTACAACTCTGGCCTTTGCCATGTCGGGTGCGCTCTCA
TGGCAG
>TCONS_00004521
AGAGTTAGCATAAGACCTTTATAACATCAAACTGCATGGATTTCTTGTCCGGCCATTCTCTAGTTTTTCTTCTT
CACCCAAAGTTTCTGACATATATAACTTTGCAGATTGAACAAAGAGTTTTTGACCATCTGCAAGTCGAAGTG
GGAAAGCTGCGGTTTTCTATGACCAAAACTCAGGAAGTGGAAGACAGATTAATTGAACTGGAAGCACTGCA
AAAGGCCCTGCAGGAAGCAACAGGTATGATAAGTTGACTATTTGCTCTTTTGTTAGATATTTCCAGACTGTTTT
ACATGGAAAAG
>TCONS_00004715
GATTTAGTGCATCTAAAGAGAACAGGAACCACAGAGGAAGAAGGGACGTACCTGTACTCCTTGTTATTTATC
CCATAAGAGACAGGCTCCATCTGCAGCTTTGTTGCAGTGCTTCCATAACTAAGTACATCTGAAGTAGCAACG
GAATATAATGGCATGTTTTGGAAACTACCGCTGCTATTACTTAGGAAACTTCCAGTTCTATTGCTGCTCCCAGT
AGTAATATGTGATATAGTAGTCGACAAGGACTGGGAAGTAGATTGAGATTCCACAGGCTTTCTTGAACGGTT
ACGGCCTCGATGCATGTGCCGCTCACAATATTTTGAGTCAGGATGTGCATCTTTGGAGCACCTCCATTTCTTTC
CATCAGTTCTTCTACATCTTCCTGGCTCAGGGTCAAACTTCTTCCCATAATAAGAACAATAACTCACTGGCAG
AGATAGCATCAAAACTCCTTCTAATTGGAACTACAAGGTCCGGTGGCACGGGCATCCCTGCCACTAAGTACT
TGTATATCATTGCTTGATGCTCCAACTCCTGCCACTGCACCGCCGTAAATGGTGGCCGGAATCCATAGTCATA
T
>TCONS_00004725
ATCTTTCCTTCTTAAACTCCGTGTGCACTCAAACAACATTATATGAAATGGGACGGACGGAGTAGTTTGGGTT
TGAGGGCTAATTGAGTGATAGTCAATTAAGGTCTTTTCCTAGCAGCTGCTTTACCTTAATGAACTAGAATCTAC
AACGTGTAACGTTTTTCCCTTTTCTGACTATAGCACTAAGTTTTATGAAGCGAACACTTTAGTCTGTTGTTGGAT
TGTTCGGTTAGCTCCTGATTAAATTTCTACAGTCTGATCACTAGACATTGTAGTTGCTGGAGTGGATTATGATT
GACCTTCAAGTCGGGTGACATTAAAGTTATTTAGCAAATGATACTAGATGTAACTTGAAATTAGAGAAGTAT
GTTTAACGAGAATTTATTCATTGTTGATCATTGTTTCATACATAAATAGATACATAAAAATGTCCCATCTCTAA
TAATGTTAAGCTTGTAAAGAGGCGGTACACACAATTCAATAGTTTAGAGCAAGCGAAGGTACTGGTTCGAGT
CTGACTGCCTCTAATAAATAAAAAATAATCTTTAGGTGCTTGGTCTAAGAAAAAGAATCAAATCTACAAGTC
AGTGGCCGTGTTGCAGGCCTAATTGAAAAAAATAAAAATGTCCTCTCTCTAACAGGTTAAGATTCTAGATGA
GGCGGTACACAATTCAACATTCATAAAGCAACAAAGTTACCTAAGACTAAATGCAGTTATTGTCATATGTTCC
TGGAAGATGATTCAACTTTTTCTGCTCAAAGCTTGTTCAACAGAATTTTGACGGGATGTAGGGTATGGCTTTAG
CTGTGCAGCATTTGGGGAAATTGGCTGTGTTGTGCAGCCAAATCTTGTAAAGCTCTGCACTTTAGATGTGTGTT
TGTGCGCTCACACTGCATACTTGAGAAATATGCCTTATTTTCTGTATTTCCTTTTAGGGGAAGGAATTGATGTTT
CTTTATTTTCACCTAGTTTCTTATTCTGGTTAGGGAAACAGCAATAGCTGGGAATGCCACCTGTTTGGAGGTTG
ATAGTCCAAAGGCTGATTATGCATACAATGAAAAGAGTGGAGAATTATGTAAGAGAACATGTTCGATAGATG
AAAAAACGGAAAGTAAGAACACTT

>TCONS_00004855
GAACTGAAACAAGCTTTATATAGAAAAAATCAGGAGAATAGTTTAGAGCTCCCTCCGGAAGCATCTAGAGA
ATCCTCAAGGCTCGACGTTAATGGCGAGGGTTCCAAGCAAGCACTCTCGCGATCAATTTCAGTGATGCTGTGA
AGGATAGTGCAGGTAGGATCGAATTATAATAGAATCTGGCAGAAGCAAACACATTAATTCAAATACCTTTAT
TATGACAATTTTATG
>TCONS_00004866
CAGCAGCACAACAACTAGAAGGATGGTGCTTTTGAAATCGAGTTAGGGAAATATGTTGGAAACCGACCAGTT
CAACTTCGGAAGAGTAAGTGGCAAGACAGGATTGATTATGAAGCTTTGGAAAAACAAAAGAATGTGTCTCC
GAAGAAACCAAAGTTACCAAAGAAGAGTGTATTGCACAAGTGAGCGTCAAATCATATGATTGTGGATCAGT
GTAAGATATTGTTGATCTATATCTATGGCCATTTGGGAAAGGAGGAAAAAGTGGCAAAGTCATTGATAGAGA
TGCTCCTATTCAACTTATGTAGTGCTTTATGCTTCAGTTGATGATAGTTATTTTCTCCTGCCTTTTATGGCCGTTA
GAGCTCGGACTTCCTCGGCAATA
>TCONS_00004929
GGTTGATTTTGATTACCTGGATGAACCATTTGGAGATGCACGCGGCCTTCTTTCAGGCTTTTGTGGAATTTTAG
CATGTGACTGCGCTTTATTTCCTATCCACTGTGAGAAATGGTCGAGTTTACCTATCTCATACTTCAACCGCGTC
TTCGATCAAATTATAAAGACCAAGTTCTTCTTTAAGACAACCGAGTCAGTTGCACGACAACATGTTTATAAAT
CTATTGGAAAGAAATGGG
>TCONS_00004945
GTTTTAGAGCGCACAAGATGCACCTGTAAAAAGTGTATATGGTTTCTCTTAATGTGATAGCAGATTAAAGGAT
GTATTTAGTACAGACCTGCACTAGAAAAAATAACACGAGAGCATTTGAAGGAGCAAGAGATAAACTCATCA
TTCCTCCGCGAAGTTCATTAGGCACGTACATGGTTCTCAATCTGGCAAATGAAGGCAAAAC
>TCONS_00005033
TGAAGGATTAGTCTTTGTGCTCATCATCGGATTCAGAATCTGATCGGACATCATCAGTTATGTGGCCTTTTGTT
ATGAGATCGTTAACCATCCGTAATATGAGTCTCATCCAAAAGATGTGAAGAACAAGCAAGCACACCAGCAG
TGAATTGAACAAAAAGTAAAGTATGGTTCCTTCAGTCCGGTGCTTCTCTGTGTCCACTATGAAAAGAATTTCG
TAGCTGCAG
>TCONS_00005091
TTCGAGGGTTTATTTTATTAGTTTTAATAATAGGAATTTTGTTTAATTTAATATAATAAACAAAGTCTATTTTGT
ACTCTATCTGTGTATCCTTTTTTATTCCTAAAAAATAGCAGATGAAATAGAAGGCTTAGAAGGGAGATAATGA
AATTATGTGATTGGGTCTTCCAAAAGCAAAGGAATGATCCGTTTTTTAGTTAACTGATCTGATGGGTCCAACA
AACAATAAATTATAACAAATATCTAAATTCTAAATAAAAAAATCAAAAATAATAGACTAAGATTCTAAATAT
AAATAAAGGATAATAAATAAACGGGATCTTCTTTTATTCGAAACGTCTCGTGATCTTCAACCAATTATGCGCT
TCAATATAATTACCGGGAGTAAGCGCTATAGCCTGTTTCCAATACTCAGCGGCTTGATCGAACCAAGCCTCTG
CAATTTCAGAATCTCCCTGTTGAATGGCCTGTTCTCCCCGGCCGGAATAGATCACGGCCATATTATTAAAAGC
TTGTGGTAAG
>TCONS_00005249
GATGCTGTATGCACTGGGTTGCCCTCTATAGTGGCATGTTATTTTGCCAAATCGACATCAGGACTGCTTGACAT
TGTTTGTCAACATTTGTGCACTGCTTTTGAATGATGAATTCTGCTCTGTCCTTGGCAATTACTAGTAACACCAA
GGAGAAAATATAAACAGAATTGATCGTTGGTGGTGTGGCCTAGCGGTCAATGAAGTGGATGAAAATCATAG
AAGATAAGATTCAAATTCTAGTACTTCGATAGAAAGCCG
>TCONS_00005326

AGATTGGTTCTCTTTCTCGAAGCACCACTCGTTTTCCAACAATTAAAGCGTCCAAACTGATCCTAGATTCCACC
TTAGTGAACCAGAGGCCAACAGGCTTGAGCAATCTGAGGATGATATCACTAGCTACCTTGCACTTAGCTTTAC
TGAGAGAGACATAGAAGCAGAGGCTTGCATAGTTTCAAGCATATGGCGTTGATGTTGAGTAATTGCTGCACT
ATTACACACCTTCTTTGTGTTTAAAATTCAATTGGATAGATGCTTCCTTGATTTTCTCCTTTCGGGTACCCAATA
TTTGCTAAAAATCTAAGAGGATTCTCCTGATCCAATCAAAATCATGCAAGCTAGTTGGGGCTATGAGAGATG
CTGGTCTCATTTTCCCATGCCATCTTTCTCTTTTTCTTTCTATTTCTCTTTCTCTTTCTCTTTCTTGAAGCATCGTCC
ATCTTCTTTGATAGGCTATCACAAACTCTAATCAGCTCAACATTCTGAGATTCATGTTCCTTCTGAACTACCAT
TGTTGCTTGATCCATCTCTAGAGCTTTATTCTCATACTCCAAATGTAGTCCCCTCTCTGTTGTGATGGTATTCTC
AACAAACGCCTGCGAACCAGAACCGCATGATGGCTCTAGTTGTTCAAATACCATAGAACAGCCAATTTCAGT
TGTCACTGCATTATCATTTTGCGGAACTGGATGGAGTTTGAGACTTTGTGTCACCCGTTGCTTCCTTCTCCATCT
CCTAACTTTATTCTCATATTCCAGGCGAATTCCCCAACATGCTTCCGCTTCTGAGGATACCTCAAATAGGCAAT
AATCATTTAGGTTCTTCTCTTCTTTATTGTCAGAAGTGCGCCACAATGCTTTAAATGGTATGTAGTACCACAAG
ACAGAAGGAAAGTTAAGGCAACGTTGGTGGATGTGAAACATCAAGGAATGTTT
>TCONS_00005328
GCTTACGAGTTTGTTGATGCTTTTGGGTAAACAAGAAATATTGCTTCCTCTTAAATTCAACTCTTCCAGAGAGT
GCAAAGATCCAAAATCCTCAGGAAGTCCACCCAATATGTTGCAATTACCAAGATGAAGTTTTATAATTTTTTA
GATCATGCCCTGATAATTTTCCAGCCTCCGCAAATGCATCCCTCCATCTTTGAACCTTCTCCATATCATCTTTGT
ATTTTTCCTCATGTTGGGAAAATGACTCGGCAAATGGTGGACTTTGATGGCGTACATCAGATGGACTCACATC
ATAAAAGATTGGAATCACAGTCTGCTCCAATTCATTTTGACACTTTATGATGTGGGCAAGCTCCTCTAAGCAC
CATCTTGAGGATGCATAGCTTTTTGAAAATATCACGACCGCAAATCTGGAGTCTTCTATGGCTTTCAAAAGTT
CATCAGAAATTGGTTTTCTTGTTTCCAGACGCTCGTCATCTTTTTCACCATAGTTAATAGGAGGTAAAAACTGA
AAGCAAG
>TCONS_00005329
ATTCTGCAATGTCCAAGAGACGGATGGACCTCTTCCAAATTCATACACCCACACAAACTTAGTGTCTCCAAGT
TTGGCATATCACCAAAATTAGGGCTTTTTGTTAACCTGAGGCTCTTGGTTAGATCCAAATGCTTCAACTTGTTC
AATCTTTATAATTTTTTAGATCATGCCCTGATAATTTTCCAGCCTCCGCAAATGCATCCCTCCATCTTTGAACCT
TCTCCATATCATCTTTGTATTTTTCCTCATGTTGGGAAAATGACTCGGCAAATGGTGGACTTTGATGGCGTACA
TCAGATGGACTCACATCATAAAAGATTGGAATCACAGTCTGCTCCAATTCATTTTGACACTTTATGATGTGGG
CAAGCTCCTCTAAGCACCATCTTGAGGATGCATAGCTTTTTGAAAATATCACGACCGCAAATCTGGAGTCTTC
TATGGCTTTCAAAAGTTCATCAGAAATTGGTTTTCTTGTTTCCAGACGCTCGTCATCTTTTTCACCATAGTTAAT
AGGAGGTAAAAACTGAAAGCAAG
>TCONS_00005363
ATCCGTTTCTTCTTTTCGAAAATATCATTCTTTTCACTGTTAGAATTCGTGGGATTGCTGAATTAGTCTGAAGCT
TTGATTCTCCTGATATTGAGCGAATTCCTACGACCCACTAAGGGTTTGTGGCTAAAGATCAATTTTTTTCATGG
ATCCCAAATATACAGGAGAAACCTTTAAGCATCTGGAGAAAGAAAGTGAACTACTCTTGAATACCCATAAAT
CAATGTCAGATGAATTGCACAAACTTCAGGTTGTCTTCTCTTGCTCTGAACACCACCATATTCCTTTCTTGCCG
AATGAAATACATAGTTGCTGCCAAAGTTGT
>TCONS_00005421
CTTGTTCGGATTTTCTCAAGCAGGAAATGGCAACACTATATCTTCGTTTGCAATTACACCCAACTAAATTGGGT
GGTGGAGAGCGAAAAGGAGCTAATTTAAAATGAACCCATTTTGGGTATGTAGCAGGCGAAGGGATTGGAGA
AATCCTGAGCGAGCTGGAAATGAACTTAGGAGGGAATTGAGCAGTGTTGTTATAGAGTAGTACAGGAGCCA

ATCTCATGGCTTTAACTTTGATGGAAACTATCTGAAAAGGATTTTTCTGGTTCAAGAATGGAACTGGTTTTGCA
GTCCCG
>TCONS_00005459
TCCAGGCTGAGGGTCCTGACCAAGGCGTTGACTTCCTGAAACTACTTGTGCCAAAGTAGGGAGCACACGAGC
CGAGAGTCTCGTTGCTGAGAAATTACTTAGCCATGCTAAGGTATGATACATGAGCGCTGAGAACCATGAAGC
GAGTGGCACCTCGTGGATTGGGCCTATTCGATTAGGTTGGGATCGAACCCGTGCCGATCACACGGTGACTG
>TCONS_00005545
GTTCTCTCTAAACAATGTTTAATATGGCTTTCACTTTCAACGATATGTTTGTAAATAGAATCTGGTGTATAGTA
TATATGGAAGAGTAGCTTAAACGACTCCCTTTTCAAGGTTTGCGTCTTTGAAACACAAAAAAGGAACCTCTGG
AGCCCTTTCCCAACCTGATCTTCTCGTCAATGTATGTGATCTCTAATTTAACTTCACTCTCGCCACCATACTGG
AAATCTAGTGCCCCTACCTTGAGTTCAGGCGGCTCGAAAACAACTTGTATCCATTTTTCATCTAGGACGTTCA
ATTTTCCTCCGAATATTAGAGGGCATTTCACAGGTTCATCCACACAGAAACGTTGCAATTCTTGAGCCACTTC
GGCTACCCTTTTGTGCTCATCCCTTGTCAGGGAAACCCCTCGATCTGTTTGCATAACCT
>TCONS_00005653
ACAAAAGGCCAAGACCAACACATTTTAAAGCAGGAGCATCATATTTCAGTTCATATAACATTCAATAGAGGC
TTCCTAGATCTTGTTTAGCTTTTCTGCTACAACAACAGGATTTGCTTTAGCAATAGCATCCCGACCAGCATTCT
TATAATCAAATGGGCAGTCATGCTTGTCTGAATAACGATGAGCTGCACAGAAAAGATTTCCACACTTGCAATT
GAACCCTGTTAAGCCCACACGCTTGCGGCAAGTAGTGCACCTAGTTGGGCCCACTTTTGGCTTCACCTCTGAA
CTCTGACCTGAGGATAAATCTGAAGATGCTTCTGTAGAGATAACCTTTAATTCTGCTGATCCTGGTTGCACATT
GATCGCACCAGTGACAATAGGCTCTTTGTCATTACTGCTTGAGTTTCCATTTACAATGTTTTCGATTGATGTAG
CTGCGAATTTAGCCTGTTCCTGCTTCAGTACCATGTCCTTGTGACACTTGGAGCACATATTCATGGTGGCTGCA
CTACCAAAGAACCCACAGTTGTTGATGCAAAGGATGGGGCCTTCTGGAGCTTGGCAGCCTGTCTCTTTGGAGG
ATTCCATTTTCTGCAGTTAACCTGCCACTTAATCGAAGCGATAAATCGAGAAGTCGAGAGGTGAAGAAAAGA
GGTAGAAATTGAGAAAGATTAGAGAGAGAGACCTAAAAAAGAGAGAAGAAAGAGAAAGAGGAGGATTGA
ATTGTAAAACTGCGGAAAATGATTTTTCGTCCCCTTTTATTTAATTGTAATTTCAT
>TCONS_00005654
ACAAAAGGCCAAGACCAACACATTTTAAAGCAGGAGCATCATATTTCAGTTCATATAACATTCAATAGAGGC
TTCCTAGATCTTGTTTAGCTTTTCTGCTACAACAACAGGATTTGCTTTAGCAATAGCATCCCGACCAGCATTCT
TATAATCAAATGGGCAGTCATGCTTGTCTGAATAACGATGAGCTGCACAGAAAAGATTTCCACACTTGCAATT
GAACCCTGTTAAGCCCACACGCTTGCGGCAAGTAGTGCACCTAGTTGGGCCCACTTTTGGCTTCACCTCTGAA
CTCTGACCTGAGGATAAATCTGAAGATGCTTCTGTAGAGATAACCTTTAATTCTGCTGATCCTGGTTGCACATT
GATCGCACCAGTGACAATAGGCTCTTTGTCATTACTGCTTGAGTTTCCATTTACAATGTTTTCGATTGATGTAG
CTGCGAATTTAGCCTGTTCCTGCTTCAGTACCATGTCCTTGTGACACTTGGAGCACATATTCATGGTGGCTGCA
CTACCAAAGAACCCACAGTTGTTGATGCAAAGGATGGGGCCTTCTGGAGCTTGGCAGCCTGTCTCTTTGGAGG
ATTCCATTTTCTGCAGTTAACCTTAATCGAAGCGATAAATCGAGAAGTCGAGAGGTGAAGAAAAGAGGTAGA
AATTGAGAAAGATTAGAGAGAGAGACCTAAAAAAGAGAGAAGAAAGAGAAAGAGGAGGATTGAATTGTA
AAACTGCGGAAAATGATTTTTCGTCCCCTTTTATTTAATTGTAATTTCAT
>TCONS_00005783
GAACTGCTTGAGGTCTCATCTTTCCAAGATGCTTGCCATTGCTTGTCGTAGAAAGTAGTCTCTTCCACCAACTT
TTTGATAGTTTCTATATCCTCATTCTTGCGGATTAGAAGAACACCGGCTACAGCTACAAACAATAGTGTTTTAC
AGAAGAAGTTTCCCATTTGATCGACGAGGCCTACACCAGTCAAGCTATCCACAAAGTAGGCCATAAAGAAA
CCAACCATTGCAGCACGACCATTGAGACGTTCAGCTTCAGGAAGATGAAACCTCTTCATCCATGCCCACCAA

GGAATAATTGAGGTGTCGAAAAGCACAGGATCTTCATTACTTGATGACTCTGGGTTATCTTCTAACCATTTCCT
TCT
>TCONS_00005858
ATATTATTGTCCTGAGTTGCTTGGAGGCTTTTCCTTTGGATGCTTAGAGCCATAGTGGTCCACAAGTTGATTTTG
ATTTGCTAGCTGCGCTTTACAAACGGGGCATACGACTTTGAGACCAACAGCTCGGGCCTCAAGTTGAGACCC
TTTTGATTTCTGATTCTTCTCCGCATTTTTTTTCTGGGCTTCGATCTTCTGCTTCCCCCTTGTCATCTCTCTTTCTCT
C
>TCONS_00005946
CTCACAATCATTTGGTCAGAAACATCGGAAGGCTATTTTGACAAACTGTCCTGGCTGCATCAAGTGGCATCTC
CATCTGTTACACTTTGAACGATTTCAAAAACAATTACTGGCCTCAGCAGAGTAGGCAGATCAAGTTATGCTCT
AGCACGAGATGGATGGCTAAACGGATCATTTTTTCGAAGACCCGAAACCCCCAAATCCCATCATGGCTGCAA
CATCAGGGTCTATTTCGGGTTCATCCTCTGCAGCCTTCTCTTTCTTTTTCTCTTTCTTCTTTTCTCGACGTTGTCTT
TTCCGTTCTTCCTCTTCCTGCTGTTGTTTCAGCAGACGCTCATCAAAATCTTGAACAGTGAAGCTCCCCGTATCT
TTCCGCTTCTTGAGCGATTCAAATCGTTGTTGAACCTGCTCCAGCGAGGCTCGTTCTACCCGCATAGACATACC
CAATGCTCGCTGATGCTTCTTCCCATTGATATGGTCCAAGTAGTTTGCAGAATCCTTTACTACACATTCACAAA
CTCGGCAAAAGTATCCAGCCTGCTGACTAAGGGGTGCAATTGGAGTAACCACCTGAGTCTTTCCCAAACGAG
ATTCAAGGTCGACTTGATAATCCCTTTGTTTCAAGGGCTTCCTCTGTACTGGAGGTCCTCTTGACTTGGATTTCT
TTCCCTCGGTCTCCTTTTCTTCACGTTCACGAGCACGCTGCAAATACTCTTCTTTATCGAACTTTTTCCGAAAAG
TATTATCAATTCCAACAACATTATCGCTGGATTGAGCCATTTATAGGTTTAGAAATAGGGCTTTGC
>TCONS_00006013
CTCTGAGACCATGTTGTCGTGCGAATGCGCTAGGAACATGCAGAAAGCACTTTGAAAGGTAATAGGGTTTCA
TGGTACAAATGAAATGAGGATGACCAAGATGCAAGTCCTTAGCAGCTTCTACATTGGGAACAGCCTTGCATG
ATGATATGATGTTGGACTTTGGTTTTCCTGGAGTGGAAACTCTTTCAGTATCCGAATTAGTCTTCTTTCCC
>TCONS_00006125
CTCTAATGCTCCTTCCATATACGACCTGTGACTTTCAATGTAAGCTCTTGTCCATTATTCCCACCAGAAAAGAA
AAGTGCCAAGCTTGTTGGAATAATAAGTCCATCTTGACAGTCGCGAACTTTTCTGTGGCTGTCTCGGATACTC
CTAGGAATACCTTGCCATGTCAATTTACGCCCAAAACCACCAACTTCTAGACTATACTTGAACATTTTGGCTT
CAGTGTCCTCGCCCATAAATCGTAGAAAGGCCATGTAAACTGGTGTCATACCAAGTGAAAATGCCTCAAAGT
GCAAGCAGAACTGTTTTCCATAACAGTCGAAAACTGTGAGCATCCATATTGC
>TCONS_00006147
TCTGCAGCTACGAAATTCTTTTCATAGTGGACACAGAGAAGCACCGGACTGAAGGAACCATACTTTACTTTTT
GTTCAATTCACTGCTGGTGTGCTTGCTTGTTCTTCACATCTTTTGGATGAGACTCATATTACGGATGGTTAACG
ATCTCATAACAAAAGGCCACATAACTGATGATGTCCGATCAGATTCTGAATCCGATGATGAGCACAAAGACT
AATCCTTCA
>TCONS_00006175
CTTTGTCAGTTCCAATAATGCCAATCTCAATGTTTTTGCCAGAGATCTGTCCCTCGAATCCCTCCTTTAGAGTC
AGTATAGCAGTGTGTACAGCATCATCGAGCTCTATATCCTCCGTCTACCTGATATAGTTGGGGACCCTTGTCAT
CAAATCTCGCAACCAAGAGTGAAACACCAAATGGCCTTACACCACCTGATTAGGTGAATTCCTGCATGACAG
CAGCAGTTTCCCTCACCAGCTGTGTGACAGGGATTGGTTCTTTATAGAGTCGGTGATATTGCTCAGCCTGCTTT
CTACTTTTCCGAACCAAAACTCGAGAATCAGGGCCCATCCCACTGTAGACAACTCCAATATTAGGCGTCAAA
ACCTGTATTTTCTGCACAGATGCTTCATCAACTAAGATGGATGGAAACTTCTTCTCCGTAGCAATTCCAACAC
CATTAGCAGCT

>TCONS_00006230
CAGGCACACTAGTCTGATAACGACTCGGATAATCAAAAAGTTTAACTGCAGCTAGCAAAAATATGCTTAGTA
AGACTTTCCACAATGTATCAAGCTCCCAGCACCCAAGAAGAGACCAAAAATGGCTGCACTGCCCAAAGTTGT
TTGTCCAATGTATCTGATTTTCATAAGGCCAGGCACCCTGTATCTAATAGCCTCATAAGTCCCATAAACAGCA
CCAACAGCGCCACCAACTGCACCGCCTACAGCAACCCCAGCAGTTATTCTCGCTAGACAACTATCTTTTGCCA
TCTCTCTCTCTCACACACACACACAGTGACTTTGGTTCTACTTCTCA
>TCONS_00006324
AGTCTTTCCACAAAGCTTATTATCGTATATTTGAACACGCATTTGACTTGGTGAAGAACCCATAAGATGTGAA
TGTCTCTGCCTAATCACATTCTAAACCATGATGACTACACGAGTTTTCACGAAAACTATTTTCACTTTTTGCAA
AAAGTTCACAAACAAACAGAACTACGCACTGCATAAATACCAGGAAAGCAGGACAGAGACAAGGAGTTCA
TAGCTGCTTTTCAAACAAAACTAGGATTATTGAATATATAATTGTGTAGCTACGGTTGTCGCCCATAATTCAA
AGAAAAAGAAGAAGACTACTGTATAACTTCCATGATCCACCAGATGCCACCAAATGTTGATTATTGACTCGA
GCAATTTCATGCTTTCTTTTCAATAAAACAGTCATTAGGTCTTCCGAGATGAATGGAAAAAGTCATGCACAGA
AACAGGAAGATGC
>TCONS_00006345
CGGCTTTCTATCGAAGTACTAGAATTTGAATCTTATCTTCTATGATTTTCATCCACTTCATTGACCGCTAGGCCA
CACCACCAACGATCAATTCTGTTTATATTTTCTCCTTGGTGTTACTAGTAATTGCCAAGGACAGAGCAGAATTC
ATCATTCAAAAGCAGTGCACAAATGTTGACAAACAATGTCAAGCAGTCCTGATGTCGATTTGGCAAAATAAC
ATGCCACTATAGAG
>TCONS_00006361
AAGTAAGAGGTTGTCTCAAGAGCAAAATAGGATGTAAAAATCGGTTTATATGAAATCCACCAAAACACACA
ACACCAGAACAAGTCCTTTTGGGCCTCAAACGCATCGAAATATTTGTAGAAAACAGAGAAATCTTCTGCTGA
GGAAAACAGCAGAGGATTTGTTGCATACACAGAACAAAAATCACTGAAAATCTTGCCGACATAAAATCAAT
CTCCCCTTCCTTTACACAAATAGCCTCTTTTCTTCCTTCAGTTCTTCAACCCCAGAAATAAATTAAAAGCCAG
>TCONS_00006451
CAGGTGGATACACCAACAATCGCTTACTGAATAACAAGTGCTCGCTCAACCAAAATCAAGACATATGGTCTG
ATCAATATCTAGGATACAGAAAACGCATAAGCAAAAGGAACAGAAACATTTCCACCCAGGTGAACTAAAAC
CAATAAAACAACATGCCGCCAAAGAGATTGAAAAGCTCCACAGAAACAAATCTAGATCAATCCCTGGCGTT
GTAGATCCGTATGTCTTTTTGTTGTAGATGTTGCCCTCTTGATCTTCATATTCTTCTTCAAGATCTGGGCGCCAC
TTATTCAGCCCTTGTTTTTCTTGGATTCTTTCCCAAAGTTGCTTGGCTTCTTATACATTAGTGATCACCATTCGTC
ATATTGATGATAGCTCCTTCCAGGCCATTCTGCCTTCCATCAGCACCATTAATAGCGTCAGCTGCTGCTCCTTG
CAGGCCATTCTGCCTTTCATCAGCATTATCATTGAAACCTTTCTTTTTATTTAGACCATGAGCAGTCATAAGTC
CATAGAACTTGCGCATCAGCATTTCTTCTTCAACCT
>TCONS_00006563
TCTAATTAACCGTACAAGATTGTCAATGGCAAGCTTACCCCCATTAATTAACAAAGCTATAGTACGATAAAA
ATAACAATTCAGAAAAGCAAACAAATTCTACCCATTTCTGAATTTACTTCTCAGAGGAGTTTGGTCCTCTCTTC
TTGCCAAAGCCAACAACAGCAGTAACGAAACGCCTATTATACTGCATCCTTTTGTGTGCCCGGCCACGGGGC
TTCTTTTTCTTATCTTGCTTGGCGACTTTGGGTGTTTGGCCTCTCACCTTACCAGCACGAGCCAATGATCCGTGA
ACCTTTCC
>TCONS_00006572
GTGCTTACAATATTAAAGAGGTTGAAAAAGTCACTAAGACATCTGCATTAAGATAAGTACTTCTCCAATTTTT
CAACTTCTTCTGTGCTTCCAATAAACAATGGAACTCTTTGATGTATCTCAACTGGTTGAATATCAAGAATTCTT

TGTTGGCCATCTGATCCTTTGCCACCAGCTTGTTCTACAATAAAGCTCATGGGTGCGCACTCATACAAAAGTC
TCAACTTCCCGTTCTTGCTCTTCTTGTCTCGCGGGTAACCATATATACCACCATATAAAAGTGTCCTATGAAAA
TCACCAACTAAACTGCCAATGTACCTAGCCGAGTAAGGCTTACAATTAGGACCGGGTTCCTTCAAGTGATCG
ATGTAATTCTTTAACCTGTCATCCCATAGCTGATAGTTTCCTTCGTTGAACGCATAGATCTTTCCGGTCTTTGGT
ATTTGAATGTTTTCTTGAGTCAGAATAAATTCTCCGAACATTGGATCCAATGTGAAAGAAAAGACTCCTTTTC
CCAACGTGAGCACGAAAATCACAGAGCTCGAGTACATGCAGTAGCCTGCTGCTAAAAGGTTGTTCCCTGGTT
GGCACACATTCACTATGCACCT
>TCONS_00006692
GGTTTCTGGAGATTGCTTTACTGATTCATTCTCCTGCTGAGTGGCTTGAGCCCTAGATAAGGTCTTCAGCTGGC
AGTTTTCCATGTTTGAGAGCAGCTGCATTACAGTTTTCAGCTGAGGAACACTTTGTAACCCGTTTTCCATATGA
GCATCCAATGCTGAAATCACCTGAGGCAACTGCTCCAATACCAGCTCTGTATTCTTGATTGTAGGTGTCTTAG
CAAAAAGAGTCTTTGGTAGCTTCATCAGTTCGGCCTGAGATGGGGAGGACTGAAGCATGAGATGGAGGAGAT
TGGAAGTGGACTGTATAGACTGAGTGATTTGATCAGCTACCATCGTTTTCGCTGACCCATCTCTGCTGCTGCT
>TCONS_00006967
GCATCAAAGTACTAATAACTCTTGATATACAATATCACAAACTTTAAATCACATTAAAAATTCAAACATACA
ACCAATGTAATGAATACAAACACTAATAAATGAAACAGCCTGTCCAGAATTCTGTACTGAAGCACGCAAGA
TTCACGAAAACTTAACCCAAAAAAAAAAAAATTACAACTTTAAAAGTAACACATCTTGATTCATAGGAACCA
GCAACATTAGCAGAAATGGAAGTTCCACATCTTTACCATGTGGGGAAAGGTGATCAGTCGTTCTTGGTAAGG
ACCCAAATAGCATAGATAATACCAGGCAGCCATCCAAAGAGAGTCAGCAAACAACAGATCCAGAATTCACA
CTTGCAGCCAAATTTGAGAAAGACACCAAGGGGAGGCAAGATGATAGCCAAAAGAATGTCTATGCAAGTCA
TCGCACCCATATTTTTTATTATTCAATAGAAATTACACAGGAAAAGAAGAAAGGACAAGGGGCATATACTGG
GGAAAGAGGATTGAAATGGGGAAGTGGCTGCT
>TCONS_00006983
GATAGGCTTTTCTCTCTCACGCATACAATCTCAGATCTGTGTATGTACAGTAAAAAGATAGACGTATATTTGA
TGTTCAGTTTGCATTTATCGCGGCGGTCTCTCATTCGCTTGGTGCAGGTCGGGACCTGCCTCGCCGGCGACAAT
GACGTCTGCTGACGGTGACGGTGGCGTATCGATAGATATACATGTTGTTTATGACGAAGTTTTGGTCCCGCCTT
GCATCAACTGAATCGGAGACTGCGGTGAATTTAATTTGTTTCAAGTAAATCAGCTTTGTTATGGATCCATCTA
ATATCTACCGCTTGTTTCCTGTGAGCGTATAACTCCAATGATAGATCTAAGAGGATTTTGCGAATTCGATGCC
ATTAGTTAAATGAAGATGTTTGATTTTATTGAGTACAGTTAATGTTATGATTTAGGTAATCTGTAATTAAGAAA
CGTGATAGATTGTATTGGAAGCTTTTCTGCATTAATTCCCTTTTCAGATAGTTTAGGAGGATGAAGTAACTAA
>TCONS_00006990
AGATACGAAAGTAATTTTAGTGGCCCATATGATGCAACAAAATAACATGCTCTGATTTTAAAGATCCAGAAT
AAATTGTTGCATTAGATTGATCCCTTTTCCGCTGGAAAACTAAGACCAAATCTCTCCATCCATACATATACGTT
AACATAGACATACTGCAAAAAATTAGAAGAGCTGGAATGCATTTAGCGGCCTCGACCACGTCCACGACCAC
GTCCGCGCCCGCGACCACGTCCCAAAGGCTTTCCAGCTGTTGGCTTCTTCGGCTTCACCCTAGGTGTTTCTTCC
ACCAGTAAAGTCTCAAGATTTAAGCTGTCAGGGAGAATGTAATAACGGATGTTGTTACCCCTCACACTCAGG
TGATCCAACGTCACTGGATTCTTTCCCTTTAGTGTAAGTTTGACAGCCTTCAGGTGTGTGTTCATGCTAACATC
CACACCTGTAATAGTTCCATGAACAACAGTGCCGTTTTTGAGCTCAATTGATACAGTCTCGTTGTTCAACTTCA
TCAAAAATCTG
>TCONS_00007018
AGTCAAACTATAATCAAACGAGATGCAATCCAGGCAAACAGAAATCCTCTCAAGAAAGTCAGCTTAAAAGA
GAATAGGTTCAGGATAATCTCCCATATATCTTAGGTGGACAAAACTAGCAAGTTGCCCCAAAAACACAAATC

AACATCCTTACAAGTAATAGAAGAACTAAACTAAGCTCCTTCATAGAAGATAGTAGATTAATTAATGCAGCT
CCAAACAACAGACAACCACCATTCTCTGGAGCTGCCAGAGAGATAGACAGCAAGAGGAACAGGGCAGCAG
ATTCACCCAAGAAAGCATCCTAGGCATCTAGAACCCTGCTCTACTTAGATCTCTGTCTCATCTTTCTGCGCTTC
CTCTTCAGTCTCCTCATACGCTTCTTCTTCCACTTGGCCCTCATCCTTGCTCAAATTTCACCGATGGCTCACTCT
TTATCTCTCGACTTTCGCCGCTTTCACC
>TCONS_00007083
GCGGGTCGGATGGAGAACAAATGCAAAAACACAAATTTGTAACTTTAAAAATTGCGCCAGCTGAGCTTGCG
AAATCGAGGGGACGGGGCAATGAAAGTGAAAATACTGCGTATCCTTTTGATTGTTTTTCTTATATACCGTGAC
GCGTGTCCAATAATCACATGGTGGCTTGAATCTGATCTATCACACCAGTAGGTGCTTCATGTTTGTTGCCAATT
ACTAGAATTAGCTGGGTTAATATTTAGCCAACTGCTTCAATTATGGCCTGTCAGTAATTTTTTTCA
>TCONS_00007103
CTTATTACAAGCGTACATTACTGAGTCTGTCACACCATGCTACAGGGATCTAAGCAACCTACAAATTCCCTCC
ACACCGAAGGTCTAATATATGATGTAAGGTAGTAATGATACAAAGATCTGTTTAGGCTATAATTCCTTCTAGA
GGACAAGGCTGCAAATTTTTAGGACCTAAAATCTAAGATTTTACGTACAGATATCTCATGAACAATCAGAGT
ATCTACCATTGACAACAACACTACAGTCACTAAAGAAAAGAAACAAGAGCAAAAGGTCAGAGAGAGAACG
AGAGAGCCTATTAAGTGGATGGCATAGAATTGCTCCATGTCGATACTCAAGGGTAGGCCTCATCAGCCTCGTT
TCCGTTTTCAGTTACTTCTGCCTCCACCTTCTTGCTTATGTCGCTGTTCATCAAAGCATCTCCATCTCCAGTGCT
CTTTGTTGCTGCACCATTTTCATCTCTCTGTTCTTGTTCCGGTTGATGCTTCTTTTCCTCAGCAGCGCTACGATCC
TCTTTCTTTGTTTCAGTAGGTTTCTGTTCCACACTTTCTTGAACTTGTACATCCTTCCCTTCCTCATCCTTAGTAG
CTTCAGGAACATCAGCTTCTTTCTCGAGAGGGGCCTCTTTCAC
>TCONS_00007147
CTTGCTTAGATCTCTCAATGTTCCCTTGTTTGCCCACTTTAGTCCACATGCATTGCAGAGTGTCCTTGGTCCATC
TGGCCCACGACGCATTGCTGGCGTACAATTTTCACTCACACCACAATGGTGACACTTTACAGGAACAGTTTCA
GAATGCTGAGTGCCATCTCCTGCAAGGCTACTCTTGGCTGAGTCGCAGCTAGAAGATCCAGAACTTGCTTTCA
AAGATGCAAATTGACCATTCTTCCGATGCATCCTTTGAGCAACCTCCTTCCTGACACTATATCTAATTTTCTTG
CCAAAACATCTCTCCTTTCGCTTCTCTCGGAATCTAACCAAGGAGGCAAATCTTCTTGAAAGATTAACATGTT
GTCGAAAGGAACTTCAATAGTGGGCACAGCAGTAGGAATGTCACATTCCCCCAATAGCAAGAGCACTGCTTG
CACCTTCTCCTGAGTGACAGCAGGAAAAAAATACACTTCACCTTCAAAAGCAAGAGTAAGCTCACTACTTCT
AGAAACTTCCACAACTCCACCACCTCC
>TCONS_00007148
CTTGCTTAGATCTCTCAATGTTCCCTTGTTTGCCCACTTTAGTCCACATGCATTGCAGAGTGTCCTTGGTCCATC
TGGCCCACGACGCATTGCTGGCGTACAATTTTCACTCACACCACAATGGTGACACTTTACAGGAACAGTTTCA
GAATGCTGAGTGCCATCTCCTGCAAGGCTACTCTTGGCTGAGTCGCAGCTAGAAGATCCAGAACTTGCTTTCA
AAGATGCAAATTGACCATTCTTCCGATGCATCCTTTGAGCAACCTCCTTCCTGACACTATATCTAATTTTCTTG
CCAAAACATCTCTCCTTTCGCTTCTCTCGGAATCTAACCAAGGAGGCAAATCTTCTTGAAAGATTAACTTGCA
TGTTGTCGAAAGGAACTTCAATAGTGGGCACAGCAGTAGGAATGTCACATTCCCCCAATAGCAAGAGCACTG
CTTGCACCTTCTCCTGAGTGACAGCAGGAAAAAAATACACTTCACCTTCAAAAGCAAGAGTAAGCTCACTAC
TTCTAGAAACTTCCACAACTCCACCACCTCC
>TCONS_00007220
GGATAACCAACGACACAGCTTATTGTCACTTCACATCTTAATTGAAAATTTGTATAGAAATTAGCAAGATCTG
CGCGAGACAACCATAATTAAAGCAGCCAAATACGGGTCCAAAGAATCCAAGTACTCATCACAGGCAGCATG
TGATAAGAATGTGAATTCAGTTAGCAGTGTGCTGACTTGGCTGTCCGGGATCGATTAGACTTATCTCCATATC

ATGAGTTGCCGACCAACCAGGTTCATATGTTTCAAGAAATCCAATCTTAGCCTTATTTGGGAGATCATGTGGG
TGAACAAATGGAACTCCCGCTGGCCATCCAATCTCCATCCTAATGTTTGTACAGGGGCTATCAAGTGACTGGA
TGTGACTTTCACAGTCCTTTAAGGTGCAGAACCTCAGCTTGATGCCCTTTTTATCAGCTATATCTTGAACTCTCT
CTTTGAGAACTCTTTGTGCCTCCAATCTCAACCTCTTTGAAGGTGGTAGAGGTTTAGATCTTGAGTTGCTCTTTT
TCCTCCTCATCATGGGCTTCATAGATCCTGTATCAGCATTTCATCGATCCAAGGACCATCTTGGGCTGTAGGA
AAGTTGAATGTTGGGAAATTGCTCCTGTTAAGCAACTGATCAGTTGATCTCTGCATAAAATCGTCAACTGGTA
CAGGAAGCTGGAATTGAGCTTCAACCCCATGACCAGCAGCCCGCATCTCTGAACAATTGAGGTTGGTCATGG
ACTTGGGCATAAGTTTAAAAGCAAGAGAACATCGTCCATATATGCTCCCCTGTTGATTACCAGTATCTGTACG
ATGAAGTGCAGGGCTGATATAGCCCGCAGTTGCCTGATATAGAGCAAGGCCAGGATAAACTATTGCCTCTTG
GGGACCAAGATCACCATCCACAAGAACCCAGTGACCATGAAAATCTCTCACATGTAGACCAGGCTTATCTGA
CTTAACAATCGTAACAAGGCTTCTGTCAACCTGATGTTCATGATCTGAGAACATCACTAACTGACCATCCTCT
TGGGTTGCCAGGCTATGGTGCTGTGCACCCTGAAAAGATGGTCTGGCATGACAACAGACGGATAACACAGAG
GATGATATTTCTCGGTTTCTGAGGGGAACACTATCAAGTATTTCAGTGAATGGAGAGCTGCGCAGATTTAAAT
AGAAGCTGATGGCATCCAATATATCTCTTGCTGCTCTTCCAAGCAGAGAGAATACATCCAATAAACCAGCAG
GAGGAAATTCAATATCACTGCTGGGTTCTGTAGGAGTGAGCCCTGGCCTGAAATCATAAGTTTCTTGCCATTG
TTGAGGATCTGCATGGTAGCCAGAAGTCTTACACCATTCTCGAGAGTCATCAGAATGAATAACATCAGCGGC
AGGATAAGAGGGTTTGTGTTGGAAGTAAAGTCGGGCAGACTCTAGACTAGACCTCAAAAGAGCTCCATCACT
TGTGGATAGCTGGATAATAGCGGCTGAATATTGAGCCAATGACTGTGACAAAGTTGAGACTGATAATTTATA
CGAATCAGAAGGAAGACCTTCCAATGGTATCAAATCAATGAGCTTCACACGACCCAGAGTTGGCAGGCCATT
GCCTGCCATGGAACCCTCTAGAGACACTGATTTAGTGATGTTTGTTCAAATTGAGACCACACCCACTGAGTAG
CAAACAGAAGACACAGTCAATTCAGAAAATAAAATATATATAAACAACAAGCAAACTCTCAC
>TCONS_00007221
CGACACAGCTTATTGTCACTTCACATCTTAATTGAAAATTTGTATAGAAATTAGCAAGATCTGCGCGAGACAA
CCATAATTAAAGCAGCCAAATACGGGTCCAAAGAATCCAAGTACTCATCACAGGCAGCATGTGATAAGAAT
GTGAATTCAGTTAGCAGTGTGCTGACTTGGCTGTCCGGGATCGATTAGACTTATCTCCATATCATGAGTTGCCG
ACCAACCAGGTTCATATGTTTCAAGAAATCCAATCTTAGCCTTATTTGGGAGATCATGTGGGTGAACAAATGG
AACTCCCGCTGGCCATCCAATCTCCATCCTAATGTTTGTACAGGGGCTATCAAGTGACTGGATGTGACTTTCA
CAGTCCTTTAAGGTGCAGAACCTCAGCTTGATGCCCTTTTTATCAGCTATATCTTGAACTCTCTCTTTGAGAAC
TCTTTGTGCCTCCAATCTCAACCTCTTTGAAGGTGGTAGAGGTTTAGATCTTGAGTTGCTCTTTTTCCTCCTCAT
CATGGGCTTCATAGATCCATCTTGGGCTGTAGGAAAGTTGAATGTTGGGAAATTGCTCCTGTTAAGCAACTGA
TCAGTTGATCTCTGCATAAAATCGTCAACTGGTACAGGAAGCTGGAATTGAGCTTCAACCCCATGACCAGCA
GCCCGCATCTCTGAACAATTGAGGTTGGTCATGGACTTGGGCATAAGTTTAAAAGCAAGAGAACATCGTCCA
TATATGCTCCCCTGTTGATTACCAGTATCTGTACGATGAAGTGCAGGGCTGATATAGCCCGCAGTTGCCTGAT
ATAGAGCAAGGCCAGGATAAACTATTGCCTCTTGGGGACCAAGATCACCATCCACAAGAACCCAGTGACCA
TGAAAATCTCTCACATGTAGACCAGGCTTATCTGACTTAACAATCGTAACAAGGCTTCTGTCAACCTGATGTT
CATGATCTGAGAACATCACTAACTGACCATCCTCTTGGGTTGCCAGGCTATGGTGCTGTGCACCCTGAAAAGA
TGGTCTGGCATGACAACAGACGGATAACACAGAGGATGATATTTCTCGGTTTCTGAGGGGAACACTATCAAG
TATTTCAGTGAATGGAGAGCTGCGCAGATTTAAATAGAAGCTGATGGCATCCAATATATCTCTTGCTGCTCTT
CCAAGCAGAGAGAATACATCCAATAAACCAGCAGGAGGAAATTCAATATCACTGCTGGGTTCTGTAGGAGT
GAGCCCTGGCCTGAAATCATAAGTTTCTTGCCATTGTTGAGGATCTGCATGGTAGCCAGAAGTCTTACACCAT
TCTCGAGAGTCATCAGAATGAATAACATCAGCGGCAGGATAAGAGGGTTTGTGTTGGAAGTAAAGTCGGGC
AGACTCTAGACTAGACCTCAAAAGAGCTCCATCACTTGTGGATAGCTGGATAATAGCGGCTGAATATTGAGC

CAATGACTGTGACAAAGTTGAGACTGATAATTTATACGAATCAGAAGGAAGACCTTCCAATGGTATCAAATC
AATGAGCTTCACACGACCCAGAGTTGGCAGGCCATTGCCTGCCATGGAACCCTCTAGAGACACTGATTTAGT
GATGTTTGTTCAAATTGAGACCACACCCACTGAGTAGCAAACAGAAGACACAGTCAATTCAGAAAATAAAA
TATATATAAACAACAAGCAAACTCTCAC
>TCONS_00007319
AGCTGAACCCAGAGCAAAATCCGAAGATCATTGGATGCCAACGTTGAATAGACACCGTACGAAGATCCCGG
TATTTCAACAAAAAGTTCCAAAGCGCCTGCCGCTGATGATGAACCAGCCATCACCAGGTCTGCCGAGTGATA
GCATAATAAACCCACCTAAGTGGAGGAAACTTCCTCTTCTCTTCCCATGTACTTGGGCAATTGCATTTCCCAG
CTATCACAACGAATAAATAACTTATCCTGCATGCATCTAACAACAAAAAGAAACAAAAGAAAATACTAAGT
CTAGCATAGTTGCAAAGCTTTAAACAACTTCATGTAGGTGGTGTGTCC
>TCONS_00007532
AGGCTTTCAAAGCATCCTGGAAACTCTAATTGTCCAGAAATTTGTCAACTCTATTGTGCCAGCTGAGTGTGCG
AAGAGCATGCTACTGTCCCTCGAGGGTACCAAGTGCCTCCTTTAAAAGCTTCTGAGCTTCTTCCCTTCTCCGCT
TGATATCCTCTATTGCTTGTTTACGTCGTTGTATTTGAAGTTCTAGAATATGGATAAGTGTAGCTCGA
>TCONS_00007533
GAGGACGCAAAGAATTGAGTAGGTGGTGCAGGTTCTTGAATATAAGAGATATCTCTTCCACTCTCCGGGCAT
ATTGTGAAGGGCGCTCCACAAGAACATCAGCAAGCTCCAATATATGTAGCTGCAATTCTCTGTTAAGTGCTCT
TAATTCTTTCTTGAAATCAACAGTAGGCCCTTTTGGGTATAATTGACGAACACCCTGATCTTCCAAACTTGGGA
GTACATCATCAGTAGTGTAGTTGCTACCAAAGAGGAGATAGGTGCCGTCAATGGGAGGGGGAGGTTCAGGA
GCAGAGTTGGGGTCTTGAAGATAGTCCTTATACAGCCTGTAATACGGCGGCGGCGGTGGGTATGTCGCTGTCG
ACATATCTGATCTGACTTTCACTTAACCAACGTTTCTTTTCAACCAAAAATGGAGATTTATCTCGGCGTTGAGT
CGACACTTGGACAGAGAAATATCGGACCTCTGC
>TCONS_00007534
GCAACTCTCTGTCCAACTCTGTCCTGACTCCAACAGTAGGCCCTTTTGGGTATAATTGACGAACACCCTGATCT
TCCAAACTTGGGAGTACATCATCAGTAGTGTAGTTGCTACCAAAGAGGAGATAGGTGCCGTCAATGGGAGGG
GGAGGTTCAGGAGCAGAGTTGGGGTCTTGAAGATAGTCCTTATACAGCCTGTAATACGGCGGCGGCGGTGGG
TATGTCGCTGTCGACATATCTGATCTGACTTTCACTTAACCAACGTTTCTTTTCAACCAAAAATGGAGATTTAT
CTCGGCGTTGAGTCGACACTTGGACAGAGAAATATCGGACCTCTGC
>TCONS_00007556
GTGAGTATCTCCAGAATGAGAAGTTCCAAAAAATGACAAACAATAGTAACTTATTTCCTGTTGAAAATGGCA
AAAGGAGCTAGAGGTTTTCTCCAGACATATGAAGAAAACAGCTTTAGTATCTGTGTTCTAACTTCTCCTTTTCC
TGGTAGTACTGCACATATGAGTAGGTTCCGACGAGGGGAGCGAGAAAGAGAGTGGCGCTGATCCAATTTTCG
GAAACTTTGTGATGGATCTTACCAGGAAGATCCTTCCATAGACCTGGCATTACTTTCTGCTGAAATGGCGATA
AAGCATACACAACTGCCTTCAGCTTCACCGGTTGCTTCCCCATCGCGTATTTGCTCCGATCGTTCTT
>TCONS_00007564
TACAATAGACGAGCAAAAAACATGGCAAGGCGCTGCATATCGTTTTCGAGGACTCTTCTCTCTAGCAGTAGA
CCATCAACAGTCAATTCCTCTCCAAGTGTACAATCCCTTCTCGGCCAGCGCCTTCAGTCTCGCCGTTCTCTTTC
CACCATACTCACAAGGACTAATGGGGTACTGGGTTGCACCCAATCGCTGCTACCGCTTCACAGCGCGGTCGC
AGCTAGCCGTCTGACATCACACATCCAAATGGAGGCGCGCACTTGCTGCCAGCTGTCTCAGGGTACTTAAGA
TGCATTATGTATTAGCTGGAAAAGCTGCTGTAACAAACATTTTGGAGGATTTTCGCTGAAATTTGCCCTGGAA
GTGCAAAACTGTAAGCTCTAAATTCAATTTGCTTTCCTAGTTATTTTTGGGCTGATGTGGGAAATTCTAGAATT
CTTGAAAGTCCTGAAAATGATAATTCTCTAGGAGAAACTTA

>TCONS_00007565
TACAATAGACGAGCAAAAAACATGGCAAGGCGCTGCATATCGTTTTCGAGGACTCTTCTCTCTAGCAGTAGA
CCATCAACAGTCAATTCCTCTCCAAGTGTACAATCCCTTCTCGGCCAGCGCCTTCAGTCTCGCCGTTCTCTTTC
CACCATACTCACAAGGACTAATGGGGTACTGGGTTGCACCCAATCGCTGCTACCGCTTCACAGCGCGGTCGC
AGCTAGCCGTCTGACATCACACATCCAAATGGAGGCGCGCACTTGCTGCCAGCTGTCTCAGGGTACCTTTTTC
TGTCGCACTTGCCCTGATCGGTAGTTTTCAATTGTACTTAAGATGCATTATGTATTAGCTGGAAAAGCTGCTGT
AACAAACATTTTGGAGGATTTTCGCTGAAATTTGCCCTGGAAGTGCAAAACTGTAAGCTCTAAATTCAATTTG
CTTTCCTAGTTATTTTTGGGCTGATGTGGGAAATTCTAGAATTCTTGAAAGTCCTGAAAATGATAATTCTCTAG
GAGAAACTTA
>TCONS_00007572
GCTTTCCACACTTTATCAATATTAAGCATCATGTCACCACATTCATGTTCATTCCAACCTTCCAATGCATGCAA
AATTCCAGCTATTCTCCAGGCACTCATCACTCTTCTTGGCAGCCAATTCTCACAAGAGCCTACATTTTCAAGAT
ATTTGGAGTAATCATAGCTGGTGTGTTGAAGTAGAAGCAATCTTTTCGAGCTTTTCTTGGTGGAAACTGTGAA
AAGGGAATAAATAATGTTCCTTTTGGTGCTTTCAATTGTTCATCTTCTCTCAATCCAT
>TCONS_00007587
ACCAAATTCCAGATTTTTTGTATTATGCAGTAGTTAAAGAAAACAAATAAAGTAGAAACCACTAGGATATTA
CCATCTATCCGTCTAACCAACTCCTTTAGCTTAAACACAAGGCGCTGGATTCCTGGTTGAGCAAATGTGTAAT
GGTCTTGCATCCCATCAAGCAATTTTGATAGACACCAATAACAGTCAGCTTCTATGTTAGATATTTTCTCAGA
AGGCAGATCAGACATCATCCATTTGTCAATGCTCCCTTCTAAGTGTTCTGACAAGAAAACAACTAAGAATGG
TGTTGCAAGATCATTTATTCCCTGCACATATCCACTGGCAGGGTGCCGGATGGCCCAAATGTAGAGTATGCGC
TCCAGGGATTTCTGAACTTGTGCTTGTTGGAAAAAAGAAACATCAGGCACCGTTCTAGGGCAGTCAACAGCA
ATCTGCCGAAGCATGTTGATCTCTTCATCTGTGCGCTCAATATCTGAAATGTCATAGTACTGAGCAACACAAT
CAAGATAATCTAGACGCTTTCTTCTCAGAACTCCCTCCCTTCTATCTGAATTAGGGGGTGCATATCCCTGTTGT
CAGCTCATTATTTAAATTTCATAAGAAGCAACAAACTATGTCTAATAAGAAAACCCGCAATCTAGTTTGGCA
GGTTTTGTAAACAATGTAGGAAGCAAG
>TCONS_00007609
CTTGAACTGAAAGGAAAACATTCATTCCGCTTCCAGGGAGTCTCCAACAAGTTGGTCAGTTCTCTTTCTGCGA
AATAAGGCCCTGACGATCGAGAAAAGTGCAATAAAAGCTACTTCCATGTGCTCAGAGCTGCTTGTCTTGATG
GAAACCTGACCCATCTTCCGGTTATTGAGGTTTGCGCTTACTGACATGTTTGTACCTCGACTCACTCTGAAGTC
AGTTTGCAAGTTTCCGCTCAACACTGTATCTTTGTTAAGCGAGAGAACTGTCATCGAGAGACTCAGACTTTCA
TTTCTCACTGGGTAATCTTTTCCTCTTAAAGTAGCTCCAAAGCTTCCACCATAAGCCGCTTGCCCAGCACCCCC
CATCCGACCAGCATTCACAGTAAACTTCAGTCTCTTTCCAATTGAAAAACTATCTTCACACTTGGTACCAAGA
AAATACTTATCCCCGAATGGTATTACAGAAATACCACATTCAGTGACATTATTTCTTAAGTTTCTTACCTTTGC
ACTACTGTGAATAGTGCAGATCAGCTCCTTGTTGGCAGATTGAATATCGAGACCTACCGCATAAGTGGGCCC
ACCTGGATTGGTGAATGCTGCAGCAAACTCGGATTGAATACTGAAGTCTCGTTTGTCCTTGCTCATTTGTCCAT
TGACTGAGGCAATGATGTTTTTTCTTATTTCTGCACTGCTCTCCAGGTTAATGCCATCAAAGCTCACATCATGG
TCCCATCCATTGGTATCAAGCACTGGTCGTGCAAGCCACTGTTCGCTGGTAACGAGACACCTGTATCTGTGGA
CAGGGCAATCTGAATCGAAGCTTGGCGGAATAGCCATATCTGGCAACAGAACCGGCTCTGGAGAGCCCTCCT
CCTGGTTATCAGACTCATCATTCGGGGCTTTACTCTCACTCGAAGAAACTCTCTTTTCTCTTCGCCTACGAGCTT
CTTCTATCAGTTGTTTTTTCAAATAGAGGATTTCCCGGTAGTCTAATTCGTCAAGATAATCCTTCTTCTGGGAAC
CACTCAACCTTTCAAACTGAGACTTGGTCAGTATCCGAATAGGAGGTAATTGATAATATTCATCTTCATCATC
CGAGTCTAAAAGAGGAACCTCGTCAATTTCATTCTCAGATCCACCGTGCCTTATCTGAGCACGATGCTTTAGA

AAGGACGAGAGAAGATGTGGAAGAGAAGGCAATCGTCCGACATTTGATGGTCCCACCTTTAGGCTGTCTTCA
AAGTCCAACAGGGTATTAACATCACTTAGAACTTTGGTGCATATGCACAGCAACAATAACTGAGACTTCCAC
GCCTGCCCATTAGGTAGAATTTTCTCTCCGGCATTATTGGTTTTGCAATTGGGATCATTCTCCACTAAAACTAC
AGGATTTTCAAGCTTTGTGTCAGAGACTGCCTGGTGAATATAATGTTGCACCAAATCTGTGCAGGTGGTGATG
AAAGATTCATAGTTAACAGGATATCCATTTATCCCTTCGGGAAGATTGAAGGAAGAATGAGTCATGACAATG
ATGGTATTAAACCAAATTGCAGGACCAAAGACTTCCGTTATAAGCTTCAACAAGGGGAAATCACTGTAGCCT
GTGTTGATCAAATCCAGGCGCTCAAAATACAGTACCATGTCAGGTTTAGATTTCCTTAAAAATCGTTTCACAG
AACGCAAAATCTTCTTGTTTTTCCTGACATTACTGGGTGAGGGAGGCAGTAAACCAGGAGTGTCAATAAACG
ATACTCTGATGCCATTTACTGTTCCCGCAATCTCTTGAATACGGTCAGTAGCGGGCTTGAACGCGTTGGTTGTT
GCTCTTGATTGGTCAAATATGGAATTTATGGTTGAACTCTTGCCAACTCCAGTCCTACCCAGTACAAGGATCTT
AAATGAGAAATCCAATTGCGGTCGGCCAGCAGCTTCTTGTTCTGCTGCTATTACACGTGCTCTCTCAATTTTAA
GATTTGCCCTTTTTAAATCCGATTCTCTTGCCCGTATCAGTGATGCCAGCTGGATTCGGTATAAGACCTTTGAT
ACCAGCAGGTTGTCCTCGGACAGGCCAAAACGTTTAAGAAGCCGCAAGAACTTAATCTGAAGAGCTTCAATC
TTTACCACTGGACTAGGCTTCTCATTGACACTAAAATCTGACTGAAAAGAATCCTCCACCATCCGTTGCTGTG
AATGGAAATGATTTGTATTCTGCTGATTATCATTGGAAGATTGGGTTGTATTGGCTAATGCTGGGTGATCAAAC
TCTTGATCAGGATGTTCCTCTGATAAAAAGCTATCTGATGCTAAAAGTGGTCTCGATGAGGCTACTGACTTGG
ATATCAACTGTGATAGGACCCAGTCCTTCAAACTCATCATGTTTTCTGGTTAACACTTAATTCCAATCACCATT
GAACAAGAGCTTCAGAATTACAAAAATGACCAAAAAATACAAAAACCCAAATTCCCAGACCAAGATTCTAC
TTTTCTGGTTTATTTACTGAGTTAAGCAAACCCCATTTGAGAAAAATCAAGATAAATCAAAACCCAGATGCTA
AAAACTGGCAAATGGAACGAAATCAAAAGGGTATGAAATTGAAAATGAATTGGGTATTCTTGAAAAAAGAA
AAAGATAGAAATGGGACCCTTTATACCATACTTAGGAGGGAGTTTTAGAATTTTGTTCTTTGATCGAAATTGA
TTTGTGGGAAGACGCGGTAGAGGGGGGTTTTGGTTTTTCAGATAAGACACAGTGACGATGTTGTATTGATTCT
CCG

>TCONS_00007681

CATGATCGATGGCAACAGAGATAATGAGCTCCCAATATCTTTGGAAAACCCAATTATCTTGAGCTGGCAAAT
TTCAGCAGGTAGTTCAACAAAGTGCTCCTCTTTATCACGCAACTCTGAATAGCCTTTCTTTCGTAGTAAGTTGT
CCAAACAGAAGAGTTGTTTTGCTTTTATAAGTGGTTGCTCAGGGTACGAAAGACGGATACCAAAAGTATCGT
AATAGTGTTCGACATGGTTTTTTGAATCTTTATATATGCTGTATGCATTGCTTTCTTTGACAACATCAGATATGA
AGAAAAATGTCTCCTTGCATGGAACATATACCAAACTATTGGCAACATCATGTACGCTTTTGGAACCATTGGC
AAGTTGCAACTGCTCATCAAATTTGGATATCTCACTATTGCCTGCATATACTTTAGTCCCAAAAATTGGTGATG
ACAAGCATCTTCTGACAAGCTCCCAGTCCACGGAAATTTTATTGTGTCCACATAAATTGACGGGAAGAAGTA
GGTAAGATTTTGAAGCTGAGTCAACATAATCTTCTTTTTCCAAAGATACGAATTCTGAAATGAACTCAGAGCG
GTCAAGAATGATCTTGAGGAACATTCTCTGGAACTTCTCTGCTAGTTGTACCTCATTGTTCTCAAAATTAGTAG
CTCCTGATGGAATAAGCTCTGTCTCCACAGATCTACCTCGAGCCAAGTTGAGATCAAGTTTCATTCTCTCAGC
CTCTTGAGGAAGAGGTGCTTTCAGAAATAGGCCAAACTTTTTATAAACCCTATCAATTGGACAAGGTGAAAA
GTTGATGTAGTACGAGTTAAGGCAAACAGGACTCTCTGTCTCAGTCCATGATTCTTTAAAGGCAGCGGGAAC
AATCATCTCATGGAGCTCTTCTCGTGCGTCTTCGCCATCTGAGCTTTCTGAGTCAGAAAAAACATGAATCAGG
TCTTCATCTGCTTGATCCGGCAAAAGATAATCAGTGAGTGCTCCAAGTTCATGCAAGCTCTTGCAGGCTCTTA
AACAGGCATCTTTCTTAGCTGCTTCTATAGAAGATTGAGGAGCACTCACGATTTGATGCATTGGAGCGTTTGA
AGGCAAAACTAATTTGCAAATAGTTCCATCTATGTCATCAAAGTAGAAGAACTGCGGCTTGGGGCAGAAAAA
CTCGTCACGTGGAAGCTTGGAACAATAATGGTGAAGCAACGAAATGCTTGATGCAGAACTGATGGTTGCACC
AGTCATATCTACTTTGTATATATTTCTTGAAAATCTATAACTGCCGTGCAAGATTTCCTGGAAGAATCTCAT

CATTCATCCGAGCTTCATTTCTTGAAAAATGCTCAATCAAATTAAGCTCCCTTTGGTTATCACTATCAACCAAA
AATGCATATTCAGACTTTGGCATGCGGGCACGCCCTCTAGACTGTATAAAACTGGCAACAGTTTCAGGAAGA
TCAAATCGTATAACGAGGCAGCATGTCTGGATATCAAGTCCTTCCTCGCCAACTTTGGTGGCAACCAATAAGT
TGAGCTCACCAGAACGGAACTTATTGAGAATGATATTTGTATTTTTTCGTGACATACTCTTCAATCCCGAGTGC
ACTCCTACAAGAAACCCGCATTTCCAAGAAGATAAAACTTTCAGATGCTGAAGCATATAAGAAAGAGATCTC
GCAGTCACGATCCTATTGACGAAAATTATGCACTTCATATCTGGTTGTACCCCGAAGTTGGAAAGGATTCCAA
TTAGACGTAAAAGCTTTTTGGAGAAATAAGGCTCCTTCAGAACTTCCACACGTGTCAAATCAGGATTCATACC
ATCTTTAGCACAACCAGAAGTAAACACTGTGGCGACCTGACTCAGATATCTGTCACACAAAGAGTCATCACT
AGCATTAACATCAGCTTCCACCATTTGATGGCGCTCATAGTGATCCCCCTTTAAGAGGATGCAACTAGCCTGT
AATGCCCCAAAGACACCTAGGTTTTCCAGAGAAAAAATTAGATGTCCATGCAGTCTCTTGAGCATCTTTTTG
TGTTCCTGAGAGTGCTATGATCAACTGCTTTCTTGTGAAGCACCATCACACACTGGTGTTTTATCTCTTCAAGC
TTCTGAGAGTAAGCTTTAGTAAGACACGCGGTGCCAGGACCATAGTAGTATACATTCACCTTAGGTGATGCA
ACAAACTGTTCCAGTTCATCCTTGTCTTCAACAGAATACACCTTAGAGCGAAGTAAGGTCTCAAGGCCTTCAA
CAGTAGCACCTTTTCCTGATATTGGAGATGCAGTCATGCCAAATATACGGGGCAGTTTTACTACATCAGGTTT
GTAAAATATCTTCATAATCTCAGCATAAGGATGATCACTCTCAACTTGTGCATAGTGGCACTCATCAAAGATC
AGAAGAGCAATAAATTCAATCCTGATATAGCAGTGGGAAAGATTATGTAATAGTATTTGAGGGGTCATAACA
AGGACCTCATACTGTTCCATTTCTTTTTCCCAGTCTTCGTGGCTCTTCAAATGCTTGGATTTCCCGCAGTATGTC
CCAACTTTGAAGTCAATAGAGTCTTCTATGACCTTGGCTTGCTGTTGCACCAACGCCACAGTGGGAGCAAGAA
ACACGCAAATGCTCTTCTGGGGTTTCCTTATCAGCTGTCCCATCTCATAGATAAGCAAGACGGCTATATGAGT
TTTCCCACAGCCCGTTCCCAGATATACAACAACATTCTCCTCTAATGCTTTTTTACAAAGATCCATCTGATACT
TTCTAGCAATTTTTCTGGGGTCCTTCTCTGGTTTCTGAACTGAAACAGGGGAATCTATGTCGCCACTGAGAGAC
AGAGCAGAAAGCTGCTCAGTGATCGGTGAAGTTGCAGCACTTGGTGGACTCTCAGTGCCGTTCTCGAAGTCG
CCGGCTT
>TCONS_00007712
GTTGTTGTTGATCTGCTGATGCTGTTGCTGCTAGCGTTCTTTTGTTCTCGCTGCTATGTGGCTGCATTAACACCC
AAACAGCGAGGATCATACTAAAGATAAAGGGCAATCTGGAGGAGATTGCAGCCATGGCGGTTTTGAGAAAC
TCAATGCCTGATCCTAGTGAGATATGAGCCTCATATTAAAATAGCAAAAATCTCATTAGTTTAAGTCTGAGGC
TGTTGTGAAAGCAGAAGGCTGTGGGGAGTTTCAATTAGTGGTTTTGCCCCTTTCTGTCATTCTCTAATGCCGAT
GATCACTCAAATGAAAATATTTTCCGATTCCAAAACCAGCCTTTTGGTGAAAACACATATAACCTCTCTATTTT
CCTTTCTTGTCACTCCTGTTTCATTTTATGTGATGCAACGAGGCTATGAAGATTTTGTTCTTCTGTGAAAGATGA
ATCAAGACATATTTCTATTGAGCTGGGAAAAAACTGTAACTTGGCATGGCAGCTCAGACGGGTCTGTGAGAT
ATTGGAAAATGTTCCATTTCTTTGGTGCTTCTTCCGTGTCTCTTAAGGCTAACTGATGCTTGCAGCCATGGTGGT
TTTACCAAAGCATTTTTGGTTTTTGTCTGAGGAAAGGCGAATCTGAGCCTACCCCGTTACTTTGAATTTGGTTTT
TGGTTTTCGCTCTTCTCCTTGGTTTTACCCCGTTAATCTGAGCTTACCCCTAAAGCTTTCACTAATGACTCACTC
CTAATTCCTAAAGAAAGAAAGCGAAATATGTACAAGAGTTTTTGGTTTTTGCTCTTCTCCTTGGTTTTGCCTCA
TTAATCTGAGGTTACCCGAAAGCTTTCACTAATGACTCACTTCTAATTCCTAAAGAAAGAAAGAGAATATATA
CAAGAAATAATTTATATTTACAGCTTTCAGGATAGTAGAAAAAGAACTGTTATGGAGATGTAACAAAACAAA
AGCTCAGGAGAACGAGAATGAGGCTTCAGAGAGTTTAGGATTTTCTGTTGAAAGGGTGGTTGCCTCTCTCCGT
ATTAGCCCATGATTTCTCCCTTACTGTAACAATCCCAACAACCCTGTAAAAATTTCATGGAATGGCCAGTTTTC
GCCTTGCTATTTAAGGTATATCCATTTTTTTCAAAGTATTTAACTTGTAGCCAGTGTTGGTGAGTATGATTGACT
GCTTGAAAGAGGCGCTGATGATTTTT
>TCONS_00007828

AAACAGACAGAAATCGATCTGTTTATGGCAATTCTACAGAGAATTCGAAACCATAATTTCCAACAAGATCAG
AGTACATCGATCTCCAGTCTCGAGACAACTCATGTGCTAGAGCACAATTTTCTGACATCGAAGAGCAATTGTA
GTCAATCAGCCAACGCATAAGTCTCAGCAACACCAGTCTACTTCATTGTGATGCGGAGTTAACGAGCTCTTGT
TTTAACAAATTTGGTTCTTGAAGCTTTCTTCTCCAGTTTCTCTCTCTTTTTTCTGGTCTTTTCATCCTCTTCAGCAT
CCTCTGAACCTTGAGGTAAAAATCCTTTGATAAATCCAGAGAGTTTGTACGCTGCAAACGCAGGTATAACTA
GATATGTGTACCAGAACTTGTCTGAGATGATAGAAGCCAACTGCACGAAACATGTAATGTAGATTACATCAT
GTAAATATCCACAAATACCACCAGTACTCATATCATATCCACCATCAAAGAGCTCGCCATCATCACCATAAC
TTGGTTTTGACATTGAATCTAATTGTTTGTATGGTAAAGCATAAGCCAAAGAAGTCAACAATAATCCTATTAT
ATGCTTCCATGTGAAACTTGAATAGAAAATTCCAGCTCTCACCAATAAATAAATTACGTTGCAGGCAAGGAT
GATTTGAAATAGTTTCTTCATATGACGAGCGTTTTCTTCCTTTCGCTTCTTTGCTCCTTGATTCGCCATCTTTGGA
TTCTTCTAAACGATGTCGTATTCTGATCTAGGAGCAAACTTAAAGCCCTAATTTTGGAATTGAATTGTATGAAA
TTGAATTAG
>TCONS_00007833
GCATTGGCAATATGATAGTTGTAATCAATTCCTGAACAAATTTGATGGAACCTTTAGATGGTGTGGCATGATT
TTTATATCAAAGGGCACGTGAAGCCAAGCCTCAGGCGGATAGTACAAGAAGTTTTCAGGCTTACTAGTATAG
ATATCTGCATATTGATGAATGTGGTAAGCAAAAGCAGACTCCTGACCAGTATCAGTGAGGAATGTAGCTCCA
AATGATTTATTGAACATTCCTTTCATTTTGTAGCGAGTCTCTTGTCTCTCTTCATTGAGCTCCCTAAGGAGAGAC
TTGTATGCTTCACTTGTATCTCTATTAGTTACTGTAGCATGAAGCCTACCAAGAAGTTCTTGTATTATGTGAAA
CTTCGCCTGTTCAAATCTGTAAGTATTGTCATTCTGAATTATGATCTCATTCTTCAATTCATGGATGATGGCAGC
AGTCCGCCACCCTGCTTTTGAAGGCCCTCTGAGATCACTAAAGAGGTGATCCCCAAAATATATCACCTCTGGA
CCTTTCCACTTTGTAATCTGCAAGAACGTTTTAAGGCATCCATGGTAGTAAATTTTACCGGGAAAGAAAGCAT
CAACTTTCGTAAAAGCCAACGTGTCCTTCTCCACATCATAGCAGC
>TCONS_00007834
CCTGCAACATAAAGCGCATCCCTCCATCCACAAAATAAAAGGGAGAATTGGTCAACAAGAAAAGTTTCTTTC
CTTTATCCTTCAGCATCCTTAGAAAGCGTAGTAGCTGATCATTCTTTACAAGGTATCTAGGGGGATCAGCAAG
TATTCCTCTGTGAACTAAGCCGCTATTATGAACATACTGTATCGCTCGATTCACATCTTCATATACATAACGAG
CATCAAATTCCAATTTAGCATCAACAAAATGTTGCACAATGTCAGCAATAAGGCATGCCTCACTAAAACAGA
AGAAATCCATCAAACAAACTAGTTCCCGCGCTTGATCACGGCCAATATGTCTTGTACCATACATCTCATCTAT
TTCTTCCTGGCTGAG
>TCONS_00007835
CTTTGATTTGTCATAGTATAACCCTCTGATGGGAAAACTAGGATCATACTTGAATTCCAAGCAAGTATCAGGA
TATTTGAACTCACTAACTAGATGCTGTTTAGCAAGATCATAAATCAAGCTCTGTAAATTGGAGGAGTAATGCG
CCAACGTGTAATCATAGTCGAATCCATAGACTTCTATGCTGTCCAACCGCACGTTCTTATTAACATATACACC
TTTAGGATTCATTTTGGGCATTTGTTTCA
>TCONS_00007919
CCCCGACCCTGTTCCAGTGGGATCTCCAGTCTGCGCAGTGAAATCCTTCTGAACCGTGTGAAACAGGCAGCC
ATTGTAATACTTAATCTTGCACAATTTCAAAAAATTCTTGCATGTCAAAGGGCAGCGATCTGTAAACAAGTCC
AGTACCATCTCCCCCAAACTCGTCACTATCATTACCGACATTATTTCTGTAAGGACGGAGGGAAGAAGGAGA
GGAAAGGGTTGGGAAG
>TCONS_00007987
TTTTGATCGAATTCCGTCTAGTAAGCCTCGAGATCTTACAGATGCTGTTGAGAATCGTCATTAACCAATCATGT
ATCGGACGCACCATCCCTTGAAGCGGATTTGACCCGCACCCTCTTTCTTTTTCTTTAAATAAAGAAAGTGGTG

GCTAGATTCGCGTTCCCTTTCCAGACTATCAAGGATTTCAAAGATCTTTCATACTGTGCCCAATCCCGAAATTG
GTTCTATACATATGACCAGCAACGAGAAAAATAAATGCAATAGCTAAATGGCGATGGGCAATATCAGTCAG
CCACAGACCCCCAGTTACAGGATCTAATCCTCCACGAAAAGTAAGAAATTCCGCATATTTTGACCAATTCAA
GGTGAAAAATGGGGTTGCTCCCTCGGCAAACTGGGATAAAGTTGAGCCAAAAGATCTCGATTCAAGATAAAT
TCATGAGGAAGTGGTAGCTCTTTAGGATCTACTCCAGCGTTTAGAAATTGGTTAATCGGTAAAGATAGATGTA
CTTGATGCCCCGCCCAAGAGAGAGACCCAAGTCCTAGTAGCCCTGCCAAATGGTGATTCTGGTAGTGAAAGA
AAGCCCATACCTAACTCGCTATGCTTGGCGCGGTCCCGTGCTACGCTATTTGATCGAGATTCGAAATACCCTT
CCTCGCCGCCACCTTCCTTCAGAGGTAGCCCGATTGGAATCTTGTACACTTTCCTTATCTTTCTTTTATTGATCC
AGCCTATACCTATTTCCTCTTGCTTCGCGCGGCCGGGCCTCTACCTCATAGAAAATCCTGTTTAGGAAGCTAGC
GCCCGCTCTCGCTCCAATATACGTGTAGGGGGAAGCACCAATGAAAGTGAACCTTTATTGTTTCCTTTTCTCTT
CGAACCGATTAGAAGGA
>TCONS_00008132
TTACCCTTTTTATTCATATCTGTGATTGAGTTCGTTATGGGATGTGTTCTTGGAGTTAAATTGGACTACTTTCTA
CTGATATCCGCCATAGGTGTTAGAAAATCTTATTTATAAGGTCCAATGTCACGTTAGAGAAGTGTATCTTTGC
AAGGGATACAGAAATCCTAAGTCTAAACTGCCAAGTTTGATAGAGCCAGAAATCTCTCAAATGCCCAGAAG
CCTTGAGCGCGAG
>TCONS_00008148
GAATTCGCAAAATCCTCTTAGATCTATCATTGGAGTTATACGCTCACAGGAAACAAGCGGTAGATATTAGAT
GGATCCATAACAAAGCTGATTTACTTGAAACAAATTAAATTCACCGCAGTCTCCGATTCAGTTGATGCAAGG
CGGGACCAAAACTTCGTCATAAACAACATGTATATCTATCGATACGCCACCGTCACCGTCAGCAGACGTCAT
TGTCGCCGGCGAGGCAGGTCCCG
>TCONS_00008228
GTCGCATACTGTGTCCACTTTTTCTGTTTTTCCCTTCGCATTTGACCTTTCCTTTTTCTCTTCTCTTTCCTTTTGTTT
CTTTCTTTTTCACATCACAATTCCTTCTATCCGAAACCTCAATTTCCTTCCTTCGAAAATTAGCGTTGTTTGGTCT
GCTGCCGTTTATGATCGTAAATCAAAAGCTTTCAATGGTTTCTTTATGTACCCATCAAATTTGTTATTTTTTACA
TCAAAGAGTTGATCTTTACAAGATTCCAG
>TCONS_00008248
CATAATACCTAAACAGAAGAGAAGAAACCTGGAATTCTTCTCGTTTTTTAAAGAGAAATTGTGGTGAGGAGC
TGGGGACATGATAGAAAGAAACCGCCAAAGAGGTGCAAACAATTTTGAGCTCATTCTTTAAACTGCCACTCT
CGACGACACATGGGGCAATGAGCCTGGGAAGTTTGTGAGTTTACCCACTTTAGAATGCAATGAAGATGAAAT
GCATGGTTGCAAGCACCCCAGATTAGCGGGCAATCATCCCCAGGGAGTTTACAATCAGGACAGCAACCATCA
AAAGCCATCCTACATATCCCGCAAGTTTCATCCTGAGCATCCCATGTCCATGAAGCAACAGCGTGCCATTCTA
TTCATGATTCCAGCGAAGCGCC
>TCONS_00008255
ATCAGGAATATTATTCTCAGGATGGCTATTAAACTTCATCAACCACTCCGAAGTTGTCAAATCGGAATAATTC
GTCGCAACCTGACCATAAGTATCTCCAGATTTAACCTTGTACGGAAAAACATGACCTAAAAACTCACCGTCC
AAACAATCACAACTAAAGGGTATGTTAATTCTTGTCCCAGCTATGACACTGTCTTGATTAGGTATGTTGTCATT
GTTGTTGTAGTCGATGATTTCTTGTCTAGTAGAAATGGAGAATAACTGTGATATGTGAATAAGATTTGTCCCCC
GCCAAACAAAGAATGAACCTAAAGCTAAATCGCACCCTTTATT
>TCONS_00008264
AAAAAAAATATGGCGAGCAAGAATGAAGTTGTGCCCGTTGAGCTTCCTGCTCCAGCTGGCTGGAAGAAGAC
GGTGGAGTGACAACCTTGGAAACCAAGGATCGAATCATAAGTGTTGGTGAAGTTACCCGGTACTTCTGTTGG

TAGGAGGTAGCAAGTGCTTGATGGATTAGTTGAAGATCGCATTGACTGGTGTATATACTACCATCGAATGACC
CAAAGAAATGAAAAGACTGAAAAAAGCAGCAGATGGACTCTTGATAGGTGAAAAGT
>TCONS_00008339
ATGGGGAGAAAAAACAAGAGATCCAGAGCTAAAGAATTATAAATACTAAATTTGAAGTTGACATGAAATAA
CACAGGAACCTTTCACATAAATCCTTTGTTGTCCTTGAGCATCAGGTAAACTGATATCTTTATTGATAAGAGAT
TCAATCTACAGCTGATCAGCAGATGTGAACCAGTTCTGCAAGCCAAACAATCAACTACATGGAGCCCAAAAC
ACAAAGTAACATAATTCAGCAGCCATACTATCTCATCCAATGCGGAAGAACAAAAAGATAATTCACAGTTAC
GGAGCATAACATGCTCGTCTTCTACTCTGCTCTGGACTGGCCAGCACGAGAAGAGAGAATGATGCCAACAAT
AAGGCCATAAAGAGCCAGGGCTTCAGCGAAAATAAGAATCAGAATCATCCCAACAAAGAGCTTTGGCTGTT
GCGCATTAGCTCTAACACCAGCATCACCAACGATTCCGATGGCCATACCAGCAGAAAGTCCAGCAAGGCCA
CAAGCAAGACCAGAGGAAAGGTGAGCATAGCCATCAAAAAGATAATAAGACTTGGTTTTTGGGTTAATGCC
AGTACTGATTATCACAGCTATAATCAATCCGTAAATACCCAAAACTCCAGCCATAACCACGGGCACAATAGA
CTTCATCACTAGCTCTGGCCTCATCACACCCATAGATGCCACTCCTACCCCACTCTTAGCAGTACCATAAGCT
GCCCCCATACAGGAGAAGACTAAGGCAGCGGCAGCGCCGAGGAAGCCGAAGAAGGGAGCCGTTTCATCGC
CGCTGAAAGTCGACGCCATTGTTTTTGCGAAGTT
>TCONS_00008342
CAAACAACTAAGTTACCATTATCCAATTACATGTCAACAAATTTCAGAATAGGGGAAATATGGTAATGAAAG
GACCAGAAAGAAATAGGGCAACCGAAGAGGGGAGACGTTTATAGGTGCATACAAAATATTTGCTCCTCCTTC
TGAACATTCATTCCTCGAGAGAAGCTGAGAGGAAAGAGGAGCAAATAGAATAAGGAACCTAGAGTTCGAGC
ATAAGTTCTTCTTTCGAATCGGAGAAATAGTGGAGAATGTATGGGGCGACCCGAATCACGGCGACCCAAGTA
CCAAACAAGGCCACGTGTGTCTTGAGCTGCTTCAAAGCAGCTGCTTTGTCAGGTTTACGCATGAACAATCCTG
GAAACATTGTTGCTTCTTTTGCTGCTCTGAGATTTGAGATTGAACGAGCCAAAAACCCTAGGTTCCGACTGAA
AAGTGGGTAACGGTGAGGTTT
>TCONS_00008470
AAATCAAGAAAGAAAATAACCCCTGCATTTTACTTTTAGTTAGGTAGGATTGGCTTAACAATCTTCTTCAAAC
TATACTAAACAGTTAGTTCCCACTTAATTACCTCCACAAATTTCCAAATTTAACTTGGGGTGCTGGAGGTCTAG
TACAACTTGGTTAATTTTTACTCGGCTTACCTTCCATTATTAAACACCTACTTCTAGTGAATTCTG
>TCONS_00008502
TCATAGCATAGACAAACAAAAAGAAAATAAAGCTACAATGTCAGTTTTCCTTCAACGAACAAAGCTGTTGCC
CAACAAAATTCCCCAACTCAGCAATGAGATGTCTGAATTTCCTAGGAGATATGTGAATTTCTCTAGGCCTCTC
TGCGAACAAAGTAAAGCTTACCCATGACATGAAGGATGGCAACAAAAGCTATGAAGCCAATACTCATCACA
AGCACAACGTTGGGGGATATCTTGAGTCCGGGGGAATCATCAGTGTAGAATTGGAGCATGTTACCACCTGCT
CCTCCTGCAGCTCCACCACCACCAGCGGCCTTCCTTCGACGCAGGTTAGCAGCAGCTGCTGCAGCAGTCCCTC
TTTGTGGTCCACCTCCACCCAAAGCCATTTCTTTAGAATGCTGAGATTAGGAGAAAAGTCTTCCCGATCTGAA
AGGAGGAAAATGAGAGAAAAATTTTGACCTGTTTTCTTTTAATTGG
>TCONS_00008716
ACAATTTGATTATTTGCAACTCATATACAAAGTAATTCCATCAAAGTGAACTCTAGCCAGAGACATATTCTCA
AACCACCTCACTGGGGGAATTCACCAAATGCAATTATCAACATGGATTCGGAGTGAACCAAATTGGAGGAG
GCCAACGAAGCATAGAATGTGAATTGCGAGGATAGCATGACAATGCTTCAATCCTACCATCCTCTAAATTGT
AATACCCTAGATCATGATTCCCATTGACACCATCATAATTAGCCTCACAATAATCATCCGTAAAGTAAATCCT
ATTTCCTTCACATCCAGGATAATCAGAAGCCAACAGCGCCAATGAAGAATTCTCTCCTAAAAACAACATCTT
CTCACCCAAACTATCCACCTTCTCCCACCTCGGCCCTTCTAAAACCAACCTAAACACACGAAATTCAACCGTC

TTATACACAACATCAAGCTGATGATACGCAGCATCAGTATCAAGCTCCAAAGACCTAGTCACCAGTAAAAGT
TCATCATTCGTTTTAACCAAATACTGTATATCACCGCCAATTTGCCTAGGCGTTTCAATAAATGAAACCCTAG
GCGAATCACCACTAACATCACAAACTGCAATTGATCCAAGTTTATGAACAGCATAAAATAATCCATCAAAAT
ATATAACATCCTCAGCAAAAAACCGCGCTTCATCAATAAATTTCCA
>TCONS_00008723
AAGAAAACACCAAGCATCAACTCCATTATTTAAACTCGTCCAGTCATCATTCACGAGCCAACATATCAGTAG
GGAACGATCTTCCAATGTTGGTTCCGGTTGTCTCCTTTCCACCATTCCCAGAGAGCAACAATGGTCCCGTCAC
GGACACCTCCATGTTCTTTATCAGCATTCCATGCATCTAGATTGAGGCGAATGTTATTAACCATCCTAACTGCT
CGATAACCATCACTCACGTCTCTGCTCTCTGTCCACAAGACTGATGCATCAAGAACATCAGGGTCGTAAGGG
GTGAGCTGCACAGGGTGAGAGGCGCCAACAGAGTGCTTCATGGCCAGTCCTGCAGCTTTATTAACCAGAGCA
AAGCTTGGAAACCCCTCTTCATCCTTCACTTTAGTGCTGTACTTCTCATCTTTATACCAGTGTTGAAAAGGATC
GGATGGATCAGAAGAGGCAAGAACGACTTTGCCGTCACGGATAGTGAGAGAGTAATTGGGATCAGCCTTTGT
ATAAACCCTAAATGAAGGCTTATCGATGAGCTCTGGATGTGGTGAGGTCTCATGGTGGAAGAATGAAGGCAC
ATGTGGTTGAAAACGATGATGATCATGATGACGATGACCGCTCTCACTCTCACTCTCGTGAGACACGTGTTCA
ACGGAGGAATAACTAGGTGGTTGCGGTGGTGGTGGGCCCTCATAATAAGGCGGTGTACGATTATAAGATCCA
CCATAGTTCTCCTCCATTCGCGGAGGCGGATAATTAGTCCGACCATAACTATCCTCCATTGACGGTGGAGGAT
AATTAGTCCGTCCATAATCGTCCTCCATAGGTGGCGGTGGGGGATAATGAGGACGGCTATAATTGGGCTCCAT
CGGTGGTGGTCCACCTACATGGGAAGTGTGATAGACAT
>TCONS_00008864
TTCAGGCAATGGTGTAGACCCAGTGGGTCGCTTTTCGTGATTTTTCATGAGCAAGTCATTGTTTCGCTCAGCCA
CAAGGAGAAGAGAAATCAGCTCAGAGTACTTTTTGAAACCTTTCTCTCTGTACTGCTGTTGCAGAACCATATT
GGAGGCATAAAACGTTGTAAACATTTTTTCAAGCATATCATAATCACTGATAGTATCTCCACAGAGTTTCAAT
TTAGAAGTAATTCTGAACATCGCAGAATTATATTCA
>TCONS_00008872
TGGCTCAATTTTATTCCCACTTCATTGCACGGGTGATTTCCCATTCAACTGCGTCATGATCTTGATCCATAAGTC
CGTCAACCTTTTTCCTGCAGTTCACCAATGCAACTTTGAATGTTTCGTGCTCAAGGGATGATGGGTACATTATC
TGCTCTTCCTCTGGTAGTTCGCAGAATTCATCAGGAGTTATTTGCAGCAACTCCACAGCAGTTTGACCAGTAC
ACCAG
>TCONS_00008900
GCAGCCTATTGTATCACCATCGTTTTCAGTATCAGTTGAGCATAGCCTAGAAATATAGAAGAATGAGGCATC
AGAGGGACTGGGATTTTCTGTAAAGGGTGGTTGCCCCTCTCTGCTATGCCCATGATTTCTCCATTTATAACCAA
AAAAACAACAGCCCTTTCAAATAAACAAGAAAATAGAAAAAAAGAAAATAGTTCATCCAAACACACCCTA
GTATATTGCTCTTCCTCTT
>TCONS_00008910
CTTTTTTTTTTAAAACAAAAATCAACAACCCTTGAACTAATGGAAATATACTACTATACAATCATTTCTTGAG
GATCAATCAACAACAACAGCCAGATCTCTCTTTCTCTGTCTTTGTTTCTGTGATTTAGTTCCTCTTTTTTGATAC
GTAACAATTGAGGCAGTGATGATATTTTATCCGGATTCTGGGCTAACTGGATCGGGCATAGAGACTATCCGAC
CCGTATCTTGGCCTGGCAAATCACCGGACGGCCGGCGATTTGAAGGTTTTTTCTTTGCATACAACCGGTGATTT
TGCAGTCATGGCGTGGTCGCCTCTCTTTTCCGCGATCTGTCTTTGCAGATCTGAGCCTAAAACTACTACTTCTTT
GAATTTCAACTACTTTTTGTTCTTTTAAGTTTTAGCTTAAGCATCAAACCAAAAGAATGAGGATTTTGACGAAG
ATTGAGCTCTTATGTGGTATGGTTATGATACTTCTGAAATCAAAAGCATAATCTACCGTACGACTCTATGTGA
>TCONS_00008942

TTGATGCAAGTAGTGCACGAGCTATGAGAGGCCATAATCTACCACAGTCTTCTGGCTCAACTCATGCCCCGA
GTCTTGAAAAGGAAAATACCGGTGATGCAATTGGAAATGGTGGCCACAAATCAATTTGATTCCTTAAAGTGA
ATGGTGAAGACCTTTTGAACAATTATGATGTTTTTTTATTGATACTATAAATATGAATACATTTGGTTTTGTCG
AGGTCTTTGCTAGT
>TCONS_00008951
CGGAATTCAAGAAGAACATATTTGCTTGTTGAAGATGTTGTTGTTGTTGCTGCTGTTGCTGTTGAGTGTCAGTA
TAGCCTATATAGTTAGGGTTCATCAGATAAAGTGTCTGCAGACCATCAGCTTGGATTTCTGAGCTCCCTTGAT
AGTACATCGCCATAGATTTCAGCACCTATATATAAAAATCTTACCTCACTCTTAAATGCTTAACCAATCAAGA
AATTAAAATCGTTTTTTTTTCTCTCCAATATATGAAACTATTTCAAGAAAGCTTAGGCCATCCAAATTTGGAA
CTCACCTGCAGATAGTACTGATGATGGAACCTTCTTGTCAGATTCTTAAGTTTTCTGCAAACTCCACTGCACAA
AAGGGGAATATGAATAAAGAGATTTTTATTAGTGCTAGTCTCACAACATATCCTCC
>TCONS_00008952
CGGAATTCAAGAAGAACATATTTGCTTGTTGAAGATGTTGTTGTTGTTGCTGCTGTTGCTGTTGAGTGTCAGTA
TAGCCTATATAGTTAGGGTTCATCAGATAAAGTGTCTGCAGACCATCAGCTTGGATTTCTGAGCTCCCTTGAT
AGTACATCGCCATAGATTTCAGCACCTGCAGATAGTACTGATGATGGAACTCTGACAAATATATGAAGAATG
TATGAAGGATCTTCAACAATAAACTAAACAGAAAATAAATTTAATAAAAAGATGAAGAAAGAAAAGTAATA
TAGAGGGAGAGAGAGAGAATGATTTTGAGAAGTTGCCTTCTTGTCAGATTCTTAAGTTTTCTGCAAACTCCACTG
CACAAAAGGGGAATATGAATAAAGAGATTTTTATTAGTGCTAGTCTCACAACATATCCTCC
>TCONS_00008971
TAACGAAATTGTTCATACATTGGTTCGATTTTTCTTTTTGTTTTAGGTGGAGTTTTGAAATTAATCGAAAGTTGA
AGAAGGCATTGCCTATTGAAGATAGTGCGTTTTACAAGTCACAAAAATGTCAGATATCAGAAAATGGTTCAT
GAAGCAGCATGACAAAGGTACGGGTAATGGGAGTATGTCCAAGAATAGTGCTCCAGAAAAGCCTTCAGCAG
AAAAGCCTTCTCCAGGAAACCTGGTAAGTATTTTGTAGCTAGATCATTCTTCCAATTTCACTTGCTTTCTCGTT
GACCTCTCGTATGGTTTAAGTGTTTGTTAATGTAGTTACCTATTTCTTGTTTCTTGGAACCTGTTCCGATTGCCTG
GAATGGTGGGGCTGGCTTGTTGTTTCTT
>TCONS_00009131
CTGAGGTAGGTGTTTATTTCCTGATAACTGAGATTCATGCTTCTTGTCCTTGTCATAATCTGCCTTGTTTGAACA
AGTCTGATGCTCATCTCTTGTTGACTTCTCAAGATTTTGTGAAGTAGCATTAGTTTCCACATCCTTCTGTTTATC
GCTGTTTTTTCCTTGATAATTTAGTCTTTGCTGAGGTTGGTTTCCCTCATCAGGACGAAGTGTGGGATTTTGTTC
ACCTTGTTCATTACGCTGCAGTCTAAGATCATCATCCCTCTGTTTGGAATTGATATCTGCCTCTGCTTGACCAAT
CTCCACAATCTCTTCCATTCCAGCAATACTGGAAGAGACGGGTGACGTGACCAGAGACATGTTAACTCCAGC
TTCCAGAAGTTTCCTTAACTCCCTCTCCTTCTGCAGTTGCTCCTGAAGCTGTGCCACATCCTTCTCAAGTGCCA
AACGACGCTCACGCAATGCATTCTTCCGATTCTCCATACTTTCTTGCAGAAGGGCATTCCCCTTGGCCTGCTCT
GCAATTTTGGATTGCAAGTCAGTTTTAGTGACCTCAAGCCTTTGGATTTCAGTCACGTCATCGAGAGGGACAT
TGATAGATTCCATCGAGAGGTTCTTCTTGGCAGGAGAGCGCCCCCACAGAGCAGGGCGCCGAATGCTTTGGG
CAGGACTACTTTGCATATCTATTGACTTTTGGGAAGTAGATGTTTTTTCCAGTTCAGCTCCCAATTGTTCACCA
CATTCATTTCCCTGCGTCCTTGAATTTTCATTACTTTGAACTGGAACACTCTCGCTTCCTTCCACTGGATCATGC
TGAATAGAAGTACGAGGTGGACTTGTAGGCAAGCACCTACTGGCTTCGTTGTCTGCTTCGGGAGACTTGGAA
CCAGATTTAGAAACTTGAGAGCTCTGCAACATTAATCTATATCCTTAGGGAGAAGTGACAGAAGTTGACGTG
GGGAGGAGCAAATATGTACTAAAAATAGTTCAAGAGGACTGCATGAAAGCAGAGAGAACACTAACATTATC
AGCAAGCTTTTTTAGAGATTTTCCATTTCCTCAAAATAGTCTTCGACTATATTCAAGTAATC
>TCONS_00009132

CTGAGGTAGGTGTTTATTTCCTGATAACTGAGATTCATGCTTCTTGTCCTTGTCATAATCTGCCTTGTTTGAACA
AGTCTGATGCTGAAGATGTTTTATTTACAACTCATCTCTTGTTGACTTCTCAAGATTTTGTGAAGTAGCATTAGT
TTCCACATCCTTCTGTTTATCGCTGTTTTTTCCTTGATAATTTAGTCTTTGCTGAGGTTGGTTTCCCTCATCAGGA
CGAAGTGTGGGATTTTGTTCACCTTGTTCATTACGCTGCAGTCTAAGATCATCATCCCTCTGTTTGGAATTGAT
ATCTGCCTCTGCTTGACCAATCTCCACAATCTCTTCCATTCCAGCAATACTGGAAGAGACGGGTGACGTGACC
AGAGACATGTTAACTCCAGCTTCCAGAAGTTTCCTTAACTCCCTCTCCTTCTGCAGTTGCTCCTGAAGCTGTGC
CACATCCTTCTCAAGTGCCAAACGACGCTCACGCAATGCATTCTTCCGATTCTCCATACTTTCTTGCAGAAGG
GCATTCCCCTTGGCCTGCTCTGCAATTTTGGATTGCAAGTCAGTTTTAGTGACCTCAAGCCTTTGGATTTCAGT
CACGTCATCGAGAGGGACATTGATAGATTCCATCGAGAGGTTCTTCTTGGCAGGAGAGCGCCCCCACAGAGC
AGGGCGCCGAATGCTTTGGGCAGGACTACTTTGCATATCTATTGACTTTTGGGAAGTAGATGTTTTTTCCAGTT
CAGCTCCCAATTGTTCACCACATTCATTTCCCTGCGTCCTTGAATTTTCATTACTTTGAACTGGAACACTCTCGC
TTCCTTCCACTGGATCATGCTGAATAGAAGTACGAGGTGGACTTGTAGGCAAGCACCTACTGGCTTCGTTGTC
TGCTTCGGGAGACTTGGAACCAGATTTAGAAACTTGAGAGCTCTGCAACATTAATCTATATCCTTAGGGAGA
AGTGACAGAAGTTGACGTGGGGAGGAGCAAATATGTACTAAAAATAGTTCAAGAGGACTGCATGAAAGCAG
AGAGAACACTAACATTATCAGCAAGCTTTTTTAGAGATTTTCCATTTCCTCAAAATAGTCTTCGACTATATTCA
AGTAATC
>TCONS_00009136
ATAAGTTTGACCACCAGAGTATTATGAATTTTACCTGCCATGTCCAACAGCATCAAAGGGTGAGAGAATATC
CACAGAGGCATTAAGAACAGCAGTAGCTTGAGCTCCAGCTGAGAAAAACCTAGCAGATACTATCTTACCATT
GCAGGAAGTTTCAGGAAATAAAGGACCTTCCTCACATGCTCCAGAAAAATGTTGAGGAGTATAATTAGTAGT
AGTTGTGGGATCATAAGCAAAGCTGGGGTGGTCTGGGTTAATGCCAGAATCAACAAAGCCGATCACAATTCC
TTCCCCGATGTTTCTATCTCCTCCTCCTTGCGTCCATACTGCCGGTATCCCCAGAAACTGCGGAGTGTAAGTAG
TCATCAGTTTAACCCTCCTATCTTCCTCCACCAACTTCACTCTTGGAGCATTCTTCAGGTTTCCAATCTTCCAGC
TCAATTATATGATCATTTGGTGTAGAAACATCAAAGTTGCTCTAGAATATATCACTCAAGTAACAGAACATCA
TTAAGGAGCCGCGTGGACAAATGGAAATTGATGTGGAACACCTTGAATTTCAGGATAATACAACAAACAAG
TAACTCAATTTCATAGCAAGAAGACAACACTCCACTTCATTAATACTAAGCATATACCAACAGTTGTCTAG
>TCONS_00009267
CTAAAAGATGGGATGGGTGATCCATAAGATCATTTGGCGAGGTTCATCCCTCAGCGTGGGGATCTTTCTGGAA
ATAAAAACTTGTTTTGTTGAGGCAATTTCCCTCGTCGCTTACCCGCATTTCTCTTGGTATACCCACCTGTGTGCC
TCCTAATTCCGTGAAGTCCTTGGAGCATGTGCGATTTATTCAGGATCCTTGTCAAGAAAGAAGTCAGTCCCTT
ATTCTTTATTTTATGTTAAAAGGTCTGGACTCGAAGAGGG
>TCONS_00009314
CAGAGTCAGAACTTCCTTCTCAATATGTAGCTGCTTATAACACAAAGCTTCTTGTATCCTTTCACTCGTTACAC
TTTTCATTGTCAACGAATGAATTAAAGTGAGATGATACCAGAAGACCAGAAAAGTTGAAAAGAGAGTGAGA
GAGAGCAATCGACAAGATGTAAGAAGAGTGAGGCTCAGAAATGCTCAGCTAAGTATAGGGGTATCCTTTAG
AACAGTGGTAATAACTCATTTGTGCTAAGCCTAAATGAGATACTCGAAATGCTTGTCCTTCTCTTGTAACTCA
AGGTTTAAAATAGTTCGTTTCGTTTTGGAAACCTCTCTTTTAGGTTAGACATAAAAACAAGAATCAAATTTGCT
ACAATTTGACGAATTATGATCAGTTTTTTGCTAGCAGATATTAAATTATCCTTAATATTTATTAGAATACTTTGT
CGGATGACAAATTCAAAGACATCATTACATCATTATGTGAGTGTGATTCAACTTTGAACCATCTATTGAGATT
TCTGTTTGCTGCTGTGTGCCATATGGATACAAAATCTGGTTTTTTATCACTTGATAACTTTAAGACAGCACTCTC
AAGTATTCAACGGCAACCCAAGTTGGAAGCCTTGAAAGGTATGTCATTATGTATTCTGTGCTGAGTGTTGACT
AGCTTC

>TCONS_00009478
CGGCTGTTAACCGATTGGTCGTAGGTTCGAATCCTACTTGGGGAGATTTTTAGTTATCGCTTTTCTGACCTAG
CGACCCCTGCCCTTCTCCTTTGTTTCTAAACTAGCAGAATCGTGGGACATCAAAAGCGGTAAGTTGATTGTTG
GTTTTTATTCCTCACTCTCGTATAGGTTAACTTGGTTCGTTCCATTCTTAGGGATAAGAAGGATCCACTGGAAA
GACTGGAGTAGTATCTTCATTAGCCGGAAGGAATTAGTCTCCACTAGCGTCATTCGAAGAACGAACAAGAAA
AGGGTTACTGTTTAGGATAGATGGATGTGGCCCAATGGCTAAAGCTCTGCCAGCTTCTTGTAGACTGAACTCT
CTTCTCTTTAGGCTCCGAGTTCTTTTTTGGGGGGATGGTCTAATTTACTAGTGGCAACGAAGTTCTTGGTAATTA
GCAAGGGGGAAGGAGGATCTCCACTCATTTCATTCATTCAGTGCCTGCGGTGAGGCGCGACCCACAACAAAC
GAAGGGGGAAAGCTTGCTTTGCTTGCTGTAGCCCTATTTTAGTAGATTAGGCTTGGTAAGCGTAAGCTATATTT
AAGTAGGCTCGCAGCTTCGCTCAGCAGAAGAGCCTGACTTTTTATTATATTAGTAAAGCGCTAGCGCCCAACC
TATACAAGGGCTTTGAAGGGATAGGGGAAGAAAAGAAAACAAAGAGGGTCATTTTCTTTTAGGAACATGGC
TCCTTCATTCACTTGCTCATGAACTCGCAGCTTCGCCAGAAGCGACGAGGGGGGGCTGGGGAAGGCCGACGA
CTACATGAGGGGGAAGCTTTCGTAGAAGCTTTACCCCTTGCTTTACAGAGATAGTTATGAACTGACTAAATGA
CTAGATTCTCCCGGAACGCTAGCTAAGCTAATCGTCTTAGTTCCCTTCAATTAAATCAATAAGCTTTTTGATTG
ATTCAGGGCGTCGCGGGAAATCTATAGATCGTAGTAGTTCGCCAACCTCTCTAACTAACATACGATACAATTT
CACTTCACGGCACGGCCAAAAAAAAGAAAGAGCTTCGCTCGGTTGGCCTTCCTACGCTGACGAATGCCTCTT
TTCTCTTCTCTGAAGTCTACAACAAACAAGTGGGAGAGGCAGGATTCGAACCTACGTAGAAAAACTTCAACA
GATTTACAGTCTGCCGCTTTTGACCACTCGGCCACTCTCCCCTTCCCGGGCCGAGGCCCCCCTCAATGGGTTCT
AACGAGAATAGAAGCGAGTCGCTTCCGGCCTGTCTTGTAGTCGTAGTCTTTTAGCTTATGTAGTCGTCGGCCTT
CCTACACGCATGCGTCCGCCAGAATGCCTCCTTGGTTCGGGACGAAGCCAAGCCGATACATACGATAGGCGA
AGGAAAGACCCCCATTTCATAGCGCCTGGGGAACGCAAGTTTGATCGAGGATTGGAGAGGAGAGGTGGAAG
AAAGGGCTCGGGATGGATTTAGGAGTCTTTGTGCGAGCCGTATGCGGTGAGAGTCGCACGTACGGTAACGAG
GGGGGTTCGCGTCTATACGTGTAGTGTGGTGCTTAGGCCTACCCACCCTATTTGTTCCATGATCTATGGGTCTA
CTGGAGCTACCCACTTCGATCAATTAGCCAAGATTTTGACCGGATACGAAATCACTGGTGCTCGATCTAGTGG
TATTTTTATGGGGATTCTATTTATCGCTGTAGGATCCCTATTCAAGATCACTGCAGTTCCTTTTCGGGCGGCTGT
AGGACGGCC
>TCONS_00009511
CTTTCTGCTAAACAGGATGGATTGAACTCATAATTCATTAGATGCTTTTTATGAATGTCAACTAAGTATCGTAA
GTAAATTGCTCCCGGTTGTTCAATCATTTGATAACCAGAGTCATTCTTTGATAAACGATCACTATGAGTCAGA
CTCAATAGAATTTGATCAATCCTATTTTCTGTCGTTAAGGTGGAGAACTGAACCAAGAATTCTCTTTCTTCATC
ATCAATCGAATCACTGTTCGCGACCCAGGATTCTATTTTATCATCAATCCAATCCCCGTTCACGTTTTTTCTTTT
TCTTATCAATGAATAGATCTCTTTACTTGTATGACTTAGATGTCTCGTATTTCTCGAAAAATGATTCGATTGATG
GAATTTGGTATGAGATCGATGATCTCGATGAGATTGATATTCCAATCTTTCTTCTTAGAACGTATTGATTTGAC
CCCATAAGCGGGACCAAGCATGTTGCCGCCAGAAGCAGAACCCCGTATTTCTTCTAGAGAATCCCCTAATTG
TTC
>TCONS_00009516
CGAGTATCGATGTAGAATTCAGTGTCAACTCCGTCAATGATCCTATCTCTTTCAAGAACAAACTTCCCTTCTTT
ATACTTCTGGCTTTCCTCATGTGCAGACTCATATTTCCAGCCAACAATTTGCCCACAACAGCAACAAAATATG
TCCGCTACCGTGTGCATCCCAGAAAGCATCACCCTCTCTTCATGAGGTCCAACAGTTACATTCACCGCTTTATT
GAATAGGTATGCTTTTCCTCTGCGGCAATGAAACCCCCTGGAAACAAGGTCGTCAGCAAGGGCAAGATGGGT
TTTGCAGAATTTGCACTTATATGTCCTACCTTCAAGATCTACCACAAATATCCTTCCCATTTCCAAAGATTTC
>TCONS_00009575

TTTTTTATATCATTCCTTAAGTCCCATAGGTTTGATCTTGTAGAATCTGACCCATTTTCTCATTGAACGAAAGGT
ACGAAATAAATCAGATTGATAAAAGTACCATGTGAAATCTTCGGTTTTTCCCCTTCCTCGATCCCTATCCCATA
GGTTAGGTACAGTGTTTGAATCAATAGAGAACCTTTTCTTCTGTATGAATCGATATTATTCCATTCCAAATCCT
TCCCGATACCTCCCAAGGAAAATCTCGAATTTGGATCCCAAATTGACGGGTTAGTGTGAGCTTATCCATGCGG
TTATGCACTCTTTGAATAGGAATCCGTTTTCTGAAAGATCCTGGCTTTCGTACTTTGGTGGGTCTCCGAGATCC
TTTCGATGACCTATGTTGAAGGGATATCTATCTAATCCGATCGATTGCGTAAAGCCCGCGGTAGCAACGGAAC
CGGGGAAAGTATACAGAAAAGACAGTTCTTTTCTATTATATTAGTATTTTCTATTATATTAGATATATTAGACT
ATTATATTAGATTAGTATTAGTTAGTGATCCCGACTTAGTGAGTCTGATGAATTGTTGGCACCAGTCCTACATT
TTG
>TCONS_00009576
TTATTCTCAAAGGATAAGATTATTCTCTTTACCAAACATATGCGGATCCAATCACGATCTTATATATAAGAAG
AACAAAAGATCTTTCTTGATCAATCCCTTTGCCCCTCATTCTTCAAGAATAAGGAAGATCCTTTTCAAGTTTGA
ATTTGTTCATTTGGAATCTGGGTTCTTCTACTTCATATTTATTTAATATGAATATTTTCCCTCTTTTTTTTATATC
ATTCCTTAAGTCCCATAGGTTTGATCCTGTAGAATTTGACCCATTTTCTCATTGAACGAAAGGTACGAAATAA
ATCAGATTGATAAAAGTACCATGTGAAATCTTCGGTTTTTCCCCTTCCTCGATCCCTATCCCATAGGTTAGGTA
CAGTGTTTGAATCAATAGAGAACCTTTTCTTCTGTATGAATCGATATTATTCCATTCCAAATCCTTCCCGATAC
CTCCCAAGGAAAATCTCGAATTTGGATCCCAAATTGACGGGTTAGTGTGAGCTTATCCATGCGGTTATGCACT
CTTTGAATAGGAATCCGTTTTCTGAAAGATCCTGGCTTTCGTACTTTGGTGGGTCTCCGAGATCCTTTCGATGA
CCTATGTTGAAGGGATATCTATCTAATCCGATCGATTGCGTAAAGCCCGCGGTAGCAACGGAACCGGGGAAA
GTATACAGAAAAGACAGTTCTTTTCTATTATATTAGTATTTTCTATTATATTAGATATATTAGACTATTATATTA
GATTAGTATTAGTTAGTGATCCCGACTTAGTGAGTCTGATGAATTGTTGGCACCAGTCCTACATTTTG
>TCONS_00009594
AAAAATAGATCCTAAATATTTGAAATCAATCATTTATCACTTGGTGGAGGAATATATAGTATTTCATTGCTAC
AAGTATGGATTATTGAAAATAATAAGACATGGATTTGGATATTTTCCTTTAACTATTCATGTCAACTAAACCG
GAGGGGGGGGGGGTTGAAGGGAATTTTGTGAAGAGAAAATGGATTATGGGGGCGAATATATAGTATTTCATT
GCTACAAGTATGGATTATTGAAAATAATAAGACATGGATTTGGATATTTCCCTTTAACTATTCATGTCAACTA
AACGGGGGGATTGAAGGGAATTTTGTGAAGAGAAAATGGATTATGGGAGTGTGTGACTTGAACTATTGATTG
GTCTGTGTAGATATATGCCTGCCACATGGGAATTCACAACCAAATGTGTCTTTGTTCCAATCGCCGTGTAAGC
CCTATACAGAGGATAGGCTGGTTCGCTTAAAGAGAATCTTTTCTATGATCAGGTCCGAATCATGTTGTACATG
AGCAGGCTCCGTAAGATCCAGTATAAGTGAACTAGATAAAACGGAATCAAGATTCCGTTTTATCTAGTTCACT
TATAAGATTAAATAGTATGTAAATGTATTCATTTCCTCTGCATTGACACGATCAATACTACTATCGGAGTGAA
ACAAGGGATCTAAAGAAGAAGAGAGGCTAGACTATATTAGTAACAAGCAAACCTTTTATGTGTATCTCCAAA
TATTTTGGAGATAAATACCAATTAGAAGGTCTGAGACGACCCAGAAAGCACTTGATCATATCATGATCTGATT
TGTAAGCCTACTTGGGTCTTGAGTATTTACTTGTAAGAACGGAATTCTTTGTTTTGTAATGGATAATTGCAACT
CCGTAAAAAAGAATTCAGTCAAATTTTTCTTACATTGAA
>TCONS_00009595
AAAAATAGATCCTAAATATTTGAAATCAATCATTTATCACTTGGTGGAGGAATATATAGTATTTCATTGCTAC
AAGTATGGATTATTGAAAATAATAAGACATGGATTTGGATATTTTCCTTTAACTATTCATGTCAACTAAACCG
GAGGGGGGGGGGGTTGAAGGGAATTTTGTGAAGAGAAAATGGATTATGGGGGAATATATAGTATTTCATTGC
TACAAGTATGGATTATTGAAAATAATAAGACATGGATTTGGATATTTCCCTTTAACTATTCATGTCAACTAAA
CGGGGGGATTGAAGGGAATTTTGTGAAGAGAAAATGGATTATGGGAGTGTGTGACTTGAACTATTGATTGGT
CTGTGTAGATATATGCCTGCCACATGGGAATTCACAACCAAATGTGTCTTTGTTCCAATCGCCGTGTAAGCCC

TATACAGAGGATAGGCTGGTTCGCTTAAAGAGAATCTTTTCTATGATCAGGTCCGAATCATGTTGTACATGAG
CAGGCTCCGTAAGATCCAGTATAAGTGAACTAGATAAAACGGAATCAAGATTCCGTTTTATCTAGTTCACTTA
TAAGATTAAATAGTATGTAAATGTATTCATTTCCTCTGCATTGACACGATCAATACTACTATCGGAGTGAAAC
AAGGGATCTAAAGAAGAAGAGAGGCTAGACTATATTAGTAACAAGCAAACCTTTTATGTGTATCTCCAAATA
TTTTGGAGATAAATACCAATTAGAAGGTCTGAGACGACCCAGAAAGCACTTGATCATATCATGATCTGATTTG
TAAGCCTACTTGGGTCTTGAGTATTTACTTGTAAGAACGGAATTCTTTGTTTTGTAATGGATAATTGCAACTCC
GTAAAAAAGAATTCAGTCAAATTTTTCTTACATTGAA
>TCONS_00009742
CTCTTTTCTACCCTTCCTAATGTGCCATTTGATTCTGATTACATATGAGTGATTATTCAGTTTGCAGCTCTATAG
TTTAGTGCTTAATAGCTATGAGATGCCTTATAAGAACTATCAATTGTTTTCTAAACTTTATTGTGTTTGTACTTT
CTATTTTCTTTTTAATAATTTACAAGGTTTTGATTTCTGGTTGTTTTTTGTTAAATAAGATGTTATATAGAAGTAA
ATCTCCCTCTGTTTCCACAAACTGGAAGATCAGACTTCACC
>TCONS_00009763
ATGAAAACAAAAAAATCAATCTTCATATGATACATGAACAGAAGTGGAATTAACAAACACCTAAGGACGGT
ATGTACCATTTTCACTTAGTTATCTACCGTAGGACTTAACCAATTGCGAGAAAGCCCTACTCATCATCAGAGA
GATCATCCGAATTTGATGAGATCATCCAGATATGAGAAGCAGAAGCACTGGCATTAATATGACTGAAATCAT
TGACTTGCGGAGAGAGCTCGCCAACAGGGATGGTACTGTTTTCTTCAGGAATATCATGAATGGTAGACTCTTG
GAGATGAAGTGCATACTCCAGACTCCACAGCACATCACCCATGGATGGCCTATCTTGACCAAACTCAGCTAA
GCATTTCTCTGCTGTTTCTCCAAACTTCCTGAGGGAATCTGGTCTTATTTTGCCCACAAGTTTGGGATCTATGAT
TTGTTCCAGTTGTCCTTTCTTCTGCCACTTCATTGCCCATTCAGCTAAGTTGACCATCTCCCTCGGAAGAGATGG
AGCTATGACAGTCCTAGCACAAAGAACTTCTAATAAAACAACACCGAAAGAATAAACATCAGATTTTTCTGT
CAGCTGTTGCCTTCTAAAATATTCAGGATCAAGGTAGCCAAAAGTTCCTTTCACGACTGTGCTAGTATGGGTT
TGATTAAGCTCAAGCCCTCTGGCTGCCCCGATGCATATCTCCAGCCTCTGCTCCCAGCCCATACTGGGTAGAT
CTGACCCATACAAATGACTCTTGAGGGTCCCATTCTCCATGTACTCAAAAACTAGAATCCTCTCGTTGTTTTCA
TCACAATATCCAATCAATGAAACCAGATGTGGATGGCGGAACCGAGAGAGCATCTCAATTTCTGTTCGGAAC
TCTGCAAGACCTTGTTGGGACTTAGAATTAAGCCTTTTCAGGGCCACCTTTGTGCCATCACACAAAACACCCC
TGTAAACATTTCCAAAGCCACCTTTTCCAATGACCAAACTCTCATCAAAGTCGTTAGTTGCTTCCTGCAAAGC
TGCAAAAGGAACTTGATAATTTTCAATAGGAATGCGATGACTCGAGGTTGAAGCAGCACTTATGGACGTTGT
CCCCTTTGAATGCTTGCTTCCCATGGTATGTGTGGACTGTAGAGTGCCACCATTAATAGATAAAGGAATCCAT
GTCTTTGACTGGGCAAGCTACTCCTGTTTTCTTCTT
>TCONS_00009807
CAGGACTATTTTACGGCATCTCTCCTTCTCTCGCGCATAGCTCTACAGAGGTAGAACGGAATTAGGAAGAAA
AGATGCTCTCAGTGTTCAGCGTGGGAACTCAGCTTCAACCAAGAGTAATAGGCATTCGCAGAAGCCAAGTAA
TGGCGGTTTTGAAGCAAATTTGCAAGAAAGAGTCCCAACCATGACTTTTTCAGTTTTGAGCTTCAAGTGTGCT
CAAAATCAGATTG
>TCONS_00009810
ACAAGAATTGAAGAATGAAGGATATGTCCAACGACACTATTCAACTTGTGGTTATTTGAAAAACTTCCATGTC
CACAATCATGACCAAGAACAAAGAGAGCCCAAAACATAGTACTCTGAGCAAACCAATACAAAGGCCAAAC
AGCCCAATTGTTGAAATAAGCAGCCGCAGCTGCCAATCCAAATACAATAGCAACATCCCTCACAACATAACT
CATAGATTTCCATGGATTCTTGATCCAACAATGCGTAGGAATAGC
>TCONS_00009870

GTGTGCATTCGATTTATCGATTCGATTTTGACCCTTATCGATAATTACTTATCGATTATCGATTTGTACATATGC
TTATCGTTATCGTTTCAATAAGGTTTCGATTTTTTTGATTTCGATTTATCGATTTTGGGGGCTTATCGATTACACC
AATAAGAAAATTTGCGGGATTCCACGGAAAGTATATCGCTATAAACGCGCCTAACTAATATGGACAAAAAG
AAGCTAAAATAAGACGAACACCATTTATAACATAAAAATTGTGTCAACAAGCACATGTAACAAAACTAAAG
TAAGGGATCAAATGTATGCCACCAACACTCCAATGGTATAAAAAAAAC
>TCONS_00009918
AAATTACAGCGCAGTTATTCTAGAAAATTATCTGATAGACTATTTTTCTGCTCGCTTGGTCTAAACAAGATAA
ACACTGGATGTATTAGCTTGTATCCTTCACTCTTACTAGAGGTTGCACTGACAACCTTAACAGTAGCATGCCA
TTCGTATCATGATAAATACTTTGCGAACAGTCCACCAAACAGCAGCTGGTATAGTGTTCCATAGGTTCTTCTTT
CTCCTAGCCAGCTCTTTTCTGCTCCAACACTTGAACAGCTCATCAGTTTGCTTTGGCATAAACCCGTTGTCTTCC
AGTCACATTGAAGGAAAAATTCCACAACTGAGTGGGACAGTGCAAAAATAAGTGACTGTCGGTTTCAGCATG
CTTTCACATGAAAAAACACATGCTGC
>TCONS_00010041
TATACTCTCCGTTACTAGAAGCCATGCATGTTAGCGCTTTACCTTGGGTTTGAATGGATACAGACAAACAATA
TTCTTGCACCTATAATCACACATGGTATATACTCTGCTGTTATTCTGGGACATGGACTTTGGAAAATCCACAGA
AGACTACATCAGAGAATCCAAACACTTAAACAAGAAGGTAATAATTCAAGAAACTTGTAAAGTGCCTACAA
TAGTGAGAATCGAATAATTAGCTCGCTTGCAGGAACCTTTTGAACTGTAATTTGGAGAGATCGTAGTATTGTA
TAAATCTACGTGGCATATACGAATAATATAGGTGTATGGCTCAAGTCAGTTGCATACACTAGGCTAGCAAAT
ATA
>TCONS_00010187
GCAAAAACAAAAGGGAAAACATATTTTAGCTGACAAAAATCCTTAATTTCATAAGCTGCAAACATAATCCCA
TAATTCAAATTCATTACATCTTGTCACAAACAAGACATTAGGAGAGTATAGGGCAAGAAATGTGGGGGAAA
AACTAGTGCTAAAGTAATAAGCAAAGTAACACTCATCTAATCACCACCAGCCTTCTGGTAAACATAGGTAAG
CCCACATTTACCACAATAGTGCCTGTCAAAGTGGTTAGCCATGAAAGTCCCGGCACCACACTCAGCATTGGG
ACACTCCTTGCGGAGCCTCTGAACCTTACCAGAATCATCAACCTTGTAAAACTGGAGGACAGCGAGCTTAAC
CTTCTTCTTCTTGTGCTTAATCTTCTTTGGCTTAGTGTAAGTCTTCTTCTTACGCTTCTTTGCACCACCACGGAGG
CGAAGGACAAGGTGGAGGGTGGACTCCTTCTGGATGTTGTAGTCAGCTAGTGTTCGGCCATCCTCAAGCTGCT
TTCCTGCGAAAATCAACCGCTGTTGGTCCGGTGGAATGCCTTCCTTATCCTGAATCTTAGCCTTAACATTGTCA
ATGGTGTCCGATGACTCTACCTCTAAGGTAATAGTCTTCCCCGTCAGGGTCTTCACGAATATCTGCATCTTTCC
CAACTATGTGCACCAAGATTATCAAGGTGAGGAGTGCCGCTGCACAAAGAGAAGATA
>TCONS_00010320
GAAGCCAAATGTATATAGTGTGAATGAATCACAACTACTATACCAAAAAAATATGGCAGCCACTAAATAAT
AAATAAGACAATAAAGCAACAATAAAAGGAACACCAGAATTTACGAGGTTCGACCAACTTTGCCTACTCCT
CGGACACAACCAATATTTTATTCCACTCCAAAAATACAAGTGAAATAATACTAAAGAGAAAAGATACAAAT
GTCTTAAACAGATGAGAAGGCAAATGAGAGGTGTGTTTAAATCCTAAACATTAGGCCT
>TCONS_00010451
CTTCGAGTCCAGACCTTTTAACATAAAATAAAGAATAAGGGACTGACTTCTTTCTTGACAAGGATCCTGAATA
AATCGCACATGCTCCAAGGACTTCACGGAATTAGGAGGCACACAGGTGGGTATACCAAGAGAAATGCGGGT
AAGCGACGAGGGAAATTGCCTCAACAAAACAAGTTTTTATTTCCAGAAAGATCCCCACGCTGAGGGATGAA
CCTCGCCAAATGATCTTATGGATCACCCATCCCATCTTTTAG
>TCONS_00010489

CGGCAGTAGTAAAGCGTGCTGCAAAATTTTTTGCAAGTCTTCTTGCGAAGGGGGTAGAAACAACAGCTTTCTA
TGTGAGAAACGAGACCTTACTCTCTTCTCTAAGAGTTGATCGGCATCCAATCTGCAACTCACACCAATGACAA
CAGCTTGCGACGTTACAGACTGCATTACATCTAGCAAGCTATAGAGTAATCGCTGTTTACCCTGTGTAAAAAG
GTCAAACTCATCCAACACAAAGATAATGGTTTTATGTGCCAACCCGCAC
>TCONS_00010764
TGAATCACTTGTGGATCTACAAAACTCTCCAGCAGAATTTCACCATCTTTTTTCTGGAAATCTGTCTCTCCTAA
GAAGTGAAGGGATGGGCAGTTGATAGGAGATGAAAATACATTGGCAGCTAATTGTGGTACCCCAAATGAAG
GTCCTCCAAATTTAGCTCCTGATATTATGATTACAAACTTTATTTCTTGAACTTTTGTTAGAGCCACTCTTTCCC
TTTGCATGCCTGGAATTGCTGCACCTAAAACTGCTCCCTATTTT
>TCONS_00010774
GCAAACCCAGTAGCTACTTGTACTAAAAAACAAGTAAGCGTAATTCCCCCTAAACAATAAAATATATTGACG
TGGGGAGGAACGTATTTACTAGTTATATCATCCGCAATTGCTTGAATCTCGAGACGTTCTTCGAACCAATCAT
ATACTTTATTGAGATAGGCGTAACTCCCCCAGTAGCTACTTGTACTAAAAAACAAGTAAGCGTAATTCCC
>TCONS_00010775
GCATTAACATAAATACGGCAGTAAGAAGCGGCAATACAAAAGTGTGTAAACTATAAAAACGGGTCAAAGTA
GATTGTCCCACACTAGCGCTTCCGCGCAATAATTCGACCAAGGGTGATCCTATTACAGGAATAGCGTCAGGG
ACACCTGTTACTATTTTCACTGCCCAATAACCAACTTGGTCCCAAGGTAAGGAATAACCAGTTACGCCAAAA
GATGCGGTTAATACAGCCAGAACCACACCTGTAACCCAAGTCAATTCGCGAGGTTTTTTAAATCCGCCGGTG
AGATACACACGAAATACATGCAGGATCATCATTAGGACCATCATACAGCTACGCCAAAAGATGCGGTTAAT
ACAGCTAGAACCACACCTGTAACCCAAGTCAATTCGCGAGGTTTTTTAAATTCGCCGGTGAGATACACAGGA
AATACATATAGGATCATCATTAGGACCATCATACTTGCCG
>TCONS_00010776
TATCCATTACAAAACAAAGAATTCCGTTCTTACAAGTAAATACTCAAGACCCAAGTAGGCTTACAAATCAGA
TCATGATATGATCAAGTGCTTTCTGGGTCGTCTCAGACCTTCTAATTGGTATTTATCTCCAAAATATTTGGAGA
TACACATAAAAGGTTTGCTTGTTACTAATATAGTCTAGCCTCTCTTCTTCTTTAGATCCCTTGTTTCACTCCGAT
AGTAGTATTGATCGTGTCAATGCAGAGGAAATGAATACATTTACATACTATTTAATCTTATAAGTGAACTAGA
TAAAACGGAATCTTGATTCCGTTTTATCTAGTTCACTTATACTGGATCTTACGGAGCCTGCTCATGTACAACAT
GATTCGGACCTGATCATAGAAAAGATTCTCTTTAAGCGAACCAGCCTATCCTCTGTATAGGGCTTACACGGCG
ATTGGAACAAAGACACATTTGGTTGTGAATTCCCATGTGGCAGGCATATATCTACACAGACCAATCAATAGTT
CAAGTCACACACTCCCATAATCCATTTTCTCTTCACAAAATTCCCTTCAATCCCCCCGTTTAGTTGACATGAAT
AGTTAAAGGGAAATATCCAAATCCATGTCTTATTATTTTCAATAATCCATACTTGTAGCAATGAAATACTATA
TATTCCTCCACCAAGTGATAAATGATTGATTACAAATATTTAGGATCTATTTTTCTTTTCTCTATAAAGGCCCA
GAAATACCTTGTTTACGTATCATTGGAAAGTGCATTAACATAAATACGGCAGTAAGAAGCGGCAATACAAAA
GTGTGTAAACTATAAAAACGGGTCAAAGTAGATTGTCCCACACTAGCGCTTCCGCGCAATAATTCGACCAAG
GGTGATCCTATTACAGGAATAGCGTCAGGGACACCTGTTACTATTTTCACTGCCCAATAACCAACTTGGTCCC
AAGGTAAGGAATAACCAGTTACGCCAAAAGATGCGGTTAATACAGCCAGAACCACACCTGTTACTATTTTCA
CTACCAAATAACCAACTTGGTCCCAAGGTAAGGAATAACCAGCTACGCCAAAAGATGCGGTTAATACAGCT
AGAACCACACCTGTAACCCAAGTCAATTCGCGAGGTTTTTTAAATTCGCCGGTGAGATACACAGGAAATACA
TATAGGATCATCATTAGGACCATCATACTTGCCG
>TCONS_00010777
ACAATTGAATCAATTTAAAAATTTAAAAAAGACCTAAAGTTAGGGATTTATCAATAGGTAATGTTGCACCAA
TGCCCAACCAAAGGGCGACTGCGGTACCAATCAAAAAGACAATCTTCGCTACTGGACGGCGAAATGGATTTT

GGAATTTATTAACATTCTCTAAAAAAGGTACTGTTAATAATCCCGCAGGTACTGAAACCATTAAAAGAACCC
CCAATAATTTATTGGGCACTGTACGAAGTATTTGAAATACAGGAAAGAAATACCATTCAGGTAATATTTCCA
AAGGGGTTGCAAATGGATCTGCCGGTTCACCAATCATTGATGGTTCTAAAACGGCTAAGCCTACATTACATGC
AATAGTACCTAGAATTACTACTGGAAAAATATATAAAAGATCATTGGGCCATGCGGGCTCTCCATAATAATT
ATGACCCATACCTTTAGCCAATTTAGCTCTTAATACAGGATCATTCAAGTCAGGTTTTTTTGTTATTGGGATAG
GTGAATTCTTATAGATCCATCCCCCGAGGGAACTGGACATGATAATTTTTAATCATCCAGCTCGAGCAAGAAT
AAAAAGAACCAAATGAATCCATATATCCATA
>TCONS_00010778
ACAATTGAATCAATTTAAAAATTTAAAAAAGACCTAAAGTTAGGGATTTATCAATAGGTAATGTTGCACCAA
TGCCCAACCAAAGGGCGGCTGCGGTACCAATCAAAAAGACAGTCGTCGCTACTGGACGGCGAAATGGATTTT
GGAATTTATTAACATTCTCTAAAAAAGGTACTGTTAATAATCCCACAGGTACTGAAACCATTAAAAGAACCC
CCAATAATTTATTGGGCACTGTACGAAGTATTTGAAATACAGGAAAGAAATACCATTCAGGTAATATTTCCA
AAGGGGTTGCAAATGGATCTGCCGGTTCACCAATCATTGATGGTTCTAAAACGGCTAAGCCTACATTACATGC
AATAGTACCTAGAATTACTACTGGAAAAATATATAAAAGATCATTGGGCCATGCGGGCTCTCCATAATAATT
ATGACCCATACCTTTAGCCAATTTAGCTCTTAATACAGGATCATTCAAGTCAGGTTTTTTTGTTATTGGGATAG
GTGAATTCTTATAGATCCATCCCCCGAGGGAACTGGACATGATAATTTTTAATCATCCAGCTCGAGCAAGAAT
AAAAAGAACCAAATGAATCCATATATCCATA
>TCONS_00010781
GTTCTTACAAGTAAATACTCAAGACCCAAGTAGGCTTACAAATCAGATCATGATATGATCAAGTGCTTTCTGG
GTCGTCTCAGACCTTCTAATTGGTATTTATCTCCAAAATATTTGGAGATACACATAAAAGGTTTGCTTGTTACT
AATATAGTCTAGCCTCTCTTCTTCTTTAGATCCCTTGTTTCACTCCGATAGTAGTATTGATCGTGTCAATGCAGA
GGAAATGAATACATTTACATACTATTTAATCTTATAAGTGAACTAGATAAAACGGAATCTTGATTCCGTTTTA
TCTAGTTCACTTATACTGGATCTTACGGAGCCTGCTCATGTACAACATGATTCGGACCTGATCATAGAAAAGA
TTCTCTTTAAGCGAACCAGCCTATCCTCTGTATAGGGCTTACACGGCGATTGGAACAAAGACACATTTGGTTG
TGAATTCCCATGTGGCAGGCATATATCTACACAGACCAATCAATAGTTCAAGTCACACACTCCCATAATCCAT
TTTCTCTTCACAAAATTCCCTTCAATCCCCCCGTTTAGTTGACATGAATAGTTAAAGGGAAATATCCAAATCC
ATGTCTTATTATTTTCAATAATCCATACTTGTAGCAATGAAATACTATATATTCCTCCACCAAGTGATAAATGA
TTGATTACAAATATTTAGGATCTATTTTTCTTTTCTCTATAAAGGCCCAGAAATACCTTGTTTACGTATCATTGG
AAAGTGCATTAACATAAATACGGCAGTAAGAAGCGGCAATACAAAAGTGTGTAAACTATAAAAACGGGTCA
AAGTAGATTGTCCCACACTAGCGCTTCCGCGCAATAATTCGACCAAGGGTGATCCTATTACAGGAATAGCGT
CAGGGACACCTGTTACTATTTTCATCGGACACTACAGTGGAAGTGTGTTGAATCAAGAACAGACAGTAAACG
CCTTTATTATGGACGCTTTATTCTGTCTCCACTTATGAAAGGCCAAGCCGACACAATAGGCATTGCGATG
>TCONS_00010888
TTGCGAAGGGGATGTTGATTGTGTTGACCGTTGCCCAAGGCCATCAAGTGCACCTTATCGGAGCGGAGAAAT
TTTCGCCGCTCCAATTCATCCTCGTGCTTTTTAATATTTGCCTTCAACTTCTCACCGCCATCAGGTAACATAGCG
CCTCGGGAGCCAAGCAACTTCTTGCGCCTGGCTATTTTGTCGGTTAGTTCAGCATTCGTTAGTACCGAAAGCTC
GTCACTTTGCTCTTCTCCATCCACGGCAATCGTCGGTTGGTGTATCTCCGCCGCTGCCGCCGGCGGTGCTTTGG
TGGTGACCACAATCTCTGCCGGAGGCTCGTCATCGGCGTCGTTGATCAGCTTATTCCAG
>TCONS_00010956
GCCTTTGATGGGAATCTATGGGAAGTTAAAGATTCGGACTTCCCTGAACTCAAATACTTAAAATTGGACAGCC
TCAATACAGCTTTCAGATTGGGCCACAAGATATCCTCGATTCTTACCTGATTTTGCTGTCAGCTGGTGCACCAC
GATCACGGTCACCAGCATGCAATCGCGAAAGCAGGTCTAGCAAAGAAGCACAATCG

>TCONS_00011084
ATAAAATTCTGTAATTTTCACCTAAGAAAGTCGCAGGCAGGCCGCGAAATTAATGACAAAAGGAAAGTCTTT
CGCTGAAATTAATCAGACTCACAACTGAAATGCAGTTGAATGGAGCTGGACACATTCGTTTAAAGAGCATAT
TTGCTACTTCTGCAAAATTAAGGTTGAAATTTACAAAATAAATAACTCATTACATGGACAAGTAACACATTCT
ATCCGACCGAAAAATAATCACCAGAACAAAGCAGAAATCTTAAATGCAGCCAAAAGCTGGTAAAACAAAC
AGGAGAGGGAGAACTACAGCCAACTAAGATGATCTGCAGAATGCACAGGATCTGATAGCAAATACATCTCC
TTTTAGTGTTTAGGCTACAGAGTTGAAGTTCTAGATTTTCTCATCTTTTCATCTTGTGAAGCAGTATGTGGTAAC
GAGGAAGATGAAATCGCGGCATGGTAAGACGAAGATGCTAGCTGAGCAAATGCTGGAACAGCACCACCTGC
TAGCCCTGCACATGCAGATGAGAAAACTATCACAGGAGGGGCACCAACAAGGAAATGCAACCACATAGGTC
CTTTATCCACCTTCTAACGATCGGCATCACATGGTTGAACCCATAGAATGCACTTGTAGTCCCACCAACCGCA
GAACCAATCTGAACCATTAAGTAGGTAGTTAAG
>TCONS_00011208
GTGAAAACTACTAATAATATTATCATTACCAACAAATATTCTAGTCACAATTCACAACTGTGATCGACGACTT
TTCATATGTTGAAAACGATGAATAATGGCATCGTTGTTCACAGTTGAGAATATTTTACGCTCTACATAGCAAA
CTAAATAACTATTTAAAAATTCATCACCTATGGTATTACGAAGCTCATTTTTTATGTAGTTCATGGATGAGAAA
GCACGTTCCACAGAAGCGGTAGCAACAGGAAGAATGAGAGTCAACTTGATAAGTAAATACACCAGTGGCCA
AGTTTGATGCAGGTCTGACTTAACCAACACTTTAGAAAGGTCACTAATTCCCTTCATGTTGAAGAACCTCTTGT
CATGACAACGAGCATAAACTATAAAACTATCAAGCTGGAAACTAAGATCCCGGAGCTTGTTGCTATCAAACT
CATTCGGATAATATTCAGCCAACTTCATTATCCTGTTCTTATCAAAATTACCAAATGAATTAAGTGGATTTAAA
CTAGCCATACCGAGAAGTAAGTCGGTACTCACGGCATCAAAACGACTTCTAAGCTCTTGAAGATGCAAATCA
ATAACACCATTAAATATCTCAACGTGCAAGTGATGAGAGTATGTAACATCAAGAGATCTACGCTTTGACTTCC
CAGGAAAATAGAGAGCATCCATCTTCGGGATCATTATCTCATGTTTATCACAGAATGAAGAGACATTATCCAT
TAATGATTGAAATTCACTATTCCTCATCCTTTGTAATCTTTGCTTTGTATGAGTGAGGAGTCTCATAGCATTGAC
AATATCTTGATCCATCCTTTGTAATGAGGAGCTCAAATCATTTGTCATCATTAGAACTTTCCACATCAAGTGCA
ACATAAAAACAAATTCAAATTCCTTAATCTTAGTCATAAGACTTTCAGCAACAAGTCTATCAGCATAATTTGG
ACCCTCACGAGCAACAAATTCAAGAACATGAATAATTGATGAAAATAGGACAATGAGGTTATCTAACGTTCT
ATAATGAGAACCCCAACGAGTATCACCTGGTCGTTGAAGCCCACGTTCTTGATTTAGTCCTCGTCCAGTATGC
ACTTCACCTGATATGAGCAACTCCTCTAATTTTTCAGCTTGATGTTGTCAAAGCAAATCTCTGCGCTTAAAAGA
TGATCCAACAATATTTAACACATTAGTAACTATACAGAAAAAGTCATCTACATCTGAATGCTTCTTTGCAAGA
GCCACAAGTGTTAACTGCAATTGGTGAGCAAAACAATGAATGCAATACGCCGATGGAGTTTCATTCAGAATC
AAAGTTTTAAGACCACGTAGCTCTCCTTGCATGTTACTA
>TCONS_00011317
GTGACTACTTTTGATGAACCCGATGCAGATGAACCTGTATCTTGACTCTCATCTTTATAGTTCTCAGCTGACTC
CTTAGATTGCTTCCCTAGGCGGTATTTCTGGAGATGTGACTTCAAATGATATAGGGTGAGTCCTTTCACCCCCA
TCGCCTTCATAATTGTTTTTGGCGTTGCCTTATCAGGGCCACCGAGTTGGGTGACGGCGTCAACGAAGCGTTC
ATGGAGCTCCGCCGTCCACCGGAGACGAGGCTTATGATCCGCCGTCAAAACCAAGCAAGCATCCCCCAACAT
ATTTTGATATTCACCAACTCCGCCACTGCCACCGCCTATGGCGTTTCCTTCCATACCAAATGGCAAAGCCGAG
TACATTTTCATTCCAAATACTACACTAACCAAAGAATCTCTAATTTCAC
>TCONS_00011396
TGAAGAAATAGCATAAATGAGTATAGTAGTTGTTTTCTACGGCTCCTTGCAACTCTTCTACACTCGAGAGTTC
ACAGAATGAATCCAATGCACCATAAAATCGAAAAGGAGGTGAATACAAGACAAATATCGCGTATGATTTAC
ATAATTGAAAAAGAAAAGAAAAGAAAAAGAAATGCACAACAGAACAAATACGGTATGCACAACTCAGGCA

AGGAGATAACAGTAAACAAACAGCTCTCCATGAATAGCATTCCTCTGATACTTCTTTACAGTGGCACCATATA
GAAAATTGGATGGGTCCTTCCTTACAGATTTACATGATATCTTGTCCAGGCTTTAGATATAGCAATCTCCTTGA
AGGCGCTGGAGGAGCCAGTCCAGGGATATCCCTTATGTCTTTGTTGTGAGGATTCACAATCTTCACAACAATA
ATGGCAACAACACCACAGACAATGAGGAAGAGGAAAAGCATGATGCATTTATCCGTGGCAACCTGCCGTCC
AATTTCCTTGACAAGCTGGGATGCCTTTTTAATGGAGAACTGAATTGTGTCAAGCTCATTGACAACACGACCC
ATTTGATCAGTTTGGCCTTTCAAGGTAGTAGCAGTTTGTGTTCCCACTTCAATTGTTTGGTGAACAACCTGTTTG
GACCGTTCAATCGCTTGATCAGTTTCATCCATTGTCTTATTTCCAGCACTGATAAGCTCCTGATTTGACATTGCT
GATGCCATTTGGACATTTTCCTCTGCTGTTGGTTCACTTGCACCTCCCATGTCAAATAGTTCAACCCTCTTATTG
CCAAGACTGCTCATATACGTTTTTCTCAAGGCCACATATGAGTTTAGCTCTTTGATCATTGATTGCTTCTCATCA
TTGAGTTGTTTGCTGATCTCAGGAGGATTTCTACTCTCCTCATCTTTAATTTCACGATCAAACTCTTTAATTAAC
CTCTTACATTCTCTCATCTTCCCTGTAAGCTCTTCCAACTGTTTACTCTGTCTGTTGGGATCTTTGATCTTATCCA
GCTTTTGAAAACCATTTGCTAGCGCTCGGAAATTATCACGAATTTCTCCATGGATCTGCTCCATTTGTGGGTTC
ATTTGCAAGTCCAAATTCGCCATTTTTTGGGCCTCCAATTTTCTCTTCTTTTTCTTCTTCGTTTATTGCCGGATTA
GA
>TCONS_00011446
GAATGACGGAAAAATGTGACATAAGGTACACATCATGCTTGGAGGTTGCCCACACCAAGTTCCTCAGCTGGA
AGTGTAAAATTGTTGATTTAACAGATCTTGAATTACGTCTGAAGTCATAGTACGAAGCCTCTTTGTTTGTGGCT
TTGCAGTCCTTCTCTGATGCCTCCCCAGATTGTGGAACATTTTCATAGTTCTTATATTGCTCTAATCTTGTTTGC
CTGTATTTCTCCCTGGTTATGCTAAGTCTTTCCCAGGGAATCCCTTGAATTTCTCTACCTCTTCTTGCCTCAGCA
GCAGAGGTATCTTGCATTTTATTATTCATGTAGTCATACTCATCTGCATCTGAATCAGAAGCATCAATATCTCT
GCCATGGAACTCATCATCCATATCATCATCTATAGCTTCCATTTCATATTCATTTGCCATGTAATCAGCATCAT
CCTCTTGTTGATGGGACATATTTTACCCCTACTAAATTACCTACAAACTAC
>TCONS_00011483
AAGAGAGCCAGCTTCAATAAGTTGGGATTGATGGATCAACTTTGACAGCATATTCGTTGATTCCTCCAGCAAT
GTTAAATACTTTCCTGAATCCCTGTGTCTGCAACCACTTGGCGACTTGTAGTGATCTCATGCCATGGTGACACA
AGACATATGTGTCCCTTTGAGGATCAAACTTAGTTGTTATTTCTGGTCCCCAGCTTCCAAATTGGCGAAGAGG
AAGAACCTGAAATCCTGGTAAAGAAGCCTGGGCCACTTCCTCGGGTTCTCGAACATCAATCAATTGAGCTTCT
TCCAAGAATGATGGATCTTGCAGCTTCACATGAAACTCGTTGGGTTGGATGTGTTCAAGTACAGATTCCTCTCT
CTCAGATAGAACTTGCAATAAATGCCATCCAAACTTAGTTTTACACTTTACAACCTTATTCAGTGGTGCACCA
AATGCAGCTTCTTCGAATTCTGGTACCATTTGCCCTTTTCTCACCCAACCAAGCATTCCCCCTTCTTCTTTTGAT
GGACAAAGGGAATACTGCACGGCCAGATCACTTAGATCCTCTCCTTCTACAGTTCTTTTCAGAAGCTCCAGTA
AAAGTTTCTGGTCATCTTCTTTGACAAGTAAATGCTGTACTAATATCTCCCGACCACCTCCAGAACTCCCTCCA
GG
>TCONS_00011548
AAGGAGGCCTAATGTTTAGGATTTAAACACACCTCTCATTTGCCTTCTCATCTGTTTAAGACATTTGTATCTTTT
CTCTTTAGTATTATTTCACTTGTATTTTTGGAGTGGAATAAAATATTGGTTGTGTCCGAGGAGTAGGCAAAGTT
GGTCGAACCTCGTAAATTCTGGTGTTCCTTTTATTGTTGCTTTATTGTCTTATTTATTATTTAGTGGCTGCCATAT
TTTTTTGGTATAGTAGTTGTGATTCATTCACACTATATACATTT
>TCONS_00011615
ATCCCTGATGCTCAATGGGCTGCTGATGGCGCACCTGCTGTTCCTGCAGTTGCTAGTGGCTGGTCTGTAGATG
GAGCGGTAGAGGGAGGTTGGGACGCTGCAGCTGCTCCTCCTGCTCAAGTTGCTCAAGTTCCTGGAGCAGAAG
GGATCCCTGCTCCAGCTCCTACTGGCTGGGGATGAGACCTGCTTCCTTTAGAAAGTTCTTTTTGATTAAGATTA

GATAATGTGAGTTTCTATGACAAGACGGTTTTGTTCACAATGCAAGTTATAAAATTTTGGATTTGGTGATGTAC
TAAATTATTTTTAATCATTTGT
>TCONS_00011807
GCAAACAAACTCAACTGAACTTGATCATAACTTAGCATATTTCTTGAGATTGACAGCAGCATCTTTGGCATTT
TCAGGTACAGGCATGTTGGTCTTAAGGGTGACTCTTGATGATGCTATCAAAGTCTTGTTTTCCTCATTGTTTGG
ATCAATCTTCCAAAGACAAACTTCCCAAACATGAATAGTTTTGCCAATGTTGACTGGTGTAGCCTCAGCAAAA
ACAAGGTCACCAATTTGAGCACTCTTGAGATGGTGTATGCTGAGATGAATTCCAGCTACTCGCGAAAAACCA
GAAGCCATATGAGCTCCCATACTTGCCAAAGCCTCAGATATCATAGCTGAAACTCCACCGTGCAACACCTTG
AAGGGCTGGCAACACTTTTCTGTCACAGG
>TCONS_00011808
GCATCTTTGGCATTTTCAGGTACAGGCATGTTGGTCTTAAGGGTGACTCTTGATGATGCTATCAAAGTCTTGTT
TTCCTCATTGTTTGGATCAATCTTCCAAAGACAAACTTCCCAAACATGAATAGTTTTGCCAATGTTGACTGGTA
CAGACATGTTGGCCTTAAGGGTGACTCTTGATGATGCTATCAAAGTCTTGTTTTCTTCATTGTTTGGATCAATCT
TCCAAATACAAACTTCCCAAACATGAATAGTTTTGCCAATATTGACAGGTGCAGCCTCAGCAAAAACAAGGT
CACCAATATGAGCACTCTTGAGATGGTGAATACTGAGATGAACTCCAGCCACTCGTCCAAAACCGGATGCCA
TATGAGCTCCCATACTTGCCAGAGACTCAGCTATTAGTGCTGAAACTCCACCGTGCAACACCTTGAAGGGCTG
GCAACACTTTTCTGTCACAGG
>TCONS_00011868
ATCCCCATATTTGAAACGGTTTCCAACGGATCTAACACTCAAGCTATTATTCTCGAAGATCTTACAAACAGAC
TTTCAACAACCTTCCCCATATTTGAAACAGTCTCCAACGTAGCTGGAAAAATTGCATCTGGTTTAGGATCCTC
AAAAATTATAAGGCATCCAGCACCCTGGTCCAGGGTCCCAGCGAATTTCTTATCCAAAATCATTTGAGATAA
CTTCTTCTCAACATGGTCACCAGGCAACTCTATCAACTCAGCAATATGAGCAATCTCAACTCTCGAGAAGGGC
TCAATCAATCTACAAAGGTTCTGCTCAAGCAAAGTGTCATAGAGGGAAGACAGGTGCCGGTGGACGATAGG
ATCTTCATCTAGCTGAGCCTTAAAGTTCCGAAGAGCAGTCTCGAAGAGCTTCAAGGAGCGCTTGGAATGTGC
ATCCGCAACAGCTTTCATTGCATCGACTTCTGGCCCCTGATATTGCAATCCAACCTTTGGGGATGATATTATTC
CTGCAACATCATCAGACTGGTTGACCATGATTTTGCAAAGCAACATATACTTGAGGCTGTATATGGCCTGGGG
ATCTTCAAGGGCATTGAATGCTTCAAATGCTTCATAGAAATAGCTGTAACCAGTTTTATAATCCTTCTCTTCCG
CATGAAGGATCCCGCTCTGCAAATCAATAGAACCTTGCTGAGCTGGAGGCACATAAATAGCATTGGCTGCTG
TTCGAGCAGCTGTGAGCGCAGCCTTGGCTTTGGGAAGATTTCTCAGCGAGAAATGTAGCTTGCTCTCCAGCAA
GTCAATCTCCACAAGAAGCAGCTTATCATCCAATCTTCTCACCTCCTTAATCAGTCCTGAAAGGAGGGTCAAT
GCTTCAGAGTATTCCTTGTTTTCCATCAAAAGAGCTGCGAGTCTTGCCTCAATTCGTTGACGAAGAAATGTCC
GCTTCTCCTTACGGGTCCACTCCACTATATCCTTGCAAAGTGTAACCTGAAGGTCAGATGTACCTGGTATTTTA
GCCACTGCATCAATGATTCCTCGAACAATTTTTGCAGTTTTTGCCTTGGGGATCAAAGAAAAGAAGGGGCGC
AACTGGGTCAGAAGGTTTTTAAGTTCCTCTGCCCTATTCTCCTGTCTAAGGAGGTCAGAGAGATTGGAGATTG
CCTGCTCTTTGATCCTTAGGGCCTCAGAAGCAGAAGATGGGTTATCAAGGATGCGGTAAAGAATAGAGATGG
CGTTAGATGGTGATGTAGCCTCAGAAGCCTGAGCTAGAGAATCAGTAGTGGCTGGGAGATATGAAGATGACA
TGGTCACAGCTTGCAGATTTTAACTCAAATTAATTTCACGGGAGAAACAGATCGGAAGGAGCTCGCGGTAGA
AAGGAGATGA
>TCONS_00011869
ATCCCCATATTTGAAACGGTTTCCAACGGATCTAACACTCAAGCTATTATTCTCGAAGATCTTACAAACAGAC
TTTCAACAACCTTCCCCATATTTGAAACAGTCTCCAACGTAGCTGGAAAAATTGCATCTGGTTTAGGATCCTC
AAAAATTATAAGGCATCCAGCACCCTGGTCCAGGGTCCCAGCGAATTTCTTATCCAAAATCATTTGAGATAA

CTTCTTCTCAACATGGTCACCAGGCAACTCTATCAACTCAGCAATATGAGCAATCTCAACTCTCGAGAAGGGC
TCAATCAATCTACAAAGGTTCTGCTCAAGCAAAGTGTCATAGAGGGAAGACAGGTGCCGGTGGACGATAGG
ATCTTCATCTAGCTGAGCCTTAAAGTTCCGAAGAGCAGTCTCGAAGAGCTTCAAGGAGCGCTTGGAATGTGC
ATCCGCAACAGCTTTCATTGCATCGACTTCTGGCCCCTGATATTGCAATCCAACCTTTGGGGATGATATTATTC
CTGCAACATCATCAGACTGGTTGACCATGATTTTGCAAAGCAACATATACTTGAGGCTGTATATGGCCTGGGG
ATCTTCAAGGGCATTGAATGCTTCAAATGCTTCATAGAAATAGCTGTAACCAGTTTTATAATCCTTCTCTTCCG
CATGAAGGATCCCGCTCTGCAAATCAATAGAACCTTGCTGAGCTGGAGGCACATAAATAGCATTGGCTGCTG
TTCGAGCAGCTGTGAGCGCAGCCTTGGCTTTGGGAAGATTTCTCAGCGAGAAATGTAGCTTGCTCTCCAGCAA
GTCAATCTCCACAAGAAGCAGCTTATCATCCAATCTTCTCACCTCCTTAATCAGTCCTGAAAGGAGGGTCAAT
GCTTCAGAGTATTCCTTGTTTTCCATCAAAAGAGCTGCGAGTCTTGCCTCAATTCGTTGACGAAGAAATGTCC
GCTTCTCCTTACGGGTCCACTCCACTATATCCTTGCAAAGTGTAACCTGAAGGTCAGATGTACCTGGTATTTTA
GCCACTGCATCAATGATTCCTCGAACAATTTTTGCAGTTTTTGCCTTGGGGATCAAAGAAAAGAAGGGGCGC
AACTGGGTCAGAAGGTTTTTAAGTTCCTCTGCCCTATTCTCCTGTCTAAGGAGGTCAGAGAGATTGGAGATTG
CCTGCTCTTTGATCCTTAGGGCCTCAGAAGCAGAAGATGGGTTATCAAGGATGCGGTAAAGAATAGAGATGG
CGTTAGATGGTGATGTAGCCTCAGAAGCCTGAGCTAGAGAATCAGTAGTGGCTGGGAGATATGAAGATGACA
TGGTCACAGCTTGCAGATTTTAACTCTGACAGCTAAGGGCATGCAATCCTCAACACAAGCCCCTGGGTTGCCA
ATTTAAGCCAACTCACATTTAAATTAATTTCACGGGAGAAACAGATCGGAAGGAGCTCGCGGTAGAAAGGA
GATGA
>TCONS_00011870
CCCCATATTTGAAACGGTTTCCAACGGATCTAACACTCAAGCTATTATTCTCGAAGATCTTACAAACAGACTT
TCAACAACCTTCCCCATATTTGAAACAGTCTCCAACGTAGCTGGAAAAATTGCATCTGGTTTAGGATCCTCAA
AAATTATAAGGCATCCAGCACCCTGGTCCAGGGTCCCAGCGAATTTCTTATCCAAAATCATTTGAGATAACTT
CTTCTCAACATGGTCACCAGGCAACTCTATCAACTCAGCAATATGAGCAATCTCAACTCTCGAGAAGGGCTC
AATCAATCTACAAAGGTTCTGCTCAAGCAAAGTGTCATAGAGGGAAGACAGGTGCCGGTGGACGATAGGAT
CTTCATCTAGCTGAGCCTTAAAGTTCCGAAGAGCAGTCTCGAAGAGCTTCAAGGAGCGCTTGGAATGTGCAT
CCGCAACAGCTTTCATTGCATCGACTTCTGGCCCCTGATATTGCAATCCAACCTTTGGGGATGATATTATTCCT
GCAACATCATCAGACTGGTTGACCATGATTTTGCAAAGCAACATATACTTGAGGCTGTATATGGCCTGGGGAT
CTTCAAGGGCATTGAATGCTTCAAATGCTTCATAGAAATAGCTGTAACCAGTTTTATAATCCTTCTCTTCCGCA
TGAAGGATCCCGCTCTGCAAATCAATAGAACCTTGCTGAGCTGGAGGCACATAAATAGCATTGGCTGCTGTT
CGAGCAGCTGTGAGCGCAGCCTTGGCTTTGGGAAGATTTCTCAGCGAGAAATGTAGCTTGCTCTCCAGCAAGT
CAATCTCCACAAGAAGCAGCTTATCATCCAATCTTCTCACCTCCTTAATCAGTCCTGAAAGGAGGGTCAATGC
TTCAGAGTATTCCTTGTTTTCCATCAAAAGAGCTGCGAGTCTTGCCTCAATTCGTTGACGAAGAAATGTCCGCT
TCTCCTTACGGGTCCACTCCACTATATCCTTGCAAAGTGTAACCTGAAGGTCAGATGTACCTGGTATTTTAGCC
ACTGCATCAATGATTCCTCGAACAATTTTTGCAGTTTTTGCCTTGGGGATCAAAGAAAAGAAGGGGCGCAACT
GGGTCAGAAGGTTTTTAAGTTCCTCTGCCCTATTCTCCTGTCTAAGGAGGTCAGAGAGATTGGAGATTGCCTG
CTCTTTGATCCTTAGGGCCTCAGAAGCAGAAGATGGGTTATCAAGGATGCGGTAAAGAATAGAGATGGCGTT
AGATGGTGATGTAGCCTCAGAAGCCTGAGCTAGAGAATCAGTAGTGGCTGGGAGATATGAAGATGACATGG
TCACAGCTTGCAGATTTTAACTCAGCTAAGGGCATGCAATCCTCAACACAAGCCCCTGGGTTGCCAATTTAAG
CCAACTCACATTTAAATTAATTTCACGGGAGAAACAGATCGGAAGGAGCTCGCGGTAGAAAGGAGATGA
>TCONS_00011902
TGAATCATAACGAACTGCAGGGGTTCGGCTTCGGAAGTCCTCCATATATGAACCCGAGGGGATCCCACGACA
TTCAAACGGGTCTTCAATAGAATATTCTTCCGAAGATGCAAAGTGGATGCCAATCACATGAAGAATAGACCG

AACTATCTCCTTAACAACAGAGATAGATATGGCTGTGTTAACCAGAATCACACAGAACACTCCTGCATCTGC
TGGAGTTGGATATTGTGAGAGTCCCATTTTGAATCGGAACCTAGTCCAAAAATGAAGAGTTTTTCTTCAAGCA
AAACTTTTGAAAGAGCTTAATTCACTGGACCTGCATAGAAAAAATCAGCAATAAATATCAATCAGCAAAAG
GAAGAAATCCCATT
>TCONS_00011968
CTTGCACATACCTGCATCCTCTGTAATGTCAGAAGTCCACAAGGTGAGATTGTCCCTTAAAAGCTGCATAATC
AAGGTGCAATCTTTGTAGGAGTCCTCATTAAGGGCATCCAGCTCCGATATTGCTTCATCAAAAGCCTGCTTAG
CCAGGTGGCATGCCCTTTCAGGGGAGTTCATTATCTCATTGTAGAAAACAGAGAAATTTAAAGCCAAACCCA
GCCGAATGGGATGGGTAGTTCATAATTCAGCCTCCGCAATAGTTGTAGCTGTCTGATATGCTTTTAAAGACAG
ATCAGAA
>TCONS_00012106
CACTCAACACTCTGCGCTCACTGGGGGTGTGTACAGACTCCGGAGGGGCTCCCAAAGCCCAAGCGCTAAGCA
CGGACAACTCACGTGCTACCATATCAATACCTGGATCCGCACGGTCAACTCACGTGCTACGCGGACAACTCA
CGCGCTATGGTATCAATACCTGGATCCGCACGGTCTACTCACGTGCTACGCGGACAACTCACGCGCTATGGTA
TCAATACCTGAACCCGCACGGTCAACTCACGTGCTACGCGGATAACTCACGCGCTATGGTATTAATATCCTCA
CAACCAGGCCCT
>TCONS_00012107
CACTCAACACTCTGCGCTCACTGGGGGTGTGTACAGACTCCGGAGGGGCTCCCAAAGCCCAAGCGCTAAGCA
CGGACAACTCACGTGCTACCATATCAATACCTGGATCCGCACGGTCAACTCACGTGCTACGCGGACAACTCA
CGCGCTATGGTATCAATACCTGAACCCGCACGGTCAACTCACGTGCTACGCGGATAACTCACGCGCTATGGT
ATTAATATCCTCACAACCAGGCCCT
>TCONS_00012174
GGATGGTCAATGGCCATTCTTTTGATGAGAGAAACCAAAGCAAACTGGAAGTTTTGAGCTCCCTGACTTTCTT
TCGTGCTTCCCATCCTTGAAGCAATTTGGTAAACTAGTGGAATAAACTTGTAAGATTGAACCTCACTTATTGTG
CCGAGCATGCTATCCACAACAATTGGTCTAAATGAGAGACCAAACCACAGCGAAATAAGTCGAAATACCAC
CCTGACATCATATTTATCTCCTATTACTAAACAGCGTTTATATTCATCAAGCGC
>TCONS_00012175
CTTGTGCTTTCTTAACCGCATTGCTGCCTGCCATTCACTGGAATTAAGTCGTTCTTCATAACTACGAAACAGAG
CATCCGCATAGTGCGCAAGATGGAAATGCAGTTGACTTCGTTTTATGGCGGAAACCTTTCCTTGTGCCATGCA
ATCCTCAGCTAGAAGGACTGCATGCTTCAAGTATTTCTCTAGTATAGTTCTTGAGTTGCTTGATCGTGTTTCAG
CGAGCCACTTCCCAATTAACCGGAAAACATCAGATGCGTTTTCATTCGTCTGGTGGTTTAGTGAAATATACTTT
GCAAGATTGATTGCCATCTGATGTTGGCCTTGAGCACGCAGAAGCTTAGCTTCTTCAAGCCGTCCAAGCCAGT
AAAGGTTGGAATGTTCTCCCATTTCTGCACAGAGAAACTTGAATTCATGCAAAGCTGAAGCAGCCTGAGAAA
AC
>TCONS_00012176
TGGCAATTTAAAATATGAAGCAAAACTCTTCTAAACGCCACAAATGGTTCCAGCAAATTCATATGTAACTGT
GCTTGCTTCAAAGTGCGCTTCCAGTCCATGTCTAGCCATGACAACTGAGTAATTGATGGGAGTACAGGTTTAG
ATGAGACCTTTGGCATCTTCAAAGAATCCAGCATTGTGCAAGATGAAGTCCACCGTGAATCCCATGTCATCCC
GAG
>TCONS_00012177
TCCCAAGACAATGTGGATATTGATACTACAGATGAAGTGGTTGTCTGATGCTTTGCCTTCAGAGGCATGGAGC
GTGACTTTGATCCATAGCTAGAAGGCCTTCCATATTGCTTTGAATTTTCTCGCTTAGAGGGATTAGAGGATGTG

CTTCTTTCCATCACGTGACACAACCGAAGCTCATTAAGAGCATCTAAGATAACTTGAATTGATTTGGTCAACT
TATTGTCCTCCGTAAAAATGTTCTCTTGCACCTGTGAGGAGATCAGTTGACAAAGATCAACATCCACGTCTCT
TCTACTCGACAAATTGACCATCACATGAGGGAACAAGAGTTCTGCAACTTCAGATTTCACCAAGACAATATC
TTGACACAATCTCAGGATCATATCATCACAATATTCAATCAGAG
>TCONS_00012178
GGACAATGTCATGGTCAGAATCCAAATATGCATCTTCGAAAATATTTTCCTCATTCTTTTGTGCTGGGTATGCT
TCATCTTCAAACAATTTTTTGTGTAATGCTTTTAGACTCCAGACAAGTAACCTTGGCGGAAGGTAGCGAGATC
GTTTCCCTAAGATTGACAAGTGCTCCCTAGGCGAGTAATTTTGACTTAGTTCCTCGTGGAACATCCGTATATCA
TGAAATAAATCGAGGTTAGGGAATGGCTCTAGTTCTTTAATGTACTCGTGTAGAGATGGGTCAGAGTCCAAG
GTAAGTTGGCTGAGCAGAGACAAAACTTGAGATGATGTGTCAGCTGACAGTTTGGGACTAGACTCGGAAGA
AATGCAACACATTACCAACTTGGAAATTAAAAACTGAAGCTGTTCACCTAGGACAACAGTGGTTCCCTCCAA
TTGCTTTATTTTGAAAAACTTTAGCAATGAAGAGATCATTCGACAACATTGGTCCAGCAAAGCATCAAGGTCC
AAGCATTGACCAATCAAGTTGAGGAGATAACTGGCAGTACTAGGAACCGCAACTCGATGCCCTAATACATCA
ATAAGCACTTCAATCCCAGCAAGCCTGTCAGATTTATGCCTGAAATGAC
>TCONS_00012179
GGGCCATAAGTTCTTCCATATACTTTGACCTAGAAGTATACTTTAACTCCCTTGATAGATTATCAAGGACAAC
AATAACAAGTTTCCTTAGACAAGGGTTAATAGCAGCAATAACACATACCATAAAAACAGCCTCCAATTCAAT
TTTTTCACTGTGCAGCGCAAGATGAGCTAGGGTTACAATTATCGTTTCCAATATGGTACGAGGCTGAGGACCA
GCAGCTAAAACCTCCTTCGCCATCACCAGTTTCTCTCTGGAGCAAGTCACAAGCTTTGTGCCAAAATTTGAAC
ATATGTCCTGGAATAGTTCAAAATGACCATCCCAAGTTTGGAAAAGCACACCAACTCGTTGAGCAAAGCATA
ACCTAACTCGATAATCAGGATCCCGAAGCATCACCAGCAGCTTTTCAATCATAGACTGACCAATTTGAGGAT
CCACTAAAATGAAATTGCAAATGCAATCAATAAGCTTGGTCCGGCCAACCCAATCAAAGAGGTCGCTGTCTG
CAATTTTG
>TCONS_00012180
GTGTATGATTACTTCCAGGACCCTTGGATCACTTTCTTTCTCCATGAGCTCAAACATGACATCCCATGTATGAA
ATGGTAAAGCTGAAAAGAACTTTGTGATGATTGATACGACTTCCATTCTCTGATTTACAGACGAAGCCAATAT
TTCGGCAGTAGGTTTTCCCCCAATTTTTACAGCATCAGTATCTTTGGAACTAATGTTCACATCCAACTCCATGT
CCATGATCATACTAGTACGGCTATTTACTGGATGAGAATCGTGACTAGAAACAGATGCAGAAGAGTCAAGGT
CGTCCCTCTTTGAATGAAAATTACTTCCACTACTTGAGGATCCTTCATACAGTTTAGCAAGAACCTTCAGAATC
CTCTCCACAGATTGAATTACTGCAGCTAGTACGTCTATGCTGTTGTCGTTTTGCATCTCATTAAACAATGGGGA
GCATAGAAAGCTTTCAAAGGACACACGAATGGTCCCAATTGTGTCAAATATTGACCTTGAACCCGACAAACC
ACAAACGAGGTCGTCATAACTTTTCTCTAAGATAGAAGCAGCTCGATCCAACAACTTCGACACATATTGACT
CAGAGTGGCAAGGCAAGGCACAATTTCTTCTCCTAGCCTAAGAGTGAACAGCACATGAAGCCAACACAAAT
AGAAAGATTAATGAAGGCATTCCATTGCAAGCAAGAGATGAAACTTAAAAGCAGAGGGCAAAAAGACAAT
GCCAATAATCAGTTCC
>TCONS_00012181
CAGGAACATAGTGTGACTGGGAAAGTCCTTTATCTAGCAATGGTGTACCACCAGTACAGAGAACATAAACAG
CTGTAGGCAATATAGCAACCAGCTGTTTATTCAACATTGAACAGTCCTTCCAGAAAGGAACAGCCAAAACAG
CTCTCAAAAGATTTTGCCTCAAATATAAAGCGTCTCTAAGATCACCTTGATAGCCTTTCCTTGAAAAGTAACA
CAAAATGAAGCACAAAACAGATATTGATGGCACGCGTTTAAGTAATCGAAGATCCCACAAGTCTTGGGGCA
CAAGGTATGCATTAATTTTATCATTGAGTATTATGTTTCTAAGTAGCCTTAATGCAGCATCCACAACTGCTGTA
AAAGTACTGAATGTGGGCAAACCACCAATAACACAGTTCCATATCGTACGCCAACCTGTTTCAACCCCATTA

AATGTGGATGACAACTCTGAAGATGTTTGCAGTGCTGGAACAGGAAACCGCAGCATCAAAGAAAGTCCTTG
AAGACTCCTCAATGTCCACAAGAGACCTTCATAACTATGCTCCATGTTTGCATCATTTATTATCCTTTCAAAGC
TTGCACATATACCTTCAAACCAATAGATCAACAAATCCTTCTTTATGCGACCATAATAATTGTGGATGAGGTA
ACAGAAAGCTGCATGCCACGGCCACTTTCCCCTTGAGAGCCTCTCCCTTATCTGGACTACAACATGCTCCCTT
CTAAGTCGTTTCTCAGATGACGATGCTATAGGTGTGTTAAGACATGCCCGGTAAAAGACAAGTGCTGCAAGC
TCCATAAGACCACACTGAGAGCTTGTCAAGCTTCCACATTTGTCATCCCTGGTTGTGTCTTTCCAGGGTAAATT
GATGGTCCAACTGCTCATTTGATCTAATTCCTTCCCTAATACATCCTGAAGTTGTTCTAATAAAGAGCTCCCAT
CAGCAAAATCTCTCGTCAAATTTAGCTGTAGCCTAGCATAAAGAACAAGCGAATCCTTTAACCCACGATCAC
GAGTTGTCATCCAACATCGGAAGACAAAATGATGCAGAGCATCATGGATTTCCAGGGATTTAGA
>TCONS_00012182
CTCGGATTGAAAACTAGGAGTATCCTTTAAGACATCCCAGACATGATTGAAAAGCAATTTTGCCACAGGTAA
AAGAAGCATATCTTTGCCTGAAAATTTGGCATCCTCTGCTCGTTGGACAACAATCCGAAGAGTTTTAGCAAAA
TTCAACTTGGGCAAACGTTTTTTGCTCCCAGATATCTCCAGGGACACACATTTAGTAAGCAATGTAACAAGAA
AGGGCCACGTCTCAGAATGAGGAATTTCGTCTGGTTTAAGCCTCGCGGTCTTCTCTCCAACGTATCTGCAGAA
CTCAACAGACCTTTGGCCTTCTTCTAGCCATGCATTTAACAACTTAATCCCTTCTTCACGAGATTTTGCTTTATC
TGATGAGAGTTTGGAGACAATGTCCTGAATATCTCTG
>TCONS_00012210
CCCATGTGCACAATTCACTTTGCAAGTGAAGAAGAAGCAGTGTTCCAAGTGCACCGGTGCTGAGTACACTGT
TCCTCAGCGTCAGCGACCGTTAATTTCTGCAGTCTTCTCTGTCCAGTAGCTGTTTCCTCAAGTCTTTGGGGGCTT
TCAGCGACAGAACGGTGTGCCTGTTGAAAACCACCAATGGCTGATCTTTGAGTATCATCAACAGGTAAATCA
GCAAAATCCTTTGCACTTTTGGCATATTAGGAATGAACCAGGTCTCCAAATTGATCTACAACCTGTTTGACGA
GGAGTATGGATGGAC
>TCONS_00012302
TTGGAGACAGAATATCTTCAAAATCCAACAATAAGAAAAGGTGCCCAGAGAAAATAAACGGAAATTCTCAG
GAGACATAGGCATACCCATATTCATCAAAATATCCACCGCGGCCTTCAGTTCCAAAGTCCTCCCTAAAGACA
ATCTGTATATTATCAATGTTGTCACGATTCCAGAGCGGCAAAAAGAAGCGATTTGCAAAACGCAGCACCAAC
AAATTCTGAACCAACTCC
>TCONS_00012380
GCTTGGCGAACAAGAGAGGCCAGTAAGGCTCTACGGTAACATTTGCTGCTTTTACAATACTTGAAATCTTCTC
AGCAGTGATAGGGATGTCCTCATCGTTGAGGATCAAACATGCGTAGGTACAAGCGATTTCTCCGATCGACAT
TTTCTACTCAATCGATTTACCTTGAGGCAAGAAAATGCTGGTCTGCTCTTTCCGCTGCTGCAACTGATTGCTAG
>TCONS_00012383
CTCAGCCACGCATACAGACTGGACGAGTATCTGGTGCATCTGTAGAGACAGGACAACCATGTAACCCGAGTC
AAGGACCTTTACAAACCGGACGAGTATCTAGTAACTCTCTCCCAAGACCTTCTTTAGAAACACAATCATCAC
GAAATGAACCAGAAACTGTTCATCGCTCTCCAGAGGATGACAATTGTGCAAGATAAATTTGAAGTTTACAGC
GGGCTGCAAGAGCATAGGTTTCAAGCCATCGTAATTAGTACAATGCAAAGGCTTTTTAGAGCACGGATAGCT
CGACTCAGCAGGCTGTACTCTAAGTACAATACTAATGAAGAAAGATTGTCTCATCGACCTACAGATGTTGAG
CTTGAGGACTGGAAATACCTAATACAATATTTTGGAAGTCAGGATTTTAAGGTACTAACTTAATACATAAA
>TCONS_00012396
TGCCTTCTAATCCCAGGTACTAATTTTGAATGGTACTCATGATCGAGAAACTGGTGGCTTTGGTGCTTCTTGCT
TTGTGACTGCCATTACTGATGCCTTAAATCGAACTTATGGAAATCCTCACAATTGTCTTAAAAATCCTCCAAA

CAAATATATCAACACCCTTCTGGTGCCAAAAAATGGCCAAATACATGTGGATATAGAATGCCTGGCTTCTCA
GGGAATTTTCAATGTGGT
>TCONS_00012446
CGTCAAACAGCAGGACAAATAAGAGTCTAAAGATAAAGAGGTAGTATCTGTACTCACCCGTTATCATCCATA
ACCAAAACTTTTAGTCCTTGAAAAGTCATCTGCATGTGATTTGCAGGCAATCTGGGCATAAAGGGAAGCTTA
GGCTCATTTGAGCGTCCAGGAATCCCAAGTTTAACAATAAAGATAGCAGTAGACCCCTTGCCAAGACCTTCA
CTTTCGATCCAAATATGACCTTCCATAAGATTAACAAACCTCTTAGAAATTGCAAGGCCAAGC
>TCONS_00012560
GATAAACTGGAGCAGGCTTTTCATAAGCTCAGTAGATTCAGCTGAGACTAGAATCTCCCTCTTGAAGCTCTTC
TCTGCATCATAATACCCTGAAACAATCAAGAGGATGCGAAGTTTCTCTAGTTGCTGGAAACTTCTTATGTTTTC
TGCTGAAGTAGAGTGATGTGATAAGAGCTCCCCTATAGGTATTCCGACTGAACTCATTCCAATATTTGAAGTC
CCCATTCTTGATCCTGTACCATAAGGTTTCCCCGATTTTGACCATCTTCTAATATCTTTCCGTAAAACTTCTCCT
GCTCTGGGGTCAGCTTCATCCAATTTTGACAAATTTATATTATCTTTCTGTAAAACTTCTCCGGCTCTGGGCTCA
GCTGCATCTGACTTTGACAACTTTTTAATATCTGTTTGCAAAACTTTTGCTCTGGGGTCAGCTGCATCCGGTTTT
GACAATCTTTTAATATCTTTTTGTAAAACTTCTCCTGCTTTGGGTCAGTTGCATCATGCAATTTGTATCTAAAAA
TTTCATAAAGACCGTTACATCCAAATCGACCTGCT
>TCONS_00012599
GCTGATATAGGACGAAGCTTTTCGTAGAATTGCAGCATCACAACATAAACATGTTTACTTTCCACAATTGAAC
CCCTTGCAAACTGAGAGGACAATGATGAGGATCAAAGCAATCAAGATACCTAACACTATCAGCTTGATCTTC
ATGTTTTGCAGCCACATTTTCCTCCGGATTTGGGTTCCAGTATTCCTGAAGTCTTGTGCCTGGTGATGAAGGTT
CTCCGTCTTATCCACAAGAAGCTCTATCTTTTCTCCACGATCAAGAACCTTCTCAATGTTTTCCATCATAACAC
CTTTAACTTCCGAAACCTGGGCTTTCACCTTCGCGATTTTGCTAATTTCATCAGGATGGTCAGCACAATACTGC
ATATGCTCCTTCAACTTAGGTCCGAATTCCTTGTTCAAGCTGTTTGGAGGAGCTGTCGCAGCCTTCCCACCCCC
ATATTTCGACACAAAATCATCCTTAACACGCTCCAAAAATGCTATTGGAACTTGTCTTCCAACAGACTCCTCA
GCAACCACACAGTATGTGAAGCCATTATCGACAAGGTAATTGAAGGTGTGACCATCACAGTTGTAAGTGAAC
TTGTTATTAGAAGCAGGGAGCTTCTGAAGGCACTGATAAGCTATGGAGTTGAAGTTACCACTGAAATCTGTGT
ATTCAGCCAATATCACATTCCCACGACCCACAAACGCATAGATCAAAGCCTTCTGCTGACCCATTTGATTTCT
TGCTGGATCG
>TCONS_00012702
GGTAAAGAAAGGTTTAGACTCAAAAAATGTGACGTCGGAGGACATAAAGTACCTACGAAGATCAGGTGAGT
AACAACGATATCCCTTCTGAACACGAGAATAACCAAGGAAGACACACTTGAGAGCACGAGGAGCTAACTTA
TCTTTCCCAGGGGCTAAGTTATGAATGAAACAAGTGCTCCCAGAAACACGAGGGGGAACAGAGTATAAGGG
TGACTGGGGAAACAATACTGCATACGGAATCTGATTCTGAACAGGAGATGAAGGCATCCGATTAACCAAAT
AACAAGCTGTGAGAACTGCATCGCCCCAAAAACGCAATGGAACATGAGATTCAATGAGAAGTGTGCGAGCA
GTCTCAATGATGTGCCTATTCTTTCTCTCTGCAACCCCATTTTGCTGAGGGGTATAAGGACAAGAGGTCTGATG
AATAATTCCTTGAGAAGTCATAAACTGCTGAAATTGAGAGGATAAATATTCTAAGGCATTATCACTGCGAAA
AGTGCGAATGGAAACACCAAATTGATTTTTAATTTCAGCACAAAAATTCTGGAATATAGAAAACAACTTAGA
ACGATCTTTCATTAAGAAAATCCAAGTACATCTTGAATGATCATCAATGAAACTAACAAAATAACGAAATCC
CAAGGTTGAATTGACTTTACTAGGACCCCATATATCAGAATGAACTAAAGAAAAAACAGACTCTGCATGACT
CTCAATACTACGAGGAAAGGAGGCTCGGATATGTTTCCCGAGCTGACATGACTCACATTCTAATGTAGATAA
ACTAGACAAACTAGGCACCATCTTCTGAAG
>TCONS_00012703

TCTGATTCAAGTCCTGTTCCAATCGTCTGTCCCGTACTGCGGTCCTGCATAATAAAAGAATCATCAATAAAAT
ATATACCACAATGGAGGGCATGAGTCAAACGACTAACAGATGCAAGATTAAAACGACAGTCAGGGACATAA
AGAACGGAATCTAGAGTGACAGAGGGTAGGGGATTCGCTTGTCCAACACCTTTTGCTTTAGTTTGAGACCCAT
TGGCTAAAGTAACAGTGGGAAGAGACTGTGAATACGCAATATTTGACAAAAGTGATTTATTACCAGAGATAT
GATCAGAAGCGGCTGAGTCCACAACCCATTGTCCAAGAGTACTAGACTGGGAAACACAAGCAAAAGAATTA
CCAACAACAGAAGTATCAGTCTGAGCAACAGAGGCTACTTGTGGAGATGTCTGCTTACTTGCTCGATATTTAA
GGAACTCATTATATCCCCTTCAGATAAAGAAAATCCCTGGTTACCTGTAGTCTCGGTCTGAGCAACATAAGCA
TTTTTGGGTGGGCGACCATGTAAAGAATAGCACACGTCACGAGTGTGTCCAAGTTTATGACAATAAGAGCAC
TTGGGTCTAGATCTTCCAAAACGACCTCCTCCTCGTCTATTCTCCATAGTTTGAGATGCCCGATTGTCCACAGA
CTGGGATACGAGGACAGATGAGTCAAGTGTCTGTGATGAGCTCCCTGGGTGACTTGGTGCTGCAGCAAGGCG
AAGTAATCGAGAGAATAATTCATCAACTGTGGGGACAGTCGGACTAGCCAAAATCTGATCACGTACTGAATC
AAGGTCATTAGGGAGTCCAGCGAGTGTAAGAACTAGAAACATCTTCTATCGTTGCTCTTGTTGCTTTTCAATA
CTAGCAGAAACTAGCATCAAC
>TCONS_00012704
CTGATTCAAGTCCTGTTCCAATCGTCTGTCCCGTACTGCGGTCCTGCATAATAAAAGAATCATCAATAAAATA
TATACCACAATGGAGGGCATGAGTCAAACGACTAACAGATGCAAGATTAAAACGACAGTCAGGGACATAAA
GAACGGAATCTAGAGTGACAGAGGGTAGGGGATTCGCTTGTCCAACACCTTTTGCTTTAGTTTGAGACCCATT
GGCTAAAGTAACAGTGGGAAGAGACTGTGAATACGCAATATTTGACAAAAGTGATTTATTACCAGAGATATG
ATCAGAAGCGGCTGAGTCCACAACCCATTGTCCAAGAGTACTAGACTGGGAAACACAAGCAAAAGAATTAC
CAACAACAGAAGTATCAGTCTGAGCAACAGAGGCTACTTGTGGAGATGTCTGCTTACTTGCTCGATATTTAAG
GAACTCATTATACTCCCCTTCAGATAAAGAAAATCCCTGATTACCTGTAGTCTCGGTCTGAGCAACATAAGCA
TTTTTGGGTGGGCGACCATGTAAAGAATAGCACACGTCACGAGTGTGTCCAAGTTTATGACAATAAGAGCAC
TTGGGTCTAGATCTTCCAAAACGACCTCCTCCTCGTCTATTCTCCATAGTTTGAGATGCCCGATTGTCCACAGA
CTGGGATACGAGGACAGATGAGTCAAGTGTCTGTGATGAGCTCCCTGGGTGACTTGGTGCTGCAGCAAGGCG
AAGTAATCGAGAGAATAATTCATCAACTGTGGGGACAGTCGGACTAGCCAAAATCTGATCACGTACTGAATC
AAGGTCATTAGGGAGTCCAGCGAGTGTAAGAACTAGAAACATCTTCTATCGTTGCTCTTGTTGCTTTTCAATA
CTAGCAGAAACTAGCATCAAC
>TCONS_00012731
CTTCAAACGGAAATACCAGAACACAGCCAGTTTATACATGACCAGTTAGTAAATGCACCCATAAGAAGCCTG
ACGCAGGCGATTTGTTGTTCTTCCAAGACATATTCCAGGAAGAGGGCCTCCTTAAGTAAATACTGTCTGATTA
GTTCCATCAAAACTGAAATATGTACTAGTAGCTTAATTCATTCTGGCTTCTTAAGGTTCAGGCAGTTATCCGTT
GCTTGGTTAAACAACTCCTGAACCTGCTTTAGCTCCTTTTCTGCAGCTTCCAGTTGCTTTTGAAGTTCTTGGCCA
ACTGCATCGTTCTCAGCCTGGAGTTCTGCAATTCTTTTCAATTGGGCTTCTTCACCCCCATCTGATAAAGGAAG
AGCAGCAACCAATGCATCAAACTGCTTGGCAGCCTTAACAAAAGCAGCACTCATTTGCTTTGGTTGCTCTGCA
ACATTGGCTGCATCTTCTGTAGGATTAGCAGGAGGAGGTTCCGGATAATTAGGTGACAGACGAACAGGAGGG
GCATCCCTCTGAAGAGTACCAAAGGTGTTGAAAGCAAGAGCTGCGATTGTATTTACTTGCTCCTGTAGCTGTG
ATATTATATCCATCTTGCAGATTTTTCC
>TCONS_00012765
GTAGAACAAGAGTTTTGGTGAGATGCGAACAATAAATAGGGTAAGAAGCGTAAGCGCAGATTCCTTGAGTA
TGATCCTTATGGGCATGTGTTAAGAAATGGTACCTCTTTGTGCTTGACTTTGGACTCCATGTGTCCACTGAAAA
TGGTAGTCCTTTTAGCATTTCTATTGGCATTTTTCCTCTCTCTACACAGTGGAATAATCCAAAAGGAGAGAGGT
CGG

>TCONS_00012789
GTTTTTATAGTTTACACAGTTTTGTATTGCCGCTTCTGACTGCCGTATTTATGTTAATGCACTTTCCAATGATAC
GTAAACAAGGTATTTCTGGGCCTTTATAGAGAAAAGAAAAATAGATCCTAAATATTTGTAATCAATCATTTAT
CACTTGGTGGAGGAATATATAGTATTTCATTGCTACAAGTATGGATTATTGAAAATAATAAGACATGGATTTG
GATATTTCCCTTTAACTATTCATGTCAACTAAACGGGGGATTGAAGGGAATTTTGTGAAGAGAAAATGGATT
ATGGGAGAATATATAGTATTTCATTGCTACAAGTATGGATTATTGAAAATAATAAGACATGGATTTGGATATT
TCCCTTTAACTATTCATGTCAATTAAACGGGAGGATTGAAGGGAATTTTGCGAAGAGAAAATGGATTATGGG
AGTGTGCGAC
>TCONS_00012790
CTGCCGTATTTATGTTAATGCACTTTCCAATGATACGTAAACAAGGTATTTCTGGGCCTTTATAGAGAAAAGA
AAAATAGATCCTAAATATTTGTAATCAATCATTTATCACTTGGTGGAGGAATATATAGTATTTCATTGCTACA
AGTATGGATTATTGAAAATAATAAGACATGGATTTGGATATTTCCCTTTAACTATTCATGTCAACTAAACGGG
GGGATTGAAGGGAATTTTGTGAAGAGAAAATGGATTATGGGAGTGTGAATATATAGTATTTCATTGCTACAA
GTATGGATTATTGAAAATAATAAGACATGGATTTGGATATTTCCCTTTAACTATTCATGTCAATTAAACGGGA
GGATTGAAGGGAATTTTGCGAAGAGAAAATGGATTATGGGAGTGTGCGAC
>TCONS_00012791
CTGCCGTATTTATGTTAATGCACTTTCCAATGATACGTAAACAAGGTATTTCTGGGCCTTTATAGAGAAAAGA
AAAATAGATCCTAAATATTTGTAATCAATCATTTATCACTTGGTGGAGGAATATATAGTATTTCATTGCTACA
AGTATGGATTATTGAAAATAATAAGACATGGATTTGGATATTTCCCTTTAACTATTCATGTCAACTAAACGGG
GGGATTGAAGGGAATTTTGTGAAGAGAAAATGGATTATGGGAGTGTTATTTCATTGCTACAAGTATGGATTAT
TGAAAATAATAAGACATGGATTTGGATATTTCCCTTTAACTATTCATGTCAATTAAACGGGAGGATTGAAGGG
AATTTTGCGAAGAGAAAATGGATTATGGGAGTGTGCGAC
>TCONS_00012841
TCCAACTCGAGGAAATTGCATTCACCCGCACTTGCTGCAAATGCAATCTTCCGGAATAGTAACCAACTGTAGT
GCTCATTGAGGAATTTTGGCCAATGTATTCTTGCTTCTGTAACTCCCATCTTGCGAGCGACTAGTTCATTTCTCG
TTGTTACAATAACACTGCTCCCATTTCCTTTGGGCAGTCCAGCATAGATTTTTTGCCACCAATCATTGTCCAAG
CTCCAAACATCATCCATAACAATCAAAAACCTCTTTCCCAAAAGGTACTGGTTTATTTTTCGTAACAGTTCAC
ACTGGTCATCACCAACGCATGCATCTCCTAAACTCCTCAATATGCTTCTCATGACTTGTTCCTCAGTAAATGTT
TGAGAAACAGACACCCAAATCCTCCTCTCAAAGTGATCCTCCACCCTTCTGTCATTGAAGACTTTCTGAGCAA
GAGTGGTTTTGCCAAGTCCTCCCATACCCACAAATGCAATGGCAAGTAAACCATCTCTTGCTTCAAATAGCCA
ATCCTTTATCTTCTCCGTGTCACCTTCCAGACCAACTACCTGAGTGTGGTCATAAAGGGAAGAAGTCCATCTT
GACATTAGATTATTGTGTGCCTCCATACTTCCTTCTTTTAGAAGTGGTACTCCAAGATATGTTGAAATGTTTTGC
TTTATCTCCGAGATTCTGTCATTGATTTCAGCAAGGCGCTTCCCAGTTTGATGGCGAAGAGATAGCCTTTTGGG
ATGGAAGCATGTAGTAGCCCGATCACTATCTGCAGATTGATTCTGGCAGTCCTCAAGTATCTCTTCAGCTTCA
AAGATTAAGTCTCGCAAACAGGACATGGCCCCTTTAAGGGTGTTGTTTTTCCTCTTGAGCCTCTCTGCATCTTT
GAGAAAGCTTTGCATGAATAGTAGCTCGTTCTTCAGTTTTTCAAACTGCTGTCTGAATTTAGTAAGAAACCTA
CTCTCCTCTGTGAGAACATTCAGAAGTTTCTCCAAGAATACAGTGACCACCGCATCCACCATCTCTTCTGCTA
CAAATTTCTGAAATGCAGCTTAATGAAAATGGATCACCTACACATGATACAAATATAACACAGCTA
>TCONS_00012933
AATAAAAGCACCAACTTCTAGTGCTCTCTTGCCAATGACCCCAATGACATTCAATATCACCTGCCAATGGACA
CAATTATCCATATTTACAAATCTATTCACATATCTACAGTGATATTTAAGCTGGATAGATGTACACCCTAGCTG
TTGCAATCCAGATCCATCACACACGGACAGTTCGAGGATTCAATGCCTTCAATGCTGCTTCAGCAAAGCACTG

TGCCGCTTCTGCCTCTGCTTCAGCCTTCTCCGCATCTCTAGCTGCCCTTTCTGCCTCAGCAGTAGCAGCTTCTGC
CTCTGCAACTGCTTGTGTAGCAGCTGCGGCAGCCTCTTCCGCGCTCATGCTCATCATTTGAGCTAACTCTGCAT
CAATTTGTGCTTTAGTAAGTATTCTAACTCCATTGTTTTCAAGCTTTGGGGAATCCTTCTGCTTCTCCTCCAGAA
GCAATAGGAAATCGTTGCCCCTGGCATCAGAT
>TCONS_00012934
CCATAGCATGTAGATTTCTCCATTTATCCTTAATGTCCACATTTGAACGAGAACGCAACACACCAGAAAACTC
TGGATCTTTGAGTATGGTGCTCCACTTGCCAACCCCATACTTAGCAACACCCAACTTAAGGGCAGCTTCTTCTT
CTGATGTCCACTTCTGCTTTGGTGCACCCATAATAGGTTGATGCCTTTTTGACACTGACGCAGAATTGAGCTTC
CCTACCTATAGAGCCATCTCACTTTTCAATAAACAAATAACTGAATGTGGCAAAAGAAAAGTAAATACCCAA
GAATAAATGATCAATAAGAACATAAGCAGGGCAAACTATAATCTGAAATTATATGACAGAAGATATTCATTT
GTTAACT
>TCONS_00013083
ACCAGTTTTGCTTCCATTTTCTACAACAGTAGAACCATGGCGGCCATCCCTTGGGCTCTCTAGACTAAAAAAG
GTGTCAAGCGAGGGTCTGTGTAATGCGGAAGACCAGCTCTAGCTCCCAAGGCTCTGCATTTAATAGCTGCCA
CTTGAGCAGCAAATGGCAACATTTTCTCTGGTGGCATGTTGGCACAGATAGAATAAAGAACTGCACCAATGA
ATGCATCTCCAGCACCAGTTGTATCAACTAGTTCTGTCTGAGGTATACTTTCAGCTGTCCCCACAAGTAACTTA
CCGCTGATTGTCCCTATCCCTTTTGCGTGTAGCTTTGCCACATTCGAGGATATGCATGCTGGCGATGTTGCATT
AGTGTCTTTTTTCTGTTTCAGCTTTTCGAACAAATCGTCGACATCCCTTTCTTCCGGGTCGAGATTGTCTATTTCT
GTCCTTTGTAGCATTAAGCAACCATCTTCACCCAATGTCACAATAACAAATTTAACTTTTGGCAATCTTAAGA
GCATGGAAAGAAGGGCGCTTGGAATAGAAGAAGCCTCAGTCCAGTTCTGAGGAAATTTGGTTGAGCAAACA
ACATAGCTTGCGAAGCTCAAAAGGTGATCTAATCCTTCTCTTTTCCTCTCAGCATCAATTAAAATGGGTATAC
CCCTTCTATTTGCCTCCTCTGCTACAACAGCAGCAGTTTCATGCAATCTCCCGTCGAAATAGACAAGCTTAGC
ACCATCCAATGATGACAACAAATTTGATTGGGAAAGATCAGTAGGTATCATGGGTGGATATCCAGGGGTGTG
AATGCAAGTGCGAGTCTTCGTTTGGTTGTCAACAATGATGTAGGTAAATGGTGAATTGCCTCCATCAGACACC
ACGATAAAAGACGAATCTACACCATCAGCTTCTAGCTCCTCCAGTATCCCCTTTCCTTGAGAGTCATTAGAAA
TCTTTGAAATAATTCTAGGAGTTAGGCCAAGGCGAGCTGCACAAGTCAAAGAATTCCCAGCATTACCACCTC
CCTGAACCTGAAAGCTAGTGCTTCGAATCTTATCATCTGGATTTGGATAAGAAGCAACAGCAGCCAAGAAGT
CCACTCCAACCGACCCACAACCTAATATGATGCGATTTTCGGGGAGCACTATCGAAGCTTGAGTCGCCGGAA
TTGACATTTTCACTCTAGTTCTAGCCGGCGAAGGGAATGGAGACGTCAAAAGGGTCCTAGAATTTA
>TCONS_00013084
ACCAGTTTTGCTTCCATTTTCTACAACAGTAGAACCATGGCGGCCATCCCTTGGGCTCTCTAGACTAAAAAAG
GTGTCAAGCGAGGGTCTGTGTAATGCGGAAGACCAGCTCTAGCTCCCAAGGCTCTGCATTTAATAGCTGCCA
CTTGAGCAGCAAATGGCAACATTTTCTCTGGTGGCATGTTGGCACAGATAGAATAAAGAACTGCACCAATGA
ATGCATCTCCAGCACCAGTTGTATCAACTAGTTCTGTCTGAGGTATACTTTCAGCTGTCCCCACAAGTAACTTA
CCGCTGATTGTCCCTATCCCTTTTGCGTGTAGCTTTGCCACATTCGAGGATATGCATGCTGGCGATGTTGCATT
AGTGTCTTTTTTCTGTTTCAGCTTTTCGAACAAATCGTCGACATCCCTTTCTTCCGGGTCGAGATTGTCTATTTCT
GTCCTTTGTAGCATTAAGCAACCATCTTCACCCAATGTCACAATAACAAATTTAACTTTTGGCAATCTTAAGA
GCATGGAAAGAAGGGCGCTTGGAATAGAAGAAGCCTCAGTCCAGTTCTGAGGAAATTTGGTTGAGCAAACA
ACATAGCTTGCGAAGCTCAAAAGGTGATCTAATCCTTCTCTTTTCCTCTCAGCATCAATTAAAATGGGTATAC
CCCTTCTATTTGCCTCCTCTGCTACAACAGCAGCAGTTTCATGCAATCTCCCGTCGAAATAGACAAGCTTAGC
ACCATCCAATGATGACAACAAATTTGATTGGGAAAGATCAGTAGGTATCATGGGTGGATATCCAGGGGTGTG
AATGCAAGTGCGAGTCTTCGTTTGGTTGTCAACAATGATGTAGGTAAATGGTGAATTGCCTCCATCAGACACC

ACGATAAAAGACGAATCTACACCATCAGCTTCTAGCTCCTCCAGTATCCCCTTTCCTTGAGAGTCATTAGAAA
TCTTTGAAATAATTCTAGGAGTTAGGCCAAGGCGAGCTGCACAAGTCAAAGAATTCCCAGCATTACCACCTC
CCTGAACCTGAAAGCTAGTGCTTCGAATCTTATCATCTGGATTTGGATAAGAAGCAACAGCAGCCAAGAAGT
CCACTCCAACCGACCCACAACCTAATATGATGCGATTTTCGGGGAGCACTATCGAAGCTTGAGTCGCCGGAA
TTGACATTTTCACTCTGAAATTTCTAGTTCTAGCCGGCGAAGGGAATGGAGACGTCAAAAGGGTCCTAGAATT
TA
>TCONS_00013121
GGCGATCGGACAGAAAGCGATTCTATGGTCTACAACTACTTACAGCGATCACGATCTCTTTTAAGTTCCGTT
AGGCTAAGAACTGTATGGAATCCGAGCAGAGTTAGCCGGAGATTGAGAATTCGAGTGCAAGCTAAAAACGG
AGCCTCCGAAAATGCTGTCAGCGGGGGCAGCACCAATTTTAGTGATCCTAATTGGTAGTAAAAGTATCAGAG
AGAATTTGAGGCTCGTTTTAATATTCCTCATATTACCGATGTATTTCCTGACGCCGTTGAGTATCCTTCCACTTT
TTGTCTTAAAATGAGGACTCCAGTATCTGAGGAGTTTGCAGGAGGTTACCCTTCAGATGAAAAATGGCATGGT
>TCONS_00013176
AGAAAAAAATTATTTTCAAAAGCTCACCTAAGAGCAGTAAGGTCATGATCATATAAACCAAGCTGATTAAAT
TTTATTAACAAGAGGACAAATGGTACATATCAACATTCTGTACTTGGATCATCGGGCGTATTCACACCACAGA
ATCTAGTTTGACAACAACAATTACCGACTCCAAAGAATCACACATCACTGAAGCCTAAATCTTCCAATCAGT
TTGTGATTGTTATCACAAATGGTCCATCTGGTACAGTTCTTCAATCTAGAAATGCTTTGATACTAGCTCTAAAT
ATTACCATATTTATATAGCACCACCTTTGTACTCATCGTTCCAATGGAAGCAAACAAAAACCTTACTCCCTTC
AACTAGTGCACTTCACATACATAGGAATCTTAATCCTTCTGCTATTGCATTCCATAGATAATCCTAAGTCGTAG
TCGTCACACCAACAACAACATTGCTCACCAACTTGCCACTTTCATCTAAAGACATAGGGTTCTGAACGACCA
CGGTTTGATTCTGTGAACGGGCTGTAGCTGTTGAAGAAGTGGGTCCTGCAGAGGATGTAGCACCGCCATTTGG
GGTGTGCACGGGAACATGGCCTCTTCCATCTCCGGCATTAACGTGTGTGATATAGTGGCATATGGCACATTTA
ACTGATGGAGCTCCGCATGGATACATCAGCATAGTACGACAATTTCCACAGTTGACATGAGCAAACTGGTTA
GGACCTGGCACAAGGTTCACCGTATGACAGCAGGCGCATCTCACACTAGTTGCCCCACGTGGATGCATCAGT
AAGGTGCGACAGCCTCCACATATCAACTGAGCCATTTCCATTCCAGGAGGTGGGACAGGGGTGAGGGCGTTG
CACACTGCACAGCAGACATTAGTAGCTCCTCTCGGATAAAGAAGAATTGTCCTACACCCACTGCACACCAAC
TGACTCTGCATAGCAGTAAAAGAAATCAGAAAGCTAGGGCCAAACAGATTTGGTAATATATTCCACACAAAT
TTAAAGCAGAATTGGCGATAAGGGGATTCCCACTTTTCTGGAAAATGTGAATAAAAAAAGTTCTAAGAAAGC
GAATTTATAGATTATTCAGAGTTTGGATTTGGTAATCTTATCGGAATTTCGCTTTTTTATTCTTACTACATCTTCT
TCTCCGTTTAATATTGAAAAAAGAATGTTTTCTTTTTTTGGACTTTTATGAGTTTCCAACGGAGGAGAGAGAGT
AAATTCGGGTAGAATTGGAAAGTTCTTTTAATTTAATCCGAACGCGTAAATTTGATTTTTCTTTTCCTTTGTCCG
A
>TCONS_00013198
AAAGTAATATACGGCACGACCAAATGAAGCAAATCTCATCATTTTAAGTTAATCCACGTGTTAATCATTACTG
AACGTGACAAGAAAGGCTACATTCCGCAACAATACAAGCAACGGAGATTCACAATGCAAGTCCAAGAGAAT
TGAAAGAATTTCAATATGTTCAACTCCAGCGTCTATGAAGATAAAGAACACAAACTATGTTATTCCGTTGTGA
CTGGTTTTGTATGCAATATAACACAAGTTTGTCCAAAAGAGGCATTTAAACATGTCCTCAGAATAGCATGCTC
CTAACTCAAAAGCCTGAAGGAACCGGCACAGCAAACTTGATTAAAGTAACTCTTTCTGTGGCGTCCATTATTT
AGCTGAAGATGAAGCAGCAGAGAGTCTCGCACTCCTTGCCTTATCAGCTTTTTCCAGGTAATCTTGATGAGAT
TGTTGCTTGGAGCCTCGGGTCATGAAGTACCAGGGAACGCCAAAGACGACGCTGATGACGGCGATACCAGCT
ACGACGTGCTTCTTTTCGCCGCTATATAGGACATGCTCCAATTTTGCTCTGAACCCTCCTCCTCCATTTCCTCCA
CCATTCATCTTCTTCGTCTCACTTCCCGTATTCTTCTCCATTTGT

>TCONS_00013251
GATAAATTGATAAGTCGGGCATTCCCGGCCCACCAAGCGAAACTAGTGATTTCTTTGTCACGGCCAGCTAAG
GCTAAAGTTTCATTAAAGAGCGTTTCCACGTGGTAGAACCTCCTCAAGGAATATAAGGTTTTCATAAGGCTAA
TCTTGAGCCGCCATCCAAGCGCGAATACCTTCGTTTAAGAGAATATTTTTGGTGAAGAAAGTCTCAAATTCAG
GATCTTTCGCTGCGCGAATTTCCTGAGAAACGAAGTCATAGGCACGTAGGTTCAGGGCTAGACCGACTACTC
CAAGAGCAC
>TCONS_00013266
AAAGAATGTGATGTAAGAGTTTCCTTTGACCTGAGATCGTCATAGTAATTGACAAGTTATTTATTGTGCTTTGA
TACCAGACACCTATGGCCCTAGGGCGATAGTTGAAAGGATATTGGGTACGATTAAATGCTTGTAGAATTAGT
GATTGATCAAGACGGAATCTGTCAACTCTTGGTAATGAGTTTAAGCTCCATGTTGTCATGAATTATAGATGAC
CAAACTAAGACCTTGGCTAGGGCAATTGAATGAAAGAAGAAATGAGTTTCTTAGGTCATTCAATGGTCGATT
ATATTTGACATGAATACATAGTTGGTCGCCTATTAGGATTTGACAGTTGAACCATATCCTAGGGTGATCCAGA
GCTATAAGGACAGAAGGAATTACTACATTATTCTTCTACGGGTTCTTGAGAGTAGATTGTATACTTCATGCTAT
CCGGTCG
>TCONS_00013322
AATTCATGTGAGATAATTAAGGAACTCAGCAGCAGTTGCAACAGCACCTCCTGTAATGGCATCCATCACAAT
CTTGTCGCGATTGTTGTTGCTTGCAGCAGATATGAGAGCCCCAGTCAATGCACCCCCAATCATTGCATTCTTCC
AATCTCTGGTTCCACGGATCCTCTCTGCTCCATACTCCATTCCAGCATATACCCCAGCCACAGTCCCCCAATA
AGCACCTTCTTTACACATTTTCTTCAACGAATTCTCAAAGTTGTGACTCGACACACTGCCTTTTTTGACAATGT
GATAAGTTTCTTCAACAGCAGTTTTGGTGACAGCAACAGTGCCAATTTTCAAGAAAGCATCAACAGTTTGGTT
GAGAAGTGGGTTGCCCATATCAATAACCAAATCCACCTTTGGAGAAGCTAATGATGCTGAAAATCTGCTCCT
CGGCATTTTTTTCTTCTTCT
>TCONS_00013331
TGAGGAAGCTCGAGAGGGATTAGGCTCCAGGGTAAAACCCAAAGCATCAAGTTTGTCATCATGTGAAATTCC
AGCCTGCAATAAGGTCTTGTTGTCATCTCTAACCTTCTTACCCTGAAAAACTACACCAATGTGCAGCCCACCA
CCAAGTATTGCAGTCACTGCTTCCATTACGGTCCTCATGGGAATCACTAGATGTACG
>TCONS_00013388
CTTCAAAACTTTATATGGTCAGGCGTAGAAGAATTTCACATCGGTGATCGTCCATTACCAAGCATCTGGCCGA
TAAGGTTTGTCACCTGCCAATATTTTGATACACAACGATCAATGCAGCTATTTTCGCCAATATTTAGCTCAGGG
TCCTTGTACCTTCTATCGACACATTTGTTGAAGCATGTCTGAGTGAGCTTGTTAAATGATTCAACCCTGTACTC
CATCTCCTTCTCGGCCATGCCAAAAATCTGCTCCTTGTCAACTGTTGTTGGTAGATTATTAGCAGCCATCTTTA
CGAACCGATGAATGGTGTCTTCTGTGCTCTTTCGTCTTT
>TCONS_00013528
TTTTTCCTTTTTATCTCAAACCCTAATTTTGAGTAGAAGTGCTTTTTAATTCACACATTTGAAGCCAAAATCAAC
AATCAATCTTTAGAGTGGCATAGAGATTGTGTAGTATTTCAATTGCTAAAACTGACCATTCAGAAGAAACCA
GTAGAGAAGAGACCCGGCATTCTCTTGGGGAGTTATGCAGACAGGAAAGATAGATGTAGCTATTTGAGGTTT
ATTATCTGTTGCAGTCAACTCTTGGCAGGAATGGCATGGCGGCTTTACTATGTAACTACCAAAATGA
>TCONS_00013556
CAAAACTATCTGTTGTATTAAAAACACTTTTACACAGCTAGACAAAGTCTCGACTTTCTTACAGTGAGACAGT
AATGTTGACATCAGAAACTGAGCCAAGCATCTTTCTCTGCACATAAATCCAGTACATATCTCTGCTCACATTA
CTAATTACCTAGTGAGATAGTATACCACCAAAAACAGAACCACAAATGAGGCCACTAGTGTGAACATCCTTC
GGCTTGATTTTGTCTCGAATACCATCTTAAATTTATCCATTGTTCCAGCTAAAACACCTCTTGAAGAATCCATA

TCATTTCCCATTCTATCAAGCATCCGATTATGAGTCTCCACCTCCTCATGTATATCACCTGTCAATCTTTTGAGC
ATATTAACTCTATCTTGCAACCCATCTATTGCCCTCTCATTCTCTTGTTCATCAATTTCG
>TCONS_00013629
AGAATCTCAAAACAGCAACATATCAGAAGAAGCATCGATAGGTTGAATAGGGCAAGGCAGCTGCCATGGCT
CAAGCAAAGTGTCGGGAATTTCAGAGAGGTCCACAGGGAATCCAGTAGCGTCAGCCCAAAATTGAGTGAGC
GAGTTCAATGAATTCAAACGTTCAGTCAATTCAGCAATCTGAGCCCTCAACACATTGTTTCTGCATCCATCG
CACGATAATTTCTCTCGACAGAATCAATCCTCTCGCGGCAGATACTGTTCTGTTTATGTAGCTGTGTTGTTTCG
CTCATCAACTCCCCCAATCGCTGTTGCTTCCTCATCCGTGACCTACGAGCAGACTCACGGTTGGATTCCATTCT
CTTCCTTTTCCTTTCGTCAAATTTTGCATACTGCTGGTTGAGAAAGAGTGCAGAATACGAATACGACGCATCTG
GATAGCGTATGTCATTAATAATTTTG
>TCONS_00013706
CGTAAATGCGACCAACTACCTCCATGAACCACCAAGTCTGATAACCTCCTCAGTACTTGTATCCCTATTTGGA
GCTCTCTCGCCGCCTACGTTCTCCTTTTGATTGCCTAAGTCCCCATATAGATCTTTAAGCTTCGCAAGATTGCTA
GATGCAGCCTTCTTTTCCTTGCTTTCATCTCTAGGTGGAGTTCGATGTTGATCAAGACTGGTCCCTAGATCATG
CAAACTTCGGCTGTGGCTTCGACTCCTACTCCGGCTCCTGCTCCTCTCATAGTCTCTTCTGCTTCTCTCTCTTGA
ACTTCGATCATAATCATACCTCCTGTCTCTATCCCTTCCCCGGTCTCTGTCATAATCATACCTCCTGTCTCTATC
CCTTTCACGATCTCCCTCATAATCATACCTCTTATCTCTATCCCGATCCCTACCCCTCTCCCTTTCTCTTTCCTGG
TCCCGGTCCCTATATCGATCCCTATCCCTATCCCTATCCCTATCACGGTCTCTGTCCCTAGGTCTTTCCTTCTCC
CTCTCTCTGTCTCGGCCCCTGTCCCTTTCAGAATTTTCTCGGTCAGATATATCACGACTTTGACTCCGGCGGAT
GCTGGGGGACCGTCTTGAATCATCTGCACCATCCTTATCATAGGGTGGTGGTGCGATTGTACGTCGGATGGGA
GATGAATCTCTAGTTGATGCACGATGAGGCGCACGCTGACCAAAAGACACTGAAAGGAAGCTTTGACAGA
AGGTGGCCGACGGGAGGTTTCCTCAGATCCGCGACTAGAGTCCCCGATCGAACCAGAGGGTTTGGTCGGCAG
ATTCATTTTTTCGAGATTGGCAACTGCTGTCCGCATAACAGGAACAGGAATGCGGGGTAATAGTGTGTCAAA
ATAATACTGTCCGAGAAATAAGTCACGCACATATACGCCCATTGTCGTCATTTGGCCACTGGATCCAGGAGA
GAATTCCTCATCATCTTTGAGATAAGGCTCATACCAAGCCCATAATGTCTTAAAATCAGCAAGATATCTCAGA
TAAAGGAACCCAACAGCTCTAATGTAAGGAGAATCTGGATGCTTTAACAGGCCATGCATTTGCTTGACAGTA
AGTTTCATCGTAAAGAACTTGTAGAGAAGGCAGAAGGCTGTTGAAGGACCACGACAGTTGCCAGTCATCCAT
GGTTCCACATGGTCAACTTGATTATAAATTTCATCAATCACTTCGTGATAAGTTTTCAGGCGCAATAGGTCTCT
GAAATAATCAGAAGAAAGAATGTTCATGCACAGAACCTTCTCCAGCAATTGGTCGATCGGTCTCCCAGAAGT
TTTAATCTCAGCCATGCCCTTGGTGATGTAGGCACCAAAGTGTTATCTTTTCCTCCCTTTGCTTGGCCAACAAT
AAAAAATGATATTGGAAAGATTAAGGTTCAGATACCAAGTTACTTGCTGGAGTTTCTCTGGAGAATTGAGCA
GACAGAAGAAGTGGTGGAAGATTATCCCAGC
>TCONS_00013707
AAATGCGACCAACTACCTCCATGAACCACCAAGTCTGATAACCTCCTCAGTACTTGTATCCCTATTTGGAGCT
CTCTCGCCGCCTACGTTCTCCTTTTGATTGCCTAAGTCCCCATATAGATCTTTAAGCTTCGCAAGATTGCTAGA
TGCAGCCTTCTTTTCCTTGCTTTCATCTCTAGGTGGAGTTCGATGTTGATCAAGACTGGTCCCTAGATCATGCA
AACTTCGGCTGTGGCTTCGACTCCTACTCCGGCTCCTGCTCCTCTCATAGTCTCTTCTGCTTCTCTCTCTTGAAC
TTCGATCATAATCATACCTCCTGTCTCTATCCCTTCCCCGGTCTCTGTCATAATCATACCTCCTGTCTCTATCCC
TTTCACGATCTCCCTCATAATCATACCTCTTATCTCTATCCCGATCCCTACCCCTCTCCCTTTCTCTTTCCTGGTC
CCGGTCCCTATATCGATCCCTATCCCTATCCCTATCCCTATCACGGTCTCTGTCCCTAGGTCTTTCCTTCTCCCT
CTCTCTGTCTCGGCCCCTGTCCCTTTCAGAATTTTCTCGGTCAGATATATCACGACTTTGACTCCGGCGGATGC
TGGGGGACCGTCTTGAATCATCTGCACCATCCTTATCATAGGGTGGTGGTGCGATTGTACGTCGGATGGGAGA

TGAATCTCTAGTTGATGCACGATGAGGCGCACGCTGACCAAAAGACACTGAAAGGGAAGCTTTGACAGAAG
GTGGCCGACGGGAGGTTTCCTCAGATCCGCGACTAGAGTCCCCGATCGAACCAGAGGGTTTGGTCGGCAGAT
TCATTTTTTCGAGATTGGCAACTGCTGTCCGCATAACAGGAACAGGAATGCGGGGTAATAGTGTGTCAAAAT
AATACTGTCCGAGAAATAAGTCACGCACATATACGCCCATTGTCGTCATTTGGCCACTGGATCCAGGAGAGA
ATTCCTGCAAAAAAATTTATGTCCAGGCACCTTCAAGCAAGAGTGGTCCTTGCATCCTCATCATCTTTGAGAT
AAGGCTCATACCAAGCCCATAATGTCTTAAAATCAGCAAGATATCTCAGATAAAGGAACCCAACAGCTCTAA
TGTAAGGAGAATCTGGATGCTTTAACAGGCCATGCATTTGCTTGACAGTAAGTTTCATCGTAAAGAACTTGTA
GAGAAGGCAGAAGGCTGTTGAAGGACCACGACAGTTGCCAGTCATCCATGGTTCCACATGGTCAACTTGATT
ATAAATTTCATCAATCACTTCGTGATAAGTTTTCAGGCGCAATAGGTCTCTGAAATAATCAGAAGAAAGAAT
GTTCATGCACAGAACCTTCTCCAGCAATTGGTCGATCGGTCTCCCAGAAGTTTTAATCTCAGCCATGCCCTTG
GTGATGTAGGCACCAAAGTGTTATCTTTTCCTCCCTTTGCTTGGCCAACAATAAAAAATGATATTGGAAAGAT
TAAGGTTCAGATACCAAGTTACCTGTCACAGAAAATAGTGAATAAGTTACATAACATAGTGAACTAAAACTT
GCAAAAACGTACACTAATATAATCACATTTCAGCTAATACAACCATCCCATGTTTGAAAAAGTAAATGATCA
TTACT
>TCONS_00013708
GCGACCAACTACCTCCATGAACCACCAAGTCTGATAACCTCCTCAGTACTTGTATCCCTATTTGGAGCTCTCTC
GCCGCCTACGTTCTCCTTTTGATTGCCTAAGTCCCCATATAGATCTTTAAGCTTCGCAAGATTGCTAGATGCAG
CCTTCTTTTCCTTGCTTTCATCTCTAGGTGGAGTTCGATGTTGATCAAGACTGGTCCCTAGATCATGCAAACTTC
GGCTGTGGCTTCGACTCCTACTCCGGCTCCTGCTCCTCTCATAGTCTCTTCTGCTTCTCTCTCTTGAACTTCGAT
CATAATCATACCTCCTGTCTCTATCCCTTTCACGATCTCCCTCATAATCATACCTCTTATCTCTATCCCGATCCC
TACCCCTCTCCCTTTCTCTTTCCTGGTCCCGGTCCCTATATCGATCCCTATCCCTATCCCTATCCCTATCACGGT
CTCTGTCCCTAGGTCTTTCCTTCTCCCTCTCTCTGTCTCGGCCCCTGTCCCTTTCAGAATTTTCTCGGTCAGATAT
ATCACGACTTTGACTCCGGCGGATGCTGGGGGACCGTCTTGAATCATCTGCACCATCCTTATCATAGGGTGGT
GGTGCGATTGTACGTCGGATGGGAGATGAATCTCTAGTTGATGCACGATGAGGCGCACGCTGACCAAAAGAC
ACTGAAAGGGAAGCTTTGACAGAAGGTGGCCGACGGGAGGTTTCCTCAGATCCGCGACTAGAGTCCCCGATC
GAACCAGAGGGTTTGGTCGGCAGATTCATTTTTTCGAGATTGGCAACTGCTGTCCGCATAACAGGAACAGGA
ATGCGGGGTAATAGTGTGTCAAAATAATACTTACCTGTCCGAGAAATAAGTCACGCACATATACGCCCATTG
TCGTCATTTGGCCACTGGATCCAGGAGAGAATTCCTGAAATAAATAGTTAATAATCAGAATGAGCCCTAAAA
ATCAACTCCCATCATGAAGATAAACATCATAGGTGAACAAACTAAAGACATAAT
>TCONS_00013751
GCAGCAGTATAGATCTTTTCCTCAAACCTCACTGCAATTTTCTTGAGCTCCTGAACTCCTCCTTCTCCACTGAC
AGGAAGATGCCTCTTTAAGGTCTCGGTTATGTTGTTGATAATCCTTTGACGAGAATCGGGTAGAAGTTGAGTG
CGCCAATCAGCAGAGTCCATAGCTCAATTCTGATCTAAGCGATTATTTCTTCCAATCGAAAATCAGTTTTACT
GCTTCTGATCAAAGAAAAAGAACAAAATGATGTTACGAGATATTCGGGAGAATATAGAATCCGAGATAGAT
TACAAC
>TCONS_00013789
CCTCCATCCAAAGATATGGCTCCATCTCTAATACAAGGGAGAGCCTGTAACATATCTGCTCTTGTTCTATATA
CATGCAAGCGTGAAAACAGGTTATAAAAGAGAGTCTCTCTTAGACCGTAACCACTGCTTGTAGCACAATATA
AGTTCACACTGTCTATATTGACCATATTCATAGCAAAGCCAAGAAACCCAGGTGGAGATTCCCCATTGGGTA
ATCTTGGCTTCAAAATATCAAGCCTCCTTTGAGGGTCGTCAGCTATGAACTCACCGCCATATGGTCATAAGTTT
TCAAGACTGATAACAAGAAATCGGCCATCCAAAGACCTCCCAATAGAAGATCCAAGTCCATGAAGACCAGA
AGTCTTGTTTATATAGCCTTCCTTATCGTATGTTTC

>TCONS_00013844
GTTATTTCCAGGTGCTTTTTGTTCAGATATTCAGCTTCCGAAAAAAGGATTTTGATGAATGTTCTTTTCATCGGC
CTGGTCATGTAAGTACAGCCAGCCTTTTATACATTTACACTAAAATTCTGTACTTTACGCCAATAACCCAAAC
TGAGCACCGAGCATTCTCTATCAACACGCTGGCTCCTGTTTCAACTTGATTCCACTTCTGCAGCCAAACATCAT
GGGCTGAGCTGGACTGAGCAAAAGACTACTAGCAAGCTCCTCATTCAAGAAGACAGGAAGTGGTGGCATTT
GGCTCATAATCCCTGGCATATTTCCCATGTCATTTAAAATCGCACTTAAATTGTTCTTCGTCTGGAAAAAGAGA
TCAACGTTGTCCTGCAACTTGAATGTTGAGAGGTTAGCTGAAATTTTATTGAGGACCTGATTATTTTCTTCCAA
TAGACGCCTCGTTCCAAGTAGTGCTGCTAAAACCAAAAAACCATTTATACAATTAGAGCCAATGCCGCACTA
AACAATGCAAGCTGCAAAACCAAAAGCACAGAAGGAAATAGAGAATAGCACTGTAGATAATCTTTGTTTTT
GATAAATTAGCAGAGAAAAAGATTAATTGGTAGTTTTAGCAAGAGTTCTACCAAATCACAATAAATGACATT
TTGTAGCGC
>TCONS_00013879
GAAAAAGATAACAGACAGGAGTAAAAAAAAAATGGATTGAACGTGCTCATCTTAGAAAACATCCGATCAA
GAAAAGAAATTTTCAACATCCTACTGTTGATGGCCAGAACAGAGTGCCAGCCACCAAAACAACAAAATGAA
AGATTAAACAACTGTTGCTGGGAATTTAAATATCAAAGTCTAAACATGTGTTGTCACGGACTACGAAAAGGT
ACCAAGTAAACCTCTGCAAACGGCCCCAGGGATATACAAGTAAACAAACAAGATGCATTGGCGGTCTGTGG
TGATACCCTTTATGTAATTCTACCTGATTGTCACAAACTTGTTGGTACCATGTTTAGCCCAGTGAGTCCTCAGA
TCAATTGGATTAGCAAAACTGTATCCTTCCGCAGCAAGTTTCTCCAAAACCTTCTTGAATGCACCCTGGCTCAT
CTTTCTTCCAGCAATCCTTGCTCCGCGTCTTTTAGTACTCATTGGCAGCGGATACATTGATATACTAGTGGTAC
AACAAGCTGACTGCCAGCCCCCACATCCCCATCGATAACATTGCTGAGGAGCACCAGTGCAGGAGCAAACT
GGTATAGGAATGCCAGAAATGTCCATATCAATTCCATTTATTACAATGTCCATGCTCTTCTTTGCTGGTTTTGCT
CGTTGAACACGGGGAGTCCCATTTTCCTTAGGTCCGGACGAGCCTTTCTTTAACTTCTTCGCTTTAGGAGCTTT
AGGAGTGGCACCACCTGTCTTCCTCTTTGAAGGACCACTTTCGTTCTTCACACTTGGTTCCTCTGCATTCACCCT
CACATCCTCAGTTACATCAGGTTGCTGTACCATTTGCATGGAGTGACTCGCAGAAGTTTCAGGGAGAACTGTC
GTGTAAGGGTTCCCCGGAAACATATGGAGAAATTTGTCTCTGTGATTTATCCAACTGTCCCTTACATAATCTAT
GTGAGATAAAGGTGCTTCTGGAACGATAGAATCCCGAGAATGGTACATCCCGTTAGCGCCGAGCATAATAGG
ATTCTCACGCCTAGTTAGGAAAGGTTTCGTATCTCGATCAACCACCGATGACATCAGCTGAAGACCTAGATGC
CCTTTAAGGGAAGGTTCATAATAACCCCAGTTCCTCATGTTCAAACCGTCGTCATCCATCAATAAATTGTTGG
TAAACAGTTCTTAGGTTTTTTTTCCTTCAGGAGAGAGAGAG
>TCONS_00013910
CTGTCTTAAAGTCAAGTTGGGAGGTGCATCTTTCTCACTTGGTCCACTCGCTGAACCATCTAATAGTACTGATC
CGTCACAACCCTGGACAAAACAGTCATGGAAGTGAAGGCGAAGAAGGCCGGCAGCCTGGCCAATATCCTGC
CAGAAAACCTGCCGGAGGCGGCTTCTGATGATGGATTCAGCATTGGGGCAAATGGAGTCATAGAAAGTCCAT
GAAAGACCCTTCACAATTGGTTGCGTACCTTGAGCTTCTGTTTGATGAATGTGGACTGAAAGTAAAGCAGAA
ATGATCAGCACAAG
>TCONS_00013971
TACCGTACACATGGGACGGCACATATCTCAGCAACCCATGACAGCACAATTATACACAAAGCTACCAGATC
GCTTTCGCCCAAGCAAGTTCCACTGAGGCGGTCCTTCACTTGGAACCCACGAATTAACTTCGATAATCCCTTC
ACCCATGGCTCTAGGCCCTCCAATTACAATTAGCCTATCACCACAAGCTCTAAAAGCCAAACCCCAACCATT
CATAGAACCTGCTCTTTCAGGCAATCTTCCTATGGTAACCCATGCTTTATTTTTCTTGTCATACTTCCTCACTTC
CATGTCAGCATAATCAGCAGCATACAATTCATTATTTACAACTGCCACCAAAGGAGGTGCTTCAGATGTAGC
AGGCATATTATCATCCCTAGCTTCACCAGCTCGGACAGGAGACATGCTCGGGATTTCTGTCCATTTTCCTGTTT

CAAGATCATACTCCTCTCCACAGGTCAATAGCTTTGACTCAGTTCCTCCGATGCCTCCGATTACGTAAAATTTT
CTGTCCATGAATACTGCAGAACACATCTTACGCGGCTTGTTCATGCTCGGTAGAGTCTTCCACCTTCCAGTCTC
TGAATTGTAAAGTTCTGCTGAGCTAAGGATATTGCCCTGTGAATCACAACCACCAGTTAGAATGGCAATCTCC
CCAAGACTGGCAGATCCAAACAAACACCTCGGTGCGTTCATCTGCATTCCGGATGACCAAGTGTTTGTCAGT
ATACTGTAACGATAAATGACATGTGACAAGATCTCCTTTCCAAACACAAGAAGCTCTGTGCCAACTGCCAAA
GACTCCTTGTCAGAGAACACAAAGCATTCATTGGGATTCATTGATGGTAGATGCATCCAACGGCGGCGACTA
GGGTCAAAAGCTTCCCATTCCAGTAGCTGGCAAGAAAAATAAACCCAATGCTCAACCACACCATATTGCCGC
CGCAATCTATAAAGCTCCCCACTCCTCACTAATGAGTGAAAGCTACTATTCAAAGATGCAATAGCTCCATAAT
CAGACCTGGAGCAATGAATGAGACAGCTAATGGAGTTGTCACGGCCAATGGCAGGAATAAGAGAACTTGAA
TCAGAATTATCTGCAGCATGACGCTGATTACTAGCTTCGTCAGCTGGTGATACTGAAGCATCACCTAAGGATA
TAACCACTTCTTCATTGAGAGCAACAGATCGCTTTGGCAACTTGCGTTGAACAACTTCGTTCTCGTGATTATTT
TCCAATGGCCTTTTACCGTGCTGAACTTCAATCCTATCAAGGCTGTAATTCATGCAAGCTGCCCAGTTGCTTTC
TCTAGGAAAATCCCTCGGTACCAAACAAGACCTGTCTTCCAACATGTCGTCCAGGTTCAGCCCTGAAAAGAA
GCACCAGTACCACAGCCTAACTTTGATCTGAATTCTCAACCTTATGCTCTTCCTCACCAAACTGATATCTACTG
ATTTGGATTCATGTGAGAAGAACAATCTGTTCTCTCTCTCTTTCTCTATGTATACATCTTCAGGTTAAAATTCTA
CTCTCTGTGTGTGTATGTGTGTGTGT
>TCONS_00014044
GACAGTTACAGGAAATCTATAGGTCCAAAATCTAAGATCCACATCATACAAGAAAACGCAGATCGACTGAA
AAATATTTTACCAATGGATCTCCAACCAGTAACTGACCCCAAAATGTAAACCGGAAAAAAAAAAAGAAAAA
AAAGAAAGAGAACATGACACTCAATAAGCCTCCAGAATTGGGAGATAACAGGCTGCAGGATCTTCCTCACA
GTTGTCACTTGTAAATCCAAAATATTGTCGGACTGATGTAACTAGAAAGGTGATAATGTCGGCCTAACAGGC
ATGTGTTTGCTAAGTTTCCCTTTCACATGAAATCATAATCATCATAACCATCATATGCATTTACCACAGCTTCA
TCATCTGCCTTATCAACATGTATCTGCTTCTTCTTTGCACCTTTCTTTTTACTTGCATTGGCTTCCTTTTCTGCTTT
AATCTTCTCATTTGCAATTGCAGTAACAGAAGAGGCAATTTCTTTAGCATCTGCACCTTTCAACGCAGTCATTG
ACAATCTCATAACAGCCTTGAGCAACCCAATATAATGGTAGCTTTTCTCATATGGTCGCAGCTTATGAGAAAT
CAGCTCAGCATATTCCAAGAAATCATTCTCACTTTTGGGGATGAAATTGTCAATGGTCTTGTCATCACCACCTT
TCGTGGCGAACAATTCAGCTGTGGACTTATAATCAGCTTCTTCAACAAGCCTTTGCTGGCGAAGTTTTTCTGCT
ACTGGATCTAGAGGCTCTTCCTTAGTATGAATTTCAGCTTCCTTGCCTTTCTTTTCAGTAGCTTTTGCAGCAGAC
TTCTTGGGGGCCTTCTCAGCAGGAGGTTCTGGTTTAGGTGCCGGAACAGGCTCCTCTTCATCTTCCCACGACTC
TTTTACATCATTATCATCTGCGTCTTCGTCATCCCATTTACTCCTTGGTTGTTCCTTCTTAAGAAGATCCGGAAC
TGGCACATCCTCCCAATCCTCCATCTTTAGTTGATTCTCTTCTGTCCACAAAAACTTGTAAATTCGAGTATCAG
TACGATGGATGGGTGAGTCTCGCCGACGATGCCTTCCGACGGTGGATAAAGCGACCGAACAGAAAATCTCCG
ATCGGTGGGTCGCTAAACCCTAGTAGACTCCGCAATTCTTATCGGCTTGCGCGCAGCTAGGGAGATTGAAGGT
ACTAGAGGAACGTGAAGAAGAATCCAGAAAATCTGGTCGTTTAGGGTTAGCGTCTGGTCTCCCATTTTAATAT
CCAGATTCTATTAGACGGATCCGGG
>TCONS_00014045
GACAGTTACAGGAAATCTATAGGTCCAAAATCTAAGATCCACATCATACAAGAAAACGCAGATCGACTGAA
AAATATTTTACCAATGGATCTCCAACCAGTAACTGACCCCAAAATGTAAACCGGAAAAAAAAAAAGAAAAA
AAAGAAAGAGAACATGACACTCAATAAGCCTCCAGAATTGGGAGATAACAGGCTGCAGGATCTTCCTCACA
GTTGTCACTTGTAAATCCAAAATATTGTCGGACTGATGTAACTAGAAAGGTGATAATGTCGGCCTAACAGGC
ATGTGTTTGCTAAGTTTCCCTTTCACATGAAATCATAATCATCATAACCATCATATGCATTTACCACAGCTTCA
TCATCTGCCTTATCAACATGTATCTGCTTCTTCTTTGCACCTGCTTTCTTTTTACTTGCATTGGCTTCCTTTTCTGC

TTTAATCTTCTCATTTGCAATTGCAGTAACAGAAGAGGCAATTTCTTTAGCATCTGCACCTTTCAACGCAGTCA
TTGACAATCTCATAACAGCCTTGAGCAACCCAATATAATGGTAGCTTTTCTCATATGGTCGCAGCTTATGAGA
AATCAGCTCAGCATATTCCAAGAAATCATTCTCACTTTTGGGGATGAAATTGTCAATGGTCTTGTCATCACCA
CCTTTCGTGGCGAACAATTCAGCTGTGGACTTATAATCAGCTTCTTCAACAAGCCTTTGCTGGCGAAGTTTTTC
TGCTACTGGATCTAGAGGCTCTTCCTTAGTATGAATTTCAGCTTCCTTGCCTTTCTTTTCAGTAGCTTTTGCAGC
AGACTTCTTGGGGGCCTTCTCAGCAGGAGGTTCTGGTTTAGGTGCCGGAACAGGCTCCTCTTCATCTTCCCAC
GACTCTTTTACATCATTATCATCTGCGTCTTCGTCATCCCATTTACTCCTTGGTTGTTCCTTCTTAAGAAGATCC
GGAACTGGCACATCCTCCCAATCCTCCATCTTTAGTTGATTCTCTTCTGTCCACAAAAACTTGTAAATTCGAGT
ATCAGTACGATGGATGGGTGAGTCTCGCCGACGATGCCTTCCGACGGTGGATAAAGCGACCGAACAGAAAA
TCTCCGATCGGTGGGTCGCTAAACCCTAGTAGACTCCGCAATTCTTATCGGCTTGCGCGCAGCTAGGGAGATT
GAAGGTACTAGAGGAACGTGAAGAAGAATCCAGAAAATCTGGTCGTTTAGGGTTAGCGTCTGGTCTCCCATT
TTAATATCCAGATTCTATTAGACGGATCCGGG
>TCONS_00014057
CCCATGTATGGTTGTCCAAAATGGATTGTATCTCACTATTGACAGCTTCTTTCCAATATGGTGCTTCAGAAGAA
GACATAGTCTCCTTAAACGTTTGAGGCTCATTTTCTAGTAGAAAGGTTAAGAAATCTGGACCAAATGATGTAG
ACTTCCTTTGACGTTTGCTACGTCTTGGATTCTCCTCATTATGCATCTCATCATTTGTTTCTTCCCGTGGACGTTT
ATTCCTTGGAATAGATGTCTCATCTTCAATTTTATATGGAAATAT
>TCONS_00014134
GTAACTTAAAGGACATAAATGAAAGAAAAGGGCCAACACCTTAAAGATACAAATAGGCTTCACAACAACAT
AGGTAAAGTTCCAACATTTGAGAGACCACATGACAACATGATACTACAGAATCTAACCTCCCAGATTCAATG
ACAATACGAACCTTATTCAATTAGGTCAAGTAAAAAGAAAACATCAAAAGACCTCAGTTCTTTGACTGTCTC
GCAATCTTGAACCACTCGGTATGGTAAGACCCCGGCACATCAATCCTCTCATATGTATGAGCACCAAAGTAA
TCTCTCTGAGCTTGAATCAAATTGGCAGGAAGTCTTTCCCTCCTGTACGAGTCAAAGTAAGCAAGACTTGAAG
ACATACCCGGTGTACTAATACCCGAGTTGATAGCTAGGCAGACTACTCTTCGCCAAGCAGACTGTCGTTCAAC
CATCTCTTTTGCAAACTCTTCATCCACAAGTAGATTAGCAAGATCCGGGTTTCTGTCATAAGCCCCCTTGATCC
GATCCAAAAATATAGCACGGATAATACAACCACCCTTCCAAATCCTAGCAAGCTCCCCTAGTTTCAAATCCC
ATCCCTTTTCAACGCTCTTTGCCCTTATCAAATTCATGCCCTGAGCATAGCTACATATTTTGGATGCATAAAGT
GCCTTCCTCACATCGTCAATCAACTGATTCTTACCCACGGACTGGTCAACAAGGACATCACTAACCCCGCTAG
CTTTAAATACTTTAGCTGCTTGAACCCTTTCATCCTTTAATCCACTAAGGAATCTTGAATCCAATGATGAAGCT
ATTGTGGGTGCAGCAACTGACAGCTCAGCAGCTTGCTGAACGGTCCATTTACCAGTACCTTTCATCCCAGTTTT
ATCCAAAACTTTGTCTACAAGATACCCGTCTCCTTTATCATCCTTGATTCCAAATATATCAGCTGTGATTTCAA
TCAAGAAGCTCAGAAGCTCTCCTTTGTTCCAGTCCGAGAAGACTTGTTGTAATTCATCATTAGAGAGCTTGCC
AACAGATTTTAGCACATCATAAGCTTCTGCAATTAACTGCATGTCACCATATTCAATTCCATTATGAACCATCT
TAACAAAATTTCCGGAACCTCCTTCACCAATATATGTAACACAAGGGCCACTGTCGACTTGAGCTGCAACCTT
TAGCAAGATGTCCTCTATGTTCTTGTACGCTTCAAAAGAGCCTCCAGGCATCAGTGAGGGTCCGTTGCGAGCA
CCCTCTTCACCGCCTGAAACTCCCATTCCTAGATAAAGAAGACCCTTCTCGGCCATTTCTTTCTCTCTCCTCTC
AGTGTTCTCATACCATTCATTGCCACCATCAATGATACAGTCTCCTTTCTCCATGTAAGCAGAAAGGGTTTTGA
TGGTCTGATCAACTGGTGAACCGGCCTTGACAAGAATGATTATGACACGGGGCTTTTGGATAGAGAGTACAA
AGGACTCTGGATCATGAAAGCCATAAAGAGGAAGATTTCCTTCCTTTTTAGCTCGTTCAACGGTCTCGTCAAC
TTTTGAAGTGGATCGGTTGTAAACAGATATAGGAAATCCTTTCTCAGCAATATTGAGAGCAAGATTTTGTCCC
ATAACAGCAAGCCCAGCAAGACCAATTCTTGTCGGTGTAGCCATGTTGGTTACCTTTAACAGATGAAACTGA
CTCAAGAAGATTCTTTACTACCTCAACCAAGAACCAAGATATAGTTGTATGAGATTTCACGTTAAGTAATTGA

AACAACAAACAATTTATGGACTGGGGTTGTTGAGATTACGATTAGATTTGTGGAAAATTGAGTATAAATAGT
GAATGCATGAAGATATAATTGAATGGTGTGAACTCACCT
>TCONS_00014221
GTGCTGAGGTAACAGCGAAGTTAGATCGTTCATGTCCACATCATACATTGCTCTGTTTGTATAACTTGGTGTCA
AGAACTTGCTTTCCGCACATTGCACATACTCTTGCCATCTTGATGCACTTGTTGCTTGCAAATCATGCACTTTGT
ATTTCCATAAGGTGTCCACCTTTTTTTCTTGGAGAGGAGTTTGTTCTCGTTAATCTTACGGCCGCCGCCTTCAGT
AGTGTTACTGGCACCTTCTTTCCATTTATCGGGCACAATTACCTTGGACAACTTCTTCTCACACTTTTCGCAAA
CCATAATTGCACTTCGCGAGTTTGATCGATTGAAAGTATGC
>TCONS_00014222
ACATTTGATAGCATTCACAAAGAAAGAACAGTTCTCTAGAATAAGAGTGCTGAGGTAACAGCGAAGTTAGAT
CGTTCATGTCCACATCATACATTGCTCTGTTTGTATAACTTGGTGTCAAGAACTTGCTTTCCGCACATTGCACA
TACTCCTTTACTATAAGCACAGGTATGACAGTACTTGCCATCTTGATGCACTTGTTGCTTGCAAATCATGCACT
TTGTATTTCCATAAGGTGTCCACCTTTTTTTCTTGGAGAGGAGTTTGTTCTCGTTAATCTTACGGCCGCCGCCTT
CAGTAGTGTTACTGGCACCTTCTTTCCATTTATCGGGCACAATTACCTTGGACAACTTCTTCTCACACTTTTCGC
AAACCATAATTGCACTTCGCGAGTTTGATCGATTGAAAGTATGC
>TCONS_00014223
ACATTTGATAGCATTCACAAAGAAAGAACAGTTCTCTAGAATAAGAGTGCTGAGGTAACAGCGAAGTTAGAT
CGTTCATGTCCACATCATACATTGCTCTGTTTGTATAACTTGGTGTCAAGAACTTGCTTTCCGCACATTGCACA
TACTCCATCTTGATGCACTTGTTGCTTGCAAATCATGCACTTTGTATTTCCATAAGGTGTCCACCTTTTTTTCTTG
GAGAGGAGTTTGTTCTCGTTAATCTTACGGCCGCCGCCTTCAGTAGTGTTACTGGCACCTTCTTTCCATTTATCG
GGCACAATTACCTTGGACAACTTCTTCTCACACTTTTCGCAAACCATAATTGCACTTCGCGAGTTTGATCGATT
GAAAGTATGC
>TCONS_00014232
GCTGAGGGCCTGGTTGTGAGGATATTAATACCATAGCGCGTGAGTTATCCGCGTAGCACGTGAGTTGACCGTG
CGGGTTCAGGTATTGATACCATAGCGCGTGAGTTGTCCGCGTAGCACGTGAGTAGACCGTGCGGATCCAGGT
ATTGATACCATAGCGCGTGAGTTGTCCGCGTAGCACGTGAGTTGACCGTGCGGATCCAGGTATTGATATGGTA
GCACGTGAGTTGTCCGTGCTTAGCGCTTGGGCTTTGGGAGCCCCTCCGGAGTCTGTACACACCCCCAGTGAGC
GCAGAGTGTTGAG
>TCONS_00014233
GCTGAGGGCCTGGTTGTGAGGATATTAATACCATAGCGCGTGAGTTATCCGCGTAGCACGTGAGTTGACCGTG
CGGGTTCAGGTATTGATACCATAGCGCGTGAGTTGTCCGCGTAGCACGTGAGTTGACCGTGCGGATCCAGGT
ATTGATATGGTAGCACGTGAGTTGTCCGTGCTTAGCGCTTGGGCTTTGGGAGCCCCTCCGGAGTCTGTACACA
CCCCCAGTGAGCGCAGAGTGTTGAG
>TCONS_00014243
TTGAACACTCAACTCCTATAGCTGCTTTGGGAGTAATTGAAGAAATCTGAACTGTATTCTTGACTTCCAAAGG
CCATTCTTTGAAACATTCTTATCTGTGCAGATTGTTCCCTTGAATCAAAAATTTCACTTTGTCCAGGTTTCATTC
CATTGGACAGATCTGACAGCTCATCCATGGAATCTAGAGCTCTAACCACCTGGCTCATACGAGGCCTTTTTGG
GCCTGAATGACGTACACAAGCTGCAGCTGCTTCAATCATCCGGAACATCTCACTCGCAACAAAGTTCTTTTCA
AGCCTTGGATCTACTATACCTTCAAAATTTTCAGTCTCAAGTGCTTGAGCAAGCAAAGGTCGAGCCCATTCAA
CCAGGCTTTCATCACCTAAGGGCTGAGACTGGTCAACAGGTTTCCGCCCCGTAATAAGCTCCAAAAGCACAA
CGCCAAATGAATAAACATCAGATTTCTCTGTTAATTTTCCACTAGATGCATACTCTGG
>TCONS_00014267

CACAGAAAGATAAACTGGTTACTTGAAATTAACAAAGTGCTCAGCATCTACATATCACCAAAGTTGTATTTG
AGTAGAAACTTCATATAGAGTGCTAAATTGACATATCCACATGCACAAAATCTGACGGCCACAGTTAGCATC
AGGCATGGACTGAGTCCATTTGTTAACCCAACAAATAAATGAGCACAAGAAAACCAACAAGTAAGAACATA
AACTTAATATTGCCATCTGCCTGTACTTGGGTTGTGTCTTGAGCATTACGAACATGTGGCCCATGATATGCAGG
AGTATATCCAAATGGGTAATTTGATGTGGTAGGATACGCTGCCGGGCCAGGGAATCCATGAAACTGGAAGCT
GAGTAATGAAGGGAACAATCCACCAAAACCAGCCGACATTGTAAAGTTACCAAATCTTGCAGTTGCTGTTGG
AAAAAATCCTCCCATATGACCAAATCCAAGATTAGGAATATTATTTGATTGAGGTTGAGGAGCCGTTTCAGGT
CGTTGTCCTGCAGGCCTGTTAGGAATTTCAAGGCCTGGGATTGGTTTCGATCTGGGATCAGTAGAAGTCCTTC
CTCTGCCATAAAGAGGAACTAATTTCTCCTCCTGAATAAGGGCCTTACAAACAGGGCATTCATGGGATTGTGA
GTGAAGATGTAGCCATCTATAAAGGCATGGCCAACAGTACAGGTGACCACAGAGTGTCACAATGGGATCTTG
CGCCAATTCAAAACAGATATTGCATTCAAAATCACCAGCATCATTGCTCCCATCCCCAGAGGAAGAAGAAGA
GCTTTCAAATGCCCTGGTAGTTGAGTCCTGCATCTCACTCAGATATATCGTTTCTGAAGAAAAGCAAGGGGGA
TTCGGAGGCAATTAATGGATCGAAGATAAAAAAGTGGTAATTCGAGCCCTTCAATTAGTATTGAACAAGTAC
TGATCCCAACAAAGATGATTCTAGGGCTTTTCACCCTCTCTCCC
>TCONS_00014268
AAGAGAGACAAATACTGTACATAATGTAATCGAGGAGGATCTCAGGACAAAGATCTCTACTCTCAATCTCTT
TTAATTTACTGCAATTTCTAAGACACCTTAAGATTCAAGAGTATAAGGTGTTTGCAGCAGATCATAAGGTGCC
CAGAGGGCATCCAGGGGACAAGGGCGGTTCGAATCGAGTCGAACCCAAGGCTTCCCCTTTCCACTCCAATGC
AACAGGCTAACCGGACCAGGGTGCAAATCTCGGCAGAGTCCACGAAAATTATCTCCGCCTAGACCATGTTGG
TTCCACCTATGATCTACAGGTGCTATATTTCCAGCAAAAACAAGCAGAAAAGGCGGTAAAGAACCCAATTCA
TAAATCCTCATTCTCTTTTGCAACTCCATCCACTCCACAATCTTTGTAGTATAATCCCCTGCTCTCCATCTATCA
AGATCAATAACCATAACCCCTGTATTAAAATAACAAGGTTTTCGGTTTTGGTTAGCGAAAGTTAAGGAAAGT
GAAGGGTTAGACCAAAACGTGGGCGTGAAGTAAGTAGTGAAATTGGCATTGCAATATTCAGGTGCTGCTAAA
ACAGAGTCCTCTATTAGAGGTGTTGCTGCTAATTTAGCAATGTCGTCAACTAGAACAAGATCAGAATCAAGA
TAAACGACTTT
>TCONS_00014283
TGTGGTTGGGCCGAAGACTCCGTTGCTGTCAATAACAGTAGCTGATGATGTATTGCAGTGTCAGCCAAGTGCC
ATGCAAATATTATGTTCCCTATCTTTTGTTTGAAATGTCCAAGCTAGTCAAATTCTGCATGTGATTAGCTAATA
GTCCGATTCTGATTTCCCAAATTAGCAGCTTCGCCTGATGTCATACCATGGACCTCGAAAGATATTGATATCA
ATTTTAGTTG
>TCONS_00014325
CTTATTATAACCCTCTTTACTTCCTCATACTGTCACAAATTACAGACCCATTTTCCAGTTAAGAGAAAAAAAG
AAATCTATCAATGGGGCGTTCCGCTTTCGTTGTGGCTGTTTTTCTCATGCTTTTCTTCTCTCTTTCTCATGGGATC
GGCAGAAAAATGATCGGTTCTTCTTCCCATGGCGATACCCATAGAGTTTTACTTGAGGTT
>TCONS_00014363
AATACGGTAGTAAAGTAAAAATGATGCTTTTCTCTTTCTGCTAACTTATTGGATATGAAATTTAGCTCGATAAT
TACTAGGCAGCAAATTTAGGAGCTTCCCTGTGCTTGAGAATCTTTTTCTCGAGCTCATCTTTCTTAGCCCCGAC
CACCCTCTCTACTTCCTTGCCTTGCTTCAGCAGCAAAAATGTTGGCATAGCTTGCACTCCAAACTCTTGTGCTA
CATCCAGTTTATTTAATTGCTTGGAGGAGTTGAAGTGAAGTTGCCACCGATTGGAAGAATGAAAAGCAATCA
CACGCGACTGTTCTGACGACGATCCTGATTCTGCCTCTGCCGCTTCCGCCGCGCCTCCGCCGAGCAAACTTGA
AAGATACGATCCCATAGGATTCTTC
>TCONS_00014502

TTTTTTTGTTCTGATCCAAAAGGTTTCAAAGATCCTTTTTCAGGTTCACTTATTGTTGTTCCCTTCTCTTGTTGCA
CATGTGTTGTGTTGTTGCTTGAGGAATCTTTGCCAGTGCTCTCTCAGAATCGAAAAAGGATTGTTCAGTGCTTT
CTTCACTCTATATTAGGAGGTGGAGTAGATCAAGAAATTGAAGAAGATGATGGAAGTGAAGAACTAGACCTT
ACATGA
>TCONS_00014560
GCATAATCTATAGCAGAAAGGTGTTGCAATTCATCTGAGCACTTTTCCTTTGGTGGAGGGGAAAGAAATTTCT
CCAACAAATCTACATCTTTTTGTTCTTCTGCAGGAACCATATCAGATTCTTCAGCCACTCCATGTCCTTCATTA
ACAGCAAAGTCCATAACATCATCGATCCGATCAGATGGTTCAGCTGACTGAGGAGGGGTTTGAGTGCTCGGT
GTCCTAACCTGAAGAGCTGGTGAAGCTTTTTCTATAGATTCAACACTAACAGTTTGAAGTTCCTCTCCGTACA
AGACTCTCCCACTGGCCATTATTTCTCCTTCCTCAGTTTCGGGAGACATATTAACCTCATCATGACCATAGTCA
ACAATTGTAAGCCTTCCTCGTCTACTCACATTCACATTCAAATCCATCGGCTGAGGATTATTTGGAGAAGCGA
CTGACATAAACGGCGTCGTAGTGGTAGACCCAATTACCCCT
>TCONS_00014561
TAATCTCTTTAGAGGCATTACTTTCATGTATATGCTTGGGTGCTGCAGCTGTAATATTTCAGAGTCAGTGAATC
TAATTAGATTGTCAAGATCTTCTATCTAACTTCTTGTCACTAGGTCGCTTCTCTTCTGCCTCTTTGCGTCTTTGTT
GCGCATAAGCAGTGTAGCCAGTACCAGCATTAGCAGCTGATAAAAGTGCTGCATGTGCACCAACAGCAGAA
ACAGGATCTCTCCGATCAACGTCCACCTTGTCCCACTTTGACTTTTTGTTCTGTCTACCATCCCGAGTTACAAC
ATCTGGAATTGGCAAATTGATTTTTGGTGTAGGAACCATGGCTGCAGGTTGTGTTCCTCCAGATATGAAGTCA
ACTTTTGGACTCCTCTTCCTCTCTTGCTCCCTTCTCTCCATTTCACGCTTCATTTCAACTTCTATTTCATCATAGA
AGTCACTTTTGTCATATCCACGCGGATCAAATACGTCTTTACTGAAGCAAGATCCTATCTGGTCAATATCTTGA
TAAGTCACTGCATGCAGCAAGAAGTCTGGATTCCGGTACTCCTTTCTGTTGCGCACTTCTGCATTAAAGCTTCT
GCCAGTAGTCTTCTTTAAAGCAAGGAATTTAATGATCTTTTCTTGCAATTCATCTGAGCACTTTTCCTTTGGTGG
AGGGGAAAGAAATTTCTCCAACAAATCTACATCTTTTTGTTCTTCTGCAGGAACCATATCAGATTCTTCAGCC
ACTCCATGTCCTTCATTAACAGCAAAGTCCATAACATCATCGATCCGATCAGATGGTTCAGCTGACTGAGGAG
GGGTTTGAGTGCTCGGTGTCCTAACCTGAAGAGCTGGTGAAGCTTTTTCTATAGATTCAACACTAACAGTTTG
AAGTTCCTCTCCGTACAAGACTCTCCCACTGGCCATTATTTCTCCTTCCTCAGTTTCGGGAGACATATTAACCT
CATCATGACCATAGTCAACAATTGTAAGCCTTCCTCGTCTACTCACATTCACATTCAAATCCATCGGCTGAGG
ATTATTTGGAGAAGCGACTGACATAAACGGCGTCGTAGTGGTAGACCCAATTACCCCT
>TCONS_00014595
CACTTACAGCAGTGATTATTTGTCTAACGTATTGTTTTAATGGCCTGGATCAGGTTTGTTAATCTTATGGAAGG
TCATATTTGGATCGAAAGTGAAGGTCTTGGCAAGGGGTCTACTGCTATCTTTATTGTTAAACTTGGGATTCCTG
GACGCTCAAATGAGCCTAAGCTTCCCTTTATGCCCAGATTGCCTGCAAATCACATGCAGATGACTTTTCAAGG
ACTAAAAGTTTTGGTTATGGATGATAACGGGTGAGTACAGATACTACCTCTTTATCTTTAGACTCTTATTTGTC
CTGCTGTTTGACGACTTG
>TCONS_00014638
ATGCCCTCTTGCTGAGCTGCACCGCCATGAGCCTGAGACATTGTTTTTAGCTGAGGCTTACCCCTCTCCTCCAC
CTTTCCCGGGGGACGAGAAGGAAAGAAGGGCGAAACAATGCTAGTAATTTGAAAAAGAATTCGATAGAACT
ATGTTCTTACCATTAACAAATTTCAGGTAGCAAACGATCTCAGAAAAAGAAACCATCCTCACCAAGATACTC
GCTGCTAAATGGGTTAGTGAAGTCGTCGTAATCAAAGTCATCATAATCAAACAAATAGTCTGAAAGGCCAGC
TTCAGACAACCCAGATGGGTATTCATCATTGGAAGAACTATAATCACAAATCTGAGCTCTAAATTCATAACC
AGAAGTGGAATCACGAGGGCACTTCAGATCCTTAATCTGTTGTCGACATCTCTTTCCTAAATCCCCTTCAAGA
TCAATACTATAACAGTGGCGCAAGTCAAGTGATTCAAGCCGCGGGCAGCCATCAAGAATGGCACACAGGCC

TTCATTTGTCAAGTTATTCCCAAAAAGTGCAAGGCGTCGCAATTCAGGCATATTTCCTGCAATAGCCAGAGCT
TCATCATTGACATTAAAATGTAAACTTCTAAATCCTCTGAATCCTCCAAATCCACTGTTGTTCAATGTAAATGA
CTTGAGCTGCGAGCAAGAACGACCGATAGCCTCTATATCAACTTTACTAATAACAGCTAAGTAAATGTGCAA
CTCCTCCAACAACGGGAAGTTCTTGGCAACTGCAGCCAAACCTCCATCTGAAATTGCTTCACAGCTGACAAG
TCTTAGATGCCTTAGCTGACTTGATCCTGGCTGCGATTCACAGCGACGCGGCACATCCAGTCCAAGTCAGTAT
CATCTATGTCCACATCAATGTCGTTTCCCATGTCAATGTCCCGCCACATGATAGGGTCATGGCACACATTCCA
CCACGTACTACAGACTCTCTGTGCATTTTGCAATATCTCTATCGCTCCCAACCGGAGAAGGATGTCCGCCGTG
ATTTCTCGAGGGAGCTCTACCCACGGCGGCGACGGCGGCGGCGGTGGTTTGGAGGAAGAGGTTGACGCTTTC
TGATTTTGCTGCTTGATTCTCCATACCAACCGTTTTGGCTTCGGCATTTCTCTGTGTTTAGGGTTTTGTCGTCTGT
GTTTTCTCCTAGTACTTTCTGATTA
>TCONS_00014789
TTAGAAAAGAGAAGAAAACACAAGAAACAAACAATGAACCAAGTTTTCTCACTTTGATTTCTTTCCCTGCTTC
TTTTTTATCACTTCATCAATGTACATTATACCTTTGCCTTTGTAAACTTCAGGGGGCTTACAGCTCCTTACAGAA
GCAGCAAACTGATGCACCCTATATTTGTCAATCCCAGTGCAGCAAATCACATTGGGTTTGAAACAGAACACG
CGAACTGCAGGGGGCACACTCAGTTCAACCTCATGACTGTAGCCAAGCTTGAGGTACAAGAGACGACCCTCG
GATTCTGCTCTAGCCTTGAAACCCACTCCAACAATCTTGAGAAACCGAAAGAATTTAGCTTCCATGGGATGA
ATTTTATCAATCAGCTTTGGACCTGCCGATGATTCTCTCACAGACGACTATCGGAGATTGAACCACTGCAACT
CGTAGTCGGTCATCAAATGGTACTCGTCGTCGGAGAACCTGAGACTGAAGAGGAGGAACGGGGAGATTGTG
AGG
>TCONS_00014930
TGCTATTAAGATATTTCTCCTTATCCACCCTAACTATCTGACCTTTCTTCATAGAGTAGACAGGTTTTTAGGCT
TTGTTGCGGTGGCAGTAGAGGAAGTTGCTGCTGAAGCTGCTTCTTTGGATTTTGGCTTTGCAGGTTTTTCATTGT
CAATTGGTTCAGAGGGATTTTCTGCTTTAATAATTAAAGGTAATCTAAATGGGGTATTAATTCTTTTTCTTGTCA
ATGAGAAAGATGAAGAATATGTGAAGGAGGTGTGTGAAAAAGCTGAAGAGAA
>TCONS_00014931
TTTGATATATTCTTGTCTGACATGTACTGACTGGTTCGAAATGTGGACCCTTTACTATATTCTCTCATAATCCAG
CTTATCAGACTTTATTAGCATCTCTGTTGGAAGCCAAGCTGGTGCAGTTGGTATTCCGATCCATCCAATAAGTG
CATATTCTCCTGTCTCGAATATGCGCAAATCCAATACCTCACCACGGTCTTCGTATATATAGTCCAAGCCTTTG
TAATAAGGTGGATGATCCACTGACAAATA
>TCONS_00014960
TTGACATGAATAGTTAAAGGGAAATATCCAAATCCATGTCTTATTATTTTCAATAATCCATACTTGTAGCAAT
GAAATAACTCCCATAATCCATTTTCTCTTCACAAAATTCCCTTCAATCCCCCCGTTTAGTTGACATGAATAGTT
AAAGGGAAATATCCAAATCCATGTCTTATTATTTTCAATAATCCATACTTGTAGCAATGAAATACTATATATT
CCTCCACCAAGTGATAAATGATTGATTACAAATATTTAGGATCTATTTTTCTTTTCTCTATAAAGGCCCAGAAA
TACCTTGTTTACGTATCATTGGAAAGTGCATTAACATAAATACGGCAGTCAGAAGCGGCAATACAAAACTGT
GTAAACTATAAA
>TCONS_00014961
TTGACATGAATAGTTAAAGGGAAATATCCAAATCCATGTCTTATTATTTTCAATAATCCATACTTGTAGCAAT
GAAATACTATATATTCACACTCCCATAATCCATTTTCTCTTCACAAAATTCCCTTCAATCCCCCCGTTTAGTTG
ACATGAATAGTTAAAGGGAAATATCCAAATCCATGTCTTATTATTTTCAATAATCCATACTTGTAGCAATGAA
ATACTATATATTCCTCCACCAAGTGATAAATGATTGATTACAAATATTTAGGATCTATTTTTCTTTTCTCTATAA

AGGCCCAGAAATACCTTGTTTACGTATCATTGGAAAGTGCATTAACATAAATACGGCAGTCAGAAGCGGCAA
TACAAAACTGTGTAAACTATAAA
>TCONS_00014962
GACCAATCAACAGTTCAAGTCGCACACTCCCATAATCCATTTTCTCTTCGCAAAATTCCCTTCAATCCTCCCGT
TTAATTGACATGAATAGTTAAAGGGAAATATCCAAATCCATGTCTTATTATTTTCAATAATCCATACTTGTAGC
AATGAAATACTATATATTCACTCCCATAATCCATTTTCTCTTCACAAAATTCCCTTCAATCCCCCCGTTTAGTTG
ACATGAATAGTTAAAGGGAAATATCCAAATCCATGTCTTATTATTTTCAATAATCCATACTTGTAGCAATGAA
ATACTATATATTCCTCCACCAAGTGATAAATGATTGATTACAAATATTTAGGATCTATTTTTCTTTTCTCTATAA
AGGCCCAGAAATACCTTGTTTACGTATCATTGGAAAGTGCATTAACATAAATACGGCAGTCAGAAGCGGCAA
TACAAAACTGTGTAAACTATAAA
>TCONS_00014963
TGACATGAATAGTTAAAGGGAAATATCCAAATCCATGTCTTATTATTTTCAATAATCCATACTTGTAGCAATG
AAATAACACTCCCATAATCCATTTTCTCTTCACAAAATTCCCTTCAATCCCCCCGTTTAGTTGACATGAATAGT
TAAAGGGAAATATCCAAATCCATGTCTTATTATTTTCAATAATCCATACTTGTAGCAATGAAATACTATATATT
CCTCCACCAAGTGATAAATGATTGATTACAAATATTTAGGATCTATTTTTCTTTTCTCTATAAAGGCCCAGAAA
TACCTTGTTTACGTATCATTGGAAAGTGCATTAACATAAATACGGCAGTAAGAAGCGGCAATACAAAAGTGT
GTAAACTATAAAAACGGGTCAAAGTAGATTGTCCCACACTAGCGCTTCCGCGCAATAATTCGACCAAGGGTG
ATCCTATTACAGGAATAGCGTCAGGGACACCTGTTACTATTTTCACTGCCCAATAACCAACTTGGTCCCAAGG
TAAGGAATAACCAGTTACGCCAAAAGATGCGGTTAATACAGCCAGAACCACACCTGTAACCCAAGTCAATT
CGCGAGGTTTTTTAAATCCGCCGGTGAGATACACACGAAATACATGCAGGATCATCATTAGGACCATCATAC
TTGCCGACCATCGATGAACTGATCGGATTAACCAACCAAAGTTGGCTTCAGTCATTATGTATTGAACAGAAGC
AAAAGCCTCAGTAACGGTCGGACGATAATAAAAAGTCATAGCAAACCCAGTAGCTACTTGTACTAAAAAAC
AAGTAAGCGTAATTCCCCCTAAACAATAAAATATATTGACGTGGGGAGGAACGTATTTACTAGTTATATCATC
CGCAATTGCTTGAATCTCGAGACGTTCTTCGAACCAATCATATACTTTATTGAGATAGGCGTAACTCCCCCTCT
GAGAACCGTATATGAGACTTTCATCTCGTACAGCTCAAGCAAAAACACCCAAATACTAGTTGCAACGGATAT
GGAATAGAAAGTTTTAAATTTATTAAGTTCTGATCCCATCTTCTCTGGAAAATCCGAAAGCTCTTCTTTGTGGC
GATAATACCAAAATATCAAGTTGGCGCAGTCATCTTTATCTTTATTTTGATACTTATGGGCCTCTTGAAT
>TCONS_00014964
TTGACATGAATAGTTAAAGGGAAATATCCAAATCCATGTCTTATTATTTTCAATAATCCATACTTGTAGCAAT
GAAATACTATATATTCACTCCCATAATCCATTTTCTCTTCACAAAATTCCCTTCAATCCCCCCGTTTAGTTGAC
ATGAATAGTTAAAGGGAAATATCCAAATCCATGTCTTATTATTTTCAATAATCCATACTTGTAGCAATGAAAT
ACTATATATTCCTCCACCAAGTGATAAATGATTGATTACAAATATTTAGGATCTATTTTTCTTTTCTCTATAAAG
GCCCAGAAATACCTTGTTTACGTATCATTGGAAAGTGCATTAACATAAATACGGCAGTAAGAAGCGGCAATA
CAAAAGTGTGTAAACTATAAAAACGGGTCAAAGTAGATTGTCCCACACTAGCGCTTCCGCGCAATAATTCGA
CCAAGGGTGATCCTATTACAGGAATAGCGTCAGGGACACCTGTTACTATTTTCACTGCCCAATAACCAACTTG
GTCCCAAGGTAAGGAATAACCAGTTACGCCAAAAGATGCGGTTAATACAGCCAGAACCACACCTGTAACCC
AAGTCAATTCGCGAGGTTTTTTAAATCCGCCGGTGAGATACACACGAAATACATGCAGGATCATCATTAGGA
CCATCATACTTGCCGACCATCGATGAACTGATCGGATTAACCAACCAAAGTTGGCTTCAGTCATTATGTATTG
AACAGAAGCAAAAGCCTCAGTAACGGTCGGACGATAATAAAAAGTCATAGCAAACCCAGTAGCTACTTGTA
CTAAAAAACAAGTAAGCGTAATTCCCCCTAAACAATAAAATATATTGACGTGGGGAGGAACGTATTTACTAG
TTATATCATCCGCAATTGCTTGAATCTCGAGACGTTCTTCGAACCAATCATATACTTTATTGAGATAGGCGTAA
CTCCCCCTCTGAGAACCGTATATGAGACTTTCATCTCGTACAGCTCAAGCAAAAACACCCAAATACTAGTTGC

AACGGATATGGAATAGAAAGTTTTAAATTTATTAAGTTCTGATCCCATCTTCTCTGGAAAATCCGAAAGCTCT
TCTTTGTGGCGATAATACCAAAATATCAAGTTGGCGCAGTCATCTTTATCTTTATTTTGATACTTATGGGCCTCT
TGAAT
>TCONS_00014965
GCACACTCCCATAATCCATTTTCTCTTCGCAAAATTCCCTTCAATCCTCCCGTTTAATTGACATGAATAGTTAA
AGGGAAATATCCAAATCCATGTCTTATTATTTTCAATAATCCATACTTGTAGCAATGAAATACTATATATTCTC
CCATAATCCATTTTCTCTTCACAAAATTCCCTTCAATCCCCCCGTTTAGTTGACATGAATAGTTAAAGGGAAAT
ATCCAAATCCATGTCTTATTATTTTCAATAATCCATACTTGTAGCAATGAAATACTATATATTCCTCCACCAAG
TGATAAATGATTGATTACAAATATTTAGGATCTATTTTTCTTTTCTCTATAAAGGCCCAGAAATACCTTGTTTA
CGTATCATTGGAAAGTGCATTAACATAAATACGGCAGTAAGAAGCGGCAATACAAAAGTGTGTAAACTATA
AAAACGGGTCAAAGTAGATTGTCCCACACTAGCGCTTCCGCGCAATAATTCGACCAAGGGTGATCCTATTAC
AGGAATAGCGTCAGGGACACCTGTTACTATTTTCACTGCCCAATAACCAACTTGGTCCCAAGGTAAGGAATA
ACCAGTTACGCCAAAAGATGCGGTTAATACAGCCAGAACCACACCTGTAACCCAAGTCAATTCGCGAGGTTT
TTTAAATCCGCCGGTGAGATACACACGAAATACATGCAGGATCATCATTAGGACCATCATACTTGCCGACCA
TCGATGAACTGATCGGATTAACCAACCAAAGTTGGCTTCAGTCATTATGTATTGAACAGAAGCAAAAGCCTC
AGTAACGGTCGGACGATAATAAAAAGTCATAGCAAACCCAGTAGCTACTTGTACTAAAAAACAAGTAAGCG
TAATTCCCCCTAAACAATAAAATATATTGACGTGGGGAGGAACGTATTTACTAGTTATATCATCCGCAATTGC
TTGAATCTCGAGACGTTCTTCGAACCAATCATATACTTTATTGAGATAGGCGTAACTCCCCCTCTGAGAACCG
TATATGAGACTTTCATCTCGTACAGCTCAAGCAAAAACACCCAAATACTAGTTGCAACGGATATGGAATAGA
AAGTTTTAAATTTATTAAGTTCTGATCCCATCTTCTCTGGAAAATCCGAAAGCTCTTCTTTGTGGCGATAATAC
CAAAATATCAAGTTGGCGCAGTCATCTTTATCTTTATTTTGATACTTATGGGCCTCTTGAAT
>TCONS_00014966
GCACACTCCCATAATCCATTTTCTCTTCGCAAAATTCCCTTCAATCCTCCCGTTTAATTGACATGAATAGTTAA
AGGGAAATATCCAAATCCATGTCTTATTATTTTCAATAATCCATACTTGTAGCAATGAAATACTATATATTCCT
CCACCAAGTGATAAATGATTGATTACAAATATTTAGGATCTATTTTTACTCCCATAATCCATTTTCTCTTCACA
AAATTCCCTTCAATCCCCCCGTTTAGTTGACATGAATAGTTAAAGGGAAATATCCAAATCCATGTCTTATTATT
TTCAATAATCCATACTTGTAGCAATGAAATACTATATATTCCTCCACCAAGTGATAAATGATTGATTACAAAT
ATTTAGGATCTATTTTTCTTTTCTCTATAAAGGCCCAGAAATACCTTGTTTACGTATCATTGGAAAGTGCATTA
ACATAAATACGGCAGTAAGAAGCGGCAATACAAAAGTGTGTAAACTATAAAAACGGGTCAAAGTAGATTGT
CCCACACTAGCGCTTCCGCGCAATAATTCGACCAAGGGTGATCCTATTACAGGAATAGCGTCAGGGACACCT
GTTACTATTTTCACTGCCCAATAACCAACTTGGTCCCAAGGTAAGGAATAACCAGTTACGCCAAAAGATGCG
GTTAATACAGCCAGAACCACACCTGTAACCCAAGTCAATTCGCGAGGTTTTTTAAATCCGCCGGTGAGATAC
ACACGAAATACATGCAGGATCATCATTAGGACCATCATACTTGCCGACCATCGATGAACTGATCGGATTAAC
CAACCAAAGTTGGCTTCAGTCATTATGTATTGAACAGAAGCAAAAGCCTCAGTAACGGTCGGACGATAATAA
AAAGTCATAGCAAACCCAGTAGCTACTTGTACTAAAAAACAAGTAAGCGTAATTCCCCCTAAACAATAAAAT
ATATTGACGTGGGGAGGAACGTATTTACTAGTTATATCATCCGCAATTGCTTGAATCTCGAGACGTTCTTCGA
ACCAATCATATACTTTATTGAGATAGGCGTAACTCCCCCTCTGAGAACCGTATATGAGACTTTCATCTCGTAC
AGCTCAAGCAAAAACACCCAAATACTAGTTGCAACGGATATGGAATAGAAAGTTTTAAATTTATTAAGTTCT
GATCCCATCTTCTCTGGAAAATCCGAAAGCTCTTCTTTGTGGCGATAATACCAAAATATCAAGTTGGCGCAGT
CATCTTTATCTTTATTTTGATACTTATGGGCCTCTTGAAT
>TCONS_00014967

GACCAATCAACAGTTCAAGTCGCACACTCCCATAATCCATTTTCTCTTCGCAAAATTCCCTTCAATCCTCCCGT
TTAATTGACATGAATAGTTAAAGGGAAATATCCAAATCCATGTCTTATTATTTTCAATAATCCATACTTGTAGC
AATGAAATACTATATATTCACACTCCCATAATCCATTTTCTCTTCACAAAATTCCCTTCAATCCCCCCGTTTAG
TTGACATGAATAGTTAAAGGGAAATATCCAAATCCATGTCTTATTATTTTCAATAATCCATACTTGTAGCAAT
GAAATACTATATATTCCTCCACCAAGTGATAAATGATTGATTACAAATATTTAGGATCTATTTTTCTTTTCTCTA
TAAAGGCCCAGAAATACCTTGTTTACGTATCATTGGAAAGTGCATTAACATAAATACGGCAGTAAGAAGCGG
CAATACAAAAGTGTGTAAACTATAAAAACGGGTCAAAGTAGATTGTCCCACACTAGCGCTTCCGCGCAATAA
TTCGACCAAGGGTGATCCTATTACAGGAATAGCGTCAGGGACACCTGTTACTATTTTCACTGCCCAATAACCA
ACTTGGTCCCAAGGTAAGGAATAACCAGTTACGCCAAAAGATGCGGTTAATACAGCCAGAACCACACCTGT
AACCCAAGTCAATTCGCGAGGTTTTTAAATCCGCCGGTGAGATACACACGAAATACATGCAGGATCATCAT
TAGGACCATCATACTTGCCGACCATCGATGAACTGATCGGATTAACCAACCAAAGTTGGCTTCAGTCATTATG
TATTGAACAGAAGCAAAAGCCTCAGTAACGGTCGGACGATAATAAAAAGTCATAGCAAACCCAGTAGCTAC
TTGTACTAAAAAACAAGTAAGCGTAATTCCCCCTAAACAATAAAATATATTGACGTGGGGAGGAACGTATTT
ACTAGTTATATCATCCGCAATTGCTTGAATCTCGAGACGTTCTTCGAACCAATCATATACTTTATTGAGATAGG
CGTAACTCCCCCTCTGAGAACCGTATATGAGACTTTCATCTCGTACAGCTCAAGCAAAAACACCCAAATACT
AGTTGCAACGGATATGGAATAGAAAGTTTTAAATTTATTAAGTTCTGATCCCATCTTCTCTGGAAAATCCGAA
AGCTCTTCTTTGTGGCGATAATACCAAAATATCAAGTTGGCGCAGTCATCTTTATCTTTATTTTGATACTTATGG
GCCTCTTGAAT
>TCONS_00015301
CCATTTTTCATCTGAAGGGTAACCTCCTGCAAACTCCTCAGATACTGGAGTCCTCATTTTAAGACAAAAAGTG
GAAGGATACTCAACGGCGTCAGGAAATACATCGGTAATATGAGGAATATTAAAACGAGCCTCAAATTCTCTC
TGATACTTTTACTACCAATTAGGATCACTAAAATTGGTGCTGCCCCCGCTGACAGCATTTTCGGAGGCTCCGTT
TTTAGCTTGCACTCGAATTCTCAATCTCCGGCTAACTCTGCTCGGATTCCATACAGTTCTTAGCCTAACGGAAC
TTAAAAAGAGATCGTGATCGCTGTAAGTAGTTGTAGACCATAGAATCGCT
>TCONS_00015302
GCCACTTTGGAATCGCCTGCACCCGTTCCACATAAGCTGCATTGGTCCCTGTACCTAAGTTAACAGCAATAGC
TACGTCGTTGTGTGTATATTTACCACCAGCCAACGTCCCAACAGTATCATTCACCAGAGCTGAGACCCGCATA
TCAACGCCCTTTCTTTTTATAGCTTTTGTGAGTTCTCCAACAACATCTTGGCCAACC
>TCONS_00015427
GCATGAAGTATACAATCTACTCTCAAGAACCCGTAGAAGAATAATGTAGTAATTCCTTCTGTCCTTATAGCTC
TGGATCACCCTAGGATATGGTTCAACTGTCAAATCCTAATAGGCGACCAACTATGTATTCATGTCAAATATAA
TCGACCATTGAATGACCTAAGAAACTCATTTCTTCTTTCATTCAATTGCCCTAGCCAAGGTCTTAGTTTGGTCA
TCTATAATTCATGACAACATGGAGCTTAAACTCATTACCAAGAGTTGACAGATTCCGTCTTGATCAATCACTA
ATTCTACAAGCATTTAATCGTACCCAATATCCTTTCAACTATCGCCCTAGGGCCATAGGTGTCTGGTATCAAA
GCACAATAAATAACTTGTCAATTACTATGACGAT
>TCONS_00015496
CTCTGTTAGAAGCAACTTTTATTGGGTTAGAAACTTTTTGGTGGGGCAGAATTGGTTGCTTTGCAGAGGAAGTT
GTGTTTCCCATTGCTGCTAAAGTTCTTTGAGTTTTGGATTTGAAAAAGTGCTGTCGTACAGTTTCTGTTGTGTAT
ATGAGGAAGCATTTGGAGATTCCAATGGGAATTATTAATAGAGCTAGTGGCCGAAGGAGGTTCATCTGAGCC
CTCTTCGCTGGAAAATTACACTATAT
>TCONS_00015570

TTGCCTGTCAATTCTGTTCTTGAGTTTCAACAAGAACTTCTCGTTATCTTCATCAGCAACTTTAACAAGCCTTTC
AAGCAAATTCTTTCTTTCTAAAACACCAAGATCATCTACATCAACTTCAGCAGCAGCACCTTGTGATCCAAAT
AATATACCTTTTCTTAATCTATCAAAAGTTGGAAGTTTTTCAAGAGCAGCCCATTTAAGTGCCTCTTCATCATC
TTCATCTCTAGATGAACGTGAGAATATTTCTACACCATTATTTCTCCATATTGAATTGCTATTTGCCCTTAAACT
CCCTCTCGTACTTCCTCTTAAACTACTCCCTCGCAAATTACTTAAATTTGCTGGCTCCAT
>TCONS_00015748
AAAAAAATCTGTTCCAAACTTTACAGACTATATGTTTTTCTTCTCTTCCAATTTCCTGAGCAGAGGCTAACAAA
TATATCTCAGCAAGTATGGGAAAAAGTTAGCTAGTAAGGCACGGTGGCAGTAACAGTTCTGTGTCTTGGATC
AGTGAGAACAGTTTGTACATTGGCAAAACCAGTTTCCTTCATCACTCTTTCAAGATCAGTCAAGTAGTATTCA
TCCAGAAAAGGTTCTGTGCTCTTCATTAATGTAAACAGCACTGGTGGTAATTCCTGAAGAATCTTCGACTTTG
GCGAGTTATCAGTTATAGCAACAGTTCCTCCAGGTCGAAGTATCCGAAATGACTCCTTCACTAAATTTCTTATT
GCTCTTTCAGGACATTCATGAAACACATAAGCAATTGAAACAAGGTCAAATGATTTGGAAGGCAAGCCAGTG
TTTTCACCATTTGCATGTATCCAGCTTATAGGATTCTTTCTCTGGTTTCTTTTCTTTTCTTTATACTGAGCAACAG
CAAGAAAATAAGGTGAAAGATCCAGACCAGTCACTTTAGCAGAAGGATATCTGTCCGCAAGATATCCCGTG
GTCACACCAACCGAGCATCCAATGTCAAGAATGTCTCTGATTGAGAAGTCTCCTGAATATTGTTGATGATGTT
TTTCGATTGCATCAAGCCAATTTCCGCGTACAATCTGATTTGCTTCTTCAAGTGAAGAAGCATTAGGTATTGTA
CGTCTCACCATCGACATAGTTGCAGCCTCTACTTCAGCTGCAGCCAGCCAGGAGAGGTTGCCCTCGTCATATG
CGTGGAAGGGATTTAGATAATAATCAGGATATGCAATGGCAGGATTCTGGATGCTCTCTAACTCTTTGTAAAC
ATCAGAATCCAGTATCTCCTTTGTCATCTCCCTCCATGGAATATTAGTTTCTCAGCTGTACTGATAAGAACTT
GTCTGGCACCAAGTTTGAGTATAGAGAAGAGAGGTTTGAAGGAAATAAGAGCTCCAACTAGAAGTGAAAGT
GGGGTTTCCCCTGTCCAACGAGGCCTTTCAAGTTGTCCTTCCTCAAATGCCTCTGCAATTTGTGCATTTCCACT
ACTACTTGTACTTGAAGAAGCTGACAATTTAAACCCCAAAACACTACGGCTTCTTCTTATTGGATTGTTAGTTA
TGGTGAACAAATTGGGAT
>TCONS_00015768
CTTGTTTACGAAAACCATAATACACAGAGAAGTTGTTTGAATTTCAATCGGAATTATGTATCACAAATTGGGA
TAGAGAGTATGAAGAGGTAGCTCAACGTCATACAATGACTGCAATCATAAATTCGGTCTATTGAGCACGTTA
ACACCAAGTTTATTGCTTACTGCCATAAGGACCATGGAGAGAGTACCTTTACAAGCACCAGAGTAAAGGTAT
AACTAAAATGAAGAACAATAAGAGAAGTAAAAGTGCAACTCAAGTAGAATTGAATGTAGTGAATTATTCAC
ATATTTACCAAATCATAAACTTTGGGGAAACCGACGGGGTGAAGTAGACTTAAGTTGGAAATAGATGTAACT
ATATTTTTTTTCATAAAAATATATTACTGTAAGCCATAAATAAGATTCATACCTTGGCTCTACAATATTCAGTA
TTGATTACATTTTACCAAATCATCCACGGGAACAAACCCTGCCCGCCCTTAACTTAATCTAATTAGGTTGGTAT
TATTACGCCTATACTCGAGTAAATGCACACACAAGATTAAAAGGTAAAATCATGCAATACCAAGTGATGTAA
GATCAAAAGAGTAAACTGTAGATACAAATCAAGAGTGACCATAAGGATGTTCTTTGTCGTTGGGAACCCTTG
CAGCTTTAAACGACATCAATGCCACTCCACTCAGAACCCGAAATCATGAGTACACTATGAAATCTTCGCAAA
AACCAAGTGGGTTTTGTTCAAATGTAGCTTAGATGAGCACGAGAGACAGCATGTAGATATAGAAATAGGAA
AAGTAGC
>TCONS_00015908
CAGCCCATTGGTCCTACTAAAGAAGATGTAAAAGAGTTTGGTAGCTTCCTCGGTACATTGGCAAGGACTGCA
ACTCTTTGCCCGCTGGATATATTGGATTGGAGGAAAATGGACACAAAAGACGATTTATGGACATATACCAAG
TTGAAGTATGATATTCCTGATGCTGCAAAAACATGAACTTTATTTTCAATTGGAAATGCTTGGAGAAGGCATA
AAAGTCAACTA
>TCONS_00016038

ACAGCATCTCCTCTCTGTGGAACCAGAAGCGGCAATTACGTCAAATATTGCTCTGAGATATATAGCTCTCCTA
CGATTCAATGATCAAACAAATATCTTGCTCTGTGATGTTTTCTCGCTAACAAAATTGATTTAGCAGCAACGA
CTACCAAAGTTTGAAATCAACTTCTGTTAATGCTTGGGGGCTTCAAAGTTTATATTCTGCAGCTTCAGTCTCCT
AGAAGCTTCTGTTTTTTGGATGTGTTCATGTAAGGCCTTATGACCCACTCGGTTTGAGCTTCATCCTGATCCATC
GTTCCTAGTTTTATCTGGTAAAGCCGTAGTTCAAATCGAGGACCAACCTCTTTCAGCTCAATTGATTTTGGACC
TCCGTGCTTTTCATAGATGTGATGTCTGAATGAAATGTAGTCTGACTGGTTAGCAAATGTGATAATACGTTTTG
TATCAGGCTTGGGTACCGGAAACAAATGCTTTAGGATGTTAACTGTCCTCTCACCAAGCTTTGTTGAAAATTT
GTCAAAAATCAGATGTGGGTACGCCTCAGACATGGTTCCAATAGATTTTTTGTCCTTTATATCATGTCTCGTGA
CCACATTGAGCAATCCAAAGTAAGCAGTTGGACCAAACGGCAGATGGCTAATGATAATACCATCAGGCACA
CCACGATGCTCATGGACCAAAATTACATCTGTAAAATCATGAGCTCGGCAGGTTTCAATTATTTCTGATATAA
CCTGACCACCACGATTCATCCGCTGAGCATTAGGGAACACAATTTTCAATTCCTTGACAAACTGAGTAAGAG
GAGCACTAGGATTTCTTGAAGTGGTAAGCAAAATTTTGGGATCTTTTTCAGTTGCATTAGCATATTCATCATCA
ATAGTTGATCGGGGTACAGCAGTATTTTCGTCTTCAAGGTCGATTTCTTTGCGAAGAGCAGCCTCTTCATTCCT
GAGCTCAGTTGGAATCGGTTTTCCCTCCTCCAAAGCTTCTTTGATTTTGCGTTTCTTCTCATATAACAAACGCTC
CTTCCCTTCTAAGCTTTTCCTGTAAAGGTACTCTCTTCGCAACCTAATGTTCCTTCTATGCATGGCTTCCAAGTC
GTTTGAAGGTTTCCTGTGCTAGTAGGCTATAAGAGATTTTTTGGTAGTAGGGTTTAAG
>TCONS_00016056
TTTTGCATCCTATTCCACTTAATAGGGTGCCAAGATTAACATAAAATGCTTGCTCCTCAAGAGCTTTTCCTCTC
TGTATTATTTCCGAGAAATAAATTGCCTCTGTTCAGGGGATGCTAGCCTTCATCACCACCTGTGTCTGCATCTG
AAGATCCATCTTCGTAGCATTCATCATTCATTTCTATCTCTTCTTCATCACTCTCTTCTTCTCCCGTATCCTCTTC
ATCAACTACATTGTCCAGCTGCGGCAAAATGTCTATTCCTTTGCTTCGATAAATTAACCCAGTCCTCATAACAT
GATGAAACTTATCCCTGTGGCGAGTCAGAGGATGCGGATTTACAAGTCTTCCACGACGGTACCCCTCTCTCAA
CGCCACAGTCGTTGTCTTGCACTTCAATGAGAGGTAAAATATTCCTGGATACCTTGTAAATAGTCGAGTAAAT
CTATGTGGAAGATTTAGTTCCTCTCTCAAGCTCCTCAAGTAATTTCTCTTTGTTTTCTTGTATAGTGTCAAGCTT
AGAAACTCATGTAAAACCCCAACTATTCTCTTTTCCATAAGATCACTATCGGGGTCAATTCCCCTTGAATCCTC
ATAAGGTGAGATGTAAGGTAGCTTCTGAAACTCATCCATCCACGCCTTGACCTTCTTCTGTGCCCCATATCCCC
TTGGGAAACTCATTGGGAATTCTAATGCAGCTTTGCCCCTTTTAAATTCTCTATACCCAGGAGGGCTGCCATCG
TCTATTGTGCCCATCTCATTCATCTTTTGCAACTCGGAGACAGCATATTCTTCTGGCCATTGAACAAGCTTTAA
GCAAGCCAATCCATTCGTTCCCTTAACTATCCTAAAATGATCAGGAAATTTCTTAATTAAATTCTTATCAAAAT
CATCGGGCAATCCT
>TCONS_00016071
TCCTCAATCACATTGTTGCTTCAAGTACTCGGAACTCAAGAAGAGAAGACCCGATAACTCCACTCATAAGAC
CCTTTGTCGCACATTCTGTATGTCGTGCTGCCAAATAGTGATCCCACTTCAACCTTGCTCCTTCAAATGAGTAT
CTGCACAGTTACAGCAGAAGGTCATTGCTGGTTCTTCCTGAACTCTGTAAGCACAGGGTAGATGTTCTCAAAA
GCAGTATATGTCTCATCCCGGACCTTTGCTCCAGTGAGGACAATCTTGCCAGACACAAAAATGAGCAGCACA
ATCTTGGGTTGCTTCATTCGGTATATCAATCCTGGAAACAATTCTGGCTCATAACTTGAAAAAGCACCATGCG
AGTACGCCAAACCTTCAAGTCTAATGGGAAATTTCACATCACAGGATCCAACTATATTCTGAATCTTGAAATC
CTTAAATTTGGCATCGAACCCAAGCTTCTGGATTATCCTTGCATACTTCCTTGCTGCAAGCTTAGATGATTGTT
CACTCTTTGCCCCGGTGCAAACCATCTTTCCAGATGCGAAAATCAAAGCTGTAGTCTTTGGTTCTCTTATCCTC
ATAATAACAGCAGCAAAACGCTTTGGATTGTATTCAGCATTACGTGCTTGCAATGCAATAGCTTTAAGATCCA
ACTTGCAATCCAAGTTGACAGTTGAGACAATATTCTGAAGAGTAGGCACAATCCCAGATGGGTGCTTAGACA
AATCCACTGGTTGGCTCCCTTCTAGACCCACTTCAGCCATTCCCCTTGTTTCGTTATCCAACGGTCTCAAAATA

CCCACAAATTTCCACTTAGTCAAGAATCTCAAAAACCAAATTCTTAAAATTACAATTTCTTTGATTCAAGTTA
AATCTTGACCCGATTGGCTCAATTAGGGTTTCCAATCAAGATCCAGAAATACAAAGATCCAACCCGAT
>TCONS_00016179
GGATCATTCAATAGCTCGGGCTGTCTACTAAATCTCAAAGCAAAGTACTAGGACACATAGGAGTGAGTAGAT
CCCGGATCAATCAACGCAAGTGCATCAAATGAACAGACAGAAAGAATACATGTAACCACTGCATTCGAGGC
CTGAGCATCCTGTCTAGTAAATGCAAAAACTCTCGCCTGACCTCTACCAGCATTGCCTTGTCCTTGATTCACA
ATAGCACGATCTCCAGCACCTCGACCTCTA
>TCONS_00016189
GCTGCTAACATGTTTAACAGTTTTCAGGACAATGAGGCTGGTTTATACTCGAACCTTTTCATTTGATGTACATT
CAGCTGCAGATGATAAATGTGTGAGTAATAAGCATAATAGCATAAATTAACCTCCCAGAGGAAAGCTGAGCT
CTTCACAGTTCTTTTTATCTCTTTAATGCTGTAACATGATCGCGGATCATCACAAAGATGGTATATTTACAAAT
GACACCATCATAGATAGTCTCGTTATGGTCATGCTAGCTATAGAGGTATAGTAGATATACTTCGTCCTATGCTT
GATATGGACATACAATAGCTTTGTACCATATCTCAGGTTATACCACCCTGTGGGGCTCAAGAAATGGAAGCG
CCATACACACGTCTTCACTCTTCAGATACTTTTATTGTTGTTGAAGATGTAGCAAAGTTGTCTATTGATGAGCA
TGATCTGAGGAGAAGGATGAGTTAGAACTATGGGTGGACCATACAAAAATCCTCCATAATGGTCTGCGCTTG
TGCTTCTGGTGCTTAGATGTCATTTCCGACTCGGTTCTTCTGAGCATTTCCCACATAACCTGAACATCCTCATA
GCCACAAGTTTGTATATCATCACGAAGCTTCAAAAGCCCTGGACCATTTTTGCGAGCTTTAACACGAGCAGA
AACAGCAAACCAAACACGCCGAACAGGAAAAACCATTTTTTGCCACCAATCATCCATTACTTTGAATTTGAT
CTTCAATTTCTTCCTTCTACTTTTACACCCACCACCTCCCAATCTACTCACCAAAATCTTTAACCCGGCCAGAC
CTTCTCTATTTTCCTTTCTTC
>TCONS_00016218
ATTCTGAAGTTTTTCAAACAGTTGGAGCATTGAGAATATATCAAGGTTGTGCAAATGATTATGTCTTTTTTAGA
AGATAGCCCAGAATGATGCCCAGTAATCCAATAATGAGAACATATATAGTTGGGATCCCACCACGACTACTG
TTACGTTCACGTCTCAAGAGCGCCAATTCTTGCTGAAGCTTATTATTTTGCTGTATAACAGAATTTTTCTCTTCT
GTCAGCTTTGAAATCAGTGCCTTTGTCTCCGACGAGCTGTCTTGCTGCTCATTATATGCCCTTGAAATATTATT
AAACTCGGATGTATTTACAGCTCCATTTTCAGACACTGAGGCCCTAGGAGAGGAACCTTCCTCGGAACCCTCC
TGCACAGGCGATGGTGGTTGTTGAGGTGGAACGTAAACGACTCTCAACTTACAATCCTCCACATGATTCCCCT
CCTCCTTATTGAACTAAAACCAAAAAGTGGATATAAACCATGCTAGTTCTCATGAATATGGCACAAAAGGCC
GCAAGTTATTCATAACAACAATTAAAGTGTTACCATCTCAGGTGTAATGTCCTTTGCAGCAGCGCCAGGGCTT
GCAACAACACTTTGAAGTAGGAACTTATCCTTGCATTGCATGTCTGCTGGAGCCTCCTTCTGTGCTTGCATTGT
AACTGTGACATCGCTGGCAGAGTGAGGCATGACAACTCCAGTGTTGGGCCTTACACAATATTTCTTTGGATTC
GTCGTCTTCACCTTGAAAGCGACATAGTTATCGGACTTGTTTGTCAATTGTATGGAGCATGAGATCTGCTTCTT
CAATTCAAAGGGAAACTGAAGCTCAAGAGGCTCGATTTGAAGTAGCTCTCCACCGCCGTTACTCATTCCGAC
GCGATTTGCTAATCGGCCGGAGAAGCGATTTTCCGGTGGCGGAAGTTGAACTTAGAAA
>TCONS_00016225
CCGAGCTTCCAAATCACTGGTTCTTGAAAGACTCAAAATTTTTGGACACATCCTCCACCTTACTAGCAAGTTC
TTGCCTCTTTTGCTGAGCCATTTTAGATAGATCCTCCATTCTCCTTGATGACCTCACTGCAAATCTTTGAAAAG
CAGGGCTGTTGGCGAGGCCATTGACGATTAATTCATTGGCGACATAAGAAATAACTCTATGAAGG
>TCONS_00016263
GCTAGCTAAGACTGGATCGTCAAAGACCGGATTCCTTTTCTTAATACCTTCCTTATCTCTCCTAAGGGTCTTGC
CCTAGGCATAACCTATATACAAAGAGTTCCAACTATCGAATCAAAGCAAAGAGGAACAAGTCGGTCTAGCTT
CTTACTCTTTCACCTTTCAAAGAAGTCGAAATAGAAAGGGGTAGGAAAGATCCCGTGTACCTTGTTCAAGTTG

CGGATCTCCTTCATTAGCTTTGAGTTCATTGTTGTTCTTCTGGTTCTCCTTTAAAAGGGGTCGTGGGGGAAAAG
CCCCAGCCCCCCTACATGCATTAAGCGACGACGGCTCAAACGAAGCAAACCATTTCTCATAAGAGAATAGA
AAATCACAAGAAAGATTTG
>TCONS_00016311
TGTATAATGACCAGCTCCTTCCCATAATGCATTTAGGAAGTTTAGCATATTACACAGTATATGTGATTTGGTCA
TCACAAGATCATCAGCTCTTTTAACATAAACCCATATGGAATCTAATATATTTGCAGCATTGCACTGCAATGC
ATCATTGTCTCCAGGTTGATTGTCACCATTGCATGATTCAGAAATTGGATTTTCCCTCAAAGCAATCACAGCG
GTAAGAAAAGAAGCCTGATAACGTGCAGCAGAAGCAAGCATCTTGAAAGTAGCAATTATGAGATCCTCACT
TTCTACCTTCTCTTGACAAAGAATGCTACAAATCGTATTTTTAAAATTATAGATCTGCTTGTCATCCAGACCAA
AATATGCATTGCTAAGCGCACATGATTGCGAGTCGTCTGCAATAATAAACAATCTGGATTGCAGTCTTGCAGC
ACCCACCTGTATCTTAGGATTTCGGAAGAATGACATCAATGATATCGCTGCGGTAACCACAGGCACTGGTTTT
GTCGTCGACGACATGATTGCCTGATGAAAGACAGTGAAATTGGGCACATCCCTTGAGAGATCAGAAAGCATA
CTGGAAAGAATATCCAATCCCAAGACAATTGCCTGTTGTAAACCTTCGATTTCTGTCAGCCCATAAAGCCGGC
TAAAATAGAGCTTCTCAAGGCCATCTGAAGTTGTACAAACAAGTCGAAATAGTGCAGTGTGGATTGAAGAAT
CACCAAGTAAAATATCCGTGACCACTTCACCAAGTTTCTGGATATTTGAAATTGACAGAATGCATTTCTTCAA
TACTTCAAGCACCTTCAGAGTCACCTTCCAGCGAGCATGCTTGACTTTATAATTCCAGAATTCATGATTAACA
AGGACATACTGAATGGAGAAAATGACAAGTGCAAGGACAACATCATTTTCCATTCCAGCATCCACAAGCTGC
ATGGTGAGGTCAAGCACTGAAAGAGTCAATTGACAATCATTCTGCTCACAGTCGATCAAAAGCATTTTTGAG
AGCCGACCAGAAAGCAACCACGATCCACTCGACAAGCCATTGGAGCCAATTCTGAAAGGATTTGTTTTAAAC
GCAACATCAAAGATATTCGCCTGGACTATCAATCTGGAAACATGATACGGTGAGCATTTTAACATTTTTGCCA
AAATGTTAACTCCCATCGACATCAACACCACATCAGAGCAATTAGGAGATAAATTCTTGATCCAAGCACAAA
TGATCTCGGCCACATTAAGACGCAAGTTTTCTGTTGGTGAGTTCATCTCATCATGCATATAGCCACCACCAAG
GTCCAGAAGAGCAGAACAAACACCCATGTTGAAAGTCACAAGTCGGCTGAGCAACCGAAGAGTCAGAAGG
ACATCACTGGTTTTCTCCAAGTACAGCCCTTGGGCCAAACGCAAAAGA
>TCONS_00016418
ATTTGGTCCGCTGCTGCTCCATTATTTTATGGTATATGTTGCCTGAGCTCCTTCTTCTAGACACTGACCTTCCAA
TGGTGGAGCAATTAGCCTACTCAGGACAATGAGTTAGCGGGTTCACATTGTTGTTCTTACTGGGCAACCAGAT
TGTGGTTAGAGAGCAGGTTTATGTGTGACGATAAGTGAAGTTGCTATATCACAAGAGAGCAAAGGAACTTGC
TTCATTATTCTTCTGTTCGATGGTCGCTCTGCT
>TCONS_00016579
TGACAAATCTCTAACAGGAGACTAGGAGAGGAGAGACCATATACCGATTAAAAAGGGAAGAAAAACACAA
TATAATAAATATACAAAACTTAGCTGCCTAGGAATATGCTACAGAGCTCTTTAGACAATGGACTATCCCCTGT
GCAGTTGCAAAAGAACTCTAAAATACATATATACAAGCATGAACTTGCTGTTCTCTGCTTCAGCGCACCTCAG
CATACAAACTACTCTCCGATTAAGTATAAACAAGCCGCACTACCAGCAGCTATAGCTTCCTGTCTTGGGTCAT
CATCTTGGCCTCCAAAAGCCAGGACCTGCACAGCAAAAGCGACTGAGTTTTGCAATAAATATTTCTTTAGAA
GAAGAAAATCTTGTTGTTTACTGCATTGAAAATAAAATTAAAAAACAAGTTAGAAGGAGAAAGGCAACCAA
CAAAAACAATAAATTGACACACCAC
>TCONS_00016613
TCACAACTTGATTGGGCTGACTGTTGCATCTAGTGACGGGCAGATAATACCGACTTTTTTTCTGCTAGGATGTT
TGACACCTAATTAAACAGTTCACTTTTTGAGATGAGGAAAACAGGCATAGAGATCAGATTTTGGATGTTTAGC
TTCAGCATGCTCCTTGCACTTCACCTCTGATGTTGTGCACATAAATGTCTGCATACACACCTTGCACTGTATAC
TCATGGCTTTTTTGTTCGTGTCGAGCTGACTTCCTTTGGAAGCTTTCGACTTCTCGAGATTCTTCTCGCGAGCCA

TCTTGGCTTTCTGACCGTTGCCTCCACCCATGATTTTGCTTGATCGGAGAGGTCGGAAAGAAAACCCTAGAAG
AGCTCTTTTTGTTTCAGCGCTAAAACGTGGCGGGGAATGGCAG
>TCONS_00016691
CACACACTCTCACCATCGCCTCTCTCTATCTCCCTATAATACTCACACTTATATTAACGGGTTGCAGATCGAAG
TTTCTGGCAGATCCGATTCAATTCTATATTCCTTCTTTCTTTGGGAGGGGGAACGGTTTGAGACAATTTCAGTA
TGTTTCGGGGAATGAATGGATGTTATGATGCCAATGTTGGTCCTAGGAAATTGAAAACTTTGTAATGAGATGT
TTTAAATTATATTGGTAACTAGCCCATATTGTAGTATTGTACTTAAAAAGAAGAAAAGTTGATGATAGCATAT
AGGAGTATCTCACTTAGGTTGTCTGTACACACAAATCACGATGCATCATCTGTCATTGCGGTATTTATTTATGT
TCCATTTTTGTTACTTGTTGTAACCACCCTTCTACATGAGATCACGATGCATCACCTGTCATTGCGGTATTTACT
TATGTTCTATTTTTGTTACATGTTGTAACCACCCTTGTACGTGGGACATCACTCTATGGTTAATGAAGTTACTCT
CCGGTGATATACATATATAGAAATTGTTAAGTGAAACTGTCTATATATGCTTTTTGTTCCCCACATTTCAAAAT
CTAATTGATCTGAGCATCGG
>TCONS_00016789
AGGAAATATGGAAAGGGTCAAAGGGACTGTCAGCATGACTTTTCTTCCATACGTGTCCGACAGATTCCCCAA
CACTGGCATTATAATCAGTGATCCCAATCCTATTATCGCATGTTGAAATCCAGTGAGATATATAGCGAGGGAG
CATTCATCTTTTCCAGGACAAATGGCAGACATGGTGATATCAGTAATGGCAGGAATCACCATGAATGCAGAG
AAGCAGTGAAGAAACACTGTCATGAATATGTGGCTCAACCCACTACTAGACAACTTTTCCATACCTTCAAATT
CTCTTGCCTACTTCTTTC
>TCONS_00016828
AGAGATTCGATCTTAATGAGAATGGACTCTGAAGCCCAGAATGAATATCAGACTTCCTCTTCAGATAGCCAT
GAGATCACCATACTGGCGCATCAAAATGATGGAACCAGCAAAGAGAGCTGAAGCTCGAAGCAGTTTCATGT
TGAGGTTCCACTTTTCTTCGTCATGAACCTGAGAGTGCCAGAAAGCTTTGATCTTGTCCACTGATATCACAGA
GTCCGCCATTGAAGCTCGTCTAGAGTGGGA
>TCONS_00016836
ACTCAACCATGCATTAAATTGTTACAGGCAAAAATCTGTTCATATAGTAAGTTGAATCCCACAATTAGGACAC
ACTCAATTGAAGTTTCTTTGGATGAGATATACCTTAAATGCATGTACACTGTTGTCATTCCTTCTTTTTCCCCCT
CAGAGACTAAGCTTATAGAATTCAACCAGATCAGAAAAATCGTTTATAAGCTCAAACCTATTTTTAAGTTCTT
GGATCCAAAATCTCGAAGCTGACTGATTAAGCAGCTGCTGTTTCCTCCTTTTTGGCAAGCTGGTTTTGAATGTG
TTGGGGAACAACATCAAAGTTGGCTAATTGCATGACATATGACGCACGGCCTTTTGTCATTCCTCGCAACGTA
CTAACATAATTGAACATTTCTGCTAATGGAACTAAAGCATCAAC
>TCONS_00016997
TTATTAACTCCAATTTCATCAGAAGCAGACCTTGGGAGCTTTCATGAAGGTGAAGAGTTTCTGAGGTGGTCAT
GCTGCTGGCCGAGGGCGAGGGTGCAGTGAAGAAGAAAAGGAAACCTAGAAGAGGGGAAGACTTCAAGTTC
GTGAAGCTGTTGTTAAAGCTGACTTCTGACTTCTGTGGGTAGTATACTTCCTTGGTGTTGGGTCTGGTGTAACT
GTTCTAAATAATTTAGCACAGATTGGAGTTGCACTTGGTGTAAATGACACAACGATATTGCTGAGTTTTTTCA
GCTTTTGCAATTTCTCTTGGGCCGTCTTGGGCAGGTGTTGTTTCTGAACACTTCGTGCGGTCAAAAACAATTCC
ACGGACATTTTGGAAGATGGTAACACAAATCATCATGATTGTAACATTCCTTCTGTATGCTTCAGCTCTGAGT
GGTACTCTCTATGCTGCCACTGCATTGCTAGGAATTTGCCTCGGTGTTCAGTTTG
>TCONS_00017034
ATGTGTACAAATCATCAATATTCTTTGAGAATTTTGGCAGTCTCGATAATGAAACGGCCTTCCTTGTACTTTTG
GGAAGGATTATAAGCTTTAACATATTTCCAACCCAACTCCTCACCACACTTGCTGCAGAATATATCAGCAACG
CTAAAGTAACCCGTCATTAGTTGTCTATCTTCCTTTTTTCCCACCACTACATTCATTGCATGCTTGAAAAGGAA

TGCTCCTCCCGATTTTGCCACAAAACGTTTAGAGAGAAGATCATCTCGAAGAGCAATCGGATTCCGGCAACT
CCTGCAGCTAAACAAAGGGTGGCTGTAGGAAGAGTCCGCCATATTAATTAGTAGTTGAACAAAACGATGATG
AGTTTAGTA
>TCONS_00017239
ACACCCTGAAAGTATCCACCAAATAATTAGTAACCTAAGGCCACTAACGGAAAACTTGGGAAGACACTAGG
GCAATCTATTCAGAAGAGAAGAACTTCACATAATAAGAAAACAGCTGCAACCGCTTCCGGACTTTTTACCAT
TATTTTTGTCATCGTTTGAAGAAACCAACCAACAATTCTCAATTCCTTCAGTTAAAGAAAAACCAAGCCACAA
GAACCACATATTTAAACAAATAACTGCCACCCG
>TCONS_00017291
GAAGAGGGTCTTCCAACAACTCAGTGGGGGTATGCGTCTCCGTACTTTGCTTCATTTTGAGCTCCTCTTCATAT
TTCTTCAATCTCTCGTTCCACATTTCCTTGTACTTGAGTTCAGCACGATGCATAGCATAGAATCCAAGTATGCC
TCCGAAAATGGTTCCTGTTATTATTCGAACATAACCCCATCTCGGCGATCTCATCGGCGTCGTCATTGATTCTA
GTCTCCTTCTCTTCCCTTTGTTCCGCGAATCCTA
>TCONS_00017365
CTATTACAGAGACCTGAAAGGGAACTCGAAAAACCAAGGATTATGTAGCAACTAAATCAGGAGTTTCAGCC
TCCACAGATAACAAAGCCGTGTGTACTTTATCCACCCAATTGTCTAGCCGATCCCTCAATGACTTGATTTGGG
GAATTCCCAAAACTCTTGGTTGCACCCAGGAAACATGAACTGTCCCTTCGACTTGATCAATTATTCCCTCAAT
GAGATGCACAGAAAGGCTCTTCATGAGTAGATATTCTACATCCTCCACATTTAACTTTGTGCGCTCTGCAATC
ACACTCAGAGGAATAGTTCTATCGTCTGCTGGGCGACTGAAGATAATTTCCATCAAGCAGAGAATGTTGACC
TTCTCGAGAAGCTTTTTCTCATTCTGCACCAATGCTGGTTGAGCACTCAAAGCAGCTTGATGGACATGGCATA
TTTGTTGGTATCGAACCAAATCACCGGTGTTAAAGGCTTCAAGAATATAATAGAGCCACTCAACCTTAGTCCC
TACCAGACTCTGTATAATCGGATGAGCAAGTAGTTCTCCAAAGTTGTATATGTTGTCTCCCAGCAATGCTGAC
AGGGACAAATCGAAAGCCAAATCCTAAACAAAATAGAAGAAACAATAACAGTTTTCAGATGCATTATAATT
AAAAGACTGAAATTGCAGAAA
>TCONS_00017486
TCCATACGCAACAAGATCAGCTCGGTCTTCAACCACAGCTTCGTTTCCATCTCCTCTATCATAACCACCAGCT
ACCATAAAAGTACCTTTGAATGCCTTCCTCATTGGTACAAGGCTTTCAGTACATTCAGTTTTCTCTCCAACTGT
TTTCATCCTCGGCTCAACCATGTGGCAGTAAGCAATGCCATACTTATTCAAGGATTCGGCCATGTAAAGTCCC
AAAGCACTTGGGTTTGAGTCTCCTGATTCCATGTAGCTTGCAAATGGGGAAAGCCGTATTCCAACTCTGTCAG
CTCCTATCTCATTTACAACTGCTTCAACTATTTCGTGTGCGAATCTGCAACGGTTCTCTAAAGACCCTCCATAA
TGGTCCGTTCGATCATTGATTTGGTCTTTCATAAATTGGTCGATTAGATAGCCATGAGCTCCATGGATCTCAAC
CCCATCAAATCCTACAAACATAAGTGAAGTTAGCTTGAGGGAGGATGTTAATTTGGCGGCAATACCAACGTG
ACAGCACATGGACAGATATTCAAAGCAAG
>TCONS_00017487
CTAGAAATGGATAGTCAGTATAACCAACAACAGGATCAGATGTATAGAATGTCTCCCTGTTATACTTGTTGAG
AGGTGCATCGAGCTCAAATCGCCTAGGTAAATCTGGATTGGCTAAGAATAGACGTCCATATGCAACAAGATC
AGCTCGGTCTTCAACCACAGCTTTGTTTCCATCTCCTCTATCATAACCACCAGCTACCATAAACGTACCTTTGA
ATGCCTTCCTCATTGGTACAAGGCTTTCAGGACATTCAACTTTTTCCCCAACTGTTTTCATCCTTGGCTCAACCA
TGTGGCAGTAAGCAATGCCATACTTATTCAAGGATTCGGCCATGTAAAGTCCCAAAGCACTTGGGTTTGAGTC
TCCTGATTCCATGTAGCTTGCAAATGGGGAAAGCCGTATTCCAACTCTGTCAGCTCCTATCTCATTTACAACTG
CTTCAACTATTTCGTGTGCGAATCTGCAACGGTTCTCTAAAGACCCTCCATAATGGTCCGTTCGATCATTGATT
TGGTCTTTCATAAATTGGTCGATTAGATAGCCATGAGCTCCATGGATCTCAACCCCATCAAATCCTACAAACA

TAAGTGAAGTTAGCTTGAGGGAGGATGTTAATTTGGCGGCAATACCAACGTGACAGCACATGGACAGATATT
CAAAGCAAG
>TCONS_00017488
TTCTAGAAATGGATAGTCAGTATAACCAACAACAGGATCAGATGTATAGAATGTCTCCCTGTTATACTTGTTG
AGAGGTGCATCGAGCTCAAATCGCCTAGGTAAATCTGGATTAGCTAAGAATACACGTCCATACGCAACAAG
ATCAGCTCGGTCTTCAACCACAGCTTCGTTTCCATCTCCTCTATCATAACCACCAGCTACCATAAAAGTACCTT
TGAATGCCTTCCTCATTGGTACAAGGCTTTCAGGACATTCAACTTTTTCCCCAACTGTTTTCATCCTTGGCTCAA
CCATGTGGCAGTAAGCAATGCCATACTTATTCAAGGATTCGGCCATGTAAAGTCCCAAAGCACTTGGGTTTGA
GTCTCCTGATTCCATGTAGCTTGCAAATGGGGAAAGCCGTATTCCAACTCTGTCAGCTCCTATCTCATTTACAA
CTGCTTCAACTATTTCGTGTGCGAATCTGCAACGGTTCTCTAAAGACCCTCCATAATGGTCCGTTCGATCATTG
ATTTGGTCTTTCATAAATTGGTCGATTAGATAGCCATGAGCTCCATGGATCTCAACCCCATCAAATCCTACAA
ACATAAGTGAAGTTAGCTTGAGGGAGGATGTTAATTTGGCGGCAATACCAACGTGACAGCACATGGACAGAT
ATTCAAAGCAAG
>TCONS_00017520
TATCTTCAAAAGAAACTGGGCAGTGGTAAGGAACCAAAATCGATAGCATCATCTTCATCAACTAGAGCGTTG
ATAAAAGATAGGACCAGACTGACAAGCACTAGAACAAAGAGGACCAAGTAGAAACCCAGCTTGAATATCC
GAATCTTCTTCTCAGACTCAATGAACCTAAAAACTTCAGTCACATCAATAAGATGCTGCCGG
>TCONS_00017639
ACCTTGTTAATCACAAGAACAATAGGAATACGTTCTTGAATCGCATGCCGAATGGCCCGTTCTGTATTTACCA
TCACTCCTTCAACAGCATCAACAATCAAAACTGCACCATCAGAAAGTCTCAAAGCAGCTGTCATCTCATCCG
AGAAGTTGACATGACCAGGAGCATCCATAATGTTGCAGAGGTAAGACTTTGAATTGCTGTCTTCCAGGACGA
GTGACATAGGGACAGACTTAATTGATATCCTCCTTTCTTGCTCATCTATTCTTGTGTCTGTATACCTCATATGCT
TCTCACTATTCTGATCAAAAGTAGATATATGGTGGGTTTGCTCCACCAGCATATCCATAAACAAAGTCATCCC
GTGATACACGTGTCCCACTAAAGCAACATTTCGAACCAATGCTGGGTTCAACATCAGACCTAAGAGAAATTG
AGTCGAAACATAGGTGGACGAATCCTTTACCCCAAGTTCAAACTTGAGATTCTTCACCGGTTTAATTATCGGC
ATTTCAAGAGGCTGTTCATCTTCATCCATCACCAATGTCTCGACCTCTTCACCGTAAACCTCTTCGGCTGTTGG
GTAGTATTTCTTGTCTTCAGCAAGGACAACTTGGTTATCCATGTCAACATCCTCTTGAGTGGCCAGCCATCCAT
TCGATGCACCAGGTTGCTCGCCATCAGATGCAGCCCTCTCATCATCACTTCTCTCAGGGAGCTCCTCATCTTCC
TCTTCCCTATCACTTTCCTGATCAGACTCAATTTCAGGACCGATATAGTTCCCAAATTCATCGTATAAACTTTC
ATCCATGGATCTTTACTACCTGATTAGAAGAAAAAATCACCCATCTTATTAATTGTCCAACTAAAGCAAACAA
AAAATCAACGGAAAAAAAACAATTTTCACCAAAACAAGCTATGCAATGGAGCATTTATGCACAGGTGAAAC
TTAG
>TCONS_00017641
CTGAGTCCTCTTGAGAAACTAGTAATAATTAATTTATTACATTTTCGCGTAGTAAACAAGTTATCTAGCAGTG
AATTTTCATATAATCTTCAATCTTTAGCTTACATAGTGGTTACATAGTTAACCAAATATGAGACTCTCATCCTG
TAAAACTGGGATCAAATGTTACAAAACTCACCTAAAATAAAAAAGGAACTTCTGGCAAAGCTAAAATGAGT
TCAACCTTTAGGCTATATAGAATCCAAAGAACGTGCTAAAAGAGGATAAAGTAAGAACTTGAACAAGTGCC
CAGTAATTCAATACGCCTGAGCCAACAGAAATCTCATACTTAATGCCAGTCGTGCGAGAATCTACCCTTTGAT
GGAGAGGAAATCACCACAGATACTCGAATGTTAGAGTAAGGGATTATCCCAACAAATAGATGAGCTCAAAG
GGAGAGTTACGCAGGAACTATTCATGCTCCTGGTATGCCAAATTTGTACTAGACTATTACATATTTCAAGAGG
CTGTCGGCTACAATATCTTTCAAGAGGCTGTCGGCTACATAACATACAAAACCACCTCAGCTATGTGATATCT
ACATCATCTGCAGGTGAAGATCAGCATCCTGCTGCGCAAGCTCCACCATCATAGCCTCATCAAAGAATTTGTT

GATGCTCACATCTTCACTCATTCCCTTTCTACGCCTTGTCTTCACCATGAACTCACGAGCCAGATGCTGAATTG
GGGCAGGCTCTAGTGGTCGTAAAACGATGCTCTTGTCAAGAGGATCTCCAGGAACAATAGCCCAATGATCAA
AAACTGAAAGACAAAACGCCTGCCCTTGGGTGTGATACCTCAAGTCGGTTTCGAAACCAAAGGACTCGATAA
CAGGTAAAAATGCCTTGACGATGTAGGCAGGGGTCCCAGGTTGAGGAACATCAGCAGTAACATGTCCACGC
CTGCGAGACAACACGGTGTATGTAGCAGAGAGGCAATCCATGGGTGTTTGGATCTCCACATAATACACAGGT
TCCATAAGCCTGGGTGTCGCCATAAGGAAAGAAGAGTAGGCTACACGTCGTGCAGTTGGAATAATCTGACCT
GTT
>TCONS_00017648
AAAGACTATAACAAAATTTAGGACAGTATAATACTCAAACCAGATCCATATGGAATTCAAAAATGTGCCCCT
TCAACGCAACCTTCTAGCCTCTCTCTCAGACTCCAACAATGTCAAAACATCACCCTCTCTGACAGGCCCCTTG
ACGTTTCTCATGATAAACCTGTTCTGGTCATCAAGAAACTTGACTCTCACCTGAGTTACCTGTCCTCTGGATCC
AGTACGACCCATCACTTTCACCACAATAGCATGCTTTGTGGCAGATTCCATCCTTGATAGGAGCAGACGAGC
GAGCACAAAGGGCAAATTGCTACGACGGCCGAAGAGGAGAAAAAATGCCT
>TCONS_00017753
CTTTTCTTCCATACGTGTCCGACAGATTCCCCAACACTGGCATTATAATCAGTGATCCCAATCCTATTATCGCA
TGTTGAAATCCAGTGAGATATATAGCGAGGGAGCATTCATCTTTTCCAGGACAAATGGCAGACATGGTGATA
TCAGTAATGGCAGGAATCACCATGAATGCAGAGAAGCAGTGAAGAAACACTGTCATGAATATGTGGCTCAA
CCCACTACTAGACAACTTTTCCATACCTTCAAATTCTCTTGCCTACTTCTTTC
>TCONS_00017755
GAGGTGATCTTATCATGAAGCTCTCAATGAAGGCTAGCAATAAAGCAAAGGCAGCACATGCTAGACCAAAT
CCTGGGAAATGAAAGGGTGCACGTTCTGATAGAAATAAAGCTGTTAAAGGACTAAAAATTAATGGGGAAAC
TACGGTCGCAAAGGAACATATGCCTGTGATGCATCCTTGAACCTTTCCCTGCTCATTTGGCCCAACCTGCTTG
GAGGCCATGCTCTTCAAACATGGCATCGCAAAAATAGACATAATAGAGATCAAAGCGCTAGCATAAGGGAC
CCATGAGGACCAAGCAATGCTATAAAGTAACATATGAACACAACTGAAAAAGAGTCCAACAGAGAGCAGC
TTCTCCTCTCCAAGAGCAGGAACTAATATGGGCATGAGAAGAAGCTGTGATATGGATCCTGCAATCCCAGAA
ATTATAAGCAAATCAGCAAATTGGTCTTTGTTGAAGTGAAACTGGGCCTTCAAG
>TCONS_00017808
TTTCTTGTGATTTTCTATTCTCTTATGAGAAATGGTTTGCTTCGTTTGAGCCGTCGTCGCTTAATGCATGTAGGG
GGGCTGGGGCTTTTCCCCCACGACCCCTTTTAAAGGAGAACCAGAAGAACAACAATGAACTCAAAGCTAATG
AAGGAGATCCGCAACTTGAACAAGGTACACGGGATCTTTCCTACCCCTTTCTATTTCGACTTCTTTGAAAGGT
GAAAGAGTAAGAAGCTAGACCGACTTG
>TCONS_00017863
TAGCCTCAAATCTTTCCGGCACCGTTACAAGGATTTTCTCAACGATCCTTGAATCGTTTAAAGTGGAACCCAA
CAATCTGACACGATTTGCTATGTTAAGAAGTCTGTCAGAATACTCCTTTATGGTTTCACTGTCTTTCATCCTTTG
CAACTCAAATTCACGCACCAAATTCAGCACTTGCATTCCTCGAACTTTTTCATCTCCTTCATATTCAGTCTTGA
GATAATCCCATACCTCTTTTGCTGACATCAGAGACATGATACGAGTGAAGATGGTGGATGAAACTGCAGCAA
ACAAGCATGCCTTTGCCTTTGACTTTCTGGTTTTCCTCTCCTTGTAATTTTTGATCTGAGCCATCGTGGGATTGTT
TGGCAAGGGAGCAATCTCGTAATCATCTTCAACAGCCTCCCAAAGATCCAACGCTTCCAAATAGGTCTGCAT
TCTAACTACCCAGATCTGATAACTTTCTCCATCAAAGGTTGGTGGTGCGATCGAAGAAAAGCTTGTTTCTCCG
TCCATGGTGTCCACAGAATCTGCTTGCACTCACACAGGCCCCTCAAGAAAAAGAGCTCTGATACCAATTGTTG
TTAACTTTAATCAACAAACCAGAAAACAAGTGAATGTTTGTGATTAATGAAAGAGCAGAATATTAAAGGAAT
GAAGAACAGAGAAGAAATCTGAAAAGAGAGAGCATAAGACTAATACAGAACAAGG

>TCONS_00018244
ATCAAATCGGAGAAGCCGTCAAAAGGAATTGGAGATTCAACTCCTTATAGATTCATCCAGTCGAGTTTGAGC
CTTCCGTTCCCAGAGCAGAAAGCCAAGTATTATAGAGAGCTGGACACTGCTGCTGATGTTGTCAAGCGAGCT
TGTCGTCTATGAGTTGATGTAATTTCTCCACTCATTGTAAATAATTAGCTAATTACTCCTTCGCTTCCGTTTATA
TGACTGCATTTCATGAGTAGACTCAACTTTAACATGTTAATTTGACTTATATGTTAGATTACTGTTAGTGAAAG
GGTTTGACACGAAGTTTAAAATTTTTATTTTGACTTATTTACTACTATGTTGATGAAAGTAGACGAAAATATTG
AAGTGAAGATAATTCTAA
>TCONS_00018363
CACAAATTGGCCTCTTAAAACCCGAACCCTAGAGTTAAAACCCTAACCCCTAAAAGTATATCTGCTACAGTC
GCTCTCCCACTCTAGACGAGCTTCAATGGCGGACTCTGTGATATCAGTGGACAAGATCAAAGCTTTCTGGCAC
TCTCAGGTTCATGACGAAGAAAAGTGGAACCTCAACATGAAACTGCTTCGAGCTTCAGCTCTCTTTGCTGGTT
CCATCATTTTGATGCGCCAGTATGGTGATCTCATGGCTATCTGAAGAGGAAGTCTGATATTCATTCTGGGCTTC
AGAGTCCATTCTCATTAAGAT
>TCONS_00018428
GGCTAACGTGGTCGCTCCGGAGCTTTATGCAACATTGGTGTCAACTCCGAATCTCCATCATCGTTCTCATTATA
GTCGTCTTCTTCCAATTCTTCATTGTGACGAAGTTCTGCAATTTTTCTCCGCATATGCCGACGCCATGCAGCTTG
TATAAAGCTGGCAGCCCACGTTCTCCATTGCTGCGAGTAAAATCTGAAAGTGTGCTGTACCTGTCGACTCTGC
AGCCGTCTGAACTGAGATGTAATGTACTTCACTTCCTCTGCTTCTAAGGCAAATGCCTCTACTTCTGTTAGTGC
CTTTACAGTCCTAGTGGATGGCGGCAAGTTTGAACCGGATTTAGGATCCAATGCCCATGTCAGCAGTTCCTCC
CCGCAGAAGTCATTTTCTTTCAGAATACCTCTGTTGAAGAAACCACTTCTTCCACCATCAGTTGTTACACTCTC
TAGGTGACCACGGATTATAAACAGCATTTCACCAACCGGGTCTCCTTCCCTAACTATGTACGTATTTTCTGTGC
ATAAACTTGGTTTCAACCGCTCACAGATTGCGTCAAGTAACCTCTCGTCCATGTTCTCAAACAAGGGCACCTG
CATGTTTCCAATGAGAAGAGCAAATAGTAGGAGTCCGGAAATGGCCAATATTATTGAAAAGAGATTTTCTAG
AGTATAGGTACTGGTTTCAAG
>TCONS_00018537
TGAACACCGAGGCAAATTCCTAGCAATGCAGTGGCAGCATAGAGAGTACCACTCAGAGCTGAAGCATACAG
AAGGAATGTTACAATCATGATGATTTGTGTTACCATCTTCCAAAATGTCCGTGGAATTGTTTTTGACCGCACGA
AGTGTTCAGAAACAACACCTGCCCAAGACGGCCCAAGAGAAATTGCAAAAGCTGAAAAAACTCAGCAATAT
CGTTGTGTCATTTACACCAAGTGCAACTCCAATCTGTGCTAAATTATTTAGAACAGTTACACCAGACCCAACA
CCAAGGAAGTATACTACCCACAGAAGTCAGAAGTCAGCTTTAACAACAGCTTCACGAACTTGAAGTCTTCCC
CTCTTCTAGGTTTCCTTTTCTT
>TCONS_00018546
GCAGATTCCTTTGTATGTGCTCCATCAAGTAGTATAGTTGCCCCAGGTACTCCAAGTAAATTAGCTTCTTCTGA
AGACAATATTTGGCTTCTGCCTTGCAAAAACGCACTCTCCAGACCAGCACGAATAGATCCACTGGACAAACT
CCATCCTTGCTTATTAAGGCACAAGGCTGAACATGTAGCTGTTACAGCATTTTGAAGTTGGTGAGCTCCAAGC
ATGCGTAATTTCACACCTAGAAGTTCAATCGACAGATCAAAGTCCTTCACAATCTGGAGCACTATATCACAC
AATTGGCGAGGTATACCACTCACGTCACAGAAGCCTCTCAAAGCGCTTCTGTTTCCAGGATCAGATGCTGATA
CCACTGGTGAAGACATAGAAAATGCTTTCCCACGAAGAATGCGCTCAATATATGGAATGAATGGACCTCCTA
GAACAAGCTAACTGGGCGCCCATGTTTAACTATTCCTGATTTTGCCACCGCTATACTTTCCAAAGAGCCTCCA
AGTGCTTCCAGATGTTCCTCACCAACAGTAGTTATGATTGATAAAGCAAGATCAGAAGCTGAAATAACATTA
GTGGCATCTCGTGCTCCGCCCAATCCAGCCTCCACAACTGCAATTTCCGTATTCTCTTCAGCAAATAGGCTGA
ATGCTATAGCAGTCAAAACCTCAAAATGACTGAGACATCCATTCTCAAGTTTCACTGCCCTCTCAAGAACCTC

CCTCCTCCTCTTGAAATGATGATTCAACGCCTTGGCTGATATTGGCTCGCCCCATCTTCCCAACGTTATGCGTT
CCCTAATAGTCTGTATATGCGGACTAGTATAGCAACCAACAGAATAGCCTTCTGCCCGCAATATACTGGATA
GGAATGCAGCAGTTGATCCTTTTCCTTTTGTTCCAGCAATATGAACAGTCTTGAACCTAGATTGGGGATTACCG
AGGAGCCCCACCAAGCGACTCATTCTACCTAGATCGAAACCATATTCGGAATCTGTGCCAGCGCCTATTGGA
ACCCCTGACTTCTCGAAGTTGTTCAGATTGTTCAAAAATTCCATTAGTTCGCCTAGTTCTGGCTCCTCTCGCAA
AGTGGAAAAGCCATGACGGGTATTCTGGACAAGTCCAGCAGCTGAGGAGAACATACTTCTCTGAAATGAAC
GACGGAGAATGCTAACATTTTTCATTTTCTTATTCTCACACTACACTGAAGAAACCATCGGCGGTTCTGCT
>TCONS_00018553
AACATTTCTGAGACTATGTACGTCTTAAGAGACCTTAGTGAAATCACTACTAGGCTGGCTATACTACAAAAAT
AGAAACTAAGGTGGAAAGTAATGACTGCCCAAGGTAAAACTATCTCGGCCAGTACAAGTAGGACAAAGGAT
GAGCATAGCTGCAGGAGGCCGGTCTGTCATACACTGCTGCATTGCCAAGCCATCCATATAGCTGACCAGTAT
GACTCCCTATTTGCCCGGGTGTACTATAAACTCTTTCCTGTTGTAGCCATGGATTTGGAACATGTGAAAGTTCT
GAAGGATCAATATCATGAAGTTCCAAACATTTGATGACAGATCTCAGATCAAACAAGTGCCTCCTTTTTGCTT
GAT
>TCONS_00018648
GACTTCACAAGCTAATGGAGTACCTTTATTTAGTGTATTTTTTCTTCAAAAAAATCCTAATAGCAATCTACTCA
ATTGAAAATTCATACAGCATGTTGTGAATGTACTTGTCACCAGGCTGAACAATAACAGAGGGGAAGTTTGGT
GTGTTCACAGCATTTGGAAACCCTTGTGTCTCTAAACAGACTCCTGCATGCTTGTTATACACAGCTCCACCTTT
ACCGACAACTCCATTCACATAGTTTGCAGTGTAAAACTGCATGCCAGGAGCATTGGTCCATAAATTGAGAAC
TCTGGAGCTCTTAGGATCCTTGATTTTTGCAGCATGCTTCAAGCCGTCTTTCACGTCTCCACAGTCGAGCACGT
AATTATGGTCATACCCTAAGCCAACTTGCTCGATATCACGACCGATCTGCTTTTCAGAGGTGAAATCAAATGG
AGTGCCTTTCACAGGCATTATTTCTCCGGTTGGGATTATGTTCTGATCAACGGGAGTAATATGATTTGCCCATA
TCTGGGCTGTGTTATCAAGAATATCTCCAGAATTATGGCCACCAAGGTTCCAATAGGTGTGCTGTGCTAAACT
AACAGGAGTAGGCTTATTTGCAGGCACAGCTTCCATGTCAAGTCTGAGTGTCGTCTTAGAGGTAAGTGTGTAA
GTTGCGGTCACAGAAAGATCACCTGGGTAACCTTCTTCTTCATCACGACTATGATACTTAAAGGTAATTGATG
GATGTTCGCCCTCCTTGTGTTCAGCAACCTCCCACACAACCTTGTCAAAACCTTTATGCCCACCATGGAGGCT
GTTTGGTGGCTTATTGATGGGTAAAGTGTAATCAACTCCATTCAGAGAGAACTTTCCTTCTTTAATCCTATTTG
CCACTCGACCCACAATGCAACCAAAGAACGGAGCTAAACCTTTCTGG
>TCONS_00018662
TAGGTGCCAATATGAGGACCAGTGGTTGAAACAGGTGGACCATAAGAGTATCTCCACTCTCCACAAGCTAAG
CACCGGTCAACCTCGTGGCTATTATGCAGCGTGCAAAACTTACAAGACCAGACTTTTGCTGCATTTGCTTCAT
TTCCTCTATCCTTTGGGGTTCCACAAGCTCCGCATAGAAGCGCTAGCGGCTGATTCAATAAAGTACACGTACA
ACATTCCCACATTATCTGAGGATCGGTTCCTAGAACTGAAATATGATCAGGCTTTGGTCCTACTGAAGTTTTA
GAGCTTTCAGAGGGTCCCTCGCTCTCTACCATTTTGGAGCCGCACCACAGATCATCGTGTAACCTCCTTTCTGC
AGCCATAGCGGCAGCCTGAATTGGGGTGAGTGCAGCCTTGATGCTACTATCACCCCCCAGACGCTTAGGCCC
CGATGGCAAAAGTGCTCCAACTCGTGATCTATTTTCTGCAGCAGCCAGTGCTTTCTGCCGCAATGATGACAAT
GGAGGTTGTCGAGAAAACCCGCCCAATCGCTTTCCGGGGAGATCAAATCCTTGTCCAGTACACAACCTTCCT
GATTTCGTCCAATAGGTTATAAAAATCAGCATTATGCGGGCCATATTCATTGTGGCAGAGTTCATGAAGCATG
GTGTGAAGAATTTGAGTATAAGGGAAGAAGTCCAACTCATTGTTTGGTCGCCGCAATCTAAGCTTAACCTCTG
CACCTCCTCCTATGTTGAGTCCTAAAAGTGATGGATTGGCAGGACAAAATTCAGAGAGCAACTTGACTTTCCA
TTTCCGTTTGCGCATTATAGGTTGCACTTGCTTCGCTACCTTTTCAAGAATTTCTCTTGCCTCATCATCACGCAC

CTTCTTCAAAGGTTTGACTTCCCAGACCTTGTTCAGATCATTAAGATCCATGTGGCTGGTGGAAAACCAAAAC
CAAGCTGA
>TCONS_00018831
GTTAGGATTCGCGGAACAAAGGGAAGAGAAGGAGACTAGAATCAATGACGACGCCGATGAGATCGCCGAG
ATGGGGTTATGTTCGAATAATAACAGGAACCATTTTCGGAGGCATACTTGGATTCTATGCTATGCATCGTGCT
GAACTCAAGAAATGTGGAACGAGAGATTGAAGAAATATGAAGAGGAGCTCAAAATGAAGCAAAGTACGGA
GACGCATACCCCCACTGAGTTGT
>TCONS_00018860
AATTCTATTGATATACAATTTGATGCCACTACAACTCACCTCAGTATAAGCTTGTCCCAATCCTGAAAACCAG
CATTCATCAAATTCTCCACTGTAACAATTCTGTGTCTTTCACTACGCCCAGTAAGCAAGAACACTTTGAATCCT
AGCCTCATAACATCTTGATAAAACTTCAAACTTGACCCAATGGCTGGTGTCACTCCCTTCTCAACCCATTCATC
AAATTTCACACTATCAAATACCTCCAACCCATAACCATGTTGCGAATAATAGGGAAGATTTGAAAGCAAAGT
CTCATCAACATCAAAATCCCACACATCTTTTCCATCTGCGCCCAATTTCATGCTTTCTGCATACGCTCCAGCTT
CACTTGAAACTCTATCAATGTCCAACTTATAAGCCCCGCCGTTAATATATTGCCTCACGTAATCAGCACATTC
CTTTGGAATAGTCTTCCATGGGCTCAAATTATTAGCCTCCACAGCAAACCTCCAACTTGTACACTCTAATTCA
ACATCAGTAATTGTTTCCTTCAATTGGGGTT
>TCONS_00018866
CAAAAACTGGATCCTCTGCCAACAAAAGGGGTGTGTCTAGGTCGACGAATTTGAAACATCCAAGTCCAGCAG
CTAGATGACCAGCAAAGCCCATGGCAAGTCTGGTTTCAACCATACCACCAATCATTAAATTTAAGCCCATGG
ACCTCGCCAAGTCAATAATTTCCAGAGCCCCAAGCACCCCCACTTTGGCAAGCTTAATATTAATGACATCAG
CAAGACTTTCTTGCACTATCTTTTTAGCATCAGCCAAACTTCGACAGCTTTCATCAGCAGCAACAGATACCCC
ATATTTATCCTTTGCGATCTGACTAACATGTCCAAGACCTTCCCAATTGTC
>TCONS_00019035
TATGACTTTTCTTTTCGATGCTTGACTTGCTCAAAACAATAACTCCGAGAACAATCAGTGCAGCACCTGCAAA
CCACTGAATTGGTAGTGGCTCCTGGAAAAGTAAAAATCCAGCCAATCCAGATGATAAAAAGTTGGTTGCAAA
GTTTGTCACAGTAGCTTGTAGAGATGATAGAGCTTTAAGGCTATTGACATAGCATCCCCACATTGTGACATTG
AATATTACGACACAACCATATTTGATGAGCTGGGAGGAGAAGAATTTGGCAGAGATGGCAGCAAGAGCAGC
GTTGAATCCAGCTGCGACTGCCCACACATAGCC
>TCONS_00019247
GGATTTACTTGTCTATATATTGTTCCATTCGATCTTTTAGGTCCCGACTTCACCTCGATGGTTAGGCCACCACGC
CCTTAAAGTCTATACGCGATAGATAGACTCCTAGAACCATGACATATTTGCTTACTTGAACATAATTTCTTTCC
ACGAAAAGAAAGGAAATGGTTCATTCCACAAAATAAAAAGCTTTTTTTACGAGGTACAAATAGAAATTCCTC
TTTATTTGATTTGTTACGAAATCGACCATAGATCAA
>TCONS_00019264
CAAAATCGAAACGCCAACCATCAAATCCAATTTCAGTTTTTAGCCAATTCATCCAGTCTGATAACTCTTTTTG
GACTTGAGGGTTAAGATGATCGATATCAGGTGCAGCTGCAAAGTCTAAACCGGTGTCATCATTCCCCGTGCC
ATCAGAATATTGCGTGTCGCCTTTGCAAATCATATGCGGACCCCAATCAAGACGATCATCAGACGTTCCGCCT
TCAAAGATGCAGTATATTCCCCTGCTATCTTGCTTATCGGCACATCTGTGGTTTATTACTATATCAGCAACAGC
TTTTATTCCCTTGAGGTGAAACTGAGTTAGATGATGGTGGTAACCAAACATGAGTAACTCCAGCTTTTGCCAA
ATCTGGAACTAAATTGATAAGAGAGTTGTACCATCCACCTTGCTGATTACTTGATTCCCAATTGAACCCCTAA
AATTCATTACAAAAAAAGAAACAAATGAACA
>TCONS_00019265

AAATAAAGATAGAGGCTGCATTTGATATGGATCTTTTGATATGGATCTATAGATCAATCTACAATATTATCTT
GATATATGTGAATATCAATTCCATATATGGGCTTGACATAGCATCCAATTCCATTTATTTGCTATATCTATTTGT
TCTGATCAATCTGAATTACTCTTATGTCTTATAGTTTGAATTACTAGATTTTTGATTTCCAATCTGAATCTCGGT
ATCTATTGAAAGAATCAAATCAATGAAGGAAAAGCAATAGATATAGGCAGATTCAGAAAATCAGGTAGTAA
TCTTTTTTTTTAACATTCCCAAGAAGTAATTGAGTAAACCATTGACAGTAGTTCCACGGGTATCGAAAAGGAA
GAGTAAACCTCACCGATTTTTAACGCCTGAACCATGATATGAAATAAGTCGATAAAGCATAAATCCAATTTC
AAAAATTCGAAAGCGGTAAATGCGCAATATGTGACTCACCTGAAGATCTTATTCTGCATAGTATCTCGTTAGA
CAACCACTATGTTTATATCCTTTCTTTCTCATACAAAGTGCGTATACACTTAACAAAACCACCTCTTCTTTGAA
CAGTAAAGAGAGAAACAAATAAAAAAGGGGTCCGGCCCTCCTCAGTATCACCTGGTAAGAGGCCGTTGATT
GGAATAGAAAATTTCGGAAGAATTAGGTTCGAATAAATTTCCTGGGATCCTCTTCCTTTCGAACTACAAACAT
GGGAATCGTTGAAATAGCTCTTTGATTGAAAGAGGATGATTCCTATAATACATTACTCCAGTCGAGTCCAATG
GAATTTCAAAATGAAGATATTTTTATTTTGTTCAAAACGTGTTGCAGAACGATCTAGAAAGCAATTGATATTG
ATACAGTTTGCTTCCTTAACTCAATTAGTAGGACCCCTAGAGTCCACTTCTTCCCCACACTACGAGTGAAAGG
GAAAAAGTAAAGACTACCATTAAAGCAGCCCAAGCAAGACTTACTATATCCATATGAATTATGTCCCCTATC
TCTATAAATATAAAGGAATTGTTCCATTATTCCTCACTAATAATAGTGGAATCAATGGCGCAGAGTCAAAAA
GAACGAAGACTATTAATAAACCAATCCAATAAATTCGCTCATTTTATAAGAAATTCCTATATGATTTCTAATC
GGGAAAGATTAAACACAAGCAAAATCTGCAGTAACATCTCAAGGGAATGTTACTAATGGAACATGTAGGAA
TAATAGGTCCTATTCTTTTTTTGTTGACCCTCATTTCAATTGATTGGATGCTTGAGCACTCAGAGATTTTC
>TCONS_00019289
CCTCGTCTGAGCTACGCATGCTTAAAGATCTTCATGCTGAGCCAATGGAAATATATTTTGACAACAAGGATGG
GAAGTGTTAAAGGAACCAGAAAGCAATAGTATCTGTCATTTTGGATGGCTGCAAGGATGGGGTTATCAAATG
GCGGAAGTAACTTGGACACAATAGCAGCATAAACAAACAGCAAAAAGGAAATGGACCCAAATGCAAGAAA
TGCCAATCCCTGAGCTTTCGAACCATACTTGTATATAGAACACAATGCCATCTGGTACAATATGATTATCTTGT
CAATACTTGAAAATTCTCAGGCTGGATATAACATAATACACCAGAAAGCGTTAAAATTAGGTCACCAGCTGT
TACCTGAGCAGAATCATCTCTTGCCTTTTCC
>TCONS_00019293
TAGCTACCAGCTTCTTTGACATTTTTGTTCACTTCTCTTCTTCCTGCTTCTCCATAAATGTTTTCCTCAAAAAATC
CCATGGCTGAATGACCCAGAGAACGCCGGTACCGGCGGCGACGCCCCACATGGCGGCGGCCTGGATATCGG
TAGACTGAGGGCGGAATTTGGGGCGGAGTAACTTGAAGAGTGTGTTTCCTGCTGCAGCTGGGCTTGTCATCTT
TGGCGTTCTCAAAATTATGAAGACGCTAGGTTTTGCTTTTCTAAACGCTGTAATAATGGAACTGAAAATGATA
CCAAATCCTAGAAAAGCAGAGCCCCACAATTTAATGTCAAAGTTGC
>TCONS_00019321
TTAGGATAGTTGTAGTTGGGGTAGTGTCTCTTCAGCATGTCAACCAAATCCTGTGATTTCGCTTTATGAGCCAT
GCATATGTGTCTACCTTCAGCTTCAGGTTTCTCATAAACCAGCACCAGCGCTTCAGCTAAATCACGTACATCT
ATAATAAACCGGCTCTTGTTTTCCAGCTCTTCATATCCTTCTTTCAGCAGCTTAATGAGAACCAAACTACTGGC
ATTTGTAGTCCTCTGCAACATTGGCCCCAAAACAAGTGTTGGAAGCACAGTTATCATATCAAGTCCAGTTTGT
TTTGCATATGACCAAGCTTC
>TCONS_00019432
TGAACCGAAAGGTTGACTATTTGGTACACGCTTTAAGTGACTACCCTCTCTAGTAGTTGTTCCTGGAGGAATTC
CCGTTTGTTGCACATTTTGAGAGTTTTGCTTGGGATGATAATATTTGCAGGAGTCCCCATACTGGCAATACCCC
GTACGAACAAAGTGATTGCAGACTCCTTTACCAAACGAATCAGGATCGTTATATAACTGAGGATTGCGCAAA

GAGTCGTACCAAAGAGCTTTAGCTCTCTGATGTTGAACGCCTTGAAGATGTCGCCTCCGAGCCGCTGCAGTAT
CCTGGAATTCCTTATCGCAATAATCGCAGTAGTATTTCCCCAACGGCATTTTTA
>TCONS_00019433
CAGACTTATCCCCAGTCAACAAAAGGGAGAGGCGGATAACCACCCTCTGGCGGAGGCCTTAGAGAGGGAGG
CAGATTGCCCAGCTTTGCTCCGGCATTTTCTCTGAACACATCACCTGCTGGAGGAATTCCCGTTTGTTGCACAT
TTTGAGAGTTTTGCTTGGGATGATAATATTTGCAGGAGTCCCCATACTGGCAATACCCCGTACGAACAAAGTG
ATTGCAGACTCCTTTACCAAACGAATCAGGATCGTTATATAACTGAGGATTGCGCAAAGAGTCGTACCAAAG
AGCTTTAGCTCTCTGATGTTGAACGCCTTGAAGATGTCGCCTCCGAGCCGCTGCAGTATCCTGGAATTCCTTAT
CGCAATAATCGCAGTAGTATTTCCCCAACGGCATTTTTA
>TCONS_00019434
AGGTGGCAGACTTATCCCCAGTCAACAAAAGGGAGAGGCGGATAACCACCCTCTGGCGGAGGCCTTAGAGA
GGGAGGCAGATTGCCCAGCTTTGCTCCGGCATTTTCTCTGAACACATCACCTGCTGGAGGAATTCCCGTTTGTT
GCACATTTTGAGAGTTTTGCTTGGGATGATAATATTTGCAGGAGTCCCCATACGTACGAACAAAGTGATTGCA
GACTCCTTTACCAAACGAATCAGGATCGTTATATAACTGAGGATTGCGCAAAGAGTCGTACCAAAGAGCTTT
AGCTCTCTGATGTTGAACGCCTTGAAGATGTCGCCTCCGAGCCGCTGCAGTATCCTGGAATTCCTTATCGCAA
TAATCGCAGTAGTATTTCCCCAACGGCATTTTTA
>TCONS_00019587
GTTGTTTTGTCAGTGCTCTTCACTGTTCTATACATCTGTAAGTGACTTTTCACATGAGCTAATGTTAGATCCTTC
ACATTCATCAACTCTAGCACCGATTTTGGTGTTGCTCGTTCATGTCCGCCTAGAAGCTGAACAGCATGAACAA
AATGAGCATGAAGAGTTGAAGTCCATCTCATTCTAGGAGCTCTAACGCTTCTTTTCACCCCACTTATCATTCTT
GAACTTCTCTTGAAATCGCGGCCGTATATTTGAGGTTGATATTGCTGGTGATGCTGATGCTGATGATGATATTG
AAGGTTTCTATTTGGTAGTTGCCCAAACCCTAGACTTAGGGTCGGCTC
>TCONS_00019681
TTGATCTATGGTCGATTTCGTAACAAATCAAATAAAGAGGAATTTCTATTTGTACCTCGTAAAAAAAGCTTTTT
ATTTTGTGGAATGAACCATTTCCTTTCTTTTCGTGGAAAGAAATTATGTTCAAGTAAGCAAATATGTCATGGTT
CTAGGAGTCTATCTATCGCGTATAGACTTTAAGGGCGTGGTGGCCTAACCATCGAGGTGAAGTCGGGACCTA
AAAGATCGAATGGAACAATATATAGACAAGTAAATCCCTTATGGGTTCCAAG
>TCONS_00019774
GAATGTGATCTCAATCTCTCCACCTGTGGCCACAATTCGGGTTGCAGCAAACAAAGAAGAGTGTCATACCTTC
TTCTCCCCTTGCAGTTGCCTGGAAGAAAACTGCTTCTCCATGACCACATTGAGAACATCGAACAGATTTTGTA
CGAGGCAAAGTTGGATCTGCTGCTACATCCTGCAACACTTGTGTGCGTTCCGCAGCAGAATGATGTATCTCAT
TTCTATACACGCAATTGTTTTCAGCAGGCTCCTGATGGTCACAATTACGGCAAGCGTAAAGGAGAATCTTCTG
CTCCTTATCTTCTTTAGGATACAAAATATTGTTACATTCTCGGCAAAATTTCATAGTACTCATTTTCGCGAGTTT
TTGTTTTGAATCGTTTAGCTTTTAGAGGGAAAATAGCGATTTGTTCTATACTCTGC
>TCONS_00019892
TTTTTCTCGACTTACATATTCACTGCTTATCAATTTTGGTTCTCTGCTAACTGGTCTTCGCTTTTAGCTTTCTCTA
CTAATTTAACTATGAATATTCAGACAGCAGCCTGATGCTTGCCTCTGCTGTTTCTCTCCAGAAGGTGATGCATC
TTCAAAATGTGAAGATCCTCAGGATGTCTTGTGACAGGAAGATGGTTGTAGTGGAATGGGCCTTCCTATTCTG
CACTGATTTCTTTTATTTACTTGTTTGCCTCATAGATAGTTGTATTAAATGAAAC
>TCONS_00020070
CTTACGTACGTCAATATCTTCAGGCAGATCATCCAGTGCCATCTCATATAAAACATCCCAGTCTGGTGTCATC
ATGAGAATTTGTCCAAAACCAGTAATTACACCAAGAAGATCTCCATCTGGACTAGGTGATACACACTTTACTC

CACCCTCCACTCGGCCAACAATTTCTGTCGTATTATCATCCGCAGTATATAGCAATAGAAGACCATATGAAGT
CCCAATAATCAATGCTTCTTTTTCCATTAGATAATCCATGGAAGTAATGAAATCCCCAGGCTCTAAGTCAATA
AGGTTATCTGATATGGAATTCCAAGCTCCTGCATTCTGCTACAGCACCAAGATGAGGAGAAGGCAGAAGTAA
ACTGTATATGAAGTTGGAAGAGGAAGCTAAGAAGAGGCGATTGTGCTCCACGTCAAATGCTGCGAAGCTGAT
AACTTCATCCTCGGAATTTAACTGAATCTTCGACAATTGCTCCTTCAATATCTTCAGATTCTTCATCTTCTATC
>TCONS_00020100
AAAAGAAACACTAACAAAAATTTCTTCAATCACCACTTATCTAAATAATAGTCAAAGCAATTGGTCACGAAT
TCCTAAATTAGTTATTGAATTGTTGCCTTCAATTCCAGTGCTATCTTCCTCACACTTTTGAATGGGATACGTAGC
AAGAATGAGAGATCAATTCCACTATTCACATCCTCTACAAATCTACAAACACGATATCACTGGTTTTTCCTTTT
GTTTGGTGTAAAGGACGGCTTCCTACAGTCTAATATCTTGTAGAGCTCATTAAATACAACCTATACAAATGAA
AGTGTAGCAAGATATGAAATAAAAGCAAAACATCAAAGCAAGAGAATTTCAATTGGGCACGATAAGGATAC
CAATTAAGCGCCATCTTTTTTAAGTGGATAAATTACAAATTAGACATCATAAA
>TCONS_00020223
GGGGGAATCAGACTAGGCTGTCACTGTTCTAGAAGAGGAAGCGTAGCTGGCACGTTGAACCATGTCTGTCAA
CGGTGAAAGATAGCCCTAAAGTGAGGGCCCCGGAGATAGTATTTCTCAACTGTCGATCGACGTGACAATCAT
GCCTGCTTGACAAAGCCTTTTTCATTCAGTCTCCCAATAAACGAAAAAGGATTTCTGGTGACACGAAAGCTGT
GCTCGACGCTCTCTTTCCTATTCCACTGCGCTAAGTGGTCTTTCCCTTTCTTGCTGGATGACTACTATGGAAAA
AGATGACGACTACTCCCAGTCTCTGGTCAATCCATGCTGATGCTAGAAGAGCTATTCCCGCCGCCCACCCGA
AAGAGACGAGGAAGACTAACCGCCATCCTGCTCGAACCTTTGCTGCCTGAGTTATATAGCTGACTGCGAGAA
GGAAGGTTCAAGCTATGGCTGCCCCATGGGATGCTCATTTGAAACCTTTGTCCGGTCAATGCGAGAAAGAGA
GAAGTGATGTAAACATAAGAATGGAAGGTTTGCTTGTCAGCTGTCGGGCCAATGAGGCTCTTCTAGCTTTACC
TACACGGCAAGGGAAAGATCAAAGGCGAGGTGGGAAATGCCCCGCACGAACTATCTGATCAAGTTTTCTTTT
TCCTATCACAGGCAGCTGGCAAAGCCGTGTTTGATCGTCGCGTACATGCTCCGTTACAGCCTCGTTCATGCGC
CAATCAATTGACAGTGAAGCAGAGAAGGAAGAGTTTTGATCACCGTGTGGGTAGTCTCTATTAGGCTTTCCTT
TCTTTCGGGGCCATTTCCTTCGCCCTTTCTTCTTAGTCTGAGGCATCTCCCTCTCGTCCTATTGTGGTAAGTTAAT
CGTAAAGTCTTGGAAGTTAGGGTTTTGCAATCATTCCAATCCCTCTAGTCTTGGTAGTTTCATTAAGCCATTCT
GCTATCATTCTAGTTGTTAGTGAAGTGACTGCCTGATGTTCGATCGTGTAACTGGCACCGCAGCCAATGAATG
TCCTCAACTCGGAGAAAGGGAATAAG
>TCONS_00020336
GAAAAACATGGTTGAGAAACTAAACATTTTGTGCAACATCATACACTATGTTGTTGAATGAGGGGTTTCTTTT
TCCATCTCCATATAAAATGTAGACATGAGGGACAGGGTAAGCCAACAAGCCTATTGCAGCCTCAAACACATC
AATGCAATATCCTTTCACTCCAGAAGGTCCTTTGTCTTTATTCACAAACTCTTCAAAAGTAACACGGAAAGGA
ACGGCAATACGCAGGGGTTTGCCATTATTGGGGAACACCCATCCTCGAGGCCGTTTTATAGTTTCTCCTGGCC
ATATAGCATTATATAGATGTTGGTTACTTGTTGAAGTATTTGCAGGCATTGTGTATAAAACCTCTGGAGTTATA
ACAGAGAGACCAGAATAGTTTGACCAGTAACCTATCGTGCGTAATCCAGTGCCACCTATATTAAGAACATCA
TATGCTGAATGAATCAAATTCTTCTGAGAATCAAACTGAATCTGACCAGTTAAACCAGTGAAGTTCAGGGCA
GTAAGTATCTCAAGTAACTTGTGCCCTTGGTCG
>TCONS_00020386
ATATATATTACAGGAAGAAGATCTATATGAAATTGACATTCCATTGAGATTTACATCTGCTGCTAGCACTAGA
ATTCATGGGCTGGCTTGCTGGTTTGATGTACTTTTTAATGGAAGCACTGTACCGAGGTGCTGACCACGGCTCCC
GGTGCACCTACAACTCACTGGTATCAGCTTCGGTGCGTGTTGGCACAGCCACTTTATGTCATGCCAGGACAGG
AGATAAATGGCAGCCTTCGCCTGGTTGCCCATAAGGCACAAAGTTACACAATTTATCTAACATTGTCTGCTAC

TGTTGGAGATGTGCTCCAAACATCATCCGGAAAACTTGATCTGAAGGAACCATATTACCGGATGTCCCAACC
ACA
>TCONS_00020411
AATGATCCGAGATGCATACAGTAAACCCAACGTGAGAAATGATGGTTCTTTTTCTTGACGTAGGACAATACT
CTCCAAGCAATCCAATGGATCCCAAATAGAAGATGGCTTGTTCGGACATAAAAGAAGTTTCACTTAGACCAC
TACAACTCTGAAACTCCACAAAAATCACACTACCTGCAACCTTATAGATTTGAAACGAGGAGGAACAAATCT
GCAATAGAATGAAAGAGGAGAGAAACTGGAACACGTAAAATAGGGTTTAATATAGAGGAAAGGGAACTGA
GGAGGAGGACAGGCAGCCCTAGAAGATTGTTTATATAGGGTTGGGAAGGGTATTATACCAGATACTTCTCTG
>TCONS_00020473
AGGAAGAACTGAAATCCATTGAAGGACTTCAACCCGCTTCTCGATCCTGTAAAGAGGTGACAGATTTTGTGTT
GGAACATTCAGATCCTCTTATACCAACAATAAAGAAGAGTCGTAGATCCTGTTGCTTTTGGAAACGGCTCTGT
GGCTCGTCATGTTTCAATATCTCATGGATGTGCTGCTGGTGCCGGTGTTCTCATATAAAGATGCCAGACTGCTG
TAAATGTAATATATGTAACTGCTGCTCATCTGTCAGTTGCTGTTGCTCAATACCTAAGTGTCAATGTTTCTCGT
GTCCGCCGTCTAATTGCTGCAGCAAGCCTGTCTGCTGCAAATGGAGTTGCTGTTGCAGCTGTAGCTTTAAATG
GCCTTCTTGCTTAAGCTGCTCTTGTAGCCCATGTACATGTTCTTACCCTAAATGTCCAAAGTTAAATTGTAGTTC
TTGTTGTAAAAAGTCATGTTTCTGCCCTTGCTACCTTTGTTGCTAGTTGTACTATTAAATCATGCAATAATTCCT
ATATAAAAG
>TCONS_00020477
CTAATGCAGTGGAAGTGGAACGCATGGTTGCAAACTCCCCAAGCCACTGTGCATTCCTCACTTGTTGCACTAG
CTTGATTAGCTTGACATTCAATACAGAGATCCATGATGTGGTTCCTACAGATAGCACAGTTATCGACCACAAT
ATCCCAAGCCCATAGAGAGACAGCGTTCCACTTTTTTATCTCAAAACGCTTGGCCTTCTTAGTGGAAGATGAT
GATGAAGCTCCGGCACCGGTAGAACCCTCGCCGGCCGGAACCATAGTCACGTCGGTGTCGACAGATGCCATT
GAACAGAAGCTATAACACGCTCCTTTGGAATTCAATATACA
>TCONS_00020516
TACAACAAAGAAACAAAGAGTTCTCATAACTTAGTCTCTTTCCTATCGAACAAAGAAATTATGCCTAATAGA
TACACATAAAGGACTTAAAATCAGCTGTTTTTTGGGCTTCCCAAATTCTTTACTCCTGTTCTTACTGCTTCTAGA
TTCCTCTTCCCGCATTAATCAGTAGTCATCGGTTGGCTTATGCAGCACCTAAAACGGGCCTTTCTGACAGTTGT
CATGATCTCTGTCTCTGCGACCTCGAGCATTCTGACCACTTGAGAGAATGGTGGCCTATTATCAGGGTTAGCA
TCCCAGCAGCGGGTCATGATCTCACTTAGGACAGGCAAACAATCATTGGGGATTGCTGGACGAACACCTTTG
TTGACAACAGCAAAAGCTGCCTGCACAGCAGTCATTTTCTGGAAGGGAAGCATCCCTGTTATGAGTTCCCAC
AGAACGATGCCAAAACTATAAACATCGACTTTGTGGGTGTAAGCTCGATGCTGGATCATCTCCGGAGCCATC
CAGCGGTATGTTCCAGTCTCTGGTGTCATTCCTTCAGTCTGCACTTCAATACGAGCAACCCCAAAATCCGCAA
TCTTGATCGACTTGTCCGCAGCAATCAGTAGATTGTCAGATTTCAGGTCACGATGTATCAGATTCAGGCCATG
TACATATTCCATCCCCCTTGCCACGTCTAATGCCTGCTTCACCGCTAACTTCAAGGGCACTGATCGATTTTGTC
TCCTGGCCAGAAACTGACGCACAGATCCCCCTTTTGCATATTCAGTCACAATACACCAGACCA
>TCONS_00020526
AAGTCATGTGCGGTAGCAATACCAAACCAAATACGACGAGTAGTGGGGTCCTGAGCTAAGCCTTGGCTAAAC
CTTGGAAATCGTAATGCCATAATACTTTTCAAATCCTCCTAGCCATTATCCTACTGCAATAATTCTTGCTAAGA
AGAATGCCCATGTTGTGGCAATTCCACCCAGAAGGTAATGGGTTACTCTTACAGCACGTCCTTGTATAATGCT
CAAGGCTCTCGGCTGAGTAGCAGGAGCAACTTTTAATTTATCATGAGCCCAAACGATGGATTCAATAAGTTCT
TGCCAATAACCACGTCCACTGAATAGAAACATTAAACTAAAAGCCCAGACAAAATGAGTACCTAGGAAAAA
AAGGCCATATGCAGATAATGAAGAACCATAAGACTGAATTACCTGGGATGCCTGTGCCCATAAGAAATCGC

GGAGCCACCCATTAATAGTAATAGAACTTTGCGCAAAGTTTCTTACACTGCCCCAAACATCTGACTGCATTTT
CCAACTGAAATGGAATATTACTACCGAAATTGCATTGTACATCCAGAATAGTCCTAAGAAGACATGATCCCA
GGCCGATACTTGACATGTACCCCCTCTTCCAGGTCCATCACAAGGAAAACGAAAACCAAGATTTGCTTTATCC
GGTATCAAACGAGAACTGCGAGCAAATAGAACACCTTTCAAGAGTATCAATACCGTCACATGAATCGTAAAT
GCATGAATGTGATGTACCAAGAAATCCGCGGTTCCTAATGGAATAGGCAACAAAGCCACCTTGCCACCCACT
GCCACTAAATCACCACCCCCCCAAGTTAAACTGGTACTTGCTGTTGCGCCAGGAGCCGTTGCACCAGGTGCT
AAAGCATGGGTGTTTTGTATCCATTGAGCAAAAACAGGTTGTAATTGTATAGCGGTATCTGAAAACATATCTT
GAGGACGCCCTAAAGCGCTCATGGTATCATTATGAATATACAAACCAAAACTGTGAAAGCCTAGAAATATAC
ATGCCCAGTTGAGATGGGATATGATTGCATCACGATGTCTAAGGACACGATCTAATAGATCGTTGTACCGAGT
AGTTGGATCATAATCTCTTACCATAAAAATGGCTGCATGCGCGGCAGCACCAACTATGAGAAATCCACCAAT
CCACATGTGATGTGTGAACAATGACAGTTGTGTACCATAGTCAGTAGCTAGATACGGATAAGGGGGCATGGA
ATACATATGGTGAGCTACAACAATGGTTAAAGAGCCTAACATAGCTAAGTTAAGAGATAATTGAGCATGCCA
TGACGTTGTTAGGATCTCATATAGGCCTTTATGGCCCTGACCTGTAAATGGACCTTTATGAGCTTCTAAAATAT
CTTTTAGTCCATGACCAATACCCCAGTTGGTCCTATACATGTGACCTGCTATCAGGAAAAGAATTGCAATAGC
TAAATGGTGATGGGCAATATCAGTCAGCCACAGACCCCCAGTTACTGGATCTAATCCTCCACGAAAAGTAAG
AAAGTCCGCATATTTTGACCAATTCAAGGTGAAAAATGGGGTTGCTCCCTCGGCAAAACTGGGATAAAGTTG
AGCCAAAAGATCTCGATTCAAGATAAATTCATGAGGAAGTGGTATCTCTTTAGGATCTACTCCAGCGTTTAGA
AATTGGTTAATCGGTAAAGATACATGTACTTGATGCCCCGCCCAAGAGAGAGACCCAAGTCCTAGTAGCCCT
GCCAAATGGTGATTCAGCATAGATTCTACATCTTGAAACCAAGCCAATTTTGGCGCCGCTTTATGATAATGAA
ACCAACCAGCAAAAAGCATTAACGCTGCAAAGACCAATGCCCAATTGCTGTACAATAGAGTTGTAATTCACT
AGTTATTCCAGATGCTCGCCAAATCTGAAAAAAACCAGAGGTTATTTGTATTCCTCGGAAACCCCCGCCTACG
TCACCATTTAATATTTCTTGGCCCACTATTGGCCAAACCACCTGGGCACTAGGCCCAATGTGAGTTGGATCAC
TTAGCCACGCTTCATAATTAGAAAAACGAGCACCGTGGAAATACATGCCGCTCAGCCAAAGAAAGATGATG
GAGAGTTGACCGAAATGTGCACTAAATACTTTTCGAGAGATCTCCTCCAAATCACTGGTATGGCTATCGAAAT
CGTGAGCATCAGCATGTAGGTTCCAGATCCAAGTGGTAGTATCAGGCCCTTTAGCTATTGTTCTTGAGAAATG
ACCCGGTCTGGCCCATTCCTCGAACGAAGTTTTTACGGGATCCCTATCTACCAAAATTTTAACTTCTGGTTCCG
GCGAACGAATAATCATTGAGTCCTCCTCTTTCCGGACAACACATACAAAGAGACCCGCCAACAGTCAAATAA
TTAGTGAACCTTAGAGATAGAGAGATATTTCTATAATTAGTTTGTTTCTCTTCTATTTTTCTATCTCCCATCTATC
TATTTTCTTTAGTTATTTACTAGAGCAATTATGATCTGGAAGTCGATCCGGGGCAAGTGTTCGGATCTATTATG
ACATAGCCTTGAGGCGCTCAACGGACCTTTTAACCTTCTAAAAACCTTTTTGGGCTTTGGATTGATCCAAAAA
CGAC
>TCONS_00020554
GTGCAAGCAAATTACCAAACGAAAATGTTATTCCAAACTTGATCGGGTTGAAGAAAACCAGCATTGACAAG
AGAGTACAGGCGATACCAGTTGACAAGCATATAGCAAAACCATAAAGCCTCTGTTTGGTAGATAAAGTGCA
GTTGCGATTAAAATCATCCATGAAAGATTCCTGCTGAGGAGCTGCTTCTTCATCTTCTACATCCATTCCCACCA
GCATTTTCATCTTCTCCAAAGCCTGGTTCATTTTCTCCATTTTTTTCCCTTTTTCG
>TCONS_00020555
TACATCTCACAAAAACCACACATTTTATCTTCTTGAACATCTTCCATGTTGTGCTCATAACGAAGTAGTTTCTT
TTTAGCCCATTAGTGATAGTATACGGATCCTGTTTGGTAGATAAAGTGCAGTTGCGATTAAAATCATCCATGA
AAGATTCCTGCTGAGGAGCTGCTTCTTCATCTTCTACATCCATTCCCACCAGCATTTTCATCTTCTCCAAAGCC
TGGTTCATTTTCTCCATTTTTTTCCCTTTTTCG
>TCONS_00020559

ATTTAATTTGATGCTCCCTGAAATCATGAGAAAGTCGCTCAAACCCGCAAGTATTCTCTTTATAGATTGCCTCT
TCCTCCAATGTAAAATTTTCTCTTTGATTGACTTCTTCACACATGCCTTTTGGTTTCTTCTCTTTAATCCTGTAAG
GAAAGAACAGAATACAGCTTAACGTTTTCCACCGCCACCACCGAGCTTAGGACCTTTCCCTCCAGCTCCCTTG
GGCATGTTGCCCTTCCCTCCAGCTTTCTGTGACTTGGCCTGCACCTCTGCCTTCTTGGCCTTCTTCTCATCCTTTG
TCTTCTTGATCCTTTCCTTGATTTCACGGAGAGCAGCCTCCCTAGCAGCATCTCTAACTTCTGGCCTTTCAGTTC
TCTTCTTCTGGATAACCTCCAGGGTTGCACCCACAATGGACCTGGAGTAAGGCTTCTTTGTTGCACGTCGCCTC
TTCTTAACAGCTTCTTGTGCAATATCCTTCTTGTGCTGCTTCCTGTACATAGCTGTCCAAGTAAGTTTGGAAGGC
TTCAGGCGATTGTGAAAGTACCGTTTGCATTTTGAGTTGAGAAACAGGAACACCTGAGAATCTGAACGAATA
AATCTGATGCCCCTCCCAGGATATATCTTGCCACCACTAAAACGACATAATTCCGTCTTGAGAACCATTGTTG
ATGCAGCTGCGAGCTCTCACGTTGCGATGTCCCGAAAATGGAGAAAAAAACCCTAGTGAACAACGCTTATAT
TGGAATTGGAGGAAAGATTTTGGGCCAAAAAGAAAAGCAAACCTAACATAAGCCCAAGAAGTGGACCTCTT
AATGGGAAAAAGGCTGCACCAATATTGGGTCAGTCCAATATTCAAAATCATACCACAAATCAATCGGTATAG
GTTGGGTGGGCCAGCCCATTGGG
>TCONS_00020647
TCCCTTTATATAACTGATCATTCGAGCTGTAACACCAACTTAACAATCGAGACAAAAAAACTAAATTCCCAG
ATCAAATGAAGGAGAGTTTCCGCCATCAGGAATTTGTGTAATAGGTATTGAATTCACTGTATTCCGAACCAAG
GACTGTGATTTATTTCTAAGAATTCTAGCAGCCTCTGCTTCCATTGCATCAAGATCGATACTTTGAAAGAACTG
GTCATCATCAAATGGATCACCAACTTCATTCTTTGCTGCTTCTTCCAAAAGATTGGTTGAGCATGGTACTGCTT
CTGGTGCAATTCTATTAATACTTTGCTGAAAGTTCAAGGAATTTCTCGTTATTGACTTTGGGTCAGTTTGAGGT
ATGTGATGATTTGCTGTTGCAGATGAGCTCTTGCTTTCATCTGTTCTGCTAGTTGGAACCTCAGAATCAGGAGT
ATAATCGTTTGAAAGGTTTGGAATTGGTGACTGAGTGAGCAATGAACGCCTGTGATTAAAGATTGGCTTAGGT
CCAGAAATCAAGGCAATGCAGCCGTGATCATCAGATGATCTATGACTTCATGAAAATATTACTAAAAGGGAA
TCTATCTCCCTGATAAAAATGGTACTTTG
>TCONS_00020722
AGAAGGCCTGAAAAGTGATTCTAACTAGAACAACAACCACTCTTCTGGGAAACAGGTTGACCTCGTATCTGC
ACTGTTGGAGGCTTTGCATTATTCGATGCTGGTTGGCTTGCCATCCTATTCTTTATGTCAGCTGCCATTGCCATG
AACGCCTGCTCAACATTAGTGGCATTCTTTGCACTAGTCTCCATGAATGGAATACCAATTTCATCAGCAAATG
CCTTTGCTGTATCATAAGACACAGCACGGTTGTCAGCCAGGTCGCACTTATTTCCAACCAGAAGCTTGTTTAC
ATTGTCACTCGCATAACGATCAATCTCACTCAACCATTGCTTAACATTGTTAAAGCTTTCTTGGTCAGTTACAT
CATAAACTATAATGATGCCATGTGCCCCACGGTAGTAACTACTGGTAATCGTCCTGAAGCGCTCTTGTCCAGC
AGTGTCCCAAATTTGAAGTTTCATTGTCTTCCCATCTTGCTCCACAGTGCGTATTTTGAAGTCGACACCAATTG
TGCTGATGTAACTGTCCAAATAAGAATCATCAGCAAATCTCAGGAGAAGACATGACTTTCCAACACCAGAAT
CTCCTATTAATAAAAGTTTAAACAAGTAATCATATTCAGGATTCATGATTTTAAGCCGACTGATCCAAAGGCG
ACGGGAACACGGTCCACCCAACGGACGGATAACTGATGGCGAAATCGGATCGT
>TCONS_00020746
AATTGGCCATAGACTTGCTCATGGATTGACTATGAAATAAGATGAAGTAAATTGTAACTCCTCCCAGATCACA
CTATTCTTTTGGATGTCCTTAAACTTTCTATATATGTGGTAGAAGATTTATGAAGCCGAGTTTTCGTAGGAACA
TGAAGTGCATAGCTACAAGAAGACCAGAGCTTCCCATCATAGTGAAAAGACCAAAATATAACTATTGGTGCT
TACTGCATACTAGTTTACTGTCAAGGACCTGCTCACCTTCTTTCCATTTAATTTTGACATGCAGCAGAAAAATA
GACGACTGAACTCTATCTCTCTTTACAACTTCTCGATCTGTATTTGCATAACGTCTGTTTCCTTTAACACTAAGA
TGTCTCTTGATGGCTACCAATATAGGATCACAATTGTTAATCAACATCATGTGTTCCAAAATTTACCTGTACCT
TACTAATTTACCTTTCTTTCTCTTTTGTTTTTAATATGCTTAGATTGGTTATGATAAGCAAGGACTTCCAATAG

GCTTGCAGCTTATTGGTCGTCCGTGGTGTGAAGCTACAATCTTGCGTTTAGCTACTGTGATAGAGGAAATCTCT
GCAGAGTATAGGAAGAAGCCAGTGGAGTACTATGACATCTTGAAAGGGAAGTGAGAATAATGTTTTACAAG
ACACAATTGAA
>TCONS_00020846
TTACTATCTTGTCCGTACTCCCTGACTAAAGCTTATCCGAGCCTGATGGCAAAGCAATCCCTATTACATGCTTC
AGATATGTTGTCAACATCTATTGTCGCACTTGGTTAATGAATCTCAGTAACAAAAGTTGTACTTAAAGCTCAA
AAAAGATATTGATTATCTTTCGAGTGTCAGACTAAATTTCGAAAAACTTCTTTAAATACAACATTTGTAGCTTC
AGTAGTTATATTCCCATCGTCATCACAAACCAATATTTTTAATCCCTTTCTACTCGTCACTCGAGAAAGAGCA
ACATATAGCTGTCCATGTGTAAAAACTGG
>TCONS_00020877
CCTAATTACGAAAGAAACCCTGTTGATTCGCCAAAGATCTACAAGCCAGGATTCTAGAGAAAGAGATGCTAT
TAAGGATACTTTAACAGAACCTTGTTTGTTACTGAGCTGGGTAACTAAAACCCTATCCCCTTCCTTTCTTAATA
GTAGATAGATGTAGAGCTGGAGACTCCCTCAATAGGGCATCTCAATCTGGCTCTCAATCTATCGTGTAAGGA
ACGAAGACCATAGGCATAGGGTAATAGCTCCTTACTTGTAATTGAGATAGGGGCTCGTTTGATACAAGTATTT
CATTTTGATCTCCTCCCTCACTTCTTTCTATAAAGAATTCTCAAAAAAGGAGTCAACCTGTCTAAACTTCTCTA
CAAAAGTCTTCCCCTCGCTGCTGGTCTTTAAACTTGCAACTGCTATCTAAGTCTAAGACAAGAGATTTTTCTTA
CTCTTAAGTTTGATTTTTCGCTAGAAGCCTTA
>TCONS_00020963
GAGTAATAATAAAAATGAACTGCAAAGAGGTATTGATTAAAAGGAAAAGTTGGTCTCCATACTTGATAAAAT
ACTAGTTCGAATAAGTATCCGCTAAAACTTCAAAACAACCAAGATAAAGATCCCAATATCAAAATACAGTGC
CAGCAATAAACAGAAGGCGGCCTGTTACTCTAAATTCTTTTACTCTTCATTAGCAGAGTCCACGTTCTTGTTGT
TGTTGTGCTTCCTAGCATACCTCTGGTTCCTCAAGAACTTGGGATCCATCCCTTTGGTGGAGCTGTGACGGTGT
TTCCTGGGTTTCTTGATTCCATTCCTGTGAGCCTTGTACGACTGATTGTGAGCTGTGTGATTCTTCGACTTGGCC
ATTTTTGGTCCTTGATTTCACAAACAACTGCAGCCGGCGTTAGGGTTTTCGGTAGTGATGAGG
>TCONS_00020978
CAGACTTCATACCCAAATATGATGTTGTCGCAAGGTAGTAATATGTAGTAGTTGCCAGTGCATTGCTTAGGTA
TTCAGAAACGGGTGGCTTATATCCTTCAATAAACCATGTTGACTCGACATTATAATTTCTTACTACTTCTTTCAT
TCTTTCAATTGCATGGCAGACAATATGAGATCTTCCAGCACTAGACAGTTCCTTTTCATAATCCTTGTAAAGAT
CTAGAATAGCTTTATAACTGATTTTCATGTAATCAGGAAGCCGATCAATTTCGTTGATATCCCATCTTTGTATG
GCATCTGTGTATGCCTCAAGTTCTTTAACTGTACCGTAAGCATCAAAGGTGTCATCGACAATAGAAATCATTG
ATATGGTCTTAACGAGCATGACGCGGGCTTGAGAGTATTGAGGCTCAAAATAAACTCCTAACGCCCAAAAGT
AACATTCAACTACTCGATCTCT
>TCONS_00020979
GGCAGCTCAAATTTCGATGGAGTCCACAAGTAGGCCAATAATGTGAGGTTTTAAGACTTTCTCCGGATGAGTA
TATCCATCTAGATTGTGTATATATGTAACCTCAACAATACGAGCAAGATTGAGAATAGGAGTTAAAAGCTCT
GCAGATACTGGAGTTGGCCTAAGAAGTCCTTCATTAAGATCCTTCCATGCTGTCTCAGCCATTTGTTGGAATTT
ATCCATTGCCTCTTTTGTTGATACACCATAATCTCTCATGCAACACTCAATCCCTGTTGCAATTTGTCCTTCTGC
AGATACAGGAGTTGGCCTAAGAAGTCCTTCATTAAGATCCTTCCATGCTGTCTCAGCCATTTTTTGGAATTTAT
CCATTGCCTCTTTAGTTGATACACCATAATCTCTCATGCAACACTCAATACCTGTTGCAATTTGTCCCCTGCTTT
TCTCAACCT
>TCONS_00020980

GGCAGCTCAAATTTCGATGGAGTCCACAAGTAGGCCAATAATGTGAGGTTTTAAGACTTTCTCCGGATGAGTA
TATCCATCTAGATTGTGTATATATGTAACCTCAACAATACGAGCAAGATTGAGAATAGGATTAAAAACTCTGC
AGATACAGGAGTTGGCCTAAGAAGTCCTTCATTAAGATCCTTCCATGCTGTCTCAGCCATTTTTTGGAATTTAT
CCATTGCCTCTTTAGTTGATACACCATAATCTCTCATGCAACACTCAATACCTGTTGCAATTTGTCCCCTGCTTT
TCTCAACCT
>TCONS_00020981
GGCAGCTCAAATTTCGATGGAGTCCACAAGTAGGCCAATAATGTGAGGTTTTAAGACTTTCTCCGGATGAGTA
TATCCATCTAGATTGTGTATATATGTAACCTCAACAATACGAGCAAGATTGAGAATAGGAGTTAAAAACTCT
GCAGATACAGGAGTTGGCCTAAGAAGTCCTTCATTAAGATCCTTCCATGCTGTCTCAGCCATTTTTTGGAATTT
ATCCATTGCCTCTTTAGTTGATACACCATAATCTCTCATGCAACACTCAATACCTGTTGCAATTTGTCCCCTGC
TTTTCTCAACCT
>TCONS_00021003
TTGTGCCTCGGCACAATTTACTACAAATAAAGAGCAAAATCAACTCAAAATCTAAGCTTGGAAAAGAACCA
GCGATTTTTCCCAGTCTTAAATCGCTCTTCAAACTTAGCTTTCGCCTCTTTAGCAGCAGTCACCTTCTTATCACG
TGACTGAAGACAATCAACAGTAACAGCTTCCTTCAAATCAACATCCAATGTGTAACGCGTCGGCATAATGTG
ATTGTAATTAACGAGCTTAATGAAAGTCTTAACCCGTGATTTCTTCGCCTGTTTTTATCAGAATCCTTACGAA
TCACTTTCTTTGGGTATTTCGATACGCCGACGACCAAACAGTGACCGTACGGCCTGTCACGTGTCCCATCGTC
AAAGGCTCGAACAATCACAGCTTTCCTCCCGGCGTAGCGTCCTTGGAGTACGACTACGGCTTTGTTTGGCTTC
AG
>TCONS_00021155
TTTCTCTTGTTTGTTCATTTCAGGAAGTTGGGTGTTACGTTTATGGTGCTTTTCGCCTTGTATGAAGTAAAGTATT
TACTTGCCCATCTCGTTGCTCTGATGGACACTTTCTTCCATGGAGACTAATGGGAAAATGTAGCTGGTTTTTGG
CTTGACATTGTCTGTATAGAAGGTATTTAGAATGGCAGATTTGCAGAGGTAGTATGACGCGCACAACATGAG
TTTGAATCCTGTTGCACCAAGTTAATGGAGAACGGTAGAGCTAGCGGACCCTTCATCCATTGAGTTCGAAGAT
AAACCAAGCGAATTCAAACTCTTGGGAAGCATCA
>TCONS_00021171
CAACAACGTTAGCTACTATGTCACATCCTCATCATCCCTTCCCAAATACAAACAAAGTCCGTCATCTTCCCCA
TCCCAATTAACGTAATCCAGCATTTAATACCCAGTAACAAATTGCATGAATTCCGGGTCGGATTCTAATCCAT
CCATTGTTTCCGGTGCCAACACCACCTCCAAATCAACGCTGCCGCCGCCTTCTCTTCCCGGAAACGCTGATAT
CTTGCCGTCAAATTTATTGGCCCGTCCGCTCCGAACAGCGATGGGTCTGCCCCACCCAAAATCATTATCGTAC
ATTGGAAATCTTGGGGAGCTTCCCATTGTGAGCATAGCTCCGTCGAAATTCCCTAGCGGGAAACACCGTGGG
TCGCTCTCCCAATCCTCTACGTACTTCCTCACCATATCGTTATCGTGCACCTTCACGTTCTTGTTCAATTGCTCC
GCGCACCATCGTAAATCGTGAGATAAAACGTCACCGGCCGACGCGTAAGTCGGAATGCTCTGAATAGCGTTG
CCGAAATATAATGGATGGAGCTTTGGCTGGAGTCTATGCCGGCAATTAACGGCCATCCGAAATGTCGTCATTT
TGGAAGCATGAAATTTCCTCGCACGTGTCACCGCACGCCAAAGCAATGCGCAAAGTGATTGAAATGATGAGA
TCTCAGCCGTTTGATTTTCAGTTATACCTTCTGTTTCTGTTTTCCTAACCGCATTCCTCAACCAAGTTAACGTCG
TTACTTTGCCGTCAGACATTTTGAAGGGATCGTTACTCTGTTTCCC
>TCONS_00021194
TTGATATACAGGTAGAGCTTCTTCGAATCATCAATACTGTCAAGGTACAGCATTGTTGCCAATATCTGGTTGCT
AAATTCTTCATCTATGTGTTGTCCGATGAAAATAACACGTTCACGGTAAAGAGCATTCCACAAATCAACCCAC
TGCCACATTCCCTCAGTTGGATTTCTGTAGGGCACCCTTGGGGTCCCAATGGGCATCATGGAAACTCGTGCAC
GTGTTGGTTTACTGTTTC

>TCONS_00021197
CTAAATAAGTAAATCAAAAGAGTACATATGTAGATTGAACTAAAAAATCGCAAAATAAGACTGCAAGTTTT
ACAACCACTATCAGCCATTCCGTTTACAAGGCATATGAACTAACCAAGACCTGCAGTGGTATCCTTTCGTGGA
GCATCCGCCTTAATACGGGGAGGCCTAACTATACGGTCTATAAGACCATATTCAAGAGCTTCTTGAGCATTGA
ATCGCTTCATCCGACTTAAATCCTTGTGAACCTTTTCAACAGGCTGGCCTGTCTTCTCAGCCAATTCCTTGAAA
AGGTAATCTCTAATTCTGAGAAGTTCATCTGCTTCAT
>TCONS_00021205
ATGGGGTAAAATACGTCTCAGCACTAACTGTCCCACTTCAAATTTCCTTGGACGCACTTTCTTGTTGTACGCTC
TTGCCATTCTTTGCTGGTACAATTGGCCATGACACACTGATGCCAACCTTTTCTCATCAATCAAACTCAACTGT
TCAAGGCGGGTCTTCACCCATTCATCATCATCAATCTCAGCCTCCGCAATAATTCGAAGGGATGGGATTTCCA
CTTCTGCAGGTATTACTGCTTCGGTGCCATACACCAATGAATAAGGAGTCGCCCCCACTGAAGTAC
>TCONS_00021211
GTAGTTTTCACCTCGACCTCAACTGGAGCAGTTCCTACTACTTCTACTGGTGCAGATTGAACCTCAACACTGG
CCATTGGAAATGGAGAAATAAACTTGAAACTAAAGATTTGAAGAAAGAGTAAAGAAGAAAAGGAGCAAAG
GTGGCCAAAGTGCTTTGAATGTGAGACTGATGATGAGAAATGATCAATTGAGGGCTATTTATAGGGAAGTAA
AA
>TCONS_00021271
GAAAATAAGAGGAAAACCTATTCCCTTGTTGAATAGGAATCGCGCCCAAAAACATCAAAAATTAATCGCGC
CCAAAAACATCAGGATTAATCGAACAGAGTCACGACTTTGCAAGCCTACTTCGACCAGATTTCGATCATCTTT
TTCCCTTGTTGAACGGATGCAGAATCGCGCCAGATCCTACATCGAACAGGATTAACAAAGCCGCGACTCTGC
TAGCCTACATCGATC
>TCONS_00021311
GGTTACTTCTCTCTATCTGTAGATCCTTTTAACTTTTTGCTTACCAGGATGTTTCTTGATCATGCACTTCCTTGGT
AACCATCTTTTATAAGATTCTCTTTTACCAAAAAAAAGGACTATGCAAATGAACTTACGGAGAGTCTGTCGTG
TCATCTATTATCTTTATTTTCATCCTTTTTATGATGTCTAATTTGTAATTTATCCACTTAAAAAAGATGGCGCTTA
ATTGGTATCCTTATCGTGCCCAATTGAAATTCTCTTGCTTTGATGTTTTGCTTTTATTTCATATCTTGCTACACTT
TCATTTGTATAGGTTGTATTTAATGAGCTCTACAAGATATTAGACTGTAGGAAGCCGTCCTTTACACCAAACA
AAAGGAAAAACCAGTGATATCGTGTTTGTAGATTTGTAGAGGATGTGAATAGTGGAATTGATCTCTCATTCTT
GCTACGTATCCCATTCAAAAGTGTGAGGAAGATAGCACTGGAATTGAAGGCAACAATTCAATAACTAATTTA
GGAATTCGTGACCAATTGCTTTGACTATTATTTAGATAAGTGGTGATTGAAGAAATTTTTGTTAGTGTTTCTTTT
TTT
>TCONS_00021472
AGTTGTCCATAATCATTGAGCCCACTGGCATAAACACGGCCATTTTCTGTAAGGAAGAGAGTATGAGCACCG
CCACAAGCAATCTCACGGAGGTTTTGATTATCGAAAACGGAAGGAAACAGAACCTTAGGGCGCCACTGCGA
CTCAAGACTCCCATGCCCTAGCCTTCCGTAATCCCCGTTTCCCCAAAGGGCTGCGAATCGC
>TCONS_00021689
ATAGAACCTTTTGATATTTCATATCCACTTTGATTTGATTTCAGTCCTTGAACTGGATCTCCTAAGCTGAGTAGT
GTGGATATGGAGAGGATGCTAATATCTGGATCCCAAACCATGACCAGCAGCATTGCAGCAGTTCCATATGCA
ATTGTCGCAAACGGTAGGCGAATAATGTGCTTCAGCTTCTGGCTGAAAACACAGTAACCCTGCAACCGTATTT
TCTGGACTTGGTATACCAGATATTGCTGAAAAATGCCCGTGATAACTAAGAGGGCAACATTACAACTGCAAA
GAAGTTGGAAAACTAATCCCCCGATCAGGTGAAATATCCATGTAGCCCCCACAGCCAAAACAGCAAAACCT
GCTTGCCAATAAGATACAGTAGAAAACACTCCCAATCAGGCTTGCAGGCTTCCTATTACCAAAAAGTGGTGC

CTCATCCAGTATGTCCAGGAACAAATTGTTTTCGTTTTCGTAGATAGAAGAAGAGTGTCTTTCTCCTGACATTT
TCTTCTTCTT
>TCONS_00021751
AAAAGTACAAGCCTTGTCATCTGATGCTGCATTTGGAAGGACTGTTATTCTGCTATCTTGCATGGGCTTTACAC
TGCCAAAGTCGGCAACTTTAATATGTCCATCTGCAGTAAGTAGCAGGTTCTCGGGCTTAATATCTCGATGAAT
CAATCCCATGCTGTGTATATATTCAAGAGCATCTGCTACTTCAGCTGCATAAAAACGCGCCTCCTCCTCAGAC
AAAC
>TCONS_00021752
ATGCTACCAGCTTCAGAAGAGGATGTTGCACCACCATTTCCATCATTAGTTCTAGCTGATCCATCCCCAATAT
GTGATGGATTCCATGAAGAATCTTGATCTTCACCACTGCTACGAGTAGAGTGGGCTTTTGGCTCTGCAGCAAG
ACATGGAGGAGTTTGAGACCTTAAACTATCCCAATCAACCTCCCTAAAGAAAGGATGGTTCTTCAGTGAAGC
GTAACCATCAGGTCCA
>TCONS_00021813
CCTTAAGATTAATATTCCCTTCACACCTCATTTTAAAAGTCACAAGCGGATAAAGCATGTTGCCCAATCCCTTT
CGGACATCCGATAGAATTGGAACAATATAGAGAACGCTAGCATGGTCCATGCACGAGAATGGGCACGCAGA
AACTGAGAGACGTGTTGCTCCAAAATGAGATTGTGATACAGAAAATATTTCCTCAATAATCTGAGTTATTATA
AATTTACTGCAGCACCTTAAACAAAATATAGGATGCTTGCACAGGTTTATGATGAGAACATCTTCTCTATTGA
TCAGGCAGTCCGCAGAGGAGGAGACACCAGTATTACACCATCCCCACGAACAAATAGGAAAGGGATGGTCC
GTCTTGTAGTCCGAACTATCTCCTCATATGTTTCATCATCAATCTCAATTGTGGTCACAATTTCTTCAACATCAC
CAAGAATCATATTAAGATGCTGGTCATAAGCGTGAAGTTTGCCACGGAGTTCTCGGTCTGAACGGAGTTTGAC
GTAGATTCTCTCATCAAGGCTGAGTCTGATGAGATCCAACGGCTCTTTCACTGTGCTCTCTTCTTCACTTCCCA
TGGCTGTTGTC
>TCONS_00021901
TACAAAGCATTCAAGAAGTCTATTTGCACTTTGCAACAAAAATTAGTGAGGTTGAACTACTATATCTAGGTAA
TCTTTGAGCTATGTCATGGTTCATCTTCATGAGCTTTTTCGGCAGCTTCTATCTCCGAATCAAGCTTGTTGACTA
GCTGCTCGCTTACATGTTTTGCCACAGATATCATGTCAAGTATGACACTAGGGTCTAATCCGGTGCCTCCTCGT
TTGGTCAACCCACATACTAACCCTTGTCTATTAACAGATATAGAAACAGCCGAGTTCATTAGGGACTCCTCCT
CTGAAGTTGCATCGACAATGTAGTGCCTTCCAACCTTTATCAAAGTAACTATGACTGGTATTCCGCTGGTGTC
AAATTGCAGAAATTCGTCATCACTAACATCCACATCTGCTTGCTCATCGGATGAAGCAGCTTCACGAACCTGA
ACTCTCGGAATACCCGTATTGCTCAGTGCAGCCTTGATTGCAGCACCTAGTGCGTCCAGCAAATTCCCGCCAG
AACTAATGACAAGGCAATCAATATACAGATCCCAACATACTTTTCCTTCTTTGATTGAGAGAGACAACGGAT
CTATTCCAGCCCCTGCTCCACTTTTACCACCC
>TCONS_00021902
TACAAAGCATTCAAGAAGTCTATTTGCACTTTGCAACAAAAATTAGTGAGGTTGAACTACTATATCTAGGTAA
TCTTTGAGCTATGTCATGGTTCATCTTCATGAGCTTTTTCGGCAGCTTCTATCTCCGAATCAAGCTTGTTGACTA
GCTGCTCGCTTACATGTTTTGCCACAGATATCATGTCAAGTATGACACTAGGGTCTAATCCGGTGCCTCCTCGT
TTGGTCAACCCACATACTAACCCTTGTCTATTAACAGATATAGAAACAGCCGAGTTCATTAGGGACTCCTCCT
CTGAAGTTGCATCGACAATGTAGTGCCTTCCAACCTTTATCAAAGTAACTATGACTGGTATTCCGCTGGTGTC
AAATTGCAGAAATTCGTCATCACTAACATCCACATCTGCTTGCTCATCGGATGAAGCAGCTTCACGAACCTGA
ACTCTCGGAATACCCGTATTGCTCAGTGCAGCCTTGATTGCAGCACCTAGTGCGTCCAGCAAATTCCCGCCAG
AACTAATGACAAGGCAATCAATATACAGATCCCAACATACTTTTCCTTCTTTGATTGAGAGAGACAACGGAT
CTATTCCAGCCCCTGCTCCACTTTTACCACCC

>TCONS_00021960
TTCAATTGTGTCTTGTAAAACATTATTCTCACTTCCCTTTCAAGATGTCATAGTACTCCACTGGCTTCTTCCTAT
ACTCTGCAGAGATTTCCTCTATCACAGTAGCTAAACGCAAGATTGTAGCTTCACACCACGGACGACCAATAA
GCTGCAAGCCTATTGGAAGTCCTTGCTTATCATAACCAATCTAAGCATATTAAAAAACAAAAGAGAAAGAAA
GGTAAATTAGTAAGGTACAGGTAAATTTTGGAACACATGATGTTGATTAACAATTGTGATCCTATATTGGTAG
CCA
>TCONS_00022089
GGTTGACAAAACAAGAAATGACAAAGTACCAGCTTAAATGATTCAGAAAGAGATTCCACTGAAGTGTAAGC
AAGATAAAGAAGAGAACTCTTGTAGAACTCGGCGAAGTCCTGGCGAGCTTTATGATACTGAGATGAAACCC
AATAATAGCTCGCATAAACAGATGGATCAATGTCAGTCATACTGTCAAGTGTAGTCTTCCCTTCATCTAAAAG
TTTCTGGCACTCTTTTTGATCCCCTTGCTCAAGCTTAAACAGGGCAATCTGCATCTTAATATAAAGAATCGGCT
CCTCTATCCGTGTCTCTTTAGTATTACGAAGTTTCTCAGTCACTCCTTCAAGGTAACCTACAGCAGCCTCTTTCT
CAGGGTATTGCCGAGAAACAATGACCGCAAAATGTGCAAGCTTGAGAAGATTGATCTTTGTCTCAAAATCAG
TGATGAAGTTGTCATACAACTGTATTAGTGTGTCACCAGCCTGAAAAAGGGGAAGTGAAACGAACTGGTCAA
GCTTGAGAGTAAGCTGGTGCCAGAGCTTTCTTTGGTACAGATCTGATAGCGTAGTGTACCAATCGGAAAGCTC
TGGATTGGCATTGCAAAGTGATTCCAAATACCGTAGAGCCGCCA
>TCONS_00022178
TGCCCCGCCAAGATACGGGACAGATTACCTCAAAGCTGGTGAAGGATAGACCAGAAAGAAAGAATCAAATC
ATCATAGCCCATTATTAGCAGCATTTAAAGTGGCTACCTTGTGTCATTTCTACCTTGTTCTAAATACAAGGGGT
CATAAAATCGGCTATTTGTGCAACTCAACTGCTGTAGCAGCAGATTAGTCAGCAAGTCAACTGCAGAGACAA
AATATTTTAGGCTCAGATCTTGTTCAAGTCTCATTCAGTCTTTCAACACCGTGATGGCTGCAAACTTTCAGCAT
GTTACCATAAACGCTAACCAGGTCGCCCTGCCAAGCTACGGCGCAAATTATCTGAATGCTTCATTGCATTTTC
TAAGAAGAATTTCATCATAGCCCCATCATTTATTTATTTATCTATCATTTTCTCCACACAACTTC
>TCONS_00022366
ACAATCAAGAGTACTGATAGAACGATAAACTCTTGTCATCAGAGACACTTGCAAGCATACAAGACCGAACA
TCAGTCCTTGATGTTGGTCCAAAAGCCACAGCCCAAACCTGATCTGTATGGTTGGTCAAAGTCTGTGTAGCCG
CTCTCATTTTAAGATCCCACAGCCTAACCGTCTTGTCACTTGATCCTGTTGCAATTGCTGCCCCGTCTGGGGAG
ACATCAACACTCAGAACCCAACTTGAATGACCCGACAAAGATGTTAGCAAGGTTTTACCCTCAGCATCATAT
ACATGTACATGACCATCATCTGATGCTGAGAAGAGTATCCTTGAATCATGAAGCGAAGGTGAAAACACGAG
AGATCGCACAGGCATGCTATGGCCTTCTAAGAAGTGAAGGAATTTGGCACGTGCTACGTCGAAAACAGAGAT
AGTCCCGTCAACTGAACCACAAGCAAGAAGTCTACCATCAGGATTCCATGCAACTGAAAGGACAAATTTCTT
GTTGCTGCTTTTCTCAGATGGTTGTGGGGCGGCTCCTTGACGAGGAATTGACAATGTGGTAATAAGTTGCCAC
TGAGTAGTATCCCATAGCTTGACCGATGAACTACCACCACCAGCTGCTGCCAGAGTGCTCAAACACTCTAAT
AAAGCTATCTATAGAAGC
>TCONS_00022385
TCAGCTTGACGCATCTCTACTCTGTAATTTTCGCGGCTTCTGACCACATATTTACTTGACAAAACATTGAGCTT
TTAAATGCAAGAGAAATGAGGCCAGTAATTACTTGAGATGCTCTTCAGGTGAAGAACCTAGGTCTTCCTTGC
ATTGTGGGCAGCACTTAGTTTCTTGAACTGCTTGATAGATGCACCTTTTGCAAACTGCAAGCAAGGAGGAATT
GTTGTAAGTCAGATAAATAAGTTAACAATTTCATGTTCTTTCTTGATTTCTAGATGGACAAGAATCAATCATGA
GGCTAATATTGATGAGAATCATAAAATTGAGTAAACAAGCTAACTGTTTTCTTTCATAAAGTAAACCAGCTAA
TTACCAATCTCAATAAACAAACATATAACAACAAAAGAAAGATAATGTCCACCTCAATAAATGAAAATACA
AACATTGATAGCTGAAGAGATGCACTTCTGATTTGATAACTAGATATTTTCTTAGAAGAGGGAAGCATTCTGG

AAAAGGATCAACTTACACCCTCCGAGTCTTAGTGTCCAAATCATGAGAATAATACTGCCACTTCTTTTAGTTT
AAAGAGCTCTTGCCCCGGAAGTCTTATACTTCTGTTAAAGTAAAGACAATTATAAGTTTATAATTCAAGTTTG
ATAGCCTATAATATATTTTTGACACTCATCTACCTTGAAGTGTACTTTGCTAGTTGGTTTGTGGTGCTAAACAT
GCCCATGGACATGTTAAGTATCTGAAGTTCCGTCTGGTTAAAAAGAAGTAGCTGAGCTTTTCGTATTGATAAT
TCTATTAATTGTGTAATAGGATTGGTGGAAGAGCCGCAGCTATTTCTTGTACTTTTTGTGTACCATCTCGATAC
CATTTTAGTAAACCCTTCCCTTACAAAAAAGGTATTTCTATTAATTGTGCTACTGTTTTATTATGGTAATAATAT
CAAAAGTGAGAAGAGTATAGACCCGGTCACACAACATACGTAAATGTCTATTTACGTGTGCATGCATTGCAA
ATGCAAAAAACACAGAGAAGCAACTCATTGAAGCTTACATGTGTGAAGGCAAAGGTTTACAGTGGTGGCTTC
TTCAAACAGTTTACTGCATAGCATACAACATAAAGCTGCATAAATCTCCTTGAGCTTGCAACCCATTGATCTA
TCTGCTTTGACTGATCTCCCAGAGTCTTTCACTTCAGATCTTTGGTCACCCTTTGCCGTATCCATTAGAGAACTT
TTGTGTCCTCTGTTTGCCTGCACTATGAGGTTATTTCTAACTTCTGAAATTATGACATAGATTACTTATAGACTA
GATTAGACAAGTTAGTCTTAATAACACGAAGAGCTGCAAGAGAAACCATAGATACATAGAACTATTCAACA
CAAAAGACACAAACATCCGAAAATTCTGTGGCCTTCGTTATGTGTTATGATGCATTGACTGTATGGATTAATG
CGATGAACATATAGATTATCAGTTGACAAGGATACTTATTTTCTCAGATATCAGAACAATGATTATCTCAATA
TGGAAAAAATGTGTGGTTCCTTGCCTTTGATTCATACTTGTGTACCTATATATGAGAAGTGGAGTATGACCGGG
CAATTGTTAGGAACACTATTTTGAATTTTTGTGAGAGGACTAAAACATAACTGTAGTTGAAACAATTCTAGAT
GGAATATAGGAAGGATTTATTTTTGAAAAATATTATATGATAAAATTGTTTCGGCATCAGCTGTACCATCTCA
TAACCCCCTTTAAAATCATTGTTTAAGTCCCAATTGAGAAAGAAGAAAAAAGTCATCATTTGAATATCCTGGT
ATCTAGTGGCTCTCATATGGCCCACTGGATAGGTCTACTCCTAGGGTATTTATAAGTTTGCCATTTCATGTTGG
TGGATTAAAGGATTCAGGTTGTACAAGCACTAAGTATAACCTGGAATGAGCAAGTGGTATAACATCTATTCA
GTTGGACAAGCTTACAGATCTTTCACACCTCAAATTGGATTTAGTACTAAGCTGCATCGAGTTATGAATAATG
TATTCCTGTTTTGTATGTCGGATCTAGTATGGACCAAGCTTTTGGATTCTACTCTTTAATTTGATTTAGTTCTAA
GCACATCTTGTAATAAGGGGACGC
>TCONS_00022399
ATTGGGCTGAAAAATCTATCACCGAAATACAAAAACAATCATGGGTTTGCAATCAAACCCAAGAAATATATG
GATTCTTCGTCCTAAATTTACATTTCTAGTCCTTTTACTTCTCATCACTTCCCCTTTGATCTCTACTCAGGAAGCT
GGGGATGAAAATGAAAGAGTGGAGTCAGGTGGACGGTTCAAAGATCTGGGAAGGCGTAGTAAAGTGAGTCT
ACTAATCTTTAGCTGC
>TCONS_00022412
CTTCATCAAATAGTTCCAAATTTATTTCAACTTACAAATATCTTTCTTAACCTTATTACAGATTCTTGATATTTA
GTTTTTTGCTACCGGAAAACAAAATGGGGTTATCGGAGATTGTAGATTCCGGCAAGGTCACCGGCCAGAAGT
CTCAGTTTTCTCAGACTTGTAATCTTTTGAGTCAATTCTTGAAGAAAAAAGGTTCTTTTGGAGATCTTAATAAT
CTTGGTATTTACCGCAGTTTTGAACCAACTGGTATGTTCTTTATATCCATTTATTTAAATCAATAGCTGTTTTCT
GTTATCATTTGCTAGAT
>TCONS_00022553
TAAAATGCAAGGGAAAGAAGGCTTTGCAATACCATATGAAACTAATATCATCAGCTTCACCAGAAATTTTAG
AAGTTGTTCACAAACCAGCACATTATTTCTAAGGCTCAAAGCCTACAAGACATTTACTATATTTATATCCAAA
TACGACCTCCACGAAGAGCTCTAAAAAGCACAAAAGCCTCCTGAAGAAAGAAACAGCCTGACTACCATAAC
TTAAAAGCACACCATTACAAGGTGCAGATGAAGAAGCCAAAGTACATAGCAAATCAAAATCACCAAACCAG
ACTTCTAAATGTCTGCATTCAGCAAGAACCACTAAAAGCAACTGCACCAAGCTGTCAAAACACCTCAGTAGT
ATGAATGCATCCCCTGGTACATACCCCCAGGTGCAACTCTAGGTGCAGGTGATACCCCGGGCACTTGGTTTTG
TTGACTGTAATGAGGACCCTGTGGTGGATAATCTCCTGAAGCCAACCCAGTTGAGCTTGGGGGTAACCCATA

CATTGAAGAGCCACGGCCTGCACCACCTAATGCACCACCAACTCCACTTAAGCCACCACCTCCCAGCGCCCC
AGTGCTGCCATATCCACCACCTCCCAATGCCCCAGGACCGCCATATCCACCACCGCCACCAGTAGCACTACT
GGCCAACGCGCCATATCCAGCTGAACCAGCCCCACCATAATGAGACCCCCCACCTCCACCATATGGACCGCC
ATACCCACCACCACTAACAGAACCCCCAGGTTGGCTCCCACCATAAGATGAACCACCACCGGGACCACCCA
CACCTATACCATTCCCACTACCGTATGCTGAATTGAGCCCATGTCCGCCGGGCCCACCAAAGGTTGAATGCCC
TGGAAATCCACCATAACCAGTAATCCCACTAGGCCCACCATACTGGCCCGGGCCAACCTGATCAACATGACC
ATCAGTTTGAACCTGAGCTCCTCCTCCAGGTTTTCCCCCTTTCTTCCCATCAATAGCTAGCTTACAATTCAACT
GATGCCCATCAATATTCTTAACAGGATCCACTAGCGAAGCCTTAGCCGCCTCCGCCGTCTTATACACAAACAA
AGCATACCCTTTTGACTTCCCAGTCGCCTTATCAAACCCTAATGGCCCTTCCTCTATTTCCCCATACATACAAA
AATGCTGTAAAATCCTCTCGGCCTGCATATCATACGGCACATTCGCTACATAAATCTTCCTCGCCGACACATC
AACCGGATTATTGGTACTCCCGCCGCCAGGCCCACCTTGAATCCCAGCTGACGCGAGCTGAGTCACTGCCATT
CGTCCATCAATCTTCTTACTCGGCTCCTTCAAAGCAAGTAAAGCTCCATCAACATGTTTAAACGTAACGAACC
CGTAACCTTTGCTTTTACCAGTGGCTTTATCGGTAATTACAACGGCTTCTTCTAACTCGCCGTAATTACCAAAA
ATCGCTTTAAGTTTTTCTGTATTCGTTTCCCAGCCGAGTCCGCGAATGAAGAGCTTCCGTTGAGTGGTGTCGCG
ATCGGCAATTGCTCGTACTTGGTCTAAAACGTCGGCGTGACGTACAACAGCGGTTTGTAGGATTTCGAGCATT
TGTTCAGGGGTGACTGATTCTAGAATCTTCCGTGCGTCTTCGATGGTGAGTCTACTGGTAACGGGGACGTCGG
TGATAACGCCGTTATCCTCGAGCTTTCGTTTCTTCAAAAGGTCCATCTCTTTTTTACAGTGAAGAGATT
>TCONS_00022597
CACCAATCGAGCTTTGTATCTGACCAGCTTGCCATTTCCATCTTTCTTGAGTTTAAAGACCCATTTGCATTTGA
GTGGTCTTTTACTTTTTGGAAGTTCAACCAGCTTGTACGTGCCATTTTTCTATAAAGATTCCATCTCTTCTTGCA
TGGCTTTCATCCACTGGTTCTTTTCTGGATGGGACAGCACCTCCTTAAGACTTTCTGGCTCCCCCTCATCACTG
ATGAGGACATACTCTGTGGAAGGGTACCTGCATGACTCTACCCTTGGCCTTTCTGATCTCCTCAGAGGTTGAG
GTTGTTCTTCTCCCCGAGTGGGGTACTCCACT
>TCONS_00022657
GTAATTGATGGATTATAATAAGCAGCGGAAGCAATCCCAGTATCAAAATCACATGTAATCTAGACAACTAGC
ATAAACGAGATTTCACGGGTTACCTCTTGAAGCGTGAACATATCTCTTCTACCACGTGAGCCTTCAGTTCCAT
AACTTCTCAATGGAATCTCCATCACCCGCACAATCGTGATCCTCGAACGTTCCACGGTCTTCTACTGTGTTACC
CAAATAATACCGGAAATAGCAATTTCGTGTGGGCGAAATTTCTAGGAAAACTTGGCTGAAAATTTCAGCCAC
CATCTAC
>TCONS_00022704
ACATCTTGTACACATCCAGATTATTGTTAGAGATACTGTTGGATCCTCCAAGACCAATGAGCCTTAACCATTG
AATCTGGTCGAGATATTTTCGGAAAATCACATAAAAACAGGAATAATAAAGGAAAACTTCTCCAGAACAGA
ACGAATAGACAACAGAAACAGAGGGCTAATAAAGTAAGCCTTCATACATCATTAGAGTCCACCAGAGAGTT
ATAGGAGCCACACAGCTTAGGAAACTTTGCAAAATCCAAAATTCAAACAGATAGGAAAACTATAATCAACA
GTAGAGAGTAGACACTAATGATTGTCCATGTTGATCAGTAGCTTAGTTTAGGGATCTAATGTGCAGTGCGCAA
TATTCCATGCAGATAACCACCAGTCATAACAAGAGACACAAAGGAGACAATACCAGCTGGAAAAACCTTCT
TTGACTTCAGAAACCTAGATCCCATTACTCCCAGAAGTGCAGCCGAGAGGACGAAACCCATTGATGATGCCA
AAACAGGTTGTGTTGGAAGCATAGCATAAACAGAGTACAAAAGGAGGCAGAAACTCCACCTGCAATTAGG
GACTTCGTGCTGCCACTTTTCAGATAACCCATAAAACCACCAGCTCCAACCAAGAAAGCATAGCCCAATGTC
AATTTTTGGGACATAGAAAGGGCCATTTTCTTTTTATTGTTATGATCCTTGCCTTCATCTGACTCCCCCTCGTTG
CCACCATCATTATTATTATTATTATTGCCACCATTATTTCCCTCCCCACCACCGCCATTTGCACTCACAGAATC
ATCTCCACCACCACCACCTGTCTCTGGCAGAATATCAATTCCTGGCCCTGCTGAATCAACAGTGAAGGTGGTT

GGCATTGTAGAATCAGAGACAACACTGGCTTTGACTCCCCAGTAGGAAGGCTTTGATCGAGTGGACAAGTTT
GGGTACACCGTGCAAGGGGTATTCAATGGATGTAGAGATGAACGAAGTGACCTCAATGGCTGACTATGAGC
AAAGAAATGCTGCCTTTGACTCCAAGCTTGGACGCGAAAGTGAGGCAGCACTATGGAGGATTGAGCAATGG
CCAAAAATTCTCCCATGATGTATATGCTGAATTATGAGAAAGTTATTTACGGGATGGTTTGTTTTCTGTAGCTT
GTGTATTCTCAGCAACTTCCACAAAAATCAGTCTTCCATCAACAATCCTTCCGTTAATGGAATTCAATGCATTC
CGAGCATCTGCTTCTGACTCAAAGGTAACAAATCCAAAACCTTTAGGCCTTTGCGTTCTTGGGTCTTTAACAA
GCCTCGCTGC
>TCONS_00022705
ACATCTTGTACACATCCAGATTATTGTTAGAGATACTGTTGGATCCTCCAAGACCAATGAGCCTTAACCATTG
AATCTGGTCGAGATATTTTCGGAAAATCACATAAAAACAGGAATAATAAAGGAAAACTTCTCCAGAACAGA
ACGAATAGACAACAGAAACAGAGGGCTAATAAAGTAAGCCTTCATACATCATTAGAGTCCACCAGAGAGTT
ATAGGAGCCACACAGCTTAGGAAACTTTGCAAAATCCAAAATTCAAACAGATAGGAAAACTATAATCAACA
GTAGAGAGTAGACACTAATGATTGTCCATGTTGATCAGTAGCTTAGTTTAGGGATCTAATGTGCAGTGCGCAA
TATTCCATGCAGATAACCACCAGTCATAACAAGAGACACAAAGGAGACAATACCAGCTGGAAAAACCTTCT
TTGACTTCAGAAACCTAGATCCCATTACTCCCAGAAGTGCAGCCGAGAGGACGAAACCCATTGATGATGCCA
AAACAGGTTGTGTTGGAAGCATAGCATAAACAGAGTACAAAAGGGAGGCAGAAACTCCACCTGCAATTAGG
GACTTCGTGCTGCCACTTTTCAGATAACCCATAAAACCACCAGCTCCAACCAAGAAAGCATAGCCCAATGTC
AATTTTTGGGACATAGAAAGGGCCATTTTCTTTTTATTGTTATGATCCTTGCCTTCATCTGACTCCCCCTCGTTG
CCACCATCATTATTATTATTATTATTGCCACCATTATTTCCCTCCCCACCACCGCCATTTGCACTCACAGAATC
ATCTCCACCACCACCACCTGTCTCTGGCAGAATATCAATTCCTGGCCCTGCTGAATCAACAGTGAAGGTGGTT
GGCATTGTAGAATCAGAGACAACACTGGCTTTGACTCCCCAGTAGGAAGGCTTTGATCGAGTGGACAAGTTT
GGGTACACCGTGCAAGGGGTATTCAATGGATGTAGAGATGAACGAAGTGACCTCAATGGCTGACTATGAGC
AAAGAAATGCTGCCTTTGACTCCAAGCTTGGACGCGAAAGTGAGGCAGCACTATGGAGGATTGAGCAATGG
CCAAAAATTCTCCCATGATGTATATGCTGAATTATGAGAAAGTTATTTACGGGATGGTTTGTTTTCTGTAGCTT
GTGTATTCTCAGCAACTTCCACAAAAATCAGTCTTCCATCAACAATCTTCTGTAACAACGCCAAAAGGTGAA
AAGAGCTTCTTCAATCTTTCATTCGTAGTGTAAAACGATAATCCTTAGTGCAATGCTCTGCCGGAAAAAGATA
TAGAAAACTGAGTCTTGACGGGCTCAGTCGTGGGGGCGTGACCGCGTGAGTCGTGAGGCGGAGAACTGGCG
AGGGCTTGACTGCTTGAGCCGTGTGGCCGGTGGCGGACTGGCAGAGAACTGGTGAGGTCTGACTTCTGAGGG
TGAGGACTGAGGGTGAGGAACTGAGTGAGTGAG
>TCONS_00023029
AGAGGCTAATAAATCAGGCTTCTATAGCAGGAATACCACCTTCGATGTTGATCATATAATCAAAGGCAAAAA
GCTTATGAAGTGATGATGCCTGCCAAAACATCACTCTTGCCCCTTGCACCTATTGTGTAAACCAAACGAATCG
CATCAAGCCACCAGTCCAAAGATTCCCATGAGTCTGCGATCATGTAAACTGGCCCATGAATCGTATCAATGC
AGAGGCCTGCACCAGG
>TCONS_00023030
CTCGTATCTGCTGAATTTGATGAAAGGCTTCCTGACATGTGTCGTTGATCAACTTTACTGATCTGTTGAAGTCC
TTCATGATTCAAAACAAACTGAGAACGCTTCCAGTCACTGTGTGGTGCAAGCGGACTACCAGCAGGTGGTGC
AGTTAGATATCCAACCTTTAGGTAGGGCAGAGGTAGCATAGATCGCACAAGCTTCAAGGCACTTGTGTAAAC
CGAGTAATTCGGTTTCAGTGCATGTGCAGCGGCAATGTAAATCGCAGACTGGCTGTACAACTCATTAGGAGA
AATCTCTTTAACATTACCAGCTCCCCCTGTTCTTGACATGTCCTCTGCTAATCCATACAGAACAGTTCCAAGGT
TGTAGATTGCCCTATGGAAATCAAATTGCAGTTGGATCGCTGCACGAAACTTACTAATTGCCGTTTTGACAAT
TGTTAGCTTTTCTCTGGCAGGAACGATTGCACTGAGTT

>TCONS_00023032
CCTGCAGGGCAAGTCCCCAGTTGTTCAATGCCTGTGGACTGTTCCAATTGAGTTGGACAGCCTTTTCATAGTTC
TTTGTTGCCTGCTTCCAAAGTTCTTCAGCTTCTTTTGTACGACCCCGGATTTTCGCTCGATCAGATATTGCAATG
GCCCAATTATAGTAAGCATCATTGAGCGTAGGACAAAGACGGGTTGCCTCCTCATACTTCTTGCATGCCTCTT
CCAGCAAATCATCTTTTGAAGGTGAAGTGGAATCTGGGTTTACATTGTCTGCACTTTCCTGGAGAACCAGTGC
CCAATTATAAAGTGCATCATAATCTTCTGGATTTCGCTCTAGTGCACTAGCATACCTG
>TCONS_00023033
ACCTCCGGGCAGCATATGTCAGAATCCGTTGGCGAGATCGGCCCTCCTCGTCAGCACCTGTGACGCTGTTAAT
CAACTCCAAGGCAGCATTACTGTCTGTCTGCTGCTGATTGCTCTGTTGACTGTGAGGAGTGTCGCCTTCTCGTC
TTTCGGCTTCAGAATCATCATTAGCATCGCCATTCTTCAATTCATCCAACAACTCTCTCATTGTAAATGTTCGG
CTACCTTCATCTTTCTTTAGCTGCTTCAAATTGCT
>TCONS_00023139
GAACAGAACAGAAACTCTGTAGTCCATTCTTAAGAAACAGAAACAATCCTCTCCGACTTAGTTCATTTCTTCT
CCGTGTTGCTTTCCAATTCCCTTTTGAGCTGAAGAGAGAAGTCATCATTGACATCATCATCATCCCAATCATCT
TCCCATTGCTGGGTAACTTCTTTCCCTTCCTCTTTCGCCTCCCACTCTTGATCAATTTCGAACTCTTCAAATTCAT
CATCATCCTCGAACAGATCCATTTTCACGTCTTCAGTTGCTGGCTTTGGTTGTTCCGTTGCCATTGCTATTTA
>TCONS_00023289
GTGATGAGCTGGAGTTGGAGCTACCATCTTGAGCATCTGTCTGAACACGGTGAGAAAAACTGTGCCCAAACG
ATTTCCATAACCTTGAAGAGAAAGAAGGCGGCAACTCTCCCATGTTCCCATCACATGGCATTTCAACTGGCTG
TGCTGAAGTGGTTTTCAGCTCTGAAAATCTTACAGCTCCTAAAATGCGTTCTGGTAGTTCTGATTGAGTTTGGC
TCTCAATAAGGAAAGCTAAATCAACAGTAAGAGTTGATAGATAATTAAATGCGAGATGAACAATTGCATTTG
CTATCACGGAAGATCCAATATCAATATCTACTTCTATGAAGTCATCTGCTATACAGTAGTTACATGTCAGCGC
ACGGCCAATTACGCATATAGCTTGCTCCCCAACTGCCTTTCTTACAATCCAAGGTCCTTTAACAACATTTGCTA
TCAATTTAAGCCTTCTAGTTCTAAATGCATCATCCCCTTTCAAGAACTGCTCCATTAAAGACCCTTCGGGGAC
AGGCTCAAGACCCACAAAATATGCAACGGCACTATAATTTTCCTTACTAGGAACTTGCAAGTTGAAAGCCCA
AACAAAGGGTTTGCAACCAGTTGGATTTTCCTCTTGAAGAGCCCTGCTGATACGGTGTGTTGGACTATTCAGG
AGACCAGAAATCTTTTTGGGACCATGAAACTATTCCCTGGTGGAGAAGCCCAACCATTTGAGCAGTTATCTGG
TTCCAGAAGAGGAACGGCACCTTCTGATTTCACCCTCTTTATCCATTCAGGTTCATCATCACCATCAAGATTGG
CCATATGCAACGAAAACTATCTGTACG
>TCONS_00023307
CTGGACTTGATTACATCAAAATCTAAGCAGTCTAGCAAGTCCAGCAAGTTATGTTAAACGATGACTTCCAAC
GAATAGCAGTAAAATTAGGTGAACATATTGATATTCATAGGGACACATGTACCAAGCAGTTGAGTAGCTAAG
AAAATAGAATAGAAATCCACTATCAGAAATCAAACTGTGAAGAATTTTATACAGAATAGAAGGTGAAGCAC
AGGTGAGAAACTGCAAATTATGAAACTATCAAGATTCCTGAGGCATGTCTCATTCTTGTCCTGCTAGTGAATG
TCTTCTAGGTATCTCCTTTGGTTCTGCCCTCTCTATTTCATTATAAAGATTCCTGAGATACTCTAATGCTTATCCT
CCTTAGGAATGTCTTTGTGAGGTATCTCCTTTGCTTTTGCCTTCTCCAATTCCTTGGCGGCGGAGGAAGACTCTT
CCTCGAGCCAATGGCGAATTTGACGGACGTTACGCTTGAAGATGGATGCAGACTGTTTGACATCGCTCCTCAG
CAATAATATAGCCGCGCTCACTCCGACCACCGTTAGTATAAAGTTCGTTAACGCCATTT
>TCONS_00023349
CTGCTACGGAGAACAACTAGGATATAGTATTATCTGTGGTCTTCTGCCAACTGTAGCCATTGAACTCTATTTTT
TCTGTATTTGCTTTAGAAGCCCAGAGACAGAAGCACAATGTGTTGATTTGTATTCACATTGCTCACAACAGTA

CTTTAGTGCTTCGATCACCTTTACACACCTTTGAGAAAGGAAATTGTTCTTGGAAAGGCAAGCTTGAATGTCG
CAAGCCTGTTTCTTACATGGCTCTTTACTCGGC
>TCONS_00023432
CTTTGGAGCCAATAGCTAGGGCATCAAGCAACTTCTTTTCATACAATTACTCGAAAATTGACCTCGCTATATA
AAGAGGTCAGAGCCATGACATCCATGGAGTATTAGACATTTAGACTCAAGGAAACCATGCTGAATTTACAAA
TAGCAAAAAGTAAATGAAAGTCCAAAATCAAGCTCGCGCGTCCAACATCATGCTCCTTGTCCACAAAGACAC
AACTGTTGAATTTCAACAGTTGATTTACCAAAAAAATCAAACTTCTTCCACAATCCAGCTGACAAGAAATCA
TTTCCCCGTGTTCATCATCAATGTGATATTATCTCCTTTCAGAAGAATCCGCCCCAGCTGCTTTCTGCTTTTCTT
CTTCACATTGACTTCCTCAGCATCATCTAGAACCAAATTCATGTACTCATCAAAGCCAATAATACGTCCTTCA
ATCCTCAGATCCTTCTGCTCAAATAGCCATATCTGAATACGAGCTTTACTCTGAAGAAACCTGAAGATCAGGT
TAATGGGTTGGGTCATAATCCTCTGTACTTTGGTGCTAGCCATGGCTGCTACTCCTGTTTCTCTCACGAGTCACT
CTTCCTCTCTGATCTCACGGAATGAAGCAAAAACCTAGTTAGGAGAT
>TCONS_00023461
AACTTATTCAACTCTTTTGTGGTTACACCACCTACTCTTGCATAGTATGCATTGTTATAGAATGCATCATCAAT
GAATTTTGCTGCCACCATCACACTTGTGATTAGTAGTCGGTGAACATTGAGGGAAGTTAAATGAGCACTAGTG
TGTTCAATGAATCGCTCCATGTAAATGTGTGCAACCACAAAGCATGAAGGGCTGCAACATGAATACTTAAAA
ATGCGATCCAAGTATTGTTCGATGCCGAGCGACGGGGTCCTTGATCCATGAAATAATGTAATCACATCTTTTC
TTTGAGTACTTTCTAATAAGCTTTCGGTTTTTTGAACAGACCTCTCAAGAAGTGTTGAAAGAAGCGATAGAAC
TCTGGGTTTTCCTGATTTTTGCTTGCCAGACACCTTCAGCCCCAATGCTAAGTATTTTTCTGAGCGTATGACCTC
AGATTCAGGTGCCAAAGTTCCC
>TCONS_00023512
AATAAACTACAAACACAAAATAGAGCTTGTTGTCTATATGTTTTCACTACTAGAAGGCTGAAAGGCATCTAG
GATTTTCAGATCATAGTAAACACATGAATAGAGAAAAATTTATGTGACACGAGCTGTTCAAGTGGCAAAGAG
TTGAGCAAAATTATGAACTATCATATAATCCAAACAGCTAGCAGCAGGCTTAGCATAGCTGTATCATGCCTTC
TTCTTTTTGGACTTGAGCATCACATATCCGATAAACATGCTCAGCAAGCAGATGAGTGCCATTCCAGCAAATA
CGGGAATAAGAATCGCATACTCTTGAGGTAGAAAATATTTGTGCACAAAATGATCAGTATCAACCAGTGGCA
GAATGATGACCCAGAAGGTATAATATGTGAAAATAGACAAACTAGTCACTGTCAAAAGCAGCCCAATCGTT
CTGTCTGCTAATTCCATCGTGACTCGTTCGCTTGTGCAGACTATAATCACTGGACATCAGCATACAGCTGTAA
AATAAAT
>TCONS_00023526
AAAAACACACAAAAATAACTCTTCAGCAGACCACCCCTGAAGCCGCCGACCACCCTTCACCCATCGAAAGC
CCTCGGTTCTTCATCTTCTTCAACACTAGAAGAACCAGACGATCCCAGCCCGCCACTATCAGACCTCGCCTCC
GTTCCCACCCTAACGCCACTGTCTCACCAGGCAAACGACCCTCCCCACGCCGGAAAACACCCATAGCTGACC
CTTCTCCACCTTCTTCTCCAACCAGATCAATAAACACAGTAACGCAGCCAGGAAGACCAACCTAGTCAGCCC
GCCTCTACACTGTGTTCACCCAGTCAAGTTCTGTCTATCTCGAGTTTTCTGGTCGAGCTTCGATAACTGAGTTTC
GA
>TCONS_00023559
ACAAAATTTACTGGGTGTGAATATACAATAGATACAGAAAGAGCAAAAACTCTGCTCAAAAACTCCATTAA
GAAGTCCAAATTTATTGTTTTCATTTCGTCATCCAGTAAACCAAAATGAGAAACATGCATACCGAGGCAACTA
ATGAAAGAATGATAGTATCCATAGACTTTTTCTTCTTTATTGCAGAAAGAATATGATTAACTGTTGGAAGGCG
ACTGCTGACATTGCTCAACTTTGAATTAATGCCACCAAATGTTGATCTTTGAAACATAAGTGTCTTTATTGTTT
CTTGAGCTTGAGAAATCACACCATCCATCTGTCCAGTACTCCTATGGAGGGAAGCACGTTCATTAAGGAGTGC

TGCTCATGAGACCCACTACCATCTTCCAGATCTAACCTGGTCCGATCGAACTCCCTAAAATCTTCAAGCAATG
ACGCATGCTCTTTCTTAGCTCTGTAACTTGAACGGAGCCGATTAAACTCCTGAAAAAGATCTTGAAGAATTTC
TTGATGCCGAGTCAGCGTATGAGAAAAGATCTCAGATCCTCCAGATGAAACCCATGCTTGCATTTGCGAATTT
ACTTGTTGAAGCTGCTTGAGCAGCTGATCTATACTGGGTTCAAGGTCGTTATCATTAGCATTACCAGTTTTGTT
TGAAACGAACTTACGATATATATGCATCTGCTCATCCAGCTGAGCTTCAAGCTTCCTTGCCTAGTTGGATGTGT
AAACCAGCATAAGGAAATTTTATTTATTAAAATATATGAGGAAGAAAAAGAAACAATTACAGATGAGATTTC
AAGAAAAAATTCACCACATGACCAACAAGTCAATTTTCCTATGTGAACCTAAGCTGAATCAAGCAAATTGAA
AGAGGATCAACTAAAATTTACCCCTGCCACATGCAAACAATTTGTCATGATTCTCGCCATTTCCACACCCTTA
GAGAACTCTCAGGTAATCTTATCATAAGTTCAAATGAAC
>TCONS_00023793
GAAGAGGAAGGGCAAAAAGAGAGACCTAAAATAAATGAAAACATGTTAGAGTTCTTCCGGGTTTGCATCTA
AACAGTTCCAAAAAACTGCTCCAAAGAGGTTGAGCTGAAGACATTCATAGCTGCTCTTTCTGGCTTGGTAGGT
GAAATGTGTAGCTCATATTTGATAAATCTCGCCAATTGAAAAATGATTTCCGGATTTGTATCTATATGCCAATC
AGGTTCCAGTTGTCGGACAAATGATGTTCGCCCATTCTCAGTGCTACAAAAGAGAACCTTGTCTTTGACCATG
CTTAACACTCCAGCATCTTCTAAAGCCACCAAGATGTTTTTTTCACTTTCTTCATCATGTACTCTCTCCATGAGA
TAAAGGTCACAAAATTTGGTGATTTCAAGCAACACTTCCAGCACGGAGGATCGGATAGTTGCTTGTTTCTGTA
GTTCCTCTGGGCTAGTTTCCTCCAGGATTACTCCAAGCAACCGACATGTTACCTTCCTCCCCTCACTCAGTCTC
TGCCTAACTATTTGCCCGAGTGTCGGCTTTACTGGCTGAAAGAACTCATCAATAACATTTTGTGTACTTGAATC
CTCTGAAGAAGGGCTAACTCCTGAAGTTGCTACATTTGCACTTAAATGACTGTTTACACCAGAACTACCAGGT
GTAACAGCTTGTCGCTTAGGACGCCTCCTCTGTGGTCCACTAGGTGATCTCAATATTCTCCATGTAAAAACTAT
TGCAAATGCAAGACCTGCTATAGCTCCTACAGATCTCGAATCCAGGTTTTGGAAGAGATTAGAAACTTTGAG
GGTGAGAAAAGCCCCAAATCGCTTCATGAGCTGGAAGAAATCGTCCTTGGAATTATCGGCCATAACTTGCTG
AAGACTTCAAAATTGATATAGAATTCAATGATTCGACGTGGTAGTAAGTTGGTTTTGTGGAGAGGATGAACG
GCTGATCAAGCTCTAGGGTTCATCTTCATTGCTCTCTACTCTTCTAGAATCTTCTAGATTGAGAGAAAGTAGAA
ATGGATCTTGCAGAGGAAGCTTCGAT
>TCONS_00023840
ACAATAGAAAGAAGATCCTTTGCAAACTCTTTCTCATACAGCGGGGAACCAACTTTTGCATCAAGAATTACTC
GCCGGACAGAACCTGATATCAACTTTAATGCATCTTCAACTGTTGGAACAGTTAGATCATGATGCTCCGACTT
AAGCTGATGAACTGCATCAAGCTCTTTAATCTCCTTTGAGCTTAAGTGTGAAACTTTAGAATTGTTGTTCCCAG
TTATCCGTTGCAAATCCCGGTCATGGAGGGCAAACAACACACCATCTGAAGAACGAGAAACATCAATCTCA
ATGCAGTCAGCCAGAGAATCAAGAGCAGTTTGGTATGCAGCCAT
>TCONS_00023841
GGGAAAGGCCTTCGATGAATCACCACCATGAGCACAAACCAGAGGATGACTGCGCAGCCAACTACATTTCT
GTGATTGCATCTGATGGAATCGACGGAGACGAAAATGGAAGTAAATAGGGGGAATAATGGCGATGAGGGCG
AGGCCAATGAGCAGAGATCTAAACACCTTTCTGGAAGTGAAAACTCTGAGCTTTGGCTTAAGCTTTCGGGAC
AAGAGGCCATGCCTCTCCAACCTGAGTCGACGCTTTTGCCTGTCCATTCTCCACAATGGCACCCACCGATTTG
TCCAATTCAACGGATTCCC
>TCONS_00023906
TAAAGGTATAGATTGAGTGGGTTGAGACTCATCTGAGCTTTCTGGGGAAGCTGCCTTTTTTAGCTTGTGATTAT
TATATGCAGCTACACCTGCAATAGCAATAGCATAACCAAAAAGATTAATCAATGTCAATTTTGTATCCGCAA
AAAGTAGGGCAGATAGCAGCACAACGACCCAATCTTTGACAACTCCAGCAACACGAATGGTCAGGGCACTT
GTATGTTGAATCACCAGAAAAACAGACAGATTCAGGGCAAAGGTACATAGAGAATTTAGGGTTAGAATAAG

AGGATGGAAGCTCCATGTCCTCTGTTCGTCCATCTTTGGTTTCTCCAAAAAGATCCATGGAATTAACAAACAA
AGAGCGCTGCATGGGCTGACATAGTACATCACAGATATGGGGTTGAGCTTCAGGCCCTTCCGTTTCACCAGA
ATCTCCATAAATATAAGCCTCAAAGCTTCTCCAACGAC
>TCONS_00023907
GCGAACATTGCACCAATTGGTATGACTGAACTCATGTATATATCTAGAGTCATCCCTTCCTCAACTTTCATTAT
CTTCAGAACCTTGGTAAGCACAAAACACAAAATTGAGGAGAAGACCATGTGAAGTAGAGTCAATCCAAGAG
GATAAGGAAAGTTTATATCTTTTGAAGACAAAACCCACTTGTTGAAGAAGATCTGGCCACTAGAGAGTGCAA
TATAGAGGAGAAGATATGAATATGTTACCATATCTTCGCTCACCCGTTTTCTCTGCGGATCCGCCATTCCTGCA
CCCCTCACACTCGGCTTGAATTCACAGTGGGACTTTGATATTTGAAAAGAAGTTAAGTGTGAAATGGTGGAGT
GGAATGGTTAAAAAGTGTGACGAGAATGAAAATACGGAAAGAGTGCTCAGTGTTCCGACTTTTAGCGCGCCT
GACTTGGTGGATTG
>TCONS_00023912
CATAACTCCTATAACACCCACTACTCTTATCCATTATCTCCATAACTTCCAATACTTATGGTTGTAACTCCCAT
ACCTCCATGATCTCCCCCATGATCTTCCTCCATGATCTCAAAAGCTTAATGACATATTTATGGAATTCTTTTGG
TATACCTCTATGTAGTACTATAAATAGAGGATGAGAAGTCATTTTGTAATACACTTGAATACATGAAAGAAAT
AGATTCTCTTCTCT
>TCONS_00023917
TGGCATATTTGTGGTAACAATCTTCTGTTCCATTTTCAGATTAACGAACTAGGGAGAAGAATTACTTACATCTT
GCACAAACCGCGGTGTTGGGTTTTAAGAACATCCTATCTAAAGTCCGTTCCAAGTTCTCATAGCTACTATGAG
CATTCAGATCTACCTTCCTTCCAATTGCAACTCCATCCATATTGACCTTCACAAACATGGAAGTCTTGATGAGC
CCTTTCTCCTTGGCAAAACTATTGCTCTTGCTGCTACTCGCATTAGCGATAATATTTTTGCTTTTGCATAATTCTT
CTGTGGGTGGTGATTTTGTCTGGTTAACTAGGGTATTCATCCGATAAGTCCTTACAGGAGGCCATCCAACAAC
CTGACTGACACTAGAACGGGGAGGTGAAATAGAATCAGCAGCTCTCTTGGTGCCAGAGGAAGCATTATTATT
ATTAATTTTAGTAGCTGAAGAAGAGGAAGATGAAGAAAATGAATTGGAACCCAAAGAAGGGAAATTTTTAG
CCGTCAAGATTTTAGCCCTTGAAGCTCCAGTTTTATTAGAACCAAGACTTAGTCCAAGACCCAATTCGAGCTC
AGACTCATCAGGGTATGATGAAGAAGAAGCCTCAGATGATAACAACACCATATTATCTTCCTTTGACACTGTT
GAAATTGACCCATCAGTTCCTCCTCCACCAT
>TCONS_00024075
TAAATTATTAGGTAGTGATATGAAACAAGTCCAAAGGGAATAGAAGTGAATATTGAGGATTCATATATTGTTT
TTAACCAGTTTGATATTGAAGCTAATTGCTGAAGTTATCTTCACAATTTTCCAAACTTTCAGGCCTTATTTCTAT
CTTTGAAATTCCAAGTTTTCAGAGGCAATGAAGATCATTCATATAATGAAAAGGAAAAGTGGAAAAGATAA
GAGTTGATTCCTTGCCTGGCTTGGCAAGACCGGTCTTTCCTATAAGGCAACCGATGATTGCAGCCATGGCAGT
GTTCACTCTGTTATTTCCTTTGGTTAAATAGGCAGAAATCTGGGGCTTATCCTCAACTTGGTTTATTCACATTTT
CTTTTTTTGACAAGTCTTTCTGGAACTATTCTAGACTAGAAGTATTTTCGTGGTTCTTTTTGTTCTATTATTTGGA
ATTCAGGTTCTCCGCTATTCTGACTGGTTACTGGTTTATTACAGATTGGAGAGAGTTATGGTATCTCCAACATC
CCAGGATCATCTGTCTTCATACATAGCCAAATTCTAACTTGTTCCGTGCTTTGAACAAGTTTTAAGCTAATTCT
C
>TCONS_00024080
GAAGTTGTGTGGAGAAAATGATAGATAAATAAATAAATGATGGGGCTATGATGAAATTCTTCTTAGAAAATG
CAATGAAGCATTCAGATAATTTGCGCCGTAGCTTGGCAGGGCGACCTGGTTAGCGTTTATGGTAACATGCTGA
AAGTTTGCAGCCATCACGGTGTTGAAAGACTGAATGAGACTTGAACAAGATCTGAGCCTAAAATGTATGGAT
TATTTGATTTTGTCTCTGCAGTTGACTTGCTGACTAATCTGCTGCTACAGCAGTTGAGTTGCACAAATAGCCGA

TTTTATGACCCCTTGTATTTAGAACAAGGTAGAAATGACACAAGGTAGCCACTTTAAATGCTGCTAATAATGG
GCTATGATGATTTGATTCTTTCTTTCTGGTCTATCCTTCACCAGCTTTGAGGTAATCTGTCCCGTATCTTGGCGG
GGCAACCCGGTATGGCATCTTCTGCAAACCGGTTTTGCAGCCATGGCATGGTCACTATCTCTTTGCATGTTCAT
AAAAGCAAAATCTGAGCCCTAAAAGTTTTACGACAACCATTTGATGGTCTTGAATGATGGTCGATCTCTGGA
ACTGGAAATCCAATTGGGTTCTTCAGTTTTTTCTTGAACACCAATTGTCAGATGCAGTTCTCCATCAATTTTTTT
GCTCAAAACTGAGGAATCGGAGAAAAATGGGGATTTCAGGGTGGTTGACCATCTCCGGCATGGCAAGGATTT
CTATGTTGCTCCAGACGAACATCCCTTTCTGAGTATGCATGCATCTATGTCTCTATCTTTGTTAATACTTGTTAA
TACTGTGCTCCTGCGTGGCTGCGTCTTGAGGCAAAGAAGGTTAATTCTTCTTCTT
>TCONS_00024081
GAGAAGAAGTTGTGTGGAGAAAATGATAGATAAATAAATAAATGATGGGGCTATGATGAAATTCTTCTTAGA
AAATGCAATGAAGCATTCAGATAATTTGCGCCGTAGCTTGGCAGGGCGACCTGGTTAGCGTTTATGGTAACAT
GCTGAAAGTTTGCAGCCATCACGGTGTTGAAAGACTGAATGAGACTTGAACAAGATCTGAGCCTAAAATATT
TTGTCTCTGCAGTTGACTTGCTGACTAATCTGCTGCTACAGCAGTTGAGTTGCACAAATAGCCGATTTTATGAC
CCCTTGTATTTAGAACAAGCTTTGAGGTAATCTGTCCCGTATCTTGGCGGGGCAACCCGGTATGGCATCTTCTG
CAAACCGGTTTTGCAGCCATGGCATGGTCACTATCTCTTTGCATGTTCATAAAAGCAAAATCTGAGCCCTAAA
AGTTTTACGACAACCATTTGATGGTCTTGAATGATGGTCGATCTCTGGAACTGGAAATCCAATTGGGTTCTTCA
GTTTTTTCTTGAACACCAATTGTCAGATGCAGTTCTCCATCAATTTTTTTGCTCAAAACTGAGGAATCGGAGAA
AAATGGGGATTTCAGGGTGGTTGACCATCTCCGGCATGGCAAGGATTTCTATGTTGCTCCAGACGAACATCCC
TTTCTGAGTATGCATGCATCTATGTCTCTATCTTTGTTAATACTTGTTAATACTGTGCTCCTGCGTGGCTGCGTC
TTGAGGCAAAGAAGGTTAATTCTTCTTCTT
>TCONS_00024127
GTCAATGTCTGCCTTAAATTTATATTTGAAGGGACTATTTATGTTTTCTTGGTTAACTGTGGTATAACTTGTTAA
TATGCAGTGTGCATTTAGTGGAACTGTGCGGAACTGGCCAATGTTTTGCTATGAAAGCAATGGATAAGAGCAT
AATGCTTAATTGAAACAAGGTGCATAGAGCTTGTGCAGAACGAGAAATCCTAGATATGTTGGACCACCCTTT
TCTTCCCGCACTCTATGCGTCTTTTCAGGTTAGAGACATAGAACTTGATGAGAGGGGGCATAATTATGTAGTT
GTAGT
>TCONS_00024147
TTTAAAGATTGAACCCATCAAAGTGTACATTATGAATCTGCCTCTGAACCTACCCGAGAATAGTGCATATTAA
GTCTATTCATCGAACGTACTCATAAATCCACCAAGAAATCCAGCACCGTTGCAATCACCACATAACATATCTT
TTTTACCTCTGCACAACCAACATAGTCCACCAGCTTTGAACCGCCCGTTGAAATGATCTACAGAGTTAACCCC
AGAGCCTTTGCACTGTGAGCACGATACTGCACCATTTCCATCACAATTTTGACAGACTATGCTCCTTACTTTCG
TGTCTTTGTCACTGTCAGTGGCCTTCACTTCCACAACTCTCAAGTTAGTCTTGGAGCTGTATGAAACATCTTTG
ATCCATTGAATCTTTCCTCCGGTGCAATTCCCATTAATCAGA
>TCONS_00024238
TTCTGATGAAGCTTAACTTGCAGTACCTTTTCTGTTCATCACATCATGATAATACAGAAGCACGCACATTGGTT
GACCAAGAATGCAGAAAAAGCACCAGAATGTCATGTTGCCCACATTCGAGCTTTTGAACTTGTTTTGCAAGA
AGTTCGTGAGTATGACCAAGGGAATCTGAAACATGATTCCAAAGAAAGCCCAAAACTTGAATAGTCGGCAA
GGAACAGCAATACACAGCTCGTGGAAAACAGCAGATACAAAGAAAGAAATCAACATCGCAACCCCCTTAG
GTATGCCATTCCTTAAGCATGGGAAATAAATGTGACGCACCATCCACTTATGTACAGGCATATTCCAGAGTCT
CCAATACTCATCAATAGTTTTTGCATTCCACCAATCTTTGTAGAACTCACGATCCCCAAATCGCAGAAGTTCC
CCAAGTATATTTAGCCAAAGATGAAAGAGGCAGTAGAACATGCAGAGCCAGACATATAAATTTGGAAGTGA
AAGCCTCAATACCCTCTCGATGGCGTATAAAGGTCTCCTTTCAATGGATGTTGTGAGTTTTGCACAATTGGGT

TAATATACTGTTCAATGATAAATCCCATAAATCCTGTAAAAATTACCAGCTTGATGAGTTGGCGCGCCAGCCA
ACCCTTCCGAATGCATGCAGTGCGAGGATAGCTAAGCTGATAGCACAAAGTTGGAGCAACCATGAAGTAAG
CCAAACTCTTGAAATTAACATCGTATGAGTAGTTGACATCTGAATGTTCACCCT
>TCONS_00024273
ACAAAACAAGAAAACCAATTTACATTCCATTAATTCTTCATATTTCAAAGAACAAAAAGCTCCCTAGTAAAA
GAAATCTTCTTCTCCCATATAACTAACTAGTTCAGACAACATATGACAAAGGAAAAGAAAAGGAGGAATAA
ATCTCAATATCGGATCCAGACATCTGACTGCACTTTAGAAAAGAATGAACAGAGCAACCCATACACTTCAAG
TAGACACGGAAAACACCCTGCAGGTGCAGTCTATCTAGTAAGGCCACCAATCTGGCTTCGTCACCATGATCT
GATTATACTCCGGCCTTGCAGTCAGATCCTTGAAAATGACAAAAGCTACAACGAAGAAGAGGACGAAGAGA
ATCTCGAACTCAGCAAAACGGACGAAATCGAATACGGAGTCTACAGCTCGAGTCAAGATCGACGGCGACGG
TAAATCGTCCCTTCTTCCACCTCTCCCTACCCTCTTTCCCATTCTGTCTTTCCTAATTTGGAAATGGACGAACGC
AAATGCCCAGCTTTTGATCAATTT
>TCONS_00024510
ACTTCCCTAAACAACTTCTGCCTGTCCATCTCAAATCAGGTATCCTCAACTCTGGAATGAAAGACAAAGCTCG
GGAATGTAGTCGTAATGTCCTTGAGATATTGGAGAGTCATATTCTTCAAAAGTGAAGGATGTTGCAAGACCA
AACTTCTCCATTCCTCCTTATCAATCTTTCCGTCATGTTTAGTATCGGCTTCTTCAAAAGTCTTATCAATTATGC
TCTCAATGACATCATCTGATAGGTTCATACCAGACTCAGCAAGGGTAGCAACCACCATTTGCTTCACCTCTTG
CCTCTCAATGAAGCCTTGTTGCTTGAGATCGTATAGCTGAAAAGAAAACTCAATCTTATCATCAATAGGAGCA
TTTGGATGGAAAACAGAGAGGGCACGAGCAAATTCCTCAAAACCCAAGATTCCATTGTGCTTTGTGTCAAAT
AAATCAAAGACCCTGTCGGCAAACAAGCTTTCCTTTTTGATCGTCTTGAATAGTGCCAATTGAAATTCTTCCTT
GTTGATCAGCCCATCATCAACCACTGCGCTGCTGATCTTCTTAAATAGCTCATAAAGAGCTTCAATCTCACTG
ACGCTAAAAACTGTTTCTCGTGCTAGGATCTCCGGATCTTCAAAGCCCGTTGATTGCCTATCCAGATCACAAC
ATTTCGACAGAGAAGCACCTAAATGCTTGATACCGTCTAGGCACTGCAACATGGTATGATCCCTACTTCAATA
TGCTCCATATCCTTTGACCATTCTCTGTTATCGTCCTATCTGAATGTAAAATGTTGCGAGTTTCTGAAAATGATC
AATTTGGAGCTATATATCTGACAAGAAATACAAGTCAATATAAACTTAATTCTATCAATAACCCCTCAAACA
AACAAATAAAA
>TCONS_00024528
AGATGGTGGCTGAAATTTTCAGCCAAGTTTTCCTAGAAATTTCGCCCACACGAAATTGCTATTTCCGGTATTAT
TTGGGTAACACAGTAGAAGACCGTGGAACGTTCGAGGATCACGATTGTGCGGGTGATGGAGATTCCATTGAG
AAGTTATGGAACTGAAGGCTCACGTGGTAGAAGAGATATGTTCACGCTTCAAGAGGTAACCCGTGAAATCTC
GTTTATGCTAGTTGTCTAGATTACATGTGATTTTGATACTGGGATTGCTTCCGCTGCTTATTATAATCCATCAAT
TAC
>TCONS_00024747
ACAAAATCAAGAAAAATTACAGAAAATTCAAAGATTCAATCAAAATAGAATAGCCAAAAGTGAGAACAAG
ACACAAATAAGAGGCAAGCAAAACCTTCTAGTTAAGCCCCTTCTCAATCGTATTTTAAAAGGAATTTTTTTAA
ACCCTAACTTAGAAACTGAAATTTACCATTAGAAAAGTGGAAACAAGATGGAAAATTTCAAGTATAATTTTT
AAAATCTTACCCAGAAAAAGATTGGCAGCTGCTTGATGCCTTGATCACTGAAGAAAGAACGTGTGAATCTGT
GATGGTGAAAAGGTGGATGAAATTTGTTTTGGTCGTTTGGGTTTGGCAGAAGGATGTCAAAAGAGAGAAAAG
GGAGTACTGCGTTGAAAT
>TCONS_00024905
GCTGTCATCGTGATCCAGAGGAACCACCCTCTCATCCACCCAAAAAATCCACCATTTCGACCAATCAACTGA
ATCTTTGTAAGGCTGCTCCACCAGTTTCCTCATGGTATCAATAAGAGTGCCTCCAGAGAGCACAACAGTGAA

AGAACCTTTTTCTTTGATGAATTTATCAGACAAATCAGCAGTGTATTTGGCGAGTGCGTTTGCTACATCTTCTT
CAGAGTCGAACTTTAACACCGTCTTCTTCCCTTTCTGGGTCGCCATCTTCGAAGCCCAAGTCTGTTATTTCCTA
AA
>TCONS_00024917
ATCGGAAAGCAATTTGAAGCAGCTAAAGAAAGATGAAGGTAGCCGAACATTTACAATGAGAGAGTTGTTGG
ATGAATTGAAGAATGGCGATGCTAATGATGATTCTGAAGCCGAAAGACGAGAAGGCGACACTCCTCACAGT
CAACAGAGCAATCAGCAGCAGACAGACAGTAATGCTGCCTTGGAGTTGATTAACAGCGTCACAGGTGCTGA
CGAGGAGGGCCGATCTCGCCAACGGATTCTGACATATGCTGCCCGGAGGT
>TCONS_00024961
GCGATTTCATGTGTTTTATCAACTTTTTGGATTATTAGTGTTAGAATATTATTATTAATCTTTATATTATGTGATG
TATGAATTCATGTTCGTTTTGTGGTATCATGTCAATTGTCAATAGCTTCTCCTAAAATGCGAAGTCTACCTTTTG
ATCTATATAATGAATGGCCTCCATCTTCTGTTGGAAGGTAATATCTCTATTTCTAGACAATAACTCTTATTTCAT
TGCTAAAATATGTATTTTTGGTGGTTAAGTATTGATGATAATAGTGAAGATATATTAAAGAGACGACTCGTTA
ATACTTGTGGAACCTGATTGTATTTGATGGTCATACTTGAATACTCGTGGAAAGTTCATGCTACTCCTCAACAG
ACAAGCAGTTGATTCGATGTGCTTAATCCACCGATTTATTGTTTGAGAAATTTGAGAAGCCTAAAATATCAGG
TTGGTAATTTGAAGGCAACTGATTCTTTATGTTTTCTCCCTCGATTGGGACTGTCTAAAAGGTAAAAAATGATG
CTCACTTTGCACTTGAAACATGTCAGCAAGCTGCGACGCTCAGGATTATCATGATAAGAAGTTGCTATTGCCT
CCTCAGATCTATTGCATCAGGTAAGAACTGAGCCTCAAAATTTCAGACGTTATTAACAAGAAAAGAAGATAA
CTATCATTAGACTGATGACATATTAATGTGACCATAAATATGTGTCATAGATAAACTGTTGCACATATTTTTCT
GATTCTTATGAGGCATTTCTTAAACTTTGGATACATGATTCAACAATAAATGAACTCCATATTCAATATTCAAG
TTTCGAAAGACTGCAAATAAGGATATTGCAACTTCTTGAAAATGAAATGGAAACACTTAGCAACAAGAGCTA
TGCCTTATGAGATCAATCAATTTGTCATCCTTTTTTCACCCTCTTTTTTTGTGACAAGTATTTCCTATAATTCATG
GCTATATCTCTGATGCGAAAAGAATTTGAGACTTGTTCCTTACATGGGATAAAGTTTTATGGTCTGTCACTTCA
AGTCTGTTGGGACAAATGTTGTAGCAGGTTATTATTTGTAAATCTTTTCTGACTCTTCAAAGTGATTCTGAGTT
GTCAATATCCTTCTAGTGTTTTCTTGGTTATTGAACATAGTGCCGTAAGATAATTTTCTTTCCTCTACCCATGCT
TGATTCCTCTTTGTTTCCAAATGCTTTTGGTCTCAACAAAAAAGGACCTCTTGCCGTGCACACTCCTAAAATCT
TCTGCTGATGGCACTTCAGTTGATTCTAG
>TCONS_00024962
GTTTGGAAACAAATACTTAGGTGCGTGTTTTTTGTTTCTAATTTTGGTTGTGTAACGTTGTTTTTAGTGTGCAAA
TTTTATTATTTGAAGTTGCCGATTGAAGGTGAATTCAAAAAGTTTGTTTTTCTGATTTTCTTTAGATTTGCTTTCT
TCGTTCTTAGAAAAGTGGATCTGAAGCAGTGAGATGCTTCTGTTCTCCAGTTTTGTTCTCTCTCTTGTGAGTTTT
ATGTAATATGTGATTCCTATAAATGACTCTCACTATTACTATACAGTTGTAGAGTGATGCACCTAAGGTGTTTG
ATAAGGAAATCTTTTGCGAGGCACTATTTGGTTCTTGAACTCTTCACTATTTCAATGTATTTCATGCATTGCAA
CAGACATGGGGGTGGTCTTCTGTTTTGAAACCATTACAAGCTTGAACCAATTACCTGAGCGAGAAAAAAATC
ATCGGCGAATTTGAGAGGAGCTGGCTCAATGAACAGGAAAACTACTATCAATTGATAGACAAGCAGTTGATT
CGATGTGCTTAATCCACCGATTTATTGTTTGAGAAATTTGAGAAGCCTAAAATATCAGGTTGGTAATTTGAAG
GCAACTGATTCTTTATGTTTTCTCCCTCGATTGGGACTGTCTAAAAGGTAAAAAATGATGCTCACTTTGCACTT
GAAACATGTCAGCAAGCTGCGACGCTCAGGATTATCATGATAAGAAGTTGCTATTGCCTCCTCAGATCTATTG
CATCAGGTAAGAACTGAGCCTCAAAATTTCAGACGTTATTAACAAGAAAAGAAGATAACTATCATTAGACTG
ATGACATATTAATGTGACCATAAATATGTGTCATAGATAAACTGTTGCACATATTTTTCTGATTCTTATGAGGC
ATTTCTTAAACTTTGGATACATGATTCAACAATAAATGAACTCCATATTCAATATTCAAGTTTCGAAAGACTG
CAAATAAGGATATTGCAACTTCTTGAAAATGAAATGGAAACACTTAGCAACAAGAGCTATGCCTTATGAGAT

CAATCAATTTGTCATCCTTTTTTCACCCTCTTTTTTGTGACAAGTATTTCCTATAATTCATGGCTATATCTCTGA
TGCGAAAAGAATTTGAGACTTGTTCCTTACATGGGATAAAGTTTATGGTCTGTCACTTCAAGTCTGTTGGGAC
AAATGTTGTAGCAGGTTATTATTTGTAAATCTTTTCTGACTCTTCAAAGTGATTCTGAGTTGTCAATATCCTTCT
AGTGTTTTCTTGGTTATTGAACATAGTGCCGTAAGATAATTTTCTTTCCTCTACCCATGCTTGATTCCTCTTTGTT
TCCAAATGCTTTTGGTCTCAACAAAAAAGGACCTCTTGCCGTGCACACTCCTAAAATCTTCTGCTGATGGCAC
TTCAGTTGATTCTAG
>TCONS_00024978
CCTACATTGGCCTCCAAGGCCATATCACTACTCTTCTAAGGAGCCATTTGAGTATTCATTACCTGCAACACCG
GAAGTTCCATTTGGTAACCGGCTCGTGCTGGTGGAATTTGAATAAGGTGAGTAGGTTGAAACAGAATTGCTTC
GTGTATACCGGTTCTGAGCAGTGCTACTTCTTGTTGCCTTAAGGCGGGTCTTCCTCCCCTTTCCCATGCATTTCT
CCAAGTGAGGAGCAAACCGTCCAGCTACTATAGACCGGCTACAGTTCATACATTCAAGTATCTCATTTGCGA
CAGGAGGATGAGTTTGCCCAAATATATCTACAATGTATTTGCCATTTGTTTCACTGCTATTGCTAGGATCGGCT
ACTGTTGCCCGAGCTTGGGCAGACAACCGCAGTTCTTCCTCTTCTTCTTCTAAATTACAATCAAGGCCTAATTT
TGCTATTCGGTGACACTCAGATGCAAC
>TCONS_00025064
ATATTCCCTAACCTTCTGAATAATTTCATATTGGTCGACTCCAGGCTCAAGTACAACATGACAGGACAAAACA
ATTTTCCCTACAGTGATGGCCCAAACATGCAGGTCATGAACTTCCTGAAGTCCTACTAAAGATTTAAGGCCAT
TCTCGAGTTTAACAATATCAACTTCCTCTGGTGTCTTCTCCATCAATAAGGAGAAGATAATTCTAAGCATGGG
TATGGTAGTACTAAGAGCAAAGATCGAGAAAAAAATAGTACAGAGAAGATCAACCACCAACCATTCTGGTT
TGAACCACATAATAGCTCCAGCAATCATCACCCCAACTGATTGTATCAAATCAGATATAACATGCAGGTAAG
CCCCTTCGATATTTATGTTCGTTGGTTTGCTGCAACTAGAGGCTGCTGATACCAGTTTCAAGCTCTCTTCTTCAT
TTCTTGGATGCAATTCTTGCATTTCGTGATCGTGATCATGATCATGATCCTTGCAAGGATTATATGAATGGCAA
TGGAGAGAATGATCATGGCCAAGCCACAAAACTGAGACGAAGTTAATTATGAGACCAAATGCAGCAATAGC
AAACATAAGCTTCCCGTTCACTTTGGCTTGTGGATGAAACATTCTCTGAATTGCTTCATAAATCAGCAGACCA
GAGACAAGCCATATTAGCTGTACAGATAGAAGGGCTCCTAAAACTTCAAGACGGTGGTACCCGAAAGAGTG
TTCTTTTGTCGCATCCCAGCCGGACACCCAAACAGCAAAAAGAGAAATAGAAAATCCAACAACATCACTGA
GCAAGTGAGCTGCATCAGTTAGAACCGCGAGGCTGTGGGCTTTCACCCCTCCTATGGTCTCCACTGCCATGAC
TATTACATAAAAGATTATGAGCCCACAAAGTTTCATAGTCGACTTTGACCTTTGTCTTGAACCCAACATACTA
TGCTCTTGCTCTGAGAATGAGCAAATTGGATTGCAACAAGACTGAGCTCGCTTACCGTTGCCATTACATTTTG
CTCCCAGCAATTGCTTTATTTCAGACTTGGAATCCTCATGTTGCTCCATCTGGATTCAGGACAAAAGAGAGAG
AG
>TCONS_00025102
CTTGATTAATAATTCTCTCAGATGAAACTACAATATGCAGGTCGTGGAGGTCCTACTTCAGCTCTTCCTGCCTT
TGTGCTGCCAACATCTTTTATTACCTTCCGAAGGTTTCCTTCTGCAAAAGGAACCATTAAGCAATGTTGCTCCA
CAAGCTGGAGAATGGTTGACTGATGGACCTGCAAATACACTTGGTATTTCTGCTCCTTTGTGCTGCCAACATC
TTTTATTACCTTCCGAAGGTTTCCTTCTGCAAAAGGAACCATTAAGCAATGTTGCTCCACAAGCTGGAGAATG
GTTGACTGATGGACCTGCAAATACACTTGGTATTTCTGCTCCTTTGTGCTGCCAACATCTTTTATTACCT
>TCONS_00025125
TCCAATTCTAACAACAGAGAATTTCAAACACTTGCCTTTCCAACAACAAATAAAACCCAAATATCCATAGTTT
CAAGTTTGTGCACTGCCAGCTTGATTCATGATAACTAGCACCAAAAGATTATTTTAAAAAGCACAATGCGAG
GACAAAACTCTACAAAGTGCTAAATGCAAAGACATACAATTCTGAAGATCCAATGTCGTCAAGTTAACGAG
ATGCCCCTACTTTTGCAAGGTCCTCAAGTGCCTTTGAGATCTGCTCCTCCGTCAAGCCCTCCAATCTGATGCCC

CTTTCAACTCCTAGGTCATATCTGGCCCAAAGCTGAGGCTCGATGGAAGTGGCCTCACGAATCAAAATAGGT
AATTTGGGGTTGTGAGTCTTGAGATCTCTGTAGTTCTTCTCAATGAACGCCCTAGTGGCAGCACTTTGAGGGG
AGGATGGAGAAAAGAGGATGCGAAGCTCCTTCAGATTCCGAGACAGATTTGCTCTCCACGCCATTTTCCCAC
AATCTTCACCTTCCAAATATTCCCTTTGCC
>TCONS_00025401
CACAATTGAATCAATTTAAAAATTTTAAAAAGACCTAAAGTTAGGGATTTATCAATAGGTAATGTTGCACCA
ATGCCCAACCAAAGGGCGGCTGCGGTACCAATCAAAAAGACAGTCGTCGCTACTGGACGGCGAAATGGATT
TTGGAATTTATTAACATTCTCTAAAAAAGGTACTGTTAATAATCCCGCAGGTACTGAAACCATTAAAAGAACC
CCCAATAATTTATTGGGCACTGTACGAAGTATTTGAAATACAGGAAAGAAATACCATTCAGGTAATATTTCCA
AAGGGGTTGCAAATGGATCTGCCGGTTCACCAATCATTGATGGTTCTAAAACGGCTAAGCCAATCAAAAAGA
CAGTCGTCGCTACTGGACGGCGAAATGGATTTTGGAATTTATTAACATTCTCTAAAAAAGGTACTGTTAATAA
TCCCACAGGTACTGAAACCATTAAAAGAACCCCCAATAATTTATTGGGCACTGTACGAAGTATTTGAAATAC
AGGAAAGAAATACCATTCAGGTAATATTTCCAAAGGGGTTGCAAATGGATCTGCCGGTTCACCAATCATTGA
TGGTTCTAAAACGGCTAAGCCTACATTACATGCAATAGTACCTAGAATTACTACTGGAAAAATATATAAAAG
ATCATTGGGCCATGCGGGCTCTCCATAATAATTATGACCCATACCTTTAGCCAATTTAGCTCTTAATACAGGA
TCATTCAAGTCAGGTTTTTTTGTTATTGGGATAGGTGAATTCTTATAGATCCATCCCCCGAGGGAACTGGACAT
GATAATTTTTAATCATCCAG
>TCONS_00025402
ATTTCACAATTGAATCAATTTAAAAATTTTAAAAAGACCTAAAGTTAGGGATTTATCAATAGGTAATGTTGCA
CCAATGCCCAACCAAAGGGCGGCTGCGGTACCAATCAAAAAGACAGTCGTCGCTACTGGACGGCGAAATGG
ATTTTGGAATTTATTAACATTCTCTAAAAAAGGTACTGTTAATAATCCCGCAGGTACTGAAACCATTAAAAGA
ACCCCCAATAATTTATTGGGCACTGTACGAAGTATTTGAAATACAGGAAAGAAATACCATTCAGGTAATATTT
CCAAAGGGGTTGCAAATGGATCTGCCGGTTCACCAATCATTGATGGTTCTAAAACGGCTAAGCCTACATTAC
ATGCAATAGTACCTAGAATTACTACTGGAAAAATATATAAAAGATCATTGGGCCATGCGGGCTCTCCATAAT
AATTATGACCCATACCTTTAGCCAATTTAGCTCTTAATACAGGATCATTCAAGTCAGGTTTTTTTGTTATTGGG
ATAGGTGAATTCTTATAGATCCATCCCCCGAGGGAACTGGACATGATAATTTTTAATCATCCAG
>TCONS_00025403
ATTTCACAATTGAATCAATTTAAAAATTTTAAAAAGACCTAAAGTTAGGGATTTATCAATAGGTAATGTTGCA
CCAATGCCCAACCAAAGGGCGGCTGCGGTACCAATCAAAAAGACAGTCGTCGCTACTGGACGGCGAAATGG
ATTTTGGAATTTATTAACATTCTCTAAAAAAGGTACTGTTAATAATCCCGCAGGTACTGAAACCATTAAAAGA
ACCCCCAATAATTTATTGGGCACTGTACGAAGTATTTGAAATACAGGAAAGAAATACCATTCAGGTAATATTT
CCAAAGGGGTTGCAAATGGATCTGCCGGTTCACCAATCATTGATGGTTCTAAAACGGCTAAGCCTACATTAC
ATGCAATAGTACCTAGAATTACTACTGGAAAAATATATAAAAGATCATTGGGCCATGCGGGCTCTCCATAAT
AATTATGACCCATACCTTTAGCCAATTTAGCTCTTAATACAGGATCATTCAAGTCAGGTTTTTTTGTTATTGGG
ATAGGTGAATTCTTATAGATCCATCCCCCGAGGGAACTGGACATGATAATTTTTAATCATCCAG
>TCONS_00025404
ATTTCACAATTGAATCAATTTAAAAATTTTAAAAAGACCTAAAGTTAGGGATTTATCAATAGGTAATGTTGCA
CCAATGCCCAACCAAAGGGCGGCTGCGGTACCAATCAAAAAGACAGTCGTCGCTACTGGACGGCGAAATGG
ATTTTGGAATTTATTAACATTCTCTAAAAAAGGTACTGTTAATAATCCCGCAGGTACTGAAACCATTAAAAGA
ACCCCCAATAATTTATTGGGCACTGTACGAAGTATTTGAAATACAGGAAAGAAATACCATTCAGGTAATATTT
CCAAAGGGGTTGCAAATGGATCTGCCGGTTCACCAATCATTGATGGTTCTAAAACGGCTAAGCCTACATTAC
ATGCAATAGTACCTAGAATTACTACTGGAAAAATATATAAAAGATCATTGGGCCATGCGGGCTCTCCATAAT

AATTATGACCCATACCTTTAGCCAATTTAGCTCTTAATACAGGATCATTCAAGTCAGGTTTTTTGTTATTGGG
ATAGGTGAATTCTTATAGATCCATCCCCCGAGGGAACTGGACATGATAATTTTTAATCATCCAG
>TCONS_00025584
TTTACTCATTCCCCAAGTTAAGTGAGCCATCCTTCATTAGCTTCACACTCAAGACTATTAAATCTCCCTCTTGA
GTAATGTCGAGAAGCCTTTCTCCAATCTGTCCCAGTTCTCATCATGGCTACAAATTCTTCATAGCTAATGCTGC
CATCCCTGTCCGTATAAACCTCCTGTAAAATGTCATTCACAATGTTGGTGCAGTCATCTGCTCCATCCTCCATC
AGTGCATCTTGAAGCTCATGTGGTTCAATATAGCCATTTCCATCCTTATCAAAGTAGGAGAAAGCCTTGTGCA
AATGTTCATCATTTGCCATCCTTTGAAGATGGAGTGAAACAGCAATAAATTCCCCATAATCCAGGGTCCCTTT
GCCGTTGGTAT
>TCONS_00025586
TGAAAGGCTCCTGTGGTTAGTTCCAATACGTATTGCACGTCCGTACTTTTTACATTTTTCCACCAATGGTGTAA
AAACCTCCTCAATGTGCTCAAGTTCTTTCTGGTAGTCTTCTTCTGTGTACTCTAATTGCTCAAACTGGGCTCGCC
TGTCAGCAAAGTTTTCAGGATTGACACGTATTTTTTCAAAGCACTCCGCAACTCGAAGTGCAATAGGAGGAGT
AAAATGAATGTCAGCCACCAGAGGGATATTGTAATTCTTTTGCACAAGAGAATTTTTAATTTCAAAACATGCA
TTAGCTTCTTTCTTCCCTTGGACTGTAATTCGAACTATGTCAGCTCCTGCGTCAGCTATTTTCATCACCTGTATA
AACAGGAGTTAGCATCAGCGCTAAACTTACTTGGCTTAAAATTTTCATGCCCATTGAGGAAGTAATGAGGTTC
TATATATA
>TCONS_00025587
TGAGAACAGAACCATCTCGATGCAACACGCCTCTATAGTCTACTTCATCACCCTGTTACAAAAATCAAAACA
TTTGTTTGCTTCACAAAATGAAGCCTCATATAGCAGATCTGCTCAGGGTTTTTACCTGACCTCCTTTTGAGCTG
GCAATTCCCCTGACCTACGTTGAAAGTCAAAATAACGTCTGTTCTTTTCTTCAAAAGGGGCCACTCCTTGTTGA
AGCTCAGCTGCTCTCTTTCCAAGGTTTGCCAACCGTCGACAGGGATCTATCTCCTCCTCTGGAGGTTCAGTTAG
GGAAACCCTTATTGTATCACCCAGACCATCCTGCA
>TCONS_00025664
ATGGAACAACTCATGACTTTGAATCAGGTTATGTATTCAACCTATTTAATATTCTGAGGCAAAAGGCTGAAAT
GCATTACCATCATGTTTGGCCAGAACTGCAAGTTGGGTGGAGAGTAATTGTTGGTTCGATAATTGGATTCTTC
GCTGCTGCTTTAGGAGTGCAGGAGGAGTAGGGGGAGGTGGTATCTTTGTTCCCAT
>TCONS_00025671
CTTTCATCCTAACATGTCCTCATCCAGTGTTAGCTTGAGTTCATAACACTTTGCTAAAGTAGATGTTCATGAAG
TGTCTTGATGGATTGAAGCCAATTAAAATAAAGATGGGTGAAAGGACGATGACAATGCCAATTGGATATAGG
AAGAATGTTGTCCTCTCCAAAAAGGGTAATACGTCATAACCTAAAAAGTTGAGGTAATGATAGTAGGAAGCA
GCAACCATGAAGAGCAAATTTGACAGCAAGACAGGAATGAAACCATGAGCTACCAAAACAGGGGAGAGAA
AATAATGTATAACGTATAGCAAAACAAACATAGGGAAGAAAGAGTTGCAGTGGACATCAAACGCGTACAAC
CATTCTACCCGTTGTTCCACGGCATGACTGCTTGGAGCTTCCTCTCGAAGGTAATTGTTGGTTAGGAACCAGC
AACATGTTGCCAAAGTAGCTCCAGTGAGTACAAAATGAAAAAGCAATACCGAGATAACTACAAAAACAGCA
TGTCCTGCACTATGATCATACGCAACACAGTATGCCAAAGTAGCAACCACCAACAGAAGACTGCATATTACG
ATAAATGCAGGATCATCACGTGCCCACTGGTTCTTTGTTTGCTTGTGGTACTTTGTGTGCTGATATACAACTTT
AGGAGAGGTGCATAGGTGAAGCATTTGCCAAAAGGTATATTCAATGTCCATTTGTTGCCACTTGATTATTCGG
CGAAGGTACTGAGGCAATACTGAATTGGGTCGGACATGAGAGCTGGTTCGACCTTTCGAAGTTGTGGGCAGC
ATCTTTATCTTTTTC
>TCONS_00025891

CTTCTACATGATATCACTACGTAATGTTCTCCAGGCTATAATACAACAGAAAACCAGCAATTAACCAAGCAA
GTCTCTAGATCCCACAAAATTAAGATATCTAGCGACCAATCATATGTAAACAATTTCCAAATCAATAATTTGT
CAGAGTCAGAATAACAAGAGGGAAAGCTAGAAAATTTACCAATTAAGAAGCACCAACGATGATGTTATTGA
TAGGAATGAAAATCAACTTCACGAAAAATCCAACAAATCCCATCACAACAAAACCGATCGCTGTACGAGTA
GCAACCTTGGTAAATTCTTTACGATCAGGCTTGTGACATCTTTTGACGAGCCTAACGCTGTCTTTGGCGAAATC
TCTAAGAGGATCGAACACAGAATCGAGGGCGTCCATTTGAATTGGATCTGATCTGAAAGAAGAAGCAGATTA
GGGTTTCTCTCTGAATTGGGTGAAATGGAGATGTATGAAATGAGCTTCGGATCGATGCAAAT
>TCONS_00025986
GGGGGATGCTGAATCGAAAAAGGTATTCCACTTACCGCTACAACATGTGGAGCTAAACTACTAAATGAGTTC
TCGAAGCCATAATAAACTGCAATAACAGAGAAATTCAACTTTCATTTTGAATATAACATGCAATTGAGCACC
ATTAAAATTTAAAGGAGTAGTCTATGTACCAGTAAGAATATCTATAACCCGTAGTTCTTTCAGTCGGCCAGCT
AGCACTAGTCCAATAGCAACTGTCCCATATAAACAGAGAAGAAAATGCCAGAGAAGAAAAGCCATAAAGT
ATCTGGTGTTTCTCTCGCCTATGCAATTGTTCATCCATCCACAATGATGATCAAATCGAGCGACACAACGATC
ACAAATGCTGCAGTGCTTTGACCTAGCAGGTCTGTATTTGGAATCTTGCAGGTTGGACATTCCTTTTCTGAGAA
TATAATATTGTCATAGGGATAAGCAGATAGATACTGCGAAACATTCTCAATATTTACAGTTCCTGGGTCAGAA
AAGCTAGTCAATAGAAAGAGTAGAATACCCCCTCCAACTGCCAACAAGCTCGTGTACCTGGAGGATGGGATT
AGGACGATCACAACAATAGTACTCGACGCAGAAGAGTGCATCGGAGCCTCTTTGACCAAAAACAGCACCCA
CAAA
>TCONS_00026045
CTTGGAGAAGAGCTTTCCTCCTTTGCCCAAAACCAGGTGCTCTGATGGCAGCAACGTTCAGTACACCTCGCAG
CTTGTTCACAACAAGTGTAGCCAAAGCTTCTCCAGTTACGTCTCCAGCAATAATGAGCAGAGGGGCTCGTAA
TTCAGTTGACTTCTCCAGGAGGGCCATAATATCCTTGATTGCTGAAATCTCCTGATCTGTAACCAGCACTCTAG
CATTCTCAAATTCAACAATTAATTTCTCCTGGTTGGTCATGAATTGTGGGGAGATATATCCCTTATCAATCTCT
ATTCCCTCTTCAACATGAACAGTTGTTTCAAAGGAAGAGGATGACTCGATTGTTAGAACACCATCATGACCA
ACTTTGTCAATTGCATCAGCAATCATGGTTCCAATTTCATCATCATTTCCAGCAGATATAGAAGCAATAGCTTT
GATGTCATCACGACCTTTAACAGGCCTAGCCCTCCTTTCTAGCACTTCAATCAAAACGTTTACAGTTTTGTCGA
TGCCCTTCTTCAGAGACACTGGATTTGCACCAGATGTAACACTTAATAAACCACGTTTAATGATTTCCCTGGC
AAGAACTGATGCAGTTGTTG
>TCONS_00026059
CTGCTTTCAATCCCCTTACTGCCATTCTTATTCAAAACAAACTTGAGGGTCCGAATTATGTTGATTGGAAACAA
AATTTGGATATTGTTTTTGTGCTCGATGAGGTGTGTCCAGAAAAACCTGGATATGATGCCACAGATGATGAAC
AAAAGGCTTACCAGAAATGGATTAAGGCTGATGAGATGGCGCGGTGTTACATTTTGGCATCCATGTCAAATG
TTCTGCAACATCAGCATCAGTCGATGGAGTCTGCTTATGACA
>TCONS_00026067
TTTTCATTCATCAAAACAAAATAATTACACTATTAACTGAAGAATAAGAATTTATCATCACAGATTCAAAACA
GAATTGACAAATCTGCAGATTATAATGACTATGTCCGGTACATGATAATGAAACAGCGGAACCATTAATACA
AGAAGCAAGTAAACAGCCGGTGGCGCGTGACGGACCTACGGTGGATCAGCCGCCGAGATTAAGAAGAAGG
AACAATAACAGTGCGCATCATATCCTTGAATTCAACGAAATCAACTTGGCCATCGTGATCTTGGTCAACTGAT
GAAATCATCATCTCCACTCTATCAATTTCATTTCCTTCAGGCAATCCCAGTTTCTCTAGTACAGCCTGTAATTC
CTTCGCCGATATAAAACCGTCGCCGTTCTCATCAAACACGTTAAACGCATCCTGCAGATCCACTTCATCCTGA
TCCGGATCCTGATCCAACCCGATCTTATCCTCATATTTTGACCCGAAGAAAACATCGTCGAGTGAGCGATGTA
GAGCCTCAAAATCTTCGAATCTAAGGCCGTTGTTTCCTGATTTGATATATGATCTCACCATTGATTCAATTTCC

GATTGATCGGCATCTAACCCTAGCAAATTAAACGCTTGACTAAGTTCGTCAACGCTGATCAAACTGTCATGGT
TTCTGTCAAACACGTCGAAGATCCGACGAAGACGAATTGAATTGAGGCTCGGGCTTCGAAGCCGGAACGAC
GATGAGGAAGAAGACTTAAGTTTGCCTCTGGTAAGGCTCTGTTTACACTCGTCCTGTACTCCATTATTGTCACC
TACTGATCCCATCGGTTTCTTCTTGTGAAAGATTTCTATAAACTATCTTGAGAAGATTTGAATAAATATAG
>TCONS_00026101
CTTCTACTACGACTAGCGTAACAGCATCCCAAACAAAGCTACACCTGAAGAAATTAAGCTTAGCTAAATCAT
GAAATTTTCTAGAAATAGGGAAGACATACCTGTGTTGGAGTCTTACTGTAGCTTTCTCCTATCTCCTTTATTCT
ATTAATGAGTGGCTGCAATTTGGTTAGAAATTCAGGAGTATAGATCTGTCCTCGAGGGCCGGTGGGTGGATTT
TCTGGGGTATATTTCCCAGTCAAAGCTCCTTGAGCGATGGGTGAATATGCAATCAACGTGACTCCCAGTTCAT
CACAGGCAGCCTTTACACCATTTTCCTCTGGAAACCTATATATCAGGCTATAATTGACTTGATTTGAAGCTAG
CGGGATGCCTCTCCTCTTCAGCTGCTCGTATGCAGCGCGAAGACGCTTTTCACTATAGTTAGATACTCCCACA
GCTTTTACAAGGCCCTGCTCCACTGCATCTCCTAAACCATCAATATAACCTTCATTTCCCCATATTCCTGGCCA
GTGAAGTTGATAAAGATCAACTGAAGAAAGTTCGAGACGTGCAAGAGAATCCTTGAGGGCGGCTAGGACAC
TTTGACGGCCTAGCCTCCACGGCAATGCAGCAAATTTAGTCGCAACAGCAACCTCGACTTCTGGATTACTTTC
TTTTCTTTCCTTGATGAATCTTCCTAGCAGTGTTTCAGAATTTATCGCACCAAATGAGAACCTTGAACCATAAA
CCTCAGCAGTATCGATGAATGTTATTCCACAATCAATGCTGGCATCAAAAGCAGTCTTTGCAGCCTTCAGCTT
CCTATCATCCCACTCAAAGTTATTCCAATAGCTAGTATCACCCCAAGACCAAGCTCCAATACCAAGCTTTGTA
ACCTTCAAATCAGACCCACCAAGTTTCACCTTATCTTCTTCTGCTTTCACTGTAACACTACTCTCAGAAGCTAC
AGCTCTAATTGTTTTCACCCTTCTCTGGCTCAAAGAA
>TCONS_00026107
AATATTGGTAATGTTACAAAATTTCCTGACATGGAAATTTTTATAAATGTCTGTGGAATTGATATCAGATGTGC
TATAAAACCCCAGCTGCAGTTTTTATAGCCGGCATGTTTTGGCTAAATAATCACATCCTTCGTAGACCTCCAA
ACCTGGCTGGCCAAGATCATCCTCTTTAAAGCAAGCACATCGTTTGCTCATTTTTCCGGTCAATGCAATCTGCT
CAGGTCGTTGAACTAATCTTGGATATCCATCGTCGCACCAAACCTCTGAACCCTCTTCTTGACTCCATCTTGAA
TTACAGCTAGGTAGTTTGGCCTCTTCATTCTTCTGTTTTTCCATCAGCTGAGCACCTCTAGCGGC
>TCONS_00026133
CCAAGTTCAGAAGGTGCACCTTATTATGAGATCTGTCAACAGCAACAACCTTCCCTTTGTCCTTCATAAGGAT
TGCTATTGCTGTGGTTTTGCCCCCTGGAGCTGCACACATGTCCAATACTCTCTCTCCTGGTTGAGGATCTAAGG
CGTGTGCAGTTATTATGCTTGGCAGGTTCTGAAGAAAAATCTCACCCTCAAGCAAATCATAAAAGGAGGGCA
GTCTAAATACTCTTTCCTTCATATCCACCGCAACTCCTTCAGATACACGAAATAACCCAGCTCTTGACATCATT
GCTGTTCCTTGGCCAATGTAGAAGCCATCTCTTTCAAAATAATAAGGATCTGCTTGTGATCCTTGTAGAACAG
TGCCACGTGTCATTGCAACACCCCATCCACCATCTGGAGCAGACTGTTCCACAGCAACTGAAACTGCAACTG
CATCACCTCTTTCGACATGAGCACTGCATGCCAAAACACCAGGCACATAAACTTGAGCACCACGAAGAACTG
CCTCCGCACACTTTCGGCTCACAATTACCTCCTTAAGAGGTTTGCCTTCATTGTACCCATAATGTATAGCATGT
GGTCCTGAACCTTTAACAAAAACTACATAGTCCATACCAGGAAATTGACACTTTGAAATACTTTCGATGGCCT
TAAGAGATTCTGCACTTCCAGTGCATTCTAAGGTTGCCTTAC
>TCONS_00026134
GTTGGGAAAGATTAATTAACAAGAACTTGAGGCGTTAACCCTCATTTTATAGATTCGACCTAGGGATAGGATT
GAACTACTTGTAGCCTTTCGGGTGTGCTTAATCTTCTAATTGTGATTAGGGATAATTCAATTAGGAAGTCTTGT
TAGTCTTCGAGAGAAGCTAATTAAGAATTATTATCCGTGGCTAATTAACATAAACTCGCTCATATTTGTAAAA
TCGT
>TCONS_00026142

TGCACATTCTCTCCCTGGCGTAAGTATTCACTCAACGCCAGTAGTACTTCCTTCAAAACACTGAAGAAAGCAC
TCACTGTCACCACTTCTCTTGTTTCAGATCAGAGACCGATTATAATGGGTCGATACACTGAAGATTTCGAGGA
GAAAGAGCTGTTGGGTAAAATATTTCTTTTCAAGTCATTTTTTAATAATTTCTTTCCTAATTTGACACTTTTTCA
AATCAAAATTTTCTATGCGTTTATAATGACTGAACCTTGAATTAGATTGGAGAGCCTGATAGGAAGTATGGTT
TGAATAATCCTTTAGTAAGGATATCAGCCACTCGCTGTGAAGTCGGAATAGATGGCATTTAAATAACTCCAGC
TCCAACCTTCTCCTTTGTGAAGTGTTTGTCAATTTCAATATATTTTGTTCTACCCTGTTGAACAAGATTGTGAGC
AATGCTGATGGCTGCGTAGTTGTCACCGTACAGTTTCATAGGCATAGTCTCTGTTCTTCTTAGTTCCTCCAAAA
TTCATTTAAGCCACACCAGTTTACATATTCCATGTGCTGTAGATCTATATTCTGCTTTAGTACTGATGCGGG
>TCONS_00026153
CAACAGACTCACCATATAAATATGCATGAGGTTCATTTGAGCCTAAATATAAAGCTTCACCAGGGTTGAGTTT
CACATAATTTAACAAGTATGCAGCTAAGACGCCAACATCAGCTGGATACTGCTTCTCAAGTCTTAAAATCAGT
TGTTCTTTGTCCGTTAGGTGCCTCGCCTCAAGACTGACGAACCCACATAGAGCCTCAAAT
>TCONS_00026174
GCTAGAAATTCGGGTGGAGCTGTTCTTCCGATAAGTGACCACACATTTTCCATCACTGTCTTTGCCCATGTGCT
CATTGTGTAAACTGCTATAATTATAGCTCCAGTAGGATCAATCCACAGTAGAAATGGATGGCTAAGACTGCT
GCCGCTAACCCGATTGAGTTGGTAATGACATCAAAGAAATGGTCTTGAGCATAGGCCCTTACAATTTCATTTT
TGAATCTTCGGCAGTAGATCAGAAGCATAAACTTGACCACAGTGACAGAAACCATAATT
>TCONS_00026344
GCAAGAAAATAATTATTGAAAGATAATTGAAATGTTTGTCTTCGACTCAAATGAGCATTGAATACAGCAAAT
TTATTCAGACGGCCGCTGTCCCAATACTGAAATATCCTCGTAACGCTTCCCAACATTGTTGTGCTCCCAGAGCT
TATGAACCCCATAATCCACTGCCCGTTCACCAAGGAAAGCGCCAGCGATGACGAAGGTGACATAAACAGAG
TTGCGGCGCATAAGCACTCTGTAAAGCCCTTCAAATAGGCCGCCTCTGCTTCTTCTGGCTGCTGTCTCCATTGC
TTTCTCAGGAGATTTCTAGCTCTGCTCCTTCGGAAGAGAAATCGGAACG
>TCONS_00026359
TTGTCCTTGTAGCCCTCGCCACTTGCTTCAGCCTCTGAAACGTCAAAATACTCACTTCCAAATCCTTCTAAGAA
GCTCGCTAGCTTTTCAAGGTTGGTAATAGCAAGGTTATGAAGATCTTCCCCAGTCCAGACTGGACAAATAAGC
AAGGAGATGATCATGACGGTTGCAACACTGACCATAATGGTGGATATCCGCTGCTTATCCAAATCCAAGTAC
TTGTCACCGGAGACTGACACCAAACTGAAGGTTGCGACAAAGAGCATGCATCCATAGTCATACCTCATTTGC
ATATGTGGGTAAAACCTTGTAAATGTACCTA
>TCONS_00026402
TCCTTTGGCACCACCCATTTCCATTTTTAATCAACCAGGTAAAGGAGGTCCAAAAAATAGTCCAAAGCGAAG
GTTGTCTGAGATGGAGCTAAAGTCAGCTACAACACATGTATTGTTAAATTGCCCTGAGGTCCAACCTTATTAT
AGGTAAGTATATATTCGTATATATTACAGACTTTTATATTAAATATACCACTGATTTAACTAATAAATATTATA
ATGGCAATTACTTTGTGGGAACATATGGGCAAGATGTTGTTTATCCC
>TCONS_00026403
TCCTTTGGCACCACCCATTTCCATTTTTAATCAACCAGGTAAAGGAGGTCCAAAAAATAGTCCAAAGCGAAG
GTTGTCTGAGATGGAGCTAAAGTCAGCTACAACACATGTATTGTTAAATTGCCCTGAGGTCCAACCTTATTAT
AGGTAAGTATATATTCGTATATATTACAGACTTTTATATTAAATATACCACTGATTTAACTAATAAATATTATA
ATGGCAATTACTTTGTGGGAACATATGGGCAAGATGTTGTTTATCCC
>TCONS_00026409
ATATATCACTGAGTCTGGTTCTTGTTTGTCAAGCCAATCCAAGGTTTGGTGGCGTTTATCTGAGTCTTTCTTCTG
CTCAGTTAGTAACAATGGATTGAATGGACCAATAGCCCATAGGTTTAAGCCATCCTTTTCTTTGGCTACTAAA

TCAAGGTATAAACCTTCTATTACTCTGGATGAATTGTAAAGTTCTCCAGAGCTGATTTTCCCCATAAGGGATTC
TTGTTTCCTCCACAATTCCCATAACTCTGGAGTAGCGCCGCCGTTTTCGACAGATGGAATGTCTTCGTATACTT
CAGTTCCAGCTAGCAAAGGCTTTCCTTTGATTTCCCAGAACAATGAAAACGTATGAAAAGCTGAGATACTATT
AAACTAGTAGCACTCAGCATTTGGAATTGCTGGCACATCCTCTACTACCCAAGTCATCATAGAATCATAAATA
AAAATCACTCTCCGATGATTTGCACCTAAGAGTTGGCGTGCAAGAGAGCACACTGGCTCCCGGAGATGTATT
GCTGCATAAAATGAAGGGATTAATTGATTAGGAAACTTATGAGAAGCATTAGGATTTGGTGGGGGATTTTCA
AAAGGTGGTGTAGGGAATTCATGAAAATATAGGTTTTTAGCTGTGAGAGGGTCAAAACCATGGGGACACGA
AACTTAGCCTGGCGAATATGAGTGGTTGCTCCAACATAGTGTACTGGGATATTATACGAGGAGATAAGTCTA
GAGAGGTGGAGAAGCTGGTTGAGATGACCTTGTGCTGAAAGTGGCACCATAATCACAGCCACTTGTGCATCT
TGAACACCATTTTGGTCATGGCTTTTTGTGTCGTAGCTCTCAG
>TCONS_00026532
CAAAAGCTTATAACACCAAATCTAGCCATGGGCGAGAAAAGAAGATCCCAATACTAGGAGGAAGAATATAA
CATTCTCATTTCTTTCCGCTCTTTTTCAGGCCAGCACCACCAATGGAGCCTTTCGCAGCCTTTGCCTTGAGCTCC
TTTAGTGCCTTTTCCTCTTCCTTCTTCTTTGCAAGGAAAGCCTTGTCATTCTCGTCGTATTCCTTCTTTTCACTCTT
TGGGGCCTTCAAAGGCTTTGCCTTTCCACCTTGCTTAGAAGACATGGTTGATTTTCTAGGGTTAAGAGTTTGGA
TCTGATTATCGCTTAATTAGAGAATGGAAATACCTTCTCCTAACCCACACAGCTCTTTTGT
>TCONS_00026574
TATGCTCTTAGAGATCGTGCTCTTCTATTAGTTATGCTCTTAGAGCTCTTCTATTAGTTCTTTTGCAGAAAATGT
AACATGTCCGTGGTGATTCCATTGCATGGTGGTGTAATCAAGCTCTGCCAGCAGTGTCTAGATTCAACTTGCA
GTATTTGTAGGGCTACCGTGCTGTCATCCTTATCTGACGTTAATGATCAGCAGAAGAGAGAGATTTTGGATAT
CATAGGTTTTGACCAGTCATTATCTTTACTAGTATTAACAAGTCCGTCATCAATCCTACCCATATATATCACAA
GTAGGCCATGACATCAGTGTGGATGGAGCTATCAGTTTGGTTTGAGATCTTGACTTTCATCGTAATGTTAA
>TCONS_00026575
ATGGAGTGCTCTTGTTTGTTGATTTGCTTTAAGGTTTCTTTAGTTCAGGCAGATCTAGCTTTGGCTCTTGATAGT
GGTGCATTCTAGTAGGTCCTTTTGCAGGAACTTTAACATGTCCGTGGTGGTTTCCATGCATGGCAGCGGGTATA
TTGTTTTTTTGGTTCTTATATTGAGTGATATATCTGCAGCCATAATCAATTACTTATATTCTATGGTGTGTACTTA
TGTCTGATCAAGCTCGTGCCAGCAATGTCTAGGCGTTCGTG
>TCONS_00026666
GATGCTTTATCAGATTTTGAGCCTGTTCTCAAGTTGGTTCCTAGTAATTCTGTGGAAGCTGTGAAAACAGAAA
CAGATGAATCTTGTCCTACAAAACCACCACCATCTTCTGCCTCTGCTAATCAGGTGGTTGTTCTTAGAGTTTCA
CTGCACTGTAAAGGATGTGAAAGGAAGATGAGGAAACATATATCCAGAATGCAAGGTGTGT
>TCONS_00026811
ATATAACCGATGAAGTCTTTCAACTTCTGCTGGAGTGAAATTGGGGAAAGTTTCACCAAATTTCTTTCTAGAG
CAAGAATTTAACAAGCTGCGTACTCCAACCACAAAAGATTTTTTCAACTTTGAGTAACTTGTGTTCTCCATTTT
CAGCAATGACGGGAGACGGGAGACTGAAAATGAGGTGAGGTCAGAATTTTGATTTTTATAAAGTTTCGACTT
TATATCCGTTTGGTATATTTGGTCAGAATTACTGGATTTGCAGGTTTGGACCAGTTTTTGACGCCCTTTCAAAC
GTAACTAATTAAGGAGATATTTCAAAGAAGTAGGAGGAGAGAGGACGGCCGGACAGAGTACGGGGGAGAA
AGGCGTCGCCGGCGTCGTCCCGGCAGTG
>TCONS_00026875
GATGCTTTAACAAGAGCAATAGGAAAGTAGAAAGATAAAAGGAAGATTGCGAGATATTAGATCTGACGGAT
GTAAATGAGTATAGATCTCAGACGGATAAAAAATAGTGGCAACTTTAATACAACGTGGCCAATGAAGGTGG
CCGAAGTGATGGCGCAGAGACGTTGGAGTCTACTGCACATAAAGTGTGGGTATCTTCATGTGTTTTTACATGT

AAGTCATAGTATGAGATTTTGTACTTCATGAATATATAAGCGTTTTATGTTTTTCTCTTCATACTTTAATATTTTA
T
>TCONS_00026877
TTAGAAATTTGAGAATTTGATATGCTCATATCATGAAATGTTTAAAATGTCATTCGTATTGTGGGACTGTTGTT
TTTATGCTGAAGTATATTCTTTTCACAAAAATAAACTTTAAGGTTTTGCAAATTGGTTAACACGTTTAAAGTGT
CCAAACAAAGATTTCAATATTTGAATTCATATTGAGATCCTAATCCTCTCATCCAAACAGGTTCTTTTGTCTCT
GCTTTTCCGTTGCTACAGTTTGAAGCGAGTTGCACAATGCCGTCTCTCCAAACAGCATTGCCTCCTGAACTAG
CCAACAATGCTATCCGACTTTATCGTGAGTGTCTCCGACGAGCAAAATATATTGGTAGCAAGTGCTGCGAGG
GGGCTCATAAACCACATGTTGCATGAATCTGAGAAGATGACTGGCCGGAAATTCAGCCAGAGTTCTTGAAAT
TGCCTGTTCCAAGCAAATTGGCTTTTAGGACCAGACTGAAAGTTTGTTATTAGCCTGATGTTTTATTAGCATAA
TTAGTGCAGACCATACAGCCGTGCTAAAAGTTTTACGAGAAGTTGAGCTTGTGATGAGGCTCATTGTAATCTG
AAGAACGTGGCGTCATTTCTGATTATCCCGTGAATACATTTTTCCA
>TCONS_00026912
AAAAGAGAAACATTTCCCTTCTGTCACTAAATTCCTCTCTAACTGGCAGCTCCACCATCCCACTCATCCAACA
CAAGTAAATTTACTTGAGAAAAGGGCAATTTTATTTCAAATCTAGCACATGGGCTGAAAACTAAATTTGGAA
AAGAAGATATGAAACAAAATTTGATTTATATTTCTACAAATATTAGGTCAAATCAAATAAATCTTCGGTATG
TATGAGGGTCCATGAAAACCTTATCTTGCTTTAGGCCCCTCATGACCTGCATGTTAAAAACAGCATTCTGTAA
AGATGCATCTGATCATGAACCACCAGTTCCAGAAGCACCCTCAGCTTGTTTTTCTCCCGTTGTTTCTCCCGCT
TTTTCTTGTTTTTCAATGCATTCTTGCTCAGCTCCCCAGAAGAACTTCCTCCAAATAACTCTGCTTGAACCTCAG
CAGCAACCTTCGCATGAGGTGGACGATAAGCAGCAGGTTTTGCTGCAGGAGGATTGGCAGGTGTAGCCTTTG
GAACGGCCTGGGTAGCTTTTGGAACGGCCTGGGTAGCTTTTGATCCTTTCCCTGGCGCTTTAGCTTCATCTATT
TTCACCGAGCCAACTGCCTTCACAAGGTCTTCAATGTCACCAAATTTTTCTACTGACTCTGGCTTCCAATCAAC
CTGATACAATCTGTCAAACATCTTCTTGAAGTACAATGATCCATTGTGGTGAAAAATTTTAACCCCATTATCA
ACTTGAAGGCGTGGAGCTGTTGTGGCAGTCATAAAGTACCGACCATCAGGAGACCATTCACTTGTCACTGAC
AATTCAGCCCTTGTAGTTCCAAGCTGCTGCTTTTCCTTGTAGTCCCAGAATGCCATGTCTCCTGGTAAGTTACC
AAAGCCTGCCAAGCATATAAAGTTCCCCAGCGGATTCCATCTGATAGTGTTGTATGGGCCTGTTCCAAGCTCA
AGCAGAGGATTGCACTTCTTGTCGAACACCGTGGCCATAGCAGGCATAAAACCATAAACAGCTGCAAATTCT
TTACCCGAATAGGACCATTGAACATCATGAATAGGGCCGTCTTTACGGAGAGGAACAAGCCCTTCATGGGTC
CCATCAGTAGTCAAGTAGTTCAACTTTGATTCTCCGTAGTAGCTTTGGTTTGTTTTATCAACATCTGATTGGACC
ACCACTAGAAGCCCTGTAGAACCAGAATTCCAGCTCAGTTGCACAGTTGAGCAGCGGAAGAAGCTGCGTCG
AGCAACAGCTTGACTTTGTAAGTCCTTTCCACATGCATATATCTGAACACTGGCTGGCATTCCCTTGGATTCTG
GAACAAATGCAGCCACGTAGGATCCAGGGGCTTTAGAAAGCTCCAATGCAGCAATCGCAGGAACTCGCAGC
CGATTAACAAATCCCTTAGAGAAATCCCTAGGATCAAAAAATTGGATCTCATTTGTTGCCAATCGACATGCA
ACAGTTTCGTCAGAACTGAAACGTACAGAAGGCCATGTAACTTTTGTCATATTTTTCTGGAAGAGTTGGTAAA
CATATTCACCATTTTCTATCTTCCACAAGACCACGTTCTTATCCTGAGGAGATGAGCATTTCTGGAAGGTCTGA
AGATAAGTTCCACAAGGAGAGATGACAGCTGCAAGAACGTTTGGGACATTAAAAGACCTAATCTCTTTCAAT
GTTTTGCAATCAAAGATGCTTATTATGGAGTCTGATTTTGTAACCATGAGTTTGGACCCATCTTCACTGAACTT
TGCACTGGTACAAGGAACCCTATCAAGCTTGATACCAGGTTGACCGCTTGGGAAAGGTGGTCCATTCCATAT
AGCAAAACCCTCTGGTTCTCTTACTAAAATATCCAACGAAGGGGAAGGCTCTACAACCGCCATCCT
>TCONS_00026949
AGAAATTCACTCTTTATGCTTTTAATATTCTTCCGCAGAGGACCGTAATGGAAGCAGTGACTGCGATACTTGG
CGGTGGGCTGCACATTGGTGTAGTTTTTTAGGGTAAGAAGGTTAGAGATGACAACAAGACCCTATTGCAGGC

TGGAATTTCTCATGATGACAAACTTGATGCTTTCGGTTTTACCTGGAGCCTAAGCCCTCTCGAGCTTCCTCAAC
TCTAGGTCCAGAAAGCCATTCTCGTGTACTTCCTTGTGACACACCTCAACCACTAACAAGGGGCCCGCCTACG
CCTACTGTAATTTGTAATGGCATACGACAGGGGACATATAATGCCTTCTCAGATCATCCAGAACCAAGTCTGA
ATTATAACTTCATTGAAAGCGATCATGATTCAGCTCCATCCCCTCCTGATGCC
>TCONS_00027060
TGAAAAGGGTGGGAGAAGAGGAAAAGGTATAGAAAATTCAGCTAGTAGCTGCTGAAAATTAGAGCATAACC
TATCCAGGATATGCAGCTTTGTCTGTTTAAAATTCAAAAATGTGATTCTTTTGAGCTCGCACAAAAATTGCTAT
TGTGCTAAATATGATTGATATGAGCTGTGAGCTTTAACAAACTCTACAGTGAAAAGCTTTAACAAACTCTGCT
TTTCCAAAACAAATTTCCATTGAAATCTGTTCTTTAATTTTATTGTTTGAATTATGTTTTGTTCATTTTCTTTGTTT
TTTCTTCTGTTCACAGGCTTATTTCTTCTCTCTCTGTGGCACTGTTTTTGCCGAGGGTCTGCCTTGAACCGTTGAA
AAGGAGGTTCGGAGGTTCGTATCACATTGAATCTGCTCAGCTGAATGGTCTGCTGCAAAGCCTCAAGGAAAG
AAGAGAGCAAAAAGAGAGGCTAAAGAACCTCGCAACTCTGTGCTTGATAGGCAGAGAATAGGAAAGAGGG
AAGAAATGTCACCTCAATTTTAACTCTGAAGGGACCTGATCTACTGAATAGCTTTTAACAAGGGATAAAATTT
GGCAATTGGTTTACTCGCCTCAGAGACTGCATTGAAGCTTCCAAGATGGTTGAACGCCTTCTGTTATTTGGTGC
CAAAATCCATTAGCTATGCTGTTGTACCTCACAAGAAGGCCAAGAGGGAGTCCCTCTTGCTTATCCCCATCTA
TCTCATATGAATCCTATTATATCTTCAGAAAGCATGAACTGATGATTAACCATGGTTTAGATACTATTCTTGGT
TAGTTATCATGTTCTCATATATGTGTAGATTACCAACT
>TCONS_00027074
AATCTTGATTTTTGTCACTACTCGAGGTTTTATCTACAAATATAATAGACATAGTTGCAACAATAAAGCTATTC
TGTTTAACCAGAAAATAATGAACATTGGGATATACCTCAATTGCTGTAATAACAAAACATTGAACGTAACTT
CCATTTTCTAATATAACTTGACTGAGCTTTGGAGACTGAACTCTATCATACATGGACTGTTTCATTTGGGAAAC
AAGTCAATGAGGTCCAAGCAGGAGAACGAAATGATCTTTGCCTTACCTCCCAGCATATTGGTAATCTCCCAA
GCATTTTGTGACAGCCTCATTGGCCTTCTGAGATTCCCTTTCTCCTCTTTTTTTCTTCTCCCTTTCCACATATTCCT
TCAACAGCTCTTTAGCCTGAACCAGCTTGGAGAGCAAATTGCGATAAACATCAAGGTCCCTATCCACACTTTT
CTTGTTTATATTCAGTGAAGCACAAAGCTTTTCTAAGATGGTTGGATCAGTTACATTTGCCAACTCAAGTAGGC
GAAAGAGACCAACCGCAAATAGCCGACTGTAGCAGAAACTATCCTTGGTTCCAGCTCGCTGTGCAATATCTT
TCAGAATGTTTTCAACTTCTCCATCTCTGGATGAGAAGTCAACTAGCGTATTGGCATTTTGAGTACGAGCCCAT
TCTTCAAATTTTTGTGCATCAGCTCTGTATTGCACAGGATCCTCATTGAGCGCCTCTATATATGCTTTGAAGAT
AGCATCACGATCCTCTTCACTTGGGTAGCCTTCCATAAGTTGATCATATACAGTGACGAAACCAAGCGCAAA
CACGGGATCATATCGGTAGGATTTCTTGTACTTTATCAAATGTTGTTGCACAATTAGCTCTTGTAACACTGTGT
TGTAGACAGTTGGAATTGGACGCTTATAAGCCTTCAAGAAATTCATTTTAGTCTCGGCCACAGTTGGCAAATC
TGGAAACAATACAATATAATGAGCTGCATTATTGCCTCATAAAATAAACATATGCAC
>TCONS_00027089
AGTTTAATTGCAAAGGCTATATAAGCAATAGGACGAACAACAGGCCTGTTTGCTTGAGATTTGCATACACAA
ATTTAATGATCATTCCAGTAATACTAAATAGAGAAATCCACTTCAAAAACACATCTCAAATTAACTAAAACC
AAAAAGCCTAACAGTAGTTGATCAAAATGTAGGCACTTAAGTACGAGCTGCAGTAGGAGCTCCGGCAGGGC
GTGGAGCTTGACCAGGCTTGGGAGCATCATCCCTTGGGCGTCTGAAGCGCTGTGGAGGCTCACGGACCCTCCT
ATCAGTAACAGAGCGGACCCTAACAACTTTGGAGTGGATGGCACATGAGACACAATATTGCATCTTCAAGTA
CAGCTTAGGAAGAGTGTACAATTCAAAAGCACAAGCTTCCTGAACATCCCTCACTGCTGCTTGCTCAACGAT
GTTCCTCACAAGGAACCTCTTGATTGCTTTGTCCTTAGGGCAGCACTTGCCGCAGTTAGAGCAGCGAATGAAT
TTAGTGTGGCCACGTCCATGCTTGTTACGACCTCCGTTTCTTCTCTTGAATGTCATTTTGGCCGGACGAGTTGTA
GCAGCTGCTGAGCAGTGGATACGATGGAACTCTAATGGCGACCACTCTGCTTCTGAAGCTATCAGCTACT

>TCONS_00027107
CAACTCCTTTCATTCAATCTACAGCCTTTCTCTTTAGTAACAACTATCTCCAACCTGTCCTTTGTTTGTTTCATA
ATCTGTGGTAGCCACCTCTCTTTTCCTTTCCTTCTAAAAGTTAGTCATAAAAAATTATTTTACAGTAAATTCGTT
TACATCTACCTACTAACAACGTTATTAATTTTATTTACAGGTTGCACCTACTGGTCTGCTTCAGATATTGATGA
TAGGTAGATATGTTTATAAGGAACTTGCAACTTTTGTGTGCGAGATAACTAAATAGAAGCAACCAAGAACAA
TATAACACCTTTTATGTGATGCTTTTGGTTGGTCCTATGAATTGGTTTACACGTAACAGGTTCCTTTTGTGAACG
TAAAGGTGATAAATTTTTTCCTCTATTTGAAAGCAAAAGCTGATAACATGTGACTTCTAAAGATGATGCTGTTT
TAGGGTTTTTCCAATGCATAATCTGTAGATCCTTTAATTTTGTGTCATATAGTGAGTGGTTTACAACTTTGAAAT
GTTCAAA
>TCONS_00027108
CTTTCATTCAATCTACAGCCTTTCTCTTTAGTAACAACTATCTCCAACACCTTTTATGTGATGCTTTTGGTTGGT
CCTATGAATTGGTTTACACGTAACAGGTTCCTTTTGTGAACGTAAAGGTGATAAATTTTTTCCTCTATTTGAAA
GCAAAAGCTGATAACATGTGACTTCTAAAGATGATGCTGTTTTAGGGTTTTTCCAATGCATAATCTGTAGATC
CTTTAATTTTGTGTCATATAGTGAGTGGTTTACAACTTTGAAATGTTCAAA
>TCONS_00027150
AAAAGCAAAACAACTGGATGTAGAAAATTTCACCAGTTAAGCTCCAGAGTGGGAGACAGGAGTAGTCACAA
AGTGTCCATCCTTGTATACATGAGCATACTGCTGTGGCAAAGCATGCTCACCCCAATCATCATCAGGCCCTGC
ACCAGGAACCATAACATTGATTTCACTTGACTTAGCTGTGGTAATTGAACCCCCCAACGAATCTTTGCTCAAG
TATAACTGGCACCCAGTTGTATTGTCGACTGATATTGTAGGAGCCGAGCCCTAGCAGCCCACATAAAAGGAG
ATTTAACATTAATCTCGT
>TCONS_00027161
AAAAAAACAGAAGAGGAAGTTTATGCTATACAGATTACAGTACCATTTTACAGATTACACCATCAATACCAC
AAAGCAAACTATACTCTAGATTTGTCATATAAAAGGAAGGTATTGAATACAAGGGCAATTACAATATTGATG
AACCAAACAACCTGAAAAACAATAAGTTTACCCCTTATTAGATCAATCACTGTGATTGCGCTTCAAGTTAGA
AGGCAAAGGAGAAGAAGAAACCAAATGCCTCCACAGATACTTCCCACTAGCCATATCTACGCAAGTCCAAA
ATCCTTTCCCAATGGCGCTGCACACCTGGGAGGAGAGAGGAAGCGAATTTGAGTCTCTGGATAAATCGTCGT
GACTGTCGTCGGCGCCGGCGCCGGCATCGTCGTCGTCGGGATCAACATTAGGCTGGGATTTGAGGTTCCGGA
GAACGAAGAATCCGGCGAGAGTTGCAGAGAGGAAGATGAGAATGATCCTAAGGGGACACATTCGGTTTGTT
TCTCTCTCTCT
>TCONS_00027269
TTTACAACAAATGATGCATCCCCTTGCCCGTTGAATTGCAGCATAATCAATATCAGCAATTATAACGTCTTCCT
CATGCCCTGTGGTTGCAATTATTTCACCCATCGGTCCAACCACAGTAGAGTGACCCCATATTATGTAGCTACT
GGCTGAATCTCGAGATGGGGAGCAAGATGCCACATAAAGCTATGACAAATGCAGTAACATGTCTTTTCAGTT
TGATT
>TCONS_00027288
TAGGATAGGTTTTCTTAGCCTTATTCTTATTCTAGTCTAATTAGGATTAGTTTTCCTTAACCTTACCAATTTTACT
CATTGTAAGGCCTATTTAAAGGGCTTAATATGCTGAAAATAAACATTGGTGATTAGTTTTTCTAAGCTATTGCT
TCAAAAACGTGATTTACTTTTTATTTCATTGTTCTTCCTTTATTCAACGATTCTTGCAATCTTGCGGGATTGAAG
AACGTGTGGGTGAGGTTTCTTTTCATCTTACATTCTTCTAACATGAGTTTGAAGAACGCTTTCGCTAGTTTTGTG
AAAAGCTTTAGCGGGGTGCTTTTGTTTTTCTCCCTTCTTTGTACTTGAGAGGTGCAAAACCTTGAAAGTACATC
AATTTGGTATCAGAGCACAAGTTCCAGGTCCAAATTCTTGAT
>TCONS_00027300

TCAAAGCTGTTATAAAATCGCTAGTAAAGTAATAGCAATAATCTACATCAACTTGTATCTCTTCTCCTGGACT
CCAGTAACTCCCATTTGTATCTCTATGATCGTGTGAAGTGGCATCTCTATATGTGGAATTTCGTTCAAAGGCCT
ATCAGCAAAATAAGCATACAAAGCCCTCAATACCGCCTGATGTGATATCACTACCACGGGTGCTCTCTGTCTT
TCAAGCTCTATAATCACTGGCTCCAACCTCTGTATGACATCAAGGTAAGACTCGCCTCTGGGGTATCTATACC
TCAGCTTGTCATTTTTACGTGACCTTCGTTTTTCATAGAGAATGGGGAAGAATGCATCATACAAGGTTAAGAA
TAGTTGTGACATACTCGTATTCTTCAGGCATATTTTTCTTTATTTCCTCATAAGTCATTCCATCACATACACCAG
CATTGATTTCATCAAGGGCTCGCCATTGTATCTTGGGGAATTCACCAATTGGACTAGCTGTCAAAACTGTTCTT
TGCAGTGTGCTGGTCCATATAGAAGCAGCCTTTTCATTTTTCAGTCGTTTTTCAACAAATTTGGCAAGCTTCTTC
GCATAGACCTCCCCAGCTTCACTTAATACAGTGTCACCACCAATTCTGCCTCTAACATTGTCTAGGCTCTCTCC
ATGCCTTGTAAGCAAAATTGGACGAGGCGTAAGATGTGTATTGACCTGCATATCAGATAAGT
>TCONS_00027301
TTCAGCCAGTGAGCTAAGACTGTTGTTTACTGTTTCATTTCTACTTCTGCACTGAAGCAACTGTTCCCTCAGAC
TCCGTACTAGCTCGTTTAGTCTCTGGATGTTCTTCTCCTGTGATAAAATAATCTTTTCTTTAACAGCTTCTTCTCT
ATTTGAAATCTGTTGTTGCTTCTGAGATATACTAGGACTCGGTTCGACATCACTCACCAATTGCTGCTTCCATT
CCAACTGAGATGTGATTATGAGTACAATCAACAGAAAAACAAGCAGTATTGGTCTCGACATCATTTACAAGC
TAATCTCACACCACAAGATCTAATCTTGAAGTGGAGTTCAACTAACCATAGGTCCTGTAATTGTGAAGTACCA
AAAAAGGTCTTTT
>TCONS_00027375
AGGGGTCATCGTCCAGGCCTAGGGTCCTGACCAAGGCGTTGACTTCCTGAAACTACTTGTGCCAAAGTAGGG
AGCACACGAGCCGAGGGTCTCGTTGCTGAGAATTTACTTAGCCATGCTAAGGTATGAGACATGAGCGCTGAG
AACCATGAAGCGAGTGGCACCTCGTGGATTGGGCCTATTCGATCAGGTTGGGATCGAACTCGTGCCGATCAC
ACGGTGACTGAGACAGAATTAAGTCAGGATAGTTGGAACTCCCAAAGTAAAAAGTGAAGTATTTTTTTT
>TCONS_00027376
ACCAAGGCGTTGACTTCCTGAAACTACTTGTGTCAAAGTAGGGAGCACACAAGCCGAGGGTCTCGTTGCTGA
GAATTTACTTAGCCATGCTAAGGTATGATACATGAGCGCTGAGAACCATGAAGTGAGTGGCACCTCGTGGAT
TGGGCCTATTCGATCAGGTTGGGATCGAACTCGTGCCGATCACACGGTGACTGAGACAGAATTAAGTCAGGA
TAGTTGGAACTCCCAAAGTAAAAAGTGAAGTATTTTTTTT
>TCONS_00027453
ATCAAGCCACAGTAGTTTACAATATCCCTAGACCCTGGACCTGCACCACTATTTTCAGTCTCTGGAGTGGGAT
GCACACTTTCTACAAAATCCCTTGATGATAATGAGTCCATAGATTGTACTTGATGAGGGTTACTCGATTGTCCC
ATATCTGGAGATGGTTGTGTTTGTGGATTAGGCCTAAAGGATGACGTCACACTAGAATTATCATGTAGTACTC
CTACATTGCTGCATCCACGACTCTTTC
>TCONS_00027454
CCTTTCTTATTATCTCTCTTTTCACAAAGATTACCTGCACCACTATTTTCAGTCTCTGGAGTGGGATGCACACTT
TCTACAAAATCCCTTGATGATAATGAGTCCATAGATTGTACTTGATGAGGGTTACTCGATTGTCCCATATCTGG
AGATGGTTGTGTTTGTGGATTAGGCCTAAAGGATGACGTCACACTAGAATTATCATGTAGTACTCCTACATTG
CTGCATCCACGACTCTTTC
>TCONS_00027489
TCTCTCTCTCTGAAAAAGTTGAGTACTTTTATAGTTCTCATCAATTCACAGAAGAAGATGGTTTTCTGCTGATG
GCTGCATCAAATGATTTGAAAAGCTGAGTCAAAGCAGAAGGGGAAAAAAAACTAAAGCTAAGACAACAAC
CCCAATATAATCAATTCAATTCCCAGAGAAGGAAAAGACAAGAAAAGAAAGAAATTGATTTGAGCATGAAA
GGTAAATAGGAAAGTGAAGGGACTGAAACAAGTATAATACTAGTACTGATCTATAAATTCTATAGATAGATA

CATTAGATCCTCAAAAAAGAGGCTATAAAGATGATGATAGAGCACGTGCACTTCCAGATTTCTATCACACCT
GCAGGGTTCATTTAATATAGTATAAAAGTTCTGCAAGAAGGAAGAAGACGTCTCCTACGTACTACTACACAA
A
>TCONS_00027493
AGCCAAAATTAAGACTGTCCTTCAACCTTGTTCTGCCACCATTCCATGAATTTAACCTGTGAAGCCATGGTAT
CGAGCCTATATAGCTGCAGCCAGTGGTTCGCGACTTCCTGTCCAAATGTCCATCCAAAGATAGCACCAGCTCC
AAAAGACAATGCAGTACCATGCAGACTCCTTGAGTACTTCCAAGCAACGCCTGCAGTTGAAACAGCTCCAAT
TAGGCCTCCTACAGCACCAAATTTTGCTGCTTCCCTAGCAGTCTTCAGAGTGGGTCCATGAAGAGGGTCAGAA
GCAATGAATTCGTCGACATCAGCTGGAGACCAAGAAGG
>TCONS_00027660
ATCATCTTAATCTTGTGTTGCTGCACCTTGGTTAAGCTCTGCGAACCTCATACCAACACGCATTGAATGCTTCA
AGAACTTCTCAGCACATCGCGTAATACAGGTCTCCTCTTGTTTGTCTAATGTTTTACGTTTGAAAGTGTCTACA
CAGCCCGTGAAACATCTTTCCACCAGGGAATTATACATTCTCAAGCTGTCACGGATTTGGAGCTGGTCAATCA
TAGAAGCCATTTTAAGCTTGTCTGCTTCAGGAAGATTATCCAAATCACCTAACATGCTTTTATCCATTTTTTGA
GCTCTTTAT
>TCONS_00027713
TGCCAAAGGTAAATGAACATAACTATGGTTGCCCAAGCTAATTCTAAAGAAACAACTTTCAATTGCCTTCATA
TTTTGGGTCTGCCATCACATAATGGTAAGCAACCACCAGCACAAAGATAAATATACCAAGAAAGTTGGCAAA
GAAGCCCAGGTCCTGGTCGTCAATCATAGTGATACTAAGACGAAATGGAGAATTGGTGTAAAATTCGAAGAA
>TCONS_00027895
GAGGGCATAAACACAAGACCCAGCATATCTCAGATCCTTGTGGCAATGAAAGTATCTTCTTGGAGGCACAGC
TTTATGGCACTGCTGTTCATATGATGAAGCATATCCCTAAAGGCTCTCTCTGAGAGGAGATGCAACCAGGAG
ATCAACAGTTGGCATTTGCTGCTGGCTGTTTGATTGTGTGGAGAGTAAGCTCATCTTTATGCAATGGTAGTTAT
AAC
>TCONS_00027925
TTATGATCAAGAAAGAACAAGGAATAAGCATAATATAGAAGTGATAGTATGGCCGAAGAACAAGAGATGG
AGTTCACACCGCATAAACATGTTTGGCACCAACTTTTGCACAAAAGAGGGATAGGATTCCTGTGCCAGCACC
CACATCAAGGACCACTTTATCCTTGATTAAGAAGGAGTTCTTGTAAATAACATTTTGGTAAGTCTTAGTTCTCA
CCACATCCTTTAACATCTCCGAAGTGAGAGTATGAGTCGAAATAATAGTCAGCGCTAGTCTTGTCGCCCCCAA
CCTCACACATGGAGGTGTCTTCCTCCACCTCAACGTTGGAACTTTCCGTTATAGTTTCATCTTGCTCTTCATACT
CACATTTCATCTTGCTTCCCGAGCTCTCATTCTCATTGTTGCTTCGACAATCCATTTTTCGCTTCCAAGTGTAGG
GGTTTAAGAAGCAAATGGTGGCTCCTATTTAGGGTTTATGGAAGGGGAGCGGTCAGCTCTATTTGG
>TCONS_00027940
TTTCCAATTCTTCCAAAGTTAATCCTGCTTGGAATCATCCACTCCAACTGCCTTAGGTATAGGGTGAGAGGTGC
CAGAACCTGGTTCTCCTGTGACAGAATTATTGGGTTTAGTAGCTGATGGTTTAAAAGGACAAGCAGGAGCTG
CTCTTTCCGGTCTCTGATACTCCTTTCCAAACATTCTATAAATCTTTGCCATGAGAATATGGAAGAGAACAGGT
AATAAATTAACGATCGGGACGAGGAACAGCGGTATTAAACACGCTACGCAAACCATTTTCGATTGCTTTAGA
AGGCTTTGTTCTCTTTGCTCTCTCGTGCGTTGTATCGAGAAGAAGATGTGCTATCGAAGAAGATGACTCGTC
>TCONS_00027966
CAACTCAAACTTGTCAGCATCCCAGTATTTCATCAGACGATCTTTCCCCACCGTGAAAAAGTAATGGGTGTTG
CGCACAAACTTAACTCCCATAACGCTATCAGCATGCGCGAAAAGTGACTTGTGGCAATCCCCGAAATCTAGA
CCCCAAATCTTCACATTTTTGTCTCCAGAGCCACTGACAAGCAGATCCCCATCTGAAGAAATATCCAT

>TCONS_00028044
AAAAAAAAACAATACAAAAATTTCTCATATCGAATTCGAAGTGCCATGCTATTATTACTTAGTATTCATATGG
CGAAGGCATAGTCTTCTTTTTTCTCTCAAATAAAAACCTCATTGGCGCCAAGCGTGAGGGAATGCTAGACGTT
TGGTAATTTCTCCTCCGACCAGGATAAAAGATCCCATTGAAGCGGCTAATCCCATGCATATTGTATGGACATC
TGGTCGCACAAATTGCATAGTATCATAAATAGCCACCCCAGGTATTACCCAGCCCCCAGGAGAGTTTATAAA
CAAATACAGATCTTTGGTCTCATCCTCGATACTGAGATATACCATAAGACCAATAAGTTGATTCGAAATCTCG
CTATCAACCTCTTGGCCTAAAAAAAGTAATCTTTCTCGATAAAGTCGGTTGTATACGTCAACCCAAGATGCAT
CTTCCTCTCCAGGACTTCGGAAAGGAACTTTTGGAACAGCAATAGGCATTAAATGAAAGAAAGAACTAAATA
CTATATTTCACTTTGAGGTGGAAACGTAACAATTTTTTTATTGTATTTATAATATTCATATTGGTTTTTATCGTA
TTTATTTTATCCATAGATTATAAAAATTCATAAAGAAAGACAGAATGAATAAACTCAAATTATTACGAATAG
GTCTTTCTAATGATAAATAAGTATGGACTCATTCGTTCATAGAAAATGGGATCAACTCCC
>TCONS_00028059
AAAAAAAAAAACTAAAAGCTCTCTTCTGATCAAAGTTGACAAAACCAAACAAGAACTCTCTGCCTGCTTACT
TTGCAGCACTGTGCTTAGCTTTCGAGTGAGATTCCAGAGCATTTTCAGAACCAAAACCCTTGTTGCATGTCTTG
CAGAGATGAGAGCCGCCTGATTTTGGAGTCTGCTGGTTCTTGTTTGCAGGAGATTTACCAGCCTGTTTTGAGG
GGTGAGGGGTCGCAACATGGCCGCTACCTTTTTTGCCATCAGTTTTTTGAGGAGTTACAAATTTTGCCTTCTTA
TCGGGGATAGGTGTTTTTGTAGCCGAATCTGCCTTCCTCTTTTTGCCAGGTTCAGCCTTCTTTGGTTTCTCCTCCT
CACTCTCATCAGACTCACCATCCTCATCGCTCTCATCTTCACCCTGAAGAT
>TCONS_00028060
TTACTAGGTTCCACAATCTTCACCTTCTGCTTACCTGAAGCAGAATCCTTTGCAGCATTAGACTTCTCTGCCTC
CTTAACCTTAGCCTCAGGTTTTCCATTGTTTGCAATAGTGAGGGGGATGTCCTCATCAGACTCTTCTTCATCGT
CTTCATCATCCTCTTCCTCCTCAAACGGGTTAGTGGCCTTGTATCCAAAAAAGTAGACGCTGCCATTTTTCCAG
TTGTGTGATAATTCAAAGTCTCTATCAAAGACCAGGTCAAACTGCTGCTGAGGCACCTTATCCGAGCTTAGTG
TCCCAAGGACAAGTTTCTTGCCATCAATGTTCACGGACAAGCAGACACTTTGTACTTTATCCTTCTTCAACTCA
CCGAGAGATGCCTGTGAAAGATGCAAAACCATCCCATCTCCAGGCTGTACAGTTAAAGGCTCTCCACTTTTCA
CTTCAGCACCCCAAAACTCCATAGCTGCTGTAGGGTTTTAGAGAAAAAGGAAATTAGGGTTTTGGGAGAGAG
AAATGGAGGCAGAAAGAGAGAGGAATGAAAATGGTAGGGTATTTTGTGTTTGGG
>TCONS_00028064
GGTGCAAATCTGCCATTAGTTTCCACAATAGCACTAGAATATTTCGTTTGAATGTAACCTTCTTGCGATGAGGT
TTCTTGGGAGATGTGAGGGGTTCACCTCTCATCTTTCTTTGCTGTTTCTTTTGATCGTATATGACTATGCATTTGT
AACCATTGCTAGGGATGAAGTGTACATTAACAACTTTCTGCTGTATAGAGTGCTGAAGTTTGATATCAATGTG
CTGTTTGCAGGCGTCTGTTCTGTACATATTCGTTCAGAATGTCTTTGTAGAGCCCGTAATTTTTTTCACAAATTC
AAGCAGCCATTTCTCTGAGAATACCATTTGCAATTGGCTTGTACACTTCCTCCTGGAACATTTGTTTAGCCATG
TGATTTTGTTAAAGCCAGCAATAACAGTTCTTTAATTTCTGTCCAGAAATGATTTTGTATGTATAAGAATTATA
ACGGGAATTTGTATTATAGGAAAGCACACTTCGATGTACTTATTTTTGAGTTAGCGTACATGCAATTTCTGCCT
CAGATGTTTCCTAATAGAGAAATAGTGTCTACAATCTCTAGATCCATTATTGCTCAAACATAAGGGTTAGTGT
TCTGTCGATGAGAGCTAGCTTAACAATGTGAATTCCTAATGATTACTTAGATCTAGTTTTGGACGTTATTAAAA
CAAAATGGGGTATTATCACTTTTAGCCCG
>TCONS_00028101
ATCAAACGGCTGGATTTGAAGGTGAGAAGTGGAGGGAATGATGTAAGAGTAGGAGTTAGTTGTGGGTTCGTA
GTGGAGTTTGGATTTGAGGATAGGATTGGAATTTTGAAGTTTGTGGAGGGCATTTTGGAGAAGTGAAATGTCA

GGGGATTTTGATAAGAGGAGAGCTAAGACTGTTATCCCTGTGCCACCGGGAACCGCCTTGCACCAGCTGTGC
TCCGTGCTACCTGCCGGCCGGGTTTCAAACTCACACGGCGGAGCTGCGGTGGTG
>TCONS_00028181
ATACTGAATTTTCAAAAAATTAAAAAAAGGTTTAACCAAACCTCAATCTCAAGCCTTTCCAATTCCAGAAATT
TGAAGTGAAATTCATAGAGTCTACATGTACATGGCAATTTAAAGCATTATTTTCAGAGAGTGTTTTTCCTTAAT
AAGTGGCATCTAATTGACAAGTTTCTTTGCACAGGCATTCTTACTATTCTAACTGACTACATCATCTACCAAAA
TCCCAAAGTTAAATAAGCTGCATTGACAGTCCCTTCTAAACCCCTCAGCACTGAAATGTGCACAAATGACTCC
CAAATTGCGACACAATATCCACTAATGCTCCGTAACCAGCTACTGCTGTTACCCCTGAAGGATGTAAAGAAA
TGGAAAACAGTGACTGAGGAGCACATGGTATCTGTGAAATAATATCTCCGTTCAGGTCAAAGCGACTAAGCA
GGGGCTCCGCTCCAACCGCTAAAACTTGATTATCATGAAATATGGCATCCTGGATAGCAGCAGTTGTCGTGAT
TCGTGAAACGCATTCACAAGCTAGAAGGTTCCAAACAGATAAAGACCGGCCACTTCCACAAGCCAGCCAAC
TCTCACTTGCATCAAGAGCAATGCAACTGACATATGGAAACAACTCCCTCGACCTCTTATCCTTATGTGGATC
AATTATTTGAATACACTTACCAGTTCTGCAATCCCATATTCGTGCTGTCCCATCCTCTGAACCAGTTATAACCT
GATTGTGAGAATTTCGCGCAACAATGCAATGCAAGTAGTCTGAATGCCCTTTCAAAACCATCTTTATTTCATTC
TTCTCCACATCCCAGCAGTATGCACAAGAATCACCAGCAGCAGCAAAAATGGATCCAGTCTGAGTATTAACG
GCCATGCAATTATTCTCTGGAATTGGAGAAAGTGCACCCCATGGACCTTTTTGTTGAGGATTGACCAAGTCAA
GAATTGGCTCTGATGTGCCTCCTTGCACCGGCTCCTTAGACCCCAACAATTCTTTCCATTTCCATCCACGAATC
CGACCATCATCACCACAACTGCATGAAATCACCTCAATAATAGTGAATCCTCATCGTTGCCATAAAATTTAAC
ATCATAAGCAGGCCCATCATGTCCTTGTACCAGCCAACTAGATTCAGCCACTGGCACCAACGAATTGCCCGC
GGAGCTCAAATCGAGGCAAGAGGAGATGGAGTAAGAGGCGATAGTGCCGTC
>TCONS_00028182
CTCAATCTCAAGCCTTTCCAATTCCAGAAATTTGAAGTGAAATTCATAGAGTCTACATGTACATGGCAATTTA
AAGCATTATTTTCAGAGAGTGTTTTTCCTTAATAAGTGGCATCTAATTGACAAGTTTCTTTGCACAGGCATTCT
TACTATTCTAACTGACTACATCATCTACCAAAATCCCAAAGTTAAATAAGCTGCATTGACAGTCCCTTCTAAA
CCCCTCAGCACTGAAATGTGCACAAATGACTCCCAAATTGCGACACAATATCCACTAATGCTCCGTAACCAG
CTACTGCTGTTACCCCTGAAGGATGTAAAGAAATGGAAAACAGTGACTGAGGAGCACATGGTATCTGTGAAA
TAATATCTCCGTTCAGGTCAAAGCGACTAAGCAGGGGCTCCGCTCCAACCGCTAAAACTTGATTATCATGAA
ATATGGCATCCTGGATAGCAGCAGTTGTCGTGATTCGTGAAACGCATTCACAAGCTAGAAGGTTCCAAACAG
ATAAAGACCGGCCACTTCCACAAGCCAGCCAACTCTCACTTGCATCAAGAGCAATGCAACTGACATATGGA
AACAACTCCCTCGACCTCTTATCCTTATGTGGATCAATTATTTGAATACACTTACCAGTTCTGCAATCCCATAT
TCGTGCTGTCCCATCCTCTGAACCAGTTATAACCTGATTGTGAGAATTTCGCGCAACAATGCAATGCAAGTAG
TCTGAATGCCCTTTCAAAACCATCTTTATTTCATTCTTCTCCACATCCCAGCAGTATGCACAAGAATCACCAGC
AGCAGCAAAAATGGATCCAGTCTGAGTATTAACGGCCATGCAATTATTCTCTGGAATTGGAGAAAGTGCACC
CCATGGACCTTTTTGTTGAGGATTGACCAAGTCAAGAATTGGCTCTGATGTGCCTCCTTGCACCGGCTCCTTAG
ACCCCAACAATTCTTTCCATTTCCATCCACGAATCCGACCATCATCACCACAACTGCATGAAATCACCTCAAT
AATAGTGAATCCTCATCGTTGCCATAAAATTTAACATCATAAGCAGGCCCATCATGTCCTTGTACCAGCCAAC
TAGATTCAGCCACTGGCACCAACGAACTAAACCAAATTCACCAAATAATTATCAATTCAAACCAAAACTTAC
CTTCACACTTCGAAAACATTGAGAAAACAGGAACTTACTTGCCCGCGGAGCTCAAATCGAGGCAAGAGGAG
ATGGAGTAAGAGGCGATAGTGCCGTCGCTAATACCGGCGACGATGAGGTCCGGATAAGAATTATGGTTTGGA
TTCGGGTTCGGGGCGAATGCAGTACGGAAAACAGTTCGGCATTGGGATTCTCTCTCCAGTAAAATGGAGTCTC
TGTATACGTCTTCGTCCCAGTTCCTGCAGTCCATCGCTGTTACCATCCTTACCAGAATATTTCGGTGTT
>TCONS_00028236

CTTTGAGATTTTCCAGAATGTCATAAGCAGACTCCATCGACTGATGCTGATGTTGCAGAACATTTGACATGGA
TGCCAAAATGTAACACCGCGCCATCTCATCAGCCTTAATCCATTTCTGGTAAGCCTTTTGTTCATCATCTGTGG
CATCATATCCAGGTTTTTCTGGACACACCTCATCGAGCACAAATTTGTACTCTTCAGCAA
>TCONS_00028253
CTTGATCTAACTGTATGAGTTTTTTTTGAAGTTTAAAGAACACATTTTTCATCTCATCTGTTACTACCTGTTTAG
TTTCCTTCAAAAGATAAGTTAGGATTTATAGTGCCATACTGGTCGTTATTGTGATCTATATTTCTGTCATTAAA
GTTCCGCTTCAAGTTTAATCTGTTCTATTTATATATAAAGTTTCTTTCTTCGCCTCTATTATGTGATAATTTCCA
TGGGCAGGAGGTTTGGACATATTGGTGAAGTGTCCTTTAGCTCTAGAGGTGAACAAATAGGCTCAGGTAACA
CAACACTTCTTGGAACCACGTATGCTTACAGTTTATCCAAGTTTAGGTTGTAGAAATTCTTTACCTACGGTGGA
GTACGGATGTTTTGAAGCTATTTTGCTGAATACTTCTTTCCATTTGGGCCTTGCAAAATGCGTCCTTACCTGCA
GTAGAATTAGTTTAATTAGTACTAGCTGGAGAACGATTCACTTCAAGTTTGTTTCTCTCTATTCTGTGTTGTGGT
TGAAGACTTCTCATGAAATTTAGCAGCTTTCGAGTAATAGAAACTTTCTCTATCTAGAACATGGCACTCTTTAA
GCTAAGCTGAAAACTGTTCTTTCTTATATGTTTCTTTTCCATTGCTCTTTTAGAGGGTTTGGTTGAGAGTGGTGA
TGGATCATACCTGAGTGCCAGATAGGATGTCAACACCATGTTTTGCATGGTTGAGCGCCTGGACCATAGTGAT
GAGAGTTGACCTGGCATCGCCCTCTTCATAAACACTCGTTAGATAATTGTGCATCTCTATCAGACCCTGTTTCA
TTGAAATCATCAGAAAAGATATAGCTGAGCCAATTAGTTATAGATCATAGCAGAATCCCAGAAAGGAGGAA
AAAGTGTG
>TCONS_00028352
AGTACTACCAAAAACCTAAAATTTTTGAAGAAACAAAGAAATTTACTTGGTTAATGCAGCATCATAATCTGT
CTCTACACGGTAATTAAAAGCATCATCGTAACCACATTCTTCTTTCAATAGCTTCACCTGGCCAAGTGGAATTT
GAGGAAGAGAGAGGCCATCATCTAGACCACTCAATTGAGTGCGTACATATGGATCAATGGAGACATAGAGG
ACTTGGAATGTCACATGTCCATCTGGAATGGAATCAGCTCTTAGCGATAAACTCACAGTACGTAGCTTAATAT
GCTCAGAATTGGGAACACCTGTCGGTGCATAACTAGCAACGTACCACTCTCT
>TCONS_00028401
CCTCATTTAGCACATTCTTCAGCTCGTGTTAGTGCATCGGTTATAACATCTATTGTCCAATCCTTGACATCTTCC
ATCTTCAAAGGTTCCCCTGTCCTTAGCCTTGCATTTGGACGAGCAAGTTTAGATTCAAATGGAGTTCTTGGTCT
CACGTCTTGAATTGATTCCCATTCCTCACGGGTAAGCATCCTAACTGACCCTTGACCATCAGGCGCATTCTTTA
TCTTTCTCTCAATCATTTCTTGAGACTTCGAGTCATCATACATCATGAGGAGCTTAGCAAAAAGTGCGGTACC
AACGACAGTGAGTATCATTGGCATAGTAGCTGTGTCAAGCCATGCTCTTGATTCTGGAACTTGAACGAATAAT
TGGTTCTTACGAGGATCTTTTCGTTTCTTCTTCTCCTTCAATTTAGCAAGATTCATCTTTAAGGTTTCACTAGGG
CGATCACGAGACAACGTGTGAACTTGTCTACTAAATTGGCATAATTTTCTACGAACACCCAGCATAATCGGA
CGAAATGAGGGG
>TCONS_00028469
GAATAATAATGAGGCTAGGCTTCAATAATCTTCATTCATCTCCAAATTTGAACCATTTCAAACATTGACGGCG
TTGCAAAGGAACATAAGCGACGACGTACACGCAGCGTCGCCGCCGTGTCTTCCTCCTCCTCCACCTCCACCA
CCACCTCCGCCGCCGCCGCCCAACAACACCCTTTCATAATAATCCACACAAAACAACGATAACGACAACTCC
ACTTCATGGCGCGTCCAATAAACCCTCCTCACAACCTCCGAATCAGAAGCTGACTGATCCACTCCTATTGGTT
TCCACTTTCGCAATCGATTCAACACTGGCCTAACACACACCATGTACAGCCGCCGGTACCCTCCTAGCGCCAA
AACCACCGTCCTCACTCCCTCCGGCGGCGGCGACACGTGGCGAAAGCAGAGGTGCTCCCACAGTGAGTCGTC
TCGAGTGATGGCGCACCATTGTCTACATACACATGCAGCAACTCCTAAGGAACGACCGTCGAGCCTCTTCAA
TATATCTATCAG
>TCONS_00028471

ATTCAAATCAATTTTGGCGCTTAATACCAACATACATGAGGAGCGAATAGTCTATATGAAGCATACGGTATAT
AGAACTTAACATCTCTTTTACAAATTCAAGAACTGTCAAATGATGCTTACAGGAAATTTCCACCAGTTTCATT
CCTATTTACCGAAAGTGAAACAGGGAAGTGTCACGAGCAAGTCTGATTCACCAAGCACTCATTAAATCTGCA
GCATCAATCAAGTTTAGCAACTTCAAGTTCGATGTGGTAAGTCCGGACATTCATTCTCCTAAAACAATAAACT
TGCCCAAGCAGCAATAAATGCAAAGCCACCGAAGGGAGCTAAGGTTGAGTATTTCCTGTCTTCCAGAAGTGC
AACTGCATAGCACGTTCCAGAGAAAGCGATAATTCCACCAGTTAGCAATCCTCCAAAAATGTTTGGATGTTT
AGTGATAGGAGCAGAAACAAGGGCAGCAGTGTGAACCAAATGGTACAGTGATGCAGTATGCCACACCTCTT
TGTAAGTGGGATTTTTGGGCTTGAAGACATGAGCTCCATAAGTTCCTAACCCAAGAGCAGCTACACCAGAAA
TAGCAGCCACTTTGTGCCAGATTTGAGGATTCATTTTTGGTTTTTCT
>TCONS_00028485
CAGATTTTCCTTGTTGATATAGGAGACAACTTTTCTATCTCTAATTTGGCTCAAGAATCTTACCAAAGCAGCAA
TTGAAGCAAAGATTGCAACAGTCAAATAGCGGCCAAGTTTAGCATCTGCAAGGAAACCACCTAGAAGACCA
AGAAGATTAAGTGTGCCCATGAAGTTGGTGACAATGTTGGCAGATTTGGAGGCTGGAAGATGCAAGTCTCCA
ACCAAGTTTGTTTGTACTTGGAAGCATCATTTTGTGGTTGAAATAGCTTACAACCTACTGAGCCGTTAGATTGG
GAATTGACATATGTTTGAATGAACGTGCTTCTTATCTACACAGAATGGGACCAGCTGTTTCAGCCTTCA
>TCONS_00028527
CTGACACAAAAAGAAAATGTAAAAAGATAGTAAAACAGCAAAATATCAAAATTGTCAACTAATAAGTACAC
CAGATCATGAACTTGAGATATCTGTTGGCACCACTTAAATATAATGGATAAAAATGAAAGAATGAGAGGAA
CAAAAGAAATCGAAAAGGCACCATAGCGCAAACTCAGAAAATATAAAATCAAGAAAAATGGACAAATCCA
ATAAATCTAAAAGTAAAAATGAGAAATATATCCACTGGAAAACATAAGACATAAAATCTGCCAAGTCCTGT
AGGAACTTGGCTTAACAGTAAGAAGTATGGATTATATGGAAGTAATGAAATAAGAAGCAAATGGCTACAAA
TCCAATAACAAAAAACCAAGCCACTGACCATAACAAACTCATCAACACTTCAATAACTTTGTTTCCTCGTGGT
CAGGCCACTGTTCATCACAAGTGCGATGAGTAGCCACTACTCCCCGGTAGCTTAGCAGGTGAAGGTTGGCCA
ACCTGCTTATTTGGGTAGGCCTGCATTAACCCGGGCGCCACTCCCCCGCTAGAACCACCAATGCTGCTACCAC
CAACACCACCACCATATACCCCTAAACCTGAAACACCCCCTCCTAAACTACCCAACATCCCACCATAAACTC
CTACAAACGGATTCACAAATCCTACATAAGGATTCACAACCATTCCACCATACAATTGATTCATCATCGCTAG
ATTCGGGTCCTGACCCATCATCCCCATATTCTGAGCAGCTGCAACTGATGGCGGCATTGCCAGCAGCGGGTGG
TGTATAACCTCGGTCGTAATACTGGCCTCTCCTCCACCAATTTTTGGCTCGGCAGCCTTCTTGCAGTACAACTT
CCGCCCCTCAAACACCTTACACGGCTCTTCTAAACATCTTTTTGCCTCCTCAACCGTTTTATACACAAACAAAG
CATACCCTTTTGATTTCCCAGTTGTTGGATCGAACCCCATTGGTCCAACCTCAATTTCCCCGAACTTTTCAAAA
AACAACCTTAATCTCTCCGGACTCACGTCACGCGGCACATTACTCACGTAAATCTTCCTATTACCAAAGTCCA
TGGTCCCACTAACGGCAGTGTCCTTCACGGCAGCCAACTTAC
>TCONS_00028672
GCCAAGAGCACCCCCGCGAGTAAAAGCGTCCACGGCCGGCTGACCAAAATGAGGATCCCAAATTGCATTAG
CAATAGGTCTTACATGTAAAGGTTCCTGTACCCACGACTCAAAATTTCCTTGCCAAGCTACATGAAACAGATT
TCCGGAAGTCCACAGAAAAATTATTGCTAATTGACCGAAGTGAGAAGCAAAAATATTCTGATAAAGACGTTC
CTCAGTAATATCATCATGACTCTCGAAGTCATGTGCGGTAGCAATACCAAACCAAATACGACGAGTAGTGGG
GTCCTGAGCTAAGCCTTGGCTAAACCTTGGAAATCGTAATGCCATAATGCTTTTCAAATCCTCCTATCCATTAT
CCTACTGCAATAATTCTTGCTAAGAAGAATGCCCATGTTGTGGCAATTCCACCCAGAAGGTAATGGGTTACTC
CTACAGCACGTCCTTGTATAATGCTCAAGGCTCTCGGCTGAGTAGCAGGAGCAACTTTTAATTTATTATGAGC
CCAAATGATGGATTCAATAAGTTCTTGCCAATAACCACGTCCACTGAATAGAAACATTAAACTAAAATTCCA
GACAAAATGAGCATCTAGGAAAAAAAGGCCATATGCAGATAATGAAGAACCATAAGACTGAATTACCTGGG

ATGCCTGTGCCCATAAGAAATCGCGGAGCCACCCATTAATATAATAGAACTTTGCGCAAAGTTTCCTCCCATG
ATAT
>TCONS_00028674
GCCAAGAGCACCCCCGCGAGTAAAAGCGTCCACGGCCGGCTGACCAAAATGAGGATCCCAAATTGCATTAG
CAATAGGTCTTACATGTAAAGGTTCCTGTACCCACGACTCAAAATTTCCTTGCCAAGCTACATGAAACAGATT
TCCGGAAGTCCACAGAAAAATTATTGCTAATTGACCGAAGTGAGAAGCAAAAATATTCTGATAAAGACGTTC
CTCAGTAATATCATCATGACTCTCGAAGTCATGTGCGGTAGCAATACCAAACCAAATACGACGAGTAGTGGG
GTCCTGAGCTAAGCCTTGGCTAAACCTTGGAAATCGTAATGCCATAATGCTTTTCAAATCCTCCTAGCCATTAT
CCTACTTGCAATAATTCTTACTATGAAGAATGCCCGTGTTGTGGCAATTCCACCCAGAAGGTAATGGGTTACT
CCTATAGCACGTCCTTGTATAATGCTCAAGGCTCTCGGCTGAGTAGCAGGAGCACTTTTAATTTATTATGAGC
CCAAACGATGGATTCAATAAGTTCTTGCCAATAACCACGTCCACTGAATAGAAA
>TCONS_00028813
AAAAGCAGAAAAGTTTGACATCAGTCCTAGAAATAACAGTTAAAATGGAAGCACAAAGCTCACATTCCGG
TAAAAGCCTTTTTTCCTCCACCAGCAGAACCAGCTGGAATCACCTTTAAGACATTGAACCTCACAGTTTTGGA
CAAAGGCCTGCACTGTCCAATGATAACATGATCTCCCTCTTTCACACGGAAGCATGGTGATATGTGCGCTGGA
ATGTTCGAGTGCCTCTTCTCATATCTCTGGTACTTCTTCACATAATGTAAGTAGTTGCGTCGAACAATGATGGT
TCTGTTCATCTTAGCACTGTGGCATGTACCAGCAAGGATACGACCTCGGATAGAAACATCGCCAGTGAATGG
GCATTTCTTGTCAATGTATGTACCTTCAACAGCCTCACGAGGAGTCTTGAACCCTAGACCGATACTCTTAAAG
TAGCGATTGCCTCCCTTTCCTGGCCTTTTCCCTTTCCCTGTCTTCTTCGAGCTTAGAAAAACACCTGGCTGCTTC
AAAAACGCCTTCTCCGTCTGCTCCGCCATGATTGAGGACTACAAGCTGAACTGGACGAGGATAGATGCTCAG
CGGCAAAACCCTAAAGTGCAACAGAGAGTG
>TCONS_00028873
AACAGTTTCTAAAGCATGATTCAGAAGAAAAACCCCAGGAACGAAAAGGAAGTAAGTAACCTAATGACAA
GGTACTAGCATTATCACAAATCCATATTCAACTTCTGTCATTGGCATCTATACTTGATCCTGCATCCGCCAGTG
CTTCAGCCATCATCTTTTCTCTATTCTGCATTTGTCTTTCCACAGCAACCATATAGAAGCCAATTGCACTCCCA
ATTGCACAGACCCATAAAAGCCTTCCTGACCATTTGTCACCAACGATTCCTCTATTCCCAAAGCCCATCAGCT
AAAACTGGAGCAAGGGAAACACGTATTAGAATGTGAATTTCTCGTCATGAACTACAGTAGAAAGGGAGGAA
GGAAAACAAATTTAAACAGTAAGA
>TCONS_00028886
ATGGTCCTTGCCATATCCTCAAGGGTCCTGTTTTTCCTCTCAACTACCCCATTTTGCTGAGGAGTCCTAGGGGC
AGAGAAATTATGATCTATTCCATTTTCATCACAAAATTCAGCAAACTTTGCATTTTCAAATTCAGTTCCATGAT
CAGACCTAATGGATGCAAGTTGATTTCCTAATTGTTTCTGGGTTTTTCTAACAAAAGCAGTGAACATTTCAAAT
GCTTCATCTTTAGAGGTTAGAAACAGAACCCAGGTAAATCTAGA
>TCONS_00028891
AGTGAAAACAACAATTTCAACAAGTAAGGAATAATCAAAAGCTTGCCCCTCGGGCTCAATATCAAACTCAC
AGTACAGTATCAGCCCCACGGGCTCACTCCCAACTCACCAGCACACAACATCAGCCCCTCGGGCTCACTCCC
AACTCACAACACACAAACAACGGCCTCTCGGGCCCACTCCCAACTCACCTGGGTACCCTGCGCTCACTGGGG
GTGTGTACAGACTCCGGAGGGGCTCCTACAGCCCAAGCGCAATATCAATAGGCCCGAAAGCCTTCACACATC
ACACTCGAGGCCTGAAAGCCTCCTCACATAACACTCGAGGCCTGAAAGCCTCCTCACATCACTCAACATATC
CTCACAGATGGCCCTCGGCCTCAATCAGTCCAGAAAATAAT
>TCONS_00028987

CCTGGCAAGAATCTGCAATCCCTTCTCGCTCATATGACCCATTCTTTGTGCCACAAATCTGCAGAAATCTCAT
CTTGTGCCGCATTCAATTCACCTTGGCATATTTCTGCATTTGTCCTGTACAACGTGCCACGAGCAACTCCCTTT
GCAATCACCAATGATCCCTTGGTGAGTCTCCACTTTTGATTTGCAAAATAGTTCTCGTATCCATCTCGGTCTAA
AGCAATTCCCAAGATCAAGTTCATCCGCAAATCAGGTGCATGTCGCACGTCCTTTAGAACCAATGTGCATCCG
ACATTTGTCTTGATACAAATGTCACCAATCCCCGCAATCTTTGAGTAACTTGTGTTACCCATTCTCACAGTTCC
AAAATCACCTGCTACATATCTACAAAAAAGATCTCTTACCGGTGTGGCATGGTAAGATGCCGCTGTGTCAAC
CACCCATTCCGACTCTGGACCTGACAGGTGCATGCATTCCTCTTCCTCATTTATAAAGAGGACAACATTATCA
TTGTTTTGCACCATGGTGGTTGTGTTGTC
>TCONS_00029017
CCCGCAAGTGGATACCATGATTCTTCTGTCTATCAATAACCGCATGCATTGCACGGTGGATGTGAAGAAGTAG
ACCATTATCTCGGCAATAATGAGCCAAGCTAGTATTTGCGGTGAATCCCCCCGTTAAGTAGTCATGCATTACG
ATGGGAATGCCCAATTCTCTAGCAAATACAACTCTTTTGATCATTTCTTCGCATGTACCTACAGTAGCATTCAA
GTAATGCCCTTTGATTTCACCTGTTTCAGCCTGTGCTTTATAAATTGCTTCGGTACAAAATAAGAAACGATCTC
TCCAACGCATAAATGGTTGTGAGTTCACGTTCTCATCATCTTTGGTAAAATCAAGTCCACCGCGAAGACATTC
ATAAACAGCTCTACCGTAGTTTTTAGCAGATAACCCCAATTTAGGTTTAATAGTACATCCCAACAGGGGACG
ACCATACTTGTTCAATTTATCTCTTTCAACTTGGATCCCTTTGGTAAAATCAAGTCCACCGCGAAGACATTCAT
AAA
>TCONS_00029018
CCCGCAAGTGGATACCATGATTCTTCTGTCTATCAATAACCGCATGCATTGCACGGTGGATGTGAAGAAGTAG
ACCATTATCTCGGCAATAATGAGCCAAGCTAGTATTTGCGGTGAATCCCCCCGTTAAGTAGTCATGCATTACG
ATGGGAATGCCCAATTCTCTAGCAAATACAACTCTTTTGATCATTTCTTCGCATGTACCTACAGTAGCATTCAA
GTAATGCCCTTTGATTTCACCTGTTTCAGCCTGTGCTTTATAAATTGCTTCGGTACAAAATAAGAAACGATCTC
TCCAACGCATAAATGGTTGTGAGTTCACGTTCTCATCATCTTTGGTAAAATCAAGTCCACCGCGAAGACATTC
ATAAACAGCTCTACCGTAGTTTTTAGCAGATAACCCCAATTTAGGTTTAATAGTACATCCCAACAGGGGACG
ACCATACTTGTTCAATTTATCTCTTTCAACTTGGATCCCATCATCTTTGGTAAAATCAAGTCCACCGCGAAGAC
ATTCATAAA
>TCONS_00029027
TGGAAAAATGTATTCACGGGATAATCAGAAATGACGCCACGTTCTTCAGATTACAATGAGCCTCATCACAAG
CTCAACTTCTCGTAAAACTTTTAGCACGGCTGTATGGTCTGCACTAATTATGCTAATAAAACATCAGGCTAAT
AACAAACTTTCAGTCTGGTCCTAAAAGCCAATTTGCTTGGAACAGGCAATTTCAAGAACTCTGGCTGAATTTC
CGGCCAGTCATCTTCTCAGATTCATGCAACATGTGGTTTATGAGCCCCCTCGCAGCATCATCCTTCAATTTCTG
AATTCTATCCGGATCTGTCTCGTGCATATGCTTTTTGAACTGCTGTCTCACCATACCAACAAGAAGCTCTGTGT
TATGTTGCTTGCTACCAATATATTTGCTCGTCGGAGACACTCACGATAAAGTCGGATAGCATTGTTGGCTAGT
TCAGGAGGCAATGCTGTTTGGAGAGACGGCATTGTGCAACTCGCTTCAAACTGTAGCAACGGAAAAGCAGA
GACAAAAGAACCTTAAATCAGAAACACTAGCCGAAACTTGTTCAG
>TCONS_00029096
AAGAAGAAGAAGAAGTAGTGATACCTGATGAAACCTTTCCCTCTAGTGCTCCCAAAAGATGTGTTGTCATTA
TGGAAGGGGATCCCCACGCTGGGACTACAAGAGGTCGGATGTCTTTTCAGTGTTTTAATCCTTCTATTGATAA
ATTAAGAGAAGAAGCGTCAAAACCACATTTCTGAAGGTTCTGCTGCTTGTTCTTCAGAGACGAGTGAAATAA
ATTCGAAAAGGTCAGTGTTTGATGCTTAAAGTTCTCATAGTAACTTGTGTACTCTATCCATTGTTGTTATACGA
TGAGCATTATGGCCGATTACTTCTACTATATACCATCTTGACTTGATCTTTTCACATCTCTTTCATCAGTAGAAA

GATAAACATTATTTCTCCTGGTATATCTTTGGCCGAATATGTTGAAAATTCCTTCTTAGTGGATGTTTGTTACG
TAGAGATGATAATAGGCGATATATTGTATTTCTAAAAAGAAGATA
>TCONS_00029128
AAATTTTCCACGCAGCTACAATGTTGCAAGAAAAGATTGCAAGTCTGCAGGTCATGACGGAACAATATGGTA
ACGCCTTGTACGATAGCAAAACAGCAAGATAAATCAAGATAAATGCCAAAGGATCAAATTGCATCCTCGCT
ACTTGAGCTGGTCAAATAGCTTCACCGCAACACATTTGTCAATACACTGCCAATAATCAAAATACTGTCCAGT
GCAATGCTTGTGCCCAGATTCATCACCTTCTATCCTTTTAGCACAAGCCTGATATTCCCTAAGTTGCCTTACAC
ACTTCGGCTTGCAAGTCTCTTCTAGTGTCTTCTTTTGGTCAACGACTTCCTCGTCCGACATGGCGAAAGAATAT
CGATGCCGTAGGTTCACCTGTTTCTCTTCGGGGGATTTGAGAGTCGCGGAGTGGAATGGAGCTGCAAAAGGC
AATTACTATGCAAGAGGAGAATCTCGAAGGTTCGTTGGGCTTTGTGATGGATGTTGGTCAATTAGCTTGCGCC
GAGTGCGTAGGGTACGCCGGGTTTAATGTAATTACTGGAATACCCC
>TCONS_00029204
AGTGAACATTAATAAACAAAATCATCACTCTTCCATAACTAATCAGACAAAAGAATCATCCATTCATTCATAT
AATTTTTTGGCATGTTGAGTTCTCTCAATCTTGCTTTCGTTCCTCGTGACTTTTTCCTCCAATTTCTCATTTTTTGA
AATAGCCTTGGCAATGGCCTTCATTTGACGTACATTCTTTGACCTTTTCATCTGTACTCCCTTCATCTTTCTATGT
GATCCACCTATATGTGGTTTTGGAGCTGCTGACTCTGAAGTATCCATTTCTTGGGGACCCGTGACCGTCTTGTC
AGAGGAGATATCCATAGCCGCACGACCATTTTTCTTATTCTTGTTTCTGTTCTTCGCCATAGCTATGCGAACAG
GGAGA
>TCONS_00029264
GATAGAGAATACTTGTCCTTGAGAATAAATGGAACCGAAGCGCATATTTAGTGTTCGAATTATCTTCGTTTAG
TATTATAAAAAAATGTTTTGGGTTTCTAACCGTGCCCACACTAGATTGGAATGGTAGGACAAAAACGTTGAAT
GGAAAAAGAAACAAAAAGCAAGCAAGCAAACCCACGCACGCAATCCAGGATAGGGCAAAAATGAAAATA
AAAAAGAAACAGGTCAAACATTGATCTCTACGTTAGCACATTCATCATCTTGAAACAAATCTATGGAGGTTC
ATACCAGCTGCAGCAACCTTAGTTTCCAACTCCAACCACAGATCTGAATCATTAACTACCCTTGCAGTGACAT
TATCCTTTTCTCAAAACCATATCCAGTTTTTTTTGGTAAGAAGTTCATTCCTGAACACTGGAAAACCAACGGAT
GTCACATTAAATTATACAATTTCAAGGGCAGGGAAGCAATTATGGGAGGCAATCGGAAGAGTCCAGGCATTA
ACAGCCAGCCGAAGTGCCATATTGATAAGCAAGTCAATAGAATACAGTCCATAATTTTTGTTATTTTTGAATA
TTTTGTTGCTATGCCTTCATGCACCACTTGCATATAGGCGTGTCCACTCCTTAGCCGTTTCAACAGCTTCAGCCT
CATTTGACTTCCAGTGCTTTGCAATGTTTTCAGAGAGTGGATCATCTGGATTTGGAGCACTTAAAAGTGCTTGA
ATGCTCAAAAGTACAGTACGGATCTGAAGTGCAGGACTCCACTTGTCTTTAAGAATATCAAGACATATCCTTC
CAAGCTTATCAATGTTAGGATGGTAGATTTTGGTGAGGAATCGAACCTTAGGAGCAGCCATCGGGTACTCTTC
AGGCAAAAAGAGTTCGAGCTTGAAGACACCTCCTTCATAAGGAGACTGTGTAGGACCAAGAATCATGACATT
AAAGTATCGCATATTATCTTCCGATGGAGATGCACTTATTCCTGGTGCAGGTTCGCTGAGAAGCCGTTGAGTT
TCCTTGATAATTCGGCGAGGAAGATTGCTGTTCGCCATTGGAATTGAACTGAAGGCTGCCTGGGTGGGTGGGG
TTCTTCGGTGGCAGAG
>TCONS_00029432
AATTTGATCTACAAGGTCATCTAATTCCTCACCCCAATCAGATGTTGTCCCAACAAGATCTTCAACTTCTACGT
CAAGCTCAGGTTGATCAATAACCAGTACTTCCTCTGCTCCATCCCCGGATATAAGATCTTCACCGAGCAACTC
TTCCCAAATATTCTCAGGCACTAGATCCATATCGGTTGCACTTGCTGTCACAACAGAAGCCGACCTTGCGTTG
CTGCTTGATTTATTGTCCAATGCAGCAGAGAACAGCATTGCCATATCGCTTCCCATATTTGTAAGCTCCGTTTC
ACGATCTCGAGTGGAGTAATTCATAGGCTGATCACTTCCGTTGGCAACTGATACAAAATCTTGAAGGTTCTCA
CTACTGGGGGTCATAGTTAGTCTCCTCTTTTGCCCAACTTCAATGCGTTGTTTATCTTTTCTTTGCACACATTTAT

CCAAGTATTGCTGGAGAAAAGTCGGATTGCTGAAAATCTTGGCTAGGAAACTCATCATCTGCTCTTGTTTCCT
CTCTGTGCTTTCGATTTTTTCTCCCATTGCAATGATCTGATTTCTAGTACTCTGCTGTTGCTGTCTAAGCTTAACT
ATTTCAGTCCACAAAAGATTCTTATCCCGCTTTAGTCTTTCGAGCTCCTCCTCCATTCCATAATACCCTATTTCA
ATACACGGCCCGGATCCTTGTTGGTTCATGCTCTGACCTACATTCCTCCTCCTCTTTATGGTCTTCAAAAGATG
CTTCTGTCCGCCTAGAAAACCTTCATTCGCAAATTCCCATCTATCCGGATCCACTTTTCTAAAACCCTAACGTG
CTTCTTTTCTTCGAGTATTCATTGTACAACCATCAAACACAAGCAGTAGAATTCATCAGAGCCACCAAACATC
AGCCGCAGAGACTATCTGAACTTCTGTCTAAAGATATGTGTTAAGCTGGCGAATGAAACTGGAGAAATTACT
GTGCTTGAAATGCTTAGGCAGCAAAGTGGTTGAAAACTTATGAGAATCCCAAACAATAAAGCTATTTCTTGCT
CTGCTCCATGAAATTACTGCATCCGTTGAAGGGTCTTCCACCATTTCATACGTCTTGCTCAAAAACGGTGGTG
GACCCACGTCATGCAGCCCCTCCATTGGTAGCGGCGCCGCCGTCACGGCGGTGGCAACGTCCTCTTCCTCCCG
CTTCACTCTCACTCC
>TCONS_00029434
CGATTGCATGATTGGCACTAACTTAGCAAGGTAAGATGGCTGGTAGACTGATAGAATAGTGTTTCTCATTGTT
GACCATCTGGAGTCCCTAGTAAAAAATAGGCCTTTCTGATGAAGAGGGGATGCTGCAATTGGAGATGGGATG
CTTCTATTTGGTATGTCCTTGAATTTCTTTATTCCAACTTCTCTGCATAGTTCTGCATCTGCTACAATAACTAGT
GGCTGTCTACCCATATGAAATCTGAAGATAGGACCATATTGTTTGGCAAGGACAGAGAAGACATCAGGGCCA
TATTTAGCCATCAAAGGAAGGTGTCCTACAAGGGGAAAAGCTGGAGGACCTGGTACTTTTCTCACAAGCCAA
TAAGGTCTATAGAAGTAGACCAAAACACCAGCTACGAAGGCTAAGATAGTGCATATTGTTGAGACCAAAGA
TGGT
>TCONS_00029435
CTCGCGGCCCTATTCCAAATGGAATATTTGCATAAGGATGCCTTTGTTTTTCCTCTTCACAGCTTGGATCAAAC
CTCTCTGGTCTAAACTTCTCTGGTTCGGGGAAGTTCTTTGAATCCTTAGCAAGAACTCCAAGAGCCAACCAAA
CCCATGTGCCCTTAGGAAGTTTATAGCCTCCGATCTCCACCTCAGCTGATGTTTCTCTGGCAACTAAGGGTGA
GACGACATAACACCTCATTGCTTCTTTGATTACCTGATCAAGGTATGGGAATTTCTGCTGAAGATCAATGGCA
GTGGGTATGTGATCATCAGGGCCAAAAGCATCTATCTCCGTGAGCAACTTCTGCTCAACCTCTGGATGGCCAG
CAACCAAATAGATAATAGAAGACAATGTAAAGGAAGTTGTTGCAGAACCAGCCAGTAAGTGCTCATAAGTT
ACAGCACTGATATAATCTGAGGTGAAAACATTCTTAGCTAACTTCTCTGACTCCCTTGCCTGCAGTATAAGCG
ATAAGAAGTCCTTTGAACCGCGATCTTTTTCCTCCATCCTCTTTGCTACTATCTCATCTAGCCTCCTGCTTAAAT
TCTTG
>TCONS_00029439
GGCGGAATTCAGTACTCAGGTCACACCACTGCTGGAAGTATGCAGCTCTTGAAAGAAGGATTTGCCAGCCTC
ATATGTCGACCAGCAAATGGCAGCAGCAGGAGCATGGAAGAGCATCCTTGGCATCCAGCCTCTCATGAGACT
TCTGTATCCATCTTTCTTCACTATTACTCGAAAAACATCCCTAATTGACCCACTTGTAAATCTGTCACACCCAC
AAACGCCCTGACACTGCAATTGTGTCTTCACAACATCAAGAGGCGTCGTAACCAATGCCGCGGAAGCACCAG
CTGCTGCCCCAGCAGTAGCATGAACTATTAATCGTTCATCGCTCGCATTCTCGGGCGATACCTCCGTTAAACC
CTTCTTGATAGCCTCATACGTAGCAAAGTGCACAGCTGTATATGGAGCATTCATTAGAAC
>TCONS_00029457
TCTTTGTCTAGTAATCAAACTGAAAAGACATGTTACTGCATTTGTCATAGCTTTATGTGGCATCTTGCTCCCCA
TCTCGAGATTCAGCCAGTAGCTACATAATATGGGGTCACTCTACTGTGGTTGGACCGATGGGTGAAATAATTG
CAACCACAGGGCATGAGGAAGACGTTATAATTGCTGATATTGATTATGCTGCAATTCAACGGGCAAGGG
>TCONS_00029474

CTCCATCCTTCGGAAGTGGAAGAAAGGGTACTCTAATTGAATACATAGTAGGGACCCGGAACGCTAAAAGGT
GGGGGAACTAGAGTTGTCACGGTAGAAAAGATAGATAAATAAATGACTATAAGAAACCAACGACTCTCTCT
TCTTAAACAACCTATATCCTCCACACTTAATCAGCATTTGATAGATTATCCAACCCCGAGCAATCTTAGTTATT
GGTGGAGGTTCGGTTCGTTAGCTAGTATTTGTTTAGTCATTCAGATAGTCACTGGCGTTTTTTTAGCTATGCATT
ACACCTCATGTGGATCTAGCTTTCAACAGCGTAGAACACATTATGAGAGATGTTGAAGGGGGCTGGTTGCTCT
GTTATATGCATGCTAATGGGGCAAGTATGTTTTTCATTGTGGTTCACCTTCATAATCTTCATGGTCTATATCATG
CCAGTTATAGCAGTCCTAGGGAATTTGTTCGCTGTCACAATCATTAATAGGAAGATTACAACTCCGAGACACC
GAACAAATTCCCTAGGACTGCTATAACTGGCATGATATAGACCA
>TCONS_00029555
AGGGATTGAAAGAGTCAAAATTCGCCCGCGAAAACTTTTTTATTGCAAAATTTAGGACAATACAACAAAGG
ACAAAATAAGGATTTGGTATAATAATACAATTTTTTTTTTAGATTTCTATTTATAAAACTAAAAAGTTTAGTT
ATCTATTCAAACAAGATATACAAATTACTAATAGATTGAATGAAATCTCAAAGAATCCCACGTTCAAGGTAT
TACTCAGTAAATACATATATATCTTAACTTAAGATTGACTATTCTAG
>TCONS_00029562
ACTTTGGGAGTTCCAACTATCCTGACTTAATTCTGTCTCAGTCACCGTGTGATCGGCACGAGTTCGATCCCAAC
CTGATCGAATAGGCCCAATCCACGAGGTGCCACTCGCTTCATGGTTCTCAGCGCTCATGTCTCATACCTTAGC
ATGGCTAAGTAAATTCTCAGCAACGAGACCCTCGGCTCGTGTGCTCCCTACTTTGGCACAAGTAGTTTCAGGA
AGTCAACGCCTTGGTCAGGACCCTAGGCCTGGACGATGACCCCT
>TCONS_00029643
AATATATTTTAGAGAGATCCTGTTAGTGGAAACAAAGCAGCACCAGATCGAGTTTGGGAGCTCCAACATACT
CGTAAGGATGACAAAGGAGAAACTGTGTGGTCAGATCCACAGTCGCAACAAATACATGGCCAACTCAATGA
AATTGTAACTCAACAACAATCTGAAGAAAATGAGCAGCCAATGAGTGCAGATGAGATTTTAGCCACTGTACT
TGGCGAACGAACTGGCTATGTTCGTGAAAAAGGGTATGGAAAGAAGCCTACCAAAAAAAA
>TCONS_00029687
TGTTGCCTCACGCGCCTCCGTGATGTCATTCGACTGGTTGTAGAAACCATACTCATGAGACCGAGCGAGGAA
GCCGTGAAGTGCAGATATATCATGGTGGGAATGAACAGTTGGAGTAGTGGTACCGTCTTGAGAAGAAGAAG
GAGCAGTGAAGGCAGAGGTAGCAAGTGGTATGCCGACAAACTGCTGCTGAGAAGAAGGCGGCGCGTGAGGT
ATGTTTAGTGAATTTCCAGGAGGATTGGAGTTGAAGAAAACGAGATTGCTTCCTCCTGCAGGTGGCTGATGAT
GTTGTGTCTCAGAGAATCCAACATAACCAGCAGGGTTCATCAGTATAAGACCATCAGCAGAAAACCATCTTC
TTCTGTGAATTGATGAGAACTATAAAAGTACTCAACTTTTTCAGAGAGAGAGA
>TCONS_00029792
ACTCGCTTTCGCTACGGCTCCGGTGGGTTCCCTTAACCAAGCCACTGCCTATGAGTCGCCGGCTCATTCTTCAA
CAGGCACGCGGTCAGAGCCCTGGCTCCTCCCACTGCTTGGGAGCTTACGGTTTCATGTTCTATTTCACTCCCCG
ATGGGGGTTCTTTTTGCCCTTCCCTCACGGTATATGGCTTTCAACCTAAATGGTTTCAATTTCAACCA
>TCONS_00029800
TCTAATCGCTATCGCTGTCGCTGCTGCTGCTGTCATGCCCATCGTGCTTCTTCTCCTTTTTGTCCTTCTTGTCCTT
TTTCTTCTTCTTCTTCTCCATGGATCTTTTCCTTTATCTTCTCAACAATCCCTTCCTTGTGCTCTCCGCTGCTATGG
CCGTGCTCCTTTTTGTGCTTTTCTTCTTCCTTGTGTTTATCGGCCTCATGGTGTTTGTCTTTCTCTCCTTCCTTGTG
GCCTCCAATGTGGAGTGTCTCCCCTATCTTGT
>TCONS_00029878
TCTCTGTCAAATTTTCCTTGTGACTTAGATGGAGCTTTAACGTTGGGCATACTAAGAACGGCACCATGCCCTCC
ATCTCCAGCACGAACTGTGATAACCACCTCATCAAAGTATTTGTGGGGTTCCCTTATGAATGCATCAGAAGCG

GGCGAAGGAGGCGTTCTGGCTTTTGCAAGTGCACACTTTACAGTATAGCTTCTATAATTTTCGTTATTGTTCCA
TTTTTTAGAAGCACTTTTTGGGAATTGTGGCAAGGGAGGGGAGAAGGTAGCAGAAAAGGAGAGCGA
>TCONS_00029941
ACATAGTAGAATCATCCTAGTAACTATAAGTTTCTTGTGTGTATCCATTTTTACCTGAACATTTTATGGCGAAA
TATGAAGCACATGCATATTGGAGGACATAATCAATCCGTCTCCAGATTCTCATTCCCTACTTTCAGTTCAATCA
AGGACGAGGGTAAAGCTAGGTAGCTTGGGGGAAAAAACCTGCATGGTTGCAAGCATGTTTTCTCTGTCATCC
TGAAATTCAATTCAATTAAAGTTATAACGGACGAATGTACTAAGCCCTAATTCAGAAGACAAGTTGGATAAG
TTTTAAGCTCCAAGTGGCTGCATTTGCCTAAAGGGGAAAGTCAGTCTTCCACGAGTCCTTGCATCATGCTGAC
CAGATTGTGCTTTACTTCAGTTGTTCAGGCC
>TCONS_00029980
AAAATGAAAAATAGAAGGCACTTCCATTCAAGTGTATCTAAAAACTCAAATCCAAGATTTTAAATTCTCGTA
AAATTCTAAACAGGTAACTAGTTTAGATGATTCCAATTTTGTTGGCAACGTCCAATGCATCATAGTCAGGAGT
CAACCTCACGTATGCTTTCTTAGTCCCATCAGGCCTAATCAAGGTATTGACTTTCTTTGTCTGGATATCATACA
TCTTCTTCACGGCATCCTTAATCTTCTTTTTATCAGCCTTGATGTCCACGATGAAAACAAGAGTGTTGTTGTCCT
CAATCTTCTTCATCGCAGACTCAGTGGTGAGGGGATACTTTAGAATCCCGTACTGATCAAGTTTGTTCCTTCCA
GGTGCACTGATACGAGGGTACTTGGGGTTTCTATCTTTCTTCAAAGTCTTGGGTCGGTGGAATGTAACTTTTGT
CCTTATCTTTTGTGACTTCTTCTTGAAGGTTGATCCTGACTTGACGGCCTTGGCAACCTTAGCTGCCTGTGCCTT
GGGGTCAGATTTTTTGGACGGATCAGCTTTAGCTGGAGCCATTTGGTTACGTACAGTCTGAAACCCTACGAGA
AATCAACCAGCTGAGAGAAGCTGCTCTGCC
>TCONS_00029999
GGCCTGGCACTTGCCCCGACGGCCGGGTGTAGGTCGCACGCTTAAGCGCCATCCATTTTAGGGGCTAGTTGAT
TCAGCAGGTGAGTTGTTACACACTCCTTAGCGGATTTCGACTTCCATGACCACCGTCCTGCTGTCTTAATCGAC
CAACACCCTTTGTGGGATCTAGGTTAGCGCACAGTTTGGCACCGTAACCCGACTTCCGGTTTATCCCGCATCG
CCAGTTCTGCTTACCAAAAATGGCCCACTTGGAGCTCTTGATTCCGTGGCGCGGCTCAACAAAGCAGCCGCG
CCGTCCTACCTATTTAAAGTTTGAGAATAGGTCGAGGGCGTTGCGCCCCCGATGCCTCTAATCATTGGCTTTAC
CCGATAGAACTCGCACGCGAGCTCCAGCTATCCTGAGGGAAACTTCGGAGGGAACCAGCTACTAGACGGTTC
GATTAGTCTTTCGCTCCTATACCCAAGTCAGACGAACGATTTGCACGTCAGTATCGCTGCGGGCCTCCACCAG
AGTTTCCTCTGGCTTCGCCCCGCTCAGGCATAGTTCACCATCTTTTGGGTCCCGACAGGTATGCTCACACTCGA
ACCCTTCCCAGAAGATCAAGGTCGGTCGGCGGTGCAC
>TCONS_00030000
GGCCTGGCACTTGCCCCGACGGCCGGGTGTAGGTCGCACGCTTAAGCGCCATCCATTTTAGGGGCTAGTTGAT
TCAGCAGGTGAGTTGTTACACACTCCTTAGCGGATTTCGACTTCCATGACCACCGTCCTGCTGTCTTAATCGAC
CAACACCCTTTGTGGGATCTAGGTTAGCGCGCAGTTTGGCACCGTAACCCGGCTTCCGGTTCATCCCGCATCG
CCAGTTCTGCTTACCAAAAATGGCCCACTTGGAGCTCT
>TCONS_00030001
CGTGCCCTCCTACTCATCGGGGCCTGGCACTTGCCCCGACGGCCGGGTGTAGGTCGCGCGCTTAAGCGCCATC
CATTTTCGGGGCTAGTTGATTCGGCAGGTGAGTTGTTACACACTCCTTAGCGGATTTCGACTTCCATGACCACC
GTCCTGCTGTCTTAATCGACCAACACCCTTTGTGGGATCTAGGTTAGCGCGCAGTTTGGCACCGTAACCCGGC
TTCCGGTTCATCCCGCATCGCCAGTTCTGCTTACCAAAAATGGCCCACTTGGAGCTCT
>TCONS_00030042
AAAAGAGCCTAGTATTTGGAACTTCTATAGCACCACTAAAAACTCTGTTGAGCCTAAAATGCAGATCCACTG
TTCTTCGTATTTCCAAGCAAGGTGTCTTTGGCTTCATTAATTTTAGAAGCAAGGTAGTGACTACCTCCTGCATC

TGGATGGTTAGCTACCATCACCTTCCTATGCGCTTCCCTCACTTTATCTGCTTGAACGCTTTCCCTGACTCCAAG
AATAAGAGCTGCTTCCCTCCTGTTCATTTTAGGCTGAAAACCTCCTTCATAAAACTTACGCATTCTTGCAGTCG
GAGGTCTGGCCTTGAATGCTTTCCATGCCTGGATTCCGTACCTTCCAGCATATGCAGCAGCGGCTATAGCCAG
TCCTGCTACCAATGGTGC
>TCONS_00030199
CCAACTCCCATCCCTTGTCTACTCGTCATGAATGTGATGTTCCTCCATTTAGCAACATAGTGGACTTAGATACT
CAATTCAACTTTGCCACAACCGTACTGCAACTCTAGATCCCTTCACGGCTTCAGCTCCATTGCCAACCTTCAA
GTCATAATACTTTAGACCATTAGGAAGGGTTGTGTATTCACTCTCTGGTATTTTTGCTCCCCTAAGAGCTCTTCT
GCTTGTGCTAGCAGCTTCAGCCACATCACAAAGACAAAAGGCAGCAG
>TCONS_00030200
TGTTCCACCACCAACTCCCATCCCTTGTCTACTCGTCATGAATGTGATGTTCCTCCATTTAGCAACATAGTGGA
CTGCAACTCTAGATCCCTTCACGGCTTCAGCTCCATTGCCAACCTTCAAGTCATAATACTTTAGACCATTAGG
AAGGGTTGTGTATTCACTCTCTGGTATTTTTGCTCCCCTAAGAGCTCTTCTGCTTGTGCTAGCAGCTTCAGCCAC
ATCACAAAGACAAAAGGCAGCAG
>TCONS_00030201
TATCCTTCAACAACTTTTACAGCAGTTCCAAATGGGCTTTGTTTAATAGAAAGCAGTTCAATATCAATCTCAAT
TGTTGCATTTGGAGGAATTTCCTGCACCCCCTTTTTCCCATAAGCTAGTTCTGGAGGAACTATGAGCAGGCGCT
GTCCTCCTACTCGCATCCCTTGAACACCGAGATCCAATCCTTTAAGTACTGTTCCTCTCTCTGATTGACCAACA
TCAAATCCATA
>TCONS_00030296
CACCACCGCAGCTCCGCCGTGTGAGTTTGAAACCCGGCCGGCAGGTAGCACGGAGCACAGCTGGTGCAAGG
CGGTTCCCGGTGGCACAGGGATAACAGTCTTAGCTCTCCTCTTATCAAAATCCCCTGACATTTCACTTCTCCAA
AATGCCCTCCACAAACTTCAAAATTCCAATCCTATCCTCAAATCCAAACTCCACTACGAACCCACAACTAAC
TCCTACTCTTACATCATTCCCTCCACTTCTCACCTTCAAATCCAGCCGTTTGATATCTCATCAACGGCTCAAATT
CTTCAACAGCT
>TCONS_00030333
AAAAAGAAAGAAAAGAATTCCTAGCATATTAGGCAGATACCAAGTAATGTGTTTTTATCTACAGTACTAAGT
CTTACATGAACCATACTACAATCAGCTTCCACCTGTATATTTCCTATATCAGTAAGCTAGAGGTGTTGTAACA
CACGTAGGTTGTAATTCTATCAAGGGCAACTAATACCCTCACCGAAGTATACTGCGAAGGTCAAAAACAATC
AAAAAGAGACTCATGAAATCACGGTGTTGAGTTCATTATGGAGGAGGCATCGAACTGAGAAACTACTCCTTC
ACTCAAGAGGCGCATTGCAGCCCCTCGTGAGAATTTCCTTGCAAACAAAAAACAGGGGACCAGAGTAGAAT
TATTCCGACACCACTCTGTTCTGTATTCAGTTTCATAGTAAACATTGCTGATATCCTTTATTTTCTTGATTTGTAC
AGGCCCAGCATCCGAATAGCTAAATGTAATAGGATGCCAACCCTTCTTCTCCATATTTGTCGCAGATTCATTC
CACTCTGTATAGGTTATCGTTCTACGTTCAAGTTCTCCTTCAAAACCATGCATCGCCAATAATGTCTGCACATA
GTGTTCATCTGGAATGCAGTTGTGCTGCTTTTGAAGTTTCATATTCTTTTTCCCCTTACTGGCTTCCAGCGGTGG
ACGTCTCTTGCAGAACATCTTGAAGACTCGAAGGACAGCATCATCATCTGCAACCACTTCCGCATGCTTCCTG
ATTAAAGTGATCCACTGTGACCCTTTCCGCCATTTGCTCATTGGTATATAGGGTGACATCCTTGGGTTGTAGCG
GGCTTCCTTTTTATCAAGAAAACTGTCAACAAAGCTCCTTGGAGAAGCCATCAAGTAGTTGTATATGTAGCTA
AAGTTGTACAGTGGAACACAGCTGTCCGACAGAAGAACAAATCTTTGATTAGCTGGATCATCAAGCGCTGCC
CCAAGTAATAGTTTCTCAGCTTGGATCATACTTGATTCTCCCCAGGCTTGATGCTATTAGTCAATTGGCGATTA
TAGAAGAAAGGCGATCTTGTAGTAGACTCATCAAACACAAAACCTGGCTCCGAATGTATATATATCGAGAAA
TTACCAGTCTCTGCATTCTCGAAGAAACTTCCCCAGAGGAAATCGAGAGGCAAATTACGACGGACTAGAAAG

AGGAAAGCAACTTTAGGATTGCCGCTATAATCAACAGCTCGAGATCTACGAGAAATAGAAGAAGTAGAAAT
AGAAGAAGAAAGTTCCGAATCGGAAAGAGAGTAGCTCTGGAGCTTCAAAAAAGCTAACACGCAAAGAGTA
AGAGACACTACAACCACTAGTTTCCACCACA
>TCONS_00030334
AAAAAGAAAGAAAAGAATTCCTAGCATATTAGGCAGATACCAAGTAATGTGTTTTTATCTACAGTACTAAGT
CTTACATGAACCATACTACAATCAGCTTCCACCTGTATATTTCCTATATCAGTAAGCTAGAGGTGTTGTAACA
CACGTAGGTTGTAATTCTATCAAGGGCAACTAATACCCTCACCGAAGTATACTGCGAAGGTCAAAAACAATC
AAAAAGAGACTCATGAAATCACGGTGTTGAGTTCATTATGGAGGAGGCATCGAACTGAGAAACTACTCCTTC
ACTCAAGAGGCGCATTGCAGCCCCTCGTGAGAATTTCCTTGCAAACAAAAAACAGGGGACCAGAGTAGAAT
TATTCCGACACCACTCTGTTCTGTATTCAGTTTCATAGTAAACATTGCTGATATCCTTTATTTTCTTGATTTGTAC
AGGCCCAGCATCCGAATAGCTAAATGTAATAGGATGCCAACCCTTCTTCTCCATATTTGTCGCAGATTCATTC
CACTCTGTATAGGTTATCGTTCTACGTTCAAGTTCTCCTTCAAAACCATGCATCGCCAATAATGTCTGCACATA
GTGTTCATCTGGAATGCAGTTGTGCTGCTTTTGAAGTTTCATATTCTTTTTCCCCTTACTGGCTTCCAGCGGTGG
ACGTCTCTTGCAGAACATCTTGAAGACTCGAAGGACAGCATCATCATCTGCAACCACTTCCGCATGCTTCCTG
ATTAAAGTGATCCACTGTGACCCTTTCCGCCATTTGCTCATTGGTATATAGGGTGACATCCTTGGGTTGTAGCG
GGCTTCCTTTTTATCAAGAAAACTGTCAACAAAGCTCCTTGGAGAAGCCATCAAGTAGTTGTATATGTAGCTA
AAGTTGTACAGTGGAACACAGCTGTCCGACAGAAGAACAAATCTTTGATTAGCTGGATCATCAAGCGCTGCC
CCAAGTAATAGTTTCTCAGCTTGGATCATACTTGATTCTCCCCAGGCTACCTTGATGCTATTAGTCAATTGGCG
ATTATAGAAGAAAGGCGATCTTGTAGTAGACTCATCAAACACAAAACCTGGCTCCGAATGTATATATATCGA
GAAATTACCAGTCTCTGCATTCTCGAAGAAACTTCCCCAGAGGAAATCGAGAGGCAAATTACGACGGACTAG
AAAGAGGAAAGCAACTTTAGGATTGCCGCTATAATCAACAGCTCGAGATCTACGAGAAATAGAAGAAGTAG
AAATAGAAGAAGAAAGTTCCGAATCGGAAAGAGAGTAGCTCTGGAGCTTCAAAAAAGCTAACACGCAAAG
AGTAAGAGACACTACAACCACTAGTTTCCACCACA
>TCONS_00030359
CTCGAGGTAGCTTTCATCATAAGGTGGTTCCTATTTGGATGTGAGATAAGCTTGTTTTTGGAATCCTCCCTTGA
AATGGCTATCTAGTCTCCATAATCTTCGAGGTTATTGACACGTTCACATTCATCTATCCATTCGCTATATATGT
CTATAGGCTCAGTCAAAGCTGTCACAGTGGTACTAAAGCTTTCCTGGCAAATCCTGCATATGGCCTTACCAAT
TAAGTTCTTCATATCAATGCGACATTCTACACTGCTTCCATGATTGCAAAAAGGACAACTGAAGACACTGTCA
AGTTTGTCCATTCTCTTCTTTGGTGCTGGCTTTGCTCTTGACTTCCTCTTGCCCATTTTTCAACCAAATATCACCC
GA
>TCONS_00030392
GAAACTGATGAAGATGAAGAATATGAAAGTGAGAGTGATTTTGATGATGAATATGGTGATGATAATGAAGAT
ACTGAGAATCAAGTTGTTCCTTTGTCGTCTTCTCCGTCAATTTCTTCTTCTTCTACCGGCAGTTATGGAGATTTA
TCTGCCGGAGACGGCGGTGACACGGCGGCTGTTTCTTCTCCGTGGAAGAAGCGATTAAGGGAAAGTGATGGC
CTCCATTCAGAGGATGAAGAGGCTTGCTCCTCGGATTTGAACTACAACAATTTATTGGAAGATAATAAGGGG
ACATCTTCTTCAATTGGATTCTTGAGACCA
>TCONS_00030426
GCAGCAAATTTTCAGGAAGTGTCTTGGAATCCTCAAGTTCGGAATTTATTAACCACCATTTACATTAAGATCA
GGCAACAGTGGCTTCCTCGATGCTGCCTATATCCTCAACCAACTCTACTTTTGGTTCCAAGTCTATGAATTGAT
TAGACTTGCTAGCTAGGTGTGAGATACTGACCCTACCTTCACGTCTAATATAGTCAGCCACGGCTTTCATTTCT
TCTAATGAGATATATATATATTTCCCTCTGTCATCCATGACACCAGAGAGTCGCCCCATTTCTTCCAGAGAGTT
GATCCGATTGATACACTCCTGAGTTCTTAGCTTGAATTCTGCAGCAAGATCCTCCAATGGCACACATTTATGTT

TCTTAATGTATTCGACAAAATCAAAAAGCAGACCCTGGCTTCCATCTTGTATCTCATTTTCAGTTGTTCCTTCA
GCATCAACTGAAATTTCACCTTTCCATTTCTCAAACTCTAAAGCAGCAGCTTCTTCCTCTTTGGCCTGTCGAGC
CTTGGCTTCTTCTTCCAGTGCACGCTCTTTAGCTTCACGCTCCTCCTCCTTTCTCCTTCTGATTTCGTCATAGCGA
CTTTGCTTTGTTTGTTTCGATTCCCGAGAAGCTTCTTCAGCCTGTCTTTGTGCTTCCCGCTCTTGACGTTTCTTTTC
CTTTTTCTTTGATGATTTTGCAGTATAATATCCATCTCCAGCAGCTTCATCATCACTGTCATCACCCAGTTCATC
TATAGTGGCTGCAGCCGAAGATGTGCTAGCAGCTGAAGAAGCTGGTCTTCGACGCATTCGAC
>TCONS_00030432
AGACACAGACACTACCAGCACCACTGACAGACACATCAGAAACCACCACGGAGACGGAGGACAAGGTGCA
GAGTTGACTCCTTCTGAATATTATAATCAGCCAGAGTCCTTCCATCTTCCAATTGCTTCCCAGCAAAAATCAG
CCTCTGCTGATCCGGCGGGATACCCTCCTTGTCCTGAATCTTAGCCTTCACATTGTCGATCGTATCAGAACTCT
CCACTTCAAGGGTGATTGTTTTCCCGGTCAACGTCTTCACAAAAATCTGCATCCCACCACGAAGTCGAAGGAC
CAAGTGAAGGGTAGACTCCTTCTGGATGTTG
>TCONS_00030529
AATTTTGCCACTATACAAGCATAAGCTACTGCATTACAAACAAGGAAAGAGAAATCAACAAGAAGACTCTTT
TCTCTTTCAACCAAAGAACTTAGGATCATATCCATTACTAGAAGTGGCAAGAATATAATAAGCAACAATATG
ACCAATAGAGCCCCAAGCAAGAACATCAACAATGTTGAAACCAACAGGGTCATTAGATTTTAGCAAACTAA
CATATTCTTTGGCTCTAGAATCACCAGCTTCAAAATGGCTCACTCCATTTTGCTCAGGCAACCCTTGTTTGGCC
ACATTCTCTCTCTGAAAATTGAAGAAAACAAATCTTCCCAAGAATAGTGATAGTCCTGTGCTCAAGCTAATAA
CAAGTGATGTGTTGAGCTCAGCTTTAACACCAAGATTGCATCTTTTCTTCATGGGTTTGTAGGAATTGGCCTTA
TTGTTGAGAAAAAGACAAGTGTCAGTTGGTTTGTTGAGGGGTCTAAGACCTTGGAATGTTGGTGCAGAGAAC
AAAGCAGAAGCCATGATT
>TCONS_00030530
CCACTATACAAGCATAAGCTACTGCATTACAAACAAGGAAAGAGAAATCAACAAGAAGACTCTTTTCTCTTT
CAACCAAAGAACTTAGGATCATATCCATTACTAGAAGTGGCAAGAATATAATAAGCAACAATATGACCAAT
AGAGCCCCAAGCAAGAACATCAACAATGTTGAAACCAACAGGGTCATTAGATTTTAGCAAACTAACATATTC
TTTGGCTCTAGAATCACCAGCTTCAAAATGGCTCACTCCATTTTGCTCAGGCAACCCTTGTTTGGCCACATTCT
CTCTCTGAAAATTGAAGAAAACAAATCTTCCCAAGAATAGTGATAGTCCTGTGCTCAAGCTAATAACAAGTG
ATGTGTTGAGCTCAGCTTTAACACCAAGATTGCATCTTTTCTTCATGGGTTTGTAGGAATTGGCCTTATTGTTGA
GAATAACACAAGAATCAGTTGGTTTGTTAAGAGGTCTTAGACCTTGGAATGTTGGTGCAGAGAACAAAGCAG
AAGCCATGATT
>TCONS_00030558
AGTCTTCCTTCGCCTTTCGTTGTGTCCAGCCAAACGCCGCCTGCAACTCCTTTTCGATTCATCAAACTCTCCCA
CCTCGTGAAATCGGCTGCATTGTTGACAAAACCGTTGCCGGAGTCCGGCGACGACGACGGCTTGAGACTTTG
CATGGTATTCACACACTTTATGCCTCTTATGGTACTGTTTGGCATCACTTAAATCAACAGTACATTTATCAGCT
TGACAACACCTCATTGAAGTACTTGACCCTTTGATCTTTTCATTGTTATTAT
>TCONS_00030598
AAGAAATTAATCATGTGTTGTTGTGGAGATGATTGTCAATGTCGTCCTTTAGGTTTTCTATTGGGTCTTCCTTTT
GCCTTTGTTGCCCTTCTCCTCTCCATCGTTGGTGTTGTCATCTGGATTGTTGGAATCATATTGAGTTGCATATGT
CCATGCTGTATTTGCGTAACAGTATTGGTAGAGTTGGCTTTGGGATTAATCAAAGCACCCTTTTCGGTTATGAA
GTGGTTTACTGAGCAAATTCCTTGTTAGAATACACCTTGTATAAATTCATCTCCCTAATTTCTAGTAATTCTTTT
ATGTAAATTTATTTCTGTGGTGAGAGAATAAATTAGATTTATTTTATCTTGTTAAATTTGTCTCCTTTGCCTACA
AAAACTATTCCTGAAATTAATTGGTATTTTGTTTTCCATATTCGGTAGAATAGTTGAG

>TCONS_00030667
CTACCGAGTACAGACGAGCAAAGTAACGGACACACCAAAAAAAGAAGGAAACCCGGAAAAACAGACCTTG
CCTCCTATTCAGTTGGCATTTTCGTTTTACAAAAACCCTAAACAATTTTGAAATAGCAAAGTGAGAAAGTTTA
TCATCTTCTCCAACCATGGCAGAAGCAAATTCTGTGACTCAAAGCTCTGAACAGCCCAACAAATTGCCTGAA
GCTGTGGCAAATCAAAACTCGCTGCCAAACATTCCTGAAAATGAAGCTGAAAAAAGTGGCCCTCAAAGATG
GTCGGGGTTTAGTATTTTTAATCTGCG
>TCONS_00030686
CGTCTACAAAGTCCGGTGAGTGCTGTTGATTACTATTCATCTAAAGTAATCTGCCTTACAGACTATTATGATGG
CAGATGTGCATGTTTCTGATAGTGCTGGACCCGCTGAAGTAATTTCGAACCCACAATCACGTGAGTTGATTCA
TTCAGTAAAAAGTAGCTTTGTGAAATCTCATGATCAAATTTGGTAATAAATTATCATAAAGAGTAGGATTTGA
TTATTATGCTTAAGGTCGATAAATCTATTTCTTTGCAATAGTTATT
>TCONS_00030707
GAACAAAAACCTTTTGGTTCATGAGAAAGAGCATATCAAAGTGATATTCCAATTTCTCCTTCCAATGAGCAA
GTTATGCAGTCCACCCAAAAAGTTGAAACAAGTACTCTAGGGAAAGGACGAGGGCGAGAGCTTAGATCTAT
GTGCTCCTTTGATGAGTCTGAAAATGAGAAACGATTCTTCCATCAGAATGGTCATTATGCTA
>TCONS_00030900
AGTAGCTTAGGCTTGATTTTAGTCACTATTCCATGTCTTGTTGCAAACGAACTTCGTACCACTGAATGGAACCC
ACATCTCCATCAATTTCAAGCTCACAAACAGATTTTACTAGGTATTGAACTGCATCATCAATTTCTTCAAATCT
TGCAAGATCCTTTGGTAACTCATTGCCTGGCCAATTTTCTAGCCAACCTTTTAACCTCTCCCTTAATTCTTCCTC
ACTTACAAACTCCTCGGACTTACCAGGTTCCATTAGCACATAAGTCTCTGTCTGTATGTTTGCTCTTCTCCTTCT
TGTAGCCAATGTCAATACAATTGTTCGTTTTATTGCACCACAAATGTGTCTCCATTGGAAAAGAAGCTTCTGCT
TTTTCTACCGCAGTGAGTTGCAGCAATGATGTATAATAGACAAATTAAACAACATGAGTAGTTAAGCACATA
TCAAAAATAGTTGCATTTCTTAGACTTTGGCGATGAGTATGGTGGTGGATAAGATGATGGTTTTCAGCCTCATC
TATATTCATACTGTTCCTGT
>TCONS_00031077
CCCACAAATCATAAACAAGATGCAACTTTGGAGCATCAAGATCGGCACCTCTAAGCATAGACACAATTGGTT
CGGTAAACTTTAAAAAGTACTCAATCTTATCCCACCATTCGTCATTCATTACAAGGGATTTAATCTCATGCGTT
TTATCTTCAATCACTTTGTCTCCCTTATAGTTTCTCCAATCATCATCCATTACCATTCTTTCCAAAGATGCTTTCA
CTTGATGAACACAACTAGTCATGACAACATGAGATGCAAATCTTGTTTCGGCAACTTTCAACAAAGACAACT
ACGAATGCTTTTGAAAAAGAGCATGAGCCATATCATGATTCAACACAAAAATTTTCAAGCTACTAACTTCAC
CAATCAAATCTAAAATCCATTTGCAATTTATAAATTGAGTTGATTTTTCGGAAGGTTGGCACATATTTTTAGGG
CAAGGTTCAAACAATGAACAACACATGGTGTCAAAAATATATGTGGATAAGTTTGCTCAACCGTAGAACCGC
AAGCTTCATATTACTTGCATTATCGGTGATAACTTGAACAACATTACTTGCACCAACTTCCTCAATTGATTTAA
TGAACAAATTAGCTATATATTCTCCACCCTTCACAATACTACTTGAATTGATTGATTTTAAAAATATTGGACCA
CCACTAGAAGCCGCCATTATGTTTATCAATGGTCGCCTCTTAATATCCGACCATCCATCGGAACAAATAGACA
ATCCTTTTCTCTTCCATGCATCCTTAATAGGTTGCAATTTTCTATCAATATGAGCTTTTTCTTGAGCTAAAAGGG
TAGTTCTTAACCTATTGTACGTTGGCGGAACATAACCCGGTATAGAAGTCTTTGGCAAAAACTCGGAATACTT
TCTAAAATAAGGAGACTTGGCAAAATTAAAAGATAAACCTAAAGCGTAGAACATGCGGGCTACCATTTTGTC
GGCGATATTCCTATTCTCAATGCCGAACGACTTCTCTAGAGCTCCAACGTTTGAACCTTTTCTTTTTTTACAATG
CGATAAATCTAACCCTTCAGGAAGCGATACGTAATCTGACTTTTTCCTCGCGTCAAGTTGTACAGATGCTTTTT
TATTTTCAGCTTCTTCATGTTCTTGCTTCAAAGCTTCACAAACTTCTCTACTTATGTCTTTACATATTTG
>TCONS_00031078

ATCTGACCCTTCAGGAAGCGATACGTAATCTGACTTTTCCTCGCGTCAAGTTGTACAGATGCTTTTTATTTTC
AGCTTCTTCATGTTCTTGCTTCAAAGCTTCACAAACTTCTCTGCTTATGTCTTTACATATTTGAACACCTTGACC
AGAAATTTTTAACAAATGAGCTTTGACTTTAGAATATGATCCCGTAATTTTTTGTTACAATAGTTACATGACCA
TACTCTATTTCCACCACCACTTGGAGTAAGTGAAAGAACTTTGACATGGTTC
>TCONS_00031116
ATAGTAGTGTGCCATAATTAACTGTAACAAAGAAAAATCAACACGATCCTCAAATAGTTGTGCGATATTAAA
CTCCATCTGCTCCTTTCTTGACAACTAATTTTCCTCAACCCACTAGAGAAGAAAACGGAATCTATTCTACTTCT
TGTAGGTAATCATCTATTTCAGTTGGTGTGAGAATTTTGAATACTTTGTCATTGCCAATGATGCCAATTTCAAT
GTTTTTTCCGGAGATCTGCCCCTCAAATCCCTCCTTCAGGGTCAATATAGCAGTGTGTACAGCATCATCAAGTT
CAATATCCTCAGTGTACCTCTTCTCGAGAAATGTCTTTGCATTTGACACGTTCTTTCCCATCGCTGAAGCCTTCC
AAGAGAAATATGAGCCTGATGGATCCACCTGATATAGTTGAGGACCTTTGTCATCATACCCCGCAACCAAGA
GAGAAACACCAAATGGCCTTACACCACCTGATTGGGTGAATTCCTGCATGACAGCAGCAGTTTCCCTCACCA
GTTGTGTGACAGGGATTGGTTCTTTATAGAGTCGATAATATTGCTCAGCCTGCTTTCTACTTTTTCGCACCAAA
ACTCGAGAATCAGGGCCCATGCCGCTGTACACTACTCCAATATTTGGTGTTAAAACTTGAATCTTTTGCACAG
ATGCTTCATCAACCAAGATGGATGGCAATTTCTTCTCAGTTGCAATTACAACACCATTAGCAGCTTTAATTCCC
AGTGATGTTTGGCCCGATCCAACCGCCGTCAAGGCATGTTCAATCTGAACCAGCTTTCCAGATGGACTGAAA
GTGGTAAGTGAGAAAGAGTACTGACTATCACCCATTTGAATTAAATGCTAATCTTCAATAGAAACCCGGATT
CCGGTAGTAAACTTAACGGAGGCGACGAGCAGCGCCGGCGAACGAGTAGAAAATAGGGAAGTGCGCGAGT
ATTGGAGCGTTAAGTAGGCTTTGGGATTCAAT
>TCONS_00031153
TGGTCCACTTTGATGACGTTCTCCAATTTGATGAGGACGTGTCATGTTCCAACGATGACGTGTATTTGAAATTC
GGGTTTGACCCATATAAAGCTTGGACCCCTTCCACGTCATCAAGCTTTAATTCCCTCTTCTTCGTTCTTGGGCTT
AAACTTGGGTACATAACAGCATCTTTAACCGAAGAATGAGCCAGCCCAAGTACATGCCCAATCTCATGGGTC
GCCACTGATTCCAAATCAACGGCAACTCTCGACCTCTCTTCATCAAAATCAACGGCCCACGTTTCCGCTGCGT
CTAAATGAAACCTCCCATTTTCCGGCGAAAAAGCGTGAGCTAACACTCCCAACACTCCGTCAAACGGCTCTC
CGTCGCCGTGATCGCCTCTAAAAAACCCGATTTTAATATCCGCCGTGTAGTAATCCTCCGCCTCCGTGAAATT
CACCGGAATCGCTGACGACCACCGCGAAAACGCACTTTTAAAAGCCGATTTAATCTCCGATATGTCTATATA
ATCAATCATGTAATTTTCAGAGAAAGCGTACGATAAAATCATCGGCGATGATTTCATCCACCTAGGTCTCCCG
TAAAAATACGCGTAATTTCTCGTTGTATGTAACGAGCTGTGATCATGTTCATGAGCTGAATCACTCATCCCAC
AACGAGACTCTAAATCTTCGTCGAAAAAATCGGTGAAATTTTGATCGGGAATATTAGATAAATAGCCAAACC
GTTGGAAGTATTTTTTCAGTTCCGACATGCCGGTGAGTTCACTTCCTTTTCCGGCGTCGAGGAATTTAACGAAA
GCGTGCCACGTGTTGTTTTGAATATCGGCGGTTAATTCAGTTAAAGGATCGTGTTTTAGGATTCTGGCGGGAA
AAGAAGGAAGGGAAAGGAGGAGAATGAATGGAGCAAAAGTGTAATAATAACTTGGAAACGGAGACATGG
AGTTAGTTTTTTCTGTTGGATTTACAAAGAGATT
>TCONS_00031276
CTCATCAAATTCAACAACAACGGCCTCTGACTGATGTAAGCATATGCCTTGACGGCCTTACTGAATACTGAAG
CATATGGGTTTTCCTTGAATATGTCACCAAACATCAAGTTGATACAATGGGCAGCACATGGAGTCCAATAAAT
GTTTGGATAGATAGCTTCCATCATCTTCCCCGCACTAACATTCTCACTAGCATTATCTGTAACAATTTGTACAA
CATTTTCCTTTCCAATTTTATCAATAGTATTTCTAAACAAGCTGTACATTTTGCTTCCATCCGTAGAAGAGTTGC
TAGCATCGTGAGATTCAAGAAAAACACTCCCTCTTGGAGAGTTCACCAACACTTTTATGATCATTTTTCCATTC
CGTGCTG
>TCONS_00031283

ATCTCGGAAGCTGCATTAAACCCAGTTCCTAAAACCCCAGAACCTCTCGTACAGAGAAAAGCAAGTTTGATC
TAATACTGTTCGATAAAAATGCAGAAGAAGAGCTTCACTCTCCTTCAGACAATTGCAATTTCAGGTGTATTCT
CTGCCGTTTCTGGCTGAGAGTCAGCTCGGAAGGAGCTAGGAGGTTTGATTGAAAGTCTCCGCAATCCCAATTC
TGACCCCGTTTCACCTCCCCCCTCTCACTCCTAATTCTAGCAGGTATTCTACACTTTTCTTCTCTATAAC
>TCONS_00031284
ATCTCGGAAGCTGCATTAAACCCAGTTCCTAAAACCCCAGAACCTCTCGTACAGAGAAAAGCAAGTTTGATC
TAATACTGTTCGATAAAAATGCAGAAGAAGAGCTTCACTCTCCTTCAGACAATTGCAATTTCAGGTGTATTCT
CTGCCGTTTCTGGCTGGTATGGGTTCATGTTCGGGAGAGAGTCAGCTCGGAAGGAGCTAGGAGGTTTGATTGA
AAGTCTCCGCAATCCCAATTCTGACCCCGTTTCACCTCCCCCCTCTCACTCCTAATTCTAGCAGGTATTCTACA
CTTTTCTTCTCTATAAC
>TCONS_00031285
GGTAACATATCACAACCTAAGGAAATATACTCTGGATTTCTCCTGCCTGATCTATCTGTGTAGACAAGATCTG
GGTTCTTCCCGATATCTTCAAGTACCCATGGAGGTAGAGGAATACTGCAAGAGTCTCCAACATTTCCTCCACA
CTGATGAAAAGACATAACAACTTGAAGTTTCAAGCCATGTTCTTTGCACATCTTAACAAGTTCAACATAACCT
TCCCAATTATATTTCAAAGGTCCATCTTTTTCAACCAAACCCCACCAAGCATCTACCATTACTCCTTCAATTCC
AGCAGTTTTCAAGGCCATTAAACTCACGTGCATCGCTCGTGGCTTGTTCAAGTTCCCTCCCATAGTCATAGT
>TCONS_00031287
TTGAGTCACACTCTGGACGTCTAGCATTTCGGCCTCCTTCTGACATGCTTTTCACGAATTGCCCTAAATTCTGC
CAATTTTCTGGCTCAAACAACCGTTTGTTCATTCTCAGGAATGTAAATGCGCTCAATGCATTTCCAGAATCTGA
CCTGCTTGTTGCCAAAACTTGAGAATATGCTCCACCATCATACCTCTCTAGAGCATTTTCTCCAGCAAGTTCTA
CTTCAGAAGTTCTAGTTGCTTTTTTTACTTGTCGAACTAAGCCTTCTGGTGAGCAATTTGCACTCTGGGGCTGTT
CACCATCCCTCATTTCCATACATGTAAAGTTAAATACAACACGATGTTTCCCGAACATACGTGCGATAGGTAG
ATAGCCATTTGTGTGTCTTGTATTATAGTATC
>TCONS_00031288
TCTCGTATTATAGTATCCTGCTGTTAACTCTGCAGCATGTGATCTAGTATTGTAATGCCAATGAATCCCAGCTA
CCTTTCCAGATAGTTTAGCCCCAGTCCCTTGATATATACCCTCTGCTGCTGCTAGGATTCTGTCGCCGTGCTCC
ACTAGCTTTCCTGAATACCACTCCAGGAAGAACTGTCCATATTCACTGTTCCATGTTCCATCTCTTTGAAAAAA
TCCAGTATCCTCAGGAAATTGGTTGTACTGTCCGGAGTCATGAGGCCCTCCCTGGCCCCAGTCATCCTTTCCG
GCCGCCTTGGCGGCTGCTGCTAGTGAAGCTCTCATCTACTTGTCATAGCACTGGAATTCTCCAATTCCAGGAA
ACCTCCATGTACCATTGCTTTCCGGATAAGATGGATATCTTAGCTCCCCACAAGGACCCATTCCCACTTGAAT
TTCCT
>TCONS_00031289
TCTTCAAGTACCCATGGAGGTAGAGGAATACTGCAAGAGTCTCCAACATTTCCTCCACACTGATGAAAAGAC
ATAACAACTTGAAGTTTCAAGCCATGTTCTTTGCACATCTTAACAAGTTCAACATAACCTTCCCAATTATATTT
CAAAGGTCCATCTTTTTCAACCAAACCCCACCAAGCATCTACCATTACTCCTTCAATTCCAGCAGTTTTCAAG
GCCATTAAACTCACGTGCATCGCTCGTGGCTTGTTCAAGTTCCCTCCCATAGTCATAGT
>TCONS_00031429
TTCTCGAAGCTTCTTGTTCGTATCTCTCATCAAAAGGTGCTATTCGATAATGTCTGTCTAGCAGACTCCCTGCT
AACCCTAGTGAACATTCAAATATCTGTCCTACATTCATTCTTGAAGGTACTCCTAATGGGTTAAAGACCATAT
CAACGGATCTTCCATCTTGTAAATAAGGCATATCTTGTCTAGGCAAAATTTTGGAAATGATACCTTTATTTCCG
TGTCTTCCAGCTACTTTATCGCCTACTTTGATTTCACGTTTCTGTAAAATATATACACGAATCGTTTCGGGATTA

TAACTAGAACCACCCCTTTTCTGGATCCACCTCACATCAATAACCCGACCCCTGCCACCTATAGGTAGTTTTA
GACAAGTTTCTTTTGAAGTAGATACCTGAATACCAAGTATAGCTCGTAACAATCTAT
>TCONS_00031456
AAGTCGTTTTTGTTCCATCCTTCAACTTCTTCGTGTGGGTTTCCTCAAATACCTCATCATGAGTAACTGGTCTTC
CTTTAGCATTTTCCAATCTTCTTCGATGAGCCGCAACACTAATTGAGCCGCCCGTGTGCACCGAGCCACCCTT
GTTGGACAAACGGGCTGCCTTTCCTTGTTCGCTCTTCTTCTTAAATTCCGGAGAATTCCAATACTCAAGAAGTT
TAACCCATAAATCATCAAGAATCCAACTTGGCCTCGTATTACTTTCTCAAGCAGTCCGAAGCAGATCAGGCA
ACCTATCAGAACATTTCTTAAAG
>TCONS_00031457
CAGCACGCGGCTCGATTCAAGTCGTTTTTGTTCCATCCTTCAACTTCTTCGTGTGGGTTTCCTCAAATACCTCAT
CATGAGTAACTGGTCTTCCAATCTTCTTCGATGAGCCGCAACACTAATTGAGCCGCCCGTGTGCACCGAGCCA
CCCTTGTTGGACAAACGGGCTGCCTTTCCTTGTTCGCTCTTCTTCTTAAATTCCGGAGAATTCCAATACTCAAG
AAGTTTAACCCATAAATCATCAAGAATCCAACTTGGCCTCGTATTACTTTCTCAAGCAGTCCGAAGCAGATCA
GGCAACCTATCAGAACATTTCTTAAAG
>TCONS_00031472
TTCTTACTTGCCTAAAATTTATTAAAAGGGACAAAAGTCTCTCTTCTCATCTGAACACAACCTTCCCTCAACCC
TCCTCTCCTAAGATAACTTGTTAAAAGTTCCAGTTTATGCAGGGGGAAACCCCCACGGAGGAATAACATATCT
TGACTACAATCAACATTTACAATTGAAGAAAAGGCAAGAAAATCGAAAGAAGGTGATCTTAAAGCAATACA
GTCATATTTAAGAAGATTTACTCAATATGCACTAAATTGGATATTAAGTTCAAAGTCATCAAAAGAAATCAA
ACACTTGTGTCCTGATCGAACGATCCTCCAATCATCAAACCACAGATGCTCTACTTTGATTCCATTGAAAGTG
GCACGGCAGTATAGATGTGAGGTGAGTTGGTTTCTTCAGATTCCACGCGCAACCAGTCCTGACCACAAACCA
AGGCTTCTCTAGCAGCTGCATTCAGTGAACTGCGATCTTGGTCAAGCACCTTGCCTCTGTTATTGAATGCCACC
TCTGGCCCAAGAGTGGATGCAGGAACTCCCAAGATATCACGTGCCATCATTGATAATGTAGGGTACCTTGGT
GTGTGAACTTTCCACCAATTCAATATGCTGAAATCATAATTGCGTGGAAAGACTGGCTCTTCCAAGTATTTCTC
AAGATCTGACGTTATGTTGTGGTTTTGTGAAGTTTCATGGAGAAATTTGTCAAAACCCCTAAGTCTGTCCCTTG
TACCACTAGAGATACTGGTTGAGCTGCCAGAAGTACCAGCTGTATCTGGATCAAGCGAAGATGAGCCCATCG
CATACTCATTTGAAAGCTCCCTTATAGCATCAGATATTTCTTTTATCTGATTTGAGGCATCAGAACCATAAATC
TGAGGATAGTAATACTCCACCAACTTCATCTTGAACCTGGGGTCCAATATTGCTGCTACCGCCAAAGTCAAAC
TGCACTTACTCCAATACTTATCAAACTTTTCTTTCATCTTCAGCGCTATATCGCTAAGAAAATTATCAGGATTTT
TACACCAATCAATCAACTGGATGTGAATATCACATATTTCAGGGAAATATAGATTCGCAGTTGAACATTTATT
TGCCGTGAAAACATTAGTAACTTCAACAAAAAGTTTTATATAGCCCGCAATAGCACTAGCATGATCCCACTCT
GCTTCAGACAAAGCGAAAGTGTAGGAAGGGTCGTGCTCTTCTAGTAGACAGAAGGCTCCCCTATGATCTAAG
GCAGCTTCAAGCATCAAGTATGTTGAACTCCACTGCTGTCCACAGTCAAGAATCAAAGGCCTTTCACCACTA
ATCGCAACTTGCTGAGCAATCTCATTGAACTTTCCTAGGGCTAATTGTGAACTCTTAACATGCCTTATGCTTTC
TCGAACCTTGTGGATCACATCTCGAAGTGCTTCCATGACATCGAAAACGATTGATTTCAGTAGCTGCACTGCA
CAACGCATGTCAAACAATTCACCATTCTTTAACAGCGGCCTGTTTTGAGAAAGCCAGTCCTTGATTCTAATAA
TCATCTCATCATAGCCTGTACAATGATCGAAAGTCATAGAAAACAACTTACGATCAATGGCCCAGTCTGTCA
AACTTTTTATAACAACTTCTGAAAGTATGTCATCTGTGTGAGAAGGATCCAGTGTTATGAAATTCAGCATTTTC
TTCTGCAGTTTCCAGTCTTCATCAATATAAAAAGCAGTTAAACACAGATACCGTGCATTTTCAGATGAAGTCC
AGACATCAGCAGAGAGAGTAATTCTTCCATGCAAGTTATGGATCGCCTCATACACCTTCTGTTTCTCTTTTGCA
TAAATTGTCATACAGTCAAGTTCAACAGCACTGTTAGTCAAAGTCTCAAATAGTGGCTGTAAGTTCTTGACAA
ATATTTTGAATCCAACATGGTCAACCATGGCTAAAGGATAACCATGTAACATGATCATACGGGCAAGATCCA

GACGGCTACGCTCTTGATCAAATCTAACACTGCCAAGGTTGATAGGCATAGTAGCTTCCTCTTTCTTTACTTCT
TGATCAAACTTAAAGGTCACAGGGTTAACGGTTTCTTCTTTCCTTTGCCCTTCTTCATAAGTGACAACTGCAAC
AGTAGTATCTTTTCTCTTCCTCTTTGCTGCAAGTATTTGGGAAACATCATAGTTGGATCTTTTTAGACACCGCAA
CAAATGATTCCTTAGATGAGTTGTTCCACTGTTACTCGATCCACTAAGCTTCTTTTTACAATGTACACAAACAG
CATAACAGATATCTGCCTTTCTAACTCTTTCAAAATGATTCCACACAACGGATGTCAACCTCTTTGGTTTCTTT
GCTGGGGTCTCAACTGGAATTTCCATGGCATGGATGAAGCTAAATTAACCAATCTGTTTTCACTGAAATGGTT
TGGCCTAAATGCTAGAAAGTGAAGAGCTTTTCGTTGACGTTGTTCAGAGAGAGATCCGATCAGAGGATCCGA
GAGAAAATAGGGTTAGGGTTTGGATTGGATTAGAACGGAGAAGAGAATATGAACCGGATGTTGAGACCGGG
AACCGGGAAGATCCGGTAAGAATGGCCCGATTGCGTGATTTGGGATTTGGGTGGTTCACACATTTGTGAAGTC
CGTTTTGAG
>TCONS_00031484
CATAATCCTCTGCCACAAAGTAACAAGCTGGCTTGCAACATTCACTGTAGAAATCGCCATGGTTTACCCCATT
GGGAGCACAAGCAGGGAAGAGAGGGTTCTTTTTAACTTCTCCCAAACCCGAGCCAATTACCATCACTGCCAT
ATCAAGATTGAAGGAATGAGGAGTATAGGTGAGAACTGATGGTGGAGTTTGTTTTCCTTTGTCCATGCCATCA
ACAGGATCAACACCGATCAATACTGAAAATTTCGGGTTAGTAGAAATATTAGCAATTTTTCCAAGAGCTAGC
GCGAATGCAACTTTTCCTCCACGACTATGGCCTGCTAGTCCAAGTTTTTTCAAGTTTGGCTCAACATCGGATGG
AAGGTAATGGTGCAATCCTTCAGCTAACCAATTTGTGATTTCAGCAGTTGATTTGATATCCTCAGTTGCATCTG
CTCCTTCCACCAAATATAACTGAGGAGCCACAACAATGAAGCCATGAGAAGAGACATGTTGAATAAGCTGA
GTATAGAAAGAATTGTAAAGAAGATAGCCATGAAGAAATATCAGTA
>TCONS_00031533
CTTGGCATTAGCCTGCATAATCACTTTACGAGGCGAATGGCCTCTTTAAGATCATAAACAGCCTTTTTCTTCTT
CTTTTCATCATCACTCTTCACGTTAACCAATGTGGGAAACGGAACCATTTCCTCTTCAACATTCAAAACTCTCC
TCATTGCCGCCCTTTTTTGCGCCTCAATTCTTCTCCTCTCTCTCTCCAGCTCCTCATCACCCTTAGCTTCCACTCC
CCCCTCCTCTTCAACCCGATCCTCAGGTAACGGAGCTACTCGGGTCGGGTAAGAAACCGGAGTTATTTTTGGT
GCATCCTTTAAGTATCGGAGATCCTCCCGTGTCCAAGTTTTACCATCCGCATTAACTGATTCTCGAGTATTTGA
TTCGGGTCGGGTACCATTTTGTGTTTGTGATTGTGGGGAAGCTTGCGATGTGGTTTCTGTAGATGGGGTTTCCG
GAGGATCCTTGGGTTTGTAAGAAACGGGTTGGACTAGACTCGGGTTTTCTGGGGATTCGGGTTGTGATTGAGA
TGATGAAGAGGAGAGAAATCGGTGGAAATGAGAGCGAAGAAGAGGAGAAAAGTGAGATGGAGTTTTGTAG
GAGCAGTGGCGGCGAGCTTGAT
>TCONS_00031593
CCAGGTTCAAGTTTCTCTAATATGACAGCTATTGATAAATTTATCAGAGAAGTTGACATACTTTGTCCACAAG
GGTCGAAGTTGAGGGAATGCTATATCTAGCCAAGGTTTTGCTCGACCGTTGAAGTGGATCACACCAGCACTTT
GAGCGTCCGCAATACTCGTATTATCTTGGTACCCGAGTCCTAGCATGTGCCAGAAGGATTCAATGGCATGGAC
ATGACCGTGAAATGCAATTAGACCTGGAGGTGTCAAAGGATGTAATGAAAACCAAGCTTGCATCGGTGAGA
AGGTTTTGCTATCCGTGATTATGTGAAGGACAACTCTTTCAGGGCGGAAAGAGTTCTGGACGAGCGATGATGC
AACAACAGAAGCAGCAAGAATATTGTCAGAGGCAAGGACAAAATGGAAATATGAATTATCAACCAGGGCA
GGAACAAGTTCCGCTGATGGTAATTGGAGACGGGCATTGGCGTTGATAGAATACTCATGCGCTAGCTTGAGA
TCAAGACAGTGCAGCTGTTTCGGGATGCCACTAGATGCCACATGCCGATAAAGGTACTCTTGAATTTTGGCAG
TTCTTGTTGTTTGTTCAAGGAGAGTTACCTGCAATAAATAGGCACTAATTTTTGAAGACATAATTCAGTAAAA
GTGTACTACTCTCTCTAATAAGTTAATCTTTTAGATGAGACAGTAATGATTCTACATGGTATCAGCTTAAGCAG
AGGTAGGTCCTGAGTTCGAGTCTCATTGTCATCCATTATCAA
>TCONS_00031597

GAATAAGTAGCCTCAGGTAGCCTAGAACAAAATTTTGCAGGACCTCATCTACATAGGAAGATCGGGGTCCAA
ACGTCTTTGGATAGCAGCTGCAGCCTCGAAGTTGCCAAGCTTGTATTGATAAGCCATCTCTTCTTTTAATTGAG
CTTGTTCTTTCATTGCTTGTGCCATTCCACTCTGCATCTCCCGCTTCAATGCTTCCACCTGTTCTTCCTTGTGCTT
TTCACGTTCTCGCAGCAATTCTTGCCTGCGCTGTTCTTCATCATCGAAGACAGTACGTTCAGTATCCACGACCT
TCTTGATACCTCTTTTGTTTTCCTTTTCCATTTTACGTCTATAATAGGCATCAAGGTTATAATATTTTTTAGAAGG
GAAGGTTGCGGTGTTATGATCCTCCATGAATTGCTTGAACAATTGCTTTTCTTCCCAGTTTGGCAGGCTCTCCA
GATTCACCTTCTTTATTTCTGCTAACCATGCGGTAAACTCTGGTCGCTTGTTCCACATATCAGTTTCCTTGATAA
TTCCATATTTGCCCCAAGAGTTCGTAACAGCACCAACTTTCCCCTTCTCTTTCTTCTTCTTCTTTCTCTCCTTCTT
CTCTTTCTCCTTCCTTTTCCTCTTCTTCTCCTTCTCTTTCCTCAGCTTTCGTTTCTTCTCCTTCTCTTTTTCCTCTCTT
CTTTTCCTCTCTCTCCTCTTTCTTCGCCTCCTCTCATCCTCCGACTCATCCGAATATTCCGAACTACCCGACTCC
GTATCTGAATA
>TCONS_00031602
ACTACTCGGCTTTAGACGGGAAATGGTTTCCGCCGGTGGCGATCTTATACAGGTAAAATCCAGTCATTACCAC
ACTGAGACCAGCAACAATGCCAGCTGGCATTATCTTTGAAGTTTGCATGTATCGCTGACCCATGACCCATGTT
AGTACAGCAGCACAAGCAGTTTCAAGAATCAAGGCAAAGTAAGAATTTTTGCGCTTGTGAAATGCTTGAAGA
CTCAAGTAACCAGCTAAAATGAGCAGAAATCCAGTACCCAAACCTCCAGCCAGTGAAGCTGTGCTTCCTTTC
TTGAAATATCCTATAATACCACCACATACAAGAATTAATCCATATGGGATTGTGAAGCAAAAATCATGCATTT
TTATCAAATGGGGTTTAAAAAAGATTGATATTTCGACGAA
>TCONS_00031621
AGCAGCAAATGGAAAATGAGTATCTTCATAGAGCTTAAGTATATACTTTAAACCAGTCGATGTACCTAAGTT
AAACAAGTATCTTCATACAGAAGACACCTTCTTGGAAGAAAGATAACGCCAGATAGGCGGTATTTACAAGTT
TTACTAAGACGTATTTACACGGGCAGTTTGACTGCATTTTTAGAACGATCCGAACGTATTCTCTCCACTGTAAG
TCATGTACAGGAAGCCATCCTCATCTTTGTGTTCCTCATAAATCGCGGACATCATGGCAGCTGTGGGAGGAAG
GATATTTTCACAAAGATAAAAATAGCCTTCTCAGCACTAAGCTTTATCCTCTTACGAACAACATACACAAATT
GCCCCACAGTCAGATCAGCAGGAACCAAGTATTTTTTCTTGTCAATGTCAGGAATGTCACTTCTTTCAGCCTTC
TCCACAATAACCGGTATTCTATCAGGATACTTCTCCCTGATACGAGCAGCTTCGGCCTGTCGCCTTTCAAGTG
GGTGTTCCAATTTGAAGGAGCTTTTGGCCATGGTGATCGATTAATGTGTCCTAGTAAAATAAGTTAGCTCGAC
GAAATATTTTACGGAT
>TCONS_00031632
TATTGCACCATTGGCAAAGAACCTGGATATATATATTCACTTTGCAGCAACCAGAGATGATTATTGCACCATC
GACAGAGAACCTGAATATATATTCACTTTGCAGCAACCAAAGATGACTATTCCACCATCGACATAGAACCTG
AATACATTCCTCTTCGTGCTGTCTGCTTCATCCAGAATGGAACATGGGATATGTATAATTAACTGACCTTGGAT
TCTTGCAAACCCTGAATCATTGCTAAAGCTGCTGAATTCTCTGCATCTTTTACTCGTGACTTCTCGTCTCCCGTC
ACAGAAAACACATTTTCACCAATTGAAACTTGCACAGAGCATGCAAAGCTCCTATTGTGAGCAGGACCTATC
TCTTTCTCTATTCACCCAGAAAGAATTCACATCGAAGCCACAATCCACATATACAGCCCCCATGACCGACTCC
ACAATGTCTGCAAGAATTTTTGGCGCTTTCATCACTCCTCCATAGAACTCCATCTCAGCCTCCTGTTGCACTGT
TATCACGAATTC
>TCONS_00031722
TCCAGCTCCCCCAAAAGAATCATCACACATCGTCATTTTGAGCTTCTGATAGCCAACCGTAACTGTGCATTGA
GACAGTCAGCATGGAAGTCATCAAGGCCGAAGCAGTGGCTGTGTGGTATGGTTGCAGTGACTCCACACAAGC
GCTCATCTCAGCCGGACACCTGAAGATACGATTGGAGAGAGCACTTCTAGAAGCTGCGCGAAAAGATGAAG

GAGCAGTTTTCGATTCGGATGCTATTTTGGAAGCGGAATTGCGGACAGATGGTGACCGGAAAACGGACCTGG
CGGCGGCCGATGCGGCGGCGGTGGCC
>TCONS_00031769
TACAAGGTGTATTCTAACAAGGAATTTGCTCAGTAAACCACTTCATAACCGAAAAGGGTGCTTTGATTAATCC
CAAAGCCAACTCTACCAATACTGTTACGCAAATACAGCATGGACATATGCAACTCAATATGATTCCAACAAT
CCAGATGACAACACCAACGATGGAGAGGAGAAGGGCAACAAAGGCAAAAGGAAGACC
>TCONS_00031824
TCTTTTTATATCTCATCTTTGTGTCATCAGGCACTGTTGAAGGCTTGGACAATGGCGTAAACCGCATTTACAAA
TTTCAAAAGAATGGAGTCAGAGAAATACCAAGAAAGTTGATGATGCTAGATGCTGTGTTAGATTATGACTAT
GCAGGACCTAATCCGAGGCACGAACCCGGTCGAAAGAGAGGTGGCAATCACCCTTGA
>TCONS_00031828
TCTTTGGTCTTCTTAACTCTAGTTCTTGTAATTGATCTCTTTGTTTGCAACTTTATCGAGCTGTCTCATATCAAAT
TGCCATAGATGTCCTTAAGTTGAGACAATGGAAGCTGCTTCTGCAAATATAGGTTGCCTTAGCAACGTAATTA
CCAGCTTAAGGCCTGCAGCATATCAGAGCTCTCTATACAAAAAATTAATGCTCAACTTTGCTATCCAGTTTAT
GTTATCTTGCATTTTCCTTGTGAGGTTTCACCTTTTTTGACACCACAAGCTGCTTGGACAATGACTCAGCAATA
AGTTGATGGCCAAACAAGTGAGAGTTCTTCAGAATCAAATTCTTTGTTGATGAATCAATAACATAGCCTTGAC
CAGACATCCACTCCAGTAACTGCAGAAGTAGTTCCTTCTTTTCTGGTTTTGAAACGTAGCGTGATATAGCCAT
ATCAAATAATTCCTGATCAATCCTGTGGCCAGAGCTGTGCATGGCCTTCATCACATCATTGATTAAGTTTACAT
TGCCAGATCTCATGAAGTTGACAGCAAACCTTTTGATAGCCGGACGGCCGATGAATCCTGCATTGTCCTTGAC
AACTACATAAGCATCTTTGAGAAGGCGCCCAGCAATCAAAATGTGCAAAATCTTCTCATGAAGGGATTTGGA
TATGGTTCTTTTGCTATATCTCAGCATGTTATATACAGAAAATGCTTCTGAATGTGCATCTGTCTTACCAAGAT
GATAAATTAAAGAGGAACAAAGTCCCTCCTCTAGCTGGTGACCTTTTCTATGCATGTCCTCCATGGTACGATA
AGCAAGCAAATATAGCTTCTCCTTGCAGAAATATCTTATAAGAATATTAAAGGTATTCCAATCAGGACTAATT
GCAAAGTCATCCATCTTCTTCATCATGCTCATTACATTCTCTATATCCCCTGCTCTGCAATAGGCAGAGAGCAT
TGCATTCAAAATAACTACATCATATTTTTCATATTTTTCTTCAAATTCAGAAGCTAATTTCTTGGCATCTTCGAG
AAGCCCACTGCGGCAGAATGCTGAAATCATAATGCTATAAGAGTATCCATCAGTTTTCACATGTTTTC
>TCONS_00031861
TTGCAAAGAAATAGATTTATCGACCTTAAGCATAATAATCAAATCCTACTCTTTATGATAATTTATTACCAAA
TTTGATCATGAGATTTCACAAAGCTACTTTTTACTGAATGAATCAACTCACGTGATTGTGGGTTCGAAATTACT
TCAGCGGGTCCAGCACTATCAGAAACATGCACATCTGCCATCATAATAGTCTGTAAGGCAGATTACTTTAGAT
GAATAGTAATCAAC
>TCONS_00032017
CCACTACTTACTTTTTCTCCTTGCTCAGCACCATAACCACTGTAACCCTTTAATGTTGACAGGTCATGTGCTCTC
TGTGCCATGGCAGAACTTATGAATTCAGGAATGGGCTCAGTAGTGTCACCAATTCCAAGGCTTATAATTTGAG
CATCAGGGTGTTTCAACATGTGTGCAGATCTCCTTCTTGCGATCTCAGGAAAAAGATAACCAGCTTGAAGTTT
AGCTAAGTTCTCATTGCGAGAGACCTTTGTCTTGTAAGAAGTCTCCGTAGAGGGTGTTGCAACACATCTGACA
ATACCACCGGTTTTGCCTGGCAGTAACACATTCTGGCTCCTGAGACGGAGTTTGTGCTGACCCAATAAAGTAG
AAGTTGATGAAGAGATTGAAGTGGAAAAATTTTGCTGAATAGCTGCCATTTTTTTCTGTTAGAACAAACCACG
TTATCTCACTGGAGTA
>TCONS_00032019
CTACTGTGATACATTACATCCTTTCTGTTTTCTTTTAAGGGGAAGCATCAGCGAATGCAGAAGCATAGACGAG
GAAGAATACATGGAGTTATAATACCACTAGAAATCTCGAGGTGCAGGATGAGCGTGATATTATATCCTCCAT

GCTATTACCTCTGTAACTACATTTACGAAGAAGAGCAGCAATAATTTGACGAGTAGCATCATCAGCTTCTACT
AATAAAACTCTAAGCACCATTTTCTTCATACACTGACCATCTCCTCCTCCTCCTCCT
>TCONS_00032038
CTACCATCACCAGACATAAAATGTTTGTTTGTTATAAGTTATGCAGATTCTTCATAGGATTCTTATCCGATAGC
AAATTTATTTCTTGTTTTTAGCATATGTTTAGAATCTGCTCTAGATACTAGTATTTCTAGACATTGTGTCCCTTG
GTCTCTCCGGATTTGTCTTTACCTCTGAATTTTCATTTGGACCTACTACTGCATGGTCCTTATCATGCTTCTCTTT
TGGTCCCCATATTCAGGCGGGAATCAATTAGACGAACCTCAAACCTGAAGGGTGCTTGGGACCGTCCTTATC
CCACTAACCGAAAAAGGTGTATATTCAAAATTTTGATGACTAACTTAACTTGAAGCAATGTGGACTACACATT
CTTTCATTGCAAATGAACATTTTCAGTAGAATTTAGGAGCATAACTCGACTGTTTGATTAACAAACTCCCACT
GGCAGCGTCTCTTTTGGCATGGTTATCATCTTTCTTCTGTTGTGATAAG
>TCONS_00032100
GCCAAATAAAGAGAAAAGAACCAATGATAGAAATCCAGAAATAGATAAAAAAAAACCATACTCTTCACAT
CAGCCAAACTGACTACATTCATGCAGACATAACCTGCTTGATAATTCCAAACGCGAACATAACAGGATAAAA
GCTATAACAGCTGAAAGGAGTACAGAGCCCTTGGAACACAGGCTACACAAATACTGAGACAGTGCTTGAAC
CTCTAATATTCATCTCTGTAAATTCACGAAATGATTACTCCATCACTTAAGATGTTGGGAGATATCTTTCGGCA
TCACTTTTCCACAACCAATCCCCACATTGATATATGCCGTCAGATGAATATAGTCCAAATACCATAGAGCAAC
AACTTTTACAGCTTTATCCGATGAACATACAGCGAAAAGGAAACAACGACAAATGCCAAATACTACATAAA
CCAGGGGACTCACCAACAGTCAGTGTTTTACCGGACCTATCCAGTGTCAGTATGTACTGCTAAATTCTTTTTGG
ATCACTTAAATTTTTGTCATTAGATCTAAGATGAACTCCATTGTCTCAGCAACCGTGAAACAGCCAAGTTATC
CATTCTCTCAGACTCCAAGGAGTGGCATACCTTCAACTTGAGATTTCTATACAAAAGACTCACCATTGGTGCA
CCATCAAACATGCAGATAAATGATCACTGGCAATATATAGCCTGATCTATGTGCGGCGGAATTTGCTTGATCT
CTGTTCCTAGCTCTTGTTCAATTCGATACAGATTGAAGCGATCCTCAAAGGTAATCAGGTTCACAGCTAAACC
AAGATGCCCAAATCTCCCTGAACGCCCAACCCTGTGTAAATACGTCTCTGAGTTCTTCGGGAAATCAAAATTT
ATAACGACATTGACTGCTTGAATATCAATCCCTCTAGTAAACAGATCAGTACAAACAAGGTTCCGGCAAGCA
CCATTGCGGAAGTCATGAAACACTCTGTTCCTGTGATCTTGAAGCATCTTTGCATGGATGTAGAAGCATGAAT
AGCCTAGCTCTGTAATCTTCTTGGCGAGAAGTTCCACACGGTTTACAGAGTTGCAAAAGATAATTGATTGATT
GATTTGAAGCTTTGAGAATAAAGTGTTGAGGCAGTGAAGCTTCTGCCTTTCTTCTACAAAAGCATAAAACTGT
GTTATACCCTTGAGAGTGAGTTCGTCCATAAGGTTGATAACATATGGCTTCTGCAGAAATCTATCTTTGAAGTC
CTTGACTGTGACAGGGAATGTAGCAGAGAACATTAAAATTTGACGATTTGTTGGCAAGAAACGAATCAGCTG
CACAATGGAAGGTTGGAACTCTGGAGAAAGAAGCTTATCAGCCTCATCCATGATAAGCATGGAGCAATCTTT
CAAAACACATATTCCCTTTCTCGCAAGGTCAAGTATTCTTCCAGGAGTTCCAACTAGTAAATGAACTGGTTGA
TAGAGTCTCATTATATCATCCTTCAAGCTGGTACCCCCAGTGGTAACCATGACTTCAATTTTTAAGTGCTTCCC
AAGTTCTTTACAAACCTGAGATGTTTGAAGAGCCAATTCTCGTGTGGGAACAAGAATAACAACTTGAATGGC
ATTGACGTCCTGATCAATTTTTTCCAATGCCGGAATGCAGAAAGCAGCAGTTTTCCCAGTTCCATTTTTGGCCC
TAGCCAGAATATCACTCCCAGTAAGAGCGATTGGGATACTTTCTTCCTGGATTGGAGATGGTCTCTCAAAACC
CTTCTCATAAATTCCCATAAGCAATTCGCGCTTAAGAAAATAGTCTTCAAATTCATTTCCCTTAGTCGCTGTCA
CATCCTCTGTCCTGTATCGAGTATCAGGTGCTGGTATGTTCAACCTCGCCTTCCAGTCTTGTGAACTTTGGTCA
AGTGCTTCAGATTGAACAGTCTTTTCAACCTCATCGATGGACGAATCAGATGCCGCCAACTGTTGGTTCCTTCT
GAGCCATTGCTGCTGCTGTGTTATCTGTTGTTGTTGGTTCTGATAATGCTGCTGCGGGTGCTGCTGCTGGTGATT
CATCGGATTTCTCTGCACATACTGCTGCTGAGGGTTTCTTTGTTGAAAATTAGGGTTTGCGTTAGGGTTAGGGT
ACATGTTTCCGCCGCCACGTCCTCCGCCACCGCCTCCCATTCCCGGCGGCGGATATCTTCCCCCTCTCGGATTC
ATCGCCAATAAAGAAAAAAAAACTAAGAATTATAACAAGCGTAAAATGAAGAGAAAATGAATTAGCAATA

TAACTTTATGAATCTGAAGAATCTGTGGTGAATTTGACGAAATTGTGAAGAAAATTTAGTGAATTGGATGAA
ATTTTAGGGGAAATAATTGCAGGGATCGTGGGCCTAGG
>TCONS_00032101
GCCAAATAAAGAGAAAAGAACCAATGATAGAAATCCAGAAATAGATAAAAAAAAACCATACTCTTCACAT
CAGCCAAACTGACTACATTCATGCAGACATAACCTGCTTGATAATTCCAAACGCGAACATAACAGGATAAAA
GCTATAACAGCTGAAAGGAGTACAGAGCCCTTGGAACACAGGCTACACAAATACTGAGACAGTGCTTGAAC
CTCTAATATTCATCTCTGTAAATTCACGAAATGATTACTCCATCACTTAAGATGTTGGGAGATATCTTTCGGCA
TCACTTTTCCACAACCAATCCCCACATTGATATATGCCGTCAGATGAATATAGTCCAAATACCATAGAGCAAC
AACTTTTACAGCTTTATCCGATGAACATACAGCGAAAAGGAAACAACGACAAATGCCAAATACTACATAAA
CCAGGGGACTCACCAACAGTCAGTGTTTTACCGGACCTATCCAGTGTCAGTATGTACTGCTAAATTCTTTTTGG
ATCACTTAAATTTTTGTCATTAGATCTAAGATGAACTCCATTGTCTCAGCAACCGTGAAACAGCCAAGTTATC
CATTCTCTCAGACTCCAAGGAGTGGCATACCTTCAACTTGAGATTTCATTGGTGCACCATCAAACATGCAGAT
AAATGATCACTGGCAATATATAGCCTGATCTATGTGCGGCGGAATTTGCTTGATCTCTGTTCCTAGCTCTTGTT
CAATTCGATACAGATTGAAGCGATCCTCAAAGGTAATCAGGTTCACAGCTAAACCAAGATGCCCAAATCTCC
CTGAACGCCCAACCCTGTGTAAATACGTCTCTGAGTTCTTCGGGAAATCAAAATTTATAACGACATTGACTGC
TTGAATATCAATCCCTCTAGTAAACAGATCAGTACAAACAAGGTTCCGGCAAGCACCATTGCGGAAGTCATG
AAACACTCTGTTCCTGTGATCTTGAAGCATCTTTGCATGGATGTAGAAGCATGAATAGCCTAGCTCTGTAATC
TTCTTGGCGAGAAGTTCCACACGGTTTACAGAGTTGCAAAAGATAATTGATTGATTGATTTGAAGCTTTGAGA
ATAAAGTGTTGAGGCAGTGAAGCTTCTGCCTTTCTTCTACAAAAGCATAAAACTGTGTTATACCCTTGAGAGT
GAGTTCGTCCATAAGGTTGATAACATATGGCTTCTGCAGAAATCTATCTTTGAAGTCCTTGACTGTGACAGGG
AATGTAGCAGAGAACATTAAAATTTGACGATTTGTTGGCAAGAAACGAATCAGCTGCACAATGGAAGGTTGG
AACTCTGGAGAAAGAAGCTTATCAGCCTCATCCATGATAAGCATGGAGCAATCTTTCAAAACACATATTCCC
TTTCTCGCAAGGTCAAGTATTCTTCCAGGAGTTCCAACTAGTAAATGAACTGGTTGATAGAGTCTCATTATATC
ATCCTTCAAGCTGGTACCCCCAGTGGTAACCATGACTTCAATTTTTAAGTGCTTCCCAAGTTCTTTACAAACCT
GAGATGTTTGAAGAGCCAATTCTCGTGTGGGAACAAGAATAACAACTTGAATGGCATTGACGTCCTGATCAA
TTTTTTCCAATGCCGGAATGCAGAAAGCAGCAGTTTTCCCAGTTCCATTTTTGGCCCTAGCCAGAATATCACTC
CCAGTAAGAGCGATTGGGATACTTTCTTCCTGGATTGGAGATGGTCTCTCAAAACCCTTCTCATAAATTCCCAT
AAGCAATTCGCGCTTAAGAAAATAGTCTTCAAATTCATTTCCCTTAGTCGCTGTCACATCCTCTGTCCTGTATC
GAGTATCAGGTGCTGGTATGTTCAACCTCGCCTTCCAGTCTTGTGAACTTTGGTCAAGTGCTTCAGATTGAACA
GTCTTTTCAACCTCATCGATGGACGAATCAGATGCCGCCAACTGTTGGTTCCTTCTGAGCCATTGCTGCTGCTG
TGTTATCTGTTGTTGTTGGTTCTGATAATGCTGCTGCGGGTGCTGCTGCTGGTGATTCATCGGATTTCTCTGCAC
ATACTGCTGCTGAGGGTTTCTTTGTTGAAAATTAGGGTTTGCGTTAGGGTTAGGGTACATGTTTCCGCCGCCAC
GTCCTCCGCCACCGCCTCCCATTCCCGGCGGCGGATATCTTCCCCCTCTCGGATTCATCGCCAATAAAGAAAA
AAAAACTAAGAATTATAACAAGCGTAAAATGAAGAGAAAATGAATTAGCAATATAACTTTATGAATCTGAA
GAATCTGTGGTGAATTTGACGAAATTGTGAAGAAAATTTAGTGAATTGGATGAAATTTTAGGGGAAATAATT
GCAGGGATCGTGGGCCTAGG
>TCONS_00032147
TGTAGTATGATCGTGCACCATTAAGAACATAACCGAATTGATCTATCAGAGAAACCAGATTACTCACAATCC
CTTTTGCAGTTTCATACATCTTGCTTGCTAACAAGCCCCTTATTATCCAGCAAGAATCCCAGTAATAAACCTCT
CTGTAACGTGATCCTGCTATAAATAACTGGATTTTTCAATGGAATCAAAGTATACAACTCTGGT
>TCONS_00032148

GCCGGGGTTCATTCCACATAGCATAGTATCGACTCAAACTATGGTTCGATCCCTGAGTATCTTGAATAGTCAC
TTCATGTATTCCTGAATTCCAAAAATGATACTCCTTGAGCAATGCAGGAAGAGATCTTCTAACCAAGTCCAAA
TCGCCTGTCCGGTTGTATATTTCAACAATCATTGCAGACAGGAGAGGAGGCTGACTGAACGTGTGAAACACA
AGATACGATAAGAATTTGTACAGCATGAAAAGATGAGCTAAGTAGT
>TCONS_00032149
ATTTGCTAAAAAGGCTACGTCAAGTTCCATTTCAGAAGGAGCGCATTCAGATCAACTGGTAGAATTGATGTTG
TACTAGTTGTTGTGAGATCAGGTTCATTCCTCATCCATCTTGAACTGAAATCCCATCCACTTTCTGCAGCTGAT
GCCAAGTCGCGATAAAATTGTCTTTTCTCACAGATGTTTGGGAGTTTGGAAGCTGTTTTACTGTCCTGAATTCC
AAAAATGATACTCCTTGAGCAATGCAGGAAGAGATCTTCTAACCAAGTCCAAATCGCCTGTCCGGTTGTATA
TTTCAACAATCATTGCAGACAGGAGAGGAGGCTGACTGAACGTGTGAAACACAAGATACGATAAGAATTTG
TACAGCATGAAAAGATGAGCTAAGTAGT
>TCONS_00032150
TTACTAATTTCGTCAGCTTGAATGGTTACTCTGCCTGCTCAGTAGCAGTCAATCTTCAAGTCTTCAGGCCATCC
AAATTCCTCCAGAAGTGCCAGTACAACACCATTCGCCCATCCAAAACCCGTTTGGGCTACATACACACCACC
ACCTCCATATGCTCCCCATTTTGTCACGTCGTATTTTTCATACATTGCACCGGTTTTCTTGTAAGCTACATAGTT
TGTTCGTATCCATCGGATAACAATGTCTTTAGCCAAGGCACTTGCCTCTTCTAGCCCAGACCTTGCGAGACCTT
CAATGATCATGTGTTGAACGGGGGG
>TCONS_00032171
GTAACATAGCATTGACAAGGCTAGGAAAGACATCAAGCACAGTTGCAGCAAAGTATGTCCAAACAGCACTC
ACAGGTCTCGTAGCAACAGAAGCAGCAACACATTGCAAACAAACAAGCATAAATGCATCCTTTTTCCTCGTG
TCTGCGTTGCACATGTTCAACATAAGTCATCTCCTGATATGAGGGTGCGTCCATTATTTTCTCGTTTGTTCTAAG
CTTATTGCACCTAAAGGCTTTTCAGAGTAAGTAATTTATATAATACTGAGAGAGCTTGAGTTGAGTGAACCAC
TTTAATTCCTTGTATG
>TCONS_00032191
CAAGATTACTAGCAAAAACTTGTGCTTTCTTAGCTTCTTGAACCCAATCAATCCTAGCAATAAACACCAACAA
CAAAACCAAACAAACAGCAACTCCAACCACAAATCCTGTCAACAATCCAGTAAGACCAAGATGAATCTTGA
AAGCCAAAACCACACCTAATGGTAAAGCTAAGAGATAAAATCCACTAATATTAGCATACATTCCTAGCCATG
GCCTAGCCGTTCCTCGTACAATTCCTCCACAAACTGCTAATGGAAAATTAACCACTTCAAGTAATGCCATTAA
CAACATCATTTTCTTAACACCATTTATAATCCCTTTATTATGACTAAACAATGGCCCCCAAATTCCTCTTGCAG
CTGCCATAACAGCACCACCAAGAAAACCCGAGCCAACGCTCATAGCTAACGATACATAGGCTGCGCGATAA
GCAGGACCAGGACTATCTGCACCAAGCTCATTAGACACACGAATAGATGCACACGTTGATAACGAAAGCAT
AACAGAAAACAACAAATAATCAAAATTTAACACAATGGCTATAACTCCAATTGCTTGTTTAGCATTTGGTAG
ATGTCCTGTTAACAAAACCAAGATTTCATAACACCACCATTCAAGACAAGTAGTAAGACAACATGGTCCACA
TAATTTCAGTAGTCTGATCCAATCGCGAATTCTTTGATCCCACCATCCTCCTTCTTTCCATTTTCCTCCCTTTTTA
TTCTCTGCGAGAAATGCAGTTGTGATGGCTGTTTTTGCAAACCATGTGAAGTTCATAGCCATCAAAGGAAGTA
GTATTCCTTTTTGCAATTTTAGCTCTGAAAGGGGATTTTGCATGAGGTTTGATGCTGGCCATTTTTGTGTTACAT
TTTCTGGTGAAGGTGGCATATTTGGTGGTGGTA
>TCONS_00032219
AACATACATATGCCCATACACATAATCATACAAATTATATAATCAATGAAACAAGCGAAAGATCGACGAAG
AAACAAACCTCGATAGCACAAGGGATTTTGAGGTAGTTGCAGCAATCACTAATCTCAGTAACAGTAGGGTTT
TCACATGCTTTTGTCACGCAAATTCTCCTTCCTTCAGCTAGTGTCTTCTTTGAATTTATATAAATTGGATACATT

ATGTTCCACTTCTTGATATTCTTAATTTCACTATCCATCTTTCTGATCTCTTGATAAAGGAGAGAATTGAAGATT
GAAACTGTTGATCGGATCGAATGAGAACGGGGATTTGCAGAATTCGGTTTTG
>TCONS_00032243
CAGATTACGTATCGCTTCCTGAAGGGTTAGATTTATCGCATTGTAAAAAAAGAAAAGGTTCAAACGTTGGAG
CTCTAGAGAAGTCGTTCGGCATTGAGAATAGGAATATCGCCGACAAAATGGTAGCCCGCATGTTCTACGCTTT
AGGTTTATCTTTTAATTTTGCCAAGTCTCCTTATTTTAGAAAGTATTCCGAGTTTTTGGCAAAGACTTCTATACC
CGGTTATGTTCCGCCAACG
>TCONS_00032253
GGGCGGGAAATCAATAGACACAGAAAGAGAAGAGCAAAAGGAGCAGAAGGCACTCCGTTTTTTATGTGAA
ATCAAGGAGCAGCAGGTTAGACTTTGACACTTTCCCGAAGAGAGCTCAAAGCAATAAGATGAAAGATGGGA
TGAGATGGTAGCCTTAGCGCAGAAGATAAATCATTGAACCTTAGGCCACTACCGAGCAGCAATGTAGGATCA
TAACTGCCTGTACTGCCGAGATGATCCCTACTTGAAAAGGCGGAGACAGATTGTTGCGACAAATCGAGTTTC
AAAAGCCCATTTATCAATCACATTTTCAGGCACACCAGTCAAAGAGCCATCGGACCCATGAGAGTCCCATCT
TTGACTAATATACACGGGCTTCTGAGAGGATGTCCTGTTTAAGCAGCAGCCAAGGCGCCAAGACCCGTTGTTT
ACAAATCAGGTCAAAAGCCCAATATAACGCATTTGGAGGGCATGGCGGTACAAGGAGCCAAGCCATCAGAA
TCAGACCCGTAAAGGTCTCGTTTTTGACAATAAACATGGGCTTCTGAGAGGATTCTTTAATCAACCAAAACCT
GCAACAGAGTCCTTAATTTCTACCACTTACAAGTGGAGAG
>TCONS_00032378
GAGAAAGAACACAGCGAAGTTATCAATTTGCTTCGACTGCCGGCTGCAAGAGTATTATTATTTTAGTCGTGAT
AATGGAGCCTCGTGCAGATACTAACCTTGATACAGACATCTGGAGTCTGAATGATCAAGATAGCAATGCAAT
TATATTGCCAGACAAGAAAAAGAAGAAGAAGACAGAAAAGGAACAGGTATCGAAAAAGCTTAAAACGAA
GAATAACATTAAGTTAAGTCAATCTCAAAAAAAAAAGCTGAAGAAACTCGAGGTAAGTGCATCCATGAATG
TTCATTGTTTTCTTATTTTG
>TCONS_00032441
CTTTCAGAAATTAGAAATAGATAGTTGGCAAAGACAACACTGGCAGATATGGCCGCTATATCCGACTTAGCA
ATATAATTGCTTTCAACTTCAGCTAATAAAGGGAATTGTTTTATTATTGTTGATTAAAGCTTTTTAAGGAACTA
GCCAAGTTTATGCTCATATTAATGGTCATACCACAATGATTGATAAATTAAAGTTTCAATGCTTCTGAAACTG
CAAGGGTAAAATACATGTGGTTCACATTAAGAAGTTAGGCAGGGCAATCACAGAGACAACTTCCTAGCATA
ATCATATTAGTTCACGTCAAGCCATTAGGCAGGGCAATTAAAGACAAAATCTTCCCAATGTTACTATGTATCT
GCCACAGGAAAGATGACACTCTAAGCCTTGACCAATTTGCAATATCCTCACCTCAACTCTAGTACTGAAAATT
CCAGTTGGTGTGACCGACTTCTTTGGATCAAAAGCATCAAGGTAACCTTCCATTGATGCTCTTAACCGCGCAC
AGTCAGGGTTATGTTGCAAGTACGATCTCAAATCTTGAAAGCACCTTTCCTCTAAAGTCATGGTTGCATAGAA
ACTAGAATCTGATGGCAACAATTTTTGAAGAACTTGAAGCACTAATAACTGGGTGAAACCACGTAGTGTATG
ATGGTGTGAGGTCAACAAAGGAATTATACAAGGCAGTAAAGCACTCAGATGCCTGGACTTGTACTCTTCAGT
CGAGTGAAGGATGATATTTGCAGCTATAAACACATAGGAAGATAAAGCCTGAGGCCGCATATTGTAATCTCG
CAGCAACGGTACTAATT
>TCONS_00032447
ACCTGCTAGAATTAGGAGTGAGAGGGGGGAGGTGAAACGGGGTCAGAATTGGGATTGCGGAGACTTTCAAT
CAAACCTCCTAGCTCCTTCCGAGCTGACTCTCTCCCGAACATGAACCCATACCAGCCAGAAACGGCAGAGAA
TACACCTGAAATTGCAATTGTCTGAAGGAGAGTGAAGCTCTTCTTCTGCATTTTTATCGAACAGTATTAGATC
AAACTTGCTTTTCTCTGTACGAGAGGTTCTG
>TCONS_00032546

AAAAGAGATCAATTCCAATTGAACAAGACTTCCACATTTTGTGAAATGTGTTAGAATAATGTTATACACAAGT
TCAGTAGAAGGAACGAATTGCAATGTCAATTGTCAAAGCTCATAATACAGATTTTAGACAACTCTATCATCA
CGCAATATAGTACATGGGATTTGGAAGTTTAAAGGACCTTTTGGCAACAGCGAAACCGTTCAAGTCACTCCA
CTGATCTCCAGAGCTCCCATGAATCCTGTAACCTTCATCTTCTAACTCCTTTCTTTTCTCAGATTTATACTGGGT
TGCCAGTTTACCATTATCGGAAGGTCCCCTCAAAATAAGTCTTTCCCAATTGCTGTATCCAGCATACACCATA
TTCTTTTCTGTATAATTCCTTTGAAATTCACTCCGACCAGTCAACAAGAATATCTTAAATCCTCGCTGTTGTAG
CTCCTTGTACAGTTTTAAACTTGCAGGAATAGCAGGAGCTTCTGCTTCATTTACCCATTTGTCAAAGGCGACTT
CATCAAAGATCTCCGATC
>TCONS_00032715
ATATATGTAACCTCAACAATACGTGCAAGATTGAGAATACGAGATAAAAACTCCGTAGAGACGGGAGTGGG
CCTAAGAAGTCCTTCATTAAGATCCTTCCATGCTGCCTCAGCCATTTCTTGAAATTTAGTCATTGCCTCTTCTGT
TGATATGCCATAATCTCTCATGCAGCACTCAATTCCTGTTGCAATTTGTCCCCTACTTTTCTCAACCTCGTATGT
GGCTGTGTCATCAATAACTCGACATATTATCACACTAGCTTCAAGAATTTTAGGATTCTTTGACAACCACTCA
AAATCCTGCTCCGTAACAGACTTCATGCCCAAATACGATGTTGTCGCGAGGTAGTAATATGTAGTAGTTGCTA
GTGCATTGCTTAGGTATTCAGAAACAGGTGGCATATATCCTTCAATAAACCATGTTGACTCGACATTATAATT
TCTTACTACTTCTTTCATCCTTTCTATTGCATGGCACACAATATGAGATCTTCCAGCACTAGACAATTCCTTTTC
ATAATCATTGTAAAGATCTAGAATAGCTTTATAACTGATTTTCATGTAATCAGGA
>TCONS_00032716
ATATATGTAACCTCAACAATACGTGCAAGATTGAGAATACGAGATAAAAACTCCGTAGAGACGGGAGTGGG
CCTAAGAAGTCCTTCATTAAGATCCTTCCATGCTGCCTCAGCCATTTCTTGAAATTTAGTCATTGCCTCTTCTGT
TGATATGCCATAATCTCTCATGCAGCACTCAATTCCTGTTGCAATTTGTCCCCTACTTTTCTCAACCTCGTATGT
GGCTGTGTCATCAATAACTCGACATATTATCACACTAGCTTCAAGAATTTTAGGATTCTTTGACAACCACTCA
AAATCCTGCTCCGTAACAGACTTCATGCCCAAATACGATGTTGTCGCGAGGTAGTAATATGTAGTAGTTGCTA
GTGCATTGCTTAGGTATTCAGAAACAGGTGGCTTATATCCTTCAATAAACCATGTTGACTCGACATTATAATTT
CTTACTACTTCTTTCATCCTTTCTATTGCATGGCACACAATATGAGATCTTCCAGCACTAGACAATTCCTTTTCA
TAATCATTGTAAAGATCTAGAATAGCTTTATAACTGATTTTCATGTAATCAGGA
>TCONS_00032718
GATCTAGAATAGCTTTATAACTAATTTTCATGTAATCAGGAAGGCGATCAATTTCGTTGATATCCCATCTTTGT
ATGGCATCTGTGTATGCCTCAAGTTCTTTAACTGTACCATAAGCATCAAAGGTGTCATCCACAATCGAAATCA
TTGATATGGTCTTAACAAGCATGACGCGAGCTTGAGAGTATTGAGGCTCAAAATAAACTCCTAATGCCCAAA
AGTAGCATTCAACTACTCGATCTCTAGCATATGGAAGTGTTGTTACGAAATTCAAATCTTTCCACCACCTGCA
GCATAACAAATCAACATCAGTACTTGATATATGGTCAATCAAAAAGGGTTCATCGTTTTAATTTATCGCACCT
TGATACTTCAGCAAGTTCTTGTTTGTGCAACATCTGGAGCAAGTTGAAATCCAATTTGGCAAATTGAAGTAAC
ACATCATTTTTTGATTGTTCCTTCTCATAGATTCATGAGATGAAGAATCGGGTCTCGACTCTAGGTACGCCCTT
GTGCAAACATTGCTCAAGGGCATGTGTCACTTGCTCCCTTAGTGGAGATTTCAAATGTGGAACTGCAGATTCA
AGATGGATAGTGGAGAAAGCAAGTGCGTTTTCTAAGATATCGTCAGCATGAGTCCTCACATGTGAAGCTTCA
TACAAGTTCAATAATCCTAAGACATCGCTAGAAAGAGACTCTTTGAATTTGCCATTTTCATCTTGGAATTTGCT
GAAAATTTCAGGAGAGATGTTGAAACCATGTTGCCTGAGCAATCGAAATTGAAGTGAAGAAGTGCTCAAATC
ATCGCAGTTTGAGTTTTGGTTATAAATTTGATCCAAAATCTCATCAATTTCTTTTTCAAAGTGGTAGGAGATGC
CAAGACGTTCAATAATGTCAATCAAATTCAATGTATCAGCCAATTTCATTGCAGTTGCT
>TCONS_00032744

TTTAGGCAAGGATGATGAGAAGTAAAATCTCATAGAAAATTAAAACCCCACAGAAAGGAAGAATCAGAAG
GGAGCTTGAAAAACGTGCTCCTAAACTGGTTGAAACTGGGAAAAAGACATTGATATTACATGGTACAAAAA
CAAGCCAAGTGTTGAATGAGGTGATGACTGAAATTTACCATTTGAAGAGGGATAATTCTGATGAATATACCC
>TCONS_00032770
CCCAACATGTTCAATGTTCCCATCACATTTGATATGATGGTCTTGACAGGATTATACTTGTAATGAACAGGAG
AAGCAGGGCAAGCCAAATGATAAATCTGATCAACTTCAAGCAAAATTGGCTCCACCACATCATGTCTAACCA
TCTCGAATTTAGGGTTTCTGAAATGGTGCAACAAATTCTCCTTCCGACCAGTGAAGAAATTATCAACCACAAT
CACACTATCCCCTCTCTCTATCAACCTATCCACCAAATGGCTCCCAACAAAACCAGCTCCACCTGTTACCAAT
ACTCTCAACTTCTTTTTCTTCAATCCTAATGGCACCTTTCCACCTGCAACACATTAAATTAAACCAA
>TCONS_00032771
CTCAACTCAAGTCTGATTCACTCCTTGTGCGGATGAGGGGCTGGCGGTCGGAGGAACTTGGCAACACGTTCTG
CTTTGGCCCCTGATTTATGGTCATAGTACTCGACGACTGCAGCGCCCGCTAACGCAGCAAGAGTCAGGGCTTG
TGCATGCAGCCTGGCATGAATAATCTTAACGCTGGTTTTCATGTTGGGTTGCGACCAATTGTAAGCAATTGAA
CCCGTTATTCCACTCAGCCATAAGCACCCAACTGTTCGGAGCTTGTGTTCCACCACCCATTCCCTCACCGATTC
CAACGTGTTCTTCTTTTCTGCCATTTCTTCTGCCTATGCAAATAAATAAATTGACTGT
>TCONS_00032964
CCTGTTCTGTGCCTCTGGGATCTGCCAAGAACAAACCAGAGATGCAGCAACAGTTACTTTTCAACTTAAAAGT
TTCAATGCCTGTACACTAAAAGCAACCCAAGAACACAGAAATCTGAATGAATACCAGAAGCCTGATTAGCA
GCTGTGATTGTCAAAACCAACTCAGCTGCTTCATTTTCTCTCACCCCATTTCCTTTCTTGCTCTCA
>TCONS_00033037
GATAATGTATAAATCAAACATGGGCAAAGAACAAGATGCAAAATTTTGAGATCGTCACAACAAAACTCAGC
TTAAACAATGTGAAGCTCTAAAGACTATCACCATATAATTCAGAAGGATGAAACGAGTCTATGCTCCAGGAA
CCTCGGGTTCTGCCTCTGCTACTGTCTCATCAGCTGGCCGATCCAACTTCACACCAACATCTTCTGAATAATCA
CCATGAACCTCCATCAGCTTGCCAATATCAAACTTGGGGGCCTTGAGGATCTTGACTTTTCGGATATAAACAT
TTTGTAGGGGGAAGATGCTTGAAGTTGCTTTCTCAATCTCTCTGCCAATTGATTCAGGGATGAATTTGGCAACC
AACT
>TCONS_00033038
ACTACCAACCTGACGGATCTGGCTGCTCTGAGCATAACAGGTACGCTTCTGCTGGTTTGGACGTTTCTTTGTAA
AAGCAATGCAGAACATCCTCAGGGTATAGCTGTCGGTAGTCTTGACATCCACATGGGCCTCAATCAAAGTTT
GCCATTTGCGAACCAGTGACCTCAACTTGTCTGTTGTGAAATCCATTCCCCAGAAGTTTGTGAGGACATTCTTT
CCTTGCACATCCTCTGCTCTCA
>TCONS_00033050
AAAGATTCTTACCTCTGCCAGGCTGAATGTATGAGAAATGTTGATTGTAATCAAACCCTTAGACAACCAGGAT
AGGAGCTCTTTCAGAGAATCTCCAAGCACATTAGGTTGATATATCTTATGGCTTCCCCAATAGAGTCCGTGAA
TCGTCCAGTTCTGATGGAAAAAAAAGTATAAAAAATGATGGTTATTACTCTCGGATAAAAGAAAATGAATAA
GCTTT
>TCONS_00033099
CGCTAATACAAAGAACATCAGATGAGAAGCTTTTCTGAACTTCCACAGCTAGCTCTTCCAAACTCAGTTGGCA
CTCCAGTCCTATCTTGATGACAAAGGAGTATAGAACAGTATCCTGCATGCTGCTGATGTTGAGATGAAGAATC
TCAAAGGAGAGTTTCTCTAACACATTAATTATCTTCACAACTTGACCTGGAATCCTCTTAGAAATGGTTCTCA
ATATAACATTGGATCCTGATATCTTTGCTTCAACATCAGCCACTGGAGAATTACAACATGCTCCAAGTTCCTT
GAATAGGTTATTATTATTAGGACTAAATGGACTTTCAGGTGTGGGACTTAGTTGCAAAATTGGCCTTGGACTA

GGACCAGGGCTAGGGCTTAAACTCTTTCGTCGCTTTTTCGCCTCCAGAGATTGTAGAACTAGGTGTAATTCTTT
GATGAATTCAATTACTCCTGCTATTATTGATGCTTGAT
>TCONS_00033121
GTGATGGTGGAAGGCCAGGCAATACCAGAATCTTTGTTGGTGGCTTTCTATTAGGAGGAGTAATCATTGGTGC
ACTTGGTTGTATCTATGCGCCTCAGGTAAAAGTGCAGTAGCACTTGTATTGGGCACCGCCTTTGGTCTTCTTTG
CTCTAAAAATACTTGTGAATGTTGCTTCATCACTTCTTGATATAAAGACGACCAAAGGCGATGACCTTTTCCGT
ATTGGTCACCGTCCTTGGTCGTCTTTGCTCTAAATGCCTTTTCCGAATTTCTTCTCTCTTGCTGTACGTGGGAGG
GGTAAGATGACTGCCCTTTAATTTCTCTTTTACAGATTAGCAAGGCATTAGCTGAAACAGATAAGAAGGACCT
CATGAGAAAATTGCCCAAATTTATATATGATGAAGAAAAAGCATTGGAGAAACAGCGCAAGATACTAACGG
AGAAGATTGTCCAGCTGAATGACGCTATTGATGACATTTCCAACCAGTTGAGGTCAGGAGACACACAAAATG
GAGTTGCTGTGAACCTAGATGAAGTTGAAACAATTATATAAGAACACTTCTATT
>TCONS_00033162
GGAACTGTAGGAGTTTTGTTTTACATTACTATCTTAGACCAAGTATGCTTGCTGGTTTCATACTGGAGGAAAGG
CGCTACTCTTCATCCACCACAACACTGATCTCACCTTTCTCAAGCATATCATAAAATTCCTTGGTCCTCCTCTG
GTTATTCCAGTGGTGCATCTTCCACAAGCCTCCCGCGGCGATGCCAAGTGCAATCCCAATCAGTATTTCCTTC
ACCACACTTGGACCCTTGTATGCAGCATGGGCAATATGAGAACCTGCCATTTTTCATACCTCGGGAGAATCGG
TGACGGACAAAAACCAGCGAGAATTGAGCAGACGGAATTGGGAGAGGTATAAAAACTGGGTAGTATCCTCG
TTTCTAGAAGCTACTGTTAGGGGAAAGATTTCGTGCGACGTGTCACACACTATTTAATCTGGTCCGCATGAGA
CTTAAAGAATCACTTGAGAGATTAGTATATCGCAATTGCTCGTATTTACTAGTGAGT
>TCONS_00033191
ATTTGACTTTCATTATTTTTATTTCGTTTTTTTTTATTGGAGTAGTGTGACTATGTTACTATTGACAGATTTCAAT
GCCATAAGACGTATCAATAGTTTTCTATTCTAAATAGAAAGAAAGTCAAATTTGTCTAAATACTAGACATAAG
GAAGCAGGGGATAAATGTGGGGAACAAAAAATTCTAGGAGGGATTATTTGTCTTCCTAGTCTTCGACACAAG
AAAGGGGTGTAGAAAAATCCTTTTTTCTTGTGTCGAAACGAAAGAGTAATGATTCTTGACCCTGTTTGTTAAA
AATTCCTAGTCTTGGTTTCGATTTTTCCAGATGTATCAGAAACCCTTTACGTATAATATACTAAGCGGTGGACA
AACAAAACAAAAAAAGAGAGGAAATTTTATTAATTAAATAAAACTTCTTCAATCAACTTATCTTATACAAAA
TTTGATGATGAAATATGAAAACAATAAAAAATAAATAGAGTAATGTAATAGAGAGAGTAAGGTTCTACATTA
GATTAGTATAGAAAGGATTTGCACGATATCTAATATATTATAGCAGCCAAGAAATTGAGTGATTCCTTCTTTC
TTCCAACTTTGAAAGTACCGATAGATACTATCATAGAAAAAGAAGAGGTGGTCGGAATAGTGAATTTTTCAA
AAACATGATCAGAAAAATGAGAAAAATGGAGTTTTTGAAAAGAAAAAGAAATCCATTTTATCATTTAGACG
AAAAAAATATTATGATTCTTAAGAACTCAACGGGCCCTTCCCCTTCGAATCAAACAAACAAAGAAGGGAATC
CCGTTGAGTTCTTACGCTTTCATGTTGACGACTCAATTCATTCGATTACTACAGGGATGAACCCAATCCGGAAT
ATGAACCATAAAAGAAAATACCTATTAAACCGATTACAAGAATACCAGCTACAGTACCTATTATCCAAAGA
GGAATCCTTCCAGTAGTATCGGCCATTTACCCCACTTCCCTCCAGATTTCATCAAGTGGTCATGCTAGAGACA
TAAACAGTCATGGATAATTAAATTATGAGATCCTTCCGAATGAGCTAAGAGAATCTTATTTATTCTCTTTCGTT
TTCTTAATTGAAGAAATAATTGGAAAATAAAACAGCAAGTACAAAAATGAGTAATAACCCCCAGTAGAGAC
TGGTACGATTCAATTCAACATTTTGTTCGTTCGGGTTTGATTGTGTCGTAGCTCTATAATTCGGATTAAGTTTAT
CGTTGGATGAACTGCATTGCTGATATTGATCCCAAAAAAAAGACGGTAGGTACAGCTAGGCCGTGAACAGCC
AACCATCGTACTGTAAAAATTGGATAGGTTCGATCTATAGTCATTAGGGCCTCCTAAAACGATCTACTAAATT
CATCGAGTTGTTCCAAAGGATCAAAACGGCCAGTTATTAATGGAATTCCTTGTCGGCTCTCTGTAAAATACTC
GTTTGGCCGAGGGCTTCCAAACACATCGTAAGCTAAACCGGTGCTGACAAATAACCAACCCGCAATGAATAG
GGAAGGTATAGTAATGCTATGAATGACCCAGTATCGAATACTGGTAATAATATCAGCAAACGAACGTTCTCC

TGTGCTTCCAGACATGCTGAGCTCCACATATTCTTGTACAGTCAAAGAAGATCGATTCCGTAAAAGATGAGAT
CAGTAAATGACAATTCACTGAAATTTCATCTTTGTGAGATCGTCAATATTGTACCGAGGGCGTCTTTAGAGTA
TACC
>TCONS_00033258
CAGATATTAAAAGACAATTTGGTACAAATTTGGCATTTGTATCACTTCATCTTTTCTTCCTTGAACAAAACATG
ACGATTCACACGAGGATCAAACTTTCTGAATTCAAGTTTCGTCTGAGTCGTTTGAAGCTTTTTGGTCTTCTTCTT
TACATAGAAAAACCCAGTTCCAGCAGCTGAAACGAGCCTCACGAATAGAGCTCCCGCCTTCTTCTTATCACC
CATTAGCTTATC
>TCONS_00033277
ACATCAAGTTGATACAATGAGCCGCACATGGAGTCCAATAGACATTCTTGTACGCTCCTTCAATCATGCCACC
CATTTTCACATTTTCACTTGCATTATCAGTGACCACTTGAACAACTTTGCTTGGACCAATCTTTTCGATGGTGTT
CTGAAACAAGGTGAACATTTTGATGTGGTCGGTGGATGAGTCGCTAGCATCAGTAGACTCAAGAAACAAACT
TCCCCAGGGAGAATTCACCAACACATTAATAATCATTTTCCCCGTTCTTGTCGTCCACTTATCCATCATAATGG
AGCAGCCGTACTTGTTCCACACAACTTTATGCTCCTAAACAATTTTATTAGTCTCCTCCACTTCCTTATTTAGAT
AAGGACCTCTTATTTCATGGTAAGTGGGAGGCTTCATT
>TCONS_00033278
ACATCAAGTTGATACAATGAGCCGCACATGGAGTCCAATAGACATTCTTGTACGCTCCTTCAATCATGCCACC
CATTTTCACATTTTCACTTGCATTATCAGTGACCACTTGAACAACTTTGCTTGGACCAATCTTTTCGATGGTGTT
CTGAAACAAGGTGAACATTTTGATGTGGTCGGTGGATGAGTCGCTAGCATCAGTAGACTCAAGAAACAAACT
TCCCCAGGGAGAATTCACCAACACATTAATAATCATTTTCCCCGTTCTTGTCGTCCACTTATCCATCATAATGG
AGCAGCCGTACTTGTTCCACACAACTTTATGCTCCTAAACAATTTTATTATCTCCTCCACTTCCTTATTTAGATA
AGGACCTCTTATTTCATGGTAAGTGGGAGGCTTCATT
>TCONS_00033286
AGAAGTGACAGAGCTAAGAATCTTTTCTTGCTTCTGCATCCTGATGTCTCCACGGATTGAGAGCATCTCTGAC
AGCCTTGGCTTCCGGAGAATTTTCCCAGGCTCTCTTTTCTGCCTCTAGAACAGTCACCTTATCATAAAAGCCAG
TTTTGATCATGAACAACTCCATGGAACCTCCAATCATGGCGGAAACAACTGAAATTTTCAAATACCAATCCC
AGAATTTCATTGCAATCCTTCTAATACAGCTCCAATTCCTGCAAATCAAACAGAAT
>TCONS_00033338
ACCCTGGCTTTCTTTGGTGCGTAGAAAAGGTAGAAACCGACGTAGATAGTTTCAATGAAGCAGCCAAAGGAG
TTTATGGTGATGATAAGGGTCGTGTTTGTCTTGAGAAATGCATAATAAATCCAAAGCATGGAACTAAAGAGA
GCAACCACATATGGAATCGATTGATAGCCTTCAGTTGATTTTTTCTTGTAAATTTTATAAAATGTGGGCAGTGG
AGAAAGGAACACAATGAACGAGACGATGTTACCAAGGACACCAAAAGCAAAAGCCCAGTGACCAGAAAT
>TCONS_00033367
TGCTTGTTGGGTATTTTGGTTTGACACTGCTTCACACCCCCCAAAAAAAAGAAGGGAGCTACGTCTGAGTTAA
ACTTGGAGATGGAAGTCTTCTTTCCTTTCTCGACGGTGAAGTAAGACCAAGCTCATGAGCTTATTATCCTAGGT
CGGAACAAGTTGATAGGACCCCTTTTTTTACGTCCCCATGGAGCTACGTCTGAGTTAAACTTGGAGATGGAAG
TCTTCTTTCCTTTCTCGACGGTGAAGTAAGACCAAGCTCATGAGCTTATTATCCTAGGTCGGAACAAGTTGATA
GGACCCCCTTTTTTACGTCCCCATGTTCCCCCCGTGTGGCGACATGGGGGCGAAAAAAGGAAAGAGATGGAT
GGGGTTTCTCTCGCTTTTGGCATAGCGGGCCCCCAGTGGGAGGCTCGC
>TCONS_00033368
TGCTTGTTGGGTATTTTGGTTTGACACTGCTTCACACCCCCCAAAAAAAAGAAGGGAGCTACGTCTGAGTTAA
ACTTGGAGATGGAAGTCTTCTTTCCTTTCTCGACGGTGAAGTAAGACCAAGCTCATGAGCTTATTATCCTAGGT

CGGAACAAGTTGATAGGACCCCCCCTTTTTTACGTCCCCATGTTCCCCCCGTGTGGCGACATGGGGGCGAAAA
AAGGAAAGAGATGGATGGGGTTTCTCTCGCTTTTGGCATAGCGGGCCCCCAGTGGGAGGCTCGC
>TCONS_00033372
GGCAGGTTTAGCGGGGGCGGCAGCAAGCTTGAAAGAGCCCTTGATTTTAGAGAGCTTTCCAGAAGCAACAA
GCTTCTTCAACTGAACTAAAAGTAATTTCCTGAAATTTGAAGGCAAATCCTTTTGCTTTTCCTCAATGAATTTC
GCTATCGCAACTTGACTTGAACCAGTTCTCTCCTTTAACGCCGCTATTGCTTCTGATATCATCTCAGCATAAGG
AGGATGAGATCTGGGTTTCTTAGGAGCAGAAGTTTTCTTTGCAGCTGTAGACGCCATTGTAGAA
>TCONS_00033447
ATGACGAGCGCAAGAATGTTTAAATGAGTAAGGAGAAATTCTTTCCTGCGAGTAACTCCCACCGTCGGTATG
AAGCAGCTTCCCTGCATCTTCTTCCATATGCACCCTGGTAACGCCAAACTTTCTATGGCCACCACCAAACTCA
ACTGGAAGATCCACATCAAGATATCCGCCACTCGCAATTGGGATATCAAACTGTGATATTTGATACCCTTTTG
GAAGGTCAGGATAAAAATACTGCTTCCTATCAAACTTGGAACTCAAAGACAATTTACAATTAAGTGCAAGGC
CAACTCTTACTGCACACTCAATCACCTTTGAGTTCAAAACCGGCAATGCACCAGGCAAACCCATACAAACAG
GACAAACACTGGTATTTGGTGGAGAACCATAGTTGTAAGGGCAGCTACAAAAGGCCTTAGTAAGAGTAGAA
AGTTGAACATGTGTTTCTATGCCTATAACTGCCTCATAATCTTTCAGAATCTTATCAACTGTTTTTGATTGGTTT
TGTGTGGAAACTTTCAACTTGGGCTGTTCTTTTTCTTGGGTTGCAGTTTGTGTTTGTGCACTTCTCATACAACAA
TACAAAACACCATGTTTTCTTGCAAAATATGAAGATGGGTATAACATAGAAGGGTTAAGCTGTATCCCTCTAA
GGAATGTCAAAGCCATCTTACCAATAAACACACAAAATTATCTTACCAATAAACACACAAAATTATGCCCAA
GTTTTATTTTTTGGTCAAAAAGCTTCAGTGACTGATGAGAGTCACATATACGACATCTTTTGAGGAGGGTCCA
AGATTTTACCCTGTGAAGCTGTGAGATATT
>TCONS_00033488
CAGAGGCTAGTAATGTAGAAACAAAGGTCCTGTTCCTTCTTCTTTGTCAGCATCAGGGATGCTCCTATAGATA
CACAAGTTGTTAGACATTTCCCAGCTCCAATCCTCCTTCCTTGTAACATACATAGGTGGCCAACGGTTAATCCT
CACATAGTGTTGTCGTATTTTCCCATCCGCAAGCAAGCTAGTTACTATGGAGTAGCAAGACTCATGACCACCC
CATACCTTGTCATCTGGTTCAGCAGTCTGACTTCCATCTGGAAGGAAATAACGGCGTAAAGGAGGTCCTGTTC
CGTTCCAGTAGGGGTCGAGCATTCGCCAATATTCCGGAGCATCCTCGGTGAAATGGAACTCCCACGCATGAT
CACATAAATCCTCCTTTGTTACTCGCTTACGTTCACCATCCATTACCGACAATGAGTAAGCAGCCAACTTTGA
AAATCTAATCATTTTTGCTACCCGAGGTAAATGTGCTTTGTCATGCCATAATTCTTCACACTTTGAGCTCCAAA
TCTTATCAGAAGAAGCAACAGCACACCAACCACGTGACACAAGGAGAGAAAGTGCCACGCTGCGCGCGTCA
AGACAGCTTAAAATCGTCAACATTATATCTGAACCAAGTAAAATTAGTGGATCTCTCATCAAATTATCACCTT
TTTCTTCACTAATTATTCTCTGCTGATTCCTCTTCTCCTTCTTCTTTTTCACTTTTTCTTCAACTCCATCCTCAAAA
ATTCCACCTTTATCATCAGAAGAGCATAACCCATCTTCAAAA
>TCONS_00033489
ATTTTGAGCAGTGGAAAGAAATGACGAAAGAAAGTAACCATTTTAATTTCTTACCAACCACTTGCAAAGCTA
GATCATAAAAAATTGGACGTTTGGACAGCAGTTGCACAGATTTAAGTGTTCTCAAGCACGTACAAGTTCTACT
AACTCATTTGGTGCGAGCTGCAGCCATGCATGACATATATGGGGTTGGATAATGCCTCGGTGAAATGGAACT
CCCACGCATGATCACATAAATCCTCCTTTGTTACTCGCTTACGTTCACCATCCATTACCGACAATGAGTAAGC
AGCCAACTTTGAAAATCTAATCATTTTTGCTACCCGAGGTAAATGTGCTTTGTCAT
>TCONS_00033643
AAAAAATAAAAACGCTTTGTGCAGATGCTCATCATTTGCCATTTTTCTTAGATGAACTGATACAGCGACAAAC
TCCGCGTAATTTAAATATCCATCTCTATCAACATCAGCGGCTTCCATGAGAATCTGAAGATCAGCATCAGGAA

TCTGATGGCCCAGCTTTTGAAGACCAATTTTAAGTTCTCCAAGGTTAATCTTGCCTTTCTTTCCTGTATCCATCT
GGTCAAATGCTTCCTTCATTCCAGCCACTTCCTCTGCTGACAAAAACTCCGCGAT
>TCONS_00033754
TTTAGCACTGCATATCCTTCCTGTTGCACTAAAATGAATTTCCATCAACTTTCCAAATCGACTAGAGTTGTTGT
TCTTAGAAGTTTTGGCATTCCCAAAGGCCTCCAGTATGTAGCTTGTTTGCAGCACCTCGCTCTCTATTCCACTA
CGGCCTCCACCAATCATAGCCAAGTATTCCATTGCAATCTTTGCTGTTTCCGTCTTCCCAGATCCACTTTCCC
>TCONS_00033875
TCATCTCCTCAAGAATCCCATTGCCTGGTAGACATTATTGACAGTAACATCTGCTACATCTGCTGCTTTTCTGG
CTCCCTCTGCCAAGACTCCATCCAAGTAACTTGTGTCTGATACAATTTCCTCATAGCGTACCTGGATAGGGTGT
AGATGATCAACTAAAGCATCTGTAAGCACGAGTTTGAACGTTCCCCAATTCATATCTCTGCATTCTTCTGCAA
CCTCCTGCTTGGTCTTGCCTGTAACTAGTTGATAAATAGAAAGAAGATTGTTACATTCAGGTCTTTCCGGATTG
TCAAACTCCATGCCAGGAAACGAATCAGTCTTGCACCGTTTTATTTTATTTGCTATTACATCTTTTGAATCTAG
CAAATTGATTCTCGACTGATCAGATGGTGCTGACTTCGACATCTTGGAAAGACCATCAGTAAGGGACATGATT
CGAGCCCCAGCGGGTGGAATAAGGGGTTCAGGAACCTTGAAGATTGAACCGCCTCTCCCTCCCAGTTTCTTCC
ACTTCCTTCCTCCATATAAATAGTTAACACGCTCAGCCAGCTCTCGTGTCAACTCCAGGTGTTGCTTCTGATCC
TCTCCGACAGGGACAAAATCAGACTGCCGAAGGATACTTTGATACATCAGATCCACACATATTCACACACTT
TCAAGATTAGAGTTGGCGGAAAATTGTGCTGGGAACTGCAGTGGCATTTGAGCAAGCTGGTACAGAAGAATA
TCAGCAGCCATTAGCACAGGATAGGTTAGAAGGGCAACACCCACATTTTCATCCCCCGCCTTCCGTGATTTCT
CTTTAAATTGAATCATCCTGTTGAGCCAACCAATAGGTGTTGCAGAACTCAAAAGCCACATCAACTCTACATG
AGCACGGACATGAGACTGCACAAAGACTGAAGCCTTAGAAGGATCCACACCACATGCTAAATAAATAGCAG
CTGTATCCCTTGTTGACCTTGGCAATTGTTGTGCTTCATATGGCAAAGTTATCGCATGAAGGTCCACAATGAA
AAAGAATGTCTCATATGTATCCTGCAAGCGTATCCAGTTCTTTATGGCACCAAGATAATTTCCCAGATGTATC
AATCCTGTAGGCTGAACACCAGAGACTATTCTCTTCTTTACAGGGCTTGAAGAAGACTCAGAATTAACAGGTT
CAGAGACAGCTACATTGCAAAGGCACCGGAAATCTCGACCGGCTTGTAAATTACGTTGACGAATTAGCTGTG
ATCTTCTCAAGTATTTACCTGAACTTAATCCGCGTAACCTGGTGGAAGAGGCGAAGCGAGGAGCAGAATAAG
TGACCATACTGAAATGAGAGAGTAGAGAACGGCCCATTGCTGCTGCTTTTTTAAGGTCTGCGAGTTGGAACG
CAGTTTGGATGAGATGGTG
>TCONS_00033876
TCATCTCCTCAAGAATCCCATTGCCTGGTAGACATTATTGACAGTAACATCTGCTACATCTGCTGCTTTTCTGG
CTCCCTCTGCCAAGACTCCATCCAAGTAACTTGTGTCTGATACAATTTCCTCATAGCGTACCTGGATAGGGTGT
AGATGATCAACTAAAGCATCTGTAAGCACGAGTTTGAACGTTCCCCAATTCATATCTCTGCATTCTTCTGCAA
CCTCCTGCTTGGTCTTGCCTGTAACTAGTTGATAAATAGAAAGAAGATTGTTACATTCAGGTCTTTCCGGATTG
TCAAACTCCATGCCAGGAAACGAATCAGTCTTGCACCGTTTTATTTTATTTGCTATTACATCTTTTGAATCTAG
CAAATTGATTCTCGACTGATCAGATGGTGCTGACTTCGACATCTTGGAAAGACCATCAGTAAGGGACATGATT
CGAGCCCCAGCGGGTGGAATAAGGGGTTCAGGAACCTTGAAGATTGAACCGCCTCTCCCTCCCAGTTTCTTCC
ACTTCCTTCCTCCATATAAATAGTTAACACGCTCAGCCAGCTCTCGTGTCAACTCCAGGTGTTGCTTCTGATCC
TCTCCGACAGGGACAAAATCAGACTGGTACAGAAGAATATCAGCAGCCATTAGCACAGGATAGGTTAGAAG
GGCAACACCCACATTTTCATCCCCCGCCTTCCGTGATTTCTCTTTAAATTGAATCATCCTGTTGAGCCAACCAA
TAGGTGTTGCAGAACTCAAAAGCCACATCAACTCTACATGAGCACGGACATGAGACTGCACAAAGACTGAA
GCCTTAGAAGGATCCACACCACATGCTAAATAAATAGCAGCTGTATCCCTTGTTGACCTTGGCAATTGTTGTG
CTTCATATGGCAAAGTTATCGCATGAAGGTCCACAATGAAAAAGAATGTCTCATATGTATCCTGCAAGCGTAT
CCAGTTCTTTATGGCACCAAGATAATTTCCCAGATGTATCAATCCTGTAGGCTGAACACCAGAGACTATTCTC

TTCTTTACAGGGCTTGAAGAAGACTCAGAATTAACAGGTTCAGAGACAGCTACATTGCAAAGGCACCGGAAA
TCTCGACCGGCTTGTAAATTACGTTGACGAATTAGCTGTGATCTTCTCAAGTATTTACCTGAACTTAATCCGCG
TAACCTGGTGGAAGAGGCGAAGCGAGGAGCAGAATAAGTGACCATACTGAAATGAGAGAGTAGAGAACGG
CCCATTGCTGCTGCTTTTTTAAGGTCTGCGAGTTGGAACGCAGTTTGGATGAGATGGTG
>TCONS_00033877
TCATCTCCTCAAGAATCCCATTGCCTGGTAGACATTATTGACAGTAACATCTGCTACATCTGCTGCTTTTCTGG
CTCCCTCTGCCAAGACTCCATCCAAGTAACTTGTGTCTGATACAATTTCCTCATAGCGTACCTGGATAGGGTGT
AGATGATCAACTAAAGCATCTGTAAGCACGAGTTTGAACGTTCCCCAATTCATATCTCTGCATTCTTCTGCAA
CCTCCTGCTTGGTCTTGCCTGTAACTAGTTGATAAATAGAAAGAAGATTGTTACATTCAGGTCTTTCCGGATTG
TCAAACTCCATGCCAGGAAACGAATCAGTCTTGCACCGTTTTATTTTATTTGCTATTACATCTTTTGAATCTAG
CAAATTGATTCTCGACTGATCAGATGGTGCTGACTTCGACATCTTGGAAAGACCATCAGTAAGGGACATGATT
CGAGCCCCAGCGGGTGGAATAAGGGGTTCAGGAACCTTGAAGATTGAACCGCCTCTCCCTCCCAGTTTCTTCC
ACTTCCTTCCTCCATATAAATAGTTAACACGCTCAGCCAGCTCTCGTGTCAACTCCAGGTGTTGCTTCTGATCC
TCTCCGACAGGGACAAAATCAGACTGGTACAGAAGAATATCAGCAGCCATTAGCACAGGATAGGTTAGAAG
GGCAACACCCACATTTTCATCCCCCGCCTTCCGTGATTTCTCTTTAAATTGAATCATCCTGTTGAGCCAACCAA
TAGGTGTTGCAGAACTCAAAAGCCACATCAACTCTACATGAGCACGGACATGAGACTGCACAAAGACTGAA
GCCTTAGAAGGATCCACACCACATGCTAAATAAATAGCAGCTGTATCCCTTGTTGACCTTGGCAATTGTTGTG
CTTCATATGGCAAAGTTATCGCATGAAGGTCCACAATGAAAAAGAATGTCTCATATGTATCCTGCAAGCGTAT
CCAGTTCTTTATGGCACCAAGATAATTTCCCAGATGTATCAATCCTGTAGGCTGAACACCAGAGACTATTCTC
TTCTTTACAGGGCTTGAAGAAGACTCAGAATTAACAGGTTCAGAGACAGCTACATTGCAAAGGCACCGGAAA
TCTCGACCGGCTTGTAAATTACGTTGACGAATTAGCTGTGATCTTCTCAAGTATTTACCTGAACTTAATCCGCG
TAACTGAAATGAGAGAGTAGAGAACGGCCCATTGCTGCTGCTTTTTTAAGGTCTGCGAGTTGGAACGCAGTTT
GGATGAGATGGTG
>TCONS_00034064
AGAAGTGTTCTTATATAATTGTTTCAACTTCATCTAGGTTCACAGCAACTCCATTTTGTGTGTCTCCTGACCTCA
ACTGGTTGGAAATGTCATCAATAGCGTCATTCAGCTGGACAATCTTCTCCGTTAGTATCTTGCGCTGTTTCTCC
AATGCTTTTTCTTCATCATATATAAATTTGGGCAATTTTCTCATGAGGTCCTTCTTATCTGTTTCAGCTAATGCCT
TGCTAATCTGAGGCGCATAGATACAACCAAGTGCACCAATGATTACTCCTCCTAATAGAAAGCCACCAACAA
AGATTCTGGTATTGCCTGGCCTTCCACCATCACTATATCCTGCTTGAACGGTCAATGAACTTCTAGATGAGGAT
GATTTTCCTCTACTCCTTGGGCTGAAGGCCAACTTGGTGGTGCCACTGACACTTTGGTCTGCTGACTTCAAACT
AGAGCCTAGCACA
>TCONS_00034099
GTAAATACGAGCAATTGCGATATACTAATCTCTCAAGTGATTCTTTAAGTCTCATGCGGACCAGATTAAATAG
TGTGTGACACGTCGCACGAAATCTTTCCCCTAACAGTAGCTTCTAGAAACGAGGATACTACCCAGTTTTTATA
CCTCTCCCAATTCCGTCTGCTCAATTCTCGCTGGTTTTTGTCCGTCACCGATTCTCCCGAGGTATGAAAAATGG
CAGGTTCTCATATTGCCCATGCTGCATACAAGGGTCCAAGTGTGGTGAAGGAAATACTGATTGGGATTGCACT
TGGCATCGCCGCGGGAGGCTTGTGGAAGATGCACCACTGGAATAACCAGAGGAGGACCAAGGAATTTTATG
ATATGCTTGAGAAAGGTGAGATCAGTGTTGTGGTGGATGAAGAGTAGCGCCTTTCCTCCAGTATGAAACCAG
CAAGCATACTTGGTCTAAGATAGTAATGTAAAACAAAACTCCTACAGTTCCTTGCTTTACTAGTAACTAGTAA
TGTAAAACAAAACTCTTGCTTTACTAGTAATCCTTGAATATTCCTACGAATGTCCTTAACTTGAGAAATGTGAT
GTTTGGGAATTTTGTATCCATACCATGTATATGCTGGTGCTGTGTTGAGGAGATTTGTGTCTTGTTTGTTCAATG

GCTGTTCAGTGTTTAGTCCTTTCAAGCTGGAAAATTGTGTTGATATGCCTGGAAATTTTGTGAATGGACTATAT
AGCTATGTGAA
>TCONS_00034176
GCAAGTTGAAGTGGAAACCCTAGAAAAAGGTTTCTTGGTAAATGTATATTCAGAAAGAAGTTGTCCCGGCTT
GCTTGTTTCTATATTAGAAGTTGTTGAGGAGCTTGGCCTTAATGTGCTCGAAGCTAGGGTTTCATGCACCGACA
CCTTTCGTTTAGAAGCTTTTAGCGGAGAGAATGAGGAGAATGAAGAAACCATCAATGCCCAAGTTGTGACAG
AAGCAGTTTTTGAAGCTATTAAGAACTGGAGGGAAAGCAACGAGCAAGGCTAATTAATCAATCAATGTAGCT
TAATTAGCTA
>TCONS_00034239
CCCAACTTTACGCTATTTCTCCCTTCAGTGACCTCTATAGTTCTAGAAGAATTCTTCACGAAGATCAAGGTTTG
TTCGCAGAGAATTAACTCCAGCTAAAGGATTTGCAGTCAATATACAACATTGTGATTTGAAGATAGTCTAACA
AAATCTGTAGTTCATCGGGTGAACTATGTCGTAGGCATAAGGCAGTCTGGAAAGTCAGTGTTTAGTTCTTCCA
AGAACGAGCCAATATTGATAATTAATACTTGAAGTCTAAAATATATGTACATAATCCTATCAGAACTAATAT
ACAAAATATCATGATATATTCTAAACGTGGAATGGTTTCCGTGG
>TCONS_00034326
AATTTTTTGGGCCCTTTTCCCTTTTTTCACCTCTTCTAGGTGTAAGTCTTGATTAAATTATCTGAATCTAAGTGTT
CCTTCCATTTTCTTACCCCAAAAAAGAAAAAAAGGTTTCGTCTTTGAGGAATTTCAGCCAAAGGGGTACAAG
AATTTATAGGAATTTCTAAGGATTTAATTATTTTATTAAGTCTGACATAATATAATGGATTCAGATTCTTGGAC
TCGTCTATTTACGTCTTCAAGGCGTTACCAATCTCGATCAGGTTTTCGTTGCTCGGAAGTCAACCTGGTGTAAC
GATTACAAGCTTTTGTTTTTCCAGCTTTTTCGTTTAATGAGCTGAGGTTTAGTGAATAGGTTTCTCTACCTGAAA
AGCATGGAAGAA
>TCONS_00034333
ATATATATAGTATATGTTAAAAGGTCCAAAATTAAACAGGAACTGTCCTTCCACTATTCTTCATTTCTGTTCCA
CTTTCCTCTTCTCCTTCATTTTCCCTTGACATTTCTTCCAATGATTTTCCAGCTGCTGCTGATATTCCATGGCATG
TTGACCTAAGCCTGGCTGGGAAAATCTCCGCGGGGACAACAAATGTTGTGGCGTTTGGACCAAAATTCGCGA
AGAAAAAGGTAAGCGAGTACATGATCACGAACCCGATTCTGTTATCCTTTAGAGTCCAATGATGATAAGGAA
TGGCTAAGGCAAACATGAACACTGTCATGAAGAAGAATCCCATCAACTGAATTGCAAATCGACCTATTTTGT
CGATGAAGAATACTGTGAACCAGTAACCAGGAACAGTACTGCAAAGGGCGATAAGAGTTTGCGCCCTTGCA
ATTTTGTAAACTTCCTCCAACGCATTCATGGTTTGCGCTGGTGGAATCCATCCAATCGCGCTGAAAATGTCCTT
TTGGAAAAGGTTTTGGCTGTAGAATGCAATGTCCAACAAGAACCATGTGCTAGCAGTTCCAAGCAAGTGAAG
TCCATGGCGACGGAGGAACTCCTTAGAAAACAAACCAAACTCATTCCCTGTGTTTTGAGAAACATTCTCAACT
TTCTCTTGCTCTTCTTCAATATCGACTTGCAACACTTTAGACATGTCGTTTGCTGCCTGTTTTAAGTTCTTCGCCA
CCAATGCAGTGTAACGAGCAGTTTCAGGCATCTTCATACGCCAGTAATAAGTCATTGCAGCTGGGATTGCACC
AAACATAAGAATTATACGCCACACGAAATCAGCCTGAGAGACAGTTGAAGCAAGAGGGCCGTCCTGATATG
TTGGTGCAGGGAACGCGCCCTTAAACGCAGCAGAAACAATGATTGCCACCATTCCACCAGCCAAAATTCCAA
AACCTTGCATTGCAAAAACAGCAGCAATGAACGCTCCACGCGTCTTTTTATTAGCATACTCAGACATGATAGT
AGCAGAAAGAGGATAATCACCACCAATGCCAAAACCTAACCAAAACCTGAAGAAACAAAGTGTAGTCATA
ACACTTTTTGGTGTATGACCAAAGGAAAGCCCTGAGGCAATTGAACAAATAACCATCATCATAAGGGTCATT
CCATAAACTTTTTTTCTCCCCATTTTATCTCCGAGCCACCCGAAAAACAGTTGACCAGCAAGGGTCCCACAGA
AGGCGACTCCATTCACAGCAGCCGAAACGTTAGGAGGGAGAGATCCAGGTTTCAATGCCCCGTCGCGGTGGT
AGTAAATGCGGCCAAGCAATTTTGTGACCAAAGAAATGCAGAAAAGATCATAAGCATCAGTAAAGAAGCCC

ATGCCAGCAATAACAATTGCTGTGAAGTGATACAATTGTGTTTTTGCTACATCTAGTGCATTTAGCACTTGCA
ATTGATCTTTAGCCATGACTATACTTTCTCA
>TCONS_00034359
CTTGTATACGTTCCAAAAACGCAGGGGACTCAATTCCAACCTTTGTTGGTGATGGTCTAATAATTAACTTGCCT
TGATAACAAGAAGTGCAAGAAAATTCATTATTTAAAAGAATCTTTAAATTCTTTAATGGATGCCCATTTGAGT
TTTCTATAATCCGTCTCATCATAATTGATCCAGGATGTCCCAATCGATCATGCCAAAGTACAAAAGTATTGGA
ATCAGTAACCTTTTGGTTTACGGTAGAATGTGCCTCAATTGCACTAATTCTTGTCCAATATAGGCCACAAGAT
AAAGATGGGAATTTCTCAA
>TCONS_00034375
CATCAGGCACAAATGAATAAGTCTCTCTATCCAAATTAGACTCAAGAAGTAAAGGTTGCCTCCTTTCAACAA
GATTTAAGGAGGCCTTATCTTTTGCAATTACATCTAGATTTTCTCGAATGGCCTTGAGACGCTTACTTATCTTA
ACATTATAAACCACAGGATTTGACTTTGAGAAGAAGATGCTTACCTTACTCACCAATTTTCGATGTGTCACTT
CAGTAGCAAAGTCATCCAAGAGATCATCAGCCTCAAAGAAAACTTTTTTAAGTTTCCTTATCCAATCCCTCAC
CTCGTGGCTACTCTGCTGCTGCTGCTCCGCGT
>TCONS_00034376
AAGCATATCTTTAGATGGTGAGGAAGGTGATTGTAGCTCAATCTCAAAATGGCCATGACACTGTCGCCTCCTC
GTGTGATACTTGACAAATCTTGATTCTTGAAATATATCCATTCATCCTCCGTTCTTTTTAGGCGTAGTAGGCTTC
CTACCGACCTTATTGCAAGAGGAACTCCTCCACACTTTTTCACAATTTCCTTTCCTATTTCCACCAAACTCGAA
TTTTCACTCTCTTTGTTACA
>TCONS_00034486
TTTTAACATTAGCCACACAAGGATGGGCCAAAATCCATATCGAACAAAATTTGATTTGCAGTCAAACGCACG
CATAGAACATGTCCAGACACTAACAATATAAAGATACTAAAGCCAATGTGCTCATTCATCCTCCTGGCTGCG
CAGCCTAGCAAGCTTGTTCGTCACAACCACATCAAGTTGTGCTGCTCGCTCAACAATTTCTTTCCTCTTCCTTG
TGGACACATTATGTGCAATCTCTGCACAGTAAATTCGGTTGTGCATCATTAAGATCTCAAGTTCACTCGCATTG
TGCACAACAAACTTCTTGAAGCCATTGGGAAGATAGTGACGTGTCTTCTTGTCAGACCCATATCCAATATTGG
GCATCAGCACACATCCCTTAAACTTTCTTCTAACGCGAGAATCAATACCTTTTGGTCTGCGCCAGCTTTCCTTA
ACAGTGATTCTGCGGTCGCTCTGGGGTCTAATGAACTTCTTGACCCTCTTCTTCACAACCTTCTTGTTCAGCAA
TGGAACCGCCATTCTTACCTTACCTTCTGCAATTCAAC
>TCONS_00034545
GAGAGAGTACCAGCGTTGCGATCGGAAGTGCCAGATTAGGTGCGGACCAGATGGCAAAATTGATGCAAACC
TGGGCTGAAAGTGCCCAAAAGTAGCAAGCAACTGAACCGAGGCTATTATTGTAAGGAGTTGCACAATTTTTG
TGTAATTCACAATAAAATCTTCATAGTTTGAGAAAATAAACCTTAATTATTACGTGTATGTTTAATTAATATGA
AATTTTATTGTTGTATGACATGATGGTGAAACGTGAT
>TCONS_00034573
CTGAACATTAAGAAAAATAGCAAGATCCATGTGTACAATTCAGCTGCCATTCTCAAAACAACTCACGAAGTG
AGACTTACAGTTCCAAATAGTGCAACTGCCGATCTGCTATGCTGCATTCCTTTTATCATTTTGCTAGGATCCCT
CATATAACCAGCCACTTGTTGACTAATGTCCTGATTAAATGCTTGCAATTTGCCTTTGATTGCGGCAGCACCAG
AGGTCACCTGGGTCTTTCTCTGCCATTTATCTATCGATTTGTCCCTAAAAGCAGCCATCCTTGAATGCATTTGA
GAAATCTTCTGCCAATCTTCATCAACTTCACCATTTGACTCAACAGAACCTTCCGAAAGCTTCAACCTTTTACC
AGAATTAACGTCCATTGATTGAGTAATTGATGGATTATTCTCAAGTAGTACCTCTTGCAATTCCAGTATAGAAT
CCAAGGTTTTTCTGGAGGAGGCAAGTAGATCTGAATATGCATCTTTAACTCTGTCCTCAGAATCACAAAAAGA
AGATCTTATTGGTTCCTGCGGAAGTCTATTGGAGTTTGAGAAAGCCTTTTGCAGCAAGAACCTTAGTTCAAGT

GTCTTGTCCCAGAGTGCCTTCTGGTTTCTAACTGCTTGGCCTTTGAGAAGATCCTCATCCTTATGGCGCTTCAG
ATTGCCCAAAAGATCTTGTTCCTTGTGCTTGAGCTCCATATATTCTTTCTTAAGTTCTTCGATTCCAGCAATTTT
ATGGTCCTCAGTCAGTTTCTCTCGGTCTTCTTCTTCTTCTT
>TCONS_00034574
CAATTTTTGGGGCCTAATGCAAGACATTTGTTGGCCTATGGGTAGAGCCGCCTGTGAACAAATTCTCAAACAA
TTTGGGGGCCATTGGTGGAAGATTTACAGGCTGAGGAGCCATGAAATTGACAATCTTTTCATGAATATTGTAC
CGTATTTTACGACTCTTTGATGCACGGCGATCAACAATTTTTCGTTTCTTCGTTTGCAATCTCTTCAATGCATAG
AATGCCGTCTC
>TCONS_00034582
ATCACGTTTCACCATCATGTCATACAACAATAAAATTTCATATTAATTAAACATACACGTAATAATTAAGGTT
TATTTTCTCAAACTATGAAGATTTTATTGTGAATTACACAAAAATTGTGCAACTCCTTACAATAATAGCCTCGG
TTCAGTTGCTTGCTACTTTTGGGCACTTTCAGCCCAGGTTTGCATCAATTTTGCCATCTGGTCCGCACCTAATCT
GGCACTTCCGA
>TCONS_00034598
GTTTTACAGAACCCCTTGCCATACCAACAATATTATGTAAGAGTCTTGCATATTCTCAAGATGAGTTTCGTAA
AAATACGTATAAGAAGATGAGTGTGCAGCCATAATAATTAGACAAATAAAAAATGATCAAATTACATAAGG
TTTGAGTAGTACACTTAGAGGACAGATGAGAACATCTTTAGAGATGGTTTTAGCATGTCATTCTCAGGCTAAA
TGCTGATTCTATGATGATTGAGAGTGTTGAAAGTCAGTAGAATCAATAAAATTATATATTTAAATATGTCTAA
GTTCATGGAGTGACTCAAGAAAATTGTACAGGGTATTCTAGAATCATTTGTATTTAATTGTATGATATTACCTG
CCTTGTTAGATGTAGAAGATAACTATTTATCATATTGTAATTACATGAGGAGGTCTTCTTGTACATTGTTTTTAC
ACTCAAGACAAGGCAAGTTCTCTCATTTGCTTGCTTTTTCACATGATATCAAAGTGTCCTGATCTTGGTAGAAA
CCTGAGTAGCTTTCGTCTACCAGCGCTGACCTTTTGTCGCCTGCCCAGTTAAAGTTTTCTTCTTTTCTGTCTTCTT
TTAGTGACCTTTTAGGGGCAAAACTCTTCTTTGCTGTGACCATTTCGATACTTGCACTTCCGCTTCCTTTTCGAT
GACCGCCCAATGACCCTCGCCTCTTTTCTAGTGATTATCTCGTTTGACCGCGGCTCTTTCAGTTGGCTGCTCTGG
AGGCATCCTTTATGACATGATTTTATAGCCACAAAAGTGTTTTGACATGTTTAAGACCACAAGTTATAGTCTTT
TTCCCCCTTTAAACTCCTGCATAGTCAAACACGTTCACATGAACTGAAATGAAGGTAGCATAACTGAACCCA
ACTAGTTCTGTGATAGAGCTTGTCGGTGTTGATATATTTGCAATTGGCACACGGGGTGACTGTTCTTCCTTTCCT
TCCAATGTGAAACTCTTTCATTCCACTTGATACCCTCAACTGGTCGCAAACATTCAAGGAAAAGCAACATTAA
GTGTTTCTATCTAAGA
>TCONS_00034780
CATTATCTGTCTTCTTGTTGTTTCACAAGAATATTCCAATTAAACAAATGGAGCACAAGGCGTCCTTCCTACGG
TTATTCCTGCTTTTTCTGGGCTTTTCCCTCTTGATCTCCACCGTTGCAGTTCCTTACTCCAGAAGCCTCAAGACA
ATTACAGGAAATCCAACATTTCAAGAATCTCATGACAAGGTACTGAATAAAGAGGTGGAAAAGAAGAGGGA
GCTATTGGTTGAGAGAGATGACTATGATGGAACAGGTGCTAATAATCGTCATGACCCATCGCCTCCTGGAGG
CGACTAGTGAAAATTGCAGAAATACATTATTTAAATGACGACTGTATTTTCCTTTATTTTTAATGTTAAGAAA
ATAAAAT
>TCONS_00034806
ATTAAAAAATAAAGGAAAATACAGTCGTCATTTAAATAATGTATTTCTGCAATTTTCACTAGTCGCCTCCAGG
AGGCGATGGGTCATGACGATTATTAGCACCTGTTCCATCATAGTCATCTCTCTCAACCAATAGCTCCCTCTTCT
TTTCCACCTCTTTATTCAGTACCTTGTCATGAGATTCTTGAAATGTTGGATTTCCTGTAATTGTCTTGAGGCTTCT
GGAGTAAGGAACTGCAACGGTGGAGATCAAGAGGGAAAAGCCCAGAAAAAGCAGGAATA
>TCONS_00034833

TAACAATCAAGGTTAGTCCGTACCAAATGCTTTCTAAATTCATAAATGTTTGGTATTCTTTTAAAGAACACTTA
GTTTGTAAAGAACAAATGCAAATTGTTTAGGTGAAAGCCAGGTACTTGTATTTGTACAGTAGAGTGCCAGGAT
TCTTCATTTAAATTAAGTTTAGCTGACAATCTATTTGTATATGGACATGTGCTTGATTGTTTGGTCATTTG
>TCONS_00034960
CAGGAGAGATGTTGAAACCATGTTGCCTGAGCAATCGAAATTGAAGTGAAGAAGTGCTCAAATCATCGCAGT
TTGAGGTTTGGTTGTAAATTTGATCCAAAATCTCATCAATTTCTTTCTCAAAGTGGTATGATATGCCAAGGCGT
TCAATAATGTCAATCAAATTCAACGTATCAGACAACTTTCTTCCAGTTGCTAATAGCATACTCCTCGTTTGTTC
CTTCAATGCTTCAATCTCTTGAGCATACTTTTCAGCAACCTGATTATCAATGGAGAATGAAAGGAAATGATCA
CCCCAGAGACTAGGGGAGAAGTCGGCGACGGGACGAACAATCTCTTCT
>TCONS_00034963
ACTTATTTCTAAAATGCCACAACTTCAAGTAATATACAACAAACTACCAATTAAAGTACAAATGAAGTCCCA
AATGAGGGACATTAACAGGGCTAATTGGAAAAATTGATCTCTATAACCCACTTCACCAGTTTTTTACATGCC
TCGTGTAGATAGTAGACTCTGGTGAACACCAGCCAGTCCAATGGAAACACTAGCGATCAATGAAAGATTTGT
TCATAAGATTAACTGCTGAATCCATAAACTTCCCAACCGACTTAAGTTCTCGTCCAATAGGTTCCGTAAACTT
CAGGTCGACTTCAATGGGCCATACCTTAAGCCCAAGTAATTTAATAGGTGCAATCTGTCCTGGCACAATGCA
ATCTGGACCTGCAGCTTCTACAATAGCCTCCGTCCCCAATCCACTTCCAATTGGATCTGGCTCCTTCATAACAG
AGAGAGCATAACTGGAATAAGGGTTTTGAGGGATACGAAGAATAGGGGAGTAATGAATTTTGGGGATTGGC
TTTGAACCAGAAGCAGCTCGCCATGGTACATCATTCGGTAGTGTAGCTGCAGCTCCTCCACCCATTTTTCTTGA
CAT
>TCONS_00035115
AGTTGACAAAGACCCATTTGCAATACATGCCTTCTTTGCAGCAGATTTAATCACTGGAATATTGAGGACATCG
CAAAAATCCAGTAGCTTCTCCTTAACACACTTGTCCAGCCTCTCTTTGACCTTCCCCCTTTGTTTTTCCTCATCT
TCAACCCAAACAAATCCTGAAAACTGCCCTATGTTTTTCTTCAAACTATGTGCCTTTGTTTTCTTGCCATAAAG
AATATTGTGTAGTATCTGCAGGTTGTCATCAGGTTTTCTCTTGGATAGTTTATAAGCCACATTCGGGATGTCCTT
CAGCTGAGTACCACTACCCTTCCTAATTGACAATGGCTTAGGCGTCGAACTTCTAGCACCTGAAGACTCGGAG
TAACGCTCCACAGTTTTCCGTTCCCTTGTCGGCCTTGAGCTTGGTGTCAGTGGCTCTTCCGTACTCTCCTTATTT
TTTTCCTCACTTTTGCTACTACTTTTCTCTTTGCCCTTCTTCACTGGTGTTTTCACTTGCTTTTCCTCCTCCTCTGAT
TCCTCTTTTTCCTCCTCCTGCTCTGATTCGTCTTCTTCCGCTTCTTTTTTGTCTACTTCCTCTTTGTCTTCTTCTTTCT
CGTCCTCATCTTCGGCTTTAATTTCATCTTTTTTCTCCTCCTCCCCTGCTTCGGTTGACTTTTTCTCTTCTTCTTGTT
CTTGTAAGGAAGATTTCTCCTCCGTCACCTCTTCTGGTTTCTGATCTTTCTCTGACGCCATAGACGAAACAATA
CTACTGAGCAGCAGTGAACGAGTGGTAAAACCCTAGTTTGGGGTTTTTGGTTTTGGGGATTG
>TCONS_00035116
CCTCCTCCATGTTTTTCTGCATCATCACCACCTTCAGCTTCATCCTCTTCTTCCTCATCACTCATGTTATTTATTA
CTTCAGTGATTATTGCCTTCACCTCTGCTTTTCTGTGCATCAAATCCACATCAAAGTGGCTACCTAGTTTCCTGA
TAATATCAGATAAAGTTGCAGTGTTAAAATCAACTTCTTGAAGAATATTTGCTACTACTGCATGCAATTCTTCT
TTGGTGGGCTCTGCTTTGGCCTTCTTTACACTTTTCCCTTTTCCTTCCTTTTGAGAAACCTTTGCAGTAGACTTGC
CTGATTGT
>TCONS_00035117
CCATAGTACATCTCCCCACTGTTCTTTCTAAAATACATATAAGATCCTGTCTACATTTCTCCACTTTAGTAACA
GTATATATCAGCAGGCTCTGAATTTCTTCTGGGGATCAATGAAATAATAATAACACAAAGTAAAACATAACC
AATTACTCAATAACTCAACATTAAATATATCAAATTTCATGGCAAATTGAAAGCTGCAATATGGCTGCTTAGA
TCTCCATGAGTTCTTCAAATGTCTTCATAGCAGAAGCTGGTAATCCAAGAAGCACATTGATGTTTCTCCTCTCT

TTCCCGTGAGAAAGAAACAGAGAAACTTCCTTCTCTGGCAAAGCCACAGGTCCCGACACAACTGGCTCTCCC
CACCCAAAATCTGTAGTGTGGAAAGAAAGCCTAGACCAAGTAGTAATAAGAAGAGTAGCAGTTAAAGATGG
CCTAACTCTTGTTGCTTCAAAATAGTCTATGGCAGATCTCATGTAACTATCTGTTACCATTTTAACAGCTTCTTG
AACCAACTTCACAGCAACAGAAAGTGGATTCTCAACTATCTCTGCAGCATTACAGAGTGCATTAGTTAACAC
GATCCCGTTTCCAAAATATCCTTCTGTAATTGAGGGATCAAATCTTGATCTTCCATCTACAGCAAAGAGCAGC
TTTGTCTTTTGATCAGGTTTCATCTGTAATGCCTGAGTTCGCGCTTTCCAGACGAAAGCTGAAAGGGCTTCAAA
TGTAGTACACTTGCAAACTCATTGTGAGCATATTCAAATTTTGGTGGATTTCTAGCCTTGAGTATGCTCCTGTC
TAGGAATGGAGGGACTTTGATTGGTACACCTCTGGCAGTTTCACCCCAAGAATTCACAAATTCCATTGCTCCA
ATCCCATCAAACATGCAATGGTTCATGCACAAACCAAGCACAAATCCCCCACATTTGAACTTTGTCACCTGA
ATATAGAAAATTGTCACTCAGTATGT
>TCONS_00035153
CCTGCATCAGGGTTCTAACTTCATCTTTTGTAGGAGGATGAAAATGAGGTCCGGGCAAACGTTTATTAATATC
ATTGTAGACAAGTAACACAGCAATATAAAGGTCTTCAAACTTCAGATTTGCTCTACCTGATTCATTTTTGAAA
TGGTCAAAGACCTTGTCTGTTATTTTTCGTATTTTTGTCTCTCTCCACTGCTTACCTTGGAATTTTTGAAGAATCT
GTCCCATGGGTTTGAACTTTTGGAGAAGACAAAGGGAAGCTTGCTACGGTAGTGTGATTTTGGTCAACTTGCA
CAGGGAATAAATACTCCAGAAT
>TCONS_00035168
GCGTAATTTCTGAAAGTTATAGGATGACTTCTCTCTTTTTTTATCGATACTCTTTTGGAACTCATTATGAATTAA
ACGGCTGTAACATTCAAGTGTTACTTCAAATGAGGAAACTTCATTTCTTGTGCCCTCTTTTTTCTCCTTTTGGCT
CGCTAAGTGGATTGGGCAGCATACTTTCGGAGATTGTGGGATGCCTGACTGACAGAGAACGTAGTTGGGGAA
AATGCCTACATATTGTTGGATAATGTAGTTGTAGAGGGCACTGATTTTCCAAATGTTAGTGTCTTCTCAACATC
TAACAGAGGTTAGCTAATTTTTGGCTGTCCTGTTTAGGTTTTTAGCAATAACTTTCTGGCAGAAAGGGTGACGC
CTGTTTATTGTGTCATTCTGTTAGCTCCTTCATGGTAGATATGAGCAAATAAGTTTCTGCTCAAATATACTACA
GTATGAGTGAAACTTAGAGTGAGGATCAGAGCTGGCAAGTCCACAAGTTCTATTCTCTTTCCTCTGGGTATGG
TAGTGAAAAAGAGTGCAACAGTTGTTGCTTGTGTCTGTCTTGTTTTGGTATATGAGGTACATATATTTTTGGGG
TTATACTGCAGCAGCGGAGCTGGAAGTAGGAGTTGATGCTATCTAGATGGGTACAACATGTTTATTATATATT
TCATTCTCTCAACATTGTGCAGAAGATTCTTGGATTTTTCCAACTCGGTTGCTGCTTGCGTAGTTGGCTCCGAA
GTTCACTCTGGCTTCAGTTCAAGCCTTACCAGATAGCTGCCGGGGCTGCATATCTAGCATCAAAATTTCTGAA
CATGGATTTGGCTTCACATCATTCTGTCTGGAAAGAGTTTCAAACACCCCCGAATGTGCTTAGAGATGTTGCA
CAACAGTTGATGGAGCTCTTTTAGGAGACATGAGATACGGCAGTTAACATCAGCAAAACATAAAAAGTCAAT
ATCAACAATTTTTTGGCTCTTCACTTGGTAGATGTATTTGTAGATTCTACCATCGTGCATAGGCTTAAAGATCA
GTGTATAGATTTCACTGTGGAATTTCTTAGTTTGAAATTATCTGTGGAGTGCTTGTCGTCTATACCTTATAGTAA
AGAACTTCATTCTCCGTGTAAATTGTTTTTTTTCTCACTGCAAAAGTGTCCATTAATTATTGAGATCGTTCTCT
>TCONS_00035316
CCTGATCCCGGAGCTTGAATTCCATCACTAACTACCCTCTAGCATATTAGCGAATAGGGTGGTACTGTGTGAT
CCCTCGCTTTGGAGGCAACTTCCTCAAAATAAAAAGGACAAGTGAAGAAGGCAGTATCTCCACCAACAGGT
AATATATCAAATTCAAAATTGGATGATCCAAAACATCAAGATCTGCAGCTTTATTAAATGCATTGAAGCACA
TCATAACACATCTAATGAGGAAGCATGAAAAACATATTGTTGTGACATAGCCAACCTCCTGTAGCTTCTTGCG
TCTCCCTTTTGATTCTACTGGAAACCGCTGTAACATAAGGAAAAGCCTTCCACCATAAAGGAGAAACCCTAA
TGCTGCAAATAGGGATACACCTGCAAAGAACATCTTGGATAAGATCATGAGTACTCGAACAGGTTTCCACCA
TATTATCAGCCACAGTATAATCTGGATAGCATAAACCACTCCATTAATTGTGAAGAAACTAGGTCTAAGCCC
ATCCGTGGATACGGCACGTGCCTGGTAGTATATCTCAGCCCAGAATAACACTAGCAGGGCATAAGTTGTGAA

GAATGCAAGACTTGGCATATCAAGCAAAATATGTTGGACAATCTCGGGGTGAAATTTCTGAACATCCCGCCG
AAATGCAAAAACTAGCGA
>TCONS_00035356
CCTTCGTTGTCCCAAATCACCCCTGAACCATTTCCTTCAGGAATCTCGACTGCACCAGTAACATTAAGCGTAG
GTCTCAAGGTTACATCAAATATGTTGACAACACAGTAGGTATTCTTCTCAAAGAGCTCGACAACTCTTTCCTC
GGAAGGGAACAGTGCTGCGGAAGGAAAAGTAGAAGGTGTAACTTGTTCAACTGTGACAGACGGATCCCCCA
AAGCAAGTGCTTGAAGGATGATAACATGAATACATGAAAAAGCTTGTTAACAACATCCTGCGCGTGGCTGAA
GGAAAGCTTTTCCGTAGTTGTTCAACCAAAGGAGTGCCATTATCTCCACTACCCCTGGGAGTGGAAGCGGTA
GAAGGAGGAGGGGAAATGGAGTGCGGAGTAGAGGGATCAGCAGAGCAGGCTGAAGAAATGCTATCGAAGC
AG
>TCONS_00035439
AGGGGGTAAACTTAGAAGAAACAACATCCAAGAATAAATAAAGCAGAAGCTAGATACTCCATTAGCGGATA
TGAAGATATTTACTAATGGCCTTCAAATATCCATAGGGAAGGAAGATGATAGATCACTTTGTGAGGACATAA
ATAGCATAGACAATACCAGGTAGCCATCCAAAGAGAGTCAGCAAAACACAAATCCAGAATTCCGCCTGGCA
GCCAAACTTGAGGAAAACACCAAGGGGAGGCAATATGATTGCCAACAGAATGTCTATGAATGTCAGTGTGC
TGGTTCTCATTTTTCCCTTCTTTCT
>TCONS_00035451
CCCAACAATCAATTATTAACAGTACTATCTTTCACTTTTCTTCTTTTCTGAGGCTTCTCCTAAAGGATACTTTTG
CATCGGCTCGCTCTCGTTAGGTAAAATTATCGAGGCGAAATCAGCTCTTTTATGCATGGCGACTGTTGCTCATC
TTGAGGATCCTTTTGGTTTTGAAGATTACTTTCCCTCAATGATTGAAACATTGGGTGCAGAAAGGTTTATGATG
GAACTATGCAACGGTTTCTGCTTGCTGATGGATGTAAGTATAGGGCTTAT
>TCONS_00035454
AGTTATGATTCGTTCCTTCATTCATGAGCAAAAGATGCACATAATATTTTTTCATTCTTTAAACTGCCATTCAC
GACGACACATAGGGCAATGTGCTTGAGCCGATTGAGAATTCACCCACTTCAAGATACAATGAAGATGAAATG
CATGGTTGCAAGCACCCCATATTAGTGGACAATCATCACCGGGGAGTTTGCAATCAGGACAACAACCATCAA
AAGCCATCCTACATATTCCACAAGTTTCATCCTGAGCGTCCCATGTCCACGAAGCAACTGCATGCCATCTGCA
TACACAAGTAACAAGTAAGCACACCATGCTGTCTTAAGTACGAAAATTATCACATGCCATGAAGAGGACATA
CTCTATTCACGAACCCGGCAAATCGCCCAACAGAAAAGGTTAAGGGAAATTTCATGTTAGTATTCAGTCCTG
>TCONS_00035518
GGCACATTGAATGCGTCAACTAGAGATTCATCTATCATTCCCTCATTGACCATATCAATAAGACTTGATCCGA
AAAATTTGAGGAGACGATAACCTGAAAATGGCGAAACTAGGACCATTATTCCTCCACGAACAATCTCCTCAG
CTCTTGCATTCAAGAACACTTCCATATCCTTGTCGAACTGAGAAACATAAGCGTTAACTACTGCAATATTTGC
GGCATCTACATAGTGAATCAATCCCTTATTCCACGCTGGGGATTTCTCATCT
>TCONS_00035539
CGACAGTTAACATAGAAGAGTAGTTATTTTAGATTGTCATGCGCACACCAAAAGCTTCTCGAACACAACACT
TCCAACAAAATTTTCCTATCTATCTCATGTTCCTTTATTTTGAATATGGTCATTTCCCCTGTCTGAACTACGAGG
AAAAATGGAGACGTCACTCCCAGGACTTCAAGTAACTTCTCGAACTCTTCATGGAACTCTTTCTCAGTGTAGT
CGAAACCGTAACCAGGTTGTGGCTTGTCACTAAATCCAAATCCGATCCAGTCAGGTGCAAAGCAGTGGAATC
CAGTATCCGCCATCTGAGACATAACAACTCGATAGCTGTATGATTGTGTTGGAGCTCCATGGAGAAATATAA
CTGTCCCACGTCTGCTTTCAGGTGATCCTGCAATGGGCAAACTTGAACATTTTGAAGTCCTTAAAC
>TCONS_00035554

ACTAACTTCATACCCCAAGAATAGACCTTTTTGTCTGTCTCTGTTTTGGATAGTTTAATACTAGTCTCCTCTTCA
ATAAAAAGCAGAGGAATACTGAAACTAATACCAAAACCAAAATGAAAAAAACTTCTAAATGAAGACCTGG
TAAGCTGCAGCTCCATCCCCTTTGCAAAAAGCTATTTGTGGGAGTGATTACTGGATCCATAACAAAATCCACA
GTCATCGCTTCTTCCCCCAACATAATTCTTGTACTCCTTGACTTATATCCTGGCATTGTTGCTACAACTTCATAA
TTCCCCCCAGGCGCCAGCAACCGGTGGTAATCGGCTAACGCTTCCCTTGCATGTATCTGAAGACAATGACAA
GAACAGTAAGAAGACTGTCCTAGAAATATGAATTATCTCACAAGTCCTATTTAATGCCAAGACGAAAAGTGG
TGTAACAGATGACATGGAATAAATAAATAAATGAATGAATTACCAGTATGTGAAAAGTTAGCTAAAAGGAA
TAATCTGCCAAACTACAAACAGACTAGCCCCTACTTCCCCAGGCTGATGCCTTTACCTTCTTATTCCCCTACGA
AAGAGAAAAAGAGAAACC
>TCONS_00035555
CTAACTTCATACCCCAAGAATAGACCTTTTTGTCTGTCTCTGTTTTGGATAGTTTAATACTAGTCTCCTCTTCAA
TAAAAAGCAGAGGAATACTGAAACTAATACCAAAACCAAAATGAAAAAAACTTCTAAATGAAGACCTGGT
AAGCTGCAGCTCCATCCCCTTTGCAAAAAGCTATTTGTGGGAGTGATTACTGGATCCATAACAAAATCCACA
GTCATCGCTTCTTCCCCCAACATAATTCTTGTACTCCTTGACTTATATCCTGGCATTGTTGCTACAACTTCATAA
TTCCCCCCAGGCGCCAGCAACCGGTGGTAATCGGCTAACGCTTCCCTTGCATGTATCGTGGAATTTATTCCTTT
TATAGCTATTAAGGCAGGTAAAGGCCTGCCTTCATCTGATGAGAAAATCCTTCCGTGTATTCCTGTCTTCACA
AGGCTTGCGACAAGATTGAGCATACTCATCTTGTTATACTCAAAAAGAGTAGGAAGCTCACGTGCATTAGGC
CATTTGTCATCACTTATCTCTAGAGTCAGTTCAAAACAGCCAGCATGTAAGTAATTCCAGTCTTGCATGCCTCC
ATAGATTGGATACCAATATGCACCATTTGTTATTCCTCCTGGAAACTCCTCGCTTAGAGACATGTTATGGTGG
GAATGACTGTAGATGCTAGCCATGTGTCTGAATGTTTCATCATCTGGACAGCCATAATAATATTTTCTTTTGTT
CTCAGTTCCATCCCATGGATAATTTGCTACAAGTGCTCCCCCGTGCAGACTAGCAGATGCTGTGAAGTGCATT
TCCTCCAACCAACGCATAATGGCTTTTGTTTCAGGTTGATGTGCGCCAGGATCATCATTTATGGGAAAGAACT
GGTCAGGAAAGTCTCGGTTCAAATCGATATTGTTCGCATTTCCACGCCTCCTCAACGAAAACCCATCTGGATT
CATTGATGGGAGTATGTGAAGATGAACATTGTCTACAATCAGGGTAGCCAAGGGATCCTTCATATAATTATCA
CAAAGCCAGTTCGCCAGAAGAATTAGAAGCTCACGACCTACAGGTTCATCCCCATGCACGTTGCCAATGTAC
TTAAAAGCAGGCTCAGCCTCTTCTTTGCCTGGCTTGTCAGAGATTTCCATCACCCACAATGGGACTCCCAGCA
CACTCTTTCCAATGCTGTAGACTCTGGATATCTTACTGCACCTA
>TCONS_00035557
GTGCATTAGGCCATTTGTCATCACTTATCTCTAGAGTCAGTTCAAAACAGCCAGCATGTAAGTAATTCCAGTC
TTGCATGCCTCCATAGATTGGATACCAATATGCACCATTTGTTATTCCTCCTGGAAACTCCTCGCTTAGAGACA
TGTTATGGTGGGAATGACTGTAGATGCTAGCCATGTGTCTGAATGTTTCATCATCTGGACAGCCATAATAATA
TTTTCTTTTGTTCTCAGTTCCATCCCATGGATAATTTGCTACAAGTGCTCCCCCGTGCAGACTAGCAGATGCTGT
GAAGTGCATTTCCTCCAACCAACGCATAATGGCTTTTGTTTCAGGTTGATGTGCGCCAGGATCATCATTTATGG
GAAAGAACTGGTCAGGAAAGTCTCGGTTCAAATCGATATTGTTCGCATTTCCACGCCTCCTCAACGAAAACC
CATCTGGATTCATTGATGGGAGTATGTGAAGATGAACATTGTCTACAATCAGGGTAGCCAAGGGATCCTTCAT
ATAATTATCACAAAGCCAGTTCGCCAGAAGAATTAGAAGCTCACGACCTACAGGTTCATCCCCATGCACGTT
GCCAATGTACTGCATTACGAAGCCTTAAAAGCAGGCTCAGCCTCTTCTTTGCCTGGCTTGTCAGAGATTTCCA
TCACCCAC
>TCONS_00035662
CCTCGATTACATCATTACAAGGACTGAAAGTAGATACGTTAATTACAAAGAATATTTATTTAGTTTTTCTTCAA
TGTGGTGAACTCTGGTCAGAGACTCTTCAAATGCATTTAGAGCTACTTTATAATGATCTAGGGATATCACCCC
TTCTTCCACAACTTGTAATGCACTTCTATACAACTGGCTAAACCATTGAACGTGTTCAGTAGACTCAGTAGAA

CTGGATTCAAGATCTGATGCACCTAATCGCTTGTAATCCTTTTTCCATCGTGATAAAATGTACTTTGAAGGGAT
CTCTTCCACACCATTGAAGTTCAGTACACACAACGCGTGCCTGCACAGATATCCATAGAAGTTGAAGCAGCT
GCAAATACAACGAACTTCGGCTGCTGCTCTGTTGTACAGAACTTCATAATCCCGTATCTCTCTCCTATTTCCCT
CTCCCAAAACACGCTCCTTCACCAAAAATATTACCATTTGCCCGTCAACATGTAACTGTGTTGTGCTGAAACA
AGAATACATCTCCTCCACCTCTAACTGAACTCTTTTGAAGATCTCTCTACTGTACACTTTTGAAAGCTGCAACT
CAAAGGAACATCTTGTTTTGAGTTCAGGGTTCGAGTTTCTTGACTCTGTATCTGCAACCGCTTCTTCCTTGTACT
TTTTCTGCAAAGCTAATTCGTATTTGTCAAGAAACTCTTTTAGGGGAGTTTGCTTGTGCAAATATTTATCGAAA
AAAGCAGTCAATGTCTCATTAGACCTGGCAGTGGCCATTCCAGCAAAAAAAGTATCTTTCAAATAAACTGGA
GCCCATTTAGTTCGGTCATCATAGAGTGAAAGAAGCCACTCATGATCACCAACTCCAAATCTTTGGATCATAA
ATCTCCATGATGCTTCGAAATCAAATGGTTTCAGAGCCTCATATATTGTTTTAATTAATGCCTTTCTAATAGCA
TCATAGTTACGCAACCCTCCCAACTTTTCTGGAACTTTCCTCATGATGTGTGATAAAGCAACACAATGAGAAG
ACCTCGGAAACACCTCACTGATTGCACTCTCCAAAATCTTGCATCTTTCTGTAATAATTGTTTGAGGGAAACG
TCCAAGTGAGCAGGTGAGCCAAGCTTTGAACACCCAAACATAAGAGGCTTTTGTCTCGCCCGCAAGTAGGCC
ACAGCCCAGCAACACTGATTGACCATGGTGATTTGTGCCCACAAATGCCACAAGTCCAATCTCATATTTGTTG
CATAAATATGAATTATCAAAATAGATCACATCAATAAAGTAAGCATATCCCGCCCTTGACCTGGCGTCAACC
CAGAATACATTCCTCAACTGCCCTTCATCATTCAGATCCATCAAGTAAAAGAAGTTTGGGTTTGTCAATTGCA
TTCGACAGAAATAATTATACATGGCTTGCGTATCTCCTTTTCTTAAATTCAGCTTGTCGTGACAATCAGATGTA
GTTTTGCACCTCCTTACACTAAAATTGGCATTCCTATTCGCACCTGCATCAATCACAAGTGCCCGATATAACTT
GATTGTGCGGACTTCAGCATCACAGTTAGAATCCAACTTTTTCTTGTTTCCAGCACCTGTCTTCTTGATGGACTT
GTATGCTTTTGCACCTAACAAGTGATTGTGTTCAAGAGTAACTTCAAGTACTCTCCATCTTTTGGAGTCCACCA
TTCTCATCCTCATCATTGCAGGACAACCAGTTCTTGTTTCCTTGCGCAGCCGGTTTACATCTTTAATCCTCTTAA
AGCCCTGGCTACTGCAGCATAGTACTGCACCATACTTCTCCCTGCTATTTCTTTTGAACCAAGAATTTTTTACT
CTAACACGAAAACCAACCTCCCTGGCATAACAATTATAATAGTTGTAAGCATCATCATACGATTCAAACTCC
ATTCCTACTGCTGGGGCAACAAATTGCTTCTGCCCATCAGTAAAACCATTTTGATCATCTAATGCATCTGACTC
TATTTGCCCACCACTTTGAGAAAATAATTCATCTGACTCAATTTGCTCACTGTTCTGGTCATCTAACCCATCTG
ACTCTATTTCTTGCGATCTCCCACTGCCTTGCCCAGTAAGATCTACCTCACTATCAAGAGAAGCTTCTTCCATC
TGAAATTGAGAACTGAATTTCGCCGGAAAGGATGGGGAGGAGTGGCCTCGCAGCTG
>TCONS_00035663
CCTCGATTACATCATTACAAGGACTGAAAGTAGATACGTTAATTACAAAGAATATTTATTTAGTTTTTCTTCAA
TGTGGTGAACTCTGGTCAGAGACTCTTCAAATGCATTTAGAGCTACTTTATAATGATCTAGGGATATCACCCC
TTCTTCCACAACTTGTAATGCACTTCTATACAACTGGCTAAACCATTGAACGTGTTCAGTAGACTCAGTAGAA
CTGGATTCAAGATCTGATGCACCTAATCGCTTGTAATCCTTTTTCCATCGTGATAAAATGTACTTTGAAGGGAT
CTCTTCCACACCATTGAAGTTCAGTACACACAACGCGTGCCTGCACAGATATCCATAGAAGTTGAAGCAGCT
GCAAATACAACGAACTTCGGCTGCTGCTCTGTTGTACAGAACTTCATAATCCCGTATCTCTCTCCTATTTCCCT
CTCCCAAAACACGCTCCTTCACCAAAAATATTACCATTTGCCCGTCAACATGTAACTGTGTTGTGCTGAAACA
AGAATACATCTCCTCCACCTCTAACTGAACTCTTTTGAAGATCTCTCTACTGTACACTTTTGAAAGCTGCAACT
CAAAGGAACATCTTGTTTTGAGTTCAGGGTTCGAGTTTCTTGACTCTGTATCTGCAACCGCTTCTTCCTTGTACT
TTTTCTGCAAAGCTAATTCGTATTTGTCAAGAAACTCTTTTAGGGGAGTTTGCTTGTGCAAATATTTATCGAAA
AAAGCAGTCAATGTCTCATTAGACCTGGCAGTGGCCATTCCAGCAAAAAAAGTATCTTTCAAATAAACTGGA
GCCCATTTAGTTCGGTCATCATAGAGTGAAAGAAGCCACTCATGATCACCAACTCCAAATCTTTGGATCATAA
ATCTCCATGATGCTTCGAAATCAAATGGTTTCAGAGCCTCATATATTGTTTTAATTAATGCCTTTCTAATAGCA
TCATAGTTACGCAACCCTCCCAACTTTTCTGGAACTTTCCTCATGATGTGTGATAAAGCAACACAATGAGAAG

ACCTCGGAAACACCTCACTGATTGCACTCTCCAAAATCTTGCATCTTTCTGTAATAATTGTTTGAGGGAAACG
TCCAAGTGAGCAGGTGAGCCAAGCTTTGAACACCCAAACATAAGAGGCTTTTGTCTCGCCCGCAAGTAGGCC
ACAGCCCAGCAACACTGATTGACCATGGTGATTTGTGCCCACAAATGCCACAAGTCCAATCTCATATTTGTTG
CATAAATATGAATTATCAAAATAGATCACATCAATAAAGTAAGCATATCCCGCCCTTGACCTGGCGTCAACC
CAGAATACATTCCTCAACTGCCCTTCATCATTCAGATCCATCAAGTAAAAGAAGTTTGGGTTTGTCAATTGCA
TTCGACAGAAATAATTATACATGGCTTGCGTATCTCCTTTTCTTAAATTCAGCTTGTCGTGACAATCAGATGTA
GTTTTGCACCTCCTTACACTAAAATTGGCATTCCTATTCGCACCTGCATCAATCACAAGTGCCCGATATAACTT
GATTGTGCGGACTTCAGCATCACAGTTAGAATCCAACTTTTTCTTGTTTCCAGCACCTGTCTTCTTGATGGACTT
GTATGCTTTTGCACCTAACAAGTGATTGTGTTCAAGAGTAACTTCAAGTACTCTCCATCTTTTGGAGTCCACCA
TTCTCATCCTCATCATTGCAGGACAACCAGTTCTTGTTTCCTTGCGCAGCCGGTTTACATCTTTAATCCTCTTAA
AGCCCTGGCTACTGCAGCATAGTACTGCACCATACTTCTCCCTGCTATTTCTTTGAACCAAGAATTTTTTACT
CTAACACGAAAACCAACCTCCCTGGCATAACAATTATAATAGTTGTAAGCATCATCATACGATTCAAACTCC
ATTCCTACTGCTGGGGCAACAAATTGCTTCTGCCCATCAGTAAAACCATTTTGATCATCTAATGCATCTGACTC
TATTTGCCCACCACTTTGAGAAAATAATTCATCTGACTCAATTTGCTCACTGTTCTGGTCATCTAACCCATCTG
ACTCTATTTCTTGCGATCTCCCACTGCCTTGCCCAGTAAGATCTACCTCACTATCAAGAGAAGCTTCTTCCATC
TGAAATTGAGTGATGGAAAGGATGTTAGCAAAGAAAATACTCTTCCCAAATATCTGCGAGATGGAATATATG
CTTTCCTTTACAACTACAAGGGTAACGTGACATCCTCTCCCAATATACCAAGAAGGTGAACAAACCGCTTTGT
TATTCAGGAAAATCCAGTAACAGAAACGAACCAAAT
>TCONS_00035758
ATCAGCACCCTTAAAATTAACCATCGTAGTGTTCAAGGTCGCATACAACTTTCGTGATGGTGAAAACCAAGA
CTAAAACCTAATTAACAAATTACTGTAGGAAATAGTCCATAAAAGCGCCCCCAACCAAAGAAAATACTAAA
ATAAAAGAGCAAAATTTTCATTGTGAACTTCATCCTCAGAGAGTTGATATTCTTCTTTTGCATGGTTCGCATCC
AAAAACCTTGAAATTCCAACAAAAAGATCCCAACCATGTCATAAGAAAAATCTCCGTCGACTTGTATCCATC
TCCGGCTTGTGAACCCATTCATATTCCCCACCCTGAGTATCAAAAGTACCATGCTGGAGACTGATTAACTTGA
GAGTAAATCTAGGACCACATTCCTGCAGACGGGCAATTGTCCTTTCCTGTGGGTTCTTCGCATCCTTGCTTTTC
TTACCTTTTGATTCTGTCTGTTTGCCCTCTTTGGTTTCAAAAATGTAGCGGTGATGGCGAAAGAATATGAAATC
ACGTTGATTGTGAAAGGTCACAACTCGACGGCCACGAAATTCAGGGTCTTGTGGGAAAAGTGACTGTATCAT
CCTCCCAACTCGATGCCCCAAACGCGTTGTGAAATTGGTCAAAACTAGCTCAGGCTTGTGACTTGTTGGCTTT
CCATGATTCTTGATATCCTTCCGTAGCATGAGCTTTGAAAGCTTAAAATGAGCAGTAGGTCCATCAGGTAAGC
CAATAATGAGAAGAGCATCTGGTTCACGGCGATTAGTGTGGACTACAATAATAGATGAAAACTCCTTCTCAT
TTGCATATTGGACAATCTTTTTCAAGTCATATGTCCCTCGTTTGTAGTAATGAGCATTTGGAATCACAGAAATA
AGATCAGAAATAAATGCTGGTCCTCGTGTAGAGTTGTAACGGGATGTAGTAATCAATATTTTCGGATCACGCT
CCTTCCTCAGAATTGCACTAAATTCATCAACATCATTGCCAGCAAATAGCTCTTCATCATCAGGTCTACAGAT
AGTCTCATCCAACTCCCTTGTATTCTCAATGGTACGAGGTACCATCTTCGGTGGAGGCTCCTCGCCTAGCTCAA
GAGCTTTTTTCTCAGCAGCTTCACGAGCTTTAGCTTTCTTACGCTTATCAAGCTTTTTCTGATGCTTGAGCTTAG
CATGTACAGCTGATCTTTTCTCCTTGTTTTTAATCATTGAAGGACGCATGCTTTCTCTGCCCTCATTATTCTCTTT
CATCTTTTTTATGGGTTTTTCTTCTTCGTTTTCCCTCTCCCCGTCTTCGTTATTGCTTCGTTTCCGCCCCATTGAAA
GTGTGTAGTGCTAAAGGTTTCTGTGGTTAAGCTTGTGGCGTCGTTAAGGGAAGGTTGGAGAGGAGCAAAACG
GCGGCTTGTTTTTGGATGGGTAAATAAGGAGTATTGATAGTTTGGCCTCTACTTTTGAGTTTAGTTGTCAATCTT
CTCTGTA
>TCONS_00035808

CAAAGATTGATTTGAGCCACCAAATTCCTTTGGTGTCCTGTGGGCTATCTTCACCCTCTGGTGGAGGATCAGA
CTGAGGTGTCTTGTGGAGTTTACTCACATCATAGCCAACTTCTTTAGCCTTCTCAATCAGCATGTTGTATATCTC
TTCGTCAAGATGTGTTTTCCTACATAATATTTTAAGCTTAGCTTCATCACTTTTAGGATCAGCTTTATATGCAGT
CCCTTCAATTGATCCTCTTTTGCCATTACTCCAAGTTTCATTT
>TCONS_00035829
AGAAATTTGATTGGGGCCAAAAGCCCATTTCTTAGTGAAATTCTTGGGTCAAAGTTGTCTTGGTATTCCAAAA
GGAGCTGGTATCATGATTTGTTCATACATATCAAATGCTTACTGTTTATCCTCATGTTCACTTGGAAATGTTGT
GTCTTCAATTGTGGTGCTGAGCATTTCCTGTTTATTTGATTCAATATTAAGACAGTTGATAGATTGATGCATGA
AGCCTCTAAAAGAATTTGGGAATTTGATAAACTTGAGTTGGAGAATTTGAAATGAGCCATGTGGTGGAGAAC
ATTAAGAGATTTGTTGGGCCAAGTTTTGAGACAGAAGAGAATTGAGCTTTGATTTGAATTGATCTTGGCCCAA
AGAATAAATTTAAAGGTGGTGGGTGGGCTCACATAAGATAAGCTGACTCCTAAAATAATAAATAGTTTGTGA
GGTTTAATGAGATTGTGGAGATGAATTGAAGAAATTGTAGTTTAGTTGCGTTAGCGGAGACTTTGCCTCTGGG
TTCGTCTTTTGAATAGTTGAGACATTCATCTGATAGGCAGTTTGAGGGTTTCTACGCTTTTTTTTTTTTAATTTAA
ATATGAACTGGATGCTTTAAAGGCTTTACTAAATTGTTGGGTTCATTTGTTTGATGTCCAAGTATGTTTGGCAT
AATCTATGATTTTCAAAATAGTTGGTACTCTGGCAACATGTACAACTGATTATGTGTGTCAATTCTGAACTTAA
TTTTTATACATGTTCGGTAATTTAGGCGATGGTGAATTGAATCTTTATTGTCTCTGTCAGTCCTGTTTTCCTATTT
TATTCACAGTAATTGAACATGAAAGTGATATTGTTATGAGTAGATTATTTGTAGTTTAATCTGAACTCCATGTG
TTTAGACAACAATAATTGATAACCTCTATGTTATAGGAAGTCTAATTTTTATGTTTGGTTCAAAGACAAAATG
AGTGATTGATCAAATGATGATGTTGTGGCTCACTCTGTTTTCTTTTTACTGGTTTACAATTGTATTTAGCCAGTT
CTTGTTCTCAAGGGGCTTATCACTCTTCGAAGCCTAATGTCGTTTACCAATAATGCTAATTAGGAAGTATGAA
CGACCAAGACTATAGGATCATGGTAACCACTCGGCAAGAACAAGTGCCATACTGATTTGAATGCTTTTGTAG
TATTTTTCAGTTTATATCTTGAATATTTGATATATTAGCTAAGTTTTCTGGGACTGCTTCAAGTTTGCAATAAGA
TATAAAACAAGTTGCTTTAGCCTAGGAAAGTTGCGATTTTAAACCCTCTAGGTTTTCAAGTTTGTCCTTCAATC
CACAAGACTAGGAGACTAAAGAGGCCTCCAATCTCTGGCTTCTATGAATGCATTTTCTTTCAACTTTAGGACT
TTAAGGACATTGATTTCTCTTACAATAGTTAGTTCGCTCCCCTCTAACCGGTTCCAGAGAAGGTCAGCTTCCTC
AGGGTTTTCAGAAGTATGAAGGTCAGCTTCCTCAGGGTTCTCAGAAGTATGAATGACCAAGACCACATGATC
ATGGTAACCATTCGGCAAGCAAATGTCACAACTTATTTCAATGCAAATCTCCGTAAGAGTGTTTTCAG
>TCONS_00035886
CCGAACGTGCCAAAAGAAAATTTACTTTGCATGCTGTATCATGAGCAACGCCGATGGATATTCAGATAGGAA
TTTTGGCAGCTCCATAAGTGAAGGATCTAACTCAGGAAGTGCCATATAAATGTCTTCTGTTAAGAAGGAATA
CTCAGATCATCATCCAACAAAAATGAGTTAGATGTTAGATTCTGAGAATCCTTGTTTAAAATCTCCCTCATCT
GTGCGACAACCTCATTTGACACACTTTGAGTTCCATACTTGTCATCCCAATACATGGTACTAATTCGATATATT
TGTCTGATTGTCAATGCCGGGCAAAGGTCCTGTCTTATCTCTTCCAGTGATTTCTTCCTCTTCTGATGAATTACC
AGAAATCCAACAGCTTGTCTGATATAATTAAGTTCATGCCAAGATGTACCTGCAAACTCTTCTTTTGCATTGA
CAATCCATTTTTCTAGTTCTGCCAAACCGGACTTCACATATTCTCCATTTGAAAATGTGCAACACTCTCGTCGG
AGTAAAAGACTGTTAAAAAGTGATATGTTGATGAAGGAGAAAACCTGGGTTGTCAGCTTGCGGATGAAAAA
CGAAGGCACATGATTTCCGCATAAGCGACTCAAGAAAGAATCCAGAAATTTGATGATGCTATCCCACTGACT
GCTTGGTGCCTGTTGAGGGATACCACCAGGTGATCTAGTTGATTTGCCTCCATGCACCCGTTGGATTTTTGGTG
CCTGAATGCACAGACCCAGCAACGGGGATATTTCCTTCTTCAAATTATCACGAATCAGACCAAATATCTTCTC
TACACATGCAGTTAATTGTTGTTTAAACAGTAAAGCTGGATATCTTGCTTCCATATGTGACATTCCATCCTCCG
ATCCAATAAATTTCAAGGGCGACTTTAAACTTTGTGCGACCCTCCCATTTAGTGCAGAACCGCCTCCAGAACG
CTGAGAAGTACTAAAGAATCCATTCGCCCTCAAATTTCTTTGCAGAAGACAGAGAAGTGCAGATGTATTCGA

TAACCAATAAGGCAATGTGACAGCCTCATCTCCGACCTTCAAAACTTCATTGATACCCGCAATGATAAAGTC
AAAAATAGCAGTCCTCTCTGACTCAAAGGCATGCCAGTGAAGGAGGCACCTGTAGATGACACAAGCAGCAA
CTGGCTTTCCATCTTTAAATCCCAAATTCTCTTTGATACACCGTGAAAGAATTTCAGAGTTCTCCTGCTGCTTTT
CACTGGTCAACTTAGCTCTACGCGAATCAGAAAATCCTTGAGCAAGAGGTGGTATGATTTTTGTTGGAGTGGG
AGATTCAAATGAAGATTTACGATCAGCAGAGGGCAGAGCGAGTGCACCAGAAAATTTATCGACAAAAGGCT
TGGCAAAACCAGCACGATTGCTCCTCGGAGTTACACCCAGTGCTTTCTGTCGGAGGATATGATTTTCATCCTCT
AAATTTGAGAGCTTCTCTTGCATACTTTTTAAGTTTTGTTGGAGTTGTGAACATGTTTCTTCAACTGCTGTCAGC
TTGGAAATTGTGTCAGTAGACTCTTCTTTAGCCTTTAGTAGCTCATGCTCCAATGCTGAGTTCTTTTCCTCCAAG
GCACTCAATGAACTCTTCAGAAATGTATTTTCATTTCTCAGCTCAGTCACTGAAAAAACTTCTCTTTCCAAAGC
AGCTTTCTCCTTCATGGATAAATCCAGTTGTCTTTGGAGCACAGCATTCTTGTTCACCTCATTGACTGCAGCCA
ACTTTGCTGCATCTAACTCAAGGCTTAAAGATTCTACAGTCTTGTGAAGTTTTGAAATTTCCACTAATTTAGCC
TCATCATTAGATAGCCGCAATTTTTTTTCTAATTGCAACCTCCAAGTGAGATCCTCCAACTGCTTTTCAAGCTT
GGTTTTTGCTATACGAAGGGCACCAGCTTCATTAGCTTCCTGTTTGAGCCTTCTGAACTCTCTTCTTGCTATTTT
TCGCCTCCAAAGACATTGTATAGAAATAATATTGGACTGCCGATGATGAAACGCTGATCGAAACTTGCACAT
TCTCCAATGAGCCTGAATGATAGTAGCAGCCTTATTCTCCTTCCTGTATAAGAACTTCTGTCGAGCAGCAAAA
CCACGAACGCAAGACTGTATCAGCAGAGAAGAAGCATAAAATTGCACATAAGCATTCCTCAAGATCCACTT
CCGCACGTATTTCTGGATTATGATCGCAGCTGATGCTTCCTGCAGTGCAGCATATAAATTACGAGCAAGATAA
CCTCTGCAGCAAGACTGCAGATGAATTGCAGCCAATCGGTTTGAGATGAAGTCCTTGCGTGCAAGAAATGTA
CGTAGTCGACTTTGTATTTGCTTCGCAGAAGAGTCCAAAATCTCAGCACGACGTGAGTCTAAAACACCAATCT
GGCCAGCCCTAAGGAACACTTTTGTCTTTCCCAACTGATAATTCCCGAGCTTGAGTTTCTGCAGTATTTTCTCT
GTGATGGTTTTTTCATCATTACTTCCATCCAACATGTCTAAAACAATCAGTCCAAATCGATCTATAAATTCATG
GTAAGTTCTGCGTGTGGGATAACCTGCCAAACTTATGCGCACAGCCTCCAGAACACCCTTAAACCGAGAAGC
TACTGAAGAGAACTTGTATGATGACCTAGACGATTCTTCTCCAAGGGATGGAAAGAGACCAGCAATAAAAG
GACACTTCGAAGAAGATAACAGATTACGGTGTTCTACCACGACATAATCACGATTTTTATCTAAGAATGCTTC
TGTCTTATATGTGACCTA
>TCONS_00036016
TCTTAAGCAAGGCAAATCTCTGACAATGGCGCACTCTCCAACATCGTAGGTCTCCCGTTGATTTTCTTCTATGT
CTAGTAGAAGGTGCAGCAGGTTATTCCGTCACTTTCAGTTGGTAAATCAAAGGTATGGCCTTCCTCTCGCTTTT
GATAAGTTATCTTCTTTAACTTTTTGGAGTCAAATTTGACACTGATAAAGGGAGCTAGAAAATGGTTTTAAAA
AATTAGGGTTTGAGATTTTTGGCTACTTATAATGAATCAATCCCATAATTATTGGGTGAGTTTGTCATGAAGTT
TCCCACAAGGTGTTTGACGCTTTGCCTCAATGAATCTCTG
>TCONS_00036046
GGTTTATAATTTGCTCCCTTTATTTTCTAAATTCCCATTCATGTCTTCTTCCTCTCTTACCATTACACTCTCTTCAT
CCCTTCGCTCTTCATCTTCATTACCTCTCCTTCTCCATCGCCATCATTTCCAGCGTGCTGTGCTGTCGTCACTTGT
ACCTGCCGCAAAAGCAGTTAACTTCGAGGCATCTGCTGCGTCGCTGTTCTATTAATGCAGATAATAGTGTAAC
TGCCTCTTCTCTTTTCCTTTTGTTTTTTGTTAATTTATTCGCTGACTTCATCT
>TCONS_00036067
TTACATTAGAACAGAAGGAGGCTAACAAGAGAATCAAATATCCTCGTTTATGAGTCACTAACATTATTCTCA
AACAACTAAATAACTACCAAGAACCTTAAGTTAGAAGTACAGATTGGAAGAATGATCAAGAAAAGGAATTT
TCCTCCCAATGCTGGACCATTTTTTGTCTATTGACCTATAGAGCTTAGTTTTCCAATAACACCTCTTTGCAAGTT
GACTTATTGTGGCCTGTACCCTTGCATCTACTGCATTGGAGTTGACGCCTAGTTACTTCATGGGAACCCACCTT
CTTAGTAGTTGGCCGCCCTGGTGGACGACGGGTGGGAGGAGGGTTTACTGTTACAGCAGCTTGAGAAGCATC

CTTTTTTTTGGGCCTTTCTAAGCTTGGAATTGGATGTATTGATTCTGAATATGTTGATCTATAACTATCAGCAGT
AAAGTATCTGGCACAATAGTCGTATGGATCACGGCCAAGGCAAACCATGACAGCTATTGCGTGGCAACAAG
GTAAACCAGTTAGCTGCCAACCTCTGCAAGTACAATCCCAATGGTCAACATCAACAAAATCTACGGTATCCC
CTCGTACCTCAAATTTGGTACCACTAGATGTCAACACTTGAAGAGAACTAATATTTAGGCTTTCCTTCTCTAGC
CTTTCCTCCATAGATGGAGTTAGCCTCGTCACCCACTGATTGGACTCATTCCGCCGGGTATAAATGAGCTCCA
TAATCTTACCCCTTATTGCATCAACCATCTGAGTGATTGGCAAGTCATGAGCATCTGATACCCAACCATAGAA
AAGCTCTCCAAAGTTTGATGTCATGTGATTATATCGCATCCCACGGAAGAAAGCATTTGCCCAATTAATAGGC
TCACTTTGCATTACCCAGTTGTAAGCATCTACTGAAATACTTTTTATGCTATCAACACACCTCTGGAAGCCGTC
AGGCTTTGATGCATAAGCTGCACCATAAAAATCCTCAACCAAAAGGCGCTTGACTTCATGAGAAAATTGGCC
TCTCACATCTCTGATAAGTTGTTCAGAAAGATAGCGTAGACAGTATCCATGAAAGACGTCCTCGCCCTGAAAT
ATTTCAGCAATTGATTCTCTAAGGCCCTTCTCTCTATCTGCCACAAAAGTAATACCACGACACATTGACAATG
CAGTTCTAAGCTGTAATAGGAACCAACTCCAGTTATCATCAGATTCAGCGTCTACTATGGCAAAAGCAACAG
GAAAAACACCATCATTCCCATCAGCAGCTGTTGCTGCTAACAGAGTGCCTTGGTATTTAGACTTTAAGAATAT
ACTATCAAGGAAAATCAGGGGCCTGCAGCCTTGTTCGAAGCCATAGAGTGAAGCATGAAATGATACAAATA
GCCTATGAAAGCTTGAGTCATCCTTTGTAGTGAAAGTTGCAAGACTTCCAGGATTTGTCTCCATCACTTTTTCA
CAAAAAAATGGCAGCTGACTATATGCCTCCTTATACGAACCTTGAAGCTGCTCTTTAGCAATCTCTTTTCCAC
GCCATGCCTGGAAATAATTTAACTGGATGCCGTATTCCTTTTGTATGTCATTGACTATGTCCTTTGGCTTGTAAT
TTGGGAAAACTTTCAACTTCTCCTTTATAATACTAGCCACCCAACTCCTCGTTGCCTGATATCCATTAGTAACA
ACAGCACCTTCACATGTATGTGTAGGATTCATTTTTTTAATGCAAATCAATTGGGTGGTTGACAATCTTGATGC
ATGAATTCTCCATGGACAGCCCTCTGCTTTGCATTTTACAGTAACTCGATGACTATCATTCTTCTTGTACTTGAA
AGCAAATTGATTTGCAATAGCATATTTTCGCAATGTCTCACGAAATTCATGTACGCTATTAAACCTTTGACCC
ACACCAGTTATATCATTCTGCCACTGCTGAGCAGCTTTAGCATTCTTGTCGTCATAATTAGCGGCAACAAATG
AAACAGGAACAGGCATTTCAGCTTCTATCCCAATTGTGTCGTCAATGTTGTTTGTATCACTTACAACATCAAA
ATTGGCATCAAGCAAGAGGCCAGGCTCGTTAGGGTCATCCACGATATCCTCCACGACACTAAGAGGAGCGTC
AACAGGGATCGCCATTTCTGATAAAGTCGTTCGGCTTGACCTACTGCCAGGCATGTTTGAGAAATCAGGATCA
ACAGCTTCTTCAGTCATCACATAGATCTCAGCAGAATCAGAGTCACCATGGAATTTGATCATGCGCTTAAGGT
CTTTGTCATTGGAGATTGTTATAAGTGTCTTCCTGTTTCCAGGAAGAAAATATTTAACTGACATGGTAGCAAG
GCTGAAGTTGAACATCTCAGCTACCTCCATCTTGAAATCATTATAGTTCATTTTGTCATCCATTTCCATAGCAT
GAGCATCTCCGCCTTTATACGAAAGGAAACCATCTTTATCAGTCTCAAATTCACCACCTGACTGACATATGGC
TATAACTCGCTTCCCAGCCATGACCTTTATGCCTGGGAGATGAAATCTTGAAGTGGGTATTCAAGAATGGCAC
AAAATTGTTACTACCTGCGAATTGGAAAAGGGCAATAGAATGGGAACCTGGAATCTGCATAAAAAGGGGAA
TGAGGGATTTGAG
>TCONS_00036068
CAAGAGAATCAAATATCCTCGTTTATGAGTCACTAACATTATTCTCAAACAACTAAATAACTACCAAGAACC
TTAAGTTAGAAGTACAGATTGGAAGAATGATCAAGAAAAGGAATTTTCCTCCCAATGCTGGACCATTTTTTGT
CTATTGACCTATAGAGCTTAGTTTTCCAATAACACCTCTTTGCAAGTTGACTTATTGTGGCCTGTACCCTTGCAT
CTACTGCATTGGAGTTGACGCCTAGTTACTTCATGGGAACCCACCTTCTTAGTAGTTGGCCGCCCTGGTGGAC
GACGGGTGGGAGGAGGGTTTACTGTTACAGCAGCTTGAGAAGCATCCTTTTTTTTGGGCCTTTCTAAGCTTGG
AATTGGATGTATTGATTCTGAATATGTTGATCTATAACTATCAGCAGTAAAGTATCTGGCACAATAGTCGTAT
GGATCACGGCCAAGGCAAACCATGACAGCTATTGCGTGGCAACAAGGTAAACCAGTTAGCTGCCAACCTCT
GCAAGTACAATCCCAATGGTCAACATCAACAAAATCTACGGTATCCCCTCGTACCTCAAATTTGGTACCACTA
GATGTCAACACTTGAAGAGAACTAATATTTAGGCTTTCCTTCTCTAGCCTTTCCTCCATAGATGGAGTTAGCCT

CGTCACCCACTGATTGGACTCATTCCGCCGGGTATAAATGAGCTCCATAATCTTACCCCTTATTGCATCAACC
ATCTGAGTGATTGGCAAGTCATGAGCATCTGATACCCAACCATAGAAAAGCTCTCCAAAGTTTGATGTCATGT
GATTATATCGCATCCCACGGAAGAAAGCATTTGCCCAATTAATAGGCTCACTTTGCATTACCCAGTTGTAAGC
ATCTACTGAAATACTTTTTATGCTATCAACACACCTCTGGAAGCCGTCAGGCTTTGATGCATAAGCTGCACCA
TAAAAATCCTCAACCAAAAGGCGCTTGACTTCATGAGAAAATTGGCCTCTCACATCTCTGATAAGTTGTTCAG
AAAGATAGCGTAGACAGTATCCATGAAAGACGTCCTCGCCCTGAAATATTTCAGCAATTGATTCTCTAAGGC
CCTTCTCTCTATCTGCCACAAAAGTAATACCACGACACATTGACAATGCAGTTCTAAGCTGTAATAGGAACCA
ACTCCAGTTATCATCAGATTCAGCGTCTACTATGGCAAAAGCAACAGGAAAAACACCATCATTCCCATCAGC
AGCTGTTGCTGCTAACAGAGTGCCTTGGTATTTAGACTTTAAGAATATACTATCAAGGAAAATCAGGGGCCTG
CAGCCTTGTTCGAAGCCATAGAGTGAAGCATGAAATGATACAAATAGCCTATGAAAGCTTGAGTCATCCTTT
GTAGTGAAAGTTGCAAGACTTCCAGGATTTGTCTCCATCACTTTTTCACAAAAAAATGGCAGCTGACTATATG
CCTCCTTATACGAACCTTGAAGCTGCTCTTTAGCAATCTCTTTTCCACGCCATGCCTGGAAATAATTTAACTGG
ATGCCGTATTCCTTTTGTATGTCATTGACTATGTCCTTTGGCTTGTAATTTGGGAAAACTTTCAACTTCTCCTTTA
TAATACTAGCCACCCAACTCCTCGTTGCCTGATATCCATTAGTAACAACAGCACCTTCACATGTATGTGTAGG
ATTCATTTTTTTAATGCAAATCAATTGGGTGGTTGACAATCTTGATGCATGAATTCTCCATGGACAGCCCTCTG
CTTTGCATTTTACAGTAACTCGATGACTATCATTCTTCTTGTACTTGAAAGCAAATTGATTTGCAATAGCATAT
TTTCGCAATGTCTCACGAAATTCATGTACGCTATTAAACCTTTGACCCACACCAGTTATATCATTCTGCCACTG
CTGAGCAGCTTTAGCATTCTTGTCGTCATAATTAGCGGCAACAAATGAAACAGGAACAGGCATTTCAGCTTCT
ATCCCAATTGTGTCGTCAATGTTGTTTGTATCACTTACAACATCAAAATTGGCATCAAGCAAGAGGCCAGGCT
CGTTAGGGTCATCCACGATATCCTCCACGACACTAAGAGGAGCGTCAACAGGGATCGCCATTTCTGATAAAG
TCGTTCGGCTTGACCTACTGCCAGGCATGTTTGAGAAATCAGGATCAACAGCTTCTTCAGTCATCACATAGAT
CTCAGCAGAATCAGAGTCACCATGGAATTTGATCATGCGCTTAAGGTCTTTGTCATTGGAGATTGTTATAAGT
GTCTTCCTGTTTCCAGGAAGAAAATATTTAACTGACATGGTAGCAAGGCTGAAGTTGAACATCTCAGCTACCT
CCATCTTGAAATCATTATAGTTCATTTTGTCATCCATTTCCATAGCATGAGCATCTCCGCCTTTATACGAAAGG
AAACCATCTTTATCAGTCTCAAATTCACCACCTGACTGACATATGGCTATAACTCGCTTCCCAGCCATGACCT
GCGAATTGGAAAAGGGCAATAGAATGGGAACCTGGAATCTGCATAAAAAGGGGAATGAGGGATTTGAG
>TCONS_00036088
CCGCCGTCCAATGCTCTTTCCCAGGATTAGACTGAAATCTGCTAACAACGGCAACGACAAAGCAGATATCAG
GCCTAGTGCATAACATAGCATACATGAGGCTCCCCACAGCTGATGCATAAGGGACCGCCTTCATCTTTACTAT
CTCTTCATCAGTCTTAGGAGACTGATCTTTAGATAGAGAAATTCCATGTCTGAAGGGAAGAAATCCTTTCTTG
GAATCATGCATGCTAAACCTGGAGAGTATTGTATCAATATAAAGACCTTGGGACAAGCCTAATATCCTTTTCT
TGCGATCTCGCAAGAGTTTGATCCCAAGGATATGAGCCGCTTCTCCCAAATCTTTCATATCAAAATGTGTGGA
CAACCAC
>TCONS_00036156
AGAAGGAGATATGCAGCCATTAGGCACGAAGTCCTTTCTCCAGCTCAGGTATTTGAGGAACATATTAGAAGC
CTTTTCAACATCTAGATCTCGAGCTCGTAAAAATCTGCGAATCATCTGATCATCCACACCCTTAGCGGAGGGA
TCTTCTTTCTGGACAAGGGCTCTCATGATTCCGACTTTACTAGGCTCAATCTCTTCAGTTTCC
>TCONS_00036157
TAGTTGGTCATCATCAATGTCTCGCAGAAGAGTTGATCTCAATTTTTTGTTTTCAACAAAAATAATCTTTTTCTT
TGTTTTGCTGTCGATGAAAGGGTAAATAGCTTTCCATGCAGTCATAAATATGTAAGGGACATGGACTATAAAT
AGCTTGCCTAGCCGCTCAGGGTAGCAGTCCTGCAATATTGAAAGAGCAGCTAAATATCCACGAATATCGCTA
TTAGCATATCCCCATCCTTCCAGGTCTCCAATGGCAACAAACTTCTCTTGCTCATTTGGCATTCTA

>TCONS_00036172
GATCTCACTGTCCTATTTCTTTCACCATATGAATCATCATCTTCTTCTTCCTTGTTCTCTTTGATGATTCCTTTCTT
TAACAGAAGACCCTCCAGAGTCCGTGTGCTGAAATCATCTTTTCCTCCCAAGTCTTGAAAACCAACCAGCCTA
TCTGAAGCTACTCCATTCCTGAAGAAGATGACACATGGTAGAGTTTTAATTCCCAGCTTTGTAACGAAAAAAG
GAGCATTCTCAGCATCCAACTTTAGGAACTTTGTATCGACGTGTCTTGGTGCAAGAGACTTCAAATGCTTATC
CATGATCTTGCAGCGATAGAACTCCCGATGGTAGAAGTGACAAATCACTTTCTCACTACCAGTAACTTCACCC
AAAAAATCTCCTTCACTAATCTCTCTATATTCTCCATGCCCTTGCCTTTTTAAAGCTTGTCGCTTCTCTGCTTCTT
TCTTGAGAGCCGCAATCCTATCTGCGTGCAATTTTTCCAGCTCAGGGTCATCCATCAGTTCATCAAGATCTATG
TCTTGATTAACAGAAGATGATGCTTGTGCCTTCTCTTGAGCCAGCACTTCCTTCTGGTAATCACGAGCAGCAG
CAGCCATTACATTTCCAAATGCTAAATTAGAGAGAGTTGATTTCACTGAATCCGGATCCATCTCCTCTCCTTTT
TTTT
>TCONS_00036173
GATCTCACTGTCCTATTTCTTTCACCATATGAATCATCATCTTCTTCTTCCTTGTTCTCTTTGATGATTCCTTTCTT
TAACAGAAGACCCTCCAGAGTCCGTGTGCTGAAATCATCTTTTCCTCCCAAGTCTTGAAAACCAACCAGCCTA
TCTGAAGCTACTCCATTCCTGAAAAGCAAAATTCTTTTAGGTGTAGAACAAAAGGAAATGACAACTGTACCC
CTTCCCTTTATCCACTGTTTTGATGAGGCCACAAGTTTGTAACTTTAAACTATGTTTGTGAAGATCACGATAGC
TCCCTCATCTAATTTACAATAAAATAGGGCAGCTCCCTTGCCTAATCAAATCCAAGACCTGAAGAAGATGAC
ACATGGTAGAGTTTTAATTCCCAGCTTTGTAACGAAAAAAGGAGCATTCTCAGCATCCAACTTTAGGAACTTT
GTATCGACGTGTCTTGGTGCAAGAGACTTCAAATGCTTATCCATGATCTTGCAGCGATAGAACTCCCGATGGT
AGAAGTGACAAATCACTTTCTCACTACCAGTAACTTCACCCAAAAAATCTCCTTCACTAATCTCTCTATATTCT
CCATGCCCTTGCCTTTTTAAAGCTTGTCGCTTCTCTGCTTCTTTCTTGAGAGCCGCAATCCTATCTGCGTGCAAT
TTTTCCAGCTCAGGGTCATCCATCAGTTCATCAAGATCTATGTCTTGATTAACAGAAGATGATGCTTGTGCCTT
CTCTTGAGCCAGCACTTCCTTCTGGTAATCACGAGCAGCAGCAGCCATTACATTTCCAAATGCTAAATTAGAG
AGAGTTGATTTCACTGAATCCGGATCCATCTCCTCTCCTTTTTTTT
>TCONS_00036194
CAAATGTCAGCTAACTAACAGATTTGACAGTGGCTATAGGAATCAAACAATTCCAAGTGACTACACATGGAT
CAACTTCTTGAAACAGTACGTGCCCAAGAGAAACCAACCAATAAGAACAAACATGAAAATTACCAGGTACA
TAAGGAAAGTCCCTCCTCCAAATATGAGTCCTAAACCAAGCACAACGAGAGCGGGTAAATAGGTTTTAACA
ATAGATGTTTTATTTACCCAACGTCCCTTGAGGAAAATGAAAATGGCCAGGTTGACTACGTTCCATCCACCAG
TCTGAAACCCCAACCTGTTTAAACCATTAGCTACAAAAGAGTGGCAGTTGCAAGTGAAAATGCTGTAAGACA
GGTGTTGGAATTCTTGAATGCTCTTCCTCAAGGCAGCATCCCACGTGTCCACATGGCTTGCAGATTCATCATCG
TTCTGGATATACTCACTTGTATGTTCAGCTGAATAAGGAGATATGCAGCAGCATTGGAAATAGCGAGTTGGTG
CCCCAAATGTGAAATTGTCTACAGAAACGAAGTTTGGCCCTGCAAAGTCCAAGATTATGCCATCCTCCCTAC
AAATCCCTATATGCCCAATAAAGGGGATAAACCATGACAGGACAGGAAGTGGTGACCATACGATGCAGCAT
GGAAAGCGATCTCTTTTTGGATCAACTAGCATGGTATCCGCAAAATTATCTTCAATCATCAGAGCATGATCTG
GATCTACCTCAGGTGCCATTACCAGAGCTTAAACACTGGATCACAAGAACAGAGCACCCAAAAAATATTAC
AATTCATATACGAATGCATTCACGTATGTCTAGTTACAACTTATGGTCTACACTATCCAAAAACATGTCTCGC
AGCAAAATCAAGCTCTTACGGAACACAACAAACCAACACTGCACCGTGCCAGAATACACACAGCTTTAACA
TTCCATCAGCATGATAAATACATTTCCCCACATGTCCAGCAACAAAAGATGTAATGTGCTCAAAGAGTTGCCT
ATCTTCAAGAAAGTGTTCAAGCTAATTATCTCAAATAAGTCAAAAATCCTGCAAAGCGTCACTAGAACTAGC
TTGAAACATGAAACGAGTTTCCTTCAGCTAGTACATTGAACCCTCTTTTGAATTAGCAATGGTATACACGTAG
TGCTGGAATAACTGTCACTGGTAAAAGAAAGTGCATATGTAACTACAAAGAGTAGAAACTTGCTACAGGCAC

TAATAAATTGTTGAAATGTATGAAAGAGGGTTCAGTGGTTTGGCAATACCAAATCAAGTGTAGAAGCAGAGG
CTGATC
>TCONS_00036210
ACTATTTCACCAAATACCTGCTAAAAGCAAAATTATTGTCAACAGTAGATGCATCATTGACATTTTGAGAACA
TCATTTACATAAGTCCCTTAAGCATTTCAAGATGTTTTCCACCTATACACCTTTCAAGAACAGCTTCCATCCCA
TAGTACAGCAAATTGTAAAAACACTTCCACTCCATCTAAGGATAATTTATAGTTGAAAATAGGCAAAATCCA
AAGATGCTGGAAACAAAACTTAGAAACTAAAATCGCCAAGCAGGGTAATTCGCTTCATTGTTCTCCTCGTAG
GGAGCTGTCGAGCTGCTTCCTCCTCTCTCAGCATAATTGCCTGTGTTTTGGTTGGACGAGCGAAAAGGCCACA
AAAATGAAAAGGGGTTCCTCCTCCCCTGGTTCTGACCGGTATTGTCCCTGCCGCTATTGTTGCTGCCACTGCTA
ACATTTCCAGTTCTTGAACTCCAATTGGTCGGGGCACTTCCAGAAGGTCGCGAAGGAAGCTCAAGGCGGCAA
ACAGGGCACGAGTTGTGCCGAACCAACCAAGGTACAATACAATCTGCATGGTAAATATGCTTACATGGCATC
TGCCTTGCTTCAGAACCCAATTCAAAGTTATCTTGACAAACTGGGCACTGAGAATCCGTGTTGAGGTGCCTCT
GTGTGATCTTGACAGTAGGCAAAGCATCTATTGCCGAACGAGGTGCCGGGGGTGGGCCCTGCCTATCATTCAT
ACTGAGCTGCTCAATCAACTGTTGCAGCCCAGGCCCCATTAAATCATCTAAATTAGGTCGCCGCTGCCCCATT
CCCGGACTTCCATTGAAGAAGTACTCAAATGCATCATTTTCCGTCATACTAACAGGAGTTTGGCCGTGAAATA
TCAACCATGGACCAGAACCGAAACTCCTTCCTTGTCCAGGAACAATGCCAGATCTTGTCCTAATGTCAAAGTT
CGGGTTTCTTCCTGCCATTCTCTGCCTCATGAATTGAGCAAGGGCGTCCATTATACCAAATCTTGGGTCCGGGA
AAGGATCCATTAATCCAAAATGAGGATCATCCTCAATAGTAGATCTAGCACCTAGCATGTCATCAAGCTCTTC
AACGAAACCTCCCCCACAATTTGGGCAAAGACAGCTAGCCCCTCGAGGCCGAATTGGTTGCCTGCATCTGTG
GCACCAGTGTGTGTAGCTCCCACTCGACATCTCTTATCAGCAGTTTCCAAGAAGATGAGCTGAAGATGAAGG
T
>TCONS_00036254
AGTTCATATTTTGCCCTCTGATCAGGAGAAAAGAGTCCAAAGTGTTTCTCAGTGATATCTCCTTCCTTATTATT
TTCATCAAACATGGCAAATAAATAAGTTTCTATAGTCTTTCCAGGTTTCTTTGGAAGCCCTGAATATGTTGCAG
TTGAGACCTTGATTTGATCTTGCAACCCTGCTGCTGCTAATGCATTGTACACATTTTGCATGGCAGGAGCAAC
AAATGGTGCATATTGACCATTATTTCCGGGAGAGACTTCATTTCCAACAGCTATATATTTAAATTTAACATCTG
GAAAATGATTTATTATGTTATCTTGGACCCATCCATTAGCTCTTGAAGAATCAGTTAGGGATTGAAGATCTTG
ATTTGGGACGTCGAGAATGATCTCAATGTTACTT
>TCONS_00036304
ATAAAAATCACTTTGGTGAGCCAAGTCAAGTACCTGATGATCTTGATATGCTTTTAAAGACAGATCAGAAAC
CTCTTTCTTGTCATTCACAATTTTGAACTCTGCAAGGTATCGATAATAATCCCCTTTCATTTTGTAGTAAAACAC
AGTTGATTCTCCAGAAGTACATGATGGAATAAGATGCTCATCAATAACGGTCATGATATTATTGCAAATGTCG
GTGAGCTCTGACTCCACATTTTGCTGGTACTCCTTAATCCGCTTCGCGTTCTGCTCATTTCTCTAGACTCTTCCT
TCTGCTCGATGGAAGACAAGATCCTCCACGATGCTCTCCTAGCTCCAACCACATTCTTATAACCAACAGAAA
ACAAATTCCTCTCTTCCACTGTCAATTCAACATCCATATTTGCAACATTCTTCATCGCATCGACCATCTCTAGG
GGATATTCTGCCTTGAAAAGAAGACCACAAAACCTGCTACTATTCTGCTT
>TCONS_00036434
GTCGATGTGAGCTCTTGGAGAAGATCGGCCTGCTATCCCTAGAGTAACTTTTATCCGTTGAGCGACGACCCTT
CCACTCGGCATCGTCGGATCACTAAGGCCGACTTTCGTCCCTGCTCGACGGGTGGGTCTTGCAGTCAAGCTCC
CTTCTGCCTTTGCACTCGAGGGCCAATCTCCGTCCGGCCCGAGGAAACCTTTGCACGCCTCCATTACCTTTTGG
GAGGCCCACGCCCCATAGAAACTGTCTACCTGAGACTGTCCCTTGGCCCGTAGGTCCTGACACAAGGTTAGA
ATTCTAGCCCTTCCAGAGTGGTATCTCACTGATGGCTCGGCCCCTACCCCCCCCCCCCCCCCGGAAGGAGGC

CATCTTCGCCTTCCACCTAAGCTGCGCAGGAAAGGCCCAAAGCCAATCCCAGGGAACAGTGAAGCTTCATAG
GGTCTTTCTGTCCAGGTGCAGGTAGTCCGCATCTTCACAGACATGTCTATTTCACCGAGCCTCTCTCAGAGAC
AGTGCCCAGATCGTTACGCCTTTC
>TCONS_00036449
TATTAATTCTGTAAGGGACGCCTTATTCTGCACTCGGGTGGTCCGATGAACAATTCAGTCTCCACATCTGATTT
TTCACTACTCTCTGTACAAAGAGCTTCACCTTCCTTTTCTCCACTTGATCCTTGTCTCTGTTGAAGGTCACCAAA
CTAGAGCTGGCAAATTCATAGAGCTTTCCTCTTGGAGAAAAAATAACCAAACCAACCTCAGCATCACAAAGT
ACTGAAAGTTCAAATGCTTTCTTTAGCAAACCATTTCTGCGTTTAGAGAAAGTAACTTGCCTGCTTGTCGCGTT
CTCTATACGCCTCATCTGAGTTTTTCCTCTCACCATCTTTACAAAATTGGCAAAAAATCAGGAAGCTTTTTGGT
TGCAGGTATGAGGAAATATAGAAGTGAAGCTAAAAAGGGGAAGTCCCAAATCTGATGTTTGTATGTGGAAG
AACAACCTGAAAGGGTAATAGGTGCAAATTTTGCTCTTTT
>TCONS_00036601
GCCATTCATCTCTTTGAGAGCAGTATTTGCAGCCTCCACTGTTGTGTACTCAACAAAAGCATAACCTTTCGACC
TCTTGGAAATTTTGTCCATTATGATTTTAACTTCAACAAGTTCACCAAAGCCTTCAAATGCTGCACGCAACGTT
TTCTCAGATGTATAGAACGACAGACCAGTCACAAAAAGCTTCTTGGTTATAACATTTGTT
>TCONS_00036768
CCATTGCGAAGGTCCGAGCAATGCCGTGAACAATAAGATTGGTTATGTTGCCAATTAAGTAAGCAGTGAGGC
CAATGTTGAAAAGCATGTAGAAGATGGAGAAAATCTTCTCTCCTGTATTATGTGCACGAAGATCACCGTAGC
CGACTGTTGTATTTTCTGCAGTATGATAATGAGTTGCTAGCCAGTAATATAAACATCCAACGGAATGCACTGC
GAATAAGGTCACCTATGACAGAGTTATAGCCATAGGATGGAC
>TCONS_00036773
GCACTTTCTCTCTCCATTTTCCCCATTTTCTCAGAGAGAAAGGAGATTACCTTTAAAGTCTTTGATTGATCTCTA
TCTTCGTTTTCCCGGCGAAATTAGGGTTTGAATATCGCTCATCGATCTCTATTCCTCGATTTCCAAGATTGGATC
TTGCTGTCTTGAATTTTCGGTATTCTTATCTGCTTGAGCTTTTTCTCAATCTTTGGATCTGATAGGTGAATAGTG
GATACTTGGTGGAGATGTA
>TCONS_00036784
AGGTCTTCTCTATACACCGGAGCCTTTACTTTATACTTTAATTTAATATTTAATCAACTAATTGATGTTATTGGG
AACTTGTATAGTTCACACTCTTTGGCTCTACCCATGAATTATCCAGTAATAGGTCTTTCACAATCAGATCTACC
TATACAGTAACGGTATTTAATTATGAAAGTTTGCTGGGTAGCTGACCCTCTTAGTCCGTTCTTGCCAGAGTGGG
AGCCTGCCTAATCTTTATGTTTTATGCTTTTTAAATAAGATTTCCTCCGCTTAATGGATAACCATTTGTTACCAA
TGGAGAATTTCTTATCATCTTAAATTCAGGTGATTGGATTTACACCAACGGAAACCATAAACTTCATACACAA
TAGAGGGATATGATAGAGTTTTTTTTAATAATGAATGGAGTTCCTTCTTCCATCCTATCCCATTCACCGGTAC
TGATCATTGATACTGTAAAAGTCGTTTTCTTGCTTTTGTGCCAGCTCATGATCTAAACGAGTCGCACATACACC
CTAGTACATGTTCCTCGACGCTGAGGGCACCCCCGAAGAGCGGGGGATTTCGTGACATTTCTGATTGGCTGTC
TTGTATTTCTAATAAGTTGTTTAATAGTTGGCATGTTGAATCGTATACATAATATGATGGGTTGGTTTAGATTG
ATCCTAACCGAATGATGATGAATTACTTCTATTTACTATTTAATAGAATATTCAATTCGAAGATAAAATCTCA
AATCACAGATTTGCGCGAAATCCATGTTATTTTCATTCAACCGCTACAAGATCAACAATTCCATAAGCTTGGG
CTTCTGTTGCTGACATAAAAACATCTCTTTCCATATCTTCGGATACAACCCATAAGGGTTTCCCCGTTATTTTTA
CATAAACCCTTGTGAGGAATTCACGCAGTTTCAGCAGTTCTTCCGCTTCTAGGACAAATTCGCCTGTTTGTGCC
TCATAAAAAGAACTAGCAGGTTGATGGATCATTACCCAAGCGTGAGGGAATGCTAGACGTTTGGTAATTTCT
CCTCCGACCAGGATAAAAGATCCCATTGAAGCGGCTAATCCCATGCATATTGTATGGACATCTGGTCGCACA

AATTGCATAGTATCATAAATAGCCACCCCAGGTATTACCCAGCCCCCAGGAGAGTTTATAAACAAATACAGA
TCTTTGGTCTCATCCTCGATACTGAGATATACCATAAGACCAATAAGTTG
>TCONS_00036812
GCACGATCACGCCAAGAACCTTGTTGCATTGCAAGTGCCTGCCCGCTGATGAGACTTGTGGCAGGAGCAGTT
TCAGAGATGTCCTTTTTGCCATTATCCTCTTCTGCTTCATAATCAGTTTCGGTGGAAGGTGGAAGTGGCAGAAG
TGAGGGAACTCCAAAGATAGCCTCATCATGAGCAGCCTTGATTTGTTCCTCCCTCCTAGTCTCCACCGCAGAA
AACCGAAAGCTTTTGCCAATGTGGTTGACTAAAGCAACATCATCTTCTGCAGCT
>TCONS_00036836
TACCAATGCTCCCATATCCGTATATTTCCTCTAACTATTCATCTGAGATTTCAAATCTGTGATAATGGACTGTA
AAGCACCTATATGACTACCAAGTAGATGCAACTCATTTCTTGGATTGCTAGGCTGAAGTTTCTTTATTGGACTC
TGATATTGTATCCTCTAAGATCTTCAAGTACATTGTTATTTGTTATTGAGGCTGAGATGATTTATTTGCTTATTT
AATTTTTTGCTGCTACGTTGAACAGACTCCAGCTGAAAGAGTCAAAGCCAAAATGAAACTTCAGCTTTCAGA
AACTGCTCGAAAAGATGAAACAAAAGGCATGGGCTCTGGATGGGAGCGTTTTGACTTCGACAAGGATGCCC
CTTTGGATGAAGAGGAGATCGAAGGTAAGCTATTTGGCT
>TCONS_00036852
AAACACAACTAGCATATATATGTATACAAAACTCACAATTTTCAATAGTGAGAAACTTGTGTGTATGCATTTG
TGGCTTTAGTCATTCGCTGCTTCAACTGTTAAACAGCATACAACCTTATCACCTGATCTATCTTGGCACGACAG
ACGGGGCAGCCCCACTTCTTTCCCTTGATCTCATTCAAACAAGACATGCATCCAGCCATGTGGCCACAAGGG
ATGCAAGCTCCTTCCACTGGAGCATCTAAGCATATTATACAAGATGAAGAAGCACCATCAGGATTACTGCTTT
C
>TCONS_00036857
GCTGAAATATAATATTTAGGTCTCACAAGACTATCCCAGAATCTTTACATAATACACGCAAAAAAATAACTG
AAGTTGTGTTCAAAACAAATAAGTCAATCTAGATATATCTTACTAATGGATCAAAACCGATCTTTTCTTTTACG
TGCTGTGTATTTGCTATCTGGAGGAACTGCGTTCCCCTTTTTCCTCATCTGTTCTTTCTTCTTCAGAACCCAATCC
CTCCCTTTGCCTTTCTTGTTCAATTTTTGCTTTTTTCTAGGTCTACGCCGATCAGATATGCAAACTGTCTGGTTTT
CTTCATCCTCGCTGCTGTCTTCATCAGAGCAACTCTCTCCATCTTCACCCTTTCCATCTGGAGTAGTGGTGCTGA
GAGACGGCGGACCACAAGTCAGAACTAAGTACTCTTTCCTCCTTTTAGAACTGTGTGGGTAGTCAACAACTAT
TCCTCCAGAAAATCCAGCTCGCATGGCAAAACCCAGAATCAGCTCCCGCTGAGCAAGATTTTCAGGATAGAT
CTGGAGTACTGCTCTTGCTCCCCTTCCCAAACTTCTATACAAGGAACCAAAGAATGCCTTCAATCTTATCCGA
GGTTCATGACAAGATTTGTCAGCATTGCACAACCACTGCACAGCTGAGATACTGATGGCACCATCCAGAACA
CCAGGTCGAAGCCCTAAGCCCTGACCCATGTCACCCAGAATTAGATCACCCTCAACCTCACGCTCCAATGCA
ATATCAAGCATTGATTCTGATATATCCCAACCAAGCCACTGGTGTCCGTGCTCAGTGAGTGTCTCCCCACTTA
GACCTGATCCGCAACCAATATCAAGAAGTAATCTTGGGATGCCATCATCTGGCAAAGCAAGAAGCTCCAATG
CTCTCTCAGAAAGTTGTGTCTGTATGTCGATAATTCGAGAAGATGAGGTGTACTTTCGAGCTTCATCGTCGTTG
TAGAAAATTTCTGGCGGTGCTAGAAGTTCTGGTCTTGACATTTTTGTGAGTTGGGGGAAGCTAATTTGCTTTCC
CTTTTTCTGAAGAAGAAAGGCCTAAAATTTCTCTGTTAAACCCTAGCAAAGCCCTG
>TCONS_00036965
AGTGAGATTTGAATACTAAAGTTAAAACACATGCTCCTGGTGATCGTTTTGTTTGTAGAAAATCTTTCTGGTTC
CAAATTTTGTTGGTTGGATTCATTCTAATATTATATCAAGGGTCTGAGAATATATGTATTCATGTGAAGCTTAT
GTGTTTTTGCCTGTCTACCAGAAATGTTATTTCTTTCATGTCCCTTTTCATTTTTCGTTCAACTACATATCCATTC
ATAATACGATCAAATCCTGTGGAATCTAGTGTCTGGCTCTACTTTAAGCCAAATATGCATCAAATATCATATT
CACTGATCAACTACTAATAACGTATGAGGACCAGCAGTTTCATGTCTGCGAACGACCAGTCACTGCATTAAC

AAGTGAAAATTGTAAATAAGTGGAATAAAAGCTGGGTAACTGAGCGTTTATAAGCCATTTTTAGCTTGATGT
GATCTCTGATGCAAAGTTTAGTACTGTTTAACTTTTTGTTGTTTCTATTCCTAAAGTGTATAATATCAGAAAGG
AAATGCTTTTACTCTTCTTGCGTGCAGTATGCACGGGCATTGAAGGAGAAAGGAGTTGAGGACAAAGTCATG
GTGTTTTCCGAGGACACTCATGCATTTTATAGACCACAGTGTGACTTCGAGAACTTCCTGAATATTGGTGCGTG
GCTTAAGAATTACTGCAAGTAGATAGATATATGTTCAGCTCTTCAGCAGGAATTTCCCCGTTCCTTTATGACCA
ATCAGAAAAGAAGATTAAGCCATTT
>TCONS_00037045
CTCATATAACTTTCCCCTAACCTCAAACAACCTTTTAGGGCTCTGCTCTTTCATTATGTCTGATGCAATTTCTGA
AAGATATTGCTCCCAGTCCATTGGAGGTATAGCTTGGTTGTTTGTGAAGGGATACCTTAGGCAAATAGTACAA
GGGGTCAACACATGCATAAGGAAACTCTGGTGCAAATAGTTCAAATAATATGTGAAAAGCTTGGATTTAGCA
GCTCAAGCTAGCAAGAAGCTAAGACAAATCAGTCCAGCCAAGGTGTTACGAGCTCAAATGTTGAACACGAC
AAGTCTCAAACGTCAAGACTGCC
>TCONS_00037273
GTATCGGCACTAAGAAGCGTAATGTTGTCTTTAATTGGCTTGTGATTCTTGTTGAGCAAAACTAAATAGTAAA
TATCCACTTTTGGTGGGTGATGAAACAGGAAATAATCAATGCTGCTTCATATGCTCCGCTGCCACCTTCCGAC
GGGCTTGCAAAAAAACTTTCTAAGTTGGCTGTCATGGGGAAGGAAAAGAATGCTGCATGAAAAGCAAGCGC
ATATATAAAAGAA
>TCONS_00037278
GCATGGTCCTTGTGATACTGCTAGAAAATCTTCTCCTTGCATGCAAAATGGTAGATGCACAAAGCACTTTCCC
AAAAAATTTGTGTCATCAACCACAATTGATGAAGATGGGTATCCAATTTATAGAAGAAGGGATGATGGTAGA
ACTGCAAATAGAGTTGGTATTGCGTTGGATAATAGGTATGTTGTACCACACAATAGATTTTTATTATTGAAGT
ATGGTGCACACATTAATGTGGAGTGGTGTAATCAATCACGATCCATTAAGTACTTATTTAAGTATGTTAATAA
AGGTAATGATCGTGTCACGACAGCTTTTTCTCAAAGTGTAAATAATGAAGACTCGGGG
>TCONS_00037350
TTATTATTACCAACTCTTTACATCAAACCTTCCTTAGGAGAAGGAACAGCTTCTGGTCAAGCTCAAATTTAGCT
GCGATGGATGGATCTCCTCTCAGCTGCATCAGAAGTCCTCCAAATGAAACAAAAATGTCTGCTTTAACATGCT
TTCCTGAACCTTCCTCTGAGATCCTATATAGCTTCCCAAGCATGACATATTCGAACTTATCAGCAAGTGATTTC
TTGTTACCCTCAATGAAATAACCTGTATCTGGTGTCCCATCCAAGTTCAAAGTAGATGCTAAAACCATCATAA
ATTAAATTTCTCTTTGACGCGCATAGGATATATCTCAGTGTTCACATCCAACAGCATGTACATATCAAACTGCT
CACTCCGTGCTTCAATGCGATTAACTCTGTCAAATTTTTTACCATCAGGATCCTTTTGAGTAACAGTAAATATA
TCTTCGAAAAGTGTTTCAACCATCTTCAACAACCAGGTGTTACAGTGATGGCGATT
>TCONS_00037351
TTATTATTACCAACTCTTTACATCAAACCTTCCTTAGGAGAAGGAACAGCTTCTGGTCAAGCTCAAATTTAGCT
GCGATGGATGGATCTCCTCTCAGCTGCATCAGAAGTCCTCCAAATGAAACAAAAATGTCTGCTTTAACATGCT
TTCCTGAACCTTCCTCTGAGATCCTATATAGCTTCCCAAGCATGACATATTCGAACTTATCAGCAAGTGATTTC
TTGTTACCCTCAATGAAATAACCTGTATCTGGTGTCCCATCCAAGTTCAAAGTAGATGCTAAAACCATCATAA
ATTAAATTTCTCTTTGACGCGCATAGGATATATCTCAGTGTTCACATCCAACAGCATGTACATATCAAACTGCT
CACTCCGTGCTTCAATGCGATTAACTCTGTCAAATTTTTTACCATCAGGATCCTTTTGAGTAACAGTAAATATA
TCTTCGAAAAGTGTTTCAACCATCTTCAACAACAGGTGTTACAGTGATGGCGATT
>TCONS_00037642
ATCCATTTCACTAAACCTTAAGGGTGGCAGAATAGATACATCACTATGAAGATTTACTAATATTACATACAAC
ATATTTTAACCTTTTGATTTCCATCTTGTGAACTCAAAATTTAGAACAACTGGTCCTTGTGCAATGCCTAACTG

ATTTATGTTTGTATCTGCTTCTCGGATTAACAAAGTGGGGGGTGCTCTTCAGTATTCATTGGATGATTTCCAGTC
ATCAGACAAGACTCAACTCATCAGATACTGATACACCTTCAGAGATCTCGTTTGACTCAGCAGCAGCAATCTT
TTTCCGCAATGCCACCAGCTTCTCGCCAGCATCACATACGTATGCAGAAGCTTGCCTACCTGAAAGAGTTGCC
CCTTCCATGCTATCGATGTAGTCCTGTTTTGTATATGAGCCAGCAAGAAAGAAATTTTCCACTGGCGTCTTCTG
ATCAGGTCTGAATGGGTCTTTACCAGGTCCTTCACGATACAGGGATTGCCCAATTTTCACAACTGATGACCAG
GTAACCTCAAGACCTTGGGAAGAAGGAAATAGTGCCAAAACCTGCTTTGACACTCTTTTTATGATTTCATCAT
TTAGTAGAGGCATGTACGGGTCACCTGGCGTAAGGACACATTGAAGCAATGAGCCTTGGCCCTCAATGTAAT
AATCTTCCGGAGATGCCAATGCAAGGTCCGCAAAGCAAGAGAAATCTGCATCTGGTGTATACAGGAGATTGT
CCAAACCTGTAGCGCGCTTCAATTGCCTCGAACGCTCCAAGTCCTGCAACTCTGTAACCCAGCCATTGTATCG
TAGTTGCACTGTAACAACAGGCACTCCAACCAATTTGTAAATGTTGTCAAATAATTCCAATTCCCTCCACTTCT
GAGGTAACAATCTTTTAATTCCAGGGACATCACATGCAGCGACATAAGCATCAGCTTTTACAATTTTCTTCTG
AGTGGCCTTTGACATTGCAAGCCCGCTAACGTACATACTTCCATCAGAGGATGTCTCATAGAGTACCTCTCTG
CACCCCCACCTCAGATGGAACCTGCCCCCCTTATCCAAGATGTACTTCTTAATGGGACCACTCAAATAAACGT
CAGGAGAACCTTTAAGCATGCGTAAAAGGGAAGCCTCCGTTTTAGTGGCAAATAATGCAAATATAGTGAGCA
TACACCGAGCACTGATATTGTCACAGTCAATGAATCCAAGAGCATATGCGACAGGATCCCACATCCTCTGGA
TGCTAGTACGCGTCCCGCCTTTAGACATAAACCACTCCGAAAAGCTTACGCTATCCAAATCACGTATCTGCTG
CAATGCACCATCTGGATCAACTAAAGCCCGCACCACTGGACTAAGGGCAAGAGCTACAGCATTTCTAGCTTT
ATCGTAAGTCTTTAGCTGATTAGTAGTCAAAAATGCATTAATTCCATGTAGGGGTGCTCCAACCGGAAAACG
GAAATCAAGCTCCCCTATTTCACCCCCTTTATTTACAAATGTGTGGGTATGGTCCTTCACTAGCAGATTTTTTC
AGCACCCACCTTTTTCAACAAACGGAACAGATTATTATAGCAACCAAAGAACACATGCAGTCCCATTTCAAT
GTGGTTTCCACGTCTATCAACAAAAGAACCCACTTTCCCACCAATAAAAGGCCTCGATTCATATATATCCACC
TCATGTCCTTGATCCAAGAGCTCCACAGCAGTCGACATGCCTGCAAGCCCAGCTCCAATAATAGCTACTTTCA
GCTTTGGCCCTCGATAATGTTCAGGTTCAGGTGGAAATAGCCCTTTTGGAGCATTTGTACTCATATCAGAGAC
CATGGAGTCCAAATCAGCACGAATAACTGACTTCCGGGTCACTAACCGGTTCGACAAACGGGTACCACGGA
AAGCCAAGAATCCAGCTGACCCATTTGGAAAAATATGTTTCTTTCCAGAAGCTG
>TCONS_00037777
CCAATATGAGAGCCCAGTGACCAACACACCACAGTAAGCCACAGAAAGAAGATTGAAATTGAAACCTAATT
TCCATGCTTCTATGTCCCTCTTTACTGAAATAGCCCAAATTGCTGATTGAATGCAACTAAAGAGGCATTGAAG
AGTTGTAAGCCTTATTTTTGCTGGATATTGTTTCATAATTGGACCCTGTTAGAACAAATTGAAGCATACTTTTA
C
>TCONS_00037857
GGCGCACTTCTAGGGGCTTGTAAGCTACACTGTGACATTGAGGTAGGGGAGGAGGTATCCCATAGACTTTTTG
AACTTGAGCCTCAAAATACTGGTAATTACGTACTTTTGTCAGACATCTATGCTGCGGCAAATCGGTGGTTAGA
TGTTGCGTTGTTGCGTCTGAAAATGAGTGAGAAGGGTCTTAAGAAGATACCTGGTTGCAGTTGGATTTACGTT
GCTTCCTAGAGATATCTTCATCTACCTCAAATTGCTTCGACTTGCAGGTATGTTTTTATTCAATTTTGCTCATGT
TTTTTGCTATGCATTTGATAAATTATCAGTTACCAAAGCTAAATAGTGCACTCCTACACTTCATTGTACTCCAA
ATTCGGCAGAATTCACATTTTCTGTTGATTCCAAATTTGATCCATTCAACAGAGGCAAAATTCATCATTATGTG
CCAATAAAACAAAATCCCCACTTTCTGTTGTCCCATTCCAACATCGATTCCAAAAAGGCTAACCACAAAATT
GAAAAGATATTGTTGGAAATTTGCTCTGTCAATTAGTTAATTTTTGGGTTGAGTTGGGGTGGACGAACGCTGC
ACCAAAGAGCTGTATGAAAGCAACTTAAATTATACTGTAAACTTGGAAAGAACACTGTAATTTTGAGATTGT
GACTCTATACAGGGGTAAATTGATCCCTTTACCTCCTCATAGAAACACATGTAGCTCTGTTTTTCTTCAGCCCA
ATTTCTGCTTTTTTGCTCTCCTCGATTCTTCAAGATTGAGGCTGACTCCATTGATTGTTGGCTGAATTTTTCAAG

GTGTTGTGCATAGATTTCAGCAATTGTAAATCATATTCTTCGTAGTCTTTGGCCCTGTTATGGTCCTGCTGTTGA
AACACAACTCAGTGAAACATGTTAAACTTTGGTTTCTTTTTCTTCGAGATTATATATTTGTTGTTCTTTTTTGTCT
TTCTTCATGTTTCTTTGAAAATTCTGGACATGCATTTGTTTATTTGTTGAAATGCCTCTTATAAAAATTCTCTATA
TAGGAGCATACTCTACTACAACGCTGCACCGATGCACCTAAGGTGTTTGATAATATGCCTGAGGCAACTATA
ATCAATTTTTTTATTTCTTTTCCTTTTTTCACACATGAGTTCTATAACTTACGCATCATTCAACAACCAAAAGGT
ATATGGTTTATTACTGCTAGATAGTATTAGTAAACAATATATATGTCATATGCTGTCGTTTTAAAGAATTGCTT
TTTGAAATAATAAATTCCATTTGCTTCTATGTATATTTCTATTGCAATATTGCTATCTAGCTTTGCGTTAACAAA
AGATTTGGATACAAAGAAATGTGATCATGCTCCCCATGTTATTATTGCCGTTACGAAGCTAATTTGGCTAGAG
AGGAAGGAAGAAAATGACAACAATTGAGTTAATAGGATGTTGACTCTTGGAACAAATATTGGTAATTTCGAG
GCATTTTGTAATACAATTTTAAAGAGCAGTATGGTACTAAAGAGCAAGGGAATAATGTACGAAAAAGAGGT
GATGTTGTAGGGTCGTAGCATGGTGAAAAATGTCAAGCTTTTACCAACACCGATGAGAACTTTCATGATCCA
ACTGAAAGCATGTTGAAGAAGGCTAACCACTAAAATTAGTGAGTTGTGGTCAATTGACATACCTTACCTTTAA
ATCTATGAGTTAAATGATAATTATTGATTTATTCTTTAGGCACCATATTCTAACAACTTAATGAAAATGACCTG
ACAATCAAGAAGTCAAGATTATTTAGCTACTAAGTTTCACTTGATTCGTCCTTTGTTCTAAATGAATGTGACAA
GATGACTTCTTATAATTAATTATATGAATGGTAGAAGTGACTTCTAGATGTTTTAGTTTCTTTTATGGTGGTTAT
CTGAAAAGAAATAAACAAAAAAGTAGTACTTCTGTTCAATGGTTGAGCGTTGAGTTAAACTTGGATATATAG
ACGCTTTGGATTACATATGGCGCAGTATTTATAAACTAAATTGAGCTTTGAGCTGTTTAGGTCATTCCGCCATG
AATTTTGAGCTTGAGTCTCTTAATTAGTATGAACAATTTTCGATTAGACGATGAGCATGGTTTCGATTTTCATTT
CTGAGAATAGTTATTGCAGCTTACTATTTGTTATTGCTCACAATACACAAGAAGGAAGCACCCTCACACAAA
AAGGGAGAAAAAGAATTAGAAGAAAAATTAACATCTTAGTTAGTAGAAGCATTTAAAGGTATTTGTCTATGA
TATTCCTGCTCTGTCACCTCAAAATAATTGATGAGATCGCTGGAATATGTTCCATTCACCTAAAGAGATGATTT
GAAGTCGTCACTACTTTCATTATATGATAGTGGTGTCTTTTTTAGGTTGATCACACATTAGGACTATCAACAGA
ATGGTCTCGCCAGGCTCTATGCCTTACCAATAAACATTAATATGCTCGAACAACTTTATAGTGATTATTTTTCT
TTTTGGGTAAGTGCAAGCTAATTTGATAATATGAGTATGAACTTATTGGATATCAGTAGTTTATTCAGCTTTCC
TTGAATTTGTACAACAATTTATCTTTTGAACTTACTATTTCTACTTTTTTGGGATAATATTTGTTTTTCCTGTTAT
ATGATTTTATCTTGATTTCTATATAGTTTTTCTAATTCTTTTTGTTCGTTCACATGAAATGCAATCTTAATTTATA
TGTCTTTTTATTATTTACTTTGTTTGATTAACCATGAAAATGATGGAAATGAATAATCTTATTGAAGAAATATTT
ATGGAACTTGGAGTTACCAACTAAATCAAAAGGACATGAAACTAGGGCGAGATTGATATAACTAGGCTGCT
ACAGATAATTGCTTAGTCCCTCTGATAGGTTTAGTGTCTCCATTTCCCATGGCTATAGCTTGCTACTGGTATAA
TATGTCAATCTTCATATGTCTATTACGCAAGGAAAAAATTGATGGACTAAGCAATATTGCTTTAGGTACTGCT
CTTATTCTACGATCAAGGCAGGAGGAGTATTGCATGTTAATCCATGCAGTAGTTCTTTGGAATATGGACTAAA
GGCTAAAATTGTTTACTCTATCTGGTTTAACAATAATTTATGGAGATGTGACATCCTCTTTCTTCATTGTGATAT
TAATCACTAATTAGACCTTTGTCATAGAATATCTAATCAATTGGGCAAACAAAGTATGAGATATATGAAAAT
GATATTATATTTTAATTGATTTAGAAGGAAATCTTAATAGTCGTTCCAATGGAACTTCTTTCAAATAGGTAAGC
CAGTGGAGAATTTGCAAACTTCCGACAAGTCCTTTTGGATTCAAATAGAGGTGTCCAGGGTCCCATAAGGTG
AAGGATGCCTACTACTAGCTGAACTTTTGTGATAGTATTATTCCCTCCATTTCAATTTATGTGAACCCATTTGA
CTGG
>TCONS_00037925
AGCAGATCAGGTATGCCCTCAAATACCCAAGCCAGGGATGCAATCAATCTATACAGGTAAATTAAGGAGAA
GAAGAACTTCAGATAATAGAAGTTAGATGTCAAGAAACAAGAGCATCAAAGGCTACCTTCTCAGTTCTTCTG
GAAAGCGCCGATGGCTCATCCATTGTCCAACATCACGACGCCTGAGAGACATTTCTAACCTCCTGCGATTTAG
AGACTGCTGAAAGCTCTGCATATTTCCAATCAGGAGTGCAAAAAGTAGGAGTCCCGTTCCGATGATAGCAAT

TGTGAAAACAACTTCCAACAGAAAATAACTCGGGACTTGATTGCCAGCCAGTGTACTAATTTGCTGGAAGCC
CCAAAAGAATGAGTACACGTATCTTGTGACAACACTCTGTTTGGTGGTGAGATCAACTGCTTGTTTATAGATT
CCAAAGTCAAAACTACCTTCAGTAAAACAAGCAACTGCATCAGTATTGTTCTTCCACTGGTCCCATGTAATAT
CCGACCGAAACTTTGTGTAATCGGTGCCATGACCACAGTCTATGAACTCCATACACCTTTCTATGTTTGAGCT
GCGACAAGCACCTTGAAGGCATAGATTAACCCTCTGTAAACCAAAAAGATACCACAATGAGCCCACTATATT
GCTTGATACAACAATTATGAGAAGATTTATGACAAATGTTGCCCATATTGACTCAAATATAAAACCACTTGGA
GACTGGCCAGCAACCAAATAATAAATCCTGTACAACCTAGAATTGTACTGGAGTAGTATAGCTGCCCACAAT
AGATTCTTCGTGTAATTTACCCCAGATGATCCAATAGACTTGGGTAAAACCAACAAAATGATGATCTGAAGA
AGAATGTGCATTAAGTAAATGATATCAGTGATGCTCCTCAAAATCACAATAATTTTTGTCATGTGCCAATTTA
GCACTATGCATTTGTTCCCCTGTTGGACATTAAGCAAAAAGAAGAACAGGGGATCTATAAATAATGCAAATA
GGCATGAGATGACAAAAAATTTGTTCCACTTCTGGACAATCTTCGCATGAGGATTCATGACACCAGGAATAT
ATGGATACAAGAAAGAACAAGTTCTCTTCGCCCATCCTTTTGTATCTCCATACAGCATATTATGGAACCGGTG
ATCAAACATATCTGAAGACTTTGAACTTTTTTTTAGTGCTTTCAAATGGTTATCGGTTGGGCATGTTGTGCAGT
AAGGATCACTGCACATACCCAGTTGACCAGACTTCAATAGATGTTCATTTTTTCCAGTGTAGTCATTATTAGTC
CAACCATTTTGTTCTCCACTGCCAATTGAAGGATATTTTTCTGTTGTTGGCAGAGTTGGTTTGTGCACTACTGCA
GTGGGAGTGGACCGAGAGCTATTCTCAGGCTTGTGACTAATATATAGTGGCCCGCTCATATGAACCAATGAA
TTTCGCCCCTCACTTCTCAAAGGACCGGTATAGCCTACAAAAC
>TCONS_00037961
GCAATTTCATAATCCGAATTGAGGTTGAACATGGCGATGAAGAGGCCTTTGCAGTGTATGTTGAGAAGGATTT
ACATGAATGGAGGAAGTTTTAGAAGTTTTAGTGCACTTCCTGAAGGCAGCACCACAACTTCCCCTTCTCAAAA
TCTCATCGATTTGGAGTATGAGTGCAGTGCCCACAAATATTGCCTTGCAGGAAGGCGAAAAAGCTGGAGAAA
ATGACG
>TCONS_00037973
TCTTCTTCTTGGTCTTCACCTTCTTCTGCATTTTCTCTTCAAAAATGTCTCCATGAGCTACAATTTGAGATCTTTC
AGTATAACACAGAGCTGTGGAGCTGAGAAGAGTTCTGAACAAATACACAGTCCTTTGTTTCGAAAGCCTGCA
AAAAAAGAAGCACTATCTGTAGCTGAACGTGATATATGAAACCGCAAAAAACCCCTTTTTACACAAAACAC
ATAG
>TCONS_00038126
CAAAGTTATTATTGAATCAAATCAAATCAAGGGGAAATGACCCTTCAACCGAAAACACTTCGTTCCCGCAAA
TTTCCTTTTCTTTAACCTCACTCTCCGCCTCATTATTATTTATTATTCTTAAATATCAAGCAAAACCCAGTTCAA
GAAAGCTATATCAGATTGACATTCTTGCACATTTATTTCGAAAAAACAAAAATGGGTGTTTTTTCTGGTCATTG
GCCTTCTGAATCTGAGGAACGTAACTTAAAGGAACCTATACTTCTATGAACATAATATTCTTTTGAGCATATTC
CACATAAGCCAAGTGCAAATATCCTTGTATTGCGTTGTATGCAAGTATCAATAGTTAATCTTGGTGTGTTTGTA
TTTGAGAGAGAAACTAAAAATGCGGCAATAACTTTAGCTAAGTTGGTTTATCTTGTTTCACTGTAATCTCCCC
G
>TCONS_00038184
TATTATTGTTTTGATTTATTAAAAGGCGAGATTCTTTTGTGGGTCAATATGCCTGGTAAATTGGATAATAATAA
TGTACATTGGCGAAGTAATAATTAGTTGATGGAATCCATGTCTCGGTTTAGAGATTGATGATACACCTTTATG
AAAGCTTATAAGTTTTCATGTGTAAACCCGGCTGGAGGATTTTGTATCCGACACATGAAATAAGTTAAGCGAA
AGTCTAAA
>TCONS_00038187

CTCATTACACATTGAAAACTAAAATTTGCCGATTGGCATATCTTTCTGGCTTATGCATCTCCAAATTCCAATTA
GTTTCTCCTTCGCTAGGCCAGTTAAGAAGCACCAACGATGATGTTGTTGATAGGAATAAAAATCAACTTGACG
AAAAATCCAACAAACCCCATGACGACGAAACCGATGGCGGTACGAGTCGCAACCTTAGTGAATTCTTTGCG
ATCAGGCTTGTGACATCTTTTGACAAGTCTAACGCTGTCTTTGGCGAAATCTCTAAGAGGATCGAAAACAGAG
TCTAAGGCGTCCATTTTAGCGCTGAAAGAGGAGTGCGGATCTGGTTTGA
>TCONS_00038361
CTCACAACAGCTTTACAAGGCCATCAAACAATCAGAATTTTGAGAATGTTTTAAGATTCACTTTCCGGGCACA
AAGTTTGTGGCGTATGCCCAAGCGTTGTTGTTAACTGGGTCTGCAAGGTGGTCAGCAAGGTTCTCCAATGGAC
CTTTTCCGGTAACAATGGCCTGAACAAAGAATCCGAACATAGAGAACATGGCAAGTCTGCCATTCTTGATCT
CCTTTACTTTGAGCTCAGCGAATGCCTCTGGGTCATCAGCAAGGCCTAATGGGTCAAAGCTGCCACCAGGGTA
GAGTGGGTCGACAACCTCACCGAGAGGTCCACCAGCTACACGGTATCCCTCAACAGCTCCCATCAAGATAAC
TTGGCA
>TCONS_00038422
GGGTCTGATCCTTTTTTCACACTTCTTTTGCTCGATTGATTTGGATCAAACGTTATTGCAGGGGCAGGCGCTAT
ATTCAGCTGTGTGTGTAGATCCTGCATGTCAACAGCCTTTAGAATCCGAGAACTTCTGAGAAAAAACAACTTG
CCTTCTCCCCATGTGAGAGCAATATCTATACTTCCCAGAGCAGATACCATCTTCGTTTGAATATTTAAGAGCCC
AATACAAAGAATTAGAACCATCACCTTACAGGAAGAAGAGCACATATTTCTTCACTTGAAGAGGAATAGAA
GAGAGAATAAGTGCTTTGTCTGTGGCTATACTCTTGCATATTATCTTCCTAAGGAATTTATATATAAAAAACTT
GTGAGTCATCTTTAATGGGATTTGCAAAACTAAAGGTACTAAAGCAGGGAGGGACCCAGGGGCAAATATCTT
AAGAAGTTCAAGATTCACTTCAATCATGAGCCTGCTATAGAAGCAATAATATAAGGCTGTTTTTAGAAGACA
CTGACAGGTTTGAATAGAAAGTTAAAATTGGAACCATTCTTGGGAAGACAAGAATCTTAACATTTGTTCCCCA
CATCCCTATCATTTGTCTTGTTCTTTTGGACCAAACAATGACCAATCCAGTCTGTTTGAACTTTATGCCAACTC
ATCTGCCACCTCATCAGATTATGCTCACGGCTTGCTTGCT
>TCONS_00038430
TAACACATTCACCCTCAACTTCCATCCGTCATTAGTAATCTTAATTCGTTTTTCCTATTCACCAGTACGCTGCGT
GCTACAACTAGCAGCCATTTTATTGTCAATCGTTTATTGTAGCATTTACCCTAAAACACTGCATTGAATTTCAT
CCACATTATGCATGTCCTAAATTCTCAGCTGTGTGCAGGCAAGGATTGTCCCTCTGCACCTTGTCATTGGAGCA
ACCTTACGCCACACCAACCTCTCATTTCCAAGCGTCAATACATCAACATCAGACAGCACTTTGATGTTACAAT
TCCAATGCTCAGGTCCATTAACCCCTCCAACTATATAAATATCATCTCCAAGCCCTATCATAGCAAACCCAAT
TCTTCTACGAAACTCAGATGCTGAAACTACCATCTCTCTTACTTCTTTTTCCTGCTTGTAAATGAACCAATTGCT
CATAACATAAAGCTTTTCCCTAACGACAGTCATTGGACCCTGGAGCCAAGAATACTCGTGAACGGTCCAACC
TTGCTTGACATTTTCCAAAACCTGAATAGTCGATAAACCTTTATGCAAGACATGAACTTTACCGCCAACAACC
ACTCCTGAGCATGCGGAATTGTGCGCGTGATGGAGATCGGGTAATTGAACCCAGACATCCTTTTCTGGATCAT
AGATTTCCGCGTTACATATTGATTTCCTGTAGTTAGTAAAACCCCCTGCAACAATTATCTTATCCTCCAACACG
CAACAAGCAAACATGGCACGAGGGAAAATCATAGACGCACACCGTCTCCACACACGGGTTACAGGGTCATA
TGACCAGACCTCATCAGTCGCGAAAATCCCATCTTGGTCACCTGTCAATGGATCCACAGCATCACTGCCACCG
CCTAAAACAAACAGCTTTCCAGCAGTAGAAACAACACTGAAGCGAGCAAGATGACTGATGTTTGAGGGGAG
AATAGGGAGAGTAATCCAACGGTCATGCATAGGATCATAAAGCTGCGATAAGTTTTCAGGTTCAAATGCAAA
TACACATAAAAACTCTTCAGACGAGTTGACCTCCTTTCTCGCTTTAAATAGTTCGGTACTTCGAATGGCAGCTC
TCCAGGAATGGGAAACAAGCTCTAACTTTGGATGAAGGTGGAACGGAACCCGTGCAATGCACCTAAGGGCA
ACAGCATTGGGAAGACCTTCAATGAGTGCAGTCATAACCACCAAGCTTGCATCTATTTTTATCAGCAAATAAT

GATTGTGGTGCGCTACTGTTGGCTGACGTCAACCTGCTAGGTCAAGAAGATAGCAGTCGGCCTTGAGATTACA
GTTCAAAGAAGATAACATAAGAATTTGCTTGGCTTTTATCACAC
>TCONS_00038527
AAATTTGTACACTATTTCTGGTGGTCCTGAATAAACATGAAGGATTTCAATCGCGAATCCTCGTAAAAATATG
GATCTGAAAACTTTGTGTGATGATTCAAATGTCTCCTCATCAGGGTTGTAGAGTCGAACACTCTCTGGCAATG
ACGTTTGAAGGAATGTATTATAGCCACCTCCAAGTTTAGCATTTTCTTCTGGTGTCAACCCTTTCCTGCCATTG
ACACTCTTTGTGAATATTTTTGGATCAACAGTTTTGAAGTCATTAGGATCTGTCTTGTGGACCATTTCCATTTCC
CAAGTCTTCAATATCCTCTGCACTTTCTCTTCAAGTGATCCTTCAGGCCATACATGAGTTCTTTCTTCTTCAAAA
AGCTTATCAACAACATCATAGGTAGGAGGGCCATTTCTCCATTTTGTGTTTTTGACCTCTTCTTCACTCAAGTA
ATCTCTGTATTTGTTTCCTCCTCCTACTATACTTTCCATTTTGATTTGATCCTAACTAAAGAAAG
>TCONS_00038813
TCTGAGGATTCATATAAGAAGGGATAGTGTGGCTTTGTGAATTTGCAAGGCTTAGTGAGGTGTTTTGTGTTGA
ATATTTTAGGATAGGCTGTGGATAGAAGAGCTGCAGCTTCATTATAAGTTTGATTAGGCCTTTTTCGAGGGGTT
CGAGGTTTTCTAGTTGAAATTGCAATAGGAGAATTGCTGGAGTCTGAAAGAGTTGATGAAGGTGAAGATGAT
TGTGAAGTCCATGAATTAGATGGAGATTTTATAATCTCTAAATCCAATTTATAAGCTGCCCTTCCACCTCCAGT
TAAACAAGACATTGTACTAATGAGAAACAGAGAAGAAATTCAAGAATTTGAAATCTTGGAATTCAATGATG
AATTCTTGAAAACCCCATTAAGCAATTCAGACTTAATTCTC
>TCONS_00038814
TCTGAGGATTCATATAAGAAGGGATAGTGTGGCTTTGTGAATTTGCAAGGCTTAGTGAGGTGTTTTGTGTTGA
ATATTTTAGGATAGGCTGTGGATAGAAGAGCTGCAGCTTCATTATAAGTTTGATTAGGCCTTTTTCGAGGGGTT
CGAGGTTTTCTAGTGGAAATTGCAATAGGAGAATTGCTGGATTCTGAAAGAGTTGAAGAAGGTGAAGATGAT
TGTGAAGTCCATGAATTAGATGGAGATTTTATAATCTCTAAATCCAATTTATAAGCTGCCCTTCCACCTCCAGT
TAAACAAGACATTGTACTAATGAGAAACAGAGAAGAAATTCAAGAATTTGAAATCTTGGAATTCAATGATG
AATTCTTGAAAACCCCATTAAGCAATTCAGACTTAATTCTC
>TCONS_00038827
CTTCTACAAATCATTGCCAGTAATCCTCATATCAGCAAGCTCTATTTACGAGGGTGCACAGGCTTAACCATTG
AGGGAGTACTTGGAGCAGTGAAGTTACTTACCAAGGGTAACTACAACTTAACGAGCTTAAGTATTTCTGGCA
TCTATAATGTGAAGAGAGAGGACTTTGAGACACTTGGTCATCTAATGGGTATAAACCAGATGCAAAAGAAAG
AAGGAACAAAG
>TCONS_00038898
GCTTAACGTCAGGTTCCCTCCACATACATATGAAAAGTAAGCCAGCCGTATTAGCTCATCAACTCGCCTTAAC
GCACCCTCACTATATAGGTGCGGACTTGCGTACGGTATCACTTGCCTAACAGCTGCCAAAAGCGGAAGCTTC
AATGTTGACGGGCTTATTGTCTCCTTTCTTATCTTAAAAAAAGGGAAAATAAGAGAGGGATCACTCCTCGGGC
AGTCGCTAACCGTTGCGCCTTCCCTTCTGCTTACACCATTTCTTTGTGAATGCCCCGCTTTGCTCTTTGTCGCGC
TACTTCCGCTAGCAACCAGCTTCGGATTCTCCTTAAGGCACCGTGTTGAGTAGGGATTTGAATAGAAACTCTA
TTTTGGGGCTCAATGTTGCTGGACTCTACTCCTCTCCCCGGGCTCGCTCCGCTCTATGCCCAAAGCTTCAACTC
AATCACTTGGACAGAGGGATTTGACCTACCAACTGCAGGCTCGGGTGCGAGAACGGTATCCATTGCCTGCTG
CCCCCTCCCAATAATACGTGTTTATCAACCACCCGCTTACTACAAGAATGTATCATGATGAGAATCAAAGCA
GTCTTGTGAATGCCCAGTTCTCCTGTCTTCTTCGGGTCCTTAATAGAACTCAAAACTAGGTTTCTTCTGTATATA
AACTATATCTTTCTCTTCCTCAGAGATTGAATTGAAAGTCGACTGAATGTGATGAAAGGTTATATGCTTAACA
CATGCAAGTCGAATATAGTATGAAGGCTTTCTCTTCTAAAGGCAGGGGTGAGGACTCCATTGCGGCATTAAGT
AAGGAGTTGGAGGAAATCAAGGCGCATCCA

>TCONS_00038899
TACTACAAGAATGTATCATGATGAGAATCAAAGCAGTCTTGTGAATGCCCAGTTCTCCTGTCTTCTTCGGGTC
CTTAATAGAACTCAAAACTAGGTTTCTTCTGTATATAAACTATATCTTTCTCTTCCTCAGAGATTGAATTGAAA
GTCGACTGAATGTGATGAAAGGTTATATGCTTAACACATGCAAGTCGAATATAGTATGAAGGCTTTCTCTTCT
AAAGGCAGGGGTGAGGACTCCATTGCGGCATTAAGTAAGGAGTTGGAGGAAATCAAGGCGCATCCACTTCG
TCATCTCAGTCTCTTTTTGTGCAAAGAGGTAGAAAAGATTCTGCCCTTCTAGCCTCGAGTAGTGGACAATGTA
TCATTG
>TCONS_00038908
AAGTGAAAAATAACATAAGATTGCAAAATGAATAATGTACGAGCATAAGGTAACATAGAATACGACATTCA
GATATGAATTGAATGATTGGGGCATGTTGAAACACAAACCGTCATCAACGCCTTCCAGATGCCACTGATTCA
GTAGAGAAGAGATACTTCACTCGTGAAAGAACAGAGTCATGGGCAGGATCATAGGGATGGTCCTTTGAAGG
AATCTCCAGAATGGCTGGAATTGGTTTGTTGTAGCTATCAACCAAAAATCTTATCATGTTGGCAACATATTGG
CTGATTAACACGATTGCAATATCTTCTCTTGTGGTGAATTCCTTAAAAGCATCTTCAATCTGCTTCACCGTTGTT
TTTGAATCCACAATGAGGTAATTTGTTTTCCTCCTCAAATCAACATTGCCAACTCCAGCCAGTAAAAATCCAG
TAATTGTATCCTCATCAGCAATCATAGCAATAAGTGCAGAGTTGCTGGTTTTAATTGGAGCTCGGTTAGCCATT
TAACACTACAACAACGTTTCTGCAAATAACATAACATCACCCAATTAACAAACATTTAAAAGCATGAATCTT
GAACACATAAAACAAATTCAGATTTCAAATTGAATTCTAAATAGTTAAGAAAAAGTCATCAAACCAATCAA
AATCTTTACAATTCTAATAATACACAAAGTTTGATGAAACCCC
>TCONS_00038946
TTTAGCTTACCCAATGATGTTGATGGGTTAAGTGAAGAGAAAATGGTACTCATACCAGAGTAGTGATCAATA
GCACCGCTATCAATGATACATTGTCCACTACTCGAGGCTAGAAGGGCAGAATCTTTTCTACCTCTTTGCACAA
AAAGAGACTGAGATGACGAAGTGGATGCGCCTTGATTTCCTCCAACTCCTTACTTAATGCCGCAATGGAGTCC
TCACCCCTGCCTTTAGAAGAGAAAGCCTTCATACTATATTCGACTTGCATGTGTTAAGCATATAACCTTTCATC
ACATTCAGTCGACTTTCAATTCAATCTCTGAGGAAGAGAAAGATATAGTTTATATACAGAAGAAACCTAGTTT
TGAGTTCTATTAAGGACCCGAAGAAGACAGGAGAACTGGGCATTCACAAGACTGCTTTGATTCTCATCATGA
TACATTCTTGTAGTAAGCGGG
>TCONS_00038952
GTCATAGAACACAGTAATACTACCGAAATTAGTGTCTCACAGAGTTATTATAAATTTGTATTTATTACTGTCTT
CAATATTATAGGTGTAGTGATATTTCACAAATACATTTTCTCTGTCGAATAGAAAATTACATCTTTGAGTTTCC
CTGCAATGGCATCTTGATTTTCTTGACCTTGTTGATAAATGTTATCACAGTTTCCACACTTTCAGCATCTAAATT
TGGTACTTTGTGCCCCTCTGGGTGATGAATCACAACAGGATTAACGAAGCACTTCACAAGTTCTTCTTCGCTTG
TTTTTTTGGCTTCCTTCTCACCTATGAAGTGCAAAGATGGGGTCTCAATTGGAGATGAATATGCATTAGCAGCA
AGCTTAGGGCATCCAAATTCGGGCGCTCCAAACTTGAAGCCTGATATTATGATCACAAATTTTATATTTGGTA
CTTTGGTCAG
>TCONS_00038953
TGTGCATAGGATCATTGATAGTGGGTACTTTTGCTCTTGTATGCCTGTTGCTGTTGATACTAGAGACGGTATTC
ACTTGGTTTTCCAGGTAGAACCAAACCGGGAGTTCCATGGTTTGGTATGTGAAGGAGCCAATGTGCTTCCAGC
AAGGTTTATAGAGGATTCTTTTCAAGATGGATGTGGAAAAATTGTTAATATCAAGCGTATAGATGAAATAATA
AGCTCTATAAATGGTTGGTACATGGAGCGGGGTCTTTTTGGAGCGG
>TCONS_00039219
CTTTCCATATATATTGGTCCTAGTAGGACAAGCCCCTGAAGCTGGTAATAACCTGACGACAGTTACATGTTCA
CCTCTGGAAGCATGATGGAGAGGCGTATCACCTTCTACATCAACAAAGTCCAACATCCTCTTCGCACATTCAG

GGTCAGGAGCGCTGTTGATCAGCAGTTGGGCTATCTCCGTGTATCCGCCAGCACAAGCATCATGCAATGGGA
TTGCTCCATCTTCATCCTTAGGCTCCAAAGAAGCTCCTCTTTCTAGCAGTAGCTGCACACAAGGGAGATGGCC
ATACAAACAT
>TCONS_00039240
GGGGCAAGGTTCTTCCAGAAAACTGAGTGATTAATATGACCTCCGCCATTGAACTTGATAGCACTCTGCAATT
TAGCGACGGTAGGAGAATCACCCTTAGTGATGGCATCATGGAGCTGTTCAAGGGCTTTATTGTAATTTGTAAT
GTAAGTCTGATGATGTTTCTGGTGATGAATCTGCATGATCTCACAGCTAATAGCAGGTTCTAAGGCACCATAA
TCGTAAGGGAGATCGGGGAGTGAGAAAGTCTGTACGCCGCGGAGTTGTTGCCGGAACCCTAG
>TCONS_00039241
CTCCAATATCCATCACCACAAACTTCATTTTCCAGATGCTCAGAAACATACTAATCAATTCTGCAACAGTTCA
TTCGCTGCCTTCAACCATGCATCATAAATTTCATCTGAAAACAGCACAAAGTGTACCTCTTTGAGCTCACTGC
CAAATTCTTTGACAGTAGATATAGCCACAGTGGCAGCTTCATCAAAAGGATATCCGAAGACCCCACATGATA
TGGCAGGAAAAGCAATATACTGAATGTTGTTCTCATTTGCCACACGCAGGCTATTTCTATATGAATTCGTCAA
ATAGGCTTTAGGATTTGGGACAGCATCATATATTGGTCCAACAGTGTGGATTACATGAGAAGCTGGCAATCTA
AAACCTGGTGTAATCCTTGCTTCTCCTGTTGGGCAGCGAACTCCAGGACGTACCTCTGGGACTTTGTAGCATG
CATCTCGTAATTCTGGACCAGCAGCTCGATGTATGGCTCCATCAGCACCACCACCACCCAGCATTCGCTCATT
GGCAGGATTGACAATAGCGTCAGAGGATCCGTCTACGGACCATTGAGTGATATCACCTTTTTGAATCTTCAAC
AAGCTCGACGGAGTTAACTGAAACGTAACG
>TCONS_00039293
TTGCAAGCCTTGTTTGCTCATGAATTTTTTTATCATCTCAGCCTTTGTCCTACCATCAAGATTAGCAATATCAC
TAGTAGGTAATGGTAATAAATCAAGTGGAGTAAAGGGATTAAAGCCATAAACAACCTCAAAAGGAGACATA
CCTGTAGAAGAATGGATTGTTCTATTGTAAGCAAATTCAATTATAGGTAAGTGATCTTCCCATGAAGTTAATTT
ACCTTTCAAAACAGCCCTCAACATGTTT
>TCONS_00039525
CTTATTCAGGGGGAGCCTCGAAAAGCACTGTTGAGAGGAAGATCCTTGGCCCCTCTTCATTCTCTACAGGGTT
CCAAACCTTTCTTCAACATAGGTGACAACGAGCCAGGCAGAGATGGAAGAGATCAAACACGGGAATAAGAA
GCAAGCTTGCCTTCCTTTTTGATCATTTTGATAGAGGGGGATGAAGAAAGTGGACAAAACAGACTCGCATTTG
TCATCGGACAAATACAGGAAAAGAATCATATTGAAAACGCTCCTAACCCAACCCCTTCCTTCGTAGAGCCCG
TGTATTGTAAGTGATCCGAACCTGCCCGGAGCGAGCCTCCCATAGAGGCAAGTGAAGTTGGTGAGCCGTATG
ATGGGCAACTATCTCCTGCGGTTCGGAGAGGACTCAGCTTTAGTTAGTACCCCCTTGGTTTCGGGGTGGACCT
TTTCACTCTATTTTATTATATACGCTTAGCGAAAAGAATGTTTTTTGATACACCTAGGACATGGATTCTATATG
AACCAATGGATCGTGACAAGTCGTTAGTACTAGCAATGACTTCCTCTTTCATTACTTCATTCTTTCCATATCCC
TCTCCTTTGTTC
>TCONS_00039527
GGATTTAGAAGGCTTGTTCAAAAGAAAAAAAGACGTGGGTGGCATTATGACTGGGGTGTCAGTTAGAAAGGT
TCTTACAATGTTCGTTCAATCAAGCAGCATTCTTTTTTTCTTAACTTAAGGAGAGGGAAGAGGGCTTTCTAAGA
AGGGCTTGGGACCTATGAACAGAACAACACAACAAAATGATGAAATGAAGAAGAATGAACCGGAGGTATC
GGAGGACG
>TCONS_00039615
CACCAACTTCACTTGCCTCTATGGGAGGCTCGCTCCGGGCAGGTTCGGATCACTTACAATACACGGGCTCTAC
GAAGGAAGGGGTTGGGTTAGGAGCGTTTTCAATATGATTCTTTTCCTGTATTTGTTCGATGAGAAATGCAAGT
GGCTCACCAACTTCACTTGCCTCTATGGGAGGCTCGCTCCGGGCAGGTTCGGATCACTTACAATACACGGGCT

CTACGAAGGAAGGGGTTGGGTTAGGAGCGTTTTCAATATGATTCTTTTCCTGTATTTGTCCGATGACAAATGC
GAGTCTGTTTTGTCCACTTTCTTCATCCCCCTCTATCAAAATGATCAAAAAGGAAGGCAAGCTTGCTTCTTATT
CCCGTGTTTGATCTCTTCCATCTCTGCCTGGCTCGTTGTCACCTATGTTGAAGAAAGGTTTGGAACCCTGTAGA
GAATGAAGAGGGGCCAAGGATCTTCCTCTCAACAGTGCTTTTCGAGGCTCCCCCT
>TCONS_00039616
TCACCAACTTCACTTGCCTCTATGGGAGGCTCGCTCCGGGCAGGTTCGGATCACTTACAATACACGGGCTCTA
CGAAGGAAGGGGTTGGGTTAGGAGCGTTTTCAATATGATTCTTTTCCTGTATTTGTCCGATGACAAATGCGAG
TCTGTTTTGTCCACTTTCTTCATCCCCCTCTATCAAAATGATCAAAAAGGAAGGCAAGCTTGCTTCTTATTCCC
GTGTTTGATCTCTTCCATCTCTGCCTGGCTCGTTGTCACCTATGTTGAAGAAAGGTTTGGAACCCTGTAGAGAA
TGAAGAGGGGCCAAGGATCTTCCTCTCAACAGTGCTTTTCGAGGCTCCCCCT
>TCONS_00039617
GACTTGTCACGATCCATTGGTTCATATAGAATCCATGTCCTAGGTGTATCAAAAAACATTCTTTTCGCTAAGCG
TATATAATAAAATAGAGTGAAAAGGTCCACCCCGAAACCAAGGGGGTACTAACTAAAGCTGAGTCCTCTCC
GAACCGCAGGAGATAGTTGCCCATCATACGGCTCACCAACTTCACTTGCCTCTATGGGAGGCTCGCTCCGGG
CAGGTTCGGATCACTTACAATACACGGGCTCTACGAAGGAAGGGGTTGGGTTAGGAGCGTTTTCAATATGAT
TCTTTTCCTGTATTTGTCCGATGACAAATGCGAGTCTGTTTTGTCCACTTTCTTCATCCCCCTCTATCAAAATGA
TCAAAAAGGAAGGCAAGCTTGCTTCTTATTCCCGTGTTTGATCTCTTCCATCTCTGCCTGGCTCGTTGTCACCT
ATGTTGAAGAAAGGTTTGGAACCCTGTAGAGAATGAAGAGGGGCCAAGGATCTTCCTCTCAACAGTGCTTTT
CGAGGCTCCCCCT
>TCONS_00039618
TGCCATTTGATGAGTAACTGTGAACAAAGGAGAGGGATATGGAAAGAATGAAGTAATGAAAGAGGAAGTCA
TTGCTAGTACTAACGACTTGTCACGATCCATTGGTTCATATAGAATCCATGTCCTAGGTGTATCAAAAAACAT
TCTTTTCGCTAAGCGTATATAATAAAATAGAGTGAAAAGGTCCACCCCGAAACCAAGGGGGTACTAACTAAA
GCTGAGTCCTCTCCGAACCGCAGGAGATAGTTGCCCATCATACGGCTCACCAACTTCACTTGCCTCTATGGGA
GGCTCGCTCCGGGCAGGTTCGGATCACTTACAATACACGGGCTCTACGAAGGAAGGGGTTGGGTTAGGAGCG
TTTTCAATATGATTCTTTTCCTGTATTTGTCCGATGACAAATGCGAGTCTGTTTTGTCCACTTTCTTCATCCCCCT
CTATCAAAATGATCAAAAAGGAAGGCAAGCTTGCTTCTTATTCCCGTGTTTGATCTCTTCCATCTCTGCCTGGC
TCGTTGTCACCTATGTTGAAGAAAGGTTTGGAACCCTGTAGAGAATGAAGAGGGGCCAAGGATCTTCCTCTC
AACAGTGCTTTTCGAGGCTCCCCCT
>TCONS_00039619
CTCCGGTTCATTCTTCTTCATTTCATCATTTTGTTGTGTTGTTCTGTTCATAGGTCCCAAGCCCTTCTTAGAAAGC
CCCAAGTCAGTAAAGTAGTTAACCAAACACAAGTAAGAAGTTCGTCAGTCCTTCCTCCGATGCCTCCCGTTCA
TTCTTCTTCATTTCATCATTTTGTTGTGTTGTTCTGTTCATAGGTCCCAAGCCCTTCTTAGAAAGCCCTCTTCCCT
CTCCTTAAGTTAAGAAAAAAAGAATGCTGCTTGATTGAACGAACATTGTAAGAACCTTTCTAACTGACACCC
CAGTCATAATGCCACCCACGTCTTTTTTTCTTTTGAACAAGCCTTCTAAATCC
>TCONS_00039620
CTCCGATACCTCCGGTTCATTCTTCTTCATTTCATCATTTTGTTGTGTTGTTCTGTTCATAGGTCCCAAGCCCTTC
TTAGAAAGCCCTCTTCCCTCTCCTTAAGTTAAGAAAAAAAGAATGCTGCTTGATTGAACGAACATTGTAAGA
ACCTTTCTAACTGACACCCCAGTCATAATGCCACCCACGTCTTTTTTTCTTTTGAACAAGCCTTCTAAATCC
>TCONS_00039660
GCGGTTCCCTCCGGTCCGTTCCCAGGTTTGGTTAGGGGAACATCCGAGCGATTGCCTTCGCGGATCCAAACGC
GCTCACAAGGCGCACAATAAGAATAAGACCAATAGAGACTTCATAAGGGACCATTTGAGCTGCAGATCGTA

ATGCTCCTAGAGAGGCATATTTCGAATACAGAGGAGTGAAAGTTCCCAACAAATAAGTACTTTTTCATATACT
TCTATGAACCGTACGAGTGCGTGCTCTGTCACCCCCCATCGCGCTTACGGCTCTATACCCCAACCCAACTTGC
TCTGGCCCCGGTGAAAGTTCCCAACAAATAAGTACTTTTTCATATCCTTCTATGAACCGTACGAGCACGTCCT
CTGTCACCCCCCACCGCGCTTACGGCTCTATACCCCAACCCAACTTGCTCTGGCCCCGGGTCCTTCCTTTCTAT
TCCTCGGTAAGCGGACCGTGGGAGCATGGGGGGCGATATCCGCTTTTGACTGATAGAAAGCATCTCTCTTTTG
TCCCTCCTGACCGAACTCGCCAAAAAAATCACAATAGAAAAAGTTGTTCTTGCCGGGAGAGTAGCGTCGGAG
ACATCTTTATCTGAGGAGGAGGCTTCGTCGTCCGGCTCCTCATAAATGATGTTAGGATCGGCCGGCTCATCCT
CGGAATCCGAAAAGAAAACAGAGACAGAACGGAATCACTTTGTTTCAGTGACATATCTAATCAGACTGAAT
AGGATTTGAAGAGCTGTCACGATCATTCACAAAAATTTCAGTGAACTATTGAAGCTATCAGTGTGATTGATCA
GACAAGTACCAAGGGCTTTCGGTAGACTTCTTTC
>TCONS_00039661
GCGGTTCCCTCCGGTCCGTTCCCAGGTTTGGTTAGGGGAACATCCGAGCGATTGCCTTCGCGGATCCAAACGC
GCTCACAAGGCGCACAATAAGAATAAGACCAATAGAGACTTCATAAGGGACCATTTGAGCTGCAGATCGTA
ATGCTCCTAGAGAGGCATATTTCGAATACAGAGGAGTGAAAGTTCCCAACAAATAAGTACTTTTTCATATCCT
TCTATGAACCGTACGAGCACGTCCTCTGTCACCCCCCACCGCGCTTACGGCTCTATACCCCAACCCAACTTGC
TCTGGCCCCGGGTCCTTCCTTTCTATTCCTCGGTAAGCGGACCGTGGGAGCATGGGGGGCGATATCCGCTTTTG
ACTGATAGAAAGCATCTCTCTTTTGTCCCTCCTGACCGAACTCGCCAAAAAAATCACAATAGAAAAAGTTGTT
CTTGCCGGGAGAGTAGCGTCGGAGACATCTTTATCTGAGGAGGAGGCTTCGTCGTCCGGCTCCTCATAAATGA
TGTTAGGATCGGCCGGCTCATCCTCGGAATCCGAAAAGAAAACAGAGACAGAACGGAATCACTTTGTTTCAG
TGACATATCTAATCAGACTGAATAGGATTTGAAGAGCTGTCACGATCATTCACAAAAATTTCAGTGAACTATT
GAAGCTATCAGTGTGATTGATCAGACAAGTACCAAGGGCTTTCGGTAGACTTCTTTC
>TCONS_00039662
GCTGCGGTTCCCTCCGGTCCGTTCCCAGGTTTGGTTAGGGGAACATCCGAGCGATTGCCTTCGCGGATCCAAA
CGCGCTCACAAGGCGCACAATAAGAATAAGACCAATAGAGACTTCATAAGGGACCATTTGAGCTGCAGATC
GTAATGCTCCTAGAGAGGCATATTTCGAATACAGAGGAGTGAAAGTTCCCAACAAATAAGTATTTTTCATATC
CTTCTATGAACCGTACGAGCACGTCCTCTGTCACCCCCCACCGCGCTTACGGCTCTATACCCCAACCCAACTT
GCTCTGGCCCCGGGTCCTTCCTTTCTATTCCTCGGTAAGCGGACCGTGGGAGCATGGGGGGCGATATCCGCTT
TTGACTGATAGAAAGCATCTCTCTTTTGTCCCTCCTGACCGAACTCGCCAAAAAAATCACAATAGAAAAAGTT
GTTCTTGCCGGGAGAGTAGCGTCGGAGACATCTTTATCTGAGGAGGAGGCTTCGTCGTCCGGCTCCTCATAAA
TGATGTTAGGATCGGCCGGCTCATCCTCGGAATCCGAAAAGAAAACAGAGACAGAACGGAATCACTTTGTTT
CAGTGACATATCTAATCAGACTGAATAGGATTTGAAGAGCTGTCACGATCATTCACAAAAATTTCAGTGAAC
TATTGAAGCTATCAGTGTGATTGATCAGACAAGTACCAAGGGCTTTCGGTAGACTTCTTTC
>TCONS_00039663
ACAACTCAAAATGAGCCTTGCGATGAGACGCGGACATTCCCCATGCTGCGGTTCCCTCCGGTCCGTTCCCAGG
TTTGGTTAGGGGAACATCCGAGCGATTGCCTTCGCGGATCCAAACGCGCTCACAAGGCGCACAATAAGAATA
AGACCAATAGAGACTTCATAAGGGACCATTTGAGCTGCAGATCGTAATGCTCCTAGAGAGGCATATTTCGAA
TACAGAGGAGTGAAAGTTCCCAACAAATAATACTTTTTCATATCCTTCTATGAACCGTACGAGCACGTCCTCT
GTCACCCCCCACCGCGCTTACGGCTCTATACCCCAACCCAACTTGCTCTGGCCCCGGGTCCTTCCTTTCTATTC
CTCGGTAAGCGGACCGTGGGAGCATGGGGGGCGATATCCGCTTTTGACTGATAGAAAGCATCTCTCTTTTGTC
CCTCCTGACCGAACTCGCCAAAAAAATCACAATAGAAAAAGTTGTTCTTGCCGGGAGAGTAGCGTCGGAGA
CATCTTTATCTGAGGAGGAGGCTTCGTCGTCCGGCTCCTCATAAATGATGTTAGGATCGGCCGGCTCATCCTC
GGAATCCGAAAAGAAAACAGAGACAGAACGGAATCACTTTGTTTCAGTGACATATCTAATCAGACTGAATA

GGATTTGAAGAGCTGTCACGATCATTCACAAAAATTTCAGTGAACTATTGAAGCTATCAGTGTGATTGATCAG
ACAAGTACCAAGGGCTTTCGGTAGACTTCTTTC
>TCONS_00039664
GCACTCATCAGTCAGCTCGGCCCGGCCGTCCCATCTCATCTTGATTTGGCTATACTTGTATGTAGCCAGCCATG
TTAGCTCCACATGAGAGCCTTTTAAATAAAGGCTAAAAAGGCACTCATCAGTCAGCTCGGCCCCTTTCCTATT
TCGCTTTGCCGCCTATTAAGAGAATAGGTCCCGAAAAAGCGGCCCGCCTTTCATCATCTATACCAGCTGCCAC
GCAAGAACCAATAGAAAGGATTTCGACGCCCCTCTCGTCATAAGAAGCAGAACGCGCCATCACCTACAGCC
CTTTCCTCTGCCGGGGACTTCCACGAAATGAAAACGGGCGGCATCGTCGTATGCTCCACTTTTGTTGCCCTGGT
TTATATCCCGGAGTACTCCTATGGCCGATCTGTCACCCAACCTACTTAAACAACCTAAAGGCTTGGCCCCGTC
CCGTTTGGAGAATGACCCCTTATGGCACGGCTTAGTCAGTTATTCTCGCAGTTCAGATAAGATTCGGACCGGG
TTGGGTTAGGTCTCTGAAAGCATGGTTCTTGCCTCCCTCCCCCCTTTGAGCTCGTCCATTCGTTGGGTGATCCCT
TGCTCTCACGTTCGAGTGTTTGCTCGTCGTTTAGGCCCGTGAGTGAGATTTCTGCTCATTGCAGTCACCTCCGG
GGTTCTTCGCACCTGGATAGTACCAGGACCCTTGTGCCCCGGCCCTTGATCAGATAAAGCTGCCCGCCTTATG
ACCCACAAACTAAAAATGAGCCTTGCGACGAGACGCGGACATTCCCCATGCTGCGGTTCCCTCCGGTCCGTT
CCCGGGTTGGGTTAGGGGAACATCCGAGCGATTGCCTTCGCGGATCCAAACGCGCTCACAAGGCGCACAATA
AGAATAAGACCAATAGAGACTTCATAAGGGACCATTTGAGCTGCAGATCGTAATGCTCCTAGAGAGGCATAT
TTCGAATATAGAGGAGTGAAAGTTCCCAACAAATAAGTACTTTTTCATATCCTTCTATGAACCGTACGAGCAC
GTCCTCTGTCACCCCCCACCGCGCTTACGGCTCTATACCCCAACCCAACTTGCTCTGGCCCCGGGTCCTTCCTT
TCTATTCCTCGGTAAGCGGACCGTGGGAGCATGGGGGGCGATATCCGCTTTTGACTGATAGAAAGCATCTCTC
TTTTGTCCCTCCTGACCGAACTCGCCAAAAAAATCACAATAGAAAAAGTTGTTCTTGCCGGGAGAGTAGCGT
CGGAGACATCTTTATCTGAGGAGGAGGCTTCGTCGTCCGGCTCCTCATAAATGATGTTAGGATCGGCCGGCTC
ATCCTCGGAATCCGAAAAGAAAACAGAGACAGAACGGAATCACTTTGTTTCAGTGACATATCTAATCAGACT
GAATAGGATTTGAAGAGCTGTCACGATCATTCACAAAAATTTCAGTGAACTATTGAAGCTATCAGTGTGATTG
ATCAGACAAGTACCAAGGGCTTTCGGTAGACTTCTTTC
>TCONS_00039665
AGCCTTTTAAATAAAGGTTAAAACGGCACTCATCAGTCAGCTCGGCCCCTTTCCTATTTCGCTTTGCCGCCTAT
TAAGAGAATAGGTCCCGAAAAAGCGGCCCGCCTTCTATCATCTATACCAGCTTCCACGCAAGAACCAATAGA
AAGGATTTCGACGCCCCTCTCGTCATAAGAAGCAGAACGCGCCATCACCTACAGCCCTTTCCTCTGCCGGGG
GCTTCCATGAAATGAAAACGGGCGGCATCGTCGTATGCTCCACTTTTGTTGCCCTGGTTTATATTCCGGAGTAC
TCCTATGGCTGATCTGTCACCCAACCTACTTAAACAACCTAAAGGCTTGGCCCCGTCCCGTTTGGAGAATGAA
CCCTTATGGCACGGCTTAGTCAGTTATTCTCGCAGTTCAGATAAGATTCGGACCGGGTTGGGTTAGGTCTCTG
AAAGCATGGTTCTTGCCTCCCTCCCCCCTTTGAGCTCGTCCATTCGTTGGGTGATCCCTTGCTCTCACGTTCGA
GTGTTTGCTCGTCGTTTAGGCCCGTGAGTGAGATTTCTGCTCATTGCAGTCACCTCCGGGGTTCTTCGCACCTG
GATAGTACCAGGACCCTTGTGCCCCGGCCCTTGATCAGATAAAGCTGCCCGCCTTATGACCCACAAACTAAA
AATGAGCCTTGCGACGAGACGCGGACATTCCCCATGCTGCGGTTCCCTCCGGTCCGTTCCCGGGTTGGGTTAG
GGGAACATCCGAGCGATTGCCTTCGCGGATCCAAACGCGCTCACAAGGCGCACAATAAGAATAAGACCAAT
AGAGACTTCATAAGGGACCATTTGAGCTGCAGATCGTAATGCTCCTAGAGAGGCATATTTCGAATATAGAGG
AGTGAAAGTTCCCAACAAATAAGTACTTTTTCATATCCTTCTATGAACCGTACGAGCACGTCCTCTGTCACCC
CCCACCGCGCTTACGGCTCTATACCCCAACCCAACTTGCTCTGGCCCCGGGTCCTTCCTTTCTATTCCTCGGTA
AGCGGACCGTGGGAGCATGGGGGGCGATATCCGCTTTTGACTGATAGAAAGCATCTCTCTTTTGTCCCTCCTG
ACCGAACTCGCCAAAAAAATCACAATAGAAAAAGTTGTTCTTGCCGGGAGAGTAGCGTCGGAGACATCTTTA
TCTGAGGAGGAGGCTTCGTCGTCCGGCTCCTCATAAATGATGTTAGGATCGGCCGGCTCATCCTCGGAATCCG

AAAAGAAAACAGAGACAGAACGGAATCACTTTGTTTCAGTGACATATCTAATCAGACTGAATAGGATTTGAA
GAGCTGTCACGATCATTCACAAAAATTTCAGTGAACTATTGAAGCTATCAGTGTGATTGATCAGACAAGTACC
AAGGGCTTTCGGTAGACTTCTTTC
>TCONS_00039666
AGCCTTTTAAATAAAGGTTAAAACGGCACTCATCAGTCAGCTCGGCCCCTTTCCTATTTCGCTTTGCCGCCTAT
TAAGAGAATAGGTCCCGAAAAAGCGGCCCGCCTTCTATCATCTATACCAGCTTCCACGCAAGAACCAATAGA
AAGGATTTCGACGCCCCTCTCGTCATAAGAAGCAGAACGCGCCATCACCTACAGCCCTTTCCTCTGCCGGGG
GCTTCCATGAAATGAAAACGGGCGGCATCGTCGTATGCTCCACTTTTGTTGCCCTGGTTTATATTCCGGAGTAC
TCCTATGGCTGATCTGTCACCCAACCTACTTAAACAACCTAAAGGCTTGGCCCCGTCCCGTTTGGAGAATGAC
CCCTTATGGCACGGCTTAGTCAGTTATTCTCGCAGTTCAGATAAGATTCGGACCGGGTTGGGTTAGGTCTCTG
AAAGCATGGTTCTTGCCTCCCTCCCCCCTTTGAGCTCGTCCATTCGTTGGGTGATCCCTTGCTCTCACGTTCGA
GTGTTTGCTCGTCGTTTAGGCCCGTGAGTGAGATTTCTGCTCATTGCAGTCACCTCCGGGGTTCTTCGCACCTG
GATAGTACCAGGACCCTTGTGCCCCGGCCCTTGATCAGATAAAGCTGCCCGCCTTATGACCCACAAACTAAA
AATGAGCCTTGCGACGAGACGCGGACATTCCCCATGCTGCGGTTCCCTCCGGTCCGTTCCCGGGTTGGGTTAG
GGGAACATCCGAGCGATTGCCTTCGCGGATCCAAACGCGCTCACAAGGCGCACAATAAGAATAAGACCAAT
AGAGACTTCATAAGGGACCATTTGAGCTGCAGATCGTAATGCTCCTAGAGAGGCATATTTCGAATATAGAGG
AGTGAAAGTTCCCAACAAATAAGTACTTTTTCATATCCTTCTATGAACCGTACGAGCACGTCCTCTGTCACCC
CCCACCGCGCTTACGGCTCTATACCCCAACCCAACTTGCTCTGGCCCCGGGTCCTTCCTTTCTATTCCTCGGTA
AGCGGACCGTGGGAGCATGGGGGGCGATATCCGCTTTTGACTGATAGAAAGCATCTCTCTTTTGTCCCTCCTG
ACCGAACTCGCCAAAAAAATCACAATAGAAAAAGTTGTTCTTGCCGGGAGAGTAGCGTCGGAGACATCTTTA
TCTGAGGAGGAGGCTTCGTCGTCCGGCTCCTCATAAATGATGTTAGGATCGGCCGGCTCATCCTCGGAATCCG
AAAAGAAAACAGAGACAGAACGGAATCACTTTGTTTCAGTGACATATCTAATCAGACTGAATAGGATTTGAA
GAGCTGTCACGATCATTCACAAAAATTTCAGTGAACTATTGAAGCTATCAGTGTGATTGATCAGACAAGTACC
AAGGGCTTTCGGTAGACTTCTTTC
>TCONS_00039667
GCCATGTTAGCTCCACATGAGAGCCTTTTAAATAAAGGCTAAAAAGGCACTCATCAGTCAGCTCGGCCCCTTT
CCTATTTCGCTTTGCCGCCTATTAAGAGAATAGGTCCCGAAAAAGCGGCCCGCCTTTCATCATCTATACCAGC
TGCCACGCAAGAACCAATAGAAAGGATTTCGACGCCCCTCTCGTCATAAGAAGCAGAACGCGCCATCACCT
ACAGCCCTTTCCTCTGCCGGGGACTTCCACGAAATGAAAACGGGCGGCATCGTCGTATGCTCCACTTTTGTTG
CCCTGGTTTATATCCCGGAGTACTCCTATGGCCGATCTGTCACCCAACCTACTTAAACAACCTAAAGGCTTGG
CCCCGTCCCGTTTGGAGAATGACCCCTTATGGCACGGCTTAGTCAGTTATTCTCGCAGTTCAGATAAGATTCG
GACCGGGTTGGGTTAGGTCTCTGAAAGCATGGTTCTTGCCTCCCTCCCCCCTTTGAGCTCGTCCATTCGTTGGG
TGATCCCTTGCTCTCACGTTCGAGTGTTTGCTCGTCGTTTAGGCCCGTGAGTGAGATTTCTGCTCATTGCAGTCA
CCTCCGGGGTTCTTCGCACCTGGATAGTACCAGGACCCTTGTGCCCCGGCCCTTGATCAGATAAAGCTGCCCG
CCTTATGACCCACAAACTAAAAATGAGCCTTGCGACGAGACGCGGACATTCCCCATGCTGCGGTTCCCTCCG
GTCCGTTCCCGGGTTGGGTTAGGGGAACATCCGAGCGATTGCCTTCGCGGATCCAAACGCGCTCACAAGGCG
CACAATAAGAATAAGACCAATAGAGACTTCATAAGGGACCATTTGAGCTGCAGATCGTAATGCTCCTAGAG
AGGCATATTTCGAATATAGAGGAGTGAAAGTTCCCAACAAATAAGTACTTTTTCATATCCTTCTATGAACCGT
ACGAGCACGTCCTCTGTCACCCCCCACCGCGCTTACGGCTCTATACCCCAACCCAACTTGCTCTGGCCCCGGG
TCCTTCCTTTCTATTCCTCGGTAAGCGGACCGTGGGAGCATGGGGGGCGATATCCGCTTTTGACTGATAGAAA
GCATCTCTCTTTTGTCCCTCCTGACCGAACTCGCCAAAAAAATCACAATAGAAAAAGTTGTTCTTGCCGGGAG
AGTAGCGTCGGAGACATCTTTATCTGAGGAGGAGGCTTCGTCGTCCGGCTCCTCATAAATGATGTTAGGATCG

GCCGGCTCATCCTCGGAATCCGAAAAGAAAACAGAGACAGAACGGAATCACTTTGTTTCAGTGACATATCTA
ATCAGACTGAATAGGATTTGAAGAGCTGTCACGATCATTCACAAAAATTTCAGTGAACTATTGAAGCTATCA
GTGTGATTGATCAGACAAGTACCAAGGGCTTTCGGTAGACTTCTTTC
>TCONS_00039668
GCCATGTTAGCTCCACATGAGAGCCTTTTAAATAAAGGTTAAAACGGCACTCATCAGTCAGCTCGGCCCCTTT
CCTATTTCGCTTTGCCGCCTATTAAGAGAATAGGTCCCGAAAAAGCGGCCCGCCTTCTATCATCTATACCAGC
TTCCACGCAAGAACCAATAGAAAGGATTTCGACGCCCCTCTCGTCATAAGAAGCAGAACGCGCCATCACCTA
CAGCCCTTTCCTCTGCCGGGGACTTCCACGAAATGAAAACGGGCGGCATCGTCGTATGCTCCACTTTTGTTGC
CCTGGTTTATATCCCGGAGTACTCCTATGGCCGATCTGTCACCCAACCTACTTAAACAACCTAAAGGCTTGGC
CCCGTCCCGTTTGGAGAATGACCCCTTATGGCACGGCTTAGTCAGTTATTCTCGCAGTTCAGATAAGATTCGG
ACCGGGTTGGGTTAGGTCTCTGAAAGCATGGTTCTTGCCTCCCTCCCCCCTTTGAGCTCGTCCATTCGTTGGGT
GATCCCTTGCTCTCACGTTCGAGTGTTTGCTCGTCGTTTAGGCCCGTGAGTGAGATTTCTGCTCATTGCAGTCA
CCTCCGGGGTTCTTCGCACCTGGATAGTACCAGGACCCTTGTGCCCCGGCCCTTGATCAGATAAAGCTGCCCG
CCTTATGACCCACAAACTAAAAATGAGCCTTGCGACGAGACGCGGACATTCCCCATGCTGCGGTTCCCTCCG
GTCCGTTCCCGGGTTGGGTTAGGGGAACATCCGAGCGATTGCCTTCGCGGATCCAAACGCGCTCACAAGGCG
CACAATAAGAATAAGACCAATAGAGACTTCATAAGGGACCATTTGAGCTGCAGATCGTAATGCTCCTAGAG
AGGCATATTTCGAATATAGAGGAGTGAAAGTTCCCAACAAATAAGTACTTTTTCATATCCTTCTATGAACCGT
ACGAGCACGTCCTCTGTCACCCCCCACCGCGCTTACGGCTCTATACCCCAACCCAACTTGCTCTGGCCCCGGG
TCCTTCCTTTCTATTCCTCGGTAAGCGGACCGTGGGAGCATGGGGGGCGATATCCGCTTTTGACTGATAGAAA
GCATCTCTCTTTTGTCCCTCCTGACCGAACTCGCCAAAAAAATCACAATAGAAAAAGTTGTTCTTGCCGGGAG
AGTAGCGTCGGAGACATCTTTATCTGAGGAGGAGGCTTCGTCGTCCGGCTCCTCATAAATGATGTTAGGATCG
GCCGGCTCATCCTCGGAATCCGAAAAGAAAACAGAGACAGAACGGAATCACTTTGTTTCAGTGACATATCTA
ATCAGACTGAATAGGATTTGAAGAGCTGTCACGATCATTCACAAAAATTTCAGTGAACTATTGAAGCTATCA
GTGTGATTGATCAGACAAGTACCAAGGGCTTTCGGTAGACTTCTTTC
>TCONS_00039669
CTGAGCCATTCCCATCCCAAGTGGTGCCCCGATTCGGCCTGGCCGGTCGGGAAGAGATTCACATAGAGACTA
CTGCTATCCGGGAATCTCTTTCTATGTTAGCTAGCGGGGGCGGGCTTTCCTATGGCAGTCCCGCTGCGGCCTAT
GAACGGCCGTCCCATCTCATCTTGATTTGTCTATACTTGTATATAGCCAGCCATGTTAGCTCCACATGAGAGCC
TTTTAAATAAAGGTTAAAACGGCACTCATCAGTCAGCTCGGCCCCTTTCCTATTTCGCTTTGCCGCCTATTAAG
AGAATAGGTCCCGAAAAAGCGGCCCGCCTTTCATCATCTATACCAGCTGCCACGCAAGAACCAATAGAAAG
GATTTCGACGCCCCTCTCGTCATAAGAAGCAGAACGCGCCATCACCTACAGCCCTTTCCTCTGCCGGGGACTT
CCACGAAATGAAAACGGGCGGCATCGTCGTATGCTCCACTTTTGTTGCCCTGGTTTATATCCCGGAGTACTCC
TATGGCCGATCTGTCACCCAACCTACTTAAACAACCTAAAGGCTTGGCCCCGTCCCGTTTGGAGAATGACCCC
TTATGGCACGGCTTAGTCAGTTATTCTCGCAGTTCAGATAAGATTCGGACCGGGTTGGGTTAGGTCTCTGAAA
GCATGGTTCTTGCCTCCCTCCCCCCTTTGAGCTCGTCCATTCGTTGGGTGATCCCTTGCTCTCACGTTCGAGTGT
TTGCTCGTCGTTTAGGCCCGTGAGTGAGATTTCTGCTCATTGCAGTCACCTCCGGGGTTCTTCGCACCTGGATA
GTACCAGGACCCTTGTGCCCCGGCCCTTGATCAGATAAAGCTGCCCGCCTTATGACCCACAAACTAAAAATG
AGCCTTGCGACGAGACGCGGACATTCCCCATGCTGCGGTTCCCTCCGGTCCGTTCCCGGGTTGGGTTAGGGGA
ACATCCGAGCGATTGCCTTCGCGGATCCAAACGCGCTCACAAGGCGCACAATAAGAATAAGACCAATAGAG
ACTTCATAAGGGACCATTTGAGCTGCAGATCGTAATGCTCCTAGAGAGGCATATTTCGAATATAGAGGAGTG
AAAGTTCCCAACAAATAAGTACTTTTTCATATCCTTCTATGAACCGTACGAGCACGTCCTCTGTCACCCCCCA
CCGCGCTTACGGCTCTATACCCCAACCCAACTTGCTCTGGCCCCGGGTCCTTCCTTTCTATTCCTCGGTAAGCG

GACCGTGGGAGCATGGGGGGCGATATCCGCTTTTGACTGATAGAAAGCATCTCTCTTTTGTCCCTCCTGACCG
AACTCGCCAAAAAAATCACAATAGAAAAAGTTGTTCTTGCCGGGAGAGTAGCGTCGGAGACATCTTTATCTG
AGGAGGAGGCTTCGTCGTCCGGCTCCTCATAAATGATGTTAGGATCGGCCGGCTCATCCTCGGAATCCGAAA
AGAAAACAGAGACAGAACGGAATCACTTTGTTTCAGTGACATATCTAATCAGACTGAATAGGATTTGAAGAG
CTGTCACGATCATTCACAAAAATTTCAGTGAACTATTGAAGCTATCAGTGTGATTGATCAGACAAGTACCAAG
GGCTTTCGGTAGACTTCTTTC
>TCONS_00039713
CTTTGAAGAATGTAAATCTTGCATACATTCAACCTGCGTCTTTAGCAAGGGGACGAGGGCAAAGCTTTTGATC
TTTGGGCTCTGCAAAAGTAAATGTTGACAAAAAAAATCTGTTTGGAGAGATTAAAAATTACAACTCAGGGGC
TTCTAGGCAGAATAAGCTAGCACATAATAGTAATAATCTTATGCCTCCTGGTTTTGATATGATGGAAGAAGAT
GTTCCCTTCACTCAAGAAGCTGAAATGATGCAAGAAGTTGGAGCTACTTCGAAGAATGTACGAGACAC
>TCONS_00039714
AGCTCGGTAAGCTTTTACTATTAGCTTTTGTGTTGCTAATTCTATTTATTCATAGCAAAACGATTTCCAATATCA
GGCTAAGAATTGCTAGGTGTTCCACAAGAAGACATCACTCATTGAGGCTCCACCTTTCTTGCCTTCTCTGGTTG
ACATGAATCCATCAAAGTCTGAAATGGGAGCAACTTCAAAGAATGTACATCATGCATATATTCAACC
>TCONS_00039726
CTTTTTAGTTTCTTCTTCCTTCTAAGTTCATTCCTACTACGTATAAGCTTTCACTTTGTTCCAAACACAAAATTTG
AAAAGGAATTTTCTTACATTTATTTGAAGCATGTCAAGCAGAAGATCCAGAGATTCGGAGTCTTCGAGGATTT
CAGATGATCAGATCATCGAACTTGTATCCAAATTGCAACAACTTCTCCCTGAGATTCGAACTCGCCGTTCCAG
CAAGGCATCAGCATCAAAGGTGCTCCAAGAAACTTGCAACTACATAAGAAGCTTGAACAGAGAAATGGATG
ACCTTAGTGATCGACTCTCTCAGTTACTATCCACTTTAGATGCTGACAGTCCAGAAGCTGCAATTATCCGCAG
TTTGATAATATGATTTATATATTACAAGTATTCTACTAACTAGTTGAGAGAGTTAGTCATGAGTGGTTT
>TCONS_00039739
AAATCCTATTGCTTTTCTGGGTTTCTAAGGATAAGCAATTTCATTGTTCAGCTTAGAATTGGTGAAACACAAGA
AAGAACAAACAACAACCGTGGCGAATAGAGCAAAGTAAAGAACAGCATGCTGTGGTGTAGTTTATCTGTTG
ACGTCTATCGGAAAGTCATCTCCTTGAACTTGTATTGCCGTGTCATTCTGTAAAATACCCTTTTTCAGAAATCA
CTTCTTTGTTATAGATTACTGTTTCTTTTGTTCACCTTGAAAAATCAAGTCTTCATA
>TCONS_00039740
CTTTTGTTTCTTGATTGTTCTGTTGGAAGATTGAGGTGTTTGTATTGAGTGAAAAGGGTTGGGAACAAGTAGTT
ATGGTGTGTCAAGCAGCTGGCCAGACGAGATTTCGAGCATTGAAACATGAAAATGGAATTGCTGGGTCTGCC
ACCATAATAGTTAGAGTCATAGCATGCTTTCAACCACTCCAAGATTGCCAGGCTGAATACTTCCGTCATTTGC
TTAAACCCGTCACGTAGATTGACCTTTCTTTTTACCGCGTTCAAGTTAAGCTGGAGTTTTTAGTTTTTTTCGTTG
TTTATCAAGAGTGCAAGAAATAAACAGCAATTCGTAGT
>TCONS_00039747
ATGAAGACTTGATTTTTCAAGGTGAACAAAAGAAACAGTAATCTATAACAAAGAAGTGATTTCTGAAAAAG
GGTATTTTACAGAATGACACGGCAATACAAGTTCAAGGAGATGACTTTCCGATAGACGTCAACAGATAAACT
ACACCACAGCATGCTGTTCTTTACTTTGCTCTATTCGCCACGGTTGTTGTTTGTTCTTTCTTGTGTTTCACCAATT
CTAAGCTGAACAATGAAATTGCTTATCCTTAGAAACCCAGAAAAGCAATAGGATTT
>TCONS_00039748
TACGAATTGCTGTTTATTTCTTGCACTCTTGATAAACAACGAAAAAAACTAAAAACTCCAGCTTAACTTGAAC
GCGGTAAAAAGAAAGGTCAATCTACGTGACGGGTTTAAGCAAATGACGGAAGTATTCAGCCTGGCAATCTTG
GAGTGGTTGAAAGCATGCTATGACTCTAACTATTATGGTGGCAGACCCAGCAATTCCATTTTCATGTTTCAAT

GCTCGAAATCTCGTCTGGCCAGCTGCTTGACACACCATAACTACTTGTTCCCAACCCTTTTCACTCAATACAA
ACACCTCAATCTTCCAACAGAACAATCAAGAAACAAAAG
>TCONS_00039831
ATACTTCTTCCTCTCATCAATCCTCCTTTAATAACGGATGGCTGCTGGATTCCATAAGCATCTCATTCTCCTTCG
CTCAAGATTGGTACGATGAGTGGTTTCAACTGTGCAAGATACACAATCGCCCACATCATCCAACAGCATACT
GTAGCAGTTATAATCAATGTCAGGTGTAACAGATTAGTGGAAGGCCCTCTGTTGCAGCAGATTCCAGCACAG
AA
>TCONS_00039849
GTCAGATTTTGGATGTTTGGCCTCGGCATGTTCCTTGCACTTCACCTCTGACGTTGTGCACATAAATGCCTGCA
TACATACCTTGCACTGGATATTCATGGCCTGCTTGTTCTTTTCAAGTTGGCTTCCTTTTTGGGCTTTCATCTTCTC
GAGGTTCCTCTCGCGAGCCATCTTCGCTTTCTGACCATTGCCTCCACCCATGGTTG
>TCONS_00039882
CTTCTATAACACCCACTACTCTTATTCACTATCTCCATAACTTCCACTACTCCAATACTTATGGTTGTAACTCCC
ATACCTCCATGATCTCCCCCATGATCTTCCTCCATGATCTCAAAAGCTTAATGACATATTTATGGAATTCTTTT
GGTATACCTCTATGTAGTACTATAAATAGAGGATGAGAAGCCATTTTGTAATACACTTGAATACATGGAAGA
AAT
>TCONS_00039936
CTGATATTTAGAGAATCTTTAGTTTCCCTTAAAAGGGAAGTTATTTGGTATTGGAATGAAATGGACATGAATA
GAGTAAAGTTGATATAGAGAATTCATTTAGCCGACCCCAATTAATATTGAAAAGACTTGGAAGGCTATGTGA
ACTCTTGGTCTAATTTGCTTTTAAATGGTAATTTGGATGCTTAAAGTCTGGAAATCTCGTTTACTCTCAATGCAT
GTATTAACTTTTAGCAGTGAAACTTTTTCTCTTGTTGATTTTATCATCTGAACGCCACTGATTCATTACGCTCCT
CATGTAGAGTACACATCCACAACGAGGAGGTGAAGTGAATCTAACTTTGGGAGGAATTGACTTGAATAATTC
AGGGAGTGTTGTCGTTAGAGAAGATAAAAAGCTTTTGACTGTGATGTTTCCCGATGGACGTGATGGGCGAGC
TTTTACTCTTAAGGAACTCTTAATGCATGTTACAAGCAATTTACTTGGAGGAG
>TCONS_00039939
CGGATGGCTGCAGCTGCTTCTGCCATTGCAACTGGAGGTGGCTGGGGAGGAACCCAGGATCGCTGAACTGGT
CGTGCCTCGGCTGGTGGAGCAGTAGATCCAAACTTCTGTTCGTTCACAGCTTCAATTTCTGTTATTCTGGCATT
CTTGCGTTGCCACCATGGCGTTGAACTGTCTCTATTCGTCGGATTGTTTTGATACCCATAATTCAAGCCATCGC
CACTCCCTTGAGATTGGAGAAGATTCCCTGAACTGTTTTGACTTTGGCCAATTTCCCAAGGCTTAGGCTTTGGA
CCAACACGAGGCTCTGGTACAGGCTGGTTAGGATTTGGTGGCTGGTCATTTATATCTCGGATGTTCGAGGGCT
TTTCTCCTCTTTGAATCATAGCCATGATGTCCATATAAGATTTTGGGTGGGGTGCAACAGATGGTTCCACAGAT
GCAGGTGGTGACAAAGATCTCACT
>TCONS_00039940
TTTTGTAAGGCACTGCTCATTGATTTCATCTCTCGAACTTGGTAGTTTAACAGGTTTGTTAGCTCCTCAAAGTAC
CTTTTCTCTTCAGTTTTAGATGCCAGCATCTCCTGACTTGCACGGGCCACATCAGCAGCAGCTGCTGCAGCTGC
TTTTGCAGCAAGAGCCGCTTCTTCTGCTAAACTTGGTCTTCCCTTCACAGCGCCTTTTTCATCTTCTTCTTCCATT
ACAACCTTGCGAATCCAAGACTTAAACCGGGGTATAATCGTGTGCTTGAAAAGTAAAGCTGTACCAGCTCCT
GAAGCAGCAAGTATACCTACAGCCATTACCGCATGAGTCCAGTGGAAATGAGAGAAATATCCCATTTTTGTC
ATGGTATTACTATGTAAAGCTGATGCAGGCAGGAGATTCTGCGATGCAGCCAGTGTTTGAGTAGTCGACGAA
GATTTCA
>TCONS_00039941

CTTCAGTAATAATTAAGCCAAGATCGAAGGTCAAACATCAGAAAGTTACCTACCGGAACTCGACGAAATGCT
TCATCTATCTCCTCCTTTGTGAGCCCCTTCCTCTCAAGGAAAGAACGCCTATACATGACTGGAGATCCCCTAA
CTCTTGGATGTGAAAGAAACTTGACAGCATTTTGGACTTGATCCTCCCGAACTGGTTCCGAGTTTACAAATAC
AGAAGTTGGAGAGTCCCCTCTTGCAGCCTCAGGCTTGTAATCAAGATTATCTTTAGCAGCTGGTTGAGAAGCG
GTTGCCTGATTTTGGGAATTGTCGTTGGGGGAAGAATCTGATTCAGTCGCCATACTCACCAACTCCTGTGAAC
TGAATTTTCAGGGAAATAAAGATTAAAG
>TCONS_00039942
CCGGAACTCGACGAAATGCTTCATCTATCTCCTCCTTTGTGAGCCCCTTCCTCTCAAGGAAAGAACGCCTATA
CATGACTGGAGATCCCCTAACTCTTGGATGTGAAAGAAACTTGACAGCATTTTGGACTTGATCCTCCCGAACT
GGTTCCGAGTTTACAAATACAGAAGTTGGAGAGTCCCCTCTTGCAGCCTCAGGCTTGTAATCAAGATTATCTT
TAGCAGCCTGATTTTGGGAATTGTCGTTGGGGGAAGAATCTGATTCAGTCGCCATACTCACCAACTCCTGTGA
ACTGAATTTTCAGGGAAATAAAGATTAAAG
>TCONS_00039952
GGCAGCAATAATAATGTCTGCTTCACGAACGATTTTTTCTGGTTCCTTGGTGCGTGAGTGCACTATAGTAACA
GTAGCATCTTCCTTCAGAAGCAGCAGAGACACTGGTAATCCAACAATGTTACTTCGACCAACCACAACGGCA
TTCTTCCCTTTTATGCTAATCCCACTTCGATGCAAAAGCTCGATGCATCCCTTGGGCGTGCAAGGGAGGAACA
GAGGTTGTCTGCCTTTCATTGCAAGCTTGCCAATATTCAGAGGATGAAAGCCATCTACATCCTTTTCCAGACTG
ATTTCACCAAGAACTCTCTCTTCATTAATGTGTTTTGGTAACGGAAGTTGTACCAGTATACCATGTACATCAGG
ATCAGCATTCAGCTCGTGGACCTTGCTAATTAATTCAGCTTCAGCTACGTCCTCTAGCAGGTCTATGTGAAAA
GACTTAATGCCAAGCTCAGCACACGATTTTCTCTTCATATTCACATAACTTTGAGAATCTTTCCTATTTCCTAC
AATGACTACTGCCAACCCAGGGACCTTGCCATACTTTTCTGAAAGGAGTCGGACTTCAGAAGCAATCTCAGA
ACGAATAGTTTGAGCAATTGCTTTACCGTCAATGATGGTAGCCTTGTGATCTGATGGCGATGCCATTGTTTCTT
CAAACGAGAAATAACTTCC
>TCONS_00039973
TTTTTTCTTCTTAAAATCTACATTGACTTGGAAGAGTTTAGAAATGTATATTTGTTCATTTTGTAGAATTCCTCA
GAACTAGCTGCTTAAGTTGATTATTGATATTTTACGTGCTCCTTCTGGTTACTTGATTAGGACCTTATTTTACTC
TTCGTGATGCATGTATATACTGTTTCATGTGAGAAGTTTCTTTTGGTAGTAACCATTCCAATTTCCTAAAGTTAG
AGGCTGAGTTCTCTATGGAAGTGCCTGCTTTTGAGATGTGACAAGGATGGCAGCCTACGTTAGCAGATAATTG
AATACTTGGCTATATGGACCTTGCTTCGCATAATGCATGTAGACTATCTGTTGCAAAAGTTTCCCTTCAATAGT
AAGAATTCAGGGTTGAGTATAGAGCTACAGGCATTGTATGCAACTTGCCCACCTTCGAGTAGTGATGAGGGT
AGCAGCCTATATTGAC
>TCONS_00040015
CTTTTCCCCACTTGAATATTACCTTAAGAAGTGGCTTTTGGTTCTTGTCATTAATGAAATAATTAATCCATCAC
GAGCATGTTTTCGTTTGAATCCATCAGAAGTTTCAATCTCAAACGTGTAATTTGTTCTCGGTCTCATCTGTATTC
TTCACCTTCAGAAGCTACACTCGGCACACAGGATGAACTAACTGGAAATGACGAAGTAGTTGTTGAACACTG
TAATATAATTGACACGAGCTTTTGGAAAGAACATCCAAGAATACTTGATGAGGAGGAGCCATTGCTGACGAT
TTTGCGGCCCCATAGCAATAAAATGCTTCGATTGTCTTGATGTTGAAAGAGGAGCTGCTCCCTAAGTTATTAA
TCATATTCAGTTTTCATTAGATCAGAAAAATAGTTTCAAATTGTCAAATATGTAGATTAGTCGTTTGCCTTTGA
TTATTCCCTTTACTCCTTTTTTTTCTTCTTCTAAGGGTTAGCAAAACACTCAGAACATTTAGAGCCCGTTTGGAT
ATAAAAAAAATTTCACTGTTTTGGAAATTAGTGTTTGGCC
>TCONS_00040082

CCACCATGGGGTACGCCTGTTCAAGTGACCAGTTTTGATTTCATAAAAGTTCGTTGGAGTTTCCTTGGTTTTGTT
AAACTTGAAGGACTTGAGAGGACACTGACTTGCAACTTCTTTAGGTGCATGAAGCTCAATCTGTTGAGTATCT
TCCAAGTGATCGATCCAATCATGAACCATAAACTGAATCCAAGAAGCAGCGATCATGTTAAATTGTTT
>TCONS_00040152
GTGCTGAGTAATATGAGCACGTCTTACCTCCAGAACATGCATAGACACCAAGCTTGAAAGGATGAGGTATCT
TACATCAGCACTATCTTTTGCAATATGAGCCCAGCAACCAAATAGAGCTAAAGCCAATGCTCTCTCTTCAACG
TCTCCGGTGTCGAATACTATCTTTATTCTACTTAGAAGTTCTCTATAGCTATCTATTTTATCCTTCGAAAGGATC
CCCTTATCTTTTCTCCCCTTGCTCCTCAATCGACTCCTCTGCTTGAGCTCGGACAAGAAAATATTCAAAATGCA
AAGCTTCATATGTTTGTCCCCTGACTTAAATGCATCTGCAAGCCTAAGGAGGATAGTATTTGCAAATAGCTTA
TCCTCCCCAGGAATTAAGCCGAATACTTTGTACTCAGCAACACTAAGATTTGACTCTCTACTCCACCACTCAA
GTTTTGGCCCAATATCTAGTATGGCTTCTGCAGATTTACCAGGTTTATTAGAACGGAGGCCCTTTTCAAGGTCA
ATGCTCCACTGCATCGCACAAGCCGATGGAGTTCTCTCCATTCTTATCGTCTCTGGAGGACAATGAGCTTGAG
CGGGA
>TCONS_00040207
CACCAACTGTCAGTAATTTCTCTCTAAACTCATCCTTCATTTGATCTTCGAGCAAGTATCGGAGCACAAGCAC
TAAGTATGCACCAAGGCGCATCATTTGTGGGTCACCATGAGGTCTGAAGAAGGTTGCATCATCTTCAGAAGG
CGATATCCAAGACCATATGACGTCCAGCAAACTTGGTATGTCTCCTAACATAAGGTTCATCT
>TCONS_00040252
CAAAATTTAAACCACAAGTTTCTTTATTAATCACTTTTTAAGATGTGCATAGTCCAAAATTTTCCATGCGACAA
CCAAGTTGATTGAAGATTGCAAGTCAGCAGCACAGGCAGGAATAAATGGTAATGCCTTGTTCAACAAGGAA
AAAACAAGATAAAACCGTAACGGATCAACAACTTATATCCTTGTTACTTGAGTTTCTCAAATAGCTTCGGGGC
AACGCATTTGTCAATGCATTGCCAATAATCAAAATACTGTCCAGTGCAATGCTTGTGCCCTGATTCATCACCTT
CTACCCTTCTAGTACATGCCTGGTACTCCTTTAGCTGCCTTACACACTTAGGCTTGCAAGTTACTTCCAGTGTC
GCCTTTGGGTCAACAACTTCCTCGTCCGACATGGCAAATAGATATTGGAGACGAAGGCTTCGATACAGCAAA
TTTACCTGAGTCTCGTAGAATCAATGAGCTGCGGATGG
>TCONS_00040302
GTCAACTTGACTTCTTACCATGAATGATTAAATGAAGGAGGTCAATCTAAATTGGAATTCGAAGTTAATACAT
GACAGAAAATCACCTCAAATATATCACAAGATAGAGCAAAAAGATTTGTACCAACATTCATCTATGTTTAGA
TCTCATTTTCATAAAATCTGGTAAATTCCGTTGAAACACAAAAATCTCCTACTTCATCCGCTGTATACAATCGC
GCTCTTTACAGTAATAGGAGGAAGATTTGTGGGTGGAGGAACCTTCCTCTGTCTATACAAGTTTTGAGTCTGCT
ACTTTTCCTGTTAGCTACCTTCTATGTTCTATACATGTATCTACTCAAAGAATGCACAAACAATTTATATGGCG
GACAAGACAGTGCGCAACATGCTTTGATGTGGTAAAGGTAATGTTGCATATATGTTGGACAGGACAGAAGGC
TGATGTTCCCTTTGGAACTTCTTCAATAGATTGATCATTCGCCTGATAGTTTCTTCTGTTGCTAAATTGTTCCCT
GCCCATAAAACCTCAGCAAAAATTGCTATTATTTTCGGTATATAAGATCCTTGAGGGCCAATAATTTTCTTCTC
CGACGTTTCCATCATCGAACAAAGCAGTTCGTGATTGATTTTAGCCTCATCCAGATTACACCTTATTGGTAAGT
GGCTAAGCCAAAGCAAGATAAACTTATAGGTGTAAATGCCTTCCCAGACAAACTGGCTTAACTTCCCACTGG
TAGAAATGGCAGCCTCGTAAGCCATGATGTTATCCGGGCGTTTTGCATCGGGATGCTCCATTACAGATTTTAA
ATGATTGAATATTACTGAAACAACATTAGAAAGCTAAGTCAGTCCTGCTAGTGGAGAAAATAAATATCTTAA
GCATGTTATAATGCGCAAATTACCTATAGTATGAGGCTTGAGGATATCACCTCCGAATTCAGCACAAATACC
AACTGCAGTTGCTACTAACTGAAAAA
>TCONS_00040344

ACAAATTCTAGTCTGATTATTTTCTGTAGAATTGCATCTGTTGCATTGTCAATGGATGCAGTTGCTTCATCTAGT
ACTAGAATTCGGCTCCTCTTCAACAGTACACGTCCAAGACAGAATAATTGTCGCTGTCCCATGCTCCAGTTTG
ATCCATCTTGTAAAACTGTGGAATCTAGTCCCGTTTCTTTTCCTTGAACAGCCTCTCTAAGCTGACATTTTTCAA
GAACCTTCCATATTTGGTCATCACTATATTCTGACAAGGGATCTAAATTGTATCTAATAGAACCTGTAAAGAG
CGTAGGATCTTGTGGAATAATTCCAATACGCGACCTAAGATCATGAAGCCTAATTGTTGAAATATCGCGGTCA
TCAATGATGATCTTGCCATGTGTAGGTTCAACGAGGCGAAACAATGCACTGATAAGAGTTGTTTTCCCACTGC
CTGTTCTGCCTACAACTCCAATCTTTT
>TCONS_00040447
CCCTCTTCTTCCGCCGCCGTAATCTCATTCATACGACGACGTTTAGGGTATAATCATGAACGCTCCATCACCTC
ACCACGAAGAAGACGAACACGAAGATGAGGTCTTCATAGATGAAAATGATATCATACAAGAAATAAACGTT
GATGAAGAAGAGTTAATTGATGCTGATGAAGATGATGAAGGAGAAGAAGAAGAAGGTGGAGGATTGTTAGG
TGAGTTATGTTTGGCTCAGTGGAAGTCTTTTGTTTTAAGTTTTCAGCTTTCTATTTGACTTAATCACTTCTAGAA
ACCTTCTGAGTATATACAGCCAAAATTTCATATATCTTGGTTACTCATGTTTCTGCTGTAATTACTTTTACTTGC
TCCCTCCATCCCAATTTACGCGAATGTATTAGACTGCACTCCAGGTTTAAGAAATAAAGAAAGACTATTG
>TCONS_00040565
TTAAGCCCATTATGTAGCATAAGTCTTTGTCTCTATCTTGAACTTCGTCCCAGTCAAATACCGGTCAAATACTG
ATACATAAATTGAAATGAGAGTACCTCTTCCTGGAGTTGCAGGGACGGTCAGAACTCTTTGTGCTAGCATCAG
CCAGAAGATGCAGAGATTCCAGCTTGCCAGTTGATGAAGATGGTGCTGGATTACTAATCTGTTTACCTTTGTT
CATCCCTGATAAAGCTGTGCCATTTAAGAGGGACAGAGCTTTATCATAGGAAGAAAGTATTTCCACAGATAA
ATACTGGCATACCTCCTGTTTCATGGGATCAAATTGCTTTTGCAACTGACACATCAATTCCCTCCCCTTAGTTA
GCTCAGTTATCACTACCTCTCTCTCCC
>TCONS_00040711
GACATAATCCAACAAGTTTTTTCATTAGAATCATGAATAAAGCTCGACTCTTCCACCTTAAAACCCTAGCTTG
GTTCTACGCCAGTGCCTGCGCTTAGCATTGTACCTGATAGTGTTATCAGTCCTCATACGAATCCAGTAAGGAA
TGGGCCTGTTTTGCCTCTGCTTCTTCGCCAGTTTCTTCTTAATCATAAATGTCTTATGTGACGGCATTTTCACCG
GTTGGAGGTTCTCGACGATAAGCTCTGAAGCTGCTTGAAGTTGTGAAGCCCTAA
>TCONS_00040725
CAAATACCCTACAAAATGACAGTAAAAGTAAAATAGAATGCTTCTGATATCTGCCACTGCTCTTCAATGGTTG
GTTTTAATTGCAATCTCTATCTTCAAGCCCGAGAGACGACTTCAATCCAGCTTCACTGAAGAGAACAAATAAT
GTACAAACCAGAAAGAGGATAGGAACCCGACCGTGCCTGTTGCAAACATAATCGCCAAGACCATGAAGAGG
GAATATCCGAGGTAAAGGGTGGCGGAAACAGGGCCGCTTAAACTCTTGAGGTCGAATATTAGGTAGTTGATG
GAGTATAGGAAAATGTATATCGCGACTGAACCAGAAGCAAAGAATGACTTCCACCACCATTTCCAGTCCTCG
ACACATAGATGCATGTAGGTTAGAACAAGAGACACCTCAGCACAAACAATGACAAGGAGGAGCATGACAA
TAAGGAGGAATCCGAAGACATAGTACACACGACCCATCCAAAGACTGGACATGATAAAGAAGAGCTCAATG
AATAGAGTACCGAAAGGAAGGGTTCCTGCACCAAGAACCAAAAGCCAAGACGGATATTTTTGTGGTGGAAT
TTCACGTGGAATCTGGTTGGTTCGGACTGGATATTCAATGTGAGGTGCCTTTGCCCCGAGGTAGCCACCAATA
AGTGTTAGGGGAACAGAGATACAGAACCAAAGTAAAATGAGAACGACGAACAGAGAAAAGGGAATGGCCC
CTGTACTGTGACTACCCCATAGCAAGAAGTTCAATGTGGTCAGGATAAAGAAAGCAATCCCGGGGAAGAAA
CATGCAGCTTTCCACGCAACTGAAACCCAGCCCTTGTGATCGCCACAGAAGATTGTCCTCCAGAGACGAACA
GCAACATAACCAGCTGCAACACCAAGAATCATGTAAAAGAATAGCATACCTGTAATCAATGTTCCTCGAGAT
GCTGGGGACATGAATCCAAGGGCAGCAAACATGATAGTCACTACAGCCATCCCCAAGATTTGAACACCATCT
CCAACCATCGCGCATAGAAGGCCAGGATTACCAGGCGCGCGGAAAACATCACCGACAACAAGCTTCCACCC

AGATAACTCCTCATTCATCTGGGCTTGAGCTTCTTTGTCGAGCTCATCATACCTTGCCAGATCCCTTCGGACTG
TTCTCAAAAAGATCACGAGCACAATACCAGCCAAGAAAGTGATCACCATTAGGGAATTGAGTATCGAGAAC
CAATGCACCTTTGCACCTTCCATCTTCAAATAAGCATCCCATCTCGATGGCCACTTAATGTCACTCTCCACAA
AGTTCACCTCATAAGTAAAAGCCACAGGCTCATTTTCTTTAATAGCCATGGACACAGTTGTCGGGTCACACTT
GATTGGAGAAGGGAACTTATTGTACGTTTTAAGGTTCTTTAACGAATCAGGCGAATGCTGGTAACTACAAGGT
ACAACCTCAAACCCAACGACCATATATCCAGGTGCATCGGATCCTGAATTTCCAACTGTTGAGATCACCTCTG
ATCCATCCCCAGTACCCATCACACGAGCCACATTGGTCTCCTCGAATTTATGAACAAGAACCGTAAACTTCA
AATGATTAAACACATAGTATTGATCTTGGACCTTAATCCCAACTGGATACCCCGTCCATCGCAAGAAGTAAC
CTTCCTTCTTGGTATAACGAATAGCGGGCAAATTATCAAGAATCAAATTAACCTGATACATTTCATCAATCCT
CTCTTTCAAAAGCTTAAATTCCTCACCAGACAAAGGTTTTGTTTGACACAAGAAAACCTCAGTCTCATTGGTA
TACATCTTAAACCTATAAGGAGAATTCTCAATCCTATCACCCATAAGAAGCTCACCTAGATTTTCAGCACTAT
CCTTCACACCTTCTTCAGGCTTACAGAAAGGCAAACTATAATAACTATAAGGCAATTCAGTGTCAATTGAAGT
CAATGAATTGACCTTAACATTCAAGAAATCACCAACCTCATATTTGTGAGGATAACTTCCAGGCAAATAAAA
CCCATGACCCAATTCAGAAACCAAGCAGATAAACAAAACCCAAATCTTGAATTTATCAAAAGATCCCATTTT
TACACCTATTTTCAGCTCAGATCTCTGATGGAAGAAAATGGGCTAAAATGGAGAGGAGAAGCGTTGTGAGAG
TAACATTTGAGCTGAGGAAGATCTAACAAAAGGGTGGTGGC
>TCONS_00040825
TCGGTACATATGTAGAGACTGTCCCACATCAAATCTTGAACCAATGGATGAGGGTAATAGAGATCCTCCTTTG
CACATGAATGGCGTACTTTTCTCCAATTAATTTCATTGTATGGTTGATCATATAACTCTTCCCTCAGTTGCAAA
ATGAGAGGTGTGATTGGACCAACAAATCTCTTCCCATAGAGATAAGACATTGGCATGTAGACCATTCGACAG
TAACACCACATTTTCGCTGGATGTATGGGAAGAAAAGATGGAAGAATCCAAAACTCGGGGGGCATTGGATTG
GTCCCTAACCACTCAAAAACTCCAAGAATCGAGAGCCATGTTTTGCCCCAGGAAGGAATCGCGGTGACGCTA
CCATGATCAAGAATCCATTTCCTTGCTCTAGCACAAGCATTGTTTTCGCCACCATCCGGTCCTTCCCCGAGGAT
CCTCATACAAATATAATTCAATGTTGTACAGAACATTGTACTGTGACCTTCTATGTGCAAACCCCATCCACCA
TCTTCATTCTGGTGACAGTATATATACCGAAGAATTTCCGTACGATGCTCGGCTGGGAAAACAGTATTAAGAT
GGCCAGTGATGTACATACACATAACAAGAGGTGGAAGAAAGAACAAAGGACCGGCATTTTCAGCAGGCCA
ATGACCATCACTAGCCTGTAATGCTGAAAAGAAATGAACAGCTCTACGCAATGAAGTTGTGGCAACTTCATC
TGTAATTTCTTCTCCTTCTTCTACTTTTACTGCTGGAATTTTTTGTTTGAAATTCTTCCCTCCAAGAAACTGCATT
CGCCAAAGAAGATCACCACTAGGCTTGATTTTATAGCGATTGTTCCAAAAATGTTGACGGGCCTCTTCGATCT
CGGCCCGTTCTTCTGGGCTTCCAGCATTTGGGTCAAAAGCCCATATTTGACGTCCAACGTAGTTGTTTGTGCTG
TACAAATATGGCCCATCTTGCCCTTGTGCAATCTTCAATTTCCACATTCTTAATTTCTTCTTCTAACCTTCTAGC
TTAC
>TCONS_00040849
CCGTATACGTTGAGGCACCTTCTCCTGCATAGATAAAACCGCCATCTATGGAGCGAATATCGTACACAGAGA
CGCAGAGACCCAAATTTGCAATAACCTTATCCACAAAGAGACCCTCAAGCTCTCCCCTGATTGATTCATTGAG
TGGAAGGCTTAGGAGATGAGGCGGCAATCTCAGTGTGTGTTCTATTTCACTCAAATAAAACATGCCTCCT
>TCONS_00040935
AGGCACTGCACTCATGTAAGTTTTCTCCTGACTGGATGATATCCTCTTTTTTTCTACATTCAAGTTGTAATTGCG
GAGGATTTCATTCTCATTTCTGCTTCATCAGGCTCAGGAGAAAATCTGTAAATATGTGCTCATATTCTGGCATT
TTCCCGTACCCAGGAAAGTAATTGATATCTATCACGTAATAGCGGTCTTTAGTCCCATGCTCTCTAATTATATC
CAAGTTGAAGAGCCTGAGACCCAGTCTACGACGAAGTTCCCTAGCAAGCCTCTCAAGTAATGGTCGGGGAGG
AAGCTCACCAACACAAGGGTCTAAATCAGCTTCCTCTGCAGAGGCTGCAACACATGAAACTCTAGGGAAAT

GGAAAACCCCAGCACTTTTTGACAGCTCGAGTTTGCTAACATCAGGTAAACTGAAACGCCTGACAACCTTAA
CAGCTTCCCCAACAATAAAAACCTTAAAGAGCACACCTCCATGATTGATAAATTCCTGCAGAACAAGCGGGG
GTTCAAGCTTCTGAAGAGAGGGTGTATCATAGGCCAGGGACAACTCATGTGACTTTGCAACCAAAGGCTTCG
CCACAAGAGGTAGGGCAAGACCAGCTTTATTCACTGCATCTGAGATGGATGAAGGATCAGTCTCAATTACCA
ACTGCCTCGGAACACCAACTGTGCCATAGCTGTCTGATAAGTTCAGGTCTGCAACATCTTCAAGCATGTATTG
GCGATTATATACCTGCTCTATGGCATCTGGAGGGTCAAGGACTGCGACATGTGGATGTGTTTGCCTATAATCC
TCAAGAATACGGCTCCACTTGCTTCCTGACAACTTGTGGAGCACAATGTCAAAAGGACCTTGATCTGAAAGG
GGTCTGCTCTGATCTATAGCAACAAATAGTATTCCCTTATTCCTAGCTAGACCTACAAGCTTGGGCTGCAAAA
AACTCTTAGTTTTCTTGGATGTAAGAGCATAGCCTACCACCACCAATTTCATCTGTTCTTGTGGAGACCTCACC
ATTCCCATTTCCATTTCCTTTTCCTCTTCCTCTACTTCTACCTCCCCCTCATACCCCACTACATTTTCACTCCTCA
CCATCTTCATCCCCCCTAAAAAAAACACCTTCCTTTTACACCAAATTTATGTATTTATGACAAATTTCTCAACA
CCCCACTTCACAAAATTTAAT
>TCONS_00041006
TTGTTAACTTGTATCCCTTTTTATTTTAATGTTTTGCTTCTTTTTGGAACTTTTGCAGATCTTAAGTTGGGAAATA
TATATCACTTGTAATCACGACTTTAGCAATTACGGAAAAGCCATCGCTTCGTTTTTTCAGTTACTTGAGCTTGG
AGAAATTGCATGAAAAAGCTTAACAAAGTGAATGGCTACTGTTATTGAGTTCGCTCTGAAATTGGGACCTAC
AACAACAACAACAACAACAACATCCCAGTGAAATTGGGACCTACATATAGGTTTTTCTCTTCCGCCTGTTATT
CTAGCTTCATTCATCAATCAGTATGGATCAAATTGCTGAGTCAGTTTTGGTTAAAGAAAGCATGTTCTCATTGT
TTATAAG
>TCONS_00041031
AGGTGATGGTGGCAACGGGTGTGCCAGTATTTTTCCTAGTCGTCATGATCGTCGTGGCAGACCTGATGGTGTG
TTCTCTAATCACTTCATACCTCAAGGTTGGTGGGAAGGAAATATGTAACTAGTTGACTGCCATCTTCCGTCTTT
TCCCCTAGTTGAAGTCTCAAGATGAGGAAGACTCGCCCTCTTCGAGAAAAAGAAGAATGGATGAGGATCGA
GATGGGGATATCGGCACCTCCTCAGGGATGATTTGGAGGGATAAGCAATGGAGTGA
>TCONS_00041047
TATTTACTTAATAAACCAAGTCAACTATTTTTGTGCTTGATAATTATCTCAAGATTGAGCTTGTTCCATAATATT
ACCAAATTATGTTTTAGCAGACGATGAAGGGAAAAACGATGAAGAAAATGACGAATACGAAGGCTTGAAAT
TTAACAAGACGCTGGGGAAAAGAGCCGAAACTAAGAATTACAAGCCAAAAGTATTGCTTGAAGATAATCTT
GTTAAGACCTTTAGCCCTGATACACCGAAA
>TCONS_00041063
TGAAGACCTTTTTCCTTTACCCTTCAATTTTCCTGCTTTGCTTGTTGGACGATGAAGGTCAAGGGGAAGAGGTC
CAGGTTTTCGTGTTTTTCGGTTAAATCTTCTCAGACATACATATCCTCTCCAAAAGCAATTTTGTACTTGTTCTG
TTGACATCCCTCTAAATATTGCTCTAATGGAGGTGCCACAATATAGGGTCCTTCTCCGCAAATTCCATATCCAT
GCCCTCGGAACATCAATTGGCTGTTTAGTGGATTCTTCATCTGCAAAAACCTCATTTACTTGGAAGTATGTCCC
GTTGAGCGGAAATCTTCCTCTGTTTGCTGTTCGACATGGAATCAGAAGTGTCCCACGAAC
>TCONS_00041203
CAAACACACAACGATAAGGTAGAGGGGCTCATAAACAATAAGTCCCCTTTCGAATTGAAGATAGTCCCAAA
GAAACCTGTTTCACATGTAAAAGAAAAGCAAACAATTTTTAACAACAGCCAAAATTCAGTGATTGATATTCA
GCATGATAGCAGGAAACTCATTACTAGGTAACCTCAAAACTTTCGACAAATTGCATCTCTTATCCATCATCAG
GACTAAGGAGACGAGATAAAGCTTAATCAGAATCCTCAAAATATTCTCCTTCATCAGATGGCTCATCAAACG
AACGCATGTCAACCTGGTAGCGAAGGATCCCCCCAATGCCACCAAATCCTCGACAGAACTGTGACCCCTCTT
GAGACCTGTTAGTGACAAACTCAAGTGAACAACCGAAGTTTTTGTACTCGTTGGCAAACCACTCCAATAGCG

GCATTTTGTCCTGGACCTCTAATTCAGCTGAGGTGTCAGGATCCTTGAAGTTGCTGTGATCAGCCTCTTGGTCC
TTGTTTAGGTGCTTAATGACAATCTCGTTGGTAACACTATTTTTCAGCGCATAACGGTTTATATCAAGATTTTCC
CAAACAACAAGAGTTTCAACAGCTCCCATCTCAAGAGCTTTTATTGTGTCATCCACACCAAATACATACTTTC
CAGTATCTTGACTGATTTCCTCAAAGAATTTTCCTATCAAGCGCTTTTCTTGTATAAACTTCACATTCGCGAGA
ATCTCAGCAGATAGCTCAATAGCCTGATTGAATCCATTTTCCCCACCATAGGACACATCAACCACATTAAGTA
TCTTTGTTTGTAGGCGTTGATCAAACATGTCAGACTGGCTCAGCTCTGTCTTGAAATCAGCTGACCCAGCAAG
TATTAGTCCAGATACATTTGGCTGGCTAGTGGCTGGATTAATGAAGAACTGAGTAGCAAGCTCTGCTGTCTTC
CTCACATAGTTATGCCGTTTCTCCATTCGAAGACGAGCAAATCGCAGAGCTGATTGACCTCCTCTTCCGTGCTT
CTTGGGAAGGTCAACAGTGAATTTATGAAGGACTTCCCTAGTGTTGCCACTTAAGGTTCCGAAAAGAGTGCC
ATTACCGTCCATGACAATGAAACCAAACTTCTCATCTGATTCCAACAGCTCGCCCAGAGGCTCCGTATGAAA
CTTGTTGTCACATAGGTACAGAGACGCATTTATTGGTTTAAAAGGTGTAAGGTCAAAGGTTACCTTCTTTTCCT
TCCCATCGTCAGTCATTATAGTTCCAGTATAAAGGACCAACCCATTAGGAGGTACCTTATTATACAGCTTAAG
CCTCTGCTGGGCGGACGTTATTGCACCAAGGACGGACTGACGATTTACTCTGCTCTTAATATTGGATGCAGTA
CCATATTCTTCTGCCAACATCCTTGTTATCCGGGATATCTGATCACCAGGAGGTATAATAAGAGAGATCATAC
TGGTACCATTTCCTCTTGCAGATTCCATGGCTTTGATTAACTTTTTCATTTTCCATATCTCAATATTCTTGTCATT
TTCTTGGCCATCTGCCATTGCAAACTGCTACTACCACAAGCTGAGCTACAAATTAATCTGTTGGAAGCAGCAG
TCGCAGTAGCTCGAGCAAAATGCTGGCTGTGGGAAAATTGTGGAAACCCTTTTGAGCTACTATTTATACGCTG
GAATA
>TCONS_00041204
CAAACACACAACGATAAGGTAGAGGGGCTCATAAACAATAAGTCCCCTTTCGAATTGAAGATAGTCCCAAA
GAAACCTGTTTCACATGTAAAAGAAAAGCAAACAATTTTTAACAACAGCCAAAATTCAGTGATTGATATTCA
GCATGATAGCAGGAAACTCATTACTAGGTAACCTCAAAACTTTCGACAAATTGCATCTCTTATCCATCATCAG
GACTAAGGAGACGAGATAAAGCTTAATCAGAATCCTCAAAATATTCTCCTTCATCAGATGGCTCATCAAACG
AACGCATGTCAACCTGGTAGCGAAGGATCCCCCCAATGCCACCAAATCCTCGACAGAACTGTGACCCCTCTT
GAGACCTGTTAGTGACAAACTCAAGTGAACAACCGAAGTTTTTGTACTCGTTGGCAAACCACTCCAATAGCG
GCATTTTGTCCTGGACCTCTAATTCAGCTGAGGTGTCAGGATCCTTGAAGTTGCTGTGATCAGCCTCTTGGTCC
TTGTTTAGGTGCTTAATGACAATCTCGTTGGTAACACTATTTTTCAGCGCATAACGGTTTATATCAAGATTTTCC
CAAACAACAAGAGTTTCAACAGCTCCCATCTCAAGAGCTTTTATTGTGTCATCCACACCAAATACATACTTTC
CAGTATCTTGACTGATTTCCTCAAAGAATTTTCCTATCAAGCGCTTTTCTTGTATAAACTTCACATTCGCGAGA
ATCTCAGCAGATAGCTCAATAGCCTGATTGAATCCATTTTCCCCACCATAGGACACATCAACCACATTAAGTA
TCTTTGTTTGTAGGCGTTGATCAAACATGTCAGACTGGCTCAGCTCTGTCTTGAAATCAGCTGACCCAGCAAG
TATTAGTCCAGATACATTTGGCTGGCTAGTGGCTGGATTAATGAAGAACTGAGTAGCAAGCTCTGCTGTCTTC
CTCACATAGTTATGCCGTTTCTCCATTCGAAGACGAGCAAATCGCAGAGCTGATTGACCTCCTCTTCCGTGCTT
CTTGGGAAGGTCAACAGTGAATTTATGAAGGACTTCCCTAGTGTTGCCACTTAAGGTTCCGAAAAGAGTGCC
ATTACCGTCCATGACAATGAAACCAAACTTCTCATCTGATTCCAACAGCTCGCCCAGAGGCTCCGTATGAAA
CTTGTTGTCACATAGGTACAGAGACGCATTTATTGGTTTAAAAGGTGTAAGGTCAAAGGTTACCTTCTTTTCCT
TCCCATCGTCAGTCATTATAGTTCCAGTATAAAGGACCAACCCATTAGGAGGTACCTTATTATACAGCTTAAG
CCTCTGCTGGGCGGACGTTATTGCACCAAGGACGGACTGACGATTTACTCTGCTCTTAATATTGGATGCAGTA
CCATATTCTTCTGCCAACATCCTTGTTATCCGGGATATCTGATCACCAGGAGGTATAATAAGAGAGATCATAC
TGGTACCATTTCCTCTTGCAGATTCCATGGCTTTGATTAACTTTTTCATTTTCCATATCTCAATATTCTTGTCATT
TTCTTGGCCATCTGCCATTGCAAACTGCTACTACCACAAGCTGAGCTACAAATTAATGTTACCTGTTGGAAGC

AGCAGTCGCAGTAGCTCGAGCAAAATGCTGGCTGTGGGAAAATTGTGGAAACCCTTTTGAGCTACTATTTATA
CGCTGGAATA
>TCONS_00041205
TTCGAATTGAAGATAGTCCCAAAGAAACCTGTTTCACATGTAAAAGAAAAGCAAACAATTTTTAACAACAGC
CAAAATTCAGTGATTGATATTCAGCATGATAGCAGGAAACTCATTACTAGGTAACCTCAAAACTTTCGACAA
ATTGCATCTCTTATCCATCATCAGGACTAAGGAGACGAGATAAAGCTTAATCAGAATCCTCAAAATATTCTCC
TTCATCAGATGGCTCATCAAACGAACGCATGTCAACCTGGTAGCGAAGGATCCCCCCAATGCCACCAAATCC
TCGACAGAACTGTGACCCCTCTTGAGACCTGTTAGTGACAAACTCAAGTGAACAACCGAAGTTTTTGTACTCG
TTGGCAAACCACTCCAATAGCGGCATTTTGTCCTGGACCTCTAATTCAGCTGAGGTGTCAGGATCCTTGAAGT
TGCTGTGATCAGCCTCTTGGTCCTTGTTTAGGTGCTTAATGACAATCTCGTTGGTAACACTATTTTTCAGCGCAT
AACGGTTTATATCAAGATTTTCCCAAACAACAAGAGTTTCAACAGCTCCCATCTCAAGAGCTTTTATTGTGTC
ATCCACACCAAATACATACTTTCCAGTATCTTGACTGATTTCCTCAAAGAATTTTCCTATCAAGCGCTTTTCTT
GTATAAACTTCACATTCGCGAGAATCTCAGCAGATAGCTCAATAGCCTGATTGAATCCATTTTCCCCACCATA
GGACACATCAACCACATTAAGTATCTTTGTTTGTAGGCGTTGATCAAACATGTCAGACTGGCTCAGCTCTGTC
TTGAAATCAGCTGACCCAGCAAGTATTAGTCCAGATACATTTGGCTGGCTAGTGGCTGGATTAATGAAGAACT
GAGTAGCAAGCTCTGCTGTCTTCCTCACATAGTTATGCCGTTTCTCCATTCGAAGACGAGCAAATCGCAGAGC
TGATTGACCTCCTCTTCCGTGCTTCTTGGGAAGGTCAACAGTGAATTTATGAAGGACTTCCCTAGTGTTGCCAC
TTAAGGTTCCGAAAAGAGTGCCATTACCGTCCATGACAATGAAACCAAACTTCTCATCTGATTCCAACAGCTC
GCCCAGAGGCTCCGTATGAAACTTGTTGTCACATAGGTACAGAGACGCATTTATTGGTTTAAAAGGTGTAAG
GTCAAAGGTTACCTTCTTTTCCTTCCCATCGTCAGTCATTATAGTTCCAGTATAAAGGACCAACCCATTAGGAG
GTACCTTATTATACAGCTTAAGCCTCTGCTGGGCGGACGTTATTGCACCAAGGACGGACTGACGATTTACTCT
GCTCTTAATATTGGATGCAGTACCATATTCTTCTGCCAACATCCTTGTTATCCGGGATATCTGATCACCAGGAG
GTATAATAAGAGAGAGATCATACTGGTACCATTTCCTCTTGCAGATTCCATGGCTTTGATTAACTTTTTCATTTTCC
ATATCTCAATATTCTTGTCATTTTCTTGGCCATCTGCCATTGCAAACTGCTACTACCACAAGCTGAGCTACAAA
TTAATCTGACGTTACCTGTTGGAAGCAGCAGTCGCAGTAGCTCGAGCAAAATGCTGGCTGTGGGAAAATTGT
GGAAACCCTTTTGAGCTACTATTTATACGCTGGAATA
>TCONS_00041206
AAGAAACCTGTTTCACATGTAAAAGAAAAGCAAACAATTTTTAACAACAGCCAAAATTCAGTGATTGATATT
CAGCATGATAGCAGGAAACTCATTACTAGGTAACCTCAAAACTTTCGACAAATTGCATCTCTTATCCATCATC
AGGACTAAGGAGACGAGATAAAGCTTAATCAGAATCCTCAAAATATTCTCCTTCATCAGATGGCTCATCAAA
CGAACGCATGTCAACCTGGTAGCGAAGGATCCCCCCAATGCCACCAAATCCTCGACAGAACTGTGACCCCTC
TTGAGACCTGTTAGTGACAAACTCAAGTGAACAACCGAAGTTTTTGTACTCGTTGGCAAACCACTCCAATAGC
GGCATTTTGTCCTGGACCTCTAATTCAGCTGAGGTGTCAGGATCCTTGAAGTTGCTGTGATCAGCCTCTTGGTC
CTTGTTTAGGTGCTTAATGACAATCTCGTTGGTAACACTATTTTTCAGCGCATAACGGTTTATATCAAGATTTTC
CCAAACAACAAGAGTTTCAACAGCTCCCATCTCAAGAGCTTTTATTGTGTCATCCACACCAAATACATACTTT
CCAGTATCTTGACTGATTTCCTCAAAGAATTTTCCTATCAAGCGCTTTTCTTGTATAAACTTCACATTCGCGAG
AATCTCAGCAGATAGCTCAATAGCCTGATTGAATCCATTTTCCCCACCATAGGACACATCAACCACATTAAGT
ATCTTTGTTTGTAGGCGTTGATCAAACATGTCAGACTGGCTCAGCTCTGTCTTGAAATCAGCTGACCCAGCAA
GTATTAGTCCAGATACATTTGGCTGGCTAGTGGCTGGATTAATGAAGAACTGAGTAGCAAGCTCTGCTGTCTT
CCTCACATAGTTATGCCGTTTCTCCATTCGAAGACGAGCAAATCGCAGAGCTGATTGACCTCCTCTTCCGTGCT
TCTTGGGAAGGTCAACAGTGAATTTATGAAGGACTTCCCTAGTGTTGCCACTTAAGGTTCCGAAAAGAGTGCC
ATTACCGTCCATGACAATGAAACCAAACTTCTCATCTGATTCCAACAGCTCGCCCAGAGGCTCCGTATGAAA

CTTGTTGTCACATAGGTACAGAGACGCATTTATTGGTTTAAAAGGTGTAAGGTCAAAGGTTACCTTCTTTTCCT
TCCCATCGTCAGTCATTATAGTTCCAGTATAAAGGACCAACCCATTAGGAGGTACCTTATTATACAGCTTAAG
CCTCTGCTGGGCGGACGTTATTGCACCAAGGACGGACTGACGATTTACTCTGCTCTTAATATTGGATGCAGTA
CCATATTCTTCTGCCAACATCCTTGTTATCCGGGATATCTGATCACCAGGAGGTATAATAAGAGAGATCATAC
TGGTACCATTTCCTCTTGCAGATTCCATGGCTTTGATTAACTTTTTCATTTTCCATATCTCAATATTCTTGTCATT
TTCTTGGCCATCTGCCATTGCAAACTGCTACTACCACAAGCTGAGCTACAAATTAATCTGACCTGTTGGAAGC
AGCAGTCGCAGTAGCTCGAGCAAAATGCTGGCTGTGGGAAAATTGTGGAAACCCTTTTGAGCTACTATTTATA
CGCTGGAATA
>TCONS_00041215
AAACAAAATGAGCTTGGCTTCAAATAATCCTTGAACAAAATAGGGAGAGCTAACACATGTAATCTACCAAA
AGTTTCAATTTGTTCATGATGTCTTAAAATCTGTTCTTAGTATTTCCTGCCGCCAAAACCACCAGCACCACGAC
CGTAGGTTGGGAGGGCTGCCACTGCCTGTGGTTCTGCAGCCTGCTTGACAACACCAGGAAGGTCAGACATGC
CAAGAGCTGAAAGCATGTCAAGGGTCTCCTTGTCGACCTCAATCAAATCAGTCTTAATGGCAGACTCATCGG
GAACAAAGTCCATACGCCTCTCACGCTCCTCCTCTTGCAATTTCAGGGAGATGCCTCTAACAGGTCCCTTCTG
AATGCGCTTCATAAGGTGGGTGGAGAACCCAGCAATCTTGTTGCGGAGACGCTTTGAAGGAATTATGGCAAC
TTCCTCCAAGATCTTCTTGTTGGTGTGGAAATCCAAGGTCATTTTTGAGTAGTACTTCTCAATTACCTGTCTAGA
TGACTTCTTCACAGTCTTGGTGCGAACACGACCCATCCTTGC
>TCONS_00041256
TGATTATATTGAACCTCTTTTTGATTGTCTTAAGACGATTAATAACCTTTTGGTTATTTAATGACAAGCTGAAAT
GAGTATTAACAGCAACACAAGCAGCATTATATGCATTTTCATTGAAGCATTTGTCAACTTTATTTCCATTTCTT
GCTTGGATAGCAAGTGCTTCAATCAGACACTTGTCCATTGCCGGAGACCATACAACATTTTTTCCCCTGTGCTT
CGTCTTCTTTCTCCCACTATCTACGTCATATTGGTCAATTCCAGTAGAATGATAGAGGGGAGTGTTGTCAGGGG
CATAATGAATAGTAGGTGATCTCCTGCGAGTCAGTGGTGGTCTGGATCTCATTGTTTGCCGGAGCTAGGCGGT
GGCGGAGCAGCTGCTGACGGTGGCAGTCGGTAGTTGAATTCACAACTTCATTTGTAGCAATTGGCTGGAGTA
AGGTGTCGTCAGGGGCTATGGTTTACTG
>TCONS_00041262
AAGAGAAAATAGAATGGCTAACCTGCTTAGTGGCATTACGAACATGTAAAGCCTTAGCTTCCATAGCCATTA
CCACCAATGCCTTGACCACAACCCCAAATGTATGCTGCTAACTTAACTACAATGTATTTACTCGTCCCATGCT
GGTAATTACAAGCATACTATAGGAATTTATATATCCATTTTCCACTAAAATCTTTGCTAGTTAAAATGGGTATC
TGCCACTAAACATGTAAACCAGGGCGTCAATGCTAGATAAATGAAGCAAGATTAGCACATTTTCGCTGCAAC
CATGTCAGTCGTATGCTGCTGCTGTTAAGAGAAATGAACATCTCCCTTAGACTGGGATTGTCGAATAGATCCA
AAGCTGCATAATAAAGATGGTCTGTAACGCCTTCTATCTCATCCAACGCTTTGACACATTTATGAATTGAAAA
TCTTCCATCAACTTGAGGCATAACAACTGTCTGCAACTTTGAAGCAGCAATCATCTCCTGTATCGCTTCTGCCA
TAGAATCAATTATCTCTTTATCCCCACAGCTCTGGTCAGAAGCAGGAGCTATATCTGATGAACACTTCCTCTTT
TTACAAGCTACATTCCTTGTTGCAGTTTGTTGTGAAGTAGCAGCGCTATGTAAAGGTGTTGGCGATGAAGGAT
TTACAGGAGCCAAGTTGCTAGTTTCTTTAAATGAGCTATCTAGCCCAGCAGATTTGTCCATCCCTTCATAGTGA
CTTGATTGGGCGTACTTCCCAACCGCACCCGAGTCAGCAAATATTGTGCACAGCTGCTCATAGATAGGGCACT
CCTTGGGTTTGCTTGATTCAGGCTTCGGCTGAGCCCCAATGTCTTCCCAAAGGTCAAAACCAATGTAGCAAGA
ATCGTCCAAAGCATCATTTTGATCAGAAGCAGATTTAAGGTTACAGTAACGAGTACGCAACACATCAAGGTG
CTTTCTTAGCTGGTTATTATTGAAATTAAGATTGGTCTGTCTATTGAATTGATCACGTATGTAATTCCATGTTTT
CTTATCAAATACATTGTTCGGCCGGTTCCCCAGCTTAATCTGCTCAACAACAAGGTCAGCAAAGACCTTGTCC

AGGGGGGGTGTCCACCTTGTTCTCAAGCGTTCTTGCTTGTGTTGCATTTCATTAGCTTCAGAATCCATCGTTGA
TAATGGAGTTACCGGCTACGGGGAAGACAGTTCTCTTC
>TCONS_00041263
CCTTACAAGAGAAAATAGAATGGCTAACCTGCTTAGTGGCATTACGAACATGTAAAGCCTTAGCTTCCATAG
CCATTACCACCAATGCCTTGACCACAACCCCAAATGTATGCTGCTAACTTAACTACAATGTATTTACTCGTCC
CATGCTGGTAATTACAAGCATACTATAGGAATTTATATATCCATTTTCCACTAAAATCTTTGCTAGTTAAAATG
GGTATCTGCCACTAAACATGTAAACCAGGGCGTCAATGCTAGATAAATGAAGCAAGATTAGCACATTTTCGC
TGCAACCATGTCAGTCGTATGCTGCTGCTGTTAAGAGAAATGAACATCTCCCTTAGACTGGGATTGTCGAATA
GATCCAAAGCTGCATAATAAAGATGGTCTGTAACGCCTTCTATCTCATCCAACGCTTTGACACATTTATGAAT
TGAAAATCTTCCATCAACTTGAGGCATAACAACTGTCTGCAACTTTGAAGCAGCAATCATCTCCTGTATCGCT
TCTGCCATAGAATCAATTATCTCTTTATCCCCACAGCTCTGGTCAGAAGCAGGAGCTATATCTGATGAACACT
TCCTCTTTTTACAAGCTACATTCCTTGTTGCAGTTTGTTGTGAAGTAGCAGCGCTATGTAAAGGTGTTGGCGAT
GAAGGATTTACAGGAGCCAAGTTGCTAGTTTCTTTAAATGAGCTATCTAGCCCAGCAGATTTGTCCATCCCTT
CATAGTGACTTGATTGGGCGTACTTCCCAACCGCACCCGAGTCAGCAAATATTGTGCACAGCTGCTCATAGAT
AGGGCACTCCTTGGGTTTGCTTGATTCAGGCTTCGGCTGAGCCCCAATGTCTTCCCAAAGGTCAAAACCAATG
TAGCAAGAATCGTCCAAAGCATCATTTTGATCAGAAGCAGATTTAAGGTTACAGTAACGAGTACGCAACACA
TCAAGGTGCTTTCTTAGCTGGTTATTATTGAAATTAAGATTGGTCTGTCTATTGAATTGATCACGTATGTAATTC
CATGTTTTCTTATCAAATACATTGTTCGGCCGGTTCCCCAGCTTAATCTGCTCAACAACAAGGTCAGCAAAGA
CCTTGTCCAGGGGGGGTGTCCACCTTGTTCTCAAGCGTTCTTGCTTGTGTTGCATTTCATTAGCTTCAGAATCCA
TCGGCGGTGGAAGTGTTGTGTAATCGAAAGGCAAAAGCCCAGAAACCTACCGTTGATAATGGAGTTACCGGC
TACGGGGAAGACAGTTCTCTTC
>TCONS_00041426
TACATGGACAGAAGCAGCTGGATAACATGACCGGCTGATCTTTATTTGTCTGAAGGCGTTTACACAATTCAGA
CTCGTATTGCAGTACACAACCATCCAAAGAATTCATCAGTATAACCTCATCAGCTGTGATGACGCATCTGATC
TGTTCAAGGTTTACCACAATAGCTTGCTCTCGCCCTAATATCGTGGAAGGATAGATAAACTTAGGGTCCAAAA
GACGGAGATCCCGCGCTGGTAAAGCACAATGTCTCATAACAGTAGCCTTATCCAATTCCAGAATCTTTGAATT
CCCATGCTCATCAATCTTTATCCAAGAGCGACTCCCGTGACCTTGGCCTCTCCTCTTAAGACCAGGAAGACCT
GGCCCGTGAAAATTGTTATTTGCCTGTACATCTGTATTAAATCTAGCAGAAGTAGATTCTTGAAAATCTGTAA
AAACAAATTGACCATTTTCTTCTGCCATTTGCTTTCACGTTCTGTGAGTTAGTAACAAATCTCAGAAACAAAG
ATTTCAGCATTGAACAAAGCCACTAGTACTCTTAAGCCAACTGTAGCACTGTTGCTTTTAGCCGAGCGTTAGT
GTAAGCAACAGCAGTAGCTCTTCTTATTATAGTTCCCGTAACCTTTTTATTTT
>TCONS_00041484
GATCTTTTGAGCTCCCACTCCTTTTTCTATTCACAACCTTCACTTCAATGCATTGCTGTGTGTCCTAAGTAACTG
TTACATATTAATTAGCAGCTAATGGCGTGAGACATGATATTGCAGAAAAAAGAGTTTTAGTGTCCAATTGGCA
ACTTAGTCCTGCTTTCCTGCCATTAAAAACCGCATAAAAGACTCTTGTACTTAAGACAATTTATGGGCTGCTAT
ATGTCGTAAGACAAATCTGATTTCAGAAAAAATTATGGCTTGTAAAGTATGGTTAGAAACAAGAACACGCAT
CTGTGACTCATGGATTGGAAACACCACTGATCTTGGTGTATGCCAGGTTGCCAGTATGAAATATAAGAGCTAT
TGGGAACTCATGGTTTGGGAACGCCATTAATCTTGGTGTATCCCAGGTTGCCAGTATTAATCTGGGCTGGAAC
TTCTATCGCCGGTCCAGT
>TCONS_00041498
TTTATTTATGCTTTCCTTCTTCTCGGGGAAAAAAACCTCCCCGATCTACCGTTTGCTTCGTAAGTTGCAAAAAC
GCCTCTTTTCATCATTCACAACTTTTTGAATTCGGACATTTCTAGAAACCTCTATTTCTCCTTCTCTCTCTCTCTT

TCTCTCTCTCATCACACGGGAAGCAATTATAGCAATCGATCTGCTTTCGGTGCATCCAGCAACCGATCGGCTC
TTATTCAAGCTGCCAGAATGGATGGAAAGGAGGTATAAAGGAAGCGAAAAACAACAGTCTTATGGGACGAT
GCTTAAAAGCAGGGGCGAGCTAGCCTTGAAGATACG
>TCONS_00041663
TTGCTGCATCCTGATTTGACCATGTGACTCTGAGAGAGTCTGTAGTGAACTAGGTACCGCTAACTTGTAGCTTG
TGTACATGAGCTTATAGTCTGTGTTCTCTACCAACTTAGAGTATTTCATTTTTGTTGCTTTTCCGCTCTTTCAGGT
TATGTTCTCTGTTAGCCTGTTAGGTAGATTCTTTAGAATTAACAAGATGTTGATTTGCAGTTTAATCGCACTTTC
TCAGTAGTTCTGGTCCGTTGGTGCAGGTTTACATGTCGCTCTAATGATGTTCACTGGCTATCTGGTGGGATATT
ATGCATTCAGGGCATTATTTAGTCACAGTCCAGCAATGAGCGCCGCCGGAGGCATTCTTGGATTGGTTGGTGG
CATGCTTGTTGAGACACTTCTCTTTATTATTAGAACTTCAAGTCAAGATAGAAAACCTACTAGAAAATCCACT
TCTTTCACTTCGAAGCAGAAAAAGAATCAGTAGGCTTCATCACAAGAAGTTGTTCCTTTCTAACTTTCTGAGA
GGAGAGTACTTATTGACCCAAAAAGGATGATACTGAGGG
>TCONS_00041669
AGATTTACTCCTCTCAGAAAGTTAGAAAGGAACAACTTCTTGTGATGAAGCCTACTGATTCTTTTTCTGCTTCG
AAGTGAAAGAAGTGGATTTTCTAGTAGGTTTTCTATCTTGACTTGAAGTTCTAATAATAAAGAGAAGTGTCTC
AACAAGCATGCCACCAACCAATCCAAGAATGCCTCCGGCGGCGCTCATTGCTGGACTGTGACTAAATAATGC
CCTGAATGCATAATATCCCACCAGATAGCCAGTGAACATCATTAGAGCGACATGTAAACCTGCACCAACGGA
CCAGAACTACTGAGAAAGTG
>TCONS_00041874
CTTAAGGTTACCAACATAGACAAATTTCCAGCCATTGAGACCGGCTCGGACAGCCAAATCCATGTCTTCTAC
AGTTGTTCTGTCTTTCCATCCTCCTGCTTCATTAAGAGCTGACATTCTCCAAACTCCAGCAGTGCCATTGAAGC
CAAAAAATGCATGGATTGCAGATCCAACTTCTTGCTCCACTGTGAAATGGTAACCAAGTGATATTTCTTGCAT
TCTTGTCATCAAACACTCATCA
>TCONS_00041934
ACTCCATCCACTTAACTATACAAGCTGAAGCAGCAGCACAAATCAGTAATCAACTGTTCTGCTGGAGGAGGT
GGAGGTAATGGCGTGGGGGAAGGGGGGAGGCGCTTTTGGAATATTGAGGAAAAGAAGCGTTGAGCACCGGA
AGTTATGAGCTTTGATGCGGGGTCCACCACGAGCTTAGTAAGCCAACTAGAGTTTCGAAGTGCCGTCGGTGG
ACGATCGTACGGAGTATTATTCTGGTTCCGACGTAACGGCCTTTTCCTAAACTTTCCTCCGGCTCCTCCTCCTTC
GTATGCCGCCGCCGCCGCCG
>TCONS_00041988
GCAACTTCAAAACTCTTTCTTTAAGGGATAAAATCTGTTCCTTATTTTGATCAGCTTCCAACCTGAGTTTCTTTT
TCAGCTGCTTTATCTTCAATTTTGCAGCTTCAAGCTCTGACACAACCTTAGCATAGTCAGCCACTTGAGCCTCT
AACCTCGTCTTCTCTGCCTTCAAGGACTCGACTTTAAGACCCAAAAGCTTAGCCTCCATATTGTTTATTTTAAG
CCGATTTTGAAGCTCCATGACAGCAGATTCTTGCTCCTTGAGGCCATAATACTCAAGCAGTTGGATCTCAAGA
GTCCTCTCCCTTTCTTTAAGAGTTTTAACAATATTCTTAAGGTTCTTAATCTCTTGCTCATGGTTATCCGCAACA
ATTTTATGTTCTCTGGGTGAATCTGCGTCCTGCTGTGGTGTTTCAACATCCTTCTTTGGAGAAAAATTAGCCTTT
GAAGATAAACTAAATTCCTTCACAATTTCATTGAACTCGGGCAGAAGAAACCCATCTCTATCTCCATCAGACT
TGCAACTTGTAGATGGAACACTTATAGAACTCTCAATAATGCGTTTTGGTATGGAGAAGTCCTCATCTCTCTCT
GGCACATCAAAAACTGAATTGCG
>TCONS_00042015
TGCTAATAGGGCCATATGCATGTAGTTGTTTTGTCTACTATGCTGTATTTCATTATACATTGGAGAGGAAAAGT
TAATTGAGGGTGGGGTGGGACCTGAAGAGAAAATCTCATAGGAGGCTCCTTAGGTTTTAACTGAAGGATTAT

GTTCTCATGTAATTTGCTTTGGTTGGTTCAGGAGGCGTGCCCCCTGGGACTTCTCCATTAATAGATGAAGGTGC
AGCTGGACTCCGGCTCTGAATCGTATGCATTGATTACAAAGGTGCTGTATTTAGAGTGCAGATGATACAACTC
TTTACTATATTTGAAGTCCATATAAATAGCACTTCAGTATCCACTTGGTCTCAAAAGGCACCCACGCGATCTA
CAAAGGAATGTTGTTCTTGCCACTGCTGGTGTTATTCTTCGCTGTGTTTATGATTTTTTCATCTCAAGCTTGAGA
TGCATCAATTTTGCCAGAGCGCTCATGATTGGTACTAGGTTATACTTATTGATGTTCTCGATGTTCTGTATTCAG
TTTCTATAAATTCTGTAATAGCCTAGCCTTTGTAAAACTAACCATTGTATGTTGGACGTACTGAATTCTCTTATC
AACAGGGAAAAGGTTCACAGCGGAAAGTGTACTTAAGAGTTTTTCTA
>TCONS_00042033
TCACAAATGCAATGTTTTGATAAAGGAATATGATTGCAACTCCTTTGCAAGATTGGTTCAAAATAGGAAGTCT
CTGATAAGGTAGTGTAAAGTAAGAAGTTTGTGTCCCCTTAATTAGAAAATTGATATAAGTGGATCCTCAGTAA
ATCTGCACCCGGCATCATGTTCAATACCTTTCAAATCCCTATGCTGTTTTGAGGAATGCATGAGAATGGCACTT
CCTTTCTTCCCAGCACGTCCAGTTCGACCAGATCGATGAACAAAGATCTCTGAAGAGTTCGGAAGTTCATAAT
GTATCACCAGATCAACATTAGGTACATCAAGTCCTCGAGCAGCAACATCAGTGGCCACTAATACATTGAATT
GACCTTGTCGGAAACCGGACAGAGTTCTCTCCCTCTGGCTTTGCGTGATATCCCCATGCAAAGCTTCACATCT
CAAAGTTTTTTGCATTGCACTCGCCAATCTATCAGCATCACGCTTTGTTTGGGTAAACACAATACACTTGCCTC
CTTTTGCATGCTCTGATATTAAGGGTCCAAGAACTGCTGGTTTCTGACGCATCTCACAAGCAATTGAATACAA
GGAAATGCCATCGGCTAATTTCTTGTCAGAATCCCCTACAAGATCAATGTGAATTGGCTTCTTCAAGAACTTG
TTGGTAATTTTCATTATCCAACTTGGCATTGTGGCTGAAAACATCATCGTCTGATGTTTCTGCCGAACATTTTCA
AGAATGGTTTCAACATCCTCCGCGAAGCCCACATTAAGCATCTGATCAGCTTCATCTAGAACAACGAACTGG
ATTTCTGATAAATTTAAGGACCTCCTCTTCAGCAGATCAATGACTCGACCAGGTGTTCCAACAACAATGTCTG
CACCCCTTTGAAGAGTACTCATTTGGCGTTGAATGGGGACACCACCATAAACACACAGTGTATCCAAACCGG
GAGCAGATTCATAAAATTCTTTCTCCACCTGGCGTGCAAGTTCTCTAGTGGGAGCCAAGAGCAATGCTAAAG
GGTTTCTTCCCCGTCTGTATAGTTTAGAATAACCAAAAAGAATTTCAATTTTAACGAATTTCTTATTCTCAACA
AATAAGCAGAATCAAATAAAGAATCATC
>TCONS_00042034
CTGTATTGGACCATATAAAAACCACCCATTACGAGCGTAACATCCTACAGACTTAGATTTTTTAATCTTCTTTT
CAACATTTTGTGTTCAGTGGTTAAAGTATCTACACTTGTTGGAAGATGCTTCTGCAGAGTATACAAGTCACCGT
TAAGCACATCCATTCAAAATTAATCAAGAAAGGAGAATGAAACTTGCCTTGAAATGGAGTTCCTCCCTGAAC
TCTGATCACCACTATCCTTGCCATCTCCGAAGTCATCAAAACGGCTTGAACGGTTACTGCGGTCAGACTGTGA
ATCTCCAAATCTACTTGAACGCCCCGAACCAAAGTTAAGGAAACCACCCAAACATTTTGAGCCTCCAGAACC
ACCAAACTTTCCTGAGCGGCCCGAGCTAGTTCCACCGTAGCCACCCCCTGATCTTCCAGAACCAGTTCCACTG
TATCCACCTGTTCGAGAAGATGAAGCGCCTTCTCCACCAAACCTGCTGCCTTTATTTCCATAGCCACCAGAAC
GACTTCCACCAAAATCACCAGATCGACTACGCATGGTGCCTCCATAGGAGCCAAAGCGGTCACTGCCTTTGC
CCATGTCACTATACATGTCTGCTGCTCCAGCCTCTACTTTAATACTTGGGAGCTCAGTAAATCTGCACCCGGCA
TCATGTTCAATACCTTTCAAATCCCTATGCTGTTTTGAGGAATGCATGAGAATGGCACTTCCTTTCTTCCCAGC
ACGTCCAGTTCGACCAGATCGATGAACAAAGATCTCTGAAGAGTTCGGAAGTTCATAATGTATCACCAGATC
AACATTAGGTACATCAAGTCCTCGAGCAGCAACATCAGTGGCCACTAATACATTGAATTGACCTTGTCGGAA
ACCGGACAGAGTTCTCTCCCTCTGGCTTTGCGTGATATCCCCATGCAAAGCTTCACATCTCAAAGTTTTTTGCA
TTGCACTCGCCAATCTATCAGCATCACGCTTTGTTTGGGTAAACACAATACACTTGCCTCCTTTTGCATGCTCT
GATATTAAGGGTCCAAGAACTGCTGGTTTCTGACGCATCTCACAAGCAATTGAATACAAGGAAATGCCATCG
GCTAATTTCTTGTCAGAATCCCCTACAAGATCAATGTGAATTGGCTTCTTCAAGAACTTGTTGGTAATTTTCAT
TATCCAACTTGGCATTGTGGCTGAAAACATCATCGTCTGATGTTTCTGCCGAACATTTTCAAGAATGGTTTCAA

CATCCTCCGCGAAGCCCACATTAAGCATCTGATCAGCTTCATCTAGAACAACGAACTGGATTTCTGATAAATT
TAAGGACCTCCTCTTCAGCAGATCAATGACTCGACCAGGTGTTCCAACAACAATGTCTGCACCCCTTTGAAGA
GTACTCATTTGGCGTTGAATGGGGACACCACCATAAACACACAGTGTATCCAAACCGGGAGCAGATTCATAA
AATTCTTTCTCCACCTGGCGTGCAAGTTCTCTAGTGGGAGCCAAGAGCAATGCTAAAGGGTTTCTTCCCCGTCT
GTATAGTTTAGAATAACCAAAAAGAATTTCAATTTTAACGAATTTCTTATTCTCAACAAATAAGCAGAATCAA
ATAAAGAATCATC
>TCONS_00042046
ACCTCGTTAGTAGCAGTTGTCGTCTGTTCTCACAAGGAAAGCAGTTAAACCTGTTTGACATACTCTGTATACA
ATCTTGCAATTCAACACTATCCAAGTATTTATTTATGGATTTTCAGTTGCTCAAAAGTTTTAAATACAGATGAG
GATAGCAGTAGAAGGGTGTATGCACGGAGACTTGGATAATGTCTATGCCACCCTATTGCAGTTGCAAGAAGT
TGAGAATATAAAGATTGATCTCCTTTTATGTTGCGGTGACTTTCAGGTAGATACATTTGTTCCTCGTTAGATTTT
GACACATCAGTTGTTATTTGATTTGTCTGCACGAGTTAATAATGGAAATCGGGCTTTGGCTCATTTTCTTTACA
TATACCAACCAATATTGACTACACTGGGATTTTGTTGTTCTGAATCTTCTATGCTTATCGTTCATTCATTGGAAT
ATGAGGCTTAGCTCATTCATTGTAACAGATTCTATTCAGTTTAATTGTGCTCTTCATTATTTGAATCATTCTTT
GTTAATACATCTTTTTGGTGTTTTGAATCTTCTATGCTTCATGATGTTGGTAATTGATATGGG
>TCONS_00042101
CCTCCTGAAACTATCTCTTCTTTTTACCCCCCTTCCCAGATTTTGAAGAACCAGCTGCAGAAGAGGATGCAGG
CTCCTGCTTCTTAGTCCTATTAGGATGTCTCCGGACCAACTCTGCAACATGGATCATCAGCTGTTTCCGGGAAG
AAATGGCAGGATTGTAAGGGGTTCCATCTTCCTTCTTCAACAAAACTCTCACTCTTCCTCTTTGCATGAAATCT
CGCGGATATGCCTTATCGAGCTGTTACAATCAATCAAGAATATAATTACAAACAAATTCTTTCTCAATCTTGT
AAGTTCGCTGATTTAAAAGTTTAAAAAACATTAATAAAACAGTATTGTTCACAATTGCCAT
>TCONS_00042218
AGCGTTTGCAGGGGCCAATGGAAGAGGATACGCTCAAATCTCATTTTGAGAAGATCATCTTGATTGGGAAGA
AATATCTTTTACGGAGGATTTAGGAAGAAGTTGGCAAGGATGACTGTGTTAGAGACAAAGCTATGGATGTTG
ATTCTGCTACAGGTGGTATGTCTTAAACCTTGCAATTTTGTGTGTTTATTGTATTCTTTTCCTTATGTCTTGGTTG
T
>TCONS_00042231
AACCAAGACATAAGGAAAAGAATACAATAAACACACAAAATTGCAAGGTTTAAGACATACCACCTGTAGCA
GAATCAACATCCATAGCTTTGTCTCTAACACAGTCATCCTTGCCAACTTCTTCCTAAATCCTCCGTAAAAGATA
TTTCTTCCCAATCAAGATGATCTTCTCAAAATGAGATTTGAGCGTATCCTCTTCCATTGGCCCCTGCAAACGCT
>TCONS_00042243
GAAAAAGCTGGCAGCTGTGCAGGAAGTGCGAACAAGTTCTCCCTTGCGACTTTCCCTCTCTCTGTTTCCATTCT
CAATCGGCGCACTTTCCCCAAACCCTATTCTCTTTGATCTTCATTTATTCTTCAGCTTTAATAGACCAAATCTAC
GATGCTGGGCGTTTTAAGGCGCAAAGCTTCTGCTTCGTGTTTAGGAAAAAATCTGCATGTGATTCGCCCTGCA
GCTTCTACATCTAGAATTCCCTCTGCCGCATCAGAAAAGGTAATGACTATTTAATTTTC
>TCONS_00042246
ACGCCTTCTTCAGCAAATCTCGGAGGATCATCTAGCTCCTCTAGATGTGCATTTGCTGAGGGTTTCTCAGTGCC
GAAGAAACAGAAATCATTTTGGAGACAGGAAATTTTTGCATCAAGACTTTCTTGTAAGATGTCCATTCCGTGC
CTGGATATTTAATAGTAATATCTCTATATGGTTCTGCTGTGATTTCAGCAGCTGTGCTTCCTAAATCCTTTGCTG
AATCCTCTGGTTTTCCACTATTCCCCCTTAATGGATCAAAAAACTCATCCCCGCCA
>TCONS_00042247

CCAAAACAAACACAATCTCGTTATAAGATATAGCTAGCAACTGCAGCATAAGAAAAGAATTTTCCATTGATC
CATTGAATAGAAATAGAAATCTTGATAACTCAACTTTAAAACAAATTTATACCATAACCCAAAAGAAGACCA
CTAACTAGGGATCCACTAAGATCACCTCCTTCCTGCACAATTTATATGTAGCTCAATAGCAGGTGTATGAAGA
TTAAAAAGGCATACTAACCGAAGAAACAGAAATCATTTTGGAGACAGGAAATTTTTGCATCAAGACTTTCTT
GTAAGATGTCCATTCCGTGCCTGGATATTTAATAGTAATATCTCTATATGGTTCTGCTGTGATTTCAGCAGCTG
TGCTTCCTAAATCCTTTGCTGAATCCTCTGGTTTTCCACTATTCCCCCTTAATGGATCAAAAAACTCATCCCCG
CCATAATCCGTAACATCAACCTGATCACACGACGGTGATTGAAGGGAGAGAGGATGAGTCTCAGCTGGGATC
CGGCGGAAGGAGTGAATCTCTACTTCAGCTTTGTAA
>TCONS_00042260
TACCTTTTCTGATGCGGCAGAGGGAATTCTAGATGTAGAAGCTGCAGGGCGAATCACATGCAGATTTTTTCCT
AAACACGAAGCAGAAGCTTTGCGCCTTAAAACGCCCAGCATCGTAGATTTGGTCTATTAAAGCTGAAGAATA
AATGAAGATCAAAGAGAATAGGGTTTGGGGAAAGTGCGCCGATTGAGAATGGAAACAGAGAGAGGGAAAG
TCGCAAGGGAGAACTTGTTCGCACTTCCTGCACAGCTGC
>TCONS_00042261
GGAAAGATTGAATTGCAACATAGCTCTATTTGCACATCTCAAGTATAGATGGTTGCAGCTGATTAAATAATCA
GGGGGAAAAACAATGTTCAAGGCCTCCAAAACTGTGACATTGTCATGTTTATTCTGGTTTTAAGCTTGAGTGT
ACACTCAGGTATACCATGAACAGTCAAATCAAAAGCCAGAGATGATTGTATATCTTCAAACATCAAGGAGCA
GGCGACGTGGATCTTCAACCACATCTTTAATCCTTCTCAGAAAGTACACTGCCTCTCTTCCATCAATCAGCCTA
TGGTCATATGTCAGCGCAATGTACATCATTGGTCTTGAGACAACGTTGCCTCCAACAACCATTGGGCGATTTA
CAATTGAGTGCATTCCCAAAATAGCCGACTGAGGAGGATTTATGATAGGGGTACTTAGAAGACTTCCATACA
CACCACCATTGGATATTGTGAATGACCCTCCAGCCATTTCATCAATAGAGATGGTTCCATTAGTTGCCTTCTTA
GCGAGTCCGTTTATTGTCTTCTCTATCTCAGCAAAATTCATCTTCTCAGCATTGCGGAGAACAGGGACGACCA
GACCCTTTGGGGTACCAACAGCTATACTGATGTCTACATAGTCCCGATATATGATATCATCGCCATCAATAAC
TGCATTAACTATAGGTTGATTCTGGAGTGCAGTAACTGCTGCTTTCACAAATCCAGACATGAGTCCTAACTTC
ACTCCATGCTTTTCAACAAATGCATCTTTGTAATCAGATCGGAGCTTCATCAAATTTGTCATATCAACTTCATT
GAATGTAGTCAACAATGCAAATGTGTTCTGAGAATCTTTCAAGCGGGTAGCAACTCTTTTCCTGAGCCTGGTC
ATGGGAACTCGTCTTTCCCGCTCTTTAGGGGGAAGTTGGGGTTCTGTAGCAGAGCGCTTAGGAGGCGGCAGTG
AAGTTTCCTTAGACTTCTCCTTGACAGGAGTTGTCTCAACTTTAGTTTTCATCTCTTCCTCTTTTTTCTCTTCAAC
GGCAGAAGGTGGCTTCGGAGCTTCTTTTTCAGATGGCTTCTCAGATGGAGCAACATGATCTACACTTTCTACA
CCCTCACCAGATTTTGAAATAATAGCAACCTTATAGCCTGGTTCCACAGTGTCTCCTTCCTTAGCTACAAACTT
TTGGATTACACCGGCTTCAGGGCTAGTAACATCAATTGTCACCTTATCTGTTTCAATCTGAGCAATTGGCTCAT
CAACTTCTACTCTGTCACCCGGATTCTTCAGGAATTTAGCCAGTGTGCCGTCACTTATGGATTCACCCATAAAA
GGAACAACAGCATCAACCAGATCACCACTATTTGAACAAAAAGGCCTGCCGCAAATATGTAGCGTGAGACT
TGAGTGAAGATTGGCCACAGCTCCCCTTTCTGGCCTCAAGT
>TCONS_00042384
ATTGTCTTCCCTGACAGAAAGCTTCTTTATCTTTGAAAGAAAGAGAAGAACTTCAGGATGGATGCTTGAAAGC
TGCTGCTTAACAGGTTTCACCTTGTCAGGTTTCAGTGGTAGCACTATAGTTGTGGCTGGAAGGGTAGCAGAAG
ACCCATAGATTTGTTGTATAACTGAAAGAGTTGGGTTTGCCTCCACCCATTCAGGTACAATATAGCCTACATT
GCAGTGCTGGCAAGGTTCCTCGCTGAATCGTATCTGGTACCCATTGCTGAAGATGTAGGGCTGCGCAGTGATG
AGAAACACACTCTTGAAGCCAATGCCTTTCTCGCCGATGTAGCCACGTTTGCGATTGCCCTTCTTAGTAGAAC
GTCCGACGCTGCAAATGGACTCGATATTTTTGCGGGAGAATCCTTTCTCGTTATTGAAGATTAAAAGCGTCGC
CTGAGCTCCGGTGTCCGTTATATCTTTGGAAGTAATCACAAATTCCAGTGAGGGGTCCACGCCTTCGTGGTAC

TCGTTATCCTCCGCGTTCTGAACAAGTTCCATGAAAAAGTGAACATCCTTAGCGTAAAGTTCAGCAGAGAGAT
TTTTGACAGCTTGATGAAGGTCCTCAGTGAGTGGATTAGTTTCACCTCCAATGGAGAACTTGCTTCTCCTTATT
TCTTCAATGTGCTCTTTTGCCGTCGCCATT
>TCONS_00042412
AGCTTTACGACTACCCCAGCAGCAACAGTTGAAGCGGCCGAAATCGGTGGATAAGTTCCTCATACAGCCAGT
ACGCAACGTTTTTTCATTACATAGTCATAATCTGACGAGCAAGACCAGGATCCTGTTGTAAGAGCTCTCTAAG
GTTATTCTCCACCTCCGCCAGATGCTTTTCCAGATACTCTTTAGAAGTCTGCAAAGATGCAATTGCGGTTTCAC
TATCCTTGAGTTTTTGCTCTTGCTCATCCATTAACACAGTCTTTGGCTCTAAGATAAACGTTCTTCCTATGGCTT
TGTATGTATTAGTATCATCAGAGAGCTGCTTCAGTTCCTCCAATGTCAAAAAAGCGCGCTTCTTTTCTGTTTCTT
TAGTTCGCATCTGCGTTTGAACCTGCTTCAATTTCCCGGTAGTCTCAATCATGCGACCTTGAATTTCAATAAAA
GCCGCTCTATTTGCTTCGTCGGCCATTTTTTCAACTTCAATTCCGAGTGCTGATACAGAAGCTGAAGCCCTAGT
ATAGGATTCTACTTTCTG
>TCONS_00042572
TAAATCTCTCGGTTTGGATTTTGGAAACAACAATAGGAAACGGAAGAGAATAAAGGAAAATCGAATAGGTA
GACAATGACTCAGAAACACAAATAGAAGCCGGAGGGCGGATACCTCATTGGCCAAACCGGCCGTCGGCATC
GAATATCTCTTTGTTTTGCTCAATCGCCAAATCCAACGATTGGAATATGAACAAGATTCTCTTCTGATAACTAT
GTTGTGCGGACTTTCCAAAATGATGTCGTACCCGTGT
>TCONS_00042602
TCATTTTGGAAAGTCCGCACAACATAGTTATCAGAAGAGAATCTTAGAAAAACTTCAAGTAACTATTGTGAG
GGAAAGAATTAATCCGCCAGCTTAATTTCAGCAATATTGGTCTTGTTTTACACGATCACAGTTCTGATGAAAG
ACATATCTATATTTTAATCTACGAGTGCCACAAATTCATTGAGTTTAACTGTTCAAAATCCGAAAATGAACAT
GTATAAATGCAAGTGAAATTGAACTGACTTTGATCAGAAAGTGATGGACCTTGTTCATATTCCAATCGTTGGA
TTTGGCGATTGAGCAAAACAAAGAGATATTCGATGCCGACGGCCGGTTTGGCCAATGAGGTATCCGCCCTCC
GGCTTCTATTTGTGTTTCTG
>TCONS_00042680
ATGAACTGCAAATGCTCTTTCACAAAAATGATCGCCATTTTGTGGTGAAAGGACAAAAAGGGAAGCAGTATC
ACTGCTACAGTTCAGGGATCTGATTACACAATTACCTCATAATTTTGCAGCAGAAAAATCTGCCAGGATCAAT
CAGACCTTATTTGAGTACCGCTGACCAATAAAGAGCTACAATGTTGCATTCTGACTAGCTACCTTCTGTAAAT
ACCTACTCCTAATTTGCACTATGAATCCAATCCTATCCACATAGATGTTCTTAGCCATGCGATGAGGGGATG
>TCONS_00042833
TTGTATCAAAATGAGGAACGCTATGCTGCCTTAACTCATATGGTGGCTTCTCATGGACTGGATCGTAGTTCAA
AGGTGCTTCGTCAGACGAATATTGGCACTTTTTTCATGCTTTAGTTCGACTGTTTCGTAAGGTTAACTAGGTCC
CAGGAATTAGTCAAAGATGGTAAAAAGTTATCAGCTGTCTGGTTCTGAGTTTTGACTCAGACACAGCATGTCC
AATGTTCTGCGGTCTGCTACTGTCTAATATACAGATTATCGAGGTTAGTCTATAATTTTGACGTTTTCCTATTCC
GAGAAACGTTCGCTTCAGACGTAATG
>TCONS_00042834
AAATGAGGAACGCTATGCTGCCTTAACTCATATGGTGGCTTCTCATGGACTGGATCGTAGTTCAAAGGTGCTT
CGTCAGACGAATATTGGCACTTTTTTCATGCTTTAGTTCGACTGTTTCGTAAGGTCTGGTTAACTAGGTCCCAG
GAATTAGTCAAAGATGGTAAAAAGTTATCAGCTGTCTGGTTCTGAGTTTTGACTCAGACACAGCATGTCCAAT
GTTCTGCGGTCTGCTACTGTCTAATATACAGATTATCGAGGTTAGTCTATAATTTTGACGTTTTCCTATTCCGAG
AAACGTTCGCTTCAGACGTAATG
>TCONS_00042836

ATTTAATTTACCAACCAACCGTCTATATCTTGCAGGATCGCTAAGAGGCTCCCCCTGTCCTGGCAGAAGTTTA
GAATTCGGATCCATCGGAGTGTCAACAGGTTTACAACCTGTCATTCCTGTCTCCTCAAGAATATCTAAGGCAT
ACTTCTGTTGTGAGATCACAATACCTGAGCTAGACTGAGCGACCTCAATAATCAGAAAATACTTTAATCTGCC
CAGATCCTTAGTCTGAAAGTGCTGAAAGAAATGTTGCTTCAACTTACTAATACCATCCTGATCATTGCCAGTA
ATAACAATATCGTCAACATAAACGACCAGATAAATACA
>TCONS_00042878
CCCCTGAAACACAAACAACAAAACCTCTTCTTCGGTCAATGGATCTTCTTCAACCTCCTTGTAGCGCTTGCAC
TTCCCCTCTTCTCCGTCGACTATCTTCTCTCAATTCCGTCAACTTCTCTTTGAATTCCATCAAATCCGCCGCAAA
CTACTCCGAAATTTCAAACTCCTGTCGCCGGCCAAAATGGTAGCAGCTATTCTCCCTCAGTTCCCACACATAA
AGTTACCGTTCTCGACCGCCAACGTGGCGTGGTCCACGAATTCCTCGTTCCCGAGGACCAATATATATTGCAT
ACA
>TCONS_00042951
GCATTTCATTAAAAGCCTTGTTCAACAACATTCCATATATAACAAAACAAGTCTACATCTTTCACTCTCACTCA
TTCATAACAGGCTTGTTTTCACTAGCTTTTGGCCTCTGTTCCTTTGGCTTCTGCTCTTCTGTTTTCCTCTTGTCGG
AGGCGCCTCCCTTCTGGCTAAGGAAGGATCGGAAAAGCGGCGAGTTAACGTCGTAAATCATCGTCGTCGGTC
GGAATTAAACCCTAATATACCCAAAACCCTAATCTCCTCTC
>TCONS_00043018
CATCAACCTCTTCCCCGCATATACTCTTTGTACAAGAAGGGTCTGTATTTTGGTTTTTCTGCAATCTCCTTTGTA
AACTGGGGAAGTGGTTCAATCCATTTGTCTCCTGACAAACTGTAGAAGAATGCAAATGAATTCCTTGATCTTC
CATTAGGTCTCAGCACCCTATGGCTTGGACTTTTGAATTTGTCGTTCGAAAGGACCTGTAAAATATATGGTAA
CAACTCTGCCAA
>TCONS_00043021
CACGATTATATAATCTTACCAGTTGGATGTAACCAGTCCAGAAACGTAACTCTGGCTCTTTCATATGGATCTTC
TTGCAAGAGTTTAGGTATGAGATTCCAAGTTTTATCAATATATTCAATGATGACAGGTGATTCCACAACAGCT
CTACCGTTGTGAATCAACATGGGTACCGTTTTGTGGACTGGGTTGTGTTGGAGAAGCAATGGACTCTTGTTGCT
CAAGTCCCTCTCGCACAAGCTCGAAAGGAGATGATGTCGAGTTAAGAAGATGTTCTGGAGCTAAATCGAATA
ATTGGAGCTTTGACGTCTG
>TCONS_00043031
AGATCCAAGCACAATGACCTCCCAACATTCAGACAATGCTTACACATCTACTTGCTTTGGGCACATTTCTTTC
ACCATCAACTGAGTTGCTCATAATAAAGATTCAACTAGCAAGATGAAACCATTGTGTGATATATTAGGTATAT
TAAGACAAGATTGACAACTCAATTTATATAGAATGGGGAATGAAGTCGGATATATTCAACTAAAGCAAAATG
CTCCTCAAAAGTTGCAGCAATCAGGTCAATTTGTAGTGCACAACTAGTAAACACTTGAATGTCAAAATTTCAC
CAAAATTTAAACTAAAGTCCCTCTAAACTCTCAGCTTAGTTCTTGGCCGCCAGCTTTTCTTTGGCTTTTTGGATC
TTCAAAACCTTCTTCACAATTTTCTGCTCTTCCACCAAGAAAGCTCGAATAATCCTCTCCCTTACTGCACTTCC
AGACAATACTCCACCATAGGGACGGTTCACAGTCCTCCTGTTCCTAGATAATCTGGATCTCTTATACTCAGTT
GGTCTCAAGTGTGGAATCCCCTGAATCCTCTTTCCAGTTACAGGACACTTAGGACCACTAGCCCGCTTCTTGG
TACTCTGGTAAATCAACTTCCCACCAGGGGTTTTGACGACACGGTGCTGGTTGGATTTGGTGGCATAGCTGTG
GCGCTTCCGGTAGGTGAGTCGCTGCACCATCTTCGGAACCTCTCTCTCTCTCT
>TCONS_00043153
AAGTATGACTTCTTTATCATGCTTAGAAATCATCACCTAGGTCCTGAAAGAATTGAAGGAAACAAGGTAGTC
GTGGTGTCTGCGACTCTTTTGATTGCTGCAATTGTTTGTGAATTTGGAACACTATAGTCCAAGTGCGATTCAAT
GTCCATTGTTCCCTCTATTACTTTAACCACCATGGACATGGAAGGCCTCAGAGTAAAATCACTTTGTAAGCAC

CACACAGCAAGCTTCATCATACGGACGACTTCTGATGTATTGAATTGCAGATCATCCTTGTAGTTTCCAATCA
TATCGATCAGCTGATCGTTTTCAGCCTTTTTCATAAACAAGGTAAGCAAATGTGGATATTCCAGTGAATGTGA
ATAGTCCACATTCTTACGCCCACAGAGGATCTCCATCACAACAACTCCAAAGCTGTAGACATCTGCTTTTTCT
GTAATGAACGCGCTAAACCATTCAGGAGCCAAGTATCCGGGAGTTCCCCTAATTCTGGTCACTATCCTGCTCT
GATCTTTATCCACCAACTTGGCCAGGCCAAAATCAGATACCTTTGCACAAAAATTGTCATCCAACAGAATGTT
CTGTGGCTTAACATCTAAGTGGACTATTCTTTGCCTGCATTCTTCATGAAGATAAGCCAATCCTTTTGCTATAT
CATGGATAATGCTGCTTCTTATTTGCCAATTAAAGGTAAACTGCGTAGAGCCTGTGCCAAAAATCCATTTATC
CAATGATCCATTACTCATGAAATCATAAACTAATAGCTTGTGCTCTTTCTCAGCACAAACTCCAACTAGTCTA
ACAAGATTAACATGATGGATGCTTCCTATGGTTTGGACCTCTGCCAAGAATTCCCGTTTCCCTTGGCCAAAAC
CATCAAGAACTTTCACGGCGACTTTCTGACCATCTTGAAGAACTCCTTCATAAACTGAGCCAAAGCCTCCCTG
ACCAAGCTTTTGCTGGAAATTCCCGGTTGCCACCTTCAGCTGTTCATAAGAAAAT
>TCONS_00043155
CTATATCATGGATAATGCTCCTTCTTATTTGCCAATTAAAGGTAAACTGCGTAGAGCCTGTGCCAAAAATCCA
TTTATCCAATGATCCATTACTCATGAAATCATAAACTAATAGCTTGTGCTCTTTCTCAGCACAAACTCCAACTA
GTCTAACAAGATTAACATGATGGATGCTTCCTATGGTTTGGACCTCTGCCAAGAATTCCCGTTTCCCTTGGCCA
AAACCATCAAGAACTTTCACGGCGACTTTCTGACCATCTTGAAGAACTCCTTCATAAACTGAGCCAAAGCCTC
CCTGACCAAGCTTTTGCTGGAAATTCCCGGTTGCCACCTTCAGCTGTTCATAAGAAAAT
>TCONS_00043368
AGGAAGTAGCAAGTCTATTTGGATCACTAATCTGCATCCATCTCTTCAAGGGCAGCTTTAGCTGCAGTCTTCG
CTTCCTCTAAAATACCATCAGGAACCTTCTGATGTTGGCGGTTGGACTCGTCACATATCCAACTCATTTCAAGT
TCAAATGCCTTGTCCTTTGCCTCATCATGTACTCCATAAATTATTTGGCCACCTCAATCACCCCTTGTCGGCA
GGTCATCTCCGATAACTTCAGTTTTTCAATTTCTGTTTTAGCAGCTTGTCTGCCCTTTCCAATAGCAGCACCAA
AGTACCTGTATGAGACACCAGAAGGTTCAACCAGGTACAGTTGAGGTCCATCTCTATCATACCCTCCAAGAA
TCACCCCACATCCGAAAGGCCTGAGCCACCAATACAGTGTGCATAAATGCACATAACTAGCAACGCGTTCCG
CAAGTTCTTTCACTGGAATTGGCTCACCATATGTACTTTCGAAGTTGGTTGCTTCAGACTTAGCCCGGGCAACA
ATTTGCCTCCCATCTGCAGCCAAACCTGCAACAGCCATGCCGGAATGGCGATGAACGGAGTGAATTCTCTGA
TTTGATCCAGGCAACATCATCTTGGATGCTATTAGCTTCTCCACTCCCAAGACGATTCCGTCCTTGCATTTGAT
GCCGACAACAGTGCCGCTGTTATCGACAGCTTTGCCGGCGTATTCGATCTGAAATACTCTGCCGTCCGGCGAG
AATGTAGTCACGGATAAGTCATAACCTGTTCCTATGCTGGCCATTTCTCCCTTCTTTTCTTCTCTACTAGAGCTT
CTACTGATTCAGTTGAGTTCAATTTGGAGCTGTGTAGTGACC
>TCONS_00043415
GGGGTTTTCAGGTTTATGGTGACCCACGGAGAAGGGAGTGGACGACTTGATCTCACAAGTCACAAGACCTCG
CTCGACAACTGGCACAACCTTACTTGGTAATCCTTAAGTTGCAACAATCATGTTTATAGTATGTTTTTCTTTACT
GTGAAGCTTTTGTTGCTCCGAAGGGCTTGCCGAGTTCCCAGTAGATGTCTGAGGAGCGAGGAGGGAGAACAT
ACTTGCTGGTTTACGTGGAAAAAG
>TCONS_00043429
GATTGTCCATGTTGATCAGTAGCTTAGTTCAGGGATCTAATGTGCAGTGCGCAATATTCCATGCAGATAACCA
CCAGTCATAACAAGAGACACAAAGGAGACAACACCAGCTGGAAAAACCTTCTTTGACTTCAGAAAACGAGA
TCCCATTACTCCCAGAAGTGCAGCCGAGAGGACGAAACCCATTGATGATGCCAAAACAGGCTGTGTTGGAA
GCATAACATAAACAGAGTACAAAAGGGAGGCAGAAACTCCACCTGCAATTAGGGACTTGCTGCCACTTTTCA
GATAACCCATAAAACCACCAGCTCCAACCAAGAAAGCATAGCCCAATGTCAATTTTTGGGACATACAAAGG
GCCATTTTCTTTTTATTGTTATGATCCTTGCCTTCATCTGACTCCCCCTCGTTGCCACCATCATTATTATTATTAT

TGCCACCATTATCTCCCCCACCACCACCACCGCCATTTGCACTCCCAGAATCATCTCCACCACCACCACCTGT
CTCTGGCAGAATATCAATTCCTGGCCCTGCTGAATCAACAGTGAAGGTGGTTGGCATTGTAGAATCAGAGAC
AACACTGGCTTTGACTCCCCAGTAGGAAGGCTTTGATCGAGTGGACAAGTTTGGGTACACCGTGCAAGGGAT
ATTCAATAGATGTAGAGATGAACGAAGTGACCTCAATGGCTGACTATGAGCAAAGAGATGCTGCCTTTGACT
CCAAGCTTGGACGTGAAAGTGAGGCAGCACTATGGAGGATTGAGCAATGGCCAAAAATTCTCCCATGATGTA
TATGCTGAATTATGAGAAAGTTATTTATGGGATGTTTTGTTTTCTGTAGCTTGTGTATTCTCAGCAACTTCCACA
AAAATCAGTCTTCCATCAACAATCTTCTGTAACAACGCCAAAAGGTGAAAAGAGCTTCTTCAATCTTTCATTC
GTAGTGTAAAACGATAATCTGCTGACGAATAGCTG
>TCONS_00043470
CATCACATGAGGAGTTGTCAACAAGCAGTTTTAGACCTGGACAGAGCTATTCATTGAAATGAATTACAGAGT
CTCGACTCCAACGGATGAACCGGGGTTGGCAGGATCAATTCTACTCATATTTTCCTTGTCAGCTTTCTCTCTTT
CAGCTTTCTCCTTTTCAGCCTTCCGCTTCGCCTTCTCCATCCTCTCCCTTTCCATTTTCTGAATGTAGACGGTCTC
TTTAGCACGTTCTTCTTCACTGAGGATTCGACCTTTGCCGTCGGTGAAGTAGCGAAGGCCCGAAGCCGATGAT
CTCCCACCACCTTCCATTAAACGACGGACTCCACTGCTTGAAACGACTGATCTCATCGCCATATTTATCTTCTC
CTGGAGATTGAACTTTAAGACTGCAAAACAAAGTCGTGACTTGGTGAGAAAGTCAGCAAAACCTACGCCGTG
TCACCTGCAAACTTG
>TCONS_00043496
CTTGGCCTTTACAAAATACAACCCGATGCCGATATAGGTAGAATCCAATTCAAAATCTGCAAAAAATTTACA
AGGGTTTAGTTAAAGGGTTTTGTGGGAGTGATTAGTCAATGGAGAGCAGGAAGTTGGTAGCAAAGCAATTCC
TCAAGGACAAAAAATTCTGGGTCGCCTCTTTCCTCGTTGTCTGGGCTGCTGCTCTGCAGGGGCACATGATGTG
GTTGCAGAGGCAAGATTCTTTCAAGCACAAATTTGGAAACTTAAACGACCAAGGCACCAATCAACAGGATA
ATCACTCATCAAATTAACTACTTTGGTATCATAATTGGAATCTTCTCTCGTATTTCAATGCTTGGAACTGGTTTT
TCTCTACAACTATCCATTGCACTTCTGGAGTTATCAATGATAGATCCAGATAGAAGTATTCAGGATTTAGCAA
ATAGCAATCTCTGTTATGAATGATGCAATTAGCATTCCCATTTTATTCTCTTGGGCTAGTCTTGTCAAATGATAT
TTGGTCTCT
>TCONS_00043497
CTTGGCCTTTACAAAATACAACCCGATGCCGATATAGGTAGAATCCAATTCAAAATCTGCAAAAAATTTACA
AGGGTTTAGTTAAAGGGTTTTGTGGGAGTGATTAGTCAATGGAGAGCAGGAAGTTGGTAGCAAAGCAATTCC
TCAAGGACAAAAAATTCTGGGTCGCCTCTTTCCTCGTTGTCTGGGCTGCTGCTCTGCAGGGGCACATGATGTG
GTTGCAGAGGCAAGATTCTTTCAAGCACAAATTTGGAAACTTAAACGACCAAGGCACCAATCAACAGGATA
ATCACTCATCAAATTAACTACTTGTAGTCATGTTTCCACCATAGGACTGGCAGGTTATTACCAAGTCATCTGTT
GGATAGTTTCCAAATTTGCTAGATTGCAGTAGCAGTAGCCAGTAATT
>TCONS_00043505
GGCTACTGCTACTGCAATCTAGCAAATTTGGAAACTATCCAACAGATGACTTGGTAATAACCTGCCAGTCCTA
TGGTGGAAACATGACTACAAGTAGTTAATTTGATGAGTGATTATCCTGTTGATTGGTGCCTTGGTCGTTTAAGT
TTCCAAATTTGTGCTTGAAAGAATCTTGCCTCTGCAACCACATCATGTGCCCCTGCAGAGCAGCAGCCCAGAC
AACGAGGAAAGAGGCGACCCAGAATTTTTTGTCCTTGAGGAATTGCTTTGCTACCAACTTCCTGCTCTCCATT
GACTAATCACTCCCACAAAACCCTTTAACTAAACCCTTGTAAATTTTTTGCAGATTTTGAATTGGATTCTACCT
ATATCGGCATCG
>TCONS_00043522
AAATAGCATTACCTGTTCCTAAAGGCAAAAGAGGTCTTGAATATCTGCATAGTGGACTCCATCAATTCATCAT
TGAAAGGTACAAACTTTGGCTTCCCTTGAGTAGTACCAGAACTCAATGAGATAGTTTCAATTGGTTTTCCAGT

AAGAATGGGAGAAAGATCACCATCAGCAATTCTTTGAATGTAAGGCTCCAAATCATTGTGAGTGACAATAGG
GACACAATTCTTGAAAGTCTGAGGATCAGTTCTGCCATTCAAACCCCATAGCTGCAAATATTCTGTCCCTCCA
TTCTGTTCCAGTATCTTTTTAAGTGTCTCTTCTTGAATTTTGCCAGCATCTTTTGTCAAATCCTCAAATTCCTCAA
TCACTTTTTCTTGATCAAACTTCTTCTCAATCTTCTCCACCAACATCTTCATGATTTTCC
>TCONS_00043562
GGAAACAGCATGTGATAGAGATGCATGAAACCAAGATACATGTGCGTACATCGCCAGAGCCTTTCACACCA
GTAGTATTGGAAGGGATAGGTACTTCACACAGGGAATTTTCATGTAAAATCCAACAGCGGGTTGAGTTGTCCT
GGCTGCAGCTGTATAGGCAACGTCCACCAAAAGCCCAAGCTAAACCTGTAACTACATGATTATGAGCATCAC
AGGAGCCAGCTTTCTCAAATCTACGAACAAGAATGTCACAACTCCACACTTCAATTGATCCTGATCCGTTGCC
AATGGCTAAGAGCATTTTATTAGGTGATTGCGCAGATACACTGAAGGAAACCACAGAGGCCATTGCAAAGTC
GGCGGCTTTTACCTCCTTCAATAGAGAAAAGGGTGAATCACTA
>TCONS_00043563
GGCAGGCATGTGCAGGGACTACCTCTTATCAACGGTTCGTACTTCTTCTCCAAGAAGTTCCAGTTGATCCTTTA
CATCTTCACCACAAATTGCAACCCACCGCTCCATCTGTGCTGTTCCAACAGGGACCCAATAACTAGATTTACT
ACCCTTTCCATCAGAACCAGAGGCGTCATCAAGAAAGACAGTAAAGCTATAGCCAACCAGCCTATCTCTCAG
TTCCATCTCACTATTCGAGTGAAGCTGTGTCCAATCCAATCTTTCATCTTCAGCACTGTTGAAGCCCTCTAAAA
ACTGCGATTGCCCATCAACTTTATCTGTCTCCACCTTTTTCAAGACTACCAGCTTGCTGATGATGTTGAGGAGT
TGCAACTTACGTGAGGAGATCTCCGAGAGACATTTAATTGCTTCAGACAAGTTTGCAGAAACTCCAAACTGA
GATCCAACAGAAGATTTCAACCATTTAATTATGATATGTTTCGCAAATATAGAAATAGACTGCTTAAAGGCCA
>TCONS_00043572
GAGGAAGAACTGGTAATAGACACAAAACCATCCATTCTGGCTCTATATTTGTTCGAATAAAATGCTTAGCCA
ATTCCATGCGTCTAACCAAAAAGTCCTTTCTTCTTCCAACTTTTCGATCTTCCCATTCATTCCCTGTGTGCCCTC
GTCAAAACCACTAAAATCATAACATAAAAATGCATTGTGGAATATTCGAAAGCTTCTTTTTTTAGTCCAGCA
AGGTGGTAAACATAAAATAAAAAAGCAGAAAGAGAAATGAGACTTTTATTTTGATCGAATTTTCTATTTTATA
TATTTTATTTTACTTTTACAAAAATTTTACAAAAAATGGGAATTTTTTCTTGTTGCTTGAATATTAACTATTCAA
TTAAAATGAAATATTACAACTATAACAGGCATTTTGGTTTTCAAATTCAAATT
>TCONS_00043682
ACTTCTAGTACACGCTAACATAATCAAAATAACCGCATCGCACCATCTAGAGAAAGATCCTCCATGGAAACT
GGTTTCCATCAGCGGTACTTAATGAAGCCAATTTCCTTTGCATTGCTGCGGAAGCACTGCCTGCAGCACATAA
GCCCATACTTCCTAATGATGGCATGGGGATTGCCACATACACGGCAGGTGCGAGAGCCAGGGCCGTAGTTTT
TGGGGTGAGCGTTCCAGATGTTGGAGTGACCCATCTTCTGAAGTCGGCTGCAGACTGCGCTTCTGAAATGTAT
GCAAAAACCCTAAT
>TCONS_00043713
ACACCGCTGCTCAACTCCCTTACCAAACTCTCTCCCTTTTTATTTCTGTCAAAAGTTTGAGCAATTTTGACAGC
AATTGAATATCTTAGCTCAGCCATTTGCAAATTCCAATAGTATATTTCCACTTCAGAAGGTATGATGCAAGGT
TTCCACCATGAGCATTTCAAACCTGGTCGACAAAAGCAGACAACTATACAGCACAGCTCTTCATCTTTTGTCA
TCAAATGCTTTAGCTTTTTCTACAGATTCCATTGCTTCTTCAAATGATTTCCAGCTAAGGCAGCATTGAGGAGA
AACGCGCGCGTGTTCATGAACCGCATAACCATCTCCAATTTGGTAAAAGCGCTAGCAGAACCCCCATTTGCA
GGCCGACTATACCCATATCCATAACCGTGATTATTCCTATAACTGTTCTGATAACTATTACGGTAATAATTGTC
ACTATTACCACCACTATAATAATTATTCGCAGAATTTCGGTAACTATTTTGATGAATATTGTAATCGGCGCGTT
TGCGATCGTCGATCAGAGTCTCGTACGCTTCGGAAACTTGCTTGAATTTGAGAGTAGCGTTTTCCTTTAACTGC

TGCGGTGAATGTGCGTGCTTGTCGGGGTGAAATTCCATTGCCAATTTTCGAAACGCTTGTTTAATATCTTCTTT
GCTTGCGCTTCGTGTTAACCCTAATATTTT
>TCONS_00043716
AATAACAACGTGTGATGCAAACAGATTTCAATGTTATATCTATTTACAAGTCAAAAGGAACAAATTCTTGTTT
CATTCCTTCATGATAGAATACCATAAGTCTCTACATTGTCAACTCACCGAGTTGGCTCAAAATAATGCTGATTT
AGATTGAGCTTAAAATCAGGTCGAGGTCTTCCTAAAAACCGAATAGATGTTAGACAGCTTTTGAAAAAGTTC
ATCAATAATTCGACTGAATTTTGAGCCAACTCGGGAACTGGCCAAAGGGTCGAATGGCTTCCCAACTGTCTCC
ACAGTCTCAACACCAAGAGATCCAAGGATTTCCCCAAATTGTTTAGAGATGCTCTGATAACTATTATTAATTT
TCTCTTCTCCCTCTGTTGCCACCTTGATTTGCATTTTCGCTCTCTCAAAATTGTCCAGAACAGATAGAAGTTTCT
CGACAACTTCCCCTTGTGCATTCGTCACAAGAGAAAGTCTTTCTCTCTCTGTTCTCTTACGGAAATTGTCGAAG
TCAGCACTGATTCTAAGAACTCGGTCCCTCTCACTTGACAGTTCTTCAGTCAATGAGGCTACTTTTCTCTGAAG
TTCAATTTTCTCATCTTCTATGGACTTGAGGGAAATTTCTATCTCTGCAACTTTTGAATCGTCATTATTTGCTAA
AGCATTTCGGTACAACTGCAATGAAGTTGCAATGAATGATGCAGTTTCATCTGCGGCTGCGTTGTCACCCTCA
TCCGAAGCATCTTCAGCAG
>TCONS_00043717
TTTTGCTCTTCTTTAGGCTCTGCTGTCTCTGGCTTTGCCGGCCCGGGGCCAGCCGACACCTTCACCATTGAAGG
ACGTAAGAGTCTGTCTCCAAGTTTGAAACCTTTGCGATATTCTTCTAATACAACACCTTCTTCAAATTCTTCTG
AATCCTCACGCATTATAGCTTCGTGCAGCAAAGGGTCGAATGGCTTCCCAACTGTCTCCACAGTCTCAACACC
AAGAGATCCAAGGATTTCCCCAAATTGTTTAGAGATGCTCTGATAACTATTATTAATTTTCTCTTCTCCCTCTGT
TGCCACCTTGATTTGCATTTTCGCTCTCTCAAAATTGTCCAGAACAGATAGAAGTTTCTCGACAACTTCCCCTT
GTGCATTCGTCACAAGAGAAAGTCTTTCTCTCTCTGTTCTCTTACGGAAATTGTCGAAGTCAGCACTGATTCTA
AGAACTCGGTCCCTCTCACTTGACAGTTCTTCAGTCAATGAGGCTACTTTTCTCTGAAGTTCAATTTTCTCATCT
TCTATGGACTTGAGGGAAATTTCTATCTCTGCAACTTTTGAATCGTCATTATTTGCTAAAGCATTTCGGTACAA
CTGCAATGAAGTTGCAATGAATGATGCAGTTTCATCTGCGGCTGCGTTGTCACCCTCATCCGAAGCATCTTCA
GCAG
>TCONS_00043754
TAACTCTCTTCAAACCGGGAAAAAAGAAAAGAAAAAGAAACCTCGAGGGAAAAATAGAAGACGGCAGTAA
TTACCCCCAAAGTCGCCCGCTCTTCTATCTTTGTCCCGCACCCTGCCTCTCGCCTTTCTTCTTGCTCTGTATCTTC
ACCTACAGCACTCTGAGTTGCTATCTCACACATTCTCAATTCCCACAAATCACAACTTACACAATCCAGAGAG
TTTTCTTTCTTCGACTTCGTGCGAGTTTTCGAGCTCCTTTTGACTCTCCAGTTCCATAGGTCATAGGTGAAGCTC
AGCCAAAAGAATTGCCGTGTTCAGAGATTTTGTACCCTTTCGTGGACTCTTTAGCTTTTCGCTTCGTGGAAAGT
TTGACATGTGAATTCCTATGCTGGAGACGCTGCGTTAAGGATTGAGCAATTTTGATTTGGACGTGAGGAGCTG
AGGGTTTCAGAGCAGTTTTTCATTGAAGTTGTGCTATGGAAGCCGCCATTCTCAGCTCTTGCACACCGCTTTCC
ACAACGGCGGACCTCAGGATTCGAGGTTCTGTTTGGTTGATATTACTTTACTTGCTTGAGGGCGTTATCCTATA
AATATAATTATATTTTCCAAGTAAAGCAGTTTTACAAGGGGATGTCATTATGTTAATACCTTCCTAGGAAAAG
CAATTGTTAAGACTTGTAATTTTAGCGGTGAATTTTTCTTGATTTGAACTAGAGAATATATTTTAAATCTCTTGA
TTTTACTGTATACAGATTGTGGCAATATATAACTTGGGTTATGCGCCATTCTTAACAAACCTAAAGCATGTTTA
TTTGCTTCACCAGT
>TCONS_00043804
GGAAAATAAACGATACCGAACAAATCTACTGTGTGTGTGTGTGTTTGCTCGTACGCCGCAAGCGAATGAGAA
AGAGAGAGAAGACAGTGCGTGTTGTAACTGTTCACTGAACGAATCAAATGTTCATTCAAATACCTTCCTAATC
TCCGCCATAGATTTACAGCTCCCAAAGTATTTTCCTTTTTCTAATAAAAAATACACATTAGAATATACATACTC

CAATTAGATTGTACTCCATACATAGTAAGTATTTTATTTTTCTCGGATTTTTTCTTTTCATGTTTTGCATAATCGC
CCTTTACAACTTTCAAAATTAACATTTGCTACCACATATATAATCAATATCAATAGAGAGAGAAATAGAGAG
CGAGAGAGGGTACAGATAATACGCATTTGTATTTTTGGGGGTATTCTTTGTGCTATTTTGTTCATTGCATGCGTT
GAATTGTAGAGATAATTACGGTTTGAATGGTATCCCCAAACAAAGGCAAAGGTGGATGCTTGATGTTAATCG
ATCCTCTCCTTGTCGGCGATGTCTAAACACAACAAGGAACGAACTATCAATGACTTATACAATGTGACTTTCA
CTCTCCCCCAACCTAAGTTTTAACTGCCTCCTAAATCGTTGCATCAATCAAAAGTTTCGATTTTTTTTATCTCAA
TCGGAATCAGTATGGAACAGCATCGTTTGTGGATAAAATCAATGTTGATTTGTTCCTGATACTTGTTTGA
>TCONS_00043813
TGAAAATATACGGAATTCCTCTATGAATCTATGAAAGGTATAAAAAAGAAAGAAAAACACAAAGAAAAGA
ATAGGCTAGGCATAAATCGATATGCTGAAATTGAAATCAAAAATAAATAAGAAGAGGAGCCAATTAGATAC
AAATAATGAATCGTATAAAAAAGAAAGAAAAACACAAAGAAAAGAACAGGCTAGGCATAAATCGATATGC
TGAAATTGAAATCAAAAATAAATAAGAAGAGGAGCCAATTAGATACAAATAATGAATCGTAATAGAAATAG
AAAATAAAGTTCAGGTTCGAATTCCATAGAATAGATAATATGGATGGGATTGTCTATAATGATAGACAAATG
AAAGACTTTCTCAAGATTCTGATTCATCCACTTGAGATTTTAAAATAAAAAT
>TCONS_00043935
AAACTTAAGAGGCCACAGCAGCACCCTTCTTCCTTGGAGCAGCCTCGGTTCCTTCTCTGAAGTTGGTCTTAAA
CCTACGTGGTACATTGCGGAGGTACCTCATGCGGCCAGTTCCAGTGGTCTTTCTCCTAAGTGCCTTGACGCTCC
AGTTATATTTCTGAGACGAGCAGCAGGATAAGCACAAGCTGAACAACGACTCTTCTGGATGTGGAAGCTAC
GACGACCACATCTCACACACAGTGTGTGTGTCTTGTTCCTCCTCTTACCAAAACTACCTGTTCCTTTTCCCATTT
GTGCAGCGGATGTGGGAAAACCTTAAAGCTGGAAAAATTATGCGAGTCGAAAAAGA
>TCONS_00043953
GGGATGTTGCCTCCCATCTGAGTGCAAATGATTACTAAGTGTTCTGCCTTCTTGGTCATTATTTTTCCCTCACAA
AATCGGAATCATTGTGAGCATCATTGTTGATGCTTATCTGTAGGCTGTGACGATAAAGCTTGCTGAATTGATC
GGGTTTTGGTCAACTGGACCCGTATCTTGACAGGGCAAACCAAGATGTCTTGTAAGCAAAGTAATGAGTATC
GGAATAAAATAGATTGTGATTGAGATTGTTGTTGTTGTTGTTGTTGTATGCTACTCTGGTTTGCAGCCATGG
TGGTGTCCTTTTCCTTTGCCCGATTTTGTCACAAATCTGAGCCTACTTATGTACTCAACCAAGAACTAAGAAAT
AAGAATGAGGCAGCATAGTGATGCCAGTTTGTTTCGATGGTTG
>TCONS_00043972
CCCATAGTTATCGTAACATGGGGCCGGAGGGACCGGTCTTCCGTACCATTGATAACATGGGCCCCCGATATG
ACCCTCGTAACATTGTCCACAACAATATCCTGGTGGTGGCGGAGGGTATTCTTGAATTGGCATTACCATTATT
GGCTCTGGTGCCGGCGGTGGTGGTTGTGATGGTGGAGCAACTGGATTAGGTTTAGGAGCTTCTTTGGGCTTTTC
GGGCTCTTTCGACTTTGGCTTTTCAACTTCTTTCGGCTTTTCTGGTTCTTTGGGCTTTTCGGGCTCTTTCGCCTTTT
CCGGTACCTTAGTCTTCTCTGGCTCTTTGGGCTTTTCAATGATGACAACCGTAGGCTTCTCAGGTTGTTTAGGCT
TTTCGGGCTCTTTAGGCTTCTCCGGTTGTTTGGGCTTCTCGGGTTCTTTGGGTTTCACAGGCTCCTTGGGCTTTTC
AGGAGCTTTCGGCTTTGGAGGGTCTTTGATTTCAATGCTCTTAATTACTCCACATCCTTTGCTACACAATTTGTC
ACGAATTTTCTCAGGATCACAACATACCACAGTGATTGTGACTGTATTGGCCTTCTCATCATACACTTGGTCTC
TAATTTGAGGGAATTTACAGAGAATTTTTTTGACCTTCTTATAGCAGCTGGAGCACTGAAGATCAACCTTGAG
CACCATTACAGTGGTCTTTTCAGTTTTGTCACCACCCATGATTGA
>TCONS_00043978
GCTTTGCCTCTTCTTCCCTTACAGCCCTTTCTTGAAGATACAACTCCGTCATTTCCGCTACCTTCCTATTCAATT
GCTCAAACTCATCCAGATCAACCAAGTAATATGACGAAGAATAAGAACCAGACAATCCAAAAGTGCGACGG
CCGAGACTAAACTCAGATCCAAGGCCGTAAACTCTCCCCTTATGCACGCCACCAGCTGCCTTAATCCACATGT

CCATGATCTCCTCTTGGGTTGGTTGGATTGGCCTACCTTGATCATCAATCGGCTGGCTTTGAATGAACTCCTCC
AAGATTCGTTTGAAATTATCATTAGTTGTCTCAGCACGCGGCTCGATCCAAGTCGTTTTTGTTCCATCCTTCAA
CTTCTTCGTGTGGGTTTCCTCAAATACCTCATCATGAGTAACTGGTCTTCCTTTAGCATTTTCCAATCTTCTTTG
ATGAGCCGCAACACTAATTGAGC
>TCONS_00044113
GCGTCCCATTTATTGATAAAGTCATTCCACATTGCTGCTGTAATGGAATCTCTCAAGGCAGAGGCACTTTGTTC
TTGTATTTGAAAAGCCAATTGTGGATCAGGCTGGTCATCTAAATCAGACGATTCTTCTACTGATGTTCCTTCAA
TGTCTTTAAACAACCAATCACTCCTTTCCTCACGCCGGATGTGATTGTGAATAACACATGCAGCTATAACAAT
GTCCCTTTGTGTGTTAAAAGCATATTGAGGGGCAAGTTTCAGAATTGGAAATCTCGTTTTCAGCACCTCAATA
GATTTCTGAATGGCATTTCTGAGTGATGCATGCCGGTGGTTAAATAACTCCTTTGCACTTCTAGGTAATAGATT
AGCACCCCGATATTCATGAAGATGACATCGAATTCCTGGAAATGGAGCAATAAATCCATCCATATTAACATA
ACCTGTATCAACAAGATAATATTTCCCTTCAGGAATTGGAGGGAAGTTCTGATCGGGATCATCTAGGACAGCT
CTCAAAACCCGTGAATCTGAAGCTGAGCCTTCCCAACCAGGGTATACAAATATAAATTGGAGATCAAATGTG
CAAGCGGCCAACACATTTTGTGTCAAAATACCCTTCTTATTACGAAAACGTGATTGATCCTTGGCTGGCACAT
GAGCAGGGATTCGCATGCTATCGATCACACCAATGCAATCCTCAAAATATGGGTAGAATCTATTACTTTCAA
GGATCTGGAGAGGAGTGCTGACTGGAGGAAGTTGAAGGAACTCGCGGGATAACGACTTAATTGCCCTCAAT
ACATTATTAAAATGCCTACTTATTGTTTCACCAGAGTGCTGATAACGCTCTTGTATAACCCTGTTACGTTCGTT
GTGGCCAACAATGTTCAAGAAAATTGCCAATTGCTCCTCTATCATAACGCCAGGTGTGTCACGTAGCATGCCT
CTTTGTCGAAGGATGCTACATAGCTTGTGAAAAACATGTTTATCCATCCTTAACATTTCCCGACACACATCATC
ATTGGCACTGAGTAGTTCGCTCAAGAAGCCAGACCCTTTAGGAGTTGAACTACGAGAAGGCTGCCTAGTTAT
ACAATTATAATAGTGATAACCAGCAGCAGCAGCAACTAATTCCATTTCATCCAATTGTATGTCGACATCATCC
ATGCTTGGTG
>TCONS_00044323
CTCGATAAATACAAACGCCATTGCTGACGCGTCAATTTCACAACATTTTCTCTGCTGTTCCCCCAATACAATTT
ATTCGCTGCGCCGTGCGCCTTTCACTTTCTCTGCAAATTGACTGCTGCTGGAGTTAAACCTCATTTTCCATGGT
GATGTTGGAGCTAGCTTGTGTACATCTCGACTAATCCACAGATTACCTGTTACCTCCCATCAGCGGAGGTACT
GGGTAATTCTGCTCAC
>TCONS_00044508
CTGGGCTTTCTCCTCAGCTTCATATTGCCCTATAAGTTGCGTTTGCTGATTTAAAGCTCTTTGCATGTCTTTATC
ACTCTTATCTGCTAATCTTCCATTTATTGACACACCTGATGCAGTCACTTGCACTTGATCATTTGCATCACTGTT
ACCAGCGCCGTCCGTCAATGATTGAGGCCCATTGTTAGCAGAACTTGGGCGAGCCTCAGAGGCACATTCAGC
AGAACTATTTTGTAACTCATCACTAGCTGATCTGGTATCCCTGCGCCTTATCCGTCGGCATGATTTACCAACCC
GTTTTGAAGAAGAGATGCC
>TCONS_00044521
CCTTGCTTGTCTCCAGCCTTAGAAAACTATGAATCCATATTCCATGATGTCTTTTTGGGGTTTCACTTCATTCTT
CTGCAAATGCAAATTAAAGAGGCATTAGATCCCAGTTTCAGAGCAGAATGGATATATAGGAATCATATAGCC
GGCCTCAACTGATTTTGGATTATGGTTTAGTTGATTAATTAGATCATGGATTCAGCTTATGCAGAGATAAACA
AACTCTAACTCCGGCTTATCATGTCAAGACCATCGTATTCCTTCTTCTGCTTGTTAGCCTAATTATCTCATCTGG
CAGGCGGATTCCTCTAGGTTATATTCTCTTGTCAGTGGACATGGTGGAGTGGAATTAAGCTGTAACGGACAAT
GCATGAAGCAGAGTGTAGCGGAGACTTTAAGGGAATTCAACACACAGCTCTTGGATTTTTCAGGTTAGTAGT
GGAAACATGTAGGTAAATATGGATCTCGACCTTGAGAAGAGAGCTTGTTAGTTATTCTAGGTTGGATTTCTGC
TTTCAGTATATTATAGATGATTATAGAGGACTTACTGAATTTGTTTAGGTTCCATCAAAGTAGTTCGCTACTAT

GGCATAGAATAGCGAAGATGTTGCAATTCATTATTCAATCGTTGGATTTGAACAAGTATAACTTGTTTTGGAA
CTCTGGATGAGCGTGGAATTAGGTTACAATAGGCTACAATTACGAATTAATTGGCATCCGAACAAACCCTAA
CATAAGTCCTAATTTGTGCTAATAAAGCAT
>TCONS_00044523
TTCTTCTACCAAATTCATTCATTCACAAATGCCACTGCGTCCAGAATGAGGTCATTCCTTGCTGTTTGTGTGTTC
AACTAAAGCTCTTCTCTCGGGCACGACGTCGACGACAGGGCAACTTTGGGATGAGAGAACTTGCACCAACTG
CCATTGGTCCTATTATATATTGTATAGATGTCCCAAAAGGCGGATTCCTCTAGGTTATATTCTCTTGTCAGTGG
ACATGGTGGAGTGGAATTAAGCTGTAACGGACAATGCATGAAGCAGAGTGTAGCGGAGACTTTAAGGGAAT
TCAACACACAGCTCTTGGATTTTTCAGGTTAGTAGTGGAAACATGTAGGTAAATATGGATCTCGACCTTGAGA
AGAGAGCTTGTTAGTTATTCTAGGTTGGATTTCTGCTTTCAGTATATTATAGATGATTATAGAGGACTTACTGA
ATTTGTTTAGGTTCCATCAAAGTAGTTCGCTACTATGGCATAGAATAGCGAAGATGTTGCAATTCATTATTCAA
TCGTTGGATTTGAACAAGTATAACTTGTTTTGGAACTCTGGATGAGCGTGGAATTAGGTTACAATAGGCTACA
ATTACGAATTAATTGGCATCCGAACAAACCCTAACATAAGTCCTAATTTGTGCTAATAAAGCAT
>TCONS_00044525
CCTTGCTTGTCTCCAGCCTTAGAAAACTATGAATCCATATTCCATGATGTCTTTTTGGGGTTTCACTTCATTCTT
CTGCAAATGCAAATTAAAGAGGCATTAGATCCCAGTTTCAGAGCAGAATGGATATATAGGAATCATATAGCC
GGCCTCAACTGATTTTGGATTATGGTTTAGTTGATTAATTAGATCATGGATTCAGCTTATGCAGAGATAAACA
AACTCTAACTCCGGCTTATCATGTCAAGACCATCGTATTCCTTCTTCTGCTTGTTAGCCTAATTATCTCATCTGG
CAGGCGGATTCCTCTAGGTTATATTCTCTTGTCAGTGGACATGGTGGAGTGGAATTAAGCTGTAACGGGTGAG
CATTACTAGTTTTATCGCCACCTTATTCTCCAGTGACCTCCCTTATTCAGGCATCACGACTGTTTGATGGCTGA
CTTTTGGTCTCAAACTAGTATACCATAAACTGGGGAAAAAGAACCGGTATCAGTTTGTTCCTGCACTATTCGT
CTGGGATAAACTATTCCAATTTCCAGCATGCATCTGTTTGATTTGATATGGTATTTCTTGTGGTTTATCTTTCTG
CTTATATAGAACTTGTTGAAACAACAAATGCTGTATCAGCCAGAATCTATAACTGTCTCTGGTGATGATGACA
GA
>TCONS_00044530
TTCTTCTACCAAATTCATTCATTCACAAATGCCACTGCGTCCAGAATGAGGTCATTCCTTGCTGTTTGTGTGTTC
AACTAAAGCTCTTCTCTCGGGCACGACGTCGACGACAGGGCAACTTTGGGATGAGAGAACTTGCACCAACTG
CCATTGGTCCTATTATATATTGTATAGATGTCCCAAAAGCAATCAATAATCACAAAACTATCCAATATCTAGG
CAGAGCTGAATGTGCACTCAGGCTTGATGAACTTCTTTCTTCTAGCTTGATCAAGAATGGGATTCACAGAAGT
ATCTCTTAGCTATAGGGCTAGTTGAGAAAACAGAGAGCTGTCAGTCTTCTGTCTAATGCAGTTGTAACCTGTTT
TGAATGTTATAGTGG
>TCONS_00044622
CCTGAAAAACTGATCTTATGAGAGCTTCATCTTTTTCCTCCAGCTTCCTCGCCTTTGCCTCATCTGCCCGCTGCA
AAGCTTCAAGCATTGCATCTACGCTCACAGTTGCATGTCTTGACTTCATCGATTTCATCTCATCTAATGCAGCC
ATAATATCCATTTCTCTCTTCGAATCCAAGGTTCTATTTTCTAATGACTTCATTGCATCACCCATTTCTTCAGCA
TCTCTTTTTTGTTTCTCCTTCTCCATTTCCTCATCTTGGCCACGCCAAGGCTCAAAATTCCTAGTCGCACCTGATT
CAACAGTATAATCGGAATTTTTTGGATCTGTCTTATATGTAATCTCCGCGGAGCACTTTGTGCACTTAAAGTAG
AATCTGAAGATTTGTATTCCTAAGTAGGTCTCGCCAACGACGTCCTCTTTGCGGGAATTGAATTTGGTTCCCTT
ATAGATGTAATTGCCGCAAGTAGCACAACGAATGCTCATAGGAAGCATCATGCGCACTTTCATCTGCTGATTT
GGGAGCTGTCTCCGCCGTGGAATCTTCGCCGGATCGAAATCCGGCGGATAATACTTGTTCAATACTTTCCTTTC
TCCCATGGCTCCTCCTTCTTCT
>TCONS_00044639

GGAAAAGGGAATATACCACAAAACAGAAACCATAAACCCTATTTCTCTCTGTCCCTCTCCCAATAAAGAAAA
TGCCGTCATTTCCTCGAGCAGGCTCAGTTACCATCTGCGAGATCAACCGAGACCTCATCACTGCGGATGAACT
CTCTGATGATGGAGCTAAGGAAACCTACGGCAAGCTTCTGGTAAGTTCTTGAACTTGTAAAAGAATTCTGAA
AGATGAATGGCTTACTACGATCAATACCCAATTAGTTAAAGTGGAAGTGAGTTAAAATATTGTTAAAAATTC
ATCCAATTAGGGTTGGTTT
>TCONS_00044643
AGTAAGCCATTCATCTTTCAGAATTCTTTTACAAGTTCAAGAACTTACCAGAAGCTTGCCGTAGGTTTCCTTAG
CTCCATCATCAGAGAGTTCATCCGCAGTGATGAGGTCTCGGTTGATCTCGCAGATGGTAACTGAGCCTGCTCG
AGGAAATGACGGCATTTTCTTTATTGGGAGAGGGACAGAGAGAAATAGGGTTTATGGTTTCTGTTTTGTGGTA
TATTCCCTTTTC
>TCONS_00044659
CAGGGGTTTTCTCGGGCCTATTCATATGGTACCTAACGTAATTCTATGATACTCGTGGGACTTCGGGGCTCTCC
GGTTCAGCTAGGTCCATAGTTCCGGAATTTATACGCGAATCAAGATCGATAAAGGAAGTTTTACAATCACC
GACTCAAACCCATTGTCGAATTCTACTCAGCGGTATATGGAGTTACTTATGGCAGAACGGGCCAATCTGGTCT
TTCACAATAAAGCGATAGACGGAACTGCCATGAAACGATTATTAGTAGATTAATAGATCACTTCGGAATGGC
ATATACATCACATATCCTGGATCAAGTAAAAACTCTGGGGTTCCAACAAGCTACTGCTACATCCATTTCATTA
GGAATTGATGATCTTTTAACAATACCTTCTAAGGGATGGCTAGTTAAAGATGCTGAACAACAAAGTTTG
>TCONS_00044686
GAGAAAACCCCTGTATAGCTTCTTCGATTTCTCGATAAAGAGCAATATGACCAATAGTGGTTCGAATGTATAT
AAAAAGGATTTGTTTTTAGACTTCTTACTATTAGATAGTGTCCATAAATCTCATAAAAAGTACCTAAAGATTC
ATAGTGAACTTCGATGGGAGTTTCTCTTGAAGCAATAACGCGTTGATCTAGTCGCCACCGG
>TCONS_00044687
AAACTTTGTTGTTCAGCATCTTTAACTAGCCATCCCTTAGAAGGTATTGTTAAAAGATCATCAATTCCTAATGA
AATGGATGTAGCAGTAGCTTGTTGGAACCCCAGAGTTTTTACTTGATCCAGGATATGTGATGTATATGCCATT
CCGAAGTGATCTATTAATCTACTAATAAGTCGTTTCATGGCAGTTCCGTCTATCGCTTTATTGTGAAAGACCAG
ATTGGCCCGTTCTGCCATAAGTACCTCCATATTCCGCTGAGTAGAATTCGACAATGGGTTTGAGTCGGTGATT
GTAAAACTTCCTTTGTTCGATCTTGATTCGCGTATAAATTCCGGAACTATGGACCTAGCTGAACCGGAGAGCC
CCGAAGTCCCACGAGTATCATAGAATTACGTTAGGTACCATATGAATAGGCCCGAGAAAACCCCTG
>TCONS_00044688
AAATACCCCGGGCGGTGCCACAATCCGTTCGACGTACAACAATGTGTTGAACTACTTCAACAAGTCTGCGAG
TGAGATATCCAGCGTCTGATGTTCGTACAGCAGTATCCACAACTCCTTTACGGGCTCCGTAGCAAGAAATGAT
GTATTCTGTTAAAGAGAGTCCTTCGCGTAAATTGCTTTGAATAGGTAAATCAATCATTTGTCCTTGAGGATCTG
ACATTAATCCTCTCATACCTACTAATTGATGTACCTGAGATGCATTTCCTCTAGCTCCCGAGAAAGACATTATA
TGAACTGGATTAAAAGGGTCAGTCATCCTAAAATTAGGATTCATTTCTTGTCGCAAATATTCACTTGTAGCAT
ACCATATTTCAATGGATTGACGTAATTTTTCTACCGCGTGTACATTCCCATAATGATGGTGTTTTTCCAAAATC
AAACTTTGTTGTTCAGCATCTTGAACTAGCCATCCCTTAGAAGGTATTGTTAAAAGATCATCAATTCCTAATGA
AATGGATGTAGCAGTAGCTTGTTGGAACCCCAGAGTTTTTACTTGATC
>TCONS_00044689
GAAATACCCCGGGCGGTGCCACAATCCGTTCGACGTACAACAATGTGTTGAACTACTTCAACAAGTCTGCGA
GTGAGATATCCAGCGTCTGATGTTCGTACAGCAGTATCCACAACTCCTTTACGGGCTCCGTAGCAAGAAATGA
TGTATTCTGTTAAAGAGAGTCCTTCGCGTAAATTGCTTTGAATAGGTAAATCAATCATTTGTCCTTGAGGATCT
GACATTAATCCTCTCATACCTACTAATTGATGTACCTGAGATGCATTTCCTCTAGCTCCCGAGAAAGACATTAT

ATGAACTGGATTAAAAGGGTCAGTCATCCTAAAATTAGGATTCATTTCTTGTCGCAAATATTCACTTGTAGCA
TACCATATTTCAATGGATTGACGTAATTTTTCTACCGCGTGTACATTCCCATAATGATGGTGTTTTTCCAAAAT
CAAACTTTGTTGTTCAGCATCTTGAACTAGCCATCCCTTAGAAGGTATTGTTAAAAGATCATCAATTCCTAATG
AAATGGATGTAGCAGTAGCTTGTTGGAACCCCAGAGTTTTTACTTGATC
>TCONS_00044755
AAATCAAGTGAAGATATATGAAGATCATGAGAGCTGGGAATAATAAGTTACGAAAGTTTGAAGGAAGCGAA
CAGCGTTGCAGCTTGAAGCAACCTCCTCCACTGTGCATGTAGACGTAAATGCCAATCCCATGATAACATTAC
ATGCTGCTGCACCTCTAGCTGTTGCAACTTCTCCTTCAGAATGTGCCAATTGATGTGAGGAAGTTGGAGCAAC
TATAATGGGAGCCGACGTCTTGTTACCAAGTATAACAGTTGACATGTCTATTTTGCTCACATCAACAAGCATT
CTTGGCCGAATAGTGATTCTACGAAATGCTTCTGTATTTTCTTTTAGTGTATATTGATCCTCAGATCCTCCTGCA
AAGAAGTCATAGTACATCTTTGGAAGAGCTTGCCTAGCTAGCTCTTCAAATTCATTTACATTAACTGGTTCACC
TGCCATTTAGCCTGTAGGATACAGAGAAGAGTACACACTCTCCAGTATCT
>TCONS_00044803
ACCCACATTTCTTCGCCCTTCTGTTTTTGCAAGACTTAATAACTCTTCAGCTTCCTCCCATCATCACCCCACTTT
GAGAGAGAAGACATGGCAAAAGGAAGTTACGAGAAGGCCATTGTTTCACTCCAGAACCTCCTCAGTGAGAA
GGAAGAACTGAAACCGGTTGTAGCAGAAAGAATTGATGAAATCACAGCTGAGTTACAAACGACAAGCTGC
>TCONS_00044825
CCAGAAAGTCCAACTCGTAAATCCACCCGATTAAGTTCTATCTCAGTCAAATTTAGAACCTTTAAGTGCAGAG
TAATCCTTCCATCACTTGGATCAGTTAAATGATATGCTTCAACATAGCAAGGATCACTGCTGCTAGTCAAAGT
ATATGCACTTGGAGGAACCTTGAGTAAAGTTGAGTCGACTCGTTTAGCCAGCAATAGTGCTAGCTCACTAGG
ATCAGGCCTCCAAAGCTCTAAAATAGCCTTGTGAACCAATCCTTC
>TCONS_00044941
CCCCATCTATAACCATTGGGACATTGCACTCTCTCGCATGCTTCATGATATTACTTACACAGTCCAGGAGAAA
CGGATCCCTTCCAAGGCCTGGACCAACAACAAGACAATCAAACCTCTCCATCCACTTCTCAACCTCAGCAAT
CACTTTAGCTGCTATTGAACTTTTCTCCTCGTCCCTAATACTGTAGGACTCTTCTAGAATAGGGTGCACAATTA
ACTCAGGACTGTAGCTTTTTATAACAGGAGCAGCATCCTTGGTACAGAATACATGGGATAAATCTGCACCAA
TCTTTAAGGCTGAAATTGCAGAGAAGTATGGTGCACCAGTGTATTCTCGGCAGCCACCAACAACAGCTATCTT
CCCAGCTTGGCCTTTATGCTTA
>TCONS_00044989
ATTTTTATAAACCATAGGCTGTGCTATAAAACCTTTTAATGGTACATACATTAAGAATTCTTAATAGAACTTGG
TTGCTAGTACATCTAGGAGTTTTAAACTGGCTTAATGCTGCTCCATTTCCTATGAAATGTTCCAAAATCTTCTT
AAATGTACCTCTCTCAACAATGCAAAGTTCCAATGGTCCAATTGAATTTGTTTTTCTTGATACAACATATCCAT
GATCCACAAATGAAGCATCCATTTCTCTACAACATTCTCCTAAAACTTTTTCTTCAACTTCTCCTTTGATTTCCC
AATAAATCACATAATGGCCTGGTCTTTTTGCTACATTTGCATGACTTGTGAAATCAACTAGCTCAGCTCTTGAT
TTACTAAGTATCTGCGAACCTCTCTCCACCACTAACTGGAGGTCTTTTTCGGTGTTCTTATCGATGTTTACTGTT
AATATCAGCTTTCTCCTGTAAAAGTGGTGAGGACAATCTCATACTCTTGTCCAATCTTGACTTTAGATAAGGG
AACAGGGTCATCTTCAATGAAGTCATCAGTGGCTAGATTGGCATCTTGGCTATTATTGCAATTTTGTGACTTGG
TTCTATGTAGTGGTATGAATTCAAAGTAAGCAAAAGTGGGTATTATTGCGAAAGTAACTTTCTCTGGTGGAGA
TGAAGGTTCCACATTTACTCCAATCCAACTTTCTGTGGACCCATAATCAGCACTCACCAAAGGCAACTCCCCA
GCATAATGCCTCAGTTTTGTTAAATATGGTTGCACTGAGCCAGTCATTATTGAGTAAACATATTTAGCATTTGG
CCATAATCTTGGAATTATGCTAAACCAATCTTCCTTTTCTACTTCCTCACAAATTGATTCAATTCTTGAAGCCA
ACTCTGGATTTGGCAAAGTCATACCTGTTGAAGACATAATCAACTAATTAAAA

>TCONS_00045017
ATAAAGGTAAACATAATTCAGCATAAGAAAGATTCATTGTCACTTCACTTTACGTTTACTACTCTATATACATT
TATCTGCCTCTTCCTCCTTTACATGAATAATAGGTTCTTCCACACAAAAATTTCCATTCCCTTTTCTTCCCAAGT
CTAAATTTTTCCATGTCAGTTCAGTGTTTTCAGACATCAAATTAGGCCCTTCCCCAACTATCCACTCATCCATG
GGATCAAGAACATCGTCTGTAATGTCATTACTTCCTTCTGGATCAAAAGCTTGTAATTGCAAATTACAATGGA
CAAATACCAAATCACGCATCCTCTGCTGCTCTATCGGATTCCTCCCTTGTGTAAATAGTTTCTCGACCAAGCTC
CTTTTTAGCCTATAATGTGAAGCACCATTGCATGTCTGACTAAGGATTCGAACAGCAAGCCTTTGTAGTTCGG
GACAATGACCTCCATATTGTGACCACCAAATTGCTGGGGAAATATTTGATAACTTCTCTTTGAAGCTACCAAA
ATGAAATGTGCCCCTCCCCATGCGATACTCATCAATTTGCAGCGTGATCTTATCCTGTATATGACGATCTTCTG
TCATACGAACAACACAGTAACAAAGACCACAGGAAACTTCCACATCAGTGTAGAAGTCACTCGAATAAAAG
AGGCTTGGGTTCAAAAAGTAACCCGAGGCATGGAGATGACTGTGGAGGTATTCATCCCACACGTCATCTATG
GCTTTCCAAAATCTTGCATAATGGGATTTCTTCTCTTTGAATTCCTTTTTGATTGTCTCTTTAGCTTGATCTATTG
TGTCATATATAAAACCAACTAGTGGCTTATTAGTACTATTTAGCAATTTTATAATTTCCACAAGGGGAATGGTT
GCCTTCACAGCCATCAAAGCCTCAGTCCAAAAGGATTCATCTTTGACCATTTCTGACATTCTTTTACCCTCACG
TGTGGAAGCCAAATTCGAATCAGAAGACTGAAACATCCTAATTAAGTGTTCTTTCTGTGATACAATGTTCTCA
AGAGTCATGAAGGGCACAATCGACCTTATCTTTGAAGGGCATACTAGCTCATCAGGACAAGCATCTCTCAGA
AGCTTTAGGACAGTAGCATTACCGTAAATAAATTGTGTCAGAGATTTTGCCTTCTCCAGAACTTGTTGTATCGT
GTCTATCGCTGTGAATTTCTGTAACATAAGTTCCATGCAATGAGAAGCATCTACTGTCCAAAAAACTGTCTTA
AGTTTTTCCACTAGTTTCTTGCCTGCTTCCATCATGCAGGCAGCAGCTGTCGAGTATGCAACTATTTGGACCAC
ATTTTCAACTCCAACTTCCTCAATAACTTCCTCGAAGAACACTTGCATGGCTTCAACATTGCTATTGATAGATG
AGATATCTGACGACCGAAGGTAAATGGTGCCTCTTGGGCAGTAAACTAGGATATTAATCAGATTTCGACCCTT
TGAATCTACCCATCCATCTAGCAAGATGCTACATCCTGTACTTGCCCATGAATTTCTTATCTTCGTTACATATT
GTTGCATCTCTTCCACTGCATCCTGAAGGATCCGCCCTTTCAACTCGTGACAACTTGGGAATATTATTGTCTTC
CCAGGACTAAGGGTGGCTTTTACCATCTTCTGGAAGCTAGGTGCTCTAATAGCGTCAAAGTCAATTCCTTCTTC
ATAAAAAAATCTCCCTATAGATTTCGAAATCTCTCGTGAGGATAAATCTACAACAGAACTACCAGCTACTTTT
GAATTCACTCTATGATGCTTTTTATTTCCATTTTCAAAACACCGAGTGTCTGTCTTATTTGGTTCACCATCACCT
TCTCGAGGGCACCAGTTCCGCTTCAACGGAAGATTTGGGTGGTAAATCTGCTCAACTTCCTTGATTAAATTGC
CGTTTTTCTTCTCATGTAGCTCGGCTTTCATTGCTTCTTTGACAAGTGTAGGAGCCTCCAAGCAAGGAGTGACA
TCTCCTCGAACGCCTCCTAAGTGGTATTTAAGCCG
>TCONS_00045024
AAATTGCTCAAATATTTAAAAGGGGATGTGGATAGACAATTATCAGCTGAAGATAGGTATCTTGGAGCTTTTC
TTGTACAAATAACTGCAGCGTTTTGGCACTCCCTTGTCAATTCGGCAGCGTGCAAGGCATTCTGGATAGCAGC
TATTGTGGCCTCACAAATTCAATGAGGTTACGGGATGATTAGACATGAAAACCAAGAGATATTCCAAAACAT
GTCTGATCAATGGATTCACTTATTCTTTAATCTGCTGATCCCTTTGAGTCCAATAGCGGAAGAACACGAACTG
AATCAGTATAAATGTGTCAAGAAGTACACATCCTGCTGCATCCACCAGCCATGGTAGGTTTGGTCCAATTTTT
GACCAATCTAAGCTGCTAGCAAGTATGCTTGCTACATATGTAGTATTCCCTAGCAGAGCAAATATGAACATTA
GTGGATTAAGACCCTCAACATTTCCCCTTCTAATATTTAGGCAAATCTGGGGAAGGCGTCCACCCATGTAAAT
AGCTGCCATTCCCCAGCCAAGGAATGATCCAACTCCGCCGCTATTAGTTGTGGTATTCACCATCAAGGTTATA
TTCACCTGTTGAAGCTTTC
>TCONS_00045050
AACCGTCTCAACGCATTACAATCACTCTGTCAAATCTAGAAACTTTTGACCATTAAAATCAGCAACAGACCTT
CAGTGGTGGACTAAAGATGCCAGGGAAAGGTGGATGTCACATGATGGGATAATGTAAAAGTGTGCCTTCTC

CTTTTCAAACTGCTGCCTTCAAGGTCGGCAATGTGACAAGTCTGAAAATGAAGGCTGGGCAAAATTCATGAG
GAATGGAGGGCAATAGTGACTGGAGGATAATATGTGATTGTGACGCCACGATGGATGGGAGAACTCTTCTTG
AAACAAGAAGAGAAACTTGTCACGGTCAGGAAACGAAATAGACACCGTCCATCAGCAATGATTGTATGGAC
ATTCAATGTTATCCAGATTTCATATCCACCCTATCAACCTTGCCATATCTTTTGAAAAGCCTTTCCAACTCAGA
CTGTCTGGTGTCAAACTCAAAGTTACCACAGAAAATTGGTCTCATCCTGCCTGTACAGAAAAAGTCAGGACT
GCTAAACCTGAGATGAGAAATGTAGATGTGCAGGCGTTCTGTAGTATAGAAAACCCTAACTGCCACAACAAG
C
>TCONS_00045150
CACGGGAATACAGTTCAAGTTCAGCAGTCAGGCGCCCACCTGGAGAGCACGGGAATACAGTTCAAGTTCAG
CAATCAGGCGCCCACCTGGAGAGCACGGGAATACAATTCAAGTTCAGCAATCAGGCGCCCACCTGGAGAGC
ACGGGAATACAATTCAAGTTCAGCAGTCAGGCGCCCACCTGGAGAGCACGGGAATACAATT
>TCONS_00045233
TCACAACTAAACAGTCTCATAGCATGACAAAAGTAGTTCATTTGTTCTGCAAAAGGATGGTTAATTCCTAGAG
AAAAGCTAAAACTGCCTCTTCCTACTACTACATACTTCAAACATGGCTGAAAAGCAGAACAAACATATACTA
GGAAATGGTTCATTTTTTCTTCTTGGCGGGTGGTTGATCCTCCTCGTCCTCGTCATCTTCCTCGTCTTCCTCTTCC
TCATCATCATCCCCGTCCTCATCGTCTTCATCATCTTCGTCGTCTTCATCATCGCTGCCTTCGTTGCCATTAGCC
GCTGGAGTCTCTTCGGGATCCCCTTCTTCATCATCATCTTCCCCTTCTTCGCCTGAAAAGTCTTCATCGTCAGCA
TCATCGTCGTCCTGATCATCAGCGTCTCCATCATCATCCTCATCGTCCTCTGTGTCACTGCCATCTTTGTTCTCA
ACTCCAGCTTTTTCCATTTTATGGAGCTCTTCGAATGGAAACCTTTTCTCAGCGCCTATCAACTCTGGCAATGC
ATTAGCATACTTGAGCATAGTTCCTGCCACAATGAGCAAAGAAGCAACAGATCTTTGAGCAGCCAAAAGGGT
CTCAGCAAATACAGCTTCAACAACAGACTCCATCATCATTCTCCCAAAAACAGCTACTTCAGAGCTCAAATT
ACTGACCTCCATTTT
>TCONS_00045239
TTTGTAGATTTAGCAATCTGACCGTTGATTTCTTTTATCCCGAGTGTTCCCAACCGGGATGTTGCAGGGACGTC
TATCTGTCAAATCTTGCTCTTGAGTTCTGAATTTAGGTTTTAACTTCTTAGGAGTTCCTTTGGCCCTCTAGAATA
TCGTAACTAACTTATATGAGTTTAGTCTTAAGTCTGTATGACTAAGTCACATGAGGAGGGAGGTGGATTGTTC
TTTTT
>TCONS_00045389
AAGCCCTTTATTGTGGGAGGTCATTGTAATTCTGGAAGCTAGAGGATAGCTTTCTGTACTGGAACAAGTTAGT
TCAAAGTAGTACTATTTAACTTACACCTACATAAATATGTAGCTTTATTTAAAAGAAATTTGACATGTATCAC
ATTCCAGCTAAGTTTTGTGTTGCCTTCTTTAGTTCATCAGATTCTGCTTTTGGAATGGATGTGATCAGGAGAAT
GCTGCAGACTGGGCATCCAACTATGCTCAGCTGATTGGAAATCTCCTGAGGGGAGGCCAATCTTCTCTGTATC
CCCTTCATTAAGAGGTAAAGTGGTTGTCAATCCAACGTCTGGCAATGTGAATATTTCGGGCCAAAGTCGAGTA
TGTGTGTGATCTAGTGGCTAAAGAATCGCACAACTGAAAGAAATTGAAGCTACATGTACATATTAGCATAAT
CTACTAATGTTTTATCTTGTTTTTGTTTTCATGTTTCTTATCATAGTAAACAAAGCATATAGCTGAATATTATTA
CAAGATCAATCTAATTAAATTTGTTTACTCTGCAGCAGTAGTCTCTCTGTGTATGTCTGCCTT
>TCONS_00045438
ATATTCTTCATCACCAATAATGTATGATGTTTCAATATTATCATTAGGCATTTGCTGCTCAGCATCTTTACACA
ATTTTACCATATCACATGATGTATCCATCATTAGTAATTTCTCAATGCCGCCACGTCCGCGCAATAACCGTCTG
ATAGCTTCCAGAGAACCCCCCATACACAATGTCGTTGGAAATGTTCTCTTACA
>TCONS_00045764

AAGACATTGTTAGCCAAAGGTGTGAATGGAATCGAAAGAAAACGGATAGAGAGGTTGGTTTTGTTTATGTAT
TTAGTGGAAGAATCCCATTGGTTAAAACCCTCTGATGAGGGAGGCAAATTTCTGAGGAAGAAGATGATTGGC
TGAAGCAGGTCTCCTCATCAGATCAGATCAGATCGAGAAAGAAAAAAGGAAGCCAATTCCATCCTTCCCCCC
TTTGATGTTATCCACAATTCCACCCAACTTCCCAGCAGTCAGATTACTGCATTTGCTTCCTACGTACACTACAG
TGTCATACTCGTGAAGACACAGAAGGTGTCGGGTGCAACTCCAAATAGTTAATTGAACTTATTATTAATTG
>TCONS_00045887
CTGACGTACTCTACTAAACTCTAGCAACAGGCATCGCTACTTGACCCATTTACTTCCTACTTGATCCCAGCTCA
AGGAGCACAACGCTAATATGATGGTGCCATTTTCTTCTCTTTGTTAGAAAGCAAATGGAAGGCACACCGAAT
CGGGAGACAAGTCTAAGGAGGCACAATTGACACTTTTGAACCAAAAACCTTTTTGCACTCACAAAACACTCA
TAATTTTTAAAAACTACAACTGTCCAAACACATCTTTTTGCAAAAACTTTTTTCTCCAAAACAAGGATTTTCTC
CAAAATTGTCCGCACGGCCTAATATGCTCTCTGCAAGCTGAATGAGGGAGCTGAATTGCCTCTTGGAACCCTT
CCAAATCTTACACAATTCCGGGTTGTAAATCTAATCTGATAAATGAGGATCAATGAGGAGCCTCTAAATGCC
GCTGTTCTGAACTTCCAGTCCCTTCCCTATATGAGACACTAGGGTGGTATATATTACACAAAACAAGTAACAA
TAATGAATATCTTAGACCACAACCATGTTATTTGTTGATCGGGTTGGATCAGAATGTATTGTAAAATGTGGAT
AATTTGGAAACTAGTTAAGGAAGCTCCGAATCTGATGCATGACCAGATGATCTCCTTGTTCTTGGGCTGTTCA
TAATCATTAGCTGACTTTCGGAATTCTGCCTTATAGTGCGTATTTGTGGAGGTCTTACCGACCGTCCCCTTGGT
GTTCCTGGAACAATGATCTCATGATGATGTTTGAGACAATGAAAAACAATATGGAAATTGCATAATTGGAAT
CGTTCTCCTTTTCTTTGTAGCTTGCACTAATAGTTGAGGATAAGGGAAGTGAAGATGCCGGTAGACTAATCTA
CAATCATTGCACAAGGTATCGCATGCATCAATTTGTAGGTTCTCTTGGAATCATTCTTGTTCTGGCAATACTGT
GTTACTGAAGCTCATAGATACATAACCTAACTAGATGCTGTGGCTGCTCATATTTCATGGTATTGCTAAGAAC
CCTAACATTATCTATGTAAAGGATCAAAGTTGATGTGATTTTAACTTTAAGATAAGAACTTCTTAACATGATT
ACTTTACATCTACCAATTAAGTGTTCCATAGTCCTCATCCGGTTATAACAAATGAGAACTTATTTTGTGGATTT
TTG
>TCONS_00045889
TTTACTTCCTACTTGATCCCAGGTTCGACTCTTGAGATTGCTGCTTGACTTTGTTGTTTCATCTGGACTATTTTTC
GTGCATTTTCGTTTAAAGCTCAAGGAGCACAACGCTAATATGATGGTGCCATTTTCTTCTCTTTGTTAGAAAGC
AAATGGAAGGCACACCGAATCGGGAGACAAGTCTAAGGAGGCACAATTGACACTTTTGAACCAAAAACCTT
TTTGCACTCACAAAACACTCATAATTTTTAAAAACTACAACTGTCCAAACACATCTTTTTGCAAAAACTTTTTT
CTCCAAAACAAGGATTTTCTCCAAAATTGTCCGCACGGCCTAATATGCTCTCTGCAAGCTGAATGAGGGAGCT
GAATTGCCTCTTGGAACCCTTCCAAATCTTACACAATTCCGGGTTGTAAATCTAATCTGATAAATGAGGATCA
ATGAGGAGCCTCTAAATGCCGCTGTTCTGAACTTCCAGTCCCTTCCCTATATGAGACACTAGGGTGGTATATA
TTACACAAAACAAGTAACAATAATGAATATCTTAGACCACAACCATGTTATTTGTTGATCGGGTTGGATCAGA
ATGTATTGTAAAATGTGGATAATTTGGAAACTAGTTAAGGAAGCTCCGAATCTGATGCATGACCAGATGATCT
CCTTGTTCTTGGGCTGTTCATAATCATTAGCTGACTTTCGGAATTCTGCCTTATAGTGCGTATTTGTGGAGGTCT
TACCGACCGTCCCCTTGGTGTTCCTGGAACAATGATCTCATGATGATGTTTGAGACAATGAAAAACAATATGG
AAATTGCATAATTGGAATCGTTCTCCTTTTCTTTGTAGCTTGCACTAATAGTTGAGGATAAGGGAAGTGAAGA
TGCCGGTAGACTAATCTACAATCATTGCACAAGGTATCGCATGCATCAATTTGTAGGTTCTCTTGGAATCATTC
TTGTTCTGGCAATACTGTGTTACTGAAGCTCATAGATACATAACCTAACTAGATGCTGTGGCTGCTCATATTTC
ATGGTATTGCTAAGAACCCTAACATTATCTATGTAAAGGATCAAAGTTGATGTGATTTTAACTTTAAGATAAG
AACTTCTTAACATGATTACTTTACATCTACCAATTAAGTGTTCCATAGTCCTCATCCGGTTATAACAAATGAGA
ACTTATTTTGTGGATTTTTG
>TCONS_00045907

AATCAAAACACCAGAAATTTACAAACATCCTTAAATCACACACAACATGAAAAGATCCAATTTTACACAAC
CATGAAACGCAAAGCTATGCTTTCTACAAGCAACAAAGATAATTTAAACCTTAGATGACATGAAATTCTCCG
CTTTCATGTACCTGCTTCACTTGCTTTTCATATTCATCTTCTTCGTCGTCCTCTTCTTCTATATCTTCTTCGTTCAA
TTCTTCTTCCTCCTCTTCACCTACCCAGCCAAAACCTCCAGTTGAACGGAAGCTGGAGCAGGGGTCTTTGCCT
CTTCCACAATTTTCATTATCATCTCCGGGCTCTGAAGGGAAAAGGTTGGATTTTCTTTTCTGTAATAAGCAAGC
TGTGCTGCTTCTGTTAATTCCCTAGGCAAATTCTTCAAAAACCACGGGTGGGTTTTTATTTCTTTGATTGTAATT
CTTCTTGCTGGATTGGCAACAAATATGCGAGAGAGAAGGTGCCTACAGTCCTGTGATATGTGAACATAGTCG
GGAATCTTATACTGCACCGACATTATTCGTTGGATAGTTTTCCGGAAGTTCTTTGGATCCTCCTGGTCTTCAAA
AGGGTAAGCACCAACCAGCATTACATAAAGTGTCACTCCACATGACCAAACATCGGCCAGCTTGCCGTCATA
TTCTCTTCTAGATAGAACTTCGGGAGCAATATAAGCGGGAGTTCCAACAGTTGATTTTGGCCTCGAATGTAGC
AGAGACGACTTTGAGTATCCAAAATCACAAATCTTCAAGCGTGGTGCTGGACTGCCATCCAGTAGAGTATTCT
CCAGTTTTAAATCTCGATGGCATATTTGCATGTTGTGACAGTAGCAGACTCCTGAAATAAGCTGCTGGAAGAA
ATATCTAGCCTCATCTTCGCTGAACCTTCCGGCATTGCAAATGCGCTCAAACAGCTCTCCACCAGCTGCATAT
TCCATTACAATAGCAAGATGAGTGGGGGTTAACACCAACTCCTTGAATCGAATTATGTTTGGATGTCGAAGCG
ATTTATGATTTATGATCTCCCTTGCTACATTCTCATCAATCTTGTGTCCCCTCTCAATATATTTCATAGCCACCA
GCTCTTTGGTCTGCTTGTGCCTCATGAGCCTCGCCACTCCAAAATTCCCAGACCCTATTTCTTTCACCAGCTCG
TAATTCTCCATCTTTTAATCCTTTAGAAAAAACACTTCTCAATATAAATAAAAACCAGGAAACCAAAACTAC
ACAACTCCCAAATTGAGTTTCAATTCATGCGTTTCAGTTCAATTATAAATTGGGAAAGTGGGTCCTAAATTTA
ACGGTCAAAGGAACAATCTTTGTGGAGGAATTAAGAAGATTCTCACAAAATCCC
>TCONS_00046065
TTTTTACAACATGGTAGCCTCTAATGGCTCACAGATGATGCATGTTCACTAGGACTTACTATACAAAACAAGC
TGCCAAAAATAATAACCAAAAACATGAAGAGCTTCCTACTTTTTTCTAGTCTTTTTTCTCTTATTACTCTTAGG
AGAAGATGTGGAGGAAGATGTTTTGAAGCCTGCCCAGTGATTCTCAGCTTTGTCTTTGTTGAATGCCTTCTGTA
AATCATCCTTAAGTTGTTCATAATGCATCTTCTTCGATCTTTGTTCCTCAGGTGTGCCACTTTCCAGCGGCAAA
GGATTTAGCATTTTGTCCAGAATGTCTGAGCAACTAGGGAAAAAACGTCGACCTGTATCCACTGTTTTCTGCA
GGGCTTCCAACCGCTCTTGTAGTGCTTTTACCTTTTCAGGTGGAAATTCATTCAAAACAACCCCTGTCAGATCT
CCACATAAGTTATTTATTTCTGAAAGGCCAGTAAACTCCGAGGTCGAATCAACATGTGCTATATCCATGGCTA
GCTTGGCTTCTATGGGAAACATCATGCGTGCCATTGCCAC
>TCONS_00046066
GGAGTGCTGAAATGGTTCTAGCTAAAACTAATCCTGACTAACATGCTGAAGATGTGAACATAGAATTTTTATC
AGAATTAACTAAGGTACCTCGATTTTCTAAGAGCAGCAACATCGCGAGTAATTCATCAGCCACCACGGATGA
TGATGTCGATATGTTTCCAATCATTGGATTCCTATTCATCTCTCTCTCCAAAACATCAATGCATAATCGGTCTTT
ATTAGTTATCTCTCCCTCTTTTGGTGTCTCATTATAATCCTTTAGCCGAGTCACCCTCAAACAAATGGATAGTG
CTGTAAGTCCATCCAGTGTCGTATCTAAGGCAGATGCTCCTTTGTCAAGTAAACTCATTATTATTGATGGTTCC
ATACGTCATATTGTAAGTCTAGCACCAATATGAAGGACTGTATATCCTCGGGAGTTACGAAGATTGACATCA
GCTATGCCCAGCTTGAGTACCTCATTTACAACCTTAGAGTTACAATAGGCAGCTGCATAATGAAGAGCACAA
GCATCATTTAAAGTGACATTAGACTCTCCCAGGAGTAACTTCAGCAATTCAATGTCATCGGACTCTAGAGCCT
TGAGAATCTTCCTAATTCTCTTCTCGTGTAGGGAACCACCTTCCATTGTGTCTTGTTCAATCACTATTTGGGACT
TAAGGCGGCGTGCCTTTATATCGGTCAAACTTTGGAAGGAAGTTCTTTCTCAAGTGTAAAAATATCCAAGTCA
GATCGTTCCACCTTCTGCACACACAGCGCTCTAAAAGTAGGTCCAGTGAGCAGTGGAAGGCCACTAAAAGTA
TAGGAATTACATCATCCACTGTAGCATCATCAACCAGATTGATAAGATGCCGCTGAACAGCCATGACAAGTT
CTTGAATTTGGAAAGTGGCAAAAGCATACATGAGTTCTACAGCATAATTAATGGCAGGGCGGCAAGCATCAT

GAGTACAAGTGATATCAACGCAAGTTGAAACTTCCGGAGGAGGAGGCTTAAGCTTTCCAGTAAACAAATAAT
TTAAGAAGACCATAAATGCTTCATAACCAATTGAGCCACCAGGCAACAAATCCTTCAGTAAATACTTAGGTT
TTCCTTCTTTCACAGAATTCTCATTGTCCTTAAATTTTTTATGGAAAAACTGACTTCGTGCAGCAAGAATACAG
CGATTAACAACGACAGTGTTTCCGTCAACAACTATCTCTGCATCACTATAGTCATATTCAGCATCAAGCACGA
GTTTTTCGAGACTACCACTTAGCTTACTCAAGCTCAGCAGATCAAGACATGTCCCTGGTTCGTCGTGGCTATA
GTCCCCGTCTGATTCATAAGAGGATGAAGCAAAACTCCAAGATGATGAAAGTTCACTGCCATTGTCCAT
>TCONS_00046078
TAGATCCTATTATCACTTTAAAACGTCTTGATTTCTTTCCTTCAAGAATGATTGGAGACTGATTCAGCGCCTTT
GAGGAATTCTGCACCTTAAGAAATACTCTCTTGTCCATTCCGTCCTCATTGTCTACGAGAGAGAAGACTTCAT
TCAGTAACAAACAACTCCCTCTTTGATCTGTACCCCAATCATATTGAAAAGCTGCAGCTTTGCAAGAACAGTT
ACTCAGACAGGCCCTTTTGCAATCTTCCAACTTTTTCCCCTCAGGCCAAAATATGCTCGAACTTAGTTCATGAC
TGAACGGAAATGCAAAATATGCGGTATTCTTGAGCTCTACGAGACTATGATACTGTGGAGAATCACAAGA
>TCONS_00046153
AGGACTCAAAGTTGGAGAAAATATTGTGCCTCCTGGTTTTGATCAAATATTTGAAAGTGATACTGATCTTTTTC
CTCATGAAGAAGAAGAGATGCAAGGGCTTTGTGAATCTGAAAAGGTAAAAAAGGTGAGGGGAATGAATAG
GTATAAAAAAGGTGCAGGACTCAGAGTTGGAGAAAATGTTGCGCCTCCTGGTTTTGATCAAATATTTGAAGG
TGATACT
>TCONS_00046183
GGCACTTTTCATTCTTCATTCGAAACTAACAAGTGCCGGGTCAGGGATTTCTGCCTTGTTATCAAAAAGGGAG
TTGGGTAAAGCAAACTCTCTCCTTGGAGGAGCACGGGCTTAGTCAAGTAGAACCGGGTTGCGCTGCTTGATG
CTCCGATCGAAAACAAATTAGTGGGGCCATGCCGGTACGACTGAATATGCGTTAGAAAGGTCAATCCCTCCC
CTACGACACAAAAAAAACCGGGCCGAACTTCTAAACCGCCCGCTTCCATAGAACAATATCGTGGAAACGTA
GACCTAAGTGGTAATATTGGATCCTTGGGAACCATCACAAGTACGGCCGACCTATATATATTTGAACGAAAA
TGCCGTGTCGTCCAGCGGAGCCTAGAAGAAGGTGACTCGCGCAGACAGCTGACTCCTTTTCAATAGAAAAGA
ATAGCCAAACCAACCCAGCTGGCTGGTCAATCTCAGAGATCTTATCGGCCGGCAAACCGGAGACGGACGAC
CACGGTCCCGGCCTTACCAGCACCGGAGGTGTACTAATCAATGAACCCACGAAACCAACTTTCTTTTATGAC
AAAATAAACCAAGCCTAAGCGGTCACGACATCTTGTTGATATTGATTGAGGAGTTTGATGGAGTTTAGCTGAC
TGAATCCATCCATAAACTTAGACCGCGGTAACCTTCGTAACCAAAGCGTCACGACAATATCCAAAGGTGACC
TTTGGATTTATAAGTAGGGGGCAAGGAGGAGCACGTAGGAATGCCGACCACTACATAAGCCACTAGTGGCTG
AGAGAAAGCGAACAGC
>TCONS_00046184
GGCACTTTTCATTCTTCATTCGAAACTAACAAGTGCCGGGTCAGGGATTTCTGCCTTGTTATCAAAAAGGGAG
TTGGGTAAAGCAAACTCTCTCCTTGGAGGAGCACGGGCTTAGTCAAGTAGAACCGGGTTGCGCTGCTTGATG
CTCCGATCGAAAACAAATTAGTGGGGCCATGCCGGTACGACTGAATATGCGTTAGAAAGGTCAATCCCTCCC
CTACGACACAAAAAAAACCGGGCCGAACTTCTAAACCGCCCGCTTCCATAGAACAATATCGTGGAAACGTA
GACCTAAGTGGTAATATTGGATCCTTGGGAACCATCACAAGTACGGCCGACCTATATATATTTGAACGAAAA
TGCCGTGTCGTCCAGCGGAGCCTAGAAGAAGGTGACTCGCGCAGACAGCTGACTCCTTTTCAATAGAAAAGA
ATAGCCAAACCAACCCAGCTGGCTGGTCAATCTCAGAGATCTTATCGGCCGGCAAACCGGAGACGGACGAC
CACGGTCCCGGCCTTACCAGCACCGGAGGTGTACTAATCAATGAACCCACGAAACCAACTTTCTTTTATGAC
AAAATAAACCAAGCCTAAGCGGTCACGACATCTTGTTGATATTGATTGAGGAGTTTGATGGAGTTTAGCTGAC
TGAATCCATCCATAAACTTAGACCGCGGTAACCTTCGTAACCAAAGCGTCACGACAACATCCAAAGGTGACC
TTTGGATTCTTAAGTAGGGGGGCAAGGAGGAGCACGTAGGAATGCCGACCACTACATAAGCCACTAGTGGCT

GAGAGAAAGCGAGCAGCACCTTGTATAGGTTGGGCGGAGCTTAAAGAAGCAAGCCCCTATCAGAGAAAGCC
ATTGCGCGCTAGCCTAGCTAGCTAACGTTTTTCAGCTGGCTGTTATGTTAGTTGTAGCGCGCCTTCCCTCCTTCT
CTCACTTTGGAAAGATTTTTCAGTATTTTCTTATGATGACCTGGTCGAGAGAGTACGATACATCGGTGTAAAA
GATTTCCTTTTTTCTGTGAGCTTGGTTTACTTGACCGAAGGGTGCAGTAGAACCTGAAGGAGAGGAATAAATA
CCTGGTCTATAAT
>TCONS_00046185
GGCACTTTTCATTCTTCATTCGAAACTAACAAGTGCCGGGTCAGGGATTTCTGCCTTGTTATCAAAAAGGGAG
TTGGGTAAAGCAAACTCTCTCCTTGGAGGAGCACGGGCTTAGTCAAGTAGAACCGGGTTGCGCTGCTTGATG
CTCCGATCGAAAACAAATTAGTGGGGCCATGCCGGTACGACTGAATATGCGTTAGAAAGGTCAATCCCTCCC
CTACGACACAAAAAAAACCGGGCCGAACTTCTAAACCGCCCGCTTCCATAGAACAATATCGTGGAAACGTA
GACCTAAGTGGTAATATTGGATCCTTGGGAACCATCACAAGTACGGCCGACCTATATATATTTGAACGAAAA
TGCCGTGTCGTCCAGCGGAGCCTAGAAGAAGGTGACTCGCGCAGACAGCTGACTCCTTTTCAATAGAAAAGA
ATAGCCAAACCAACCCAGCTGGCTGGTCAATCTCAGAGATCTTATCGGCCGGCAAACCGGAGACGGACGAC
CACGGTCCCGGCCTTACCAGCACCGGAGGTGTACTAATCAATGAACCCACGAAACCAACTTTCTTTTATGAC
AAAATAAACCAAGCCTAAGCGGTCACGACATCTTGTTGATATTGATTGAGGAGTTTGATGGAGTTTAGCTGAC
TGAATCCATCCATAAACTTAGACCGCGGTAACCTTCGTAACCAAAGCGTCACGACAACATCCAAAGGTGACC
TTTGGATTCTTAAGTAGGGGGGCAAGGAGGAGCACGTAGGAATGCCGACCACTACATAAGCCACTAGTGGCT
GAGAGAAAGCGAGCAGCACCTTGTATAGGTTGGGCGGAGCTTAAAGAAGCAAGCCCCTATCAGAGAAAGCC
ATTGCGCGCTAGCCTAGCTAGCTAACGTTTTTCAGCTGGCTGTTATGTTAGTTGTAGCGCGCCTTCCCTCCTTCT
CTCACTTTGGAAAGATTTTTCAGTATTTTCTTATGATGACCTGGTCGAGAGAGTACGATACATCGGTGTAAAA
GATTTCCTTTTTTCTGTGAGCTTGGTTTACTTGACCGAAGGGTGCAGTAGAACCTGAAGGAGAGGAATAAATA
CCTGGTCTATAATTTTGACAAAAGGAGGACCGCTATTGGGAAATATCTTCTTCCTCTTGGTCGTCATCAAAGA
AGTTGACGTCCGGGGCTTCCTCTTTCCCTTTAAAATAAAACCGGTGAAGTCCATCCTAAAGTTCAAAATCTAC
ATCCAACACCAGAGGTTCCTGATGAGAACAAATTGTAAGGCGTTGAAAGAAACCCTTAAGAAAGATGTCCCT
CGCTGCAAAACATATTTCGCTAGATGGCAAGCCCCTCTTTGACTTTGATATAGAACACATAAAGGGGGAGA
GTCACAGTCTCCCTTATTT
>TCONS_00046186
CGGGTCAGGGATTTCTGCCTTGTTATCAAAAAGGGAGTTGGGTAAAGCAAACTCTCTCCTTGGAGGAGCACG
GGCTTAGTCAAGTAGAACCGGGTTGCGCTGCTTGATGCTCCGATCGAAAACAAATTAGTGGGGCCATGCCGG
TACGACTGAATATGCGTTAGAAAGGTCAATCCCTCCCCTACGACACAAAAAAAACCGGGCCGAACTTCTAAA
CCGCCCGCTTCCATAGAACAATATCGTGGAAACGTAGACCTAAGTGGTAATATTGGATCCTTGGGAACCATC
ACAAGTACGGCCGACCTATATATATTTGAACGAAAATGCCGTGTCGTCCAGCGGAGCCTAGAAGAAGGTGAC
TCGCGCAGACAGCTGACTCCTTTTCAATAGAAAAGAATAGCCAAACCAACCCAGCTGGCTGGTCAATCTCAG
AGATCTTATCGGCCGGCAAACCGGAGACGGACGACCACGGTCCCGGCCTTACCAGCACCGGAGGTGTACTA
ATCAATGAACCCACGAAACCAACTTTCTTTTATGACAAAATAAACCAAGCCTAAGCGGTCACGACATCTTGT
TGATATTGATTGAGGAGTTTGATGGAGTTTAGCTGACTGAATCCATCCATAAACTTAGACCGCGGTAACCTTC
GTAACCAAAGCGTCACGACAACATCCAAAGGTGACCTTTGGATTTATAAGTAGGGGGCAAGGAGGAGCACG
TAGGAATGCCGACCACTACATAAGCCACTAGTGGCTGAGAGAAAGCGAACAGC
>TCONS_00046187
CGGGTCAGGGATTTCTGCCTTGTTATCAAAAAGGGAGTTGGGTAAAGCAAACTCTCTCCTTGGAGGAGCACG
GGCTTAGTCAAGTAGAACCGGGTTGCGCTGCTTGATGCTCCGATCGAAAACAAATTAGTGGGGCCATGCCGG
TACGACTGAATATGCGTTAGAAAGGTCAATCCCTCCCCTACGACACAAAAAAAACCGGGCCGAACTTCTAAA

CCGCCCGCTTCCATAGAACAATATCGTGGAAACGTAGACCTAAGTGGTAATATTGGATCCTTGGGAACCATC
ACAAGTACGGCCGACCTATATATATTTGAACGAAAATGCCGTGTCGTCCAGCGGAGCCTAGAAGAAGGTGAC
TCGCGCAGACAGCTGACTCCTTTTCAATAGAAAAGAATAGCCAAACCAACCCAGCTGGCTGGTCAATCTCAG
AGATCTTATCGGCCGGCAAACCGGAGACGGACGACCACGGTCCCGGCCTTACCAGCACCGGAGGTGTACTA
ATCAATGAACCCACGAAACCAACTTTCTTTTATGACAAAATAAACCAAGCCTAAGCGGTCACGACATCTTGT
TGATATTGATTGAGGAGTTTGATGGAGTTTAGCTGACTGAATCCATCCATAAACTTAGACCGCGGTAACCTTC
GTAACCAAAGCGTCACGACAACATCCAAAGGTGACCTTTGGATTCTTAAGTAGGGGGGCAAGGAGGAGCAC
GTAGGAATGCCGACCACTACATAAGCCACTAGTGGCTGAGAGAAAGCGAGCAGCACCTTGTATAGGTTGGG
CGGAGCTTAAAGAAGCAAGCCCCTATCAGAGAAAGCCATTGCGCGCTAGCCTAGCTAGCTAACGTTTTTCAG
CTGGCTGTTATGTTAGTTGTAGCGCGCCTTCCCTCCTTCTCTCACTTTGGAAAGATTTTTCAGTATTTTCTTATGA
TGACCTGGTCGAGAGAGTACGATACATCGGTGTAAAAGATTTCCTTTTTTCTGTGAGCTTGGTTTACTTGACCG
AAGGGTGCAGTAGAACCTGAAGGAGAGGAATAAATACCTGGTCTATAAT
>TCONS_00046188
GGGATTTCTGCCTTGTTATCAAAAAGGGAGTTGGGTAAAGCAAACTCTCTCCTTGGAGGAGCACGGGCTTAGT
CAAGTAGAACCGGGTTGCGCTGCTTGATGCTCCGATCGAAAACAAATTAGTGGGGCCATGCCGGTACGACTG
AATATGCGTTAGAAAGGTCAATCCCTCCCCTACGACACCAAAAAAAACCGGGCCGAACTTCTAAACCGCCC
GCTTCCATAGAACAATATCGTGGCAACGTAGACCTAAGTGGTCATATTGGATCCTTGGGAACCATCACAAGT
ACGGCCGGCCTATATATATTTGAACGAAAATGCCGTGTCGTCCAGCGGAGCCTAGAAGAAGGTGACTCGCGC
AGACAGCTGACTCCTTTTCAATAGAAAAGAATAGCCAAACCAACCCAGCTGGCTGGTCAATCTCAGAGATCT
TATCGGCCGGCAAACCGGAGACGGACGACCACGGTCCCGGCCTTTACAGCACCGGAGGTGTACTAATCAAT
GAACCCATGAAACCAACTTTCTTTTATGACAAAATAAACCAAGCCTAAGCGGTCACGACATCTTATTGATATT
GATTGAGGAGTTTGATGGAGTTTAGCTGACTGAATCCATCCATAAACTTAGACCGCGGTAACCTTCGTAACCA
AAGCGTCACGACAATATCCAAAGGTGACCTTTGGATTTATAAGTAGGGGGCAAGGAGGAGCACGTAGGAAT
GCCGACCACTACATAAGCCACTAGTGGCTGAGAGAAAGCGAACAGC
>TCONS_00046192
CATTGATTAGTACACCTCCGGTGCTGGTAAGGCCGGGACCGTGGTCGTCCGTCTCCGGTTTGCCGGCCGATAA
GATCTCTGAGATTGACCAGCCAGCTGGGTTGGTTTGGCTATTCTTTTCTATTGAAAAGGAGTCAGCTGTCTGCG
CGAGTCACCTTCTTCTAGGCTCCGCTGGACGACACGGCATTTTCGTTCAAATATATATAGGTCGGCCGTACTTG
TGATGGTTCCCAAGGATCCAATATTACCACTTAGGTCTACGTTTCCACGATATTGTTCTATGGAAGCGGGCGG
TTTAGAAGTTCGGCCCGGTTTTTTTTGTGTCGTAGGGGAGGGATTGACCTTTCTAACGCATATTCAGTCGTACC
GGCATGGCCCCACTAATTTGTTTTCGATCGGAGCATCAAGCAGCGCAACCCGGTTCTACTTGACTAAGCCCGT
GCTCCTCCAAGGAGAGAGTTTGCTTTACCCAACTCCCTTTTTGATAACAAGGCAGAAATCCCTGACCCGGCAC
TTGTTAGTTTCGAATGAAGAATGAAAAG
>TCONS_00046193
GTTTATTTTGTCATAAAAGAAAGTTGGTTTCATGGGTTCATTGATTAGTACACCTCCGGTGCTGTAAAGGCCGG
GACCGTGGTCGTCCGTCTCCGGTTTGCCGGCCGATAAGATCTCTGAGATTGACCAGCCAGCTGGGTTGGTTTG
GCTATTCTTTTCTATTGAAAAGGAGTCAGCTGTCTGCGCGAGTCACCTTCTTCTAGGCTCCGCTGGACGACACG
GCATTTTCGTTCAAATATATATAGGCCGGCCGTACTTGTGATGGTTCCCAAGGATCCAATATGACCACTTAGG
TCTACGTTTCCACGATATTGTTCTATGGAAGCGGGCGGTTTAGAAGTTCGGCCCGGTTTTTTTTGTGTCGTAGG
GGAGGGATTGACCTTTCTAACGCATATTCAGTCGTACCGGCATGGCCCCACTAATTTGTTTTCGATCGGAGCA
TCAAGCAGCGCAACCCGGTTCTACTTGACTAAGCCCGTGCTCCTCCAAGGAGAGAGTTTGCTTTACCCAACTC
CCTTTTTGATAACAAGGCAGAAATCCCTGACCCGGCACTTGTTAGTTTCGAATGAAGAATGAAAAG

>TCONS_00046194
GTTTATTTTGTCATAAAAGAAAGTTGGTTTCATGGGTTCATTGATTAGTACACCTCCGGTGCTGTAAAGGCCGG
GACCGTGGTCGTCCGTCTCCGGTTTGCCGGCCGATAAGATCTCTGAGATTGACCAGCCAGCTGGGTTGGTTTG
GCTATTCTTTTCTATTGAAAAGGAGTCAGCTGTCTGCGCGAGTCACCTTCTTCTAGGCTCCGCTGGACGACACG
GCATTTTCGTTCAAATATATATAGGTCGGCCGTACTTGTGATGGTTCCCAAGGATCCAATATTACCACTTAGGT
CTACGTTTCCACGATATTGTTCTATGGAAGCGGGCGGTTTAGAAGTTCGGCCCGGTTTTTTTTGTGTCGTAGGG
GAGGGATTGACCTTTCTAACGCATATTCAGTCGTACCGGCATGGCCCCACTAATTTGTTTTCGATCGGAGCAT
CAAGCAGCGCAACCCGGTTCTACTTGACTAAGCCCGTGCTCCTCCAAGGAGAGAGTTTGCTTTACCCAACTCC
CTTTTTGATAACAAGGCAGAAATCCCTGACCCGGCACTTGTTAGTTTCGAATGAAGAATGAAAAG
>TCONS_00046195
GTCGTGACGCTTTGGTTACGAAGGTTACCGCGGTCTAAGTTTATGGATGGATTCAGTCAGCTAAACTCCATCA
AACTCCTCAATCAATATCAACAAGATGTCGTGACCGCTTAGGCTTGGTTTATTTTGTCATAAAAGAAAGTTGG
TTTCGTGGGTTCATTGATTAGTACACCTCCGGTGCTGGTAAGGCCGGGACCGTGGTCGTCCGTCTCCGGTTTGC
CGGCCGATAAGATCTCTGAGATTGACCAGCCAGCTGGGTTGGTTTGGCTATTCTTTTCTATTGAAAAGGAGTC
AGCTGTCTGCGCGAGTCACCTTCTTCTAGGCTCCGCTGGACGACACGGCATTTTCGTTCAAATATATATAGGTC
GGCCGTACTTGTGATGGTTCCCAAGGATCCAATATTACCACTTAGGTCTACGTTTCCACGATATTGTTCTATGG
AAGCGGGCGGTTTAGAAGTTCGGCCCGGTTTTTTTTGTGTCGTAGGGGAGGGATTGACCTTTCTAACGCATATT
CAGTCGTACCGGCATGGCCCCACTAATTTGTTTTCGATCGGAGCATCAAGCAGCGCAACCCGGTTCTACTTGA
CTAAGCCCGTGCTCCTCCAAGGAGAGAGTTTGCTTTACCCAACTCCCTTTTTGATAACAAGGCAGAAATCCCT
GACCCGGCACTTGTTAGTTTCGAATGAAGAATGAAAAG
>TCONS_00046196
CCAAAGGTCACCTTTGGATATTGTCGTGACGCTTTGGTTACGAAGGTTACCGCGGTCTAAGTTTATGGATGGA
TTCAGTCAGCTAAACTCCATCAAACTCCTCAATCAATATCAATAAGATGTCGTGACCGCTTAGGCTTGGTTTA
TTTTGTCATAAAAGAAAGTTGGTTTCATGGGTTCATTGATTAGTACACCTCCGGTGCTGTAAAGGCCGGGACC
GTGGTCGTCCGTCTCCGGTTTGCCGGCCGATAAGATCTCTGAGATTGACCAGCCAGCTGGGTTGGTTTGGCTA
TTCTTTTCTATTGAAAAGGAGTCAGCTGTCTGCGCGAGTCACCTTCTTCTAGGCTCCGCTGGACGACACGGCAT
TTTCGTTCAAATATATATAGGCCGGCCGTACTTGTGATGGTTCCCAAGGATCCAATATGACCACTTAGGTCTA
CGTTGCCACGATATTGTTCTATGGAAGCGGGCGGTTTAGAAGTTCGGCCCGGTTTTTTTTGGTGTCGTAGGGGA
GGGATTGACCTTTCTAACGCATATTCAGTCGTACCGGCATGGCCCCACTAATTTGTTTTCGATCGGAGCATCA
AGCAGCGCAACCCGGTTCTACTTGACTAAGCCCGTGCTCCTCCAAGGAGAGAGTTTGCTTTACCCAACTCCCT
TTTTGATAACAAGGCAGAAATCCCTGACCCGGCACTTGTTAGTTTCGAATGAAGAATGAAAAG
>TCONS_00046197
GTCAACTTCTTTGATGACGACCAAGAGGAGGAAGATATTTCCCAATAGCGGTCCTCCTTTTGTAAAAATTATA
GACCAGGTATTTATTCCTCTCCTTCAGGTTCTACTGCACCCTTCGGTCAAGTAAACCAAGCTCACAGAAAAAA
GGAAATCTTTTACACCGATGTATCGTACTCTCTCGACCAGGTCATCATAAGAAAATACTGAAAAATCTTTCCA
AAGTGAGAGAAGGAGGGAAGGCGCGCTACAACTAACATAACAGCCAGCTGAAAAACGTTAGCTAGCTAGG
CTAGCGCGCAATGGCTTTCTCTGATAGGGGCTTGCTTCTTTAAGCTCCGCCCAACCTATACAAGGTGCTGCTCG
CTTTCTCTCAGCCACTAGTGGCTTATGTAGTGGTCGGCATTCCTACGTGCTCCTCCTTGCCCCCCTACTTAAGA
ATCCAAAGGTCACCTTTGGATGTTGTCGTGACGCTTTGGTTACGAAGGTTACCGCGGTCTAAGTTTATGGATG
GATTCAGTCAGCTAAACTCCATCAAACTCCTCAATCAATATCAACAAGATGTCGTGACCGCTTAGGCTTGGTT
TATTTTGTCATAAAAGAAAGTTGGTTTCGTGGGTTCATTGATTAGTACACCTCCGGTGCTGGTAAGGCCGGGA
CCGTGGTCGTCCGTCTCCGGTTTGCCGGCCGATAAGATCTCTGAGATTGACCAGCCAGCTGGGTTGGTTTGGC

TATTCTTTTCTATTGAAAAGGAGTCAGCTGTCTGCGCGAGTCACCTTCTTCTAGGCTCCGCTGGACGACACGGC
ATTTTCGTTCAAATATATATAGGTCGGCCGTACTTGTGATGGTTCCCAAGGATCCAATATTACCACTTAGGTCT
ACGTTTCCACGATATTGTTCTATGGAAGCGGGCGGTTTAGAAGTTCGGCCCGGTTTTTTTGTGTCGTAGGGGA
GGGATTGACCTTTCTAACGCATATTCAGTCGTACCGGCATGGCCCCACTAATTTGTTTCGATCGGAGCATCA
AGCAGCGCAACCCGGTTCTACTTGACTAAGCCCGTGCTCCTCCAAGGAGAGAGTTTGCTTTACCCAACTCCCT
TTTTGATAACAAGGCAGAAATCCCTGACCCGGCACTTGTTAGTTTCGAATGAAGAATGAAAAG
>TCONS_00046198
AAGGGAGACTGTGACTCTCCCCCTTTATGTGTTCTATATCAAAGTCAAAGAGGGGCTTGCCATCTAGCGAAAA
TATGTTTTGCAGCGAGGGACATCTTTCTTAAGGGTTTCTTTCAACGCCTTACAATTTGTTCTCATCAGGAACCT
CTGGTGTTGGATGTAGATTTTGAACTTTAGGATGGACTTCACCGGTTTTATTTTAAAGGGAAAGAGGAAGCCC
CGGACGTCAACTTCTTTGATGACGACCAAGAGGAAGAAGATATTTCCCAATAGCGGTCCTCCTTTTGTCAAAA
TTATAGACCAGGTATTTATTCCTCTCCTTCAGGTTCTACTGCACCCTTCGGTCAAGTAAACCAAGCTCACAGAA
AAAAGGAAATCTTTTACACCGATGTATCGTACTCTCTCGACCAGGTCATCATAAGAAAATACTGAAAAATCTT
TCCAAAGTGAGAGAAGGAGGGAAGGCGCGCTACAACTAACATAACAGCCAGCTGAAAAACGTTAGCTAGCT
AGGCTAGCGCGCAATGGCTTTCTCTGATAGGGGCTTGCTTCTTTAAGCTCCGCCCAACCTATACAAGGTGCTG
CTCGCTTTCTCTCAGCCACTAGTGGCTTATGTAGTGGTCGGCATTCCTACGTGCTCCTCCTTGCCCCCCTACTTA
AGAATCCAAAGGTCACCTTTGGATGTTGTCGTGACGCTTTGGTTACGAAGGTTACCGCGGTCTAAGTTTATGG
ATGGATTCAGTCAGCTAAACTCCATCAAACTCCTCAATCAATATCAACAAGATGTCGTGACCGCTTAGGCTTG
GTTTATTTTGTCATAAAAGAAAGTTGGTTTCGTGGGTTCATTGATTAGTACACCTCCGGTGCTGGTAAGGCCGG
GACCGTGGTCGTCCGTCTCCGGTTTGCCGGCCGATAAGATCTCTGAGATTGACCAGCCAGCTGGGTTGGTTTG
GCTATTCTTTTCTATTGAAAAGGAGTCAGCTGTCTGCGCGAGTCACCTTCTTCTAGGCTCCGCTGGACGACACG
GCATTTTCGTTCAAATATATATAGGTCGGCCGTACTTGTGATGGTTCCCAAGGATCCAATATTACCACTTAGGT
CTACGTTTCCACGATATTGTTCTATGGAAGCGGGCGGTTTAGAAGTTCGGCCCGGTTTTTTTGTGTCGTAGGG
GAGGGATTGACCTTTCTAACGCATATTCAGTCGTACCGGCATGGCCCCACTAATTTGTTTCGATCGGAGCAT
CAAGCAGCGCAACCCGGTTCTACTTGACTAAGCCCGTGCTCCTCCAAGGAGAGAGTTTGCTTTACCCAACTCC
CTTTTTGATAACAAGGCAGAAATCCCTGACCCGGCACTTGTTAGTTTCGAATGAAGAATGAAAAG
>TCONS_00046260
ATGGGCGAACGACGGGAATTGAACCCGCGCATGGTGGATTCACAATCCACTGCGAACGAGAGTTGTTGAAA
CTAGCATATTGGAAGATCAATCGGCCAAAATAACCATGAGCGGCTACGATGTTATAAGTTTCTTCCTCTTGAC
CGAATCTGTAACCTTCATTAGCAGATTCATTTTCTGTGGTTTCCCTGATCAAACTAGAAGTTACCAAGGAACC
ATGCATAGCACTGAATAGGGAGCCGCCGAATACACCAGCTACGCCTAACATGTGAAATGGGTGCATAAGGA
TGTTGTGCTCAGCCTGGAATACAATCATGAAATTGAAAGTACCAGAGATTCCTAGAGGCATACCATCAGAAA
AACTTCCTTGACCAATTGGGTAGATCAAGAAAACTGCGGTAGCAGCTGCAACAGGAGCTGAATATGCAACA
GCAATCCAAGGTCGCATACCCAGACGGAAACTAAGCTCCCACTCACGACCCATGTAACAAGCTACGCCAAG
TAAGAAGTGTAGAACAATTAGTTCATAAGGACCACCGTTGTATAACCATTCATCAACGGACGCCGCTTCCCA
GATTGGGTAAAAATGTAAACCTATAGCTGCAGAAGTAGGAATAATGGCACCGGAAATAATATTGTTTCCGTA
AAGTAGAGACCCTGAAACAGGTTCACGAATACCATCAATGTCTACTGGAGGAGCAGCAATGAAGGCAATAA
TAAATACAGAGGTTGCCGTCAATAAGGTAGGA
>TCONS_00046383
GAACAACAATGACCTCCAATATCCAGAACCCACGCCTGATTCATCCACAAGATTCCCTTATACCTACTACTGT
ATCAACAACACAACTTTCCTTGCTAAATTCTACTATTTCTTCTTAATTAGTTTACTATCAGATTGGTTATTTCCA
CCACCAGCAGCCTGTGCCGCTTTCCTTGCTGCTTTTTCTTGAAGTGCCTTCGCGTCTCTTTCTCGTCTTTGTTCAG

GGGTTAATCCATCACCTTTGGGCTGTTTGGGTTTGCTACCTGCCCTGGCTTGTGCCCTTTCACGATCTCTATCTC
TCTGATTTCCGCGTTAGCTTG
>TCONS_00046409
CAGTTGCATTCAACATTTTTGTGAAAATAAGATGATCCCGAGAAATGTTACATTCAATAACTTGAAACTCATT
ATCAGCATATGCAGAAGGATTCTGTCTATGCCCACCAACTGACATCATATGGGATCTTACAAGTCATGATAGC
GAACATACATTTCTAAGAGAAACAGACTATCCTGAGGCTAATCAGTCACGCTCACTGATGGCTGTTAAGTGCT
CATCAATTTCCTCGGTAGTTAACACTCTGAAGACGGGATCTTCTTTCTTCACAACACCAACCTCAATTTCACTG
GCCTTAAAATCCTCTTGCAGGACAGATTGAAGAGCAGAAATTGCAGTCTGAACAGTTTCCTCATAGGAGAAA
GCAGGGTCATTTTTCATTTTCTTCTCCAAAAAAATTAATTGCCTCTTGTTCTTTAGATCCAGCACTTGTAGCCTTA
TGACCAAAAAAATGACCAGCAGGGTCACACTTGAAGAGCTGGGGTCCTTTCTCCTCATCAATGCCCAAAATC
ATAGCAACTACTCCAAGGGGTCTCATATAGGCATGCTGTGTGTAAACCTGAGACTTGTCTGCAATCCATTTAG
ACAGTACATCCACTGGCATTTCATATCCATATTTAAAACGAAACTCAGCAGCTTCATTTCGAGCTTGTTGAAC
CAAGGTTCTCGCATCAGCTGTCATGCCAGTAGCCAATAGTCCAAGATACTTGGTAATCGGGAACAAGTGGGA
TACACTGGTCTGATCCAATAGCTTGTCAGGGACTTTCTTCTGAGTAACAACACAAACGGAGTCCTTTCCACGG
ACGCCAATAGAGGTAATTCCGGCCGCCTTCACAGCCTTAAACGCATATTCGACTTGAAAGAGACGACCTTCG
GGAGAGAAGATCGTAATGTGACGATCGTAGCCGCCTCCACTCCCTCTACTCATTTTTAGATTGTTAGATTTACT
TTTTTGTTTTTCAGTTCGATCTGATTACTTGATTCAGACTGGTTAGGGTTTTTA
>TCONS_00046498
GGGATCTCATAGTGTTTCAGATATTCTGGTAAGGAAATGCACAAAAATTGATGGTGCAACATGTGTAGTTTGT
TAAAACGAAGTGCTTTTGTTTATCGTGAATGTTTGCTAGATTATTCTCATGTAGAGAACATAAGATATCTTCCA
TTCTCTTTGTCAGTTCAAACTACACCTTGAGAGTTTCAGTCAACTATCAAGAGCTGTGGGCTGCTAGGATATAT
CTTAAATAACCTTGTTAAGTTTCTTCTGTTGTTTCAGGGATTTCTTGATACTATAGGGAGAGTTGATTATCAGCT
TTCTCATTGAATTCAACTCTAAGTTGGTTGCAGACATAAATCCTGGTGTGAAAGCTTTAATGCTGTAAGTTGCT
GAAAAAGTTAGAAAATCATTATGCTACAACTTTACAGCACATTTACGGAATACCAGATGGGAAAGAGCTGG
GTCCAAGCTTTGCTAAACTGAGTTTGGTTTTGGAAGCCCAATCAGCTGTCCTATTGGACATGACGGCAGGTAT
GGCGGAGTGTCATCCAACATATAGACTCAGTTTCGAAAGTTCAAATTAGCTTGGATCATCTCTGCAACCCGAA
GCTTGATATTGAAACTTGAAAGTTGAGACCTATCTATTTCTCAAGATCTGTTTGATGATTTAGAAGGTCATAGC
TTATACCGACTTGGAAATCCTGTTGACCTGACTTGTTACTGCTGAGGATTGATATGAAGGGTTGTAAATGGAG
ATCATCTCAATCAACAGAGTTTCAGCCGGAGTTATCTTCCACTGAACTCCAAGATGAATTCTTCGGACTCGTA
CTACAGGTGGCATCAATCATAACTTATGAACACCTAAAAAACACTTTTTAATTGGGGAATCAATTAAATCTCT
GATTGGTTCTACAATGTGTTGTTGCTGCAGGGGAAGAAAAACTTTGGTTTTGTCATGTTTGTGGCCATATGTGG
CATTACGCAACTAATGTCATCCGTTCAGTCTCAATCAGTTGACACCCTAAAGCAAAGAGGGAATTACTTAGAT
TAGCAGTTTTTCAGTTCAGCTGCATCTAGATGGGGGAAAACAATCAAGAAACCTTTCAGGTTAACGGAGATG
CTCGAAATCTGGAAGCTTAGAAACAGGTGGTTAGATCTGTTTTTGTTCGAATGAGTATATCAACGAGGCTGTC
GTGCAGATTGCAGTAGTGAAGCAAATGGGGGCATCAAGATTTGGGATTTGAGAAACACCT
>TCONS_00046537
TTCCTTGGCCGTGTGGAACATGGATTAGCATTATGTCATTCCTACAAGTGATGACCCACCCAGGATTTCTATGT
ACGGATTTAGAGATCAAATATAATATGTTTCTTTCCATTCTTCGTGAGCCACTTATTTCTCCGAAACAAGAGAT
TAAAGTCATCTTCCTCCTTTTTCCCAAGAAGTCGACGGCGTTACACCATTGGGATACTTCGAATAAACTTGAG
TACATATAGGATACACCGGTGCTAAAACCTTTTCTCAAGATATCTTCCAAACTGTTGGGGTCGTTGCTTCGGAT
CTTGTTCTCCCGGTGTGAAACCAGTTGGTTCCGTAGTTTTAGAATTCTGCTGATCCCAAGTACTCCATCTCCAT

CATTGCATATAGGAATATAGAAAGTAAAGAGGAAAAGGCATGACCAGAAGAATTGTGTGAAATAAGTAAAT
TTATCCAG
>TCONS_00046538
ATTTCTATGTACGGATTTAGAGATCAAATATAATATGTTTCTTTCCATTCTTCGTGAGCCACTTATTTCTCCGAA
ACAAGAGATTAAAGTCATCTTCCTCCGATGCTTCCTTGGCCGTGTGGAACATGGATTAGCATTATGTCATTCCT
ACAAGTGATGGCCCACCCAGGATTGCTATGTACGGACTTAGAGATCAAATATAATATGTTTCTTTCCATTCCT
CGTGAGCCACTTATTTCTCCGAAACAAGAGATTAAAGTCATCTTCCTCCTTTTTCCCAAGAAGTCGACGGCGT
TACACCATTGGGATACTTCGAATAAACTTGAGTACATATAGGATACACCGGTGCTAAAACCTTTTCTCAAGAT
ATCTTCCAAACTGTTGGGGTCGTTGCTTCGGATCTTGTTCTCCCGGTGTGAAACCAGTTGGTTCCGTAGTTTTAG
AATTCTGCTGATCCCAAGTACTCCATCTCCATCATTGCATATAGGAATATAGAAAGTAAAGAGGAAAAGGCA
TGACCAGAAGAATTGTGTGAAATAAGTAAATTTATCCAG
>TCONS_00046567
TTTTTATGTGAAATCAAGGAGCAGCAGGTTAGACTTTGACACTTTCCCGAAGAGAGCTCAAAGCAATAAGAT
GAAAGATGGGATGAGATGCTAGCCTTAGCGCAGAAGATAAATCATTGAACCTTAGGCCACTACCGAGCAGC
AATGTAGGATCATAACTGCCTGTACTGCCGAGATGATCCCTACTTGAAAAGGCGGAGACAGATTGTTGCGAC
AAATCGAGTTTCAAAAGCCCATTTATCAATCACATTTTCAGGCACACCAGTCAAAGAGCCATCGGACCCATG
AGAGTCCCATCTTTGACTAATATACACGGG
>TCONS_00046666
CACACCCTTAATGTTGAAGTAATGAGCTTGAAGAAGTGCAGTTTGTGGCATGCTAAAGGTAATGTTGTTGAGA
GAAGCTGTAAACCGAGTTCCATTTAGACAGGTGGGACAAGGATTTATTCCTAGGCCTACTGTAAATAAGAGG
TGTCGATCAACATTTAGGGGAACGTTTGCTGGATATTTAGGGGTATTTAGGCTTTTGAGTTTAGAGTTATAGGT
CAAAGCAAAGTTTGTGTCATTTTGT
>TCONS_00046667
TGCCACACTCACCTAGTACAAGTACTTCTTCCCTATCTGGTTGAGGGAAAGGGAAGGGAGTTCCTTGTTGTGG
CATGATAACTATAGCACCATAGACTGTTGCCCTTAGCCAAAGAATGTGTGCATGCCACCATAAGGTTCCTCTT
TGTCCTGTTATATTGAAGTCATAAACATAGCTATTTCCTGTCTGAATAGGGCATTGTGTTATGTAAGCTGGCCC
GTCTGCCCAACCATTGCGATATTGTTTCAATCCATGCCAGTGAATGGACAAGTTATACTGTGCAAAGTTAGTA
ACATTGATTTGAACTCTATCTCCTTCTCTAGCATATATAGTTG
>TCONS_00046766
GCAGTAGCAAGATGGATCAACAATCTTCTTATGCGCAGGATAAGACTCAACATGGTACCCTGAAAGCGCTTC
CAATCATCTTATAATGTGAAGCTTAAGCACAGCATGCCATTAGAACTCATCTTCAAATGTCTCAACAATTAAC
CTCAAATCTCAAAGGCCAAGCTGGTTGCTGATAAACTACAGTATCCTGTCCACAAAGGTCTTCAATACAGAG
GTACATAGTGGATGAACTAAAATACCTTTTTATTCTTGTAGCCCATACATCCACTCTTCTATTAGTTCAAGGTT
AGGCTTATAACCAACATCCTTCATATTGATGGTTAAATGACCCAACATATCATATATCATGTCGCTCTGAGGA
TGCTTCCTATCTCTAGACACAAAACAGCTTGGATAGCCAGAAGTACCAATCCAACTACATCCAACCTCCTTTG
ACAATCCCATTTTGCGCATTCCTCTTCTTACGTTATCGACAGACTGCCAGTTTCCTTCCTCTGCATATATATTTG
AAAGCAGAACATGATAACCAGATATTCTGTCACTCCCCTCTAATTCAAGCAGCTTACTGGAAACAATTTTCCC
CAATTCAAAATTTCTATGGACTCTGCAGGCAGCAAGGAGTGATCCCCATATTCCCAAGACATTACCCTCTTCG
CCCAACTGTTTAGCAAAATTATGAGCTTCATCCAACCGTCCAACTCTTCCTAACATGTCAACCACACAAGCAT
AGTGCTCTGCTGAGGGCTGAATCCCGTATTCTTCGTCCATCAGCTCAAATATTTGAAGACCTTCATCAACCAA
TCCAGTGTAGCTGCAAGCAGACAAGATTGCTACAAAGGTAACAGCATCTGGTTTTAAACCATTCTCCCGCAA
AGAGTAGAACAGCGCAAGAGCTTTTTACCCATCCCATGCTGGCCATATCCCAAAATCATATTCGTGTATGTC

ACAGAGTTCTTCTCAGGGGATTTTAGAAAAATGCTTTCAGCATAATGAATGCTGCCAGATTTTGAATACATGT
CTACCAAAGCCGAGACAACATAAACATTGTTCTCAAACAAATTGCGAATTGCAAAACAATGTAGCTGATTGC
CTATTGCTATACTTCCTGACTGACTGCATGCTGGGAGAAGTGATGCTAAGGTAACAGCATTCGGTTTCACATT
CTGCTCCAGCATCTCCTTGAAGACCACAAAGGATTGTTCAATTAATCCATTTTGAGTGTTTCCAGCAATCATA
GCATTCCATGTGGCCTGATCTTTATAATTTGTGCAGTTTGACTGAAATATTGCTTGTGCTTCTCTAATCATGCTA
GATTTGGCATACATGTCTATCAGATAACTTTCCATCCCTTCAAATTGAATATTGTGCCTCAGAAGATAAGCAT
GGGTCTGTTTACCTATTTTCCTGTCCCTGAGATTTGATGCCGCAGAAAGCAGAATAGTAATTGTTATAGCATCA
ATTGCAACCCCCAGCTTTTGCATCTCATATACTAGCATCAAAGCCTCATCGTCAAGTCCATTTTGTACCAAAG
CAGACACCATGGTGTTCCACGACACTATATCTCTTTCCTCCATTCCACTAAAAACTTTAAAGGAATCACCAAC
ACGGTTACACCTAGAATATGTGGCAACCATAGCATTTAGCAAAATAACCTGTGAATCCATGCATTTCTTTATC
AGACATGCATGTAGCTGTTGAGCAAACTCCAAATGCTGCAACTGTGAAGTTGCCGTAAGAGCGGATACAAAT
GTCACATCATCAATAGGAACACCATCCTCTGCTTCTACAGCTCCAAGAAAGAGATCAAGTGCTTTAAAAGGA
AAATTGTTCTGAATGTACCCGCTAATCATAGAGTTCCAAATCTCTGTATTTCTTTCAAAACTATTCTCAAATAT
TCTGCTCGCCAAATCAATGCAACCAAGATCAGCATACATAACAATCGCAGCACTAACAACAAACAAGTCATT
TACGTATTGATTACCCAATTTCACAAGCGAACCATAAAGAACATCAGCGACTCTAACATCCCCTATTTCAGAG
ACAGCTGGAAAAATATTAACAAAACTAACAACAGTCGGCTTAATGCCTATTCTCATCATCATAACAAAACAC
CTAACTGCTTCTGAAAACCTCTTCCTTTTAACATACCACGAAAATATGGTATTCCAAGCAACAACATTTCTTTT
ACGCATAGTTCTAAACACTCTTTCAACTAAATCACAATGAGAACCATTTTCGAATCCGAGACAAGTGGAGTA
CATATTCAACAAAGAATTACACACAATCCTACTAGGGTGAATACCCGAGCGAAGAATGTGGCAGTGCACGG
CTTTACCTACAAGAATCTGTTTCGTCTCAGCGCAAGCTTTGAGAACAGAAGAATAAGTATACTGATCACATGT
CGATGAACCCACATGTTTCAATCGGGAATAGAATGAAATGGCTTCATGGGACATGTTGTTACAGATAAAACC
AATGATAATTGTGTTCCATAGGACAGTACTCGGTTGAGGAATTGTGTCGAACAGTTGGCGTGCGAGATGGGG
TTGGCCTTGTCTGCAAAGTTCGCCTAAACGGTAACGAATTGTTCTGGGTTTGGACTCGTTTTTTACAGTGCATT
GTTGTTCGTCGGTGAGAGTGTTGCCGTGGAGTAGAGAACAGAAGTTTGGGTGATGGGTTAAGGTTAAAGAAG
CTGGGGATTGGGAGAAAGTTGAGGACGAGAAAGGAAGAGGAAGAGCTGAGGAGGACATTGTCAAACGCAT
TCGGACCTCAAAACTAACAGAACTCCCAAGCTCAGGAGCTCTTCGAGGATAAACAGGTAGTTATAATTTCAA
CTGCTACTTATCTTTATGTGAAATATCAAACCGCACCTACATTTTAC
>TCONS_00046812
TCATGATACGGCCAGGTATACTTGAGATTAGACGAACCAAACCCGGATTTGTTTGCATGGAACAAACCGGAT
TTGATAATCAACTATACGTCCGTAATTTTTGGTAGTAATCCGATTACAGCTTCGTAATTAATAGTATCCAAAAA
CAATTCTGAATTCTGAAGTAGAAGAGAGAAGCTTAATCATCGTGAAGTAGCTATTTGCTGAAAAACGCTAAT
AGAAATCAATTATCATCCTTTAATCTCTTCACCGTGGTCACACATTCAGTATTTTCAACACCGGAGTATCATTT
GCGTTTTGATTAACATCTGGAGGCGTGTAAAAATGGATTTGCTGACTAGCTGCAAGGTAATTACTTAGTTTTTA
TAGAATGTAATTAGAATTTTTCTTTCTATTACAGTTGACACTTATTTTACTCGTACTAGTTATACTCGGATAAGT
AACAATACAAAAAAGTTATACTTGGATAAGCGTGAGATTTAGAGAATTTCTAGTTTCTAACTTTCCG
>TCONS_00046859
CTTTCTAGTCTTTTCATCAACATCATGAGGTAAACGGAGCCAAGCTGAGTATACTAATGACTCATAAACTGTA
ACATAAGGTGAATGGATATCATTCTGCTCACAGTATCCAGATATACGTGCAAATGTTTCTTGCTTCTTAGGAT
AGCCAGAGATCTTGATACTACCCTCAATATATCCTCCTGTTTTCCTTCCGGCCAATACATCCATCAATGTTGTT
TTTCCAGCCCCACTGACTCCCATTAAAGCTGTCAAAACACCTGGCCTGAAAGCTCCACTTACTCCCTTCAGAA
GTACCAATCTATCTTCAGTGGCGCCCTGATCTTTCATTTCCTGAGGCATGTCGACAGAGTATATAACTTCATCA

AAGGTGATGGAATGCGGTTCAAATGGAAGAACCATTCCCTTTTTCTTACTCTGACCCTCACTATTGGACTGTTC
TTTCTCATTGCTAGTTGTTTTGGCATCTTCA
>TCONS_00047002
CTCATACACATACAAAGTTCTTGACTGCGAAAGGCACCAACCTGGGAGAGTTGTGGAAGGAAATTCGGCTTT
TCCAATGTGCCTGTCCCAATCTCACCAAAGGTAAAAGCAATTATAACAGCAGCCAGCAGATTGGTGAAAGTA
TAATCAAGGTAAGTGTGTTGAGGAAATCTCCCTCTTCTTTCAAGTAAAGTTAGTAAAGCTGGCCATGTCCCTA
AGAAGAACAAAGACAATAACATGCAAACAATAGCCCCTCCTTTGCTCTCCACCACATACATCTTCAAACCAC
TGGATAAAACCCTTTCCGGTGAAAGTGCTATCAAATTGGAAATGCTCCAAAAATCCTACTGTTTGAGACCCCA
CAAAAAAG
>TCONS_00047050
TTCATTCTGATGTTTTAGTAGTTGCAGGTCCAAATGTAAATAAAACAGGCAACATAACCATTAGCCAAATGCG
ATCAAAATACGTCCCACTAAAACTAATTTCACGTTGCAAGTTCTGCCGAGCTAAGAAATTTCAGTTTGAACTT
AACTACACACAAAATGCCCACAGAATTGCGGAATTTATATGTAGCAAACACTGAGGAATCTAAACGATTTCG
CACATACATTAGAACATACAATAATATCTTTGCATTTACATCACTTGGTGTGAGCTACGACAAAGATTTAGCG
AGAAGAAATCATGGTATTTATACTTTTAGAGTTCAAGGACAAATGTATCATTTCATAGATGATCTATTTCC
>TCONS_00047106
AGTCATCACTCATCTTTGCACTACTTCTTTGCACCACTGGAGGACTCAGGCTGTGGTTTTATTATATTTAATTGA
CCACCATCCTTAGTAGCAAATGTAGCTGCTTTTACTTCATTGCAGTGATTTATAAACTTTGTCAACTCCCGATC
GACATCCATCTGCTTTGTATTCTTTGACGGCTTCACAGCTCTTTTCCCTTTGCGAATTTGAGGGGCTTTTCCATG
ACGACTAGGTACTGATTTCTTCTTCTGTTGCCCTTTGAATAGACCTGCCTTTTGTGTCATTTTTCAACCCTCTTTC
>TCONS_00047112
ATAAACCCTAGCATCCCACTAAACCCTAGCATCCGTTCAAACTAATTGAGAAAGAGGGTTGAAAAATGACAC
AAAAGGCAGGTCTATTCAAAGGGCAACAGAAGAAGAAATCAGTACCTAGTCGTCATGGAAAAGCCCCTCAA
ATTCGCAAAGGGAAAAGAGCTGTGAAGCCGTCAAAGAATACAAAGCAGATGGATGTCGATCGGGAGTTGAC
AAAGTTTATAAATCACTGCAATGAAGTAAAAGCAGCTACATTTGCTACTAAGGATGGTGGTCAATTAAATAT
AATAAAACCACAGCCTGAGTCCTCCAGTGGTGCAAAGAAGTAGTGCAAAGATGAGTGATGACT
>TCONS_00047125
CCGAAAAGATATCCGAGCAGGGGCTTCACAGCAAATTGACCAATATAACCAGCAAAAATTGCAGCTGGTTTC
TTGAAAGCTTCAAGAAAGTCCTTTTCACTTGAGTTGACTCCAACTGCAAACATTAAAATCCCCAACGCAGGG
GCATAGTACCTGTTGGTAAACCACGTAAAAGAAGGTGGATATATAAGAGCCAAAATTGTGCTACCAATAACC
ACATGGGGCAGAAAAGAGTTTGCTCCCTTCAAAATTGTCAATAGGGTGTTCTCTTTTTGTTTCAGAT
>TCONS_00047162
GGAAGAGTAACCGAGAACACGATGAGCGGAGAAAATCAACCATGGTTGTTTGTGCCTATTCGCTGATGCAA
AGCATTCCTCAATGAACTTGTATTGTTCAGATCCTTCTCTCCAGTCATGTTCAGTGTCTCCTATGCAGAAGTGG
AACATGCCATAATCGGCTGCATACCAGAACTTAGCTCTGTTTTCTGCTGGAACGTAGTATAATGTTTCGGCTG
GTACACCACATTCTCCACCGGAGTCCAGACCAGTGTATAATGATCCGCTGTTCTCCCAAGTTCTTTCATGATTA
CCACTCGCAATCATGAAAGGTACTTTCGATGTTATTGGTTCCACCTGAGCTGTAAATTGGTCCCATTGT
>TCONS_00047411
CATGAAGCATGCAGCAAGGATGGAAATGAAAATTCTAGAGATAGAGAAGGCAAGGACCAGAAACAATTAC
AAATGCTTTAAATTAAAGTTGAGATCAAATAATACATGCAACATTTTACAAAAAATCCATTGTTGTCACTGAA
AAATGGAGCATCAGAAAAACTGATATCTTTATTGAGAGATCTAATCTACAGCTGATCTGCACGTAACCAATT
CTTCTAGCCACGCAAACAAATACATAATCTTGGAGCCCAAAACAAAACAAAATGTGATTTAACAGCCAAAC

TCACTCGTCCAACGAGGAAGCACACAAAGATACTACACAAATATGGAACAAGAGAGGATCTTTCTCTACTCT
GCTCTAGATTGACCAGCGCGAGAAGAGAGGATGATGCCGACAATAAGGCCGTAAAGAGCCAAGGCTTCAGC
GAAAATAAGAATCAGAATCATCCCAACGAAAAGCTTTGGCTGCTGTGCATTAGCTCTAACACCAGCATCGCC
AACGATTCCAATAGCCATACCAGCAGAAAGGCCAGCGAGACCACAAGCAAGTCCAGATGAAAGGTGAGCA
TATCCATCAAATAAATAATACGACTTTGTTTTCGGGTTAATCCCAGTACTAATAATCACAGCTATAATTAACC
CATAAATACCCAAAACTCCAGCCATAACAACTGGCACAATTGACTTCATCACCAATTCCGGCCTCATTACTCC
CATCGATGCTACCCCTACCCCACTTTTCGCTGTTCCATAAGCTGCTCCCATACAGGAGAAAACTAAGGCAGCT
GCAGCGCCGAGGAACCCGAAGAACGGTGCCGTTTCATCGCCGCTAAAAGTCGATGCCATTTTTTTCTTCGAAT
TGGAATTGAAGCGATTCGCGATCTGAGAGG
>TCONS_00047484
TGTAAATGGTGGAATCTTGCATCGAAAGTGCAGTGCGGAAACAAAATATTGCGTGTGCCTTACACGAGTTAC
CCCAAATATCTGCTGGAAGCACAATTACAGCGTGACGGTGACATCTCTCAATTCTTCGCCATCGATTTCAAAG
TTCGGAAGGCGGTGTATAATTCCCATTTCCCAGTCGCAGCTGAGAATGGATGAATTAAAGTGCCAGACTTCGT
ATCAATCAAATCATATCCCAAATCCATCTGTATCTGACTCAACTCAGCTTCCTCCTGATTCCAGGCAAGACAG
TTTGTTCAATCCACAAGAAGAAGAACATATTGCCAAAGTAGTCTTAAAACCTGAGTGTTGCAGCTGGAATTTT
ATTATGGGATCTCCAGGTTGTTACTCCCATGCCTCAACTACTAGGCCATCTAATCTGATAGGCTGATATATTTC
CTCCTCATTTTCCCCAGCAATCACATTCCTATAGGTGTTAAATTGCAG
>TCONS_00047514
TATTTGTTGTTTCCTTCCACAATGGTAACCCTTCACATCTTGGACCCTTCAAGCGCCAAACAACCTGCAAGATT
GGAACACTTCAAATTGTTAGTTACCTCCTCAAACGGGGAGGGGAAAAAAAGAAATAGATCATCCATGATTCA
TGCTGTAGCAATTTTATCAAATCTTACTTCTGCAACCTTGATTGGGAGCGCCTGGTACACAGAAAAAATTAGT
ATGTCAGTGAAGTTGTAGTAAAAGAAGTACAGGAGAAAACAATGAAGGAAGACTACCATAAACGAAGTAG
GAGTAATAACAATCAAGCATAGGAAAGGGAAAATTCCAATTTATTGATTTTGTCAACTTGATTGACACAACT
GGTGATTTAGAAAGGTGAACAGCAGAATCAAGCTGCATGCTGTATAATTCTAATTTAGTACTAGAATATACTA
GGGTCTCTTGAAAAACTGTAGGCAAAAGCTTTAGAGCATGCCTTTGTGATATCTGTGGCTTAACTTTCTCCCTC
TCCTTTGGTCCTTGTGGTGTTTCGTCCTTTCTGATAGGCACATTTTTTACCTGCAATAAGTGTCATTAAGGAAGC
AAAGGTTAAAAACAATTTTATTAAAAGTAATTCATCTCATAAAGATGTTTGTGGTATACTGTCAATGTCATGC
AGCACTATACCAAGTTCCTAAACTCAAGATTGTAGAAAGTCTCGATGTATCTTTGCTTTGCTTGTTTCTGATCT
CTGTACAATGTCCTCCAAAAAGTATAGATGGACCCCATATTCAATACCTTGTTTGCATAGATCTTCCATGTGTA
ACTGCAAAAATTTAGGATTAAAGGATTAAGAAATTTTCTGCAAGATATGTGCTTCTTTGCAATTTGAATGGCT
TATCTGTGAGTTACCATTCATATATACGGTTTAGACCCTGGACTGAAATCCTGTTCCAATACTCAGGATCCTCC
CTGCATTTTTGGAAAAAGTTGGCAATTTTGTTGCTTGATTCATCCCCATTATTTGGATCAATATGAAAGCCTGA
GACCCCATCAACAATAATCTCAGCAGGGCCACCTTGGTTGGTAGCAAAGGTTGGTAATCCACAGTTCATTGC
CTCAATGACTGTTAGACCAAATGCTTCATACAATGCAGGCTGCACAAAAGCTCCTTTGGAATCTGCTATTGTG
CGGTAGAGTTCACTATTTCTGTATCTGTCAGTCTGAGCTGCTATCCATCTAATCTGACCCTTAAGCTGGTATTTC
TCTATCAGCATGTGCATCTTTTTTATTTCAGCTGCTTCTTCCCTATCCTTGGATTTTGTAGGATCAAAGGAACCA
CCAACCACAACAAGGTTAACTAAGCTTCTGAGCCTCTTGTTCTTGCCATACCATTCTGTTAGTCCAGATGTGTT
CTTCACTGTGTCCAGCCTTGCCATGGGCGGAAAGCAGTCAAACGCTTCTGCTTTTCTGTGTAAGGGAAATACA
CCGATTGGTCTGCCCCAGGTGCAGCAATATTAAACTTAGGATCAAAGACATTGATGCCTGAAGCAACTCTGT
AAAGCCCTGGAAGGGTAAATGCACTATGGCTCTCATACTGTCCTGGTCTATCTTTGCTGCATATGTTGAAGTAT
TCAAGAATGAGATCCATTAAGTTGCAAACTTTT
>TCONS_00047574

GTGAATAACAAATATACTGATGACGCAATGACTTACGTTACAAAATTGATTTCGAGCTGTACTCCACTTTTCT
GTGTTCCTCTGCAGTCCAAGCGACCCCTTTTCGCCGTTCTACCTCTGCTCCAGATAATCTACTTCTGCGGCTGG
AACAACCTTGCATTCTCCCGTATCGAGGTAGCGGAACTTTTCCAGCCTCGATATCATTTATATCATCAACAAG
TTTTTTATAATGTTGTAAGATTTCTGGAATAGATTTCCGTGGAACTGCTGCAGCAATCTTCATCAATTGATCGC
TATCTCCAGCATAAATGGCTAGGGCAGTTTCAAATGCTCTGTCTTCCTCCTTAGTCCAAGAGGAGCTATTGCAT
GTCTGGTCAGGGTTCATTTTGTTAGATCTACAGTGCTTTC
>TCONS_00047609
TTTTTTACTTAATTTTCTTTTGTTTCTGATTTTTGGGGTTTTGGTCTTTAAATCTTAATCTACAAATCCTAAAAAT
AATTCTGGGTTCTTCTTTACTGTGCTTGTTAAGGCTGTAGCTTTCGATTTGCTTTTAGGTTTATGCTTTGATTATC
GAGATAATTGGGGTACTAGCAAATCCCTAATTTTGATTTTGATTTTGATTTGATTTTTGATCCACAATTGAATCT
CCAAATGCAAAAATAGCTTTTACAGTTACGTTTTTAGGAATCAGAAGTAATTTTTCTTGAATAGCCATCGCCT
AAAGCTCTTTCTCAGTGTCGACTGCTCTTTCGCTGGTAAGTTGAAAACTTCTTTCTGAAATTTTAGACCTTTTGA
TGATTGCATGTTTGGTATTTCACTCAAAACCGTGAAATGTGATGTTAGTTGTAATGGGTCTTCATTTTTCTTGAT
ATTTTTTTCGATTGTTCG
>TCONS_00047634
TTTTTCTTTTACGCAATCGCCAATGTGAAAGCACAGATACTACTCCTACTTCTACAAACATCCGCACATGCCTA
GTCATTGACCTTGGTCTCTTTTGCACGTGCGACGGAAACTCAAAGTGGGGTCCATTATCTTGCTCAAAACGGC
TTTTGCAGGTACATGAATATATCCTGAAGCGGCGACATTTCATTACAGCCCTGGAACTGAGAACCTAGCAAG
CTTTTATCTCCGACGTCCGGGATGGCATCCATGTAATTAAATTGAAGGTCGACGGCAGTCTTCTCCCAATCGCT
CAATTTCGGCTCGCTCTGAACTTCTCTCTCACACGTGACCTCCGGCGAAAGCACCTGCTCCGAGCAGCTGGAA
TCAGTGTGAAGCTTCGGCAGAGAATCTGATGAATCGAAGTACATAAAGTCACTGTAAACCTGCGACGGCCTT
GTCGAGATCGGCAACGGCAGAATTTCCGGCTTCACATCCACCTCGCTCGCCGGCGGCGACATTCCGACATTC
ATTTTTTGACTATTCAATTGGTTCTTCTCCATTGTACCCTTCTTATTGTATATTCGGCATAAAACCCAATCGTCT
AGCCTTAAGCTATTGTTCTTGTTACGAGCAGATCGATCAACATGAGCAAGCCGATATTCGTGCATAATCCAAT
TCGTCTTTTCTCCTTTTGGAGCTTTGCCTGCGTAAAATACCAGAGCTTTCTTAATTCCGACAGGCTTCGGATGA
CCAATCGGCTTATCTGCACCCGTCGCCTTCCAATATCCATTTCCCGCAGCTCGATTTGGCCGTGAACCGTTCGG
ATATTTCCGATCTCGCGGTGAAAAGAAATACCACTCTTTCTCCCCATATAACGCCAAACTTGTACAGGTCAAT
TTCAGCAATAATCGGGACAGCAATCGGCTGCGACGCACATTTTCGACAGAGATAGTGCATCACAAGCTCTTC
ATCAGTTGGATGGAATCGGAATCCAGGAGGCAATTGCAATTCCACCGCTGCCATTCTTTTACAATGATTTTGA
CTTGCTGTATTTGTGTATTTATTTTTCTTTTGATTGATAGGTA
>TCONS_00047635
TACACCAATTTTTTCTTTTACGCAATCGCCAATGTGAAAGCACAGATACTACTCCTACTTCTACAAACATCCGC
ACATGCCTAGTCATTGACCTTGGTCTCTTTTGCACGTGCGACGGAAACTCAAAGTGGGGTCCATTATCTTGCTC
AAAACGGCTTTTGCAGGTACATGAATATATCCTGAAGCGGCGACATTTCATTACAGCCCTGGAACTGAGAAC
CTAGCAAGCTTTTATCTCCGACGTCCGGGATGGCATCCATGTAATTAAATTGAAGGTCGACGGCAGTCTTCTC
CCAATCGCTCAATTTCGGCTCGCTCTGAACTTCTCTCTCACACGTGACCTCCGGCGAAAGCACCTGCTCCGAG
CAGCTGGAATCAGTGTGAAGCTTCGGCAGAGAATCTGATGAATCGAAGTACATAAAGTCACTGTAAACCTGC
GACGGCCTTGTCGAGATCGGCAACGGCAGAATTTCCGGCTTCACATCCACCTCGCTCGCCGGCGGCGACATT
CCGACATTCATTTTTTGACTATTCAATTGGTTCTTCTCCATTGTACCCTTCTTATTGTATATTCGGCATAAAACC
CAATCGTCTAGCCTTAAGCTATTGTTCTTGTTACGAGCAGATCGATCAACATGAGCAAGCCGATATTCGTGCA
TAATCCAATTCGTCTTTTCTCCTTTTGGAGCTTTGCCTGCGTAAAATACCAGAGCTTTCTTAATTCCGACAGGCT
TCGGATGACCAATCGGCTTATCTGCACCCGTCGCCTTCCAATATCCATTTCCCGCAGCTCGATTTGGCCGTGAA

CCGTTCGGATATTTCCGATCTCGCGGTGAAAAGAAATACCACTCTTTCTCCCCATATAACGCCAAACCAGGGA
GATCCCATGGGTCGTACTTGTACAGGTCAATTTCAGCAATAATCGGGACAGCAATCGGCTGCGACGCACATTT
TCGACAGAGATAGTGCATCACAAGCTCTTCATCAGTTGGATGGAATCGGAATCCAGGAGGCAATTGCAATTC
CACCGCTGCCATTCTTTTACAATGATTTTGACTTGCTGTATTTGTGTATTTATTTTTCTTTTGATTGATAGGTA
>TCONS_00047644
AAACATATTTTAGATAATTTCTACCACGTGATTTAGTGCTTGACAAAAATGAATGCAGGAGCAAAGAAAAAG
GGTTTGAATCCAAACAAAGCTCAAAGTCCTATGAAGAAGATAGGTTTTGATTTTCAGTTTCGATCAAGTGCTG
AATTGGCCAAGCAATTTCAGATTAAGAAAGAAAAACTAGCTCATGATAAGGAGCATAGAATTGTGAAAGAG
CAAGCACCAGTAACTCATAAACGGAAGCATCTTATGCCTACAATATCTAATGCAAGAGAAG
>TCONS_00047649
GTTTATGAGTTACTGGTGCTTGCTCTTTCACAATTCTATGCTCCTTATCATGAGCTAGTTTTTCTTTCTTAATCTG
AAATTGCTTGGCCAATTCAGCACTTGATCGAAACTGAAAATCAAAACCTATCTTCTTCATAGGACTTTGAGCT
TTGTTTGGATTCAAACCCTTTTTCTTTGCTCCTGCATTCATTTTTGTCAAGCACTAAATCACGTGGTAGAAATTA
TCTAAAATATGTTT
>TCONS_00047739
AAAAGATAAGTTGTTCTTACTTTTCCTATGTCGTCACGAACTTTATTCAACACCATAGTTACTTCAGCCTCCAG
AGTCTGGGCAGCATCACAGCCTGGTTGCACGGCAAGCTTTCCTTCATTAAATTGTTGAATACGATCTTCACATT
TCTTGTAATTTTGATGAATCTCTTCTTGTTTTTGCTTAGCTAAGTTTTCCCCAGGTGTGACATCATCAATCCCTAT
AGAAAATCCATGATTACCAATCCACCGAGCACTTAATTTTGCAAGCCGATTCATGCATGTTGCAGCAGCATGT
GACTTGTAGTCCCTCAACAAAACAGAGTATAGACCATCCTTATTTCCATTTCCTAACGTAGCCTTCCCGAGTTG
CCCGCTGATCAGTTCACTATTACGGAAATAAACAAATCCATCACTTGGGCACATTGTTTCCCCTTTCCCGCTGT
AACTCTTTTCTGTGACTGTTAGATTTACATAGACTCTCATCTTTGAATATGGACGCAGCAGCACGCCAAAGAG
TTGTTTCCCAGTCCAAAGCTCAACCGGCTGTAAAAAGCCATCTCAGTCATTTGAAGATCCAAAGACAAGACA
AACAATAAATTCTACTGGCTTCAGAACGTAAAAAAAGATGGAAACAAAACTCAACCAAACCATACCTACTA
ATAAAAAAAAGGAAGGGTGTGCACTATGATTTTGGTTAGTGTTAATATGCATATTCCAATCCAAATCAAATAT
CTAGGAAGAATGATGACTTGTTGCTTTCGCCAAAAGCTTCAAAGATCAAAATTTTCAATCAAATGATATTTTT
G
>TCONS_00047757
TTTTTCATTAATTATTGAATTGAAGCAAAATCAACAATCAATCTCGTTTTAGGACTCATCGCTGATTGTGGCTA
AGATATAGACTAGTACTGCTTTGTATTCAACACTTATTGCTTCCAGATGTCTAGGACTTTACAACGTTGAAAAT
AAATACGGCTCGTGAAGCTTTGCCATTAATTGCATTCTAGAGTGGAATAAGGATTTTATAGTAATTCAATCAA
GTAATACCGACCAAAGTTGCAAGTTTCAGAAGAACCAGTGTAGAGAAGAGACTTGGTGTATTTCTTGGTGAG
TACACTTGCCAGAAGTCGTAGATGTGGGTAATGTTTGTTTATTATGTGTTCAACTGCCTCATGATAAATTCTTTT
ATATCTTACTTGAATGTTATTTATGGATGTCAATTTGGAGACATCAGTTTCTGGTATATCTATCCCTTCATTTGC
TAGCACACATGCATATCAGTGCACACTCCTATATTTGTTTAGGTTCTTGTCCCGATGCAAAATTGTAAATTTTC
TGAGAGGTTACTGGTTTGTGGGAAATGGCATGAAAACATAATCATGTAGGAAGCCGAAATGATTATGCATTT
TATTTTCCAAGCAAGAAAAGAAAAAAAGGAGATGG
>TCONS_00047758
ACTTTTTCTTTATATCTTCAACCTTCTTTCACTGGACGCCTCTCATAATTTTCAGATAAAAGTTTGTATTTTTTCA
TTAATTATTGAATTGAAGCAAAATCAACAATCAATCTCGTAAGGACAAATGCTGGACCTATTCCAATTTCATC
TTGGATTGTTTTAGGACTCATCGCTGATTGTGGCTAAGATATAGACTAGTACTGCTTTGTATTCAACACTTATT
GCTTCCAGATGTCTAGGACTTTACAACGTTGAAAATAAATACGGCTCGTGAAGCTTTGCCATTAATTGCATTC

TAGAGTGGAATAAGGATTTTATAGTAATTCAATCAAGTAATACCGACCAAAGTTGCAAGTTTCAGAAGAACC
AGTGTAGAGAAGAGACTTGGTGTATTTCTTGGTGAGTACACTTGCCAGAAGTCGTAGATGTGGGTAATGTTTG
TTTATTATGTGTTCAACTGCCTCATGATAAATTCTTTTATATCTTACTTGAATGTTATTTATGGATGTCAATTTGG
AGACATCAGTTTCTGGTATATCTATCCCTTCATTTGCTAGCACACATGCATATCAGTGCACACTCCTATATTTG
TTTAGGTTCTTGTCCCGATGCAAAATTGTAAATTTTCTGAGAGGTTACTGGTTTGTGGGAAATGGCATGAAAA
CATAATCATGTAGGAAGCCGAAATGATTATGCATTTTATTTTCCAAGCAAGAAAAGAAAAAAAGGAGATGG
>TCONS_00047774
CCTTCCAAAGAGTTCCTCAAATTGATCTATAGACACCATTAATGCAGATGGCTCAGCCAACTTATTTACTGTCT
TGAACCTAGCAACTGGAGCAACTATGTTGGCACGCCCAGGGTTTGAACAACCGATCTCCCCTGAAAGGTTTA
GCAGCCGTACACACGGATACCCGTCAATGGCCATGTACATTGACTTCTCAAGATCAGGAACTGACTGGAAAG
AGTTCACTTGGTCTGACGCTGAGATAGAGAGGAAAAACTGAGCAATGAAGCAGAGAAGAAAAAC
>TCONS_00047775
CCTTTAGCTCCCAATTTCTGGCTTTAAAGGCAAAAGGTTTTGAGGTAAGGGCTCCAACTGGACAAATATCAAT
CACATTCCCTGAAAGTTCACTTGTCATAAGTTTTTCAACATAAGTTCCAATTTCTTCTCCACTACCACGACCCA
ACATTCCAAGGTCCTCTGTTCCAGCTACTTCTGTAGCAAATCTGACGCACCTTGTACACTGGATGCACCGGGT
CATCACAGTCTTCACCAATGGGCCCAAATTCTTGTCAACCACAGACCTCTTCATTTCAGTGAAACGCCCACGG
TCTGAGCCAAAGGCCATAGACTGATCTTGGAGATCACATTCTCCTCCCTGATCACAAATTGGACAATCCAGTG
GATGATTCATAAGCAAAAACTCCATGACTCCTTCACGAGCCTTTTTGGCAATAGGTGTATCTGTCTTGATCTTC
ATTCCTGGAAGTGCAGGCATGGCACAAGAAGCAACA
>TCONS_00047783
AGCAACATATATTTTAAAGTTTGCGCATAAGCAGAGGAGCACCATACTGTGGATGAATCGTCACAATTGCAA
ATGGAGCATGTGTATAAGATGGGGATAGTTCAAAGGAGAATTTTTGTAGTATCATAGCTACTGCCATTTTTGC
TTCCATCATTGCGAAGTTTTGTCCAATACAAACACGAGGTCCCCAGCTAAATGGTATAAATGAGACTTGTCCA
TTTGTTGCCTTAGACACTCCTTCACTGAATCTTTCTGGTTTGAATTCCTTTGCGTCTTCACCCCATATTTCCTTAT
CATAATGTACTAAGATTGCTGGTATAACGAGTAGCACTCCAG
>TCONS_00047793
GGACTCGCACAGTGACACTGCTCCCAATCTTCCACCGATTGGTACTTCGTTCGACGGGTAGCGATGGTGATGA
CCAACTTCAATGGTACTGGTGTAGGTTTCGGTGCTGGCATTGGTTGCGGTTTTGGAGTAGGATGGGGTTTCGG
AGGTTTGCTTCTTTCCAATTTCTTACTTAACAGTTTTATTGTGTTTGTATCAGCTAAAA
>TCONS_00047812
CTCTCTCTCTCTCTCTATCTATCTATCTATGTCATCTCTTAATGTTAATTTAGAACTGTTATTCTCTAGACATGGT
CAGTTCCCAGCTGAAGCTTCATAGGTTTCACACTGGTCGTGAATCCAGGGTTGTGACAGCTCTTACTCTTTTGA
AGACTCACCTTTTCAGCTACCAAGGCCATGTCTCAGCTGCTTTGGTCCTTGGTGGTGTGGATGTCACAGGGCC
CCATCTGCATACTGTA
>TCONS_00047977
TAGCAGCTATCTCTAGAGGGAAGTTGTGAGCATTACGTTCATGCATAACTTCCATACCAAGGTTAGCACGGTT
AATGATATCAGCCCAATTGCCGTCAATAAGGTAGGGATCATCAAAACACCAAACCATCCAATGTAAAGACG
GTTTTCAGTGCTGGTTATCCAGTAACAGAAGCGACCCATAGGCTTTCGCTTTCGCGTCTCTCTAAAATTGCAGT
CATGGTAAAATCTTGGTTTATTTAATCATCAGGGACTCCCAAGCACACGAGTTTTCTACAAATAAAAATATAA
AATGGAAGGCTTGTTATTCAACAGTAAAACATGACTTATATACTC
>TCONS_00047978

CTCTAGAGGGAAGTTGTGAGCATTACGTTCATGCATAACTTCCATACCAAGGTTAGCACGGTTAATGATATCA
GCCCAAGTATTAATTACACGACCTAAAGTGGTAAACCAGATAACTACTACAGGCCAAGCAGCTAGGAAGAA
GTGTAACGAACGAGAGTTGTTGAAACTAGCATATTGGAAGATCAATCGGCCAAAATAACCATGAGCGGCTA
CGATGTTATAAGTTTCTTCCTCTTAACCGAATCTGTAACCTTCATTAGCAGATTCATTTTCTGTGGTTTCCCTGA
TCAAACTAAAAGTTACCAAGGAACCATGCATAGTACTGAATAGGGAGCCGCC
>TCONS_00048006
GGAAGATAACAGGGAAATTATACCTCAATCATTCAAGGGCATCAGTTCAATCTTCTTCTTCTTGTTTCACGAA
GAAGTCCACTATCTAAATCTGAAAGCCTCGAGAAACCACTTCTCCTCCTGGAAAATAATGAAGATCGACTCA
CAGGCAGATTTCCAGCATCAGTTGTAAGGTTTTCCAACTGAAGTGCAGCATCAGAAACTTCATTTGATTGAGA
GGAAGGAACAGATGACGTAGCAGAGCTTGTCTCCACAATTGAATCCAGAGTCTGACGTTCTGATCGTATGAT
AGCAGCATCTCTTCTCAAAGAATTATCTTCATCAGCTGCAACGTGACTAATTGGTAAAACTTGCGAATTGTTTC
TTTCGCGAATGCGGTCTGTGAGTGACGCTGCAAGGTCCTCAAACATCTGCTGTGCATTTTCTAATTCCGATGAA
AGAGAGGCAGCACCCTCTGATAATACCCTCGGAAACACTCGTGACCGTAGTCCCCGGCTGCCTATGGGATCA
GCATTTTGCAACTCATGAGAGCTGCTCAGAGAACTTGAACTAACGTCACCAGTCGCAAGTTGCTGTGACGGG
TGATCCCCCAATCCAATACTGGTCCTAATTCGCCTAAGTGCTTCTGCAACTGGGATATGAGACAAACCCCGAG
TCACACGCTGCTGTCTAACACTCTCTACTCTATGTGCTTTTGGCCTTGGTGGTATTTTGAAACCACACTCCACA
AGTTCTTTTGAGCAGTAACTGTGGCCATTACCATAAATTGGAGTAATATTTTCATCTATAACCTCTCCCTTGCA
AACAGGACACTCTTTCGTACTCGAATCAACATAAGGCAACTGATAAAAGCATGACCAGCAATATAAATGGC
CGCAACAAGTCAAGATAGGTTCTTTAGCCATATCTAAACATATATTACAATCAAAGAAACTTCCACTTCCACC
ATCCTTACCTACTTTTTTAACCACTAAATCCATCTCCAATGCCTTTGCTACTAAATGGGATCTATCTCTTTTGCA
TCCTTTTTCTTGCTTCTCATTATCCACGTTATTATTACCAATATCATCAGAAGAAGAATTACGAGTTTGGGAAC
GCCACCTATGGTGTTGCCAAGTTCCAGCTGCAACAGCCGCAAGCTGCAGAATTCTTTGCTCTATCCTACTATT
AGCAGTTTCCAAATCATTCAACAAAGACCCTAATCCTAAAGCAGTACCAGAAGATGACTCCGTAGGTTCCTG
GTTCAAATCAAGATCCATTACTACACCATTCATCAAATTATCCGCCATCGAAGAAACCCTTTTCAAGTCTGCA
TAAAAGAGATAATCCAGGTATCCGAATTAAAGGAATCAATAGCATGTACACTGATTTGGCCGTCCACCATGG
GTTCTTCTGCTTCTACAAACAGCTTCCGAGGTCTTCAGAGAGA
>TCONS_00048207
TCCCTATGCCACCAACTCCGTGAATACCAATCATGCGAACTCCATCTTCACATTCATTTTGCAATAACAACTCT
ATATCTTTGACACGAGAATCTACTCCTACTGGGTGCCAAGCAACATCTAGAGGTGTCTGGTTAACCTCTTGTA
GGACTTGCTGTATAATTTTTTCAATAAACTTTGATTCATGCCCGTCAGCAACATTTTGCAAATCCCATCCAGAT
AAATTTGCAGCTTCAGTAAGTGCAGCTCTCCATTTCTCCACCCTTTGAGCTCCAAATGGTCGTTCCTTGTGTTTA
GCCAAATATTCACCAAATAACCCAGTTTGCTTTCTCACTTCAGAAGGATCAACATCATAGAAAATAGGCAAA
ACCATCTGCTTTAGTTTCTCTTTGCATTCAAGAATTTTAACTAGTTCATTTAGACACCAACTAGAGGAAGCATA
ATTTCTTGAGAAAACAATAATGGAAATTCTTGACT
>TCONS_00048333
AACAGACAACATTAGATGTTCTCGGCCAACGAAGCCTTCCTTTTTTGGTCTACATCCTTTGTAAGGTAAGTTGG
ATAGTTCAATACTTAGATGTGTTACAAGTCAAAATAAGGTAAGTATGATTCCAAGATTTCATTACTTTCAGTAT
ATCATATATACATGTCAGTTCAGTTTAATTAAGTTATTCATATGTTATCTGCCTTACATAATTAGTACATTTTTT
TGTACTAATGTCCCATAGTCGGGGAACTGCATTCATGCCTGCAGGTTCAGGTATCCAGTTTGGCAGGCTCATG
TCATAGACAGAGTTGATATCCAGCTGATGACTGGCAAAGCTCCATTTCATTAGGACTGCAGCCGATTCTAGAG
GTTATCAGACTATCCTGTGTGTGTTTGTGTATATATATATATATATATATATATATATATATATATATATATATA
TATATATATATATATATATATATATATACTTATGGGTTAGAGTCCTGTCCTGACCATATTTTGATGTCATATACT

TTTAGAGGCTTTGTAGACACAGGTTCATTATGTACAGTATATCAGAGGCCTTGACGACCTGTGTGTATTCATGT
TCTGATTATGATGGTTCGCTGCTACAGCATTTGCTCACTTTCATTCATACCTTATGAGTTGTGGCCATGTTGGCC
CATGATAGTTACAAGAAGAATAAGTTCAGCCAAGTCAGCCTATGTATGCTCTAGTTTTCATCGAACGATCATG
ATTTACAGAGTGGTTCGCTCGGGCCAGTACGACACCGGGTGCCAGCCACGCCTCCCCAAGTTGGGGAGTGAC
ACTTTGGCCCCAAGATAAACAAGGAAGGGGATAATGGATCCCTGTCTCACCGCCCCAGTAGAGTGAAAGAA
CCCCTTCTTAATGTAATACCACATATTAACTATTGATATTCATACCAAGTCTATCCATTATTCTGCAAACCCAA
ATCTCCTCAGTGTTACCAGCAAGAAGTTTCAATTCATTCTGTCATAAGCTTTGACATGTCGAGTTTAAGGACTA
CATTCCCTCATCTTCTTTTCCTTTTAATCCCATGTATGATCTCTTGAGCTAACAAAACTTTTTGGTTATCAACCTT
CCATGAACAAACTCCCTTTGGTTTTAGAGATGAGCTTCGGAAGGATAGGGGCTAATATGGAGCTCATAATCGT
AGAATAATCTTATTAGAATAATTACTTAGATTGATTGTCTGAACTCAGAAAAACACGAAGGACTTTTCACCCT
AGGAATTACAAATAAATATGTGTTACTGTAAAACTTAGAAAGTCTAGAGCCATCGAAGAATAACTGTACCAT
ATTAGTTAGGTCAAAATTTATAATTTCCAATCAGCTTTAAAAGAATGCCCCTCCATATCCATCTGGTCCAAGA
GCACTATTGGGATCCATGTAAAAAATAGCTCTTTTAATTTTCTCTTTATCATGGATGCTGTCAGAACATCATTA
TCTGCTTGAGTTACAATCTGATTAATGCATTGTGTAGGTGTTGAAGATGAGAAACTCCTTCTGCAGAAAAAAG
CTTCTCAAAGTAGTTAACTGCTTCCCCTGCTCCCAGTAATCCAATCTCCATTCTAATTCTTTATTTTTGTATATA
GTTAGTTTTCTCCTTTTCACCTTTTACCGTGATAATGTCATGAACATTTTTCCGGTTAGAATCTTCCTCTTCATGT
CATTTACCAGAGAGATAGTTATTTCCTATGACAAAAGGATATATAACCTGATCTTCTCACTATAAAATTTAAT
AATCACATGCATAGGGAATTCATCGCCAATCGCTGATTTTGATCCAGTGTTGATTTTCTTTTGTTGCAACATAA
ATATCTCTTCATAGGTGATGGATTAAAAAGAAACTTGTATGAGAAGTGCTATGACTACAAAAAAGAATTAAA
AACACGACACAAGTAGAACCGCAAAATACGTTGTGCTTTCTTAGCTTCATTATTATCTTTTTATTACTTTTTCTA
TGTTACCTTTCATATAGTCCAATTTCTATAAATTATAGGGTAGACATTCCATTAAAGTGATGATCATCTTCAAA
ATACGCTTCATACATATTTATACTTTATTATATTAAAATAAGTAAAAGAAATTATCTTATAAAAATTTGATTGT
TCATCTTCATAAGTTCAGAGGATAATGATCCACATTCATGATTCATAAAAGCTTATACTTAATTATTGTTTCAT
GTATATATCATTATCTAAAGTTTTATTTAATAATATTACTATTAATTTATAAAATATATATTGTATAAATTAATA
AACACGTGGCGAGAGACTAATTTTATTAAAATCTTTTTAAAAGAGAAACAAAATTAAAAAAGTAGCCCCAA
GAAAGGGCTATTTCAAGAAATGAGCGGGAGAAGGATTGCCTCCAAAACAAGGTGCATATGTTCAGACAGCA
AAATAGGAAGAGACATATACGGGCGACGTGTCAGCAAAACAATAAGAAAAGGTCTCCCCGGGGATGCTCCG
TTAAAGTTGGCGTTTGTCTTTCTCCAATGCCTTTCTTCTTTGCTTGACTTTCGGTTTTCCGTTACCAAAAAAGTCC
TCCAAATGCGACGTCGTTGTCACTTCCACTTCCCCTCATTTACACACCCAAAAAGAAAAGGCCAAATTAAAT
ATTTTAAATTCAAAGCAAGTAAGAAAGTACACATGTATTATTTCTCGTTCATCGCTGCAGAAAAAAAGTATTA
TCTTCATTTTGATATCACGTATTTACCATTTCGTTTAATATGGGTTTGAATGTGATTGTCCCCAATGCAGGATAA
TTTGTGTTGTTGATAGCGAATGCGAACCTTATTCACGCCGAGACCATGACTACTCAAAATGCTGACGCTCCCC
AGGTAGAAGTGGGTAGTCAGCCGCCTGCACCACCTGCGGCGAATGACCAAGCACAGTTGCATTCTGGGAATA
AGGTTTACAAGAGTGGCCCTCTATTTTTATCATCAAAAGGCTGAAACACTAGAGGATTTGCTTGAGTGGAAAG
CAGCACTTGAGGAGGCTTTGGCAAGTGCACCAAATGCTGATCCTGTAACTACCCAAAATGGAGTATCTAGGA
ATGATCAGGGCAATGCAGTTGACCATTCATCAGAGCAGTGTAAGCTTCTCATTGATATGGTTATAAATGATGT
GAACCTTTGACGTATATCTGTCAGTTATAAGAACAAATTGTGGGGAAAATCTGCCTATTATATAGCCTTACTT
ACATTTGATTTACAAGACAACTCTTTTGCGAG
>TCONS_00048385
CTTTGCCAACACCTTCATCTTCTTTGTTACACCTTGCAAATCAAACCTAGCCAAGCCCGGATAAAAATCGGAC
ACATTCGGAGTACCTAACAACTCAGTCATCTCAGTCACAACAAGCCTAAACTCTGCACCAAGACTAGATCTTT
CCTCTCCCTTTACTGTACCACCCCATAACATACTTGTAATCACATTAAGCACAGTCAAAAACATCTGTTCACC

AATATTTACTGCTACACCCTTCTGATTATAAAGGTAATTAATTGTCTTTCTAAGCTCTTCTCTTCTTAGTTCATA
AACAGAATCTAAAGTAGAACCACTAAGCATATCACGAACACAAACTTTCCTCAACATACGCCATTTTGGTCC
ATAAGGAGTCCAAACTATATCTTTACCACCATATGTAGCTTCTCTACCAGCACCAGGCACATCTCTATTAGCA
AAAATTGTATCTTGATCCTTAAGAATCTCACGAGCTAAAGCAGGAGAAGTAATAATAATCCCAACTTTTTTAC
CAAGCCATAGTCTACAAATGGGGCCATAAATTTGGGAAAGAGAAGCAAAATAGGTGTGAAGTTCAGGATCA
AGAGAGAGGAGATTACCTACTAAAGGCAAAGCTTTAGGCCCTGGTGGCAATGGGGGTTGTTCCTTCTTTGACT
TATTGATGAAAATCCAAACAAACCATAGTAAAGCTATAATTCCAAGAATATAGGAGAAAAAGACACCATTTT
GCTCCATATTTTGTTTCTGCAAATGCCATGGAAAAGAATCAAACAGAGGTTTGAG
>TCONS_00048386
CTTCTTTCATAACTGCATAGAGATATGGTAAATGCTGAATATGAGACTCTTCCACTATGTTATCTTTGCCCACA
ACTGTCTCCAGTTCCTGTTGTAATTTCCTCAGGACCTTTGGGTTGTTCGTAATTTCAGCCATGGCAAATTCAAC
AGTGTTGGAGGTTGTGTCAGTTCCACCAACAACCATATCCATGAGTAAGGCTTTGAGTTCAGTCATGGTAAGA
GGCATTTTAGCATCTGCTTCATCTTTCAACTTGAGCAAAACTTGCAAAAAATCCTTGCTTTCTTGGCCAACACC
ACTTCCTATCCCACCATTTCTATCCATTTTCTGTCTTTGATCAATCATAGTCTCAAATATCCTATCAAATCTCTTT
ACCAACACCTTCATCTTCTTTGTTACACCTTGCAAATCAAACCTAGCCAAGCCCGGATAAAAATCGGACACAT
TCGGAGTACCTAACAACTCAGTCATCTCAGTCACAACAAGCCTAAACTCTGCACCAAGACTAGATCTTTCCTC
TCCCTTTACTGTACCACCCCATAACATACTTGTAATCACATTAAGCACAGTCAAAAACATCTGTTCACCAATA
TTTACTGCTACACCCTTCTGATTATAAAGGTAATTAATTGTCTTTCTAAGCTCTTCTCTTCTTAGTTCATAAACA
GAATCTAAAGTAGAACCACTAAGCATATCACGAACACAAACTTTCCTCAACATACGCCATTTTGGTCCATAA
GGAGTCCAAACTATATCTTTACCACCATATGTAGCTTCTCTACCAGCACCAGGCACATCTCTATTAGCAAAAA
TTGTATCTTGATCCTTAAGAATCTCACGAGCTAAAGCAGGAGAAGTAATAATAATCCCAACTTTTTTACCAAG
CCATAGTCTACAAATGGGGCCATAAATTTGGGAAAGAGAAGCAAAATAGGTGTGAAGTTCAGGATCAAGAG
AGAGGAGATTACCTACTAAAGGCAAAGCTTTAGGCCCTGGTGGCAATGGGGGTTGTTCCTTCTTTGACTTATT
GATGAAAATCCAAACAAACCATAGTAAAGCTATAATTCCAAGAATATAGGAGAAAAAGACACCATTTTGCT
CCATATTTTGTTTCTGCAAATGCCATGGAAAAGAATCAAACAGAGGTTTGAG
>TCONS_00048388
TTGGCAAAAACGGAGTCTTGATCACGAACTACTTCTTTGGCTAAGGATGGTGAATTCAACACAATGCATAGTT
TGCTTCCTAGCCAAAACTTGTATATTGGACCATACTGTTGAGACAACTCTGTAAGCTGGTGGTGCAAATTGGG
GCGGAGGAATGGTAAGAATCCCACAATTGGAAGGCCACGAGGCCCTGGTGGCAACCTAGGTTCTGTCTTCTG
TGGTATATTTGATGTCCTTTTGTACCATATAATTGTCAGGAACACAATTGAAAGGGTAATACCATGAGTAAGG
CTTTGAGTTCAGTCATGGTAAGAGGCATTTTAGCATCTGCTTCATCTTTCAACTTGAGCAAAACTTGCAAAAA
ATCCTTGCTTTCTTGGCCAACACCACTTCCTATCCCACCATTTCTATCCATTTTCTGTCTTTGATCAATCATAGT
CTCAAATATCCTATCAAATCTCTTTACCAACACCTTCATCTTCTTTGTTACACCTTGCAAATCAAACCTAGCCA
AGCCCGGATAAAAATCGGACACATTCGGAGTACCTAACAACTCAGTCATCTCAGTCACAACAAGCCTAAACT
CTGCACCAAGACTAGATCTTTCCTCTCCCTTTACTGTACCACCCCATAACATACTTGTAATCACATTAAGCACA
GTCAAAAACATCTGTTCACCAATATTTACTGCTACACCCTTCTGATTATAAAGGTAATTAATTGTCTTTCTAAG
CTCTTCTCTTCTTAGTTCATAAACAGAATCTAAAGTAGAACCACTAAGCATATCACGAACACAAACTTTCCTC
AACATACGCCATTTTGGTCCATAAGGAGTCCAAACTATATCTTTACCACCATATGTAGCTTCTCTACCAGCAC
CAGGCACATCTCTATTAGCAAAAATTGTATCTTGATCCTTAAGAATCTCACGAGCTAAAGCAGGAGAAGTAA
TAATAATCCCAACTTTTTTACCAAGCCATAGTCTACAAATGGGGCCATAAATTTGGGAAAGAGAAGCAAAAT
AGGTGTGAAGTTCAGGATCAAGAGAGAGGAGATTACCTACTAAAGGCAAAGCTTTAGGCCCTGGTGGCAAT
GGGGGTTGTTCCTTCTTTGACTTATTGATGAAAATCCAAACAAACCATAGTAAAGCTATAATTCCAAGAATAT

AGGAGAAAAAGACACCATTTTGCTCCATATTTTGTTTCTGCAAATGCCATGGAAAAGAATCAAACAGAGGTT
TGAG
>TCONS_00048393
AGTTTCCAATCATAGGAATGCAGCAATGAGGCCAATACAAACATAAGCATTTTCTCAGCTAAAGGAAGGCCA
GCACAAATTCTCCTCCCTGATCCAAATGGAAGGTACTTGAAATTATTTCCGGAATAGTTTAAATTTGTAGAGT
GACTCAAGAACCTTTCAGGCTGAAATTCCAATGGATTTTCCCACACTTGAGGATCCCTATGAATCGCATAAAC
ATTCAAGAATACTTTAGTTCCCTCAGGTATTTTGTATCCACCTACTGTTGCAGATTTGCTCGGGTGCTTAGGGA
TAAGCAGTGGTAATGCAGGGTGTAGACGTAGAGTCTCCTTTAAAACCGCATCAAGATAGTGTAGTTTCAGCA
AGTGAGATTCTTCAGCAATGTTATTCATC
>TCONS_00048398
GTTCATTTCAATCTGATACTCAACTGTACTTAGTAATTAGTATTAAAACATCAAAAGATAAGGACATCTAAAG
GCAATGTGCTAGTCAATTGAGAAATAATATCCCCACCTCCTAGGTAAGATGAAATATAAAGCTAAACATTCA
GAATGAGATCCACGAAGCACCGACCCTATCGCGAGGAATTAGGCGCAGTTCAGATCCATGTTTAAGAAAGA
CGTTACCTGCACTTTGTTGAGGATTAATTCCCACTCCATCATTAGTGATGATGGCGCTTGTTTGGGGAGGGACT
TTGAATTCCTCCGCCGCAAATTTGAGAACAGCGGTGAACGGAGCTGCCTCCGGTACGCTAAAAACTTTGAAT
GGCAATTTGGGATCGGAAGTAAGAGTGACTTTGAACGACACTTTTCCTCCACCAGCAGCAGCACCTCCACTC
GCCATCTTTTCTCACTACCGCTACTGTATTGGAATTTTTCTGC
>TCONS_00048444
CGGGGTAAATAGAAATGGAAAAGAGATGTTTCTATTTCATCCAAATTAGAAGCTTTTTCTACATCCATATGAT
CTACTAAAGTAGATCGGTATTGACCATATAATAAAAGCAGATCTTGGTCCATGGAGTCCCAAGAAGGTAAGA
ACTCCAACCTGTCCGATTCTTCTTCGGCCAAATACGATATACAAGTGGGACCTGCACTATGAGCAAGCTGTGC
GAAAAACCGCTTCTTTTTTCCGGTTCCGAAACAACTTGGGCGAAATAGGGTTCTACCGGGAAACCATGGATTT
TTGAGTTTTCGCCATTGGTTACTGGTTGAGCCATGGAATGGAATGAGATAAGAATCCCTGGAGATCTTTCCTCT
CCACTTACTCGTAACAGGCTTTTCTTTTGTAGAAGACTTCTTCCGAATGGCATAATTGTCCGACACTACGTTCC
TCGATCTTCAAAGAAAACTGACTCCTCCTACTCCTCCTTCTCCTTTAGGATAGGATCCTCCTTGGTCCTTCTTTC
ACTAATGGTCTCACCATTTT
>TCONS_00048445
AACAAAGATATGAACGGGGTAAATAGAAATGGAAAAGAGATGTTTCTATTTCATCCAAATTAGAAGCTTTTT
CTACATCCATATGATCTACTAAAGTAGATCGGTATTGACCATATAATAAAAGCAGATCTTGGTCCATGGAGTC
CCAAGAAGGTAAGAACTCCAACCTGTCCGATGCTTCTTCGGCCAAATACGATATACAAGTGGGACCTGCACT
ATGAGCAAGCTGTGCGAAAAACCGCTTCTTTTTTCGGTTCCGAAACAACTTGGGCGAAATAGGGTTCTACCG
GTAAACCATGGATTTTTGAGTTTCGCCATTGGTTACTGGTTGAGCCACTGGAATGGAATGAGATAAGAATCCC
TGGAGATCTTTCCTCTCCACTTACTCGTAACAGGCTTTTCTTTTGTAGAAGACTTCTTCCGAATGGCATAATTGT
CCGACACTACGTTCCTCGATCTTCAAAGAAAACTGACTCCTCCTACTCCTCCTTCTCCTTTAGGATAGGATCCT
CCTTGGTCCTTCTTTCACTAATGGTCTCACCATTTT
>TCONS_00048447
AACAAAGATATGAACGGGGTAAATAGAAATGGAAAAGAGATGTTTCTATTTCATCCAAATTAGAAGCTTTTT
CTACATCCATATGATCTACTAAAGTAGATCGGTATTGACCATATAATAAAAGCAGATCTTGGTCCATGGAGTC
CCAAGAAGGTAAGAACTCCAACCTGTCCGATTCTTCTTCGGCCAAATACGATATACAAGTGGGACCTGCACT
ATGAGCAAGCTGTGCGAAAAACCGCTTCTTTTTTCCGGTTCCGAAACAACTTGGGCGAAATAGGGTTCTACCG
GGAAACCATGGATTTTTGAGTTTTCGCCATTGGTTACTGGTTGAGCCACTGGAATGGAATGAGATAAGAATCC
CTGGAGATCTTTCCTCTCCACTTACTCGTAACAGGCTTTTCTTCTGTAGAACACTTCTTCCGAATGGCATAATTG

TCCGACACTACGTTCCTCGATCTTCAAAGAAAACTGACTCCTCCTACTCCTCTGGAATGGAATGAGATAAGAA
TCCCTGGAGATCTTTCCTCTCCACTTACTCGTAACAGGCTTTTCTTTTGTAGAAGACTTCTTCCGAATGGCATAA
TTGTCCGACACTACGTTCCTCGATCTTCAAAGAAAACTGACTCCTCCTACTCCTCCTTCTCCTTTAGGATAGGA
TCCTCCTTGGTCCTTCTTTCACTAATGGTCTCACCATTTT
>TCONS_00048569
TGGATACGGCTTTTTGATAAACAAGATCCAAACTTGATTTTCTAATATTATTATCTCTCCATAAACTTCATCCG
AAAAACATAATAATCTTAAACAAACAAATATCCATCAAATTACCCAAAAAGACCTACTTTTTTCTCTTAAGA
GGCCCAGTTTTATACCCAGATTTAGTAGGTTTACCCCAAGGAGTCAATGAAACCCTGCCATGACTTCCACTAC
TCTTACTCCTCCCTTCACCACCCCCATGTGGATGATCCACCGGGTTCATCGCCACACCCCGAACTGTCGGTCTC
CGTCCTAGCCACCTGGCATGTCCCGCCTTCCTCAACTTCTTCGTCCCGTGTTCCGGGTTCGATACTTGCCCTAT
AGTAGCCCTACATTTAACGTCGATCAATTTCTTCACCCCCGACGGCAACTTCACTTCGCAGTATTTAGTAGAA
GCGCTGGGTTCGGTCAGGATTTTGGCGGACGTGCCGGCGGAGCGAACTAACTTGCCGCCTTGTCCGGGTCGG
ATCTCGATGTTGTGAATCAGGGTTCCGATTCGCATCATGCCTAATGGCATGCAACTTCCGATTTGGGAACTGA
TGTCTAGACTGAATTGGCTCATCATTGATTCCAGCTCCTTTGACGGTTTCATTTTGGACGCCAACGCTTCAAAT
GGACTGGTAGAGAAAGAGCGGT
>TCONS_00048633
TTTTTTTACCAGAAAAACCCTAGCTCTCTCTTTTTATCTCCCCTCATATTTTTACCAATTCATACTCCATATTTAA
TCAATTGATTTTTTCCGAGCAGAATTGATACAAGGGATTCATATAGTTGACCCCATCTAGTTTGAGATTGAGG
GAATTCGTTGGCTGATCTGATGTTTATTGATTATCACATGGATGCACTATTTCTCTATTTGCATGTCACTCTGTA
TTTAATGATGGCTCTATGTTACTCGGTATAATCTCTGGTCCTCTTGCAAAAGGTCTGCACTATTGTTCTTTCCTT
GATTGAGCTTCATGCTTCCATTTTACATACATAGACAGACCAGAAAAACAAGCAAACACATGGCTCTTCTAA
GTTTTGTGTTCGATCTGTTTTCCCTGTTCTTAGCAACCTATGCCCTATGGATAGCTAGAGCACTTACTCAAGGTC
ACTGAAATTTATGATTTGTTGAAACCTGTCATGTAGTGAAATTCTTGCTCTAGATCTCTGTTCTTTTGTCATGTG
TTGAACCATAAAATAGGAAGGAGATACTAGTTAAACAAGAT
>TCONS_00048642
TGATTGCCTCGAGGGTGATGTAGCTGACGATTGGGCTACAAGAAGAGACTTTTCAATGTCTGCCAACCTGATC
TCTAATTTTGCATGCATGACTGAATAAAGTAACTGCTGCCAGTGCCAGAATCTTAGTCGAGTCCCACTCCGAA
ATGCAGAAGGTATTTCAGCCAGCAAGCTCAAAAGGGATTCGTTATTGTCACCATAAAACTCTGCTCG
>TCONS_00048660
CGAGCAGAGTTTTATGGTGACAATAACGAATCCCTTTTGAGCTTGCTGGCTGAAATACCTTCTGCATTTCGGA
GTGGGACTCGACTAAGATTCTGGCACTGGCAGCAGTTACTTTATTCAGTCATGCATGCAAAATTAGAGATCAG
GTTGGCAGACATTGAAAAGTCTCTTCTTGTAGCCCAATCGTCAGCTACATCACCCTCGAGGCAATCACCAGAG
CCTGCTAGTGGGTTAACATGGGAGTACTGTAC
>TCONS_00048749
TAAGGATGGTAGACCACTGGATGTTGATTCTGCCAATGCGATCGTCCTTGACACCAACAGTGGCCATGAACG
AGTACCACAAACATCAAGGATACCTAGTGTTGCTGAAAATACTTCACTATCTCAAGGTACTACTCATCTTTAT
CCTTCGGTGCTACTATTTGAGTTTTCTATTGTTTGAATTTTCCTCCAATTTTTTTTATCATTAGAATCT
>TCONS_00048792
TGTCTGCCTGAGCAACGTTTTCCAAGAAAACTTAGGTTCTAACAAAATCTGTAAGTAGACAAGAAACTTCCTA
AAATACGCTCAAGGGGTACAGCTGGAAAATGAGATACTTCGTTCACTGAATTCTTTGTATTCTTCTAATCGAA
GAGACTGAAGCCCATGTCATCGTCTGACTCCTCTTTCTCTTCCACTTTCTTTTCTTCCTTCTTCTCCACAGCAGC
AGGTGCAGCAGCAGCGCCACCTGTAGGTGCAGCAACTGCAACAGCACCACCACCGGCAGGTACTGAAGCCA

ACTTTTCTCTGCCAGCGGCAATCAGCTCAGTGATATCTTTGCCCTCAACTTGAGAGAGCAGCAGTTCAATCCT
ATCATCATCAGCCTCAGCCCCAACAGATCCAAGGATGTTCTTCAAATCTTTAGCCTTAGGGCAGGAGTTGCCA
CCCAATACGGCCAACAAGTAAGCTGCGATTACCTTCATTTCTCTTCTCTGTTAAAAA
>TCONS_00049045
GAGTATGATATTTGATCTGTTTCTGAAGGAAGGAGTAAGAACCAGAAGCACTCATTAATTTACATCATTATAG
TCGTGTAAATTAAACTATTACAAGCTGAGCAATTTCAATGTGACTGGAAAACCTCAGGCATATTTTGACAAAG
TTGATGAGACACTGAAACCACAGTCAAGTGTCTTACAAAAATTTGAAAACACCATATAGTCAGGGACTAAAA
TATGCTGAAGCATTCTAATCATCATCCTCAAGAACACTCCGACGCCTCTGGATAGTTACTTTTGTCTGCTTAGT
AGTTTGGGTGTTAATTTGTTCAAGTAATTGTATCAGTTTCTCCTCCGAAACCTTTTCCGTGATCTGCCCCATTTG
GGCAGCTCTGAGTACAACATCTTCAACTCCTCTGGCCTTTTCAGGCTTAACAAGAGCAATTCGAGCAAGCCTT
GCCCTTGCTTCAGAAGTCAGAATCTGAGTAAGCATCATTTGCCTTCGTTCATCAGCTTCCCTTTTGGCTTCCTCC
TGGGCGCTTTGCTTGTCTGAGCTTTGAGGAGTTCCCATGCCCTGTTGAGCCATGAGCTCCTGCATTCTCCTTTGC
CTGATTGCCTCTAATTCTGGGTCAGCCATCTTTCAATGCTGAATTTGAGCAGGGAATCTCAGGGAAAAGGAAT
GCAAGAAAATAAAAACACGGCGTTTGTCAGACGCGAGAAAAGGCCACCCTGG
>TCONS_00049186
TGACAGCATCTTGAGGGGAAACAAAATCAATGTAACCTTGGATATTGCGTCCAACTATGGAAGCCAGTTAAA
ATTGATTGATTTACAGAACAATTACATCGATTCATTTACAGAAAGACCTGGATATCGTTTTCAAATAATACTT
ATGCATAATCCAGTTTGTCAAGAAGGAGGATCAGAAAACTATTGTGGCAACCCCCAAGAGAACTTTGGATAT
TCAACCCCATAAGATAATTGCTTCAGAACTCAATGCAGTTCTGATCAAATTCCC
>TCONS_00049202
GGAATTTGATCAGAACTGCATTGAGTTCTGAAGCAATTATCTTATGGGGTTGAATATCCAAAGTTCTCTTGGG
GGTTGCCACAATAGTTTTCTGATCCTCCTTCTTGACAAACTGGATTATGCATAAGTATTATTTGAAAACGATAT
CCAGGTCTTTCTGTAAATGAATCGATGTAATTGTTCTGTAAATCAATCAATTTTAACTGGCTTCCATAGTTGGA
CGCAATATCCAAGGTTACATTGATTTTGTTTCCCCTCAAG
>TCONS_00049234
TTTCGCGATTCATAGGTAAATCGATTTTGATTCTCTATTAACCAATAATGTGGAACTATTAACATGGTTAAAAC
AAACTGTTTGAAGTCTAGACGCAGCATGGTACTCTTTCTACCACTATGTTAATATAGAGGTGGTTTCAAAATA
AATATTTTATCGATATAGGATACTCATATTGATAAAATGATTTGAACCGCGTAT
>TCONS_00049257
CCAGGGGTGGTCTGATAACATCCAGAAGTAGAGGAAGATGGTGGATTTGAACTTAAAGTTGCAGTTCCAGAA
AAGTCACAGGTTGCACCAGATGCAGCTTTCTTCTGATAATAGCTATTTACAGCATAGCTACAGTGATCTTTAA
TAGTGTTAGGGTTGTAACAAGGTCCATTTTGAAGAATACCAGTACAATCAGCTCCATTTCCACAAGCATAATC
TATACTCTTCTGCATAAGGCTATCTCCTACCCCATCTTTGCACAAACAGTAATTAGCACCACTTGAGTAACCA
GTGAAAGCCAAGAAAACTATTAAAGACAGTACAAAAGCAGTAGCC
>TCONS_00049294
GACATTCTTTAAACCAGGCATGTCAAGGTTGTGAAACTCCCTCAGTTTAGCAGCTATTGAAGCAGAGATCTCC
GGATCACGAAGATCCTCAGCAGATAATGTCCTTGAATGAATGAACTCCTCAACTCTACCATTTGGAAACTGA
CCAAGAAGTTTAGGTCCTTGACCTTTCTTTGACAAGCACTCAAAAGTTCTAATCTCCTCATCCCTATTGAAGAA
ACGCTCTACTCCTTCACCATACATGCGAACCAAGACT
>TCONS_00049409
AACAGCACCTGCACTAGTCTGATAAATAATTTGCATACAGTTTTCCCAAAATCCAAGTCGTTGGTCAGAGGAT
GCCCCATTGCTACTATTCTCTTGAGCAGCTGTTTTAAGAAATTTCTCTATCTCCCCGCGCCGGGAGAGGGGATT

TCTTGTATATAACCAGCATCCCCGCGCCGATATAATGGTTCCCAGCTGCAATGCAAGCACATATTATGGTCAG
CTATCAACCGCAAAACATATTAAGTATTCACTAAAAATTGAGCTGCAAGAGAAGACTGATATTATGTATCCA
TACAGCAAAATAGACCCCAACCAAAGCAAAC
>TCONS_00049410
ACAACAACCTCGAATTAGAGTACTTTTTAATTATATTCTGCTTCAAGTATGCTTCTTCTCCATTGCAATCCTGCC
ACTAAGAAATGCCTGTGGAAACATTCTCGAGAACAGTCGGCCTTTTGCTTTGATAAGCAGAGCATATATTCCG
TAACAGGGGATGTGGACCACAGCCAATCCAAGATACCACAAAGGTGCCCATGGAGTGTTAAGTTCCAAGAA
AGGATACGGCCACCAATTTAAGCAGCACGCGTGTAGAGTCCACTGGAAAACAATATAACAACAGCTCCACA
GCACAAAATATGTGATACCAAACCATGTAAATTG
>TCONS_00049480
TTGGATATTCTTTTTGGGTTGTTTGAGTAGAGTCTAGAGAAGTAATGAGCAATTTTCTTACAAAGGCTAATTGT
TTACTGATGCATTAAGTAACCATTGCTTCTTCATGGTCCTCGACTGGGCATTTTGATTAGTAGTAAACAGGGTA
TGTGTTTGAGGTGAGAAATTATCTTTAGCTAGAGAAAGTTGCCTGCTTGATAATCAAGTTTGAGCAGTGCTGT
ACCATTCTGTAAGCACTTAATAGGTCCAATATATGTTCCTCTGCTTTTCTGTATGTAATTGTTTTGTTGATTTGA
TTCTCTGAGTAACACAAGTACTTCTCTTAATGATAATTTTCAGGAAGAGCGAACCCGAGCACACTATAAGCA
GTTAATAGAGAAAGTGATTCCAGTTGTGGATAAGCGAAAACCACCTCGTGTCCTGCGAAGAGTTTGTTCCATG
CAGTGGTAATGTATCATAGTGCATCAGGCATGAGTTCAAAATGAATTCCTTCTGATATAAGTTCATAATAAAG
GAGCCTAATCGTGCTGGGGTAAGTGATATAACATCAAGTCGGTTAATTTAGCAGATGGTTATTCTTTCTGAGT
GGTAACACTTCCTTTGTGAAGGACAGTATTGTATAGTATCATGTCCTGGAAAGTCTATAACGGGATCTTCTATT
TATCTAGCGGAGGGAGTTCACTTAAGTTAGCACAACTATTTTTGTTGGTTGTTATAGGGGTTTTAGAATGCAAA
TGCCTGATTTGTTAGATTTGATATGAGTAAATCATGGGAATGAAATTCGGACTAACATTTTGTACTTGTTTGTC
AATGGCTCGTTATAGCTTGCACTCGCGAGAC
>TCONS_00049529
TGCAAATGAACTGCAGAAGCTGCTGGATCAGAGTGATAGGAAGAAGCCTAAACAGCACGGTGGCTTTAAAA
AATGACATGTCGAGTGTAAAACCTCTATCTAATGCTCAGCTTCTGGAAAAGCTGGGAGAGCTGCTTCTGAAA
ATGCTGCTGTTCTACCTGTTGAACAACTCAGTGCAGCAGAAATGGAGCGCTGGACTAAGTTACTGCTGAGAA
AGGCAAATGTAGTTCATTTTTACATGGTTCGGTGTTGCAGAAGATACTTCATAAGCAACTTGTCATTGGAGGT
GTTCTATCAGAAGCTGAATTTTTGGCTGCAAG
>TCONS_00049596
CTTTGTTCTTCTTGACGTTCTCCATCTCTTGACTAATGGTCCTAGACTGCGTACTAGGGACATAATGAAGTACT
ATTTAGACGAGCTCAGTTAAAAGCTCTTGTTTCTATCCACAGTCAAGAGGTCAGTGTTGTATCCTTAATTTGCA
GTACTAATAAGAGTGCAGAACCAGTAGTTTATTATTTCTAGTTCCTTTAGCATTTTAGAAACCATGATTGTTTA
ATGGGGAAGTTCTGCAGGCTGGCAAGGGAGGTGAACTTACACATGATGAAACAACAATCATTAGTGGAGCA
TTAGATTTAACTGAGAAGACTGCTGAGGAGGCCATGACACCCATTGAGTCAACATTTTCATTGGATGTCAATT
CAAAGCTGGACTGGGAAGCAATGGGTAAAATTCTTGCTCGGGGTCATAGCCGAGTTCTAGTCTACTCAGGCA
GTCCGAAGAATATTATTGGACTTCTAC
>TCONS_00049757
ATTATATGAACGAGTTCAATCTTTTATACATGATATTTTTTTCTAAATGTTGGCTTTATTTTTTTAGAAGAAGTT
CTGTATCCCAAGACGGGGAGAAGCTTATGTCCTAAAGTCACTAGGAAAGAAATGGAAAGATTACAAGTGCG
ATTTAAAAGGTGAGTATCTGCCAAAATATAAGACTAAAGACGCTTTGCTGAAAAATAGACCAAGTGCATAGC
GAGGGATCAATGGAGTGGTCTTGTCTCCTATTGGCTTTCAGATAAAGCTAAGGTCCTTGACGCCAACAGTGGC
CATAAACGAGTACCACAAACATCAAGGATACCTAGTGTTGCTGAAAATACTTCACTGTCTCAAGGT

>TCONS_00049895
CTGGCAATGATGGTGAAGGATAATATGACCAATTAATCCAGCATAAGCTGTTAGGAAGAAACTTAATGGGGT
CATGAGAGCAAAAATACATGACGTTCTTGTGAGACGGTGATACAAATATTCTTAGTCGCTTCATATGTCTGAA
GACTTCATTGCTCAGGTGAATGGTATTCTCCTCTGAGTTGGACATCAACGGGATTAGTATGACTTCTATCGCTT
CTCCGCCCTGTCAAAAATGGATCTTGCAATGGTGGTCTTGCCAATACCACCCATCCCTGAAATCCCGATAAAT
CGAACATCTTCACATTTCATCATCAACATCGAGCTTACAGCCCGAATTTGTGATTCTGCTCCAACTAGAGTGC
CACTGATCATTGACTTCTGGCACAGATCATTAG
>TCONS_00049921
ATAAAACAAGGGCTCAAGTCAACAGTAGACAACATCTCAGCTCTCTCCCCTAAACTCAAATTGGGAGACTTT
TGCCACAATAGATGAAGTAATTCCATTGGCATTCTTCCCAACAAGCAGTGCCCTTCAAGATTCAATGGAAATA
AAGATGTATCCAAATGATCTCTAGCAAGTTCAGCAAAGCTGATAAACAAGAGGACAAGAGAGCCAGCCCTT
ATTTCAGCTGAAAGTTTATCCATTTCGGGGAGAAAGAATTTTGCTTGAGCTCGATTTATTCCATCAACTCCAGT
GAAAGAAGTTGATATGCATGCCCGCCTAAATTGATAGACAGCAGAATG
>TCONS_00049950
TAGCAGATAAAACGGCCTTAGATTATTGCGTTAAACATATTGCACTTATCAGATCCCATTACAGAGGAAGTTC
ACCGCTGTCTGCAATGACAACTTTGCTTTTTGGTGTTCCACTTTGTCTTCCTTCAGCTTCAATCTTGTAAACGAC
ATCCATTCCAGACAACACCTTCCCAAAGACAACATGCCGGCCATCCAACCAGCTAGTTGTGACAGTTGTGAT
GAAGAATTGTGAACCATTGGTGTCGGGACCAGCATTTGCCATTGACAAAAGCCCTGGTCCAGTGTGCTTGATC
TTGAAATTTTCATCAGCAAATTTTTCACCATAAATGGATTCACCTCCACGCCCATCACCAAGAGTGAAATCAC
CACCTTGAAGCATGAAACTGGGGATTATTCTATGGAATGTGCTCCCCTTGTAATGAAGAGGCTTGCCACTCTT
TCCAACACCTTTTTCCCCTGTGCACAATGCTCTGAAGTTCTCTGCTGTTTTAGGAACTGTTTTACCAAAGAGAC
CCATAACAATACGACCGGCAGGTTTACCATCTATCTCAACGTCAAAGAAAACTTTGTGAGTTACTTCTTTCAG
ACTTTCCTGAGATTTGGCCTGCAGAGGATGCTTTCAAGTCAATAGTAATAAAAGACTTTCTCCCCCTATTTGTC
ACAAAGACAAGAGCACACATATGCACACACATGGACATTGGCCCCACATAAACCAGGAAAGAGGAATGCC
TAGGCCACCTTTTGAAATATGCCAAGTCATAAGTCCATGTTAGACCCAAAGGAAAATAGGAAAAAAAAG
>TCONS_00050362
TCTCTCTCTCATCTTTAGTCTCCCTTTCTCCTGAAAGAACAACCCTCGGTGAGTCTAGCACGGCCACCAGTCGG
CTGGCACAACACAGTCTGGCAGTTCCCGCACACCACCACAGTTTGCGAGTGACTGAACACAGTTGTTATGTTA
AAGCAACCCTGGCACTTAACATCCATAAAGAAAGAGTTAGGAGACTGAACAAGACGCTTGAGTTTGTGCTTC
CTCTTCTCCAGCTCGGCAGGAGGGTTCAACAAGTCGACATCATTTGG
>TCONS_00050367
TTCGAGAGTTGACCAGAAAACGCGGGAGTCCACGGGTATTTCGGTACCCGATTCGGACCCGACCCAATTAAT
TACCCATTTCCTTTCCTTCAGCATTCTACCAGAGGCAGACGCTCTTCTTCTTCTTCTGGTCTCAATAGTCTTTTTT
CACATATCACACACCACCAATAATTCCTACTCAAAAGTGCCCTAAAACCCTATAAGCTTTGTGTATACATAAA
TACGATCTTTATGTATTTATATTTTCTGGGTATTTTCTATTTATAGGAATTAGGCTTTCTTCTACAGGCAAACTCT
CAATGGATGATCATGATGACGATTGGGAAATTCTGCTCTCTGCTTTAAAGGATATGTCCTCTATGTGCTGTCAA
GGTGCGAACGAATATGGCTACACATGTCATCGTGCAACATGAAAGTATACTAAAAATATCCTTCACTTCCTTT
ATTCCAATTTATCAGCTTTACATAGTCAGGTTAAGTGTAGGAGTTATTGACTTTTCATAGCTGATTGTAAGAGA
ATAGCAAATCAAGTTTCGTAGTTGTTCTTTATAGTCAATTGGGAAGATACTATAGAAATTTCTGCGTTGAGTGA
GTCTGGATTAGTGAGCTTGATATTTGTTTATATAGTGAACTGGAATGCAGTAGCTTCTCAATTTTGCTCTGTGTG
TTGTCTAATTCATAACTTCTAACATCTTCATCAATCATTATGCTTAATGAAAGATAGTGGCAGATGCCTTCCTT
TTTATAATTAAATATTATTAAGAAAGCGACACTTCGGTACCGTTGCTGCTTCCAA

>TCONS_00050387
AAGAGGGAAACAAAGAAAGAGAAATAGCACGCTGAAGTCACTTTAATTGAAAGAATTGTGTTAAAGCATGT
TAACCGGACATATCTTTGCATAGGCAACCACAGCAGGGAGCAAGTCAACTACAAGGTGAACACTGCTTTAAC
TCCAACCAACAGCTTCTAGCTGCTAATAACTTAATCATCATATTTCGTGGAGCATCTTCAAAGAGCTTCTGTGC
CAGTATCCAAATAATGGAAAAGAAGCACTGATGTTCAGTTTCATAAATAGTCTTCGAGTATGGTGGAGAGCA
ATAGTCCTTTCACAAATTCAGATCTCTGGAACTTCTCTTCCTGATCCTTGTCCATTGAAGGAAACGGTTGGGAC
CTTCTTTCACTAACATGGTCATCTAGGCTGTTTACAGATGAACTTGCTTCAGCTGAAGTGCAGGATTGTCTTGT
ATGAAGTCTTTTGGAAGGCTGCTGATTGCCGACAAATGATAACAGTAACTGGTTGGTTGCCGTATTAGAGGAA
GGAGTGACATGGCTGGATTTCTTTTCCAAATAAGCACTCAGCAACTCTTTCCTCAGTAAGGATAAGGCTGAGA
AAGATCCACCATTTTGAAGTTTCTTTTTGCTGAGAGCTTAAGGTTTGTTAAGGTAAATCAAC
>TCONS_00050476
AGACTCCACCCCACTACTTTGGAAGCTTTAATGTGTGCTCGCACGTGGTTATGGAATGACTTAAATGGTTTGA
CTTCAACCGTTGATCAAGTTTCATGTCCAACATTGCTTGATGAAGAGGACGAGCCGGATTCAAGTGGTTTATC
GCAACTATGAGACCCTTGACATGCTTACATCCCTTTTGTTCCTCTCAAATTTGGATTGCCACATAGCTACTGTT
TCAATTTATAGTATCTGTTTTTATTGCTGTTGGTACTATTTGAATTTAAGTATGACTTCGGCCTA
>TCONS_00050620
ATTACAAATGCATACTGTTTCCACTACTAAAATCACAATCACTCTTCACAAGCAGATCTTAAAAGAACATGAC
AGTGTTCAAATCTTTCACTCATATACAGAAGAAAATCAAAGATGCAATTTTTACAAGATACTCGAAAAGAAT
ACACATTAATCAAGCCAGATAGCATACCGATTGGTCACATCCCGTGAATCAAGTCTTTGACAGACCAAATAT
GAGGACAAAAGTAGCAAGAACAGCGGCAATGGTGCATGTAATGTATCCTGGTTTCTCTCGAGGATCAATTTG
TGCACAAAGCAGGACAAAAGCAGCAAGATACCAAGGGATTCCAGCAAGAAAGAAGCCAATTATGAACAGG
AACCAGCCAAGACCAATACCACAGCAAGGAAGGCGATGCTCTCTTTCAGGTCTTCCCTCAGGATCAGAAACA
GTATAACCTGGAACAGCCTGATAACCATGAGCATAATATTCAGGTGATGATGCGGTGACGCCAGGTGGAGGA
ACTGGCTGAGGAAAACCGATCACCGGTTGA
>TCONS_00050667
GGGCACTAGGTTTCCTTTCTATTTGATCCGTGATTAGGAAGCTAAACTATATCATTGAGAGGAAAACACGAAC
TAAACACTGGGAAACTATATAAACTATCCTTTTCCTTTCATTTGTTTTTAATTGAATCAGAAAAAATAAACCAT
ATAGTTCAGCAGAGCTGGAACCAAATAAGTACAATGCTTTTACAAGAGTTGATGAGGTACCACCCTCTTGTG
CTCTTCTCCTTCTCCTTTTCAAAGACTTGTTTTCCGTATGCCTCCACGTTCTATATAAGAACCTTGTTCGTCTGTT
TAGCGTAAGAAGAAGAGGAAGGAACACGCTTGTTCCTAAACATCATATAAGCAAGCGGCAATACAACTCCG
ATCATCATTACAGCTGCTCCCATCATATACCTCATTGGGCTCGGCGCTTCCACTTCTAGTACTGTCATC
>TCONS_00050668
AACTATATCATTGAGAGGAAAACACGAACTAAACACTGGGAAACTATATAAACTATCCTTTTCCTTTCATTTG
TTTTTAATTGAATCAGAAAAAATAAACCATATAGTTCAGCAGAGCTGGAACCAAATAAGTACAATGCTTTTA
CAAGAGTTGATGAGGTACCACCCTCTTGTGCTCTTCTCCTTCTCCTTTTCAAAGACTTGTTTTCCGTATGCCTCC
ACGTTCTATATAAGAACCTTGTTCCTACGTCTGTTTAGCGTAAGAAGAAGAGGAAGGAACACGCTTGTTCCTA
AACATCATATAAGCAAGCGGCAATACAACTCCGATCATCATTACAGCTGCTCCCATCATATACCTCATTGGGC
TCGGCGCTTCCACTTCTAGTACTGTCATC
>TCONS_00050681
GCAGATGACAGTACTAGAAGTGGAAGCGCCGAGCCCAATGAGGTATATGATGGGAGCAGCTGTAATGATGA
TCGGAGTTGTATTGCCGCTTGCTTATATGATGTTTAGGAACAAGCGTGTTCCTTCCTCTTCTTCTTACGCTAAAC
AGACGTAGGAACAAGGTTCTTATATAGAACGTGGAGGCATACGGAAAACAAGTCTTTGAAAAGGAGAAGGA

GAAGAGCACAAGAGGGTGGTACCTCATCAACTCTTGTAAAAGCATTGTACTTATTTGGTTCCAGCTCTGCTGA
ACTATATGGTTTATTTTTCTGATTCAATTAAAAACAAATGAAAGGAAAAGGATAGTTTATATAGTTTCCCAGT
GTTTAGTTCGTGTTTTCCTCTCAATGATATAGTTTAGCTTCCTAATCACGGATCAAATAGAAAGGAAACCTAGT
GCCCC
>TCONS_00050689
CCCATATTGTCCATAAATTAACTTTTAAATACAAACATGCATAGAGCTCTTATTAAATGCGAAAAGACTCTCC
ATCTAATCAATTATCCATTTGTAATGAAGGACAAGTAAAGACAAGCCACACAATGCAATTTAAATAAATCAT
TTGACCTCCTTATCAGAAAATAATGTCCTCGTCTTTCTGTGAAGCATGGGATAGCAAGTTATGGGCTGTACCT
AGCGAAATACCTCTCTTCAAGGAAATTTCTTCTGCAGCCTTCTCCTCTGCATATTGCTTGGAAGCCAGCATCTG
TCTCTCTACCTCCCTCTTCTTGTAACCTTGTATCTTCTTGAGACGAAAGAAATCTTCCCTTTCCAGTTCATCAAG
TTCCCCTTTGATGTAAAGCACTGTGTTCTCCAACCGTGGCTTCACGACATTCTCCAATGCATTAACCCTCCGAT
TTGTGGTCTTGATCGCCTCATCAAGAGTCAAGAATGATGTCTGTAGAGAGGCAAGCTCAACAAGCAACTCAA
TAGATTTCACGTAAGCAGCACGGCAAGCTTGCACCTGTTGCCCCCCTCTAGCTAATCCAGTCAGGTCATTCTTT
GTCTCTCCTTCAGAGAAATGCTCGAATTTAGGGAGCTTTACACCGGCAATATTGTCTTGGCGGGATCGAACTT
TAATGGTTGCATTCTGGACATTTTCAAGGACAACATGCTTAATGTTCTCGCCAGCAGCATATTTTGCCTCTGTC
AGAGCGAAGGAGGAAGTTTTCATGACATCTCCCATTGATTCCTTCGTTGACACGATGTCCTTTAGAATCTGAC
GGAACTGCACAGTTAAAGCATCACTCTTCTTCTTGAGCAGAGCATGGCCTCTTGTAGCACCAACAAGGCGAG
CTTTGATCACACCAAGCATTGTAACTGTAGGAACGACATTCAAACGCTGGCTTTGCCCGGACATTTTGCCAAG
CTACTTTAGAAGTCGAATTCGTCGGAAAGGGAGAGAAAGATCGCGAGGATTTCAGACACGAATCAGATGGG
AGAG
>TCONS_00050718
CTCAATTTGTCTCTGCTGCTCCTTGCACGATCGAGCAACAATTTCATGCACCGTATCACTTGAATTGAGTTTCT
GGAGAAGGGCAGAGAGGTGCCTAGGTTGCTGATTCACAACTTGAAGTGGCCAACTATCCGGTCCAGCAGTTG
GCTGAGAAACACCATCTACAAATTCTGGGCTTCTATTAATCGCTTCTATAAAATTCTTGAAGTGATC
>TCONS_00050719
CCATAAAGATGCCACAAGAGAGACCATGAAGCTGCCTCGTCAAGCAAAATCCGTGCCTTCTGTCTCATCAAT
TTGTCCTCCATAACTCTGTGCATTTCATTAGAACCATACCTAATTGATTCTGAGACGTCACGGCAACAATTCTC
AAAATGTAATATCAAGTCAGGGATAGAAATTAACCCTACAGCACATAGAAACATAAACTGTTAGATATCAAT
TTTTACATTATTTTCC
>TCONS_00050726
CCATGAATGATGGAAAATAATGTAAAAATTGATATCTAACAGTTTATGTTTCTATGTGCTGTAGGGTTAATTTC
TATCCCTGACTTGATATTACATTTTGAGAATTGTTGCCGTGACGTCTCAGAATCAATTAGGTATGGTTCTAATG
AAATGCACAGAGTTATGGAGGACAAATTGATGAGACAGAAGGCACGGATTTTGCTTGACGAGGCAGCTTCAT
GGTCTCTCTTGTGGCATCTTTATGGAAAAGGAAATAAGTCGTCAACCTTTT
>TCONS_00050785
AAGATGCATAATAAAATGGTGAACTAATGATTGGGGCATATGTAAACCATTTTTCTCTCATCGTCTTCCAGAT
GCCACTGATTCAGTAGAGAAGAGGTACTTCACACGTGAAAGAACAGAGTCATGAGCAGGATCATATGGATG
GTCTTTTGAAGGAATCTCCAGAATGGCTGGAATTGGTTTATTGTAGCTATCAACCAAGAATCTGATCATGTTTG
CGACATATTGGCTGATTAACACTATTGCAATGTCCTCTCTTGTGGTGAATTCTTTAAAAGCATCTTCAATCTGC
TTCACTGTTGTCTTTGAATCGACAATAAGGTAGTTAGTTTTCCTCCTCAAATCGACATTGCCAACTCCGGCCAA
TAAAAATCCAGTGATTGTATCCTCATCAGCAATCATGGCAATGAGGGCTGAGTTACTTGTTTTGATTGGAGCT
CGGTTTGCCATCTTAACTGAA

>TCONS_00050812
ATTAGAAAATATAACCCACAAAAGCGCTTTAAATGAGTTTAAAAAGGCATCGCGGTAAGTTTAAGTCATCCA
CGTCGTCAATCATGTCAAATTACGAGAAGTAGAATATGCAGTAGTATAAACAAATGATAATACCAGCTACAA
GATCAGGAAGTGAAAAGACAGAAGCAGTGTTCTGCTTCTAAATATTTATGCACACTTCAGGCTGCTTCAGATT
GCACAATAAAATCAACAATTATTACCAAAATTTCACAATTTGGGTGATTTATTGCAACCTTGCATAATCCTTA
TTATCAACTTCTATGCCACCCAAAGCCCCTACAAATAAATATATCAATAGACACGTGCCTTAGGTGGGAATGT
CTCGACCTCGTCATCTAGAGTGTTCATATGAACTGGTCTGTACTCTAGCCGGACCTTCTCATTCTCCCAATATC
CTAGTGTATGTTTCATCCACTTCTCATCATCTCTTTTCGTAAAATCTTCACGAGCATGAGCTCCTCTGCTTTCTTT
TCTAGCCTCGGCTGAGTGCATGGTGATGCATGCATTGATCAAAAGGTTTTCCAGCTCAATAGTCTCTATAAGA
TCTGAGTTCCATATTAAACTTCTATCCTTCAACTGAACATCATGGAAACTCTCCCATGTTTTGTCAATGAGCTG
GCAACCTTCCTCTAGTGTTTCTTGTGTGCGGAACACAGCAGCATTGTTTTGCATAACTCTCTGCATATTCAGCC
GAATCTTTGATGTAGGCAGTGAACCATTTGAGTTCCTAATCTTGTCCAACCATGCAATGGTCCTCTCTCCCGCA
TCCTTCTCAAGTGGCTTCTGGTTCTCTCCTGGTCTTTGGATTTCAGCAACCCTATTAGCACATGCCCGCCCAAA
AACAACAATATCTAGAAGAGAATTTGCACCAAGACGATTGGCACCATGAACTGATGCACATGCTGCTTCACC
AGCAGCCATCAGTCCAGGAACCACGGCATCAGGATTATCTCCTTTAATAGTGACCACCTCTCCATGGTAATTT
GTGGGAATTCCTCCCATATTATAATGCACGGTAGGTAAAACGGGAATTGGCTCCTTAGTTACATCAACACCAG
CAAAAATTGCAGCTGTTTCAGAGATACCAGGAAGTCTCTCCTTAAGGACCTCTGGAGGTAAGTGATTGAGGT
GAAGATAGATATGATCCTTCAATGGTCCTACACCACGCCCTTCTCGAATTTCCATTGTCATAGATCTGGAAAC
AACATCTCTTGACGCAAGATCCTTGGCTGTTGGGGCATACCTTTCCATAAATCGCTCACCCTCACTATTCCTCA
GAATACCACCTTCACCACGAGATCCTTCAGTAATGAGACAACCAGCTCCATATATACCAGTTGGATGAAACT
GCACAAACTCAAGATCCTCAAGAGGAAGCCCAGCACGCGCGACCATAGCATTACCATCTCCGGTGCAAGTA
TGAGCAGATGTTGCAGAGAAGTAAGCTCGACCATAACCCCCTGTGGCCAAAATAGTAGAGGCAGCACGGAA
CCGATGCAAGGTACCATCCTCCATATTTAGTGCTATTACACCTTGGCAGCTCCCATCACTGTTCATTAGTAAAT
CCAAAGCAAAATATTCCACGAAAAACTGTGTATTATGCTTCATGGCCTGCCCATAAAGAGTATGCAATAGAG
CATGCCCAGTCCGATCGGCAGCACAAGCACAACGATATGCTTGTCCTCCTTTTCCAAAGTCTAGGCTTTGACC
ACCAAATGCACGTTGATATATTTTACCATCTTCAGTTCGAGAGAAAGGCAAACCATAATTCTCAAGCTCTATA
ACTGCTTTGGGCGCTTCTCTACACATGTATTGGATAGCATCCTGATCACCTAACCAATCACTTCCTTTAACTGT
ATCATACATGTGCCACCTCCAATCGTCCTCTGTCATATTGCCTAATGCTGCATTTATACCGCCCTGAGCTGCAA
CGGTGTGAGATCGAGTAGGAAAGAGCTTGGTTATGCAAGCAGTATTGAATCCATGCTCAGACAACCCTATTG
CCGCTCGAAGCCCTGCACCACCTGCACCTACCACCACTGCATCGTATGTGTGAT
>TCONS_00050967
GAGTACTGACTTATCAGAGTTAAACTGAGATGACAATCTAAAAGAACCAGGTCGTAGAGTCACTTGCTGCAG
CTCCATTCAGCCATGTCTGATGTACGTGTGTCCAAGTGAGTCCATCTGCGACAGTCTTATTCTTTGAACTTAAT
AGGACTGTAGTTATGCAACATCGCCTGTCTTCACCAGAGAGCTCCCACTTCTTGAAACTCACCAACCATGAGT
CTGAACCAACCTGTACAGGTAAAACTTGATCGACCCAAATACGAAATTGTTTCCCCTGTTTGTCGCCATGACA
TGTCCTGAATAAAGTTACACATTCCTGAAGGGATTTCTCAACACCAGATGGGTGGACAAAAGTAC
>TCONS_00050968
CACTGCTTTCAGGTTTGACAGGTAATGCTCAGAATGCTCAATTTCTCTACGCCTCCATTTCTCGAAAAACAGGT
AAAATTTGACAACTTCATAGGCAGGAACAAAGTTCTCCATCTTGCAGTCTGACATATCCATAACATCTCTAGG
GAAGGTACTTGGACCTAGGTTGAAATGACCAATAGCTTGTATGATGCCGGCAGCACACCTCTCTGTTGCATGA
ATTACTTTAGGATTATTCTTCGCATTTGCAGCATGCCATTGCAATAATTCCTCCTGTGCATTAGCAATCATTAC
ACCATACACATCCGGGATACTGAATAGCTCAGCATCATTCCCGGAGTCACCGCAGGCAAGGGTGTTGTTTGG

TAATTTTCCCTCACTCTTCAACTTCTTTTACAGATAAGCAAGTGCTTGTCCTTTCCCAGCACCTTGTGGTAATAT
ATCCAGATCCATCCCTCCACTGTAAATTATTTGACATCCAGCCCACGTTCTTCCAAGCGCTTGGAAAGAGTTT
TCATTACATCTTGTGCTTTGTCTTTCTGAACATAGAAACTGACCTTGTGTGGTCGCTGCTCTGTCTCTGACTGTA
GAGTGAGTTCAGGAAACTTGCTAGTCTCCTCTGTTACTATCTTTCTGTCCCACTTGTTATTTAGTAAAGCTTCCC
AACCATCATCAGGCACCATCGAGTTACCATATGTTATTTCAGTTCCCACGGACATAATAGTAATATCTGGGGT
TAGCATGGGCTTTTCTTTCCTCAACTCCTTGTAAAGTGTAGGTGATCTCCCAGTTGAGAACACTAACAAAGAG
TTATCACGATAATTGGCCTCCCATAAGGCATTAAATCTGAGCAGAGAAAGGTTCTCGGCATCATGATGATCTA
CCATTGTATGGTCTAGATCTGAGACTATCATGAGACGTGCAGCACTGGTTAGCCGATCCATGAGTGATGTAAA
TTAGCTTCGCTCTGAAA
>TCONS_00051173
TTACACTGGGGTCAAATGATTAAACAACCCAAAATAAGGTCTTTGGTTTACCATTGGGACCCGGGTTGAGGTA
CACGTGTTGAAGAGTTACTGGGCTAGAGGAAATATAGTTCTTATAATTATCAGATTGTGCGAGGGAGAAGGC
ATGCGTATTGTGAAGAAAAGTGTCTGCTTCTTCTCGTCCCCCTTTCTCTGAGTCTAAGTTCTCATCCCCTTCTTA
TCTT
>TCONS_00051223
CAATGAAAATTACAAAGGTAGTAATTTACTCAAACTTGGTCTGGCTGAGTTGCGTGTTTGGTCTTTGTGACCTT
TAAGCTGGCCTTATATTCACCATAGAAATAAGAGGCGAAACCCCAAAGACACATAGCTAAAGCCATCCACTT
TTCTGCATTGAAGCTTTCAGGTAAGAACATAACAGCAAAGATCTGTTGTACTGGAACTAGAAGGAGTTAAAT
ATCCCTGACAATAGAGCTGAGGAGCTGAAGATCAGCCCAAGATTTCCAATAATCATAATTTGCAAAGCTATT
GCTGATAGTGCTAATATCATATAATACTTGTTCTGCCCCAGTCCAAATTCTTCAGCCTCTCTATGGATGGCCTG
AAAATCTTCGTTCATAATCATGGCGACGACACAAAACAAAGTGGAGAAGATGCATATAAGAAACTGCATTT
GCATGACAAGATCAAACGTAACAGCAACTCCAGCTTTGATATGGCTATACTCCACCCTGCATTTACAAATCC
ATGAGAGCAGCACCAACAATTGTCATGACAAACCCTAAAACGTATTGACGATTCGACTCTCCTTTAGGCCTAT
CTCCATTCATGTGCAGCCCTGCTATTTGGCCAACTGCCATGAAGATGGAGTTGATGACAACAAGCAGTGTTTT
GATTGACATGAAATTCTTGA
>TCONS_00051224
CAATGAAAATTACAAAGGTAGTAATTTACTCAAACTTGGTCTGGCTGAGTTGCGTGTTTGGTCTTTGTGACCTT
TAAGCTGGCCTTATATTCACCATAGAAATAAGAGGCGAAACCCCAAAGACACATAGCTAAAGCCATCCACTT
TTCTGCATTGAAGCTTTCAGGTAAGAACATAACAGCAAAGATCTGTTGTACTGGAACTAGAAGGAGTTAAAT
ATCCCTGACAATAGAGCTGAGGAGCTGAAGATCAGCCCAAGATTTCCAATAATCATAATTTGCAAAGCTATT
GCTGATAGTGCTAATATCATATAATACTTGTTCTGCCCCAGTCCAAATTCTTCAGCCTCTCTATGGATGGCCTG
AAAATCTTCGTTCATAATCATGGCGACGACACAAAACAAAGTGGAGAAGATGCATATAAGAAACTGCATTT
GCATGACAAGATCAAACGTAACAGCAACTCCAGCTTTGATATGGCTATACTCCACCCTGCATTTACAAATCC
ATGAGAGCAGCACCAACAATTGTCATGACAAACCCTAAAACGTATTGACGATTCGACTCTCCTTTAGGCCTAT
CTCCATTCATGTGCAGCCCTAATATGATTGATCCAAATGTCATCAAAACCACAGCATTTATAGAATAATGTGT
GAACTTATGTTTCACCATAAAATACGCGAAAATCGCCGTGAAAGACAGTGATGTTGAAGCGAGGAGGGAGG
AAATTGAAACAGGAAGATAAGACATGCCGAAAGTGTAGAGGTAGCTACAAACACCAAGGAGACCTAAAAT
AGCACTGGCTAATATTAGTTTTGGTGTGAGTAAAACTCTATTAGTTTCTGTTTTAGCTTTTCTTCTTTTTGCATAA
GAAATGACAATTGGCAAAACCAAGATTGGAAACCCTGCTGTAAGCAAAAAAGCTTGCAACCATTTCCTTTTT
CCACCATGCAAAAAGTAAATCCTAACAAGCATAACACCTGCTATTTGGCCAACTGCCATGAAGATGGAGTTG
ATGACAACAAGCAGTGTTTTGATTGACATGAAATTCTTGA
>TCONS_00051229

TATCGTATTTAAGCAGTTGCTTGAGATTTTTCAGATTCCTGCTGTTGTTTCCACTGCTCTGGAGTTACAGCTTT
CGTAATAGAGAGAGCATGGATTTCCTTCATCTCCTCAGCTAGTGCACCATTAACCAGTCGGTGCCTCTCCAGC
AATCTTTTGCCTTCAAATTGTTCAGTCACAATGACAATCGAGAATTTTGCACCACATCCCCCAGATGTATCAAT
CACTTCCAGATGAGAAGGGTTTAGCTTTGCCGTTAATGAAGATTCAACCTGCTCCTTTGTCACACCCATGATC
CAACCCTCTCT
>TCONS_00051230
TGCACCTTAGAAATAGGCAGTTCAAGATTCATGAGGTCCTAATCAATTACATAAAACTTACGAAACTCCGGG
TACAATAGCAAATGCTAGAATGATCACAAGGATTTCAAAATTAGTAGTCTTTTGCTTCGAAAACCAGATTGTT
ACACAGACCACCTCAACCTTTCCCCAGTAATAGTCTATTGAGTCAACTGAAGCACCTTAAACAAAATATAGC
GTGATTGCAGATTTAGATACACACTTATGACCAGCACAGCTTTTCTCCTGATCAAACAGTCCGCAGTGGAGGA
GACACCAGTATTACACCATCTCCACGAACAAATAGGAAAGGGACGGTCCGTCTTGTCGTCCGAACTATCTCTT
CATATGTTTCATCATCAATCTCGACTGTGGTCACAATTTCTTCAACATCACCAAGAATCATATTAAGATGCTGA
TCATAAGCATGAAGTTTGCCGCGGAGTTCTCTATCGGAACGGAGTTTAACGTAGATTCTCTCGTCGAGACTGA
GCCTTATGAGATCCAATGGCTCCTTCACTGCACTCTCCTCTTCACTTCCCATTGC
>TCONS_00051245
CATGCTCTGTTTCAATAGTTTTTGGTCATGCACCATGGCTTCTCGATTTTTCCGCTCCTCCTCAAGCTGAATCTT
TACTTCCTTTAGCTGGCATTCTACACTTTCTCTTTTACGGCGTTCTTCTTCCACCTCTTTTTGTTGTGCTTGCAGTT
TCTCTTGAATTCCATTCATGGATCTCCTACTTGTTGAATTAACTCCCGATCCTTGACCACGGCAATAACTTGAC
GTTTCTCCAACAACTTTCACAAATGCAGCATTAACAATCGGGTCCAAATCAGTATCCTCTTCAATTTCTTGAAT
TTGTTCAGCAACATATTCATTCACCTTATCATTTACTTCAGCAGAGGCATCATTAACCCAATGTCCGTTCTTCA
TGTGAGTCAATTCCCAAAG
>TCONS_00051313
GAATTTAGCCAGGGAATCGAATGTGAGCTCTCTTCCATAGAAGGTAGAGTACAATGATTTAAACAGACTAGT
AGAGACAGCAAAAACAGGCAAGTGCTGAAGATCAGGGTCATAATCTAGACTATTGCAGGCCACATGATGGG
CGTTGTGTGTCCAATTCCACCAAGCAATACTAATCCCAGTAATGCAATTCCCTGCTAGAATTTGTACCAATTTG
TTGAAACCACGAGTTGTCATGATTAAATAATGACCAGAATCATGACCCAAGTAAGAAACCTGCATCCAAGCC
AACCCTAATAACCCCCCACAAAGCATACGAATCCAGAAACTATTACTATAAAGAACACCATAAACAGTCAA
GAAAAGCAAAAATGCTACAAAACATAAGGAATAAATCACCCCATGACCTTTCTCTTCAAACAAACCA
>TCONS_00051314
ATTAATTAGCCATGAGTATTAACAGCTTCCCAGAGCAGATTCCTAGCTTGCATTGCCGCTACCCTAAGTGTCC
TTATTGTCCACCTATTGGCCTCAAAGAAAGACAAACTCCTGTAGGGCAAATTGTGCTTCTTGCATAGATCCTG
TACAATAGGAGAAATTTTCCTCAATTGGCACCTAGGCAGCCTTGGGAAGAGATGATGCTCAAGCTGGAACTG
CAATCCTCCAAAGAACCAATCCATCTGAGGAGAACAAGCAATGTCAATAGTCCCGGCTGTTTGTTTCTCGAA
CCAATCGTTCCCCTTGGGTTGTCCAACATAGACATCTGCAGCAAAATGGTTCAGACAGAATTGAACGTGTTGA
ATCCCTGTCACAGCAAAGCTTATGAGGACAAATAACACCCTCTCTGTCCAATTAGGCAAGGTGGAAACAAGA
AGCGGAAACCAAGTCCAGAAAACCATGATCCCCAATATGTTCATAAGTCTATTACTCACTTTTCTCCTTGAGA
ACAATAGCAACAATGTCTGGATAAACAGATTCACCCTCGAAACACAGACAATCGGATAGAACGTAAAATGT
TGATAGCTGACGAAGAATTTAGCCAGGGAATCGAATGTGAGCTCTCTTCCATAGAAGGTAGAGTACAATGAT
TTAAACAGACTAGTAGAGACAGCAAAAACAGGCAAGTGCTGAAGATCAGGGTCATAATCTAGACTATTGCA
GGCCACATGATGGGCGTTGTGTGTCCAATTCCACCAAGCAATACTAATCCCAGTAATGCAATTCCCTGCTAGA
ATTTGTACCAATTTGTTGAAACCACGAGTTGTCATGATTAAATAATGACCAGAATCATGACCCAAGTAAGAA
ACCTGCATCCAAGCCAACCCTAATAACCCCCCACAAAGCATACGAATCCAGAAACTATTACTATAAAGAAC

ACCATAAACAGTCAAGAAAAGCAAAAATGCTACAAAACATAAGGAATAAATCACCCCATGACCTTTCTCTT
CAAACAAACCA
>TCONS_00051327
TGCGACTGCGACTGCGACTGCTGCCTTCTTCCTCAGGTTAGTGCTGCCTTCTTCTTCGGTTACAAGTATTTCTGT
TTTGAACGATTTGTTCTCTTGTTTTGAATACAAGCAGTGACATTTTCGGCTAAATCATTCTCTTGTTTTGAATAC
AAGTATTTTCGGCTACAAGCAGTGACATTTTCGGCTACAATTTTATCTTGTTTTTTCTATTTCTGTTGCTCTTTGT
TTTGAATGATTTATTCTCTGTTTGGA
>TCONS_00051434
CCCCAGAAAGGTCAGCGTTCCCTAAAAGACGGTTTCTATCATTAAGTCATACCATCCTCAGCTGTTGACATTA
GATATATCTTCTATTATCCCTTTACCATCCATGGCATCAAAGAAGAAAAAGTTCTTAAGAGGATCTCCTTTGCC
AGATATTGCTTTTATCACCTCCTGTCCAAGAATTCCTCCCATGATTGCGCAGACAGGAGGAAATTCGCTTCTA
CTTGCTATCAATCTTTGCAGAAGAGAATCTGGGATCTGAGATTCGTTTAAGCAATGTGCCTCACAGAGTTCCT
TTCTCATCTTCTGAACATTTGGAAGGTCATCAGCAGAAGTTTCTCCAGGATTCCGGCCCTCAAGCTCTTCAAAC
CTTTCTATAACTCTCATTGCAAAGTATAGCTTTGACATCCTCTTGGGTAGTGATCTCCAGGGGACTGCAATGGC
TTCCTCAAAACTTGGATACTGCAATGTGCATTCCATT
>TCONS_00051447
TTGAGTAATGCTACTTTTTTCAGGCTGCAATTGGGACAAATGGGAACAGAAGGTGAACAATTGACGGAGCAG
GAAACGGCCATTTATGATCGTCAAATTAGGGTTTGGGGTGTTGATGCTCAGAGAAGTTCTGCAAGAATATTGT
TTTAGCTGGAGTAGGTAGTTTGACGCTAAATGATGATCGCTTGGTAACAGAAGAGCTATTATTAGCTAATTTC
TTAATTCCTCGCGATGAAAATGTATTCCCTGGGAAGTCCATTGCTGAGCTTTGCTGTGATTCATTGAAGGATTT
CAACCCTATGGTTTCTGTTTCGGCGCTCTCTCCAATTTTGACGCTGATTTCTTTCAGAAGTTTGATGTAGTTGTT
GTTAGCTGTTGCTCCCTTTTGACGAAAAAATCAGTAAATGCTAAGTGTCACAAGTCGCCAAAGCGTGTTGCAT
TTTACAGCGTAGAATGCCGAGACTCCTGTGGTGAGATATTTGTTGACTTGCAGAATTACAGCTACTGTAAGGT
TATAACCAACTCATTTCTTTGTTTATTCCTCTTTATTCTGCTGGATTATATCCTTTTCTCTCTT
>TCONS_00051473
TGATTCCCCCCTTACCTTAGCCAAAATTTTCCACATTGTAGCAGAAATTACATCAAAAGGCTTCACTTTAGCTG
ATTGTTTAGGTCCAAAAATCTTGGAAATGTGTTGGTTTAGTTGCTTTTCTGTAATGTGAAAAGAATGTGATATC
ATTTTGCAAGTGTTGGTGATTCTCCAATGATCACCAACAGGATCAACCCTCTTTAGAGAAAATGGCAAATTCT
CAACTGTAGATAATAATGGATTGATGAATTTATTGGTTCTTGAGTTGTTAAGAGACTGAGGTGGGAGTTGATG
ACCAGCCATGATTTGTGCCCACATGTTGATAAAATTTGAAGCTGAAAATACATCTCCAAGAACATGAGCCCA
GCTTAGCCCTATTGACATCCCTCCACATTTGAACCAAGTGAACTGAATGAACACAAGAGGTGCAAAGCCCAA
CTCAGGACCAAGAACTTGATCATAAACAAGCTGATCATTAACAGAAGAATCATTCATAGCCAGCCACTCCTC
AATTGTTTTGCTACTCTTAGCTTCGATAATCCTAACACCACCGTCATTACACTTTATGAACGGCCGGCCACCGC
CGTCCTCCGACCTCCTAATCCTGCCGGAAGTCTCATAATATAGCTCAAGAAAATGAAACATGGGTTTTTTAAA
GTCATTTATATTCAAACCTTCAACTGCATCACTGTTGAAAAAATAAAGACCCTTAATGTAATGCAACTTCATG
ACTAAGTCCATGTTAGTAAGTTCATGAACTTTATCCGCTC
>TCONS_00051727
GATGAGATTAGTAATCTCGTCTTGAAACTACAAGCTTTGTTGCCAGATTCCTCTTCAAGATGCACTTCCGAGGT
ACCAGCCTCGAAGACATTGGAAGAGACGTGTAATTATATCAAAATGCTGCACAGTGAGGTTGATGATCTCAG
TGAGAAGCTCTCTCAAAAACTAGCTTCCACAGACTCCATATTAATGCCGTTGATCGAGACACTCAGAC
>TCONS_00051734

TGTTTCCCCGCATAGAAAGCACCTTGAACACAGTGGAATCCCTCTCTTTTTGAGATTGTCTTGGGTCAAGACA
ACCTCCTTTGCTAGCAGCCAAGTAAAACAAGCTACCTTATATGAAACTTTTACTTTCCAAATATGCTTCCAAG
GCCAATTAGTTAATTGCTGATTTGAATGGTTTAAGAGCTTATAGGCTGAACTTACCTTATACATACCTTTGTTG
TGTCCCTGCCACCATAGTGTATCCACCCCACTCTGTAACCCTTTAAAGTCTTCCAATTGTTTGTAAAACTTAGT
TAACCTATCCACCTCCCAATCATTTAACATCCTCCTAAATATAAGATCCCAACCAGTTATCTTTCCAAAAGGA
GGTTTTGTTGCCATTATTTACTCTGATGGAAGAATGACTCTTCATCACAGGCCAAAGTGACCTAATGGACTTCC
ATAAACTAACTCCATATGCTGTATTAACTACCTTTGATACCCACTTGTCTTCCTCACCATATTTAGCTTTGATTA
CCTTGCTCCATAAGGTTTCAATTTCCTGTGAGTACCTCCATAACCATTTCATCTTTAGGGCCTTGTTTTGGTTCT
TCAGGTTCCT
>TCONS_00051904
ACATTAGTCATTAATGTAGTGACAAGACGAGAACAATTTCAGTATGAGACAGGATAATCTGCTGTAACAGAC
GAGAGTGCGGGAGAAGTCAGTCTGCTGTCCAGCTCTGTTCAACAATCTCGCGCACCTTCCTGTTGTATTCACG
CTTGTTCTCACTGAACATGCGTGCTGCTTCTGAATTAGCTGGCGAGTTAGGATTTGGATCACAGAGCAAAGAC
TGGATTGAAGTCAGTATAGCAGCTACATCATATATGGGACTCCACTGATTTTGCAGGATGTCCAAGCAAATAC
TTCCATCAGCATAAATATTTGGGTGGAACATTCTGGAAATAAATCGCACAGTTGGTGGTTTGTTTGGATAGTC
CTCTGAGAATTGAAGTGTCAGCTTAAATGTACCTCCATCCCAGGGAGTATCATCAGGGCCGAAAATGACTGC
ATTCCATAGCATTATATTGTTGTCATACGGAGCTCCACTGATGCCGGCCGGGGGATCCTGCTGTAACCGCTTA
AAATCCCTCATCAGTCTCTTCCTCGCCGGTGTCGACATCCTCACCGCGAGCAAAAAGCGGCGAGAATTGAAT
GATAGACGTCGACGGGAGGTTAAATGATAGGCGGCGGCCTTATGTTCGTCGCCGGCTTAAAAGGCAATGGAG
ATTTTGCGTTGGCGTGATATA
>TCONS_00051905
ACATTAGTCATTAATGTAGTGACAAGACGAGAACAATTTCAGTATGAGACAGGATAATCTGCTGTAACAGAC
GAGAGTGCGGGAGAAGTCAGTCTGCTGTCCAGCTCTGTTCAACAATCTCGCGCACCTTCCTGTTGTATTCACG
CTTGTTCTCACTGAACATGCGTGCTGCTTCTGAATTAGCTGGCGAGTTAGGATTTGGATCACAGAGCAAAGAC
TGGATTGAAGTCAGTATAGCAGCTACATCATATATGGGACTCCACTGATTTTGCAGGATGTCCAAGCAAATAC
TTCCATCAGCATAAATATTTGGGTGGAACATTCTGGAAATAAATCGCACAGTTGGTGGTTTGTTTGGATAGTC
CTCTGAGAATTGAAGTGTCAGCTTAAATGTACCTCCATCCCAGGGAGTATCATCAGGGCCGAAAATGACTGC
ATTCCATAGCATTATATTGTTGTCATACGGAGCTCCACTGATGCCGGCCGGGGGATCCTGCTGTAACCGCTTA
AAATCCCTCATCAGTCTCTTCCTCGCCGGTGTCGACATCCTACGAGCAAAAAGCGGCGAGAATTGAATGATA
GACGTCGACGGGAGGTTAAATGATAGGCGGCGGCCTTATGTTCGTCGCCGGCTTAAAAGGCAATGGAGATTT
TGCGTTGGCGTGATATA
>TCONS_00051906
ACATTAGTCATTAATGTAGTGACAAGACGAGAACAATTTCAGTATGAGACAGGATAATCTGCTGTAACAGAC
GAGAGTGCGGGAGAAGTCAGTCTGCTGTCCAGCTCTGTTCAACAATCTCGCGCACCTTCCTGTTGTATTCACG
CTTGTTCTCACTGAACATGCGTGCTGCTTCTGAATTAGCTGGCGAGTTAGGATTTGGATCACAGAGCAAAGAC
TGGATTGAAGTCAGTATAGCAGCTACATCATATATGGGACTCCACTGATTTTGCAGGATGTCCAAGCAAATAC
TTCCATCAGCATAAATATTTGGGTGGAACATTCTGGAAATAAATCGCACAGTTGGTGGTTTGTTTGGATAGTC
CTCTGAGAATTGAAGTGTCAGCTTAAATGTACCTCCATCCCAGGGAGTATCATCAGGGCCGAAAATGACTGC
ATTCCATAGCATTATATTGTTGTCATACGGAGCTCCACTGATGCCGGCCGGGGGATCCTGCTGTAACCGCTTA
AAATCCCTCATCAGTCTCTTCCTCGCCGGTGTCGACATCCTCACCGCATAAGAAATACGGAATTGTTGACCTA
CGAGCAAAAAGCGGCGAGAATTGAATGATAGACGTCGACGGGAGGTTAAATGATAGGCGGCGGCCTTATGT
TCGTCGCCGGCTTAAAAGGCAATGGAGATTTTGCGTTGGCGTGATATA

>TCONS_00052010
GCATCTATCGATCCTAGAAACGCGGCAACTCTCACTCATCAATAGAGCAGAAGGGCTGTCAAACTGCTATTC
AAAGAACATCTCTTCATCACCGGAATAGTTGCTGACATCTGAAAATTCATAGTAATAATCGTCTTCTGAGATG
AGATCGATGTCAGAAAATCCAGATGGGTAATCCTCATCAAATGATTCACAATCATGCATTCCTGCATCAAGTT
CATAATCTTCGGTGGAGTCATGAGGGCGTCTGAGATATATGATCTGTTGAGAACATCGCTTCCCTACATCGCC
TCCTAGATTGACATTAAAACATCGTCTCAGATCAAGTGATTCAAGGTTAGGGCAACCATTAAGAATAGCTTCC
AACCCTTCATTTGTCATCTTATTTCCAACAAGTTGAAGGTTGCGCAGTTTAGGCATGTTCTCTGCAATTCCAAT
TGCCTCTTCATCGTATTCAATGCAGGGATGTCTAAATACCTGGTTGTTCAACTTAAATGACTTCAAAGCACGA
CAAGAGCGGCCAATAGTTTCAATGGATTCTTTGCTGATTAAGATGTGGTAAAGGTGCAGTTCCTCCAATAATG
GACACTTCTTCACTGCCGCACTCAACCCCTCACCTGAGACATTATAGGAGCATAGGAGTCTAAGACGTTTGAG
TTGAGGTGACCCGACATGACAGAAAATCTCTTGACCTGAACAGCAAAAAACTAGTTCAGAAAACACTCGCTT
TAATAGATGTTTGTTATTGTGCAAACCTTTCAGCAATATAATAGAGCAAACTATCGCTGCCAAAGTACTCAAG
ATTGATATCAATTAATTGACCCTGGCTACGATCCACTGCTTCACGGCATATCTGCTCCAAGTCACCTTCCAAC
ATGTCCCAGACATTGTCTCGCATGTTGATAACTCGCCACATGGCTGGGTCTTGGCATAATCGCCGCCAAGTTG
TACAGACTTTATCTGCATTTCTCAGTATCTCTATCGTCCCCAGCTTCTGTAGTATCGTTGATGTAATCTCCGGCG
GTAACTCTAGCCACGGCGGTGCGAATAGATTTTCCATGTCTACTTCCATTTTGG
>TCONS_00052090
TTATCTGTGTTTTCTCAAAACAAAAGTTGTGAATCTGGTTGTGTTTGAATTTGCCCAAGTTTTCCATCTCGCTCG
TTGAGAATCGTTGGTTGCTTTGTTTCGCTGATTGCTTCGCTTATTCGAGTTCTTCGATCTTCTTCTTCTTCCCTAA
CATAAAAATTTTGGTAAGGTTTCACATTTTGATATTTTTTCTTTTGCAAAATCAGTGAAAAGATTATTGGGTTG
GGATTCCTAATTTGATAATTTAATTTTCTGAAGTTTATTTAAAATTGCGATTCGGTATTCTCAGCGGCAGATTTT
A
>TCONS_00052101
GTAACGTTTCTACTACAATTGCTATGGCAGTCATCCACTTTCCAAATTCGAGATGCATATTCATAGTCAGGATG
TAAACTTATATGCCTTTTGGTGTCGTTTGTGACTTGTAGAGCAGCATTGAAAGAGAAGAAAACAGAGTTAATC
ACAGGCCCAAAAGCAAGCTGTCCCATCAACAGTTTCTTCAACGTACTAACGATATCCCGATTTGGCAATACC
CTCCCCACAAAGTTAAACCAATAATGTTGAGCTGTTCCTAAAATTAATAGTCCAAAG
>TCONS_00052138
AGGCACAACAAACCTGAGCAAGAATATCATTCATAGCATCACTGGCATCGGCATCATCTTGTCCCAAAGCAC
ACAAGAGCCTAAGCAATCTTATATGGAGGAAAGGATCTGTGATACCAGAAACGTCATACTCGGGTGCATATG
GACTGTTCGTGAGATCCTTCAGAATTTTGACTAAACCATCTGTGCATTTCTTTCTGAAATATTCAAGAGCCTCT
GTACTAATTTTACATAGATCTATACAAAGCTGGACGCCTGTAATGAGAACTCCATGGTGTTTCTCACTTAATA
AGGCTGCTGCAGGGTTTATAAAATTTTCGGCGAGATCGGG
>TCONS_00052198
CAATGTGTAGGTGATATTGCTGCCACAATGGGAGACACTTGGTAACCTTCACTCCATTGTGGGAAGTTCTCTC
CAGATCTGGTTGAAAAACAGAGACTAGTAAACTCTGGTGTCAATATGATGTTAATAGCAGGTAGCAGCTGCA
CCATGGATTCTTGTAGCATATCAACCATCTGCCGGAGACAGCTGACACCTCCAATCAGTTTGTTTTGGTTAATA
ACAGAAGAACCAATATAAAGAGCGACAATTCAACCACAAACTTCGGGCTCGATACACGTTCAAACAACTGC
TGCCACCTGTAAGTTCAACCAGTAGAGAGCTGCTGGTAAAGCAAATTTACTTCTCCATCCATGTAATCTCCAT
TAACAATTTCCTCCTGTGCAAATTCTTCCATTATAGTACGCTGTCGTTCATTCAACACTGTCGGGAAGTCAATG
CGGAACCGCACATATTGATCTCCATGGTTCACCAGAAACCCACTCTTTGGCAAACCTTTGCCTCGTAGTACCA
CAAGTTGTCCAGGCTGAACCCCCTTGGGTATCTGTATCTGTGTCTTTCCGGATAGAGTTGGTACCTCAACTTTA

CCACCAAGAATAGCTTTTGTAAAGCTGATATGATAATCCACATAAAGGTCTGCTCCATCTCTAGCAAAAACA
GGATCTTTAGCAACCTTTAGCTTTATAAATAGGCTGCCTGGTTGCATCCCTCGCTTGCCAGCATGACCAGCTTT
AGGCACACGAATAGTATCACCAGAGTCAAC
>TCONS_00052224
GGATCATGGTTCGATTTAAGAATCGAGACGATAAAAAGGAAGGTAAATATGGATGCTTATTGATGTTTATAG
ACAACTTGGGCTTTAGGTGAAGCACGCTGAAAATTTGTTAGCTCAGCAAGTATTACAGTTCCAGAATCTTCAA
CTAGACATGGTTGGATTAGCATTCTCACCAGCACTGGAGACTTGGCTAACAGAAGCTTCATAAGCTGCATCTC
ACGGATTGTGCCATTAGTCCGTATTAGCTTAACTTCCCTGAGGTGATTAAATGACACATCCGAGAATTGTTCT
ACTTCAAGACATTCTAGAGCAGGTATATCGTTGCCATCACCGGCCTCCACCTTAATTTCCACATATTGTAAAC
ATGGGAAGCTTCTTAGCAAGCAAAGAGCACATGAAACCTCTTCCAAGACAAACAGACAAATACTTGATATG
CAAAGATGTTTGACAAAGTTAAGATTAAAGGGAAGCCTTGTTGGTACTTCACCTGCTCCTGCAAAAAAGAAC
GTGTCATTCAGATGGAGATGCTCGAGAGCAATAAGAGGCTCAAAAAACTTGGCAATATTACATTTTTCTTCCA
CATAATTTTCCTGTGTAAGTAAAAGTTTTGCCAGAAGAGGGATACTCTTAAAGCAAATACATCTTATATTGCC
TGTGAAGTCAAAGGATCTCAGCATCGGAGCATTAATTTCAATGATGTCACTTAAACCTGAGATTTGCAGCACC
AATTGCTCGAGCAACGGGGAATGAGAGATTAAACTTTCAAGCAACTTGGAAGAAATTGAAACGTTATATAAT
TTCAGGCTAGTTAACCTATCAAATCCTTTGAAGGTTGGAGGAGGAAGTATTAAACAATTTTTTAGAGTCAGAT
GCCTTAACTGCAAACATCCGAAAAATGAAGAAGGCAATTTGTACTGGTTACCCCTCGGAAGTCTTAGAACAA
GATGTTGAATGACATTCCTAGAGAGGAAATACATCAAGTTATCAAGATTAGGACAACCTTCCAAGCCAGGAA
TGCATAGAGTAAACTTCGTAATTGGTCCTACATGAAGGGTCAAAATGTGGTAGATAATCTTTGTGAAGTTACT
TGTAATGTATGTTAGATCCTTTTTAGTTTTCCAAAGTGCTTGATCAAGCGCCAACTGTGGAAGTCTACACCAGT
TATATCTCCATTTCTTCGCCAAGATGCTTGTCCTCACAGCATCTCGTAAAGGCAAACGGATAAGAATTTCATC
AACTACATTCTCGGGAAGATTGTTAAGTACATCAGGGGGTAAAGTTCGAACACAA
>TCONS_00052283
ATTGTAATAATGCCAACTATACTATCCGTAGCCACTTGCTACTGAAAGGTGAGACGAACGCTGAAATATCCT
AGGTACTTGAAACCTTAGCCATGGCCATCATTTCCTGAACTAAATCCTCAATGAACTTGCATTGTATATCCTTG
TTCAACAGAAACTCCTTCCATTTCTTCTGATTTTCCCTAATTAATTTACCTGGCTGCTTGTCAATCTCCACCCCA
GCTTTCATATCCCCACTCACCAGCTTTGCATTCAGAAACTGGTCACCTTTCTGGGGCAACATAACCACTTGAC
AGTCATTCACTAGTGCCTCTATCACTGAACTGAACCCTGCATGACACACATAGCAACCTACACTAGAGTGAG
CTAATATATGCTGTTGTTGCACCCAACCTGAATGAATAATTCCCTTGTCTTTAACTCTTTCTAGAAACCCTTCTG
GTAAAGCTCGGTTTAACTCGACTGAGAAATCGACATTGGCAGGAAAATTTAGGACCAGAAAGAAAGGTAGC
CCAGTTTGCTCCAGACCTAAAGCCAGTTCTTTGATCTGTTCATCCTTCAAGAAAGTTTCACTTCCAAAAGAAC
AGTAAATGACTGTTCCAGCTTCAAACTTGTTTAACCAACTAGACCATCTCTCTTCCAATTTACCAGAAGGTGG
ATCAGGGACTACTGGTCCTACAAGAAAAACAGGTTTATTGAATTGTGCTTTCACATATTCTATATAAGGGCCC
TCCATTTGAGAACAAGTCTTGGCTAGTATAGCAGAGCAACCCTTGAGTCCTGAGAGTACACGGTCATATACA
GTAGGACCACCATGGAAGCTCTTGAAAATGTATAGAAAATCTCGAGCCTCAAAGGTTTTAACTGAGGTGATA
GAGGTGTTAGGAAATCCAGGTGGAGGTTTTTTCATGTCTTCCAGAGTTGGATATTTTTTGGGTTCAGGAACTCT
AGCAGGACAAGTAAGAAAAGCAGTGGAAAGTGCAACAAAAACAGAGTAAAAGACAGTCTTGATCCCTAAT
TCATCAGCCATTTTTGGGAGCCATTCTTGAGCAAAATCAAAAAGAACAAAATGGGGTTTGAGATGGGTGAGT
AGAGTCCTGATTTGTGGTTGCATAAGGTCTAAACCAACCTTGAGAAGTTCAGCAGTTACTGGTGTCAACTCAG
CAGTACTTTCTGCACCAGGAGGTAGACCTTCAACTTGGGGAATAGTGAGAGGGACTATATGAG
>TCONS_00052494

CTTATAGACTGACTACGTTTTGCGATACTTTTGCTATACGTTTCATGTTTTCTTTTCATCATAGGAGTATAATTTT
GTTCAACTTGCTGATCAGAATCGTTTTTCGTTTTGCTTTTTCAATTTTCTTTTGGAACTTTTCTCAGATCATAAGT
TTTTAGAAGATACAATATGTACTCACTCCCAGCATTGCGGAAATGCCGTTGCTTCGTGTTTCAGCATCTTGAAG
ACATGAAGCATAGTGTCCGACTTCTGTTTTTCCCTTCTGCAATTGCAAAGAAGGATTAGAGATTGATCTCCAA
ATTCTGAACAGATGACATGACAAAGACAGTTGAATGGAGTTGAGAGTGATGGCTCGCAAAACCTTTACAAA
GTGAATGGTACTGTCTTTATGGTAGGTCTCTGAAGTTTGTTTTGCTCCTTTTACTCTAATTTCATGTACCAATCA
TCAAGGTATGGAT
>TCONS_00052495
GAACTATATATGAGATAAGGAAGTTTCATGTTCCTAAGGTTTCGTATTTACTACATGATATAATCACTAAGGA
TATTTTCCATATTATTCTTATTGATCCTTGTAACTTTCATTGATAATATAATCATTACCAAATTCTCTACTATTTA
ATTATTCTGTTAACTTTGAACATCTTTTTTTTCTTATTATAGTGGTGTCCGGGTCAATTTGCTCGCACCTTGACTA
TCCCACCGAAAAGCCTGCTGCAGCCCACCGTATACGTGTCGCGAAATCAGCTCACCATGGTGGGGCAGATAG
GCTTATACCGAGTGTTATAGCTTAGCTTTGGACCTGAGATCTCAGAAGTTGGAGTTGCCAGTTGGTGATTCTCC
TCCTTTGTTTTGTTGTATCTTTCAATCACCGGCTTCATGCTGTCAAAGTAAATATATTCAGAATTGTCTACGTTG
TACTGATTGAGCAAATATTT
>TCONS_00052499
GATCATAAGTTTTTAGAAGATACAATATGTACTCACTCCCAGCATTGCGGAAATGCCGTTGCTTCGTGTTTCA
GTTGAATGCTGAAATTTGACATTGGTTTGTAATGAACTTAGTTTGTCCTTTCTGAAGACTATTGCATGCAAAGC
ATATTGATGCAACTTTGAGCATCTTGAAGACATGAAGCATAGTGTCCGACTTCTGTTTTTCCCTTCTGCAATTG
CAAAGAAGGATTAGAGATTGATCTCCAAATTCTGAACAGATGACATGACAAAGACAGGTTATAGAGTTGGAT
ACCTTGAGTGAAGCAGTGGTACCTAGAACCTAGTGCTATCCATGAATGTCATTATCTGCTTAATAACAACAAG
AGGGACTGTGTAGAAATAAGAAGATAAGGTGCAGATTAACACCAACTGTCATCTTGGTAGAAAGTTTAGGAC
CGCTATCGTCTTCTCTTTTTTCAAAAAAGGTTTGACTCGTCTACAAAATAGTACCACATTCAAGTTTGACAAGT
TAGGAGATGAGTGTGCCCTAGCTGACCATCATCA
>TCONS_00052500
CAGTGCATCTTCATTCATCTTCTCCTCAAATTGTCCGATTAGCTTTTCGATCACTCACAACACTATGAAATTGC
AATTTTTTTGGCCATTAATGGCGGTGGTTCATTCACCGCTTTTCTGTTTTGTTTCTAAGTAATATAGAAAAAATC
TTTTTTTAGTATCATATGCTAAACGCAATTTTCATCTTTGCTTATAGACTGACTACGTTTTGCGATACTTTTGCTA
TACGTTTCATGTTTTCTTTTCATCATAGGAGTATAATTTTGTTCAACTTGCTGATCAGAATCGTTTTTCGTTTTGC
TTTTTCAATTTTCTTTTGGAACTTTTCTCAGATCATAAGTTTTTAGAAGATACAATATGTACTCACTCCCAGCAT
TGCGGAAATGCCGTTGCTTCGTGTTTCAGCATCTTGAAGACATGAAGCATAGTGTCCGACTTCTGTTTTTCCCT
TCTGCAATTGCAAAGAAGGATTAGAGATTGATCTCCAAATTCTGAACAGATGACATGACAAAGACAGGTTAT
AGAGTTGGATACCTTGAGTGAAGCAGTGGTACCTAGAACCTAGTGCTATCCATGAATGTCATTATCTGCTTAA
TAACAACAAGAGGGACTGTGTAGAAATAAGAAGATAAGGTGCAGATTAACACCAACTGTCATCTTGGTAGA
AAGTTTAGGACCGCTATCGTCTTCTCTTTTTTCAAAAAAGGTTTGACTCGTCTACAAAATAGTACCACATTCAA
GTTTGACAAGTTAGGAGATGAGTGTGCCCTAGCTGACCATCATCA
>TCONS_00052605
AAAAACAACCGATAAATAGTATATGTAAAGATGTACCCTGTTAAAAGGGAGGAACTTGTATATGAAATTACA
TTTATTTTTCTTTCAATATTTTTAGGCCCAAAACAGGCTATAAAAATGCATTTCAAGTCTATCTTTTTCAATGGA
AAAGACAAAGAATAATGATGTTAATCCATGTCAAACTTCACTTTGGATCGCTAAAGAAGCCGTTGATATTTG
GCATAATCTCTGGAGACTTGTTTCGAACAAACTTCCTAAGAGCATGCTCAGCATATTTCTCCCCTTCTGAAAA
ATTGTCCAGCACCCCTGCTGCCCGGTTTTCCTTTGTCACCCTGACTTCAGGGTTGCCGCAGATATTACGAACCA

ATTGCATTCCACAGATTCCTACAGCAACGCCTACGGCTGCAAAAAGTGGATACACCTCGGGCCTCAGCCAAC
GGTTAAGGGTGGAGGAAGCCATAGAAAAACGGGG
>TCONS_00052664
CGATTCTGTATCAGGCTCAAATAAGCTCCATGACTGCAAATTTATTCAGCAGGCCGCTGTCCCAAAACTGAAA
TATCCTCATAACGCTTCCCAACATTGTTCATCTCCCAGAGCTTATGAACCCCGTAATCCACCGCCCTTTCGCCG
AGGAGAGCGCCAGCGATGACAAAGGTAACGTAAACAGAGTTACGGCGCATAATAAGTCTGTAAAATCCTTC
CATAACCCCGCCGCCGCTTCTTCTAGCTGATGTTCCCTCCATTT
>TCONS_00052752
CAGATTCGAGAACAAATACACATTCTCGTGATTTGTATCCAAGGGTCACTTAGACATTGAAAAATTGGATTAT
GAAATTGCGAAACATAATTTTGGAATTGGATCAATACTTCCAATTGAATAAGTATGAATAAAGGATCCATGG
ATGAAGATAGAAAGTTGATTTCTAATCGTAACTAAATCTTCAATTTCTTATTTGTAAAGAAGAAATTGAAGCA
AAATAGCTATTAAACGATGACTTTGGTTTACTAGAGACATCAACATATTGTTTTAGCTCGGTGGAAACAAAAT
CCTTTTCCTCAGGATCCTATTAAATAGAAATAGAGAACGAAATAACTAGAAAGGTTGTTAGAATCCCCCTCTT
CTAGAAGGATCATCTACAAAGCTATTCGTTTTATCTGTATTCAGACCAAAAGCTGACATAGATGTTATGGGTA
GAATTCTTTTTTTTTCGAATTTTGTTCACATCTTAGATCTATAAATTGACTCATCTCCATAAAGGAGCCGAATG
AAACCAAATTTCATGTTCGGTTTTGAATTAGAGACGTTAAAAATAATGAATCGACGTCGACTATAACCCCTAG
CCTTCCAAGCTAACGATGCGGGTTCGATTCCCGCTACCCGCTCTATATCTATTTATTCTAAATATTTTAATCTAT
TCATTAAATCAAATTTAGTTTATTAGTATTAGTACATCATTGAATATACAATTCCAAAAATTCTTTCACATCCG
ATTCTTTCTGTTTTTTTTTTCAAACAAAAAGTTAAAATACGAAAAAAAATCAGAATGAAAAGCGTCCATTGTC
TAATGGATAGGACAGAGGTCTTCTAAACCTTTGGTATAGGTTCAAATCCTATTGGAC
>TCONS_00052764
ATCCTTAACCAACGCCTTCCTTGATTCACTCCTTTGCTCTGGGAGCACGAGCAGCACGGGTTCATGTTTGCGCT
GACGGTGGAGCGAATAGGGTGTTTGATGAATTGCCCCGCTTTTTTCCTCATGACGACCCTTCTGATATTCGTAA
GAGGTACAAGCCATATGCTATAAAAGGAGATATGGATTCAATCAGAACTGATGTTTTTGACTTTTATAGAGGC
CCGGTAAGCTAGAGTTTGCTTGTGATGGTTCTGTAGTTTCTTAGCAAATCTGTTTTCCAATAAATTTTAAAAGC
ATCCACATTTTGATATTTGTGTAGGGATAAGTGTAAAAGAACATTTAGTTTTGGAGAATAACTAATTTGCCTTA
AAAGACAGAATATTTAGTGTTGAAATGAAATGGAACCTGAATGGTGCGGCAGAATAGATACATTGGATTGAT
GTAGCCGATCTCAATTAATTTGGATTGAGG
>TCONS_00052801
ACCTCTTATATAAAACTGATCACACATCTGCCTGAAGGAATAGTGGTCTCCAGTACCAGTATCATAATTGTCC
TCAAACACAAGATGCCTGAACCCAGCTTTAAGTGCTTGCTTCAGTCTTTTCAATTCATTTTGATGGTCATCAAA
AAAGACAAGAACCTGGCTAAGATCCTTGACTCCGTGTTTCTTCATAACTTTTCTCCAATCAACGCTTCCAAAA
TCAACAAAATCTTTTCCAGCAAAGTATGTGCAGTTCCCATCAACATAAGCAGGTCCTTTCTTGAGATACTTCTC
AGGATGACGGGGTGTAAGTGAGATGATTGGTGTATCTGGCATTGCTTGTCTCAAAACCCAAGTTGAGTGTCCC
TTGAATGCGCCACTCTCAATCATTAATTCTGGTTTTAACCACCGAGTCATAAACCAGAGCCCAAAGCTGTGGT
CAAAACCCATCCCATACATGTTATTCTTGATAGGTCGTGTCTCATATATGGGCACAAACTCCTCCAAGCCTTTG
ATTAAATCCTTTGTGGTCCATTCAACAGTTTTTCCATGTTTCGATCTGCACCAGGGAACGCCGAGAGATCCGA
AGTCGGTTTCAAGGCCATCAAAGCCGAAGAATCGCAAACGAGAAATGTGATCGGAAGAGGAAGCTGAGATA
CACACTAAATCACGAGTGTGATAAACGAAAGAAGTGAGCACAATGATTACGAGCCCTATGAGAAGGCATGG
CCAGAGGTGGCCGGTTCGAATCAAGTAATTTGTGATCTTCGTCGGTACGGAAATGTGCTTCCGTGTTTTGGATT
GGTCGCCGTCGTCGTCTCCGTCGTCACTGAACGGCGGCGCCCTGCGAGAAGAGAGCGCCCTTTCTAGCATCGT
CCTCGTTTATGCTTTC

>TCONS_00052862
AAAAACTCTCCTTTTATGTGGATCTCTTAATTCATTTGTCAGCTTTTGCAGATACGCATTTAGATGTCATCCAA
AGAGTAAAGATGGGAGACGTCATCTTGTAACAGTTAATGACATTGCGTTTAACCCATCCATTATTGGTTCATT
TGTCACTGGTGACAATGACGGCTATGTCACCTTGTGGGATGCTCGGGGAAGGAAACGGATGTTTGAGATGCC
AAGGTATCCCAAGAGTGTTGTCT
>TCONS_00052877
ATTTCTGGATATTTATTATTGGTGAAGCATTAGCAGTGTTATCAGATAGGTTAATTTTTGTCTTTATTCAGTTGC
CTTCTTCTCCAGATATTGTATTTGCCAGAAATTGCCTTTTAAGTAAAAATATTACATGATCAATGTCTTATGTG
ATTATCCTGTTTGTTTTACAGGTGACAAAGAGATGAAGTTTCTCTGAATTGGTCTTGTACATGGAGGAGAGCC
ATGGCATTTGCAAGCTTTAATGGCTCTCTTTGAAAGAGATGAGGAGGTGTTCTATTTTTGTTTTTTCGTACCATT
GTCAACTCTTATCTTATCATCGATACTTGGGTTCTGTGATAAAACACATCTCATTCTCTTGCTT
>TCONS_00052880
GCTGCTGTTTGTAGTAGTCGATAGAATCAACTTTCTCACCCCAAAGTCCGAGAAAGCCTTTCTTTTTAGTTGGC
CTCTTTTCTGGATTTCTTTCAAACTTAAGCTGGTTGTAGTCCAGCCAATTTTGAAGTCTAGCCCTTTTTCTGACA
AGTTTGGCAAACTTGTTGGCATTATATACAGCCTGGTGACAAAGATAGTGGTCAGGATGATTCTTCTTAAAGA
AATTTTCAACACTATCTGTTATTGAGCGCTTAGATTCATGAGGAACATTCCTAACCAGAACCGTGAACTGTTC
GGCACGCCTGTCTTGTGAAGCCAAAAATTTTAATCTCATTGCAG
>TCONS_00053090
GCTTCTGATCCGTCTCCTTATTGAAGTAGTGCTCCACTTCAATTTGCGCTATTGAATCTGGAAATTCCTCAGAT
ATCTTGCGAGTTCTGACATCAAAGGTGCGATAAAAGACAAAGTCAGCTGTGTCCAAATCTTCGTCTTTTGGCT
GAGGGTTCCTCCTATCATTTGATATGCAGACAACATTGCATTTACCAGTGATGGCTTCCACACGATTACAATTT
GAAAGCCCTATGCCTTCTCCTGAAGCCAAGAGTAACTCATTGTCAAGCAGCTTGATATTAC
>TCONS_00053236
ACATCTGAATTACCACCCAAAGAAGGGACAGCCAAATGAGTTATGTACTCTGCATCAACCACACCGACTTTC
CTTGTACGATCACCCTGCGCACAATAACCGAGCTTCATATCCAAGCCCCATGCATGGATCAGGTCATTCTGGA
CCATATACCAAGCACAGCGCCAGGCAGCTTTTGAGAATACAGGAGCCATCATTTCTACCCACCCCACACAAG
GAGGTGACGTGCTGTTGTTATCACACCTTTGGCCACTTTTATTTAATCTGTAGAACCTCCTGTGTACCTTGGATC
TCCCTCTGCGTACAGTAATATGATGATGTACCTCTGATTTACTAGCATCAAGTGCTGGCTGCGATATCTCCAGC
CCTTCTTCTCTAACAATAGATATATACTTTTCTGGATGGAAGTTTTCAACTCCTAGGTCCTCATCCCACAAGAA
AATGTAGTCGTACTCAGAAACTATATCTGGGTGCAGAAACCTCTTCGCAAACCACCATTTTGTTTGGTTCATA
GCAGAGACATGTAAGACACGGCTACTCCAATCCAAATCATTCCACTCATCCACCACTCCATCATAGTGAAAA
AGCATCACAACAAAGTCATGTTCAAGAAACTTCTTAACAATTTCGTTCACCATCTCTTTTTGCTTTATTCCAAC
TGCAATCCCCAACAAGTTAACTGAATGTGGAGACTTCTTCTCAACATCTCCCCACAATGGTCGCATTTCCAGG
TTCGTAGTTTTAGAAACAATTCCTTCTGGGAGTGCCTCACTCCCTAATGGCCTGCATTTTTTCTGGCATTTATTA
TTTTGCATATTCTCTGCCACGTGACTCTCTTCTTTTTCCTGTCTCGGTGGTACCTCGCATTTTCTGCATTGTCCAT
TCTG
>TCONS_00053354
CTCCACCAGTAGGTGCAGCAACTGCAACAGCACCACCACCACCAGCAGGTACTGAAGCCAACTTTTCTCTGC
CGGCAGCAATCAGCTCAGTGATGTCTTTTCCCTCAACTTGAGACAAGAGCAGTTCGATTCTGTCATCATCAGC
CTCAGCACCAACGCAACCAAGGATGCTTTTCAAGTCTTTAGCTGAAGGTGATGCATTGCCACCCAAAACGGC
CAACAAATAAGCTGCAATTACCTTCATTTTTTCCTCTCTGTAT
>TCONS_00053440

GCTCTTCTTTCGTCATGACAGAACGTTTAATAGATTGAAATAGGAAATAAATCATGTCAAAGAGGCGAGGCA
AACATGCAATCATCTGTTTGTGCCTTTTTGCCTGAGATATGGCTGGATCTTGCTCCTCTAGTTCCTTACTCTCTT
TTTCTCCTATCGATTTCAGAAGATTCTCAGGAAGAATGTCAAAAATATCATCGGTTGGAAACTTTTCACTCTCA
CAGACTTGATCAGCAGCATTT
>TCONS_00053527
ACAGTAGGTGAAGTGCTGATTGAGATACAACTTCATAACTGTCAAAGGAAGAAAGAACTATTTAGGGATATG
AGATATGATTGCTCTACTTAGCTGATGGATCATCCACTAATGAGCTTAGTAATCAAATGGAAGTAGAGGAAT
AGCACTTGGTTGCAGAAAATTTCCAAGTAAAAGACAATTTAGAGGGGGAGCTGAGACACATTGGCAATAAT
CGAAATTATCAGATCCAGGGTGAATCTCTGACAGGGAGAAGAATTTGATACCTAGCAAATTTTTGTCGACTTT
CTATGTTTCCTTCCAGGCGCTTGCTAATTAGGTTACAACCTTGTGAGTTTGTCTAATTAGTTTATTTGTCAATTG
GCGAGTGTATTGTCTCAGCTTTGCTACTTTATTCTTGCATAATGTCCTTTGAGCTCTTTTGCTTGTTATTCCGATT
CTTCTGTTAATTTGGCTGGATCAAAGCTCTGAATATCACGTGGCTTATCTAATAGGTGCACTCAGATATTGATA
GCTCATTAAATATTATGTTTATCAGCATGATCATCAAAATCTTCTCATTACAGTCTCAGTTTACTTAGTAGCAA
GTTAATCACAGTTATCAAGAATTCTGCATAGATCCATAATGAGTATGTGTTAGAACTGGAGCAAAAAAGCTA
ATCTGT
>TCONS_00053530
TCTAACCACCAAAACCATAGAGAGTACGGCCTTGCCTCTTGAGCGCGTAAACAACATCCATAGCAGTCACAG
TCTTCCTCCTAGCGTGCTCAGTGTAAGTTACAGCATCACGAATCACATTCTCTAGAAAGATCTTGAGCACGCC
ACGTGTCTCCTCGTAGATAAGCCCACTGATACGCTTCACTCCTCCTCTACGAGCTAAACGCCTGATTGCTGGC
TTTGTGATACCTTGGATGTTATCACGAAGGACTTTACGGTGTCGTTTTGCTCCTCCCTTTCCTAATCCTTTTCCTC
CCTTTCCACGTCCAGAC
>TCONS_00053805
TAAGCTCTAATTCTGCACAACTTCCCAATGTTACTAAATGGTTTGAACTGTGTATTTATGGACAGAATTTTGCT
GTCTATAAAAAATTTGGCAGCATATGAAGCTCCTTTTTGAGGATATTATTAATCAGTCCCATGCGTATTCCAG
GGCCAAAAATTGAAGCATCATCCCTTGCTTCCATATGTATAAAGATGATGCTGGGATTTGTGGCCATTCAAGT
GGGCATAAAATGGGGATACTTTATTGCAAGGTGGTACAGGCGAGCTAATGTTCTCATGTAGTAATATGCTGTG
TCCCATCCACAACACTACAAGGGAGGGTGGGGCCGTGGCTGAAGGGGCGTTTCACCCGGCAGAGGTGCCAG
TTGATACCTCAAGAGATGATTCCAGATACCTGTTTGGTAGTCATCCTTGTCTTTTTCTTTATTTTCTATGTGATCT
TATCATCCTCCCAATTTGATGATTCTCTCATCTACCAAATTACTCTAGGACATCTGCACTTTGGATATTTCTCCA
AGCTAAAGTTATACATTTGCACTTTAGCAAAATCATGTATTTCAAAACGAGAATCTGCTCAAGTATGAGTGCT
TAAGATTGCTTAGTTTTAATGAATTGCACCCAGATTTCTATACTTCACAAATAATTTTTGGCAATTTCTCTATGT
TCTCTGTTATGTGAATGGAAGAGACAACAAAAAAGTTGAGGGTGTTCAGAATTCCATTATTGCCGTTTGAGGT
GTTGAGTTCCAGTTAGACCTTTTTTGAGTTTCTATCTTTTGAAAGGTGTTTACAGCAGGCCAAACCGGGCATGT
GGAAGCATGATGAGGG
>TCONS_00053863
AGTTTATTATCTTCAAACATGCAGTTTATAACAAGTACAGAGTGATTCTAGAATGTGGGACTTTGTTTGCATTA
ATAGGTCATATATAGGAAATCCTCGAAGATAATATGCCCCTGAGAGAGACTGTTGCTGTCTTTGTTGTGACTT
GTGACTCAGACCTTAAGGGAGAGTGAAACCCCCAGTGAAGCAGTGCTTATGGCATCCATGACATTCATCTCT
GCTCCAATTGTCAGACACAGTCTTGAGAGAAGATCGTGTTGAAAAAGAGTAGCCAAACTGCCATCACTTGCA
ACTCGAGCTTTAATCAGCATAAAAGGAAACGGGGAATGCTGGGCTCCCAGGGTAACAGTCGTTTGTTTGCTG
GAGAATTGATACATTAGCTCCGCTGCAATCCCTGTGTGTGTTAAGGGTAGAATTTGACGGTAAGCAAGAGCTC
TCAAAGTGTCAGCTTTATCGCTCAAGGTGAGTGAAGCAGTGAGAATGTTAGTATTGAAGCTCAAACCAGCAT

CGCATTTGGCCACTGTATCAGTTGCTGCGTCAAGTGAGATATCTGTTCCAGTGTTGAAAAAACCATTTCCAAT
GACACCAGAAAAACTTATCAGGGGGCTTTTTGTCAAACTAATGCCTGCTGTATACCCAAAATAATCACGCAA
GTACTGCATTTCCACCTTATTAGACCTCTGATATGGTAAATAGAACTTGAGAAGTGCCCTTATTTCCTGTCTAG
GTACGCTGGCTCTAACTACAGTAATCGCAGAATGATCATACAGTAACTTTGAGAAAGCAGAATATAAGGAAA
TTGAAGAAATAACAATAGATACCTCCACTGCTGAGGTTTAAGCTCCAATCTAACCAATTGAAGTGATAGCAA
ATTGGCGGCTGCATGTTATAATCTCCATACAGAAGATCTGTTGCATCTTTGCCAATATCAGAATAGATCCCTG
GCTGCTTGCTCATTTTTTC
>TCONS_00053873
TCAACCAAGAGTAATGTACATCAACTTCATCTTTACATAGCACTTATTCGTTGATGAGAAGGTCAGATTCAGT
TTCTACTGGTAAATTTAAGATGGTTCAGTATTAGAGCTTTGGAAATTTAACCTTTTAAACCTCCAAATGCATCT
CAGTATCATCCATAAATGACTGACCAAAAACTTATCCAAAAGTGGATACAACTAAGAGCCTAAGAAATAGG
ATGCGAGATATGGCAACCCCATCGGCCCCATTAGACAAATGATTTGTGGGTATCTTAAGAGGTGGGATTTAA
AGCAAGCAAACGAGGACCTCATCGTCCAACTGCCTGAGCCACCAAGAGATATGTTGTGTTCTCGCACCTGGA
AATGTGTGTTAGAATCGCACAGATCACCAAGGTACATATTGTACGTATCTGATAGACCACGACTCAGATTCA
GCTAATTACCAGGCCCAAAAGAATTGCAGCCAAATCCCGGCTACAGCCAGAAATCTCTTTAGGAAGTCACAG
AACCAACTAGCTTGAAACTTCCAGCTCTTCCTCCTTGCTCTTTTGGTTTCTGATCCTCTCTGATTGAGAAACAA
GCAGCTCATGAACTCTATTCTTCAACTCATGAAGACCAATCTCATTCTTGACTGACACATGTAACGCTCCATC
AGGTCCCATTGTCCTATACCTCACCAGCTCAACGTCATCAATATTACATTCATCTGTTGCAAAGATGACAGGA
GATGCTTGCAGCAAGTCACATTTGGACACGATATCAAGCCAAATGTGGCTACTGAACCTCTCTCTCATTTCCT
TATAGATCAAAAACTGATCAGAAGGTGACATCCCACATTCTCCGGATAAATCATGAACATAAAGTACTGCAG
TCGGCAAATGAGTGAGAACAGCGAGTGTTAACTTTTCCAAGTTATTCCTGTCCTCATCAGATCTCCTTAAGAT
ACCAGGGGTATCAGTAACCTGAAAATTCTGGTAGCTCAAAGTAATATGACCCATCAGAATTCCTCTAGTTGTG
AAGGGATAGTTGCAGATCTCAGGCTTCCCTGTTGAAAGTACGCGAACCAAAGATGATTTTCCAACATTGGGA
GCACCGACGAGACAAAGAGTTGGTGTTTCCAGATTGATAACTGGCATTGCCCGTAATGTCTTGGCAATGTTCA
ATAGTTCATCAACAGCTTTTCCATCACGACCAAAAATTTCCTCAATTCTTTTCATTCCCTCATTCAACCGCTCCT
CTGCTTCTCGTTTAGATAATGACTTTGCACATAATGATGCATGTTCCTTTCCAACTGATACCACCCTTTTCCTTA
AAGCTTCAAGCTTTCCTAAAACCTCTTCATAGTTTCCATCTCCAAGCGTTAACTCAATCAATGATCGTTCATAA
GGGTGCAAATATCTTTTGTTTGGGAAGTTCTCCTTGTATGTTCTCAAGGGCACAGCTATCTCCTTCATTAATGC
GTCAAGTTGTTTTGCGCCTCTATTTCTCTCGCGTTTTGCAAGATTAACAATGCCCTTTGTAGGCGAGACCCTCTT
TGCTTTCTTCAGTGCTGAATGTAGAATATCTACAGATGGCATCACCATCGGTAATTTTTGAAATGCACCTATCT
TTTCTACCTCCGGCGCCAATTTTTCCTTTTTTTCTTGTTGGTCCTGTGTATTTGATACTACATAGCTTCCTTTCAC
AATTTCATAAGTAGTAGTCTGCATTTGCTTACAGAAGCATGACACTGGATACAATACACGCACAGGCACCAA
GTACAGACCTTTAGAGAGCTTAGAACGTCCAGAAAACAGAG
>TCONS_00053874
TCAACCAAGAGTAATGTACATCAACTTCATCTTTACATAGCACTTATTCGTTGATGAGAAGGTCAGATTCAGT
TTCTACTGGTAAATTTAAGATGGTTCAGTATTAGAGCTTTGGAAATTTAACCTTTTAAACCTCCAAATGCATCT
CAGTATCATCCATAAATGACTGACCAAAAACTTATCCAAAAGTGGATACAACTAAGAGCCTAAGAAATAGG
ATGCGAGATATGGCAACCCCATCGGCCCCATTAGACAAATGATTTGTGGGTATCTTAAGAGGTGGGATTTAA
AGCAAGCAAACGAGGACCTCATCGTCCAACTGCCTGAGCCACCAAGAGATATGTTGTGTTCTCGCACCTGGA
AATGTGTGTTAGAATCGCACAGATCACCAAGGTACATATTGTACGTATCTGATAGACCACGACTCAGATTCA
GCTAATTACCAGGCCCAAAAGAATTGCAGCCAAATCCCGGCTACAGCCAGAAATCTCTTTAGGAAGTCACAG
AACCAACTAGCTTGAAACTTCCAGCTCTTCCTCCTTGCTCTTTTGGTTTCTGATCCTCTCTGATTGAGAAACAA

GCAGCTCATGAACTCTATTCTTCAACTCATGAAGACCAATCTCATTCTTGACTGACACATGTAACGCTCCATC
AGGTCCCATTGTCCTATACCTCACCAGCTCAACGTCATCAATATTACATTCATCTGTTGCAAAGATGACAGGA
GATGCTTGCAGCAAGTCACATTTGGACACGATATCAAGCCAAATGTGGCTACTGAACCTCTCTCTCATTTCCT
TATAGATCAAAAACTGATCAGAAGGTGACATCCCACATTCTCCGGATAAATCATGAACATAAAGTACTGCAG
TCGGCAAATGAGTGAGAACAGCGAGTGTTAACTTTTCCAAGTTATTCCTGTCCTCATCAGATCTCCTTAAGAT
ACCAGGGGTATCAGTAACCTGAAAATTCTGGTAGCTCAAAGTAATATGACCCATCAGAATTCCTCTAGTTGTG
AAGGGATAGTTGCAGATCTCAGGCTTCCCTGTTGAAAGTACGCGAACCAAAGATGATTTTCCAACATTGGGA
GCACCGACGAGACAAAGAGTTGGTGTTTCCAGATTGATAACTGGCATTGCCCGTAATGTCTTGGCAATGTTCA
ATAGTTCATCAACAGCTTTTCCATCACGACCAAAAATTTCCTCAATTCTTTTCATTCCCTACAAAGATATCCTA
TTACTTATCAACTTATGGATTATATCAAATCAATAAGAAATTCAGTAAAAATGTGAAACTATCAGCCTACCTC
ATTCAACCGCTCCTCTGCTTCTCGTTTAGATAATGACTGCCATAAATTGCATCAATTACTTGATTTGCATCTTTG
CACATAATGATGCATGTTCCTTTCCAACTGATACCACCCTTTTCCTTAAAGCTTCAAGCTTTCCTAAAACCTCT
TCATAGTTTCCATCTCCAAGCGTTAACTCAATCAATGATCGTTCATAAGGGTGCAAATATCTTTTGTTTGGGAA
GTTCTCCTTGTATGTTCTCAAGGGCACAGCTATCTCCTTCATTAATGCGTCAAGTTGTTTTGCGCCTCTATTTCT
CTCGCGTTTTGCAAGATTAACAATGCCCTTTGTAGGCGAGACCCTCTTTGCTTTCTTCAGTGCTGAATGTAGAA
TATCTACAGATGGCATCACCATCGGTAATTTTTGAAATGCACCTATCTTTTCTACCTCCGGCGCCAATTTTTCCT
TTTTTTCTTGTTGGTCCTGTGTATTTGATACTACATAGCTTCCTTTCACAATTTCATAAGTAGTAGTCTGCATTTG
CTTACAGAAGCATGACACTGGATACAATACACGCACAGGCACCAAGTACAGACCTTTAGAGAGCTTAGAAC
GTCCAGAAAACAGAG
>TCONS_00053989
TCTATTGCTAATAAGGAAACAAAATGAACAGCAGCTTAACGTTTTCCTCCGCCGCCACCAAGCTTAGGACCTT
TTGATGCACCTCCTTTGGGGAGGTTGCCCTTTCCTCCAGCTTTCTGGGACTTGGCCGTCACCTCTGCTTTCTTAG
CCTTCTTTTCATCCTTTGTCTTCTTAATCCTTTCCTTAATTTCACGAAGAGCAGCCTCCCTGGCAGCATCTCGAA
CTTCTGGCCTTTCAGTTCTTTTCTTCTGGATAACCTCCAAGGTTGCACCCACAATGGACCTAGAGTAAGGCTTC
TTGGTGGCACGTCGCCTCTTCTTAACAGCTTCTTGTGCAATATCCTTCTTGTGTTGCTTCCTATACATAGCTGTC
CAAGTAAGTTTTGATGGCTTCAGCCTGTTGTGGAAGTATCTTTTGCATTTTGAATTGAGGAATAGGAACACCTG
AGAATCTGAACGAATAAATCTGATGCCCCTCCCAGGGTAAATCTTGCCACCACTGAAACGACAGAGTTCTGT
CTTGAGAACCATGGCTGATGCTGTTGCGTGCCCTCCCTCTCGATGAAGAGAAACCCTAAG
>TCONS_00054011
TTTGTGTTACAGCCCAAATCACACAGCAGAAAAACATTACATAGAGAAACAAAATTAAAAAGAAAAGCCAC
GACACACAAACCTAAGAGCTGATGATCACAGTTCCATGGCTGCAGTCTTATCGTTATTATTACAAAAGCTTTT
ATAGCACACACTTAAATTACTGTGAAATAATCTTAACAATTGCAAGGGTCACAGGTGCAGTTTGATCCACACT
TGCACCCATTTCCTCCTTCTGCTGCTTTCTCTCCGAATTCCTCAAAGTTGTTCATGGGAGCAACACCCTGAATG
ATGGTAAAAGTAGTGGACTTCTCCAAGTCTGGGTACATCCCACATCCTCCGCAACCGTTACCGCACTTGCAAC
CAGACCCACAGCCACAGCTTCC
>TCONS_00054028
CCCCTCTGCATCTTCACTCCATTACCAGTTGAATAATCTTGAGCTTGCTGTCAATCTTGCCAAAAACGGAAAC
CTTCCTGGTGCTGAGAATTTGGTAAGTTAAAATCATGTTTCTTAAGGCACTATATTTTGAATTAATTTCTTGATT
GGATGGAGATTAGAGTGTCGAATGGTGGGATATTTTGAATTTGGTATGTTCATTATATAAGGATTATTACCAG
CTATAAAGTGGAAAGAGAGAAGTGGAACTACGAAAAGT
>TCONS_00054047

TAAAGCCGAAGAAATTACATAGAATAGAAGACTATAGACTAGTTGTGCAATGTTACTTGAATCTTCTTTTTGG
CTTTATTACTGCTAACAGCAGAAGATAGGGGGCTTCGGTCTTCCAATCTTCATTCAAGTTGCTTATCTGAGGTC
CTCTACAACGGAGCTGAGCTTTGGGGGGTATTCCGAAGAAGAATTAGGGTTGTTGATAGAACAAGATGACTT
AAAAGCTTCAATATGAGACTGGCATTTCAAATAGTCGCCTTTATTTTCTTCCAAACATTTCTTCAGAGCCTCCA
CATCGCATGCTTTCTTCTTCACACTCTTGTTTTCCTTCTCCGTTTTCCCCTCCATTTTT
>TCONS_00054054
CAAAGAAAAATGGAGGGGAAAACGGAGAAGGAAAACAAGAGTGTGAAGAAGAAAGCATGCGATGTGGAG
GCTCTGAAGAAATGTTTGGAAGAAAATAAAGGCGACTATTTGAAATGCCAGTCTCATATTGAAGCTTTTAAGT
CATCTTGTTCTATCAACAACCCTAATTCTTCTTCGGAATACCCCCCAAAGCTCAGCTCCGTTGTAGAGGACCTC
AGATAAGCAACTTGAATGAAGATTGGAAGACCGAAGCCCCCTATCTTCTGCTGTTAGCAGTAATAAAGCCAA
AAAGAAGATTCAAGTAACATTGCACAACTAGTCTATAGTCTTCTATTCTATGTAATTTCTTCGGCTTTAAC
>TCONS_00054074
AACTGAACATGTTCCGAAGGAATTCAACAAATGATAGGTGTCATGTCCTTAGAGCATTCTCTGGGTCTGAAAC
TACATCTTGGGTATCTGAAGGTTGGTGTCTTCACCAGTAATCCCGACACGAGCACAATCCGTCTTTTAAATGA
TGAAACCTCTTAGCATCTCGCATAATAATTTCCCGACCAACAAGCCTCGAAGCAATCTTCAATGCATTATGGC
AATCACCACAGACACGCAGATTCTTTATTATTCGGATTGGTGATCGTGCAGAGCTACTCATAAGACCTTGAGC
AAAAGCAAGTCTCTCGCTGTGAGCCATTAAAGCGTCCTCTTTAGTTTCTTGGTCTACGTCATGTAACACAAAC
TTAATCTCTGGTATATAACCGTCCTCCTTCATTAGCTGCTTTAAACCCCTAAGCAATTCATATATCTTCTCATGG
TCGGGATGTGATCTATCTCCTGCTCGATATTCATGGACTCTGCTTCTAGCCTCAAGAAGACTTTGAGCAGATTT
TTTCTTTTCTTTGTCCTTAGCAATATCTGATGCTTTTACAGCTAAAAAACCTGCCTTTGACTGTTCATCCAAACG
AGATGGATCTAGGAGTTCAACAATCTCAGCACAACGATCCCCAAGCTCCAAGTTCCCATGAATTCTACAGAG
ATTCATCATGGTTTCCCATACTTCAATACTTGGCTCTATAGGCATTTTTTCAATGAACTCTATCGCTTCAATAAG
ATATCCTGTACTTCCCAACATATCTACCACTCCCACATAATGCTCCATGGAGTGAACAATACCATAATCTTTA
CTCATTGATTCAAAGTGCAACATGCCTTCAACTATATCACCTACAACACTACAAGCATGAAAGACACCCAGA
TACATTTGGCCATCAGGTTTCATCCCCGTTTTCTTGAATTCTGCAAATAATTCTATTGCGTCTTCTCCAAGACCA
TTCTTAGCAAGCCAAGTAATCATCGTGTCCCAAGAAGTCAGATTACGCTGAGGCATTTTTTGAAACACCGAA
AAGGCATCATTCATGGAGCCACATTTGCCATACATTTCAAGAATCTTATTGTACGTTTTGACGTCAAGATGAG
GATGAGATCTCACAAGGTGCTCATGAACTGATTTAGCCTCCTCAAGGGATTTATCCTCACCACAGACATCCAT
TAGTGTTATATATCGAGACAAATTGACTGTGACATGTTGCTGCTCCAACAAATGCAAAACTTCAACTGCCTCC
TTCACTTTGCCTTCTTTACAAAAGTTATCGAGCTCGTCAATTGAGCCTTTTTTAATGCTGCTATCAGCAGAATC
GATTGACTGTTCAACAGGTGTTGAACTTCTCATTTCTTGATTACCTACTATTCCATTCTGAGATTGATAAGAAG
CTTGATGTCCAGTACTAGAAGGGTTAAAGCCGCTTACATTCTGCTGATATTGCCCCATCATCTCCCTTGAGTAA
CCACTTGAATTCGTATGCCTTTCCATATCATACGCACTTGCACTTTGTGGATACGTTTCAGAATTTCCTGCTTGA
ACATTCCTTACATTTCCAGGGATATA
>TCONS_00054346
ACTCTTTCTTGTTTTTGAAAAGATCCTTCACATTCTGAAGGATGTAGTCAATGAACTTCTGAAACAATGGACTG
CATAGATTTGGTAAAGGCCACCTTCCTGGATCCAGTGAAAGGAGAAACTCATAACCTTGTTTGACTTCAGCAG
AAGATAAGTCTGCCCTTGGACCTGAAATACTATCTCCGACTTGCATTCCATGTCCTCGCAACTTCCCATCCTTA
ATCCTCCCTTTTTGAGTTGCCCAATACAAAAATGTATCAAGAAATCTCCTCTCTATTTCAGACATTTGTTATCTC
GCGGAGTTGTCCTTAGAATGGTGCAGACAGCTCTGGATGATTGTTGTGTTCTTCTTCTTATTATGATAAAAAAG
CTGCAAGAATGATCATGGACCTTTGGTAACTTTGACAGAGCAGAAAGAAATGATGTAGGCTTCACACCCTTA

TTACCAATTTGTTCTACACATAAGTTTCCTGCTTAGTATATTGTTGCTGAGCGTTTACCAAAAAGAAGAATGAA
CAAGAGATTTGAAGAGTA
>TCONS_00054354
GGAAGTCACTTCCTTTCATGCATATAACTTCATCCCGAAGTTTGACTTTGTGTATAATTGTTATTAACACGACG
TTTATAAAATCTCGCTCGCCCTCTTCCTCGGGTTGGAATTGCTTGCTCCATAGGCTTATCATCCACAGAACCAA
TAGTTGTAACACAAGAGGCCTCTAAAGTCATCTGGCTAGGGGTATTATCATAGTTAACCTGTCGATCAGGACG
GGCAGGGGAGTGGTGTATGTCGTCATCATCAAACCCGTCTCCAAAATTATTCATATCAAACTGCTGAGAGGG
TGGGATCTGATAAAGTACAAGTTGAATGTCAACATTATGCAATTCAGAACCATCCCCATCCCCATCCCCATCC
CCAAAATGTGTTGAACTAGATGCCATATCAAAACGGGGCTCTGCAAACTGTGATGAATCCATACCAAGCTCA
GCTTCAACATTTAAATTTCCATCACCCTTAACAGAACCACCTGCACCACGATCACGCTCCAAATCCGAATTTG
TATTAGACATTTCAGACCTTTCTTCAACATTAGTTGCTGCTATCGCACGTCTCTTTTTGCGTCTCCCTGCCCTTC
CACGTTTATGTGGATTAACAGCATGATCAACTACAGGCTGCTCAACTGGAACATACATTGGCTGAACACGAA
GCCTCTGAGCCTCGATTGCTGCTGTCAAACCATCACTGCACATCTCCATGACTTTCCTTCCTGCAACCTCCATT
GATGGTTGTTGTCGCAATTCAATACCATATTGATATATCATATGCAACTGACGTGCCATAACCTCCGCCACTG
GTGCAAGGGATGCATACCCTTTTGGAAATCGGATACTGGGATTTCCGATAATCTGTCGAGTAATCCGACGATA
CCAGACAAGGTAATCATCATCATATGCAACGGGTCCATCTGCTAGCCGGGCTTCACAAACTCGGTTAGCACG
ATCATTCCAGACAAGTATGGGCCTCGCATGTTCTCTAGCCCAATCCGTGTTTTGTCGGCCACGACGATCATGC
ATGTCGTGATTGTCTGCAAAATTGCAGGAAACAGGTATGACTTGACCCATTCCGAATTGTCTGAGGACCCTGT
TGGGCTGATGCATCTCCAACACGTCCCAACATAGGAGCGGCACCACAGCACGCCAAAAGTACTCTCCTGATC
GGCAATACTCGGGAAGATTCTCCATCACATCTGGAGTATACGGCTCCCAAACAAAATGTTCATCAACGAGGT
TATCTAGCTGGTCCCGAAACAATGGCAACACGTATTGCGTCGTATGTGTCCAACTAAGATGAACGTTCCATTT
ACATGCGTGTGGTGGACCAGGGAGATCATTCGAAAATGTAATAGCACCCTCTCTTGCCTGACGTCTGTCTGGA
TGAACCATCTTCAGTCTCTCCCATGCCCATACCTGTAGAAGTGGTATGAAGCCACATAACTCATTGTTTTTGG
CCGGGTAGCTCTACACAATGCACGGTACAAGCACGCCAACACAGCACTACCCCAACTATACATACCAATCTG
CGTGATGTCATCGAGCAGCGTCAAGTACATTAACTTAACCTTGTTTCCTGATGTGTCAGGAAACAGTATACCC
CCGATCATCCATAACATATAGCACCTAGCCCTCTGATGTACAACTACCTCATCTGCATCATCATCTAGCAAAG
GCAGTTGCTCCATGTATGTAGCTAGTGCACTAATCTCAAGCCTACTACCAAATAAACAATCACTATGTGGTCT
AAAACCAAGCAACGCGTCGCACATATCCTGCCACTCTTCGGTCTTTCTTGTACTATCAATGCCCAATACTGGC
CTTCCCTCTACTGGCAATCCATACAAAATAGCTACATCCTGTAGCGTAATAGTGGCTTCCCCAACTCTTAGGT
GAAAAGTATGCGTCTCCGGACGCCATCTCTCAACAAGAGAAGTAATAAAAGCATGGTCTAATTGTATACGTC
CTACTCGATAGACCCCGTAAAATCCTGCATCAATTAATATTTGCAGGACACGTCGATGAATTGGACGATCATG
AACCAAATTCCAAAAATTACCATCTGATCGTCTACATAAAATAGGCTTTTCCACCTCACCATTCCATACGTCC
TTCGACCTGTGATGGTTTTGTCTTGTCAGTACGCTATGGTCTAACGGCCCAGGATGTAGCTGTGTCAACCTCTG
ATCCTCCATG
>TCONS_00054358
AAGAGATGCATTAAAATTTCAAGATAAAAGGTTCCAACATCAGAGATGCACATGTGATCCACGGAATAGAG
TTCAAAGTCCTCGCTGACTTACCACTACTAAATATATGGGAATTCACATGTCTTTGGGGCTCCGCGGTCAGGTT
CTAGTAATAAAGTGACCCTTCCCACCAATTCCCTCTAGAAGCACTATATTGGTCGTTTCCACCAGAATCATAT
TCTCCTCCTTCCTTCTTGTA
>TCONS_00054520
TTTCGTTATTCAAAAATGGTGCAAATCAACACCGTAGACTGTCGAAGAATTTCATTTCCATGGAACTTTCAGC
TGGATACTAAAATTATAGAGTTCAATTTTGTCAATTCATTTTCTATGGTTTGTGTAGCCCCGTTCCACTGTGAA

GCGCCTCCATAACTAGTAAGTCACTGGAGCTATGGTTTTCATGCCTTTGTTATTCCAATTTATGTTAAATCTATG
AAATGTTAATTTAGTTAATTCAGATCTTACCATCGGGTTATTTTAGAGAGAGCTTGGAGTTAACT
>TCONS_00054552
AACAAACACGACCAACTTGCCTCTGATAAACCACCTTGATGTATCGTCAGTTGCTTTATGTAGAATCCTCAGC
AGATTTTGGTTCATCTTTTGACTTCAGTGCGGCCGAAGGTGATGGAGAACCTTCTTGGCCCATTTCAGTTCTCA
AATCGTTGATGCTCTGCTCCAATTCATCAATCCGGTTCCCCATCTCATCAATTTTTGAGATGATTGATTCAGAC
ATTGTCTGAAACCTGGTTTGCATTTGCTGAAGAAGATTCTGGACAAACACAGTCATATCAGCAGTGCTTTGTTT
CGTATTATCTGCATCATGCCCGTCCTGCAAAATGATCCAGAAACTATGGGTCAGAAG
>TCONS_00054613
GGGTGCTATTTCTTCAGAGATACTCCATTTAATCGAACTTAAATGAATAGAACAAAAGATTACTGTTTCTCCA
CTTCTTTCTGCTTGATTCCATCAATGCTTTCTAATGGAGGCAGAGTCAGATCAGGTTTGAAAAAGTTGTGAACA
ACGGAGGCACCAGCAAGGAGGGAAACAACGACAACAACAGTAGACCAAGCCATTGATGGAGTCCCTGTTG
G
>TCONS_00054623
TCGTCTAATAAAGAAGTGGCTTAGAGATTAGTAAGTTGATTGTTGGTCCCAGTTGGTGGCTTGTTTCCCTATCT
TGTGAAGAACTTTGATGACCTCTTCAGTAGTCACATTTCCAGTTACTGTCACCTTATTTAGTTGTTTATCCACAT
CATATGTTTCAATATCTTCAATTTTCTTGATTGCCTTCAAGATTTTCTTGATGCATTCGTCACAGTGCAGACCAA
CTTTAAGCTCCACTACCTAGTGCACAAATGAAAAGT
>TCONS_00054632
CCGCTTTATTTGCATCAGGTTCCTCCTCATCCACTAGAACAACAGCCTCCCCCTGCTGCCGTCGTTCTAATTCA
CCCTCGTGCAGTTTGATATTTGCCCTAAGCTTTTCACCTCCATCGCGTAACCTAGCACCAATGGTGCCGGACA
GAGACCTTTTAAGCCTCCTAATTTTTTCGGTCAATTCGGTATTTGATAAGTTGGAAAGTACTTCTTTGTGCTCCT
CTCCG
>TCONS_00054855
CAGAGAATTTTGGGAAGTGGCAAACTTGCGGAGAATGTCCACTGAAAGAGACCTTCCACTGAATGGCTCTAC
GACTTGATCTCTCAGTGAACAGCACCCACCAAGTTCCCAAATCGATGGAAACCCGTCTGTAATACCTGCATTC
ATGCATCACTGTGATCTGAAGCTTCCTCAGAATCAGACAAGGGCAAGAGGGTACATATCCCAAGCACATGCC
CGTTAAGGCACACATAATACTCAATTGCATCTTCATAAGCACCCAATCTACAGCTGGCACTAGCTGGCATCAT
TTTTGCTTGCAGCATGCTCCACAGTTTCGCCTTACAGTAAGGGCACACTTCTGTTGGATGGAGCTGAGCCTCCC
TCTTAATCAACATTTTACGAACTTTTGAGGAAGCAAAGGATTTAAATATTCCTCGAAAAAATCCAATATCCCC
CTCATCTCCTTGGTCAAGATGCTCGCAAGGATCAGAAACATACAAAACATCTGTCCTGCATTGTGGTAACAG
AAAGCTCTTTCCAGAGGTCCTAGAGAACCTAGTTCTATAAACAAAGTGTCCTGGAATGTGGATGCTATTGAAT
AAGCCACCTTTATTACATCCAGAGCAGTAAATAAGAAGCTTTCCAAGAGCCCTCCAGTTCCCATCAACACTAT
GACTCCCACTAGACTGTAAATCAAGCATCATTTTGGGAGCTCTAGTCTTACAAAACTCCTTCCAGAGCACTCT
CTTGGCAAGGTCATCAAACCATTTGCATACACAAGATAAAGTTGCAATTGACTTAGGATTCCAATTCAACTGC
TGAAAAACCAGAAATATAATTTCTTCGCTAAGATGCCCTTTTGTACACTTACAACTTGCTGAGTGTATGCAGC
GATACTGCTTAGTAATGATCATCGTCTACAACTGCAGCCACCACAATTGAAATGACAACAAAAGTTAGATCA
TA
>TCONS_00054856
TGGTGTAACTTAACTGCTTCACAACAAACATCAATGAAATGTTACAGATTCCAACGTGTCACTCAAATAAGAT
GCACATTTGAGATCATAAAATATACAAAGAAATTCTGATGGTGCTTTGTTACAACTGAGACACAATGGACAG
GAATAGGTTTCTATTGGACTGCGCTTGTCAGAGAATTTTGGGAAGTGGCAAACTTGCGGAGAATGTCCACTGA

AAGAGACCTTCCACTGAATGGCTCTACGACTTGATCTCTCAGTGAACAGCACCCACCAAGTTCCCAAATCGA
TGGAAACCCGTCTCTAACACCTGCATTCATGCATCACTGTGATCTGAAGCTTCCTCAGAATCAGACAAGGGCA
AGAGGGTACATATCCCAAGCACATGCCCGTTAAGGCACACATAATACTCAATTGCATCTTCATAAGCACCCA
ATCTACAGCTGGCACTAGCTGGCATCATTTTTGCTTGCAGCATGCTCCACAGTTTCGCCTTACAGTAAGGGCA
CACTTCTGTTGGATGGAGCTGAGCCTCCCTCTTAATCAACATTTTACGAACTTTTGAGGAAGCAAAGGATTTA
AATATTCCTCGAAAAAATCCAATATCCCCCTCATCTCCTTGGTCAAGATGCTCGCAAGGATCAGAAACATAC
AAAACATCTGTCCTGCATTGTGGTAACAGAAAGCTCTTTCCAGAGGTCCTAGAGAACCTAGTTCTATAAACA
AAGTGTCCTGGAATGTGGATGCTATTGAATAAGCCACCTTTATTACATCCAGAGCAGTAAATAAGAAGCTTTC
CAAGAGCCCTCCAGTTCCCATCAACACTATGACTCCCACTAGACTGTAAATCAAGCATCATTTTGGGAGCTCT
AGTCTTACAAAACTCCTTCCAGAGCACTCTCTTGGCAAGGTCATCAAACCATTTGCATACACAAGATAAAGTT
GCAATTGACTTAGGATTCCAATTCAACTGCTGAAAAACCAGAAATATAATTTCTTCGCTAAGATGCCCTTTTG
TACACTTACAACTTGCTGAGTGTATGCAGCGATACTGCTTAGTAATGATCATCGTCTACAACTGCAGCCACCA
CAATTGAAATGACAACAAAAGTTAGATCATA
>TCONS_00054891
AATTCTTGGAACGGAACAAAATAAAGTTGGGTCGAATGAATAAATGGATAGGGCTGCGGCTTCAATTAAATT
ATAGGGAAAGAAAAAGCAACGAGCTTTTGTTCTTAATTTGAATGATTCCCGATCTAATTAGACGGTAAAAAT
AGCATGTCGTATCAACGGAAAGTTCTGAGAATATTTCATTGTTCCTAGATGGGTAGAAAACCGTGTTAGAATT
CTTGGAACGGAACAAAATAAAGTTGGGTCGAATGAATAAATGGATAGGGCTGCGGCTTCAATTAAATTATAG
GGAAAGAAAAAGCAACGAGCTTTTGTTCTTAATTTGAATGATTCCCGATCTAATTAGACGTTAAAAATTTATT
AGTGCCTGATGCGGGAAGGGTTTCTTGTCCCATGAGTGGATTCTCGATTTTTTTAATGAATCCTAACTATTACC
ATTTTCTATTATGGAGATGTGTGTGTAGAAGAAACAGTATATTGATAAAGA
>TCONS_00054892
TTAGAATTCTTGGAACGGAACAAAATAAAGTTGGGTCGAATGAATAAATGGATAGGGCTGCGGCTTCAATTA
AATTATAGGGAAAGAAAAAGCAACGAGCTTTTGTTCTTAATTTGAATGATTCCCGATCTAATTAGACGGAATG
AATGGTAAAAATAGCATGTCGTATCAACGGAAAGTTCTGAGAATATTTCATTGTTCCTAGATGGGTAGAAAA
CCGTGTTAGAATTCTTGGAACGGAACAAAATAAAGTTGGGTCGAATGAATAAATGGATAGGGCTGCGGCTTC
AATTAAATTATAGGGAAAGAAAAAGCAACGAGCTTTTGTTCTTAATTTGAATGATTCCCGATCTAATTAGACG
TTAAAAATTTATTAGTGCCTGATGCGGGAAGGGTTTCTTGTCCCATGAGTGGATTCTCGATTTTTTTAATGAAT
CCTAACTATTACCATTTTCTATTATGGAGATGTGTGTGTAGAAGAAACAGTATATTGATAAAGA
>TCONS_00054893
TTAGAATTCTTGGAACGGAACAAAATAAAGTTGGGTCGAATGAATAAATGGATAGGGCTGCGGCTTCAATTA
AATTATAGGGAAAGAAAAAGCAACGAGCTTTTGTTCTTAATTTGAATGATTCCCGATCTAATTAGACGTTAAA
AATTTATTAGTGCCTGATGCGGGAAGGTTGGGTCGAATGAATAAATGGATAGGGCTGCGGCTTCAATTAAATT
ATAGGGAAAGAAAAAGCAACGAGCTTTTGTTCTTAATTTGAATGATTCCCGATCTAATTAGACGTTAAAAATT
TATTAGTGCCTGATGCGGGAAGGGTTTCTTGTCCCATGAGTGGATTCTCGATTTTTTTAATGAATCCTAACTATT
ACCATTTTCTATTATGGAGATGTGTGTGTAGAAGAAACAGTATATTGATAAAGA
>TCONS_00054986
TTTGGAGAGGAATTCCTGGGAACTATCCATGAAACTAACTGATGCTCCACTTCTCTCCAGTTGTGCACTTTCTT
AACCATGGGATGTCCATTAAGTGACTCACTCTTGGACCATGGATATGAATTTTCATAATCAAAAAGAAGAAC
CTTGATTCCAGCTTCCGCGCACTCGATAGCATACCTTGGATTGTCATCAATCAAAACATTTGCTCCCAAGGAC
CTGCAAATATCTGATTTAGCTATAGACTGCCCATTCAATGCAAAGTGATTGCCAAAGTGCATATCCTGAAACA
ATCCAGGATAATGCCTCTCAATCCACTCAATTGTGTGATCTTTAATGGCGTTTTGCCGGGACGTTACAATTGAC

AGGTTACAGAATCTAGACATCTTTTGAAGAGCCTGTCGAGCACCGGGAATTGGATGAATTCCCGTCTTGAAAT
AAGAGGTCTTGAAGAATTCATGGACACGGATATCAGCTTCATCTCTTGAGCATTTCCATATCTTGAAAAATTC
ATAAACATGATATTCAGACACCGAGTGGTATGATGAATAACGATCTGCAATGAATTCGTTGAGAGCTGAAAC
AAAGTTCCCAAGGACTTCATCAACATCAACAGCCACAACAATTTTGTCATGTAAATCCTGGTTAAAGAATCC
AGCACAGCTTCCTCTGTTAGATGTATTGCTGTTAGTATCAGAAGGCTTAGTAGCATTACTAGTACTAGCTCCCT
CTGCATCATCATCATGTAGCAAGAGAGGGAGAGATGGAGCAAAAGCATGTGCCCTCTGAAACCCATTCACAT
ACTTATTGTTACAACTTGTATTTGAATCATTAGAAGAAGAAGAAGAACAGCAGCCCCAAAGCCTGAGAGCCG
TCGCATTATTAAAAGGGTATTTTCCTACACCACCACCACCATGTGAATCAATGGTAGTAGATAAAAACTTTAC
ACATGACAAAAGCTGAAAATCTTTAGGGCCAACAGAAAAGGTAGGTGCTATTGCCATTAAAGGACGAATCC
AATTATTACTTCTCCTTCTTATCATAACGATACTGGAAAAAATGCCCTCCCACACATCTAGCAAGACCCCACG
AA
>TCONS_00055068
TTCCAATATAAAGGATTAGTCAATCAAATAATAGATAGTAAATGGGTCGGTTTGAAAATAAATGAATTGCTA
GTCGTAGAATATTATTCTCGTCAGACTTAAACCTCAGACTGAAACCGAGGGTTCGGAAAATTTTCTTCCCTTTA
GGCGAAGTAAATTAACCTAAGGTTAAGTAAGATAAATCCGAGTTTGATCTGGATTTCCCCCATTTTTGTATCA
TAGACCAAGGATCTATTTTCATTCCTTGTCTACTCTTTTCTTTTCAGTATTTTGATTATGATTATTTTTGAGTATT
CTATTTTACGCGTCACAGCAATTAGGAATTGAGAATCTATATCTTTAGATCAAAGAAGGGTCTAGCTGTTGGA
ATAATGGTATCCATTGCCCGAGTGGGGATAGCATTTCTCTTCTGCATGTCCATGGAGTTTTGAAAAATCCAAA
CATCTCAGAGATAGATAGAGAGGTAGGAATTTCTCGAACGAACCGCACTCCTTCGTATACGTTAGGAGTCCA
TTGATGAGAAGGGGCTGGGGAAAGCTTGAACCCAATTCCTACGGTAATGAATATGAGCGCAATTGAAATTCC
TGGGGAGTTATACATTTGTGTATTGATAAGACCGTTTACTATTTCTTGAAGCTCAATCTCT
>TCONS_00055074
TAGACAAGGAATGAAAATAGATCCTTGGTCTATGATACAAAAATGGGGGAAATCCAGATCAAACTCGGATTT
ATCTTACTTAACCTTAGGTTAATTTACTTCGCCTAAAGGGAAGAAAATTTTCCGAACCCTCGGTTTCAGTCTGA
GGTTTAAGTCTGACGAGAATAATATTCTACGACTAGCAATTCATTTATTTTCAAACCGACCCATTTACTATCTA
TTATTTGATTGACTAA
>TCONS_00055122
TCATTCAAAGATTTGTTTTCTTCTCGAGTTGAACGTAGCGCGGACAGTAATTCAGCCTTTGAAGAAGTTCCACC
TTTCAAGTCTTTCGCGTTCACTCCACCCCCAAATCCAAACACATGGCTACGAGTTTGAGGTCCACAACATTTTT
CTACAATCTCTATGCTAGGTAGAGATGGATCAGCTTGAACTATTTCTTCGATTTGAGCATGTTTTTCAATTGCTT
CAGGTTCAACAAGCTTGTTATCTTTCTTACGGGTCTCAAAGAAAA
>TCONS_00055158
TAGACGATGTAATGGCTCAACTAGATGATGTTATTGTACACAATAAAGACATCCTTTCGTGAAATAAGGTTAC
ATTAGACATTAGTCCTAGTTCCAAATGGTAACCGTGCTCATGTGAGCTGAAGGACAATGCTCAGTTAAGGTGG
TACAAAATACTTAAGATAGTCCAATTTTCAGATGATATTAACTTTAAGAGGAAATGACATCAACTCACCTTTT
CTCTTGCTTCGACCAGAAGTGTTGGCAGTAGGAGTAGGTGTGCCATTATCCATAGGACAAGGAACCTCCTCCA
TTCCAGAGGTTTGATTACCCAAGCATTGGAGAGTCTCAAATTCACAGTTATTGAAGCCTTGTCCATTTAATAC
AGTGCCGTCTGAGCTTTTAACTTCTGCACATTGCCGCTCTTCATCGGCTTCTGGTATGGAAATGATCCAATTTC
CCCTTCAATCCTTCCTCGGATGTCGACAAGCAAGCACTTGAGCCTAGCAACCTCAGCTTCCAATACAGCCTGA
CCCTGCAGCCTCTTGAATAGTTGCTGATTTATAGCCCTCAATCTGATAACCTCATCCTCTAATGAAGCAGCCC
GAGCTTTCTTTTTCTCACGATACTTGCGAACGGCTTCCTTATTACCCACGGGTCGTTTCTTTCCTTTCTTCTCTGA
AGACTCTGCAGTATCATCACTGGGGTTCTTATCCTCGTCCTCCTCCGAGGGAGGCACGAATTTGGTGTGAACA

TGGTAACAAGTGTGTGTATGAGAATTATCAGGGCCAGGCGGGTTGCAAGTGTGGGCGTGAGTGCAGGCATGC
GTATCCTTGAGAATATCATTGAAAAAGCTGTCCATGGAACTACTAAATTCACCAAAATTCGAACTTGATAAC
ATTTCTTGGTTAGAAAATTCCAGCTCTCCGTCAGCCATCATAGCAATCCCAAAAGTAGTAACCAGTAAGGTGA
CAATAGTGAAGCAGAGTCTGATGAAGAGGAGAGAAAAAGAGAGAAAATAGGGTCAGAGATAATGAAAAA
AGAGGAACCCTAGAAAGGGCTAAAAAGGGGGAGTTGAGATGTATTGTCAAATCTTTTAATGGCGGAAAAAA
AAGAATGGAGGAAACAGAAGGGGCCACTAGTCAAGATTCCACACACACATGCACAACACTTGCTGCTACTG
CAAAATTCAGCTCCGTTTG
>TCONS_00055201
TTGTAAATAGAAATTGATAGGAGCATTGTCAACAACAAAATTCTCCAAGTTGCAGTATGCTGCAGAAACGAA
CTGTCTTCACAGCCTCCTTCCCCTTCTACCACCCTTTCTGCGAGTGCTATCTGTGGGAATTGGAGTCACATCCTC
TATGCGTCCAATTTTCATGCCAGATCGAGCCAAAGCCCTAAGAGCAGACTGGGCACCAGGACCAGGAGTCTT
AGTCTTGTTACCTCCGGTAGCCCGAAGCTTAATGTGAAGAGCATTAATTCCAAGTTCCTTGCATCGCTGAGAC
ACATCCTGAGCTGCAAGCATGGCAGCATATGGAGAAGATTCATCTCTATCAGCCTTCACCTTCATTCCACCAG
TAATGCGAACCATAGTTTCTCTTCCAGACAAATCAGTTACGTGAATGAATGTATCGTTGAAAGAGGCAAAAA
TGTGAGCAACACCGAACACCAATTCACCCTCCCTCGTCGCTGGACCAAGTGTTACGGTCTCTTCCTTTGGCTC
CCTAGTCTTTCTCCTCGACATGGATTCGGCTGGGATTGGTAGTGAGAAGCTCAAGAGATACTGAAACAGCTAG
CGAATAACCCTAGTTT
>TCONS_00055324
TACTGGTCTAAACAAGAACCCCGTAATCATTCTGTTGGGCAATGTCCTCTCTAGTTTCATGGGAGACCTGGGG
AACAACATCAATTGAAGCATGAGGCTTATCTTCTGCTACTTCACTGGAGAGCCGGCCATCTTCTTCAGCTAGA
TGATGGAAGTGTGATTCGTCAAGTCCATTAGCATCTTCGCAAAGCTCCAAATCATCGACACTGTGAGCCACAT
GAGATATCCGGCCGTGATCATCCTCACTTACTGTAATACATAAATCATCACTGCCCATGTGATATAGGTGAGA
GTCATCTGCATCAAAAGTTGCAGCAGGTAACCGAGATTGCACAGGTATCCCTGCTGCATAATATTGCTCTATC
TCTTCTTTCCGCTTACGTTTGCCCATCCCCTTCCTTCTTACTCTCTCCTTTCCACGTACTCTCTTTCCAACTTCACT
TTGTGAGTTTGTTGCAACAACTACTGTACTTGCAATCTGATCCCTCAAGCATTCATAAGTAATATATCGGATTC
TTGTGACAGATTGTAACTGTTGATCATCCATCCCATGAGTTGAAAGGGTGTCTGCTATAAGGGCAATCTCCCT
CAGTGCGGCATTCATTCTTTGAAACTCAGATGAGATAGGCACAGGCCTCCCCACCAACTTCCTTGTAACTCTC
AAATACCACTGCATGTATTCACTTTCTTCCACATCTTCCTCTGCCTCCACAATGTAGAGATGGCGATTCGACCA
TTCATTAAGCTCTGACTCCATTTTTGTTGAGAGATCTACACCACCATCAACTCCCCGGCTCTTCCTTTCCCACTT
TTGTACCTCTTCAGGGACTGTCTGATGCATGCCAAACTGCCTTAGGCAACGATCCGGAAGGTGCCTTTCAGCC
TTGTCATAACATATCAACATGGTCTTTGATCTCCCGAGAATGAGGCTGCGTAATATATGTTCTGGTATGACAGT
GTGACTAATGTTTGAGTAAGGACACCAGTCAACGTCAGATGGCTTCATAGAGTCCAATGCTTTTCGGTAGAAT
GCCACATCACGGTTTGATGTTGGACTGCTTTGTTTACCTTTCCACCTAAGTACAAATGGGAAACATTCATGGAT
TGGATCATGATTAAGCTTTGGCCGACCAACATTGAGGTGGTAGTAACTCCAACACTGTAACAGTGTCAAACA
GCCACTAATTGTGCTTTGAGACCTGAGGGAAGCATTCCCAAGCGCTCTGTACAAGAAGGATAAGGCTGCAGC
ACCCCAAGCATACTTGCCAGCTGCATCAAAATTTTCAAACAATGGAAGGTACATGACTGGTACCTTATTGCCA
GTGGTAGTTGAAAAAATTGTGCTACCGACAAGGTAAAGAAGATAAGCACGTGTATGACGCTCAATATCCTCC
ATGGAAGCATCCTCAGGACACTGAGAGAAGGTCTCTTTCAACCAACTAAGCTTCACCATCCCACCACTCGTA
TATCCCGAGTCAGGTGCTTTCCCCAACAACCTGATACAGACTGCTTCACATGAAGTATATGTCACACCTATAA
CAGCTTCACCATCAACTGGTAGTCCAAGCAAGTAGGCGACATCCTCAAGGGTCACAGTCATTTCCCCAACAG
TGAAATGGAATGTGTTAGTTTCTCTCCTCCATCTCTCAACTAGAGCAGAAATGAGTGGGTTATCCAAACTAAT
TGCCGGTATCATTCTTAAATAGCCAAATCCAGCCTTCTTAACCAACTCAATCTGTTTCTCCGTGAGCATCCACT

GATCGAGCTTTGATGTGTGTTCATGGCACCTAAGAGCACTACGCTCCTGCCCTTCCCAAATAGCAGATGATAC
ATGTTTATCTTGATCGTACAACACAACATCATCAATAGGTCCAGGATTAGTTGCATAGTAGTCCACCATCACT
ACTTATGACATATCAGTTAAACCAGCAAGTCAAGGCAATGGGCGATGAATTTATGTCTGTGTCTCTCTTCGTG
TATGTGTGTGTGTAGAGAAAGAGATAGAGTGACAAAGAGAGAGATGAAGGGTCAACGGAGAAGAAGATCG
CCGGAGAAGAGATCGACGGAGATGGAGTTGAGAATCGGCGAGGCACAATTAGG
>TCONS_00055325
CTTAGAAAGAATATCATCCCTTACAGAATAAAAATCTCATCTTTACTGGTCTAAACAAGAACCCCGTAATCAT
TCTGTTGGGCAATGTCCTCTCTAGTTTCATGGGAGACCTGGGGAACAACATCAATTGAAGCATGAGGCTTATC
TTCTGCTACTTCACTGGAGAGCCGGCCATCTTCTTCAGCTAGATGATGGAAGTGTGATTCGTCAAGTCCATTAG
CATCTTCGCAAAGCTCCAAATCATCGACACTGTGAGCCACATGAGATATCCGGCCGTGATCATCCTCACTTAC
TGTAATACATAAATCATCACTGCCCATGTGATATAGGTGAGAGTCATCTGCATCAAAAGTTGCAGCAGGTAA
CCGAGATTGCACAGGTATCCCTGCTGCATAATATTGCTCTATCTCTTCTTTCCGCTTACGTTTGCCCATCCCCTT
CCTTCTTACTCTCTCCTTTCCACGTACTCTCTTTCCAACTTCACTTTGTGAGTTTGTTGCAACAACTACTGTACTT
GCAATCTGATCCCTCAAGCATTCATAAGTAATATATCGGATTCTTGTGACAGATTGTAACTGTTGATCATCCAT
CCCATGAGTTGAAAGGGTGTCTGCTATAAGGGCAATCTCCCTCAGTGCGGCATTCATTCTTTGAAACTCAGAT
GAGATAGGCACAGGCCTCCCCACCAACTTCCTTGTAACTCTCAAATACCACTGCATGTATTCACTTTCTTCCA
CATCTTCCTCTGCCTCCACAATGTAGAGATGGCGATTCGACCATTCATTAAGCTCTGACTCCATTTTTGTTGAG
AGATCTACACCACCATCAACTCCCCGGCTCTTCCTTTCCCACTTTTGTACCTCTTCAGGGACTGTCTGATGCAT
GCCAAACTGCCTTAGGCAACGATCCGGAAGGTGCCTTTCAGCCTTGTCATAACATATCAACATGGTCTTTGAT
CTCCCGAGAATGAGGCTGCGTAATATATGTTCTGGTATGACAGTGTGACTAATGTTTGAGTAAGGACACCAGT
CAACGTCAGATGGCTTCATAGAGTCCAATGCTTTTCGGTAGAATGCCACATCACGGTTTGATGTTGGACTGCT
TTGTTTACCTTTCCACCTAAGTACAAATGGGAAACATTCATGGATTGGATCATGATTAAGCTTTGGCCGACCA
ACATTGAGGTGGTAGTAACTCCAACACTGTAACAGTGTCAAACAGCCACTAATTGTGCTTTGAGACCTGAGG
GAAGCATTCCCAAGCGCTCTGTACAAGAAGGATAAGGCTGCAGCACCCCAAGCATACTTGCCAGCTGCATCA
AAATTTTCAAACAATGGAAGGTACATGACTGGTACCTTATTGCCAGTGGTAGTTGAAAAAATTGTGCTACCGA
CAAGGTAAAGAAGATAAGCACGTGTATGACGCTCAATATCCTCCATGGAAGCATCCTCAGGACACTGAGAG
AAGGTCTCTTTCAACCAACTAAGCTTCACCATCCCACCACTCGTATATCCCGAGTCAGGTGCTTTCCCCAACA
ACCTGATACAGACTGCTTCACATGAAGTATATGTCACACCTATAACAGCTTCACCATCAACTGGTAGTCCAAG
CAAGTAGGCGACATCCTCAAGGGTCACAGTCATTTCCCCAACAGTGAAATGGAATGTGTTAGTTTCTCTCCTC
CATCTCTCAACTAGAGCAGAAATGAGTGGGTTATCCAAACTAATTGCCGGTATCATTCTTAAATAGCCAAATC
CAGCCTTCTTAACCAACTCAATCTGTTTCTCCGTGAGCATCCACTGATCGAGCTTTGATGTGTGTTCATGGCAC
CTAAGAGCACTACGCTCCTGCCCTTCCCAAATAGCAGATGATACATGTTTATCTTGATCGTACAACACAACAT
CATCAATAGGTCCAGGATTAGTTGCATAGTAGTCCACCATCACTACTTATGACATATCTCAGTTAAACCAGCA
AGTCAAGGCAATGGGCGATGAATTTATGTCTGTGTCTCTCTTCGTGTATGTGTGTGTGTAGAGAAAGAGATAG
AGTGACAAAGAGAGAGATGAAGGGTCAACGGAGAAGAAGATCGCCGGAGAAGAGATCGACGGAGATGGA
GTTGAGAATCGGCGAGGCACAATTAGG
>TCONS_00055359
CTCTTCAACATTTTTGTTCACTTCTCTTCTTCCGGTTTTTCAACAAAAGTCTTCCTCAACCAGTCCCATGGCTGG
ACGATCCAAAGGGCGGTGGTAGTGGCGGCGACACCCCAAGTAGCGGCGGCCTGTATATCGGTGGACTGAGG
ACGCAATTTGGGGCGCAGGAACTTGAAGATCCCGGGACTGGTCATCTTCGGCGTTGTTAATTTCTCCGTTCAC
TGTAACTGAAACTGATACGATGAGCCCTCGA
>TCONS_00055374

GAAGTTAGAGAAGACCCGAACAACCGGAGACGCTAGCTTATTTCGAGGGCTCATCGTATCAGTTTCAGTTAC
AGTGAACGGAGAAATTAACAACGCCGAAGATGACCAGTCCCGGGATCTTCAAGTTCCTGCGCCCCAAATTGC
GTCCTCAGTCCACCGATATACAGGCCGCCGCTACTTGGGGTGTCGCCGCCACTACCACCGCCCTTTGGATCGT
CCAGCCATGGGACTGGTTGAGGAAGACTTTTGTTGAAAAACCGGAAGAAGAGAAGTGAACAAAAATGTTGA
AGAG
>TCONS_00055387
AAAAGGTCGGTATAGGTAAGACCTCAGATATATCAAGGTGGTCAGAATGGTCAGAAGAGGTAAAGAAAGGT
TTAGACTCAAAAAATGTGACGTCAGATGACATAAAGTACCTACGAAGATTAGGTGAGTAACAACGATATCCC
TTCTGAACACGAGAATAACCAAGGAAGACACACTTGAGAGCACGAGGAGCTAACTTATCTTTCCCAGGGGCT
AAGTTATGAACGAAACAAGTGCTCCCAAAAATACGAGGGGGAACAGAGTATAAGGGTGACTAGGGAAACA
ATATCGCATACGGAATCTGATTCTGGATGGGAGATGAAGGCATCCGATTAACCAAATAACAAGCTGTGAGAA
CTGCATCGCCCCAAAAACGCAACGGAATATGAGATTCAATGAGAAGTGTGCGAGCAGTCTCAATGATGTGCC
TATTCTTTTTCTCTGCAACCCCATTTTGCTGAGGGGTATAAGGACAAGAGATCTGATGAATAATTCCTTGAGAA
GTCATAAACTGCTGAAATTGAGAGGATAAATATTCTACGGCATTATCACTGCGAAAAGTGCGAATGGAAACA
CCAAATTGATTTTTAATTTCAGCACAAAAATTGAATGATCATCAATGAAACTAACAAAATAACGAAATCCCA
AGGTTGAACTGACTCTACTAGGACCCCATATATCAGAATGAACTAAAGAAAAAACAGACTCTGCATGACTCT
CAATACTACGAGGAAAGGAGGCTCGGGTATGTTTCCCGAGCTGACATGACTCACACTCTAATGTAGATAAAC
TAGACAAACTAGGCACCATCTTCTGAAGCTTGGATAAACTTGGATGTCCTAAACGTCTGTGAATTAGGTCCGG
AG
>TCONS_00055481
TTGACAAGACATACAAACAAATGCAAGGAAGATGGTAATCAAGAAACGCTATGGTATATGGTCCTTACATCT
TGCAGCTTGCCTTTATTTATTGCAATTCTTAATCATCTTCTCTTTCGGGTGGAAGACCACAAGGCAAAAGCATT
TTCTGCATAAAATTGTAGCGTTCTCTTCCAATTGATTCATTTTCTGCAATGGCAAAATACGCCAACATTGGGTA
GTGTCCAACATAGGACGCGTAGCTGTCACGCTGAATGTTTACTGCCCACTCAAATCTAGTCAAGTCGGCATGT
CCAGTTCCAACGTATTTAGCTTGTAAGTGCTCAAGCTGGGAGTTGATATTGAACCTGTCACTCGCCT
>TCONS_00055486
ACCCTGAACCCTGAACCCTGAACCCTAAACCCTAAACCCGAAACCCTAAACCCTAAACCCCTAACCCTAAAC
CCTAAACCCTAAACCCTAAACCCTAAACCCTAAACCCTAAACCCTAAACCCTAAACCCTAAAACCTAAACCC
TAAACCCTAAACCCTAAACCCTAAACCCTAAACCCTAAACCCCTAAACCCAAACCCTAAACCCTAAACCCTA
AACCCTAAACCCGAAACCCTAAACCCTAACC
>TCONS_00055487
AACCCTGAACCCTGAACCCTGAACCCTAAACCCTAAACCCTAAACCCGAAACCCTAAACCCTAAACCCCTAA
CCCTAAACCCTAAACCCTAAACCCTAAACCCTAAACCCTAAACCCTAAACCCTAAACCCTAAACCCTAAAAC
CTAAACCCTAAACCCTAAACCCTAAACCCTAAACCCTAAACCCTAAACCCCTAAACCCAAACCCTAAACCCT
AAACCCTAAACCCTAAACCCGAAACCCTAAACCCTAACC
>TCONS_00055488
TGAACCCTGAACCCTGAACCCTGAACCCTGAACCCTGAACCCTAAACCCTAAACCCTAAACCCTAAACCCTA
AACCCTAAACCCAAAACCCTAAACCCTAAACCCTAAACCCTAAACCCTAAACCCTAAACCCTAAACCCTAA
ACCCTAAACCCTAAAACCTAAACCCTAAACCCTAAACCCTAAACCCTAAACCCTAAACCATAAACCCTAAA
CACTAAACCCTAAACCCTAAACCCTAACCCTTAAACCCTAAACCCTAAACCCTAAACCCTAAACCCTAAACC
CTAAACCCTAAACCCTAAACCCTAAACCCTAAAACCTAAACCCTAAACCCTAAACCTAAAACCCTAAACCC
GAAACCCTAAACCCTAACC

>TCONS_00055489
TGAACCCTGAACCCTGAACCCTGAACCCTGAACCCTGAACCCTAAACCCTAAACCCTAAACCCTAAACCCTA
AACCCTAAACCCAAAACCCTAAACCCTAAACCCTAAACCCTAAACCCTAAACCCTAAACCCTAAACCCTAA
ACCCTAAACCCTAAACCCTAAACCCTAAACCCTAAACCCTAAACCATAAACCCTAAACACTAAACCCTAAA
CCCTAAACCCTAACCCTTAAACCCTAAACCCTAAACCCTAAACCCTAAACCCTAAACCCTAAACCCTAAACC
CTAAACCCTAAACCCTAAAACCTAAACCCTAAACCCTAAACCTAAAACCCTAAACCCGAAACCCTAAACCC
TAACC
>TCONS_00055490
TGAACCCTGAACCCTGAACCCTGAACCCTGAACCCTGAACCCTAAACCCTAAACCCTAAACCCGAAACCCTA
AACCCTAAACCCCTAACCCTAAACCCTAAACCCTAAACCCTAAACCCTAAACCCTAAACCCTAAACCCTAAA
CCCTAAACCCTAAAACCTAAACCCTAAACCCTAAACCCTAAACCCTAAACCCTAAACCCTAACCCTTAAACC
CTAAACCCTAAACCCTAAACCCTAAACCCTAAACCCTAAACCCTAAACCCTAAACCCTAAACCCGAAACCCT
AAACCCTAACC
>TCONS_00055491
TGAACCCTGAACCCTGAACCCTGAACCCTGAACCCTGAACCCTAAACCCTAAACCCTAAACCCTAAACCCTA
AACCCTAAACCCAAAACCCTAAACCCTAAACCCTAAACCCTAAACCCTAAACCCTAAACCCTAAACCCTAA
ACCCTAAACCCTAAAACCTAAACCCTAAACCCTAAACCCTAAACCCTAAACCCTAAACCCTAAACCCTAAAC
CCTAAAACCTAAACCCTAAACCCTAAACCTAAAACCTAAACCTTAAAC
>TCONS_00055492
AACCCTGAACCCTGAACCCTGAACCCTAAACCCTAAACCCTAAACCCTAAACCCTAAACCCGAAACCCTAA
ACCCTAAACCCCTAACCCTAAACCCTAAACCCTAAACCCTAAACCCTAAACCCTAAACCCTAAACCCTAAAC
CCTAAACCCTAAAACCTAAACCCTAAACCCTAAACCCTAAACCCTAAACCCTAAACCCTAAACCCTAAACCC
TAAACACCAACCACCTAAACTACCCTAACTCCTAAACCACTAAACCCTAAACCCTAAACCCTAAACCCTAAA
TCCTAAACCCTAAA
>TCONS_00055493
TGAACCCTGAACCCTGAACCCTGAACCCTGAACCCTGAACCCTAAACCCTAAACCCTAAACCCTAAACCCTA
AACCCTAAACCCAAAACCCTAAACCCTAAACCCTAAACCCTAAACCCTAAACCCTAAAACCCTAAACCCTA
AACCCTAAACACTAAACCCTAAACCCTAAACCCTAAACCCTAAACCCTAAACCCTAAACCCTAAACCCTTAA
>TCONS_00055494
TGAACCCTGAACCCTGAACCCTGAACCCTGAACCCTGAACCCTAAACCCTAAACCCGAAACCCTAAACCCTA
AACCCCTAACCCTAAACCCTAAACCCTAAACCCTAAACCCTAAACCCTAAACCCTAAACCCTAAACCCTAAA
CCCTAAAACCTAAACCCTAAACCCTAAACCCTAAACCCTAAACCCTAAACCCTAAACCCTAAACCCTAAACC
CTAAACCCTAAACCCTAAACCCTTAA
>TCONS_00055495
AAACCTAAACCCTAAACCCGAAACCCTAAACCCTAAACCCCTAACCCTAAACCCTAAACCCTAAACCCTAA
ACCCTAAACCCTAAACCCTAAACCCTAAACCCTAAACCCTAAAACCTAAACCCTAAACCCTAAACCCTAAAC
CCTAAACCCTAAACCCTAAACCCCTAACCCTTAAACCCTAAACCCTAAACCCTAAACCCTAAACCCTAAACC
CTAAACCCTAAACCCTAAACCCTAAAACCCTAAACCCTAAACCCTAAACCCTAA
>TCONS_00055496
CCTGAACCCTAAACCCTAAACCCTAAACCCTAAACCCTAAACCCCTAAACCGAAACCCTAAACCCTAAACCC
CTAACCCTAAACCCTAAACCCTAAACCCTAAACCCTAAACCCTAAACCCTAAACCCTAAACCCTAAACCCTA

AAACCTAAACCCTAAACCCTAAACCCTAAACCCTAAACCCTAAACCCTAAACCCCTAAACCCAAACCCTAA
ACCCTAAACCCTAAACC
>TCONS_00055655
TCCCGCTAAGGAAAGTTTCAGATGGTGTGATCTCGATCTTATCAAAGGTGATACTTTCTGTTGATCTTTTTTTCC
TTCATCCAGAATTAGGTACGACTCCTAGATCTGGTGAATACCAAGGCAGCAAGGCAATATGAATGAAATGAT
TCAAAAGAAAAACTATGCGAAGGAATTCATCCAGATAGACAAAATTATTGAATATGCTATAAATTGAGAGG
GTAGTTTCCATTATGTCTATGAATATTTAGAAATAAGTTCTCTTTTTGTCTTAACTAGTTATTTCATTTGGTTCCT
TATTACATTGTCTATATGAAAATACGAACACCCTTTCATGCTGAAGCCTAAAAGTTTCCAGAGCAAGTCCCTT
TATTCTTTTCCTCTCTTTCTTTTCCCTATTTCCAGAAATAAATATGTCAACAGTTTAAACAGTGTAGTGATGAT
GAATCTTGCTAGTTACTATTTGCTTGTTTGGAATCGGAGTCTCGTTTGTGAATTTTGAAGTTCTTTTCAATTTAG
GAACTCGAATTGTTTGTTTGGTTATTGATAATTAAAGATTGCTGATATTCTCTAAGTTGAAATTATAATCTGTG
ATTTTACAGCTCTAACATGTAAACACTTATCATGTAATAAATATTCTTCAGGAAATAATTCGTCAGCGAAGAG
AGGTTTAGATAAAGCAATTTTGAAACTTACTGAGACCAAATTAGTTGAAAAAACCTTAATGAAAACTTAGAA
ATATTCTATTTCTGTGTATGTTTACATCGTGACGGTAATGTATAGGTAACCTTGTCTTGTCTTTATGATAAGTTT
CACTGTATCTTTGTCAAATATGTTTTATGTTGAAAGTCAGTCTTAGCTAGAGATAGTTGCTCATCATTGTCAATT
TGTCATTCTGGCACTAGTTACACTGGCATTTCCCTATATCCATATTTAGACATTTGATGTCAAGGCAAATCATA
GTTTAGCAACATCTGCACCTCTTAACTCCTGCTGTTTCTGTTCACGGTATTTGTATTCTGTTTCAGAAATTCTTC
AGATAAGCCTGAAATGGAAAACAAAAAGCAATTGGATTGAG
>TCONS_00055656
CCCGCTAAGGAAAGTTTCAGATGGTGTGATCTCGATCTTATCAAAGGTGATACTTTCTGTTGATCTTTTTTTCCT
TCATCCAGAATTAGGTACGACTCCTAGATCTGGTGAATACCAAGGCAGCAAGGCAATATGAATGAAATGATC
CAAAAGAAAAACCATGCGAAGGAGTTCATCCAGATAGACAAAATTGTTGAATATGCTCTAAATTGAGAGGG
TAGTTTCCATTATGTCTATGAATATTTAGAAATAAGTTCTCTTTTTGTCTTAACTAGTTATTTCATTTGGTTCCTT
ATTACATTGTCTATATGAAAATACGAACACCCTTTCATGCTGAAGCCTAAAAGTTTCCAGAGCAAG
>TCONS_00055658
AAATTTTCCTTGTGCTGTTTTATCTTTCTTGTTCTATTGTATTCATGTTCTATGGCTCCCACCCTCTCACTAAAGT
GCTGAAATACTTATATAGGATTTAGTTGTTGCTGTTGAGAAGATGAACTCATTCCTCCGCACGAAAAAGACTA
GGCCATACTCCTTCCAACAAGATGAAATTGCGGTATTAGATTTAGGGCATAAAGCTAGATCTTATCTGCTGCT
GCTCATAAAAATGAGTCGAGTAATAGTAGAGACAAGTGGTGGCTTGATTTGTTATAGAATCTTGTAGTGGGC
AATGAGTGCAGAGATGAAACCCATTGATGTTAACAAGTTAGGAATATTCAGCTGAAGTGTGTGCTTTTAATG
ATTTGATCTTCTTGATCAGAAAAAAGGGCATAACAATATTTAATGCCCATTTAGTATGGTATTAAAGGCTCAT
TAATTTGCATCTTGAAGTGCT
>TCONS_00056051
AAGAAGTCACGATTATCAAGAAAGCTTCGAATATAAGATATGATCTTTGCTCGGGTCTTAAATATGTACCGA
ATCTCTAAGTTCAGCATCAAATCCAAATAACGCTGTCTATATCGTGTTTCCTGATCTTTCAAAACATATGTATC
TGGATTTCGGCCACTTCCCGGGACCCATGCTTCAATTTCTTAGTTGTATCCGTCTGGGCGCCTTTCTGCCGTGG
CATCATATGAAGACAAT
>TCONS_00056052
GTGCTTAAGCATGTGAAGGAGTGATAATGAGAGCAACTTTTCTCTTCCATCGAAACTTGTTTTTCTAACACCAT
GACCAAATCTATCACAGAGAACATCTGCCATAAACTAAGAAGACCTAGCGAATCCCAAACCTGGACACCCA
GTAATTCCGACAATATCTCCACGCTTCACACCAGAATGAAATTTAATAAACGCGTCTTCATTCAATTCAGAAT

CCCTCGCACTTGCCATAACTTGAACTTTGGCACCTCCACCATGTAAATCATAGAAAAGCAGCTTTGCAGAAG
ACGCGCGTTTATTCATTATCC
>TCONS_00056060
ATTTTATTGAGTTTTCTTTTCTCCATCCCAGCAATAAAAAGCCTCGGGAGGAATCTGCCAAGCCTCATGATGG
ACAAGCAAAACCTCAGGATGGACCTACAACACAAGATGTGGAAAAGCAGGTGTCACAGCTCACAATGAAG
ATCATAAATATTTGACGTTGAGTTTCTGCTGCTCCTGGAGTAGCTTCTTTTTGCACTATCTTTTTGACGTTTTGAT
TTATGATAATTGTCAATTGTATGGCAAATGCTAGCCTTAATTTGTTAGTATTGGAAACTTTGGTCATA
>TCONS_00056061
ATTTTATTGAGTTTTCTTTTCTCCATCCCAGCAATAAAAAGCCTCGGGAGGAATCTGCCAAGCCTCATGATGG
ACAAGCAAAACCTCAGGATGGACCTACAACACAAGGAGAGAACTACCTACTAACGTGCTCATGCATTGGTA
CAGATGTGGAAAAGCAGGTGTCACAGCTCACAATGAAGATCATAAATATTTGACGTTGAGTTTCTGCTGCTCC
TGGAGTAGCTTCTTTTTGCACTATCTTTTTGACGTTTTGATTTATGATAATTGTCAATTGTATGGCAAATGCTAG
CCTTAATTTGTTAGTATTGGAAACTTTGGTCATA
>TCONS_00056097
GTTCATCCTTTTTCGCCTTCTACCTTATTTTGCAGATTCGTTTGCTTCCGTAGTAGTACTTTTATTTTCTATCGGG
CATTGGGATACATAAGGTTTCAGCTTGAAGTCAGTCAAATCTGGAACTACATAGTTAGGCAGCTTCTCTTGCA
TAACAACATAACCACCTTTGCGAGTGTGAAAACCAGTAGGCTTGCAATTCTTTCCCTTGTAGTAGCCCCGAGG
AACCCTTTTTGAAGTAAGAATATCGAGAGACGAAGTACGTTTTCTTCTGAAAGTTCTCCCTAAACCTAATAGC
AGTCCCAGCGGCATTGTCTTCTAATTTAAAA
>TCONS_00056134
GGGTAACCAACTTCATTAGGAGGCAAGCTATAACTCTTGAAAATAGGATCTTGATTCACACTTCCTGGATTCT
TAATGTCTTGCCAACGTCGAATTTCGATCGTAGTGAAACAATATGAACTCGATCACGAACAGAGTTGATGAA
GATGCAAAGTACTCAGATTTTCCAGCATCATACCATTTTGGTACGTTAAGGAGTCCAGCACTAGTGAGAACCT
CAGGCAGTAGCATTCTAGCAACACCTAACATGGCCCATAGACCGTTCACTAGCTCGGCTTGAACGAACCATT
TCAAGTTCTCTGGGTCCTCAGCGAGCCCCAGTGGATCGAAACCATTGTCACCAGCGAGTCTGCCATCAAGAT
AAGTAGGAGAGGTTAAGCCTGGAAGCCATTCACCTTTCTTAGCTTCAACTTTAAATGAGTTGTAAGATGAAGT
TGATGGTTTAAAAAACACCTCCCTATTTAGTTTACCAGATGCTCCACTGAGGAACCTTGTCTTGGCGGCGGAT
GGCCGGAAGACGGCGGCGGAGGCTTGCGATGTGACGGTGGCCATTTGGG
>TCONS_00056135
CGAATGTTTGGATAATGGTATTATGCCATGGGTCAGAAAGATGCTGCAACAAGTTGTCAAATGGTCCTTTTCC
TGTCACATTGTGCTGCACTATAAATCCCAAAAATGCCAACATTGCCAATCTCCCTGCCATCAAGATAAGTAGG
AGAGGTTAAGCCTGGAAGCCATTCACCTTTCTTAGCTTCAACTTTAAATGAGTTGTAAGATGAAGTTGATGGT
TTAAAAAACACCTCCCTATTTAGTTTACCAGATGCTCCACTGAGGAACCTTGTCTTGGCGGCGGATGGCCGGA
AGACGGCGGCGGAGGCTTGCGATGTGACGGTGGCCATTTGGG
>TCONS_00056181
CCCCCTTCCTAGTCTATTAATAACAAAACGGATTTTTCCAATGTATAAAATAAAAATTCCAATGGCTTTGGCT
ACTCTAACCTTCCCGACCACGATTTTTTCTTTTTTTTTTTAGGTATTTCACTGCGAAATAAGAAAGAAATAAA
AAATTGTATTTTCCTAGGTATCAAAAATCTAGTAAATAAAAGAAATCAAAAAATAAATAGTGGGTTCCTTCGT
TTCTATGGTTACTTCTTAAACGGTGAGGTCTTCTCTATACACCGGAGCCTTTACTTTATACTTTAATTTAATATT
TAATCAACTAATTGATGTTATTGGGAACTTGTATAGTTCACACTCTTTGGCTCTACCCATGAATTATCCAGTAA
TAGGTCTTTCACAATCAGATCTACCTATACAGTAACGGTATTTAATTATGAAAGTTTGCTGGGTAGCTAACCCT
CTTAGTCCGTTCTTGCCAGAGTGGGAG

>TCONS_00056182
TTCCTAGTCTATTAATAACAAAACGGATTTTTCCAATGTATAAAATAAAAATTCCAATGGCTTTGGCTACTCTA
ACCTTCCCGACCACGATTTTTTCTTTTTTTTTTTTAGGTATTTCACTGCGAAATAAGAAAGAAATAAAAAATTG
TATTTTCCTAGGTATCAAAAATCTAGTAAATAAAAGAAATCAAAAAATAAATAGTGGGTTCCTTCGTTTCTAT
GGTTACTTCTTAAACGGTGAGGTCTTCTCTATACACCGGAGCCTTTACTTTATACTTTAATTTAATATTTAATCA
ACTAATTGATGTTATTGGGAACTTGTATAGTTCACACTCTTTGGCTCTACCCATGAATTATCCAGTAATAGGTC
TTTCACAATCAGATCTACCTATACAGTAACGGTATTTAATTATGAAAGTTTGCTGGGTAGCTAACCCTCTTAGT
CCGTTCTTGCCAGAGTGGGAG
>TCONS_00056183
TTCCTAGTCTATTAATAACAAAACGGATTTTTCCAATGTATAAAATAAAAATTCCAATGGCTTTGGCTACTCTA
ACCTTCCCGACCACGATTTTTTCTTTTTTTTTTTTAGGTATTTCACTGCGAAATAAGAAAGAAATAAAAAATTG
TATTTTCCTAGGTATCAAAAATCTAGTAAATAAAAGAAATCAAAAAATAAATAGTGGGTTCCTTCGTTTCTAT
GGTTACTTCTTAAACGGTGAGGTCTTCTCTATACACCGGAGCCTTTACTTTATACTTTAATTTAATATTTAATCA
ACTAATTGATGTTATTGGGAACTTGTATAGTTCACACTCTTTGGCTCTACCCATGAATTATCCAGTAATAGGTC
TTTCACAATCAGATCTACCTATACAGTAACGGTATTTAATTATGAAAGTTTGCTGGGTAGCTAACCCTCTTAGT
CCGTTCTTGCCAGAGTGGGAG
>TCONS_00056184
TTCCTAGTCTATTAATAACAAAACGGATTTTTCCAATGTATAAAATAAAAATTCCAATGGCTTTGGCTACTCTA
ACCTTCCCGACCACGATTTTTTCTTTTTTTTTTTTAGGTATTTCACTGCGAAATAAGAAAGAAATAAAAAATTG
TATTTTCCTAGGTATCAAAAATCTAGTAAATAAAAGAAATCAAAAAATAAATAGTGGGTTCCTTCGTTTCTAT
GGTTACTTCTTAAACGGTGAGGTCTTCTCTATACACCGGAGCCTTTACTTTATACTTTAATTTAATATTTAATCA
ACTAATTGATGTTATTGGGAACTTGTATAGTTCACACTCTTTGGCTCTACCCATGAATTATCCAGTAATAGGTC
TTTCACAATCAGATCTACCTATACAGTAACGGTATTTAATTATGAAAGTTTGCTGGGTAGCTAACCCTCTTAGT
CCGTTCTTGCCAGAGTGGGAG
>TCONS_00056185
CCTAGTCTATTAATAACAAAACGGATTTTTCCAATGTATAAAATAAAAATTCCAATGGCTTTGGCTACTCTAA
CCTTCCCGACCACGATTTTTTCTTTTTTTTTTTTAGGTATTTCACTGCGAAATAAGAAAGAAATAAAAAATTGT
ATTTTCCTAGGTATCAAAAATCTAGTAAATAAAAGAAATCAAAAAATAAATAGTGGGTTCCTTCGTTTCTATG
GTTACTTCTTAAACGGTGAGGTCTTCTCTATACACCGGAGCCTTTACTTTATACTTTAATTTAATATTTAATCAA
CTAATTGATGTTATTGGGAACTTGTATAGTTCACACTCTTTGGCTCTACCCATGAATTATCCAGTAATAGGTCT
TTCACAATCAGATCTACCTATACAGTAACGGTATTTAATTATGAAAGTTTGCTGGGTAGCTAACCCTCTTAGTC
CGTTCTTGCCAGAGTGGGAG
>TCONS_00056203
GGCAGGCTCCCACTCTGGCAAGAACGGACTAAGAGGGTTAGCTACCCAGCAAACTTTCATAATTAAATACCG
TTACTGTATAGGTAGATCTGATTGTGAAAGACCTATTACTGGATAATTCATGGGTAGAGCCAAAGAGTGTGAA
CTATACAAGTTCCCAATAACATCAATTAGTTGATTAAATATTAAATTAAAGTATAAAGTAAAGGCTCCGGTGT
ATAGAGAAGACCTCACCGTTTAAGAAGTAACCATAGAAACGAAGGAACCCACTATTTATTTTTTGATTTCTTT
TATTTACTAGATTTTTGATACCTAGGAAAATACAATTTTTTATTTCTTTCTTATTTCGCAGTGAAATACCTAAAA
AAAAAAAAGAAAAAATCGTGGTCGGGAAGGTTAGAGTAGCCAAAGCCATTGGAATTTTTATTTTATACATTG
GAAAAATCCGTTTTGTTATTAATAGACTAGGAAGGGGGGAAAGAATAACTGAAAGAAAGGAAATCGATTAG
TTATTCGTCAAAGTTTCATTTATTCAATGACCAGAATTAA
>TCONS_00056204

GGCAGGCTCCCACTCTGGCAAGAACGGACTAAGAGGGTTAGCTACCCAGCAAACTTTCATAATTAAATACCG
TTACTGTATAGGTAGATCTGATTGTGAAAGACCTATTACTGGATAATTCATGGGTAGAGCCAAAGAGTGTGAA
CTATACAAGTTCCCAATAACATCAATTAGTTGATTAAATATTAAATTAAAGTATAAAGTAAAGGCTCCGGTGT
ATAGAGAAGACCTCACCGTTTAAGAAGTAACCATAGAAACGAAGGAACCCACTATTTATTTTTGATTTCTTT
TATTTACTAGATTTTTGATACCTAGGAAAATACAATTTTTTATTTCTTTCTTATTTCGCAGTGAAATACCTAAAA
AAAAAAAAGAAAAAATCGTGGTCGGGAAGGTTAGAGTAGCCAAAGCCATTGGAATTTTTATTTTATACATTG
GAAAAATCCGTTTTGTTATTAATAGACTAGGAAGGGGGGAAAGAATAACTGAAAGAAAGGAAATCGATTAG
TTATTCGTCAAAGTTTCATTTATTCAATGACCAGAATTAA
>TCONS_00056205
GGCAGGCTCCCACTCTGGCAAGAACGGACTAAGAGGGTTAGCTACCCAGCAAACTTTCATAATTAAATACCG
TTACTGTATAGGTAGATCTGATTGTGAAAGACCTATTACTGGATAATTCATGGGTAGAGCCAAAGAGTGTGAA
CTATACAAGTTCCCAATAACATCAATTAGTTGATTAAATATTAAATTAAAGTATAAAGTAAAGGCTCCGGTGT
ATAGAGAAGACCTCACCGTTTAAGAAGTAACCATAGAAACGAAGGAACCCACTATTTATTTTTGATTTCTTT
TATTTACTAGATTTTTGATACCTAGGAAAATACAATTTTTTATTTCTTTCTTATTTCGCAGTGAAATACCTAAAA
AAAAAAAAGAAAAAATCGTGGTCGGGAAGGTTAGAGTAGCCAAAGCCATTGGAATTTTTATTTTATACATTG
GAAAAATCCGTTTTGTTATTAATAGACTAGGAAGGGGGGAAAGAATAACTGAAAGAAAGGAAATCGATTAG
TTATTCGTCAAAGTTTCATTTATTCAATGACCAGAATTAA
>TCONS_00056206
GGCAGGCTCCCACTCTGGCAAGAACGGACTAAGAGGGTTAGCTACCCAGCAAACTTTCATAATTAAATACCG
TTACTGTATAGGTAGATCTGATTGTGAAAGACCTATTACTGGATAATTCATGGGTAGAGCCAAAGAGTGTGAA
CTATACAAGTTCCCAATAACATCAATTAGTTGATTAAATATTAAATTAAAGTATAAAGTAAAGGCTCCGGTGT
ATAGAGAAGACCTCACCGTTTAAGAAGTAACCATAGAAACGAAGGAACCCACTATTTATTTTTGATTTCTTT
TATTTACTAGATTTTTGATACCTAGGAAAATACAATTTTTTATTTCTTTCTTATTTCGCAGTGAAATACCTAAAA
AAAAAAAAGAAAAAATCGTGGTCGGGAAGGTTAGAGTAGCCAAAGCCATTGGAATTTTTATTTTATACATTG
GAAAAATCCGTTTTGTTATTAATAGACTAGGAAGGGGGGAAAGAATAACTGAAAGAAAGGAAATCGATTAG
TTATTCGTCAAAGTTTCATTTATTCAATGACCAGAATTAA
>TCONS_00056207
GGCAGGCTCCCACTCTGGCAAGAACGGACTAAGAGGGTTAGCTACCCAGCAAACTTTCATAATTAAATACCG
TTACTGTATAGGTAGATCTGATTGTGAAAGACCTATTACTGGATAATTCATGGGTAGAGCCAAAGAGTGTGAA
CTATACAAGTTCCCAATAACATCAATTAGTTGATTAAATATTAAATTAAAGTATAAAGTAAAGGCTCCGGTGT
ATAGAGAAGACCTCACCGTTTAAGAAGTAACCATAGAAACGAAGGAACCCACTATTTATTTTTGATTTCTTT
TATTTACTAGATTTTTGATACCTAGGAAAATACAATTTTTTATTTCTTTCTTATTTCGCAGTGAAATACCTAAAA
AAAAAAAAGAAAAAATCGTGGTCGGGAAGGTTAGAGTAGCCAAAGCCATTGGAATTTTTATTTTATACATTG
GAAAAATCCGTTTTGTTATTAATAGACTAGGAAGGGGGGAAAGAATAACTGAAAGAAAGGAAATCGATTAG
TTATTCGTCAAAGTTTCATTTATTCAATGACCAGAATTAA
>TCONS_00056321
CCCTCACTGAGGTGCTCTTCTACTATTTTCTGATTACAAGCAGCATGATCATTAAAAAATCAAGATCATTCAG
AAACTCACCTATAGCTTCAACCAATCGCAGAACCATTTGTCCTGTGTGAATTCCTTTGACACAGCTGCTCCAA
TAAACTTTGTAAGCATCAAAGATGTAGACACAACCATTGAGGAAGCAGAGCAGAGAACCAGACAATGAACC
TCCGATAGCAAGGAGTGCGAGAAAACGGAAGTCAAATATCGCCCTTTCAATAGTTGATTCAGTGTGCTGAAC
AAATCTAACAACTGGGTTTCCATTAGGATTTGCCAAAGCATAGTTGAACCCGTTATCCGGGGTTTTAGAAGA
>TCONS_00056378

ATAACCATAGGTTCCAACAACTCGTGTTGCAGAGGCTTCTCCGTCATTCGTTATCCCCATAAGTTTTGACAAA
ACAAAATCTGAAATCTTTGCTCTAAAAGAATCGTCAAGAAGGATGTTGCTTGTCTTTATATCTCGATGCACAT
AATGCGGCTTGGTGTGCTCATGAATGTATTCCAGACCTCTAGCAGCATCAAGTGCAATTT
>TCONS_00056486
ACCCTAAACCCCTAAACCCTAAACCCAAACCCCTAACCCTAAACCCCTAAACCCTAAACCCTAAACCCTAAA
CCCTAAACCCTAAACCCTAAACCCTAAACCCTAAACCCTAAACCCTAAACCCTAAACCCTAAACCCTAAACC
CTAAACCCTAAACCCTACCCCCTAAACCCTAAACCCTAAACCCTAAACCCTAAACCCTAACCCCCAAACCCT
AAACCCTAAACCCTAAACCCTAAACCCTAAACCCTAAACCCTAAACCCTAAACCCTAAACCCTAAACCCTAA
ACCCTAAAACCCTAAACCCTAAACCCTAAACCCTAAACCCTAAACCCTAAACCCTAAACCCTAAACCCTAAA
CCCTAAACCCTAAAACCCTAAAACCCTAAACCCTAAAACCCTCCAAACCCTAAACCCAAACCCTAAACCCTA
AACCCTAACCTAAACCCTAACCCACCCTAAACCCTTAAACCCTAAACCCTAAACCCTAAACCCTAAACCCTA
AACCCTAAACCCTAAACCCAAACCCTAAACCCTAAACCCTAAACCCTAAAACCCTAAAACCCTAAAACCCT
AAACCCTAAACCCTAAACCCTAAACCCTAAACCCTAAACCCTAAACCCTAAACCCTAAACCCTAAACCCTAA
ACCCTAAACCCTAAACCCTAAACCCTAAACCCTAAACCCAAAACCCTAAACCCTAAACCCTAAACCCTAAA
ACCCTAA
>TCONS_00056487
ACCCTAAACCCCTAAACCCTAAACCCAAACCCCTAACCCCTAAACCCTAAACCCTAAACCCTAAACCCTAAC
CCCTAAACCCTAAACCCTAAACCCTAAACCCTAAACCCTAAACCCTAAACCCTAAACCCTAAACCCTAAACC
CTAAACCCTAAACCCTAAACCCTAAACCCTAAACCCTAAACCCTAAAACCCTAAACCCTAAACCCTAAAACC
CTAAACCCTAAACCCTAAACCCTAAACCCTAAACCCTAAACCCGAAACCCTAAACCCTAAACCCTAAACCTT
AAACCCTAAACCCTAAACCCTAAACCCTAAACCCTAAAAACCCTAAACCCTAAACCCTAAACCCTTAAACCC
TAAAACCTAAACCTAAAACCCTAAACCCTAAACCCTAAACC
>TCONS_00056488
ACCCTAAACCCCTAAACCCTAAACCCAAACCCCTAACCCCTAAACCCTAAACCCTAAACCCTAAACCCTAAA
CCCTAAACCCTAAACCCTAACCCCTAAACCCTAAACCCTAAACCCTAAACCCTAAACCCTAAACCCTAAACC
CTAAACCCTAAACCCTAAACCCTAAACCCTAAACCCTAAACCCTAAACCCTAAACCCTAAACCCTAAAACCC
TAAACCCTAAACCCTAAAACCCTAAACCCTAAACCCTAAACCCTAAACCCTAAACCCTAAACCCGAAACCCT
AAACCCTAAACCCTAAACCTTAAACCCTAAACCCTAAACCCTAAACCCTAAACCCTAAAAACCCTAAACCCT
AAACCCTAAACCCTTAAACCCTAAAACCTAAACCTAAAACCCTAAACCCTAAACCCTAAACC
>TCONS_00056489
AAACCCCTAAACCCTAAACCCAAACCCCTAACCCTAAACCCCTAAACCCTAAACCCTAAACCCTAAACCCTA
AACCCTAAACCCTAAACCCTAAACCCTAAACCCTAAACCCTAAACCCTAAACCCTAAACCCTAAACCCTAAA
CCCTAAACCCTAAACCCTAAACCCTAAACCCTAAACCCTAAACCCTAAACCCTAAACCCTAAACCCTAAACC
CTAAACCCTAAACCCAAAACCCCAAACCCTAAACCCTAAACCCTAAAACCTAAACCCTAAAAACC
>TCONS_00056490
AAACCCCTAAACCCTAAACCCAAACCCCTAACCCTAAACCCCTAAACCCTAAACCCTAAACCCTAAACCCTA
AACCCTAAACCCTAAACCCTAAACCCTAAACCCTAAACCCTAAACCCTAAACCTAAAACCCTAAACCCTAA
ACCCTAAACCCTAAACCCTAAACCCTAAACCCTAAACCCTCAACCCTAAACCCTAAACCCTAAACCCTAAAC
CCTAAACCCTAAACCCTAAACCCTAAACCCTAAACCCTAAACCCTAAACCCTAAACCCCAAACCCTAAACCC
TAAACCCTAAACCCTAAACCCTAACCCCTAAACCCTAAACCCTAAACCCTAAACCCTAAACCCTAAACCCTA
AACCCTAAACCCTAAACCCTAAACCCTAAACCCTAAACCCTAAACCCTAAACCCTAAACCCTAAACCCTAAA
CCCTACACCCTAAACCCTAAACCCTAAAACCCTAAACCCTAAACCC

>TCONS_00056491
AAACCTTAAAACCTTAAACCCAAACCCTAAACCCTAAACCCTAAAACCCTAAACCCTAACCCCTAAACCCTA
AACCCTAACCCCTAACCCCTAAACCCTAAACCCTAAACCCTAACCCCTAACCCCTAAACCCTAAACCCTAAA
CCCTAAACCCTAAACCCTAAACCCTAAACCCTAAACCCTAAACCCTAAACCCTAAACCCTAAACCCTAAACC
CTAAACCCTAAACCCTAAACCCTAAACCCTAAACCCTAAACCCTTAAACCCTAAACCCTAAACCCTAAACCC
TAAACCCTAAACCCTAAACCCTAAAACCCTAAACCCTAAACCCTAAACCCTAAACCACTAACCCTAAACCTA
AAACCCTAAACCCTAAAA
>TCONS_00056492
ACCCTAAACCCTAAACCCTAAAACCCTAAACCCTAACCCCTAAACCCTAACCCTAAACCCCTAAACCCTAAA
CCCTAAACCCTAAACCCTAAACCCTAAACCCTAAACCCTAAACCCTAAACCCTAAACCCTAAACCCTAAACC
CTAAACCCTAAACCCTAAACCCTAAACCCTAAACCCTAAACCCTAAACCCTAAACCCTAAACCCTAAACCCT
AAACCCTAAACCCTAAACCCTAAACCCTAAACCCAAAACCCTAAACCCTAAACCCTAAACCCCTAACCCTA
AACCCTAAACCCTAAACCCTAAACCCTAAACCCTAAACCCTAAACCCTAAACCCTAAACCCTAAACCCTCAA
CACTAAACCCTAAACCCTAAACCCAAACCCCTAAACCCTAAACCCTAAACCC
>TCONS_00056493
ACCCTAAACCCTAAACCCTAAAACCCTAAACCCTAACCCCTAAACCCTAACCCTAAACCCCTAAACCCTAAA
CCCTAAACCCTAAACCCTAAACCCTAAACCCTAAACCCTAAACCCTAAACCCTAAACCCTAAACCCTAAACC
CTAAACCCTAAACCCTAAACCCTAAACCCTAAACCCTAAACCCTAAACCCTAAACCCTAAACCCTAAACCCT
AAACCCTAAACCCTAAACCCTAAACCCTAAACCCAAAACCCTAAACCCTAAACCCTAAACCCTAAACCCTA
AACCCTAAAACCAAAACCCTAAACCCTAAACCCTAAACCCTAAACCCTAAACCCTAAACCCTAAACCCTAA
ACCATAAACCCTAAACCCTAAACCCTAAACCCTAAACCCTAAACCCTAACCCCTAAACCCTAAACCCTAAAC
CCTAAACCCTAAACCCTAACCCCTAAACCCTAAACCCTAAACCCTAAACCCTAAACCCTAAACCCTACACCC
TAAACCCTAAACCCTAAACCCTAAACCCTAAACCCTAAACCCTAAACCCTAAACCCTAAACCCTAAACCCTA
AACCCTAAACCCTAAACCCTAAACCCTTAA
>TCONS_00056494
ACCCTAAACCCTAAACCCTAAAACCCTAAACCCTAACCCCTAAACCCTAACCCTAAACCCCTAAACCCTAAA
CCCTAAACCCTAAACCCTAAACCCTAAACCCTAAACCCTAAACCCTAAACCCTAAACCCTAAACCCTAAACC
CTAAACCCTAAACCCTAAACCCTAAACCCTAAACCCTAAACCCTAAACCCTAAACCCTAAACCCTAAACCCT
AAACCCTAAACCCTAAACCCTAAACCCTAAACCCAAAACCCTAAACCCTAAACCCTCAACCCTAAACCCTA
ACCCCTAAACCCTAAACCCTAAACCCTACACCCTAAACCCTAAACCCTAAACCCTAAACCCTAAACCCTAAA
CCCTAAACCCTAAACCCTAAACCCTAAACCCTAAACCCTAAACCCTAAACCCTAAACCCTTAA
>TCONS_00056495
AAACCCTAAACCCTAAAACCCTAAACCCTAACCCCTAAACCCTAAACCCTAAACCCTAAACCCTAACCCCTA
AACCCTAACCCCTAAACCCTAAACCCTAAACCCTAAACCCTAAACCCTAAACCCTAAACCCTAAACGCTAAA
CCCTAAACCCTAAACCCTAAACCCTAAACCCTAAACCCTAAACCCTAAACCCTAAACCCTAAACCCTAAACC
CTAAACCCTAAACCCTAACCCCTAAACCCTAAACCCTAAACCCTACACCCTAAACCCTAAACCCTAAACCCT
ACACCCTAAACCCTAAACCCTAAACCCTAAACCCTAAACCCTAAACCCTAAACCCTAAACCCTAAA
>TCONS_00056496
AAACCCCTAAACCCTACCCTAAAACCCAAACACCCTAACCCTAAACCCCTAAACCCTAAACCCTAAACCCTA
AACCCTAAACCCTAAACCCTAAACCCTAAACCCTAAACCCTAAACCCTAAACCCTAAACCCTAAACCCTAAA
CCCTAAACCCTAAACCCTAAACCCTAAACCCTAAACCCTAAACCCTAAACCCCTAAACCCTAAACCCTAAAC
CCTAAACCTAA

>TCONS_00056497
AAAACCCTAAACCCTAAACCCTAACCCCTAACCCTAAACCCTAAACCCCTAACCCCTAAACCCTAAACCTAA
CCCCCTAAACCCTAAACCCTAAACCCTAAACCCTAAACCCTAAACCCTAAACCCTAAACCCTAAACCCTAAA
CCCTAAACCCTAAACCCTAAACCCTAAACCCTAAACCCTAAACCCCTAAACCCTAAACCCTAAACCCTAAAC
CTAA
>TCONS_00056498
AAACCCTAAACCCTAAACCCTAACCCCTAACCCCTAAACCCTAAACCCTAAACCCTAAACCCTAAACCCTAA
CCCCTAAACCCTAAACCCTAAACCCTAAACCCTAAACCCTAAACCCTAAACCCTAAACCCTAAACCCTAAAC
CCTAAACCCTAAACCCTAAACCCTAAACCCTAAACCCTAAACCCCTAAACCCTAAACCCTAAACCCTAAACC
TAA
>TCONS_00056499
AAACCCTAAACCCTAAACCCTAACCCCTAACCCCTAAACCCTAAACCCTAAACCCTAAACCCTAAACCCTAA
ACCCTAAACCCTAACCCTAAACCCTAAACCCTAAACCCTAAACCCTAAACCCTAAAACCCTAAACCCTAAAC
CCTAAACCCTAAACCCTAAACCCTAAACCCTAAACCCTAAACCCTAAACCCTAAACCCTAAACCCTAAACCC
TAAACCCTAAACCCTAAACCCTAAACCCTAAACCCTAAACCCTAAACCCTAAACCCTAAACCCAAAACCCTA
AACCCTAAACCCTAAACCCTAAACCCTAACCCCTAAACCCTAAACCCTAAACCCTAAACCCTAAACCCTAAA
CCCTAAACCCTAAACCCTAAACCCTAAACCCTAAACCCTAAACCCTAAACCCCTAAACCCTAAACCCTAAAC
CCTAAACCTAA
>TCONS_00056500
ACCTAAACCCTAACCCCTAAACCCTAAACCCTAAACCCTCAACCCTAAACCCTAAACCCTAAACCCTAACCC
CTAAACCCTAAACCCTAAACCCTAAACCCTAAACCCTAAACCCTAAACCCTAAACCCTAAACCCTAAACCCT
AAACCCTAAACCCTAAACCCTAAACCCTAAACCCTAAACCCCTAAACCCTAAACCCTAAACCCTAAACCTAA
>TCONS_00056501
ACCTAAACCCTAACCCCTAAACCCTAAACCCTAAACCCTAAACCCTAAACCCTAAACCCTAAACCCTAACCC
CTAAACCCTAAACCCTAAACCCTAAACCCTAAACCCTAAACCCTAAACCCTAAACCCTAAACCCTAAACCCT
AAACCCTAAACCCTAAACCCTAAACCCTAAACCCTAAACCCCTAAACCCTAAACCCTAAACCCTAAACCCTA
AACCCAAAACCCTAAACCCTAAACCCTAAACCCTAAAACCCTAA
>TCONS_00056502
ACCTAAACCCTAACCCCTAAACCCTAAACCCTAAACCCTAAACCCCAAACCCTAAACCCTAAACCCTAAACC
CTAAACCCTAAACCCTAAACCCTAAACCCTAAACCATAAACCCTAAACCCTAAACCCTAAACCCTAAACCCT
AAACCCTAAACCCTAAACCCTAACCCCTAAACCCTAAACCCTAAACCCTAAACCCTAACCCCTAAACCCTAA
ACCCTAAACCCTAAACCCTAAACCCTAAACCCTAAACCCTAAACCCTAAACCCTAAACCCTAAACCCTAAAC
CCTAAACCCTAAACCCTAAACCCTAAACCCCTAAACCCTAAACCCTAAACCCTAAACCCTAAACCCAAAACC
CTAAACCCTAAACCCTAAACCCTAAAACCCTAA
>TCONS_00056503
ACCTAAACCCTAACCCCTAAACCCTAAACCCTAAACCCTAAACCCTAAACCCTAAACCCTAACCCCTAACCC
CTAAACCCTAAACCCTAAACCCTAAACCCTAAACCCTAAACCCTAAACCCTAAACGCTAAACCCTAAACCCT
AAACCCTAAACCCTAAACCCTAAACCCTAAACCCTAAACCCTAAACCCTAAACCCTAAACCCTAAACCCTAA
ACCCTAACCCCTACACCCTAAACCCTAAACCCTAAACCCTAAACCCTAAACCCTAAACCCTAAACCCTAAAC
CCTTAAACCCTAAACCCTAAAACCCAAAACCCCAAAACCCAAAACCCAAAAACCCTAAACCCTAA
>TCONS_00056504

CCTAAACCCTAACCCCTAAACCCTAAACCCTAAACCCTAAACCCTAAACCCTAAACCCTAAACCCTAAACCC
TAAACCCTAAACCCTAAACCCTAAACCCTAAACCCTAAACCCTAAACCCTAAACCCTAAACCCTAAACCCTA
AACCCTAAACCCTAAACCCTAAACCCTAAACCCAAAACCCTAAACCCTAAACCCTAAACCCTAAACCCTAA
CCCCTAAACCCTAAACCCTAAACCCTAAACCCTAAACCCTAAACCCTAAACCCTAAACCCTAAACCCTAAAC
CCTAAACCCTAAACCCTAAACCCTAAACCCCTAAACCCTAAACCCTAAACCCTAAACCTAA
>TCONS_00056505
CCTAAACCCTAACCCCTAAACCCTAAACCCTAAACCCTAAACCCTAAACCCTAAACCCTAAACCCTAAACCC
TAAACCCTAAACCCTAAACCCTAAACCCTAAACCCTAAACCCTAAACCCTAAACCCTAAACCCTAAACCCTA
AACCCTAAACCCTAAACCCTAAACCCTAAACCCAAAACCCTAAACCCTAAACCCTAAACCCTAAACCCTAA
CCCCTAAACCCTAAACCCTAAACCCTAAACCCTAAACCCTAAACCCTAAACCCTAAACCCTAAACCCTAAAC
CCTAAACCCTAAACCCTAAACCCCTAAACCCTAAACCCTAAACCCTAAACCTAA
>TCONS_00056506
CCTAAACCCTAACCCCTAAACCCTAAACCCTAAACCCTAAACCCTAAACCCTAAACCCTAAACCCTAAACCC
TAAACCCTAAACCCTAAACCCTAAACCCTAAACCCTAAACCCTAAACCCTAAACCCTAAACCCTAAACCCTA
AACCCTAAACCCTAAACCCTAAACCCTAAACCCAAAACCCTAAACCCTAAACCCTAAACCCTAAACCCTAA
CCCCTAAACCCTAAACCCTAAACCCTAAACCCTAAACCCTAAACCCTAAACCCTAAACCCTAAACCCTAAAC
CCTAAACCCTAAACCCCTAAACCCTAAACCCTAAACCCTAAACCTAA
>TCONS_00056507
CCTAAACCTAAACCCCTAAACCCTAAACCCTAACCCCTAAACCCTAAACCCTAAACCCTAAACCCTAACCCC
TAAACCCTAAACCCTAAACCCTAAACCCTAAACCCTAAACCCTAAACCCTAAACCCTAAACCCTAAACCCTA
AACCCTAAACCCTAAACCCTAAACCCTAAACCCTAAACCCCTAAACCCTAAACCCTAAACCCTAAACCTAA
>TCONS_00056508
CCTAAACCTAAACCCCTAAACCCTAAACCCTAACCCCTAAACCCTAAACCCTAAACCCTAAACCCTAAACCC
TAAACCCTAACCCCTAAACCCTAAACCCTAAACCCTAAACCCTAAACCCTAAACCCTAAACCCTAAACCCTA
AACCCTAAACCCTAAACCCTAAACCCTAAACCCTAAACCCTAAACCCTAAACCCCTAAACCCTAAACCCTAA
ACCCTAAACCCTAAACCCAAAACCCTAAACCCTAAACCCTAAACCCTAAAACCCTAA
>TCONS_00056509
AAACCCTAAACCCTAAACCCTAAACCCTAAAACCCTAAAACCCTAAAACCCTAAACCCTAAACCCTAAACC
CTAAACCCTAAACCCTAAACCCTAAACCCTAAACCCTAAACCCTAAACCCTAAACCTAAACCCTAAAACCCT
AAACCCTAAACCCTAAACCCTAAAACCCTAAACCCTAAACCCCAAACCCTAAACCCTAAACCCTAAACCCT
AAACCCTAAACCCTAAACCCTAAACCCTAAACCATAAACCCTAAACCCTAAACCCTAAACCCTAAACCCTA
AACCCTAAACCCTAAACCCTAACCCCTAAACCCTAAACCCTAAACCCTAAACCCTAAACCCTAAACCCTAAA
CCCTAAACCCTAAACCCTAAACCCCTAAACCCTAAACCCTAAACCCTAAACCTAA
>TCONS_00056510
AAACCCTAAACCCTAAACCCTAAACCCTAAACCCTAAACCCTAAACCCTAAACCCTCAACCCTAAACCCTAA
ACCCTAAACCCTAAACCCTAAACCCTAAACCCTAAACCCTAAACCCTAAACCCTAAACCCTAAACCCTAAAC
CCTAAACCCTTAAACCCTAAACCCCTAAACCCTAAACCCTAAACCCTAAACCCTAAACCCTAAACCCTAAAC
CCTAAACCCTAAACCCTAAACCCTAAACCCTAAACCCTAAACCCTAAACCCTAAACCCTAAACCCCTAAACC
CTAAACCCTAAACCCTAAACCTAA
>TCONS_00056511

CCCTCCTAAACCCTAAACCCTAAACCCTAAACCCTAAACCCTAAACCCTAAACCCTAAACCCTAAACCCTAA
ACCCTAAACCCTAAACCCTAAACCCTAAACCCTAAACCCAAACCCCTAAACCCTAAACCCTAAACCCTACAC
CCTAAACCCTAAACCCTAAACCCTAAACCCTAAACCCTAAACCCTAAACCCTAAACCCTAAA
>TCONS_00056512
CCCTCCTAAACCCTAAACCCTAAACCCTAAACCCTAAACCCTAAACCCTAAACCCTAAACCCTAAACCCTAA
ACCCTAAACCCTAAACCCTAAACCCTAAACCCTAAACCCAAACCCCTAAACCCTAAACCCTAAACCCTAAAC
CATAAACCCTAAACCCTAAACCCTAAACCCTAAACCCTAAACCCTAACCCCTAAACCCTAAACCCTAAACCC
TAAACCCTAAACCCTAAACCCTAAA
>TCONS_00056513
CTCCTAAACCCTAAACCCTAAACCCTAAACCCTAAACCCTAAACCCTAAACCCTAAACCCTAAACCCTAAAC
CCTAAACCCTAAACCCTAAACCCTAAACCCTAAACCCAAACCCCTAAACCCTAAACCCTAAACCATAAACCC
TAAACCCTAAACCCTAAACCCTAAACCCTAAACCCTAACCCCTAAACCCTAAACCCTAAACCCTAAACCCTA
AACCCTAAACCCTAAAACCCTAAACCCTAAACCCTAAACCCTAAACCACTAACCCTAAACCTAAAACCCTA
AACCCTAAAA
>TCONS_00056514
CAAACCCTAAACCCTAAACCCTAAACCCTAAACCCTAAACCCTAAACCCTAAACCCTAAACCCTAAACCCTA
AACCCTAAACCCTAAACCCTAAACCCTAAACCCTAAACCCTAAACCCTAAACCCTAAACCCTAAACCCTAAA
CCCTAAACCCTAAACCCTACACCCTAAACCCTAAACCCTAAACCCTAAACCCTAAACCCTAAACCCTAAACC
CTAAACCCTAAACCCTAAACCCTAAACCCTAAACCCTAAACCCTAAACCCTTAA
>TCONS_00056515
AGCCCCTAAACCCTAAACCCTAAACCTTAAACCCTAAACCCTAAACCCTAAACCCTAAACCCTAAACCCTAA
ACCCTAAACCCTACACCCTAAACCCTAAACCCTAAACCCTAAACCCTAAACCCTAAACCCTAAACCCTAAAC
CCTTAAACCCTAAACCCTAAAACCCAAAACCCCAAAACCCAAAACCCAAAAACCCTAAACCCTAA
>TCONS_00056516
CTAAAACCCTAAACCCTAAACCCTAAAACCTAAACCCTAAACCTAAACCCTAAACCCTAAACCCTAAACCCT
AAACCCTAAACCCTAAACCCTAAACCCTAAACCCTAAACCCTAAACCCTAAACCCTAAACCCTAAACCCTAA
ACCCTACACCCTAAACCCTAAACCCTACACCCTAAACCCTAAACCCTAAACCCTAAACC
>TCONS_00056517
AAGAAACCCTAAACCCTAAACCCTAAACCCTAAACCCTAAACCCTAAACCCTAAACGCTAAACCCTAAACC
CTAAACCCTAAACCCTAAACCCTAAACCCTAAACCCTAAACCCTAAACCCTAAACCCTAAACCCTAACCCCT
AAACCCTAAACCCTAAACCCTACACCCTAAACCCTAAACCCTAAACCCTACACCCTAAACCCTAAACCCTAA
ACCCTAAACCCTAAACCCTAAACCCTAAACCCTAAACCCTAAA
>TCONS_00056530
GGGTTTAGGGTTGAGGGTTTAGGGTTTAGGGTTTTGGGTTTAGGGTTTAGGGTTTAGGGTTTAGGGTTTAGGGT
TTAGGGTTTAGGGTTTAGGGTTTAGGGTTTAGGGTTTAGGGTTTAGGGTTTAGGGTTTAGGGTTTAGGGTTTAG
GGTTTAGGGTTTAGGGTTTAGGGTTTAGGGTTTAGGGTTTAGGGTTTAGGGTTTAGGGTTTAGGGTTTAGGGTT
TAGGGGTTTAGGGTTAGGGTTTAGGGGTTAGGGTTTAGGGTTTTAGGGTTTAGGGTTT
>TCONS_00056531
AGGGTTTAGGGTTTAGGGTTTAGGGTTTTAGGGTTTAGGGTTTAGGGTTTAGGGTTTAGGGTTTAGGGTTTAGG
GTTTAAGGGTTTAGGGTTTAGGGTTTAGGGTTTAGGGTTTAGGGTTTAGGGTTTAGGGTTTAGGGTTTAGGGTT
TAGGGTTTAGGGTTTAGGGTTTAGGGTTTAGGGTTTAGGGTTTAGGGTTTAGGGTTTAGGGTTTAGGGGTTTGG

GGTTAGGGTTTAGGGTTTAGGGTTTAGGGGTTAGGGGTTAGGGTTTAGGGTTTAGGGGTTAGGGTTTAGGGTTT
TAGGGTTTAGGGTTT
>TCONS_00056532
AGGGTTTAGGGTTTAGGGTTTAGGGTTTTAGGGTTTAGGGTTTAGGGTTTAGGGTTTAGGGTTTAGGGTTTAGG
GTTTAAGGGTTTAGGGTTTAGGGTTTAGGGTTTAGGGTTTAGGGTTTAGGGTTTAGGGTTTAGGGTTTAGGGTT
TAGGGTTTAGGGTTTAGGGTTTAGGGTTTAGGGTTTAGGGTTTAGGGTTTAGGGTTTAGGGTTTAGGGGTTAGG
GGTTAGGGTTTAGGGTTTAGGGTTTAGGGGTTAGGGGTTAGGGTTTAGGGTTTAGGGGTTAGGGTTTAGGGTTT
TAGGGTTTAGGGTTT
>TCONS_00056533
AGGGTTTAGGGTTTAGGGTTTAGGGTTTTAGGGTTTAGGGTTTAGGGTTTAGGGTTTAGGGTTTAGGGTTTAGG
GTTTAAGGGTTTAGGGTTTAGGGTTTAGGGTTTAGGGTTTAGGGTTTAGGGTTTAGGGTTTAGGGTTTAGGGTT
TAGGGTTTAGGGTTTAGGGTTTAGGGTTTAGGGTTTAGGGTTTAGGGTTTAGGGTTTAGGGTTTAGGGGTTTGG
GGTTAGGGTTTAGGGTTTAGGGGTTAGGGGTTAGGGTTTAGGGTTTAGGGGTTAGGGTTTAGGGTTTTAGGGTT
TAGGGTTT
>TCONS_00056534
AGGGTTTAGGGTTTAGGGTTTAGGGTTTTAGGGTTTAGGGTTTAGGGTTTAGGGTTTAGGGTTTAGGGTTTAGG
GTTTAAGGGTTTAGGGTTTAGGGTTTAGGGTTTAGGGTTTAGGGTTTAGGGTTTAGGGTTTAGGGTTTAGGGTT
TAGGGTTTAGGGTTTAGGGTTTAGGGTTTAGGGTTTAGGGTTTAGGGTTTAGGGTTTAGGGTTTAGGGGTTAGG
GGTTAGGGTTTAGGGTTTAGGGTTTAGGGTTTAGGGGTTAGGGGTTAGGGGTTAGGGTTTAGGGTTTAGGGGT
TAGGGTTTAGGGTTTTAGGGTTTAGGGTTT
>TCONS_00056535
AGGGTTTAGGGTTTAGGGTTTAGGGTTTTAGGGTTTAGGGTTTAGGGTTTAGGGTTTAGGGTTTAGGGTTTAGG
GTTTAAGGGTTTAGGGTTTAGGGTTTAGGGTTTAGGGTTTAGGGTTTAGGGTTTAGGGTTTAGGGTTTAGGGTT
TAGGGTTTAGGGTTTAGGGTTTAGGGTTTAGGGTTTAGGGTTTAGGGTTTAGGGTTTAGGGTTTAGGGGTTAGG
GTTTAGGGTTTAGGGTTTAGGGTTTAGGGGTTAGGGTTTAGGGTTTAGGGTTTAGGGTTTAGGGTTTTGGGTTT
AGGGTTTAGGGTTTAGGGTTTAGGGTTTAGGGTTTAGGGTTTAGGGTTTAGGGTTTAGGGTTTAGGGTTTAGGG
TTTAGGGTTTAGGGTTTAGGGTTTAGGGTTTAGGGTTTAGGGTTTAGGGTTTAGGGTTTAGGGTTTAGGGTTTTA
GGGTTTAGGGTTTAGGGTTTAGGGTTTAGGGTTTAGGGTTAGGGTTTAGGGTTTAGGGTTTAGGGTTTAGGGTT
TAGGGTTTAGGGTTTAGGGTTTATGGTTTAGGGTTTAGGGTTTAGGGTTTAGGGTTTAGGGTTTAGGGTTTAGG
GTTTAGGGTTTGGGGTTTAGGGTTTAGGGTTTAGGGTTTAGGGTTTAGGGTTTAGGGTTTAGGGTTTAGGGTTT
AGGGTTTAGGGTTTAGGGTTTAGGGTTTAGGGTTTTAGGGTTTTAGGGTTTTAGGGGTTTAGGGTTTAGGGTTT
AGGGTTTAGGGTTTAGGGTTTAGGGTTTAGGGGTTTAGGGTTAGGGTGTTTGGGTTTTAGGGTAGGGTTTAGGG
GTTTAGGGTTTAGGGTTTAGGGTTTTTAGGGGTTTAGGGTTTAGGGGGGGGTTTAGGTTTAGGGGTTAGGGTTT
AGGGTTTTAGGGTTTAGGGTTT
>TCONS_00056536
AGGGTTTAGGGTTTAGGGTTTAGGGTTTTAGGGTTTAGGGTTTAGGGTTTAGGGTTTAGGGTTTAGGGTTTAGG
GTTTAAGGGTTTAGGGTTTAGGGTTTAGGGTTTAGGGTTTAGGGTTTAGGGTTTAGGGTTTAGGGTTTAGGGTT
TAGGGTTTAGGGTTTAGGGTTTAGGGTTTAGGGTTTAGGGTTTAGGGTTTAGGGTTTAGGGTTTAGGGGTTAGG
GTTTAGGGTTTAGGGTTTAGGGTTTAGGGTTTAGGGGTTAGGGTTTAGGGTTTAGGGTTTAGGGTTTAGGGTTT
TGGGTTTAGGGTTTAGGGTTTAGGGTTTAGGGTTTAGGGTTTAGGGTTTAGGGTTTAGGGTTTAGGGTTTAGGG
TTTAGGGTTTAGGGTTTAGGGGTTAGGGTTTAGGGTTTAGGGTTTAGGGTTTAGGGTTTAGGGTTTAGGGTTTA
GGGTTTATGGTTTAGGGTTTAGGGTTTAGGGTTTAGGGTTTAGGGTTTAGGGTTTAGGGTTTAGGGTTTGGGGT

TTAGGGTTTAGGGTTTAGGGTTTAGGGTTTAGGGTTTAGGGTTTAGGGTTTAGGGTTTAGGGTTTAGGGTTTAG
GGTTTAGGGTTTAGGGTTTTAGGGTTTTAGGGTTTTAGGGGTTTAGGGTTTAGGGTTTAGGGTTTAGGGTTTAG
GGTTTAGGGTTTAGGGGTTTAGGGTTAGGGTGTTTGGGTTTTAGGGTAGGGTTTAGGGGTTTAGGGTTTAGGGT
TTAGGGTTTTTAGGGGTTTAGGGTTTAGGGGGGGGTTTAGGTTTAGGGGTTAGGGTTTAGGGTTTTAGGGTTTA
GGGTTT
>TCONS_00056537
GTTTAGGGTTTAGGGGTTTAGGGTTTAGGGTTTAGGGTTTAGGGTTTAGGGTTTAGGGTTTAGGGTTTAGGGTT
TAGGGTTTAGGGTTTAGGGTTTAGGGTTTAGGGTTTAGGGTTTAGGGTTTAGGGTTTAGGGGTTAGGGTTTAGGGTTTAGG
GGTTAGGGTTTAGGGTTTAGGGTTTAGGGTTTAGGGTTTTGGGTTTAGGGTTTAGGGTTTAGGGTTTAGGGTTT
AGGGTTTAGGGTTTAGGGTTTAGGGTTTAGGGTTTAGGGTTTAGGGTTTAGGGTTTAGGGTTTAGGGTTTAGGG
TTTAGGGTTTAGGGTTTAGGGTTTAGGGTTTAGGGTTTAGGGTTTTAGGGTTTAGGGTTTAGGGTTTAGGGTTTA
GGGTTTAGGGTTAGGGTTTAGGGTTTAGGGTTTAGGGTTTAGGGTTTAGGGTTTAGGGTTTAGGGTTTATGGTT
TAGGGTTTAGGGTTTAGGGTTTAGGGTTTAGGGTTTAGGGTTTAGGGTTTAGGGTTTGGGGTTTAGGGTTTAGG
GTTTAGGGTTTAGGGGTTAGGGTTTAGG
>TCONS_00056538
GTTTAGGGTTTATGGTTTAGGGTTTAGGGTTTAGGGTTTAGGGTTTAGGGTTTAGGGTTTAGGGTTTAGGGTTTT
GGTTTAAGGGTTTAGGGTTTAGGGTTTAGGGTTTAGGGTTTAGGGTTTAGGGTTTAGGGTTTAGGGTTTAGGGT
TTAGGGTTTAGGGTTTAGGGTTTAGGGTTGAGGGTTTAGGGTTTAGGGTTTAGGGTTTAGGGTTTAGGGTTTAG
GGGTTAGGGTTTAGGGTTTAGGGTTTAGGGTTTAGGGTTTTGGGTTTAGGGTTTAGGGTTTAGGGTTTAGGGTT
TAGGGTTTAGGGTTTAGGGTTTAGGGTTTAGGGTTTAGGGTTTAGGGTTTAGGGTTTAGGGTTTAGGGTTTAGG
GTTTAGGGTTTAGGGTTTAGGGTTTAGGGTTTAGGGTTTAGGGTTTTAGGGTTTAGGGTTTAGGGTTTAGGGTTT
AGGGTTTAGGGTTAGGGTTTAGGGTTTAGGGTTTAGGGTTTAGGGTTTAGGGTTTAGGGTTTAGGGTTTATGGT
TTAGGGTTTAGGGTTTAGGGTTTAGGGTTTAGGGTTTAGGGTTTAGGGTTTAGGGTTTGGGGTTTAGGGTTTAG
GGTTTAGGGTTTAGGGGTTAGGGTTTAGG
>TCONS_00056539
TTAAGGGTGTAGGGTTTAGGGTTTAGGGTGTAGGGTTTAGGGTTTAGGGTTTAGGGTTTAGGGTTTAGGGTTTA
GGGTTTAGGGTTTAGGGTTTAGGGTTTAGGGTTTAGGGTTTAGGGTTTAGGGTTTAGGGTTTAGGGTTTAGGGT
TTAGGGTTGAGGGTTTAGGGGTTAGGGTTTAGGGTTTAGGGGTTTAGGTTTAGGGTTTAGGGTTTAGGGTTTAG
G
>TCONS_00056901
CTTCCGAAGAAAACTTGCTCCAGATTCATTTCCAGTATATTGAATGGCAATCGTACCGTGTGGAAGCACGTGG
AGAGGTCTACCTCAGTTTCTAACAAAATTATCGGTTACTAGTAAATTTTCAGCATCTCCATTTTGTTCCTCAAG
CAAGACAAGGGCATTGGCGTCACCAAGTTCCACACTTTTTTGTCAAGAATCGCTGCGTGGACTTTACAGCCCA
GTGTCGCACAAGAATCAATGCCACCAGAGAGGGGCTCGTTTCATTGTTAGGGCAGATAGAGATTACTATGAT
GTCCTAGGTGTGTCTAGAAATGCAAGCAAATCAGAGATAAAAAGTGGTAGGGGAACTTGAGTAGTCTCTGAT
GCAAAAACTATGAGAACAATGGGGAGTTTTAACACAAAACACAAACAGTTCAGAGGAGATTGGAGTTGGAT
GGATCTTTCGCAGTTATGGTAAAACATGTCTATAAAAAAATCTATTTCCACTTAATTTTCCATGCTACTTTTA
ATATGCGAAATTTACAAAGACGGTAAACAAAGATGAAGGATTAGTTGAGAAACAGGATGAAGGCACTGGTT
TTGATCCCCGGAATAACTGATGGGTGAATCAGAAGTGGATCACAATGATATTGATTGACTAAGGAGTGTAAG
AAGAAGGACGGACAAGCAGAAGAACCTGCTTTTAGTGCCTTGTTGAACTTTCTCTCTGGACATAGTTAAACAT
TAATGATTCTGTCCTTTGTGGAAAAAAAATTCAGAGAGCTGTTCTCTGTTTGACGAATTTGTAACACGAGAAAA
CTCAAATCCATGTTTATGTTTTCCCACAACTGCTTCAATGAGCGCAATTGAACAGGGGTGTTGGCGT

>TCONS_00056930
ATCTTTGCCCATTGGTTCATGGCTCGCTAAGTTTTCCTTTGGAAGCTTGACCTGGTGGCCGACGAGAACTCCTC
GCTCTTCTGTTATGGATGGGGTCAGGACCATTTGGAACTCTTCTTCTACTCACGAAGTTGAGATCTATCTTGGA
ATGAATACTTCTCTTTTGTATTTTCATAAGTTTGGTATTCTTCTTCATAATTCCTTCATTTTCAAATACTCC
>TCONS_00056933
GGAAAATATTAGAAAGTTCTCTAAGGGCTTTTTGGGATTTTTGATACTGCTAGGATTTGTTTGGTTATTGTTGGT
TGGAGTATTTGAAAATGAAGGAATTATGAAGAAGAATACCAAACTTATGAAAATACAAAAGAGAAGTATTC
ATTCCAAGATAGATCTCAACTTCGTGAGTAGAAGAAGAGTTCCAAATGGTCCTGACCCCATCCATAACAGAA
GAGCGAGGAGTTCTCGTCGGCCACCAGGTCAAGCTTCCAAAGGAAAACTTAGCGAGCCATGAACCAATGGG
CAAAGATTATAAGGCTTGTGATATGCTT
>TCONS_00057055
CCCCCCCCCCCCCCAAAAAAAAAATCTTGAAATAGAAGTTAAGAATCGGTGAAATCCTATTTCATTTGGGTG
TTTGTTTAGCCTCTGAATATAGTATGGATACTCTGATCTTCATTTCCCTCTTCATTCTTATTCTACATGTTAAAGC
ACAAGCAAATACAAATACAAATAGTTGCTCACCAATTAACTGTTCAAATAACTCTGATATCAACATTCAGTTT
CCTTTCAGACTGCAGGAATTTCAACCTCAACACTGTGGTCTCTCTGGTTTTGAGCTATACTCCTTGGCGTGTAT
TATCCTGTTCATATTTGTAGTGGTGATGCTGTTAAACATAAAGAAGTTATCTCATTCTACAAAGAGAATGGAG
GTGGAGAAGGAGAAAGAGAATCAGAAGGAACCTCATGCTGCCATTTTAGGGGACTATGAAGCTTTGAAGCC
CAAAAGAGAATTCATGGCCAATGCTAACAGATGTTGAATGATCTAATTTAGGAAAAAAATTGTTACAAAGAC
ATTCAGAATCCAAATGCTTTCTAGTTGTTTTAAAATGATTGAGAAACGCTCTTAAATATGATATGAAGCTAGC
ATTTGGCCATAGATCCCAAATTTGTTTTGAAAAATCTGATTTGGGTGAAGTTTGGTTTGAAGATAAAAATGTGT
TTGGACATACGTTTTCAATTTCCAACTTTATTTTGGAAAAACATGTATATAATTCTCAGTTTTGGAATTTTGGCC
CAAAATCAGCTCAATAGTAGCTGATTTTGGGATTTGGCCAAAATATGGGCAAAATTTATGGCCAAACATGAG
TTTTGAAAATAAATCCCAAATTTATTTTGGCAAAAATCATCGTCAAATGGGGCCGA
>TCONS_00057059
TTTACCAGTCTTTGCCAGTCACCTATTGAGTGGCATCTTCAGTAAATCTAGCATCAAAAGGAGCAGAAGGGTT
GGGGTGTGTAGAAGCAATACTCGCTCCAGAACCATGTTGCAATTCTATCACTAGCCATCTGACGGCTTTACTC
ATCTGCTCCAACCTAACAGCACTAATGGGTTGAAGGCCACTTGTTGCTGATGATTGCCCAGGCTTCCCTGAAA
CAGAGCCCGTAGCCTCATCTACAAGACACTCGGAACCTATTCTTTGCTTAACTGACTCAACAAACTCCTCTGC
CTGATCACAAGCAACCCTGAACAACTCCCACCGTTGCTTAAAGTTTTCTAACGCAGCGTCAGTTGCTACTGTT
TTCTGACCATTTGAACTCTCTTTTGATTCAAGTGCA
>TCONS_00057117
GCTTCATCCAGCACCATGTATTGCCATTTGACACATCTGAAGTATTTCTCATCAGAGACTAGCAGTTGTTAACT
GGTAATGAGAATATGGAATCCAGCATCCCGTCGATATAGACGTTTTGGGTTAATATTCTTCCGAAGTACCATT
TGCTCTTGTAACCCTCCCCAATATGGAAGAGTTTTTAGATCAGGGCAGAAACGACCAATTTCAT
>TCONS_00057176
GACAACAGCGGATGGGGTACCCAGATGCTTCACCCTGATTGATTTTCTCACTGCAAATCCAGTTTCTTGTTGTA
ACGATCTTCTGCTTCTCGCTTATCCTTATAGATCTTTTCCATCAGTCTTCTTATCCCTAGTGGTACTGTCATCTTG
AAGATCCTTTAAGATGATCCCCGGCACCACAAAGTTGGCAATCCAGTACATAAAGAGCATTACTGCACTTGT
CTGTAGCAAAACTGACAAGTCTCTCGGTGAATTAGCCGCAGCTATGAAAAAGGAGGAGGAGGAAATAGGTA
TATCTACAGCCC
>TCONS_00057222

CTCTCATGATTTCTTTTGAAATGTGTGTAATTGCCATTTGTGTCGGCTTGCAGGAAAGATGTGAGGGCAGTGTT
CCATGTAGAAGCTGGAAATATCTTTAGCTGCATATAAAGGGCGATTATCTCTGTTTGGAGCTGTGAGCATAGT
AGTAATTAATCCACCTGTGCTTGTTCCTGCTACTACATCAAAATAGTCTATAATCCTTGCATTTGCTCCATCAA
GCTCCTGAAGCTTGGATTCAAGAAAGGCAAGAATGGTTCCAGGAATAATGCCTCTGATTCCACCTCCATCAA
TGCTCAAAACTGTTATAAAATTCTTTCCTTTGGTAGCTG
>TCONS_00057281
CCGCTGCTCCTGCCTCATGGAGGGCAAGTAAAAAAGAGCCTGGCATTCTGTCAGACAGTCCAAGAGAACGG
CAGAGGTCAGCAGCTTGCCGACGACGTGAAACCATTGGAAGCAGTTTGAATAAGTCATCCTGGTTGAAGTTG
GAATTAATACCAAAAGTAGCTAGAAGCTGTAAGAATGCATGAGCCTCCAAGGAATTCCCGTTATTTGCATCA
ATATCAAGTTCATCTAACTTAGGTTTCCACTCCTCAGCTATTAATTTTGCACGTTCCTTTACATTTTCAGGTATT
ATACTTGGAAAAGAATCGATTTCCAGCATGGTTAATAAAATGCTAAGGCATTCCATCAGCATAATACAAGTT
CGTCGAAGGCCCAACAGATTTGCATCTTTTTTAGCATCTGAAATTGACACTTCTGAGGGGTAGAAACCCTTGA
GTGAGTCCAGAACCAGAGAGCCAGGGTCATCTGCGGCTCTAAAAGCAATTGGAATCTCCTCCCTTACAGCAG
CTAGGTTCTTGAGGTTGTCTGATATAAATTTATGGAGGCCTTCTGAATCCATGTCTTGGCATAGTTTCACTAGC
TCTGGATAAGACTTGACTTCCACAATTGCATTCTCAGACTGTTTTCTATTATTCTCTGTATAATTCTCAGTTGCC
CCAGAAACCATGGGCTTTGCCTCCAGAAAAGATGGGCCACCTCGATCCTCAGCATTAATAACTACAGGTTCT
GCAGAATAAGGCTTCCTTTGCTTCTCCAGAGCAACAGCAATAGCAGAGACAGCTGCATCCCTTTTCTCCTGGA
GCCTCTCAAGTGAAGTTTGCTCCTTAGCAACAACAGCTGCTTGACGCTTCTCTAATAGTTCTTTAGACTGAACT
ATTTTTGTTTCAAACTCTTTCTCATGTTCTTCCAGTTCAGTGAAACGCCTTTTCAAGGACCTCTGTAGCCCATCA
AAGTGTTCTTCGAGTTGCTTCCATTTCAGATTGAGAGTAATAGCACGGTGACTTTCCAACTCAGCAAATGCTTT
CTGTAGTTGATGTATCTTAGAGGTCGTAGAGTCCATCAATGTTCCAACTGATTGTGCGTCTTCCATGGCAGACA
GTCTGGATTGGTGGTGACAGGTGGAGGAAAATGATAGGTCGGCGGCGAATTTCGCCGAAGAATCAGACCATC
TTGGAACAAGGGTTTCTTTGTCGGGATGCCGCAAAGAGGGAAAAGAGGAAAGTGTCAGTTGGAATGTTGACT
GCTATTTTATGACAGCTTCTCGACACAAACCACCACACGCGCGCG
>TCONS_00057335
TCAGAAGCAAGATATACTTGTAAAGGAACAACATAATTAAATATGAATATGTCAAAGAGATCTGAAAGAAT
AATGCGTACAAGGATAGCATGAGATAGGATCGCTTTCGCATGGATAGCTCAAGAGGGCTTAGCTCTCTCTTTT
TCCAGCATTTGCTTCCGATAGCAAAGGTTCAAGTTCTCCTTTCCGATACAGCTTAATAGTATCTGTACAACCAC
CAATGTGTTTGGCCCCAATGAATACATTGGGAACCGTATGCTGTCCAGTAAGCCTTTCCAGCACTTTCTGCAG
CTGTGGTCCTTGGGGACCCATTTCATCCAATTCAATAACAAGTGGATCTACACCAAGTTTTTGAACAAAGCT
TTCACCTCCATTGAGTACGAACACCAAGTTTTGGAGTAAACAACAACTGGGTTTTCAGTAATGGTCTTTTTCAC
GCTCTCTTCCAAACGGGACCCGAATGAACCCGACATCGCCCGAATCTG
>TCONS_00057561
CTCAAGCAGATTTAGCTAGCAGAGGCTCGTCAGCTACTCCTTTTCCTTTTAAAGGTAGGATGTATGACTCCGTT
GCATGATTGTAGTTTATCAATCCTTGCCATACAATCCTTCATCCTATTTAATGCAACGGGTATAGCGGAAGCA
CTCTCCAAATTCTTCTTTTTAAGGAGCTGTATTTTCTCAGCTTCTTTCCGCTCCTCTCCAGCTAACATGGCAATA
TTAGTTGGTCCTCCAACATCGGAGCTGCAGGGATCACTAGAGGGCCCAGATTGAATACGGATCCGTTGTCAA
AACGGGTCCCCAACACGGGTCTTTGAGCCGCAAAAATGGAAGAGATATCAGTTTCAAGCTGAGCAAGAATT
ATGCCTCGGTACTCCGCTATTCTCTCAGCCTTTTGCTTAACTTCTTCCTCCAGTAATTCTGCTTCTTCC
>TCONS_00057649
CATTCTATACCTTCCTTTCTTCATTCCCCTCTTTCCTTCTATTCTCTCTTTCTCTCTCTCTAGTTTGTGGCACATTTT
TATGGGTTTGAGAGAAAATATTGCAGTGCAGAAAGCAACACTGAAATCTCCACAAGGTTTCTTTAAAGCTTTT

GGCGCGTATGAGTGGAGTAAATCCAGCAGCTTCAAGCCAAAGGCTGCAAAAACTGGAAACTGTAAGCCTAA
TGGCATCTTTAGCTGATGGAAAGGTTAGGGGCCACCTTAGCCAGGGCTTTGTTTTAGTCATCTGCACCTGCAA
CTTCTTCATTCTCATTCTCATGATGGGAGTT
>TCONS_00057682
TTCTGTCTTGGTAGATGGAGATGCAAATGATGTGATTCCCTTCATATTTTGACTGTCAGCCCCCACTGCAGGTG
AAACAACAGTCTCCTCCTCTATGGTGTCTCCTGTCTCCTGTTCTTTTGCGAGTCCACCACCAGCACGACTCTCA
ATGCTGGCTACCTCAGTTTTACTAACAAATGATTTATCATGAGAAAGATCCTCCGACTGTAGCTGGTCAACAT
CTTTTTCTGCAGCCATGTTTGCCCCACTCTTACCTGCCAGTGAATTTTTGCATGAATCCCCCTTATCGAGTAGA
GCAGCATTCGTGCAATCGCTTACATGGATTTGAACTAATGTAGGTTCGACATAACATAGAGATGATGCCTGTG
ATTCACCAGCCTTCATGCGTCTCCCAGCTTCCTTTTCATCATTTTCATTCTTGCCATCACTCTCACATTTGTCATT
CATAGCCTCAATCTTACCATCTTCACCTTTAGAATTAGTTTCACTCTTTTCAGCACCTTCACCCTTAGAATCACC
TTCACTCTCCTCAACCAGACAATCACTAACCTGAGCCATTGATCCTGAAGTTGATACAGCCAACTCATCACAA
GAAGATACAGGTAAATTTGGATGGCTGTCATATTTACTTCTCGAATATCCCTCTTCTTTGGTCTCTGATAGGGG
AGTTACCAAGGGGACCTCTCTAGTCATAGCATTTTCTTTTTCCTTGAGATTGTCCACCCCTGCAGAAGAAAGA
GTAACATGGGATTGCGATGGATCTGTTTGGATAGCATCAGACTGTGGCTGAACTTCATTACACAATATATCTT
CAAGGAGTTTCCCAACAGAGAGAACTGAAGGCGTCTGAGATGCAGCTGACTGAACTTCCATTATTTGTTGAT
CCTCACTGAGAACCTCCTTAGTTGGATCAGCTCCACAGCTTATATTAACAGTATCTACCCGTTCTGCAACATG
ACCACTGGATTGTGATGCGTCGGATTTGATAGCATTGCTATGTAGCTTAACTTCAGAACGCACCATAGCTTGA
GGGATTGGCCTTGCACTAGGATCTGGAGTCATTTGAGATGCTTCTGACTGGACAATTGCTGTTTGTTCCAGCAC
TTCCTGAGCAGGAGAATCGCCAGAGTTGACCATGTTAATCTGCAGCTCAATAGGAGGACCAGAGCAACTTTG
AGGTGCAACAGATTGGACCACAGCTGCCTTTCTCTGAGCATCATTGAGAACCTTTTCAGCAGTAGCAGTATCC
GGGTTTACATTATCACCCACCTGACCAGGAACCAGAATAGCATGGGATTGAGATGCATCTCTGTTGATAGCAT
TGTTCTGTGGATCAACTTCACTACGGACCATATCTTTAGGGATTTGTCCACCAGAAAAATCTGGAGTCCTTTGA
GATACAACCGACTGGTCTACAACTACTTGTTGAGCTTCATTCTCACGCCTGCTTCTTGTAGTGGGCTTCCTCCC
AACGGATAACCTGGTTCTCTTTTGTGGGGGTTCAATTGATCTTGGATCCTGAGCACCTAACCCCTCAGTAACA
GTTGTGGTGATCAAATCCTGTCTCTTCCCCCTGCGCCGTGGTGGCTCACTTCCAGTTTGAGGCTTGCGTCCTCG
TCCTCTACCTGGCTTAGGTGCAGTGGGAGGAGTGTTATACTGTGAAACCGATGAAATTGCAGGCAATGGTGG
CACTGGATCGGAAGTATGACCAGTTCCTACACCAGAATCTCCTTGACCAATATTCTTCAAGATAGAAGAATC
ACATAAAGAATCATCATGACCTGAAATAGCAGAAGAGCAAGAAAATGTCCCCTTATCTGATCCAGAATCT
>TCONS_00057768
GAGCCGGTTCCCCTGCTTCCCAGAAAGATGTTGGCTTTCCAGTCTTTGAAACGGTCTGGAGAACAGCTTCTGG
TTGAACATTGCCCTTAACTGTCACTTTTTGCTCCTTCAGATCAATGTCAAAGTTTTCTACACCTTCCATTTTCCC
CAACACCCTTTTTACAGCCCCTACACAGCCTTGGCATGACATGCCAACCTTGAGAACAACAGTCTGAGACAT
GATGATGAGAAGAAACTTGAGAGGTTTTTGAAGAAAAAAATGGTTTGATACAAAAAGTCAAGAGAGAAAAT
TTGGACCTTTGAATGGTG
>TCONS_00057973
ATCCGTTGGACATAGATCCAATTTTTCACTTTTTTGGATTCCTTGGAATTTCTTTTTCCTGTTCCTGGTGGTATCA
AAACGCCGGTATGTCGGGATATCTTATCTGTCTCTCCAGGAAAATGGATATCTCCAGAAAAGATTTTAAGTTC
AATTCGTTTTTTTTTTCTCTCCACCCGGACCAACCCACCGACTCGGCTTCTTAGATTTAAGGTGATTTGTGTATC
TACC
>TCONS_00057985

CTATAATCCTTAAATATCTCTTTAGTAAAACGAACTTGGAACACGAGGTCATTAGGCTTCAAATCCTTTGGTTT
TTCCGCCAAAGCAAATGGCTTTCTTTTATATAACGGCATTTTCAAACCCAGATTCAGAAGAATATTGTGAATTT
GGGTTCTTTTTGAGTGTTGAAGTTGATGGTGAAGAGAGAGGAAGGCGGTGGTAGTACGCTTTGCTTTGGAA
>TCONS_00058069
TATTGCCACAACAAGAACTACAATCCCAAGCGCCGATAGAAGCAGACACATGCAAGAGCAACCACCCTTTG
TGCGCTTGTTCAAAATTGCAAGCTTTTTCTGCACTCGCTGAAGTCTTGAGTCTGTAACATCAACATGTTCGTCC
AGGTTATCAATAAGTCTTGTTTGCAAGCCAAGTTCTTCATTAACTGCCAGTGCTATATGTTTGGTGCTCATCAC
TGTCTCCTCCAAACTCTCAAGGCCCTCATCTTGCTCTGATTTGATAGAAAAGCCAACAAATAAGGAAAATAA
CAGAAAA
>TCONS_00058078
AAGTTACCCATAGCACACAGGAGATCAAGCAAATGTCTTCAAATTTTCAACTCCAAGCTTCTGCACTTCCACC
TTGAGTGACTTGAGGTATCTCACAGCTTCATCAAGAACACTGACAGTATTCATCCTGCTGGAACCAGGGACA
ATTCCCTTTAATGCCTTAACCATCTTCTTAAGTTTCCGGCGTTTTTTCTCACTGCAATTGCTAGTACTGCCAGAG
GACTTCCTGAAAGAAGTGGTCCTGCTCTTTTTGGATTGACAATCATAGTTAGAGTATGATTCAGGGGAACTGT
TTCCATAGTTTGCATCGGTGCGTGCAGTGCTCACCTCTTCATCATCATATTCCTCATGTTCTTCCTCTTCAAAGC
TGAGTAATGCATTTATATCGGCTGAATCTTCCTTCAGATATGAAGAAACTTCTGTATTCTCATTATTTGCAACT
TTTCTCTCCATGCTGTCTTGGAAGTAAGTTGCATTAAAATTCAAATCAGGATATGGAAACTTGGAAGCCATGG
CAGGGTGGAACATGATCTGGCTTCGATAATCAGTCTGATCAAAGATGATGAAATTCTTTGGACATGCCTCAGA
GGACTGAAATTCGAAACCATGAACAGGCACAGAAGGGTTGAAACCAGTAGGAAGTGCCATTGTACCGAAGA
CTGATGGAGAAGGAATATCCAATAGATTACCACCGCCTGCTTGGTCTTCAGGAGAGAAAAAAGGCTTTTGAG
GGGAAAATTGATAGTTACTCTGCATGAGTACTATGACAAAAGAACCAAGTAAACTGGAATAGAAATGCGTTT
TCCAATTGGAAAATAGTTCCAATCAGCATTCAGTGCTTAATGAGGTCATTCTGATTTCTAATACACTGAAGAA
AACCAGCCAATCCAGGCTTTTGCTGAATAAGAGCAACAAGAAGCAAGCAAATCCCAAACCTCCCCAAGCTT
TCACTTGACCAGACTACGTTACAGGTTTAAGCAACTGACGGAAATATTCAGCCTGACAAACTAGCATCAATT
GGCAATAAGCAACACTCCCTGCATGCATTTTAATCTCACCAGCTTTCTTGACTGCTCTGTATCTCTTCAAGGTA
AATGCAATGTACATTCTCACCCTCCAAATGGACTTTAGTTTCAAGAAATATCACAGATGGAAAATAGATATCT
GCAATCCGTCATATCAAACAAGCAAAAATTAGTTAAACATTAAGGAACTTCGGAAAATAAAAGAACAGCTT
ATTTTCTCACCATTGAAAGATAATAAGTTCATCGCCAGAACTACACTAGAGGAATACGGATTTTAATTTCCAT
TTCTCTAATGCCAAACCAACTCAAATTATCTTCCACTATAATTTTTGAGTCGTAGTCAGTTTTCCTATATCTTTT
ATATTACTGTTCTCTTCAGTAA
>TCONS_00058079
AAGTTACCCATAGCACACAGGAGATCAAGCAAATGTCTTCAAATTTTCAACTCCAAGCTTCTGCACTTCCACC
TTGAGTGACTTGAGGTATCTCACAGCTTCATCAAGAACACTGACAGTATTCATCCTGCTGGAACCAGGGACA
ATTCCCTTTAATGCCTTAACCATCTTCTTAAGTTTCCGGCGTTTTTTCTCACTGCAATTGCTAGTACTGCCAGAG
GACTTCCTGAAAGAAGTGGTCCTGCTCTTTTTGGATTGACAATCATAGTTAGAGTATGATTCAGGGGAACTGT
TTCCATAGTTTGCATCGGTGCGTGCAGTGCTCACCTCTTCATCATCATATTCCTCATGTTCTTCCTCTTCAAAGC
TGAGTAATGCATTTATATCGGCTGAATCTTCCTTCAGATATGAAGAAACTTCTGTATTCTCATTATTTGCAACT
TTTCTCTCCATGCTGTCTTGGAAGTAAGTTGCATTAAAATTCAAATCAGGATATGGAAACTTGGAAGCCATGG
CAGGGTGGAACATGATCTGGCTTCGATAATCAGTCTGATCAAAGATGATGAAATTCTTTGGACATGCCTCAGA
GGACTGAAATTCGAAACCATGAACAGGCACAGAAGGGTTGAAACCAGTAGGAAGTGCCATTGTACCGAAGA
CTGATGGAGAAGGAATATCCAATAGATTACCACCGCCTGCTTGGTCTTCAGGAGAGAAAAAAGGCTTTTGAG
GGGAAAATTGATAGTTACTCTGCATGAGTACTATGACAAAAGAACCAAGTAAACTGGAATAGAAATGCGTTT

TCCAATTGGAAAATAGTTCCAATCAGCATTCAGTGCTTAATGAGGTCATTCTGATTTCTAATACACTGAAGAA
AACCAGCCAATCCAGGCTTTTGCTGAATAAGAGCAACAAGAAGCAAGCAAATCCCAAACCTCCCCAAGCTT
TCACTTGACCAGACTACGTTACAGGTTTAAGCAACTGACGGAAATATTCAGCCTGACAAACTAGCATCAATT
GGCAATAAGCAACACTCCCTGCATGCATTTTAATCTCACCAGCTTTCTTGACTGCTCTGTATCTCTTCAAGGTA
AATGCAATGTACATTCTCACCCTCCAAATGGACAAGTAAAATCGGTTACAGAATCACGCGAAAGAATACCAC
CAAGCGCACACACTTCACACAATAACTCAAAGAAGAAAACGCCCACGGCATGTTGTTCAGTCCAGCTCAGT
ATATTATAGTCTTCAATCCAAGCCACCAAATGAAGAAAATATCAAAAGATCTTTGAAACCCAAATCTTTACT
A
>TCONS_00058080
GGACTTGCATGAAGAAGTAGATACAGAATTAGGACGTAACAAGATGCTATATCTCACCAAGTAAAATCGGTT
ACAGAATCACGCGAAAGAATACCACCAAGCGCACACACTTCACACAATAACTCAAAGAAGAAAACGCCCA
CGGCATGTTGTTCAGTCCAGCTCAGTATATTATAGTCTTCAATCCAAGCCACTGTCACAGTAGCAAGCACAGC
CAAATGAAGAAAATATCAAAAGATCTTTGAAACCCAAATCTTTACTA
>TCONS_00058094
TCCAGAAACTTTTTCCTATTGCAGAGAAAATCTAGTAAAGATTTGGGTTTCAAAGATCTTTTGATATTTTCTTC
ATTTGGCTGTGCTTGCTACTGTGACAGTGGCTTGGATTGAAGACTATAATATACTGAGCTGGACTGAACAACA
TGCCGTGGGCGTTTTCTTCTTTGAGTTATTGTGTGAAGTGTGTGCGCTTGGTGGTATTCTTTCGCGTGATTCTGT
AACCGATTTTACTTGGTGAGATATAGCATCTTGTTACGTCCTAATTCTGTATCTACTTCTTCATGCAAGTCCTAA
GTTTTATTTGTAAATATGTAATTATTTCGGTTGAACTATATCTGCTGAGGATTTACTGAAGAGAACAGTAATAT
AAAAGATATAGGAAAACTGACTACGACTCAAAAATTATAGTGGAAGATAATTTGAGTTGGTTTGGCATTAGA
GAAATGGAAATTAAAATCCGTATTCCTCTAGT
>TCONS_00058113
TTAAACAAAATGGTATGAGTATGCTTTTGTCAAGGTGGTTTCAATCATCCAAGTCCACCATCACTTCAATATC
GTTGCGTAGTGTTATTTTCTGCACATAACCGATTCCAGCGCAAGGTATGCATTTACAGATGGTCTCTTTCCCCC
TTTTGTTATAGTAACTGACCAATCCTGTCCCTTGGCAACTCCGACATTTCCCAGTCCACTCATAGACGACTGCC
TTGCAATCCTCAAATACGGGGTCACACCGGAGCCGGCGGTCAATTTCCTCCGGCGAAACAGGAGAAGTATCA
GGGTAAGTCATAGGAAGAGGAAGAGCTTCACTATCATCAGCCAAATAACTCTCTCTGTTACGTGACCAACGC
CTTTCCTTTGCATTCACTTTTGTGTTCTTTTTAATGTTATTATTCCCAATTCGCTTATT
>TCONS_00058196
TATAATGAAGTTGACTTGTCCTTTCTTTAATAAGGAGAGAAATCCATGGCCTCACACACACATCTACCTTCAA
ATGTATTTCCCCAAATGGTTTCCACTTGTTGGATCCATACGGTCAATTGTTAGCCTTCCTCATATACCTCCAAG
ACTCATCCCCGATAACCTTTAACCCCTTTTCTTTCCCTGGACTCATAGCAATTTTATCCGGTCCATGAACCTTAT
AATAGGACAACACCCCATCT
>TCONS_00058199
CATGCTTGCTTGGGCTCTTTCTTGAAGAATCTGAATTTGAACTAATACAAAGAGAGGAGACGTTGCAATTCCC
TCCTAAGCCTCCTGCAAAGAGAAACTGGTGGGGAAGTATAGTGACACCTTTTGCTGGAAATAACATGCTGGT
CCTTTACAGCTGCGTGTCAAATAACTCAAGCAAGTACTTTGTGAAAGTGCTGCACAATGAACGTCTGATTCCT
ATTACAGTAAGTTTTCTGATCACATTCTTTTGAACATGAACCACAGGTTGTTTCACCATATTATTATTCAACAT
CATTGCATTTTGAGTATCCTTAATAATTTGTTTGACGTGAGGAGTATAAAAATGAAG
>TCONS_00058200
AGATGGGGTGTTGTCCTATTATAAGGTTCATGGACCGGATAAAATTGCTATGAGTCCAGGGAAAGAAAAGGG
GTTAAAGGTTATCGGGGATGAGTCTTGGAGGTATATGAGGAAGGCTAACAATTGACCGTATGGATCCAACAA

GTGGAAACCATTTGGGGAAATACATTTGAAGGTAGATGTGTGTGTGAGGCCATGGATTTCTCTCCTTATTAAA
GAAAGGACAAGTCAACTTCATTATATGTTATATTTCCCATATAACCTGTTAACTTTACCGATT
>TCONS_00058210
ACTTTTTATAGAGTTTGGTATATGGGCTATTGATTTTCCTTTTCCTCTGCTTTCATAGGTTATGCCGTTTCAGCCC
TAGGATCCAGTAAACTTCAATTCCAAGAGAATAGTATTTTTTAGGACATGGAGCAAGTGTGCCTTTTCTCTGA
ATGCTTTGGGTATCGAGCTTTTTCATTTCAGGAAGTTCTTGATTGGCGGTTCCTCATCCGTAGAGATTTCCTTCT
TGTTTCCTTTGTCAATTGCACATAGTGAACAGCTCCTCTTGGCTGTTCAGTACAGGAGGCAATTTGCAATTTCC
ATATAAGCTAGTGTGTTGGATGTTTAATGCAATTCCTCTAGTAAGCAGCACACCGTATGATGTCAGTTGTGAC
TGTACTGTTAACTACCTCGTGCAAATTTATTTTTACTAGCGCATTGCCGATACGACACCCATTCAAGACCCTTT
GGAAACCATCCTCTGAGGGCTAAATCAGCAGATGCATGAATACATAG
>TCONS_00058223
CTGATTTAGCCCTCAGAGGATGGTTTCCAAAGGGTCTTGAATGGGTGTCGTATCGGCAATGCGCTAGTAAAAA
TAAATTTGCACGAGGTAGTTAACAGTACAGTCACAACTGACATCATACGGTGTGCTGCTTACTAGAGGAATT
GCATTAAACATCCAACACACTAGCTTATATGGAAATTGCAAATTGCCTCCTGTACTGAACAGCCAAGAGGAG
CTGTTCACTATGTGCAATTGACAAAGGAAACAAGAAGGAAATCTCTACGGATGAGGAACCGCCAATCAAGA
ACTTCCTGAAATGAAAAAGCTCGATACCCAAAGCATTCAGAGAAAAGGCACACTTGCTCCATGTCCTAAAAA
ATACTATTCTCTTGGAATTGAAGTTTACTGGATCCTAGGGCTGAAACGGCATAACCTATGAAAGCAGAGGAA
AAGGAAAATCAATAGCCCATATACCAAACTCTATAAAAAG
>TCONS_00058233
CTTCCCTTTCAATGCCTTTTCCGATCATCTTTTATGCTGCCGCCGATTATGGAACAGGCCGGTTTACCGCCGTC
GTTTGGCGGCGTTGGGATTAAATCAAATAGGAATCCTCGATCTAATTTCGAACTCAAATTCCCAATTACAAGC
CCTACCTGAGAAGGAAGTCCAGATTTGCCGAGCCGATTTTCAGCTAATTTGAGCTGTATTTCTTCTCCCACTAC
TGCTTTCACC
>TCONS_00058310
ACTCATGTCCAAAGGAAATTAGACAAAAAGATGGAAGATAAGAAATGAAGAGAAGCATTTTAGTCCTTGAT
CTCTCATGGAAGACGAAGACTAACATCTTAATACAGCATGCTCAATTCACATAGGAGGAATATGCGGCTCAA
ACTCAACTAAATTCGATTTTCTGGACCATCTATCTGGCTCAATATGAGAACTCACGACCTCGAAGCAAAATAT
ATTTCTTAACTCAGACGGCAATGTCCTAGGCACAAGCCTACGATAATCTATCATATACTCTACAATATTTAAA
C
>TCONS_00058374
GACCAGAAACTTGTTCTACATCCAGATTGTTTTCTACATAATATTGGAAGTTGAGAGTGGGGATGTTGGCTTG
CTACTAGGCATTCACCAATAATAACTTGAGCCTTAGAACTTATTATGGATCTACTTTCTGTGGAGTAGATCAA
GAAATGGATGATGAAGACGATGAAGAACTAGACCTTACTTGAAATGAATTTTGAATTGTTATAGATGAATGT
TCTAGGAATCTTTTGTTACCAACATTTTTGAACTTATTTACTCTCTTGAAATGTACAAAACTTATGCACTCA
>TCONS_00058379
TCTGTTATGGTATTGCTGTCGTGCCGTTCCAGTAGTGTTTGTTAATTTGTAGTTTTGAGTGGGGGGAATGAAAA
AAGGTAAATAAAGACCTTAGCCAAAAGCAAGGGATTCCCGGGGCCCGAACGAGAGCAGAGGCAGCAGGGC
AGGTATTCTCAAAAAAGAAAGAAACGAAAACAGGCAGGGAGGTAAGAGCGAGTAAGACTAGGTTATAGCA
AGACAACAGAGGTTAGATAGCTCTTACCGGAGGAAGAGGGTTCAGGTCACCCGTCCCCTTGACCCAGACAC
GAACTAATCCTTCTCTCTCTCGAATGTATGCCCGGTTTTCGTCGAATCTTTTTGATCACCTTCAAAAACAAACC
GGAATTCCGTCTTGACATGATAAATAGTGATGCCCACCTGCAGAGCGTAGTCCTCCCTGCAGCCTTGAAGTAG
ACTATTTCTCGAGTCTGATCTTATCCCCAAAGCCTTTTTGCAAAAGGAGTAGGTAGCGAGATAGATGACTAAA

AGCTGATGATCTTTGCCTCTTACTAGTAAAGGGAAAAGCCCTATATATCTTATTAGTCAAGCCTAAAGGCCCT
TTACTAATATAATAGAAGGTAAGACCGGCTTTCGCGCAGCAGCTGCGCCCTTGCTTCTTGCCCATAGAGAGTT
AGGATATTTTGTGAGCTCCTTCGCCTAGCGGAAACCTCCGCTTTTCGGCCGTAATCCCGTGCTTTACCAACGCA
TTCAATGGATTCCACCATGAACCGGGAGCGGGCGAAGACATGCAGCTTCCATTTTCGGGGTACACAGTCACG
TGCTGAGCAAAGAGGTATATATCACACTCATTCACAAGCGCAAGGTGCGGCATCCGCCTGAACGGGGAGAG
GATTTCCCTAAAACAAAGATAGGCGTGTCAGGAAACAATGTGAAGTCACCCCTATTTCGCTGTCCAGGGAGC
TGTTGACTAATGACCTAAAAAATGCGTGACGTTTGGGAAGCATGAGCAACCCTTCGCGCACGAGATAGCAAT
GACAGGAGATTGACCGGTCGGGGTCGAGTGATCTCAGGGGTTTAGGGGTGCTTCTCCTGCTGCGACTACCTCT
CTATTTTCAAAAGTATATAGAAATGGTAGGCACTGGGAGTGAGTGAACTACCCGGGGGAGAAAGGGGCAAC
CTCTCTATAGAAGGGAAGGGAAAGGGTTATCCGCTCATAATGATTGTAGAGTCTCTCGGGGTTGGAGGTCTA
ATAGTAGTCACATCAAGTCCTCCCCCTCTTACTCAGAATAGCTAGCGAGAGGAATACTACTGATTATCTATGA
AAGAAGGCTTTTAAGAATGAAAAAAATCTTATCTTAAGGATAGAGAGCCCCCCGCCTGCCTTTTCAGATACG
AGTGAGTGAGCGCTTTTTCTGCTATGAATGAATGACCTCTCCATTTATTTTCAAGCTTGCTCTTCAAACCGGCC
TATCGCTCTCCTCTATGAAAAGGTTCTTCCGTAATCACTCTCAATAAGACATCCATTCCCCTTTCAAATGAAAA
GAGAACTAGACTTTTCTCTTCATCATTGAAATCCGCCATTCAATCGCAG
>TCONS_00058380
TCTGTTATGGTATTGCTGTCGTGCCGTTCCAGTAGTGTTTGTTAATTTGTAGTTTTGAGTGGGGGGAATGAAAA
AAGGTAAATAAAGACCTTAGCCAAAAGCAAGGGATTCCCGGGGCCCGAACGAGAGCAGAGGCAGCAGGGC
AGGTATTCTCAAAAAAGAAAGAAACGAAAACAGGCAGGGAGGTAAGAGCGAGTAAGACTAGGTTATAGCA
AGACAACAGAGGTTAGATAGCTCTTACCGGAGGAAGAGGGTTCAGGTCACCCGTCCCCTTGACCCAGACAC
GAACTAATCCTTCTCTCTCTCGAATGTATGCCCGGTTTTCGTCGAATCTTTTTGATCACCTTCAAAAACAAACC
GGAATTCCGTCTTGACATGATAAATAGTGATGCCCACCTGCAGAGCGTAGTCCTCCCTGCAGCCTTGAAGTAG
ACTATTTCTCGAGTCTGATCTTATCCCCAAAGCCTTTTTGCAAAAGGAGTAGGTAGCGAGATAGATGACTAAA
AGCTGATGATCTTTGCCTCTTACTAGTAAAGGGAAAAGCCCTATATATCTTATTAGTCAAGCCTAAAGGCCCT
TTACTAATATAATAGAAGGTAAGACCGGCTTTCGCGCAGCAGCTGCGCCCTTGCTTCTTGCCCATAGAGAGTT
AGGATATTTTGTGAGCTCCTTCGCCTAGCGGAAACCTCCGCTTTTCGGCCGTAATCCCGTGCTTTACCAACGCA
TTCAATGGATTCCACCATGAACCGGGAGCGGGCGAAGACATGCAGCTTCCATTTTCGGGGTACACAGTCACG
TGCTGAGCAAAGAGGTATATATCACACTCATTCACAAGCGCAAGGTGCGGCATCCGCCTGAACGGGGAGAG
GATTTCCCTAAAACAAAGATAGGCGTGTCAGGAAACAATGTGAAGTCACCCCTATTTCGCTGTCCAGGGAGC
TGTTGACTAATGACCTAAAAAATGCGTGACGTTTGGGAAGCATGAGCAACCCTTCGCGCACGAGATAGCAAT
GACAGGAGATTGACCGGTCGGGGTCGAGTGATCTCAGGGGTTTAGGGGTGCTTCTCCTGCTGCGACTACCTCT
CTATTTTCAAAAGTATATAGAAATGGTAGGCACTGGGAGTGAGTGAACTACCCGGGGGAGAAAGGGGCAAC
CTCTCTATAGAAGGGAAGGGAAAGGGTTATCCGCTCATAATGATTGTAGAGTCTCTCGGGGTTGGAGGTCTA
ATAGTAGTCACATCAAGTCCTCCCCCTCTTACTCAGAATAGCTAGCGAGAGGAATACTACTGATTATCTATGA
AAGAAGGCTTTTAAGAATGAAAAAAATCTTATCTTAAGGATAGAGAGCCCCCCGCCTGCCTTTTCAGATACG
AGTGAGTGAGCGCTTTTTCTGCTATGAATGAATGACCTCTCCATTTATTTTCAAGCTTGCTCTTCAAACCGGCC
TATCGCTCTCCTCTATGAAAAGGTTCTTCCGTAATCACTCTCAATAAGACATCCATTCCCCTTTCAAATGAAAA
GAGAACTAGACTTTTCTCTTCATCATTGAAATCCGCCATTCAATCGCAG
>TCONS_00058381
GGGGGAATGAAAAAAGGTAAATAAAGACCTTAGCCAAAAGCAAGGGATTCCCGGGGCCCGAACGAGAGCA
GAGGCAGCAGGGCAGGTATTCTCAAAAAAGAAAGAAACGAAAACAGGCAGGGAGGTAAGAGCGAGTAAG
ACTAGGTTATAGCAAGACAACAGAGGTTAGATAGCTCTTACCGGAGGAAAAGGGTTCAGGTCACCCGTCCCC

TTGACCCAGACACGAACTAATCCTTCTCTCTCTCGAATGTATGCCCGGTTTTTGTCGAATCTTTTTGATCACCTT
CAAAAACAAACCGGAATTCCGTCTTGACATGATAAATAGTGATGCCCACCTGCAGAGCGTAGTCCTCCCTGC
AGCCTTGAAGTAGACTATTTCTCGAGTCTGATCTTATCCCCAAAGCCTTTTTGCAAAAGGAGTAGGTAGCGAG
ATAGATGACTAAAAGCTGATGATCTTTGCCTCTTACTAGTAAAGGGAAAAGCCCTATATATCTTATTAGTCAA
GCCTAAAGGCCCTTTACTAATATAATAGAAGGTAAGACCGGCTTTCGCGCAGCAGCTGCGCCCTTGCTTCTTG
CCCATAGAGAGTTAGGATATTTTGTGAGCTCCTTCGCCTAGCGGAAACCTCCGCTTTTCGGCCGTAATCCCGT
GCTTTACCAACGCATTCAATGGATTCCACCATGAACCGGGAGCGGGCGAAGACATGCAGCTTCCATTTTCGG
GGTACACAGTCACGTGCTGAGCAAAGAGGTATATATCACACTCATTCACAAGCGCAAGGTGCGGCATCCGCC
TGAACGGGGAGAGGATTTCCCTAAAACAAAGATAGGCGTGTCAGGAAACAATGTGAAGTCACCCCTATTTCG
CTGTCCAGGGAGCTGTTGACTAATGACCTAAAAAATGCGTGACGTTTGGGAAGCATGAGCAACCCTTCGCGC
ACGAGATAGCAATGACAGGAGATTGACCGGTCGGGGTCGAGTGATCTCAGGGGTTTAGGGGTGCTTCTCCTG
CTGCGACTACCTCTCTATTTTCAAAAGTATATAGAAATGGTAGGCACTGGGAGTGAGTGAACTACCCGGGGG
AGAAAGGGGCAACCTCTCTATAGAAGGGAAGGGAAAGGGTTATCCGCTCATAATGATTGTAGAGTCTCTCGG
GGTTGGAGGTCTAATAGTAGTCACATCAAGTCCTCCCCCTCTTACTCAGAATAGCTAGCGAGAGGAATACTA
CTGATTATCTATGAAAGAAGGCTTTTAAGAATGAAAAAAATCTTATCTTAAGGATAGAGAGCCCCCCGCCTG
CCTTTTCAGATACGAGTGAGTGAGCGCTTTTTCTGCTATGAATGAATGACCTCTCCATTTATTTCAAGCTTGCT
CTTCAAACCGGCCTATCGCTCTCCTCTATGAAAAGGTTCTTCCGTAATCACTCTCAATAAGACATCCATTCCCC
TTTCAAATGAAAAGAGAACTAGACTTTTCTCTTCATCATTGAAATCCGCCATTCAATCGCAG
>TCONS_00058387
CGATTGAATGGCGGATTTCAATGATGAAGAGAAAAGTCTAGTTCTCTTTTCATTTGAAAGGGGAATGGATGTC
TTATTGAGAGTGATTACGGAAGAACCTTTTCATAGAGGAGAGCGATAGGCCGGTTTGAAGAGCAAGCTTGAA
AATAAATGGAGAGGTCATTCATTCATAGCAGAAAAAGCGCTCACTCACTCGTATCTGAAAAGGCAGGCGGG
GGGCTCTCTATCCTTAAGATAAGATTTTTTTCATTCTTAAAAGCCTTCTTTCATAGATAATCAGTAGTATTCCTC
TCGCTAGCTATTCTGAGTAAGAGGGGGAGGACTTGATGTGACTACTATTAGACCTCCAACCCCGAGAGACTC
TACAATCATTATGAGCGGATAACCCTTTCCCTTCCCTTCTATAGAGAGGTTGCCCCTTTCTCCCCCGGGTAGTT
CACTCACTCCCAGTGCCTACCATTTCTATATACTTTTGAAAATAGAGAGGTAGTCGCAGCAGGAGAAGCACC
CCTAAACCCCTGAGATCACTCGACCCCGACCGGTCAATCTCCTGTCATTGCTATCTCGTGCGCGAAGGGTTGC
TCATGCTTCCCAAACGTCACGCATTTTTTAGGTCATTAGTCAACAGCTCCCTGGACAGCGAAATAGGGGTGAC
TTCACATTGTTTCCTGACACGCCTATCTTTGTTTTAGGGAAATCCTCTCCCCGTTCAGGCGGATGCCGCACCTT
GCGCTTGTGAATGAGTGTGATATATACCTCTTTGCTCAGCACGTGACTGTGTACCCCGAAAATGGAAGCTGCA
TGTCTTCGCCCGCTCCCGGTTCATGGTGGAATCCATTGAATGCGTTGGTAAAGCACGGGATTACGGCCGAAAA
GCGGAGGTTTCCGCTAGGCGAAGGAGCTCACAAAATATCCTAACTCTCTATGGGCAAGAAGCAAGGGCGCA
GCTGCTGCGCGAAAGCCGGTCTTACCTTCTATTATATTAGTAAAGGGCCTTTAGGCTTGACTAATAAGATATA
TAGGGCTTTTCCCTTTACTAGTAAGAGGCAAAGATCATCAGCTTTTAGTCATCTATCTCGCTACCTACTCCTTTT
GCAAAAAGGCTTTGGGGATAAGATCAGACTCGAGAAATAGTCTACTTCAAGGCTGCAGGGAGGACTACGCT
CTGCAGGTGGGCATCACTATTTATCATGTCAAGACGGAATTCCGGTTTGTTTTTGAAGGTGATCAAAAAGATT
CGACGAAAACCGGGCATACATTCGAGAGAGAGAAGGATTAGTTCGTGTCTGGGTCAAGGGGACGGGTGACC
TGAACCCTCTTCCTCCGGTAAGAGCTATCTAACCTCTGTTGTCTTGCTATAACCTAGTCTTACTCGCTCTTACCT
CCCTGCCTGTTTTCGTTTCTTTCTTTTTTGAGAATACCTGCCCTGCTGCCTCTGCTCTCGTTCGGGCCCCGGGAA
TCCCTTGCTTTTGGCTAAGGTCTTTATTTACCTTTTTTCATTCCCCCCCTCAAAACTACAAATTAACAAACACTA
CTGGAACGGCACGACAGCAATACCATAACAGATAGACTTACCGACAAGAGAGAGCGAGACTGACCTGAAC
CCTTTTCCTCCGGTAAGAGCTATCTAACCTCTGTTGTCTTGCTATAACCTAGTCTTACTCGCTCTTACCTCCCTG

CCTGTTTTCGTTTCTTTCTTTTTTGAGAATACCTGCCCTGCTGCCTCTGCTCTCGTTCGGGCCCCGGGAATCCCTT
GCTTTTGGCTAAGGTCTTTATTTACCTTTTTTCATTCCCCCCACTCAAAACTACAAATTAACAAACACTACTG
>TCONS_00058388
CGCACTGCGATTGAATGGCGGATTTCAATGATGAAGAGAAAAGTCTAGTTCTCTTTTCATTTGAAAGGGGAAT
GGATGTCTTATTGAGAGTGATTACGGAAGAACCTTTTCATAGAGGAGAGCGATAGGCCGGTTTGAAGAGCAA
GCTTGAAAATAAATGGAGAGGTCATTCATTCATAGCAGAAAAAGCGCTCACTCACTCGTATCTGAAAAGGCA
GGCGGGGGGCTCTCTATCCTTAAGATAAGATTTTTTTCATTCTTAAAAGCCTTCTTTCATAGATAATCAGTAGT
ATTCCTCTCGCTAGCTATTCTGAGTAAGAGGGGGAGGACTTGATGTGACTACTATTAGACCTCCAACCCCGAG
AGACTCTACAATCATTATGAGCGGATAACCCTTTCCCTTCCCTTCTATAGAGAGGTTGCCCCTTTCTCCCCCGG
GTAGTTCACTCACTCCCAGTGCCTACCATTTCTATATACTTTTGAAAATAGAGAGGTAGTCGCAGCAGGAGAA
GCACCCCTAAACCCCTGAGATCACTCGACCCCGACCGGTCAATCTCCTGTCATTGCTATCTCGTGCGCGAAGG
GTTGCTCATGCTTCCCAAACGTCACGCATTTTTTAGGTCATTAGTCAACAGCTCCCTGGACAGCGAAATAGGG
GTGACTTCACATTGTTTCCTGACACGCCTATCTTTGTTTTAGGGAAATCCTCTCCCCGTTCAGGCGGATGCCGC
ACCTTGCGCTTGTGAATGAGTGTGATATATACCTCTTTGCTCAGCACGTGACTGTGTACCCCGAAAATGGAAG
CTGCATGTCTTCGCCCGCTCCCGGTTCATGGTGGAATCCATTGAATGCGTTGGTAAAGCACGGGATTACGGCC
GAAAAGCGGAGGTTTCCGCTAGGCGAAGGAGCTCACAAAATATCCTAACTCTCTATGGGCAAGAAGCAAGG
GCGCAGCTGCTGCGCGAAAGCCGGTCTTACCTTCTATTATATTAGTAAAGGGCCTTTAGGCTTGACTAATAAG
ATATATAGGGCTTTTCCCTTTACTAGTAAGAGGCAAAGATCATCAGCTTTTAGTCATCTATCTCGCTACCTACT
CCTTTTGCAAAAAGGCTTTGGGGATAAGATCAGACTCGAGAAATAGTCTACTTCAAGGCTGCAGGGAGGACT
ACGCTCTGCAGGTGGGCATCACTATTTATCATGTCAAGACGGAATTCCGGTTTGTTTTTGAAGGTGATCAAAA
AGATTCGACGAAAACCGGGCATACATTCGAGAGAGAGAAGGATTAGTTCGTGTCTGGGTCAAGGGGACGGG
TGACCTGAACCCTCTTCCTCCGGTAAGAGCTATCTAACCTCTGTTGTCTTGCTATAACCTAGTCTTACTCGCTCT
TACCTCCCTGCCTGTTTTCGTTTCTTTCTTTTTTGAGAATACCTGCCCTGCTGCCTCTGCTCTCGTTCGGGCCCCG
GGAATCCCTTGCTTTTGGCTAAGGTCTTTATTTACCTTTTTTCATTCCCCCCACTCAAAACTACAAATTAACAA
ACACTACTG
>TCONS_00058389
CGCACTGCGATTGAATGGCGGATTTCAATGATGAAGAGAAAAGTCTAGTTCTCTTTTCATTTGAAAGGGGAAT
GGATGTCTTATTGAGAGTGATTACGGAAGAACCTTTTCATAGAGGAGAGCGATAGGCCGGTTTGAAGAGCAA
GCTTGAAAATAAATGGAGAGGTCATTCATTCATAGCAGAAAAAGCGCTCACTCACTCGTATCTGAAAAGGCA
GGCGGGGGGCTCTCTATCCTTAAGATAAGATTTTTTTCATTCTTAAAAGCCTTCTTTCATAGATAATCAGTAGT
ATTCCTCTCGCTAGCTATTCTGAGTAAGAGGGGGAGGACTTGATGTGACTACTATTAGACCTCCAACCCCGAG
AGACTCTACAATCATTATGAGCGGATAACCCTTTCCCTTCCCTTCTATAGAGAGGTTGCCCCTTTCTCCCCCGG
GTAGTTCACTCACTCCCAGTGCCTACCATTTCTATATACTTTTGAAAATAGAGAGGTAGTCGCAGCAGGAGAA
GCACCCCTAAACCCCTGAGATCACTCGACCCCGACCGGTCAATCTCCTGTCATTGCTATCTCGTGCGCGAAGG
GTTGCTCATGCTTCCCAAACGTCACGCATTTTTTAGGTCATTAGTCAACAGCTCCCTGGACAGCGAAATAGGG
GTGACTTCACATTGTTTCCTGACACGCCTATCTTTGTTTTAGGGAAATCCTCTCCCCGTTCAGGCGGATGCCGC
ACCTTGCGCTTGTGAATGAGTGTGATATATACCTCTTTGCTCAGCACGTGACTGTGTACCCCGAAAATGGAAG
CTGCATGTCTTCGCCCGCTCCCGGTTCATGGTGGAATCCATTGAATGCGTTGGTAAAGCACGGGATTACGGCC
GAAAAGCGGAGGTTTCCGCTAGGCGAAGGAGCTCACAAAATATCCTAACTCTCTATGGGCAAGAAGCAAGG
GCGCAGCTGCTGCGCGAAAGCCGGTCTTACCTTCTATTATATTAGTAAAGGGCCTTTAGGCTTGACTAATAAG
ATATATAGGGCTTTTCCCTTTACTAGTAAGAGGCAAAGATCATCAGCTTTTAGTCATCTATCTCGCTACCTACT
CCTTTTGCAAAAAGGCTTTGGGGATAAGATCAGACTCGAGAAATAGTCTACTTCAAGGCTGCAGGGAGGACT

ACGCTCTGCAGGTGGGCATCACTATTTATCATGTCAAGACGGAATTCCGGTTTGTTTTTGAAGGTGATCAAAA
AGATTCGACGAAAACCGGGCATACATTCGAGAGAGAGAAGGATTAGTTCGTGTCTGGGTCAAGGGGACGGG
TGACCTGAACCCTCTTCCTCCGGTAAGAGCTATCTAACCTCTGTTGTCTTGCTATAACCTAGTCTTACTCGCTCT
TACCTCCCTGCCTGTTTTCGTTTCTTTCTTTTTTGAGAATACCTGCCCTGCTGCCTCTGCTCTCGTTCGGGCCCCG
GGAATCCCTTGCTTTTGGCTAAGGTCTTTATTTACCTTTTTTCATTCCCCCCACTCAAAACTACAAATTAACAA
ACACTACTG
>TCONS_00058390
CGCACTGCGATTGAATGGCGGATTTCAATGATGAAGAGAAAAGTCTAGTTCTCTTTTCATTTGAAAGGGGAAT
GGATGTCTTATTGAGAGTGATTACGGAAGAACCTTTTCATAGAGGAGAGCGATAGGCCGGTTTGAAGAGCAA
GCTTGAAAATAAATGGAGAGGTCATTCATTCATAGCAGAAAAAGCGCTCACTCACTCGTATCTGAAAAGGCA
GGCGGGGGGCTCTCTATCCTTAAGATAAGATTTTTTTCATTCTTAAAAGCCTTCTTTCATAGATAATCAGTAGT
ATTCCTCTCGCTAGCTATTCTGAGTAAGAGGGGGAGGACTTGATGTGACTACTATTAGACCTCCAACCCCGAG
AGACTCTACAATCATTATGAGCGGATAACCCTTTCCCTTCCCTTCTATAGAGAGGTTGCCCCTTTCTCCCCCGG
GTAGTTCACTCACTCCCAGTGCCTACCATTTCTATATACTTTTGAAAATAGAGAGGTAGTCGCAGCAGGAGAA
GCACCCCTAAACCCCTGAGATCACTCGACCCCGACCGGTCAATCTCCTGTCATTGCTATCTCGTGCGCGAAGG
GTTGCTCATGCTTCCCAAACGTCACGCATTTTTTAGGTCATTAGTCAACAGCTCCCTGGACAGCGAAATAGGG
GTGACTTCACATTGTTTCCTGACACGCCTATCTTTGTTTTAGGGAAATCCTCTCCCCGTTCAGGCGGATGCCGC
ACCTTGCGCTTGTGAATGAGTGTGATATATACCTCTTTGCTCAGCACGTGACTGTGTACCCCGAAAATGGAAG
CTGCATGTCTTCGCCCGCTCCCGGTTCATGGTGGAATCCATTGAATGCGTTGGTAAAGCACGGGATTACGGCC
GAAAAGCGGAGGTTTCCGCTAGGCGAAGGAGCTCACAAAATATCCTAACTCTCTATGGGCAAGAAGCAAGG
GCGCAGCTGCTGCGCGAAAGCCGGTCTTACCTTCTATTATATTAGTAAAGGGCCTTTAGGCTTGACTAATAAG
ATATATAGGGCTTTTCCCTTTACTAGTAAGAGGCAAAGATCATCAGCTTTTAGTCATCTATCTCGCTACCTACT
CCTTTTGCAAAAAGGCTTTGGGGATAAGATCAGACTCGAGAAATAGTCTACTTCAAGGCTGCAGGGAGGACT
ACGCTCTGCAGGTGGGCATCACTATTTATCATGTCAAGACGGAATTCCGGTTTGTTTTTGAAGGTGATCAAAA
AGATTCGACAAAAACCGGGCATACATTCGAGAGAGAGAAGGATTAGTTCGTGTCTGGGTCAAGGGGACGGG
TGACCTGAACCCTTTTCCTCCGGTAAGAGCTATCTAACCTCTGTTGTCTTGCTATAACCTAGTCTTACTCGCTCT
TACCTCCCTGCCTGTTTTCGTTTCTTTCTTTTTTGAGAATACCTGCCCTGCTGCCTCTGCTCTCGTTCGGGCCCCG
GGAATCCCTTGCTTTTGGCTAAGGTCTTTATTTACCTTTTTTCATTCCCCCCACTCAAAACTACAAATTAACAA
ACACTACTG
>TCONS_00058391
CTCCCCCCCCAAAAAAAAGAGAGACTTCATTTCTTCCACCTTCCCTGTATGAGTAGTGAAGAAGTATAGAGC
ACTTTAGTTGGTTGTTGACCAACGGAAAGCATGGAAGAAAAAAAGGAAGAATTTACGCAATTTCTTTTCTAA
GAAAGAGATCATAGTGATTGATGGCTCTTTCAATCCGCCGAAATGGAACGCTTTGGCGTGTACCAAAATGGA
GAATTTGGGTCAGATTTATAAGCTTACGACCACTGAACAAACTTGGTTGACGAACATAGTTTATGCGCCGCTA
ATGTAGCGGCTTGTCGAGCATTTGACAAACTCACACCATCCATTTCAAATAGGATTTGTCCCCTGGACACACG
AGCAATCCAA
>TCONS_00058485
ACCTATCTACCGCTGGATTGAAGTTTGCAGCATAGCAGAGTTGTGGAGTTAGTATGCAGATTGGTTAAAACAG
GGCAAAATGGCAAAATGCTCAAACACTTGAGAAAAGAGGCATCATATAGATCCCTGGAGTAAGGTTAAGAA
ATATGGATACCTTGTTGCTAAAAAACACCCGATCGTACTTGTAGCGATGTGCTGGATACACCCAAGAATTGCA
GATGAAATGAACTTCACCATGTCCAGGAATATCTTCTAAAGCGACGGTCTTCAGTTTCTTTGCATCTCTTTGCT
CAGCTTTAGGAGAGTGTAAATTACCAGGATCAGCATGGACAGCACTAATGAGCTGTAGGATGACGCGTTTGC

CAAAGAGCTCGTGCATTCGATCAAGGAAGGCAGAACCAGCATCCTTGAAGTTCAGAACATTTTTCTTCCTCA
GTACAACTGTCCCCTTGATCTTAATAGGACTGGTTTGTTTGCTAGAATCGCTTTCTCTTGGAACATCATGCTGCT
TCCCGCAAACCATTTCCAGCATTTTCTCCAGTACCTCTGTACAACATGGTGGAAAGATCCCCATTGAACAAAA
TGGAGATACAGAACAAGTCCTAAAGATATGTTTTTGAGTTGAAATA
>TCONS_00058487
GTTCAACTTGTGTGATTTCAGATTCAGCGATCCATTTATGTCATGTGACTTCAGATTGAGATGCTATTGGGAAT
TCCCTTTCCAGTAAGTCCAGTAGTACTCTTGTCACCAGACGCGTTTGGGTAGAGTAACGTATAAGGCATCTGT
ATAGGTCCTTTTCGGTTCTTCAATGTATTGTCATTGTTCCTATCCATTATTCTGTTCTCGATTTCTACCAGTTTGT
CACGGAATCCCTCAAATGCATGTCGTGGTTGACTGTCAGAAGTCCATTCAGGAGTATCTCTTTGTCCAAGATA
AATCTCATCTGAAGAATGCATGGATAACATCTCTATCAAAGAGATGCCAAGAAGAGTTTGGAACTGGGCTGT
GATGGTCTTTAGGTAGGCTCGCTCATGGTTTGACTCAAGCTCAACATACTCAGGAGTGCCTGGCATTGGCATA
AAGCGCCGGCTAACGGTTGGGCGATTTGGGAGATAGCCAGCATAAGGATACTGCCCAAAATTAACTGCTGCA
TGAAGAGCAGAAGCCACCCATATGATTATGGTGCATGTTTGGACAAGCTCAGCTCTTGTCTGCATCTGAGGCC
ACCATGGTTCATCTTTCAAGTCACCATGACCCACGTTGCGAACTTCTGTCCACCATGATTGAAGTTCAGAATC
ACCTTGGATCATGTCATCCGCTAAGTAATAGAATAAGCAATACTCGTCCACCCAGGTTTCAATTGCTGACCAA
ATCTCAAGTCCATCAACGGCGTAAGGATAATCCTCTATCAGAAGTCTAAGACCATGAGGCTGGCTTGGATCT
GTGGCCTACCTCTTAAGTAGATCTGCAGGGAGTGCCTGCTCAGTGAAAACCCAATTCTTATAGATGGAAGAA
GACATTTCCATGGCATATTTGGATGGAAATACTGTCAACTCAAGTACTCCACCTGCATTAATGAGGATCTGAC
GAGCCAAAGCATTGATGTTC
>TCONS_00058527
TCATTGAGAAAACTGAGTGGAATTTCATGGTTGTAAAATTACAGAGGAGCTTTCATGGTTATGTCTGCCTTCA
TATGTAGTAATCAAATAGCTTGAATCATGTCCATCTCTTTCTATCCTCTTCTTTACCTTGCACCCTCCACTTGAG
CATTTGTAGTAATTCCTTAGATTTGTGTTACTTTTCACCTTCTTCTTCCCATACTTCCTCCATTTATATCCATCAT
ACAAGATCTCAAGTTG
>TCONS_00058587
TCCACCGGTAATGTGTGGAAGAGGGTAAGATTTCTATGTGGATAGGACGTATCAACACCTTTTGCATCTTTTC
ATTTTAAAGGAAATATAGTTGAATAATATAGTGTTGGGTCCTGAGGACCAGGATGGCCGAAGATCAAAACCT
AAATGTGCCAGGGGTTGATCATCGAAGCCTGGGCAATAACGATTAAGGAATTTGAGCGCTGATGTAGCTAAG
AGCCACCTTCATAGTCAATTCATTAGGGTTGTCACCTTCTACCCGGAAAAGTATCCACCGTTTTCTTTATCTGT
TAAGCCTCACAGCTATGGGACTTCCTAAAGAAAACTGCAATCGCAGTTTATCAACCACTCACAAGACCACCG
AGATCTTCGACTTAGCTCCCAAAGGTAATCCACCCGGCCCTTTCCCAACCTATACAAGGAGAGCGGAAGATA
ACGAAAGGGAGACAAGGTCCTAACTAAATAGAACACAAACTCCCATTCCATCTATCATATCAGTCGAGTCGA
TCCGATTCGTTCTTATCTATAGATAACGCAAGAGAGAACTTATGACCTCGCGGATGGAGAGGGATTCGAACC
CCCGGTATTTCTATAAATACTTCGGTTTTCAAGACCGACTCTTTCAACCGCTCAGACATCCATCCCCTTCGCGC
TTCGCCCGCCCTATCCATCCGCGCGCTGGCAGGTAGGGGCTTATCTATGTGATTCGCTACGCTTGCCTGCGCCT
ATCGGCTGATTCTATTGCCTGCCGGTTAGCGAAACTTTTCCGGTAGTACGAATCGCTACGCTTCGCCTCTTTCT
ATATGATAATATGCATCTTGGTTGCTGCGCTCCCTGCGGGTAATAGCAAGAGAGTGAATAACGAATGATCCTC
CAAACTAAAAGAGTGATTGAGTCCGACTCAAGCGAGTGAGTGGAAGGCGTGGCAAGAGAGCGAGTTCGACT
CGCGAACGATCAGCAAGTGAAGGACCGATCCTTTTGCGCAAATACTGTTGCGAGATTGGTACTTGGCAGCT
>TCONS_00058588
TCCACCGGTAATGTGTGGAAGAGGGTAAGATTTCTATGTGGATAGGACGTATCAACACCTTTTGCATCTTTTC
ATTTTAAAGGAAATATAGTTGAATAATATAGTGTTGGGTCCTGAGGACCAGGATGGCCGAAGATCAAAACCT

AAATGTGCCAGGGGTTGATCATCGAAGCCTGGGCAATAACGATTAAGGAATTTGAGCGCTGATGTAGCTAAG
AGCCACCTTCATAGTCAATTCATTAGGGTTGTCACCTTCTACCCGGAAAAGTATCCACCGTTTTCTTTATCTGT
TAAGCCTCACAGCTATGGGACTTCCTAAAGAAAACTGCAATCGCAGTTTATCAACCACTCACAAGACCACCG
AGATCTTCGACTTAGCTCCCAAAGGTAATCCACCCGGCCCTTTCCCAACCTATACAAGGAGAGCGGAAGATA
ACGAAAGGGAGACAAGGTCCTAACTAAATAGAACACAAACTCCCATTCCATCTATCATATCAGTCGAGTCGA
TCCGATTCGTTCTTATCTATAGATAACGCAAGAGAGAACTTATGACCTCGCGGATGGAGAGGGATTCGAACC
CCCGGTATTTCTATAAATACTTCGGTTTTCAAGACCGACTCTTTCAACCGCTCAGACATCCATCCCCTTCGCGC
TTCGCCCGCCCTATCCATCCGCGCGCTGGCAGGTAGGGGCTTATCTATGTGATTCGCTACGCTTGCCTGCGCCT
ATCGGCTGATTCTATTGCCTGCCGGTTAGCGAAACTTTTCCGGTAGTACGAATCGCTACGCTTCGCCTCTTTCT
ATATGATAATATGCATCTTGGTTGCTGCGCTCCCTGCGGGTAATAGCAAGAGAGTGAATAACGAATGATCCTC
CAAACTAAAAGAGTGATTGAGTCCGACTCAAGCGAGTGAGTGGAAGGCGTGGCAAGAGAGCGAGTTCGACT
CGCGAACGATCAGCAAGTGAAGGACCGATCCTTTTGCGCAAATACTGTTGCGAGATTGGTACTTGGCAGCT
>TCONS_00058591
AAGTACCAATCTCGCAACAGTATTTGCGCAAAAGGATCGGTCCTTCACTTGCTGATCGTTCGCGAGTCGAACT
CGCTCTCTTGCCACGCCTTCCACTCACTCGCTTGAGTCGGACTCAATCACTCTTTTAGTTTGGAGGATCATTCG
TTATTCACTCTCTTGCTATTACCCGCAGGGAGCGCAGCAACCAAGATGCATATTATCATATAGAAAGAGGCG
AAGCGTAGCGATTCGTACTACCGGAAAAGTTTCGCTAACCGGCAGGCAATAGAATCAGCCGATAGGCGCAG
GCAAGCGTAGCGAATCACATAGATAAGCCCCTACCTGCCAGCGCGCGGATGGATAGGGCGGGCGAAGCGCG
AAGGGGATGGATGTCTGAGCGGTTGAAAGAGTCGGTCTTGAAAACCGAAGTATTTATAGAAATACCGGGGGT
TCGAATCCCTCTCCATCCGCGAGGTCATAAGTTCTCTCTTGCGTTATCTATAGATAAGAACGAATCGGATCGA
CTCGACTGATATGATAGATGGAATGGGAGTTTGTGTTCTATTTAGTTAGGACCTTGTCTCCCTTTCGTTATCTTC
CGCTCTCCTTGTATAGGTTGGGAAAGGGCCGGGTGGATTACCTTTGGGAGCTAAGTCGAAGATCTCGGTGGTC
TTGTGAGTGGTTGATAAACTGCGATTGCAGTTTTCTTTAGGAAGTCCCATAGCTGTGAGGCTTAACAGATAAA
GAAAACGGTGGATACTTTTCCGGGTAGAAGGTGACAACCCTAATGAATTGACTATGAAGGTGGCTCTTAGCT
ACATCAGCGCTCAAATTCCTTAATCGTTATTGCCCAGGCTTCGATGATCAACCCCTGGCACATTTAGGTTTTGA
TCTTCGGCCATCCTGGTCCTCAGGACCCAACACTATATTATTCAACTATATTTCCTTTAAAATGAAAAGATGCA
AAAGGTGTTGATACGTCCTATCCACATAGAAATCTTACCCTCTTCCACACATTACCGGTGGATGCTACAGCCG
CTATCACTTTGGCTGCAGGATGGGAATGAAGGTCGAGGAGGCAGGGAAGAAAAGGTTATTCGCTATTGGCTT
ACCCTTATATCAGGC
>TCONS_00058592
AAGTACCAATCTCGCAACAGTATTTGCGCAAAAGGATCGGTCCTTCACTTGCTGATCGTTCGCGAGTCGAACT
CGCTCTCTTGCCACGCCTTCCACTCACTCGCTTGAGTCGGACTCAATCACTCTTTTAGTTTGGAGGATCATTCG
TTATTCACTCTCTTGCTATTACCCGCAGGGAGCGCAGCAACCAAGATGCATATTATCATATAGAAAGAGGCG
AAGCGTAGCGATTCGTACTACCGGAAAAGTTTCGCTAACCGGCAGGCAATAGAATCAGCCGATAGGCGCAG
GCAAGCGTAGCGAATCACATAGATAAGCCCCTACCTGCCAGCGCGCGGATGGATAGGGCGGGCGAAGCGCG
AAGGGGATGGATGTCTGAGCGGTTGAAAGAGTCGGTCTTGAAAACCGAAGTATTTATAGAAATACCGGGGGT
TCGAATCCCTCTCCATCCGCGAGGTCATAAGTTCTCTCTTGCGTTATCTATAGATAAGAACGAATCGGATCGA
CTCGACTGATATGATAGATGGAATGGGAGTTTGTGTTCTATTTAGTTAGGACCTTGTCTCCCTTTCGTTATCTTC
CGCTCTCCTTGTATAGGTTGGGAAAGGGCCGGGTGGATTACCTTTGGGAGCTAAGTCGAAGATCTCGGTGGTC
TTGTGAGTGGTTGATAAACTGCGATTGCAGTTTTCTTTAGGAAGTCCCATAGCTGTGAGGCTTAACAGATAAA
GAAAACGGTGGATACTTTTCCGGGTAGAAGGTGACAACCCTAATGAATTGACTATGAAGGTGGCTCTTAGCT
ACATCAGCGCTCAAATTCCTTAATCGTTATTGCCCAGGCTTCGATGATCAACCCCTGGCACATTTAGGTTTTGA

TCTTCGGCCATCCTGGTCCTCAGGACCCAACACTATATTATTCAACTATATTTCCTTTAAAATGAAAAGATGCA
AAAGGTGTTGATACGTCCTATCCACATAGAAATCTTACCCTCTTCCACACATTACCGGTGGATGCTACAGCCG
CTATCACTTTGGCTGCAGGATGGGAATGAAGGTCGAGGAGGCAGGGAAGAAAAGGTTATTCGCTATTGGCTT
ACCCTTATATCAGGC
>TCONS_00058676
ACACTTGGTGTAAGCCCATCTACTCCAACTTCCATGAGCAGGTTCTCATTATACCAGTGACTGTAGGCTGCAG
CGAATTGGCAACTGAACCAGTCAAGATGAAGCTCTCCAGGCCAACTTGAACACTACCGTCATCTGAATGATA
CAAAGCAAGGAATTTGTCTTCTCTATTTTGACCAAAATAGTTGTCCCCTTAATATTACTGTACAAGCAGGCAA
>TCONS_00058682
TGCTTTTCTGTTTCGGGGTAGCTCTGTCGTTTTACCTTTCATTTTTCCTGTAATGACATGTCTTATTTCTTATAGA
ATGCCATGGCATGTTCTTTTTCTTTCTTTCTTAAAATCCATCAACATTCAAACACCATCATATAAAATGAAATG
AAGGAGAAGTTTTTTCCGAACTGATGCATGATTATCTTGACCTTATCTTTTTGGTACATATTTCTCACTCCCGGT
TGCACTTGTTTGTTATTTCAGACTTTAGGCTCTTAAAATACCTTGAAACGCCTCCAATTAGGCGTAGCTCGAGT
GACAAAGCTCAGACCTCAAGGACTTCATGGTCCTGGGTTTGATTCTTCCTGGAGGGGGGTTCTTTAAGAAATT
TAAAACTTACTCTTCCTCTTATCTACTAATCTCTTCAACTGGAGTTTGGTTCTGATTCCTTTTATTTCCAACTCCT
GTTGCATTTCTTCATAACAGTGGGTAATTGTGTCTCCTGGAAAGGAGAAAAAAGGGTGCTCAGGCCCAACCA
TCCTTATTTGTAGCTGTGGCTGCGCTTCATTGCGTCTTCTGTAAGCCTGCATCCATGCCAGTACTTTGCTAATGT
CCCATTGTTGTCCCAACATGAAAAAGACCCTTGTCTTTTGCAGGTAAATGCTGAGCTGCTTGCCTGCTTGTACA
GTAATATTAAGGGGACAACTATTTTGGTCAAAATAGAGAAGACAAATTCCTTGCTTTGTATCATTCAGATGAC
GGTAGTGTTCAAGTTGGCCTGGAGAGCTTCATCTTGACTGGTTCAGTTGCCAATTCGCTGCAGCCTACAGTCA
CTGGTATAATGAGAACCTGCTCATGGAAGTTGGAGTAGATGGGCTTACACCAAGTGTAGTGGCTGGATTTAA
GAATTCTATTCTTATTCCTCT
>TCONS_00058700
TGACTCTCAATACTACGAGGATAGGAGGCTCGGGTATGTTTCCCGAGCTGACATGACTCACACTCTAATGTGG
ATAAACTAGACAAACTAGGCACCATCTTCTGAAGCTTGGATAAGCTTGGATGTCCTAAACGTCTGTGAATTAG
GTCCGGAGGATCTGTAACTAGACATGCCTTGGAGGAGTTTAGTGAGTTAAGGTAGTAAAGGCCTTCTGATTCA
AGTCCTGTTCCAATCGTCTGCCCCGTACTGCGGTCCTGCATAATAAAGGAATCATCAATAAAATATATACCAC
AATGGAGGGCACGAGTCAAACGACTAACAGATGCAAGATTAAAAGGACAGCCAGGGACATAGAGAACGGA
ATCTAGAGTGACAGAGGGTAGGGGATTCGCTTGTCC
>TCONS_00058733
GGATGAGACCACAAACAGTAACAAGAGCCATGACCAATGAGTGATCAACATGAGGTTCCACAACCAATGTT
AATACATCACTTCCAAATAGTACTCCTGAAGTTGCTTGTTTTTGTTTTACCTCTGCAACAAGTCTGCCTGCTTGG
GTCACAATCTTAATGATGTTGTGCCAAGGATAATGTTATAGCAATTTGCTTCAGCTTGATTACAT
>TCONS_00058945
GATACAAAGGATCTACATTCAATTAGCTGACTCTTTCACCTTCTTAACAATAGCTAGAACTGCTCCAACCACA
GCTACTACAGCCCCTGAAATCAAAAGGGTTGACTTGAGATCCTGTGCTCCTCCTTTCTTCTCCTCCTTGACTTC
CTTCGGCGTTTCAACCTCTTTGGTCGCTGTGTCAACACCGGCGGCCGCTAAAGC
>TCONS_00059039
AAAAAAATCTAGAAATGCTGATGCTCATCCTGGACATGTCGACAACACAATTTGGTATCACCACTTTCACGAT
TTTAGAACATTTTGCCGATGCCGTCTTGTCTTGGGATAGATTCATTGATATCCATCTTACGCCATTTTCCCATTA
ATGAGGCCATTTCATCAGCTTTGTCATATAATCCCAGTAGGCCACCTGTATGTATGAAGAGAATCTTTCTTCCC
TCCCACTTTGTTGGATTCTCGTTCATGTCTTTTATCATTCCATAAGCTGCTTTACCACTGTAGACAGGGTCGAGA

ATAACACCTGTGGCTTCAGCAACTTGCTTCACAAATTTAAGCTCGTCAGCTGTGCTCATAGCATACCCAAGGC
CTTTCGCATTTTGAATGCTAACAATATCACGTGAGCTAACGCCTGCATTATTTCCATCAAGTAGGCCTTGAAC
ATATTCATAAAAATAATCTGGATCGTCACAAACACAAAATGCATTAACTTTTGCTTTTAAGCCACTGAGCCTG
GATGCAATTGACAAACCAGCGACCGTACCCCCACTGCCACAAGCTACAACAATGTCATCGAATTTCCATTCA
CTGCTCGAGTGCTGAAGTTGTTGCTCAACTTCCCGGATTGCTTCAATATAGCCCCAGGTTCCTAAAGAATTGG
ATCCACCAACAGGGATGACATACGGCTTTCTTCCTTCATTTAACAGCTTTTCCTTCAATAATTTGGTAAGAGCC
TCACTGCCAACTTTTGCATATTCCTCTTTTGAGACAAGATCGATGTGCGCTCCAACTAATCGCTCAACAAGGA
GATTCCCTATCAATCCAGGATCTTTATCCACAAGTAACTTTGAAGTGCGTAAGATGAGATAACAGTCAAGGTT
TAAGTACTTGGCAGCGACAGCAGTAGCACGACAGTGATTACTTTGTATGCCACCTATAGTCACTATGCAGTCA
GCCCCCTGTGCCACAGCATCTCCCAACAAGAACTCCAGCTTCCTGACCTTGTTTCCACTCAACTGCATTCCTG
ATATATCATCACGCTTTAACCAAACCTCAGTGTCCTTGGGCAAATTAGGCAGGTTCCACTTGTGAATTGGCGT
CGGAAAATGTCCAAGGGAAAAAGTGTGAGAAGGAAGGGGACTAAGATGAGAGGCCCATTGAGGAGGCTCG
TAAGACTTCTTTGTGAGAAATTGAAAGGGCGATGGCGAAACCTGGAAACTGGAATCCTCCATAGATGATTTG
GTAAGACAATAATTTCTGTTCAAATTTAACGCCTTATTATTGAATTGTTGCAAAGAAAATGGTGAACTGTAGC
ATACTCTACTGAAGCTACTCCATTGGCAACTCAACATTTC
>TCONS_00059151
CAAAGTTGGCTTCGGTCATTATTTATTGAACAGAAGCAAAAGCCTCAGTAACGGTCGGACGATAGTAAAAAT
TCATAGCAAACCCAGTAGCTACTTGTACTAAAAAACAAGTAAGCGTAATTCCCCCTAAACAATAAAATATAT
TGACGTGGGGAGGAACGTATTTACTAGTTATATCATCCGCAATTGCTTGAATCTCGAGACGTTCTTCGAACCA
ATCATATACTTTACTCATACTTCGGAAATACAGAAACAAAGAAATTCCACGATCGAACTACCAAAAGAAAAT
AGAATGGACTAGAAATCGGAGCTCTAAATGATTCATTTTGGACTCAAAAGCAAGAACTTTGCGGTTCTTATAG
ATCTAATTCATTGAAATTCCATCCAATAAAACGGAAGAATTATAAATCTCCAAAATAATAGATAGAAATACC
GCAAATAAGGCCATTGCGACACCCATCAAAGGAGTAGTTCCCCACCCAGGAGCTACTTTGCCATATTCCGAA
TTCAATGGTTTTAATAAATCTCCTACCGCAGTTCGTCTTGGACCAGATCTAGAACTGTTTTCAACAGTTTGTGT
AGCCATAAATCCTATTGTATTCATTGAGATCTGTTGACTTTGTATACCATTCCGTTGTAAATAAACGATCCTAT
CATAGATA
>TCONS_00059169
GCTCTTTCTGTGGAAGTTGCCTTTGGTTTGATATCGGCATAAGAGGGTAAAAGATGGTTTCACGTATATTCTGA
AATAGCTAAGTCTTGTTTGACACAGAAGAAAGTTGGTACCTGGTCAGTATAACACAAAGTTTGCAAGGATTTT
CTGATGCAGTTCAGTGGCTCACATCAACTTACATTTTCTCAGCAGTTTCTTGGTTACTCAAGAACAGATATCTT
TTTGGCATTTTAAACACTTTATGCAACACATCTGTCTTTTTCTGGTTGATTCAACTCT
>TCONS_00059184
GGTAATCATCGCATTATGCTCATTTTAATTGGGGGAAAAAATGAAACACAGAGAAATTACATTCAGTACATA
GTGCTCTGTATGAATAGAGAGCATTGACGAACAAGCTTAGACAGAATTTGATTGTCATGTTAACTGTTATCTA
GTCAAATACTGAATATCATGGTACAAATATATGTTCCTTTAAGAGCAGCAGCAGAATCTAGAGCATAGACAA
AGATACCTTAGAGCTACTGCGCTCTGGCAACTGGCTCCAATGCTTCAATAGTCACCACGCTATTACCGCGAAT
TACCACCATGCCAATTTCATTCTTCTCATTGCCATTAACTTCAACGGTGTTGTCAATGACCAAATTCATGAACT
GATCAAACCCACGAAGAGTGCCGACAACTAAGCGGTTGGCATTCAGCTTGATTTGAAGTTGCTTGTCCATATA
CTTTTTGAGATCCGGAGGTTGACCCGACCTGCTCATCCTTTCAACCCTTGTCTTCTACTCTTCGCTCACTGCTTG
AGGAGATGGAGAATGCAAGAATTTTGGAGGTTTTTCAGAGTCCCGGTAGAATTGTTTTTCAACTCTCAATCCC
>TCONS_00059442

TTCTCGTACTGCACCTATTTCAAAACAACAGCTGCATTTCTGAGAGCCAAATACCTCTGAACATAGTCATTAG
GAAGCGATCTTATTATCTTAAAAACCTCGGTGTTAGCATAACGGAGTTTAGTTCTTGGGTCAAAATATGGTGC
CTCGAATCCTGTTACGTCGCATATCTTTTTGCATGGATGCATAGAAGGTGGTGATTCAATGTTAACATAAGTA
GGTAGGTGAGGAGGATAATTATCGTAATTTTCAGCTTGAATGATCTGCTTGAGATGCTTCCAATGACGGCCTC
TGGCCTGTCCTTTAGGGTACTTGTCGTACATCTGAATCTTCTTGAATTTCATGTGAGTTGGGAGTACTAGCTCTG
CTTCTATCACTTCCCGCTCCATCTATAGC
>TCONS_00059443
TCTCGTACTGCACCTATTTCAAAACAACAGCTGCATTTCTGAGAGCCAAATACCTCTGAACATAGTCATTAGG
AAGCGATCTTATTATCTTAAAAACCTCGGTGTTAGCATAACGGAGTTTAGTTCTTGGGTCAAAATATGGTGCA
AGTAGGTAGGTGAGGAGGATAATTATCGTAATTTTCAGCTTGAATGATCTGCTTGAGATGCTTCCAATGACGG
CCTCTGGCCTGTCCTTTAGGGTACTTGTCGTACATCTGAATCTTCTTGAATTTCATGTGAGTTGGGAGTACTAG
CTCTGCTTCTATCACTTCCCGCTCCATCTATAGC
>TCONS_00059497
CGGTCAAAGAAAATCCTCCTGAACTGGAAGTCTGAGAATTCCAGCCATCTTCACTACACAGACAATCCCTGC
CGCACACGTCCGAAGATCCTGCTGTTGAACTGCAGGAACAAAATATGCCATCATCTTCCAGCCGCCGCTTAA
CCTTCTTGGTGAGGTATATATTACGCTTTATATAGGTAAAGGGTGTGGGTTGCCATTTATTCAACCAATCTGGA
AGTTCAAAGTCAACAGGATCTCCAATTTCGCTTGTCAATTTGTTGAATACATGCCCAATCCCACCATGGACAG
CAC
>TCONS_00059609
TGAAGTCAACGTCAATACCTGGGTGAGTGTGAGAACCTGAGCTTTGACTATCATAGATACTACTAGCGAAAC
CACCAAAGCCTCGCCGGCGCAGGATAGCAAGTTCTGACTAATTACAGCGACGTTGACTGAACCACCGTTAGT
CTCCATCGGTAAGGTTCGCCACAAATACACCAACCACCACCAGAGAAATAGATCTGAGCTTGGATAAAGCA
GATCGGAGAGTTTTAGAAGCTAAAAGATGAAAAAAATCAGATTAGAAGCAGCGTTGATTTCTGACCGTTCGA
TCAAGCAGAGAATCAATAGAGACCAAGAGATTGTGATAGAAAGAGAGAGGACTCTTCGTTCTGGAGCGTTT
GGGTGAAGGGGAGGGGGTTACTGAGGAAGCTGCCCGTAATCTCTCTACAAGGGTTCGGGCAGTTAGGGCAGT
GCTTTTCTCTGAAAATATCAAGGCGGTTGAGC
>TCONS_00059612
TAACCAAACAAGATATCTCCAGGTAAGATTTGCCATATCACTTGCTTTCAGCATCATATAGATCTGTAACTAA
ATAAGTGCCATGGGCAAATGCACCCGCCAAAACACCATAGAACAAACGATCCCTAAGAACTGGCTTGTGCC
TCAGAGCTATCCTAATCAGGGGAAGGACAGGTGCATAAACAAGTGGAACCAAATACTCAAACGCGGGTCCT
TTTCTCCTCCTTATAACTTGCGGCTCAGCAGAATCAGACGGCGAAGCCATGGAGGGCAGAAATTTTGAGAGA
TACAAAA
>TCONS_00059629
AACTCAGCTTCCCAAAGAGGAAGTTCAGCAGAAGTTGTGATTATGAGAAACGGCAAACCAATATCCTTCGGC
AAGGATAATAGAAATAAGACCACCTTCAAAATCCGCTCCTGGTCATCAATGACGACAGCACTCTGACCCTTC
TGCCAATATTTACGAAGAATGTTGATATAAGTAAGATGATTATGGACCTGAGGCGCGCTTCCAAATAGAGGT
CTCGGCAGTTCTT
>TCONS_00059639
ATTTTTTTCAATATTCCTGCAGAAAAGAAAAGCCGACTTTCAAGAACTGCCGAGACCTCTATTTGGAAGCGCG
CCTCAGGTCCATAATCATCTTACTTATATCAACATTCTTCGTAAATATTGGCAGAAGGGTCAGAGTGCTGTCGT
CATTGATGACCAGGAGCGGATTTTGAAGGTGGTCTTATTTCTATTATCCTTGCCGAAGGATATTGGTTTGCCGT

TTCTCATAATCACAACTTCTGCTGAACTTCCTCTTTGGGAAGCTGAGTTCTTACATTGGGCTTGCTAACATTGTT
GTGTACAAAGGTAATAAAGATATACGAGCCATTATAAGAACACTCGAGTTTCACAACAAACAAGGTGC
>TCONS_00059641
AATTTCCTCAACTCATCTTCAGGTCCTTTACCAGTTAGCCACCTTTCAAAAACACCATTTTGCATAAGATAGCC
AATTCTCCTCATAGTAATTTCCCTTTGTTGTTCTTTAGATTGGTTATAATCAGGTTGAACAAATACTTTACCACC
TCTTAATTTTGGGTTAAAAAGATTACTTTGCAACATTAGACCAAATAGCCAGTCTCTTTCTTGCAAATTATGAC
CATCCATAAGTTTTCTCATTTCTTTTGTGTCAAAAACAGAAGATGGTTCTGAAAATTCAGGTGGGTTGTAGCTC
AGACATGGGAAAGATTGGAGTATTGAATTAATGGGTGTTGGGTTTTCTTGGTTTAAGTGTCTG
>TCONS_00059680
TATTAGCACATCAGAAAGAGCAGTAGGTCCAGGAGGAATATGGATGATGCGACTGCTATCGTTGTTGTTTACT
GCAGCAACTAATGCCTCAAGCTTCTCAGTCTTCCCCTCATCTTCTTCACCAAAACTAATAACATCAAGAGCTA
CACTATTCTTCTTCAACTTTCTTCCAATCATCTCCAAGACCTTCTTGTCCTGCCAGCAAAAACAATGATCCTCT
GCTGTTGCTTTTTGTTTTGCCGATGCTTCAGAGCCAACTGTGCCACCTGGATTCCAGCAGCCAAATTCAATTCA
CCACCGATATCTAATCCTGGGTTTTGGAACCACAAAATAAACTAACTGCGTCAGTTTGAGCTTCAAATCGATT
GCGCGGATAATCTCCATTGCGCATCC
>TCONS_00059740
CTCAAGATCAAATGTTCAACTACTCCCAACTCTAAAAACTTTCTTGCAAGCTTAATAGAACGAAAATATCATG
AGTGCAAACTTTGAATACCCACCACTTTGAACTTGAAAATGTCTTAGCATCTTCTTGGGATAGCAAAGGTCTT
CATCTCGCACGGGAAGGGATCCTCAGAATATATGCACAATGTCATATGCGAATGTCCAGAAGAGCACGAATG
ACTG
>TCONS_00059748
TTAATTTTCCCCCCTTGGTTGCTTTGGTTCCTCAATGCCGTCCTGGGATTTGAGATCTGTTTTCTTTTTCAAGAAT
TTCGGAGGCTTTTCGCAGTCTAAGAACGGATCAAGTCAAAAGCCAGAAGCTTCTGCTGGATTTTCTCAACTAT
TTCTGCCGCTGATAGCACCCAAGAAACACCAGGTATATATTTCTTACATTTGACGTACTAAGCTTAAAGGAGC
TATGTTAAGGGGTTGGATGGAAATGAGTAGCTGGCACGAGTTATTCCATCCGTAACATATTGGATACAAGAC
TATAAGATGAAGGAAGGATATGGGATGAGTTGGTCCTGATTTTTGTGAACACTTGGGCAAGATATTTGCAAA
CGCAGAAGACTAGACAATGCTGTGACCTATCATGAGTCATGTACAGCTACTTGTAATCTGACAAAAAGAACT
TTGGCTAATTTTTCCAAGATGCACTTGGAACTATACAAGTACCATAGTTTTATCGGAAGCATGTTATGTTCAAA
CTTCCATCTGTCTTTTAGGCCACAACTCATTTTAGATTGATCATGAG
>TCONS_00059755
TGACACTGACTTAACGTTTCTGATCTTTGCAGTCATTCGTGCTCTTCTGGACATTCGCATATGACATTGTGCAT
ATATTCTGAGGATCCCTTCCCGTGCGAGATGAAGACCTTTGCTATCCCAAGAAGATGCTAAGACATTTTCAAG
TTCAAAGTGGTGGGTATTCAAAGTTTGCACTCATGATATTTTCGTTCTATTAAGCTTGCAAGAAAGTTTTTAGA
GTTGGGAGTAGTTGAACATTTGATCTTGAGAAAACTTTTTGGTTAGGTAATTTATATTCAGAGTTTAAAAGCTC
>TCONS_00059801
ATAAGATATTTTCAGTAACTTGTTTTTCTCTACTTTGTAGCTGCAACATTCATTCATGGATGATATGGCCTAGAT
TGATATGAGTAGACCCTTACAGAACATACAAATAAGAGTAGAAAATAAACTGGAAGTAAAAAAGAGAAAA
AAGGATCCTTTAAATGCCTCTGAAAAATGCAACCTTTGAGAAGAGTTTGATGTGGTTCTTTAGTTTCTGTCAAC
TCTTTTGTGCTAACATCTGAGAACTTATAGTGCGAAATGATAATAAGCTTTAGCATCATATTTGTTTCATTATA
ATTTTATTTACTTATATTTATATATTCTTTGTAGAGACGATGCGAGATAAATAAGGTCAATCGAACTAAGCTGA
AATCTAATCATTTCATGGGGTCAAGAGCTTTTGTAGCTGCTCGTGCTGAAATACGAAGATTGAGCTCCACCAG
ATTATCAACTTTGGGTATATGAAGCTAACTATTGGATCAAGACTAATTGCAAGTGAAGTGTCACTGCATTCCT

CCACTTCTTAGTCTATATTTCTCAGTTAGACTTTTATGTTGGACATATTCACTTTAATTATGTTTCTTTTAATTTA
AGGAGCTTTTATTTCAATATAGTTTTAATATTAGTTTTATGATCAATGGTGAATATTTTTGTTACCTTTGATTGTT
TTTGTTTTTCTGTTATTAC
>TCONS_00059848
TAGCTCTCCTGCATCTGAGGCTAATTTTGGATCTTATTCCGGTTTGCATGCAGAAAGACCAGCTTTTGACAATA
AATCCAATGAATACTTGCACTGGGTTCCAAAACTCTTTGCGACCTCATCACTTTAATCTCCAGAAGAGACTTT
GAGTCTCCTAAATCTTTCATATCAAACTGGGTTCTTAATCTTCTCTTAAGCTCATTTATTGAAGCTAAGTCATTA
CTAGAGATAGCAATGTCATCCACATAAAGCAGAAGAAGGACA
>TCONS_00059908
CACGGCTTTTTGCTTCTTCCTGAACTTTATCAATCACCTCGTCAGGAACTCTAAGATACTTTATGGTATTGCCA
CGAATATAACATTCTGGCATTCTCCAAAATCTATCTCCATCCTTTGATGTACAGATGACTTCACGAAGATGGA
TGTTCATCCAAGTATCACAATTCACCAAATGTCCGTTATAAGTCTCTCCATTTTTTAGCTCCACCAACATGGGA
TGCCCCTGTGCAGTCTTGAGCAACGAGAGCGGAAGCATCTTTAGATGCGTGAAAATCCGAATTCAAAATTGC
CTTGTTAATCTTCGTCTTTCTCTTCAACTTTCCTGGAAATAACGAAAACGTAGGGTT
>TCONS_00059941
AACAACTCCAGAATGTAAAAGGAAGTAGGTTACTCATATAAATAATCTGATCAATCCAAAAAAATGTAAAG
TAAAAAGGAACTACCAGTGAATGAGTCATCATTTGCTGATTCAAATCTTACCTGTGCCACAATACTTAGACGC
TGATCAGCAAATCTTGTCATACCCTGGGAAGATTCTGAAGGCAATTCATTGGAAAATAAATGGAAAAAGCAG
AGAGAGGAATGTGAATCTGAGTGTAATATA
>TCONS_00060152
CTCCGCCTTCCTTCTTCGCCGGAACAATTTCAACTTCCTTTTTCAGGTGCTTCTTCAGCGTCTCGGCCAAATCCT
TCACGTCCATTGATCCTGTCACAGTCACCAAATCCTTCTGTTTGTCTATTTTCATCTCCTTGTACCCTTTGAATTT
AGTGACAATTTTGTAGATCTTCTGAATACATCCTTGACAATGGAGATGCACCTTCAGCACAGCCGTGGTAATC
GGAGGCTCTTTCTCTTTGCTTTTCTTGTTGTCTTCTCCTCCTCCTTTGTTTTCCTTTGGATCCTTGTTTTCCTTGTTC
TGCTTCTTCTTTTCTTCGGCTGCGCCGCCGCCGCCGCCATCTTTTCCCTTGCCGTCTTTCTTTGGTACCGGCGAA
ACCAGTTCAACATGCTTGTGTGTTTTCTGCTCCACTTTTTCTCTCAGCATCACCGGATCAACTTTTCCGATTACC
GTTAACTTGTTCGAATCAGCATCGCACTGAACCTTCTCCACACCTTCGAAACTTCGAATTGCTTTGACAACTTT
GGAGACACAGCC
>TCONS_00060344
AATATTCAGTTAAAATAAGTCGTATCTTGCTCAGACCAATAAATAGAGCTGAGGTTCTAGTTAAAGCCGCTAA
TAGAAAAGCGAAATAACTAGTTATAGTAAGCATGAAAGGGCTAAATTAAATGAAAGATGTTCTTTTTATACA
TATGTTTAGAAAGCACTTACCTAAGTTTCCATTTTTGCAAAATAGGAGGATAGGCAAAAATCTCAATTTCAAT
TGAAAGAAGAAGTTGAATGAAAAGTTTTTGATTCATCTATCATTATAGGCAGACAGCACTAACAAAGATAAA
GTGACAGACATATAAAAGAAATTGATTCATCATC
>TCONS_00060369
GTTAAATTGTCCGCCACAAACTTCCTTCAAGAGACTACTAAAAGATGTAGTGTCAAACTGTCCATTATTATCT
GGTGGCATACCATTAAATGGTTCATCACAAGCAGCCTTCAAAAGACTAGTAAAAGAAGTATGTTCAAATTGT
TCATTGTCAAGTGGTATATCATTAAATGGTTCACCACAACAAGGCGTCTCCTTATATGGTAGGCTACTAGAAG
CTAATGATGGATAGTGGAGGATATCTCCAGGCTTGGATACCTCCTGTTTTCCAACATTACTTAGAGCTTCATA
ATCAACACCAGCTTGTGACTTTGTGCAATCTTTATCCATAATAAGATGTTCAAGACCAGTGAGCTTCTTGAAC
CTCTTAACATCGTCTGGGTCAATATAACTGGTATCAGGGTCATTGATGAATACAATGTTTGCAATAACATTAT
ACACATGACAACATAAATGGGGATGCAGGCTGGGAGGAAATAGATCACAAAACCGAGGATAAGCTGCAGC

TTGGCCGTGGTATAGTGTAGGAAGCTTTCCGTGAAAGAACTGGTGCAACCATGGTTGTGCCAGGACAACAGA
CCGATCTGCCTTTCTTAATGCATGATAGTAAACCCTTTCTACTTCTATGGTTCTTAGGCATGAACCAGCTAGTT
CATTGGAAGTGTACGGAGGTAAGCCCAGATATTTTCTTTGTAGAACAGATTTTTTGCGATATTCTCCCTGGAG
ATCTGGTCCAAAGTAGGCTCCAGTGATGAAGTAAAGGAGGAAAAACTGATCATCCGTGGGAACCAATCCAC
AGAAGTTCTTGTCCATGCTTGATCAAAACAGTAAGAACTATGTGAATCAATGAGTTCTCCAGTTGAGCTGCTA
TTT
>TCONS_00060440
TCATCGCTAAGCGGAAGTTTGGAGCCATACCTATTAAAATTATGTTTCTGTCTTCTTTTCTACAACTGTTGTCA
ATGCATCTTCGATAGCTTGGGCAAAAGTTGCAATCTTGTAAGTGAAGAATCCAACTAACATGGGTATCAAGT
CAAGGTGCATGAACCCGTACTCCGCCTCAAGAATACCATTCCACCGGTTATAGATCATAACCAAAACAACAG
GAACCAATAGCCTCGGTTGCCCAGCTGCTCCCCTGCAAGTAAAGCTTTCTCGTCAGTTAATTAATCAGAGCTC
AA
>TCONS_00060449
CTTAAAGTGCTGAACCATCTACTGTATACATGGTCTGATAAAGTATACCTAGCTAACCCAAAATTATGAACAT
ATTTCCAAGTATAACATGAAAAAAGTTACATTACTTGATCCTATTCTCTTCAAATGAAGCAAATATGTAAGAA
AGATCAAGCATCAAACAATCATCTACCTATTAGTACACTGATTGTGACATTGTTTGAGGATGGCAAATCCTGA
TTCCCACGCCACATATCTTCATCAACAGATCTTCTTATTGAGGTATAAGTGGGTTGCCTAGGCAAGGGCAATC
TCATATTGTCAGTTTCCAACATCAAAACTACAGAAGACATGTTTGGCCTGTGAACAGCCAAGTCCTGAACAC
AAAGCATTGCCAAGTGTATACATCTCAACGCTTCATCATGCTGACATCCATCCCATATTGAACGATCTACTAG
GTCCATTGGTCTACCTTCGTCCCACTTCTCCCATGCATATCCAATAATGCCTGAATGCTCATCCGACCGAAAGC
TTGTATTTCTACGCCCACATATAATCTCAAGCAATAGTATGCCGAAGCTGTAGACATCAGACTTGCCAGAGAA
CAGACCTTCCATTGCATACTCAGGAGCCATATATCCATATGTGCCTACAACTCTGTTTGTATTTGCTTCATTTTG
GTTTCCTCCAAATATTCTGGCCATGCCAAAGTCTGAAATTTTTGGGTTCATCTCTTCATCCAACAAAATGTTAC
TAGCCTTTAGATCCCTATGGATTATCCTAAGTCTTGAATCTCTATGGAGATATAGTAGCCCTCGGGCAATCCCT
TCGATAATGCTGAAGCGTTTTCTCCAGTCTAACTGGGATTTCCTAGCAGGGTCAAATAAAAATGTATCTAAGC
TTTTGTTGGGCATGTACTCATAAAGCAGAATTTTTTCTTCTCCTTCTATGCAGCAACCCAAAAGCCTAACAAGA
TTTCTGTGTTGTAATTTTGCTATTAGAGTGATCTCATTCTTGAACTCCTCATCCCCTTGTCCAGACTTTTGTGAA
AGCCTCTTTACAGCAACTTCTTGACCACAAGGTAACCTTCCCTTGTAAACGGGGCCAAAACCCCCTTGCCCAA
GCTTGTTTGCAAGACAAAAGTCGTCAGTAGCTGTGGCCACCATGCTGAAGCTGAAAAATGCTAATTCTGAAC
CATTTCCTTGATGCCCTTCAGCATTGAGGTCGCCCGGTCCATATAAGTCAGCGGAGAACTCTCCACTCCTAAC
AAGATGGTTTTTGGGAATTTCATTCATCCTCTTGGATTCTGAGAATTGGAAGTCAAAGTTATAAGTA
>TCONS_00060458
ATTCACCTTCACACACTTGGCCCCTTTAAACTCCTCCATTTCACTCAACTTATTAGCAGCAACTGAAGCTCCAA
TAAAATTGGGTATCCGATGATGAACGGGCCTTGGGAATTGGGCTATATTTTGGCCTTCCATTAAGTCCCAAAT
CCGTTTTCGGATAACCCATTTCCAAGCTTTCGGGTCTTCTTGGTCTTGGGTCTCTCTTTCTGAGGTTTCAGCCAT
GGCTTTACGCGCCTCGGCGTCGAGTCTGTGACGCTCCGCCTCGCATGCTGCGTCGTCAAAAACTGCGTCGTTT
CTCTTTTGGGAATCC
>TCONS_00060613
GGCTATATAAGATGTGAATCAAGGAGATTCACGCGTTGATTCACCCTGTATTAAGTCAAAGGAGAACTGGTTT
GGTAGTGCATTCACTGAGAACTACATTATTCTACAAATGCAAGAATCTGATTCATCGTCATCTGAGTCACTGA
CATCACTTGGCTGAAATTCCAAAACCTCAGCAAGTTTATTTCCATTTGTATCATTCCACTGCACTTTCCTCTTTG
GTTTGCCTTTATCTTTTGACAACCCACCTTTTTTACAAGGCAACAACAATGCTTTAGTCTCATCATCCTCATCTT

TAGAAGCTTCCACACTATCTACATACTTATTTTTCTCCCCTTTAAATTCTTTATTTTTTGTATCCATTTGCCAAAA
TACACAATAAAAGAAAGC
>TCONS_00060701
GGAAGAAGGATGATACACAGGAAATGGAGTTTGCTGACAGGCCCCGCTGCTATTCTCGGCGGTATCGTCGGT
ACTATCGTCGTCGCCAATTTTATCTTCGTCCAAAATGATCCGTTCCTTAAGCCCGATCGGAAGCAGGAGAAGG
CACCTTCAAACAAGTGAGAAATGCGTGTGATTTTCTTGGGTTTGGGGTTTTCTGTTGGTTTGATTTGTCA
>TCONS_00060707
AAAACCTACATTCAAGCTGATGCTCAAAATTTGAGTTTATTGGGCCGTAATGTAGTGACAAATCAAACCAAC
AGAAAACCCCAAACCCAAGAAAATCACACGCATTTCTCACTTGTTTGAAGGTGCCTTCTCCTGCTTCCGATCG
GGCTTAAGGAACGGATCATTTTGGACGAAGATAAAATTGGCGACGACGATAGTACCGACGATACCGCCGAG
AATAGCAGCGGGGCCTGTCAGCAAACTCCATTTCCT
>TCONS_00060854
AGGAGATGTTTGTGGCCACAAGAGCAAGAAAACCTGGGCGTGTGTACAAGGACTCAGGCGAAAATACAACT
AGTAAAATTGCTGAAATGGAAAAAATTGAAACACAACAAAGTGTAGATGGCAGTCAGTCTGTTGATGCATTT
TCATCTGTCATGGGTCCTGAACATCCAGGATGTTTAAGATTATATGGACGGGGGGTTACAAAGACTACGTTGA
AAGGAAAAGTGGGCCA
>TCONS_00060862
TTCTTTGCTGAAATTCGAGGTCAGTTTTCTTGGATTTTCAGCAGTGGAAAGCAGAATCCATTTGCTCCAACAGT
GGTAGGGATATACAATTTTCTTTCGGAAGAGAATTACAAGGTAATTTTGCTTAATTAGTTGATTCTTCTATTGC
CATTTTCCATAGAATTAAGTAGACATAGTATTTGGACTTTGGAGCAATAATTCTATTCAACTGGCCATATTATT
TTCTTTGTTTCTTCATCGGCTTGTACTTTTAGCTGCCCATTGTGTACATCTTACAGTGGACTTTTGGTGCAGGGG
GA
>TCONS_00060881
AATTTACACAGCTCTGTAGCCTAAGTTGCCAATTGAAATGCTAGAAAATCAATATAACTGTAACACCTTTCAT
TATATGATCTTACTTTCAAAACATCTTTCACAAATTTTTAAGTCAAAAAACAATATGGTTGGTACTTCCAACT
TCATCAGGTATTTAAGCTCCAAAATTACATAACATAAACAGCAAAGTCAGATTCACACTCTCCAATAGCAAA
TTGGAGTTGGCTTTTACCTCTTCCCCGCCTCTCAAACTGAATGGATCATTGATGACATCATGAGGAGCTTAATT
TTTGTAGCTTAGATTTGAGCATGTCCAATTGTTTTTGTTCTGATACTGTTGCTTTCTGCACAGTTGTACGGAATA
GTATGTTGCCATTTCTCTGGTAGACCGTCCGAGAAGCAGGAAGGTTATTGAGCTCCGCTACAGCAACCTCGTG
ACTTCTTACCTCCTTGAGGAGATGGCGCTGCGAGTCTGAATTGGTGGGTGCTGCAGACATGTCTTGCTCCTTCT
T
>TCONS_00060919
ACGAACCTTGTAGGTACATAAAATATGCAAGGCGTAGAAAGAGAAAAAAAAATTAAGGAGCTATTCCATGC
TGGGACACAAATAGTGAGTGAAAAATGGAGACTGCTCAAATCACAGTAACAGCATCTTGTGCTGCCATAATA
GTTACTGTAGTAGTGTGTGTATGGAGAGTGCTGAATTGGGTTTGGTTCAGACCAAAGAAGCTGGAAAAGCTA
CTGAGGAAACAAGGTCTCAAAGGCAATTCCTACAGGATTTTGTATGGGGATATGAAGGAGCTTTCTGGTATG
ATTAAGGAAGCTAACTCCAAACCCATGAATCTTTCTGATGATATAGCCCCAAGATTGGTCCCTTTCTTTCTTGA
CACCATCAAGAAATATGGTAATAAATCATATAGCATTTTTGTCTCTGC
>TCONS_00061102
TCATAAGCAATTCCTCTTCCAACAGCATAGCCAAAACCAAGACCAATTCCACACCCAGCACCGAAACCAAA
ACCCAGTTGCAACCCAGGAAGCCCCGGACCAAAGCCAGTGCCTCCAATAAGGCCGATTCCGAGACCAAAAC

CACAGCCTATGCCAAGTCCAAAAGCAGGTCCAATCTTCCCCAATTGCTTCGTCTTAACTATCGGTAGCTTCCA
TACCAAACCCTTTTCGTCGTCTCTGCCGTTCATTTCTCTC
>TCONS_00061166
CTTTGAGCAGTGAGACATATTCAAGTATTTAAGCCTTTCAAGTGTTCCAATTGACTCATGAACTACTACCAAG
TATTTACAGTTGCTAAGATTCAACTTTTCAAGATTTGGCATCATGCTGAAATCTGGAGTGGTAGTTAGCCCATT
GGAGTAGCTGAGATTCAAATACTTCAACTGCTCAAGGTTCGATTTCTTTGACATAACATCACATATGTCGTCG
GGAATCCACAACCTGCTGTATTTCTCTTTTGTTTGTCCCTGGCGAACCATGTACCAAGCAGTTTCTTGAACTAA
ATCATGCATCTGAAATCTGTCATCATCAGATATAGATAACAAAGATCTTTGAACAAGCACTGGTATTCCAATT
TCAGGTTTGAAACCGAAAGCATGGAATTTTTTGACCACAGGTTCTCTTCGTTTCCCCTTAAAGAAACAAGCAA
TATCAAGCAATATTTGCTTGTCTAAATCATTCAATGCATCAATGCTCAATTTAAGTTTCCCTATCACATCATCA
TGAGGAATACCTTCCAACCTCGTCAACTCGCTTTCCCATTCTTCTTTTTCTCTTTTGTGCAAAAAACATCCCAA
AACTTTGAGAGCCAACGGGAGTCCCCAAGCACATTTCACCACTCGTAATGCTAGTTCCATGAAATGATCCTGC
GGTTGAGCTTCCTTGAAGGCGAACCTGTTGAAGAGCATCAAAGCCTCATTAGTTCCTAATAGGCTAACTTCAT
ACACTTGGTCCACTCCACAAGCCGATAGCAATTGCTTGTTTCTTGTTGTGATAATAATTCTACTACCAGGCCCG
AACCCTTTGTGTCCTCCAGCAATCAAGTTTTCTATCTGCTCATCTTGATCCACGTCGTCGAGAACGATCATCAC
TTTTTTATTGCTCAATCTGTTCATCAGTAAGTTGGTGCTGGTGTAAAGATTGTCTTTGGTCTCTTTCAGTGTCTCT
GAAAGCAATTTTTCTGCCAAAGCTTGCAGTCCAAATTTGGATGCAGCTTCTCTAACATTATCAAGAAAGCAGC
CACCTTCAAACATTTCTTGAAGTTGATCAAAAACAGCTCGTGCAATGGTTGATTTGCCGATTCCTCCCATACCC
CATATCCCAATTATGCGAACATCAGTCTCAGAATTTGTATTCAGCAGAGATATTAAGTTTTCTACTTGGGGCTC
GATTCCGACTAGATTTTCTGCAATTTTAGGTCGTACATGGCCCAACTTGTTCAAAATT
>TCONS_00061201
GGTGGAGTGGATGGCGTCGTGAATCGAGAGCTAGGAGAGGACAACTCTTCAAATCAATATTGGTACTTAGCC
TTACATGCACAAGTAAAGTAGCAGAGACTTGTACCTGACTGGGAAGAGTCCAGCTTTATATAGATATCTTGCC
CACTAGCACCAAGATCTCTGATGTCGATCAGCTCATCAAACCAAAGCAAGCAGCCTTCATTTGTCCCTGTTAT
GTCTGGATTTGCATATGCGACACACGAACAGTTTCTCAAGCACAATTCCTCGCATGTACTAAGATTCACTCCA
TGATTGTACCAAGAAAAACGAGTGTCTGGCAACTTGATGCCTGGATACTTCAAGAATTTAACTTTCTTTTGAC
AATTCAATGTTGTCTTTCTAACACAGCCACTTAACCAATTTTCCCTTGCCCATTCTGTTGGGTTTTTGGGTTCAA
ACTGATCCAAACAGCTGCAGATTGAAGAGTTGCCATTATTGCACAAACTATATCCTTGACACCGACTGTAAAT
GTCACAGTCATCTGCTGGTGCGCTACCAAAGTAATTATCCCAATTCTGTGTTTGATTATTCCATAGTGAAAGCT
GTAGAACCCCATCTAGTTGCATCACTACCCTTGCAAAGATAGAGCTGTCTGTGAGCTCATACATGATAGATAT
TGTCTCAGGATCGGACACATATATATATTTATAACCAGGACTTGGTAGCCGAGATGGTGCACTAGCAAATCCT
CGACCATTCCATGGTCCAAACCTGTGGCGTTCAATGGAACCGTTCATGATAAATGGCTGTGGGAATCCATGA
GGATCGAATGTCCAAGAAAACTCACCCCTGGAAGGATCATTTGTGCTCTTCCATGACCAGAGGGAACGCCGA
AAACCAGTCTTAAGATCGATTCCAACCCTCATTCCTGGCAAAGATGTGTCACTGGGATAGTCAAAACTCTGCC
ACAAGTAATTCAC
>TCONS_00061202
GTGGATGGCGTCGTGAATCGAGAGCTAGGAGAGGACAACTCTTCAAATCAATATTGGTACTTAGGACCAAAA
CCACCCTCTCCAAGCTTGTTGTTCAGTGAAAAGTTATCGGTAGCATCCAAGATGGTCGCAAAATCAAACAAC
GGTACATCTGGAACTTCATCTTTACTTTCATTGGTGTTGAACATTTCAGAGATCTTTCTTCCTTTTCCT
>TCONS_00061276
ATAGAGTCATCTGCCGACCAAATAAAGATACCATGAAGTTTTCCCCGACTCCTCAGTATGCTGCATGCATCAT
AGAATCCATGTTTAGGAGACAAGCCACCACTGTTGTCTGTTCCAAAGCTCACTAGAATCTTGCCTCCTTTATA

GTTATACCTCTGGGTTTCAAAGTACTGCAGGAATTGAGTAATGGTTGTGCCCTTTTCATAGGCATAAAATTGG
AAGTTGACATAGTCTATTAGATGACCATACTTTCTCCAGAGAGCTAAATAGTGCACCTGCACTGAATCATCTG
CATATGGTGCTATAGATGTGTAAGAGACGACATTGTTTTGTTTAAGATAGTACAACAGTCGCCCGATGCACTC
AGCGAATGTATCTGGATCTGCATTGAAGTGTTCATAATCTATATCTATTGCATCCAGGTTATACTCTTTCACTA
TCTTTGTGACTGAATATATTGCATTTCTTACCCAAGAAGTAATTGAAGTAGGAGCAAAGGTGGCATTTTTACC
ATGCACTGTGTCACCACCGAGACTCAAACCTACCTTAACGTTTTTATGCTGGGCTTTGATGGAAGAAACCTTT
GAAGGGGTTAGATTATCAGTGTCCCAATAAACCAGGAAGTTACCATTAGTGGCTTCTGGTGATTCTGTGTTTG
TGTAGTCTATGGCAAAGGAGAGGAGAAAGTGAAATTCAACATTTGGATTAATTGGGACATCAGAGAATGTGA
CATTCTTGTATAGAGCTCCAATGTATTCTCTGAAAACCCCAGGGCAGCTTGCTGGAACTGCTTCAGTTGTAGC
GGAAATAGTCAAGAGAGATTGAAGGACAAGAATAGTGATGCAGAATTTGAAGGGCTTCATACTTTCTTGGAG
TTTGAAGGATTCCTTGTACTTCTTATTGGTATTTAACCAGAGTAAATGCCGATTAAAATACTTCTCCTTTGACA
AGGCATTTGTTAAATTCTTCTTCAGACCATCATTTAAGGCTTAATTTCTGTTTGTTCTTTTGTGAGTAACATTTCT
TTTGCAGACCTGGTGTGGTAGTTGGTGAACATCAG
>TCONS_00061302
AAGTAGACAACCGGATCAGTGTAGAAGTAGCCAAGAGGATTCAAGAACAACTGGCTGCTATCATGACTGGA
ATGCAACAGGTGATCTACTCTTAATGTTTCGGTTCTTAAACAGTGGTGACAATGAAGGAGGAGCTTGAAGCTA
AGGCACTCAATGTTTTGCTTTTTGGTTATATGTAAATGCGTACAATGACAATGAAGTATTACGTAGTACATATG
CGGAGGAACTTAAGGCTAAGGCACTCAATATTTTGCTTTTTGGTTATATGTATATGCGTACAATG
>TCONS_00061314
AAAAAGCAAAATATTGAGTGCCTTAGCCTTAAGTTCCTCCGCATATGTACTACGTAATACTTCATTGTCATTGT
ACGCATTTACATATAACCAAAAAGCAAAACATTGAGTGCCTTAGCTTCAAGCTCCTCCTTCATTGTCACCACT
GTTTAAGAACCGAAACATTAAGAGTAGATCACCTGTTGCATTCCAGTCATGATAGCAGCCAGTTGTTCTTGAA
TCCTCTTGGCTACTTCTACACTGATCCGGTTGTCTA
>TCONS_00061343
ACCTTGATCAATAGGTGGAGGCAATGGAATATCAATTTGCATATGCTCTTTCTCGAGTAGATGGCGGCAATGG
GCTATCAGTTCACAGTCCGACTGCGGCTCACCTAAAATTTCGCCATGGTGATACCAAAAAGTATAATTTTGAA
TTATTCCATATACTTTCAAATGTGACTCAACTGTCTCACGTGTCCCTAAAGTTGTGTTACAACATTTGACACAA
GGACATCGTATTTCGTACAGTTCCCCTGTTCTTCTAAAAGCATAATCCAAAAACTTTTGCACCCCGATTGAGTA
GGCTTCATCAAGTCGATTATCAACAAGTTGCATCCATTGCTTACTAGGTGCCATAACCTGCTTTGACCCTCCAG
AGAGATGAGC
>TCONS_00061424
AAAAAAGGAACTGAAGGTACTGAACTGAAGCTCCTCATATGACCCTTCATTTTAGGCGGATAAACCGGTGGT
TTTTTCACAGATCCAAACTCTACCTAGCTAGCTAATCTTTTTGGTTTGCTCTCTTTTTTTTCTCTCTTTTTCTGACA
TGGGTTTTGTATTTTTCTACATTTTCTTTAGTGTTTGTATGAATGTTACTCATGATGTTACATTTCATGTCCAAGA
AAATTTTCATAAGGGTGTTTGGGGAGGAAATTTTGAAATGACTTTTCTTTAAAGATTTTACCTTTTTGGAAAGA
AATTATTGTTGGTGTAAAAAGTTTTTGCCTTTCTTTATTTGTGTATTGAGATTTCCAAAATGTATTTTAAGATATT
TATATACTGCTGAAAGAATTTCATAAGGGTGTTTGGGAAATTTTGAATGAAATGACTTTTCTTTAAAGATTTTA
CCTTTTGGGAAAGTGATAATTGTTGGTGGTATTGGTTAATTTTGAGTGAAGCTGATCTGTGTAGCCATTTGAAT
CTCATAGATTGT
>TCONS_00061427
AGGACATCCAATCTGGCTGCATCAGACCAAACACAGCAAGCACCTGTATCATTGCTCCAGGAAGATCATTTG
TCCAATGGAGTCCCAAGCAGCTAATGACCAGATCAACAGAATTTTCTTTCACAGGCAAATATTCTTCATCACC

AATAATGTATGATGTTTCAATGTTATCATTAGGCATTTGCTGCTCAGCATCTTTAAACAATTTTACCATATCAC
ATGATGTATCCATCATTAGTAATT
>TCONS_00061453
TTCAGCAGAGAAGAAAGACAGCGATAGTAGAAACATTGCTAGCAAACTGCTTTCCTCAGTCTGCTCTACGAC
GTCGAACAACTTTGGGTTGATCAAATGGCTGGGTGAGTGAAGCTGAATGGTTGTGGACATGGAGTCCACATC
TTTTAGAAGGCCAGATGCCAGTAGTATCTTACTGATGTATCTGTGGTCTGGATTGTGGTTCTGGCATAAGGATG
CAAGTTGATCCATGTTTGCTTTGTGGATGGTGGAGTTTGGTTTTCTGAGCTCATGAACCAATTCATCCTTGCTTT
CTAATATCTTATGGTTGGACTCGACGCCAAGGTCAAGTCTGGTCCTGTAAGGTGAATGATCTAGGTCATCTCG
ATACCAAACATCAGGTACTGCAAACCCATCATCTGTTAAGGTGGAAATGGACAGAGAAGATTATTAGTAGTC
AAGTCATGCTTCTTCACCAAATTCAATTCTAGCAAAACTCTTTCTGCAGGATTGAAAGTTCCTTCCCTTCGTTG
ACGAGATGTTGTTTGCACTGCCTTTGATGTTCTTCCTATCTTCTTATCTGGGGAAGTAAGATGCCAACCAGAGC
CTGCAACATTATTTGCTCTAGGTGTCATACCTTTGGATGTTTTCTTGTCAACTATGTCATCTCTGTTATATACAA
GGTCTTGAGTGTAAACTTCTTGAAGTTTGGAGATATTTGCCGTTGGCGCAGCAGAACTTGCAAATTTGATTTTA
TCACTTGGTTTGGCTGGTTTTGGTGTCAAATTTGAAGTTTCTAATCTTTTTGGGAGGTGAGTCACCTTTGTGGTC
AGAGATAGTGGGCTCCGTTGCTGCCCCTGCTGCACTGACTCCGCGTCAGATGCCCTCAGAGCTCTTTGGCCTT
CTAATTTTTCTCTTATTTCTGCATGGCT
>TCONS_00061493
CCTGGATGCTCAAGAAGAACCCAACAGATTTTGTATGTCTTTCTCAAACTGAAGAGGTGGTGTCCTGGGTGCA
TATCGTTCCACAATCTTTCCTTCTTTGTTCACAAGGAATTTGGTGAAGTTCCACTTGATAGCATTTCCAAGGAA
ACCACCTTTTTCTGATTTTAAGAACTTGAAAAGTGGTGCAGCGTTATCCCCGTTCACATCAACCTTTTCAAATA
CAGGGAATTCAGCTTTGAACCTGGTGCATACAGTTTCCTGAATCTCCTCATTTGTTCCAGGTTCTTGCCACAAG
AACTGGTTACAAGGAAAAGCTAAAATTTCAAAACCTTGATCTTTGTACTTCTCATACAGAATATTTAGCTCCT
TGTAGTTTGAATCTGTTAAACCACATTTAGATGCAACATTGACAATAAGAAGGACCTTTCCCCTATAATTGCTT
AAAGGTACCTCATTTCCCAGTATATCCTTGACAGTGAAATCGTAAATGGATTTGGGGGATCCTTCAGCCATAG
TTTGAGGAGAAGAAAAAGAAGGGTATCTATAGAATAAGAACAAGGCAAGAACAAGAAAGAAGAGAGCTGC
CAAATTGGT
>TCONS_00061589
TATGTCCAAGCGTTGGTGGACACAATTACCCCATACCTGTGTTGGTGGGAGGTAGCAGGTACCCCATGGAAT
AGTCGAGGTGCCAGCAAGCTGGCCTGAACGCCACGGTTATCAAAACAAATTGCTAGGCACTGATCCATGGCA
AACACTCCTACAAGATTCTGCATTTGTTTCCTCCAGTTTGCCAAAAACTTCTCTGTTCCACCTTTTTATGTCTGT
CTTTAGAAGTCTGAGTTTTTGTGCCTGGATAAAATCATGAGTACCACTGACCGCATATCCATCTCACCGTTCTT
G
>TCONS_00061605
GGTCAGTGGTACTCATGATTTTATCCAGGCACAAAAACTCAGACTTCTAAAGACAGACATAAAAAGGTGGAA
CAGAGAAGTTTTTGGCAAACTGGAGGAAACAAATGCAGAATCTTGTAGGAGTGTTTGCCATGGATCAGTGCC
TAGCAATTTGTTTTGATAACCGTGGCGTTCAGGCCAGCTTGCTGGCACCTCGACTATTCCATGGGGTACCTGCT
ACCTCCCACCAACACAGGTATGGGGTAATTGTGTCCACCAACGCTTG
>TCONS_00061826
CCAGGAACAGTCAAATGCCAATTTTTCTCTAAATGTAGCAATACATCCTTGGACTCCAGAGTTGAAGATTTCC
GATGTTTTGCCAAATTGCAAGCAAACGTAGTAACCGAATCAATAAAGTCATCAGCAATCTCTAAAAGAAGAT
CTTCAACTTCAGGATCAAGCTTTCCCCGCGAATCCACCTGTAAAACTAAATCCTGTATCTTTCTCTTCCCAAGA
AGCTGATTGTTTGCTTCAGTCCCCTGG

>TCONS_00061829
ATGGAGTAGAAGGATTAAGGATTCAGCTGAAATGTTGGTTTCTGCATCTGCATTGGTCGCTCCTGTAAATTGG
TCTCAAATGAAAGGATTAAGATGATATATTGCCTCCTTTGTCAATTTTAAGGAACTGGCTTGGATTTGTTTTGG
AGAACATAATGTTGTCTGGTGAGAAAAGAATGCTGTAGGGAAACGAGCATACTGTTGATGGAGGCATGACC
GGTCATCATCAAAGCTGAAATATGATTTCTAATGTATAGGTTAAACACCACAGAACTATTTTATTGTTGGTAT
AATCTAACTGTTGTACATGATATGAAAACTTAAACACAGGCAAAAGTGTATCTCTATCTTAATATGCTTTAAT
GTGTTATAACATATAGACCTAAAAACAGATAACATGGAATGTGCAAAAAC
>TCONS_00061868
GTTTTACCTTGAAGGAGTACATAGTTCATTTCATATGCTTGCTGTGACAATTTTTTTAAATCCAAGCAGGAACA
CATCCTATATAGAATACGTACAAGCTACTATTTTCATGGGAGGCCTTGAGTAATTGGGGGGAGGCTCCCAAC
AAGTTACAATCCTAAGCAAAATCACACTATCAGTCAAACCATTTAGAAGCTACACTACAAATCCACATTGCA
AATCT
>TCONS_00061898
TGGAGGAATATGTTGGCTTCCATGAACATGACATGCACTTTGGACTGGATGCCCGATCCGTCTAAACTGTCTA
ATTCTCAAAATCTGTTGTAAACTTCACTTTGAAACTGAGTTCTTTAACCATGCTTGTGCCGGGATATAAGAAG
ACCTAAAATCGATCAACGATGCCCTTTGTGGGTTTTCACCAATTGACCTCTCTCATTTGCCTTTTTCTCAACTCA
CCAT
>TCONS_00061909
GGGCTATCACAGTCAGGTGCGAATTGATTACCCCTATTTGTAAACTACCCCTCTTCCTATTTAGGGAGTTGAG
GCGAAAAATGGCTTGATGAACCGTTCGCCACGCACCGGCCACATTCACTTGCTGGTGACGGCGTTTTTTTAGC
TATGCATTACACACCTCATGTGGATCTAGCTTTCAACAGCGTAGAACACATTATGAGAGATGTTGAAGGGGG
CTGGTTGCTCCGTTA
>TCONS_00061939
TTTTTCTTTTTTGTGTCAGGAGGCACGTGCAGGAGAGAAGCTCAAGGAATGTCAGGATGGTTTGCGAGCGCAG
TTGGAGTAAGCAGCTGATCTGAACTTCTTGATTCCACCGTTTGGCCGAACGTCTTCAATACTGTAGCCGAGGG
GTGCTTCGTAGAGTAAGGATTTACTACTAGACTTTTTACCACCTTTAGACTCCATTAGATCATTCACGATATCA
AACTTTTTGAATTGGTTTAAATCTGTCTATCCCCGAATAAATTAATATCAACCTCCAATAATCCTACATCAACG
AAACAAACGAATCAGGAAACAAACTCAACAAAAAAAATATTAA
>TCONS_00061948
CAAACTCAAAATTAGATATAAAAAGCAACGACGTAAATAAAATAGAGCTAAAAACAACACGAGTAATAAG
TAGCATAATTTTATAACAAAAAACCGGATATCAGAATCTCAATTCAGAGCCATTAGGACAAAAACACTACTC
AAATGTCGCTGAAATTTTAAACTAGAACTTAAAGCATAAACCTAAAGATGCGGTTCGGTTCGATTAGGGCGA
AATACGGTTTTACTTGGATCGCTGCCTCATCTTTCGGCGCTTCCTCTTCAATCTCCTCATACGCTTCTTCTTCCA
CTTAGCTCTCATTCTGAATTGGCGATGATCAAAGAGTTCTTTTTACTTATCAGAGCGAATCTATAGATGGAAG
AAGAAATGCGGCAGTGATGGGAGGAAGTGAAGTT
>TCONS_00061972
CTAATATTTGAGGCACATTTTGGGCAATATTAATCAAGTATGGTGATCATGAGACGGACATTAGCCTGCTGCA
CAAGAATCAGAGAGTTTAGCATTGACTTTGCTGAACAAGAGAGAATTACAACATATGATGGCCTCGAGAGCT
GCATACGGAACTGTCAATCTTATGACAATGAAAGCGCGACTAGTAGAGGTGATGAG
>TCONS_00061976
AGACATGCATGCTGCTACAAGCTTCCCTGCCAATGGACTGAACTCGACTTCGGCCTGGTGTTGCATGTACAGA
ACTTATATTTTCTTCAACAAACTCAATGTCCATATCAGTAGCATTACTAAACAATCATCCATCAGCTCTACTCG

TCTGTTCGATCAACGACTGTCTCACCTCAACTACATACAGGACTAGTGTCTGATAATCCTGAGATTCTTGTAGC
AGGAAGTCAGCAAGCAGCTGCGGATGAATTGGGGTTGCTCGAGATATCTACTTGCAAAATTTCTCTTATCCTG
ATCCAAATGCTGTACCCTAAGTTTATAAGAACCCGAGAGTACGCTTCCATGATTAAATGCCCAATGTCCGTGC
CATACTGGATTTTTGTGACATTGAGGAATGTCTGAGGAAGGTTAGGATATTTAATTTTGAGCTGCTGCAGAAG
AGCTTCTGCTTGGCTCAATAAGACCTCCAATTTGTCTAGCTCAGATATTGGATCTTTTACGAAAGACCAAGGT
CTTCGGACTGGAGACTTTCCACTAGCTTGATCAGTTATTCTTTCTTTCCATGCAAGAATTGCTCCCTCTAACCTG
TTAACAACTTCAAGTGCACTGTTTTCAGATCTCAAGTTCAAGGAATCGAACATTTCCTCAGCAGAGGTTGATT
CTGCGGTCAGGATTCTATAAAGGTCCTCGCCGAGGCTTTCCCTCCCAGACTTTGGAAGTGCATCCTTGATAAC
TGATGGAACCGGCATTTCAGCTAAGATGTTTTCATTAATAGCTTTTGCTGCCTTGAATATTTGATTGGCCAATT
TTCCCTGATTCACCAATCTCTTTCTTTCAGTGTCAGAGAGCCCAGCGATGGGTACCTTGGGTGAAGGAAGGCA
CCACCTTGTGCTCTTTTCTTCTCCTCGACTACTCACTTCCGTATACCAAAACTCAGTATTAACCATTGAATCCA
GCATTCCAAGAAGCATGGAATCCAATTTCTGAAGTGCTGGAAGATTCATATGGATGTCTGCACGAGCTTTTGG
TGTCATTATCTCCAACCTCTGGCCATTAGAACCACTTTGTTTTGCAGGAACAAACTCAATCATATGTTTGGTAG
GTGAGAGCAGCCAATCCATTTCCCCTTGCCATTTCCTCTTCCTTTCCTCTGCAAGTGGCTCCAATTTCCACAAC
TCTCCAAACACAGATGCAGCAAGATTTGTAATGGCATTGGACAATGCTAAAGCAGTCGAAACTCCATTGCTT
CCTCCCGTCACATCCTCACCAAGCAACAATTTTGCAAATTTGTCTTTCATTGTTTCCACAT
>TCONS_00062049
TGGGTATATATATAGTGGCCTCAAGAGGATTTGTTGAATAATCACCTCACAAATGGAATACCAGAAGGGACT
TTTCTCATATAATCCTCATAGTCAGAACCAAAAAACTGCTTTAGGAAAAATTCCTCATAAGGTATCCTCCCCG
AGAAGAATTTCCACACAACAACTGCAAATGCAATGGTTGATATTGGATTACAAAGCATTATTTGAGTACCAA
TAGACCAGATGAAGAAACCATTATAACCAGGGTGCCGGACATATCTATAGATCCCATGTGTAACCAACTGAT
GACTTTCCTCGTGATAAACCTTTATTAGATGAGTGAAGGCTACGCCTGCGGTCACAATTGCCAGCTTCCTTATG
ATTTCTCCAAGAACAACCAATGCAAGGCCGAAATTACTTATCCACCAATATTCCTTTAAGCCAGGAAAGAAA
TAGAGTTCAATGAGGTACTCTATCAAGGAGCATATCATTGCCAGTAGATAATGTTTGCTTATCAAAAGCGACT
TAAGAGTTACGTTTGACTTCCCATGAATGAAAAGTGCCAGAATGTACTCGGAAACATGGAAGAATATTATTG
AAATGAACATTTGCGATAATTGTCTGCATGCTGTATATCCAAAAAGTTCTGCCATTTCACACCAATAAGTCAA
ACAAACCTCTCGTCGAAATACAAAAATTATTTCAATCTGAGTCACCAAAGGCTAGTACGCATGAAACCATAT
GTTGCAAATTTTAACTCCACACCTACAAATCGGTTTCGCCGGAGAGAGAGATCGGAGTTTCTAATTCGGATGG
CCAAAGAGGAAATACCCGG
>TCONS_00062141
AATATTATCAAACCAATTGCAACTATAAGCCATATCCACCATTTAATTGCTTCCCGAATGGAGGCACTATATC
CACTCTGCTCATCCCAAAATCTTGACCACAACATAACACCACCATACTTGCTCGACTTCTTGATCACCGGAAG
AATTTCGCCGATCAACACATCCGCCGGAATAAACCCACTTCCGGCAGCCTGTGGTGCCGCCGGAAGACCCAA
AAATATCCTTGTAGCGTTCACCGACGAGGTCCACCGGATCCAAGAATTTTCAATATTATCAGTATTATTAGGA
GTATACTGACACGGAGGATTATTGTAAAATTGAACCCAAACATTGTCAAAAAGACCTGTATTTAAAGCAGTA
CCAAGCAAACTATCGGGAAATGGACATTGTGGAGCTGCTGTTAAGTACACTTTTTTCCAATTCTACCATAAT
CTTTCAAGTATTTAGCTAGATCGTCATAGTAAAGAGACGATCCAAGCTCAATATCAAAATCAATTCCGTCTAG
AACAGCACTACCTAAAGGCCTAAAAGAGGAATTTGAATTTCCACCCAAAAATGTGTTCCATAAATAACGTGC
AAAAATTTTGGCGTCTTCTCTTGAAGCTAAAGAGTAATTTCCAGCTCCACCACCAATGGATAACATTACTTTG
ACGCCTAGATTTTGGCAAAGCTTGAGTTCTGGACCGACGGCGG
>TCONS_00062149

GCTGCAAAAACTTATGGTGAGCATCTCATATTACAGTCACTGAATTTGAAACCACCATGTAACAAATTCGGCT
TAAAGATCATCTTTCTTTGCAGAAAGACCAAGAAGTTTAGCTAGCTCCCCGTTTTCATAGGCCTCCACGGTAT
CATCTGATCCTCCGATATGCTTTCCGTTAATGAAAACTTGTGGCACAGTACGTCTGCCAACGATCTCACTAAG
GGCATCCTGAATACTCCAGCCATCATCTCTCTCATCAAGCTCAACAACATAAGGTTTTTGCTTCAACTCTTTAA
ATACAGCCTTTGCTTTCCTACAGTATGGGCAATAGGATTTGGAGAAGATAACAATGGAATGAGAAGAAATTG
TTTTCTTGACAAAGGCAGAGGCATCTCCTTCTCCAGCTTCAGCAAATGTTGATGTGTACAACAACACAGTCAC
TGATACTA
>TCONS_00062346
ACTTAAGTAACCCACAATAGACAATGCCTACGGTAATGAGGAGTTGAATTGCAGGCATAAACTAGAAGCATT
TTAATCTGGTGTTGGTGCGCCTTGGTTAAGCTCTGCAAATCTCATACCCACACGCATAGAATGCTTCAAGAAC
TTCTCAGCACATCGCCTGACACAGGTCTCCTCCTGTTTGTCCAAAGTTTTGCGTTTGAAAGTGTCTACACAATC
CGTGAAACATCTTTCAACCAGGGAATTGTACATTCTCAAGCTGTCACGGATTTGGAGCTGGTCAATCATGGCG
GCCATTTTAAGCTTATCTGCTTCAGGTAGCGAATCCAAATCTCCAATCATACTCTTGTCCATCTTGATTCCTAA
ATCTGATCCAACGCTATATGCAACTACTGCTTGTGTTTTGG
>TCONS_00062429
GCTACTGTCTGCACAACTAAGCAGATTTTCACTAGAACCTATGAACCATCATCGTCTTCGAGGTTGTTGACTCT
TTCACACTCGTCAATCCATTCACTATATATATCAATAGGCTCTGTCAGTGCCGTAACAGTTGTGCTGAAGCTCT
CTTGACAAATCCTGCATGTTGCCTCCCCAATCAAGTTTTTCATATCACTGATGAAGAACAGAGATTGAGATCG
CCTTATGTAATCTGCAAAATAAAGATTGAATTT
>TCONS_00062430
GCTACTGTCTGCACAACTAAGCAGATTTTCACTAGAACCTATGAACCATCATCGTCTTCGAGGTTGTTGACTCT
TTCACACTCGTCAATCCATTCACTATATATATCAATAGGCTCTGTCAGTGCCGTAACAGTTGTGCTGAAGCTCT
CTTGACAAATCCTGCATGTTGCCTCCCCAATCAAGTTTTTCATATCAATGCGACATTCAACGCTGGTGCCATGG
CTACAGAAAGGACAGCTGAAGACGGTGTCAAGTTTGTCCATTCTCTTCTTTGGAGGCGGCTTTGATTTCGACTT
CCTCTTCCCCATAGCTCAAGCAACTGATGAAGAACAGAGATTGAGATCGCCTTATGTAATCTGCAAAATAAA
GATTGAATTT
>TCONS_00062501
AAAGCAACTTCTGTAGCAACAAATATACAAAACAATTCTCAATCAATTCCTGAACATCAAGTCCACCTCATT
ACCTGCAGAAAACTCAGGAACTCGTGAACCAACAATTGAGGTAAAATTCAATGCTGGGAAAAGCAAACCCA
CAAAGGAATCTTCAAAATACACCGCAAAATTTACGAATCCCCAGCAATGAATCGCCGGCAAGCTCAGTTACT
GTCAAAGTTTTGGAGTAAAATTAAAGCAATATCAGGAGTCCAGGTAAAAA
>TCONS_00062502
TCAATCAATTCCTGAACATCAAGTCCACCTCATTACCTCAGGAACTCGTGAACCAACAATTGAGGTAAAATTC
AATGCTGGGAAAAGCAAACCCACAAAGGAATCTTCAAAATACACCGCAAAATTTACGAATCCCCAGCAATG
AATCGCCGGCAAGCTCAGTTACTGTCAAAGTTTTGGAGTAAAATTAAAGCAATATCAGGAGTCCAGGTAAAA
A
>TCONS_00062551
ATTCTTCATAATAAATGTTTTTATCAATAATACTAGAACGGTATCTTGAAAGATAATATGAGACCAAAAGCTT
GTACCAGGGATCTCATTCCATTTTCATAAATAGATTGCAAAAGTTTGCACACTAACAATTTCCTTTCCAATTCC
TTCTACAACAAACCCAGCATTACATCCTTATATTGTCTTCTGCTTTGCCTTCATTACAATCTTATACAACATCCA
GAAAATTACAAGCATATCTTTTACGTTTTTTACTTCATGATTGTCAGCCGTGACAATCGTGGAGAGACAAACG
AAAATGATGCAACTCAGAATGCACAGTATGGGAAGGAAGATTCCTCAATGGCGTGTGTTTGGAAAACAAAA

TGTTACTGCAAAATGCGATCAAATTAGTCAACGACTAGATTCCTACTATTCAAAACTGATGGCACCATGTTGA
AGCTAAGAAATTTCCGCAAATCGAAACAACTCGTCATGGAGGCAATTTGTGGGAATCAAAATACTCAGCAA
GGAAGTCGGAAATTCCCACTGCTCATGTTGTCTGTCTGTACATAAACATACTGGAACAAGTCACATGTCGGAC
ACAGACCACAGATATGCTTCCCTTTCGGTTGCTTGTTTCTCATTGGTCCTCTTGAACAACTGCTTCTCATATCCT
GTACTTCGGTCAACCCCATCCCAGTGTCGCCCTGGTCT
>TCONS_00062689
TGTACTTTCTTGAGTTCAAACAGTTTGCTCTGTCAGAAGAAGATTATTGAAACACCCATCGTCAAACAGCCCA
CAGCCACCCATGGACTCAACCGTTCACCTATTCTAGCAGCCTTCCAAAAGAAACCTCGAAACCCAGTTCTCTG
TTCAAGAAGTGCAGGGAAATGCATCTTCACATAAGTGCATGTACAAATCCCTAACAGCACTACCGTCAGAAA
GGAGTGGAAATTGAAGAGAGC
>TCONS_00062704
CATCTCTCTGATTATCGATAGGTGACTGACCTTGAATTTACGCCCGACTTGCTCTTCTCTTCGAGGTAGCAGAC
AAGCGCTTGGACAAACGGTTTGGATCTGGAGATCAGGGACTGTTCTAAAATCCCCCATCTTCATTTATTCACT
GGAATTTCGCTCTTTTCCTCTTGATTTTAGGCTTGGTGGAAGGTAGAACTGATTAAGAGAGGAA
>TCONS_00062738
GTTGCATGACACTAATATACTTGCGGTAGAAAACTGAGTAAATATCTTCATCCGTACAGGCATGTTTACATAG
ACAACATCAAAAAATTAGAAGAGCTAGAACGCATTTAGCGGCCTCGACCACGCCCACGTCCACGCCCACGT
CCGCGCCCACGCCCCAAAGGTTTTCCGGCAGTTGGCTTCTTAGGCTTGACCCTAGGTGTTTCTTCCACCAGCA
GTGTCTCAAGATTCAAGCTGTCAGGAAGGATATAATAGCGGATGTTGTTACCCCTCACACTCAGGTGATCCAA
TGTCACTGGATTCTTTCCTTTTAGCGTAATTTTGACAGCTTTCAAGTGTGTGTTCATGCTGACATCCACACCTGT
AATAGTTCCATGAACAACGGTGCCGTTCTTGAGCTCCATGGAGACCGTCTCATTATTTAACTTCATTAGGAAT
CTCACTAGCTTCATTGTCGTCGACGAAGATGTACAGACGCTTAGGGTTTTACAGAAGCAGAGAGCAAATTGC
TTTCTTTCCAATT
>TCONS_00062775
CTTATTAGCTTTAAGAAACAAGATTCCTAGTTTGATTTGACCATGGAATTATTGTCTTGGAGCTTTCTGTTACTG
TTCTCCTCTGTCATCAGAGTCTCTTTCTTGTCTTTGTAATTGTTGTTTTGTGGTGGTTTAGTTTTTACCGGTGGCCT
TGGCGGCCTCCTTGTTGTATGAGTATTTAGGCATCTTATGGCTATTATTGTCTTGAGTAGACACGGCTTGAAGC
TGGGAAATATTACCATGGATTTGG
>TCONS_00062780
TTTTCACGAGAACCGCATGAAACTGGAAAGAAAAGCAATGAGGAAAATATGGTGTGCCATAAAAGAGACAA
CCAGAAAGATCATAAATCAATGACTAGTCCCAAAACACTCTTCGACTCAACTGCAATTTAGGTGAAAGAGTT
TAACTACTATTCTTTCATGCGACTCAATTTGTGATTGAACAGCTATTTAGTTAGCTTTGAATGCAGTACAGCAC
TTTATTCAAGGTTAAATAGAGTATTGTCCAAAAATAATCGCTCAAATCATTATAGGGCCTGTGGAAAAGAATC
ATTAAAGGACCCATAGCAGGTTATTCTCAAAGGTGGAATATTTTGCAGCCCCTCCCACTTCAGCAGTTGGTGT
GGAAGCTTGCAACCAATATTTCCTCTCTTGTTCTAGGCTTTGCTCCTCACACTTCTCTCTGTTCCACCCAATTGT
GGCTATGCAGCCAAGGGCTTTGCATCATTTCGAACAAACTGAATACCATGGCCTGGATATATAGTTGAGGAA
CAAAACCAACATTTCTCCAATC
>TCONS_00062782
TGTAGCATAGTGTCCAAATCCATGGTAATATTTCCCAGCTTCAAGCCGTGTCTACTCAAGACAATAATAGCCA
TAAGATGCCTAAATACTCATACAACAAGGAGGCCGCCAAGGCCACCGGTAAAAACTAAACCACCACAAAAC
AACAATTACAAAGACAAGAAAGAGACTCTGATGACAGAGGAGAACAGTAACAGAAAGCTCCAAGACAATA
ATTCCATGGTCAAATCAAACTAGGAATCTTGTTTCTTAAAGCTAATAAGTCATTCGTACTTA

>TCONS_00062822
TTGACCGTCGCGACAACTTGCCGTCACAACAATGCAATTTTCAATTAGAAAAGTTAGAATGAAAAGTCAAAA
TCTTGGTTAAGAGCCTCTTCTAACAAAAACTGAGATTGTTCCATTAACTCAGGACTCAATCTAAACATCAAAA
CACAAAATTCCATCTGATTAAGAGCTCCATCTCCATCAAAATCACCTTCTTTTAGCATACTCTTAAGATCATCA
TCACTCAAATCTTGAAGCCCTAACAAAGCAGAATTCTTTTTTAGACTCTCAAATGTAATGAC
>TCONS_00062823
GGAGAATAACACAAAAGGGTGCAGTAAACATCTGTATATATATTTCAATGTGTTTTATGTGCTTACAAAAGTT
AGAATGAAAACTCAGAATCTTGGTTAAGAGCTTCTTCTAACAAAAACTGAGATTGCTGCATTAACTCAGGAC
TCAATCTGAACATCAAAACACAAAATTCCATCTGATTAAGAGCTCCATCTCCATCAAAATCACCTTCTTTAAG
CATACTCTTAAGATCATCATCACTCAAATCTTGAAGCCCTAACAAAGCAGAATTCTTTTTTAGACTCTCAAAT
GTAATGACCCTTTTATCCTTATCCATTAATAGCTGAAACCCTTTACATAATTCACCAATCAAACCATCCCCACC
TAGTTTTTCAGCCATAAGTGGCAAAAAATCATTGAAGTTGGATTTTCTTGAATTAGCC
>TCONS_00062897
AAACAGGAGGCTGATGCACAAGAAAGAATTTGATTCCTCCTATACAGCATTCACCTCAGATGGAGCAGAGTA
TCTGGTCCAGAATACCGACATCAAACTGGTTGGTGTTGATTACTTGTCTGTCGCAATTAATCCCAAGGATCAG
CTCGCTAAAGTTCATCAACTTCTTCTGGCTCCTAAAGTAAGTATAACATCCATTTGCCCATTTCGGTTTACT
>TCONS_00062898
AAACAGGAGGCTGATGCACAAGAAAGAATTTGATTCCTCCTATACAGCATTCACCTCAGATGGAGCAGAGTA
TCTTGGTCCAGAATACCGACATCAAACTGGTTGGTGTTGATTACTTGTCTGTCGCAATTAATCCCAAGGATCA
GCTCGCTAAAGTTCATCAACTTCTTCTGGCTCCTAAAGATATAATTCCGGTGGAAGGCCTCAAGCTCGATGAT
GCCGTCCCAGGAGTTTACACAATTCACTGCCTACCTCTGAGGCTGAATCATG
>TCONS_00062904
CTTACCCTTGAAACCACCTTTTTGTTGGCCACTGAAGTTTTTGTTGTTGGGAGATTTGCCAGAGAATTGGCCAC
CAGATTTTGGGCTCTGACCTTTCCCCTTGTTACCACCATTGGCTGCTGCAGGTTTACCATTTGCTTAGCAAAG
GCAGGTGTCGCTGGACCTTTCTTACCGCCTGACTTGTCGGGTGTAGCTTGTTTTGCTTTTTTGGCAAAACCAGC
CTTAGCGGGTGGAGCAGGTCTCTT
>TCONS_00062923
GATTGCCAACTATACACATCTATCTCTCTTTCACCACCCCAACTTCTCTCCCATTTCTTGAATCTCTATATCCAA
TATTATTCTTTAATTGATGTGGTTAATATCTCAGAAATGGCAATCGCAGCCGCCATTATTCTTCCTATTGGTCTT
CTCTTCCTTTTATCCGGCCTCATTATCAACTTCATTCAGGCACTTCTCTTCATTCTAGTTCGACCCTGTTCGAAA
AACATATACATAAGAATCAATAAAGAAGTTACAGAATTGTTATGGTTGGAACTCATATGGCTTTTCGACTGGT
GGGCAAATGTTAAGGAAAAGAGGATGCACTAGTAATCTCCAACCATAGAAGTGACATTGACTGGCTTGTC
>TCONS_00062951
CCTCCCACCAACAGCTTGTATTATCTATACTATATAGCAGATATTACTTTCTATGGAGCTGAGAGATCTATGTG
AGAAGCGATCAAAAAGCATCTGGATGAACGCAAGCAAAAAAAAAATGGCAAACAGAAAAGAAAACACAT
GCGTAGTAGTGAAGAGGTTAAAATCAAGATGGGCCACAATGGAGTTGACCACCCAAATGACATACTGTAATT
ATAGAATGCTATCAACGGATTTCTATACAGATAACTAAGGAATCAGATCTCTATCTAACTCACCTATACATTA
GTGATCACCATTCGTCATATTGATGATAGCTCCTTCCAGGCCGTTTTGCCTTTCATCAGAGCACCATTAGTCAC
GTCAGTCGCTGCTCCTTGCAGGCCATTCTGCCTTTCATCAGCATTATCGTTGAAACCTTTCTTCTTATTTAGACC
ATGAGCAGACATAAGTTCATAGAACTTGCGCATCAGCATTTCTTCCTCGACCTGAAGTCTGTGCAATTCATCT
GACATTGATTTATGGGCATTCAAGAGTAGTTCACTTTCTTTCTCCAGATGCTTCAAGGTTTCTCCTGCGTATTTG

GGATCCATAAAAAAAAGTTTGATCTTTCGCCACAAACCCTTAGTGGCTCGTAGGAATTCGCTCAAAATCAGG
CAATCCCGC
>TCONS_00063108
ATAATCTTGAACTTTCTTTCCTCCATCCCGCAAAATCTTAATAGTAGGGAAACCCTGGATCTCATACTGCGTTG
CAAGTGCTCTATTTGCTTCATCACTAGCATCATACTTAGCTAGAACAATTGGAGGGTCGTGACTGCTCAACTCT
GCGGCAGCTTTTTCATACTCAGGAGCAAGACTCTTACAGTGCCCGCACCTTGTATTTCTGCAAGAAGCTGTTTC
TGTAGCTTGAACCTGTAACCAAAATTCAACGACGGTGAAGTTGTGCTTGGCAATAGTATCAGTGAGGTTAGA
ATGGTCCAAGGTCAACACATACTCCTTTTCCCCTTCTGCAGCAACACAAACTGCAAGAAGAAAGGCTGCTAC
AATTACCAAAATGCCCCTCATTTCTCTC
>TCONS_00063171
CCTGCTGCCATGCTTGGATTGCCAAGCCCCTTTTATCCATTCGCCCACTCAAAGCATCGCGTAAAATTGAAGG
AAAGTAGCGCAAAGTTTTCTCAGACCAGGTATGCTGGCTAGTTGCTAGAATCTGTTCTAGCATTGTCTGAAGA
TACAGTAACTGATCAAACTCCCCGATTCCACGAGTTTTGATTATGATTGCAAGAGTAACAATTGTTATTCTATT
TAAAGTCTCAGTAAACTCAGTTGGACCCTTCCCTTCCCTCTTCACATAGAAAAAATCTCTCAACGACAAAATC
AGATTCATACATAAATTCTCAGCCAATCGGAAAATGCGATGATCTCCACGTTTCCCCTTCAGTGTAGAAATCA
TTTTGGTGACATCATACATCAAGGTCTTGCTCTTGCCTTGATACCTGTAGAGTGAAAGGAAACGATAGTTAAG
AATTTCAAACACCAAGATCGTGTTTCCAGGCTTGGTCAATGTGGTGGGATTCCTCGATGTCAG
>TCONS_00063398
CAATTCAAGAAGGCATCGATGATTGCTGTGTAAGCAACTCGATCAATATGCATGTTGTTGGATGATTTCATTT
CTATGACAATCTTTTGAACCGAAAGAAGATCCTGAGCATGACCAAAACCCTTTAGCAACGTAGTATAGGTGA
TTACATCAGGGAAAATGTCATCATCATCAACAGTTTGTGCTCTGACCTTCATGTCCTCAAAGAACTGCATTGC
AACATCCAACTTTCCAGTCTCCACACATGCAAGGATTAGTTTATTATATGTGAGCTTATCCAGGTTCAGTCCGT
GTCTTAGTATTTCATCATGCAAACTTAATGCTGCCTCAGGACAACCAGTGCTTATGTACCCCTTCATCAATAAG
TTGTAGACCAAGACGGATGGATATCCGCCCTCGTGAAGTACAAAACCATATCGTGCAAGAAGACCATTAGCA
CGGCGTAGATCCCCTGCTTCAATTAGAGAATTCAGCAGACCACTAACAAGGGGTGGCGAAAGGTTTGGACTT
CCAACAGCAGTACCTTGTTCAATAGCTTCAAGTAATTCAAATGCTTGATCAATTCTTCGGGCATCACCCAAAC
CCTTTAGAAGTGTAGCATAAGTGATATTATCAACACCACAACCCTCTGGGCCTGACATTTCATCAAAGACCCT
TAAGGCCGATTCTATATCAGCACAATGAACACAAGCCTGCATCACTGCATTCATCACAATGGTGTTCAACCG
CCCATGTCGTTTTTTCGCAATCTCAATTTCCTCAAAAATCTGGTGGAGTTGTCTACGGCGAGTGAGCTCAACGA
TGCGGGAAGTGAGGGGTTT
>TCONS_00063448
CACCTGACAACTTCCCAAAGCACAAGAAACTTCAGCTCCTAATCCCGGGCATCATAAGCTTACCAGTGGGAA
ACAGAAGATTGTGAGTCCAGGTTGTTGTAGAATTGAGTCAACCGCCGCCGATTTTCGGGTTTCCGGTTAATAG
GTTTCTTCCGCAAACCAAATATTTCTTGGACCAAAGGTACATGAACCAGAACTAATTTCCAGGCCAAGAACC
AACCTAAGAGGAGTAAGGAGAGGACGAAGATGATAGCAAGAAGGGGACGAAAGAGGAATAGACTCAGAT
ATTCCCACGATGATACGGCATTCAGCTCGTAGGA
>TCONS_00063449
CACCTGACAACTTCCCAAAGCACAAGAAACTTCAGCTCCTAATCCCGGGCATCATAAGCTTACCTATACAGT
CAGTGGGAAACAGAAGATTGTGAGTCCAGGTTGTTGTAGAATTGAGTCAACCGCCGCCGATTTTCGGGTTTCC
GGTTAATAGGTTTCTTCCGCAAACCAAATATTTCTTGGACCAAAGGTACATGAACCAGAACTAATTTCCAGGC
CAAGAACCAACCTAAGAGGAGTAAGGAGAGGACGAAGATGATAGCAAGAAGGGGACGAAAGAGGAATAG
ACTCAGATATTCCCACGATGATACGGCATTCAGCTCGTAGGA

>TCONS_00063563
AAAAATTTAACACAGATTCACCAGATTGCCTTGTGCAGTTCTTGTAATGTATATTATGGTCAATGTAGTTCTTG
TTCAAGGTGATAACATTTCTAGATTCTTTTCCTGTATTCCATTACTTCAGTTTTTGTTTGAGATATAGGTTGAGA
AAACAAGTTGTAAATTCCAAGTTGGGAAATGTAGCCTTTGATCAGCACCTTTCAATAGGTGAATTACAGGTA
AATAGGAATCATG
>TCONS_00063598
TTTGCTACAGTTGTTTCCATATCAAGAATTATGCCTACATCCACCTGCTTTACAGCATTAGTTCCATCTTCACCA
CTTATAGGCATTATGTATTGGAAAAAGCTAATGATGGAGACAAGTTGAATCAAAAGAATTAGGAAGTGGCAT
CTTGGATTTTGCATTTCGAAAAATCTGTAATCCCAGAGTTTGGAAAACAGTTGTGCTTATGCAAG
>TCONS_00063666
ATGTTTGATATAGCTCCAGTCCTCCCAAGATGCTTCATTTTCACTCAAATTAGCCCATTGAACCAATACTTTCA
CCCCAACTCCATTATTGACCTTTACCATTTTCCTCTGAAGAATAGCGACTGGTTGAATCAAGATTTTTCCTTCTC
CATCGCAGAGGGGAGGTTGTTGTGCTGGAACAATGGAGGGACCTATGCGACGCTTCAACTGGGAGACATGA
AACACTGGGTGTATTTGTGACCCAGGTGGAAGCTGTAATCGGTACTGCTTCAGGTTTGAGTGGAATCTGATGA
TCATGGGTTCTCCTTGGTGGCAGACACTTTGGTTCAGCAAACACTCCTGGGAACTCAGCTATAATGTCCTGAA
TCTTGTCAGCCAAGCTTTTGGATCTCCTTCTTCGAAGTAAGGGCAATCAATTTTGGAGTTGAAATTATTCGAGT
AGGATCGCCCCACAGGAACCTCATGATTCTCCATCATCCT
>TCONS_00063670
CACTTTTTGTCTTCCCACCAACTGGAATTGCGTTTCAATGGCGATAACGAGTGGCTTGGCAACCTCTTGTGCAG
TTGCAGGCTTAAAATCTAAGGCAAAATCAGTCGTCGAAGCATTAAGCCGTTTGGTTTATTGTACGAGATCGCA
CTTGTTGAGCCCTAGATGTGACGTTGGTTCGAACTCAGTGGTTAAAAATGGTGTTAAGGAATCCAAAGCGTCC
AGTTTT
>TCONS_00063734
AATGAAAGGAAAAACTGCATATAGGACCTATCATTCATACCGACTTGGAAATCATCTTTATCCATTTGGCGGC
ATTGGTAAGTGCAGAGCATTTGGTCTGAAGTTTCAGGATGATCGTGAGAAGACTCCGGTAACGGCGTATCCTC
GTAGATGATGAGCTGCCGATTGCCGGAGTTCGGACTGCCATCTTCAGGTTGCTTGCTCTGCGCCCTTCGCTTCA
ATGGTGGTGTTCTCAGCATCATGATTCCTTCCTTTTTC
>TCONS_00063840
TGGCTTGTAAATTGGAACAGGTGGTGGCATAGGTTTTACTACCGGTGGCTCGTATACCGGAACCGGAGGAGG
CAGTGGCTTCACTACTGGTGGCTTGTATACTGGAACTGGAGGAGGAAGTGGCTTTACTACTGGTGGTTCTGGT
TTGGGCTTAGGTTTGTAAGTGGGAACTGCAGGAGGCTTTGGTTTTGGGGGGCATGGCTTTTTAACTGGAGGCTT
TGGTTTAGGTTTGTAAATAGGAACTGAAGGAGGATTAGGTTTTACTACTGGTGGTTCGGGTTTAGGCTTGGGTT
TGTAAATGGGAACTGAAGGAGGCTTTGGTTTTGGTTTGTAAACAGGAACTGGAGGAGGCATAGGCTTTACTA
CTGGTGGTTCTGGTTTAGGCTTGGGTTTGTAAATGGGAACTGGAGGAGGAAGTGGCTTTACTACCGGTGGTTC
TGGTGTAGGATTGTAAACGGGAACCGGAGGAGGATTTGGCTTTGGAATTGGAGGTGGACATGGTTTTTTGAA
AATTGGAGGAATAGGGGGAAGTGGGAAAGGTGGCAAATTAGGGAAATGTTTCATCCAAGGATGATCTTTAG
GGAAAGGTGGCAACGTTGGCAATGGTGGATATTTAAAGTGAGGCCACAAGAAAGCAGAAGTGCATAATGCT
GTTGAGAATTTAATATTTCCAGCAGGTTTT
>TCONS_00063914
CCAATTCCAGAAGGCATCAGGTTTGATTCTTCTAAGTCGAAAGTTAGTTTTATGTGCTGAAAATTTTGAAGAA
AGTTTGAGTTGTAGCTGAACTTCGACTCGATCATCTGAGTTAATTGATTTGAATGAATTCGGCTCGCGTTGCTT
ACTACTTTGTTTCTGAATGTTGAGCTAAAATTGATCTTGGTATTCCTTGCTGTTGGATTTGATAGTGTCTATTGC

TATTGAAGTCTAATTCCTTGCTGCATCAGTTGGATTAACTTGTGATCTTTGAGAAAATGTATTCATTCTGTCCA
AAACTGTGAACTTGTAAAAAAACTGAATTCACTCTTGTTTTACTGTTAAGAAATGAATCTCTAGAAGGAATGT
GAGCTCAGTTCTTAAGCTGGTTGCCATCCTCATATTGAACTGAGATATTGTTACTGCTGATTTGTGAGTGAGAA
TTGCTAGACTTGCTGGAGTTCAAT
>TCONS_00063939
GATCTGGTACTACAGGCAAATGGCGTGAAGTGGCAGAATTGCAGCCTTGATACAAAGTCGCAGCTTGAGAGC
CCAATCCATACACACGTCTTTTCTTCTCTCCGCCGACAACATCCAAGTATAGGCGATTTATATCGGCATTTTGA
ACTTCTTGTGAATCATCGGATGAGCTCGGAATAGCAGCAGCCATCGCAACTTCCATTTTATCACCAACAGTCT
TAGACTTGGTATCCACAAAGTTCCC
>TCONS_00064094
TCAGTAATTGCTTTGAGCTTTCTTAATCATCGCTTATTCATCCAGAATGTTATTTGTTCAGTGTTCACTAAATGA
ACAATGTTAACTTATACAAGAATTTGTCACATAATGTATCAGTCTGTTTCAACATTATTGCTTAACAAATTATA
ATACTGACTTATCTTTCAAAATGACATCATTGTACCCAAATATTCTTACAAAACAAGCGATCAACTGTTCCTGT
ATCACTTCTTCAGCAGGAAGCTCTACATCTGCCTCTTTCTTCAAAGATGTAACATCTTTATCCGCAATCCCGCA
AGGCACAATATGCTTAAAGTAGTTTAAATCAGGATCCATGTTGAATGCTAATCCATGAGACGTAATCCCAGA
TGAAATTCTAACTCCAATTGCACCAATCTTTCTGTCCTCGACCCAAACCCCTGTTTCGCATCTTTTTCCAACCTG
TGCTTTCACACCATATATAGATGCTAATTCAATCATGGTTAGCTCAAGCTTCTCCACATATTTCGAGCCCCCA
AACCAATATCTCTCAATGAAACAATCGGATACAAGATCGCCTGGTGAGGACCATGAAAAGTAATGTCACCTC
CTCTTTGTGTATAGTGAAGTTCAGCCCCCACGGCTTTGAGCTCCGAATCAGGTATGAGTAGATTGTGAACTGTT
TGCCTTTTGCCAAGAGTATAAGTAGGTGGATGTTGAAGAGATAATAGGGTATCAGTAATTTTAAGAGCTTTTC
TATCAGATGCTAGCTTCTCTTGCAGCTTCAGTGCCTCCAAGTAATTGACGGTGCCCATTTTCCAAACCTCAAGG
CTTCGCCTCATTCTTCTGTAATCAGAGGAGTGACACACCAAAAGCCTATTACCCGGTCAATTCTCTGTTCAAAT
TTCTGTCACGCGCCGGTGAGAGCAGCTAGTCCAACCCACCCACCTCTCTCCCTCTCTCTATATCTCGTTGGCTA
TGGCATTTGGAGAAACTTTATGTTAT
>TCONS_00064214
CAAACTGTCATTAAGTCAATAGACAACAAATTTAAAAGCGGGAACATTTCACTCCTTGGTGGTCTTGTTTATG
AGTGATTTGTGAATGTGAGGGATAACACCACCACCAGCAATGGTCCCTTTAATAAGCGTGTCGAGTTCCTCAT
CTCCACGAATGGCAAGCTGCAAGTGCCTTGGTGTGATCCTCTTCACTTTGAGATCCTTGCTTGCATTACCAGCC
AATTCAAGAACCTCTGCTGTCAGGTATTCCAGAATTGCAGCAGAGTAAACAGCTGCCGTCGCTCCTACCCTTC
CATGGGCTGAGGTTCTTGACTTGAGTTGTCGGTGAATTCTACCCACTGGAAACTGGAGACCAGCACGTGATGA
ACGAGAAACAGGCCTTTTCTTGTCTTTCTCTTTGTTGGCTGCTGCAGCTGCTGCCGCCGCTGTTGTCTTTCCTGC
TAGCAGTCCTTTGCCTCCTTTCCCCGCCATCTTCAAATCTTAATTAATTATCACATAAATTAAAAAGGCAAGA
AAGGAAAAGGTCAGTATTTTGTTTTTG
>TCONS_00064230
GCTTGAAGCAAGTGGAAATGCTCAAGGGAATTTCTTTTTCAATGGCAGTAGGAAAGCCTACTTGAGTAGTGC
GTTGGCTCTACACTTGGACAGCTTCATCGAGACTTCACATACGTAGATTGCCATGTTTATATCCGAACGAAAA
CACCTTACTGATGTTTTTGTTTCCAGACTGAAATCTTTAACTATAAGAAGGGAATCGATTAAAGCCAGATTAC
CGCCTATGGTACACTAAGAATTTAATACTCAATGAGAAAAGGTGTTAACTAAATAGAAAAATCCCGATTTCG
ATCATCTTTGACGCCTCCTCCGTATCGAGATTCAGTCGCACCATACCTG
>TCONS_00064231
GCTTGAAGCAAGTGGAAATGCTCAAGGGAATTTCTTTTTCAATGGCAGTAGGAAAGCCTACTTGAGTAGTGC
GTTGGCTCTACACTTGGACAGCTTCATCGAGACTTCACATACGTAGATTGCCATGTTTATATCCGAACGAAAA

CACCTTAAAGATGTTTTTGTTTCCAGAATGAAATCTTTAACTATAGTAGGGAATCGATTAAAGCCAGTTATCG
CCTATGGTACACTAAGAATTTAATACTCAATGAGAAAAGGTGTTAACTAAATAGAAAAATACCGATTTCGAT
CATCTTTGACGCCTCCTCCGTGCCGAGATTCAGTCGCACCATATCTGGTTCTTGGTAATGACATCTGTCCTTTTG
CACCTCATCCGCCGTCTCCAATAAAATAAAAACTTGTCTAAAGACTAGATGTTGTAGGGTACGTCTTAAAAGT
GCTCCTCCGCTACGTCGTGCCTTACGTCAAGAGGTGGTCGTGGCTTCGCTCCTGCGACCGAACCGAATCTGTG
TAATCAAGTTTCATGCTCGAGCTCTAGGAGAATGGTGATCCCTTGGAAAAGGAAAATCGACAAGGAAGATAT
GGAGTCAACGGCAAAATTACTCTTCATTCGTTGTATGGACCTCAACCGGCTCTACCTAAGAACATAACGAAA
GAAATATATATATATATATATATATATATATATATAAGATGATCCATGATTTCACGGCACTCTGAAGAATCAT
GACAGATTTGTTCGGGAGGAAAAAAAGAAGGATTCGAGGTTGGTTGAAATGCCCCTTCTGTGGCTGATAATG
GGGAGCGACAGCTGGACAGATCGATTTAGATATGGAGGTCTTATCCGGTGGATGGACGTCTCATTTGATCCTA
GTTTTGTCAATGATGGAGATTCAGAGATAGGGACAGGTACTAGGGG
>TCONS_00064243
TTATCCTATTCTGTTTTTTGTTACTTGGCAGACTTTGGGTTTCCTTCAACACTTCCACATACGGATGGCTCATCT
GAAGGAGTTTCTTCTGAAACGAGAGCCCGTCCAAATGTAAAACCTGTCTGGATACGCCCTGCTATTCTAGATT
CATAAATGTTTGAGGAGAAGCAGCCACTTTGGAATTATCTTTGGTATATCTAAGTAATAGATTGCATGCTGAT
GATATATCTGAAGTCTGAACAAAGTGGAGAATGGAGACTCGTAATCCTGAAGTGTTATCAGGTCTTGGTGCA
CAAGGTAGCATACTGTCGAGGAGAAATTTTACGTACTTAATTTTACGATTTATATACATGTAGGCCATATGTTC
TCCCATGTCTCATAAATAGTCAAAATCATAGTTGGCATCCCTTTTCTTATTAAATGTATGTAAAATCTTTTGATT
CTCCTATGTATAATTAAGTCGCTCTTTAGAACCAAACAAAGCTATGCTTTTGATTCTCCTACTGTAGAATTCTC
GAATCTGAAAAGCTGTTTGGAGCAAACTTTGCTATACTTTTAGAACATAGTTATGCTTGATCAAATTGTCAGT
ACGATTACAGATGAGAATGGTGACTGAACATCACTACAATGTTTTATGCATTTTTACCTCTGCC
>TCONS_00064248
TGGTGCGACTGAATCTCGATACGGAGGAGGCGTCAAAGATGATCGAAATCGGGATTTTTCTATTTAGTTAACA
CCTTTTCTCATTGAGTATTAAATTCTTAGTGTACCATAGGCGGTAATCTGGCTTTAATCGATTCCCTTCTTATAG
TTAAAGATTTCAGTCTGGAAACAAAAACATCAGTAAGGTGTTTTCGTTCGGATATAAACATGGCAATCTACGT
ATGTGAAGTCTCGATGAAGCTGTCCAAGTGTAGAGCCAACGCACTACTCAAGTAGGCTTTCCTACTGCCATTG
AAAAAGAAATTCCCTTGAGCATTTCCACTTGCTTCAAGC
>TCONS_00064252
TTATCCTATTCTGTTTTTTGTTACTTGGCAGACTTTGGGTTTCCTTCAACACTTCCACATACGGATGGCTCATCT
GAAGGAGTTTCTTCTGAAACGAGAGCCCGTCCAAATGTAAAACCTGTCTGGATACGCCCTGCTATTCTAGATT
CATAAATGTTTGAGGAGAAGCAGCCACTTTGGAATTATCTTTGGTATATCTAAGTAATAGATTGCATGCTGAT
GATATATCTGAAGTCTGAACAAAGTGGAGAATGGAGACTCGTAATCCTGAAGTGTTATCAGGTCTTGGTGCA
CAAGGCAATTCCACCTTTATTGCTAAAGCTATTGGCTGAAGCTTTGAGGTATGAAATCCCTTTGTTTATATATG
TAGAATTCATGTTTCCCTCTGTCGTTCTCGTTTTAGACTCTAAATACTTTCTAACTGTCATGTTTGGTGCTAATA
ATCTTAACCTCAATCTTTTTCTTGTGTAATGCTGCTAGTCCTTGCCTCTGTCAACAATAAAGACATGCCGCCGC
CTCATTAGGATAAAACACTTCATGAACCTTGTTGTTGCTCTTAGTATGGGAATAATCTAAGCTGCTACTTCATT
ATGATAGAACTCTTAAATGAGCCTTGTTCTAAGTATGGGAATAATCTAGGTTACTTTCAGTAATGCACACCTT
ATGGGACCTTATTTCACCTCCTTCTTAGACTCGTAATGAGTATGCATCTGGAAGGCTAGCTGAAGTGCTAGAA
ATAAATGTTCA
>TCONS_00064258
GGAAGAACGATGACATTACCAATACAACATAGTTTCTAGTCGCGATCAGCATCTTCCAATTCTTCATCCTCAG
GTACACCACGAAGATAGAGAACATTATTACATCTAATCAGGATCTCTCCGAGAGAACCAGTGCATTGTCCAT

CAATGTATTCTTCTGCGTTTGCAAGCTGCAAGTTCATGTATGAATCCACGGAGACCAAATACCCTTTGTACTCC
ATTCCCCATTTAAGCTTTACCATCACAGGCTTTCCCGTCAAATTGTTCAAATAAGGCTTGGGATTAACTGGTAC
TGACATGGCTTCGGTTGATTTGCTGGTGAGACGGCTAAGCTGCTACTGAAGCTCG
>TCONS_00064292
TGAAAATGAGAACTTACTCATGAGTAAAGACATCTGCAACCAGAATAGCACAGATGCAAGCATAATCATTG
ATTACTTTCTAGGATGAAAATTGGGAAAGATAGAAGCTTGATCAAGAAGCACTTCATCAGCTTGTTCTTCAAG
CCAGGAAATGAAAACTTTATGAACAATCATCCTAACCAGATCAACTTCCACAAATCTGGTTGGAAATACAAA
AAGATATCACCAACCAATTTCAGAGGATATAAAAGATGTTGTACATTATACAAAATATGTATATTCAAATGA
GTAGTGGGAAGAGGCAAACTGAATAAACTTGAGAGAATACAAAAAGATATCTCATTCAAATGCCTTTCTTTA
ATCCATCTTTATTGCATAATCAAAAGCACATTAAAAACTTTAAGATTGAGAATTC
>TCONS_00064407
GGTACTTCTAATGTCAAGTTACCTTCTAACATCTCAATAACTTTGCTCATTGATGGCCTTACTGCTGGATCGGT
TTGTATGCACCACAGACCTACTAATATCATCTTCCTTGCAGTTTCTTCTTCTTCTTTGTTTATAATCCCTTGTAGT
TTCAGCTCATCGTCTAGCTGAACGTGCTGATACGCCCAGTAAGGAAAATATATCTCACTTGTATGGCTTACTTC
TTCTTTCACATTTTTTCTTCCTCCGACCATTTCTAGAACCATCATTCCATAACTATAGACATCAGACTTGTGTGA
GACTCCTCCAAAACTCCTACTGATTACTTCAGGAGCAATATAACCAATCGTCCCTCGTGCTCCTAACATGGAT
ACAATGCTTTCTTTCCTTGTGCAGAGTTTAGCCAAACCAAAATCTGATATTTTAGGACAGAATTCCTCATCAA
GGAGTATGTTATGAGGCTTTATGTCGAAATGCAAAATTCTTGTGTTGCATCCCCGGTGAAGATACTCCAATCC
TTTAGCAATTCCAACTTTGTAGACCCCTCCATAACCTCCTTCACCAAGCTTATCGTTGAATCCGTGTGTCATTTT
CTTGATTTCCCTATAGCTGTATCTCTTTGTTGCCAGTGATCCATATTGCTTAAGTAAGGCCTCTAGACTCTGATC
TTCTGTTTTGCCGTTGATTAGTCTTATGCACTTCTTGAATGTTTTTCTATATTGTGTGTACATTATACCAAAGAA
GATTAACATCCCAATTCCAGCAACTGAAATTCCTATTGACAGAG
>TCONS_00064446
TCTGATCTCTAATTATAAAGACAATTATGTAGATTTATCTACGGAAACAAGCTGATTGATACAACAATTTGAA
ACAAGGACAATTAAGAGATTGTTTAACACGTACAATATTATATCTTCTCACAAGAGATAGCTAGAAATTAAA
TACTAAATATCAACAGATGAAGAGGATGGAAGGGTCGCAGCAACACTCATCAATCAGGAAACAGCAACACA
GAGCTGCCAGACATCCCTCAAGAAAGCCAGGTTCCCTTCTGGGGGGAGGAGCTGTATAATATTGTGGTGGCG
GCATCACTGGAGGACCTTGATAATATCCTTGAGCAGGGTAGGGATACGCGTACTTTGGTGGCTCACTCATCCT
CG
>TCONS_00064508
AGCTAATTTAAGATCATCTCGGGAAACCATTTGACTAGCAAATTCCGCCAAACCTACAACTTTTATTGACAGT
TTATAAGTTACATTGGAAAAAACAAATAAAACATAAGCTGTGTGCATCACTTGGACAACTGCTGAGAAACAT
CCTTCTTTTCTTCTTGTTTTGGTCCTGAAATAGCTGCTGTTGCTGCAGCAGTCATGGCAGCAGCAGCTGCACCT
AATTTTACACCTCCTGCAGCTTTAACAGCACCACCAATTTTTGCAAAGGCAGCTATTCCTCCTACAAGAATGG
CTCTCAATGCAACTGTTGGTTTTATACTCATATTTGTCTACGATTAGATTTCGCCTGAGGAAGTGATACGGGG
>TCONS_00064512
TTTTTCCCCGTATCACTTCCTCAGGCGAAATCTAATCGTAGACAAATATGAGTATAAAACCAACAGTTGCATT
GAGAGCCATTCTTGTAGGAGGAATAGCTGCCTTTGCAAAAATTGGTGGTGCTGTTAAAGCTGCAGGAGGTGT
AAAATTAGGTGCAGCTGCTGCTGCCATGACTGCTGCAGCAACAGCAGCTATTTCAGGACCAAAACAAGAAG
AAAAGAAGGATGTTTCTCAGCAGTTGTCCAAGTGATGCACACAGCTTATGTTTTATTTGTTTTTTCCAATGTAA
CTTATAAACTGTCAATAAAAGTTGTAGGTTTGGCGGAATTTGCTAGTCAAATGGTTTCCCGAGATGATCTTAA
ATTAGCTCAGATATATGGGTCCTTACATTGCGGGACTCGCTAGCTCTGTACTAATGGAAGAAGGAACTTGTTG

AAGTGAAGCATTTGTCATCACTTAAATTCTCTGGTAGATGCAGATTTTTCGCTCTCCAGAATCTGAGGTATGTA
ATTGCTGTGTACT
>TCONS_00064567
ATTGTACTACTCTTGTCGGTTATAACTAGTGCTGTGTGTTCTTCGAGAATATTCATGCTTTTGACTTTGTGTCAT
CCTCTATCTTCTGGTATTACCGTTAAGCTCGGTTTGCCTTATGATCTGCCTATAGTTTAAAGAAATAAATAAGC
AGGGAACTTGTTCTGTGTGTGTCGTTACTTTATTCTTTTCCATTTTCCTCCAAAGTATGGACCGCAGGTTCGTTA
GTTTTTGGGTTATTATCGTATATGCTTACTGATTCTTCTGCCATTATATTTGAAGCGCGTCTGAGAATGTGTCAA
CATTCTAATCTTTTTGATGCAGTATGGACAGCAGATGATGATTGGTCACCCTCGGCAAGTTGTCTATATGCCGA
ATTACCCCCCTGAAATGCCGTTTAAGGGAAGAGAGTATTAACCAGTTTGCTAACAATATCGCGTGGTGGGTTG
ACCCAATTTGCTGATATAGAAAATAATTTCTGACGCTAGAGGTTGTGGATCATTTATTGGTGAACGAGGTTCA
GCTGGTTGATCTCCAAATGGTTTTGATTAGAGCTCTTCCTTGTAGTGGAATTTCACTGATTCGAATTGTCATTAT
CACAGATTTCTACTTATTGGTGGAAAAATAAACCAAAAAAGATGTGAAGAAGAGTCGAGTTACACTTAGTGT
>TCONS_00064568
ATTGTACTACTCTTGTCGGTTATAACTAGTGCTGTGTGTTCTTCGAGAATATTCATGCTTTTGACTTTGTGTCAT
CCTCTATCTTCTGGTATTACCGTTAAGCTCGGTTTGCCTTATGATCTGCCTATAGTTTAAAGAAATAAATAAGC
AGGGAACTTGTTCTGTGTGTGTCGTTACTTTATTCTTTTCCATTTTCCTCCAAAGTATGGACCGCAGGTTCGTTA
GTTTTTGGGTTATTATCGTATATGCTTACTGATTCTTCTGCCATTATATTTGAAGCGCGTCTGAGAATGTGTCAA
CATTCTAATCTTTTTGATGCAGTATGGACAGCAGATGATGATTGGTCACCCTCGGCAAGTTGTCTATATGCCGA
ATTACCCCCCTGAAATGCCGTTTAAGGGAAGAGAGTATTAACCAGTTTGCTAACAATATCGCGTGGTGGGTTG
ACCCAATTTGCTGATATAGAAAATAATTTCTGACGCTAGAGGTTGTGGATCATTTATTGGTGAACGAGGTTCA
GCTGGTTGATCTCCAAATGGTTTTGATTAGAGCTCTTCCTTGTAGTGGAATTTCACTGATTCGAATTGTCATTAT
CACAGATTTGTAAGCTTTCAAATTTACAGTCTCTCATGGAGTTTATTTACTGAGCTACTTATTGGTGGAAAAAT
AAACCAAAAAAGATGTGAAGAAGAGTCGAGTTACACTTAGTGT
>TCONS_00064760
ACTATCTGGTTGGGAAGAGGTCCAAATTGTTGAAAGCCTACAGATTTGGCATTTCTTTGTGCAATGTTCCAGA
TCAGGTATCAGAAGGCTGATTTTCTAAGCTTCCTCAGAAAAGCAGTCGATATGAAAACTCTGAGTAGAATCC
ACACTTAGGTCCTCTTTTGAGACAGACTTTGACAAGAATTTTCCAGCACAAAAATTCCGGTAAGGGCGATAC
ACAGGAGGCAGTAGCATTGCAGACTCTTTGTCCATTGATAGTAGCCGGCTACACTTGCTGCATACTTTTGAGA
ACAAAGTTTGGTAGCTGCAGACCCAGAGAAGCAAAGAAAACAAGGCTGTCTCGATATCGACACCTGTAAAA
TGCTGTAAAGCCATAGCAGCATGCTCCGTGATGTGCCTAAATACATGATGAACTGAAAATCCCCTCGCATGT
ACATAGCTACCACCCTCGTCTGGCGAGAAGAACGCTAGAGCATCAGGATCCAATGAACCAGCAGGGGAAAC
TGATACTACGGCTCTAAAAACAGAAGGTATCAACAATTCGATTATAGCAACCTTGTCTGGAACAACATCATC
AGATGATCCTTGTCTTAACGTTGTCGTGCTTTGGAAAGTATTATCTTTCGATAATTCAATAGAATTTTCACTGGT
CGAAGAGGGCAATGAGGTAGACCGCTTTAACCAATCCAAACGCTGGTAGGAGAAAGTTGTTACATTTGGAA
CCTCCATTTTCAGATGTGTCAGAACTTCAGACACTGTTTTCGGTTCACAAGTTTCTTCTTGATCACTTGATGGA
GGACCACAAAGTTTCTTTGTAGCACCCTCCTCACCAGCATTGCTATCAAGGTGGGGGAAAAATCGTCGTTTCT
GATCATTAAAGGCCTTCAGGGCTAACCTAGTCCTATCAACTGCAGATGCACCAGCCTGACCAGCAAGTTGTCT
CTTCCATGCATATGCAACAACAGCACCATCGGGACAAACCAAGGGCATGTGGAGGCCCCACGAATTACTTCG
TAATCGAAGGGAATCATTGAGGACACCAGAATCTTCCAACTGCCTTCCAACAGAGCGAAGGTCTTGTAGGTG
TTGTCTCATAGAAGCTTCTTCTTTGAGGATAAGATGCAAAGGTTTAGAAGAGTGTTGTGGCTGAGCCGCCACA
AATAGGGCTTCCAGAAGCCGATCTGCGCCAAGTCTGATATCAGCTATGAGTCTACTCGCCTGCCCCAGCCGCT
CCAGTGCCATTGCCACCTGCTTTGGCGGCGCATCTCCATTCGCCTGGCTGGACTCTGCCCCTGAAGCTCCTGGT

CCCGCCGCCGTGTGGGGTTGTGGTGTTGGTTGCTGCATTTTTGTATGTCTGAACCTTGGGAGGGTTTAATGTTG
GCGATTATAGCCCCCGTGGATGAGGTGTTCTTATGAATATCCCCGTGTACATGTAGAGAATGCTTCTTTATCAA
AG
>TCONS_00064763
GGAAAGTTTGATGGCTTTTTTATTTTCTTTAAGCATTGGACACCAAGAAGACATGCAAATTATAACCTATTAGT
TTGTTTCTACTTGGTATCCTACATATTGTGGTGATTCACAGTCCCCTACTTTCTCCTCCCAATGAGAATGTAGGC
TACGATAGCCCTACATCTTGGACACCAAGAAGACATGCAAACGATAACCTATTAGTTTGTTTCTCCTTGGTAT
CCTACATCTTGTGGTGATTCATAGTCCCCTACTTTCTCCTCCTGATGAGAATGTAGGCTACGATAACCCTACAT
CTCTATTTAGTTACTTTTACAGAAGGTACTAATCGTAAACTATGATCATTTAAACTTGTTGCTTCTCTTTTGGTC
CCTACCCCAGAGTGCCGTGCACATTATCTCACCCCTGTCATGTTGCTTATGAGTGGCGGTTAAGTGGATGGAC
AACACATCTAGGATTTGTTTATTCGAGTGCTGAGGTGGAAATTAGGAACTTTTTGATAGACTAAAAACTATAA
GCCAACTTGTACTAGATGGGAAGAGTTCAGTTGTTCCTTATTGTCATATCCTTGCTGGGTGTTGTTTTCTTGAAT
GTTCGTTTGCTTAGAATAGCTTGTGGATTCTCTTTATTCTGTTTAGAGGCGAATTTATTTGTATTTGGTTACCC
>TCONS_00064851
AGCTGGGTTAGACCGTCGTGAGACAGGTTAGTTTTACCCTACTGATGACAGTGTCGCAATAGTAATTCAACCT
AGTACGAGAGGAACCGTTGATTCGCACAATTGGTCATCGCGCTTGGTTGAAAAGCCAGTGGCGCGAAGCTAC
CGTGCGCTGGATTATGACTGAACGCCTCTAAGTCAGAATCCGGGCTAGAAGCGACGCATGCGCCCGCCGTCC
GCTTGCCGACCCGCAGTAGGGGCCTCTGGCCCCCAAGGGCACGTGTCGTTGGCTAAGTCATCGCGGCGGAAG
AGCCGCGGTGGCCGCCTTGAAGTACAATTTCCATCGAGCGGCGGGTAGAATCCTTTGCAGACGTCTTAAATA
CGCGACGGGGTATTGTAAGTGGCAGAGTGGCCTTGCTGCCACGATCCACTGAGATTCAGCCCTTTGTCGC
>TCONS_00064905
CTTGTACCCTATGACTCGAATTGATGGATTATCTTGTGTTATTGTTAGATTAACAAAATACTACAGGATGACAT
AGGAGATGGTGTTTGAGGCTCGGGTGCCATAGTTGTAGCTTGAGAAAGATGAGGAGAGCCAAAATTGCAGC
AACCGCAGCTATGACAAAGAGTCCCCAGAAGCTAGCTAGGCCAAGACTGTTGGAGGAAAGTGAAGTGCTAG
AATCTGAACAAGTAGATTCCCCAAACCACGCTTTCTCTATTTGTACCATCTTTTCACTTTCTGTCACACTTAAA
ACTGCTCTTGAAACATCAGGTACTAGCGGAGATCCCATTGGGAAGACCTAGATGAAAATCACAAGAATGATG
AAATC
>TCONS_00064911
CTTTGAGAAAGAATCAGACCCCGCGGTTCACGAATGACTTCCATGCCTAGGAATAAATGCATAAGAGTTGAA
GAACTTGGCTATACCTGCACAGCTGAGGTTTGCACTTGCATCAGAATTGCTTGTTCCCTGTTGTCCTGCCTTCA
TTTGTTGAAGAAGTTGCAAAAGTTGAGCCACATTATCTTGGGTAAGAATCTGATTTTCTGCTGCATTAGCTCCT
GTATACTCCAT
>TCONS_00065200
CACTCCAAATTTGCAATCCTGCTTATTTTACTCGCCATCTCAAAGGGCTTTACACCTTTCAATAACGGTAGCAA
AGCCAAAACAATACCCAACAACATCTCAATCCAAACAGCAAAAACGTCACAAACTGATAAGAGATCGAATC
ATGTCTAGTGTCTGCAAAACCCTAGCCGATGAAGAAACAAATGCGCTGGAAATGGACAGAATCAAAAGCAA
AAGTGAA
>TCONS_00065210
GAGAGAAAGATATGAAGAGTACAAAAAGGACATGACAATGGCCATGAACTCTGGCTGTGAACTGAAAAAA
TTCCGCTTGCAATGAAGTTATAAAGAAGTACAAGAAGTTGCTCTATGGTGCTCCAGAGTTTGAACAAAGTGTG
AGAAAGACCGAAGACATCTTTAACGAGGCCCTTGCATTATATCATGTAACATATGATAATGCCAGGATCACA
TACGGCATCGAAAAATGTGGGTTTGCCTGGAAAGTAGCTGGTTCTGCTCTCTGCAGGATCCACGCCATGTATC

AGAAGGAAAATGCCTTTCCTATTTTGCCATCTATTTTACAGGACATATTGTAGAGCTGTTGTAACTCTGTAGGA
TACTAACATTGAAGTTAACAAATACTAGCATTGTTGCTAGTGTATTATGGAATTTCAAATTGTACAGGCTCGTT
AGCGATCGGGCATTTCGAAATGTGTAATATCATCAAGCTGCTTCTAATCTATATGTTAGATAGTTATGTATAA
ATTCTCCTATTCCCATATGTAGGAGTAGAATATG
>TCONS_00065272
GGTTTCACAGACATGGTATAAGAAAATAATCTGTGAGGAAAATAGAGAGTGAGCGATATTGTAGTGAGGTG
GGAATATCAAAAGAGGGGTTATTTCTTTTGAGTGTTGTAGTGGTCTTTGGAGTATTTTACTCGGACCTACAAAG
TGTAAAATTCCTTACTATAGTGATATCAGTTGCTCCTCTCGGGGCCGTGGTTTTTCCCTTATTCAAAAGGGTTT
TCCACGTAAAAATCTTGGTGTCATTGCTACTCTTTTATTCTTGTTAATTACTGTATCTCGGTGCTACGTTATTATT
CCGCTTTTAGTACCGTGAATATTATTT
>TCONS_00065288
TAATTAACAAGAATAAAAGAGTAGCAATGACACCAAGATTTTTACGTGGAAAACCCTTTTGAATAAGGGAA
AAAACCACGGCCCCGAGAGGAGCAACTGATATCACTATAGTAAGGAATTTTACACTTTGTAGGTCCGAGTAA
AATACTCCAAAGACCACTACAACACTCAAAAGAAATAACCCCTCTTTTGATATTCCCACCTCACTACAATATC
GCTCACTCTCTATTTTCCTCACAGATTATTTTCTTATACCATGTCTGTGAAACCTCACTCT
>TCONS_00065289
GAGATACAGTAATTAACAAGAATAAAAGAGTAGCAATGACACCAAGATTTTTACGTGGAAAACCCTTTTGAA
TAAGGGAAAAAACCACGGCCCCGAGAGGAGCAACTGATATCACTATAGTAAGGAATTTTACACTTTGTAGGT
CCGAGTAAAATACTCCAAAGACCACTACAACACTCAAAAGAAATAACCCTCTTTTGATATTCCCACCTCACT
ACAATATCGCTCACTCTCTATTTTCCTCACAGATTATTTTCTTATACCATGTCTGTGAAACCTCACTCT
>TCONS_00065387
AGAAGATAGGAGGACGGCTGAAACTGGATGTCTATATTTGGCCAAATAGAAGATTGAATTTGCAGAAGATGT
GGTTGTAGCTTAACTGGTACGAACTGCATCAGATTTTCAAACCAAAGTGGAGAATTTGGCAACCAGAATGGT
GGAGAACAGTGAGCACCGAAGAGCAAAAAGAATTCGCACTTCAAATGGGGAAATGTTTAGATTGAGCAATG
ATGGAGGCCTACTGAGTGCGACACTGGAGGAACTTCAAGAATTTGGGGATGATATAAAGAGTCTCCGTGACT
AAACGGTGGCTTAATTTACTGATGCTGTAAAGAAGTGCTTTTGTAGGTGCTTAGTAATGTAAAAAATTTTCTAT
GTGAGATATTTTTTTGTTTTAAAATGCTTAAAGTTTGAGAGATAATAGTAACCACTTCACTAGGATATGAGCA
AGGCAAGAAGAAAAGATTGGTGATATCAAAATCCGTATCAGTGATGACCTGTCAATACTTAGATGGTTTGCA
TGTTTGTGACAGACTCAATGAAAAGGCATAAATTTTAGCTTCTGCTCTTATGGGGCCACAATCT
>TCONS_00065396
GTCACTTGTAGTTATTTATCATTCAATGAATGACTGATAAAGGATCCATTGATATTAATCTAATCCAATTAGAA
TGCTTGGTACTTTGTAGTTGTACATAAGCAAAGTATTGAAAATCATATTTACTCTTTCTATTTCTAACCATCGG
GGAGATTCATCCTATATTATTCCTAGATTATTCCAGCAAATAGCAGAATCGTGGCTAGGGAACTATACTAGCG
ACCTACCCAATTTATTGTAGAAATTTTCGCGATCAATGATTGGACCATGCAAACTAGAAATGCTTTTTCTTGGC
TAAAGAAACAGATTACTCGATCTATTTCCGTATTGCTCATGATATATATCTTAACTCGGACATCCATTTCAAGT
GCATATCCCATTTTTGCACAGCAGGGTTATGAAAATCCACGAGAAGCGACTGGGCGTATTGTATGTGCCAATT
GCCATTTAGCTAATAAGCCCGTGGAGATTGAGGTTCCACAAGCGGTACTTCCTGT
>TCONS_00065398
CACTTGTAGTTATTTATCATTCAATGAATGACTGATAAAGGATCCATTGATATTAATCTAATCCAATTAGAATG
CTTGGTACTTTGTAGTTGTACATAAGCAAAGTATTGAAAATCATATTTACTCTTTCTATTTCTAACCATCGGGG
AGATTCATCCTATATTATTCCTAGATTATTCCAGCAAATAGCAGAATCGTGGCTAGGGAACTATACTAGCGAC
CTACCCAATTTATTGTAGAAATTTTCGCGATCAATGATTGGACCATGCAAACTAGAAATGCTTTTTCTTGGCTA

AAGAAACAGATTATCGATCTATTTCCGTATTGCTCATGATATATATCTTAACTCGGACATCCATTTCAAGTGCA
TATCCCATTTTTGCACAGCAGGGTTATGAAAATCCACGAGAAGCGACTGGGCGTATTGTATGTGCCAATTGCC
ATTTAGCTAATAAGCCCGTGGAGATTGAGGTTCCACAAGCGGTACTTCCTGT
>TCONS_00065410
GGAACCTCAATCTCCACGGGCTTATTAGCTAAATGGCAATTGGCACATACAATACGCCCAGTCGCTTCTCGTG
GATTTTCATAACCCTGCTGTGCAAAAATGGGATATGCACTTGAAATGGATGTCCGAGTTAAGATATATATCAT
GAGCAATACGGAAATAGATCGAGTAATCTGTTTCTTTAGCCAAGAAAAAGCATTTCTAGTTTGCATGGTCCAA
TCATTGATCGCGAAAATTTCTACAATAAATTGGGTAGGTCGCTAGTATAGTTCCCTAGCCACGATTCTGCTATT
TGCTGGAATAATCTAGGAATAATATAGGATGAATCTCCCCGATGGTTAGAAATAGAAAGAGTAAATATGATT
TTCAATACTTTGCTTATGTACAACTACAAAGTACCAAGCATTCTAATTGGATTAGATTAATATCAATGGATCCT
TTATCAGTCATTCATTGAATGATAAATAACTACAAGTGACAGAGACAGACGATTTAAAT
>TCONS_00065415
GCAGAGGTCCAACATGTCAAGATGTACAAGCAGGTCTTCGAGGAGGTAGTTGTTGATGAGCTGATCTTTGCA
ACTTGGGACGCAATGTCTCAATAACAACTGCTGATTTTTATCAACTCCTGCACCTTGCTTGCATAGAGCCCA
GTCTGCGGAATCAAAGAAGGCACGTTCATGGTCCTTTGAAATCAAAGGCTTCTTCTTTGGCATAAGTCCTCCA
TATTTTTTACCAGTGGCAGTAGTCTCGTGATCTTGAGAGGAGACAGAAAACTCTTCAATACTGCTGCTATCTG
CCATCTTAGTATGTCACAACCACCAGTCTAGCTCTCCCCTTGAAGCAGAGGAGATAACAAGACCAAGCAAAG
GCAGAAATATGGTGAATAGGCTAA
>TCONS_00065419
GATCTATTCAAATGACAATTGCGTGCTCAACATCTTTAAAAGAGTTGCTTAACAACATTTCAAATAATCTAAA
ATAAGTTCACATCTGGCAGCTCTCACTCATTCATAACAGGCTTGTTATCACTTGCTTTTGGCCTCTGTTCCTTGG
GCCTCTGCTCTTCAGTTTTCCTTTTGTCGGAGGCGCCTCCTTTCTGGCTGAGGAAGGAGCGGAAAAGCGGAGA
ATTCACGTCGTAAATCATCGTCGTTGGTCAGAGGCAAATGATGGAGTACACAAAACCCTAATCTCCTGTTTGC
TC
>TCONS_00065433
AGGAGGATAGTACTGTTGTAGTTGAGAAGCTTCTCAATGAGGTCAACATGTGTCATCAGCATTCTACGACAGC
AATATCGAACCAAATTCAGTGCATCAAGTGCATCCCCTTCGGAGTAATCAGCTTGGAGAAGATCAAGATATT
CATCCCATTTGTTTCCAATGACCTTGCCGCAAGTGAAACAGCGCACCGGAATGATCATCCTGCCAAAA
>TCONS_00065451
GCCAGGTCTAGTCACTGTGAGGTAATTTAATTTACCAACTAACCGCCTATATCTTGCAGGATCGCTAAGAGGC
TCCCCCTGTCCTGGCAGAAGTTTAGAATTCGGATCCATCGGAGTGTCAACAGGTCTACAACCTGTCATTCCTG
TTTCCTCAAGAATATCTAAGGCATACTTCCGTTGTGAGATCACAATACCTGAGCTAGACTGAGCGACCTCAAT
ACCCT
>TCONS_00065548
AATAAATCGAAAAAAGAAAAAGGAAAAAACGGGGAGATATTGGGGTCGGTTAATGTAGGTGTGAAGCAAA
CAAAAAGCGCGTTGAATGGGAAAGCAAAAGCCAAAAGGAGGAAAAAAGAGCAATGATTAAGGGAGTGAG
AGTATCAGGACGCTCATTTGCCAAATTTGAAAAAGCTTTATCCAAAATCCATCAGCACAGATCATGGAACTC
AACGTGAAGATAATAATACTACAATTACTCTCTTTGACGGCCGTTTTGTTCATCGGCGCAACAGTCAGAGCTG
GAGAAGTAGAATACGTCAGACCTCCACCTCGTAAAGCCTTTCGTTTGCCATGGGATCCAAAGCCTTCCTCTCA
ACCTCAGCAGGAGGACCTAAAATCTTTCCATGGACATTA
>TCONS_00065695

CCAATTTATAATGAGCATATCGCGAACGCTCTCTGCAGTCGCATAGTATGCCTTGGGAAGTTCAAACTTCTCA
GGAGAGAATGATGGCGTGAACTCTGCGTGATACTTTATACTTGATAAAACAGATGTGGAGTCTGGCTGGAAA
ACATCCAATGTGAATCCTTCATCAGAGGAAGAATCCTTCATCTTCTGTTTTTGATCACTAGCCA
>TCONS_00065818
CCCAGATCTTTCTTACTGAGGTTCTCGATTTGTGATACAAATTCCAGATCTAAATTACAGTTGAACACCAGGAT
AAAACACTAAAACTCTGAGAAACAATGTTGGATGCCTAAGAGTACAATAATTTACTATGTACAATCTAATTTT
GGCAGATAGTACAACAGATGAAACTCAATTATCCATCTCACTTCAAGAAGGATTTCTCCATCACCGTTCAGC
AGTCACCACTGTGTTGTCACCGTCTGGAGTGATGATAGCAGCAGCAGCCATGGCGTCCGAGCCATTAGGTTC
ACCATTGCTCTGTTCACCAGATTTTGCAGTTTGATCCTGAGGTCCAGGAGTTGGAACTTTAGAAAGATCTCTTA
AAATTCCCTTATTGGCCCACTTGAGTCCACATGCATTACAAAGAGACCTTGGCCCAGCTGGTCCACGACGCAT
CATAGGAGTGGATTTCGAACTAATACCGCAATGCCTACATGATGTTTCCTGCTCTTCTTGACCAGAGCCTTCAT
TCCAATCTGCAGATGACGAACCTGCTTCGTCAGATATTGACTTTGAAGATGTAAACTGACCCTTCTTACGCTG
CATCCTCATCGCAACTTCCTTCCGTACAGTATAACGGATCTTTTTATCGAAACACCGTTCTTTCCTCTTTTCCCT
AAAACGATTCAAAGAAGCAGCTCTTTGTGGTTGATTCAATCTTCCAGGAAAGTCAGCTGAAGCCCTCTGACTT
TGGGCTGCCACACTAACAGTAGGGATTCCAGCAGGGACTTCATATCCCCCCAACAACAACAGCACCGCCTGA
ACCTTTTCTGGAGAAACAGCATCAAAAACATAAACTTCGCCTTGAAACGACAGCGTAAGCTGATCGGAAGCT
CCACTACCAGCAGCAACAGCAGCTTGAACTATTTCGGTATTAGGGGGACAATACAACGCGTTGTGTGAAACG
CCTTCCACACCATTCATCTCCATGGACTCCCCGCCGTTGTGAAGCGCGTGAGAATGACTATGAGACTGGTGGT
GATGGTCGTAGCGAATGTTAGGGTTGTTATCCATAGACTCTCTTCCTACATCGCCGCCGCCGCCGCCGCCTTC
AGTTCCAGCGGCGACGTCGTCATCGTCGTCGTCGTCGATCTGAGTTTGCTGGTGGTGAAGTGCAGCGTTAATT
GTCTCCCGTCCGTACATGTTAGCTCTGCGATTTGATTCTGCCATTTTCTGACGTCCTTTTATTTGTTTTTCTAAA
AT
>TCONS_00065949
CAGAAATATGAAGAAACAAACTATATCGAGTTCCATAATGTATACAATGAAGATTTCTGTAGCAAACAATCA
AAACTGTTCACTTATTTAAATTTAGAGGCAAAATTACATTCAGTACAAACAAAATGTACTTGCTGCAGACTCG
CTAAATCTATTTAGCACCAGTAGCTCCTCTATATATCCTCAGGTCCATTGATATCCTTGTTTGCAATCACAGCA
CCATCTGGAATTTCTAGC
>TCONS_00065966
GCTAGAAATTCCAGATGGTGCTGTGATTGCAAACAAGGATATCAATGGACCTGAGGATATATAGAGGAGCTA
CTGGTGCTAAATAGATTTAGCGAGTCTGCAGCAAGTACATTTTGTTTGTACTGAATGTAATTTTGCCTCTAAAT
TTAAATAAGTGAACAGTTTTGATTGTTTGCTACAGAAATCTTCATTGTATACATTATGGAACTCGATATAGTTT
GTTTCTTCATATTTCTGTAGCCATATT
>TCONS_00066178
CTGGTAAGCAACGATATTCCATAATGTGATGCACTTGAAGAAGCGTAATATGAAGTCACTGTAAGTTGAGTA
AACTTTGGAAGTGCACATCCTCCAAATGCTGCACTAGCTGAAGAGAACATGAGAAGTGGAGGAAGCCCTAT
AACAAATGGAAGCCACTTTGGTCCCTTCACATGTCTCTGGACGAAAGGGATACCTGTATTGAAACCATGAATT
GCACTGAGCAGTCCACCAAGTCCAGAGCCAGCCACAGCCGTGACATCGTGCAGTACAGGAGTAATCGCCCA
TTCTCCACTTTTCGAAGTGGGGTTCAAGAGAGAAAGACAACGAGGGCATCGAGCAGAGCCGAGGCCAGGTTT
GATTGTCGGATCATACTCGAGATTTCCTCTCTTCAGCTTTTCTTCGTATACCTCTCGTGTTCCCATATTCC
>TCONS_00066242
CGGTCTTAACGCCCCTACTACTACTGTGCAGCCTTTCCTCGGGTTCGTAGAGTCGGGTTTCCCGTTTACCCACA
ACGGAAGAGCCGCCCCCACCAGGCAGGCGGCCACGGGTCATAACGCACTCTTTGCACAACAAATCCACTTTG

AAGTTGACTTATTCGCTCGGCCAATCGTCGGAATGTGTACGAGATACCATAAGGGCCCAATATCTCAATAGC
ACCTTTGTCTAAAGCTTCGAAGGAGACTTCATATCCGAAACGCAGGAACGATCTGACTAGAAAGTCATTCAA
AAGGGTCATAACGCACTCTTTGCACAACAAATCCACTTTGAAGTTGACTTATTCGCTCGGCCAATCGTCGGAA
TGTGTACGAGATACCATAAGGGCCCAATATCTCAATAGCACCTTTGTCTAAAGCTTCGAAGGAGACTTCATAT
CCGAAACGCAGGAACGATCTGACTAGAAAGTCATTCAAAACTTGATCGAAAAACCAACGTTTATTGAAGAA
GCTATAGAGTCGATTACTAATAGTACTAATTTGAAAGGCTTGTTGGAATTGATCTGCTACGGGATTAACATTA
TACGCAACATAAGCACCTGAAGTACTAAACGGAATAGGTATTAGTTTGGTAATGGTTGGAGCAGCAAACTCG
GATTCGGCAAGAATCTCATTTTTTGGTAGTACGAAGGGGGAATTGGCCCAAAAATTGGATGGGGGTCAAGAC
GCTGTCGGGCGCCGGTGGCTAACTCAAGTGTCCCGCGAACTGCGCGAAATGGTCGCCTATTACACGGCTCAC
TAACTCTGCCTGGGGTGTGGATACCTATTATTCGTCGGGTAATTTGTCCGCGTATTCCCAGACAATAGAAAAG
AGTCTTTCTTTCTCCTCTCTTGAGGAGAATTTAAAGGGAATGTGATCCCCTCTATTGTAATGAAATAATTGAAG
CTCATTTATTCTTTGAAGCAGAGCTTTAGCAAAACCCACTTTTGCCTCATATAAGGCCAAGGGGGAAATAAAG
TTGTCAAACATAGCATCGGAAAGGGTCATCTTTCGCCCGATATTAAAGAGGTATCTTAACTCTTCTTTAATATG
GGAGCGTAGACATTCCGGTACAACTTGATTATCCGGTAGATCTAGCGCCATTTGCCATTCATCAGGCTTGGGA
GAGGAGGTAGACGCTCCCCCACCCGAATCCTTTCCTGCGCAGTCTACCGTTTCTAACAACAATTCATCAAAGA
AAAAAGAGAAGGAAAGCAAAAGAGACAGAGTCCAAACGCTTTCCATAATAGGTACACGACAAATGTCTGG
ATTATTTTTATAGTAGAAATGCCATAAAGTGCGACCCAAGATCCATGATACGAAAACCAAAAGCAAGATAA
GTAAGCACACGAAATCCTGATGTATTTCTAACTCCAACTCGAACAATAAATAATTATACAATAGAAAACACC
ATCTAAAGAGCTTTATCTCTATCATAATCGCTAAAATAGTTGAAAAAACGATCGCTGAAAATTCAAGTAAAA
AGGGAAGCGTTTTTGAGAACATAGGAGAGGAATTCAAATTAGGAGAAATTCAAATGTGAA
>TCONS_00066243
CGGTCTTAACGCCCCTACTACTACTGTGCAGCCTTTCCTCGGGTTCGTAGAGTCGGGTTTCCCGTTTACCCACA
ACGGAAGAGCCGCCCCCACCAGGCAGGCGGCCACGGGTCATAACGCACTCTTTGCACAACAAATCCACTTTG
AAGTTGACTTATTCGCTCGGCCAATCGTCGGAATGTGTACGAGATACCATAAGGGCCCAATATCTCAATAGC
ACCTTTGTCTAAAGCTTCGAAGGAGACTTCATATCCGAAACGCAGGAACGATCTGACTAGAAAGTCATTCAA
AACTTGATCGAAAAACCAACGTTTATTGAAGAAGCTATAGAGTCGATTACTAATAGTACTAATTTGAAAGGC
TTGTTGGAATTGATCTGCTACGGGATTAACATTATACGCAACATAAGCACCTGAAGTACTAAACGGAATAGG
TATTAGTTTGGTAATGGTTGGAGCAGCAAACTCGGATTCGGCAAGAATCTCATTTTTTGGTAGTACGAAGGGG
GAATTGGCCCAAAAATTGGATGGGGGTCAAGACGCTGTCGGGCGCCGGTGGCTAACTCAAGTGTCCCGCGAA
CTGCGCGAAATGGTCGCCTATTACACGGCTCACTAACTCTGCCTGGGGTGTGGATACCTATTATTCGTCGGGT
AATTTGTCCGCGTATTCCCAGACAATAGAAAAGAGTCTTTCTTTCTCCTCTCTTGAGGAGAATTTAAAGGGAA
TGTGATCCCCTCTATTGTAATGAAATAATTGAAGCTCATTTATTCTTTGAAGCAGAGCTTTAGCAAAACCCACT
TTTGCCTCATATAAGGCCAAGGGGGAAATAAAGTTGTCAAACATAGCATCGGAAAGGGTCATCTTTCGCCCG
ATATTAAAGAGGTATCTTAACTCTTCTTTAATATGGGAGCGTAGACATTCCGGTACAACTTGATTATCCGGTA
GATCTAGCGCCATTTGCCATTCATCAGGCTTGGGAGAGGAGGTAGACGCTCCCCCACCCGAATCCTTTCCTGC
GCAGTCTACCGTTTCTAACAACAATTCATCAAAGAAAAAAGAGAAGGAAAGCAAAAGAGACAGAGTCCAA
ACGCTTTCCATAATAGGTACACGACAAATGTCTGGATTATTTTTATAGTAGAAATGCCATAAAGTGCGACCCA
AGATCCATGATACGAAAACCAAAAGCAAGATAAGTAAGCACACGAAATCCTGATGTATTTCTAACTCCAACT
CGAACAATAAATAATTATACAATAGAAAACACCATCTAAAGAGCTTTATCTCTATCATAATCGCTAAAATAG
TTGAAAAAACGATCGCTGAAAATTCAAGTAAAAAGGGAAGCGTTTTTGAGAACATAGGAGAGGAATTCAAA
TTAGGAGAAATTCAAATGTGAA
>TCONS_00066245

CGGTCTTAACGCCCCTACTACTACTGTGCAGCCTTTCCTCGGGTTCGTAGAGTCGGGTTTCCCGTTTACCCACA
ACGGAAGAGCCGCCCCCACCAGGCAGGCGGCCACGGGTCATAACGCACTCTTTGCACAACAAATCCACTTTG
AAGTTGACTTATTCGCTCGGCCAATCGTCGGAATGTGTACGAGATACCATAAGGGCCCAATATCTCAATAGC
ACCTTTGTCTAAAGCTTCGAAGGAGACTTCATATCCGAAACGCAGGAACGATCTGACTAGAAAGTCATTCAA
AACTTGATCGAAAAACCAACGTTTATTGAAGAAGCTATAGAGTCGATTACTAATAGTACTAATTTGAAAGGC
TTGGCCCAATATCTCAATAGCACCTTTGTCTAAAGCTTCGAAGGAGACTTCATATCCGAAACGCAGGAACGA
TCTGACTAGAAAGTCATTCAAAACTTGATCGAAAAACCAACGTTTATTGAAGAAGCTATAGAGTCGATTACT
AATAGTACTAATTTGAAAGGCTTGTTGGAATTGATCTGCTACGGGATTAACATTATACGCAACATAAGCACCT
GAAGTACTAAACGGAATAGGTATTAGTTTGGTAATGGTTGGAGCAGCAAACTCGGATTCGGCAAGAATCTCA
TTTTTTGGTAGTACGAAGGGGGAATTGGCCCAAAAATTGGATGGGGGTCAAGACGCTGTCGGGCGCCGGTGG
CTAACTCAAGTGTCCCGCGAACTGCGCGAAATGGTCGCCTATTACACGGCTCACTAACTCTGCCTGGGGTGTG
GATACCTATTATTCGTCGGGTAATTTGTCCGCGTATTCCCAGACAATAGAAAAGAGTCTTTCTTTCTCCTCTCTT
GAGGAGAATTTAAAGGGAATGTGATCCCCTCTATTGTAATGAAATAATTGAAGCTCATTTATTCTTTGAAGCA
GAGCTTTAGCAAAACCCACTTTTGCCTCATATAAGGCCAAGGGGGAAATAAAGTTGTCAAACATAGCATCGG
AAAGGGTCATCTTTCGCCCGATATTAAAGAGGTATCTTAACTCTTCTTTAATATGGGAGCGTAGACATTCCGG
TACAACTTGATTATCCGGTAGATCTAGCGCCATTTGCCATTCATCAGGCTTGGGAGAGGAGGTAGACGCTCCC
CCACCCGAATCCTTTCCTGCGCAGTCTACCGTTTCTAACAACAATTCATCAAAGAAAAAAGAGAAGGAAAGC
AAAAGAGACAGAGTCCAAACGCTTTCCATAATAGGTACACGACAAATGTCTGGATTATTTTTATAGTAGAAA
TGCCATAAAGTGCGACCCAAGATCCATGATACGAAAACCAAAAGCAAGATAAGTAAGCACACGAAATCCTG
ATGTATTTCTAACTCCAACTCGAACAATAAATAATTATACAATAGAAAACACCATCTAAAGAGCTTTATCTCT
ATCATAATCGCTAAAATAGTTGAAAAAACGATCGCTGAAAATTCAAGTAAAAAGGAAGCGTTTTTGAGAA
CATAGGAGAGGAATTCAAATTAGGAGAAATTCAAATGTGAA
>TCONS_00066300
CTAAAAGTACCAAAATTCTAGGGGTTTTTTCTCGATTAAGCTCGACTACAACTCTTTGTTCTCACTGAGCCTTA
ATTCTAGATTCCATTAGCGAGGATGAAAATGCAACTGATAGCTGTGGGATAAATAATTGTAAGTTTTCTTAAA
CTCTTTTTAATATAAACCGTTGTGATTTTGGATGAAAATTTCAGTTTTAATGTTCCTTTTTACTCGATTTAGTGAT
TTTTATTTTATTTTTGGGAGTAATTGAGTGATGTAGTGTGATTGAATGATGATTCAAATCCATATGACACAAGC
A
>TCONS_00066301
AAAATTCTAGGGGTTTTTTCTCGATTAAGCTCGACTACAACTCTTTGTTCTCACTGAGCCTTAATTCTAGTTGAG
TCGTAACTTGCAGATTCCATTAGCGAGGATGAAAATGCAACTGATAGCTGTGGGATAAATAATTGTAAGTTTT
CTTAAACTCTTTTTAATATAAACCGTTGTGATTTTGGATGAAAATTTCAGTTTTAATGTTCCTTTTTACTCGATTT
AGTGATTTTTATTTTATTTTTGGGAGTAATTGAGTGATGTAGTGTGATTGAATGATGATTCAAATCCATATGAC
ACAAGCA
>TCONS_00066305
CCCTCTGAAGTCCAACGGAGGACACCAAGCCCAGCCATGAGCCTTCTGATCGTCCCATTGACCCGTCCATAA
CCATCAAGTGATGCAGAAATCTAGCCTCAAAGACGACACGCCTCATTTGGAATCATGTGAATATGGAACATA
ACACGTTTTGAGCACATCCAAGTCTAATGTCGTCATCAACTCTTTGAATTGTCATCTAAATTGTCCATTCTTGT
GCGGGTTACTTGCAGTCCCAAGTCATGATGGATATGTTAGTAAGTGGTTGGCTTTATTTAAATGTTTATAAGCT
ACCTTCTGAATGATGACTTTCCTAATTATTCGTTGCTATCGGTAATGCTTTAGAAAGTAGAAAAATTTGGTTTA
AATACGTCCCACCTGTTGAGGGCAATATTCTAGTTAAGCTATGTCCGATTTTGTTTAGGGCAATAAATTGCTGG
CTAATTTGGCTGATTTGGTTACCCAATTTGTCAACTTTCCTACACTAAAATGTTGTTAAGTTAGTTAAAACGTA

TAAGCAACTTGTAATGTGTTAGTGACAAACGTCACTTGGGTAAGAAACAAGAATGTTCAGCTTAATTGGCAC
ATGTCAATTTACACTTCATAAAATGATGTATTTTGAAAATCAAAGTATGGAGTTCACCAAATATGTTTTTCTA
GTTCTGAATCTTTTGGTTATGTGTACTGATAACCTCTAA
>TCONS_00066308
GTAAGGGTGAGAGAAGAACAAAGATTGCTCCACTAGCATTGAAGAACCATCAAGTGATGCAGAAATCTAGC
CTCAAAGACGACACGCCTCATTTGGAATCATGTGAATATGGAACATAACACGTTTTGAGCACATCCAAGTCT
AATGTCGTCATCAACTCTTTGAATTGTCATCTAAATTGTCCATTCTTGTGCGGGTTACTTGCAGTCCCAAGTCA
TGATGGATATGTTAGTAAGTGGTTGGCTTTATTTAAATGTTTATAAGCTACCTTCTGAATGATGACTTTCCTAAT
TATTCGTTGCTATCGGTAATGCTTTAGAAAGTAGAAAAATTTGGTTTAAATACGTCCCACCTGTTGAGGGCAA
TATTCTAGTTAAGCTATGTCCGATTTTGTTTAGGGCAATAAATTGCTGGCTAATTTGGCTGATTTGGTTACCCA
ATTTGTCAACTTTCCTACACTAAAATGTTGTTAAGTTAGTTAAAACGTATAAGCAACTTGTAATGTGTTAGTGA
CAAACGTCACTTGGGTAAGAAACAAGAATGTTCAGCTTAATTGGCACATGTCAATTTACACTTCATAAAATG
ATGTATTTTGAAAATCAAAGTATGGAGTTCACCAAATATGTTTTTCTAGTTCTGAATCTTTTGGTTATGTGTAC
TGATAACCTCTAA
>TCONS_00066344
CCCAATTAAAAAACACCAATTGTTCCTCCACCAATCCATGATATATGACCCTAAATTTTTGTTACTCCAACAT
CTCTCCGAGCAATTCCACACTTCTTTTGTTAATGTCACCACCTCTTAACAGTGTTGTTAATCTGGGGTACAAGG
TTGGATAATCCAGGCTACCAGGCATATCCATTTGGTGAAAGACACCTTGTGCACCGTTAGGAATTTCTGGCTC
CAAAGTCACCTTTCCTTTTTTATTATAAGTGTTGTCATTGTTTTCCCTTTCTTTTCCAGGATCTGCTCTTTTAGAA
CCCTTGCTTGTCTGGGCCGCTCTACTGTAAGGCATTAGCTCTCTAGCTGCAAGACTATTTCCTTGACATACTTC
ATTAACACCTTGGATAGCAAGGTTAGCACAACTGCCTGATCTAAGATTAGCCTCAAAAGTGACATTAACAGT
TGCACACTGTTTTAAGGCTTCTTTAATGGCACCAACAGCGATGCTGTAACTTTCCTGAGTCAGTGATCCCTCTT
CACCCAATATTATTGCCCGTCGGCACAAATCATTGTAGCGACGGACTTTAGCTTGCACATCTTCCAGTCTTTCA
CTTATGGAATGCCTGCTCATGGCAGCATTGGTCCAGCGTTGCAATATATACTTAGACGGAATATTGAAGACAC
CAGACATTTGAAGAACCACAATAGCATGTCTACAAAGGTAACCTTTGTACTCAAATGAATGGCATGAGCAGT
ATATGTCCGACCTCGGCGCATCCCACTCCACCATGAAGTCCTGATTTGCATCAAAATCTTTGACAGCATATGT
TATAGATGTGCCGTCTTCTGATTCTTTTTTTAGATGGCATGCAGCGGCTCCTAATACTTCAACTTGGAACTTCTT
GAATATTTCATGAGTATATATGAGCAACATCTGTTTTTCGAAGGGCGAGGGAGACTTAAGCTCAGGCATCTCA
TGCCATGCATCAAAATTAGCTTTGGCTTCCTCTTCATACCAGTCTTCAAGGATTAGCTTATGTTGTCCAATAAA
GTCTCTAAGAGACGTTTCACTCTGGATATATTTGTCAAAGAAAGAGTTCATGCTTTCGGATCTTGAAGCCGTG
GATAAACTGGCAAAAGATACATCCCTCATAAAAGCAGGCACCCAAAGCTTGCGATTTTCATATAATGATTGA
ACCCACTCGTACTCTCTAAGATTGAACTTTTCAATCAACTTCCACCATCTGTGTTCAAATTGTTCTTCAGTCCAT
GATCTATATATGCACTTAGTAAATTTTGCCATAAATGTATCATGCCACAGACTGAGATATTCAAGATGCCTTG
GGATCTTCTCCAATATACTCCACAGACTAAAGTAGTGCCCGGTGTCCGGAAAGATTGCTCCAACAGCTGCTTT
GATGTTGTCGTTTTGATCAGACAACAAAATTCTTGGACCTCGTCCACCCATTGCCAGACACCATGTCCGCATC
AACCATATGAACGTATGCACTGTCTCATCAGCAATTAGTGCACAACCTAGTAAGGTGGGTTGAATGTGATGGT
TTGCTCCAATGAAGAGAACCAAAGGTATTTTGTATTTGTTTGTGAAGTATGTAGTATCAAATGACACCACGTC
ACCAAAAATTGGAATAATTATCCATGCCTTTGGCGTCAACCCAGAAAACATTCCTCAATCGATGCTCCTCATTT
AAGTCCACGGAATAGAAAAATTTTGGATTCTCTTCCTGCATTTGCACAAGAAATTCAAGCAAGACCTGCGCA
CCTCCTTCTTCTAAAGTCAAACTTCGACCTCTGTCATGTTGATTCCTGAAAAGATTTTCCAGATTACCAGAGAA
TTGGTATGCACCGTACTGTTTTGATACAGAAGCCAACATCTTTTTCCTTCGAACTTTTGCGTCATTCTTAAGTGG
ATCAGCATTCCTGTGGCTTCTGAAAAAGTGCACTTGAGCTGGTAAAAGCTCATGGTTATGTTCTTTTACGAAA

CTATGAATATACCACTTCCCATTCGACGACCTTCTCTTCACGTGCATACTAGCTTTACAACCTATCTTAGGAGA
AGGCCTAGGGTTGATTGCATCATCAGACTGCTGTTTGTTTCCATATCTTATGCATGAAAACTTTGCATCAATAA
ACTCTTTGGATGCCCTAGAACGACGACTACTCAATTTGGCGGTGCCAAAACCCGCTGATTTTGCATATTCTTTA
TAGAACTCATAGGCTGCATCATGTGAATCAAATTCCATATCATCTCGAGGCT
>TCONS_00066357
CATAAAAGCAAGTAATTTTGTTACTTAATGATGTAATATCATTTTGGTGTGACGTGACGGCCCGTTTCCAATTT
CCATCAACCCAATCAAGATGGAACCTCAGGTCACGCTGAGAAAACTCGAGCTCCTCATCCGGATCCTTGAAA
GCGACCCGGAACTTGCAACCTATACTACCAGTACCAGTACCAACAACACGAACAACAACTCCAGTCCACCA
CCCTGCAAGATGAAATGCATCAACAACGTCGGTCACGCGAAAACACAGGCGCTCATCCTCCATCCAATTTGG
CGGGGCAGGCCGTAAAATGGACCGTTTTCTAATACGAATCACTGTTGTTCGAAGCCGGAGTTGTGGACTTTGG
TGGTGCGATTATAGTGGCTTCATACCAAGCTCCACTGTACCCTTTTTCTTCTGTGCTGACTTCGACTTCAGAAC
CAATGCTGAAGAAGGGTTCAATAAATGAATTACTAGTAGGTGACGATACATCGTTTTCCAATTTTACTACTTTT
TCCTCAATTGTCGGCATAGTTGCAGTGATGAAATGCTATAACAAAGGCTAGGGTTTTGTGAATTGTGAGTAGA
ATTGGAGTTTTTAGGTATTGGCGTGGAATTGAGCTGCTATTAACGCTAG
>TCONS_00066393
TCAATAGTTGTTTGATTTAGTGCCTTGTATCTCCATCTTTCTTTCTCAACAAAATCAGCATCCAAGAATCTAGCT
ATAAAACTCCACAACCTATACAAATCTTCAAAATTTGTCATCATCCCAAGTGAAACAGTTATAACTGATACA
CCACAATTCAATTTCCCCTTCTTATTATCTCTCACTTCACCACTTCTTGTTTCCAAGATTGTCTTTTTTTCATCAT
CATACATGTTTGAAAACCATATATGCTGCAAAAATGCACAAGAAAGCGATATATTGTTGCGATCCGCTAGCTT
CTGTACTAGTGTTGGATCAATCTTTTGACCTTTCCAATCAAACACATTGAATGCCACAGCTGGTCCTCTGTTGA
AACTTATCTTTGGTCCATAGATTTTCACTAAAGAAGGATGAATATCTCCAGCATGAGGATGTTGAAGGCGCGA
TAACGCGTTTATCAGCCAGTTTACAAGAAAGGCTCATTGAGGGAAGCAGATTGTTGAGTTGGAGGAGGAGGA
GCTGATGACGAAGACGCGATTGAATCATCTATTGAATGATATCCCTTTTGTTGGTAGTAAGAAAAAAGACCAT
TGCCAATATAATCAAGACAAACATGTTTTGATATAGAAAGATGATAGTATTCTTGTGCTCGGATTTCATCAGC
TAGATTAGTTTGGTTGTAGTGTGGATATGCTTGTTTTAAGCTTGTAAACAATTCTAAACATGAAGGCAAACACT
CATGGTTAGTGAATTGAGAATTTGGTTGGATAGAAGAAGCCATTGTATCGATGAATTCGCGATGACAAGTGA
TGATTGTGCTGATTTTAGAGTAGTGATTTTGGTTTCCATGGGAACTTTGGCAGCAACAATTGAAACAACCTTCT
CTTGTCATTCTAAGACAAGGTGATTGCATTCTTGATTTAGTATCTTTTGGTCTTTTTGCTGGTTTTTTGGTTGCTG
CAGCAAGTGAAAGTGTAAACTAAATTGAGTTATTTAGCCTAGTCTAGAGGGCAAAAGTTAGCACTTTACAAA
CATGATTGACCATTTCAATCTCAACTTACAATGCCCTCTCAAATGTATTTTCTCATAGGACATTTAGTACTATA
TTGAAATTTAG
>TCONS_00066394
TCAATAGTTGTTTGATTTAGTGCCTTGTATCTCCATCTTTCTTTCTCAACAAAATCAGCATCCAAGAATCTAGCT
ATAAAACTCCACAACCTATACAAATCTTCAAAATTTGTCATCATCCCAAGTGAAACAGTTATAACTGATACA
CCACAATTCAATTTCCCCTTCTTATTATCTCTCACTTCACCACTTCTTGTTTCCAAGATTGTCTTTTTTTCATCAT
CATACATGTTTGAAAACCATATATGCTGCAAAAATGCACAAGAAAGCGATATATTGTTGCGATCCGCTAGCTT
CTGTACTAGTGTTGGATCAATCTTTTGACCTTTCCAATCAAACACATTGAATGCCACAGCTGGTCCTCTGTTGA
AACTTATCTTTGGTCCATAGATTTTCACTAAAGAAGGATGAATATCTCCAGCATGAGGATGTTGAAGGCGCGA
TAACGCGTTTATCAGCCAGTTTACAAGCAACTGAGTACTTAAACTTACTGACTTGTATGATATGTTAAAGAAA
GGCTCATTGAGGGAAGCAGATTGTTGAGTTGGAGGAGGAGGAGCTGATGACGAAGACGCGATTGAATCATC
TATTGAATGATATCCCTTTTGTTGGTAGTAAGAAAAAAGACCATTGCCAATATAATCAAGACAAACATGTTTT
GATATAGAAAGATGATAGTATTCTTGTGCTCGGATTTCATCAGCTAGATTAGTTTGGTTGTAGTGTGGATATGC

TTGTTTTAAGCTTGTAAACAATTCTAAACATGAAGGCAAACACTCATGGTTAGTGAATTGAGAATTTGGTTGG
ATAGAAGAAGCCATTGTATCGATGAATTCGCGATGACAAGTGATGATTGTGCTGATTTTAGAGTAGTGATTTT
GGTTTCCATGGGAACTTTGGCAGCAACAATTGAAACAACCTTCTCTTGTCATTCTAAGACAAGGTGATTGCAT
TCTTGATTTAGTATCTTTTGGTCTTTTTGCTGGTTTTTTGGTTGCTGCAGCAAGTGAAAGTGTAAACTAAATTGA
GTTATTTAGCCTAGTCTAGAGGGCAAAAGTTAGCACTTTACAAACATGATTGACCATTTCAATCTCAACTTAC
AATGCCCTCTCAAATGTATTTTCTCATAGGACATTTAGTACTATATTGAAATTTAG
>TCONS_00066449
CACATCACATAAAGATTCCAGCCCCAAACTCTCCTCAGTTATCTGTCTGTACTTCTTAGTCACTTTTCCAATCC
TTTTCATATCTGCAAAAGCTGTCTTCACATCTGCAAAGGCCCGGGTACCACCTCTCCACCCACATTTAGCTCG
AAAGCATGTCCATGTAGCTGCATGCCCATCCTGAGTAATGAAGAGAGATAAGCTCTTTTCATTTGAATCCCCA
CCTGTGCACATTGGACAAAGCAAACCAGAGTATTGCCCTGGTCCACAAGAACCAATATCTATTCCAATCTGG
CTTAATTTCTCCTTCAGTGCCTTCACATGTTCTGACTCTGCAATTCCCTCCACTGGGTCTTCCAGAACTACTCCA
GAAACTGGTGGGGGAATACGGTGGGGCCTGTAAGACAAGTTTGAAGTTCCACGAATTGGGGAGATGGGTTTT
GAAGCAAATGTTGAAAAGATGAGTCTTTGGTTTTGAAAAGGGTGGGTTTTTGAAGAAAGGACAGGAATAATA
GTCTTGTGTATGGTAATGGATGGCTTGTGGAGAAAATATTTGGAGCCCATGGCAAAGTTATTAGAGTTGTTTA
GTACAAGTCTGCGATAAGGTAGAAGAATCATCCTGCTATTTTACAATGTAGAATTTGCAGTCATGGTGTGTGA
GTGTTGTGTTTTTGCAAGAAACTTCGGAATGCGTGGGGTTAAGGAAGGGTTCTAT
>TCONS_00066613
ATAATCGTGTTAATACCTGTCAGTGTATGTGTTCAATGCTTCGTGATCAAAACCTTCCTTCCCACTGACAAACA
TCTCAGCTGTAACAGAATTAGCATGAAACTCAACCATTCTTTTGAAGTCCCTTTCGCTCCTTAAATTAATAAGA
GTTTTCCTATTTCCTGGAAGAAAGTATTTAATTGACACTGTTTTAAGCTCCAGATTGCACATCTCTGCCAATTTT
ATCTTCAGATGATCAAATGGAGTATCTTGGTTGATATTTACAGCATTTGCCTCTCCTCCATTATATGACAAGGT
TCCATCGGCATCACTCGCAAATTCACCACCAGACTGGCAAATTAAAATAAGCTTTCCCTTCACCATGGCATAA
ATCAATAATCACCATTCACCAAATGAAAGATTGGTTAATCAGTGTATGCACAAACAGCA
>TCONS_00066656
AAGCCATCCCTTTTCTAATACCTTGATGGAAGCCTAGTTCATGTTTTGGAAACATTGATAACCACGTGAGCGA
CGTCCAGAGAAAAAAATGTTTAGGGGATACTGCTGCAAATTAACTAAAAACTCTTTCTTTGAGACAGCCCAA
CTCTAACCTTGGTTGCCTTGTGTGTCCCTTGAGGCCTTTGCAGCACTCTCGCGTAGTTCGTCCGGAAGATCACA
GTTCATCATGACTCTGAGGTTGTTCCTTGTTCATCATCTCTAGTTTATCTTGGAGCTCCTTCAGCTTTTCTTCGAG
ACAAAGAGCTGGGGTAGACAAAAATGCCATATATTCGCAGCGGCTGGGCTCATCTACATCAGTCAACTCATT
TTTCAGGCCACATCTTAATTTGACCTTTAAACTTCTATCTGGCCCATTCCAGCATTTGTCCCCGCTTGAGAATTG
CAAGGTTCTATAGGAATCCTCGAATTTATCCCAATTCCCCAAACGAGTTGTACTGTAGCCCTCGACCTGGGTT
GCTTCTTTGAAAGGACAAA
>TCONS_00066658
GCTTCTTCAACCTCCTTCTTTCTAAGAGTGACGCCTTCCTGATATGTAGCAATCTTTTTATTCAGTTTTTCTCTAG
CTACTTTGCCAGCCTCCCAGCACGTATTTGGGCACTTGGTTTTGCCATCATACTCGTCACTCCCATCACAGCAA
TCACAAATGCGATCATTAACCCTTGACGAGTATATTGAAAGAGGAATATGTCCTGCATTTTTGCAATAAAATT
TCCATTGGGGCATGCTGAGGTCCCT
>TCONS_00066817
CAAGAACAAAGTGACAAAAACTACCTCACGATTGACGCCCAAATAATTGACTACAAGAAGCACTTGCAGGA
CTTCAGGTCACAAGGCAGCCACCACCTTCAGCTGGCGTCTGCCACCGTCGAGGCAAAACTTTGTCGTGGGAT
GGGCAAGGATAAGCATTTCCACTCTAATCGTCCACCTCTTAAATAAACTAAAGAGTTGGTAGAATACACAAA

TCACTCCACCCAAGCACAAATCCAAATCAAGGGAACAAATAAAGAAGAAGCTCAATTCCCATACAGCAAAT
GTTCAAATCTTCCTATAAGTACTTTCTTTCTTGCAACTTGAAAAGAGAAGAACACTAAAGTACAGAAAGTCTG
GCTGCACTAGGCATGGATGTAGAGGCAAACATTGCTACCGGACCCATCAGTTTGTCTGCATTGCTTCTGAATG
ATACTTCATCAGATCAGCATCTAGATCTTCTGCAGATACCTTTTCTCCACGATCTCCTCTTCCACGTCCTCTGCT
GCCTCCTCGAAATCCGCGACCGCGTCCTCTACCACCCCG
>TCONS_00066824
GATGAACTGTACAAAGGGATCATCAATGCTTGACACACAATTTTAGATACAAAATGCCTAAAAAGGTTAGTA
GGACAATAAGATGAAGGATAAGCTCACCACTCATAATAAATTCTTCAGCAAGACCATAAGAGAGCCAGTGA
TTGAGCTCACCAACCCATCAACGTTCTTCTGACTTTGTCTACAATCTGATTTCTACCAACCATACATGACATGT
CAAATCTACAATTTTCGTCCAGAAGGAATCTGTAAACCTCCCACACGCGATCACTTGTACAATGCCTTCCAAT
TTCCGCCTGCAAAAAGATATTAAAGGTCATCCACCGTGTGAAGAGGGATGAAGTGATGTAGTGAACCATCCG
GAGCTATAAAATTAAAAAACATGGGCATGATCACCAGGTCTATAAATGGGAGGTTAGTCACTTAAAGAGAT
AACGCCTCCATTCAATTTTCTTTACAACTTTGTCCTTTAAGCAAAGGTGCATACACACACAATCCATCACTTTG
AAACATTCCTTCACCACTAGATACTTACCGAGAAGATGCAGATCCCTAGAGTCTTCATTGCCAGTGTAACGTC
ATAGAAGACACGTGGTCTGCCCTTTCCAGATAGCTCCACAGGATTAGCAACCAAAAGTTCAGTATCTGGCCC
ACGATTTGTGATGATAACACGTAGAGGATGAAGCATCTCCGCTTTTAAGCGGGAACAAAGAACATCTTGTTT
ATCTGGATCCACAATCTTTTTCCCATCCTTCTGAATATTGCAGTCTTTCAATGTGCGCATAATGTCATAAAGAA
AACCCTTGTGATCAACACAACCAATCTGAAGTAATGTATGCGCGGGGCTCATCGAATTATCTATTGTCACACT
AGCCCTCTTCAAATTCATCATATCTGGGCTAAGAACCTGTGTGCGAAATTCATTATCTGATAGTTCGCACCTA
AATAGTTCCTCAGCAACTGATGGAGAAAGAGAAGAGACACATTGCAGGTTGTCATACTGTGGGCCTGCTAAC
TGAAGCTCACAAC
>TCONS_00066825
AATTTACGGATGAACTGTACAAAGGGATCATCAATGCTTGACACACAATTTTAGATACAAAATGCCTAAAAA
GGTTAGTAGGACAATAAGATGAAGGATAAGCTCACCACTCATAATAAATTCTTCAGCAAGACCATAAGAGA
GCCAGTGATTGAGCTCACCAACCCATCAACGTTCTTCTGACTTTGTCTACAATCTGATTTCTACCAACCATACA
TGACATGTCAAATCTACAATTTTCGTCCAGAAGGAATCTGTAAACCTCCCACACGCGATCACTTGTACAATGC
CTTCCAATTTCCGCCTGCAAAAAGATATTAAAGGTCATCCACCGTGTGAAGAGGGATGAAGTGATGTAGTGA
ACCATCCGGAGCTATAAAATTAAAAAACATGGGCATGATCACCAGGTCTATAAATGGGAGGTTAGTCACTTA
AAGAGATAACGCCTCCATTCAATTTTCTTTACAACTTTGTCCTTTAAGCAAAGGTGCATACACACACAATCCA
TCACTTTGAAACATTCCTTCACCACTAGATACTTACCGAGAAGATGCAGATCCCTAGAGTCTTCATTGCCAGT
GTAACGTCATAGAAGACACGTGGTCTGCCCTTTCCAGATAGCTCCACAGGATTAGCAACCAAAAGTTCAGTA
TCTGGCCCACGATTTGTGATGATAACACGTAGAGGATGAAGCATCTCCGCTTTTAAGCGGGAACAAAGAACA
TCTTGTTTATCTGGATCCACAATCTTTTTCCCATCCTTCTGCCGAATAAAAAGGTCTAATTCCCTCTGACCCTTA
TTAACTGAATATTGCAGTCTTTCAATGTGCGCATAATGTCATAAAGAAAACCCTTGTGATCAACACAACCAAT
CTGAAGTAATGTATGCGCGGGGCTCATCGAATTATCTATTGTCACACTAGCCCTCTTCAAATTCATCATATCTG
GGCTAAGAACCTGTGTGCGAAATTCATTATCTGATAGTTCGCACCTAAATAGTTCCTCAGCAACTGATGGAGA
AAGAGAAGAGACACATTGCAGGTTGTCATACTGTGGGCCTGCTAACTGAAGCTCACAAC
>TCONS_00066828
TTTCTATTCAATGCATTCTAATCATAATTCATGTGTGGTAAAACAGTGGTCAAGCAAGAGGTGGTGGCTTCGG
GAGGTCACGGGGTGGTAGAGGACGCGGTCGCGGATTTCGAGGAGGCAGCAGAGGACGTGGAAGAGGAGAT
CGTGGAGAAAAGGTATCTGCAGAAGATCTAGATGCTGATCTGATGAAGTATCATTCAGAAGCAATGCAGACA
AACTGATGGGTCCGGTAGCAATGTTTGCCTCTACATCCATGCCTAGTGCAGCCAGACTTTCTGTACTTTAGTGT

TCTTCTCTTTTCAAGTTGCAAGAAAGAAAGTACTTATAGGAAGATTTGAACATTTGCTGTATGGGAATTGAGC
TTCTTCTTTATTTGTTCCCTTGATTTGGATTTGTGCTTGGGTGGAGTGATTTGTGTATTCTACCAACTCTTTAGTTT
ATTTAAGAGGTGGACGATTAGAGTGGAAATGCTTATCCTTGCCCATCCCACGACAAAGTTTTGCCTCGACGGT
GGCAGACGCCAGCTGAAGGTGGTGGCTGCCTTGTGACCTGAAGTCCTGCAAGTGCTTCTTGTAGTCAATTATT
TGGGCGTCAATCGTGAGGTAGTTTTTGTCACTTTGTTCTTGTCCACTGGTTTCACAATTGCAGTCCTTGTGCACT
TTGCAGTTTTGCTGTATGTGTCAGACTTAATTTTATCCATGTTGATAATGAGTTCTACAAACTGTTGACTTAGGA
AGAACGTAAGTGGGATTAATTTTGGGCGACTAAAGCTGCCTCCCCTGACTAGAACTGCCTCCCCTGATGTACT
ATTGCAAATATATATATTTCTACCTTTTATGACATGTTTATAACGAGCTTGAAAAACTGTTGCATTAGGAACAG
GTAGGTCGGAACATAATTGGGTGACTAAGGTGAATCCTTTGAATGATTTTGTCTTATTGCAAATACACGGGAT
GAGTAAGCTG
>TCONS_00066893
GCTCTCTGATTACAGAATGGACATAATAGAAGTTAAAATAGATTAAAAGCTCAGGAATAAATGAAATGAAT
AGAAAATACGCTACTAATCTATGAGTTTTTAACCAAAATCATCCTTCTTGTTTCACTTCAACTTCCACTGCATC
CTTATAATATTCTACTTAACCAAAAAAGCATCCTTTAAGTTTACCACAACTAACCTAAAACATCCCTCCATTA
ATATCTCCATCCACAGTTGAAGGGTCCTTTATTCTTA
>TCONS_00066995
ATTTTTTTCCTCTCTATTTTTGAAATTTATTTGCATAATTGAGAGAGAAAAACACTGGCTGTTTCAGTAAGTTCA
GCTCTTTTTCTCTTCCTTTCCATTTCTTCTTGAACTTCTAACCTTACTCCAAATAGCATTGGCCTCTCATCTTGTT
GCTTTTTTTCTTCTTTCTATAGGTATTGAGCTGCCGGACAAAGCTGGAGAAGTTGCTATGTTTAAAAAGAGTTG
GAAGTAAATCTCTAGCAAATTCTGCCGGCTGCCACACAACAAACGCCGTCCCATCGGAATTCCAAGAAACGA
CGTCGTCCGTCGCCGGATCCTCCACTAACATGTACGTTTTTAGCAAGAACGGCGACGGCGATGACTTCCTTAC
ATACTC
>TCONS_00066996
GGTGTCTGTTTGTCCACGCTTTTCTACGACGAATATCACATAGTAAATCCCTTTCTCCCTTTCTAAACTTGTCAT
TACTGAACTCCCACCGGCTTGTTGCTATTTTACGGAAACCATAGGTATTGAGCTGCCGGACAAAGCTGGAGA
AGTTGCTATGTTTAAAAAGAGTTGGAAGTAAATCTCTAGCAAATTCTGCCGGCTGCCACACAACAAACGCCG
TCCCATCGGAATTCCAAGAAACGACGTCGTCCGTCGCCGGATCCTCCACTAACATGTACGTTTTTAGCAAGAA
CGGCGACGGCGATGACTTCCTTACATACTC
>TCONS_00067031
CGGGGCAGGTTGAAAAAGAGAAACACTGTTAAACAGGGCAGTGCATGTTGAATTTGGGTCATGGCAATGCTT
CACCTGCCCCGCCCTGCCCTGTCCTATTGCCATCCCTAGCCCCTATGAGAAAGCTTCAGACTTCTGAAGGCTA
TAAATAATTCACATAGAAAAGAAGGAAGAAACTATCTGGGCAGGAAACCATGGCGAGAAATGGCAATCTCA
AACGCTTGCATTCTTTTTCCTCGAGCAAAACCATTGCAATCATAAGATGAAATCTCATTGATGTTGAGATCAA
CACAAGTCTGCACAAGTTTCTCTCCGACACGTTCCGCTGCTTCTACAGTGCTGCATGGTGGATCACCCCGGAT
TGATTTTTGCAAAGTGCTCCCGTAAAACAAAGACTTCTTATTTTGGTCATCTACCAATGTAGCATATAACCGTA
TTTCTGAGCAGAAGACTGAAAGTCTTGGTTTTTCAGGAGTTCCA
>TCONS_00067038
TAATAGTAGCACTCAACTTCTTTACAAAGAAGTAAGGATCTTCAAGCTACTTTTCTTATGTTGGGCTTATATCC
AGCCCAAACCCAATATACCCCTAGTCGAGTCCATAAATTGACCGCTTCTCCATTCTCATTATCATCTGCACAG
ATAGATAAAAACCATGGCATGCAAGAAGGTGAATTGCAGACAACCTGTTTGATTAAATTCCCGAAAGAGCG
GATGGTTACTCCAGTGAGCCGTGCTACCTTTTAATCACGCAAATGTCCATTTTTGAGAAGTGGGTCTGCTGTAA
TTTCTATTAGCAAGTGAAAATATTTGGGAAACTAACGGTAAGAATGACAACTATTGCTCTGGGTAATATTTGT

TCGCTACAAATGAAGACTTGTGAGCTCTTTGGAATTTGTGTAAATCACTCATATACCCTTCCTATCCAAAATA
AAAGCTTAGAAATAAAGCCATTGGTTATTCAAGCCACAGCTAAAGCTAATTCTCGAACTGAGAGTGCTAAAC
TTCGAAATAGACGAATTAGAAAAAAAGTACGTATGTTGTAAAATTATG
>TCONS_00067046
TAATAGTAGCACTCAACTTCTTTACAAAGAAGTAAGGATCTTCAAGCTACTTTTCTTATGTTGGGCTTATATCC
AGCCCAAACCCAATATACCCCTAGTCGAGTCCATAAATTGACCGCTTCTCCATTCTCATTATCATCTGCACAG
ATAGATAAAAACCATGGCATGCAAGAAGGTGAATTGCAGACAACCTGTTTGATTAAATTCCCGAAAGAGCG
GATGGTTACTCCAGTGAGCCGTGCTACCTTTTAATCACGCAAATGTCCATTTTTGAGAAGTGGGTCTGCTGTAA
TTTCTATTAGCAAGTGAAAATATTTGGGAAACTAACGGTAAGAATGACAACTATTGCTCTGGGTAATATTTGT
TCGCTACAAATGAAGACTTGTGAGCTCTTTGGAATTTGTGTAAATCACTCATATACCCTTCCTATCCAAAATA
AAAGCTTAGAAATAAAGCCATTGGTTATTCAAGCCACAGCTAAAGCTAATTCTCGAACTGAGAG
>TCONS_00067073
GGTTAAACGTGTACCCAAAATTAGATCATAAACAAGGATCAAGCTTCTTTCCTTGTTATCCTGAAACAGTATA
TCCCGAGGGCTGATGTTATTTCATGAATAACAGACGTGGCAAAATGCAGATAGAGTGACACTGAATATGCAG
TGTAAGCAAGAAGAACCCAGAACTCATCAACCATAGGAACTCCATCGTTAAGTCTGGCGGTGAGCGCATTTG
CAACGGCAAAAGGCAGACAAAGCAGAGACATGCACATATTTGTTTTCAGACCCTTGGGTTCATCACACAAGT
GAGCCAAAATCATCCTTCCCACAAGAAATCCGAATGCAAGCCCAGTCCCCACGACCATAAGATGAGGATAG
TTCTTCATTAAGTCGGATGGAGATAAATAGTCCCATAGAAGCACTCCTCCCAGTAGCACAACAAATGGGTAA
AGCATGGCCAGTGCCCGTGGCATACTTCCTTTTGTTGCCTGAACAACCTTATGAACATTGTTAACATTGCAATA
GACCGTTGGAATAACAGCAAAAGCTACCATCAGCAACAACACGGCTTTATTGGTTGGAATTTCGTTCAGAAA
AGGAACCCAACTCACAAGTGGCATAGACTTCCCAAACTGTTGGGCCCACCACTCAGCTCCCACTAGAGCTGT
AAAGAAATGGCTGAGATATATCAACATAAGACCCTCTGTCGGTCCATTTACTACCGGAAGAATTAGAGTGTT
GGTGAAATAGCTTTCCCAAGTAGCGCAGTAGAAAGGAACAGCTGATATAACCCAGAACCAGAAAGTATCTC
GTCCACACATAGCAGTGCTCCCAAAAGCCAAGGCTTCAAATGCACATGCAAGCGCATCACATCCATGATCAA
AAAGCTCTCCTAGTGGACTAGAGGAATTTGTACGTCTTGCTTGCTTCCCATCAACAGCATCAAACGTCTGATA
GAGGAAGAGAAGCAATCCATGAGCAAAATTAACCCATCTTGGAGGAGGTGAATCCAATTGCGGAGAATATA
TATATCCGAGCAGTGCAGATGTGATTAAGAACAAAAATCCTGTAAGTGTAATCATATTCGGTGGCATCCAAA
GGGGGAAGAAATTGACACAGCGACTCCAAAATGGCTGCAACACATACTTAGCAACATAGGAGTGATCTACC
CCACTGTATTTGTACCTATGAAGCGCTTGCACTCCATGTGCCCCTATGTAACCCATCCTTTCTTTCCTTCCTCTC
GCTCCCTCTGGAATCACCTATAAACCAATTGGAATCTGCACAAAAAT
>TCONS_00067198
TTTCATGCTTTGGAATCTTTACTGAGTTGCAAGTGTGTTGGCCAAAGTATGTGGTCTGGTAAATGATTGAATTT
TCTTGAATTGTTTGGACTTGTTTTGTGGCCCTGCAATCTTGATCGTTCTTGTGTGTGCACCTAAAGTAACACCTT
GGATACTTTGAATTGAGTATATTCTTCTGCCCATATTTCCTCCATGCACAACCATCTTCCATTATTGCA
>TCONS_00067261
GTCAAAACAAGGCTTTGCGTAGTAAAGTTCGCATGGCTTCTTTTTTCCAATATTTCACCACCCTGTTGACATAT
TTCTAAAACTTGGTTCTGAATTTTATGATATCCCTCAGATGATTTATATGAATTAATGAGAAGATCTACCACTT
CTTCTGTCGGTTTTCTTCCAGCTAAACCATGTTCGATATCCTCATAAAAATCTTTGCTAATTGTTTTAAGAAAAT
GATCTGACGGATT
>TCONS_00067366
CCATGAAAAGATTGATCAAGAAACCTAAAATTTTGATGACTGTAGAGAAAAGGGTTGCCATTAATTTCTTGA
AAAGAAACACCAGATGATAATTGTGGATCCATGAAATTAACTCCTTTTTCTTTAGAATCAGAAACAAGAAGG

AAATCTGAAGGGATAAGTGAGGGATGATCAACAAAAGGAAGTAAACCAGAAGCAGAAACTCTAAGCCTAT
CTCTTCTGCTTTGTTGTGGGACATGATATGCTTCAAATCCCTCAGCCATATCTCACTGCAAAGTGAAGAGATG
GGATGAAATGAAAGGCTATTATTATTATATCTGCTGATAACCCTTTTTCAGTTCTGAGAAAGAAAAACTGAGG
ATTTAC
>TCONS_00067393
GAGGTGAAAGGGTTATTTGAACAAAATGAAACAATCCAACTTGGAAAACAAATGCAGCAATTCTGAATGAA
TGACCAAACCTCACAGTTATTTTCGGTAGAAGATGAACATATACAACTATGGTAAAGCAGTAAAGCTCCATG
AAGTGAAGTCCTCTCACCATCATTTGTAGGAATACAGTATGTCTCTCACTACTATCTCCTGTCAATGTTAAGGC
ACCTATCTGCATTCATACGAACCGTAATTCTTCAAACACTTCCTAGTTCTCTGTTTAAGTAATCAACAAACATC
CTTTTTGCACTATCAGCATCTGAAGGAGGGGGTGGCCCGCTCAAGCTTGTCTGTCCAGCAAGCTTGATGAAAG
TTCTTCGTAGCCTACGCAGCTCTGGCAAGCTAACAGATCTCGAGATATCTACAGTAGGTAATCCTGGACCAAG
AGATACACCATTGGGAGGTCCCCCAAGAGCATGGGACTGAGCTTCCACAATAGCATTTGTAACTTCTTGCGTT
GCCCTGTCCATCTCATATAAAGAATTTGCCTCAGAACCACGTGCACTTTGTGTTGCAATGGTTGGCTGTAGAA
TCTTAACATCCCTTGTTTTAGAGTCCACTTTTTTTGTTAGATAGCTAACAGCATCAACTATAGCTGTAGACAAT
TTCTCAATTCCATCTTTAGCTGGCCATAACTCAAATAATGGAGAATCCCATCTGTTTTTGCTATCTGGTGTTTCA
AATCTTCTTACAAGATCCTCAAATATATTATCATCATAAGATGGTTCTCCTCTCTCATGACGTTCTCCGTTCCAT
ATTCTACAAGATTCCTCATCAACATCACAATGGAGGACACAATATCTAATCCCTGCTGCTCTAGCCAAACACC
ACAATTCATATCTATATCCCTTGATGCTATTCAGAGAATCCACTATAACTATACTGTCCTTTGACAAAGATCTA
TCAACTTCAGACCTTAATACGCCTCTTAAGTTCTTTTCCTCGGTCATGTTCGCATAACTTTGGTTGCGACTAAG
ATGAAACGAAGTCTCATCAATAGTTCTCACTGACGGCTTTGATTCTGTTTCTTTAAGCGCTTCAGCTAAACAAG
CCGCAGCTGTTGATTTCCCGCTACATGGTTGACCACAGATAACAACCAATGCCATCTTATGGGTGTTGTCCAA
AAGCTTGTGAGAGTGGAGAAGAAAAGTTGGTTAAGTTAAAGAAGAGAAAGTAGAGAATGGGAGAGAGTTAT
AACTAGGGTTTATGGTGTTGCTTGCCGATGTT
>TCONS_00067422
AAGTTTTCAATTTTATCTTAATTTCATATTCTTGAGTTGAAAATAAGTATTATCGTTTATGCTGCAGGTTGGTTT
CTTTTGCTCTCAAAATTTCCTTTATCGTTTGGGAGTAGTTATCAAAACCCTCTTCCCCAAAGGCTATATTATCGT
TTATGCACAACTGTTTCAGTAGTTATCAAAACCCTCTTCCTCAAAGCTGCAGGAGAGTGATTCCCTTCCAGCA
GTAAAAGTGGACGGTGATGATCGTCAGCAAGCACGTGGTCTTGCCACTCTTTACTCTCATCCAGTTGTACATT
ATTTGCCGTTGACATAAATGAAGGTCGGTTGCGGATACTCAAAGAGACAGCCAAATTGCACCAAGTTATT
>TCONS_00067436
GATTCTTAGCTAGTTTCCAAAAAAAGATTGAGATTTAGTGAAAAGCCCTCTCATATTCTCCCTCAACATTGAT
AGAATTGATAGGCTTACAGGTTTCCATTAGAGCTAAATTTCCTGCACTCCTGTGAGCCTGATTGAATTGTTGAG
ATCTACGTCCATTTGATAAAATGAAGCTTGCTGTTGATCCTGTATTCTGCAAAAAAAGATTCTAATTTCTTGTG
CTGGCTAGTTCGCAAGTTGTACAGAAAAGAGACCAGAGGCAATAAACTTTCATGATACCATGGTGTCCTCTA
CCTAGTTTCAACAGTAACATCCAGTACCTTCGAGTTTGAGTAGAAGAAGATGAGTCAAAGACTGTCGGACTG
CTATTTGCCTCCACCAAGTGTTGTGAGATTTATAGTCCTTAGTGTGAAAATTATCCAACTAATGTGTTCTTTAGT
CAAGGATTTTCACTCTTGTTTCAAATCATTAAACTAGGTTGTTGATCTTATGATGTCTTATTCCTTGAATGATTT
TCGCCTTTTTATTTGTGTAATTTATGTTATATAATTGATGCTTGTTTATACGTACACACTACCCTCTCCAGACCC
CACTT
>TCONS_00067557
CTTGATATTTTGCAATCCATTACATGCAGCATCAATAGATGTGCGGTATAACTTAATGGTATGTACTTCTGTAA
TAGGTATGGATTCCTGCTGCTTTTTGTCAGCGAGAATCATTTTCTTATGTGACTTATAGAACCGCCTGACCTCT

GGGCTTACTGGATGATTGTGATGAAGCTCAACTTCCACAATTCTCCATCTTTTTGAGTCCACAAGCTTAATTAC
TAGCATTGCAGGACAACCAGTTCTTGTTTCTGGCCTAGGATGATTTGCTCCACTTTTCTTCTTGAAACCTGCAC
TACTGCAGCTGAGTTTCGCTCTATATCTTTCTTTCCTCTTTGATCTAAACCAAGAGTTGCTCACTCTAATACCAA
ATCCTTGTTCCTTGCCATACATGTTATAAAAATCATATGCTTCATCAAAAGACCCAAATTCCAGACCAATAGT
TGGTTGTGGGGACTCCTTTTTGGAATCTTTATCATGAACTCCATCATCATGTTCCTCCACGGAGCAGTCTTCTTC
GTCCTCATATTCTTCAGAGTCATCAAACACTGGGTCACTGTTAAGAGAAACTGCCTCCATCTGTTTCAGATTCT
TCATCATCCACTATGATGGAGTGCAAGATAGAAAAGCAGTGAAATTCTCATTGATTTTAATATTTGATTTGTC
ATTTTGGAATACTCAGAAGAGAGTGGCTACCATCTTACCCAGAGAAAACAGATCTCCATGTCATCCT
>TCONS_00067616
CACATGTAATTGATTCTTTGTATAGAAGTCCAGGTATCAGTGGTAATACATACTCTTTGTTTTGTTTCTATAAA
AAACCTCTTCAACTTTTGCTTTTCTTCATTAAAAAGATCAAAACAATCCCTAGTTACAGTACTACGAGAAGGG
ATCCGAAAATAAGGTTGCGCCACTTTCATAAAGTCTCTAAAACCTTCTTTCTCAACAAAGCTAAAAGGAAGCT
CATCAACTATTACCATACGACATAATGCCTTCCTACATTCTTCTTGTTCAAATTTCCAAGTAACAACAGCTACG
TCACCCTGACTACCCCCCGGAGCAGATTGAAAGCCTAAATTTGATTGTTTTTTATCAATAATAGCAGGGCGTT
TAGGACACTTAAGCATATGAGAAAGAAGTGTCGACGTACCGTCTTTGGTCTTAAATACATACTCAATAAAGC
AATAGTCACATTTTGCTTTTGTACTCCCTTCAGAAGTAATAATTTCTGTAAAATGATCCCACACAACAGACCTT
TTTCTCCTTTTTTTGGTTATATTTGACTCCGTTGTTTGAGCTTGACTTGTTGCATTTGAATTAGAAGCCCCACTTT
CACCCATCATCACCTTATCACTTTGTATATTTTCAGCCATGTATGAGTCACTGCAACAACAAAAGAAAAGGAA
TGAGAATAAAGACATCAAGTTGTAAACTAATCGTATTTAGTTATTAAAGTGGA
>TCONS_00067668
TTTAACTAGACATCAACATAAGCTATCATATCATTTCTAGATGAAATGAACACTACTATCTGTATATGATTTGA
TTCATTGGGAAAGAAACAAGAGACATGTTTCTTCACTTGGCAAGCATAGCTTGCTGAGCCTGGTTTTCTTGTTG
CTGCTGATAGTTCAGTGGCCTCCTGAAGAGCATGATGTGTGGCTCTGGTCGATGAATAGCATAGTGCACCCAT
CCTCTGCTCTGCTGAACTCCAATTGCACGCCACTCATTTTCAGAGAGAAGACGATTCTTCGGTAACAATTTCG
CTACATCAGGAGGAAGAACAACATGCCTGTACTCGTAGGTGTCATCGAAGTACTTCTCAGAATACTGGATCT
GCCCCATTTCTCAGTTCAAAA
>TCONS_00067759
AAATCCTCCAATAAGAAGAGAAAGAAACAAGTTTGATTGTTGTATCTTGATCCACGAAGAAAAAGCATCAGT
GCCATATCTTGATATACTGGTCATGACTCACTGAGGCTATGGTTCCGGTCGGAGGTGAATCTGCAAATGAAGA
AATTATACCATCATGAGCTTGGTGTGGCCAAGTTCTGTGGCTCTGGTACATTGGATTCCACAGCTCCAGTAAC
TCATGAGAACCAATCGCCAAGAGTTGAGCGCGGTCGGGGTGGAAAGTGCATGACTGGAACTTATTGCCACTG
GAACGCAGTTCATGTAAACACTTTCTTCCGCTAACACACCATATCCGTGCACTATCCTCGCTCACGGATGCCA
GGAAATTCCCGCTCATATCCCAACAGATAGATCTCTGACATCTCCGACATGTCCCTGTATATCAGAGAAAAG
GAGACCGCATTGATAGGATACAGTAATGCTGCAGATAAGAAATTTAAGACAAGCTTGATTTGGAATAAAAC
ATCTTACTTGTAACTGTTGTTGGATGCTGTTAGTCTCCACATCAAATATATTTATAATATTTCCTGTAGAACATG
CCAAAAGGTTCCCAAATCGAGGTTGGAATCTAACCTGTCTACTACCTCCCTTAAAAATGAGTTTGCAATCACC
CTCACTGACATCCCACAGTCTAATCTCGTCATTGCTATCACAAGAACTGAGAAGACCCACCTTTGTAGGATGA
AAGTCTAGGGACATCACATGATCAGCATGCCCCAAAAGTTTTTGGAAAGGGTTGCTTGGCTTGGCTGCATCCC
ATATCATCACAGTTCTGTCAAAAGAAGATGTTGCAAACACCGTTGAATTTGGTTTAAAACGGATATCTGTAAC
GAGATGAGCATGACCTTCTCCAGTGTTAACATCATTATTTTCCAAGTCCCAAATCACAACCTCATGTCCAGCA
GCAGCCAACAACTTCCCTTGTGAATGGAAGTGGCAACATAGGAGCTCGCTGTGTGTTGCGTGAAGACTTCCA
ATCTCTTTAATAGAGATGCCTTTCTGGTCAGATTCAATGCAAGCAGCAATACTTTTCCCCAATGCAGAGATCG

AATTAATTGCACCAATCAGACCAGCATCTCCCTGGGAGAAGTAGCTGTTAATATTGCCATTCGAAATACCATC
GGTAGTAGCTTTCTCTTTCCCAACAGCTGCCAGAGAACTCAGTGAAGCTTTCCTCTTTCTGCCCCTCTTATTTG
AAGACTGCAGTCTCAGCTGGTTCACTTGCTCCTGCACTGCAATTTGATTTCCTCCTGTAGGAGCTAATATTTGC
TGGTCAACTG
>TCONS_00067832
ACATAATGCCAGCAAATCGTTTAAACGTCCTAGGGATACGAACATGAGGTTTTACTTCAAATAGGACGCCTTT
CTCAGTCTTCACATACAAAGCTTTTAATCTCCCAGCTTTATTCACCCGGCTATCTAAAATTGAAAGCATTGCCT
GATGAGCAATATCAGGTCGATAATCAGAAGGGTTTCGATTATTCTTCTTAAGAAAGTTGGAATGTTCATCTGA
GTTGAGAAGTTGGTAAGTCTTTCCAATTTTAGCAATTTCCAGAGAAGCTCTTTCGATGATGAAAATAACACCA
GGTTTGGTGCTTTGATCAGTTGAAACAACCGGAATACCAGCCAGTAAATCTACAACTCGTTCAACTGCCTCTG
T
>TCONS_00067833
AAGTCTACAAAATCTTCCATTTGCGCTCCACAGCATTTGTGATCATGGATATGCAGTATGCAGCACTCAATGG
ATAATCAGAAATCGATAGATAATCTTCGACATAATCTTTATCAATTTTCCCGTGGGCCATTGCCCCAACCACA
AAAACAAGGTTCATGTCCTTGTTGATGCCATTAACATATTCCTGAATATCCACCAGCTTCTCAGAACTATGAG
AGAAGCCTATCTTCCGACAGTCGATGGGTAGGTACCGCGAAACAGGGTTTTCTCTAACACGTAACAGTTTCTC
TCGCTTA
>TCONS_00067850
TGAGGGGCTATCCATGTATTTGGCCTTGAGTTTACCAAGTACATGAATAGTACACAACTAAGTTTTATATAGA
CTGCTGATATATCAAATTTTCTTGTGGCTCATTTAGAGCTGCATACAAGACCAAACAACCAAACAGTGACACT
TTACTCTGTTACGGCTTATCCTACCTGTGTAGATGCCTCCACTACTTATTTACTCACCTAACTAGCAAGCAGGA
AAGCTTACAGCTGGTATTCATATACATTTACCAAATAGACCTTTTTTTATAAGGTAAAAATTTATTGAAAATGT
ACCAAGATGGTACACAATAGTACAACAGGCAGCCCAGATAACAACAATCGCATACTATCCTCTCCCTCCTAA
TATCTCCAGAAAGTCAATATACTGATTCTGATTTAAATGGAATGGGTTCTCCATATCTGTTTCAGTCTTCTAGA
TACT
>TCONS_00067903
AGTGTGATAATGGTGGATGAAGCCCACGAAAGGTCCCTCTCGACGGACATCTTATTGGGACTACTGAAAAAG
ATACAAAGGCGTCGTCCTGAGCTACGTATTGTAATATCATCGGCTACAATTGAAGCAAAATCAATGGCTGCTT
TCTTTAATACCAGGTTTGTTATTCTTGGGATGAGTGTTTACTGAAGCTTTATAATTGTATACTCAAAAGTCAAA
AGTCCAAATCTCCTGCCTGCTTTCCGTTGATGCAATTTCAGTTGAGAAGACAATCCCTACAGCTGATTTCTCTT
T
>TCONS_00067934
AGCAGATCTATGGCCAACTTTTCCCCTGTTTGTCCAATGCAGTCCTCAAAATTGGACCACTATGATACCAAAT
GAACCACAAAAATATCTAATAACTCTCAAACACAAAATTGAACAGTTACAAGTCGAAAATGGTGGTCCTAA
GCAACAGGCCAGAATGAAAATTGATATTATATGATTGTCATATATTATTTTTATCAAACAGCAAATGCAGCAT
ACTGCTCTCTATCAGTGATGTGCCTTAGCTTCAGCCTTCTGGTGAGACTTGGCCTTAGCCCTGAGATACTTGAG
CTTCATGCTACCATAGACAAGTCCGAATGTGAATGCCGAGGTACGAGCCACCAGAGCAAGGGTGCTGGTACC
AGAGTAAGGTCCGGGAAGAGGCGCCATTGTTGACTTGCAAAGTAGAGCTTCTTCAAGATCTCTTTTTAAGCCC
TAGATTTTTCACAAACCCTGAAAGGCCTGAACTTACCAACGACGGTGGG
>TCONS_00067944
CAGCTCACAACATATAAGCCCACCGTCGTTGGTAAGTTCAGGCCTTTCAGGGTTTGTGAAAAATCTAGGGCTT
AAAAAGAGATCTTGAAGAAGCTCTACTTTGCAAGTCAACAATGGCGCCTCTTCCCGGACCTTACTCTGGTACC

AGCACCCTTGCTCTGGTGGCTCGTACCTCGGCATTCACATTCGGACTTGTCTATGGTAGCATGAAGCTCAAGT
ATCTCAGGGCTAAGGCCAAGTCTCACCAGAAGGCTGAAGCTAAGGCACATCACTGATAGAGAGCAGTATGC
TGCATTTGCTGTTTGATAAAAATAATATATGACAATCATATAATATCAATTTTCATTCTGGCCTGTTGCTTAGG
ACCACCATTTTCGACTTGTAACTGTTCAATTTTGTGTTTGAGAGTTATTAGATATTTTTGTGGTTCATTTGGTATC
ATAGTGGTCCAATTTTGAGGACTGCATTGGACAAACAGGGGAAAAGTTGGCCATAGATCTGCTAGCACAATA
ATCTTGTT
>TCONS_00068073
GATCTATCAGGATTTGCCATAACATGGATTAACGAGACTTCTTACATCGCATTTGTGAAGAAGCACAGAATAT
CATAGGGGATTGTCACTGGAGGTTAAACTTGGCCACTGCTTCAAATTTGGAATTAGTACTGGAGATGATGTTC
AATGTAAGCTTTCAAAGGTGGAGTGATGTCGCTTAGTGATGGTGGTCCTTCAAGGACATCCCAAGCCCAGTTT
CTCAAAGG
>TCONS_00068107
ACATGAATACTAAAGCAGTAGTAATGTCAGGCTGACTATTCCTGAGAAAAACAAAAGCCAGAAAACTCCAG
TAAAAGCATGGTCTATTCAACTTAACAGAGAACCTAGTTAGCGCTCAGACTAAGACCACTAGTGGTAGAGTC
AACTATGAAACATTAACTAATTGGTGCAGTCAAATTTAGAAGCTCACCCTGTCATCATCATGGTCCACCCTGT
CTCTCAACCGCATCTCAAGCATCTCTCTTCCCAGCCAGTCGACTGAACTAGATGAACTCTTGAATCCATTAGC
ACTTCTAGAACTGCCTGCTCCTCCATGGCCAAGGCCAGTGGACGCCATAAATGAACCTTGTGGGAATCACTG
ACACTAATCTCTGCTCCTCTCTGCATAATTCCTTCTGGTGCAGTCCTCCATCCCATATGTGTTCTTGTCTACCTC
GATGAATCCGAAGCAGTATTCAAGCATTTCTTCTACAAC
>TCONS_00068172
ATACTAGAGCCTCAGATTCTACTAGATATCGAGTACTAGCATTTCAACATGTTCTTTCACACATCTTAATCGTC
ATCACAGCCTAAGTTAACTGAAAACATAATAGAATTGCAAGAGATTTCAGTGTCTTTATCTTCAGCTCTTTGA
GGCAAGCTTTTCCTTTGCCTTCTGAATCTTCAACACCTTTTTCACAATTTTTTGTTCTTCAACCAAGAAAGCGCG
AATGATTCTCTCCCTTACAGCACTTCCAGACAGCACACCTCCATAAGCACGATTCACAGTCCTGCGGTTCCTG
GACAATCTGGATCTCTTGTATTCAGTAGGTCTCAGGTGTGGAATCCCTTGGATTCTCTTCCCAGTAACTGGACA
TTTTGGGCCGCTAGCTCTCTTCTTTGTGCTCTGGTAAATTAGCTTTCCACCAGGAGTTTTGACAACCCTATGTTG
ATTGGATTTGGTGGCATAGCTGTGCCGCTTTCGATATGTAAGTCTCTGAACCATCTTTTCAAGTAGATGGAGTT
TTCCGCTTCTCTACAATGGTAAGATAAGAATGGACCAAAAGAGTTTGTATGAAGTGACGAGATG
>TCONS_00068286
GCAACAATCACTAGAAGTTCCTCTTAAAGACTTTAAAATACAAGCAAAAATGATCAATCAGCAATCCTGCCA
CAGCTTAGGCCAATGCTGTCACTATGAAGTCCTAAATTCATTTTTGAGCAACCGTGCAAGTCGATCCAACAAA
CGGTAGATAAATACCAATACTTTTTCAAGAATCAAACACGTAGACAATACCTGAACAAACTGTAATTGAATC
AGCAAAACGTGAGCTTTATTAGAATAATAAAAAACAAATCCAAGTACATAAAAACACAAAAATCAGAGATA
ACACAAGGGCAAAACCCATATAAGTCAAATCCATATATCAATTTAAGCAAATGGGTATCACCTGCTATTGGA
ATAAGGTGATCTGCTTCTATCTCTCCGGCTTCTGCGGCTATGTCTGCGTGCCACAGGCGAGGGTGACCGTGAA
TATCTCCTTCTATGTGCTGGCGACCTCGATCTAGACCTTGAAATATCCCTATCTCTACCCCTACCCATCTCTTCC
TCAACTAATTATATGGAACACACAAAAATTCACCTGTTACTACAACAAAAAGATAGATCTTCCCCGGTGCAT
AACGATTGAAATAGAATAAAACCCTAAAAAGTGGAATGAAGTGAAGAATAG
>TCONS_00068436
ATATTTTAAGCAAGATCGCGATAATGATCATATGGTGGTTCACGGTCAACACAAAGGATAGCATAGAGAGCA
TAGATAATTCCAGGAACATAGCCCAATATGGTCAACACCAAGCAAATCAAGAACTCCACAGTGCAGCAACC

ATGGCGAAGGCAAACACCAAGAGGAGGAAGCAAGATTGCAAGTAAAACTTCACATAGAATTGCACATCTTG
AGTCCATTCTTGGGGGGTAGG
>TCONS_00068482
GGCATCTGGAGCAAGCAAGCTATTTTGCTGGAGACGCTGGATAGCTCTTTAGAACTTGCCATGATTAACTTGA
TCTTTGGTCTGGCTAGACAAGAGTGATGATACTAGGACAGCAAATCTTCTTTCCTTGACAGGAAGAGAACTCC
CAGCTTTCTCACAACCCATGGAGTCTACAGGTGCATCCTCAGAGGACCTCATCTTGCGGATTCCTTCAAGAAT
TTTTTCCCAGT
>TCONS_00068538
TTTTTTTGTTTTCTTTATTTGATTTGCGTTTTCTTTGGGGACTTAGATTCCAATTTAATGTGTCTCACAACCGAGA
AAAATTAGGGGGGTTATTTTGGTTTTGGGTCTGCGACGAATAGGTTCAAGAGATGAGAGAATTAAGGATACC
CACCAGAAAGACTAATCCAATCCATAAGGATGTACCAGAAAATACAACATTTTTGTTACTTGACCAGCCATC
AGGAGAAGCAAATACAACGGGTACGCTAATCAATAAGATTAATGAAGTAGCAATTAATGCAAAAACAGCCA
ATTGGAAAGCAAGAGTCATGCTTTTAATCCTCCAAGCTACCAACAAATGAACTATATACCATTTGATCCCTCT
ATCAGCCAAAAAATATTAATTGTGATAAAATATGTCATCGAGGGATTTTACTTTATCATGAATCCATTGATTT
>TCONS_00068565
CTACAATCGAGGAACTGCATATCTAGATCCTCAAGTTTTTGGTATGCATCAAGGAATCTCTTAAGCTGCCGTG
GGCCCACAAAAATTAAAGGTTCATGAGGAGTTTCATTGAGCAAATCGCGTTGGAGAGCAAGTATTCTTGCAA
GACCAGTATGATGATCAGCATGAATATGAGAAATCCAAATACACCTTAAACCTTTTACGGCTTCATCAGCAC
CTTCAATGCCAAATCTTCTTTTGAGCTGTCCCAATGTTCCTTCACCACAATCTAACAGAATACTTCCCTTTGAG
AAAAGATTGACAAAAATAGAACTAACATTCCGATATTTTGAAGGCTGAGATGAACCAGTTCCAAGAAGAAC
AATCTCCACGTCCTCTCTTGTTACACCTTTCAAACAACTAGGTAATGCAGTTTCATGCAACCAGGGCTCTTCGA
TCATAACTTTGTTCGCCTGCATAGTCGCACTGCCTCCATTTGTAACATTGTTGTCATGCAACATCTGGGTAATA
CGTTCAGAAGCATCTGATATTTCAGGAATTTCTGATATTAACTCTTCTATGATTTTTGGCCGAGAAGTAATTTC
AGGAATACCAGATCTGTCTAACCCAAGCTGCGCATATGGACGAAGATGAAACTTGAGGAGGTTCTCTGCCGT
AACTTGACATGAAGAGAACTCACTTGGGGTCTTAGAGACTGAGAGTAAGCTCTTCAACTGCGGAAGGGACCA
AAAACCAGGCGCAGGAAAGAATTGAGGGCACAAATAATTCAGTCTTGAGGCAATTCTTGCACTCGACTTCAG
AATTGGAATTT
>TCONS_00068568
CACACAATCAACTATTTTGCACATTTCCTTGGATTCTTCGGATGGATATGAATAATATGGAGTAAGATAATGT
ATAGACGACAATTCTTGCAGATGAGATGGTGTTGGGCAGTCAACAACGAGTACAATAGGACCAGCAACAGA
AGGCCCCAATACATCACTGGGACGAACCATGATATCTTGGCGATCTGACTTTACTGAATTCCCAAGTTGCAAT
TCACGGTATTTTGGGCCAGGCCTCAACCCAAGAGCAGCAGCCTTTTTGGGATCAAATTTTCCCTTAATTTCAG
GCAACTCACAAATATAAACCACCGAAAGATCCCCAGGCTTTAATAAAAATTCAGCTGTGGAATGCATCCTCC
GCACATTGACACCAACTAGAGGATCATCCAGCTCAGAGGATCCTTCCTTTGTCGTGTCAGACACTTTTGAGTA
ACGTGGTCGCAACAGAACAGCAGATATTTTGACAACCTCATCGTTAATAGGAACATAGAGATCATCACTCGT
CCCTGAAGAAGCAGTACTAACATCAACTGATCCAACAGTTGGGCCAAAACTCCGTGCATGGACCATGGC
>TCONS_00068681
TCTCTCTCAGAGCCCTCGCGATCCGAAACATGGCCACTTTCGCCGCCAGGTCCGTCCTCCGCTCCGCCACTTC
CTCCGCCAGAACTGCCGCCACGAGAGTTGCCGCCGCTGCCAAGCCTAAGGCCTCTACTTCTCCCTTTCGCCTC
CCCACTCAAAAACCCCTCACTGCTCGCATTTTCAGGTCGCCTGTTGAAATGAGCTGCGCAGTTGAAACAATGC
TTCCTTATCACACTGCCACTGCTTCTGCGTTGCTGACTTCAATGCTTTCCGCTACGCCTCGGAGTTATGGTTGGA
CTCTTGAGGATTGCAATGATGATCTATGATGAATGTCGGAGGGTTCAAGCGAAGTTTAATGTCCATGCACTCT

GAGACAATGTCTGAAAAATAGAGTACCATGGATGATTGGTAAAATGTTTGGGATGCAGTGCATGTCTTTGTA
GTCCAATGGATCTAGATGGACAATGTAACACTAGTACCCAAGTTATTTCTGATGTATTTGGAGTGAAATGA
TGATCTTTG
>TCONS_00068682
TCTCTCTCAGAGCCCTCGCGATCCGAAACATGGCCACTTTCGCCGCCAGGTCCGTCCTCCGCTCCGCCACTTC
CTCCGCCAGAACTGCCGCCACGAGAGTTGCCGCCGCTGCCAAGCCTAAGGCCTCTACTTCTCCCTTTCGCCTC
CCCACTCAAAAACCCCTCACTGCTCGCATTTTCAGGTCGCCTGTTGAAATGAGCTGCGCAGTTGAAACAATGC
TTCCTTATCACACTGCCACTGCTTCTGCGTTGCTGACTTCAATGCTTTCCGCTACGCCTCGGAGTTATGGTTGGA
CTCTTGAGGATGGATGATTGGTAAAATGTTTGGGATGCAGTGCATGTCTTTGTAGTCCAATGGATCTAGATG
GACAATGTAACACTAGTACCCAAGTTATTTCTGATGTATTTGGAGTGAAATGATGATCTTTG
>TCONS_00068731
CTTAAGAAAGGGACAATCTCCTCGACTGATAACCGAGCAATCCCAAAAGGAGACCACATGGCGACCCATCC
ATAGGACTAATCTACCAAATGAACCCCTCCCTGGGCAACGGACTACTTCTTTTAGAGCGGGCTATTTCTAGG
GCGAGGAATATATTTCTATTTAACTACTCCATCTGGTCAAGAGCCAGCATCTCAATGCAACATTATTCCATTCT
CAGTCTGTAGTGCTTCTGCCAGCTAATGCTAATAGAAGATAAGAGATTGTTGAAAGTAACTGAACAAGGTTG
TACAACTTTGGTTCATTAATATCAAGTCTAAAGGGTCATGTATCCATAGTAAAAGTCCCCTGTGATCAAGCAT
AGTTTGTGAAAGTGGGATCTTTTTTCCCACTTTAGCTCAATTGCATTGTGAACCTTCTTTGCTCAATCGCTTTCC
TTAGGCACATGTCTTCAAGTCTTGTAGAAATTGATGAAGAGGAGCGGAGATGGAATGAAAGAAGCAATGAG
AGAGCCTAATTCCCTCCTGACCGAGTATAAGCATGTTCCATTGGCCTTTATTACATTAGATTAGTGGGACTAC
GGCTGGCTCTTTCTCCTCAATCGGTGGAAATGCCATTCATAACATCTTTGGCAACCTTTCTTTCACAGGTGGTA
GAATTATAGACCATATAACCTTGCCAACTAAAGCGTCCTACTCTACCGCAGTAAGGGCTTAAGCGATCGAAA
TGACCTAACCTGGCTCCATCTAGAAGGTTGGAATCTTCCGGGTCTATCTGCTCTTCAATACCTTTGCCTCAACT
GGGATTTCGAACCTTTGTTTCGGTACCAAAAAGTGAAGTGGGTCAGAACTCATATGAAGTTTGAAGTTAGAA
CCCAAGCGAGAGTCGTGGAAGAATCATTCTATAGGCAGAAATAAAGGCTTTCTTGGTTTCAGAATGGTTACA
GGAAAACTCTATTTTTTCATGCTTTTATGGCAGCAAGAAGGGGAAGGAGCCCTTTCTGGCTAATATCTGCTGG
TCACGGGTCGAATTAGAGTACTTCAACTTGGAAGCTGAAATCCTACCTTTTCTTTATGCGTAAATCTGGTAAA
AAAGCTCCCTTGGTGCTTTATAAGATTGCTTGAAACTCCGGGCATTTCGCTCCCTCCAAGTATTATATATGGTT
TTCCAAAAAAAAGCAGTTTCGTAATAAAGCTAATGAGACCATGCCCCGGATAATGGTGCATCACCTACTCGG
CCTCATCTTGCAAGGGAAAGGGGGATCCCCTGGCTAGGAGGGTATTCAGCAAGTGCTTCCAAATCTCCTCAA
CATAAGGGCAACAATTGATAGTTGAAACCTCTTTTTCAAGTCCCGGGGGACTAGTTGCTTGGCTCCGACCATT
TCCGCTAAAGAAAAAGCCATTCCTGACTAAAGAAAAGTCAAAACAAAAGGTAGGCGGTCAGACCTAGGGCA
TGTAGCATAGAAAAACCTTCGCCCTTAGCGAAGGGTTTTCTGAAAGCTCCATTTGAAGCAGGTGCCTAAACG
GATGATTCCAGTTCAACTCATTTTTGAAATGCTACCTCAAGAGAAGGGGGGCCTCTGGCCCGGAAGATGCCTT
ATATGGATAGGGTATATACTACTACGATTGGCTCTATTCCCTCAGAGTCCCAATCTTCTTGAAAAAACGTGAT
ACGCGGAACCTTGGTTCGTGAATTTTCCAGTTGGAATTCCTACATTTAGTATTTAGTAGTTTCATTCGACAACT
AAAATTCATGCTTTTGACGTAGCATCGGGTGTTCACATGGAAAACCTTTCTATAGTGCCCCATTTCCTATGATA
TCCAAACCCGAAATATGGTGCTTTTGCTGTGCCATTGAGAAAGGAAAGCGCACTCAATCAATTAC
>TCONS_00068811
GCTCCATTTCTCTGTATTCACTGAGGAAGAAATTCTCCGACTCTTATCTCATCTTGTTGACATTTATTGATGTTT
ATTGAGGAATTTTACAGCATTGCACTTTTATCTTATTGTGTAATCTTTTTGAACGAGAAGTTGTTTTCCCCTCCT
GTTCTTAGTAGCTTCTCCTAGTTTCTGTTGTCATAGATTGGACTTATCTATTTTTGTTTGGATAATTTAATTTGTG
AAGAGGAGCACAGCAGATGAGCCTCAAGTATTAACCAGAATGCTTCTGCCCTTTGTGGCAAGTGAAATACCA

ATTTGTGAAATGTGACTTAGCTTAACAACTGTTGACCCCTGTAAAAGGATCAGGCTCTGAAATGAAAAGGTTT
GGTCCAAGATATCACTCTGCTGCGGAGATAAGGCAGCTATCTCTATGAAAGAACTTAAGCATCCAACCCAAA
ACCTTAAAACAGAGGCTGGACCAACCTAAGAGCTTTATAAAATCCTTTGGAAGCCCTCATGCATATCATGTG
AGACAATTACCAGTGTGATAATGATGTTACCAAATGTTGGCTCTGATACCACGTTGAATTATGTAACTCGCC
>TCONS_00068857
TTAAAATCCAGCCGCGGGTTTATCGTTGCAAGCTTCATTGAGAGGAACTCCACCTGTCTTTGAAGAGATTGTA
CATAATTAATGATTTCATCCAGCATTACTGCTTTCCCAGTGACCTTGTTGCAACCAGGTACAAGATCCTGAAG
ATATTTCATCCGTTCACTGATCTTCTCCCTCCTTAGTCTTTCTGCAAGACTATGGCTATTTGTTGCCTGGCCTCTT
CGAGCTCGAATGTGTATGTATTCTTCTTTAGGTGGATCTGCCGATTGAGACCCCTGTTTACCATTCTTTCCACC
AAGCCTGTTTTGCTCTCCCTTCTGAATTTCTGTTTGATCTTTTGTTCGTTCAGCTGGCAGTTGTTGTGCTCCCTTC
GTTTGATCAAGTTCTGT
>TCONS_00068870
ACATAGTTCTGCATTGGAGAACAGAGAATATACATAGTAGGTGTGATAATGTACACTTGAGAAGAAAAGTTC
CAATTCAGTTATCCTATTTCTTGTCAGCCACATCAAAACCTTCCGCAGAATGGTACATGGACAGGAAAAATGC
ACTTTGATTCTTCTGCAGGAATCTGAGAAAGTCTTGTAACTTCTGCCTTAATTCTGTTAATGTTTCATCTTCAAT
ACTTGCGTAGAACAGAATCTCCGGCAACTTAACAAAAAGCCGTAATAAATGCTCAGCTCCATAGATAGAGG
ATGGAGAGGCATCGTCTCTAATTGATTCTTGGTATTGTTCCCGCTCGTTCTTGTAAAGGAGCATAGCAGGCAG
TGCTTTGTCGAAGTAGCATTGCAAACCACTAAGAATCTCTCCAACAGAATCAGATATCACTCCATCCTTTTTC
AGCCGATAGTCATGATACTTCTTTAATATTTCATCTACATTTGGAGAACGTGGAAGTTTGACGAGCTTGCCCA
AGTGGTTGACAAATTCACAATCATCAATCAGCTGTTTCTTTAATTGTGATGGTATTTGGATATTGACAAGCTTT
TCTGGAGGAGGAGCATACTTGTCCTTCTGCGAAACATCACTCTTTCGCTTTCTGCCTCTTCCCCCTGTAGAGCC
TTTTGTTTTCATTTGTGATCCACGTCCACCCTTTGAACTCTTATCTGTATCCTGTTTTTTCTTAAGCTCCTGCTGCT
TCTGAATATTCTCTTCTGTGAGTTTCATCAGTCGATCTATGCCCACCCATTCATCCCAGTTTTTGTTCCAACCCT
TAGCTTCATAAAGTTGTTGGCTATGAAAGGCGAGTACTTTTTCACCTTCTTGAAAACGACTGGAGTCAACCAC
GTCAATTTCAGTATCATGAGGGGTTCCACCAACTTCATCAGAGATGGTAGTGGAATCGTCCGACACCCCAAC
ATTTGAGCTC
>TCONS_00068975
ATAGGGTCTCTTAGGAAGACTTAGACGAGATCGACTTTCACAAAACAGTCCCAGTGTCCATCTTACCTATATG
ATGAGCACTATAATTTGGCGGTATTTCCAGCCGACGAAATCTAAACTGAATCTTGTATACTGGTCCTGGACCG
CAGCTGGTGCACGTATTCTCCATAACAGAAGCTTCTGATAGAGATCCAGGTAGCCAGACAACATTTCTGCATT
GTGGGTAACCCAAGCAACCAACCATAAAATTACCATCCGGTTTTTGTCTGAGTACCATATCTGATTCTAGACA
AAGTCCACATCTCCGCACGACATCTCCAACTGCTTGTTGCTCGCCACCGCTGGATCTATTTGATCTTTCAAAGA
AGATAGCCATGGCTTCAAAGAGTTTTCCTTTGTTTGCCCTGGCATCTAAAAAACAAGCTTTCATCTGCTGCAA
GGAAGTTTCTAGAACCTCTGTCTTTCTCTTGGTTCCCATGCTAACAGCTTTCATATCACATTCCATCATAGCAC
GGAGATATGGTTTCCATAGTTCATAACCCATATCATCATATCCCATAACTAGCGCCTCCCCTAGTTTAGTTGGC
GAGAACCGAGTGCTCGAGTCTTTAATTGCATAAAATCGATCAAGTAGTTTCTTTATATGGTCATGCATTGTTGC
ATCAGTTCCAATGCCAGCCTTGTCCATACAACCCAATAGATCAGCCTCACTTAATAGGGGAGGAGGTCTAGT
AACTCCTGAATCAAGGGTCAAACTTGTTGGTATAAACTGTTGACCAAAAGTGTACGGGGGGATCACTGAACC
TCCCCATGATTCAAATCGGTACACGTCAAGGTAGTTTTTTGCAAGTATCACTCTCCCACATGTAAAAAAGGAT
TCACCAGCAATATCAATCTCAACTGTAGTTTCAGCCCCAACAGCAGGTTTAGAAACACAGGCCAAGAAATGG
CGAACAACCAACTCATACACTCTATGGTGGTCTTGGCTCCACCTAGATTCTCCAGCAGAAAATTTAGTTGGAT

GAATGGGTGGATGGGCTTTGTCATCATGCCCACCTCCACTAGGGTTTCTCCATAGTCCTGATTGAGGGTCTAGT
AGGCGCTGCG
>TCONS_00068976
CTACTTAGAAGAGACCAACAATTAGAGTGACGAAGCACACATAATGGTACCATAAAAAGAATGAATCTAAT
TCTGCGAGTTAAGATTGGCCTATTCAGTGCAAAAACATCTTTAATGCAAGAGAATTCAAATTCATCAACCACG
ACGTGGACAGACATTAGCAAAGTGACCTGGATCACCGCAGATGAAACATCGGCCAGTTATTGGTTCACCTGT
TGCTGACACAAATGACACACCACTGGTATTTGGACCACCTTGGCCACGGCCTCCTCTTCTGCTGCTAGTGGAC
GCAGAAGTATTTGTACGCTCACCTCCATTGAGGGTATCTTCCCAAATGAAAAAATTGCACTCCCGTGATTGAC
ATGAAAAGAACTTTCTTCCCCTGTTGTTG
>TCONS_00069012
TCACCTGATTCTGACCCAACCAACTTGCATACTCAAGATCAATCTTTTTTGCAGGGATGCTAGCATCTATGAG
GAGGAAGACTGAGACAAGGGTTGGCCGGTTTAGGAAATAATCTTTGGTGAACTTAGCCCAATCTGTCCGTAC
TTCATGTGGCGCAGCTGCATACCCGTAGCCTGGTAAATCCACAAGGTACCAGCTATCGTTGATCCGGAAATG
ATTGATGCATTGTGTCTTCCCTGCAATTAGACAGAAGAAC
>TCONS_00069014
ACATTTAAAGAGGAAATTAGATCTTGAATTCCAACCCGAAGAGGAATAACATATCTGTCTTGAGTTTTTCGTT
AATCTGGATGAGGGTTGCTATATTGCTAAAAAGGTTTAGGTGCTTACCCGTTTATCACGTCATGTGCACTACTT
CCATAATTTTGCCATTTTGGGGAACAAAGGAGAAGAATCCTTATCAACATCGGGTACACTAGTGCATCAAAT
ACCCCATCTTTTTTTGGGTTTTGGGTGAAAGTCGCCATTCACCAATATAACGAATGATCTTGCAGAGTATATTG
AAGCAGGTTAATAATGTTTGCCTAATTCATTCTTTATCGTCGAGAGGATGTAATTGTTATGCGGGTATACCAAT
TCTAGCATTACTTCTTAACTCAAATTTAAGATGTTGTTTGTAGAATGACATAATTGA
>TCONS_00069050
TTAGATGGTCTAGAGACGACGTACCCGTTGATGTCGTTGATATTCCATCTAATGCACAACATTCAAATGATAC
AATTATAGAAACATCGGAAGAGGAGGATGAGTTTGACGATACTGATTGGGACTGGATGGAATCAGATGATTA
AAGAGGGAAATTCTGAAATTCTTTTAAATATATTCTGGTTTGAGATTTTTGGTTTTGGATCTTGTGATATTGTTG
CACTTGTTGATAGCAATCGGTCGAGGCTTGAATC
>TCONS_00069069
GTTTATTGTTGCTTCAGAAATTCTTTCCACACTGTTTGAGGGAACCCCGAGCATTAGGAAAGATGCACAAAGG
TTCATTCAGCTTCGGGAGGATTACAAGAGTGCAAAGCTTGCAGCGAGACTCAGCTCCTTATGGCCTAGCTCTA
GTTAGAACCAAAATCACATGCTAATTAGAGTTGAACTTTTATTATAGTGGTGAGCTGGACAGAGTGCAATTGT
ATTGTGAGAGCTTTTCCACCACCTC
>TCONS_00069090
GAGGTGGTGGAAAAGCTCTCACAATACAATTGCACTCTGTCCAGCTCACCACTATAATAAAAGTTCAACTCT
AATTAGCATGTGATTTTGGTTCTAACTAGAGCTAGGCCATAAGGAGCTGAGTCTCGCTGCAAGCTTTGCACTC
TTGTAATCCTCCCGAAGCTGAATGAACCTTTGTGCATCTTTCCTAATGCTCGGGGTTCCCTCAAACAGTGTGGA
AAGAATTTCTGAAGCAACAATAAACACATT
>TCONS_00069142
CAGAGAAAGTTTACTAACACCAACTTCAATATCTTCATAACCTTCCTCATCCATGGGACATATTTCTCCATTGC
CTGTTTCTGATAAAGGCCGCCCTCCGGACACATCTTCATTTCTAGAACCAGTGTGTTTGTTCTGGGTGGAGTAC
AAGCCAGAGGGAACTCGTAATGATTTTTTTCAGGGGTTTTCACATTTGAGGATCCATAAATAACTCCACAAG
CAGAATCTTTGCGTGAGGCTGGGGTTTCTAATATCTTCGATAATGAAGCTGGGGGTGCATAGGTATGGTTAAG
TTTCTTTGAAATCAACCATTTCTGTAGAACTGAGGACAGACGTGATATAATGATATCGTCAGTTTGGACGCCT

GTAGTCCATGTACTGTATTTGTTTGGGGAGGGGCTTTCTACCATTAGTTCAAAATCATCAACTTCTTGAAAATA
TGCTCGCTCCTCATCATAGTTTGCAGCTTCAACTTTTTACCCCTTGGAAGAGGTGGTTTTGCTTTTCGTCCAGG
CTTCCTTTTTTGAGGATGTAATTCGGCA
>TCONS_00069231
TGGTCATATGTAACACCTCCAAAGAAATCCATAAAGCTTCCAATTGAATTATAAGGCATAGCAGCATTCGTGT
AATGAATTTCCATTTGCTGATTCCAGTTCATGGTGGATTACTAGTCAATAATTCTTCAGGAAAGCAGAGAGAT
GAATAATCAACATATGAAACTCAGATTCCAAGATAAGCAATGTGGATCCTCTTCAGATTTGACAATCATTTGC
TTTGCCTTTTTTACGCAGTCGTCAAGCTCCTCATCAAAGTCACTCTCTTTCTCCTCTTCTCTCTCTCTCTCTCT
CTCTCTCTCTCTCTCTCTCC
>TCONS_00069244
GAAAGGTTGGCTCAAAAGGAGATTGTAAAATCTTTTTTCTTCATAATTCTTTGTGTTGGGGTTGTGTTGATATTC
TTTGATCAACTTGTTATTCATTAAGAGGTTGATCTGACTTTTCTGTCAATTTTACCGATGGTGTAGATGCATTCC
ATAAAGATACAGAAAACTCAACCAATCATCTACAGTCCAGGGACCCAAATTGATGATTTTGAATTTTTATGG
ATTAGCTCACAGAAGAGTCCAAGATGGATTTTCCCTAAGGATGGTTAGGAAACTGATGAGGATAAGAAGATG
CAGCTTTAACTGATACCTTTTAAGAATCTAGGGTAATGTGTGCGGTTGAGGTTCAGAATTCTTGGGTACTTGGA
GTTTCAAGAGCAAAAGTCAAGGCTCATTTCATAAGGACACATGTTTCTTTTGCGTGTCACAGAAGAACCAGAT
GATTGGCTGGAGAAAACTGTCCGCAAACATCTATAGGTAAGTCTTGAGTAGGGCTGTCAAATTTGGCCCAAG
CCCAATGACCC
>TCONS_00069346
ATCAGTCATCAAATGATCATCATTTGGTGAAGGAAGAAACATCAAGTATTCCAGCAGTAACAAGGACATGA
AAATGATATAATACGACCCAATCCACATCGTGGTCCAAATAGCCCATGTCCTAGAATGTATGCTTTTGATCCA
GGGGATGGGCAGATGAAGTCAGCAGTAATGGGACAGAGCTGATCTGGAACTAGCTCAAGTTGTAGTAATGC
AACATCCAGAGGTCCTCTGGAGACATGAACCACCTTTGCATTTGTCCATACCCAGGGATCCATAAAATCCAA
GCGAACACGAATGGTCCTACTGCCTGTGTTTGCCAGGTTAACTCTAAAGCTACCCTGCTCATTGTCAACCAAG
AAATGCGGAGTTCTTAGTTCCTTTTGAATCAAAT
>TCONS_00069396
AAAGAGTAACAACGATACCAAGATTTTTACGTGGAAAACTCTTTTGAATAAGGGAAAAAACCACGGCCCCG
AGAGAAGCAACTGATATCACTATAGCAAGAAATTTTACACTTTGTAGGTCCGAGTAAAATACTCCAAAGACC
ACTACAACACTCAAAAGAAATAACCCTCTTTTGATATTCCCACCTTACTACAATATCGCTCACTCTCTATTTTT
CTCACAGACTATTTTCTTATACCCTGTATGTGAAACCTCACTCTTTCTTTTT
>TCONS_00069404
AAAAGAAAGAGTGAGGTTTCACATACAGGGTATAAGAAAATAGTCTGTGAGAAAAATAGAGAGTGAGCGAT
ATTGTAGTAAGGTGGGAATATCAAAAGAGGGTTATTTCTTTTGAGTGTTGTAGTGGTCTTTGGAGTATTTTACT
CGGACCTACAAAGTGTAAAATTTCTTGCTATAGTGATATCAGTTGCTTCTCTCGGGGCCGTGGTTTTTTCCCTT
ATTCAAAAGAGTTTTCCACGTAAAAATCTTGGTATCGTTGTTACTCTTTTATTCTTG
>TCONS_00069409
TAGTGGAGTTTCACATTATTGCGATTATAATTGCCTTACTTCTGGAGTTTACAAAATGCCTCAAACGAAATGAT
GAAGATTAAGAGAAAACAAGGATCACAAATTTTATTCTCTCGCTAACCTAAAAATGAGCTTGGATTGGGTTC
CCAGCCCTCTAATAATGCCTCCTTTGGAAGTTCAACCTCAACTAGTGCAAGCGATGGCTCTACTCTTTGAGTA
CATGCTCTGCCTTTGGAGTTTACAACTATCCACTGTCGATCTCATCGAAACCCAGTAGAAGAAAGGGCAGCTT
CAGAAACAGAAATACCACGGCATGATTTCACTGGGTATATGAATATTGATTTAACTTGTGCAATTCCTACTTC
TGCCATATCAACAAAGAAAACAACTACTCTCTCTTTCTTC

>TCONS_00069512
GTCAGCTGTATATGCCAAAAATCCCTTGCTCAACTATTACTAAAACTTTTTAGCATCTTTAAGCTGCTACAGCT
ACCTTCTTAACTCCTTTCTTCTCTGTTTTAAGAGGGGATGCCACTTCAGGTTTCTCAATTCCTTGTATATCCGTA
ATCCTCAATTTTGTTAATTCCTGTCGATGTCCTCGTGTTCGTCGATAATTCTTCCGTCTCTTCTTCTTGAATATAA
GTACTTTAGCATCTAATGCGTGCTCCTCAACGACAGCATGAACAGCTGCATCCGGCAATATAGGCCTGCCAAT
GATTGTCTGAGTCGTTGAGCCCAGCAGCAAAACCTTGTTTAGTATTAACTTGTCATTAACTTCGCAGAACTTCA
GTTTCTCAGTAAAAATGGAATCACCATTGCTCACTTTAAACTGGTGAGAACCAATCTGAACAACGGCGAAAA
CGGGTTCATATGGTTTGAAGACCCGGTCCGATTTCTGAAGT
>TCONS_00069519
ATTCAAAGTTCATATCATAAGGGAAGAAGTGCACTAATTTCTCATACCGTGTAGAGACATTATTTAAAATTGA
CAATGTAATCCCTTACAGGAGATCCAAGGGAGTTCTATTGAGACATCACAAAAGCAACAAACATACGAAAT
CAAATAACACTAAAAATGCAACAATATTATAGTGTTACTACCATGATCACTTCCACACCGCCGTATGCAACA
ACAGCCACATCACAGAACTGCAATTATCCTTGAGATCTTGTAATCACACAGCTATGAGGACTCAATTTTCTTC
ATCATATTAGTATGGGAGAGGAAAAAGAAAAAGCAACTATGTACATCCTTACACCTTCCTGCCAAGTCACTG
GTTCTTGAAAGACTCGGAGTTTTTGGACACATCCTTGATCAGGTCAGCAACTTCTCTCTTCTTTTGCTCAGCCA
TTTTAGATAGATCCTCCATTCTCCTCGAGGTCCTTACAGCAAATCTTTGGAAACCAGGGCTGTTGGCGAGTCC
ATTGACGATCAATTCGTTGGCTACATAAGAAATAACCCTATGAATAAAGTTTCCTCCTCCAGCCATGGCTGAT
TGCTACAACTTCT
>TCONS_00069584
TGTTTTCTGAAAAATTTGACCGGCATATTTTGTACTTGGACATCTGGAAACTAGATGATTAGTTACCATATTCA
CATGAAAGCATTCATACTGAGAGTACTGCAGTAGGTATCGACAGAAGCATTCTTAGCGTCTAGGTAACATTA
ACTGTAAGGCCCAGATTTCAGTATACAACAGAATCAAAGATCAAGTTTCACACTAGGCCCGAAATCAAGTGC
CTTATCTACTTCACTAGCACCAAAAGCACAGCACCAAGAAGTGAAGGGAAAGAACTATCATCGTGAGTTCAT
AATCTTCGAATTTATAAACAGCTGGGTATTGAGTGTTGCTCCTCAAAAGAGGTTCAAGTTCTTTAGTGAGCCT
GATTATTCAGAAGGATGAAGGGCCTATCGAAGAGTATCCAACAGCCTTGTGTTCTTTCAAAGTGCTGGCTAAC
CAGTCCAGTCCCTCGTATAGGCCATCCCCTCTAAGAGCACATGTACCTTGTATATGCCATTTTCGGTTTTTAAG
CTCATAGAGACCAAGGCCTTCACACACTTCCATTGGTGTCATTGCTCCTTTCATGTCCTGTTTGTTAGCAAAAA
TCAAGATGATTGCATTAAGCATGAATGGATCTCTGATGATGGCCTGAAATTCTTGTTTCGCCTTTCCAATCCTC
TCTCGATCCAAAGAGTCAACGACATAAATCAGGCCATCTGTGTTATTGAAGTAGTGCCTCCAGAGTGGCCTTA
ATTTCTCTTGTCCACCAACATCCCACACGGTAAAAATAACATTTTTGTACTGTACTTTTTCCACATTGAATCCA
ATGGTAGGAACTGTTGATAGAACTTCTCCTATGTGCAGTTTGTATAATATAGTTGTTTTGCCAGCCGCATCTAG
CCCAAGCATTACAACCCTCATTTCAGAATTTCCGAAGAAGGTATCGAAGAGCTTGCGAAAGGCTTGTCCCATT
TCGCCTCGCCTTTCTCTTCTTCTTCTTCTTCTCTCTCTCCAAAAGCAAATCAGATGAAACTGGAGAGCGAAGCT
CGAAGAATCGATGCCACGGAAAAAGATATATTGTGATGATCTGATGATAAAGCAGCTTGTTAATTGGGTGAT
CAAATAGATATATGGAAAGAGTACAACTTGTGCTTTAAGAGAAGGTTGCGTTGTTTTTCCTTTTGGTTTCACTT
TCTGCTGGCAACTTCTCTGAGTTTCTTTTATTTGCTGTTTTTTTT
>TCONS_00069617
GAACAAAATTAAGCTGGAAGCAAATATATAGACAGATATGAATAACATGACAACATATTTAACATACAAAC
TACTGAAATATTACAAGCAAAGAAAGGAATTATGAAGCCCTAAATCACTAAACAGATGAAACATTTTGAAG
ACTACCACAAACAAAGCCTAAGCTCAACTGAAATTACTACAAACATTCATCATCATAATTTCAAAAAACTAT
TCGGGCTTAAGAGTGTCCGACTTTACCTGTGAGCTATGGGTGGGGACTGGCAGTGCATAGCAAAGACAGCAT
TGCATACATCCCTGAATATTAAGAGGACTTGTTTCTCCTCAAAGAAGCCAGCTCCTCGGTTCTCAAGGACACT

GACCAAGGATTTCTCACAATATTCCATAACAAGGAGAGCTTCCTTTGTACGACCCATATCCAAGAAGGTATG
CGCATTTAGTGTGACAATATTAGGATGGCCCTTCAGCGATTTCATCACTGAAATTTCTTTCAGTACTAGATCAA
GTGATTCTTCATCATTAACAATAATATGCTTTAATGCATACTGCTTTGAACCATGTAGTACATCTCGGGCTAAA
TAAACACAAGAAAATCCGCCCTCTGCAATTGCATTGCGTACATGAACCTTGAGACTGCCAATATCAATGGTG
CGACCTTCAAGCCCAGCCTGTTCCTTGGGCATAAAAGGTTTGAATCTCCACATCATAAACAATTCAAAATTGG
TGTAAAAAATCTACCTTGATTAAGTATGTTATCAAG
>TCONS_00069664
TTCACCCTCAATATAGGGCCCAATACGAAGGCAAGCGTATAGACCTTGTGCTTTAATTTGCTTGATGAATGCT
ACTATGTCACGTCCTCCACTAAAATCATACTGTCCAGGACGAGGCTCATGAAGATTCCAAAAAACATAGGTC
TCAATCACATCTATTCCACCTTGTTTTGCTTTTGATATTAGAGATGGCCACATATCAGGGGTGCTTCGAGGATA
ATGAATAGAACCAGAAAACAACATCTTCCTTTGTCCATCTATTATTAACGATCTTCCGTCATAACTCACATTTG
CTCCGCCGGCAGCCACCGCTCCTGCCATCACCAACACTG
>TCONS_00069743
GCCCTTGAACATATGTTGCATATCACCAACAAAACAAAGGCCACATTGAGAAAGAAACTGAACAGAGCCAT
TTCAATTATCCAGCTCACATTAAATGCAACTGAGTTAACTCTTTATTTCAAAGTTCAAAACGACATACAGTTA
CTTCCACGCCTTTTAGTTGATAAAACCACGTTTTCACAACCAACGTCAGTGATGACCACCACCACCATCTCCA
CCTTCTTTTTTCTTTTGTGAAGCAGCTCTTAACTGGCGCTGCTCCTCTTTGTAGATTCGGTTCCTACGTCGATACT
CTTTTGAATGATGAAGGCACTCGAAGTAGTCTTCTCTAAGAAGGGAACAGTCTTTGGGTTCCCTGCATCGAGA
CATACACTCACTGAAGTCCATCCAGAAATCATAGCATCGCCCTTTGTTCCCTGTTATTCCCCATCCTGACGCCA
TTGGAGCAAACTCTTGTTCCTCTTCCTCTGAACTTC
>TCONS_00069775
GTGGACGCTGAGCGGACAAGTTTTGCTGACCACTCGTCATCACAGATTCACAACTATTGTTGTCTGTGGTGGT
AAGGGCTGCAGTTTGAGTCTGTTGGCGGAAAAAGCTATTCTCATACACCAACTGTGAAACTTGCTTCTGCAGC
CTATCATTTTCCTCCATCAACAGCTTATTCATTGCTATCAATTTTCTGTTCACGGCTTGAAGACGCAACGATTCT
TTACGTTGCTTCTCTCTACATCTTCTATTCTGAAACCAAACTTTGATCTATTTTGGCTCGATGTTGGACAGAATT
GGACACTCCCGAATCAGCTGCTGACGACGGAGTGAACTGGGCTTTGGACATTCATGATATAATCTCTCCAAA
GCCTCAACCTGCTCAGGAGTGTACCTCACATATTTCCCATTGTCCATCCCCATTTTACTGTCTTTACAGGCCGA
TGTCACCGCCATCATCACTTCAATAAAAATCAATTTTGAGAACCAAAA
>TCONS_00069839
TCATTCTTAACGAATAGGATGTTTCGCCCTCCTTGGGTTTGTTAAAATTTCCAGCCAGACCCTCAGAGCTGGGC
TCATAGGACCCTTGAAGATTATAATTTCCATTTGGATTTCCACTTTCTTTTGAACGCTTACTAGATTGAGGCGG
CTGATGGTTGTGCTGGCCCTTATATATAATCTCTGTCACCTGGCCATCCAGGGAGCGCTCAACCTTCTTCTTGA
CTGGACAATTTGAATGTGTACACTTGTAATAGCTTCGAGGATATTCACTTCCCTTGACCTGCTTCTGCCCATAC
TTTCGCCAGTTGTAGCCATCATCAGCAGGTTTGTCAACAGCAAAGGAAGCAGGTTCTGACCTCTGATCAGATA
GGGAAACATCTGATGACTCTTTCACAATGTTAGGATTTAATGCAGGAGGAGGTATCTGTTGGTTTGCTGCTGC
ATTTGATGCGAAGGAATGGAACTGTGACAGTGCCGCTGCAGCTGCTGTTGAAGAAGACGGATAGTCAGGCCG
AATGTGCAGTTGAGACTGAGCCTGGGCTGCCTGAGTTGTAAATTGAGCAAGCACTTGCTGGCCAGGTGAGAA
GAACCCATCAAACAAACCACCTGGACTCAATCCAGGCGGTACAGTAAACGTCGTAGGCGACTGACTTATCAC
CAAACCCGTTGGCCTATTCTGCTTATACTCCTGTGCCGTCGCCGCCGTAGACGGCGGAGGAACTGGCTGGAAA
CCCGGTCGAACGCCGGAAAACCCCGCCGGAGATGACATTGCACCGGCGAGAAGCTGAGAAAAAGAACGAC
AGTCCGAATCCGGATCATTATCGGAGAAAAAACTAGATACTAAGGTCATTGGACCCGGGCTAATACCACTT
>TCONS_00069905

ATAGGCTCACAATTTCCGCGAGTACCAATGGTAATGGTAAATGGATTCAAAAGCCATACCATCACCGAATAG
GTGCACAATTTTTCAGGAACCCCTCTTAGCTTTAGAATGTTGTTGATAAGAAATCCCACGAGAAGATCTGCCG
CACGAACTCAATTGTAAGTTTCAGTAAGCATATCTGAAGCGGAGAAGATGAATTTTCCCCAAGAAGGATGAA
TGAAAGAATTGGGCACAAGAAGAAAGGCAATTAAGGGTGAGTAGCGATATGTGGATCTTTCATAAGGGGAT
TTTCCAGCAGCGACTAAAGCAGCTGCATCTGAGAAAACAAGATAGTCCACATCTGTGTATCTAACTTCCATAT
GCCTATCTTGCCATTCTCCGTAAACTATCAAAAATACCCTAAGGAATGCTGAGAATATGAGCAGAGACTTGA
GATTTACTGTCACCAT
>TCONS_00069930
CTGCGAAGACTCAGGCCCTAGACTCACTTCAATTAAAATGCAATCTACTTCTTCTACAATTACACTAACATCC
ATACTTTAATATTCCCTCACGCTCTCTCTCACTCAACACTCTTGACCCAAAAGAAAGAAAAAAACATTTGGAG
AAATAAGGAAAAAGAGCTCAATTTTCTCGATCTTCCTCTCTGTCAAAGGTTACTTACCTTCTCAAAAAAGAAC
AAAAAGAAAATAAAAAGGAATTTGCGATGCTATGTGATGGAAGAGCTCAACATGGTGCTTGAATCATTCTAC
AAGCAAAAAACATCATTTACCAGTAATAACAGCCGGAAATGAGGAGGACTAGAGATTATTTTTCTCAATTA
AGTCTTCCTATAACATACTTGAGAGAGTGTGAACTTTCCTTGCACTTCAATTTGGGTTCCTAGAGCTTCAAGGA
AAATGTTTTTCTTAACTTGAAAAACAGCATGGAAAGCCATCCTCACTGCTGAGTTTGAGGACAATTTATAAAA
GTTGGTGTTTCATATGCTAGAATATGGTAGAGAATGCTAAC
>TCONS_00069951
CACTCTTTGCACATAATCAGGGCGTCGTTTGCTTGGCATGCGAAGCAATCCTGTAATCTGCAGCTCCATGACA
GCCCTGCAAAACCTTGCTTGGCTCAATGCATCAGAATTGTTTTGAGAACTGCTGGTGTCCTTTCTTTTCTTTGGT
GAAGCCAACTGTTTCAATCCTTTCTTTCCTGAGCTAGAAATAGTTGCTTTAAAAGATTGAAACCAGTCATGAA
GATTGATGAGATCACCATGCTCCTGCGCCAGAGTATACATTATTGAGGTGTCATGCATAGATGGTACTAGTGT
ACCCACACTCTTGCTGCAGCATCCACACTTGAGAATTTTGTGGAAATCCAAGAGATCAATTTGAACTCTTCTT
CTCGGATCACCAACCAAAGCTGATTGGAGTTTGTCAACATCCCTGAAGCAAACAATTTCATGAAATGGGACA
CACTCAATGGGCTGCATATTCTCCCTTATCAAGTTAGTTGCCAAAGTCATAGCTTTTTCACTTGATGTGCATGC
ATTTAGACTACGTCGATTGACATGCCTCTTCAATATATCTATTAATTCTGCTCTGCGACTTTTG
>TCONS_00069952
GCACCATCTTCTGAAGTTTGGATAAGCTTGGATGTCCTAAACGTCTGTGAATTAGGTCCGGAGGATCTGTAAC
TAGACATGCCTTGGAGGAATTGAGTGAGTTAAGGTAGTAAAGGCCTTCTGATTCAAGTCATGTTCCAATCGTC
TGTCCCGTACTGCGGTCCTGCATAATAAAAGAATCATCAATAAAATATATACCACAATGGAGGGCACGAGTC
AAACGACTAACAGATGCAAGAATAAAAGGACAACTAGGGACATAAA
>TCONS_00069953
ATTTTGCGGATGACCTGATTTGTAAAATCAGCCTTTTTCTGTCTGTAATGGTGATGATTATTTGACAACAAGCT
CATGCCTTTTGCACTTTCAAGGTTATTATCAGATGACCTGGAGTTGTACAAGCCAGGATCCAATGCCTCCCAA
TACAAGTCCATCAGTGCAACTCTATGATATCTTCCAGCTTCATGCAAGCACATAAGGAGAGATCTCCATACTT
TCTGCACTCTCTGCAGTTCCGATAATCCAAGGGCTAGAGTCATAACATTTGGTTCTACATGATTATTCTGTTGT
CCTGAAAAAGTCCTGTACGATGGAAGATCGAAAGCATGCTTAACCAATGCCTCTGGCAATTTAGCAAGTTCC
TCACTGCACGACCTCTTATTATCTCCGTCATCAACTGAAAACTTGAGCATGAAGCTAAGAGGCTCAACG
>TCONS_00069960
GTCCTTTTATTCTTGCATCTGTTAGTCGTTTGACTCGTGCCCTCCATTGTGGTATATATTTTATTGATGATTCTTTT
ATTATGCAGGACCGCAGTACGGGACAGACGATTGGAACATGACTTGAATCAGAAGGCCTTTACTACCTTAAC
TCACTCAATTCCTCCAAGGCATGTCTAGTTACAGATCCTCCGGACCTAATTCACAGACGTTTAGGACATCCAA
G

>TCONS_00069963
ATTTAATGTTGGATGATAAAGAAGACATGTGATTGATGCTTTTGCTTGAATATGTTTCGTGGTGGTTTTACATT
CACATTAGTAACCATGTGCAGATCAAGTTGCTTCAGCTGCATCCCTTGAAGTGACAAAATCTTATAACTTTTTT
CACCTATGATTGTTAACCATTTCAGAGGTCGTGCAGTGAGGAACTTGCTAAATTGCCAGAGGCATTGGTTAAG
CATGCTTTCGATCTTCCATCGTACAGGACTTTTTCAGGACAACACAGGAATAATCATGTAGAACCAAATGTTA
TGACTCTAGCCCTTGGATTATCGGAACTGCAGAGAGTGCAGAAAGTATGGAGATCTCTCCTTATGCATTCTTG
AAGTGCTTCAGATGGGTATCATGCTACCTCAAAGTTGCCCAGTTGACAATTTATCGAAATACTGTAGTTTTGG
AGTCGAAACCTTGTTTTCTAAGTTGAGTACTTCAAAT
>TCONS_00070085
AAAGAAACCAGGTGAACCCCTGATATTATTATGTGAAATATCTAAACTTTCGAGCAGCGAGTGCTTTTCAAAT
AGGCAGGATGGTACGCTTCCATCATCCAGAAAATTATAACTCAAATCCAACCTTTTGAGATTCCACAGTCTAC
ATAAAGCTTCTGGCATGATCCAGTTGCCAATAGCATTGCCAGATGCATACAGAGACTGAAGATGTGTGAATC
GAGTCAACAGGATGCCATATTCCAAGTCCAAGTTGTCTTTTATTGGTAAGAAAAATATCAAGAACACGTGAA
TCATTGTTCGGATCACTGCCACAAAAAATACCAGCCCATGCACAATAGTCTTCTCCAACCCATTCATTAATTA
AAGCAGAGCCATTGGGATAGTTAAGGGAGTCCCTCAGCTCTAAAAGTGCCCTTTTTTCCTCTTCCATTAATTTT
CCGTCGCAACCCAAGAAATTCAAGCACAACAGTATCCAG
>TCONS_00070144
ATGAATGTAATCTGGTTCTGGTCGATGTTGCGTATCAAGAGTACATTCCCAAATGAAGGATCCTTTCATCTATT
TTCTGCTTGCTAGAACTGATTGGCCCGTCTTATTTATAAGTTGGGTCCGTGAAAAATCAATGTGGCTGAATTGC
AAGATGATACAGCAGCTGTTGATGCTGTTATATTTGCTGTTGGTTGTAATTTTGTTGCTGGTCACAAACTACCA
TTTCTTCGCTGAATGGCAAGGAGATGCTGCAGCTGTAGATGCTGTTTTGACTTGGTTGATTATAATTTGTTGCT
GGTGACATACTACTGTTTGTTGGTTGAATGGCAAGCATATGCAGCAGCTGTTGATGCTGTTTTAGCTTTGTTGT
TGTAATTTGTTGCTGGTGACAAAATACCATTTCTATACAAATGTTCAGGATTCTCATGATCGGTAAGGATCAA
AACTCCTGAATAGCTGAAGTCATGCTAGGTTTA
>TCONS_00070148
GCTTCTGCTCTTATGACGGTAATCCTTGTCTCTCCCACGGTACCTATCACGGTCATACCTGTCCCTACTTCTACT
TCGGCTTCTCCTTTTAGAATCCTTTTCCCTATAATGATCACGATGCCTTGAACTAGGACTGCGGCTTCTTGACCT
TCCTCTTGTACTATGTACAGGCTCCAAAATTCTACCTTTGTCAATTCTTTCAGCATTTGGGCCGTACTTTGCAAA
TTGAACCATTATTTCTCGACCATCTACAACTCTTCCATCGAGCTTTTCCACTGCCTTTTGAGCCTCATCCTGATA
CTTGTAGCGTACAAATGCAAAACCCCTAGAATCTCC
>TCONS_00070156
AGAGGTAGTTACTCATAGGCACCATGAAAACTTCCTACTTACTACTGCCTTTAACACATTCTGAACAGCAGAT
GTAGACAACCTAAAACAAATGATGCTTCAAAGAGTTTACCAGGCTAAGTCCGTTCTAGAGTTAGTCATCTTTG
CGATCTTTTGCTGAAACTCCATTTTGTTCACCATTAACATTTGAAGCTCCCTTTAACTGCTGGAGGACCTTCTTT
TGATAGCGATAGCCCTTATCAGTTCCATAAATGTAATCGCAGTAGGTGAAAACTGAAGCAAAATTACTCTGG
CTTTGGCCCCCAACGTAGTGATGGTAGTCATGGTAATCCGGTCCACCATAAAAAGGTATATACTTTGTGATAG
TCCAAGGAAGGTCATATCCACTGTGAGTCTCAATAGCTTCAATCTGTCTCAACGCGATCCACAACCAGAATGT
AACCATATGACCAGGCGCAATAGCGGGCCCAATAGCTGAAGGAATCCCGAGGATCAAAATCTCTAGCCAAT
GCGCATACGGCGCTGCAAAGCCTATTGGAGCTGTGTATTCGTGGTGAACCTTGTGAATATTTTCATAACCCCA
TTTACAATGCAAGAACCTATGGATCCAATAGTTAAAATAGTCCTCCACTATAAAATATGTTCCCAACTGCAAC
AAAATTTCCCATAACGGCGGCAAGGGCAAGCTTGTTCTAATCCCAACCATCTTAACAGAGGGGTAAGAAACA
AGCTGAAGGGGACCCACAACAAGGATGAACATACGCATAACAGACTTATAACAATTGAACACCTCAGAGAG

TGAAAGATTGACTTTTGGTTGAATTTTATAAGACTGAATAGAGTTTCTGAAGAAAAGTTCAAGGAAAAGGTA
ATAAAGAGGGACACAAGAGAAGATTAAGAAGAGAAAAAGGATATTGTGGCAGTAAAGATAGTAATCAGAT
TTGGTTGCAGAGTAATTGAACCAAATTGTCTCTAGAGTTGTAAGGGATCTTCCAATGGCAGATTCTGCCTCTTG
GATTGTTTTAAAGGGCAACATTGAAAAGGGTTTGTAGTAAAATGGGGTTTGAGTAGAACTGTAATGGAATCTT
GGATATTGGCTATGG
>TCONS_00070159
CTCCATCTCTCTTTCTCCCTCTTCCGCCATAGCCAATATCCAAGATTCCATTACAGTTCTACTCAAACCCCATTT
TACTACAAACCCTTTTCAATGTTGCCCTTTAAAACAATCCAAGAGGCAGAATCTGCCATTGGAAGATCCCTTA
CAACTCTAGAGACAATTTGGTTCAATTACTCTGCAACCAAATCTGATTACTATCTTTACTGCCACAATATCCTT
TTTCTCTTCTTAATCTTCTCTTGTGTCCCTCTTTATTACCTTTTCCTTGAACTTTTCTTCAGAAACTCTATTCAGTC
TTATAAAATTCAACCAAAAGTCAATCTTTCACTCTCTGAGGTGTTCAATTGTTATAAGTCTGTTATGCGTATGT
TCATCCTTGTTGTGGGTCCCCTTCAGCTTGTTTCTTACCCCTCTGTTAAGATATGACCTTCCTTGGACTATCACA
AAGTATATACCTTTTTATGGTGGACCGGATTACCATGACTACCATCACTACGTTGGGGGCCAAAGCCAGAGTA
ATTTTGCTTCAGTTTTCACCTACTGCGATTACATTTATGGAACTGATAAGGGCTATCGCTATCAAAAGAAGGTC
CTCCAGCAGTTAAAGGGAGCTTCAAATGTTAATGGTGAACAAAATGGAGTTTCAGCAAAAGATCGCAAAGA
TGACTAACTCTAGAACGGACTTAGCCTGGTAAACTCTTTGAAGCATCATTTGTTTTAGGTTGTCTACATCTGCT
GTTCAGAATGTGTTAAAGGCAGTAGTAAGTAGGAAGTTTTCATGGTGCCTATGAGTAACTACCTCT
>TCONS_00070165
GATTGCATTAGCGATGCAGCTTACATGGTTTGCCCAACTCGCCATGGACGACACAAATTAATTGAATCCTATT
GCGACAACTGTTGCACTTTACCTCCAGTTGCTCGTGCAGTCGCTTTTGCACCTCCATCTGCAGCTTTAGAGCCG
TAGTGATTTCCACTCCTGATGAACCATCCAAACTTGATAGCATATCCCTCGATTCTTTCTTATCAGGTTTTTTCC
CATCAGATGAGGAATCTGGAAGATATTTAGCAAGTCGATATTTCTGTAAATGGCTTTTTACATGGTAAATGGT
TAGGCCTTGTACACCCATGACTCTAAGAACGCCTTTCGGTGTAGCTCTATCCGGGCCACCAAGTTGTGCGACA
GCATCAACAAATCGTTCATGAAGCTCATTGGTCCAACGCAGACGTTGCTTTGAAGCAAGACTAGGATTGTTG
ATGTTATTGCCTCCACTGGTCGGATCCATTGAGCCGTCATCACAGTCTAATGACTGACTATGCACTGCTGCATT
GTTGTGGGCTAAGCTTGAACCTGGAACGCCTTTGGGCTGATACATTTCATTTAT
>TCONS_00070217
TTTAACTTTAATTTAAGGATGTCAAGCAGAAGGTCAAGACAATCATCAGCAGGTTCCTCAAGAATTTCAGATG
ATCAAATAATTGACCTCGTATCCAAGTTGCAACAACTTCTTCCTGAGATTCGAACTCGTCGCTCCAACAAGGC
ATCGGCATCAAAGGTGCTACAAGAAACTTGCAACTATATAAGAAATTTGAATAGAGAAGTGGATGATCTTAG
TGATCGTCTTTCTCAATTACTCTCAACCATTGATGCTGATAGTCCAGAAGCTGCAATCATTCGGAGTTTATTAA
TGTAACTATTAATAAATGTATTATGAGTTTTAAATATTGGAGTTATATTTTACTGCGTACCAATTAAGTTCTTGT
TTTTCTTCTTAATTTCTCTAGACTCAGCTGGTCACTAGCTAGGCCAATCATGAGTTAGAATTTAGAACTGAAAT
ATTAGTAATATCCC
>TCONS_00070244
CAAAAGTAGGCTAAAAATGAAAAAAAGGACAATTTTAAAATATGCCCTTTTCATTTTTTACCCTAGAGACCA
CACTAAAACCTCCAAAATAGACATAGTCCTCCTATATACTCTATTTGAGTACACCCATTCTATACTAACAGTC
ACAATACTAGTACTAGTGACCAAATTAGTATACATAATATTAATACTAGTATACTACTAATATACTTCAAACT
TAAAACCCCATTTAGCTAAAATATTAAATTCTTGAGCAGCCAAATTTTTTTCAGTGGAATTGAGCTCCAGTAT
AAGTCTGTCCAAAAGACCAATGAGCAGGTGCAACATTGTAGGAAATCAAACTGCGGCCATCACCTGTAGTA
ACCTTAAATGAGAGGGCTTGGCCATTAAGATAGTTGTTGTTTTGCCAGTTTTGTCCCCAGTTTCTTGACATTGG

TTGCCAACCAGTTCTTGATCCTTTTACAGCTACAGCATGAACATCACCACCACCACCAACATTTGTTACAAGT
ACAAGGTTGAAATATGAGTGTCCATTGATTGTGAACCTGATACCACC
>TCONS_00070251
GGACATGAAAGCAAAAAGATACACATAAAATAATAGCAGTATCAGCGAGAAGTTAGGGTTTTTATTCACATT
TGCACGCAGACATTTTTATTCCTTCAAAATTCCACTTTCAGTCTGCAAAAATTCCAATCATCAAAACCCAATA
ATTTAATCAATTATTGGTGATTGTCAAGTAGTGAAGAATAATACTATTATCAACACTTCGTGCATGGTATTT
>TCONS_00070296
TTGAGGTCATATCTAGTATTCAGAGTTTGCCTCGATTTGGTACCGCTCTCGCGGCCCGCACCGAAACAGTGCTT
TACCCCTAGATGTCCAGTCAACTGCTGCGCCTCAACGCATTTTGGGGAGAACCAGCTAGCTCTGGGTTCGAGT
GGCATTTCACCCCTAACCACAACTCATCCGCTGATTCTTCAACATCAGTCGGTTCGGACCTCCACTTAGTTTCA
CCCAAGCTTCATCCTGGTCATGGATAGATCACCCAGGTTCGGGTCCATAAGCAGTGACAATTGCCCTATGAA
GACTCGCTTTCGCTACGGCTCCGGTGGGTTCCCTTAACCAAGCCACTGCCTATGAGTCGCCGGCTCATTCTTCA
ACAGGTTGTCTCTTGCCTGCCCATGGATTCAGCAGCAGTTCGAAAGGTTGCCCTATTCGGGAATCTCCGGATC
GATGCTTATT
>TCONS_00070375
GCCAAGTTTTCCCATTCCATTCATAACAAATTATTAACAATCATTAATCAAAAACATTTGTTGCTGAAAAGGG
TCACTGGTTCCAAGAAGTACAACAGTTAAAACATAAATGCGAAAAGGTAGTCACCAAGTCACTCAACATAA
GCCAGAAGTAGTGAACTGTGTCGTCTCATAGAAGGTAATTTGCCTGAAATCAGATGCAGCCACCCAACAAAA
TGGAATCAATTTCCTCCTGAGACACGGGTGTTCTTTTGGGTTGGTCAGAAGCCTTCCCTGCAACAGACATGCT
AGATGGGGACCTTGAGAAAAATGTTTGAGGAGCTTCTTCAGCAGCTGATGTTTTCTGATGTTGGGAATTCTGG
GATGTGGAACCAGAAGGCTTAGAGTGCCTCTGTGGAAATTTGATGCGTGGGATTCTCTTCATTGCTTGCGCTC
CGCTCATTTTGCTCTCTTCTTCGATTCGTCTCTTCTATCCCTTCCAGGGTATAAGCGAAAGGTATAAAGACAAA
AGCTGTTGAAATTAGA
>TCONS_00070432
TCTTGAATTCGATGTCATGAAGTGAAAATGTGCCAATGATGCTCTGAGGGTAAAGCTTCTTTAATTTTTTCAAG
TCTTTTCAGAATCTCCAAAAATGAAGGTCTCTGGTTCATATCTGGTGCCCAACACTGCTCCACCAACTCTTTCA
ATTCTGGGGTAAAACCTTTTGCCCTGAAAATAGGTCTGTGTCCTTCTGCCACGTACTTGGCTGCTTCATATGGT
TCATAGTGGGATAATGGTGGATCACCTTCTAGCATCTCATATAAGATCATTGCGAAAGAGAACACGTCGACTT
TCTTGTCATATTTCCGGTGCTTGAAAACTTCAGGGGCCATATAGCGATAGCTCCCCGTCTCCCCCGTCATTTTA
TAAACATCATGGGAGTTCTGAACCCTGATTAGTTTGCTTAATCCAAAGTCTCCAACTTTCAAATGGTCTGCATT
GGAGTTGACTAGAAGAACATTCCTGGGCTTTAGATCTCTGTGGATTATAACATTTGGCTCATTGTGAAGATAA
GTCATGCCTCTTGCTATATCCATAGCAAAATTGATTGCTGTCGATGGACTAAGAGCACCTTTTTCCTTGAGGTG
CTGATGCAGATCACCCCCCCTTAAGTATTCAGTTATTAACATTAAAGGTTTTTTCTCGGTAACAGCTCCAAGA
AATTGCACTATATTTGGATGACGGAGCTTCACCAACAAATTTACCTCATGCCTGAAATCCTGAATCACCAATC
GATCATCTGAAAGGTTTGGGAGGATGCGCTTAATAGCTACAGGTGTTCCGCGCCAGCAAGCTTTTAATATCTC
ACCAAAAGACCCCTTCCCAATGATTACAGAATTTGAGAAGTCCAGCTCAGCAGGATCAATCTCCCAGTCACA
CTTCTTTGGTAGAGGGGGTGGAACAGCCCTTGGTTCAAAAATGGCTTCCATTTTGCCCATAAGATAGTCCACC
ATATGTCTTTAACAATTCAATCATGCCATGTTTCTTAGCTCCTTCTGCATCGGCTAGAGGCGTGTTTCTCCAAC
GATCCTGAGCGTTGACGTCGGCGCCATAATCGATCAAGCACTTCGCAACATCGATCCAGCCATGTAACGCCG
CGACATGAAGCGGAGTGCGATTATCGTAATCCCTAGCTTGAACAAGCGTCCGATCTTCTTCTAGAAGCTTCCG
AACGGCAGCTGCATCGTTATGGTGAGCGTGCCATAGAATTTGCGATGTTTTGCTTACTCTTGACTTCTCTTTCTT

CTTCTCCGTTGACGCTGCTACCGATGCGACAGCTTCTCCGCCACCGGCAGCGGAGCCGGAGCTTCCTTCGCTT
CCGCTCATTTTGGATGGATCGCTGCGTGTGTGTG
>TCONS_00070530
AGCATAAAGAAGATGGGAATGGGAATGCCAGTACTTACTGTGCACATTCAGCTAGTTTCCCAAGATTTTGATT
GTCCAATATGGCCAAAATCAGTGTCTTGTTCTCATCCAGGTACTTTTGAATCTGCTCAGTGGTGATATTGTTAG
GAGGAAAAGAGGACATGACTGGAATCATAGGTGGTGGCTGTTGCATCCTCGTATACTGCTAAACCC
>TCONS_00070585
CACATAACAACTACTAATGAGATTTGTTGATTTAAACACTTATGCTCTAAGCATCATTATACAAAAATAAATC
CGTACAAAATCAATCTTCATCTGAACTCTCATACGCTACTAGCAAGCTTTTCATTGCATTATCTGCCTTTGTTTC
ATTTCCCATGTCAGTTGATCTAGCTGGCAGGGTCTCTTTCTGCTTCTCCTGATTAGAATCAGTGTTATTACTTGG
AGCAGCCTGTGTGCTTGCTGGGGAATTACTCCCATCAGATGCGGTAGAAACTTTCTGCTTCTTAGGTTTGACTT
CTAAAACCCTTTTAAGTAAATCTTGCTGCCTAATCCCACTCTTTGCCTTGGGCTCAACACGAAGAGGCAGGGG
AATTGACGCTGGTGAAGAATCAGCTACAGATATCTTGAATGCTTGGAATGCACGTTTTTCGGTACGTCTCAGA
AGGCGTTCAGCATCCTCCTCGGCTTCCATCTGCTCCTTTAGATAAAGCAAGTCATCACTGTCCAGGGCGCCTCT
CCCATGAAGGTAGCCTCCACGTTCCCCAGCAACATCTTGCATCTGAACATATCAAGCACCCAGTAGGAAAAG
CCAAGATCCCTTTGAAACCATGCGTTGGAAAGTGATTTCCTCGTCATCTATCCTGTCTCCCTTTCCTTTCCCGA
ATTTTTTATCTTTAGGATCGGTGAGATTGGTGTATTCTCGAACTTCTCTGCCAGCCATGGCTTCAGTTAGATTGA
TTAGAAATTGGTTGCTACGTGCA
>TCONS_00070586
CACATAACAACTACTAATGAGATTTGTTGATTTAAACACTTATGCTCTAAGCATCATTATACAAAAATAAATC
CGTACAAAATCAATCTTCATCTGAACTCTCATACGCTACTAGCAAGCTTTTCATTGCATTATCTGCCTTTGTTTC
ATTTCCCATGTCAGTTGATCTAGCTGGCAGGGTCTCTTTCTGCTTCTCCTGATTAGAATCAGTGTTATTACTTGG
AGCAGCCTGTGTGCTTGCTGGGGAATTACTCCCATCAGATGCGGTAGAAACTTTCTGCTTCTTAGGTTTGACTT
CTAAAACCCTTTTAAGTAAATCTTGCTGCCTAATCCCACTCTTTGCCTTGGGCTCAACACGAAGAGGCAGGGG
AATTGACGCTGGTGAAGAATCAGCTACAGATATCTTGAATGCTTGGAATGCACGTTTTTCGGTACGTCTCAGA
AGGCGTTCAGCATCCTCCTCGGCTTCCATCTGCTCCTTTAGATAAAGCAAGTCATCACTGTCCAGGGCGCCTCT
CCCATGAAGGTAGCCTCCACGTTCCCCAGCAACATCTTGCATCTTTGAAACCATGCGTTGGAAAGTGATTTCC
TCGTCATCTATCCTGTCTCCCTTTCCTTTCCCGAATTTTTTATCTTTAGGATCGGTGAGATTGGTGTATTCTCGAA
CTTCTCTGCCAGCCATGGCTTCAGTTAGATTGATTAGAAATTGGTTGCTACGTGCA
>TCONS_00070666
TAGGAACCTACTGATCTAAATATTTTGATATATGTACAGAACTAAGAATAATACAGCTATACACAGAATTCA
CATTAACATACTTGCACCTTGAAAAAAGAAATTCAAGGAAAAGCTTACATCAACTTTCCAAATCTTGTTCTCA
ACCTTAACCTTATCGGCACTTGGACCAAAAATCTGCCTCAGAAGTGCCATAATCAGAGTTTTCTTCCCTGACC
CGGAAGGGCCATAAAAGAGAAGATGAGGACAATCTCCCGCTAACACCAGCTTTTGAAGATTTTGAGCAACA
TCCTCGTGGACAATAACTTTGTCTAGGGTTTTGGGGCGGTACTTGTCAACCCACAACATCTTCAAATTTGAGCC
AAACTACTCTAAGAGAATGAGGAAAACAGGAGCCTGATTCGCGACCAATTTTCCTTGG
>TCONS_00070667
CCTCAACAGGGGCCTAGGTGGCTCTAACAACTTATCAGTACCATCTTGAGCAATTTCTGTAATAAATATGCTG
TTCATTGCTTGCGGTGAGGAGTCAGCAAGTTCTTTAAGGCCCATTAGACTAGGAATAGAGTTGCCCACTGTTT
TAAGTTGTTCCTGCAGTGTTTCAGGCATCTTCACCCTTTTCCCGCTGATAAAATCGCACAGGTACATGACACCG
GCATTTGTAAGGTCCTGCTTGCCTTCCAAGGATAGTAGTTTGCCATCCAGACCATAAACCAGCCCTTTTAATTC
ATCCAAATCACAATCAATCTTGGATAGATCACCACGCACACCATTAACCTCATTCCTTAACAGTTTTGACATC

TCCATTTGCTCATCCAATTTCCCATCCACGTTCTCAATTCGCTGTGTCAAATGCTTCTTTGTGGCAGCAAGAGC
ATCAGAGACATGCTCCAGATGTTTTGTCAGATTTGAAACAGCAGTTGCCATATTCTTTTTCGTTACATACATAA
GGTCCGAGAATGACAGACCCTTCCACCACATGTATCCATAACCTAAGGCACCCACTGCAGCAGCTGGCACTA
TCAGAGATGTCCAGTCACCACTAGAACTGCCATTTAGAACAGTAATTGGTCGTGCCGAAGCCAACTGTCGAA
CCTCCATTGCCAACCTACGAACCTGTGCAGCAATGACATCCGATTCTCCACCATCTGATTCCCCTGATTTTTCA
TAACTTTTGACCAAATTCTGAAGCTCGCCTATCACATCAGATAATTTACCGTTTTTGAAAAGCAAAGTACCTG
TATACCCTGCTCCGACAATAAATACGATCTTAGTTAAGCCTACTCCAGCTTGCATAGCCATGGCCGGCGTTAG
TCCGGTGATCAACAAATCCTAGGTCTTTTTATGACTCTCCGGCGAG
>TCONS_00070734
CTTGGGGGACAAAATTATCTACCTGTGTTGGTGGGAGCTAGTAGGTACCCCGTTGAATTGTTGAGGTCGCACA
AGCTGGCTGGGACACACAGTTAGACAAAAAAGAGATAACATATCCTGTTTGTAGCCTACTTATTACTAGACA
TCAGCATTGCTTTCAATTCATTGGTGTTCAATTCCCTCCTCTATCAGATGCTGTTAGAAGCCCGCTAAAATACT
GTCAATTCCTAGAAGGGTACATTTGACAAGAATTATTGC
>TCONS_00070756
TTTGAGAATAGGAGTTAATCCAGCTTGAAATCCTCAAGCCATATAAGAACTTCATTCCTCATTATAAACTCCC
ATGTGCGACCAGGATCATTGTAGCGAGCAAGCATACAACCCGATTGAGGTTTGAGACCGTCCTTAATAAGAC
TGGATCTAAGTTGTTTCTCCTTTTCGCGAACAATATCATCTGTTGGCTTTCCACTAAATTTTAATGCAGCCGCG
ATTCCTCCTTCTGTCTTTCTTAAGCTAATACCTTCTTGATTGGGAGCTGGTAAGCTGTTCAAAGATTTATCCGAC
GGAAGAACTATCTGGATTGACACTTTAGATTTTTCAGCATCAAATGCCTGAGTGAAGACAGGAGTAGTCA
>TCONS_00070930
TACTGAGAAGTAGCACTTTGATGAAGGCACCCACAAATTAGAGCTACCAGTATCAAAGATGACAGTGAACTT
CTGAGGGGGAGTGCCTATACCAATCTCCCCAAAGTACTGAGCATCCATATAGTTCTTCAGTGCTACAATGTCT
GTATCCTCAGAGGCCCCAAGATTACCACGGAAGTTATTATACTTCCTAATGGAGGCCCTCAAAGCCTCCCCCT
CCTTGGACTCGACGCGCGCAGCAAGTCGATTGTTTTGGTCAAATTTTATCTTTTTCAGTCCAATTCTAACCAAG
CCATCATTTGACGTAGACAAGGCCAAAGGAAATAACAGCGATGAGAGTAAAATGGTGACAAGAAAAGCTTT
TGCTCCCATATTGACCAGTTTGATGAAGCAAGAAAACGGAGAGAATGAATAAGAATAGAAGTTTGTAGATGC
TTTATTGCATGAATAAATAGGAGAAAAGAAGAAAAATGGAACCAACTTTCAGACCCACTAAGCTGAGATCA
GC
>TCONS_00070984
CAGATAAGGAATAAGTCATGAGATTTTTGCAGGAAAGAAGTAATAGATTCTATTTGGTAGATTAGAGAAAAA
AGATGTTCCCACGGAGGAGGGAATACAAAATTTACCATTCTTCAACATAATGCTTTTGGAGAGGTGGTTGTAG
TGATTCAATCAAGAAACTATGAGCTATGGTCAAGGGTATTGGTTTATTTATAGAGCTTGAATGTATAACTTGA
GGAGACATGACATTTTCGCATGGGGTGAGCCTATGGAAGGGATTTTGAAGTGAAGGATGGATTCACTACATA
CTGGCTACACTATTGAAAGTGGAGACAGTATGCTTTTGAAAGGATTTGTGGTATGATGGAAAAACTGAAGAC
GCATACACAAAGCTGTTATATATGCTGCATATGCAGAATTGTAAGGCTAGGGTGACCTATTAGTTAAGAATG
GAGGACAAGGGAATTATGTGGGACATGAGTGTTTGAAGGAGACTTACTAGTTTGCAAGCGAGATGAAGTGG
AGGAGTTTCTATGAAAAGATCTGCAGATATGGGAAATGAGGGAGGACAGAGATTATGTGAAGGAGATTACG
GATGACAACATTCATGGTGAAATCACTCTCCTAGAGCTATTAGAAGGAAGGGAAAGAGGAGATATGGGGAA
AAGCCTTGAAGATGAGGGTACCAAAGTAAGTTCTTTTTTCAAAAAAAAATAGCACGCAATTTGTGATGCATC
ATAAATCGCGGATAAGCTTAAATAGATGAGACAAATAGCTGGCTTTACCTCTCTTCTATGGACTTTGGTCTAC
TCTGGTGAATAATCTTGATTGTCGCTAAGGATGTTCCATGAGACAATGTCGACTGGAGATTAGAGAAAATCG
GCATTGAGAAGTATGTGATTTGGAGCACAACACTGTTATGTCTCTTCTGGCTTGTGTGGAATGAAAGTAGGAG

ATTGTTCCATAATAGCAGGAACGACGTAAGAAGTGTGAAATGTTCTTTCTTTTTCATGTATATTACTGGTGTAA
AAGGAGAGGTCCTTTCTATGTAGGAAGTTGGGCCAACTCAAGGCTTCAGATAATTGTGTTCAGCCACAAAAG
CAGTATGTAAATACTTCAAAGACTAGCTTAATGTACTTAAAATGAGATTTACTTGACTTAAAAGATTCCGAGC
CCATAAAGGGAGTCTTTGGCCTGAATTGTTCTTATATTCTGAATCATGCTAATGCACTCTTTTGGTTACTAGATT
GGTACTTGTATTAGATCAATACTCACGGGGCTGTAGGCTGAAGTGCTGAACCATGTCATATGATTTCATCCTA
TCTAAAAATCTGGAGCTGAGACTGGTGATCCACTTCATTGGAAAGAAAGAGATTTGCAAAAACCCATTATAC
AGCTGAGCTTGCAAAGACAACTTGGCC
>TCONS_00071037
GTCTCAACAGGACAAGTATTCATTGGGAACCACTTGGAACTTCTACTCAGTAGCTTCCACGCGTTTTGCAGCA
TCAGCTTCTTTCAGCCTTTCCAAAGCAGAAATGCGTCCATTCAGAGATTCATAAACATCATTCATCTGTTGAG
AGAGGGAGTGGTGGGCGGTTTGGTAATCCTTGTGGAGAACGTAGAGACCAACGGCGGACGCCACCGCCGCA
CCGGCGAAAAACGATGCAAATCTCACCCTTAGCACGTATCCCATTTTTTACTGAATTGAGAATGTACACCAA
AGCGAAGCTGTGTCCTTTTTTGGGTATTTAGGGAAGGAAGGGGAAGAATTGAAATTAG
>TCONS_00071041
CTTTCTAATTTCAATTCTTCCCCTTCCTTCCCTAAATACCCAAAAAAGGACACAGCTTCGCTTTGGTGTACATT
CTCAATTCAGTAAAAAATGGGATACGTGCTAAGGGTGAGATTTGCATCGTTTTTCGCCGGTGCGGCGGTGGCG
TCCGCCGTTGGTCTCTACGTTCTCCACAAGGATTACCAAACCGCCCACCACTCCCTCTCTCAACAGATGAATG
ATGTTTATGAATCTCTGAATGGACGCATTTCTGCTTTGGAAAGGCTGAAAGAAGCTGATGCTGCAAAACGCGT
GGAAGCTACTGAGTAGAAGTTCCAAGTGGTTCCCAATGAATACTTGTCCTGTTGAGACATCAAAATAGATTCC
CTAGGTTCTAGTTGCTTCTGACTCCGTTATGATGTATTTGATCTTCTGCATAAAGTTTAGCTTTTTCTTTTGTTTT
GCCGTATGATGGGTCAAAAGGCACGTTTTCTGGAAGCGGTAATGAGAACTTGAAGTCAATTTTGATCCTTGGC
GTTTCTGAACCTATGAAATGTACTCTTGCCTCTCTTTACAATAAGATTCAGCTTGAACAACTAATTTCTCAGCT
AGCTATTAGTTGGATAATCGTAGGCTAAAATGATCATTATTGGTGATTCTGGATATACCTCGAATCGCACTTG
GCAGTTCTGTTTGGGTAAATATTACTTAAAGTATTTGCTGAGTTTAC
>TCONS_00071053
AACCCGCTTTTGGCGATGTGTAGCTCTGCAACACCAGCACTTTGATGGGCATGTGTAGCGTACTTCTGGCGTT
AACCGTTAACCCTTGAAGCCAGTTCACTTTTGCTGTCCTTGTTTCTGCTAATTTGATCTTTATGAGACAGTTTAC
CAGGCTGCTTAGCTGTGACCGCCCAATAAATTTTGGAACATGCTCTGTTTTGCTTTGGTCTCTGCTTTTCTGCTA
TCCATGTTATCGTCTCTCTGCCTGCTTGCTGATAGGCATTGTTCATAAACCAGGATATTACGTAATATGTATGC
TGACAAGACAGGAACTCCTGCTCAAGCACGGACATCAAACTTAAATGACGAGTTGGGGCAGGTTGATACCAT
CCTCTCGGATAAAACTGGTACTTTGACATGCAACCAAATGGACTTCCTAAAATGCTCCATTCCTGGTAATGCA
TATGGAACACGAGCCAGTAATGTTGAACTTGCAGCTGCAAAGCAGATGGCAGAGGACCTTGGCGGGTAA
>TCONS_00071154
TACTGCTACCTTCTTAACACTTTTCTTCTCAGTTTTAAGAGGGGGTGCCACTTCAGGTTTCTCAATTCCTTGTAT
ATCCGTGATCCTCAATTTTGTTAATTCCTGTCGATGCCCTCGTGTTCGCCGATAATTCTTCCGTCTCTTCTTCTTG
AATATAAGTACTTTAGCATCTAATGCATGCTCCTCAACGACAGCATGAACAGCAGCATCCGGCAATATAGGC
CTTCCTATGATTGTCTGA
>TCONS_00071245
GGGAACGTATTTTTTTGATTGTTGGTAATGTGGGGATGTATTGTCTTACCTTATCACATCTCTCTTTTCCATATCT
ATCAATAAAATCCTTCCTCCAAGAACTAGCACTGTAATCTGTTGTTAGTAAGGCTCCAAGTTAATAAATCGGC
TAAGACCATAATAGTATTATGCTTTTGATTTTTCAATTGGCTTTTCAACTTCTATGAACTCTACTGTTGTGTGGG
CCTGACTTAGCTTATTCTCTTGTTTTTGGCGAACTATAATGTCAAGCGCTTATCCAGGAGCACACCCATGATTT

TTTCTGGAGCTCAATACTTGCTTGATTGTGGAGACCTGGAGAAATATCAAGTATCACTGTAAGTGGTGGATCT
TGAGGAATTTCTACAACAATGTGAGTGTCAAGTTTTCATTGACCAGATATATCATAATATCACCACGAGACGG
CAGGGCCAAGATATTTTATATTGTCATGGTCCAAGCATGAAGTTGTTGAAGTCATTCATTCAGAGCCCTAGGT
TTTAGCTCAATCTTCACAAGATGAACTGAGCAAGAGGGATAACTGACCGAGAGGGAGGTTAAGGGGACTCT
GTCCTGAAGACTGAGGGGACTAACATGAAGTAGTGGTCAGTGACATGAAAACAAGGCTCCTTTTTTTGAATG
GCAGTGGTGAAAAACTACTGGTTTGGTCCTTGAAGCAGCTATTAGTTAAACAAAGATTATCGAGGACCACAG
TTCAAATTTCAGTGGATTCAAAAAGCACTAGGTGATTTCTTCCTAACTGTCTTAACCTTGTTGG
>TCONS_00071246
ATAATGTCAAGCGCTTATCCAGGAGCACACCCATGATTTTTTCTGGAGCTCAATACTTGCTTGATTGTGGAGA
CCTGGAGAAATATCAAGTATCACTGTAAGTGGTGGATCTTGAGGAATTTCTACAACAATTTTTCATTGACCAG
ATATATCATAATATCACCACGAGACGGCAGGGCCAAGATATTTTATATTGTCATGGTCCAAGCATGAAGTTGT
TGAAGTCATTCATTCAGAGCCCTAGGTTTTAGCTCAATCTTCACAAGATGAACTGAGCAAGAGGGATAACTG
ACCGAGAGGGAGGTTAAGGGGACTCTGTCCTGAAGACTGAGGGGACTAACATGAAGTAGTGGTCAGTGACA
TGAAAACAAGGCTCCTTTTTTTGAATGGCAGTGGTGAAAAACTACTGGTTTGGTCCTTGAAGCAGCTATTAGT
TAAACAAAGATTATCGAGGACCACAGTTCAAATTTCAGTGGATTCAAAAAGCACTAGGTGATTTCTTCCTAA
CTGTCTTAACCTTGTTGG
>TCONS_00071403
CCTCCTCCTCCATACCACTACACTTCACCTCCCCCACCCGTAAAATCACCTCCCCCTCCGGTATACATTTATGC
TTCTCCGCCACCACCAGTCCACTACTAAGCAGTTTGAAAAGCTTGGCCATTTTCCACAACCACGCAAATTTCA
GTTTAAATCACAAAATAAGTGATGGCTTCTTGAGGGAAAGATTCAGAGGCCCATGGTCAAAAGCAATTAATT
TAATCAATAAGATCATCAACTTTGCAATTCAGAGGTTGATTCCTAGCGACTTGGACCACTTTGCTTCTACTTCT
CTGGTGGCCTTCTTACACATTGTTCTCATTGTGTCTTCACGTGGAAGTGGCTTCAACAGCTTAGTTTTTTATTAG
AATATTAATTATTTAGTTGAT
>TCONS_00071410
GTGGGGACTTGTATACGTATTTTGGTGATGGAGGAGGTGGTGACTTGTATATATACTTTGGTGATGGAGGAGG
TGGAGATTTGTAATAGTAAGGAGCAGGAGTTGGCTTAGGCTTCATGCATTCTTCATAAGGAGTTTTGGAGGCA
TAAGCAAATGGTTTTGCATAGAGCACAACTTCATATTTATTCTTTGACTTCACTTTGAGGCTAGCACCTTTAAT
GCCCCAGTGTAGATTTGTGGGAATGTCACATTTTGAACCCTTTGGTGCTTTGTGGAGTTTAGCCTTGCATGCAT
CTGCTCCATATTTGGCATAGGCAAATCCTTCGACTGTGATAGCAAACTTGCCATTGATTTTAGTGGTACCATAG
CTCTTGATTTTCTTGTCACCAACTTTGCATGTGACCTCAACAACAGCACCTTTGAGGTGCTTCTTGTTGTGGGA
GTTCTTTGGGTATTTCCAGTCATAGCATCTGTAGCAGTAGACTTTTCCAACAACCTTAACGATCAAGGAATGA
TGAGGAGCATAGTAATGAGTTGGGGGTGGTGGTGAAGTGTAGTAGTATGGTGGAGGAGGTGACTTAACTGGT
GGTGGAGGAGAGGAGT
>TCONS_00071412
CTTCCACGTGAAGACACAATGAGAACAATGTGTAAGAAGGCCACCAGAGAAGTAGAAGCAAAGTGGTCCA
AGTCGCTAGGAATCAACCTCTGAATTGCAAAGTTGATGATCTTATTGATTAAATTAATTGCTTTTGACCATGGG
CCTCTGAATCTTTCCCTCAAGAAGCCATCACTTATTTTGTGATTTAAACTGAAATTTGCGTGGTTGTGGAAAAT
GGCCAAGCTTTTCAAACTGCTTAGTAGTGGACTGGTGGTGGCGGAGAAGCATAAATGTATACCGGAGGGGGA
GGTGATTTTACGGGTGGGGGA
>TCONS_00071453
GTGAAGATGGGATGGAAACTCAGTTTTTTCCTTTTCCTTTCTGGTCTGAATGCCTACTGTCATTATAGAAGAGG
TGTAAACGGCCGGCATCATATGCAGACAGAAAGTGGTGATGGTTGTCAACTGATGCTGCAGCAGTGTTGTTG

GGAGCTGTAGCAGCAGCTATTGTGCTGCCATTGAAGAAACTTTGTGATCCCTCATACCTCTCCCGTCAATAGT
TCGGTGAAAGAAGAATTCTGTGAAATGCGATGCACATTTTGGTGATGTGCAGTTCGAGTCGACTCATTTTCTTT
CTCTTTCTCCTTCTCCTTCTCCGCAGCCCTTTCCCTTGCCCGTTCCCTCGCCTTGACGCGATTTTCTGACCGGGAT
AGAGACAACCCAGAACCTTTGCTTGTCTCAGAAGTGCTACTACAAGCAGATTTCGACAAAGACACATGTTGT
TGCTGTTGGTAATTCGGATCACCTTCCATTTCAACCTCAGCTGAATCAAGACCTTGCTCAGTTCCAGCACTAGA
CCTTTTATCCTCACTGATCTGTTGTGTGTCTGGAAAAGCATTGAGAGAAGGAAGCTCAGAGATTGAAGGAGCT
GCTGCTTTTAACAACCATTCAACAGCTTTGCTTGGTTGATCATAACCAAGTCGATCTTGCAAATCATAGAACT
GAATAGCTGTGTTAACAGACAGCCTAACTCTCCGATCTCGGAGTCCTTTTGAAGTCCACACTTTACTGTGCCT
ATCTTTCCCTCCTGATGCACGAGAAACTCGAACAATCCGATTTGAAGGCCACCCACAAAGCCTACCAATACC
ACCACCGCCAAGGCCATGACTCCCTTTCACCGCTTCATTTTCATCTTGTGACTCAAATCTACCATTTCCATTGC
GGATTCTTGGAAACTTACACCCTTGTTTTTGAATCTCATCCACCTCCATACCACTTTCATACCTTTCTCAAACA
ACCACTCTAAACCAACCCCACCTTTCTTGAAAGAGAGAGAATAACACCAATCAGCAAAGAGGGTATTTTTTT
TTTCCTCTCTCTATGTGGATTTGGGATTGTAATTAGGGTTTAGGGATGGAGGAGAAAAAGAAAGGTAGATAAG
AAAAAAAAGTGTAGGTAGGTGTATGGGTTGATGTGGGGGGTGTATGTAGATGTGGGTTTTGGATGTACTTTGC
ATTGATGATGATCACACAGAGTACTTTGGCTTTTTCGGTGATATTTGCATACGCACACACAAAAATAAAGAGA
AAAAAAGTTGGGTTCGATGTATCGCGTTTGGTACCAGAGAGGGAGGGGGGGGCTGGTTGAAAATGTCAGAA
GAGAGGAGAGGCTAAAAGAAGGCTCCTTTTTGCAGTGCTTTCAAAAGCAAAGCCTGTCTACTTGCTCAGAAC
TCTTAGCTCCTCAGCCCTGTAAGAAAATTTACAGTCAAACTCATAACCACAAAAATCAGAAGCCCCTACCCC
CC
>TCONS_00071454
GCAGAATGAGGATTTAAGAAGCACAATAATCTTATGGCACTGAAGCAGAGAAAAGCATGCTTCACTCAGTTG
CAGCTGCTTCCATCTTCAATTTCCATTTGTGAAGATGGGATGGAAACTCAGTTTTTTCCTTTTCCTTTCTGGTCT
GAATGCCTACTGTCATTATAGAAGAGGTGTAAACGGCCGGCATCATATGCAGACAGAAAGTGGTGATGGTTG
TCAACTGATGCTGCAGCAGTGTTGTTGGGAGCTGTAGCAGCAGCTATTGTGCTGCCATTGAAGAAACTTTGTG
ATCCCTCATACCTCTGGAAATGAGGCAACAACGAAGAAGAATTGGACTGAAGGGTCCCCCTATTATAACCAG
CAAGGCCGGAAGAAGACGATGATGATATGCTAAAGTTCAAATTATACTCATTAGCACTATTACTGGCATGGC
CATTAGTTCCCAAATGGTCACTTAAAAATGGAAAATGCTGCAGCTCGGGATGATGGTCTCCCGTAATACTAA
ACATTGGTGATGACACAGGGAGTGGCTGTTGTTGTATAGGATTTCCCAAATGAAAACCAGAAGTAGAGTTGT
CGTTTCCACGAAGACCTAAAAGTCCTCCCGTCAATAGTTCGGTGAAAGAAGAATTCTGTGAAATGCGATGCA
CATTTTGGTGATGTGCAGTTCGAGTCGACTCATTTTCTTTCTCTTTCTCCTTCTCCTTCTCCGCAGCCCTTTCCCT
TGCCCGTTCCCTCGCCTTGACGCGATTTTCTGACCGGGATAGAGACAACCCAGAACCTTTGCTTGTCTCAGAA
GTGCTACTACAAGCAGATTTCGACAAAGACACATGTTGTTGCTGTTGGTAATTCGGATCACCTTCCATTTCAA
CCTCAGCTGAATCAAGACCTTGCTCAGTTCCAGCACTAGACCTTTTATCCTCACTGATCTGTTGTGTGTCTGGA
AAAGCATTGAGAGAAGGAAGCTCAGAGATTGAAGGAGCTGCTGCTTTTAACAACCATTCAACAGCTTTGCTT
GGTTGATCATAACCAAGTCGATCTTGCAAATCATAGAACTGAATAGCTGTGTTAACAGACAGCCTAACTCTCC
GATCTCGGAGTCCTTTTGAAGTCCACACTTTACTGTGCCTATCTTTCCCTCCTGATGCACGAGAAACTCGAACA
ATCCGATTTGAAGGCCACCCACAAAGCCTACCAATACCACCACCGCCAAGGCCATGACTCCCTTTCACCGCT
TCATTTTCATCTTGTGACTCAAATCTACCATTTCCATTGCGGATTCTTGGAAACTTACACCCTTGTTTTTGAATC
TCATCCACCTCCATACCACTTTCATACCTTTCTCAAACAACCACTCTAAACCAACCCCACCTTTCTTGAAAGA
GAGAGAATAACACCAATCAGCAAAGAGGGTATTTTTTTTTTCCTCTCTCTATGTGGATTTGGGATTGTAATTAG
GGTTTAGGGATGGAGGAGAAAAAGAAAGGTAGATAAGAAAAAAAAGTGTAGGTAGGTGTATGGGTTGATGT
GGGGGGTGTATGTAGATGTGGGTTTTGGATGTACTTTGCATTGATGATGATCACACAGAGTACTTTGGCTTTTT

CGGTGATATTTGCATACGCACACACAAAAATAAAGAGAAAAAAAGTTGGGTTCGATGTATCGCGTTTGGTAC
CAGAGAGGGAGGGGGGGGCTGGTTGAAAATGTCAGAAGAGAGGAGAGGCTAAAAGAAGGCTCCTTTTTGCA
GTGCTTTCAAAAGCAAAGCCTGTCTACTTGCTCAGAACTCTTAGCTCCTCAGCCCTGTAAGAAAATTTACAGT
CAAACTCATAACCACAAAAATCAGAAGCCCCTACCCCCC
>TCONS_00071557
GAAATCTTTAGTTCGATATACTCCATATAGTTTGTACCTATCAAAAAAATATACTCTATGTTTGCAGTCTTTGT
GCTTGCTGAAATTTGTTTTCCACTCTGTGAGCTTGAAGCTAGTTTGACCTGAATTTATATGTCTAGAATAAAAT
AAACGGAAACTTAGTTTATAAATATTTCATAGTAGATGGTGGAGCAGAGATGCCTGATGAGGATGG
>TCONS_00071719
AACCATGCCCAAATTTGGGAATCACTCTAGGTATCTGCTGCAGGTTGAGTCAATCCAGACTGTACAAGCAAG
TTGTTCAGGGATTCCGGCGAGTGCACCTGGTATTTGACTTTACCATTGAAAAGGGACACCTCTGCTAGCTCAA
GTTTCTCCGAAGTAAGACTTGTACTATCCATTGTCTTACTGAGCACCTTCAGAACAAGTTGAACCGCTTCCTCT
CTTGTGATCTCATCCTTGTAGTCCTGCTTCAACATCGACTGTGCTGCTTGATTGTTAGCACCGATGGCTGCTGC
CTTCCAACCGCCATAGTTACCACTTGGATCACTCATGTACAGTTGGAAGCCATAGTTCTTATCCCAGCCTGCA
AAGAGAAATGAGACACCAAATGGACGAAGCCCACCAAACTGTGTGTAGCCTTGCTTGGTATCGCACAGAGA
CTGAACTAGCTGTTCGACAGGCATTGGTTCTTGATAAGCAAAAGTATACCGTTGAGCTTGTACTCTAGCTGTG
TTGATGAGGATGTTGGCATCTGACATTATTCCAGCCACAGCACATGCAACATGGTCATCAATCTTATACATCT
TTTCAGTGGATGTTGAAGTCTGAAGTAGCTTTGAGGTAACCTTCTTTTCACCTACCAAAACTACGCCATCTTTA
GCTAAGATACCTATTGCACTCCCTGCATTGCCAATGGCTTCCATTGCATACTCAACCTGATATAAACGACCTT
CTGGGGAGAAGATAGTTGTGCGGCTATCATATCTTCTAGACATTGTATATTAGCTGGATTTCGCTGGAGAAAG
AGATGGAAAGAGTATCAAGTGTCGACTTGCG
>TCONS_00071788
CAAACAGGACTTTCAGCACCCTCTTCAACACTCAATTTGCCACCATTGATAGTCATATCAGTTTTGACATAAC
CTGGACAAACACAGTTGATGAGAAAACTTGGGTATTTTTTGGCTAATATTCTTGTGTAAGCATTCATTGCTGCT
TTTGATAGTGTATATGCAGGAAAAATCAGAGACCATTTTTCAGTTTCCATCAAACCGTCCTTTAAATCTTGCAA
AAAAGCATTCAAAACCTCATCCACTCTGTCTTCTGTTAGATTCTCAGCATCACTTAACACTCCTACAGCCCATT
CATTGCGTACATGCAAGATATCAAGCTTGCCAAATCTGGCCTTGATGAAATCTTTGAGTTGTGCAATAGTAGA
AGGGTCAGTCACATCAAGCTTATGAAAAATAACATAATTAGAGAGGCCACACTCCTTTAATATATCAATAGC
TTCTGCTCCTTTCTTCTCATCTCTAGCTGTTAAGATCACCACTATCCCTTTTGAGGCTAGCTGTCTACATATTTCT
AGTCCTATTCCTTTGTTGGCTCCAGTTACAACTGCAATCCTGATAACAAATTTGAAACCATACTAAATATAATT
AATGCCCATCTTACTCGCACAAAGAGTG
>TCONS_00071868
TACATTTAAAGGTACATTTTATTACACTTTTTTAAGTAATGGTTGACTTATAAGGCATTTTAGGTAGGCTTCATT
TCTTGACGTGAGGGAAACTCCAAGTTATGAAGGAAGTTGCATTGCGTGGTCCCCTACTTTTGACTTTCTTCTGC
AGTTTATCTTCATTACTTCCCTGTAATCTTCATTGTGCAGCAAGTGGTTGCTTACTGAAAATTCGGTTTAGAACT
TCGGCCAAGCGGATCCCACTTTGCGCTAACCTCTTCTCCACTACAGGAAGCCGAGAAAGGAAGTAATCGTCT
CCCAAAGTGCTTCCCGGAGTTGCATTTCTGTAGGCAAATTTGCAGGCCAAACGGACACTTTCAGAAGCATAC
GGGTCAGGACAGACCATCTTCTTGCAATTTTCCCAAGATGGAACATCATCGGACCATTCATCAGTGATGTTTC
TCAGAAGAGCTTGTATCATTAACGTGAGGTCAGATTTGTAGTATGTCTTCAATGCGGATTCAATAATCATGGT
ATCCCATACATGATGCAAATTAGTCTTCCTCCTGTACCAACGGACTACTATAGTGTTTCCACCAACATCTCCA
GTGAAACCAACATGTAGAGGCTGATGTACATCACCAACAAAATGAGACAAGAACATAAGCGCTTCAGTCAA
GTTGTATTTTTCTGAATTCAAATCATAGTATCCCTGTGAAAGTTGCATTGAGTAGTTGTATATTGCTCCTGTAAC

GCACCTGTCCTTGTGTCCATGCCCGTCATGGCAGTCTCGGCAATATTTGTAATTGCACAAGAAATCAGGTGTA
TCAACATAATGTAATGGAGAACTCCAGCGGTAGTACGGGTAGTGGTGCTTAACCTCATCAGCCCAGGAGCAG
ACAGCTGCAAGATCACCTTCAGCTTGATCTGGGAGTAACTCTTTGACTGCAGCCAAAGCATCTTCTGTTAGAT
ATTCCTCAGCAATTTTGCAGATAACATAGTGTCCTTCTTTTCCCCAACCAAGAATCTTTGGAACCATCATTAGT
ATAACAGTTACTCTTACAAACCATATTAGCTCAAA
>TCONS_00071876
GGGGATTTCTTGCAGGTAGAAGCTCTCAGCTGTCACTCAAGGGATCTGTTGCATTACATTGTTTTGTTAATTGT
AATTTAAACATATTTATTACATCAAGATGAACTTTGAAGTTTGCTTTCCTGCGAATTCAGATTATGATTGGTGG
GGATTGTAAGAGATGGTAATGGTATTGTCATGTGGAATTTAATATCTGGCCTTCTGTGTTTCCATACAAGCTGT
AAGTGCTGCCATTTAGTTCCATCAACTTTACTTTAGGTGTTAAAGATGCCTTTTCATTCATGCAGGATTTTTCAT
CTACTCTAGTTAGGTATGGCATTTGGACAAAGCCTGTCTTTAATTTCTTCCACGAACATAAAGGGTTTTGTCTT
TCTCTTCATTGATCAGGTAGTGCTTTTGTCTTGATGTCTTGCCTGGTCAAAGTGAATGGTGTAATTTGAGATAG
AGGATGCAAAAAGAAACTGAGAATGGCTTTGAAACTATAAGTCACCAATAACAGCTGTAGATAGCAATGAA
TGAAACAGACATAGCAGAGGACCAAGAGGTTGGAAAGGAAGGAAGGCATCTAGA
>TCONS_00071905
GATAATCCGTCTATGCCCCATCTGGCTGGACAAAAGGATTGGATATCTCATCTGTACCATCAGTTGGAGCGAC
AAGCATTACTGCCGTACCCCTGCAGACTATTAAGCCAAGAGCTCGTGTCTGCTCGGTAGTCTTCAGGGGATCA
TCAGCATCTCTAAGGTACTCCTTGGCTTCATCCAAGACAAGATTTAGCAGCTGATCATAGCCTTTAAGTGTCC
CTTCCACTTGTCTTCCACCTGTAAGCTTGACCTGCACACCCTTGTCGACAAATTTTGCCAAATCTAAAACAGTT
TCTTTTCTGCCAGACTGAAACAACACAAACACGAAATTGTATAAAGCAACTATCGCAATTAAAAAG
>TCONS_00071924
CTAACTCCTGCATCCAGCTTTTTACGCCGCTCTTCATATGATGGCTCAGTTGGTCGAGACAGTAGAGGAGTGT
AGGAAGCTTCTTTCTTCTTAGTACGCTCACTTGATATATTGTCATCAATTTCAACAGAAGTGCTTATGATAGCT
TCATGAAAAATTGCGGCTTGAAGTTTATAACTTCGGCATTCCCGCTCCAAATGTTCATTCAAAGCTTGCTTTGC
ATCTGATGAACAAATAAATTTGAGACCTTCGAATTCACATGATTGAAGAACTAGAGGTATCTCTGGAGCCATT
AGAAACTGAGGTTTGCGTGGTGTTCTTTCAATATCTAGAAGTGCATCTATTACATTAGGGGTTTCAAGACCCT
GGCCAATCAGAAATAGTACAGCAACCATGCACCGGATTTGATGCCAAAGGAAAGCACTACCTTTGATTTTCA
TAATCATGAGTTGATCAGCTTTGTAACTTTCATTAAAGGGGAGAATTTCAAATGATACTATGTGCCTCCGGTA
GTTGTGCACATTGCCTGCATCCATTTTGCAAAAATTTCTGAAATCATGTTCCCCTAAAAATTTCTCAGCGGCAG
TTTCCATTGCTGTTATATTCAAATTCCCATGCCAAAAGAAGTACTTGTACACCCTGCTCAAACAACTGAACCTT
GCACTGAAACCAACTGGAATAGGAGACCAGCCCATAATGCGAATGTCATTTGGCAGAACTTGATTTAGTACC
CTCACATAGTCCATTTCTCCTGGGTGAAGAAAATTTAAT
>TCONS_00071928
CCTTTTGATGATGCCTCCAGCCTAAGAACAGAAGACCAGACGAACAAGATAGAGATTGAACATTCTATTATT
TTCTGTTGCATCAAATTTTTCATGTTTTCTTGACAGCTTTTCAATTATTGAACCAAAGTTCAAGAAAGTCAGGTT
TCCGCATTTCAGGAGGTAAAATGTAATCAATCATCTCATACTTATCAATCCCTTGTCTGCTTTTGCTTGTAGTA
ATCTCGCGTAATTTGCCATGATTCCCAC
>TCONS_00071965
TAACCTGGCTAAGGCAATGAAGGAGCCAACGTCCTTTGTTCAGAAGAAGAAGACGGGTTTTCTTGGCGAAGC
TCCTTAATTACAGCAGCCAATGCCTCAGGGTTAAAACCCAAATCGCAGAGAGCAATTAGAACAGAAAGAGT
GTGACGATCGAGGCCTGTGTCCAGAATATTTGACATATGAAATACCAACTCTAGAGAATCACGTGCTGTACG

TGCAGCCTCCGGATCCATTTCGACAGTTTGGGAATTTGCTTCCTTTCAGTGAATCGAGGATAAGAAGGTTGCT
GCTTCAAAATAGCTGCACTGC
>TCONS_00072056
TCTCTGTCCTTTTCCTCATAATAACAAAACCTCTCAGATTCACAAAATCAGAGGAGGATTAGGACGAGGACG
AAGAGCAAAACTCGCCAATTCTATGTCGAAATTCGCTGAAACGCGTGATTCGTAAAATCAACAATCGGCGAT
CCATCTTTCATCGACAAGGCGTTTAAGAAAAACTCAAATCCACTATTTTGGTTGAATTTCCGTATATATTTTAA
TAATTTGTAGAGAAATTAAAGTGTAAGGATCTCAA
>TCONS_00072115
TCAACTACTCATGATCTTTAACCAAAAATATAAAAGGGAAAAAGAGTAGTTGCAATTTAAAGATTGACATCA
ATTTAGTATCTTTGAACTCTTTGCAATAATTCTCATATATCTTCCTTTCTCACTTTCCAACTTCACAACACTTTGA
GCTTAAGTGATTCTGCCTTTTGGTCACATCAGAGTTGGTGGATTCGAGATATTGCCTATTCAGATTGATTCTTG
AGGACTTTTCCTTCACCTCAAAGTTTAATTATTCACAAGGCAAGTATATTTCACTTTATATCCATACATAAAAT
ATTAAAAGTAAAATGAATAATTGTGCATTAATTTGATTCTTTTC
>TCONS_00072127
TGCCTACAAGCAATTATCAAAATCTGGTTGTTTAACAATCAAAAATGTTCCAATCGCATCTTCAAAAACAAGC
TAAACGCTCTCTAAAGAGAAATCCATATGTTTACTACAAAGACTAATTCAATGCTTGGTCACACTCAGTGAGA
CTTCACATATTCCTGAACAATGTGGAGTCCTTCTGTTTCCTCTCCAAAATCCTTTACGACAACACAACCGCAAC
CAACAACTTTCCTTGCTTTGCCTTCTGAATCAATCTTGCACAGACCAGCCCATTCGCCGAGGGTCTTGGCACTA
GGAACTGTGATCAGGTTAACATTGTGATCAGCACATAGCGCTTTGACCAATTTCACATAGTCTGGTTGGTCGC
AGTCCTCTGCCAATACACAGAGTTGGGCAGCATGCTTCTCGATAACCTTTGCACCCTCATGAAGACCTTTAGC
TAACCCGCCATGAGCCCGTGATTTTCTGAGGACAAGTTGCAATGCCGTCATGATATCCATGGGCTCACCAAG
AGCGGGAGCGGGAGCTGGGGTCTCAGCAACAACAGCCTCTTCACTGCATTACATTAAATCAAGGAATAAAC
AAAATGACATGGAAATGAGAAGATAACCATTTAAATCGAGAGCTTCTAAATAATAAGAGAAATGTCATCAA
GTAAAGATGGTTAGAAAAGGCGAATACAAGACAATCAAAATGATAAAACATGAAAAGATGGGAGGGGGCT
TTATTTCCTAAATCAACTCTACAATCCATGACATACAAAGCAACAAAAACCAAAAAGAATGCGGTATCTTTA
CTAAAAAGAGAACTGATATGGCTTGTTCATGTTAACCAAATGCAAGTTACCCAATTT
>TCONS_00072128
TGCCTACAAGCAATTATCAAAATCTGGTTGTTTAACAATCAAAAATGTTCCAATCGCATCTTCAAAAACAAGC
TAAACGCTCTCTAAAGAGAAATCCATATGTTTACTACAAAGACTAATTCAATGCTTGGTCACACTCAGTGAGA
CTTCACATATTCCTGAACAATGTGGAGTCCTTCTGTTTCCTCTCCAAAATCCTTTACGACAACACAACCGCAAC
CAACAACTTTCCTTGCTTTGCCTTCTGAATCAATCTTGCACAGACCAGCCCATTCGCCGAGGGTCTTGGCACTA
GGAACTGTGATCAGGTTAACATTGTGATCAGCACATAGCGCTTTGACCAATTTCACATAGTCTGGTTGGTCGC
AGTCCTCTGCCAATACACAGAGTTGGGCAGCATGCTTCTCGATAACCTTTGCACCCTCATGAAGACCTTTAGC
TAACCCGCCATGAGCCCGTGATTTTCTGAGGACAAGTTGCAATGCCGTCATGATATCCATGGGCTCACCAAG
AGCGGGAGCGGGAGCTGGGGTCTCAGCAACAACAGCCTCTTCACCAGACATTTTCTCTTCTTGGATCTAGTTC
AAG
>TCONS_00072150
CTGGACTCTTGTGTAGCTCTTAGGGCATCAGCATCTGCAGTGACTCCACCAATTCCTGCATTGTTCACCAAGAT
ATCAAGCCTGCCAAATTGGATCTTAATGAATTCAGCCAAAGAAGCAATAGTGGAAGGATCCATAACATCAA
GCTGCTGAAACACCACATTTTCTTTAGCCAAACCAGTTAACCCTCTGAGCTTCTCCACAGCTTCAAGCCCTCTC
GTCTCATTTCTAGC
>TCONS_00072213

GTTAATATTCTTTTGTTGGGTAGTGTTGGCAAATGAGGGGCCATATGCTATGGTTGTGGCTACAAATTCTCCAG
TTTTGCATGTCTACTCTTTCCTTCACCAATCACTACATCCAATTTCAGTAAGGACCCCTCTTTCCCTTTAGCCAA
ATCCACTTCCTCTAACCCCACTCCCTTTCCCCTCCTACTCTCTCTGTACTCTGTAGCAAAGGTAAGTCTTTTTTC
CTTTACTGTTATTGACTTAATTCCCTCATATCTATCTTTCTTGTTCCAAGATTAAGTTTTTCTAGTTGCTTTGCTTA
TTTTTTCATGTGGGGTGTTCATCAAATTCCAAGATTGCAGCTTTATTATTATCACCTCATTTTTTTCATTAGTGGT
GCCTCTTTGTTGCTGAAACTGAGGTTTTTCTCTGCTTACTTAGCTTTATTTTGATTGGTTTCATGTGGGTG
>TCONS_00072324
CTCTTTCTGCTTTTAAGTGATTGCAATCTTTTGACTATCGTAGATTTATCATAAATAACTAATGGTTGCACTCTC
TTTGACCACAAACAGGAAAATTTGGAGCTATTCTTGCTTCAATACCTCTACCGATCATTGGAGCTTTATATTGC
GTCTTGTTTGCCCTCATGTCAATGTTGATGTTGCTGGATAACACTTGATGAATTTAAATTGCTTTTGGGATTGAA
GCTACAAGTCAAGATGAAAGTTTAGAAGAAACACAACTTCATTATTATTTTGCTAGGAATGTTATGTAAAAC
ATGATATCATAGCCTAGCTCAGGATGTTAGCTATGTTTTTTTT
>TCONS_00072335
GTGCATATATACCGAAATCCTCTAACAAGAATACAACATGACCCTCAAGATACAATCGCAAATGTTCGATAG
TCATTATAATGTACATTACAAGATGGCCAAAAAGCTCATAAGCTTCCGAAGAATGCTGCATATTGTCGCAGA
ATCCAACTGCCACATTCCCGCACCACGCTGAGGAATTTGTACAGAGCAAGAGGCAGAAACTGTCGGTCCAAC
TATACATGTTTAATACATTGGATACCAAAACATTCCCTTAATACCCTCTGGATCAGGTTCAAAACCTAGATTCT
TATAGAATTCCACAACTTGACTATCCGCGAACAATGAAATATTTCCAATGTCTCTTTGGAGAAGGGTCCTTAT
CAGTTTCTCAATAAGAGCTTTTCCAAGTCCTTGTCCCTGATAGGAAGGATCAACAAGAACATCCCAAATTGTT
GCATTGAAAGCATGATCTGATGTTGCACGAGCCATGCCTATCAGCTTCTTTTCTCCATTCGCCTCTTCTCCTGA
AGAGAACTTCCTGGAATGCAAAGTTGCAACTATATAGCTATTTTTAAGAGCAGCAGCTAGCTTAGACAATGG
CCTTCGAGGCCATCCAACCTTATCACATAAAGCTTGGAGATCATACACATCAACATCTCCGCCAGAAGAGAA
TATAATCTGTTCAACTGTTCCATCTGGTTGAGTCTTTTCAATTAGAACAAATTCCTCAGGCAAAGGCTCTTCTT
CCTCTTCTTCTTCATTGGATGGTGATTCTATAACCTTTATGGTGTTATTCTTAACAAACCCGGATCTGATGGATT
CCCAAAAGCCAGCCTTCAAGTTAGAAACCTTTGATTGCCTCTTAACTTTCACAAAACCCAAATTAGTCTTGCA
GGTAAATGGCAATGGATTTGAAAATTGACAACGGCAGCAATTAA
>TCONS_00072336
CCTCTAACAAGAATACAACATGACCCTCAAGATACAATCGCAAATGTTCGATAGTCATTATAATGTACATTA
CAAGATGGCCAAAAAGCTCATAAGCTTCCGAAGAATGCTGCATATTGTCGCAGAATCCAACTGCCACATTCC
CGCACCACGCTGAGGAATTTGTACAGAGCAAGAGGCAGAAACTGTCGGTCCAACTATACATGTTTAATACAT
TGGATACCAAAACATTCCCTTAATACCCTCTGGATCAGGTTCAAAACCTAGATTCTTATAGAATTCCACAACT
TGACTATCCGCGAACAATGAAATATTTCCAATGTCTCTTTGGAGAAGGGTCCTTATCAGTTTCTCAATAAGAG
CTTTTCCAAGTCCTTGTCCCTGATAGGAAGGATCAACAAGAACATCCCAAATTGTTGCATTGAAAGCATGATC
TGATGTTGCACGAGCCATGCCTATCAGCTTCTTTTCTCCATTCGCCTCTGCCAAGTAACTTCTCCTGAAGAGAA
CTTCCTGGAATGCAAAGTTGCAACTATATAGCTATTTTTAAGAGCAGCAGCTAGCTTAGACAATGGCCTTCGA
GGCCATCCAACCTTATCACATAAAGCTTGGAGATCATACACATCAACATCTCCGCCAGAAGAGAATATAATC
TGTTCAACTGTTCCATCTGGTTGAGTCTTTTCAATTAGAACAAATTCCTCAGGCAAAGGCTCTTCTTCCTCTTCT
TCTTCATTGGATGGTGATTCTATAACCTTTATGGTGTTATTCTTAACAAACCCGGATCTGATGGATTCCCAAAA
GCCAGCCTTCAAGTTAGAAACCTTTGATTGCCTCTTAACTTTCACAAAACCCAAATTAGTCTTGCAGGTAAAT
GGCAATGGATTTGAAAATTGACAACGGCAGCAATTAA
>TCONS_00072350

CTTTGGATTTGAGACAATAACTCCAAAAGCACTAAAAGTGTCATATAGAAGCTTCTCATCAACATCAGGATC
AAGGTTCCCAACAAACAAGTTGGCACCAACATCAACACTCTTTTTATCTTGTGATGCCTTATTCACTCTTATTG
GTTTCCCATAAAGTTTAATCATATTCAGTACCTTTATTGCATAGTCAGCATCTTCTTCACTTCGGAATTCCACA
AAGCCATATCCCTGATGAGCATTAGTAACTCTGTCCTTAGGGACATAAACATTGACTACTGGACCTGCTTGAA
CAAACAACTCCCATAACAACTCTTCAGAAACCTGAGGGTCGAGGTTGCCGACATAGGCGGTGGCGTCTTGGT
TGCGCTCCGCTGCGTGTTGGCCCAGCAGGTTTGCTCCCACTCCCGGAGCTATACGAGTCGTCATACTCAGAAA
GAAGAATGCTC
>TCONS_00072371
TTCCTTCTTCTCTTTCTTTCCTCCTGTGAAGAGCTTATAACCTCCATAGAAAGCCAAACCCCAACCAGTAAGAG
AGACGATCACGAAATGCTCTTCTTTCCATTTTGATGGGCTCATTGGGTCCTGCCAAAAGTTCACCTTTGGAGG
ACCATGATGGTCACCGCCGCCAGCCAGACCGCGCCGCTGGATAAGGAGGGCGCCTTGGCTCG
>TCONS_00072434
TTGAAATGTCTACACCATTCCATTCACCGCAGGTACCTCGCAGGTCCTCACCCCCAAGGGCTAGGAGAAAAG
CTCTTTCATTTGACTACCTTAACAGAGGGACAAATGGCATTGAACGTGAATTTGTGAGAGCACTTCCTGATGC
TATTTTCATTCAGTTAGGGTTGCTAGCTCGCTGCATGTTTTATGACTAGCACTTCCTCTCTGAGTTTATCACCTT
CTATCTTTACTAGTCTCTCCTTTCCATGGGATGCTGGACCATTACCTGTAGAAATCTTA
>TCONS_00072564
ATTTCTGGCTACTCAACAGTCTGCACATTCTTGAAGCAGATCATTCATTCGGATCTAACTTGTAGGAAACATC
ATCTTCTTCGTAGTTGTTGACTCGTTCACACTCGTCAATCCATTCACTATATATATCAATAGGCTCTGTTAGTGC
TGTGACAGTGGTGCTGAAGCTCTCTTGGCAGATCCTGCAATTTGCCTCGCCAATCAAGTTTTTCATATCAATGC
GACACTCCACGCTGGTGCCATGACTACAGAAAGGACAGCTGAAAACAGTGTCAAGTTTGTCCATCCTCTTCTT
TGGAGGTGGCTTTGATTTTGACTTCCTCTTTCCCATAGCTCAAGTTACTGAAATCGATTGAACAGCTGAGATAA
AAATCGCCTTTTTGTGATCGCTGTCGACTCGAAACCCTTGAC
>TCONS_00072566
GACAGGTACAAAAGTGTAAAAGCTCTTGGTGTGCTCGGAGCTAAAATGGACAAAAGTGTTTGTTCTTTCCTGC
AAAGAAGAAGCCGGGGATGGCTCATTGAGCTCAGCTTTCCATTCGCGCAGCATCTGGTGGACTTGCTCTTCAA
GCAACCCAACATCATAACTTCGGCTTTCTTTCCTCGCAGATTGTAGATTAGTTAAAACACCCTGCAGATCATC
GACACGGTTCTTTGCCTTGTCCTTGAAGAGCTGGTGCGATGCAGACTTGCACGCACCCCTCTTCGAAACCTTC
CTCATCCAATCAAACAGAACAATACCCTCAACTCCTCACCTTCAAAATAAGCAAGAAAACTCGATCCT
>TCONS_00072594
TAGAGCAAATGATTTCTATCACCAACTTATGGAAGCTCTGGAAAGCAAAATCTTCAAATGAACTAATTTCTAC
AAAATCTTAACATAACAAGGAGAGAAACTGTTTAGAAGATGCTAATATACACAGAAGGCAAAAGAATGGCA
ACCTGGAATGACAAAAGCATAACCAAAGCTTGCTATTATTTTGCATCATAGCATAGGGCCAGAGTAACGGTG
CTCGATGAAGCCCTTGAATCTTCAAATTCATCGCGCATCCAGTCCTGGGCACACATCAAAGCTTGCAAGGTAT
CTGATCTTAGCGAACTGCGATACTGTTCAAGAGCTTTATTCCCAGTGTTGAATACAAACTCTAATGAAGCTTTT
GACATAGGCATTCCAAGAATATTCCGTGCCATCATGGACAGAATAGGATACCTTGGAGTGTGAACCTTCCAC
CAGTTTAATATGCTAAAATCGGCTTTTCTCGGGAAAAGCGGCTCTTCCAAGTATTTATCTAAATCTGATTTTAT
GTTGTTGCTCTGTGAAGTCTCATATATGAATTTGTCGTACCCTGTCAGCCGATCATTATTAGCACCAGCACCAG
CACCACCAACTTGAGATGCTTCAGCTTGACCGTTAGGAGCTAATGGTGAGTAAATAGCATGGCCATTATACA
AAGCCTTCATACAATCTGAGACAATATTAATACAGTCTGGAGCACTATCACCATATATTTGAGGATAATAGTA
TTCAACCAATTTCATCTTGTATCTTGGGTCTAAGATAGCTGCAATTGCCAAAGCCAAGCTACATTTTTTCCAGT
ACTCATCAAATCGGCTTTTCAACTTTAGAGCCAAAGAATTAACAAAATCATCCGAGTTTTGACACCACTCAAT

CAACTGCAAATGAATATCACAGATCTCTGGAAAGTATGTATTTGCAGTAGGATACTTGCTTCCTGCAAAAACA
CTGGAAACTTCAACAAAGAGCTTTAGGAAGCTAGCAATGGCACTTATTCTATCCCACTCTGTGGCAGATGGA
CACATTCCATACCCAGAATCATGTTCTTGCAAAAGGGGAAATGCATCTTTGTACTCCAAAGCAGTTTCAATCA
TGATATATGTTGTGTTCCAATAAAATGAATTATCAAGATTCAAGCACTTCTGGCAATCAACCCCAACAATTTG
AGCCATCTCAGTGAACTTTTCCTGGTTTGCCTGTGAACTTCGAACATACCGAATACTTTCCCGGACCTTGTGGA
TTATCGGGCTTGCTGTTTCTAAGGTATCTTGGACCATCAACTTGACAACATTAGCTGCACAGCGTGTATCAAAT
AATTGACCGTCACAGTAAAGAAACCTGTGCTGGCAAAGCTGCTCTCTGATTCTACTTACAATTTTGTCATATGT
CGAGTAGTTATCAAATGTCACAGAAAACAATTTCCTGTCAATATCCCAATTTCTTAGACTAGTCATGATAACT
TCTGAAAGCATGTCTTCTGTTTGGGAAGGATCAGTTGTTAAAAAATTCAGAATCTTCTTTTTCAGTTGCCAAGA
GTCATCTATGTAATGTGCTGTCAAACACAAGTATTCAGCATCGCCATTAGCAGTCCATGTATCAGCACTAAGG
CTGATTTTCCCAGGCAATTTGTCCAATTCTTCATATACTTTCTGTCTCTCCATCAGATAAATTTCCCTGCAGTCA
GCCTCAACTCCATCAAATGTTGCAATGTCAAATAAAGGTTGAAGATTTCTGACAAATATACGGAAACCAATA
TGTTCAACCATAGACAAAGGATAGCCATGTAATATGATCATGCGAGCAAGATCAAGCCGGCTTCGCCTATTA
TCAAAGTTAATCCCATTGTTGAGTAACCCATCTCTTGTGTGCCCTTGTTCAAACTTAGTCCTCACAATGCTGAC
TGCTTCAGCCTTCCTCTGCTCTTGATCAAAACTAAAATTCGAGATGGCAAGGGTTCCCTCCTTTTTCTTTCCCCG
TGTAAGCAACTGACTTATGTCATGGTTTGATCTCCTACGACATCTGATCAAATGATTTCTTAAATGTGAAGTCC
CACTGGTGCTAGACCCACTAAGTTTCCTTTTACAGTGCCTACAGATAGCCACAAAAGTATCACCTTTTTTTACT
CTATCAAAATCATTCCACACAACAGATTTTAGTCTACTAGAATTGACTATAACTGCTTCCTCAGCTATCTCCAT
TGCTTCCGACTGTAGCAATACCTGAGA
>TCONS_00072636
CTGGAAACAAAGTGTTGCCTTTACTTCCTTCTCCAAACATATTCCTCAAAAACTGGAAATCTCAGAGAAGTAG
CGGCAGATCTGAAGGAGCGATTATTAGTAAGAAGAAAGAGTGGAGAAGAGGAAGAAAGGGAGAGAAAAG
ATGGGAGAGAAGTGGGGGAGAGGTATAGGAAGAAGCATAATAATAATAACAGTAGATGGAGAGAAGTGGT
GGTGGTGTAATAGTTGTGGGGAAAGGTTTCGGTTAAGAAAAACGATTCCATTCGTTGGCTTTGGCGTCGGAAT
TGGTGTTGGTGATGCAAATCACAAGACTCGTTGAAGAAATCTTGCCCTTTTCCTTGCCTTGTTAGTACTCTCTCT
CTCACTTTCTCTTTCATAATACTCTGTCTTTGGCAGCTTTCTTTTCGTTTTCC
>TCONS_00072728
ATCTTAAGGCCACCATCGGGGTTGACTGAGAGGATGTTGGCATCAAGGACTGCAAGTATTTTGCTGATTCTGT
CAGCAATTTCTACTGTTGATTCAATGTTTGGCGAATGGATGCGTATAATCCAACACCGTACTTCACTCCCCCGC
GGAACACAGAAAGAAGCTTCTCATCAGACCTGGATTCCTCGATGGTGATCACGCTAGCATCCATGTCCTTAAT
GGAATCGCTCACGTCATTGAAGTTTGAATAGCACATGTGAGTGTGAATCTCTTTCTCTAATAATTCCAGCAATT
GATTTATTTTCCCCAGTTTCCAAAACCAC
>TCONS_00072737
CCTTGAGAATGTATATCCATGGCTCCTCAGATAGCCATGAGATCACCATACTGGCGCATCAAAATGATGGAA
CCAGCAAAGAGTGCTGAAGCTCGAAGCAGTTTCATGTTGAGATTCCACTTTTCTTCATCATGAACCTGAGAAT
TCCAGAAAGCTTTGACTTTGTCCAACGATATCACTGAATCCGCCATTGAATTGAAGATG
>TCONS_00072831
CAAAATAGTGTGCATGATAAATAGACTCAATTGTATAATTTGAAGATCATACACTACAATAGAGCTCAAAAG
ATGGCAAAAGTTCAATCAAGAATTAGTATCAGCTGGTTGATCAAAAGGCTTGTTAGAGCTTCCTTGTTCCATT
TTAATCTGTCCTCCATCTATCATTACAGAACCTGAATTTCCACCCATCAAGCTTGTTGATTCTGCAAATGTCGA
CTCTACTTCTCTTAGGCGCTCCTCTGTTTTCATGTCTTCCT
>TCONS_00072913

TTTTGTGCTGCTTCTTTGCCCAATTTTGGTATTTCAACCACCACTATATCTTGCCTAGCATCAAGAGAAGGAGC
CATGTATTCAGATATATTGGGATTCTCTTTTAGAATTTCCTCTGTTAAGTGCATGTACCTTTTCTTAATCATTGA
TTTGTCACACATGCGCTTAAATTTCTCCTTAAGCTTAGTCATATGCTCGCTATTAGTGACTCGAAAATAATAAT
CAGGATAAGTGCTTTGATCAACACAGTTCGAAGGAGTGGCTGTGCCGATGGCCATGATGGTGGCCGGTCCCT
TAGCACGCTGTGCCCTTCGAACCTCCTCGACGGTGACCATTTTCGCTGGAAAAATGGTGTTTTTACTAAGAT
>TCONS_00072914
TTTTGTGCTGCTTCTTTGCCCAATTTTGGTATTTCAACCACCACTATATCTTGCCTAGCATCAAGAGAAGGAGC
CATGTATTCAGATATATTGGGATTCTCTTTTAGAATTTCCTCTGTTAAGTGCATGTACCTTTTCTTAATCATTGA
TTTGTCACACATGCGCTTAAATTTCTCCTTAAGCTTAGTCATATGCTCGCTATTAGTGACTCGAAAATAATAAT
CAGGATAAGTGCTTTGATCAACACAGTTCGAAGGAGTGGCTGTGCCGATGGCCATGATGGTGGCCGGTCCCT
TAGCACGCACTATATCTTGCCTAGCATCA
>TCONS_00072965
TATGATGCACACAAAACCCAGGTCCTGCCCTTTTTCTATCATCATCATGAGGACCAAGTGGTGCTTCTCTTTCT
CCACTGTCCAATTTTGATCCAGAATCATATACTAATCCGCGAGGTGGCTTGTCAAGTGGCCGTGATGCTTGAT
CATGAGGCACTGGTAAAGGACTTAAATGCTCTCCCATAGGCGCCTTCAACCTTTTATTCCTAGCTGATTCCAT
ACTTCTAGATTGTCCAAATGGAACCTTTTCATAAGACCCTGGTGAAAACGGATTTGGTTGATTTCCATCAAAA
CGATTACTTTGATTTTGAAACATTTCAGATTCTGCTGGATTAACCAAGTGTTGTGTTCCACTGGGAATTTCAGG
ACCACTTAATGGTGCTCTTCC
>TCONS_00072966
CCTCCCCATCAGGATTTCGGGCAGACAAATAACCCATATGATGCACACAAAACCCAGGTCCTGCCCTTTTTCT
ATCATCATCATGAGGACCAAGTGGTGCTTCTCTTTCTCCACTGTCCTTGAAATGCATCGAATCAGGAGGATGA
TATGGTGGCAATTTTGATCCAGAATCATATACTAATCCGCGAGGTGGCTTGTCAAGTGGCCGTGATGCTTGAT
CATGAGGCACTGGTAAAGGACTTAAATGCTCTCCCATAGGCGCCTTCAACCTTTTATTCCTAGCTGATTCCAT
ACTTCTAGATTGTCCAAATGGAACCTTTTCATAAGACCCTGGTGAAAACGGATTTGGTTGATTTCCATCAAAA
CGATTACTTTGATTTTGAAACATTTCAGATTCTGCTGGATTAACCAAGTGTTGTGTTCCACTGGGAATTTCAGG
ACCACTTAATGGTGCTCTTCC
>TCONS_00073001
AGAACAATACTTCTAGTAGAATAATATAGCTGCATTTGCCATTGCCTCTACTATCATTATTCTGGTTTGCTTTCT
TCCTTCTCTTTTGGTTTGGCTTCTTCCACCTTCTTTTCCTTAGGTTTAGCTTCTTCCACCTTCTTATCTTTAGGTTTG
GCTTCTTCCACTTTAGCTTTTGGTTTGCCTTTCTGTGGCTTCATGAAGCAGAAACAAGAACAACATGGACATTG
GAACAAGAACTTCATTCTCTCTCTTTCTCTTTCTCTCT
>TCONS_00073002
GGGACTAATTTAATCTTAACTACTAGTGCTACTGCTTCAAGACAGAGAGAGAATGAAGTTCTTGTTCCAATGT
CCATGTTGTTCTTGTTTCTGCTTCATGAAGCCACAGAAAGGCAAACCAAAAGCTAAAGTGGAAGAAGCCAAA
CCTAAAGATAAGAAGGTGGAAGAAGCTAAACCTAAGGAAAAGAAGGTGGAAGAAGCCAAACCAAAAGAG
AAGGAAGAAAGCAAACCAGAATAATGATAGTAGAGGCAATGGCAAATGCAGCTATATTATTCTACTAGAAG
TATTGTTCTTCATTTC
>TCONS_00073050
TTGCTCCCTGGAAAGCATCATAATACACGCCTTTATGTACATGTACAAGAATCCTTGAGGCAAAAGTGGTTAC
AAATTTATTTGGTTTACATAAGATGCCAGTTTATTTTCAATGAAATTCCTTATACTAGTTGCATCACCCTGCCG
GACAATTCTTCCATCTTCCATGTAAACTGCACCATCTGCATACTCAAGTTCTTCCAAGCGATGGGTCACCCAC
AAAGCTGTAATTTCTTTAGATGTATCCATGGAGTTTCTCACTGCTTTTATTACTCCAATCTGATCAGTTTCATCC

AAAAAAGTAGTCAGTTCATCAAGTAACAAAACTTTGCAAGCTTCTGCCAGAGCACCAGCAGTAGCAACCCTT
TGTTTTTGACCACCACTAAGAGTGTGAACTGGTTTTTCAAATACTCAGACATCCCAACTGCATCCAAAGCTCT
CGCCACCCTAGTCCTAATCTCATTTGGTGTTAAGTTCAATTTGCCAAGACCAAAAGCAACATCAGCTTCCACT
GTAGGCATCACGACCTGATGATCAGGGTTCTGATACACAAAGCTCCTTGGTCTCTCCACATACAAGAATCCAT
CATCTGGACTCAATA
>TCONS_00073151
CCGCCTCCGCCGCTTCCGCGAGACTGAGCTTCGTTGACGGTGATGTTACGACCGTCAAGGTCCTGGCCGTTCA
TCCCTTCAATAGCGTCCCTCATGGCTTTCTCATCCTTGAAGGTAACAAATCCAAATCCTCTAGATCTACCAGTT
TCTCTGTCATTAATAATCGTTTCGGGGAAAGTGGAATAATAGTAACAAATAGTAACAGTAACAGATAAACCA
GAGAGTAGTAACAGTAACAGTAACAGTAACAGAGAGAGTAGTAACAGATCAATTTTTCCAGTAACAGAGGG
TCAACGAAGCCGATTCAAATCGGCTCGAATCCGATCTCTGCTCTGTTTTGCAACAGACCTTCGAGTCGAGAAT
TTCGCCGAACTGAGAAAAAGCATCCCCAAGTGTTTGGTCAGTGGTAGCCCATGCTAGCCCACCGACGAAGCA
CCTGTATTCAACTTCTGCCATTTTTTTCTGATTAATAAGAA
>TCONS_00073197
GCTCTCTCGCTCTCATCTCTGAAGGCTTCTTCCTGTTTTCCTTGGCAAACACAACAGTGATCTCCCGCCCAAGT
AGAACCTGGCCATCCATCTCATATTTGGCATCTGCAGCATCAGCAGGGTCCAAGTACTGCACAAAACCAAAG
CCACGCAGCTCCCCAGTGTAATAATCTCGTGGCAAATAAATATCCTTAATGGGACCAAACTCGCCAAACGGC
CCTCGAAGATCTTCAGGCCTGCAATCACGGCGAAGGTTGCGAACCAAGAGACTAGTAGGGGGATCACTCAG
ACGACCACCATAGCGACCCCTCGGGCTAGGGCTGCGACGTCTCTTGCTGTAACCTCTGTGTGGACTAGGACTG
TAGCTGCAGCTTCTTCCCCTCATACTTTCACCTGTCTGCAAGCTCAGACATTACAAACAACCAAAACTAACAG
GAAATAATAAAACAAGGACCAAAATACATTTTTTTTCTTCTTCCTATTGCATTTT
>TCONS_00073198
GCTCTCTCGCTCTCATCTCTGAAGGCTTCTTCCTGTTTTCCTTGGCAAACACAACAGTGATCTCCCGCCCAAGT
AGAACCTGGCCATCCATCTCATATTTGGCATCTGCAGCATCAGCAGGGTCCAAGTACTGCACAAAACCAAAG
CCACGCAGCTCCCTTGTGAGAAACAATGTTTTTAGACTTCAAATCCTTCAACACTTGAATATCTTCATTATTGT
ATTCATACCCAACTTCCTTCAATCTTCATGTACATTATGATCAACTTGACAGAACTCTTATTACTTCACTCGTGT
CTTCAACACCAGTGTAATAATCTCGTGGCAAATAAATATCCTTAATGGGACCAAACTCGCCAAACGGCCCTC
GAAGATCTTCAGGCCTGCAATCACGGCGAAGGTTGCGAACCAAGAGACTAGTAGGGGGATCACTCAGACGA
CCACCATAGCGACCCCTCGGGCTAGGGCTGCGACGTCTCTTGCTGTAACCTCTGTGTGGACTAGGACTGTAGC
TGCAGCTTCTTCCCCTCATACTTTCACCTGTCTGCAAGCTCAGACATTACAAACAACCAAAACTAACAGGAAA
TAATAAAACAAGGACCAAAATACATTTTTTTTCTTCTTCCTATTGCATTTT
>TCONS_00073301
CATTATATTCCGAAAGAAATAATAAATGGACTAATTGTTTAGGGCATTTTGCCATATTTGAGGGCGTTGAGAT
CAGCATAGTACTTTGTTATCCATCCTTCAATGATTTCAACCTCTGTTTTCCTTTGCATGGTTAACCATTCTGGAT
CAGTTGGCTTGCTAGTAAGAATGTCTAGACAATGGGAACCGTTACGCGTGTGGACAGCTCGTAGACTATGAG
ATATGTCTTGCAAAACCCCTGCACTACTGTAAGGATCTCTTAGCCCATTAGAGAAAATGATGTTGCTAGCAAA
CCTATGAAGAATTAATTTTATGTCATGTCCACCATAATAAGTTGTGATCCAGTGTGGACGAGGAGAGACACC
ATAGTTGCTCTTGCAGTCGTCTATAAACTCATCCAAATTAAAAGGAGCTGAATAGAACATTGTATCATTTTTG
CCTCGCCCTATAGGCATCACCATCTCACTGCATGTTTGCCATCTCCATCCCATGTTGGTTTCACTAGGTTGATT
ATACACATTTGTGTTGTAACAACTTAAATTCCCTTGAGATGCAACAATTCCAGCATAAATACGATCAAGAACA
TGACTTCCTTTGTGTGCACCATCAATTCCTCCACAAACCACTGTCACTGGATATGTTGGTGGCTTATTATACTG
AGCTGCTGTTGCATATATTGAATCCAAATAATCCTTCAACTCAGAAG

>TCONS_00073378
CAAGCTTCTGATTTCGTGAAGGTTGGGGGAGGGGGTTATTTAAAACAAACCAAGACTTTGAATTCACATCATC
TTTAAAAAACTAATCGGTTAAGGAAGTAATGACTCATATCAAGTTACAGTAACGATTAAGTTCAACCGAGCT
GGGTCCTAAAGTTGAACAACCTACATTTCACCGAGTCCATACCACCTTGAAACACGCATAAAGTAGGTCCAC
CGAAACAGAAAAGACGACTCAAAGAATTGTGCCTCCAGTTTGATGCAACATCGCATCAATTTCATCACCATC
TTCCATCTCCAGCTCATCTGGTGTCTGTTCTGCTCTAAGACGACGACCGTCAAACAAGAAAGCAATTGAATTG
AAATCCACCGACTGTCGGTCGCAATAAGCATTCATCAGCTTCTTCAGCTGGGTGCTTCGTTTGATCCTAAAGA
ATACTTCATTGCCATCCTGGCCTTTGACTTTGAGATTGATGTGACCAGCCTGATCGCCGGCGGGCTTCTTGTCT
TCTTCTGCTCCCGACATTGTTCGCTTCTCCTCCTCTATCTCTGCAAAAAGAAAAAAG
>TCONS_00073395
AGGTCAAAGAAGAAGATGGGTCATCATCATAATGAAGGAAATAGACCATATGATGATCCATTCTTGGCATGT
TGTTGTTGCCCTTGTTTTGTAGTTTCTTCTACTTTCTCTGTGTTAAAGAGGTGCCTCTTTGTAGTATGTTTCCCTGT
TTTGCAGTGCTTTGGATTGGATGAACACAGACATCAACATCATCACCACAGACATTTCTGACAACCCCAATTT
AAAGGAAAACACTGATTTTTGAGTCT
>TCONS_00073420
CTATCATCTGAACTGAAGAGTTGATAACTTCACTTGTCCAAGTCAGGATTCTTACACTAGGGCTTCAACATCG
TCTGGGTTCACGGTAGCTCCATCTTCAGTGTCTTCTGACCTCAACTGAGCAGAAATGCCATCAATAGAAGAAT
TAATCTGTTCAATCTTCTTTGCAAGTATCTTGCGTTGTTTCTCCAATTCCTTTTCTTCATCATATATAAACTTGGG
CAATTTTTTCATGATGTCCTCCTTATCTGTTCCAGATATTGCCTTACTGATCTGAGGTGCATAGACACAACCAA
GTGTGCCAACAATTAATCCTCCCAAAACAAAGCCACCAATAAAGAAGCCACCATTGCTTGGCCTTCCACCAT
CACTATATCTCGCTTGAATCAATGATCTTCCAGATGAGGATGGTTTTGCTCCACGCCTAGCACTGAAGAACAG
CTTGGTGGAACAGCCACTTTCGTCAATTGATTTCAAAGGAGAGCCAGATGATAAATGGAGAGAAGGGCTTTT
GGATATAAGCAGCGAATT
>TCONS_00073488
CTTCAGTGGAGGTGAACAAAGCCGCTTCTCAAACTTTCTTTCTGTCCCAGGAGACGTCTTTTTCAGCGAACATT
CTTGAATACTTAAGGAGTTGAAGCGTGATGAATCCGCCAACACCTTTACCATCTCTGGTAATTGTTTGGACAC
GGCATGAAGTCGTTCATTCACTGGAGATATTCTCCATGTTACTTCATCACCTCCATCATCCTGACTTTGCTTGAT
CAGCATTCCTGGAGAAAACTTGAAGCGCTTCACAGACACGGACTTGCAAAACTCAGGCGTTATTTCTGCTTG
ATTTTTCTCCTCCCCTGAACCTGCAGTTAC
>TCONS_00073585
CATTTACGTACATTTGAACTAGCCTAATGCATCATAAAGGATTCTCACTGATTTTTCCATTCTTGAATAGCTCTT
AGCATCCTTGCAGGTCTCGCCACCGGAGATAAACCAACCCTGCATGGTAATGAGTATAAGCCCCTGCCACAA
CAAGTACATGAAATAGTTGGTGGCTGTGGCCAGCAATATCAAACTTCCCGGGCATCCATCTTTCTGGAACTCT
CATGGCATATACCAGTGCTCCAATGCCATAAAATATGCCCATCAAAAGTTCATATCCAGTTGTGTGAAGCGCC
TCGGGTTGGTGCCAAAATAAAACCAGCTTGTGCAGAATAGGTACAGCACCTGAAAAGCCCATTCCAAAGAA
TAGAGATGCTCGAATGGTTCGATATTCTGGGGTCTGGAACACAGGAAGGAGTGAACCCAAGATGGTGGCAAT
TCCCAGGAGGGTAATAAATCCCAGGTACAGGTTGCAAAAGAAGGGGTAACACATGAAGGAATAATAGACG
GGAGGGTAAAATGAAGTAGATATCAGGGCAGCGATACCAGCATAGTCGAGCCTCAGCATGATGTATGATAA
ACGCTCTGAATGGCAAGAAAGTAAATGACATGTGCTGCTTGCTAACAAGCAAAACATGGCCCCACCAAGGA
AGGCGTAGAATGGCCAGCGTGTTATTGGTCTCACCAGCAATGGTGCTATTATATTTGCCACATCTTCCTTTACA
CTCCGGTCCAAACATTGAGGGTCTCATTGTGAATAGTGAAAATGCTGAGTAAGGCCTGCTTCAAAGGCCATTC

GGCCCGGTAATGGCCAAGAATGTATTCGTTGTCTTTCAAATAGGCAGGCAACGAATGGTACTCCACCAGCTG
GTACTTCACCTTCTTCCATAATCTCTTCCCTTTCCCTTCCTTTGAACCACACAACTTTTGATCTTCACAAC
>TCONS_00073612
TTTGTCTTCTATCACCTTCCTCTCCACATCAATGTCTAAGTAAGAGTTTTTGTTGAGTGTCTCACCCAAAATGA
ATGATTTCTCTGGTATAACTCCTTCCCCTTCCCCACAAACATAAGCAGTGGATCCGTGAACAATAATCTTCACT
TTTTCACATCTTTTAGCAAACTTGAGGACGTTGACGGCACCTAGGACATTGATATTCATGGCAGTATCATATCT
CTCATCAAATCTAGTAGTTGCAGCTGAGTTTATGATTATGTCGATTTCTTTGA
>TCONS_00073728
AGAAGATTGAGCTTCATTAAGGATCTCATCAGCTTTTTCCTCTACCATTTCCCTATCTGCAATTGCAATTGATC
TTCCATATAGATTTTCCAGATTGGAAAGGCCATTGGGAGAAAGACCAAAAGAGAGAAGTGAAGTGTAAGCTT
CCATATACACTTTGCTGCATTTTTTCACAGTTTCTAGACCTTTCAATGTTATGTCTTTTTC
>TCONS_00073836
GGAGGACCACCTTGTCTGTGCTTCTTGTCCCAAAGGTCCTTAATTCCATTCGAATCACTATTCTCTGGATGCAA
ATCATTATGAGCTCTCTCATTGTCTATTCTGGGTTCTCTGGTATTTCCAAAAGAACTCCTAGAAGAACGTCTTG
GATGTTGTATAACCGACGAATGCCGTTTCAGAGAAGACAAGACATCATCTGTAGTACTTTTAATTTCAACCCC
AAAAACACAGGAATCCCATTGCTTCAACCACATTAGTACCTGACGATTTGTTTGCTCATCACTGAGAA
>TCONS_00073859
TTGGGAGGAATTGACTTGAATAATTCAGGGAGTGTTGTCGTTAGAGAAGATAAAAAGCTTTTGACCATGCTGT
TTCCCGATGGACGTGATGGGCGAGCTTTTACTCTTAAGGTATTTCTCTTCTCTCTACTCTCATCTCTCTCTCTCTC
TCTCTCTCTCTCTCTCTCTCTCTCTCTCTCTCTCTCTCTCTCTCTCTTCTAAACACAAATATCATCCCAGGAAC
TCTTAATGCATGTTACAAGCAATTTACTTGGAGGAGTTGGGCAGCAAAATAAGTTGAATTTCTGGTTATTCTTT
TCTAAACAGTTTGAACCTCCATGG
>TCONS_00073884
TGCTCGATCTTGGGTCTGAGGTATGGGTCGAATCATGATAAAGAAGGTTCGGTGGTTGTATAATTCCCAAGCG
GAAAGTCGTATACTTTAAAAGTAGATGGAGTGATCAAGCGGCCCTTCTTGCGCCAACATACATCCAATGGCA
TAGAGTGATGCGTCAGTATGAACATGGAACTCCTTATTCCAATCCGGAAACCGAAGGATTGGGGCTGACAGC
AAACACTCATCTGCATGCTTAAAGGCTTAATCCTGTTCCGGTTCCCAAGCGTTATACCTTTCTTCTGCAATGAT
TCCATTCTGGGATTCCGCGCTTCGAAAAGTCTTTTTTGATGCGTCTGTAATAGCCTATGTGTCCCAAAAAAGAA
CGTGCTACGCCCGGCCCCCTTTTCAGTAGATGAGATTAGGATGAAAGGGCTTTCTTTCATTAACATTCAGTGA
AGGTGCATAGCTTCTTTGAATGTCCCGCTGATTGACTACTACATCTAGGGACGGGACTGATCCCGAAAGAGA
GGTCTTTCTTTATGGTTATGAAATCGCTTTGATTGAAAAGCAAGTCGATGATGCCATAAGTCAAACGGGCGGG
TGAGGCGAGAGTCCTTCACTGCACTCACTGGAGAGAGGGTAACATATCCTATATAGTAGTAAGGTCTTATCCC
ATTATTAGTAGCCGAAGTGTGGGCCGGCTAATTCAGGAAAAAACTTTCACATGTGCATAGGAAGACTAGAAC
ATGTTTAATAAGCCTTAGTCGACCTCCGGAGGACAACAATTTTCTTTTCCAATCTGAGATTCTCTTTTTCATTTT
CTCCAGTAGAGGAAGGTTCTCTCTTTATTCTCTTGAGGGACAAGAAAACATCCAGGTACTTGATAGGAAAACT
ACCATTTTTTTTTTGATTCCTCTTCAATGATCTGGAATTTCTGCGAGGAGCTCTGTTCGAGAGGAAGCACTGGC
TCTTATCGAAATTGACCTTTTGGCCATTACAACATTACAATCAGCTTCTTACTTCTTTCCAGAAAGATTTTCTGC
TTCTTAAGATTCCGTTTATGGCTATTATATAATATTAGAACATCGTTATCAAAGAGAAAGTAGGAGAGTGCTG
GGCACGATCTCGAAGCATGGTAAGATGTTATCATTTTCAATGATACCAAGTCCCGGCTTAGCACTTCTTCAGC
TAAAATCAACAGGCTAGGTTTGGAACCCTTGTCGCAGCCCTCGAGAAGCTGGAAAGTACCCATGTGGCACAC
CATCCACAAGCACTCCAAAGTCTTCATTACATAAACATCCATGTATCAAATTCCGCCATTTATCACAAAAGCC
CATTCGATCCAAAAAGATGCTTTTGCCATATCCTGACTTTCTTTTGACCCCCCTCCCTCCTATGTTGTCTTTAGG

TAAAAATCCCCTATCACTTAAAGCCACCTCGTGAGCCAAGGAGATCTTCTCTACAATAGTGAGTCCTTTAACA
AAAGCGCCCCTTTTTCCTTAACTATTTTTATAGGGAGAATCAAACTAATTCTTCGAGCTAGAGAGAATTTTATA
GAAAAAGTTACAAAGCTCTCCTTCAAATA
>TCONS_00073885
TGCTCGATCTTGGGTCTGAGGTATGGGTCGAATCATGATAAAGAAGGTTCGGTGGTTGTATAATTCCCAAGCG
GAAAGTCGTATACTTTAAAAGTAGATGGAGTGATCAAGCGGCCCTTCTTGCGCCAACATACATCCAATGGCA
TAGAGTGATGCGTCAGTATGAACATGGAACTCCTTATTCCAATCCGGAAACCGAAGGATTGGGGCTGACAGC
AAACACTCATCTGCATGCTTAAAGGCTTAATCCTGTTCCGGTTCCCAAGCGTTATACCTTTCTTCTGCAATGAT
TCCATTCTGGGATTCCGCGCTTCGAAAAGTCTTTTTTGATGCGTCTGTAATAGCCTATGTGTCCCAAAAAAGAA
CGTGCTACGCCCGGCCCCCTTTTCAGTAGATGAGATTAGGATGAAAGGGCTTTCTTTCATTAACATTCAGTGA
AGGTGCATAGCTTCTTTGAATGTCCCGCTGATTGACTACTACATCTAGGGACGGGACTGATCCCGAAAGAGA
GGTCTTTCTTTATGGTTATGAAATCGCTTTGATTGAAAAGCAAGTCGATGATGCCATAAGTCAAACGGGCGGG
TGAGGCGAGAGTCCTTCACTGCACTCACTGGAGAGAGGGTAACATATCCTATATAGTAGTAAGGTCTTATCCC
ATTATTAGTAGCCGAAGTGTGGGCCGGCTAATTCAGGAAAAAACTTTCACATGTGCATAGGAAGACTAGAAC
ATGTTTAATAAGCCTTAGTCGACCTCCGGAGGACAACAATTTTCTTTTCCAATCTGAGATTCTCTTTTTCATTTT
CTCCAGTAGAGGAAGGTTCTCTCTTTATTCTCTTGAGGGACAAGAAAACATCCAGGTACTTGATAGGAAAACT
ACCATTTTTTTTTTGATTCCTCTTCAATGATCTGGAATTTCTGCGAGGAGCTCTGTTCGAGAGGAAGCACTGGC
TCTTATCGAAATTGACCTTTTGGCCATTACAACATTACAATCAGCTTCTTACTTCTTTCCAGAAAGATTTTCTGC
TTCTTAAGATTCCGTTTATGGCTATTATATAATATTAGAACATCGTTATCAAAGAGAAAGTAGGAGAGTGCTG
GGCACGATCTCGAAGCATGGTAAGATGTTATCATTTTCAATGATACCAAGTCCCGGCTTAGCACTTCTTCAGC
TAAAATCAACAGGCTAGGTTTGGAACCCTTGTCGCAGCCCTCGAGAAGCTGGAAAGTACCCATGTGGCACAC
CATCCACAAGCACTCCAAAGTCTTCATTACATAAACATCCATGTATCAAATTCCGCCATTTATCACAAAAGCC
CATTCGATCCAAAAAGATGCTTTTGCCATATCCTGACTTTCTTTTGACCCCCCTCCCTCCTATGTTGTCTTTAGG
TAAAAATCCCCTATCACTTAAAGCCACCTCGTGAGCCAAGGAGATCTTCTCTACAATAGTGAGTCCTTTAACA
AAAGCGCCCCTTTTTCCTTAACTATTTTTATAGGGAGAATCAAACTAATTCTTCGAGCTAGAGAGAATTTTATA
GAAAAAGTTACAAAGCTCTCCTTCAAATA
>TCONS_00073886
TGCTCGATCTTGGGTCTGAGGTATGGGTCGAATCATGATAAAGAAGGTTCGGTGGTTGTATAATTCCCAAGCG
GAAAGTCGTATACTTTAAAAGTAGATGGAGTGATCAAGCGGCCCTTCTTGCGCCAACATACATCCAATGGCA
TAGAGTGATGCGTCAGTATGAACATGGAACTCCTTATTCCAATCCGGAAACCGAAGGATTGGGGCTGACAGC
AAACACTCATCTGCATGCTTAAAGGCTTAATCCTGTTCCGGTTCCCAAGCGTTATACCTTTCTTCTGCAATGAT
TCCATTCTGGGATTCCGCGCTTCGAAAAGTCTTTTTTGATGCGTCTGTAATAGCCTATGTGTCCCAAAAAAGAA
CGTGCTACGCCCGGCCCCCTTTTCAGTAGATGAGATTAGGATGAAAGGGCTTTCTTTCATTAACATTCAGTGA
AGGTGCATAGCTTCTTTGAATGTCCCGCTGATTGACTACTACATCTAGGGACGGGACTGATCCCGAAAGAGA
GGTCTTTCTTTATGGTTATGAAATCGCTTTGATTGAAAAGCAAGTCGATGATGCCATAAGTCAAACGGGCGGG
TGAGGCGAGAGTCCTTCACTGCACTCACTGGAGAGAGGGTAACATATCCTATATAGTAGTAAGGTCTTATCCC
ATTATTAGTAGCCGAAGTGTGGGCCGGCTAATTCAGGAAAAAACTTTCACATGTGCATAGGAAGACTAGAAC
ATGTTTAATAAGCCTTAGTCGACCTCCGGAGGACAACAATTTTCTTTTCCAATCTGAGATTCTCTTTTTCATTTT
CTCCAGTAGAGGAAGGTTCTCTCTTTATTCTCTTGAGGGACAAGAAAACATCCAGGTACTTGATAGGAAAACT
ACCATTTTTTTTTTGATTCCTCTTCAATGATCTGGAATTTCTGCGAGGAGCTCTGTTCGAGAGGAAGCACTGGC
TCTTATCGAAATTGACCTTTTGGCCATTACAACATTACAATCAGCTTCTTACTTCTTTCCAGAAAGATTTTCTGC
TTCTTAAGATTCCGTTTATGGCTATTATATAATATTAGAACATCGTTATCAAAGAGAAAGTAGGAGAGTGCTG

GGCACGATCTCGAAGCATGGTAAGATGTTATCATTTTCAATGATACCAAGTCCCGGCTTAGCACTTCTTCAGC
TAAAATCAACAGGCTAGGTTTGGAACCCTTGTCGCAGCCCTCGAGAAGCTGGAAAGTACCCATGTGGCACAC
CATCCACAAGCACTCCAAAGTCTTCATTACATAAACATCCATGTATCAAATTCCGCCATTTATCACAAAAGCC
CATTCGATCCAAAAAGATGCTTTTGCCATATCCTGACTTTCTTTTGACCCCCCTCCCTCCTATGTTGTCTTTAGG
TTAAAATCCCCTATCACTTAAAGCCACCTCGTGAGCCAAGGAGATCTTCTCTACAATAGTGAGTCCTTTAACA
AAAGCGCCCCTTTTTCCTTAACTATTTTTATAGGGAGAATCAAACTAATTCTTCGAGCTAGAGAGAATTTTATA
GAAAAAGTTACAAAGCTCTCCTTCAAATA
>TCONS_00073897
CCTCCCAAAAAAGCCCTTCAAAAGAAGAAAAAGCAAATAACAAACAACTGCATCACTTGACTTCTCTGGTC
ACTGCTTTACATTTCAACTGGTGAAAACATCAAAAACTAATTCAAACCACATATCGATAAGTAATGTAACGG
CCTGCAGTCTCACTTGGTCTGATAATCTTCACGACTTGGCCACGTTTTAGCCCATAATATCTCGCAATAGGATC
AGTGATCTGAATTCGAGGAAGCTGTGTTTCTTTCACTGTGTATCGTTCCAACAAGGTTTTCTTTTCTTCAGGAGT
AAGTGGCTGGTGCTCAGGAACTAGAACATGATTTTTTATGTTTACCAACAACTCTGCTTCCTGGAAAACCTCC
AAGTGAAATTTTGTAGATATTTCACTTATGCAAGTACGAGCAAAAAAGAGTCAAATTTTGTTGTACAACCAGG
ATTGCTTGGAAGACATCTTCTTTTTTCATGCGTTCTGTATAAGTTTTCATTGTCTTAACACCAACCTTTGCCTCCT
CTGGAAAGAACACATATATCAGCTCAGAGCTGTTGTTGCGCTTGGCTTTCTGAATTACAAGGTCTTCTCTCTTC
ATGTTGTCACCATACTTCTGAAGGAACTGATGTTTTGACATATTTATCTCAAACTCTCCGACAAGGTAGCCCCT
ATCAGCTAACATTTCCATCAGTGTCTTACGAACTCTGAAAAGTCTTGAGAGCTCCTCTTCTGATAGAGTCATCT
TTTCCCTACTAAATTCAAAAATTAGAAAGAATGCATTTATAAGTAAAGAATAGGGTA
>TCONS_00074025
GCGATTATGCAATTTAAATGTGTCAGCAAACTAGCAAGAGGGGCGTAAAGGAAATGAAATCCTGTTTATACA
TGTTGTTCGTCTGTACATATGACACTAATAATAGATACCTGTAGCAAGAGACAAAATGACCAAAATCTGGATT
CCCCGGAGATGTTTCTTGGTCTAACTTGAGAGCTGCAGGACCCATCTGAGCTGATTGAGAAGCGCTTTGGCAC
GAGCTGCATGTTACATGCTGATATGGCCCAGTAGGGTAAATAGTTGAACTACCACTGCTACCACTGTTACAAG
TACAGCCCGACTGGTTGCTGGCCAAACTTGTGGTAGAAGGAGATGGGCTGAGGCCTGGACTAGAAAGGTTCA
TCCTACTATCAACTGTGGCTAACCCCTGCTGCCACTGCTTAAAACAGAAGTTACATACTCTTATCCTGTCTCCA
TCTTCCCCGCCAGCCTTGGGCTCCTCATTTGGGATAGGAACAGAGTTTGATGCACATTTGGCACAGAAAACTC
GGCCACAGAGACGACAATGATGCCTGCGGTTAAATACAGTAAACTGAGAATCGCACTCGTAGCATACCCTAC
AGCTCTGGTCGGGCATCCAGAAATCCCTGGACACACTCACCTGCTCTGTCCTTCTCGGGATCCATGATTTTACT
ATGTCAACTATCTCATTGAGCTTCTTATTTTCAGGTGTATCCATGAATGCAACTTCCAGGATATCATGCTCATC
ATTATCTAATAATCATCTGCAACTGCTCGTGCTACTACCAATATGTATGTGTCTCCAATATATACAATTTCAGA
TCCTCTATACACCGAACCAACCACCAGCCACCGCCT
>TCONS_00074180
AAAGGAAGCGCCATCATGAGTTGGTATTGTATGTGGAAGCAGGGTGAAGAAGACACTAATGTGAGCTTGGAT
CGGAGCTATAGAGTTTAAGGTGTTTCCTATTTAATTCTAAAACAGTTGCAGAGATCCTGTTGAGAGGTTGAGA
ATGGGGAGAGAGGAGGAAAATGTGTAGGCTAGAACCTTATGCATTTTATGAATGATACATTGATATGGAGAG
GTTCTCATGCATCTATCAAGCTGGTCTGGAAAGTGCATTTTTTGCTAGGCAGAAGCCAAGTCTTGAGTTCGAA
GAAGCGACTAGATTAGGATTACCTGCGCCACAATACTGCTTAATGGAGGTTGGATCTGATGATTTCAGCATCG
CTTATGCAATATTCATGTGATTTTTTGCATGCACAGCCATGGAAACCTACAACGATATGCATTTGTTTTCAAGG
GTTGTTTTCAGTCTTTAAGAACTGGATAATGATGAGGAATTTGGTCAACACAATGGAATGTGCCAGGGCTGTA
GCAAACACTTCCTGATGACATTTCCGAAGGCAAAATTGGTTATTGTGGTTGTAATGGAAATGACAAAATTCAT
GTAGGATTTGCAACGAAGTGCTCTCAGTTAAGTCTTTGGTGGCTGTGATTTTCGCCGAAGTTGATATTGCTGGC

AATGAATTTAGTTTCTTTACTGAAAGGTGCTTGGATCCAAACATCTCGAGAATAGGCACTTAGGTTCTTGATCT
CAAGGTTTTAGAAATCAAGGATCAATCACTTCAGGGTGCAGATGAAACCTGAAGCTATATTTCTTATAGAGG
GATCGGCATGGGCTTCTATTAGAGTTGGACTTAAGATCCTTGGTGCAAGAACTGGAGATCAAGTTTGAATTAT
AATAGTCACCAATTCTTTGTGTATTATTTCAGCTGTTTTAGGCTCCTT
>TCONS_00074284
ACTGGTGCTTGAACTTCCATTCACAAAGCTCTCAATAGATGAAGCAGCAAATTCAGCTTGTTCTTGCTTAAAC
ATCATGTCCTTGTAGCACTTTGAGCACATGTTCATTGTTGTTGCAGTTCCAAAAAACCCACAATTGTTAACACA
AAGGGTAGGAGCTTGAGGAGCTTGGCAACCTGTGTCATTTTGCTCCATCTTAAATTTTCCTGTTCGCAGAAGA
CACAACAAAATCCTTTCTTTAGAGAAC
>TCONS_00074305
CCACCATGACGGTCAGTTGCTTTCTTCACGTAATTGAACTGTACAATACCAACAGGCAGCTGGGAACTAATCA
AGACACACAGCGATGGCATGATCTATTTTTTGCAACCAAAATGCAAACAGTTGTCAACAACTACAGGAAAAT
ACTAATGTTCATTTAGAAGAATGTCTCCCTATACCAGAGAAAACTGAAAAGTGAGCAGAGGCCACGCACCAC
ACAGATCATCAAGACTGGAAGACAGTTACAGTCGCAATGTAACCATTCTTCATCACCTCAAAGGCAAGATTT
TGGTAGCCATATACAAAGGTAGATCCAAACGGATTGCTTGTAGAGCCTTGCGTTTGGATAGCAGCTCGACCA
CAATCCCCTAGTATCTCCTTAACTTGTTCCCACTTTGTGCTGGCTGTTATAGTGCCTTTAGAGGAGTTTACATCC
TGATGATATGACCCAACAAAATCAGAACAATGGATAACAAAATTGCACTTCATATATGAATTGAAATCAGCA
TGCCCAGGATAGTTGGTGTGCAAGACAAACTTCTTGATCTTATGTGTCTG
>TCONS_00074343
TTACCAGAATATCAAGCCTCCCATATTGTGTTTGTATAAACTTTGCCAAGGACTCAATGCTCTGAGCATCTTGA
ACATCAAGTTGATGGAAAACAATATTTGAAAAACCTTGTTTATTAAGCAAAGATGTTGCTTCCATTCCCCTCTT
CTCGTTTCTGGCTGTTAAGATAACCGTCACACCTGAATTAGCAAGCTGCTTGACAGTCTCAAAGCCAATTCCC
TTGTTTGCTCCTGTAACCACC
>TCONS_00074349
GATTGTTAATGTCTCCTCCCTTAGAAGTGAACTAAAGCGAGTTCCAAACGAGGAGAGGAGGAAGGAACTTGG
AGATATTGAAAACTTGACAGAAGATAAAATTGAAAAGATTCTGCAGAAATTTTTGCATGATCTGAAACAAGA
TGCTCTAGAAGTGAACGGGTTGGCAGATAATGCTACCGGCATATAGCATATCGAAAGTGTCTCTTAATGCTTA
CACCAGAATTCTTGCAAGAAGGTGCCCGAAAATGTGTATCAATTGTGTCCACCCTGGATATGTCAACACCGA
TATAAACTGGCATACAGGAACAATGCCTGTCGAAGAAGGAGCCGAAGGCCCTGTTATGCTAGCTCTTTTGCC
AGATGGAGGACCTACTGGTTGCTACTTTGATCGTACTGTAGTAGCCGAGTATTAGATCCTGTTATGAAACAAG
GCTTAGTGAAAGACGTGTTTCTTGTATTTGGTTGGATCTCTGAGCTGGGATGATGTCTGGTGGTTGCATTAGTA
GTTGAAAAAATGAGGGAAGCAAAGAATAAAATTGGTCCTTTTATGTGTCCATATCACGAGAGTTGTTTTTGAA
TGAAACATCATTAACATTTTTCCTTGATTTAATCATCCAGTCCGATAAATCAGTTTCATGAATCACAATTACTA
TTTGGTTCTCCAGGACAAAATGAAGATGCCCTAAGGACCTTGAACATAGATCCTGTAGACTTGGTAAGAGAA
TTTGCTCAAAGATTATTATGGATTTCACATG
>TCONS_00074375
CGAGTCCAACGAATATTACAACACTTTGCACATGGGCATTGAATTTCTTCGCCTTGAGGATCTCCATAAGAGT
AAGCAAAATCTAAGAAAGATTGAACACCCTCGATATACTCTTTGCTATACCTTGGTAAATCCATCCATTCCTT
GGATGGAACATTTGAATTCCTAGCCATGACACTTAAGCTAAGCCAAATACAACTTTAAGTCCACCGCAAAT
>TCONS_00074387
CCGAGGGTCCTGACCAAGGCATTGACTTCCTGAAACTACTTGTGCCAAAATAGGGAGCACACGAGCCGAGG
GTCTCGTTGCTGAGAATTTACTTAGCCATGCTAAGGTATGATACATGAGCGCTGAGAACCATGAAGCGAGTG

GCACCTCGTGGATTGGGCCTATTCGATCAGGTTGGGATCGAACTCGTGCCGATCACACGGTGACTGAGACAG
AATTAAGTCAGGATAGTTGGAACTCCCAAAGTATAAAG
>TCONS_00074392
CTTTATACTTTGGGAGTTCCAACTATCCTGACTTAATTCTGTCTCAGTCACCGTGTGATCGGCACGAGTTCGAT
CCCAACCTGATCGAATAGGCCCAATCCACGAGGTGCCACTCGCTTCATGGTTCTCAGCGCTCATGTATCATAC
CTTAGCATGGCTAAGTAAATTCTCAGCAACGAGACCCTCGGCTCGTGTGCTCCCTATTTTGGCACAAGTAGTT
TCAGGAAGTCA
>TCONS_00074484
CTTTGGTGCAAGGAAGAATAAAATGCATTCCCAGTTACACCATTAGATTGTTAAAAATATTACCAGGAAACA
CATGTTTAAAAGTTACAGGCGCTATGCACATTATATGATTTAACAATCTTCATTACTGTTTTATATCCAGTGGT
CCAAGCAAACCACAGTTGGTGTTCTAGTATGTCAATGTCATACTAAATTTCTAGGAGTACATGCTCTTACATCT
CAGCAGCAAATGATTTACCACAACCACAGGTTTGCGTAGCATTTGGATTTTTGAAAGAGAAGCCCCCACCTA
TCAATGCATCGCTATAGTCCAGTTGCATTCCAAAGAGGAAGAGAAGACTTTTTGGATCACAAACAATATTAA
ATCCATTGTATTCAATGATCGAATCATCTGGTCTAGCATTTTCTCGTTTTTCAAACTCCATTGTATATGACATGC
CTGAGCATCCACCTTGTTTAACACCAATTCTAAGACACAGGTCCTCATTCCGCTCATTCCTCATCTTATTCAAG
TGCTTTAATGCATTGTCGGTTAAGGAAATAGCAGGTGCTACTCCTCCAGATGCGGGTGCATCCATCCAAGTCT
TCAATACAATGTAAT
>TCONS_00074652
GCCACCCACTGCCACTAAATCACCACCCTCCAAGTTAAACTGGTACTTGCTGTTGCGCCAGGAGCCGTTGCAC
CAGGTGCTAAAGCATGGGTGTTTTGTATCCATTGAGCAAAAACAGGTTGTAATTGTATAGCGGTATCTGAAAA
CATATCTTGAGGACGCCCTAAAGCGCTCATGGTATCATTATGAATATACAAACCAAAACTGTGAAAGCCTAG
AAATATACATGCCCAGTTGAGATGGGATATGATTGCATCACGATGTCTAAGGACACGATCTAATAGATCGTT
GTACCGAGTAGTTGGATCATAATCTCTTACCATAAAAATGGCTGCATGCGCGGCAGCACCAACTATGAGAAA
TCCACCAATCCACATGTGATGTGTGAACAATGACAGTTGTGTACCATAGTCAGTAGCTAGATACGGATAAGG
GGGCATGGAATACATATGGTGAGCTACAACAATGGTTAAAGAGCCTAACATAGCTAAGTTAAGAGATAATTG
AGCATGCCATGACGTTGTTAGGATCTCATATAGGCCTTTATGGCCCTGACCTGTAAATGGACCTTTATGAGCTT
CTAAAATATCTTTTAGTCCATGACCAATACCCCAGTTGGTCCTATACATGTGACCTGCTATCAGGAAAAGAAT
TGCAATAGCTAAATGGTGATGGGCAATATCAGTCAGCCACAGACCCCCAGTTACTGGATCTAATCCTCCACG
AAAAGTAAGAAAGTCCGCATATTTTGACCAATTCAAGGTGAAAAATGGGGTTGCTCCCTCGGCAAAACTGGG
ATAAAGTTGAGCCAAAAGATCTCGATTCAAGATAAATTCATGAGGAAGTGGTATCTCTTTAGGATCTACTCCA
GCGTTTAGAAATTGGTTAATCGGTAAAGATACATGTACTTGATGCCCCGCCCAAGAGAGAGAGACCCAAGTCCT
AGTAGCCCTGCCAAATGGTGATTCAGCATAGATTCTACATCTTGAAACCAAGCCAATTTTGGCGCCGCTTTAT
GATAATGAAACCAACCAGCAAAAAGCATTAACGCTGCAAAGACCAATGCCCCAATTGCTGTACAATAGAGT
TGTAATTCACTAGTTATTCCAGATGCTCGCCAAATCTGAAAAAAACCAGAGGTTATTTGTATTCCTCGGAAAC
CCCCGCCTACGTCACCATTTAATATTTC
>TCONS_00074653
CCCAGAAGGTAATGGGTTACTCCTACAGCACGTCCTTGTATAATGCTCAAGGCTCTCGGCTGAGTAGCAGGA
GCAACTTTTAATTTATTATGAGCCCAAACGATGGATTCAATAAGTTCTTGCCAATAACCACGTCCACTGAATA
GAAACATTAAACTAAAAGCCCAGACAAAATGAGCACCTAGGAAAAAAAGGCCATATGCAGATAATGAAGA
ACCATAAGACTGAATTACCTGGGATGCCTGTGCCCATAAGAAATCGCGGAGCCACCCATTAATAGTAATAGA
ACTTTGCGCAAAGTTTCCTCCCGTGATATGAGTTACTACCCCTTGATCACTTACACTGCCCCAAACATCTGACT
GCATTTTCCAACTGAAATGGAATATTACTACCGAAATTGCATTGTACATCCAGAATAGTCCTAAGAAGACAT

GATCCCAGGCCGATACTTGACATGTACCCCCTCTTCCAGGTCCATCACAAGGAAAACGAAAACCAAGATTTG
CTTTATCCGGTATCAAACGGGAACTGCGAGCAAATAGAACACCTTTCAAGAGTATCAATACCGTCACATGAA
TCGTAAATGCATGAATGTGATGTACCAAGAAATCCGCGGTTCCTAATGGAATAGGCAACAAAGCCACCTTGC
CACCCACTGCCACTAAATCACCACCCCCCCAAGTTAAACTGGTACTTGCTGTTGCGCCAGGAGCCGTTGCACC
AGGTGCTAAAGCAT
>TCONS_00074654
CCCAGAAGGTAATGGGTTACTCCTACAGCACGTCCTTGTATAATGCTCAAGGCTCTCGGCTGAGTAGCAGGA
GCAACTTTTAATTTATTATGAGCCCAAACGATGGATTCAATAAGTTCTTGCCAATAACCACGTCCACTGAATA
GAAACATTAAACTAAAAGCCCAGACAAAATGAGCACCTAGGAAAAAAAGGCCATATGCAGATAATGAAGA
ACCATAAGACTGAATTACCTGGGATGCCTGTGCCCATAAGAAATCGCGGAGCCACCCATTAATAGTAATAGA
ACTTTGCGCAAAGTTTCCTCCCGTGATATGAGTTACTACCCCTTGATCACTTACACTGCCCCAAACATCTGACT
GCATTTTCCAACTGAAATGGAATATTACTACCGAAATTGCATTGTACATCCAGAATAGTCCTAAGAAGACAT
GATCCCAGGCCGATACTTGACATGTACCCCCTCTTCCAGGTCCATCACAAGGAAAACGAAAACCAAGATTTG
CTTTATCCGGTATCAAACGGGAACTGCGAGCAAATAGAACACCTTTCAAGAGTATCAATACCGTCACATGAA
TCGTAAATGCATGAATGTGATGTACCAAGAAATCCGCGGTTCCTAATGGAATAGGCAACAAAGCCACCTTGC
CACCCACTGCCACTAAATCACCACCCCCCCAAGTTAAACTGGTACTTGCTGTTGCGCCAGGAGCCGTTGCACC
AGGTGCTAAAGCATGGGTGTTTTGTATCCATTGAGCAAAAACAGGTTGTAATTGTATAGCGGTATCTG
>TCONS_00074655
CAGAAGGTAATGGGTTACTCCTACAGCACGTCCTTGTATAATGCTCAAGGCTCTCGGCTGAGTAGCAGGAGC
AACTTTTAATTTATTATGAGCCCAAACGATGGATTCAATAAGTTCTTGCCAATAACCACGTCCACTGAATAGA
AACATTAAACTAAAAGCCCAGACAAAATGAGCACCTAGGAAAAAAAGGCCATATGCAGATAATGAAGAAC
CATAAGACTGAATTACCTGGGATGCCTGTGCCCATAAGAAATCGCGGAGCCACCCATTAATAGTAATAGAAC
TTTGCGCAAAGTTTCCTCCCGTGATATGAGTTACTACCCCTTGATCACTTACACTGCCCCAAACATCTGACTGC
ATTTTCCAACTGAAATGGAATATTACTACCGAAATTGCATTGTACATCCAGAATAGTCCTAAGAAGACATGAT
CCCAGGCCGATACTTGACATGTACCCCCTCTTCCAGGTCCATCACAAGGAAAACGAAAACCAAGATTTGCTT
TATCCGGTATCAAACGGGAACTGCGAGCAAATAGAACACCTTTCAAGAGTATCAATACCGTCACATGAATCG
TAAATGCATGAATGTGATGTACCAAGAAATCCGCGGTTCCTAATGGAATAGGCAACAAAGCCACCTTGCCAC
CCACTGCCACTAAATCACCACCCCCCCAAGTTAAACTGGTACTTGCTGTTGCGCCAGGAGCCGTTGCACCAG
GTGCTAAAGCATGGGTGTTTTGTATCCATTGAGCAAAAACAGGTTGTAATTGTATAGCGGTATCTGAAAACAT
ATCTTGAGGACGCCCTAAAGCGCTCATGGTATCATTATGAATATACAAACCAAAACTGTGAAAGCCTAGAAA
TATACATGCCCAGTTGAGATGGGATATGATTGCATCACGATGTCTAAGGACACGATCTAATAGATCGTTGTAC
CGAGTAGTTGGATCATAATCTCTTACCATAAAAATGGCTGCATGCGCGGCAGCACCAACTATGAGAAATCCA
CCAATCCACATGTGATGTGTGAACAATGAGGAAGTGGTATCTCTTTAGGATCTACTCCAGCGTTTAGAAATTG
GTTAATCGGTAAAGATACATGTACTTGATGCCCCGCCCAAGAGAGAGACCCAAGTCCTAGTAGCCCTGCCAA
ATGGTGATTCAGCATAGATTCTACATCTTGAAACCAAGCCAATTTTGGCGCCGCTTTATGATAATGAAACCAA
CCAGCAAAAAGCATTAACGCTGCAAAGACCAATGCCCCAATTGCTGTACAATAGAGTTGTAATTCACTAGTT
ATTCCAGATGCTCGCCAAATCTGAAAAAAACCAGAGGTTATTTGTATTCCTCGGAAACCCCCGCCTACGTCAC
CATTTAATATTTC
>TCONS_00074673
CTTGTATACTGTTGAGGCTACTGTTAGTGAGGAAATATTTATACCATTGGGCCGACAGTTTTGGACTTCGATCC
AACGTGAGAGGATGGACATGATAAAGCCCAAATTTAATGGTGTAACCAAATCTCCATTCGAAATTATCCATC

AAGCTCCATATGAAATAACCACGCACATCCGCACCATTCCTGATTGAACGAGCCAAAGATGCTAGGTATGCT
TTGTGATATTTAATTCGCTTCATGTCTTGATCCAAGTCATATCCTCCTCCTTGATT
>TCONS_00074683
GGAGGGGCTCCCAAAGCCCAAGCGCTAAGCACAGACAACTCACGTGCTACCATATCAATACCTGGATCCGC
ACGGTCAACTCACGTTCTACGCGGACAACTCACGCGCTATGGTATCAATACCTGAACCCGCACGGTCAACTC
ACGTGCTACGCGGACAACTCACGCGCTATGGTATTAATATTCACACAACCAGGCCCTCGGCCTTACTCAATC
>TCONS_00074684
GGAGGGGCTCCCAAAGCCCAAGCGCTAAGCACAGACAACTCACGTGCTACCATATCAATACCTGGATCCGC
ACGGTAAACTCACGTGCTACGCGGACAACTCACGCGCTATGGTATCAATACCTGAACCCGCACGGTCAACTC
ACGTGCTACGCGGACAACTCACGCGCTATGGTATTAATATTCACACAACCAGGCCCTCGGCCTTACTCAATC
>TCONS_00074685
TCTGCGCTCACTGGGGGTGTGTACAGACTCCGGAGGGGCTCCCAAAGCCCAAGCGCTAAGCACGGACAACTC
ACGTGCTACCATATCAATACCTGGATCCGCACGGTAAACTCACGTGCTACGCGGACAACTCACGCGCTATGG
TATCAATACCTGAACCCGCACGGTCAACTCACGTGCTACGCGGACAACTCACGCGCTATGGTATTAATATTCA
CACAACCAGGCCCTCGGCCTTACTCAATC
>TCONS_00074686
CTGGGGGTGTGTACAGACTCCGGAGGGGCTCCCAAAGCCCAAGCGCTAAGCACGGACAACTCACGTGCTAC
CATATCAATACCTGGATCCGCACGGTAAACTCACGTGCTACGCGGACAACTCACGCGCTATGGTATCAATAC
CTGAACCCGCACGGTCAACTCACGTGCTACGCGGACAACTCACGCGCTATGGTATTAATATTCACACAACCA
GGCCCTCGGCCTTACTCAATC
>TCONS_00074687
GGTACATGATTGAGTAAGGCCGAGGGCCTGGTTGTGTGAATATTAATACCATAGCGCGTGAGTTGTCCGCGTA
GCACGTGAGTTGACCGTGCGGGTTCAGGTATTGATACCATAGCGCGTGAGTTGTCCGCGTAGAACGTGAGTTG
ACCGTGCGGATCCAGGTATTGATATGGTAGCACGTGAGTTGTCTGTGCTTAGCGCTTGGGCTTTGGGAGCCCC
TCCGAAGTCTGTACACACCC
>TCONS_00074688
GGTACATGATTGAGTAAGGCCGAGGGCCTGGTTGTGTGAATATTAATACCATAGCGCGTGAGTTGTCCGCGTA
GCACGTGAGTTGACCGTGCGGGTTCAGGTATTGATACCATAGCGCGTGAGTTGTCCGCGTAGCACGTGAGTG
GACCGTGCGGATCCAGGTATTGATATGGTAGCACGTGAGTTGTCTGTGCTTAGCGCTTGGGCTTTGGGAGCCC
CTCCGAAGTCTGTACACACCC
>TCONS_00074689
GGTACATGATTGAGTAAGGCCGAGGGCCTGGTTGTGTGAATATTAATACCATAGCGCGTGAGTTGTCCGCGTA
GCACGTGAGTTGACCGTGCGGGTTCAGGTATTGATACCATAGCGCGTGAGTTGTCCGCGTAGCACGTGAGTTT
ACCGTGCGGATCCAGGTATTGATATGGTAGCACGTGAGTTGTCTGTGCTTAGCGCTTGGGCTTTGGGAGCCCC
TCCGAAGTCTGTACACACCC
>TCONS_00074690
GGTACATGATTGAGTAAGGCCGAGGGCCTGGTTGTGTGAATATTAATACCATAGCGCGTGAGTTGTCCGCGTA
GCACGTGAGTTGACCGTGCGGGTTCAGGTATTGATACCATAGCGCGTGAGTTGTCCGCGTAGCACGTGAGTTT
ACCGTGCGGATCCAGGTATTGATATGGTAGCACGTGAGTTGTCCGTGCTTAGCGCTTGGGCTTTGGGAGCCCC
TCCGGAGTCTGTACACACCCCCAGTGAGCGCAGAGTGTTGAGTGTATTG
>TCONS_00074691

GGTACATGATTGAGTAAGGCCGAGGGCCTGGTTGTGTGAATATTAATACCATAGCGCGTGAGTTGTCCGCGTA
GCACGTGAGTTGACCGTGCGGGTTCAGGTATTGATACCATAGCGCGTGAGTTGTCCGCGTAGCACGTGAGTTT
ACCGTGCGGATCCAGGTATTGATATGGTAGCACGTGAGTTGTCCGTGCTTAGCGCTTGGGCTTTGGGAGCCCC
TCCGGAGTCTGTACACACCCCCAG
>TCONS_00074692
GGTACATGATTGAGTAAGGCCGAGGGCCTGGTTGTGTGAATATTAATACCATAGCGCGTGAGTTGTCCGCGTA
GCACGTGAGTTGACCGTGCGGGTTCAGGTATTGATACCATAGCGCGTGAGTTGTCCGCGTAGCACGTGAGTG
GACCGTGCGGATCCAGGTATTGATATGGTAGCACGTGAGTTGTCCGTGCTTAGCGCTTGGGCTTTGGGAGCCC
CTCCGGAGTCTGTACACACCCCCAG
>TCONS_00074721
TACTTTCGAGGATTATGTTCTGATATCAACTCCTGGATAGGTCCATATAACCTTTCTGTTGGAAGAAAACTTGT
CGTGCAAAAGCATGCCATTGATATCCTTGGACCAACCTTATTTGCCAGTACTATATGTTCAACACTTTTGAACT
TGTCATTTGATATAAGCTGAAAAAGATCTCCAATATTGACCACGAGAGCTCCGGGAGTAGGGGGAACATCTA
CCCAATGGTTTTGATGTAGAACTTGGAGACCGCCAATATTGTCTTGGAGAAGCACGGTGAGGAAGTTACTGTC
A
>TCONS_00074844
AGCGACAACAGTTCCACCTCCGCCGAAGCGCACTCATTGTGTGGGATGATATCGAAGATTTTGCTCATAAAA
GTTTATCAACATAAGTGGATTATTCATCTTCAGAAACTTGTTATCCACCATAACTTCCTTTCCATCAGACCTCTT
AGCTAAAAAGGTCACGGACACATCCTGAAAATCACTCGATACAGATGCCGAATAGCTCATAGGCTTTATAAT
TTCTGCTATGGCACAGGCATCTTTGGAATCATCCCACTTATTTTTGCAACCCAAAGCATTGCCCACAAATCCAT
GATCGTGAATTCCTTCCTGCGTGAATGCAACAAAAGGACCACTAGTAGCATTTGGTACAGCATCTTGGGAATC
ATTCATTACACCTGAGGCAGTTTCCGGTTTAAACCTCCGAACAGAAGCAGGCTTCCTCCTTTTAGCTCTGGTAC
AACGACCAGATTGAAGTGATTCTGTTCCATTGGCTGCTGAAAATCCACTTGTAAGGCCATCACCAGATCTATC
CACAGATGTCTCTTTTGTTGCTATCCCTCCGTTTAATTCACTAAGCTTTAAATCCAATTCATTTTTCTCATTAATT
TCTTTTATAGAGGAATCCAAATTCAAATCATTGTCGTTCGCCACTCCGTCCACTTGGATATCATTCAAACCACT
ACCACTACTATCCACATGATTAATAGCATCCGAGCTAGCCAGAGGAGATGAAAGCATAGGTTCCTCCATTAT
GCTAACCTTCACTGCAGGCACATCATTTGTAGC
>TCONS_00074996
AATAAATCTAAGTGTAGTGCTTGGTGTATTGATTTTTTTTTGGAAAGGGAGTGTGTGCGAGTTGTTTATTTCAA
GAATAGGCTGGACCACCCAGCTGTACCATTTTTCGTTATAACTAGGAAAGAGAGGTGCATGATCTCGCGAAT
TACTTCTGAATAAATTCAGAAATTATATGTAAGAACCATAGCATTTCGCGATTCATTGGTAAATCGATTTTGAT
TCTCTATTAACCAATAATGTGGAACTATTAACATGGTTAAAACAAACTGTTTGAAGTCTAGACGCAGCATGGT
ACTCTTTCTACCACTATGTTAATATAGAGGTGGTTTCAAAATAAATATTTTATCGATATAGGATACTCATATTG
ATAAAATGATTTGAACC
>TCONS_00074997
AATAAATCTAAGTGTAGTGCTTGGTGTATTGATTTTTTTTTGGAAAGGGAGTGTGTGCGAGTTGTTTATTTCAA
GAATAGGCTGGACCACCCAGCTGTACCATTTTTCGTTATAACTAGGAAAGAGAGGTGCATGATCTCGCGAAT
TACTTCTGAATAGATTCAGAAATTATATGTAAGAACCATAGCATTTCGCGATTCATTGGTAAATCGATTTTGAT
TCTCTATTAACCAATAATGTGGAACTATTAACATGGTTAAAACAAACTGTTTGAAGTCTAGACGCAGCATGGT
ACTCTTTCTACCACTATGTTAATATAGAGGTGGTTTCAAAATAAATATTTTATCGATATAGGATACTCATATTG
ATAAAATGATTTGAACC
>TCONS_00075078

CTCCAATATCGACCTCTTCAACGGTTGGCCTTGGCAACCCTCTCAATCTCATCCTTCTTTTTAATGGCATAGCT
GTTCGAAGAACCCTTGGCAGCATTGACCGGTTCATCTGCAAGGCACTCAGCTATGGTCTTGATGTTCCTGAAA
GCACTCTCACGTGCACCAGTTGTCAGCAAATAAATTGCCTGGTTAACACGGCGAAGTGGAGAAATATCAACA
GCCTGACGCCTCACAACACCAGCCGAACCAATACGGGTTGCA
>TCONS_00075090
ATGAACTAACATAGTCTTCTCAGATGAACAAGGGGAATAACCCCGACGTGTCTAGGATATCTATTCCCCAGG
TGCCGAAACGCCTTGCTTCAGTCTTTCCCTCTTCATCTTCTTCTTGGAGACTGGCTTCTTTTTCTTTGGAGGAAG
GGTTTTCTGTGCATACATGTAAAGCACATAGTTCCCAATAAGGAATGCCAACACGAGTCCGCCAACAACAGT
TAAGACAATTAATCCTGGGTTGAGTCCTTTCACTTCAACATCCTTGGCCATATTCTTCACGTTATCAAAAGACG
GAGGAGGATCAAAATCAGCTTCGGAATCCATGCCTTGAAACGCCGATCGTC
>TCONS_00075209
CTCGGTGCAACTGTAGACCTGAGGCCTGAGGGTCTTTAGAGGAGCATCTTTATCAATGGAATATCTCCAGTGC
AGCTGGACTAATGGGGATCAGACCAGAAGGTGATACAAGTGCATCAACAAGAACATCATTTGGAGTTAGTG
GTATAGTGCCCTCATCCAATATCTGCACTGAGTAAGAAAGCGCAATCTTGAGAGGTTGCTTCCAATTTCGCTC
CTTTGCACGTTCCTGATATCTTGTCAAGAAGGTATCGTAATAACTG
>TCONS_00075246
CTATGCTCCAGTCATAGCAGAGCAGCACTATGTTTGTTAATCCTCCTTGTTACGAGCTAGTCTGGCCCGGTGTC
TAAGCTCGGCTTTTTTCCACCTCACAATCAGGTAACATAGTGAAAAGAGGGTATTAACCAAGATTATTATCGC
AATAACCAACAGAGGCCCAGACCAGAGTGTGGAAAGAAAAATGCCGTGTGAATCGAAGTAATTCTGGCATG
CAAAGCTTTTCCAGTTTGCAGCTAATAA
>TCONS_00075427
CCAGGCCGCATTCTCCACTGATTCAGCTGAAGCCGGTGTTAGTTCCCCAATAACTACTACTACTACTACTAAC
ATCTCTATGCTTCCCACAACTTCTACCCTCAACATGGCTTCTCTTAAATCTTGTTACTTCCACATGCCAAGGTTT
ACTTCTGGGCAAGAAGCCATTGAAATGTAGGTGTTAAGAGTTTGTGCAAGGCTAGCTAGGAGGGATTAACTT
AGCTTGAAAAGTTTGAGTAGTACCATATACAAGAAGTAAAAGTCCTCTAGCTGTATAGCCAAAGTATACCAA
CAACAGAAGTAAAGATTGGTAAGGAAGATCTCCTCAAGATTGTTGTCATCATCAATACCATACTACTTACAA
CAAATACATAATGTATACGTAAAGGACAGATGAACTGATTAATGAGAGTGGGGTCTCTCTCTGTGTTTATTTT
GTACCCTCTTTTGTAGTTGTGTGCTGGACCACACTTTGAACAATAGCAAAAAATCAAAGGCAGGCTAGTAGTA
ACCCTTAGTTATGATAGTAGTTTCCTCTCTTTCCTCTGTTTTTTTACTGTTGTTAACTTGCTATCATGTAAAAATT
AAAGTTGAAATATGAAAAATGCCTTCTAGATCATCACTTTCTGCTAAATGGGGTTCTAGATTTAGCTTCCAAT
ATTTTCAAGAATGAG
>TCONS_00075428
CCAGGCCGCATTCTCCACTGATTCAGCTGAAGCCGGTGTTAGTTCCCCAATAACTACTACTACTACTACTAAC
ATCTCTATGCTTCCCACAACTTCTACCCTCAACATGGCTTCTCTTAAATCTTGTTACTTCCACATGCCAAGCCA
TTGAAATGTAGGTGTTAAGAGTTTGTGCAAGGCTAGCTAGGAGGGATTAACTTAGCTTGAAAAGTTTGAGTAG
TACCATATACAAGAAGTAAAAGTCCTCTAGCTGTATAGCCAAAGTATACCAACAACAGAAGTAAAGATTGGT
AAGGAAGATCTCCTCAAGATTGTTGTCATCATCAATACCATACTACTTACAACAAATACATAATGTATACGTA
AAGGACAGATGAACTGATTAATGAGAGTGGGGTCTCTCTCTGTGTTTATTTTGTACCCTCTTTTGTAGTTGTGT
GCTGGACCACACTTTGAACAATAGCAAAAAATCAAAGGCAGGCTAGTAGTAACCCTTAGTTATGATAGTAGT
TTCCTCTCTTTCCTCTGTTTTTTTACTGTTGTTAACTTGCTATCATGTAAAAATTAAAGTTGAAATATGAAAAAT
GCCTTCTAGATCATCACTTTCTGCTAAATGGGGTTCTAGATTTAGCTTCCAATATTTTCAAGAATGAG
>TCONS_00075429

CCAGGCCGCATTCTCCACTGATTCAGCTGAAGCCGGTGTTAGTTCCCCAATAACTACTACTACTACTACTAAC
ATCTCTATGCTTCCCACAACTTCTACCCTCAACATGGCTTCTCTTAAATCTTGTTACTTCCACATGCCAAGAAA
AGTTTGAGTAGTACCATATACAAGAAGTAAAAGTCCTCTAGCTGTATAGCCAAAGTATACCAACAACAGAAG
TAAAGATTGGTAAGGAAGATCTCCTCAAGATTGTTGTCATCATCAATACCATACTACTTACAACAAATACATA
ATGTATACGTAAAGGACAGATGAACTGATTAATGAGAGTGGGGTCTCTCTCTGTGTTTATTTTGTACCCTCTTT
TGTAGTTGTGTGCTGGACCACACTTTGAACAATAGCAAAAAATCAAAGGCAGGCTAGTAGTAACCCTTAGTT
ATGATAGTAGTTTCCTCTCTTTCCTCTGTTTTTTTACTGTTGTTAACTTGCTATCATGTAAAAATTAAAGTTGAA
ATATGAAAAATGCCTTCTAGATCATCACTTTCTGCTAAATGGGGTTCTAGATTTAGCTTCCAATATTTTCAAGA
ATGAG
>TCONS_00075434
CCCCACCAAGAAAAACATCATTCACTGAACAACTCCTCCTATGAAGATGAGCATGAGGGGATTCCTCACAAT
TCCCATAAGGGAAAACAAGTGAATCACTTCTACCACTAGGACTCATAATTGGATCCAATGATGACTCGTCAA
GAAATGACAAGACTTGATGATCTTGAACTTGAACACTAGTATTACTACCAAACTCATCATTAAGCTCATTGTT
ATTATTGTAAAAAGGTAGTGATAGTGAAGTAGTTGAAGACCCAGAAACAGAATTAGTTGAACCATTGACAAC
AGCAGCATAAGATAGTGAACTAGAGCTAGCCATTATTGGTCTAGGGCTTCTTGAAAACACAGGTGACCCACT
AGACCAAGGTGAATTAGGTGAAGAATGAACAAACCCATTATTAGGAATCATGATTCTTGGTGAAGATTGAGG
AAAAGGGTTCAACTTTGTAGCATTTGACATGGGGTTAAAAGGTGAAGGAGAGTTAGGAGTTGAAGTTGCAGA
TGAAGTGTTTGATGAAAGTCCTAAACAAGCTTTAGCTTGGTTAATTAGTGAAACCAAATGGGTTTCTGGACCA
AAAGCTAATCTTATCATCTCCTTTTCTCCTTGGTCTTGTATCAAAATATAACCCATGATTTTTGAAGCATTTTCT
GGATCCAAACTTTGAATCCTTGATAAAACTATCTTTGTTGCTTCATATGCATCCATGGCAAAAACATTCACTAC
TACTAAACTACTTCATACTACTACAAACCCCTTTGGTTTTTC
>TCONS_00075435
CTTAAACATGTTGACAGCTCTCCTCTATCCATCGAATGCTGTTGTTGCAGTTGCTTCTTGTCTGCAACTTTTCCC
TTTTCCTTGTAAGGCTTGACAAGTACACGAGAATCACACACAAAATGAGGGTTCCCTTTAGCCAAGATGAGCT
TCACGGTTTCTGGGTAGACAAATGTAACGAATCCAAACATACGCTTCTGCTGATACGGAATTCTAACATCTTG
GACTGGCCCATACATGCTAAAGTAATTAGACACATCCTCCTCCTTAAATGTGCTATCAGCAGGAAATGTTAGG
TAAATCTGTCGCGAACTTGAATTTGAGATACCGCCCATCGCCATTGCTGAAAAATCACTTCTATCAGGCCGGC
ATCGACCAAATTTGTGGAATTCTTCTCCCATCATAAACGCTGCCGCAGCCGATCTTTGGTTATCACTCAAGAT
GTTCATACACTTGTTGTAAGCAAGTGGATGATGATGAGCACCAGAAGCCATAAGGGAGGCAGCAGCAAACC
TCTGTTGTTGCTGCTGTTGTAAGGCTTTCATCCTCAGAAAATCATCAAAAGAGTCAACTTTATTATTACTAGGA
GAACCAACAATAGCACTAGACCCATCAGAATCTTGAAATCCACCAACACTATGCAAAAACTTACAACTATTC
CCATTCTTGCAAAACCCTCTTGCATAATACATGCAAGGCCTCCAACC
>TCONS_00075436
TAGCAAGTTAACAACAGTAAAAAAACAGAGGAAAGAGAGGAAACTACTATCATAACTAAGGGTTACTACTA
GCCTGCCTTTGATTTTTTGCTATTGTTCAAAGTGTGGTCCAGCACACAACTACAAAAGAGGGTACAAAATAAA
CACAGAGAGAGACCCCACTCTCATTAATCAGTTCATCTGTCCTTTACGTATACATTATGTATTTGTTGTAAGTA
GTATGGTATTGATGATGACAACAATCTTGAGGAGATCTTCCTTACCAATCTTTACTTCTGTTGTTGGTATACTTT
GGCTATACAGCTAGAGGACTTTTACTTCTTGTATATGGTACTACTCAAACTTTTCTTGGCATGTGGAAGTAACA
AGATTTAAGAGAAGCCATGTTGAGGGTAGAAGTTGTGGGAAGCATAGAGATGTTAGTAGTAGTAGTAGTAGT
TATTGGGGAACTAACACCGGCTTCAGCTGAATCAGTGGAGAATGCGGCCT
>TCONS_00075437

TAGCAAGTTAACAACAGTAAAAAAACAGAGGAAAGAGAGGAAACTACTATCATAACTAAGGGTTACTACTA
GCCTGCCTTTGATTTTTTGCTATTGTTCAAAGTGTGGTCCAGCACACAACTACAAAAGAGGGTACAAAATAAA
CACAGAGAGAGACCCCACTCTCATTAATCAGTTCATCTGTCCTTTACGTATACATTATGTATTTGTTGTAAGTA
GTATGGTATTGATGATGACAACAATCTTGAGGAGATCTTCCTTACCAATCTTTACTTCTGTTGTTGGTATACTTT
GGCTATACAGCTAGAGGACTTTTACTTCTTGTATATGGTACTACTCAAACTTTTCAAGCTAAGTTAATCCCTCC
TAGCTAGCCTTGCACAAACTCTTAACACCTACATTTCAATGGCTTCTTGCCCAGAAGTAAACCTTGGCATGTG
GAAGTAACAAGATTTAAGAGAAGCCATGTTGAGGGTAGAAGTTGTGGGAAGCATAGAGATGTTAGTAGTAG
TAGTAGTAGTTATTGGGGAACTAACACCGGCTTCAGCTGAATCAGTGGAGAATGCGGCCT
>TCONS_00075524
GGGGCAGCTAGGTTGAATCATTATCTGGGTCTTCTTCAGTATCACTCTGTGATTGAGGACCCCAAAATTTTGAT
TCCAGGATGCTTGAATCAGAAGAGATAGGAATCATCGCGTTGTTGAAGTCCTGAAAATACAAGGCACTATGG
TCTTCGTCATTACACGCTGTAACTATTCTACATCCATCGACAGCCATTGCAGAACACCCAAAGCCTGGAGGGG
AATCACATTGAAGACAACTAATCAAAGAACATGTTCGTCTACCATTCCCTGTCTCCCAAACATGTACTTCGAT
ATCCTCCAGCCCACCACTAACAATTTTGTAGGGATCCATGTACAAGAGATTTACATTGCCTTTATGTCCATCTA
ACTCCGCTACTGCTTCTCCCTTCTGTAGGTCACCGCCTCTTCTGACATCCCAGAGCATTGCTCTTTGAGCTAAC
CCTGTGCAGACCAATGATTTCTCTGGCAACATTTGAAATGAATGTAATTCTTCTTGATGGTTCACTTTAAAGAC
TTGACGCATTGTTCTTATATCAATTGCAACAACCGAGGAACCAGCCGCAACATAGATTAGTGATTCATGACAC
TTCATTGCCTTAGGAGCGCCAGGCACAGTGCTCTTTCCCACACAGCATGAAGAGCGAACAGCAGATGAAGTA
GAAGTGTCCCAAACCATCACCTTGGAGTTCCTTGATATGCTCACTAGAAGAGATGTCTTGTGGCCGGCAACTG
ACATCAGCACTACAGGTTTCTCATGCCCGTAAAGTGTTGCCTTTAAAGCTTGTTGGCCGCGCTTACCACTAGA
ATCAAGGGACCATAATCGAACAGTTCCATCTAAACCACCGCTTGCAAGTATATTACTAGGACAATCACCCAA
CAATTTGTCTGAAAGTGTGGAAACTGGACCATTATGGCCTCTGAAACATCGCTGACAACGCCCCTTCCACCAT
AGCCGAATTGAATGGTCAAAGCTTGAGGTTACCAAAAGATTGTCATTTTTCTGCATCTTGCTACTATACAGGC
ATGTCTCTTCAAATGGAATTAATCTGCAGTTAGTAGGATTATGGCTATTTCTA
>TCONS_00075525
GGGGCAGCTAGGTTGAATCATTATCTGGGTCTTCTTCAGTATCACTCTGTGATTGAGGACCCCAAAATTTTGAT
TCCAGGATGCTTGAATCAGAAGAGATAGGAATCATCGCGTTGTTGAAGTCCTGAAAATACAAGGCACTATGG
TCTTCGTCATTACACGCTGTAACTATTCTACATCCATCGACAGCCATTGCAGAACACCCAAAGCCTGGAGGGG
AATCACATTGAAGACAACTAATCAAAGAACATGTTCGTCTACCATTCCCTGTCTCCCAAACATGTACTTCGAT
ATCCTCCAGCCCACCACTAACAATTTTGTAGGGATCCATGTACAAGAGATTTACATTGCCTTTATGTCCATCTA
ACTCCGCTACTGCTTCTCCCTTCTGTAGGTCACCGCCTCTTCTGACATCCCAGAGCATTGCTCTTTGAGCTAAC
CCTGTGCAGACCAATGATTTCTCTGGCAACATTTGAAATGAATGTAATTCTTCTTGATGGTTCACTTTAAAGAC
TTGACGCATTGTTCTTATATCAATTGCAACAACCGAGGAACCAGCCGCAACATAGATTAGTGATTCATGACAC
TTCATTGCCTTAGGAGCGCCAGGCACAGTGCTCTTTCCCACACAGCATGAAGAGCGAACAGCAGATGAAGTA
GAAGTGTCCCAAACCATCACCTTGGAGTTCCTTGATATGCTCACTAGAAGAGATGTCTTGTGGCCGGCAACTG
ACATCAGCACTACAGGTTTCTCATGCCCGTAAAGTGTTGCCTTTAAAGCTTGTTGGCCGCGCTTACCACTAGA
ATCAAGGGACCATAATCGAACAGTTCCATCTAAACCACCGCTTGCAAGTATATTACTAGGACAATCACCCAA
CAATTTGTCTGAAAGTGTGGAAACTGGACCATTATGGCCTCTGAAACATCGCTGACAACGCCCCTAAAAATG
AATCCCACAGGCAAAACTTCCACCATAGCCGAATTGAATGGTCAAAGCTTGAGGTTACCAAAAGATTGTCAT
TTTTCTGCATCTTGCTACTATACAGGCATGTCTCTTCAAATGGAATTAATCTCATAGAAGTA
>TCONS_00075573

GCCACCTCAGTCTTTGTCCATCTCTTTCACCCGCTTCAGTAGTAGATTGTGTGAAGACAGAAACCCAGAAAAA
GCTCAAGAAAATCTAGAGCTGCTACCACCCCAATGGATTTGTTTAAATTTCTTACCAGAATTTCTGCTTCTACC
ATACTTTTACTCTTCTTGCTTTCTTGCTTCAGCTTCTCTTCAACAGCTTCTGCATTATCTGAAATACAAGATTGA
AGGAAATCAGTATAGTTGAGTCCCATGTCTGAACCAGTGACTCTGTACTTCGATCGTTTGTCTGTTGACTATGA
>TCONS_00075680
TTCATATTACTGTGCTCTGGCAACAGGCTCCAGTGCTTCAATTGTCACCACGCTATTACCTCGGATTACCACCA
TGCCGATCTCATTCTTCTCATTGCCATTAACCTCAACAGTGTTGTCAATGACCAAATTCATGAACTGATCAAAC
CCACGAAGAGTCCCCACAACTAGGCGGTTGGCATTCAACTTGATTTGGAGCTGCTTGTCCATATACTTTTTAA
GATCCGGAGGCTGACCCGATCTGCTCAT
>TCONS_00075724
GAGAAAAGGAGCTTCACAAGATTGAAGAGAACCCTCCTTTCACCACCTTTCGAGCACTTGAAATGCCATCTC
AAGTATAAGCAAAAATTCTATCTGTAATTTTAGTCCGACAGATTGGACACTCGGCACAGGCAAGGGAACAAG
ATTTACACACTGCAAATTAAGTCGACATTAGACATCTCCTACCAAGGGAGCGAGGATAAGCTGAGCACATAA
TCAGAAAGTTGACTTACAGCAAAAATGTCGGCAAGGAAGGAGCATCGCAGAACTTGGTGATTCGAAACAGA
CTTTACATACATGTGAATTTGCATCAACATTTCCCAAATATCTGTGCTCCTTTTCTTTCATCTCTTGCATTCTAG
CCTGGTGGCGCACAGGTAAAAGATCTTTAATATTCCATGC
>TCONS_00075726
ATCTCAACCTTGGAGTACTTTGACAATCTCCCCCGCTCTTGCCTCAATACCTTTCAGATACTCAATCAGAACTC
GCTGCTGGTATGAACCTCCCTCCGTACGTATCAAAGTCCTTTCCATTCTCTTCTTAATAACAGCATACAATTCC
TCAGGTGAAATATCACCTTTTCCAATGGTCATGCCATCTATCAAGAGCTTGTTCCATTCTTCCTCTGGTGCTTTG
ATAATGCTCTCAAGAAACTGTTCAGCTTTTAACACTTCATTTCCTTTTTTTGCATAACTCCGCTTTGACAGCACT
CTGGAAGCATACAATTGCAGAACTTTTTGTAACATAGCTTCAAGTCCTGGCTTGTCCCCGTCTTTTTTAGCTTG
CCGCAGCTGTGCATTGACTTCTGAGAGGAACCCTTCATCAAGTTGGCCTTCTTGCTCCCTTTGATTTATTTCTTT
CTCCATAAGACTGAGAGCCTCAGGATCCCTAACAGGCCAAGAAATTTCTTCTACTTCATCAAGCACAGGTTCT
AGGATTGCTTTGAGCACATCAGTAGACGAGTCTATCTTTTCTTTTGTCTTGTGAACAAGACGATCTACGATGTT
CATTACCGACAACGCCAGCTCCTCGTAGTCTTTTTTGTCATCGTCAGACTTGCAGGTGTCTGCTCTAGCTGCAA
GTCTTATCCAAAAGCTCTCGTTGAAAGCGAGAACGTTCTCCACAACTAGCTGTGGCAGTTCCTTATCACTTGC
CTCCCTCAGTGTATCTATAAGCTTATCCACCTCCACAGTCTTTTTGAAAGATTCCTCAGCATCTTTACTGACTG
C
>TCONS_00075727
ATCTCAACCTTGGAGTACTTTGACAATCTCCCCCGCTCTTGCCTCAATACCTTTCAGATACTCAATCAGAACTC
GCTGCTGGTATGAACCTCCCTCCGTACGTATCAAAGTCCTTTCCATTCTCTTCTTAATAACAGCATACAATTCC
TCAGGTGAAATATCACCTTTTCCAATGGTCATGCCATCTATCAAGAGCTTGTTCCATTCTTCCTCTGGTGCTTTG
ATAATGCTCTCAAGAAACTGTTCAGCTTTTAACACTTCATTTCCTTTTTTTGCATAACTCCGCTTTGACAGCACT
CTGGAAGCATACAATTGCAGAACTTTTTGTAACATAGCTTCAAGTCCTGGCTTGTCCCCGTCTTTTTTAGCTTG
CCGCAGCTGTGCATTGACTTCTGAGAGGAACCCTTCATCAAGTTGGCCTTCTTGCTCCCTTTGATTTATTTCCTG
AATGTTAAAGTAAATTTCTCCATAAGACTGAGAGCCTCAGGATCCCTAACAGGCCAAGAAATTTCTTCTACTT
CATCAAGCACAGGTTCTAGGATTGCTTTGAGCACATCAGTAGACGAGTCTATCTTTTCTTTTGTCTTGTGAACA
AGACGATCTACGATGTTCATTACCGACAACGCCAGCTCCTCGTAGTCTTTTTTGTCATCGTCAGACTTGCAGGT
GTCTGCTCTAGCTGCAAGTCTTATCCAAAAGCTCTCGTTGAAAGCGAGAACGTTCTCCACAACTAGCTGTGGC
AGTTCCTTATCACTTGCCTCCCTCAGTGTATCTATAAGCTTATCCACCTCCACAGTCTTTTTGAAAGATTCCTCA
GCATCTTTACTGACTGC

>TCONS_00075778
GTTCTGGAGATTTTCTCTTGGTTCAGTAAGGTCGAAATTTTCTTGCAGCCCTCAACTGCTGTATCCGCTTCTTAT
CCTGTTCAAATTTGCTCTGCAGCACCATGATCTCATTTCTTTTTGCTTCTCTTTTCTGAAATCGGTAGAAATCTA
ATCCCACATCGTTCTTTTTCTTTTTGGCCATATTATCCATAACAGCAGTCTGAGAAACAGAACCAACTGCAATT
CCAGTTTCAGAATCTGTTGTCTTCTTCCTGCCTTTGTGGTGTACAACAACGGTCCATCCACCTTCAGCAGCACG
GGCTTCTTTTTCTTTCCTTTCCTGTTCTTTTTTCGCCTCATGATCAGTTATAAACTCATCAATCCTTTCTTGCAAC
ACCTTTATCCCTGGCCTACTCTGATAATAATCTGTAACCCACTTTCTCACTCCTTTAGAGTAATCCTCGTCCCCT
GAAGATAGCTCATAAATCTCTTCATCTTCAGCTTGTTCTGTCTTCTCTGACAAATCATCCTCCGCTATTGAAGA
TGGACGATCCTTC
>TCONS_00075787
AGAAAAGAAAAGGAAGAAGAAGAAACTGGCACTGCAAAACAGAAGAAGCAAGGATGAAATAGATGAATG
CCATCTATCATATGATAAAGATGGGAAAACGAAAAAGAGCAAATCATCAAAGCAGAAGAAGAGGAAAAAT
GACACACTTGCTGAAAGTGGTGTAGTGGATCAATCTAATGACGTAGTGGATAGGCTTGGTCAAGAAAATACC
ATATCAAAT
>TCONS_00075848
TCCTGCTCACTGTAATTAAACGGCGGAATATTGCCAACAAATTCACTTTACAATGGAAGGCTAGCTTAACCTC
TTGACCCAATCACTGCTGGTTCCATTTTGATCACCCTTAGTTTTCTATATTCTGTAAAAGCTCCAAGTCCCCAA
CAAAACTTAGGTGCGTCAATAAATTGCCAGAATAAGAAGCAAAAGAATCGGTCTGAACTACATTTCTTAGTT
AGGTAGCCATCTCATCAACTTCAGAATGAAATACAAAACTTGGAAAAGTAGTAGTTATATCCCTCAGATATG
AAAGGGTCATTATTTTCAATAATGAAGGATTTCGGCCCACAAAATTGTGCCACTCAGAGTTGTCAATCTTTCC
GTCCTGATTTGAATCTGCCTCCAAAAATGTCTTATCCAGTATTATCTCAACGATCTCGTCGGCCAACTTCATCT
CAGACTCGCATAAAATTGCAATTAACATTTGCTTAACCTCTTGCCGCTCAATGAACCCCGTGCCATCCAAATC
ATAAAGCCTAAATGAAAAATTGATCTTCTCTTCTTGTGAGGCATTTGGGTGGAAAACATTAAGTGCCCTTACA
AAATCACCAAAATCGATAACTCCTTTTTGCTTTACATCAAAGAGATCAAAAATCCGATTTGCAAAAAGGTTTT
CTTTCTTTCTGTTCTTGAATAAAGCCAACTGAAATTCTTCCTTGCTAATTAGCCCATCATCAATTACCGAACCG
CTAATATTCTTGAACAGCTCAAACAATGCTTCAACTTCGCTTACACTAAAGGCTGTTTGAGATGCAAGGACTA
CAGGATCTTCGTGTCCAGGGAACTGCTTTTTTGCTGTAGACTGGAAACAGCCCATCTACTAAAGAAGGACGA
CAAACGAGGAAGAACACGACTAGACTAGTACCGAGCAACCAGCTTCTCTACAATATATCGTTCAAACCATCT
TATCCATGCAATTACA
>TCONS_00075855
CGTATTGAATGTATCTCCTTTGTCCCATACGCTTCTATTCTGAATGTTCTTTTAATTGTGAACCTACAGGGAGCC
ATTAACTTACTCATTGATGTCTACAAGAAGGAATTTGTCAATATGGGTGGCTATCTTACTAATTCTTGTTAGGT
TAATTTGGAGAGGGCGGGGCACTTTATACAAGCCATTGGTTCTTATGAGAATGCTGTCTTTAGGAAGCGTATC
CAGTTTACTAGAAGCAAATTTTTGAAGACATACAGATTAACCATCATTTTACTGGGAGTTTCAAGAAAATTTT
CATTACAATCGAGGGATATTAGTGATATGTAATTTTTTGCTAAATTTTTATGAACACAATATTGTGATTAGCTT
ACATTATTGATGTA
>TCONS_00075984
GAACCACCTTTTTATCTTGTAAATCCTTATCAAGATGTGCTAATCGTAGGTTTATATGATTCATTAAATTCAAT
GCGACATCATCACTGAGCGGCCCACTTGGAACAGCACCTAAGTTATGTCTGCCAAAAAGCTTCCTGATAGTG
ACGTCTCTTTCATTCTTTAACACCGCAAGAGCTTCAGCCTCAGCTTGAAGCTTGCTGATCTCCACGATATAGTC
TGTCATGGACTGCTTAAGAAGGGAACTCTTTGTCTCTGAGTCATTCATTTCCCTCTCCAACTTGCTAATTTTCGA
TTCCAGAAGCGCTATCCTTTCATCAAACTTTGTTTTCCATTCACTCAGCTCTTCATCAGTGTCTTCATTCTCCTCT

GCAAGTGCTGCATACTGTTTTTCCTGTTCCTTGAACAATGTTTTCCTTTCAGCAGTTTTGGTGGCTATCTCTCGC
TGCAATTGATGAAGGTCCTTTAAAGTTGCTTCAGTATGGTGGGTCTTACTCTCTAGAGTCCGAATCTCTTTTTC
AAGCTCCTCCATCTGACTTTTCAGTGACTCGGTTTTCTCTTTATCTT
>TCONS_00076237
ACACACTTGAGTAGCACCAGTTATTGCGGTAGTGCTGCCTGGAAGAGCTCGCTTCTCCTTGCTTTCTCTATACA
TCTTGAATCAAAGGTGAATATAAAGAATGAAACAACAGCTTAGGAGGATCTGCAATTAAATCTGAAGTGCA
GCTGCTATGGGAGCGGCGAGGCTCAGAACCTCAAATGGCTTTCTGTCTTTCACCTCCTTCACTGTTCGAGGGC
ATGAATCTGTGATCCAGAAGTAAGTCATCCCACTCTCCGGACTACCTCCTTTGTCGTGGTTAAAACGCTGCCA
TGATCTGTTTGGAAAAATTCCGTGGGTCACATAAGCACTTATTTTTTGAGCTCCATGTCTAGCCAACACTTTCT
GGCATTCTGTTAGGGTGCCACCTGATTGTACAAGATCATCCACTATCACCACATGACGTCCGGCAGGATCTCC
CTCCTTAATCCGTACAATTCGTTGATCACCTTCCCTAACTTTAGCACAGACAATCATTGGAAAATGCTGAAGC
TGCTTGTGAAAACGTTTCCATGCACCATCATCAGGAAAAGCTATAGATATATTGTCAGAGTCAGGGAGTTGTT
GTAGCCTATTCAGAAGCAACGGGATCCCGCTCTCGAAGCATGGTAGTATATTGTCTCCAAAGTAGAACCTCTC
CTGCAAGGCATGGATATCAAAGGTAACTAAACTAGTTGGTCCTCCTCGAGGAACTGGGATGTTTGACAAAAT
CCTGGCAAGCGTAAAAGCTGTGGCAACATCACCTTCATCCTCCATACGCTCAGATGTGCCGGTTGGGAAGAA
CGGAAGGACAAGTGTGAATGAGGAAACGAAGAGCTTGGGCAATGCATAAATAACTGATAATTGCTCAAAAA
TCACTCCAGGAGAACTAAATGAAGCCAGGAAAGCTGCATGCTGTCCACGGATGCCATGAGCATTAGATATGA
ATAGGTTTGGGAATCCATCTTCAAAAGTCCTCCAAGTGATACTACGGAGCTCAATGGCATCAGATTGAGAAG
CAATTCGCTCAGCAAGATCTTTCATCTCATCGCAATAGAATAAGCAAACTCTTTTCGAATGCTTACCAGCAGG
ACCCAAAGCAGCAGCTGCAGACATTGGGATTGAAGGAGGTTTCAGAATAATATGAATTGGATCAGTCGCTGA
TACACAACCCACCGTCCACCTTTGGTTACTCTCAAATTCACATCGAATGGAAAAATCCTTATTACGCTTGAAG
GATCGCGAGAGGATAAAAGTGGAATTATGGAATTTGTCAAGAGTTTTTGAGCAATGTGAAGAGAAGACGGG
CGGCAACGGCGACGGCGGAGTTGTGGCTGACATGGGGTGGAAGTGTG
>TCONS_00076257
TGATATCAGCAACATGCCTTGAACCTTGACAATACAGGGAGCTAGTTACCATGACCCCACCGCACCAGCAGT
TGGATTTTGGAAAGCAGAGAAATTCCAGAAAGTCTTTAATGAGTATGGCTGAGAAATTGTCACTCCCTCTTCC
GTGATTTTTGCAATCTGAAGCTTGTTGACTGCAGATCTATCCCTATACAGAAGAACACGCATACATTTCTCCA
ATAACTTAACACCTTCTTCATAGCTCAAGTCTTCGCGCCATTCATCACGGATAATGGGCTTTGCAAGGTGATTT
CCAAATCCAGTTGCCGCGTGATTGTCCTCATAATGGACACCAATCATACTAACCGTTCCAAGATACTTCTGCC
CATTTTTCACCCCACCAAGTACAAGGGAATTCCATAGTGGGTTGAACTTGTTCCTTCGATTATACATCATGCG
AGTTAGATAATTATGCACTTCTTTGGGACCCAAGGAGTTTCCATCATCCACATGTTGTCATACAAGATAAGCT
CGTCAAGGTTCCGCAATATCTCCTGAAAATCACTAATTTCACCACTTGCGCCAAGAAGGGAGTGTTTTCCCAC
TGACTTCAATCGCTCCACACTCTTGTAACGCAACGTTGAACCATAGGAACCTCCCATATCAGCAGCCAACAG
AACACCATCCTTGTACTTGATGCCGATAACTGATGTCCCCGTCACATATGGATACTGTGTTCTCTGAATATCAG
CTTCAGGGCTCAATAAACTACACTTTGCCGGTGCAGATTCCATCAACTGCGAAAAT
>TCONS_00076266
CTTATTTTGTCCTTTATTGTATTGTCCTAAATTTTGCAATAAAAAAAGTTTTCGCGGGCGAATATTGACTCTTTC
AATCCCTATTTCATTTGTAGGGTTAACTCGTGACTTCTCAGATCTCCGAATACATGAATTAATCTCTGGTTCGTT
CCGCCATCCCGACCAGTGAATCATTAAGATTCCTTTTTCAATAGAATCTTTTGCATTCACAAGTTCCGTCGTTC
CCATCACTTCTTACTTAATGGTTAGGTCCGAATTCTACAATGGAGCTCAGAATGAAATTGGTTCTTGAGTCAAT
CTTCTCAGTCTTTATTGGCTCGAAGCTCTTGATTTTTTGTTCTATTTCTATAAGAAGATTCATTTTATTATGGTAT
GAATGCGTATTGATGCTTTATTACACTGCCTTTTATGAGATTACTCATAGACCTTACATATTGGAATTTTATATC

ATTGGTATTCTTTTTCTCTCTTTCTCTCATCCTTCCATTTATCCACATCTTTTTTGTCTATTTTGCTTTACAACTTA
GAATCAGATTTCCTTTTTTGTTTATGCAAAAGATTTCAGTTGCTACAAAGATATGACCTATATATCATATCTTG
ACTGGTTCTTTAGATCCAGATAATGCGAAGTGATGGGTTGGTTATTAGTTCTATAGTTTTTAGTTCATACTATGT
GGGCTGGTCTTTTTTAATCCTAACCCTAAAAAACCAACGAGTCACACACTAAGCATAGCAATTATATCAAATG
GTCAATCAAATTTTTATTCAACCTTATAGAATTAAGAATTATAAATGTTCCCCTTGATTGATTAGAAAAAGAA
TGAATTTTTCTTTTTTTGTTCAATCATTGGATAGAAGGGAAAGACAAGTAGTAAAATTATTCCTCGTCTAGAAA
TATCCAAATTTTGATGCCCAATACTCCATAGATAGTTCGAACTGTATAAGAGCAATAATCAATTTTCGCTCGA
ATCGTTTGTAGGGGAACCCTACCTTCTCTGATCCATTCGACATGTGCAATTTCTTTT
>TCONS_00076375
AAATACAAATACAAATACTATAGTCATGAGAAATAAGTGGAACAAGAAAAAGTAAAAGGGGTTGATTTTTT
TGGATTTTAGGAAGCTTCTTTGCAAGTATAAAAAATCAAAGGGTGTAAGTGTTCTCATTCCTTTTTCTTGAATT
CCATAAGATTTGATTAGTTTCAATTCTCTTGGCATCTTCTTTAAATTTTATTGCTGGATTTGTACCTACTAAAGA
TATATTTATTGGAAAGATACCATAAAAAAGGATTGCTGCTGTTGATTTTTCTCTCTACTGTGTACTGA
>TCONS_00076396
CTATATCTTGTAGGATCGCTAAGAGGCTCCCCCTATCCTGGAAGAAGTTTAGAATTCGGATCCATCGGAGTGT
TAACAGGTCTACAACCTGTCATTCCTGTCTCCTCAAGAATATCTAAGGCATACTTCCGTTGTGAGATCACAAT
ACCTGAGCTAGACTGAGCGACCTTAATACCCAGAAAATACTTTAGTTTGCCCAAATCCTTAGTCTGAAAGTGT
TGAAATAGATGTTGCTTCAATT
>TCONS_00076397
TATCACTGTAATCGAGCCCAAATATTTGAGTATATCCCTTGGCAACAAGACGAGCCTTAAGACGATCAACCTT
ACCATCCGGACCAACCTTGACTTCATACACCTAACGACAACCAACAGTAGATTTACCTGAAGGAAGGGGGA
CGAGCTCCCAAGTACCACTCGTATGTAAAGCAGACATCTCGTCAATCATAGTCTGTCGCCATCCTGAATGAGA
CAATGCTTCACTTGTAGACTTAGGGATGGAAACAGAGGACAAAGAAGATAGAAAAGCATAATAGGGTGATG
ACAAACAATGATAACTTAAACCGACATAATGGGGATTAGGATTAAGGGTGGTCCGTATACCTTTCCAAAGTG
CAATCGGTGTACTAGGAAGAGACAAGTCCGCAGTATGAGCAGGGTCAGGTGCATGACGTGAATCAGCTGGG
CCTGATGCTGGGTGCGG
>TCONS_00076408
ATGGAGAAATTGTCTCCTATCCTCCTGTCATGTCTAACTTGGAAACCAACCTCTTTGCCAAGACTAAAACCAA
GCTCAAATGCCACTCGTTTGGCAGTTGCAAGTACTGCAACACGGCGTGGTTGAGTCACACCAATAATACCAC
CACGACCATTGGAATGATTTGAACCATAGCCAGCTTCATAAAGGAACTGAGGAACTTGGGTTGTCTTACCAC
AACCTGTCTCACCACAAACTATTACACACGTGTTCTCATTTATAGCTTCCATTATCTCCTGTTCCATCATGACT
ATCGGCAGATCGCTCCTCTTATTTTCAACCTCCTTTGGTCTTGAAACGTGCACCACAGTTGGAGTTGCTAAAGC
TCTTTGAGGAAGA
>TCONS_00076413
TGACTTTAAACATTCTGTAGTTCTATCCTCTTCTTGCTTATTTTGCACCGAAGCATCTCCTCCGCAAACTAAAA
GTCCACTCTGAGAAGTCAACTCTTCAGGAGACCCTGGAGTGACCAGTGCTTCACTTTGTACTTCACTTTTTCCT
AAGGAGGACTGCAAAAGATTGCTATTGACTATTGGACTCGACTTCATTTTGTTAGAATCATCCAAGACATCAC
TAGTTAGGCTGTTGGTTGTCCTCTTCTTGACTGGTCGATCTCTATGTGGCATTTCTAACCCCGCCCTAGAAAAC
TGCACTTCTCTCATACGCTTCTCTCGGCTAGTTTCACCCTGACCCAAGTTCCTTGAAGACCACATAAGTGAATA
AAGATCATCTTGGATCTGATACTTCCTGAAATGGGAATTCAAGGTCTTAATGCTTTCAGCTAAAAGGATTGAT
TTCTCCTTGTATTCCTCGAGTTTCTTCAGCTTTTTTTTCTGAGATTGACTTAACTTAATGTTATTCTTCGTTTTAAG
CTT

>TCONS_00076414
TTGCTGATAATCTGACTTTAAACATTCTGTAGTTCTATCCTCTTCTTGCTTATTTTGCACCGAAGCATCTCCTCC
GCAAACTAAAAGTCCACTCTGAGAAGTCAACTCTTCAGGAGACCCTGGAGTGACCAGTGCTTCACTTTGTACT
TCACTTTTTCCTAAGGAGGACTGCAAAAGATTGCTATTGACTATTGGACTCGACTTCATTTTGTTAGAATCATC
CAAGACATCACTAGTTAGGCTGTTGGTTGTCCTCTTCTTGACTGGTCGATCTCTATGTGGCATTTCTAACCCCG
CCCTAGAAAACTGCACTTCTCTCATACGCTTCTCTCGGCTAGTTTCACCCTGACCCAAGTTCCTTGAAGACCAC
ATAAGTGAATAAAGATCATCTTGGATCTGATACTTCTTCAAGGTCTTAATGCTTTCAGCTAAAAGGATTGATTT
CTCCTTGTATTCCTCGAGTTTCTTCAGCTTTTTTTTCTGAGATTGACTTAACTTAATGTTATTCTTCGTTTTAAGCT
T
>TCONS_00076486
TGTTTCTGGATCAACTCAATGATTGGGAACTCACCTGTCTATCTACTTTTGGATTTACTTAGGATCTGTATAGA
GGTAAATATCCATCCCATCTTTGTATTTTTTCGTTGTATGGGTTTCTCTTGTAAATGGACATGAATTGTTGCTTCT
ATCATGAAAGTAAGCACATCTGAAACTCATATGTATTTGTGATATTACGCTTGCGTAATTGTATTCTGGAATG
AAGTGGGATTGTTGTAAATTTCACGGGATAATGCATTGTTCGTCTGGACTAACGGTAATCTGGAAAAGAAATA
GGAGCTTCCCAG
>TCONS_00076866
GAGTAGTGTTGATATAGAGAGGATGCTAATACGTGGATCCCAAACCATGACAAGCAGCATCGCTGAGGTTCC
ATACGCGATTGTAGCAAATGGTAGGCGAATAATATGCTTCAGTTTCTGGCTGAAAATATAGTACCCCTGTAGC
CGTATTTTCTTAACTTGATAGACCAGATATTGCTGAAAAATGCCTGTGACAACAAGGAGGGCAACATTACAA
CTGCAAAGAAGTGGGAACACTAATCCTTCTATAGGGTGAAATATCCATGTAGCTCCCACCGCCAAAATAGCA
AAGCTTGCCAATAAGAAAAAGTAGAAAATACTCCCAATAAGGCTCGTAGGCTTCCTATGACCGGATAGAGG
TGCTTCATGCAGTACGTCCACAAACAGATTATTTTCGTCTGGGGATGCAGAGATTAAAGAAGACTGTATTTCC
GTTGACATCTTCTTCTAAATCTTCTTTCCTTTCCAATATTTCCAGTTTAAAA
>TCONS_00076867
CTTTTCCATTTCCAAAGCACGTTCAAGCTTAGCTGCTCGGCTGCTCCATTCTCCCAGAATAGCTCTAAGTCTCT
CATTTTCTTCTACATACTGATTATTTGCATGGCGAATACGTTGAATCTCAGTATCCTTCTCCTCTATTATAGATC
GAGCAAGCCTCAGCTCAGATTGCAATTGATTCATCTCAGCTTTAAGTGTGCGTAACTCTTGATCTCGAGTTGCC
AATAAGTGAGCAAGATCAACCTGAGGTGCATTCCCATCATTAGATCTTTCGTATTTGCTTAAGGTTTCTTGCAG
TCTAAGAATCTCCTCACTCAAAAAGTGTATATTTTCTTGCTGATATTGCAACAATGCCATCTGCTGATCTGATA
>TCONS_00076941
GTTCTTTTCCCAAATTAAGAGATTATGTGGGCATTTTAGGGTTGTTCTTGGGCTCAGAGGGGGTTTTGACGAAA
AAAAGAATTTCAGTTTCTTCAAAGGGTCCGTCTTTTTCTACTCCAATTTGGTGGGAAAACTGAAGGTCAACAG
CAGAGCAGCAGCAAAACGCTTCCTTTTTCGATTCAAATACTCATTTGCACATTTGGAGTAGATTTTTCAGGTCG
GGGTGGGGGGTGGCCTGCAGAAGATAAAACTGTTTGGAACTGTGGATGAGTAGATGACAGGGTAATCTGTAT
TGAGTCTTTGGAATGC
>TCONS_00076999
CCTTCGTTGAATACACTCTCTCATGAGATTCATCGAGAATCAAATTTGTAGCCTGGTCAAATCCTCTTAAAACT
CCCACTATATTTCGCCCATCATTTGTGATAACTGAGATGGTTTGATCGACAAGAGACTCAAGCCCAGGTCCAC
TAGCCATGTTGCCAAATCAGAAGCAATTTACTTGTCAGATCTTACAGTAGACTGATGCTTAAAC
>TCONS_00077169
ACAATACGTGCTTTGTCCCAATTACATTATACCGGCACAGACCAAGTAACCAGCAACTTTGTTCCTCGAAACT
ATAAGGGAACAACAGTTGTTCCTGAAAGCTTGTGACCATTACCGCTCTTCCAGTCACCTGAAGCCAGATGGA

GCAGCCTGAGCACCTGGTCCGGCTTTCCTCATAGCTCTAGATTGGGCGAGTATCTCATCATAGTTCCTTTTCTC
AAATGCATTGTCTCTTGAATCGATTATCTTGTCTGCAATGCCATAGTCAATGGCTTCTTGTGCCCGCAGATATT
TAGGGCGTTGAATATCTTTCTCGATTTCTTCCTTTGGTTTTCCAATTCCTTTCGCTAATAGTTCAAGGTAATAAT
CAGTGTTTGCATCTAGTTCTTTGGCCTTGATCCACATATCTATCACTGCTCCACTGGATTTGCTGACTTTAGGTA
AATACAATTTTGTAGATGAATTTGGCTGCATAGCACGGAACCCCTTCTTTCCCAGTGACAGAAGCATTGCTGC
TTGACCATATGCCATGCCACAGTTCACTGTATAGATATCTGATTTGCAGTATGCCATTGTGTCAGCGACGGCA
TACGCCTCTGTTTCAGAACCAACAGTCTCCATCTTCTCATTCTGTGTGCCTGATGAGTTTATATATAGGTATAT
AGGCTTTGATGGATTGTCATAATCCAACCACATAAACTGAGCAACAAGTAGCTCTGTAACAGCTGGTACAAT
AGGCATGCCCAGATAGACAATTCGAGCATCTAAAAGCAAAGATGGCAAATCAGGAGGCGCAGTTCGAGGTC
TTCCAGATCCTCTTGCTCCTCCACGGTACATGCTGACGCTCATGCTGTATTTCGAGGGGCCCCGATCATTCATG
CCAGACATACTCCACATTCCACCATTGCTCAGATAGTTATTGGCTGAAGATATACTATCCTCAGTGACACTTT
CTGACTGTCGCCGGGCTGGGTTATCTTCTGTCATGAACATATCCATCTGTGAAGGACTAAGGCCATAAAGATA
CTGAGGGGCACTTTTATAGTTGTCCAGTAGTCCATCTTTGAGATTTTCCTGTCTGAATTTTTGGGGTATATGGTC
CAAAGACTTAGCTACCGGAGACCTGTAACAATAACCTCGAACGCCGTTTGGCCTGCGACTGTGAACAGAAGT
TGCAAAGGAGAAATTGGGATTGGATATGAAAGTTGATTTACCAGAACCCAGCTCGTGATTTTCAGTAATCGTC
AAGCTAGAAAGTGGAGAGAGAAGCAAAGAAGAAGCCATGGTTACGTTTTTGTTTT
>TCONS_00077198
TCTGGGTTTATACCAAATCGACCGTAGAGAGAGTTCATTAGTATCTTGTAGATATATACCATAGCTGCATCAT
CCTTTATCTTTGCTTCTTGTCTTCTAGCGAAGATGTCTGATACAAAGCCTTCAAAAGGACTCTTCTTTTTCTCAT
ACAAGTAGCCCCTTAGCGGGAAAATTCTATAACCCAAGTTTCGGGCATAAAAAAATTCTTCGCTAAAATAGA
CGCCTACGAATTTACCTGTTGGGAAGGTTAAAGCATTATGTCTATCCTTATAGGGTAAGAAGGCTTTCTCTATT
GTAGAGAGGACACACTACAAAAGCCTCAATAAAGCCAAAGAAGTTATCCAATTCCGCCTTTTCTAAATTATT
ATGCCAGACGTCCTGGCCTGGGAGCCCCGTTCGCCTGGCATCGGAAAAGTTTTCATTATAAATGGATATAAG
GAGTTCACATCGTAATAGTCTAAATTTTAACCATAGGGCTTATAGACATCGGCATGTCCTCCATAATAACCAC
GCCGAATGAAGCGTTCTTGGTTTCGAGTTGGTATATGGATGGGCCAACTCTTTGGGTCGTAATAGTGCATACG
AAAGATTGATAGAGCTAGCGATGACAACGTTATTGTATCAACGATGTCTATTTTGTACAGATTCCAATAAATC
TCTTGTGCCTTCAGCATAACGCCACCTAAGAGACGAATATCTTGTTTCAGATAAGCCAACAATTGTTGACCTA
TCTCCGGAAGATACTCAAGTCGTAATTTCTCATAGGGAATGGTGCCTTTAGAACCAAATTTCGGGCATAAATC
CTGGGCCAAGTTATTTAGCGCAGCGGGAAGGAGAAGGTAGGAATCCCTTATACGGAACAATAATTTCTTTTTT
TCATTTCCACGATAGACTTTTAACTCGTACATTTTATGCTTCCTCATCACCGTTTGGAAGGAGTACTTGCCGAT
CTGAGAAGTAAAAGCTCTCGTTACTATAATGCCATCGTATCGTGAAAAGTTATGGAAATAGACCGTTCGAATT
TCTTTTTCATCTGATACCACAGCCGCTAAACGCTCTATAAAGTCGAGCATCATACGTTCACTTCGTTTTTTGAA
ATCTGATATTGAGAAATCGTTATCTTCACTGAAATATGTTTCAATATAATATTCAGACTTGGAAGCAAGATCTT
CACCCGGCTTAACCACTAAGAACCCCACTGCGCAAGGAACATGAACATCGTCGTGGAGAGCAGCCTCTATAT
CGGCAACAATGAATGGGCTCCTTTTTATTTTCTTAATTGCTTTCAGTGCGAGTATATGATCGGGACGACGACTC
TTCTTACCCATAGCTACCACTTCTACAGGTTCATCACTCATTGTCCCGCCATCCGTTAATTCATCACAAAGTTG
CTTAAAAATTTCCTCTAGGCTAAAAGGGTGGACTTC
>TCONS_00077199
TCTGGGTTTATACCAAATCGACCGTAGAGAGAGTTCATTAGTATCTTGTAGATATATACCATAGCTGCATCAT
CCTTTATCTTTGCTTCTTGTCTTCTAGCGAAGATGTCTGATACAAAGCCTTCAAAAGGACTCTTCTTTTTCTCAT
ACAAGTAGCCCCTTAGCGGGAAAATTCTATAACCCAAGTTTCGGGCATAAAAAAATTCTTCGCTAAAATAGA
CGCCTACGAATTTACCTGTTGGGAAGGTTAAAGCATTATGTCTATCCTTATAGGGTAAGAAGGCTTTCTCTATT

GTAGAGAGGACACACTACAAAAGCCTTAATAAAGCCAAAGAAGTTATCCAATTCCGCCTTTTCTAAATTATT
ATGCCAGACGTCCCGGCCTGGGAGCCCCGTTCGCCTGGCATCGGAAAAGTTTTCATTATAAATGGATATAAG
GAGTTCACATCGTAATAGTCTAAATTTTAACCATAGGGCTTATAGACATCGGCATGTCCTACATAATAACCAC
GCCGAATGAAGCGTTCTTGGTTTCGAGTTGGTATATGGATGGGCCAACTCTTTGGGTCGTAATAGTGCATACG
AAAGATTGATAGAGCTAGCGATGACAACGTTATTGTATCAACGATGTCTATTTTGTACAGATTCCAATAAATC
TCTTGTGCCTTCAGCATAAGAGACGAATATCTTGTTTCAGATAAGCCAACAATTGTTGACCTATCTCCGGAAG
ATACTCAAGTCGTAATTTCTCATAGGGAATGGTGCCTTTAGAACCAAATTTCGGGCATAAATCCTGGGCCAAG
TTATTTAGCGCAGCGGGAAGGAGAAGGTAGGAATCCCTTATACGGAACAATAATTTCTTTTTTTCATTTCCAC
GATAGACTTTTAACTCGTACATTTTATGCTTCCTCATCACCGTTTGGAAGGAGTACTTGCCGATCTGAGAAGTA
AAAGCTCTCGTTACTATAATGCCATCGTATCGTGAAAAGTTATGGAAATAGACCGTTCGAATTTCTTTTTCATC
TGATACCACAGCCGCTAAACGCTCTATAAAGTCGAGCATCATACGTTCACTTCGTTTTTTGAAATCTGATATTG
AGAAATCGTTATCTTCACTGAAATATGTTTCAATATAATATTCAGACTTGGAAGCAAGATCTTCACCCGGCTT
AACCACTAAGAACCCCACTGCGCAAGGAACATGAACATCGTCGTGGAGAGCAGCCTCTATATCGGCAACAA
TGAATGGGCTCCTTTTTATTTTCTTAATTGCTTTCAGTGCGAGTATATGATCGGGACGACGACTCTTCTTACCCA
TAGCTACCACTTCTACAGGTTCATCACTCATTGTCCCGCCATCCGTTAATTCATCACAAAGTTGCTTAAAAATT
TCCTCTAGGCTAAAAGGGTGGACTTC
>TCONS_00077200
TCTGGGTTTATACCAAATCGACCGTAGAGAGAGTTCATTAGTATCTTGTAGATATATACCATAGCTGCATCAT
CCTTTATCTTTGCTTCTTGTCTTCTAGCGAAGATGTCTGATACAAAGCCTTCAAAAGGACTCTTCTTTTTCTCAT
ACAAGTAGCCCCTTAGCGGGAAAATTCTATAACCCAAGTTTCGGGCATAAAAAAATTCTTCGCTAAAATAGA
CGCCTACGAATTTACCTGTTGGGAAGGTTAAAGCATTATGTCTATCCTTATAGGGTAAGAAGGCTTTCTCTATT
GTAGAGAGGACACACTACAAAAGCCTTAATAAAGCCAAAGAAGTTATCCAATTCCGCCTTTTCTAAATTATT
ATGCCAGACGTCCCGGCCTGGGAGCCCCGTTCGCCTGGCATCGGAAAAGTTTTCATTATAAATGGATATAAG
GAGTTCACATCGTAATAGTCTAAATTTTAACCATAGGGCTTATAGACATCGGCATGTCCTACATAATAACCAC
GCCGAATGAAGCGTTCTTGGTTTCGAGTTGGTATATGGATGGGCCAACTCTTTGGGTCGTAATAGTGCATACG
AAAGATTGATAGAGCTAGCGATGACAACGTTATTGTATCAACGATGTCTATTTTGTACAGATTCCAATAAATC
TCTTGTGCCTTCAGCATAAGAGACGAATATCTTGTTTCAGATAAGCCAACAATTGTTGACCTATCTCCGGAAG
ATACTCAAGTCGTAATTTCTCATAGGGAATGGTGCCTTTAGAACCAAATTTCGGGCATAAATCCTGGGCCAAG
TTATTTAGCGCAGCGGGAAGGAGAAGGTAGGAATCCCTTATACGGAACAATAATTTCTTTTTTTCATTTCCAC
GATAGACTTTTAACTCGTACATTTTATGCTTCCTCATCACCGTTTGGAAGGAGTACTTGCCGATCTGAGAAGTA
AAAGCTCTCGTTACTATAATGCCATCGTATCGTGAAAAGTTATGGAAATAGACCGTTCGAATTTCTTTTTCATC
TGATACCACAGCCGCTAAACGCTCTATAAAGTCGAGCATCATACGTTCACTTCGTTTTTTGAAATCTGATATTG
AGAAATCGTTATCTTCACTGAAATATGTTTCAATATAATATTCAGACTTGGAAGCAAGATCTTCACCCGGCTT
AACCACTAAGAACCCCACTGCGCAAGGAACATGAACATCGTCGTGGAGAGCAGCCTCTATATCGGCAACAA
TGAATGGGCTCCTTTTTATTTTCTTAATTGCTTTCAGTGCGGGTATATGATCGGGACGACGACTCTTCTTACCCA
TAGCTACCACTTCTACAGGTTCATCACTCATTGTCCCGCCATCCGTTAATTCATCACAAAGTTGCTTAAAAATT
TCCTCTAGGCTAAAAGGGTGGACTTC
>TCONS_00077201
TCTGGGTTTATACCAAATCGACCGTAGAGAGAGTTCATTAGTATCTTGTAGATATATACCATAGCTGCATCAT
CCTTTATCTTTGCTTCTTGTCTTCTAGCGAAGATGTCTGATACAAAGCCTTCAAAAGGACTCTTCTTTTTCTCAT
ACAAGTAGCCCCTTAGCGGGAAAATTCTATAACCCAAGTTTCGGGCATAAAAAAATTCTTCGCTAAAATAGA
CGCCTACGAATTTACCTGTTGGGAAGGTTAAAGCATTATGTCTATCCTTATAGGGTAAGAAGGCTTTCTCTATT

GTAGAGAGGACACACTACAAAAGCCTTAATAAAGCCAAAGAAGTTATCCAATTCCGCCTTTTCTAAATTATT
ATGCCAGACGTCCCGGCCTGGGAGCCCCGTTCGCCTGGCATCGGAAAAGTTTTCATTATAAATGGATATAAG
GAGTTCACATCGTAATAGTCTAAATTTTAACCATAGGGCTTATAGACATCGGCATGTCCTACATAATAACCAC
GCCGAATGAAGCGTTCTTGGTTTCGAGTTGGTATATGGATGGGCCAACTCTTTGGGTCGTAATAGTGCATACG
AAAGATTGATAGAGCTAGCGATGACAACGTTATTGTATCAACGATGTCTATTTTGTACAGATTCCAATAAATC
TCTTGTGCCTTCAGCATAAGAGACGAATATCTTGTTTCAGATAAGCCAACAATTGTTGACCTATCTCCGGAAG
ATACTCAAGTCGTAATTTCTCATAGGGAATGGTGCCTTTAGAACCAAATTTCGGGCATAAATCCTGGGCCAAG
TTATTTAGCGCAGCGGGAAGGAGAAGGTAGGAATCCCTTATACGGAACAATAATTTCTTTTTTTCATTTCCAC
GATAGACTTTTAACTCGTACATTTTATGCTTCCTCATCACCGTTTGGAAGGAGTACTTGCCGATCTGAGAAGTA
AAAGCTCTCGTTACTATAATGCCATCGTATCGTGAAAAGTTATGGAAATAGACCGTTCGAATTTCTTTTTCATC
TGATACCACAGCCGCTAAACGCTCTATAAAGTCGAGCATCATACGTTCACTTCGTTTTTGAAATCTGATATTG
AGAAATCGTTATCTTCACTGAAATATGTTTCAATATAATATTCAGACTTGGAAGCAAGATCTTCACCCGGCTT
AACCACTAAGAACCCCACTGCGCAAGGAACATGAACATCGTCGTGGAGAGCAGCCTCTATATCGGCAACAA
TGAATGGGCTCCTTTTTATTTTCTTAATTGCTTTCAGTGCGAGTATATGATCGGGACGACGACTCTTCTTACCCA
TAGCTACCACTTCTACAGGTTCATCACTCATTGTCCCGCCATCCGTTAATTCATCACAAAGTTGCTTAAAAATT
TCCTCTAGGCTAAAAGGGTGGACTTC
>TCONS_00077202
AAAAGAGACCTTGATGTCTTCGCCCTAAGCGCATGCCAGGAAACTCATTCCTCTCATGATTTCCATGACACGA
ATCTTACTCTAGGGATCGAAATAAAAGGTAAAATACTCCTTACTCGTAATTAAACCCACTAATCATCATATCT
TTTTTACCATGTTCGATTTGCAGAATGGGGATATTCGAATCCGGTCCTTCTGACATATCTGCATCCCCATGTAT
AGTAAGAGTCTTTCCAACGAGGAACTTCACTATCGTATAGTTGAATCGACTCGCCTCTCCGGTCTCCCCAGGT
GCTCATGTTACTTAACAAAAAAGCATGAAAATGATGACGTAGGGTGGAAACCCTTCGTGACTGCAATCGTAC
GTTTTCGTCTTAAGAGAAACAACCTCTGAATCCGGTTTTGTCATTAGGATAGGAGGGAAATCGGCAGCTCGAC
GGGGAGCTTGCCCGGCC
>TCONS_00077206
CCCAATAAGCCTTTTTGTTTGCGTAATTATAGTATGGAATCAATTCAGGTCAGGCTTTTTAACTGAATAATTAC
CAAGGAGTTGATTGATCACCAATTTAAAATCTCCATAAGAGTTGCAACTCTTTCAAATCCACAGCCATCTCAA
GTCAAGTATGAGGGCTTGGTACTCTGCGAGATTATTGGAACAAGGTTTCGACAAGGTGAAGGAGTGCGGCAG
CACTTCTCCTTCAGAAGTAAGAAAG
>TCONS_00077224
CGGGCAAGCTCCCCGTCGAGCTGCCGATTTCCCTCCTATCCTAATGACAAAACCGGATTCAGAGGTTGTTTCT
CTTAAGACGAAAACGTACGATTGCAGTCACGAAGGGTTTCCACCCTACGTCATCATTTTCATGCTTTTTTGTTA
AGTAACATGAGCACCTGGGGAGACCGGAGAGGCGAGTCGATTCAACTATACGATAGTGAAGTTCCTCGTTGG
AAAGACTCTTACTATACATGGGGATGCAGATATGTCAGAAGGACCGGATTCGAATATCCCCATTCTGCAAAT
CGAACATGGTAAAAAAGATATGATGATTAGTGGGTTTAATTACGAGTAAGGAGTATTTTACCTTTTATTTCGA
TCCCTAGAGTAAGATTCGTGTCATGGAAATCATGAGAGGAATGAGTTTCCTGGCATGCGCTTAGGGCGAAGA
CATCAAGGTCTCTTTT
>TCONS_00077228
CTTTCTTACTTCTGAAGGAGAAGTGCTGCCGCACTCCTTCACCTTGTCGAAACCTTGTTCCAATAATCTCGCAG
AGTACCAAGCCCTCATACTTGACTTGAGATGGCTGTGGATTTGAAAGAGTTGCAACTCTTATGGAGATTTTAA
ATTGGTGATCAATCAACTCCTTGGTAATTATTCAGTTAAAAAGCCTGACCTGAATTGATTCCATACTATAATTA
CGCAAACAAAAAG

>TCONS_00077348
CATGTTTCCCTTCGGAGTGACTTGGTATCCATTTTCTAATCCATCCCTTTTCAGATGATCTACACGTAGAAAAC
CGCCATCTCCGGCAATGCTTTCCAATGCAGCATCGTACAAGGTTGGATCCTTTTTCTTTATTTGGGGATTTGTTC
AAAATCTCTCAAAGTATCATCTTGTTAGTACTTTGCTTTCTCAAAATATATAGCGGCACTCATTGGCGAGCCTT
TTGCTTTTTCGAGTAACTTGTGGAGGCCTTTAAACTCTTCGAGACCCCCCATTTCACCATTTAGCGGACATTGC
TCCTATTCC
>TCONS_00077355
TTCCATCTTTAACTAGAAAGAGAACAGACAAGAGCATTTGAACTGCACTCTGGATATCCATTAGCACGATAT
AGATACAATCTTCTGTACAGAATAAGCAGTTTGATCTTCATATATACAAGCTATTACTACAACCTATAAAAGT
TCTAAAGATCAAGAACATCAGTAGCCGGCCAGCTCTAACAAGGAGATGATCGGTTAAAAGGGTTCCTTCCAA
AGGCGGATAGTGGAAAATGCAATGTCGCTTTCTTCATTCCTCTAGCTAGTCATTGAACACCATGCTATCTAC
AATATAGGATCGACAAACTGATGAAAGAACAGCAACAGGGGCAACTTGTTGCAGGAACCTAGTGATGTAGT
CTAGACTGTGTCATCAAGACAGCAGGATTATAATAAAAGGGAAACACAGTAACAGTTCGCCAATACTCGCCC
TTCATCTGCATGAGATAAAATATTAGTTAGAATCAGCAGAAGGTTAGGCAGTAAGGTGCTTTGAGCGACAAC
TGTAGGCCGATATCTTCATAATCAAAATATTTTCCCTAGCAGGTTGCTTCCCTCAGCAAATGATGTGCCAGAA
ATGGCTGTGCTGCATCATGTTCAAAATTGAATCAACTCCTACAAGTTAGTCAATTACAAATGGCAAGCTTCCC
TGTCCATTCATCTGCTACCCCATAATCAATACTGATCTCCCTAAGAGGGGGAATATTCTCCATTGCAAAGAGC
ATCAGGTGGGGGAAAGATACAGTGTTGTGATCATAAAGCACGGGCTGCACCAACACGTTGGGGCTTGAACTG
TGACTCATATAGCATGCTACGTTCCTCATTCTAGACACATCCATCGCGAAATCCAGAGGAGGAATGGAGGGG
TATGCTGGCCGCACATAGTCAGGATATATTTGAGACAAATCTCCCCATTCTGCCCACCTCTCAGGAAAGCGAC
TTGGATAGACTAAACTATCACCATTCATCGTAAAGATTTGGGCTTGCTCTCGTGTGAGTACGACCCCAGTAAA
TTCACAGATGAAGGACCCAGCTTGGATCAAGTCCAATGACCTAACTCCCCAACCAGTCTCTCTAGACCGAAA
CACTTCAAATCTGTGCCTCAAACCCTTTTGACTCACTCGATTCCGACAAGTTGGAGGACACTGACAATGTGGT
CCACATTCAAAAACTAATGGTTTACCTCTCACCAATATCCCATTATAATCATAGGCAAATTGGCCACCGTTTC
TCATAGCACAAAAACAATTATCCACACACCCATTAACGCACTCGCAGCCATTTCCACTGCCCACATTCTGGTA
CACATACGGAGGAAAAACAGTCTTCAACAGATAATCAAAATAAGCAGGATCGTGATTATCATCGATATCGTT
GAAAAGAAATACTGGCACGTTTTCTTTTTTCCTGGATATATCTAGACTAACATAACCCGCAGGCCTTGCCACC
AAAGGTCTAATCCTAAGATTCTGCGCAAACCTAAGAATGGCACTTCCCAACTCTTCCTGATTCTCAATCCTAA
CAAGCTTGTACTTATACACTCCAAATCCAGACTTTCCAACATCAAACCAACATTCAACAATTCTATACAATCC
ATCATACACATAAACTTTACCACTAGCACTACCTTCGTACTTAAAGCCGCGAATTACCCTTACCTCAATTCCA
TAGTGCATACTCCGCTCCAACGCCAAATTCCCACATTCCAGCTTCTGATGCATACATTGCCTCGACAACTTAT
CCTGTCCGCCGTGCCCAGTATATATAATCACATCTCCACCATCCTGATCATCCTCGTATCCACCCGAAACAAT
CACACTCGTAGCAATCGGCTCCCTATTCGAGCTCTGACTCGCAGGGACATAATCAATGCCAGCTTGAACCTGC
CCATGTAAACCAACAACGCAAAGCTCCATCCTGAAAAAGAACACATCACCAATAAACACCCCCGGGATCGC
CCCAACAATACGCTTATCGCGATTCATCCACAACCCGTGTTCTCTTAAAACCTGACAAGCCTTTAAATCACCT
CTAGTCTTCCTATGAGGCACCACACCCATGTTATTCTCATCGTCCGCCATCGCTAAAACACGCAACGAATCAT
ACAGCATTCGGGTTCTCCTAACAGCATCGCGGAAATAACGTTGATCTTCAGGTTTAAGATCTGTAACCCTAAC
TAATTCAGATGATCTTTGCTGATATTTTCTTCTAGGAATTACCATTTCTGAAATCTGATTATCCTCATTATTAAC
CGGAACAATGGCACGTGAATTATCGAGTTCCGCCTCGATGTCACGGGTATCATCATTCTCAGGATCAGCAAC
AATCTCAATATCTCCGTAACGCTGCATTCGTTTAGCAAAAGCTTCACGAAACAGCTCAGAAATTCTGTTATAC
TCAGAATACACATTTGTTCCATCAGACCCAGATGGGTTTTGTTCAGGTGTAGTAATTGAGCTGTGTGATAGTG
GATTAGGAGTGAAATTGGAGTTGAAATTGGGAGTATTAGGGTTGGAGAAAAGAGGTGGGGTTTGAAGATCA

GCTTGAGTAAATTCATCAAGGGGTTCAAGTTTAGGTTCAATTTTGGGGGTAATTAATGGAGTTCGAGAATTAG
AGGTTAAATTGGTGGATTCAGGTTGAAGATTAAGGTCTTGAAA
>TCONS_00077610
CTGCTCTGCATTGCTGAGATACCAATGACTTCTCGGAATGATTCCAGATTCTCAATCAGTCACACCATTCAAC
AAACAAGGCCTACCTAACAGGCAACAGCTTCTATTTAAAGAAAAAGAGATTAATGTCTACATCACATGCTAC
ACGAGATTGCTATCAGCAGTAACGGGAAGATACAAACGAAACAAGCTTGTGTAGAGGAATAGCACATAACA
ACTGAGCATCGGCACCAAATCTTCAATTTTGCTCGCTACCATTTGTTATGGCTTAAAATTAAGGTCCACCTCAC
CACCACACTGGACTCAGCATAATCTTCCCATTTCCTTCCAACAGAACAAGAATCTTCTATAGCAAAAGTTCAC
TTATTTATAGTATTCTCCTCTCCATTCTCTTCTTCACTATCTTTTGGCCCCCCACCAAGAATTTCAAAATCATCA
AAACCTGCACCTAAGGCTTCAATATCATCAACTAGTGGACCTAAGGGAGCTATGCTGCCTGAAGGTGGGTCA
ACTGTCATCCAGTCTGAATTTTCCATGTCCTCCGAGCTTAGTTCCCTTCTGGAAACCCAGTCATGAACAAGGTC
CATTTTGTCATAGGAGATGGAGTCCATACAGTCCGG
>TCONS_00077647
CACAGCATCTCAATGAGGTTTCAGTATCACATGCCTGATTTACATGCTTCATTTCATAGTAGTAGCGCCACCAT
AGCAGGAAAAAAACTACAAACTGGTAAGAGGCTCGTAGCATATAAAGCAGTTAGCAGGGCCTTCTGCACAA
AAATCAAGAGGTTTGTGCATTTGCATCATAAGAAGTAATCAGGTGTTCTTCGGGTTACATCTGGTTCCCCTCTC
CTTGGAGCTGGTTCAAACTGAATGAATGTGCGCTCCCTGGAATCATCAACTTCCAAGATGGATGCCATATTGC
CACAACGGTAACAATAATTAGGTGCACTAAAGATGGTAACCACCTTTTGATCATGGGCCCAATTGAATCCCT
CCATAACCAGCTGGTGTGCTCTAGCAATTAGCTTCAAGTTGTTGGTGTGGTTAAACTGCTCAGAGATATCTTG
GCCAAATGTATATCCAGCACCCCTAGGTGAGATACCCCAACCACAACGATCATCAGGATCAGACCATAAAA
GATCACACATAGCACCCTCATGTGGAACTTCTTGTACACGGTCAAAATTGCGAATATTATCAAGAGTTTCAAT
AGATGGAGACAAACCACCGTGTAGGCAAAAAATCTCTGACTCAACCAAAGCAGTCAGAGGAAAGTAGTCAA
ACAGATCTGTGAAAGTCTTCCACACATTGGCATTACCATACTTCCGTAGGCATTCATCATAAAACCCATAAAC
CTGAGTAATCTGGCGACTCTCATGATTACCCCTTAGGATTGTAATTCGCTGGGGATAGCGCACTTTGAGAGCC
ACCAAAAGCGTTACTGTTTCAACAGAATAGTATCCACGATCCACATAATCTCCCATGAATAAATAATTAGTGT
CAGGACACTTTCCACCAATTCGAAAAAGCTCAGCAAGATCATGGAACTGACCATGAATATCACCACATATAG
TCACAGGGCTTTTCACAGGCTGCACGTTGCTCTCTTCCATTAGAATCTCCTTCGCTTTCTCACATAGTCCTCTTA
CCTCCTGTTCAGACAAGGGTTTGCACTGCATAAGCTGAGCAATTTGTTCGTCAAGATTTCCATGAGATGCGCT
CGATGGCACCGGATCCATAGCTGATTGATCTTCTTTTCTACTGCTTCTGGAGGGAAATTTGTGCAGAAACCCT
AGCGGAAAAGGGGGATAAAATCAAAC
>TCONS_00077653
GCTTGTAAGATAGATGCATAACCCACTTTTTGTTTTTAAGTGATTTAAATATTTGAAACTTTTTAGCTTATCAAT
ATTTTTCTCTAGCTCTCTTCCCTGAGCAGGTCACAGCACCATGTCCTTCACTTGAGTAGCAATGTAGCTGTTTG
CCCCTCTGGAGTGTTTTTGACTTATCTCTCAGCCATATGTGAGGATGATGTTCAGGGGAAGAAGCTAATACAT
GCAGCCATCAATCCGCTGTTTTGCGTTCCGGTTTCTGGAAGTTCTGAAAATGATAATTCTGTGGAGAACTATA
GTGAAACTAGAGATGCAAAACCTGCTTTACTTTGGAGTGTCTTGTATACTCAAGAGCTTACTAAGGTATCTGG
AATACCCTCTTTTACCTTTTTTGTTTTTGGTTTGCTCTACACAGCTACCAGCTTCCATTTTGTTTCAATATGAATT
TTCTTCAGTTCTTGTTTCATTTCGCTAAAGGAAATGTGCTATTCGTTTTGATAATGCTTTTTGTTTGTAAAAATAC
TGAAATGGCTATCAAGCGCCTTTCGCATTTTAGGCCTTTGCTGTCAGTCAATTTTGTTTCCTTCAGGGTTATGGA
TTATTTCGGCCTGGAATCTTCTGCA
>TCONS_00077669

CTGGGTATATATGTACAGAGGCGAAATCAATCGTCGAAAGCATGGAGTTGCGGATGAAATCTGATCCAAGAT
CAGCAGCCCAAAACTCTGGATTGCCAGTTGATTTTTGGGACTTTTACGACCGTAGAATCCTTCTAGGCCTACT
GTAAGCAGATGTTTCCTATCTATTGATTTCACAAATGTTGACATCTCTTCTATCCAATCTTGGAGTGTGTCACC
AGAAGGATCGGTCATGCAGCGGGGCTCGTTTATTAACTCCCAAGCAAAGATAGTTGGATCGTCCCTGTACTC
AATCCCAGTGTATAAGTTTCTCCTTGTTAGCACCGTCTTGACATAATGTTTGAAATAACGACGAATGGTAGGG
TCATAGAAGAATGAATCATTGGAAGAACTTAAAGCAACACCTTCTTTCTCTGCCCATTTTACATACTGAGTCT
TCCCACCAAATGCATTCAGATTGTTAACCAAGCTAAGCATTAGCCTAATACCGTTCTTTCTCGCTTCTGCAATA
ACATGATCCAATGCTCTAAAAACTCTTTCATCAAATCTACCAGGAGAAATCTGCAGGGCATTATATCCACCAT
CATTAAAAGCCCAAGTTCTACATACAGTGAGTCCCATCTTAGCACCAGCTTGTAAAATTCCTCTAATTCTTGAT
CTTTTACTATAATCCACAGCATGATCCATTAACCAATAAGAATTCCAACCATTTATGTAAAAAACTTTACCAT
CAACAAAGAATTGAGTCCCATTTCTCTCCACAAAACTTAGCTCTATTTCTTTGTTGTAACTGAACCACAATAA
ATCTCCAAATGAAAAATAAAGAAAAGTGACAAATAATGCTAAACCAATTACTGGATATAATAGGCC
>TCONS_00077708
TTACTGAACATCAGATGTTTTGTATGTGCGGGTGATTCGGATCTGTGCGAATGCATAAATTGCTCGGACGATTC
TAGCATAATCAGTTCCAAGCATCTGAATGACAACATCTGCTAGAAGTGTTCCCCACAGAATTGGACCAAGAA
ATGTCATCATGCTCCTTAGGCCAAAGTAACGGGAGGCAGCACCTGCTAAGCCCTTTTTTGCCGCCAGCATGGC
CAATCTGGACTCCAGGTTAAGAGCCGCCACTTCTCCACCCTTTTTAAGGACTTCCTTACTTATCTGATAGTTGG
CAGCCTCCAATAGCATCTTTCCAGAAAATCTCCTGGCTAACCCGTTGTATATCCTCCCCAATGTTAGCACCCC
GCCACCCT
>TCONS_00077721
CCATTATCCGCAAAAACGTTTCTGGCTATTTCAGATAGGAAGTTATCCTAATAAAGGCTTTCTTGGAATCTCA
ATCTTCCATCAAAAAGGAAAAGTTCCTCTCAAATCAATTATTCTGCACTGTGGAGCCCTAAGCTTTCATCGCA
TTACGACGCTGTTTATCAAACCAGCTTTTGGTAGACATCAAGGCATTCACTAAATCTGACTCCAAGGAAGTCA
AGTGGCTATCAGGTTCACGCGCATCCATAAAATAATCATCATAGCCTGTAACAAGAGACAATTGAATTGACA
AAAGATCACGAGCTAAATTTGATAGGGCAGGATATTTAGGACTTGCAGCTTTCCACCAACTCAACTCATCAA
AGTTCTTAGTCCAAGGCACAATAGGTTCTTCTAGATAGCAGTCAATTTCTGACTTTGGTGGCTGACTAGTTGTT
TCGATGAATTTGCTAAACTCTTCTGAACAGTCAAATCCAAAACTAGGATCATCAACAATTTCGTTGGTTCAAG
AAGATATTCACCCGTATCAGAATCGTCATCGCTCATAGGATACTCCATTGTGGACCTGTGCACCACATAGTCA
TCGAGAAGGCTTCGGATTGCCTCCAAGATAGATGCGAGCGGTAAATGATCGTTATCTTCATACTTCAAGAAA
CAGAACTCTAAGTACTTCACTTTGTAGCGTGGATCCATAATAGTAGCTAGAGCCAACACCAAATATATATTCT
TCCAATACTTGTCAAACTTTTTCAGCATAATATTGGCTAAATTTCTGACAAATTCATCTGAACTCACAAACTCT
TTCATCAGATTAAATCGAAGCTTATTGAGATTGTGGAAGTAGAGACCTGCAGTGGGACGTTTTGACATAAATA
GCACTTCTGCTGCAGTATAGACATGTCCTACAAGTTTACATACTCCCCTAACTTTCTCCCACTCTTGGGGCGAA
GGTATATCATAATCCTTATAATCATCTTTCAAGTATTCTCCTTTAGCCTCTAACTCCAAAGCTTCCTTTAATTTA
TGAAATGTCACATTCCAAACTGGTAGTGATTTGCCCCAAAATACTATGAGTCGAATGTCACTGATTAAATCCT
CTATCATCCGAAATGCTTTTTTCACCATTGAACTAAAAATGTCTGCACAACAAGATACGCGAAACAATTGTCC
ATCTAATTGGAGTCTTTTCTTCTCAAGAAGTTTACTTTTAATGACCTCAATAATCTCATCAAAGTCCAGATAGC
TATGCACAGTGACAGCAGATACTTTATCTTCAATACCAAAATCCGAAAGACACTTAATTGTCTCGCCAATGTA
ATCGTCATACGCACCAAAAGATCTAACGTTAATAACCCAACTCCTTAGCTTCCAGTCTTCATCAATGAAATGA
ACTCTTAAACAAATGAAGTCAAAACAATTTTCCTCAAAATCCGAGGAATGCCAGTAGCCAAAGTCATGGAAC
TTGTTATACGTTAACCTCTCAACTGAGAGGCTAACCCGTCCATTCAAGCTTCTCAAATTTTCTTTAACTCTCGCT
TTCTCTTCCTCATACAATTTCAAGCAATACCGTGAGAGAATGGCAGATGAAAACAAACGAGTTTGTGGATTA

AGATAAGCAATTAAGTTTTTGAAGTACACGTTTTCTATTGTTTGGAGATATACCAGATTGACAGAAAAACCTT
GCCATAGCTTGATAAGCTGTTTCTTCCTTGAAAACATGCTCTTTCCCAAAGTTACTACTGCCCGCTTCCCTGAG
ATTTGCATTTCCTGGTCCACACTCATGACCTTTCCCATTCCTTGGTTC
>TCONS_00077729
CTTTGATCTGATTTTCTGTTAGATAAAAAAATGGCAGCTCCTCCCATTCTGAACAAGCAATTAGGTAACAACA
TTGATCTCTCCTTACCTAGATAGCTATTCGGGGTGATAATCTCGGTGCTAATGACCACAACATTTGAATTAGG
GTAATGTTGAAGAAGATCACGAGCTAAATCAATGGATATGACTCCTGCACTGCATCCCATTACAGATAAATT
AAAACTCTTTATATTACTCCTGAG
>TCONS_00077797
CCAAATTTATCCAAATTGTCATGTAATATGTCTCCCGAGCTGTATTGCCCAGTGGTAGCTTTTGCATATCTATTT
TGACAAGAAAAGAAGTGGATAAGTTTGATGGGTAGTCTCATAGAAGCAACTGAGGGGTGGAATTTCAAAAG
CATGGTGTCATTAACTATTTGGATATGGATGTGGATGTGGATGCACAAAGCATTTCTGAAGAGTAACTGCCAA
CAGAAAAATACAATGGTATTCAGCTTAGATGGGCGCCCAATCAAACACTATGAGGTGCTTCCATCATTTTCTT
TTGCTGCTGGTTCTTCCTGAAGTTTCTTCTTCCAATATTCATCAAGTGGTTGTGCAAATATAGCTTTCCCAGATA
CAACTCCAATTATAACAGCGCCAATTACCAGCGGAAACGACAAAAATCTTCTCCTTCCGAACATCTTTGCAGT
GCTTTTCTCTCTGTTTAGTAATTTCGCAAAGAGATCGGAGCTATCAGCTATCGGCTGGCGGAGCTAATGTTTTG
GTCAATTACATTCACG
>TCONS_00077997
GTTTCCGGTCACCAGAAAAATCTGGAAATGAAAAACTTCCCGAGAAAATTTGATTGAATTGGACTGTTGTGG
AAATCGGTAGCATTTGATTTACAATACAAACTGATTCGTTTCTTCAGACGGACAAGGAAAGAGTATCACATA
AATTGGGATGGAGGAATAGCTAACTCCAATCCAAAAAATGGGCTTGAATGGAGTGGAATACAAATAGATGA
TTTCAAATAACGGGGCTCAACTAGTTTGCGATCGAGCATAGTTGATTGACACTAGCGAGTTCTTCTTGAATCA
TCAGCTTGCTGCTACTAAACTGTTTGTATTAGCTTCATTTCTTGTCTGATAATTGTGGCAATTAATAATTTCAGA
GTCGGTTATTGAGTAAAGTACTGGCCTTTGCTCTAGGAAAGTAAAATGGAGAGACTGAGAGCTCGAGAGCTC
AAATTTGTCAAGAGTTGGGGTGACAAAACATGATTAAGGAAATCTTATAAACATTTGGTTTATTGGTTATATT
GTTAATTATCCAAAACTAGTCCATATATCTAATGATGGATTATAATGTAAAACAGTCTTTTTTCTTATGTTGGA
CTC
>TCONS_00078058
TAGAGTTACAAAACCCCGTCCCATTAAAGTTCAGAATCATGAAAACACATACTAATATGTCCATTAACAAAG
GGGAGAAGATTCAGAGAGTTGTGCCTCCAGTTTGATGCAACATTGCATCAATTTCATCACCATCTTCCATCTC
CAGCTCATCCGGAGTCTGCTCCCCACGAAGACGACGACCATCAAACAAGAAGGCAATTGAATTGAAATCAA
CTGACTGCCTGTCACAATAAGCATTCATCAGTTTTTTTAGCTGAGTGCTTCTTTTGATTCTGAAAAAGACCTCA
TTCCCATCCTGGCCTTTGACTTTGAGATTGATGTGAACTTGATCGCCGCTGGGCTTCTTGTCTTCCTCTGCTTGC
GACATTTTTCTCTCCCAAAAAGCTCCTCTCTCTCTCTCTCTCT
>TCONS_00078117
ACTGTTGCTAATATTGCTAAATTATTGCAGCTGCTGAAGGAGGGGGAGCTGGGAAAGAACGTTAATGTGATT
ACAGAAAATGGGTCGATATCTGTGCCATTAATTTTAGAAGCAGCAAAAGAAAATGAGGTTATAGTTAAGGAA
GCAGTAGAGCAGGAGAAGGTGATTAGGGAAGTTCCTAAAGAATCAGAAAGAGAGGGTAAAGAAGCCAAGA
AGAGGCGGCCATTGAGAATGGGATCTGAAGGAGATGAAGTTCGACTGATGCAGGAACAATTGTTAAAATTG
GGTTTTTATTGCGGTGAGGAAGACATGGAATTCTCCAGCTTCGCTAGTGGGACTGAGAGTGCTGTGAAAACTT
GGC
>TCONS_00078124

TTTCACAGCACTCTCAGTCCCACTAGCGAAGCTGGAGAATTCCATGTCTTCCTCACCGCAATAAAAACCCAAT
TTTAACAATTGTTCCTGCATCAGTCGAACTTCATCTCCTTCAGATCCCATTCTCAATGGCCGCCTCTTCTTGGCT
TCTTTACCCTCTCTTTCTGATTCTTTAGGAACTTCCCTAATCACCTTCTCCTGCTCTACTGCTTCCTTAACTATAA
CCTCATTTTCTTTTGCTGCTTCTAAAATTAATGGCACAGATATCGACCCATTTTCTGTAATCACATTAACGTTCT
TTCCCAGCTCCCCCTCCTTCAGCAGCT
>TCONS_00078157
CCAAATATCTGACACCATATGCATCCTGATTTCTCTTTGACCATCAAGGTTACCCGGTCCACTGCTGCCTCCAC
TTGCATAGGAATTATCCTCAGCAGCCTGCCAAACGGACTGCCACGCCTGCGACGGCGGTGGCTGAACGTACA
TTGAAGGATCAACCCCAGGCATAGCAGGCCTGCCCATCATCACACCCGGTGGAGCAGGCTGGCCCATTGGTG
GGTAATAGTAAGGCACACCACTAGCTGTGGAGCCCACAATCCCAGCAGGCCCAAGACCAACACCACCCTCT
TCCTTGATCTCATCCCTCGGAACAATGTCAACAAGGAAATCAAAAATGTCAGTCCGCGTAATCGCGGCAGCG
ATGTCGTTCTTCTGTAAAGTTCGACGCTTGTTTTCCTCAGCGTGAAGCCAGGAACGAATAGTGAGTTCCAGAA
TGAAAAGCTCACACGCTTTGGCGAATAAAATGGGAGCTTCAGCGGAGATCATGCGGACATCCTCGTCAGCTT
TCATGATCTTCTTGATTCGGGCTAGAGGAAGTTGGTGGTTCTTGAAATCGTTAACCTGTTCGATTTCTTGGCGC
TGGTAGGTCCAGAACATCTGGAGCTGCTGCTGTTGCTGCTGAAGGAGGTGGTGGTACGGTGGCTGCGCTGGAT
ACGCCGCTGCTGCTGCCGCCGCCGCTGCTGCATTCGCCGCCGATTGCTGGTTGTTTTCCATTGGCGTTTTAGAC
GAGGTGGTTCACCGGCG
>TCONS_00078170
GAATCCTCAAATATGCGAACTGAATCCTCAATTCCTTATCCAAACTGCGCAATTTAGAACAGCCAATAAGCA
CTGATGACATTGTGAACTCATCCGCTGAATATATACAAGCTCTCATCAAACAAAAATAAGACAGAGCTGTAT
CTTCATGCCCTGATTTACAGCAGACTGAAATCAACGAATTCCATGAAACAACATCCACCTCCTCCATGGACA
AAAAGACACCAGTACCATAGTCCACTAGCCCAAGGTTTCCATACATATCAATCAGTGAATTCCCTACCACCA
CATTAGACAAATCAACACCACTCCTTAGCATGTTTCCATGTATTTCCTTGCCATGAAAAGCAAACTGCACACT
CGAAATCAGAATCGAGAAAGTATATCCACTAGGCCTAATACCATAGTTTTGCATTTCCAAGAAACCACCAAA
AGCAACCTCAAACA
>TCONS_00078180
AAATTTCAAGAACTATTTGAAATGAAAAGAAACTCTAAATTTTTGTCACATCACTTAATCAGTAACATCTAAA
ATTTGCATTGTTCATTTACCATATAAGCTAATTCCATGCTGAAGCAAAGGTAGTTGAAAAGCTACTCCTCATC
CACCACAACACTGATCTCACCTTTCTCAAGCATATCGTAGAATTCCTTAGTTCTCCTCTGGTTGTTCCAATGGT
GCATCTTCCAGAAACCTCCCGCAACCATGCCAAGAGTAATTCCAATCAGTATCTCCTTAACTACACTTGGACC
CTTGTACACAGCATGTGCAACATGGAACCTGCCATCTTTTCCTCTTCTCAGAAAGCAAGTCGGTGAGACGGT
GACGCGAGAGAAGCAAAAGGAC
>TCONS_00078232
GGGGTTGCTCTGATGGTAAGCAACCTTCACTTCCAACCAAGAGGTTGTGAGTTCGAGTCTCCCCAAGAGAAA
GGTGGGACGTTCTTGGAGGGAAGGATGCCGAGGGTCTATTTGGAAACAGCCTCTCTACCCTAGGGTAGGGGT
AAGGTCTGCGTACACACTACCCTCCCCAGACCCCACTAAGTGGGATTATACTGGGTTGTTGTTGTTGTTGTTGT
TGGT
>TCONS_00078233
GGGGTTGCTCTGATGGTAAGCAACCTTCACTTCCAACCAAGAGGTTGTGAGTTCGAGTCTCCCCAAGAGAAA
GGTGGGACGTTCTTGGAGGGAAGGATGCCGAGGGTCTATTTGGAAACAGCCTCTCTACCCTAGGGTAGGGGT
AAGGTCTGCGTACACACTACCCTCCCCAGACCCCACTAAGTGGGATTATACTGGGTTGTTGTTGTTGTTGTTGT
TGGT

>TCONS_00078234
CAACAACAACAACAACAACAACCCAGTATAATCCCACTTAGTGGGGTCTGGGGAGGGTAGTGTGTACGCAG
ACCTTACCCCTACCCTAGGGTAGAGAGGCTGTTTCCAAATAGACCCTCGGCATCCTTCCCTCCAAGAACGTCC
CACCTTTCTCTTGGGGAGACTCGAACTCACAACCTCTTGGTTGGAAGTGAAGGTTGCTTACC
>TCONS_00078235
CAACAACAACAACAACAACAACCCAGTATAATCCCACTTAGTGGGGTCTGGGGAGGGTAGTGTGTACGCAG
ACCTTACCCCTACCCTAGGGTAGAGAGGCTGTTTCCAAATAGACCCTCGGCATCCTTCCCTCCAAGAACGTCC
CACCTTTCTCTTGGGGAGACTCGAACTCACAACCTCTTGGTTGGAAGTGAAGGTTGCTTACC
>TCONS_00078323
CACGAATGTAAGCCATTGATCTCAGCATTGATCCTAGAGTATCCGTTGAAACTTGAAATTTCACTTCAGTTTCT
CCGGAAGGTGTCTTAGTATCTTGAAGCTTTAAATTGATAACAGCTACTCTATTTGCCGGCAGTGAAAGCTGTT
GACTTATGTAACCCATGGATTTCAGCATTGGCTCCAGAGTGACTCTTGTAACTCTAAACTGAACTTCTG
>TCONS_00078334
ACAACAATTTTCTTGCAAATGATACGGCTGCTGGAAACAAGGATCTCTGCCTACTCTAAGGGCTTCAAATGGT
GCTCGAACAAAGATGCCATTTCAATAGCTGTCAATGCTGCTTCGGCACCTTTATTCCCAGACTTTCCACCAGC
ACGATTGAAAGCCTGTTCCAAGGTATCACATGTCAGAACACCAAATATGCAAGGAGTACCTG
>TCONS_00078335
TGACAGAGTAACTCTTGAAGGTGTCCAAAGCACCCTCCAAAAGCTTCTTCGTGATCAGATCGTTAAAACGTG
CCACCACAATAGCAAAGCGATGTCCCTGTGCAGATAGAAGAGAACCCCTCAACTCTCGAACAGCTGATGTCT
GAACTAAATCAGTACGTTTTTGTTCCTTGCTTGCAAATCCAAAACCTTGGGATTGAGTGAAAGAGAGAGCAG
CAGTTAATTTAGGTGAAGAAGAAATGGTTGTGTTATGGAAAGATAAGGAGAGGAAGAGACGATGTGAGGGA
TTGAAAGTTGATTGACGAAGTGGAACAAGACCGATTTCTCCAAATGCCG
>TCONS_00078393
CTTGGTCCGAGAGTTGATACAACTTTGGGTTGCTGAAGAATATGCAGTAGGTAATGAATTAAAGAGCGCAGA
GGATGAAGCAAAGGGCTACGTGATGGAGTTAATAGATAATAACCTTGTAATGGTTGTCCAAAGGAGCTTCTT
CCGTGAGGTGAAAGCAATTCGCATGCATGATCTATTACGTCAGATGATTCAAGTTCTAGTGACCAACAATGTT
ACTTTCAAATGCAT
>TCONS_00078489
ACTACTCAGCTGATAGATCAGGTGAGCCAGCCATGATCACTAAACTCACCAAACCAGCAGCATATGAAAATT
TGAATCATAGGTTTCTATATGCTTAAGGAAAATGGGATTTGCAGATTAGGATACGAAAGTTTATTTATATACA
ATGGAAAGCCCCTATAATCCATGGACGACCAGTGGAGTTCATAAAAGAAACCAAAGAAAGGTCCGCGACAA
AGCTGTCCCCTCTCTCCATATCTCTACTTACTGGCTGGGTGCAGATAACACTGTAATAATAACAAAGCCAGAT
AGAGATACTGTTCGTTCAGAATTGATTCAGATGGCGAGGACCCACGGAAAAATCCACCTTATTCATGCTCCG
CACAGCAACTTGCCCGGTCAATATTATATGTCTATGCTAAACTTGGTGATCTATTTTCGTTGCC
>TCONS_00078632
TTCTATCAACTTTTGTCCCAAAGGATATCTATCAAATGTACCTAAATATACCAGTTCTAACTACATTGGAGAA
GATGAGACCAAGGATCCTGCATTCCCTTCAAGTTCTCCAGTTACCAATCCTGTGAGTAGCAGAGGGTCCGATT
CATCGATATTATTCACCTTCCCGCATGTCTTCTTATTGTGGCCCAGACCCTTACACTTGCTACACTGCATCTGA
CGTTTGACTATGTCAAAAGTGTCCACCTTTTTCATCTTTGGTCGGCCAGGTGGACGTTTTGTTGGTGGAGGAGT
AACAACAATGGCCACATGGTCCATATCGGGTTCACCTTTTATTGGTTTTTCGAGATTAGGTATGGGGTTAATTG
ATTCGGCATATGTTACATGGTAAGTCTCAGTTGAGAAGTATCTGGAGCAATGATCATAGGGGCTCCTCCCTAG
GCATTCAAAAACAGCAATAGCATGGCAACATGGCAACCCGTTAAGCTGCCACTCTCTGCAACTACAATCCCA

CTGATCAACATCAACTACTTCAGCAGATTCACCGCGCACTTCAAATGTGCTCCCGTGTGAGGGTAACACTTGA
AGCAACCTTGCTCGTGACGTTTCACTTTGAAGTTTTTGTTCCATCACAGGGGTCAATGGCATAACCCACTGACT
GGAACCAACACGCCTAGTATAGATCAATTCCATTATTTTACCTCGTAATGCATCAACCATTTGAGTTATTGGC
AGCTCGTTCACCTCCGCTACCCAATCATAAAAAAGCTGGCTGATGTTGGATGTCAAGTGGCCATACCTTGCCC
CTCCAAAAAATGCATTTGCCCAGTGTTCAGGCTCACTTCTATTGACCCAATTGTAAACTTCGGGTGAGATAGC
TTTCAAATTCTCAACACACCGGTCGAAAGACTCACGCTTTGAGGCACAAGCGGCAGCGTATAAATCTTGGAT
CATAAGGCGCCTGGCTTCATGTGAAAACTGCCCTTTTAAATCATTATTAAGTTTTTCAGCAAGATAACGCAGA
CAAAAGCCATGGTAATACTGCTCACCAAACACATTTTGCAATGATTCTCTTATACCTCTTTGGGAATCAGAAA
CAAAAGTTATTGGACAGGATGTTGGGACAACAGATTTAAGCTCTGAAAGAAACCAATGCCAGTTGTCGCCAG
TCTCTTCATCTATAACTGCGAAGGCCACTGGAAAAACGCCATCATTTCCATCTACACCTACGGCCGCCAAAAG
TGTTCCTTGATACTTTGCATAGAGAAGAATGCTGTCCAGGAAAAGAAGAGGGCGGCAACCTTGTTGAAAACC
AGATATTGAGGCATGAAAGGCAATAAACAGACGATGGAAACTCGAGTCCTCCTTAGTAGCAATTGTAGCAA
CACTACCAGGATTAGTTTCTCTAATTTTCTCACAGAATAAGGGTAATTGACTGTATGCCTCTTTGAAAGAACCC
TGAAGCTGCTCCCTTGCCTTCTCTTTTGCACGCCTTGCCTGAGAATAGTTCAATTGAATGCCATATTCACGCTC
AATATCTTTTGCTATATCCTTGGGCTTGTAGTTCGGAGCGACCTTCAACTTTTCCTTTATTATACTACCCATCCA
TCCTCTCGTTGATCGGTACCCAGCTTTAACAGCAGCTCCATCACATGTATGCTTCGCATTCATTTTCTTAATGC
ATATTAGCTGAGTCGTAGGCAACCTTGACGCATATATGCACCAAGAACAACCTTCAGCCTTGCATTTAGCGGT
TACCCTGCGACTATCATTTTTCTTATACCTATAAGTAAATCCATGGGCAATTGAGTATTTATGCAGAGCTTCAC
GAAATTCAGCAAAACTATTGAACCTTTGACCCGCACCAGTTATTGTGTTCTCCCATTGCGAAGCTGCTCTGCG
ATGCTTCTCCATGTTCTCCTCATTGTTGCCGGGATAGGAACACAGGGGAGTTGTGTCAATGGAAGCATTAGCA
CCAAGCAGATCATCACAATGGATCATATCCACAGGGGTGGGAGGAGTAAGCGCTGCCTCTGACGCAGTTGTC
GACCTACTGGCAGAGACATTGGGCACATTTCTCACAACAGCTTCATCATAAATTACAAAAATCTCAACTTGAT
CAGCATCCTTGAAGAAATTAATCATACGTTTGAGGTCCTTATCTTTAGATATAGTAATGAGAGTCTTCTTATTC
CCAGGAAGAAAATATTTTATTGTCATTCCTTCAGTAGCAAACTGAAAGTTCTCTGCTACTTCTTTCTTAAAGTC
AGCCAAAACTGTCTGGTCATCAATGTCCAAAGCATAAGCCTCTCCACCTGTATACGTCAAAGTTCCATCCTCA
TTGTTTGTCACAAACTCTCCTCCAGATTGACATATTGCAATTATTTCTTAGAAGCCATCACTAGGCCAGCACA
AAATCCTCAACCAATTCAAAAACTTTCTGATGATGTAGGAAGA
>TCONS_00078640
CCTCCATTATCACTTCGGAGACGCTTTAGCTTTCGACCCGTCTCCCTTTCTACTAGAGCATGAAACTTCTGGAA
AACTTGAAACACCTTTGGCATAAGAAATGAGTGATTTCTTGGCAAGAATCTGCAATCCCTTCTCGCTCATATG
ACCCATTCTTTTGTGCCACAAATCTGCAGAAATCTCATCTTGTGCCGCGTTCAATTTACCTTGGCATATTTCTGC
ATTTGTCATGTACAACGTGCCACGAG
>TCONS_00078745
TAAAATGCAAGTAAAAACTAGAAACTCCATGAAAGCTCTGTGATTATCTAGGCTGGAACAAAGGTGATACTC
TCCGTCAGAACAACATCCTGCATCTGCTCCACTGTCAGCACTGAAGCTTCAGTGTCAAAGTTGGCCTTCTCCA
ACTGCTGGTGAATCTTCTCAAGTATTGCAGATTGATCATCCATGTGAAAGTTGCGCATGTACTTCAGCATCTCC
AGTTGCTGCTCTTGTAAATTTTGTTGCATCTTTAATCTTTCAGCAAACCCGTCCGGACCAAGCTCGGCCTCTTC
ACAAGCCATTTCTTCTTTTGCAACAAATTCATAAAAACGAATCATCAGGTCTTGATCACTCTCATAGGTCATGT
TTATTTTCCCCAAGCAAGCAAGACCAATTCTTGGATCAATTCCCATCTCGGAAAGTATCTCCTCTTGGATGGC
AGTGGTAACAGCAACAGCCTCCTGCTTGTCGCTGACAGCATCAGCAATCCTTTTCTTGACATCAGGTTGGGAG
GTGAGGAACGCGAAGCGATCAAAAAGATGAAGCAATTGCTCCTTCGATAGCATTTCTGAGGCTGCCATTTTC

GTCCGCCGATTGCTAGAAAACAGCAGACTAAAATTGGAATGTATAACTAAGTGCAGTGACTATTTCCAGGGC
GACGACGACGTTTCGTTTGCTTGATATTAGCCTTTTGCCTCTTCCCGTGTGTGGCCAAGG
>TCONS_00078797
CAAGAGTTGCAGTTCACACACGCCCGGTGTGTAACTGAACTGTTTGGCTTGGAACAACTTGTAGCACTCCAGG
TTGCTTTGAGAGCTCAGAAACCTGCTCAGCAGTCAGCTTTGCTGAGAATCCACTAGCTGCATGCTTGTAACTG
TATATCAACGCCTCTTTTGCAGCCTCCTCACTGCCAAGAACAGAAGTTAGGGTTTTGATATGATAGTCCTCGG
CTTCTAGATCTTCAGGTTTCTCAGTGTATACTATATGAACTTTGGCTTCAATGGAGGGTTGTGAATCTGCCATT
GTTACGATAGCTGCAATCAACAAGAAAAGCAAAAGAATTGAAGAAAGTATTTGATCTTTCTGCAT
>TCONS_00079069
AAAAACCTTGTACAAATTCCATAACCTAGGCACTCTCAATACGAATGATCTTCAATAAATCCATTGGTTTTT
AGAAGTTAATTAAAAGGAACATCAGCTGCATGAACAAGATATGTCACTGCCAAAGCCAACAACATTAATAC
ACATGCAATCCCCTGGTCAATTGTTGTACCGTCGCTGGTCGGAGCCGGTGTCGGAGCAAAACCTTCTTGTCCC
TGAACAGCAGGCAAAATGATCGAGAGAACAAATACTAAAACGAAACCAAACATCAATTTA
>TCONS_00079154
AGTGCAGGTACTCACAGCCTTCTCGCGAGCCCCGGGTTCGATATGGACTCCACGTCGTGATTGCATCAAAGTT
TCATCAGCTTTCTGAGGATTGAGTCTAAGCTTAGACAAGACTGTTTTGTTGAGCAAAAACGCCATTCTTTCAC
GAATCAAGACAGAGGCAGAGAGCAAACGCAGAAGAAGAAAATGATGATTCGAGTCTCCGGAGACAGAACC
AAACAGAAAATTGCTAGTGTATATTTTTTGTGAAATGGGGTTTTGGAGGAGATGTGATTTACAGAGAACTCCC
TAATAACCTAAGAGGGTCACTAACCCCCCTAATGTTTCAATTTGTACAGACAAATCCTGAATAAGATATAATG
AAACGTA
>TCONS_00079157
ATTAGGGAGTTCTCTGTAAATCACATCTCCTCCAAAACCCCATTTCACAAAAAATATACACTAGCAATTTTCT
GTTTGGTTCTGTCTCCGGAGACTCGAATCATCATTTTCTTCTTCTGCGTTTGCTCTCTGCCTCTGTCTTGATTCGT
GAAAGAATGGCGTTTTTGCTCAACAAAACAGTCTTGTCTAAGCTTAGACTCAATCCTCAGAAAGCTGATGAA
ACTTTGATGCAATCACGACGTGGAGTCCATATCGAACCCGGGGCTCGCGAGAAGGCTGTGAGTACCTGCACT
TTCCCCTCTTTTTGTTTCTCGAATTGCT
>TCONS_00079253
CTATTTCAGCACGAGCAGCTACAAAAGCTCTTGACCCCATGAAATGATTAGATTTCAGCTTTGTTCGATTGAC
CTTGTTTATCTCGAATCGTCTCTTATGCTCTGGATTCGCCCACATGTCACAAAGCCTGTTCCAATTTTCCGGGGT
CAACTCTGGCACTTCAATTTTACGAGCCTCTTCCTTGGAGCTTGCACTTTCAAATAGCTTTTTTAATTTGTAGCG
CCATTGTCGGCTTCTATTTTTCAGAATATCCTCACAGCTATCTTTGGTGTAATGCTCGTCCAAGTTTATTTCAAA
CTTCT
>TCONS_00079301
CAGTACAAAATGGTGAACATGAGCATAAGCTGATAATAGAACTTGCAACAGAAGAAATATACTAATACTAG
AGTTATAACTAGCTATAACTATGTATGAACACTTGATTAGCTATATGAATTTTCCCAGTAGCTTTCACTTGCTA
GATCAGCATTGGAGCAAAAGAACTATGTAGCACTCTAAGAGCTAGCTTGCTGCTCAGCATCATC
>TCONS_00079350
AATAGATTGTAATGGCCTTAAAATATCGTGGAGAGCAAGTACTAGGTGGTATCTATACTGGGATTAATACAA
AGCAGATATTCATGACGAGTAAAATGGTAGAAGAGTACAGTGGGCTTAAGCTGCAGCCACATAAGGCCATT
GTTGGAGCTAATGCATTTTCTCATGAGAGTGGCATCCATCAGGTTTTGTGTTATTCTTCTCTAA
>TCONS_00079414

AAGAAGATCTTGAATAGAGAGTTTTACCCGGGGACATGGTTCTTCTATACAACTCCAGACTAAAGTTGTTCGC
GGGCAAACCTAAGTCCAAATGATCCAGATCATTCAAGGTGGTGAATGTCTACCCCTTTGGTTCTATAGAATTA
GGAGTTGATGACGGGAGTAGAACATTTAAAGTCAATGCCAAAGAGTCATGCATTATTTGAGCCACGTGGATG
AAGGAAGATTGGTATTGGAGATTGAGTTGGGGGACCACAATTGGTACCTGAAAGGTGTGTTACGTCGTTCCA
CGAAGTTAAATATGACGCTTTTGTTTCTGTTTGTTCTGTTGAGACTGTGCCGTAAGAATAGAGTGCATAGATTC
AGGAAAGTTTCTGCATTGCGATATTCAGGGGAATACATCCTCCCGGTGCATCATTTGGTGAATAGGGGGTAGG
GTAAAAGAGGAAGGGGATCCCTGAGTTGGTAAAGGGAAAACGAATGGTAGTTCAATTGCTTTTGAATTCCAT
AACACCTACTAGTCTATCCCCTTTTAAGCTCGGAAAACAAAGACCAAATACTTAAGTTTTGTATTATCAAGTA
TTAGGAATTTACTTCACTACCTTTTCTCATTGGATGATCAAAGAGAAAAGGAAGAAATCCACTTAAAGAGGA
ACCTTGAGTGTCATTCAAGAATTATTTCAGATTCGTCGAGTCAGGACTTAGCTCGATGCCTTTTAATCTTGAAG
CCTTCCATATGAAAACACCAATCAGAGTGTCCCACTTTCATCACTGTTCAAGT
>TCONS_00079450
GACTCCTTGTCTACCTGACATCATTTTAAAATGGTGTCGTCAAAGGCGAAGTCCAAAGCATCAATGTGGGCAA
TGTGGGCTTTATCCGCAAAGCGAAATTAAAGTGCAAGCTTTAGTGGAGAAACCACCTGCCAAACACGGCGAA
GACGCTTCTTGGCGTTAGCTTTACGAGTAGAAGTGAGCTTAATTCTCTTCCACAGAAGGCCGCCGGAATCGTG
>TCONS_00079752
TAGCTTCACATCATTCACCTTCATCATGTCCTTGATGATAACATCTTGAAGACCTCAAAATTCTTCATGCTAGT
ATGCACTTCTAACTTCTCATTCCCTGTATGCACTATAAACATCTTCAATCTCCATCTCGGATTCATCTTCCAGA
AGATCTCCCTCCAAACTACCCATATTATGCTCGTTATTTCCACTTGAACACGTTCATCTTCTGACTCATTCTCA
AGCAAATCATCATCCACGAATTCCTGCAAATCTTCAATAGTATCCATTGTTTCCTTCTTCTTTCTGTATCCAAC
AAAGCTTAGAACCTGTTCTCTCCAAACAGTCAAAGGGTGCTTCTCAATCAACTCAGAACCTTCATAAGCACTA
TTAAGAAATACATGAAACCTCTTACCCCTTTCTGAAACATAAAAGATACCAAAATGTTTAAGCAACAGCCTC
ATCAGCTTCTGAGGCATTACGAACTCCATCCTAAAATGTGTCAGATAATCTGTCACCAACCTCTTTTCGATTGT
AAAGCTCAGCAGCTCATGCATCACTGCAACCGCCCTCTTATTGAACTCCAGTGTTCCAGCCTTTAGTTCCCTG
GCATCTGCATAAGGCGACAAGTATTCCCGCTTCTGAAAATTCTCTATCTTCCCCTTATATCTAGACAAAATTTG
TTTATAATCAGGTGGGAATTTCATTGGGAAAGACAAGGAAAGAAGACCAGCTACGTGATCATCTGGGACCTG
CATACCACCCAGAGCCTTCCTTTCTAACTCTGTCATAGCCCAACTCGATCTCCAATTCACAAGCTCTAAATACT
CACTTTCATCATAAGGTTTAAATGGATAAACAACTCTAAAGTAATCAGGAAATCTGTGCACCCAATGGTTCCT
AAAATCCACTGGCAACCCCAAATCTCTTCTGAAATGAACAATCTTATCTAAGGCAAGGCGCTTGTCCACGGA
CATCATCAGCATCCTAGTTACAAGCTCTGCAGCTTTATCACTCTGCTGTTCAATAATAGCCTCCTCTTCTTGGA
CTAAATCTTCAGCCTCCTTGGTCATACCCACACCATAAAATCCCTCTATGATCCTTGTACAGTTCAAAGAGCTTG
GGGGACTTCCGCAGAAAAGCAGAGATTTTATGGGGCTTTGGGAGGTTTATTTGCCTCCTATACTGATCAAGTT
CCCTAATTGATATAATTTTTTGAGGTTCCTGTTTGAGGACTTCAAATATTAATAGGACTTTGGAGAGGATCTTG
TGCTTTTCTGTGGCTATTTCAAGGTCATGCACCCTCTTCTTCTTGCTTCTGTCTTGGACTCTTTTACTGGTGGTCA
TTGATCGGTGCCATTGCAAGATTGATGAAGAATCTATGAACAAGAATGCAATTGATGCATTTAGTTCAGCAG
AAAAACATCCACGTCTACCAATAAGGTTGAGCATTGTGCCAAACCAAAATAATCAG
>TCONS_00079804
TTACAAATTAAAAGAGAAAAGAAAATCTAAGGCTGCTTATGCCTCTGGGTATAGATATAATCAAGGTGAGCC
TCTAGTGTTCTTCTCATCAAACATTCTTCTTCCCCTAGACCTTCACATTTATCGTCCACTTCTACTTTCACTGCTG
CCTTATCCTTACTATGCTCCATTGGAGTAACGTCGTGATAAGGCGTACTCATCGGCCGGGAAGAAGCGCAAG
>TCONS_00079845

TATTGGTATAAACGACCCTTCCCTCGCGTTCGGCTATAGCAAGAGCCCCCGAATCTAGAGCTGCTTGTCGTTC
CAACCCAGTTCCAACAATGCATTTCTCGGAGCGAGAAAGAGGAACTGCTTGACGTTGCATATTAGAACTCAT
TAAAGCTCGATTCGCATCATTATGTTCGATAAAAGGAATGAGGGAAGCTCCAATAGAAAAATATTGAAAAG
GAAAAATACTTCGAAGATGAACCTGTTCTTCCTGAATATCCCGATTTAAGGCTAAAGAATTTCCTGCCGCTAC
CATATAGTATTCATCTCTACCTGGTGATAAATAAAGCATCCGTACCCCGGTTGACCTCTCAGAAATTTCATAA
AAAG
>TCONS_00079863
GCCGCTTTTTACTAATATAGTCCCGAGTTGGTAATTAGAGTTAGAAGCTTCACAGCTTCCTCCACTCACCTCCA
CGGGCGAATATAGTCTACTAGACTCTTTCTTGGCTAGTCTAGTAGACTATATTCTAGGTCATTCGGAAGGGCTC
CGTATAGTTTTGGGGTGTAACGTTGTGGTTGTTCCCTCTCCTTTCAGTCGAGTGACTTCGTACCTATCCTTACCG
AGAAAAACGAGTTCCAGAGGCATCTTCCATTCATATCGATTTGGGTTTTTCCGCACCATATTTTGATCTGCCTC
TTCTTCGATCCGGAATTCCCAGCAAATCCTTGACTCCTCGAATACAATGGAATCTCACACCTGGCGAATCTTT
CACTCTACCTCC
>TCONS_00079864
GCCGCTTTTTACTAATATAGTCCCGAGTTGGTAATTAGAGTTAGAAGCTTCACAGCTTCCTCCACTCACCTCCA
CGGGCGAATATAGTCTACTAGACTCTTTCTTGGCTAGTCTAGTAGACTATATTCTAGGTCATTCGGAAGGGCTC
CGTATAGTTTTGGGGTGTAACGTTGTGGTTGTTCCCTCTCCTTTCAGTCGAGTGACTTCGTACCTATCCTTACCG
AGAAAAACGAGTTCCAGAGGCATCTTCCATTCATATCGATTTGGGTTTTTCCGCACCATATTTTGATCTGCCTC
TTCTTCGATCCGGAATTCCCAGCAAATCCTTGACTCCTCGAATACAATGGAATCTCACACCTGGCGAATCTTT
CACTCTACCTCC
>TCONS_00079865
GCCGCTTTTTACTAATATAGTCCCGAGTTGGTAATTAGAGTTAGAAGCTTCACAGCTTCCTCCACTCACCTCCA
CGGGCGAATATAGTCTACTAGACTCTTTCTTGGCTAGTCTAGTAGACTATATTCTAGGTCATTCGGAAGGGCTC
CGTATAGTTTTTGGGTGTAACGTTGTGGTTGTTCCCTCTCCTTTCAGTCGAGTGACTTCGTACCTATCCTTACCG
AGAAAAACGAGTTCCAGAGGCATCTTCCATTCATATCGATTTGGGTTTTTCCGCACCATATTTTGATCTGCCTC
TTCTTCGATCCGGAATTCCCAGCAAATCCTTGACTCCTCGAATACAATGGAATCTCACACCTGGCGAATCTTT
CACTCTACCTCC
>TCONS_00079866
GCCGCTTTTTACTAATATAGTCCCGAGTTGGTAATTAGAGTTAGAAGCTTCACAGCTTCCTCCACTCACCTCCA
CGGGCGAATATAGTCTACTAGACTCTTTCTTGGCTAGTCTAGTAGACTATATTCTAGGTCATTCGGAAGGGCTC
CGTATAGTTTTTGGGTGTAACGTTGTGGTTGTTCCCTCTCCTTTCAGTCGAGTGACTTCGTACCTATCCTTACCG
AGAAAAACGAGTTCCAGAGGCATCTTCCATTCATATCGATTTGGGTTTTTCCGCACCATATTTTGATCTGCCTC
TTCTTCGATCCGGAATTCCCAGCAAATCCTTGACTCCTCGAATACAATGGAATTTCACACCTGGCGAATCTTTC
ACTCTACCTCC
>TCONS_00079910
AAGTTCAGTTTCTGAAAGATCTAATACCGCCAAGAAACTACAAGTCAGCAGCAATAGAAATTTACATCTACT
TAATCTTCTTTTTAGGCCTCAACTGGTTGCTGTGTCCACACTTCTTCTTGCGGCAGTTGACAGCACGAGGATGC
AATCGAGCATAGCACTTGCGGCATATCATCTTGTCCTGATTGTATTTCCTAGCCAAAGCCATCAACGATGGCT
CAATGATTCCACCACGAAGCCTCAACACCAAATGTAGAGTCGATTCTTTTTGGATGTTGTAATCGGCCAAGGT
GCGGCCATCTTCGAGCTGCTTACCAGCAAAAATCAGCCTTTGTTGGTCCGGTGGAATTCCTTCCTTGTCTTGTA
TTTTGGCTTTAACATTGTCGATTGTATCACTGGATTCAACTTCGAGAGTTATGGTTTTACCAGTGAGAGTTTTCA
CGAATATCTGCATTTTCGTTACGAGCTTCGCCTTCCTTCTTCCTTCAGCTCCTCGCG

>TCONS_00080082
ATATGTTGTGATACTTGATGTGGGTTGTGTTCCCTTATTTGTTGATGATGGTGAGGCTAGTGAGGTACATGATT
GAGTGAGGCCAAGGGCCTGGTTGTGAGGATATTAATACCATAGCGCGTGAGTTGTCCGCGTAGCACGTGAGT
TGACCGTGCGGGTCCAGGTATTGATACCATAGCGCGTGAGTTGTCCGCGTTGCCCGTGAGTTGAC
>TCONS_00080083
ATATGTTGTGATACTTGATGTGGGTTGTGTTCCCTTATTTGTTGATGATGGTGAGGCTAGTGAGGTACATGATT
GAGTGAGGCCAAGGGCCTGGTTGTGAGGATATTAATACCATAGCGCGTGAGTTGTCCGCGTAGCACGTGAGT
TGACCGTGCGGGTCCAGGTATTGATACCATAGCGCGTGAGTTGTCCGCGTTGCCCGTGAGTTGAC
>TCONS_00080090
AAAAACATTAATCCTTCCATCTTCAAAGATCTTGCACGCTCAAACCTGGAGGAAGTGACTGCTTCAACTTGTC
GGCCTTCTCAAAGTCGGACACACAGAGTGTGTAGAGGTACTTGGAGCAGCGAACCTTGAATTTGACCATATC
CTTATTCTTCTTGATCTTGACAGATCGTGCATCCTTCCTTCTTGCTGTGAGAAGGAAATCCTTGATTTCGTGGAT
TTGCTTCGGCATGATAGGGTCTTCGGGTTAGGGTTTGTTGTTGCTGTGCTGCTGCTGCTGCT
>TCONS_00080110
CTATTCTCTTTTCAGAAGGGAGGGAAGGGGGATGGAACAACAGGTATTAAGAGGAGGGAGACTCGTTTTAAA
GATATGTCCTATACTACCACAGCCGCCAAAACTTTAGAATGTGAAGCTTGGTTCAACATCCATCGCCCAGGC
AAACTTTGCTAGAAGAGACTTTTTGAACATCTTTTCTATGGGAACCTCATGGCGCTTACACCATGCCACTGTG
ATCCTAGGATCCAGATAGTTGATTTTTGATGTGCCCAAAGCCACA
>TCONS_00080310
ATCATACTCCTCTTTTCCACTGCTTATGTTAATAAATGAATAGGAACCGAAAATGTTTCCTTTAAGAAATCTCT
TCTCATATATTGCTTCTTTCACATTCGGACCATCCATAATTTTGTTTTCATAGAACTCTCTATTTGGAAACAAAC
TTATTGCAGGATGCATCCTATATTGAACATTAAGGAGGTGCTTCGTGTGCCCAATCATTACCAGCCTCTCAAA
TAAGCTCCTTCCAAAGTCAGCCTTCTCAGAGATCTTGCTCTGAACCATTGCAGGCAATGGTTTCTCATCCCCA
ATAAGTATGGCATGACGTATACCAG
>TCONS_00080400
CTTCAGCAGAACCCTGCCTCCTCTTTCGTGGAGGGCGCTTATTAGAACCTCCTTCATTAACTGGGCAGCTCTCT
ATTATCCTTTGTCTAACATCATAGGTGCGTAGAAGCACTTCAAATGTTCTTACATCAGTAAAATGTGATTTGAG
TGCTTCACAAGAGCGTTGTACTTGCTCAATGCATGGAGAAAAGGAGTACAATACCCCATCTGGTTTCAACATT
TTTCCAGCAGAAGGAATGAC
>TCONS_00080405
TTCCTCTTCACTTTCTTGATCATCTTCCTCAATTGACTCCACAAGACTCCAATGACTGTGTTGGAACTTCAAAA
ATAGAGAAGGATATGCTTCCGGGAGCTATGAAAAGGCAAGACTACGTTTTGCTCATGCAGAAACTCTACTTC
CATTTTCATGCTTGCTTGGGCTCTTTCTTGAAGAATCTGGTGAGTGTTTATCAATAGGATTATTTTAGGCTTCTA
CTGCCTCCAGTTTCTTAATCAATTTTATACACTGAATGTTGCAGAATTTGAACTAATACAAAGAGAGGAGACG
TTGCA
>TCONS_00080407
GCAAAACGTAGTCTTGCCTTTTCATAGCTCCCGGAAGCATATCCTTCTAAAGGAGGAGAATACACATAAAAC
GAAGAACACCAGATATCAAATTTCCATCATTTAAAGGATTAGCTTCCATAGAAGATATGTACAGGCAAAAAG
ATTTCTAGAGAGATGAATGCGAGCAATAAAGGAAGGTACTAGTTACTAACAAATAACAATACCAAGTAATT
GGTATAATTAAACAAAATAGTAATTGGTATACTAAAGGCTCCACTTTCTAGCCTTATACAACTATCCTCTAGT
CAGTAGTTGAAGGGATAGCAGAATAACACAGGCAATAGAGTCATGGAATAAGAACAGAATCGGTAAAATTA
ACAGGTTATATGGGAAATATAACATATAATGAAGTTGACTTGTCCTTTCTTTAATAAGGAGAGAAATCCATGG

CCTCACACACACATCTACCTTCAAATGTATTTCCCCAAATGGTTTCCACTTGTTGGATCCATACGGTCAATTGT
TAGCCTTCCTCATATACCTCCAAGACTCATCCCCGATAACCTTTAACCCCTTTTCTTTCCCTGGACCCATAGCA
ATTTTATCCGGTCCATGACCCTTATAATAGGACAACACCCCATCTTCAAGCACGAACCATCTCGCACGCCATC
CTTTCCCATAATTCACCCATTTATATAACACTCCAGCTACACTAACACTTCCATTTCCACACTAACACTTCCAT
>TCONS_00080409
CAACATGACCAAAAATAATTGTTTCTTATTCCTGCTCATCCTCGGATTCTGTGTTCATTTAACTAAATCGAATC
CACATCCCCAATTCCCATGTCAGCCACCTCGTCACAAATATCCCTTCTGCAACAAAAATCTCCCTATCTCCAC
TAGAGTTCAGTCCCTTATCTCCTTACTCACCATTGATGAGAAGATCTTACACCTCTCTGATAA
>TCONS_00080513
CCCAATCATGACAAGCAGCAAAAAAATGATCAGCTCCTCTGCTTCTATTCCAGAATTGATATTTTCCAGAAAT
TCTTCCAATGTAATTGCTGAGATGATTTTCCAATTCTTTCTGATGAGTAAAATTTTGTTTTGAAAGAGCTTCTCT
TAGCCTCAGAGAACTAAAAGGCAAATAAAATAAGTGAGCCTTTTTGGGATCTTTCACAAGAAATTGTTTGTTT
TTCTCCATCAGTTTCATAAACCCACCCTTCAGAAGCGTAAATACCTCGTTGATATGGCTGATGGAATATTGGTT
TTTTCCCTTCCTTATAAACATAAACTTTAAGGAGGCGTTCCATTAGCTCATAGCTCCTCGTAAACTTGGAATAA
TTTCGAAAGATAGATGCATGAACTTCTGGAACGTTTACAATTATCGGAGAATTTTCAATCTGAAACTTTGCATT
TCGGAGTTCCAGTTCACGTGCAGAATGCCATTGAGATCTCACTAAACTAACAGAATTCTGCAACACTAAAGA
GTTCATGTAGGAGATTGATGTCGGTC
>TCONS_00080581
AGCTGTTGCTGTGGCATTCTCTTCTCGACCTCTGCCCTGAATCCTTCTACACCAAGTTCATCGATTAACCTCATC
ATTCTACATTTCTGTCTGTTCCCTCTGAAACCAAGATCTCTAAAAGCTTCTAGTATTGCTTTGCAAACTGGAAC
AACATCATCGGCTGGAACCCATGCATCAAGAGGAATTGCCTCATCACATCTTTTTGCGCTGAAGAACCCACC
CACAAGCAGGTTGAATCCAAATCGTCCATCTTTC
>TCONS_00080642
CTGAACAATCTCTAGCAGAACTTGTTCATAACACACCAAGATTACATGATATTTATAACAAACATATCAAGA
GTGCTCCCGAGCAAAATTCTATCACTAACATATGCCAAATTTTCTCAGAAAAAATAGAACCCATAAAATCAT
ACAGAAAGTCGCTCCTAATCGAAAAGACTGAATCCCATGTCGTCATCGCTTTCTTCCTTAGGCTCTTCCTTCTT
TTCCTCAGCAGCTGGAGCGGCCGCAGCAGCA
>TCONS_00080658
CCGCGGCGGCGCCTCGTTAAATTTCAAGGTCATGAATTAAGACCAATTTTAGGACAATTTGGTCCTTTGGAGC
TTTCCTACTCCACATAAAAATTCGGTATCTAATTTGACGATTCCTCAAATTTGGCTCTGGAAATTTTTGTAAGC
TCCACCTGGTGACTGTTCAGCCTTGACCTAGTAAACCTAGTTGCAGGGTGAGCATAGTTTGGTAAAAATATCA
TTTTCCGGTGGTTCCTCTCCGTCGACCAGCTGGCGTTGATTTTGGCCGCCTTTATTCCGTCA
>TCONS_00080709
GGGAACAAATATCTATTTCCCGACATGGTGAGGCTCAAGATCTTTGAGGAGGCAAATAGCAAATTCCAAAAG
ATCAAGGGGCTCTTGGTGTGTTTTCATTCAACTTCTCATACACAAGGGTCCAAGTCATCCAGTTTCCCTGTGCA
GTCAAAGTAGCTGTTATGGACTTGTATAATTCATTCATATAACCTTCAACGAAATTGAAGGTGATTGATTTTTT
TGCATCATCTATGTGTAGGACCTGTTTTGCATGCCTCTCTTTTCCTCCCAACTGACCCTCATGTAACGTGAAATT
TTTTATCTTATCAGGAGACATGGTGGAGGTTTGGTGTGGATTTGATTTGAAGTGTTCATGAAGCAAATCTCCAG
CACACTTCATCTCTATTTGAGAGATCAACTTGCCTTTGAGACCCATTTGTTAGCAGTGTGAGATTTTCTTACAC
GAGTTTGTGACCCTCATGTAACGTGAAATTTTTTATCTTATCAGGAGACATGGTGGAGGTTTGGTGTGGATTTG
ATTTGAAGTGTTCATGAAGCAAATCTCCAGCACACTTCATCTCTATTTGAGAGATCAACTTGCCTTTGAGACC
CATTTGTTAGCAGTGTGAGATTTTCTTACACGAGTTTGCTGATTTAATTTA

>TCONS_00080792
GTCATGCCAATTCTAGGAGTAAGGCCAGTGGCTCCAGGAGTCGCTGAAGGAGTTAAGAGTGGGTTTGGCGTT
TGAACTTCCCTTTTTCTTAGGAGTGACACCAGAAAAATCTGAGGGGTGCAGCATAGGATTATCCCCTCCAAGT
AATGGTGTCTGAGACTGAGTCAATCTTCGTTGATTTCTGCTTCCATCATAATAGCATCTTGCTTAGTTGAAGG
GGTTCTTTGAGGTGTTCGCATAGGAGTCATTGCATGATGTGGTGTCTGGGCATAATTTGCAAGAAGAGCACGT
GTTGCAGCATTCCCTTCGGAGAGTTCATCACCCCCTAGTAGATCACTGGCCATACCTATCTTTGCTATGGCTTC
CAATTCATGGTCTGGAATCTGTGGTGCAGGAAGATTGAGCTTCAACCTCTTCCTCACTGCTTCTGGGTCATTAA
GTTTATTTGCATGCAATATGGTTGTAGGGGCATCCTGCCTTTCTGCAATTTTATTCCTAGCAATATCCTGCTTTC
TTAAACGAGCTTCCTTATCGACTCTCCTTTCACCTTCTAGTTCTTCAATGGTAGTTGGAAACTTAGGTTGCTCTA
CTGGACGGTCTTCCTCAGCGACATCATAGAAACCTGGAGGAGGCTTCTTTTCAAAGGGTATTTCAGCATTGTA
ATCAATTTCTCTCCTTTTTCTCTTCCTTTGACGGGCATCTATTCCAGCAACCTTCAGTTCTCTCCTTTTCTGTAAG
GAAGCAAACCTTCGAGCCTCTTCAAGCTGCTTTTCTCTAGCTTTCCTTTTAGCCTTCTTACCTCTTGTGTTGGCC
TACCGAGCCCGTGCTTCAGACAGCATTTCTTTCTCATCCTCGTCCATATCAACAGGATCAGGACGAGCAGGCT
TTGATTCTGGATTAGGATCAATCTATCCAGGCTTCAATTTCTTGGATCATCATTAGGGTCATAGTTTTCATCCT
TAGCACATGCTGGATCGAGAAGCGTTTCATAGCGTTCAAGGCACTGGGATGGTGTAAGACCAACAATTGGCG
C
>TCONS_00080801
CTTTCATAAGCTTATCAGAAGTGAGTAGAGTTTTTCCGTAAGAACCCTCTTTCCTTTAGAGGCTGGTGATTGGG
TGAGAGCCTTTTACAGGCGTTGGCTCTCACTCTGACCAGTAACCACACACTCTTCTTTGCAATGGCTGTCTTCT
GTCAGTCTCCCCTCAATCTCAGCCTCATCACCTCCTCACGCAATCTCAATCCTAATCCCTCCTTTAGACTCAAT
CTCTCCTCTCACACTCGCACCCTCGTCACTGAGCCCCCACCAGTTTTCACCTGCGTCAAGTCATTTGCTCCCGC
CACAATTGCCAATCTCGGCCCCGGTTTCGACTTCCTTGGCTGCGCTGTCGACGGCATCGGCGACTACGTCACT
CTCCGCGTCGACCCCAACGTTCACCCTGGCGAGGTTTCTATTTCTGACATCTCCGGCGCCGGCAAGAAGCTCA
ACAAAAACCCCCTCTGGAATTTTGCCGGTATCGCCGCC
>TCONS_00080984
GAAGGTTTAATAGATTATTACATGGGTAGCTAAGGAAATCTGCAGCAAGATATTGAATCTTTGCCTTTTAAAG
CTAAAATAGGCTGAGAGTGCAATTTTGGCATTAGACTGTTGCGGGGAGATATTAGGATCCCAACGCATCAGG
GTGAGTCCTTCAAAAACACCAGTGAGGCCACGACTTCCCCGTGCCATGATGCAGTAACCGATGCTATGGAAA
GAGTTCAGGAAAGCCCTCTTGGAAGACTTGAGGTCACCATGGGAAGGCGAAACATAATTCTGTTTCAGCAAT
TTCCATCTTTCTTTTTTTTTTTTTTCTGTTCTGCCAACACAGTAACTTTATTGGTACTGAACATGGCATTACCTTT
GCGATTAACGAACTACTTATCGAGAATATCCCTACCTTTAGTATTGTTAGCTTGATCAGTCATTTTCTTATGAA
TCTCATGCTGGTTGAATGCCGGTTCATAATTTGATCTTTCGTCTCTGGACGCAGCATTACATGTTTCCTTTTTCT
CTCAGTGCAGGGGAAAGAAACCTGCACCTGGTGTTTTACACCAAACTACATAAAGTTACCATGATTAAGTAT
AAATTGATTTGAGATAAAGTGATCAAAGTGGATGCGGAAGACATATGTCATGTATTCCTTCGTGTGGTAAATT
TTCTCCTAGTGCACTTCTGCAATCACATAATCAAATGTGATTGCGATCTTTAATAAGATGATAATATTGACTTA
GTTTGAGCTTGCTTATTATAGCGAGAGAATCAAAAGGGAAGAGGAGCAAGGAGAAGAAAGAGAGAGGACA
CTCAGATTCTTGACCACATTTCTATCTGTTTCTTTTTCTATAGTTGTAGTGTCCGGGCCAATAATTCGACTATTT
CATCAGGGGTTTCTTTTTCTATAGCTTCCTTTATATTCCACCAATTTCATTGTGTAATATAAATGTGAATTAAAG
GAAACTACTACTTTTGAATCTATTCTTTCTGTGCCTATATTAAAGAATCCATACTTGTCAGTCCGTCGACTCCCT
TCCCTCGGGAGTGCTGGCCCTTCCTCG
>TCONS_00081064

ACCTTGTTAATCACAAGAACAATCGGATTACGTTCTTGAATCGCATGCCGAATGGCCCTTTCTGTATTTACCAT
CACTCCTTCAACAGCATCAACAATCAAAACTGCACCATCAGAAAGTCTCAAAGCAGCTGTCATCTCATCCGA
GAAGTTGACATGACCAGGAGCATCCATAATGTTGCAGAGGTAAGACTTTGAATTGCTGTCTTCCAGGACGAG
TGACATAGGGACAGACTTAATTGATATCCTCCTTTCTTGCTCATCTATTCTTGTGTCTGTATACCTCATATGCTT
CTCACTATTCTGATCAAAAGTAGATATATGGTGGGTTTGCTCCACCAGCATATCCATAAACAAAGTCTTCCCG
TGATGCACGTGTCCCACTAAAGCAACATTTCGAACCAATGCTGGGTTCGACATCAGACCTAAGAGAAATTGA
GTCGAAACATAGGTGGACGAATCCTTTACCCCGAGTTCAAACTTGAGATTCTTCACCGGTTTAATTATCGGCA
TTTCAAGAGGCTGTTCATCTTCATCCATCACCAATGTCTCGACCTCTTCACCGTAAACCTCTTCGGCTGTTGGG
TAGTATTTCTTGTCTTCAGCAAGGACAACTTGGTTATCCATGTCAACATCCTCTTGAGTGGCCAGCCATCCATT
CGATGCACCAGGTTGCTCGCCATCAGATGCAGCCCTCTCATCATCAC
>TCONS_00081091
CAATATCGGAGTCAACGCCGGCGCCGGCAGAATCATTTCTCTTAGCAAAACGACCTTTAATTCTTGGCCTTGT
TTCAGCATAAGCTTTTCTTGAAGCATATCTTATAGTTTTCTCAAATTTCCGATTTTTCTTCTTTTCTCTGTATCTTA
AAACTCTTGCTTCTCTATTCATTCCAATTAATTTTTCAGCTTGAGCTGAATTTCCCATTTCAATATTGCTATTCA
CACTTCTAACAAAAGGGTATGATATTTCTGAAATTGAGCTCCCATCTGGTACAATTCCAACATCTAAAGACGA
TGATGACACTGGAAGTTTGGTAGTCATTGTGTTTGGAGAAATCCATGGATCAGAATTATAGTCTTCTTCATGGT
GTCCGAGACAAGCTGTAACTTTACTGAAGTCAGTGGCTACGGCCGTAGTCGT
>TCONS_00081217
CGCGGGTACAGATACTAGTAATGCAGAAAAGGAAAAGCATGTAACACTATCCTACCTAAATCTACACAATG
ACACCTTGGATCATTCCCGATTCAACCATACTAATTACATGTTTAAAGAAGAATATCAGCAGCACTCCAATCC
ACAAGGTGATCTATTCACACAACAGGCTATAAATAGTAACAGAGCATAACATCTATTTCCTCTGCTTTTTTGC
CGGATTCTCCAGTTGGGACTCAACTGTATTTGATAGCTGCTCACGGATATAAGCCATTGACCTCAGCATGGCA
GCTAAAGTATCTCTTGAAACCTGAAATTTCACCTCTGATTCTCCTGAAGTTGTTTCTGTATCTTGAAGCTTCAG
ATTGATGACAGCAACTCTATTAGCCGGGGTGGAAAGCTGCTCGCTTATATCAGCCATAGACTTCAACATGGG
CTCTAGTGTGACACGCGATAGCTGAAACTTAACCTCTGATTCTCGTGAAGGAGTCCTACTGTAATCTTGCAGC
TTCAGGTTGATGACAGCAACTCTATTCGCAGGTGTAGAAGTTTTATTCTCCATCACCCAAGTCAATGACTTGA
GGCAAGGAAGAATCACCGTGTTATCTGCATGACCATCATCACGGTGAAGAGACACAGGGGCCATGCGCTGG
GCATTTAAATCAGCAATAGTCCTAGCAGGAATACTAGTGTCATTAAGATTAAAGCGCTTTGTCATTTCATCTT
GCCGTGGCCACAATAATGGCGTCATTTCCGGAGCAGAGGCAGACGTCCTCCGATCATCATAGCCAACACCGC
TAACCTGTGAGCTAGGAGGTGAAACTTTAGCAACATTTCTTTTTGCAAGAGCCACCTTCTTTCCTCCTTCTTTA
AGAGCAGCTAATGCAGCATTATATGTTTCTTGAGCTACAGCCCCTTCTTCAGCGTATCTTATGGCTTCCCTGCA
GAGACTGTTGTATTTCTGTGCCATTGATTCGGTACCATGTAGTTGTACATGTTCATCTGATTCAGCACGGCATT
TTGCATTTATTGTCCACCGCTTCAAGATGTAACGAGATGGCAATGTAAGAACATTAGTTACTGTAAAAACTGT
AAGAATATGTCTACAGAGGATACCACTACATTCAAACATCTGGCAACTACAATTTGCTTTCAGTTCAGATATA
TTCAGCGAGACAATGTATGCTTTCTGGTCATCCTCAAATTTTGCAACCCTGAATGTGCTGAGAGCCCCATCTCC
ATCAATTCTGTTTGCAGTGTAGACAAATGTCTCCACTAACTCTTCTTGAAATTTTAAGAATATTTTCTTCGTGTA
TAAAGTTGCTGCCTGTTTCTCCATAGGAGATGGTGTCCTTAGTGGTGGAGTAGTGCATATTGTATCAAAATCG
GCTTCTATTTCCTTCTCAAATGAATTCTCCAAAGCTCTTTCATACTGCCTGAAGAACAATGGCAGTGTAATTTG
TTGATTCACATAGCCATCGAAAAATGAGCATTCATATTCCTGGTTAGGAGAGAGGGCTGCAAAGAAAGTATC
ACGAAAATAAGCCGGTACCCACTGCCGACGTGTATTATATATGGATTGAAGCCAATCATTCTTCTTCAGATCA
TACTTTTCAAGGATTGTCTCCCAATATGATTCAAATTCTTCAACTGTCTCCGTCAAATTGATACAATTATAGAG
TTCAACTTGAAAATTTGGGTACATGTGACATACATGAGCCATTCTGTCCTGGCCACCCCTTAATACATGCCAC

TTGTTGATACAATGCCGTGTTCCAGGAAAAACTTGAGCAACTGCAGATTGTATAACAGTGTCCTGATCAGTAA
TTATGGAGACAGGGGCACGGTCATTCATGGCAGCATGGAAGGTCTTGAATAGCCAGACAAATGTAGCCTCAG
ACTCATCTAGAAGAAGTGCACAACCAAACAAAATTGTCTGACCATGATGATTTACTCCAGTGAATGGAGCAA
ATGGGACTCTGCACTGATTAACTCTGTACATTGTGTCCAGTATAACGGCATCACCAAAATGACTATATGCATT
TCTTGACCTTGCATCAGCCCAAAATACATTAGCCATGCGATTATCTTCGTCCAATTGTATGGCATAGTAAAAT
CCAGGATTCTCAGCCTGCATTTTCTTGAAGTAATCAAGAAGATTCTGAGCATCTCTTCCTAATGTTCTTCGTTG
ATTGCATTGTCTAGGAGAAAAACTCTGAGGTACAGTATCCTTAACTGTTTGATCTGACTCTGCAGGACGAGTA
CTCTTAGCTCCACGGCTAATCTCTAAAGGGATGCGGTTACCATCTACAGAGACATACATTACACCACTAGGA
ACAATTCCTACACCCTGATTATTCTCAGGTCCATTCTTTTTTGCCACCTCAAAATGCCTGTGTGATGTGTGGTA
ATGCACCTTATTAGGATGCACAAGGGAGTGACTATGCTCCTTCATATATTTGGTCACAACCCACTTGTTTTGAC
CTTTTAACTCCACTCTAAGCATTGCATCACAGGATTCACCGTACACTGTTCTCAAACCATTACATAAGAAATC
AGTCTTCAGTCGATTAAACTGATAGACATGGGTGGTAAAACCCAAACGCCTAGCGTATTCATCATAGAAATT
CTTAGCCTGATCTTCAGAATGAAATACCATACCCTTTTGTGGCTCAACAACCTCATCCCGTTCAGGTGCATTTG
ATCTTCCATTAACATTTGCTTCTCCACTCTCGCTGGGTTCATCATCACCGTCATCAGTTATGCCACCTTGGTGTT
GCATATTCCCCTGTTCGACATCGATCACTTCAACATCCATCCCAGAACATGTAAACCACTTTAGAAAAACGCA
TCACATCTCTAGAGGTGCTCCAGCACATATCCCTATGATCGGAGGAGAGAGAATCAAGGCCAAACGGATCTC
AAACAATTTGCGTTATGAAAAACGGAAGTCCAAAGTTGTGTGAATTCTTCAGAAC
>TCONS_00081218
GTACAGATACTAGTAATGCAGAAAAGGAAAAGCATGTAACACTATCCTACCTAAATCTACACAATGACACCT
TGGATCATTCCCGATTCAACCATACTAATTACATGTTTAAAGAAGAATATCAGCAGCACTCCAATCCACAAG
GTGATCTATTCACACAACAGGCTATAAATAGTAACAGAGCATAACATCTATTTCCTCTGCTTTTTGCCGGATT
CTCCAGTTGGGACTCAACTGTATTTGATAGCTGCTCACGGATATAAGCCATTGACCTCAGCATGGCAGCTAAA
GTATCTCTTGAAACCTGAAATTTCACCTCTGATTCTCCTGAAGTTGTTTCTGTATCTTGAAGCTTCAGATTGATG
ACAGCAACTCTATTAGCCGGGGTGGAAAGCTGCTCGCTTATATCAGCCATAGACTTCAACATGGGCTCTAGTG
TGACACGCGATAGCTGAAACTTAACCTCTGATTCTCGTGAAGGAGTCCTACTGTAATCTTGCAGCTTCAGGTT
GATGACAGCAACTCTATTCGCAGGTGTAGAAGTTTTATTCTCCATCACCCAAGTCAATGACTTGAGGCAAGGA
AGAATCACCGTGTTATCTGCATGACCATCATCACGGTGAAGAGACACAGGGGCCATGCGCTGGGCATTTAAA
TCAGCAATAGTCCTAGCAGGAATACTAGTGTCATTAAGATTAAAGCGCTTTGTCATTTCATCTTGCCGTGGCC
ACAATAATGGCGTCATTTCCGGAGCAGAGGCAGACGTCCTCCGATCATCATAGCCAACACCGCTAACCTGTG
AGCTAGGAGGTGAAACTTTAGCAACATTTCTTTTTGCAAGAGCCACCTTCTTTCCTCCTTCTTTAAGAGCAGCT
AATGCAGCATTATATGTTTCTTGAGCTACAGCCCCTTCTTCAGCGTATCTTATGGCTTCCCTGCAGAGACTGTT
GTATTTCTGTGCCATTGATTCGGTACCATGTAGTTGTACATGTTCATCTGATTCAGCACGGCATTTTGCATTTAT
TGTCCACCGCTTCAAGATGTAACGAGATGGCAATGTAAGAACATTAGTTACTGTAAAAACTGTAAGAATATG
TCTACAGAGGATACCACTACATTCAAACATCTGGCAACTACAATTTGCTTTCAGTTCAGATATATTCAGCGAG
ACAATGTATGCTTTCTGGTCATCCTCAAATTTTGCAACCCTGAATGTGCTGAGAGCCCCATCTCCATCAATTCT
GTTTGCAGTGTAGACAAATGTCTCCACTAACTCTTCTTGAAATTTTAAGAATATTTTCTTCGTGTATAAAGTTG
CTGCCTGTTTCTCCATAGGAGATGGTGTCCTTAGTGGTGGAGTAGTGCATATTGTATCAAAATCGGCTTCTATT
TCCTTCTCAAATGAATTCTCCAAAGCTCTTTCATACTGCCTGAAGAACAATGGCAGTGTAATTTGTTGATTCAC
ATAGCCATCGAAAAATGAGCATTCATATTCCTGGTTAGGAGAGAGGGCTGCAAAGAAAGTATCACGAAAAT
AAGCCGGTACCCACTGCCGACGTGTATTATATATGGATTGAAGCCAATCATTCTTCTTCAGATCATACTTTTCA
AGGATTGTCTCCCAATATGATTCAAATTCTTCAACTGTCTCCGTCAAATTGATACAATTATAGAGTTCAACTTG
AAAATTTGGGTACATGTGACATACATGAGCCATTCTGTCCTGGCCACCCCTTAATACATGCCACTTGTTGATA

CAATGCCGTGTTCCAGGAAAAACTTGAGCAACTGCAGATTGTATAACAGTGTCCTGATCAGTAATTATGGAG
ACAGGGGCACGGTCATTCATGGCAGCATGGAAGGTCTTGAATAGCCAGACAAATGTAGCCTCAGACTCATCT
AGAAGAAGTGCACAACCAAACAAAATTGTCTGACCATGATGATTTACTCCAGTGAATGGAGCAAATGGGAC
TCTGCACTGATTAACTCTGTACATTGTGTCCAGTATAACGGCATCACCAAAATGACTATATGCATTTCTTGACC
TTGCATCAGCCCAAAATACATTAGCCATGCGATTATCTTCGTCCAATTGTATGGCATAGTAAAATCCAGGATT
CTCAGCCTGCATTTTCTTGAAGTAATCAAGAAGATTCTGAGCATCTCTTCCTAATGTTCTTCGTTGATTGCATTG
TCTAGGAGAAAAACTCTGAGGTACAGTATCCTTAACTGTTTGATCTGACTCTGCAGGACGAGTACTCTTAGCT
CCACGGCTAATCTCTAAAGGGATGCGGTTACCATCTACAGAGACATACATTACACCACTAGGAACAATTCCT
ACACCCTGATTATTCTCAGGTCCATTCTTTTTTGCCACCTCAAAATGCCTGTGTGATGTGTGGTAATGCACCTT
ATTAGGATGCACAAGGGAGTGACTATGCTCCTTCATATATTTGGTCACAACCCACTTGTTTTGACCTTTTAACT
CCACTCTAAGCATTGCATCACAGGATTCACCGTACACCTTTTGTGGCTCAACAACCTCATCCCGTTCAGGTGC
ATTTGATCTTCCATTAACATTTGCTTCTCCACTCTCGCTGGGTTCATCATCACCGTCATCAGTTATGCCACCTTG
GTGTTGCATATTCCCCTGTTCGACATCGATCACTTCAACATCCATCCCAGAACATGTAAACCACTTTAGAAAA
ACGCATCACATCTCTAGAGGTGCTCCAGCACATATCCCTGAAGATGCCATACATAACCACGTCACGACCTATT
TACCATAACAATCATCTTTAGCTGCTATTCTTTTCATCCTATGATCGGAGGAGAGAGAATCAAGGCCAAACGG
ATCTCAAACAATTTGCGTTATGAAAAACGGAAGTCCAAAGTTGTGTGAATTCTTCAGAAC
>TCONS_00081280
CTTAATTCTGTCTCAGTCACCGTGTGATCGGCACGGGTTCGATCCCAACCTGATCGAATAGGCCCAATCCACG
AGGTGCCACTCGCTTCATGGTTCTCAGCGCTCATGTATCATACCTTAGCATGGCTAAGTAAATTCTCAGCAAC
GAGACCCTCGGCTCGTGTGCTCCCTACTTTGGCACAAGTAGTTTCAGGAAGTCAA
>TCONS_00081370
CTATTGATCACTTGAAGATGGTGATGAAGGCTGATCAAACACTATCTTTGCAAGTTGTGTGTATTTACTCCCTA
GGTCATCATATTTATCCTCTAGCCCTTCCAAACGTCCTTTTAGTGATTCATTTTCTTTTAAAGTAGCATTCAGCT
TTTCCAACAATGCACCTTTAGAGGAGTTACCACCTTTCAACTCCTTAGCGGTTATCCCACCACCAAATCCAAC
TACATGACTCTTTTGTTGAGGTCCGAAGCACCTTTCTACAACCTCAATGTTTGTAAGAGACGATTCAGATTGCA
CCAATTCTTTGATTTGATCATATTTCTCACTGGTTTCAGGTTCGACAAGCTTGTTGTTCTTCTTACGAGTCTCAA
AAAAAATAGTTGCCATATCTGGTGGATTACCATCTTTACCTCCCAATTCATAAATGATCTCCCTAATCGGTTTA
CTACCCGTACGATGAGGCATACTCAACTTAGACCTATTGACTGAGTTTCTTGTACTTGTCTCCTTAAATTTTTCA
GTAAAAAAATGCTCCTTGACCAGCCATACCCAATCACTCTTGTCTACCCCCTGTGGCACATCTTTTATAGCAT
AACGGAATGGCTTAGACTTAATATTCTTGTGCATCGATCCTCTCCAATTGTTCCATAACTTTCTCATATGTTTCA
AGACATTATCTCGTTGATCATTTATGTCATCACGTAACAGCTTCCCACATGTGTTGTAGCTTATATTCCTCGAT
>TCONS_00081371
CTATTGATCACTTGAAGATGGTGATGAAGGCTGATCAAACACTATCTTTGCAAGTTGTGTGTATTTACTCCCTA
GGTCATCATATTTATCCTCTAGCCCTTCCAAACGTCCTTTTAGTGATTCATTTTCTTTTAAAGTAGCATTCAGCT
TTTCCAACAATGCACCTTTAGAGGAGTTACCACCTTTCAACTCCTTAGCGGTTATCCCACCACCAAATCCAAC
TACATGACTCTTTTGTTGAGGTCCGAAGCACCTTTCTACAACCTCAATGTTTGTAAGAGACGATTCAGATTGCA
CCAATTCTTTGATTTGATCATATTTCTCACTGGTTTCAGGTTCGACAAGCTTGTTGTTCTTCTTACGAGTCTCAA
AAAAAATAGTTGCCATATCTGGTGGATTACCATCTTTACCTCCCTTAAATTTTTCAGTAAAAAAATGCTCCTTG
ACCAGCCATACCCAATCACTCTTGTCTACCCCCTGTGGCACATCTTTTATAGCATAACGGAATGGCTTAGACT
TAATATTCTTGTGCATCGATCCTCTCCAATTGTTCCATAACTTTCTCATATGTTTCAAGACATTATCTCGTTGAT
CATTTATGTCATCACGTAACAGCTTCCCACATGTGTTGTAGCTTATATTCCTCGAT
>TCONS_00081383

CAAGAGTCATAATTTCTATTCTAATAACAACCTTAAAAGCAAATTTTCTTCCAGTAACTTGTGCATTGATGGA
CCATATTGCCAGCATGTTGGTTTACTCATGATCATCCCGACTCGCACGCCTTCTTCTATATCAGTTGGTGAAAC
TTTATCACCCAATCCAACAACAAACTTTGCGATTTGCTTGATGTTTATAACATATTTTGCATCTTCAGTGTTGG
GACTAATTATCTTTGTACACCTCG
>TCONS_00081414
GTTTCTTCTGAGACATCCTGAAGCCCTAAGTTTCTTAAGCTGGCAGTGACATCTTCCTGCTGTTGTTGAAGAGT
TGTTTTTAACTCTTGAAATTCGGATCTCAGCTGGTCCACTTCAACATTCAGCGATTTCTTGAGCTCCGACAATT
CATCACGATAGACCTTTACTTGGCCATCCAACTTTGATCTCCAGCTTTGAAGATCTGTTTTCAAGCTCTGAATC
CTAGTGAGCAGCTC
>TCONS_00081435
TACCGCGGCTACTGGCACCAGACTTGCCCTCTAATGGATCCTCGTTAAGGGATTTAGATTGTACTCATTCCAA
TTACCAGACTCATAGAGCCCGGTATTGTTATTTATTGTCACTACCTCCCCGTGTCAGGATTGGGTAATTTGCGC
GCCTGCTGCCTTCCTTGGATGTGGTAGCCGTTTCTCAGGCTCCCTCTCCGGAATCGAACCCTAATTCTCCGTTA
CCCGTCATCACCATGGTAGGCCACTAGCCTACCATCGAAAGTTGATAGGGCAGAAATTTGAATGATGCGTCG
CCGGCACGATGGGCGTGCGATCCGTCGAGTTATCATGAATCATCGCAGTAACAGGCAGAGCCCGCGTCGACC
TTTTATCTAATAAATGCATCCCTTCCGGAAGTTGGGGTTTGTTGCACGTATTAGCTCTAGAATTACTACGGTTA
TCCAAGTAGTAGATACCATCAGACAAACTATAACTGATTTAATGAGCCATTCGCACTTTCACAGTCTGAATTT
GTTCATACTTACACATGCATGGCTTAATCTTTGAGACAAGCATATGACTACTGGCAGGATCAACCAGGT
>TCONS_00081470
CAAGGCGTTGACTTCTTGAAACTACTTGTGCCAAAGTAGGGAGCACACGAGCCGAGGGTCTCGTTGCTGAGA
AATTACTTAGCCATGCTAAGGTATGATACATGAGCGCTGAGAACCATGAAGCGAGTGGCACCTCGTGGATTG
GGCCTATTCGATCAGGTTGGGATCGAACCAGTGCCGATCACACGGTGACTGAGACAGA
>TCONS_00081861
CTGCAAGTCAAATTCTCTTAGTGGATAACTCTTTTCAAAGATATTGTTTGGAAGCTTCTGTAACGTGTTTTGCA
AATCAAAGTGGAATGTGTTTCCATATGAGCGACAAGGAGCAAGCGACCAAACAACAATAGCATTGCAAGCC
GTTACAGTAAGCACATTAAGAAGGGCAAGATCCCACTCATGCTTTATCCTCTCCTTACGGTTTTTGATCTCCC
>TCONS_00081930
TCCTTTTCTGCAGATCCAACTCTTTCCTGGGATTCATCTGCCCTTGCGATTTCACGGCTTGTGCTAGCTTCAGTT
CCCCTTTTAATATTTTCGTTTTGGTTCCGATTCTGAAGGGTGGCAGTTGAGTTGCAATTTGGGGAAGAACCAGT
AGGACCTTGAGATGTAGAAGAAGAAGAAGAAGCACTAGAATTTGAGTTGTTAGGAAGATCTAAAATACTGA
ACCCAAAGAATAGAAGCAAAACAAGAAGAATCATCACCACTAACTGAATCCAAATTAACCATGGCACTTTG
TAGTTCTCAATTATTAGCTCTTCCCCAAATTCATACAT
>TCONS_00082022
AAACATGCTTGGCCCTGTGAAATCCTGATTGAAGGGGCTTGTTGGTCTCTCAAATGGCTTGTTCATTCCATTCG
CCATGGTGCTAGTACCTGCTTTGATGAGTCACGAGCTTGAAGGGACTTATTCTCGGAATCCAAATGCAAGTCC
TGTAGCAAATCCGCCGTTTCTTAAGCAATGTGAATTAAGGAGACAAAAAAACATGCCATAAA
>TCONS_00082064
CCCTTTTCATAAATTAATTAGGTAATAACAAGATACGTGGCGTAATATGGACCGGCCATAAGTACATAACAG
TAAACGTGGCATAATATGGACCGATAGTCTAAGAAGGACGACGAACAGAGTTTATGTAATCTTCAAGTGCTT
GGATTTGCTCTTCAGAAGGTGGTGTTTCGTTGTTCTTTATGGATCTGTGCACATCATATGTTGTTGAAGCCAAG
AACAACACCCCAGAAGAGAAGAGTACAATTTGTCTTGGAAATATGTGCTTCCCAAAAATCCTCATCTCTTTTC
CT

>TCONS_00082067
AGGAAAAGAGATGAGGATTTTTGGGAAGCACATATTTCCAAGACAAATTGTACTCTTCTCTTCTGGGGTGTTG
TTCTTGGCTTCAACAACATATGATGTGCACAGATCCATAAAGAACAACGAAACACCACCTTCTGAAGAGCAA
ATCCAAGCACTTGAAGATTACATAAACTCTGTTCGTCGTCCTTCTTAGACTATCGGTCCATATTATGCCACGTT
TACTGTTATGTACTTATGGCCGGTCCATATTACGCCACGTATCTTGTTATTACCTAATTAATTTATGAAAAGGG
AT
>TCONS_00082102
TTGGTTTCAGCACCTCTATATGACAAAAACTATAGTAGCAGCTAGCATAAGGTAGTACATAATTTGGATATTC
ACGCAGCAAGGACAATCTTCTGCAACTCGTTTCATTCCTTAGTCCAACTAAAGCTTAGACTTCAGTGTTCTCCA
CTCATCAAATGATATCATCACCTTTCCAATTGCTCTCCTATCTTTGAGTGCAGTAAAGGCAAGATGGGCCTCTG
CCAGGCTGAATGTATGAGAAATGTTGATTGTAATCAAACCCTTAGACAACCAGGATAGGAGCTCTTTCAGAG
AATCTCCAAGCACATTAGGTTGATATATCTTATGGCTTCCCCAATAGAGTCCGTGAATCGTCCAGTTCTTCACC
AAAGCAATGTTCGCTGGGATGACAGGTACTTCTCCACTTGCAAATCCAATAACCAGAATTTGCGCTCCCCAAT
TTAATAGCTTCAAGCTATCTTTTGTAAGCTTCCCGCCGACCGGATCATACAAGACATCAACCCCTTTGAGCTTT
CTTGACTTCAGGAAGCCCTTGACACTTTCGATGACGTTTGCATTGCTCAAGTCTACTACATGATCAACTCCTAA
AGATTTCAAAAATTGCACCTTTTCATTTCCCCTAGCAACTGCAATAACTGTTGCTCCACAAACCTTCCCAATTT
GTACCGCTGAGAGCCCAACACCTCCAGCCGCTCCAAGTACCATTAACACCTGATTGGGACGCAGCTGTGCTC
TATGCACCAGAGCCACATGGGATGTCCCATATGCAACAGGAAGTGCACCCGCTGCCACTAGATCACACCCAT
CAGGTACTCG
>TCONS_00082256
ATATTACCAATCTTCACCTAGCAACAGTAACTGTACAACTATAACACTGGATTGGAACTTTCTTTTCCCTCTTC
AATAACGGACTCTTGTTGATTCGGATGAGCCATATCATATGTTGCATGGACACCAACAAGTACGTAGTAAAT
GAGCATTACAGCACTGCATAGGATGAACCGATAAAATGCTGCATTACCCAGAGAACCTATCAAAAATATATT
CATCCCAATTGACAATGATGGCAGCCATGGCACGAGAGGTACACCCCAAACTTTAGGAAATCTTTGTTTTGG
AAGCAGTGCCATTCCTAAAGTGCCCAACAACCACAAAGCGCCAGTCACAGTATACCCGACCCAACTTTTTC
ATCCATACTCCAGAGACATGTTGCCCCAATCGATGAACCAATTACAATGGACAAGCACAGAAGAAATTTAGC
CAAATCGTACTTCGGAGTAACATCCGTAACGTAGTACCGCCTAACGAGTAATGCAACAGCCATAAGCATGAA
AATGGAAAGTGTACTAAACGAGAACACGCTTGACAAGACATCCAAGCTCGTGAACAAAGCAAGAATGCAGC
TAGTTATAGTCGTCAAGAGAGTAGCATAAATTGGTGTGCCAGTTTTTGGGTGAACCAGAGCAAACCACGGGG
GAATCATATGTGCTCTCGCAATCTGAGTTGTGTATCGAGCTTGTCCCATCGACCCAACAAGCATACTCGTAGT
CATTCCCTTAAGTGCACAAATCCCCACCAAATACTTTGCCCAATTCATACCTATGCCCTCAAATGCAACTGAA
TAGGGCGCATTAACATCCACTTCAGTGAACTTAACCATCATAGTCAAAGCCAACGCCATCAAACAATAAACC
ACAGTGATCACAGACATTGAACTAACCAACCCAAGTGGTATATCCCTTGATGGCCTCTTAGTCTCCTCAGCCA
TATTTGCAACCATATCAAAACCCCCATACGACCAGTACACAACTGCTGCAGCCCTAAATACCCCACCAGCTC
CAAAAGGGAAAAAAGGCACTAAATTTGAACTCTTCCCATGAACAAACCCAACAATTACAATAAACAAGATT
ACACCAGCACTAACTATAGAACTGACCGAATTCAATATCGACGTCCTCTTTGTCCCGGTCATAGCAATCCCAT
TTGCAACAGAAAGAATCACAACAGCTAAAGGGTCCAACAAATTAAACCCTTCAGCAAAAGAATCAATCTTG
ATTCTTAAAAAATCAGCATCACTATTAATAATACTAGCAAAATAAGATGTCCAAGATCTTCCTAATCCAGCA
GCCCCCACAATAGCTTCTAATAAAATATTCCCAGCAGCTATAAATGCAACAAAATCACCCAACTCAACTCTC
AAAAATGAAAAAGACCCACCAGCTATTGGAATATCAACAGCAAATTCAGTGTAACAGAAAACAGAAAGCA
AAGCAGAAAGACCAGAAATAGCATATGAAAGGACAATAGCAGGGCCAGCATCAAGTCTAGTTTCTTGACCA
GTAATGTTGAAAATACCTGAACCAACTACTGAACCAAAACCAAGCCACAGTAGGTCCCACCAAGTGAGGCA

TTGTTTCATTTCATATTCTGACTGTTTTTTAACTCAACTAATTCTGTGGTCTCTGTTGAACGGCAAAAAAGGCG
GTTCTTGAGACGATTTGGGGTTTGAGAAAGGGCAGT
>TCONS_00082317
CGTCAAAATAAAAAAGAATCAAGCTTTTAGCGTTTTGATGTCATTTATATATACGCTGAATTGCTTCCTGTACA
ACTATAAGAGCTACAAGCTAGGGGTAATTAATGAGGACGACTCAGGTTGCTTCTCTTGTTGCTTTATGTGATC
TGCCACATGAGAAATTACTCCAGCTCCAGCATTGTGAAAGAATTCATTCTTCACATGGATAAAGTTATGCGCG
AAAAAGGGCCAGAACGCCGAGTGAAAGCTAACATCAACTTTCTTCCACCCCAACTGTTGCAAGCCACGTATC
ATTTCCTCTTCCATACTTTCATGATATTCTAATGTACTCTGCGTGCTTAGTGCATTTTGTGCTGCCTCCTTTGCTT
TGGCTGCCTCGAGGGGGTAATGAGGTCCTTCACATGAAGCTGGAGGACAATATTCCACATCTACTACATGTTT
GTAGCCATCCAACGATCGCCAAGGGGGCTTGACGAGTTCTGTCTCCCTTCTTATAGATGATGTACGCCAACCG
ACCATATGATCATAAGAGACATTAGCATATAGAACACGACATTTGAAAGCACCAAGCGCTGATATAAAATTT
CCGTCATCGCAGTCTGATGCCATCCTTAGCAGCAGAGGTGGTTTAGTAGGTTTGCCATCCGTAAGGAAGAGCT
GACTACCAGTTTGACCAACAAAAATAGGAGCCATAGGTGCTGCAATTTTCTCCAAGATTGGCAACCCGAAGA
GAAAAGGAAGCTGATTTTTCCCTCTCACACCCAGATGTGGTGTTGCCAAGGTGATAAAATTGACTGGCTCCAG
GCCAGCAATCAAACCTTCATTTAAGGAGCCTACTGGTTTGAGGTTTGCATTAGTTGAAGTGACTGAATCATCG
GACTGCTCACTACCATTTGATGAATAAAGTGCGGCAACTGCATATCTTGCAATCAATCCACCAAGAGAATGA
GCTAAAAAGGATATCTTCTTTAGGCTTTCTCTTTTCTGCACAACCAGCCTAACTTCATCTGCTAGTCGTTCTCCA
GCTCCATCAATCCCTGTGAAGGTTTTAGTATACGTATTACATGAACTTGCATATATCAAAAAGCTTCTTCCCAA
CCGCCTCCTCAACTCTGCTTGAACATACGTCCAGTCACCTGGGCTTGCTAAGATACCATGAACAAGGACTAG
AAGATGCTCAGGTTCTTCCTCCATGTCTATCAAGCCTTTCGGTGACACAATACTTCCTTCAGTTGTACTCATCG
CATGGGCTTTATAGCTTTGACTTCTCAAGATGTTGTTTATCCCAGAAAAGCTGAGGAATTTCCAATTGAACCCT
CGGCGTTGATCAAAGGATGAAGAAGATGAAGAACAAGAAGGAGAAGATGAACAAGAATTGCCAGACAAAG
AAGGGCGAGGAGAATAATTATGAACTAATGTAGCTGATAACGCCATATATATTGCAACAAAGTCTAGAGAG
AGGAAAATAGGACCAATTGGAAATCTCTCTTTCTTGCTTTTTCCGTGCAATTCGCTATGCACAGTGCGTTATGT
CAGCATCAGGAGAGCAACAACACCCACC
>TCONS_00082365
GAACCCGGGGCTCGCGAGAAGGCTCTCTTTGCAGCGGATCCCTCCTTGAAACGCTTTAAGTCGCATAAACAG
AGTGTGCGGAACCTTAAAAGGGTGGGAGATGTTCTAGCTATTGTTGTTGTAGCGGGATGTTGTTATGAAATAT
ATGTGAGAGCTGTTATGCGGGAAGAAGCAAGGAAGAATGAAGAAGGAAGCGCATAATGGCAGATCGTTCTG
TTTTTGCATATTTTCACTGCCTTTATTCTTTGAAACATGGGGGAAATGGGATAATGAAGGATGGGTATCTCTAA
GTACATGAAACTCGTGTTTTAACTTTTGTTTGGGGTTGGTGGTATGAAAATAAGGTGGTTCCAAACAGTTGAG
GCTGCATCTCAACGCCAAGAACTTTGCATGCAAATTTTGGTTTTATTTGAACTTTATGTTTGAATGCTCTAAAG
TGTATGTGATGCCTGGTGAGATTTTCTCACCTT
>TCONS_00082367
AAGGTGAGAAAATCTCACCAGGCATCACATACACTTTAGAGCATTCAAACATAAAGTTCAAATAAAACCAA
AATTTGCATGCAAAGTTCTTGGCGTTGAGATGCAGCCTCAACTGTTTGGAACCACCTTATTTTCATACCACCA
ACCCCAAACAAAAGTTAAAACACGAGTTTCATGTACTTAGAGATACCCATCCTTCATTATCCCATTTCCCCCA
TGTTTCAAAGAATAAAGGCAGTGAAAATATGCAAAAACAGAACGATCTGCCATTATGCGCTTCCTTCTTCATT
CTTCCTTGCTTCTTCCCGCATAACAGCTCTCACATATATTTCATAACAACATCCCGCTACAACAACAATAGCTA
GAACATCTCCCACCCTTTTAAGGTTCCGCACACTCTGTTTATGCGACTTAAAGCGTTTCAAGGAGGGATCCGC
TGCAAAGAGAGCCTTCTCGCGAGCCCCGGGTTC
>TCONS_00082378

ACCATCTCTTGACTGACAGCTTACCAAGTGGAAATTTCTTGAGACATGAGTCGGCTGCATAGAAACAATGAA
GTATGGATTCCCCAATTTGGAACATTGTGCATCTATGAGGGTATAGCCTTTTTCTTGCCAAGTATTTGTCATCTC
AGAAGCTTTTACTTTATAAGCTGCACTATTCAAATTACCTTCCACGTCTCCTATTTCAAGTTTCACATTGTTAGT
GACCTTCATTTTTGCTTTTTGAGATGCACAAAACAAATCCTCTTCCACGTCTGGCTTTTCAAGCTTCACCCTGAT
AGGCTCCTT
>TCONS_00082379
CTTACTTCGTAGTGGATACCTACAAGGTGACACATCCAAACTGGTAGCAGTTTTCTTTTCACTATGTTGATTGG
TGGTTGTCATCTTAGATGCTGAGGGATTCATTACTGGTAATTTGTTGCAGTGCTCTTCCACATCTCTTTTTTCAA
TCTTCACATCTTTCAGTTGCTTACTTCGCAGTGGATACCTACACCATGATGCATCCAAACTGGTAGCAGTTTTC
TTTTCAGTATGTCGATTAGTGGTAGTCATCTTAGATGCTGAGAGATCCACCACTGACACTTCATTGCAGTGC
>TCONS_00082380
CTTTTTTGCTTTTTGTACTGCACAAAACAAGTCCTCTTCCGTGTCTCGTTTTTCAAGGTTTACCTCTTTCAGTTGC
TTACGTCGCAGTGGATACCTACAACATGACGCATCCAAATTGGTAGTAGTTTTCTTTTCACTTTGTTGATTAGC
GGTTGCCATCTTAGATGCTGAGGGATCCACTACTGGTACTTCATTGCTGTGCTCTTTAAATTTATATGCTTCAG
CATACACATTCTGTTGCACATCTTGCTTTTCAAGTTTC
>TCONS_00082381
CCCACTATCTTTACAAAATTCTCTGGTGTCATATCTTCACTTGGGCCAGAAGGATATTCAATCTCGGAAGCACT
TAAATCAAATATGGAGACAGAGAAATGAGAGTTTCCATTATATTGGAAGAGTAAAAAGTAACCACAACCAA
TTGAATAGTACTCCTTGAACTGGTCCCAACCCTCCTTCAGCCATGCCATGCCATTAGAGTTTTGCAATTTTACT
TTCCATATCGCACCACTTGGGACCTCAAGGGACACAAGATCTCGCATGTTGGCCCCATACTTCATTACAAATT
CATCTGGGATGTGTAATTTAGAGGCATGAGGAAAGAGAATAATCTTGAAGAACTGAGGGGTTATCTCCGCCT
CATTCACCGCTTGTCGCCGTCTCTTTACCATCTCCG
>TCONS_00082389
AAAAAGAAAAAGCAAAAAAGAAGAAGAAAACACCATCACTACACATCACACTTTTCTCACATAAGGGCCTC
TGCTTTTTCTTCTTCATCTTTGATGAGCTCTTTGATTTTGACTTAGCTTCACTCTTCTCAGATCAGTGATTATGCA
TCTAAGTTGTAACAAAGTGGCTTCAATTTTTGAGTGAAGTTGAAGTTATGATGTTCGATCTAGCTGAAAGTTA
AGCTTTTTCATCTGAATCGAACTCTATGTCTGCTGCTCTGTAGATCCAGTTGACCCAATTTGGCTGGTTAAT
>TCONS_00082530
GTAAAACCATTACTTGAATCAATAACTCCGTAACGCTGCAGAATTTGAGCTTCTTATTCTTCAATTTCTCCTTT
AAAATCTTCATCTTCAATTCCTCCACCTCTTTTGAATTCTCCTCATTCTTGTTTTGGTCAATGATAGCAGAATTG
AGAGTGGTCTTCTTCAACGATGAGACTAGAATTGCAGCTTTGGCACATTGAATCGCGACTTTCGATTTGAGAG
GTTCAGCTATTTCTCTTTCACTGGTGATGTCCTCCATTTTCTCTGTGTTTTCCCGCGCTCTACTGCTTT
>TCONS_00082639
CTTCATTTGCAAAAGAATAAAAGCCATTAACATCCTAGATATATAATGGAGGGCAAATCTTGCCATGCTGAA
AATCTCAGTTATCTGTACTCTTCCGTCAGTGAATTTTGGACCTTCAATTTGTTTTGTTTTTAGTTTCACATATAAT
GATATTTATTTGTGCTTGTACCACAATTATGCCTTCCCAATAACAGATTACTTTTTGTTGATACACTAACATGTT
AGGCCAGTTTTTTGTATGCTTTTTCCTTGTTATTGTAAAAATTGCTGTATCAAGGATACATTAGTCACTTCCTTT
GGTGTGAGAAGGAAACAAGCCAAAGTTTCCAGGTAAAACAATCGGATGTCACAAAGGAAAGAGGTTCCAA
ATTTTGCAAATACTTTTTATCATCATCAAAATGCGTTACCTGTTGTATTTGAAGGTCCTTCTCTCGCAGTAACGC
TTCGTAATCTGAAGGGAATATACAAGCCCTTGGATACCTTAACTCACTTTCCAACCTTGCTAACTCCCTCTGTA
AATGCTTCACCAATGCTTTATCTGACATGGCTACGTTCACTTGGGCGTTAGTAGTTACTTCCTTAGCACAACTT
GCAAATAGTAGAGTATTTCTTGATTGCTCAACATGACTTCGTGCAGGACTCATTGTACAGATGATGGCAGTTC

TACCGTTGCCACTCAATGATGGCTGTAATATCCGGGTTAGCTTTGAATCTCGGAAAGGAATGTGACCAGTCCT
ATCCTTGCTGCATTAAGTTATACAACAACTTGAATTAGTTACAATTGTCGGCATTGATCAGCGGAGGGTCAGC
AAGCTGCGATTTATGTGACAACCTTCTTTCAGTCTTGCACCAGCTGACAACGACTGAGATGCCCGCTCACTTC
CAGCCAAATCAACAAAATTCTGTGTTTCAATGGAAAAACATTCAGCAGTTACCTTAGTATAATAGCAACACC
ACACTATATATCAATTGCTCTTCAGAGATGCATTTTCTTACCACAGTAGCTAAAAGGGTACCCAGGTTGTCCC
CCCCTAAGTGCTCACGAGCAGAACTTTCAATTGTCTGCAACAAATTATACTTCAAACTTCTAGTTGTATTAAA
AGGTCAATAGCATGGTAGACAAGAAATTTCAGTTGTTTACCAGTCTGATGATTTGGTGAGATCTGGAGCTTG
TTTCATTGAAGGCAGTCTCCCCAATCTGTCTCTGAGCTGCAACATTTAATTGTGAACTCAAGACCATGGTTAAT
TAATTATATGCCAAAAAAAGCTATTCATTCTGTTACCTTCACAAATAGAAAGTAGCCGAATCACATGGCCCC
AGTCCCTCAATATTTCCTCTGTGAGTTTCTCAACAACAGTCCCTCTCTGTTGAAATTGATCGGCATGATTTAAG
ACGGGAAAAGATATACAAAAACAAGCAGTAACATTTTGTTGATCAGTGAAGCAAACTAACCTCTGGATCGTC
AAGAAGTCTAAGTGGAGCACTATCTTCACTAAGGAGATCTCTGACAGATTCATTGTATATCTCCATAGCAGAA
AACTTCAAAATAAAGACTCTCTCTTTGTG
>TCONS_00082678
TAATATATCATTCAGATTCAGGTGACCCACCAGGAACCAGGATAGCAATATTGTTTCCATTAAGCAGAATCTG
ATCAAGTTTTGTTACTCGCTTCCCTTCTGGAGTGATCTCATATTCAGTGACATCTTCAAGAACCATATTAACAT
AAACATCAAAGCCTCTAAGGGTACCAACTAGCTCCTTATCCCCCTTCATTATCACCCATATCTTTGACCCAAT
GCAACGATCAATCAACTCAGATGGAAGAAGCTGAGAAGGGTTGTTTGAAGACATTGGGAAATTTCGCAAAG
GTTTTCTCTTGCTCTTTAG
>TCONS_00082768
GACAAATGTGCTAGCCACATTTAAGAAAATCAAATGGAAGCTAACAGAGAAGATACTAATCCTCTTCTGATG
CAACGCGCTGCTTCTTCCATTTATAAGCAGCTTCCAAGATCTTGAGGTTGGTGGTTTGCGTTTCCTCGGGAAAA
AGATAATCGATATACTCCTCGTACGCTGTTGGACCATCCTCCATGTCTATTTGCCTTCTCTTCTTGAGTTTCTTT
GGTAGCTTTGCACGGACTAAGTTAACATCCCCAAGCTCACCAAAACCGCTTTCCATTTTCAGCCATTCTTCTA
AAAGCATTGCTCGTTCTTCTTTCAATTCAGGAGCAGAAGTTCGGTAGTACGTAATGGCCTTTTCAAAAACATC
TCTGGCATGCTGCAGGCGCTTCTTCTTCTGTTCAATATCTTCCTCAACCTCTGAATCCACAGCCGAGGCCTCAA
AATTCGCATAACTTATCCACACCTTCAAGTGTTTCGTGCGGTTAAGAAGTCTTTCATACAGTGCTCTTGTTCTA
TCAAATTCACCCTCAAAGATCTCGAAGTCGATGTAAGCCTTCCATAGTAACTCTGGCATATCCAGCGCAGGTT
GGTCAATTGCAAGCTCAAAAATAGCCCTGGCTCGCTCCGTTTCATCCAAGGATCTCTCTAACTCAGCGAATTT
GCTCCAAGCATAGCAATTTTCTGGTGACCATTCTAAGTACTTCTCATAAAGCTTTCTACAACGGTCTATATTTC
CAAGATGCAGCTCTATCTCAATGTACTTCTTAAATATCTTGTCTTTAGGAGCTCTTCCAATCGCTTCTCCAAGT
AGCAGCCGTGCTTCCTTGAGTCTTAACTGCCGGATCTCAAACTGAGCAGCCAACAACCAAATCTTTGCAAATG
AGAACTTCTGATGAGGAATCAGCTTAAGACACTCCCTGTAGACATCTCTGGTCCTTTCCATGTCTTGTGCGTCA
AGCTCTTCATATAATGCATAATTAATCCATAAGTAAATGTATCGCTGCCAATACCGCTTCTCTTCAGCAGGAG
GAACGTTAGCAATGGCTCTCTCATAAACCTCTCTAATCCTCTCTTTATTCCCTACACTCTCTTCCAACCGAAGA
TAATCAAACCACGCGTCATAGTTCCGCGGATTCTTCCTTACTTCATCCTCATACTGAAATCTCCTTTTCCCAAC
AATAGCATCCTCAATACCTTCCCTATCACCATACTGTTTCTCAAAAGCCACAAACTTCCTGTACAAATCCTCA
GCTCGCCCTTTCGGAATATGATCAAGTGCAAACTTATAAATGCACCTTGCCCTCTCTGTCTCCTTACACTTCTC
CTCAAACTCCGCGAACGCCACAAACAGCTGCTCTGCTTCCTCGTCGTCCGCCAACTTATCCACGGCCCTCTCA
TAACAATTCCTCGCCCTTGCTACCTCGCCATTCTTAAACTCAAACTTCGCAAACCTAATCCATGCACTAACCTT
TGGATGACACTGCACGAATCTCTCGAAAATGGCCCTCGCCCTATCCACCTCATTATACCTTAACTCAAACTTA
ATGTAACTTAACCACCCTTGCTGGTCCGGCATCCATGTCATCCACCTCTCAAAAATCTGCCTTGCGCCAGCTA

CATTCCCAAGCATCTCTTCCATGTGAATATACTTGTACCATAACTGATCAACCCTAGGCAAGAGGGTAACAGC
ACGATCCCACACATTACGAGCATGATTCACGAACTTATTCTTCATCTCCACGTCAGCATACTTCAACCACATC
GTATGATCCCTGTAATCGACCTCAAGTGCTCGCTCCCAAACGGAACGAGCACGCTTGAAGTCCTTTTGTGATT
CTTCCCATTTAGCATATTTCACCCACACGCTTTTGTTCCAGCGGACTCGGCTAATTAAGCTTTCGAATTCCTTGC
GCTTGCGGAGGCGGTAATCGGCGAGTTCGGTCGGGTCAGTGATTTTTTGCTTCGGCGGTCGGATTTCGGCCTCT
TGACGTTCTCTAGCTTCCCGAAGGATTTGCTCGGCGGTGATTTGGATTGGAGCTGGGGTTTTGTTCTTCACCCG
TGTAGGTCTAGGAAGCTTTACCTCTGTTTCTTTTCGAGTCAGGTACCCCAGATTGGGGTCTGCATCTCTGTTCG
CCATG
>TCONS_00082853
ATCGATGTTTTCACCTTACTTAACTAGAGAGCAGATGCTGGAGTGGGAGGCCGGGCTTTCGCCTTTAAAGGTA
TAAGCTGTGATGTGATAGGCACGAAGTCAAGCAACCTATGAGTAAGTACTCCCGGTCTATACCTTGGCTAAA
GTATCTTGCCAGGTATGCTAAGCTACCTATCCCTAAAGAGAATAGGCATCTTGTTACGGATAAAACTACGTTT
AAGAAAGAACGATCATATGATGTCCAGAAAACGCATTAGAAAGCTCTATAGAGGTACGCAGTTACTCCACC
GATAGGGAAAGGGGAGAGGGATTGCGAAAACTGAATTACAATAGCTCAAGTTCAGTGAGTTTTAGGTATTGG
TATTGAGAAGAAGAAGGCTTTTTTTTTTTCTTTTTTTTTTGTCCCGAAGTCATTCTCCATTCCAATTTGGAATAG
CAAGCGTAAGCGCAGGAGTTTTCTTGCTCTCGCTATCGTACCTCAGCCTTTCCCCAAGCACTAACTAAGCCTT
CATCCTCTCCCTATGCTCTTCCTGCGCTGTAGCTTTTCTCAATCCTCTCGCTATCGGTAAAGCAACTATGTACCT
TTCGGTAGGTACAGTGTCTTTCGCTTCCCTTACTCCACTCTTTTCTCCTTTTAGGGAAAGAAAGAGCTTTGTAA
AGCGATTCATATCGATTACGTAACGCCCTCTTCCCTGCTAGTATCCCTGTCCCATTAAGGTTAGGTGGGGGAA
TGGAACTGAAAGAAAGACTGTTGGAGAATATAGGTCTACTTTTTCTGCTGGCTTGGTTGAACTGGCTAACCCT
GCATTGCTTTCAAAGGAATGCCTTTTACTATTGTTACTTCCCTCTCAACTTGCCATAGGACTTGTAAAATTCCC
ACATCCGATTCGTAAACAGACAGGCTTGACCCAACCCTCAGACTGTCTTATAATAAGATAGTCATAATAGTCT
AGTGCTAAAGGCCTAAAAAGAAAATGAGCAAAATCTTCCTCCTTCAGGTGTGGCTTAGCATTAGGAAGTTCT
TATAGAATGCGCTGTGAAGGAGAAGACAGAAGCACTGACATAGTGAATGTCCGAGGAGTCAATAAGTGCCT
CGCTTTACAGTAAAGGAAGACGAATCCGCGAAAGGAAAGGTAACTGAAAGCGTATCCGCTTTGGGACCTGA
AGGATTTTCTGTTCTGACCTTCCTGAGCACGAGAGGCGTAACGATCTGGGCACTGTCTCGGAGAGAGGCTTGG
TGAAATAGACATGTCTGTGAAGATGCGGACTACCTCCACCTGGACAGAAAGACCGATTGAAGCTTAACTGTT
CCCTGGGATTGGCTTTGGGCTTTTCCTGCGCAGCTTAAGTGGAGGGCGAAGAAGGCC
>TCONS_00082854
ATCGATGTTTTCACCTTACTTAACTAGAGAGCAGATGCTGGAGTGGGAGGCCGGGCTTTCGCCTTTAAAGGTA
TAAGCTGTGATGTGATAGGCACGAAGTCAAGCAACCTATGAGTAAGTACTCCCGGTCTATACCTTGGCTAAA
GTATCTTGCCAGGTATGCTAAGCTACCTATCCCTAAAGAGAATAGGCATCTTGTTACGGATAAAACTACGTTT
AAGAAAGAACGATCATATGATGTCCAGAAAACGCATTAGAAAGCTCTATAGAGGTACGCAGTTACTCCACC
GATAGGGAAAGGGGAGAGGGATTGCGAAAACTGAATTACAATAGCTCAAGTTCAGTGAGTTTTAGGTATTGG
TATTGAGAAGAAGAAGGCTTTTTTTTTTTCTTTTTTTTTTGTCCCGAAGTCATTCTCCATTCCAATTTGGAATAG
CAAGCGTAAGCGCAGGAGTTTTCTTGCTCTCGCTATCGTACCTCAGCCTTTCCCCAAGCACTAACTAAGCCTT
CATCCTCTCCCTATGCTCTTCCTGCGCTGTAGCTTTTCTCAATCCTCTCGCTATCGGTAAAGCAACTATGTACCT
TTCGGTAGGTACAGTGTCTTTCGCTTCCCTTACTCCACTCTTTTCTCCTTTTAGGGAAAGAAAGAGCTTTGTAA
AGCGATTCATATCGATTACGTAACGCCCTCTTCCCTGCTAGTATCCCTGTCCCATTAAGGTTAGGTGGGGGAA
TGGAACTGAAAGAAAGACTGTTGGAGAATATAGGTCTACTTTTTCTGCTGGCTTGGTTGAACTGGCTAACCCT
GCATTGCTTTCAAAGGAATGCCTTTTACTATTGTTACTTCCCTCTCAACTTGCCATAGGACTTGTAAAATTCCC
ACATCCGATTCGTAAACAGACAGGCTTGACCCAACCCTCAGACTGTCTTATAATAAGATAGTCATAATAGTCT

AGTGCTAAAGGCCTAAAAAGAAAATGAGCAAAATCTTCCTCCTTCAGGTGTGGCTTAGCATTAGGAAGTTCT
TATAGAATGCGCTGTGAAGGAGAAGACAGAAGCACTGACATAGTGAATGTCCGAGGAGTCAATAAGTGCCT
CGCTTTACAGTAAAGGAAGACGAATCCGCGAAAGGAAAGGTAACTGAAAGCGTATCCGCTTTGGGACCTGA
AGGATTTTCTGTTCTGACCTTCCTGAGCACGAGAGGCGTAACGATCTGGGCACTGTCTCGGAGAGAGGCTTGG
TGAAATAGACATGTCTGTGAAGATGCGGACTACCTCCACCTGGACAGAAAGACCGATTGAAGCTTAACTGTT
CCCTGGGATTGGCTTTGGGCTTTTCCTGCGCAGCTTAAGTGGAGGGCGAAGAAGGCC
>TCONS_00082855
ATCGATGTTTTCACCTTACTTAACTAGAGAGCAGATGCTGGAGTGGGAGGCCGGGCTTTCGCCTTTAAAGGTA
TAAGCTGTGATGTGATAGGCACAAAGTCAAGCAACCTATGAGTAAGTACTCCCGGTCTATACCTTGGCTAAA
GTATCTTGCCAGGTATGCTAAGCTACCTATCCCTAAAGAGAATAGGCATCTTGTTACGGATAAAACTACGTTT
AAGAAAGAACGATCATATGATGTCCAGAAAACGCATTAGAAAGCTCTATAGAGGTACGCAGTTACTCCACC
GATAGGGAAAGGGGAGAGGGATTGCGAAAACTGAATTACAATAGCTCAAGTTCAGTGAGTTTTAGGTATTGG
TATTGAGAAGAAGAAGGCTTTTTTTTTTTCTTTTTTTTTTGTCCCGAAGTCATTCTCCATTCCAATTTGGAATAG
CAAGCGTAAGCGCAGGAGTTTTCTTGCTCTCGCTATCGTACCTCAGCCTTTCCCCAAGCACTAACTAAGCCTT
CATCCTCTCCCTATGCTCTTCCTGCGCTGTAGCTTTTCTCAATCCTCTCGCTATCGGTAAAGCAACTATGTACCT
TTCGGTAGGTACAGTGTCTTTCGCTTCCCTTACTCCACTCTTTTCTCCTTTTAGGGAAAGAAAGAGCTTTGTAA
AGCGATTCATATCGATTACGTAACGCCCTCTTCCCTGCTAGTATCCCTGTCCCATTAAGGTTAGGTGGGGGAA
TGGAACTGAAAGAAAGACTGTTGGAGAATATAGGTCTACTTTTTCTGCTGGCTTGGTTGAACTGGCTAACCCT
GCATTGCTTTCAAAGGAATGCCTTTTACTATTGTTACTTCCCTCTCAACTTGCCATAGGACTTGTAAAATTCCC
ACATCCGATTCGTAAACAGACAGGCTTGACCCAACCCTCAGACTGTCTTATAATAAGATAGTCATAATAGTCT
AGTGCTAAAGGCCTAAAAAGAAAATGAGCAAAATCTTCCTCCTTCAGGTGTGGCTTAGCATTAGGAAGTTCT
TATAGAATGCGCTGTGAAGGAGAAGACAGAAGCACTGACATAGTGAATGTCCGAGGAGTCAATAAGTGCCT
CGCTTTACAGTAAAGGAAGACGAATCCGCGAAAGGAAAGGTAACTGAAAGCGTATCCGCTTTGGGACCTGA
AGGATTTTCTGTTCTGACCTTCCTGAGCACGAGAGGCGTAACGATCTGGGCACTGTCTCGGAGAGAGGCTTGG
TGAAATAGACATGTCTGTGAAGATGCGGACTACCTCCACCTGGACAGAAAGACCGATTGAAGCTTAACTGTT
CCCTGGGATTGGCTTTGGGCTTTTCCTGCGCAGCTTAAGTGGAGGGCGAAGAAGGCC
>TCONS_00082888
GCTTGATCATTCACATAACTCCCATCTTTCCTCTTATGAGTCTCAGTCCACACTTTGCCTCGATCAACTTCTTTC
CCCATTTTCTTCATCAAATCCCTTCTTCTAGCCAGACTCATAGCACCACTAGTATGAGGAAGTGTTTGTTGTCC
TCGAATCCGTTGATTTCTCAAGCAAAGCGCCTTTGTTTCTTCTTTGGACCGATAGTTAACATACAATGCCCATT
GGTCCATGGGAATGCCTTCTGGAGCATTCTTAATGATATCTTCTTTGCTCAATAATGAATCATTGGCCTCATCC
CATAGTTTAAGCCTGAATTCCTTCCACTTTTTGTTAAG
>TCONS_00082915
ATGGCATGTTCAAGATTCACACTTGCTTCTTCTTAACACGGTATGGGTATTTTTGGTATATGCTTATGCTAGTCA
TAACCAGTGGAAGTGCCACAAGGCAGTATGGCGAAGGAGGTTTTCCATCAAAGATGTATTGGAGCAGAGCA
GTGACAAGAAGCGCAGACACAATGATAAATCCCTTCCTTACACCACCGGCATACGAAGTCACAAGACCCAC
AAGAATTCCACCAACCGCATTTAAAATAACAGGGATCAAAGTTAATGCAGTCCACCCGTAGAAGAATCCGT
GCTGTCGAATAG
>TCONS_00082976
CCTGCAAGGCCAGGCCGTAACCCCCATTTGCATCATGTTCAAAATATAGTAGCTTGAATTTGCTTTGGGCAGC
TGAAGTAACTCTTACTACAATCCCTGCCTCTGCTTGTTCATCACTAATTGTTACACCAAAAAAATGAGCATTTT
GCTTTTCTACCTTTCCACTGACTGAAGTGCCAATTGGAAGGGGGCGGACTGTGACAGTTCCATTCAAAGCTTC

TTCAAGAACATTAGCAGAAATAGTTGTTTTGATAGGGACACCCAACTTGCTAAACAATGCTGCGAACATTGT
GTTGACAGTTCCAAGGTTGGACAAATCAATTTCCATATCCATTCCTTCAGCTTCAATAGCCTCAAAGCCGGCC
ATATCATA
>TCONS_00082985
CTGTGCTAGAATTATCATTGACTTGATAAGAATAGGCACCTATCTTTGACTTGTCAAGCTCAGATGAAATATC
ATCATCGTCCAAATTCAATGTCTCCGTTTTAGTTTGTCTGGCCATGGCTGTAGAAACTCTCGAAGATGGAGAC
CTTTCTTTGAGTATAAGTGCAATTGTTGATACAGTTTTCCCAAGTCCCTGATCGTCAGCAAGAATCCCACCACA
ACAGGGTACACCAGCTTTTTCC
>TCONS_00083034
AAAGGTTTATTGTTTGAGGATCGAGGCCATGAGCAGATCGTTGGATACTCAGATGCTGATTGGGCAGGATCA
CCTTCTGATAGACGTTCTACTTCTGGATATTGTGTCTTAGTAGGAGGAAATTTGGTATCTTGCAAGAGCAAGA
AACAGAATGTGGTTGCTCGGTCTAGTGCAGAAGCAGAATATCGAGCAATGGCTATGGCGAC
>TCONS_00083043
CTGCAACCCATCATTCCAGTCTCCTCAAGAATGTCCAAGGCATACTTCCGCTGTGAAATAACAATACCCGAGC
TAGACTGAGCGACCTCAATACCTAGAAAATACTTCAGTCTGCCCAGATCCTTAGTTTGGAAGTGCTGAAAGA
GATGTTGCTTCAGATTAGTAATACCATCCTGATCGTTGCCAGTAATAACAATATCATCAACATAAACCACCAG
ATAAATACACAGATTCGGAGCAGAATGCTGATAAAACACAGAGTGATCAGCCTCACTACGAGTCATGCCGA
ACTCCTGAATAATTATGCTGAACTTACCAAACCAGGCTCGAGGGGACTGTTTCAAACCATATAGTGACCTGTG
CAATCTGCATACACAACCACTAAATCCCCCTGAGCAACAAAACCAGGTGGTTGCTCCATATAAACTTCCTCG
TCAAGATCACCATGGAGAAAAGCATTCTTAATGTCTAACTGATAAAGAGGCCAATGACGTACAGCAGCCATG
GACAAAAAAGACGAACAGATGCTACTTTAGCCACGGGAGAGAAAGTATCACTATAATCAAGCCCAAAAATC
TGAGTATATCCTTTTGCAACAAGACGAGCCTTAAGCCGATCAACCTGGCCATCCGGGCCGACTTTGACTGCAT
AAACCCAACGACAACCAACAGTAGACTTACCTGCAGGAAGAGGAACAAGCTCCCAAGTACCACTCGCATGT
AAAGCAGACATCTC
>TCONS_00083045
AGGCTCGAGGAGACTGCTTTAGACCATAAAGTGACCGGCGCAAGCGACATACAAGGCCACGAGACTCCCCC
TGAGCAACAAACTCAGGTGGTTGCTCCATATAAACTTCATCCTCAAGGTCACCGTGTAAAAAAACATTCTTAA
TGTCCAGCTGATAGAGGGGCCAATGGCGAACAACAGCCATGGATAGAAAAAGGCGAACTGATGCTATCTTA
GC
>TCONS_00083046
GCCATAGCCATTGCTCGATATTCTGCTTCTGCACTAGACCGAGCAACCACATTCTGTTTCTTGCTCTTGCAAGA
TACCAAATTTCCTCCTACTAAGACACAATATCCAGAAGTAGAACGTCTATCAGAAGGTGATCCTGCCCAATC
AGCATCTGAGTATCCAACGATCTGCTCATGGCCTCGATCCTCAAACAATAAACCTT
>TCONS_00083047
GACATAAGGTAGCGCTGGAGGTCAGGAGAATAGCATCGATACCCCTTTTGCGTTCTCGAGAAACCAAGAAAT
ACGCACTTAAGAGCACGGGGAGCTAACTTATCTGTTCCTGGAGTAAGGTTATGGACAAAACAAGTGCTTCCA
AAGACACGGGGTGGAAGAGAGAACAAAGGTAAGTGGGGAAACATAACAGAGAATGGAACTTGGTTCTGGA
TAGCTGAAGATGGCATACGATTAATAAGATAGCAAGATGTAAGAACTGCATCCCCCCAAAAATGCAACGGA
GCATGAGATTGTATGAGTAGGGTACGAGCAGTTTCAATAAGATGTCTATTCTTTCTTTCAGCT
>TCONS_00083048
TACCTCAGAAATATCTAAGTGATTACCTGGACCTGTGAAGTATCATTGGGTTTCAAAGAAGGTAACATCAGC
GGACATAAGGTAGCGCTGGAGGTCAGGAGAATAGCATCGATACCCCTTTTGCGTTCTCGAGAAACCAAGAA

ATACGCACTTAAGAGCACGGGGAGCTAACTTATCTGTTCCTGGAGTAAGGTTATGGACAAAACAAGTGCTTC
CAAAGACACGGGGTGGAAGAGAGAACAAAGGTAAGTGGGGAAACATAACAGAGAATGGAACTTGGTTCTG
GATAGCTGAAGATGGCATACGATTAATAAGATAGCAAGATGTAAGAACTGCATCCCCCCAAAAATGCAACG
GAGCATGAGATTGTATGAGTAGGGTACGAGCAGTTTCAATAAGATGTCTATTCTTTCTTTCAGCT
>TCONS_00083049
TCGTCAAGATCACCATGGAGAAAAGCATTCTTAATGTCTAACTGATAAAGAGGCCAATGACGTACAGCAGCC
ATGGACAAAAAAGACGAACAGATGCTACTTTAGCCACGGGAGAGAAAGTATCACTATAATCAAGCCCAAAA
ATCTGAGTATATCCTTTTGCAACAAGACGAGCCTTAAGCCGATCAACCTGGCCATCCGGGCCGACTTTGACTG
CATAAACCCAACGACAACCAACAGTAGACTTACCTGCAGGAAGAGGAACAAGCTCCCAAGTACCACTCGCA
TGTAAAGCAGACATCTCGTCAATCATAGCCTGTCGCCATCCAGGATGAGA
>TCONS_00083050
CTGCAACCCATCATTCCAGTCTCCTCAAGAATGTCCAAGGCATACTTCCGCTGTGAAATAACAATACCCGAGC
TAGACTGAGCGACCTCAATACCTAGAAAATACTTCAGTCTGCCCAGATCCTTAGTTTGGAAGTGCTGAAAGA
GATGTTGCTTCAGATTAGTAATACCATCCTGATCGTTGCCAGTAATAACAATATCATCAACATAAACCACCAG
ATAAATACACAGATTCGGAGCAGAATGCTGATAAAACACAGAGTGATCAGCCTCACTACGAGTCATGCCGA
ACTCCTGAATAATTATGCTGAACTTACCAAACCAGGCTCGAGGGGACTGTTTCAAACCATATAGTGACCTGTG
CAATCTGCATACACAACCACTAAACTCCCCCTGAGCAACAAAACCAGGTGGTTGCTCCATATAAACTTCCTC
GTCAAGATCACCATGGAGAAAAGCATTCTTAATGTCTAACTGATAAAGAGGCCAATGACGTACAGCAGCCAT
GGACAAAAAAGACGAACAGATGCTACTTTAGCCACGGGAGAGAAAGTATCACTATAATCAAGCCCAAA
>TCONS_00083051
TACATATCTAACACTCCCCCTCAAGCCGGTGCATACACATCATATGTACCGAGCTTGTTACACATGTAACTAA
TACGAGAACCAGTAAGAGGCTTAGTGAAAATATCTGCTAGTTGATCATTCGACTTTACAAACTTTGTGACAAT
ATCTCCTGAAAGTATTTTTTCTCTGACAAAGTGACAGTCGATCTCAATGTGTTTAGTCCTCTCATGGAACACCG
GATTTGACGCAATATGAAGAGCAGCTTGGTTATCACAAACTAGTTCCATCCTGCTGATTTCTCCGAACTTTAA
CTCCTTGAGCAACTGCTTGACCTAAACTAACTCACACGTTGCCATAGCCATGGCCCGATATTCCGCTTCGGCG
CTAGATCGAGCAACTACATTTTGTTTCTTGCTCTTCCACGAGACCAAATTACCTCCTACTAGAACACAATATCC
AGATGTAGATCGTCTATCAGAAGGTGATCCTGCGAATCAACATCTGTGTACCCAACAATCTGCTCGTGGCCTC
GATCCTCGAATAGCAATCCTTTGCCTGGAGCTGACTTTATATACCGAAGAATACGAACAACTACATCCCAGTG
ACTATCACAAGGAGAATCCATAAACTGACTTACAACACTCA
>TCONS_00083091
AAAGGATGTTTGAATTATTTGTGCTAGGGTGTACTGGTGTTGTGATGTTCCTTCATGGAGCCAATTTCTTCTTCC
ATGGTCTCTCTCATCATATGGCCATTCGTTCTCTCAGCTTCTTAGGATTTGTTGGTTGGTAAAGAAATGTCGAG
AATGATAATGATGATCGATCAGCTAAAGGAAGCTTGCACGAACCGCCATGCTTTTTGTAAATTATA
>TCONS_00083141
CAGATGACTATCAATCTGTATACTTGTTTTGCAGCTCGAAAGATACTCAAATTGCAGGGCACACAAACGATTT
AGATACACAGCATAAGCTATATATTTACCAAAGTCAACTAGTTAGACAGCGCAAATTAAGGCTAAACACAG
ACTCTTTAACACGTTCCAACAAACAAAGTCTCCTCACACTATTAATTAATATATGTACCAACATTAACATCAG
TTGGTTACTTATATTGCCATAGCTTGCTCCATGCCTTTCCCGTGAATTCCGAAAAATACCTTCCCAGCATTGTC
CAAGCTACTCGTGTCAATCGCAATTACCCCGAACATTGCCCTGAAAGCACCACAAACCACCGCAGGAGCTGA
CGAATTTGTCTTGTGTGAAACCCTGACTATCTTCTGCAAACCTAAGCGCTCTCCATCAGCAGCACAAGATGTT
ACCACATTGGACGAAAGCTCCGCAATAGCCCGGTTGAGATCCTTGGGGACATATCGATATTCTTTCTAAGCA
ATTGCAGAGAAACCGAACCCTCAATTGCCTTCTCACCCAAAACGCTCAAAGCTCTGTTGTTCTCTCCTGAGTA

TGATGCGTGCGTCATGGCACGTCGTAGTAATTCCACATCCTGAAATGTGTACTGGATTTGATTTTGAAGGGTTT
GGAGAGCGATTGAGAA
>TCONS_00083260
TGCAATGAAATCCTCAAAAAACTTCTCTGCCTCCTCATTCAATTGTTTTGATGTCCTGTTTCTGTCATTGCTTCT
CTTCCTACGTGGTTGTTTCTGCCCAACAGCAGAATTTTTTGTATCAGGAAGTTCTTTGACAATCTGCTGATCCCT
ACCTCTGTGGTCTTCTTGCTCCAATTTTGTTACATGATTCTTTCTGGCCACATGACCTGACTTGGC
>TCONS_00083261
CACAAAGTAAAAGACCACCTGAACTAATTCTATTTCGTTCTCTGTACCTTTCAAACAATAGCTCTTCACTTCGT
TGAAGTCTGTTATATTCAGCCATATCAAATCCTGAACTTTGGCAACTTTCAAGTTCTCCGGATACGTCTCCACC
ATCTTCAACTGTATAGATTGTTCTATTCTTCGGTTGACTATGCACAATTGCTGGGCTCCAACTCCCATGGCTGC
TGACGGAATAACTACTATCATTCTGAGGGGAGGTGACGTCCTGTCTTGCATCACATATTGACGTTTTCCTACTA
ACAGGAGTTTGCTCTTCATTCTTGGGAGTGAAAGAACTATCATTGCTTGTCTCCCACTGCAACCAAGGCAAGA
T
>TCONS_00083267
CTGCGATTCTGTTGAAGGCTTGTTTTATAGGTGTGAGCTCTGTGAATTCGATGTCCACCCTCTCTGTACTCAGTT
GCCTGAAACATTGCGCCATGTCCTGCATCAGGCCCATCCTTTAAGGCTTCTAGGCTCGTCCGAATCAGGAACC
TGTGCTGTTTGTAGAGGAGCATGTAACCGTTCTTCGTGGCGTTACAGGTGTCAACTATGTGAATTCGATATTCA
TATGGGTTGTCTTGTGGTACAATGTGAAAAGAAAACAACATGGCT
>TCONS_00083268
AGCCATGTTGTTTTCTTTTCACATTGTACCACAAGACAACCCATATGAATATCGAATTCACATAGTTGACACCT
GTAACGCCACGAAGAACGGTTACATGCTCCTCTACAAACAGCACAGGTTCCTGATTCGGACGAGCCTAGAAG
CCTTAAAGGATGGGCCTGATGCAGGACATGGCGCAATGTTTCAGGCAACTGAGTACAGAGAGGGTGGACAT
CGAATTCACA
>TCONS_00083278
TGAAGATGTCCTAAACCAATTATTTCATCTGCATGGGTGCAAAACAACAGAGATCGTCATACCGGAGCTTCGT
GAGCTGCGCAGAGCCCATACTGTTAGTTTTGGATCTGAATCACTATGCTTATTGAATCCTGATTGCGAGGATG
GGTATATCTATTTTCCATTCAAGCTATTAGTACAACTGCTTGCAATCTTGTAGTTTGGACTAATATAACTTTTTT
TCATTTTATGTACGAAAGGAGTAAGATTGACTAATGATACTCGCACAAATCTAGCACTCTTCAGATCATTTGC
AACATCAGATTATGTTTCTGTCCAGCGCCTTAGTTAAAAAATAAGTTACTGAATTACTTAATACATGTTGAAA
CAATTTGTTCAGTT
>TCONS_00083312
GTTCAGACAACTGTATTTGACCATTCCAAATGAGTATTTTTCGCAAAGATAAATTTGAAGCACAACTCATGTT
TCATGAGTTAGTTGAGATTATGTAAAGTAAATCCAACTCTGAGTTCTTCTGAAGGTTGATTTGGAGATTCGGAT
GGCTTTTTCTCTCCAGAAACTTGCTGCGTCAAAATTGATGGTATGTTTTCCTGTATCTTCTCCTTTTGAGGTTTTA
>TCONS_00083377
AGATCCAACATTACCTTTTCCATTCAGCAGCATATTGTTTGGGGATGATCTCCATCTTCTGTTTATCATCATCCA
TCACAGCATCCAAAGCCTGTTTGGCCATACTTTTGCAGTTGACAAACCACTGGGGCTTTACAAGAGGTTCCAC
TACATCATTGCTTCTTGAACAAATCCCAAGGCGCATCTCGTTATCCTTGACATCCCTTTTCCTTTAAAGCTTCA
GCCACAGCGAAACGAGCTTTGAAACGAGGCATTCCTTCAAAGTCTGCACCCCCGTTGCTATTTATTTTCCCAT
CGTCAGTAAAAATATTTATGAATTCAAGTTTGTGACGCTGTCCAAACTCAAAATCATTTGGATCATCGGCTGG
AGTTATCTTAACAGCGCCAGTCCCAAAGTTCATATCTACAAGTATTTCATCACAAACTATTGGAAGCTTTCTTC

CATTGAATGGATGAATAGCAAATTTCCCATGAAGGTGGCAGTATCTTTTGTCTTCAGGATGTATAGCAATAGC
AGTATC
>TCONS_00083394
CTTGGAGGGCTTCCACCATGAGATGGAGGGGAGGGATAGTAACCACCACCTCCATGGCCTCCTGAAGGAGG
CGATGGAGTAGGGTTGTGGTGATGCCCACCACTAGGGGCGGGGTTACCACAGTTTGCAGGTGGTGTTCCTCCA
TGGCTACTGCTTCCATGCGACGGCGTTCCATGAGAAGGTGATGTTCCATGGCCACCTCCTGAATGTGATGGAG
ATTTGTGAGAACCTGTTGGGGGA
>TCONS_00083421
TTGACCGTGCAGGTCCAGGTATTGATACCATAGCGCGTGAGTTGTCCGCGTAGCACGTGAGTTGACCGTGCGG
ATCCAGGTATTGATATGATGGCACGTGAGTTGTCCGTGCTTAGCGCTTGGGTTTTGGGAGCCCCTCCGGAGTCT
GTACACACCCCCAGTGAGCGCAGAGTGTTGAGAGTATTGAGTGTTGAGTGTTGAGTGC
>TCONS_00083422
GCACTCAACACTCAACACTCAATACTCTCAACACTCTGCGCTCACTGGGGGTGTGTACAGACTCCGGAGGGG
CTCCCAAAACCCAAGCGCTAAGCACGGACAACTCACGTGCCATCATATCAATACCTGGATCCGCACGGTCAA
CTCACGTGCTACGCGGACAACTCACGCGCTATGGTATCAATACCTGGACCTGCACGGTCA
>TCONS_00083489
CAGTCCAAGCTGACTATCTTCTCGTTGTTGGAGTTTGCTTGACTCTCAGCGTCGCAAATATTGTTGGTTTTACTA
GATGTTGCAAAGATGCTAAGAAGCAGCTCCAAGCATTTGCAACCCAGACTCTTACTTCTCGATTATCCTCTAC
TCTACAGTCGGTATTTAGTGTTGTTTGACCTATTTATACGCAGCAAGCTAGTTATGGAAAGAAACTGTTCGTCA
TTTTCCACCACTACGAACAATCATGTATTGACATGGTCTTTTCTCAGACTTCTCCTTCACTGAGGCTTGTATATG
TAGAATATGGACGAACTTGAAAAACATTTTTGTTCTCTTCAGTTTTCACGAATTCAATTGCTTCATTGTTTCCTG
TTGAGACTGTTTTTAGCTC
>TCONS_00083492
CAGCAAGCACCCCCGTCTCATTCTCCATAATTTTCTTCCTTTAGATCTCTGCAAGGAGTTAGAGTTTATACACA
AGAGCTGTTGTACGGTAGGGTACAGGCCCAATGTGTTGTCTACAACACTATCTCATCTTATTGCCACTAACTCT
GCCCATTTCATCTTGCCCATCATTCCCATTCGAGAGAGGTTAAGGGAGAAAGCAGAGGAGTATTTTGGGTGCC
AGTATGAGTTGTTTGTTGAATTCAGTGGTCTAATCAGGTTCAAACTCCCACTTCT
>TCONS_00083495
AGTTTTGTTGCATGTGCTTGTTGACACAATTTTTATATTATAAATGGTGTTCGTCTTATTTTAGGTTCTTTTTGTC
CATATTAATTAGGCGCGTTTATAGCGATATACTTTCCGTGGAATCCCGCAAATTTTCTTATTGGTGTAATCGAT
AACCAAATCGATAAGCCCCCAAAATCGATAAATCGAAATCGAAAAAATCGAAACCTTATTGAAACGATAAC
GATAAGCATATGTACAAATCGATAAGGGTCAAAATCGAATCGATAAATCGAATGCACACCCCTA
>TCONS_00083562
TGGGTTTGAGATTTAGCTTTACAAATAGAAAAAGGAGGCAACCGCAGCAGAGCTTCCTCTGTCTCAATCGGG
TCTGATCTCAGTATTGGGTCTTGAAGAAGTGGCTTTGTGTCATCTTTAGCGGGTCTGAAAGGGGGTTTTGTTAC
CTTCTTCACTTCATTTTCGCCGTTCATGTTACTGATATGGATGCTTTTCGTTGAATCCTCGCTTGGCTTCATC
>TCONS_00083685
CAAAATGAGGATCATACCTCTGCAAAGCTTTGACGAAGAGCTAGAAGTTTTCCTTCTTCACTCCCAACGAATA
CCAACTTTTGCTTTATTGTTTCTGAAGCAGAATTTTTCCTACCAATAATGACTCGAACAGCATCATGCATTATT
GTACGCGCTCTGTCTTCAACTATATCAGGTAACGTAGCACTGAACAACGACCGAAGAATTGAAGGATTTGTG
CACGCATTCACCACAAAATCAACCTGCTCTAGCAAGCCAGGCTCAAAAAGCTTATCAGATTCATCTAAGACA
AGAAATTCAACCCTACTTAAATCAAGCTTTCCTTTGCGGATAGCAAACTGTAAGCGCAATGGTGTCGAGATG

AGTATATCACATCGCAATTTAGAAAAGTCTCCACTTTTAGCGAGCTTTTTAGTCATCAACCTGATGTAGAACTT
CTTCCCTTTAGCCAACTTTCTGCATTCTCTTGTAGTCTGAGCAGCTAACTCTTTTGTAGGGGAAATAATTACAG
CTCGGACACCATCATTTGAAGTATCCTTGAGTTTCACAAGTATTGGAAAGACAAAAGCAAATGTTTTTCCAGA
ACCGGTAGGTGCACAAGCAAAGCATTCCCGTCCAGATAAGAGGACTGGAATAGCCTGTCTTTGGATTGGTGT
TGGTTCTTTAAATCCCAGTTCTGCCAGGTTGCGTAATAGATATAATCGACATTTATATCTGTATAACCATATAC
AAACAGAACATTGGTAAAAGAGA
>TCONS_00083701
CCCAGCCAATCACATAGAATAGACCATGAAGGCTAAAAAGAGCCATAGTAGTATGTCCCAGCCAAACATGA
TATCGAGTGGCATGTTCAAATGGGATATCTATAGCTCGAAGAAGAACTGAACCCCGTGCAACAGGTATAAAC
AAAAATGCTAAGCAGGTTAATCCAATGAATCCAAAACGACGGCCTGTTAGCTCCAGCAACATACCGCTGAA
GAGATTACAACAGAGGATATTAGACAATGACGCAGAACCTTTTCTCTTTCATGTCTTGTACATGAAACGAGG
ATAAGCGGTCAACACTCCGTATACTGTACATAATAACAGCCCAGATGATGTACACTGAGAAGAGTACAACTC
CAATCATTTCTGCAGCTGTAACAACCCCAAATGGTCCATCCACC
>TCONS_00083727
AGACTAAAAGCAACAATAATAACATCAAGATCAAAAAATAAATAAATAGATAGCAGAATATCTTTCAATGA
CGAAATTCGAGTAAATTCTAAAACACAACAGAACAAATTCCAGGCTTGACTACAGCTCCAGACTTGAGGAA
CACTTTACATTTCAGATCAAACACTCCCCAATGTGATGGATAGGAGCATAGAGAGTTCATGATTCCGCAGCA
GCAGAATGCATACAGCAGAACCATTTTTTTGATTGAGGCTTTCCAAGAGTTGCTCCATGCTTATTATATGCAG
GGTCACCCTTTGGTGGTGAGTTTGTATTGCCTCCGCTTCTCTTCTCATTTCGAGCTTTGTTAAAGATGACTGTGA
AACCCTCAGCAGAAGCTGGGTCATTGACATCCCACTCTCCAAATTTTGGAAGTGGACGACCTTTATCCGCCAT
GAGTTTTAAGGTTTTGGACAGAGAAAGAGAAAAAACAAACAGAGAAAATTCCTTCGTACAACTTTTGTTTTG
TGAATTTTGTAATTGGATTTATTTGTTTATTATTGGAAGAGAAGGGATAATTTTCTCTTACTTTTTCTCTCTATCT
CTCTCTGCAAATGAAATGGAAAAGTTCAGAGAGAGAAGGAGCCAAGGAGGTGAAGAGAGAGAGAGATG
>TCONS_00083913
CTTCATTGTCACCACTGTTTAAGAACCTTCAGAAGATTTCTAAAGTAGGATAGCTGAACTCTTCAAGGTTCTG
GAACAATTTCAAGGAACGAAACATTAAGAGTAGATCACCTGTTGCATTCCAGTCATGATAGCAGCCAGTTGT
TCTTGAATCCTCTTGGCTACTTCTACACTGATCCGGTTGTCTACTTCCGCATTCACGTGCTCAAGTTGCTCTTCC
AACTTCCTTTGCAACTCTTGTTCCATGTTACTTTTTAAGTCGGCAGCCATTTGTTCACGTTCTTCTTGCAACTTTC
GACTCA
>TCONS_00083919
TTAGAAACAAGCATCCAAGACACAGCAACAACACAAGGCAGCACAACAGCCTTTCCAGAAGCCATCACCTC
TGGATTGAGTAGTAACTGGAACAGAGGAATCACCTTCAACATCTCTGGTTGGATAACCAGCCGGTGGCGGTG
GAGCCACTAAAGCACCACCCTGTGGTTGCTGCTGTTGAGTGTACGCAGTTGCTTGAGCCTGGTTTTGGTCGTA
GCTATTCATGTTGAAAAGATGAAGATAAGAACAAGCTAAAGATCAATGAAGATATGATATTATATTCTTC
>TCONS_00083983
CTTAGAGGCGTTCAGTCATAATCCAGCGCACGGTAGCTTCGCGCCACTGGCTTTTCAACCAAGCGCGATGACC
AATTGTGCGAATCAACGGTTCCTCTCGTACTAGGTTGAATTACTATTGCGACACTGCATCGGTAGGGTAAAAC
TAACCTGTCTCACGACGGTCTAAACCCAGCTCACGTTCCCTATTGGTGGGTGAACAATCCAACACTTGGTGAA
TTCTGCTTCACAATGATAGGAAGAGCCGACATCGAAGGATCAAAAAGCAACGTCGCTATGAACGCTTGGCTG
CCACAAGCCAGTTATCCCTGTGGTAACTTTTCTGACACCTCTAGCTTCGAATTTCGAAGAGCCGACATCTAAG
GATCAAAAAGCAACGTCGCTATGAACGCTTGGCTGCCACAAGCCAGTTATCCGTGTGGT
>TCONS_00083984

CTTAGAGGCGTTCAGTCATAATCCAGCGCACGGTAGCTTCGCGCCACTGGCTTTTCAACCAAGCGCGATGACC
AATTGTGCGAATCAACGGTTCCTCTCGTACTAGGTTGAATTACTATTGCGACACTGCATCGGTAGGGTAAAAC
TAACCTGTCTCACGACGGTCTAAACCCAGCTCACGTTCCCTATTGGTGGGTGAACAATCCAACACTTGGTGAA
TTCTGCTTCACAATGATAGGAAGAGCCGACATCGAAGGATCAAAAAGCAACGTCGCTATGAACGCTTGGCTG
CCACAAGCCAGTTATCCCTGTGGTAACTTTTCTGACACCTCTAGCTTCGAATTTCGAAGGTCTAAAGGATCGTT
AGGCCACGCTTTCACGGTTCGTATTCGTATTGGAAATCAGAATCTAACGAGCTTTTACCCTTCTGTTCCACACG
AGATTTCTGTTCTCGTTGAGCTCATCTTAGGACACCTGCGTTATCTTTTAACAGATTTTCCGCCCCAGCCAAAC
TCCCCACCTGACAATGTCTTCCGCCG
>TCONS_00083989
TTTGCAGCGACCGCCGCGCCCTCCTACTCATCGGGGCCTAGCACTTGCCCCGACGGCCGGGTGTAGGTCGCGC
GCTTAAGCGCCATCCATTTTCGGGGCTAGTTGATTCGGCAGGTGAGTTGTTACACACTCCTTAGCGGATTTCGA
CTTCCATGACCACCGTCCTGCTGTCTTAATCGACCAACACCCTTTGTGGGATCTAGGTTAGCGCGCAGTTTGGC
ACCGTAACCCGGCTTCCGGTTCATCCCGCATCGCCAGTTCTGCTTACCAAAAATGGCCCACTTGGAGCTAGGT
ATGCTCACACTCGAACCCTTCTCAGAAGATCAAGGTCGGTCGGCGGTGCACCC
>TCONS_00083994
CCGGCGGAAGACATTGTCAGGTGGGGAGTTTGGCTGGGGCGGAAAATCTGTTAAAAGATAACGCAGGTGTCC
TAAGATGAGCTCAACGAGAACAGAAATCTCGTGTGGAACAGAAGGGTAAAAGCTCGTTAGATTCTGATTTCC
AATACGAATACGAACCGTGAAAGCGTGGCCTAACGATCCTTTAGACCTTCGAAATTCGAAGCTAGAGGTGTC
AGAAAAGTTACCACAGGGATAACTGGCTTGTGGCAGCCAAGCGTTCATAGCGACGTTGCTTTTTGATCCTTCG
ATGTCGGCTCTTCCTATCATTGTGAAGCAGAATTCACCAAGTGTTGGATTGTTCACCCACCAATAGGGAACGT
GAGCTGGGTTTAGACCGTCGTGAGACAGGTTAGTTTTACCCTACCGATGCAGTGTCGCAATAGTAATTCAACC
TAGTACGAGAGGAACCGTTGATTCGCACAATTGGTCATCGCGCTTGGTTGAAAAGCCAGTGGCGCGAAGCTA
CCGTGCGCTGGATTATGACTGAACGCCTCTAAGTCAG
>TCONS_00083995
CCGGCGGAAGACATTGTCAGGTGGGGAGTTTGGCTGGGGCGGAAAATCTGTTAAAAGATAACGCAGGTGTCC
TAAGATGAGCTCAACAAGAACAGAAATCTTGTGTGGAACAGAAGGGTAAAAGCTCGTTAGATTCTGATTTCC
AGTACGAATACGAACCGTGAAAGCGTAGCCTAACGATCCTTTAGACCTTCGAAATTCGAAGCTAGAGGTGTC
AGAAAAGTTACCACAGGGATAACTGGCTTGTGGCAGCCAAGCGTTCATAGCGACGTTGCTTTTTGATCCTTCG
ATGTCGGCTCTTCCTATCATTGTGAAGCAGAATTCACCAAGTGTTGGATTGTTCACCCACCAATAGGGAACGT
GAGCTGGGTTTAGACCGTCGTGAGACAGGTTAGTTTTACCCTACCGATGCAGTGTCGCAATAGTAATTCAACC
TAGTACGAGAGGAACCGTTGATTCGCACAATTGGTCATCGCGCTTGGTTGAAAAGCCAGTGGCGCGAAGCTA
CCGTGCGCTGGATTATGACTGAACGCCTCTAAGTCAG
>TCONS_00084046
CTGCTGTATGTGAACTAGTTAAATCGCACATTTAATTCGAATCAAGGAACGGAATGCATTGAACATAGAAGA
AAGGACATGAAAGCAAAATAATTTCTTTTTCCCAAGACGCTACATGTGGAAGGTTGATGGTTAAGATTATTCT
TCTCAAATTGTATCTTTCAGTCTTCAGCGATGTTGCTGAATGCGTAAGTTAGTGTTGCATATCTATGTTTTGTTG
TTCTCTTAGTGTGGCA
>TCONS_00084104
ATCTCTTAACATTAAAAAAGCTTAGAAGTAGAATAAAACATTGTGTATAAACAAATTCTCTCTAGTGTTGTAG
CTTTGGCAGTGATCTTGAATATGGCGAATCAGCAGCAAGCATCGAGTAGGCCATGGATACTCGAAGTCGTAC
CTCTTCTAGTAGTCATACTAATCGCTGCTCATGTGCTCGCTTTGGTGTACTGGATTTATCGGCTAGCAACTGAG
AAACAGCCTCAGAGGAGAAAAAAGCACTAGCAATATGTAGATTAGATTGAATTTTCATTTCGCTTCAGCTTTT

AGGAAAATGCAAGGGAATCATTCTATACGGTTCAGAAGCAAGCTTTACACTCAAGGCTTTGGTGATTGGTCA
GCCCTCTTTGCCTAAGCATACACTGTCCTCTTTTTCTCCATGAACTTGTGTGCAATTATGCTGGAAAAGTTTGG
GATTTGTTACATGTTAATGGATCGCGTTAATCGTTTGCCATTGTTGAGAAACTTCTTCATTCTTCTATTAACGCC
AGCGCT
>TCONS_00084105
TCTTAACATTAAAAAAGCTTAGAAGTAGAATAAAACATTGTGTATAAACAAATTCTCTCTAGTGTTGTAGCTT
TGGCAGTGATCTTGAATATGGCGAATCAGCAGCAAGCATCGAGTAGGCCATGGATACTCGAAGTCGTACCTC
TTCTAGTAGTCATACTAATCGCTGCTCATGTGCTCGCTTTGGTGTACTGGATTTATCGGCTAGCAACTGAGAAA
CAGCCTCAGAGGAGAAAAAAGCACTAGCAATATGTAGATTAGATTGAATTTTCATTTCGCTTCAGCTTTTAGG
AAAATGCAAGTAGGGAATCATTCTATACGGTTCAGAAGCAAGCTTTACACTCAAGGCTTTGGTGATTGGTCA
GCCCTCTTTGCCTAAGCATACACTGTCCTCTTTTTCTCCATGAACTTGTGTGCAATTATGCTGGAAAAGTTTGG
GATTTGTTACATGTTAATGGATCGCGTTAATCGTTTGCCATTGTTGAGAAACTTCTTCATTCTTCTATTAACGCC
AGCGCT
>TCONS_00084106
CCCAAACTTTTCCAGCATAATTGCACACAAGTTCATGGAGAAAAAGAGGACAGTGTATGCTTAGGCAAAGA
GGGCTGACCAATCACCAAAGCCTTGAGTGTAAAGCTTGCTTCTGAACCGTATAGAATGATTCCCTACTTGCAT
TTTCCTAAAAGCTGAAGCGAAATGAAAATTCAATCTAATCTACATATTGCTAGTGCTTTTTTCTCCTCTGAGGC
TGTTTCTCAGTTGCTAGCCGATAAATCCAGTACACCAAAGCGAGCACATGAGCAGCGATTAGTATGACTACT
AGAAGAGGTACGACTTCGAGTATCCATGGCCTACTCGATGCTTGCTGCTGATTCGCCATATTCAAGATCAC
>TCONS_00084107
GTAACAAATCCCAAACTTTTCCAGCATAATTGCACACAAGTTCATGGAGAAAAAGAGGACAGTGTATGCTTA
GGCAAAGAGGGCTGACCAATCACCAAAGCCTTGAGTGTAAAGCTTGCTTCTGAACCGTATAGAATGATTCCC
TTGCATTTTCCTAAAAGCTGAAGCGAAATGAAAATTCAATCTAATCTACATATTGCTAGTGCTTTTTTCTCCTC
TGAGGCTGTTTCTCAGTTGCTAGCCGATAAATCCAGTACACCAAAGCGAGCACATGAGCAGCGATTAGTATG
ACTACTAGAAGAGGTACGACTTCGAGTATCCATGGCCTACTCGATGCTTGCTGCTGATTCGCCATATTCAAGA
TCAC
>TCONS_00084133
TGAAAAATATGGCTTTCATTTCTTTACATACTTTTTTTGAATACATTTATGTTTGTTATTTATTTCTCTGGTATAA
TTTTTAGCGTGCAAGTAAAGAGAACAACGAGGAACCATCTAAGTCTGAAATGTTTATTGCAACTCGTACAAA
ACAAGGGAAGCAAGTTCATGAAGATGCTCAAATTGCAATATCTGAACTTCAGAACCGTCAAAACGCTGGGG
AATCATCGAATGAAGCATTT
>TCONS_00084226
ATACGAAACACTTCACAAAACTTTAAAATCAACATACTATAAAATTAGATCCATACCCTTAAACGATCGGAG
AGGACGGAAGGGGTGATGAGCTTATACTTGGGAGCTTCAGAGAGGAGCTTGTCGTAAGTGGCCTTATCGAAC
AAAACCATGTTGTTCACCTTCTCCTTTTGCTTTCCCTTGCTCCACTTCTTCTTCTTCTGCTTTCCACCACCAGACT
TGGCGGGCTTGGACGACGGAGGAGGAGCTGCCTTCTTCGGAGCCATTGATGGCGGAACTGCGTAAGGGGGTA
GGGGGAGGGGGAG
>TCONS_00084243
GAGAGCTAACATTCTTCATTGTCTCTTGTATAAACAGGTATTTCCCATTTCTTGGTATTCTTTTTAGGATCATCG
CTGAGGCTGAGATCAGTATCATCTTCTTTCATTGTTTTAATAAATGTTTTTGCTCCCATCATAATAAGCCTTTCT
ATCATGAAGAGCTGGTAATTGTTCTCAATCAATGGATCCAAAATGCTGCTGTAATTTGATGAATAGGCCAGCT
TGTACCTCACAGCCAGAGGAGAAAAGAAGCCTGGTGTCCTCTCATTAGAAGCAATAAAAAGGGTTCGCCCAT

GCCGGACCCATTTTGCTATTCTGCAAAGAATGAACTCTGGACGTGTATCCCTGTCCAAATGAGGGTGCAAACT
TCGATCAACCCCAAACCTGTCCTTCCTTGTCTTCAGAACATCACCTCTCCGAACATGCATAGCATCATA
>TCONS_00084244
AATACGGCAGAAGAATGGAGCTGTGATTTTTCCTATCCTTGCATTCTGCAAACCAAGAAAGTGGGCTTGCAGT
TCGATTGATAAGCAAAATGTTTGAGTACAAACTTTTTTCTTTGAGATCAGTCCTGCTAACTCCTTCAACGTGGG
CAACCCCTCTAGAACCTAATTTCATGCTTGTTGCCAGGACATGATGCCACATTTCGGAGTTGTCCAAAATTAC
AGGTACAGTGTCAGAGATGAGATCCACATCATACAAAGAATCCATAGCACACGAACTATCTCCCCACCT
>TCONS_00084288
GGGTTCAGCAATGCATCAGCTAAATTCCAGATTCCTCCAATAGCTTGAGGATACTGTGTTCAGGCGCGCTGAT
TGACGGAAGAATAACACGATTCATGGGTGGTCTAGGTCTTGTTCCAGCTGCCTCAGGCTCTGCAGTTGTTCCTT
CCTGCATAACTGGAATATCTGCCCTTCTTCTCACTGCAGACACTCGCTCCTTCCATATTCTTCCATCATTCAGTA
TTGAGGGTTCTGGTATTGGACATTGAATTGGTCGATCATTGTGTAGAGGCTCAGTTGGGTGTTCAACTGGTTTG
AACTCAACTGCAATTTCAATACCATGAGGTGTGATAGAGGGAGCTCCAGCTATTGTAGCAGCACCGGTTGTTT
CTGTATTT
>TCONS_00084290
GCTAGAAATTCCAGATGGTGCTGTGATTGCAAACAAGGATATCAATGGACCTGAGGATATATAGAGGAGCTA
CTGGTGCTAAATAGATTTAGCGAGTCTGCAGCAAGTACATTTTGTTTGTACTGAATGTAATTTTGCCTCTAAAT
TTAAATAAGTGAACAGTTTTGATTGTTTGCTACAGAAATCTTCATTGTATACATTATGGAACTCGATATAGTTT
GTTTCTTCATATTTCTGTAGCCATATTCTTCTAATGTCCGTACTGTTGG
>TCONS_00084295
CAGAAATATGAAGAAACAAACTATATCGAGTTCCATAATGTATACAATGAAGATTTCTGTAGCAAACAATCA
AAACTGTTCACTTATTTAAATTTAGAGGCAAAATTACATTCAGTACAAACAAAATGTACTTGCTGCAGACTCG
CTAAATCTATTTAGCACCAGTAGCTCCTCTATATATCCTCAGGTCCATTGATATCCTTGTTTGCAATCACAGCA
CCATCTGGAATTTCTAGC
>TCONS_00084532
TTTGTTTCTGGTTCTTTAGGTTGGTAAACTGCTTCTAAACACTCTTTAGCTCTGTGAACCTTTGACTGAAGGTAA
AAGCTCCCATTTTTAGCATTTCGCTCAAACAAGTTGTCATACTTCTGCACAATTTCTTCCCATGATGAATTCTC
AGTGACGCCAAGAATCTGCCTTGCCTCTCCCTCCGTCATGGTTTTACTAGCTCTTTTAATATTCTGCACTGCTTC
TTGAGCAACACCATTTTTCGAGGCATCTGCCATCA
>TCONS_00084567
ACTAGATATAGCATGTAGGAATATAGTTCTTCACACCACTAACAAATTTCTCATTCTTGCTGATGAAACATAC
CAGTTCTTTTGGTTCAACAGATATTTTCCTCGCTCCCTTGCTTAGATCTCTCAATGTTCCCTTGTTTGCCCACTTT
AGTCCACATGCATTGCAGAGTGTCCTTGGTCCATCTGGCCCACGACGCATTGCTGGCGTACAATTTTCACTCA
CACCACAATGGTGACACTTTACAGGAACAGTTTCAGAATGCTGAGTGCCATCTCCTTCAAGGCTACTCTTGGC
TGAGTCACAGCTAGAAGATCAAGAACTTGCTTTCAAAGATGCAAATTGACCATTCTTCCG
>TCONS_00084735
GGAAAGTTAAATTAAGACAGGAATTAGGCAAAAATATCTTCATTACAAAAGAATGGAAGTAGACCATTCTC
ATCATTCACACGCCCCATTCCAGGTAAAGTAATAAAACATATTCGCCATGTCCAGAAATTTGTATCAAAAGT
ACAATACAGAAACAAGGTACAGAGCATGAAAAAGCATTTATGAAGTTGTAACTGCAGTAGCACCAGAAGTC
ACCGATGGCGGTTTGAGAATGATTTCCACATTGTCATGAACCCCTTGCAGGAGGAGCTCGCCATCGTATGTAA
GTATTTTCCTTTTCTTTGCAGCTCTTAATGTCCCAACGAGCGCCTCAAAGATGTTGGCACATCTATCATCATGA
AACAGCACACCGAATGTTACCTTGTAAGAACCATCATCTTTGGCTATGCCGAGTCGCTTGATCTCTTCTTTGAG

TCGTTCAACCTCTTCTTCAACGTTCATGGTTCCTGTCAATGGAGTTGAACAGATCTATCTTATTCTCACATTTTT
CTCATTCTTATATACTCCC
>TCONS_00084932
CGGCATATAATCACACTAGCTTCAAGAATTTTAGGGTTCTTTGACAACCACTCAAAATCCTGCTCCGCAACAG
ACTTCATACCCAAATACGATGTTGTCGCAAGGTAGTAATATGTAGTAGTTGCCAGTGCATTGCTTAGGTATTC
AGAAACGGGTGGCTTATATCCTTCAATAAACCATGTTGACTCGACATTATAATTTCTTACTACTTCTTTCATTCT
TTCAATTGCATGGCAGACAATATGAGATCTTCCAGCACTAGACA
>TCONS_00084940
TGATGTCTGCCACACGTACTATTCCTCTGTGGATAAGATTCTTGCAGTTCGCCTCGACATCCTCGACATCATAA
GGTTCAACACCCCATGCTTCAGTTTCTTCTTCTTTTAATAGTTTGGACACCACAGAACAAGTATCAGGTCCCAC
GTGCAAAACTTTGCGCATGCTGTCACCATATGCCTTCTTTAAGTAAGGAATAGCCAGCTGAAGCTCCGAAGTG
CAGGTAAATTCACCTTCAAGCCTGCTGAGTGCTGAACTTCCAGATGTACCT
>TCONS_00084944
TATAGCAGATATGATTGACTCTGTTTCCTGAATCAATTTATCTAAAGGTAGCACTATGTGCACTTTCAGCAGA
ACAGACAAGTACCTCTTCTTTGTTCTTCTTGCCTCCAGTGAAGAGTTTGTACCCACTATAGAACAGCAAGCCC
CAACCGGACAAAGATACAATCACAAAATGTTCTTCCTTCCATTGGGCAGGATTCATCGGCTCTGACCAGCAA
TTGACCTTAGGAGGCCCGTGATGATCAGCTGCACCAGCAAGGCCACGGCGCTGAACGAGAGAAGTGGCTGA
GGAGGGGAAGCGAGTGAAGGCTGCTGCTCGACGAGCCGCCATTGTCAACGAAGTAGCCATGGCTTTGTTTGT
TCTGAGATCGAAGAGAGAAAGAAAATGAAATTAGCTGGGGATGTAGGC
>TCONS_00084946
TGATAGGTGTCATGTCCTTAGAGCATTCTCTGGGTCTGAAACTACATCTTGGGTATCTGAAGGTTGGTGTCTTC
ACCAGTAATCCCGACACGAGCACAATCCGTCTTTTAAATGATGAAACCTCTTAGCATCTCGCATAATAATTTC
CCGACCAACAAGCCTCGAAGCAATCTTCAATGCATTATGGCAATCACCACAGACACGCAGATTCTTTATTATT
CGGATTGGTGATCGTGCAGAGCTACTCATAAGACCTTGAGCAAAAGCAAGTCTCTCGCTGTGAGCCATTAAA
GCGTCCTCTTTAGTTTCTTGGTCTACGTCATGTAACACAAACTTAATCTCTGGTATATAACCGTCCTCCTTCATT
AGCTGCTTTAAACCCCTAAGCAATTCATATATCTTCTCATGGTCGGGATGTGATCTATCTCCTGCTCGATATTC
ATGGACTCTGCTTCTAGCCTCAAGAAGACTTTGAGCAGATTTTTTCTTTTCTTTGTCCTTAGCAATATCTGATGC
TTTTACAGCTAAAAAACCTGCCTTTGACTGTTCATCCAAACGAGATGGATCTAGGAGTTCAACAATCTCAGCA
CAACGATCCCCAAGCTCCAAGTTCCCATGAATTCTACAGAGATTCATCATGGTTTCCCATACTTCAATACTTG
GCTCTATAGGCATTTTTTCAATGAACTCTATCGCTTCAATAAGATATCCTGTACTTCCCAACATATCTACCACT
CCCACATAATGCTCCATGGAGTGAACAATACCATAATCTTTACTCATTGATTCAAAGTGCAACATGCCTTCAA
CTATATCACCTACAACACTACAAGCATGAAAGACACCCAGATACATTTGGCCATCAGGTTTCATCCCCGTTTT
CTTGAATTCTGCAAATAATTCTATTGCGTCTTCTCCAAGACCATTCTTAGCAAGCCAAGTAATCATCGTGTCCC
AAGAAGTCAGATTACGCTGAGGCATTTTTTGAAACACCGAAAAGGCATCATTCATGGAGCCACATTTGCCAT
ACATTTCAAGAATCTTATTGTACGTTTTGACGTCAAGATGAGGATGAGATCTCACAAGGTGCTCATGAACTGA
TTTAGCCTCCTCAAGGGATTTATCCTCACCACAGACATCCATTAGTGTTATATATCGAGACAAATTGACTGTG
ACATGTTGCTGCTCCAACAAATGCAAAACTTCAACTGCCTCCTTCACTTTGCCTTCTTTACAAAAGTTATCGAG
CTCGTCAATTGAGCCTTTTTTAATGCTGCTATCAGCAGAATCGATTGACTGTTCAACAGGTGTTGAACTTCTCA
TTTCTTGATTACCTACTATTCCATTCTGAGATTGATAAGAAGCTTGATGTCCAGTACTAGAAGGGTTAAAGCCG
CTTACATTCTGCTGATATTGCCCCATCATCTCCCTTGAGTAACCACTTGAATTCGTATGCCTTTCCATATCATAC
GCACTTGCACTTTGTGGATACGTTTCAGAATTTCCTGCTTGAACATTCCTTACATTTCCAGGGATATA
>TCONS_00085140

CTATAGTTGAATATTGAGTTGGAAGCCATTTATAGACATCAAGAACGGACCAACATTCCAGTGCGAAGATGG
ATCATCAAAATATTGTAGCAGATGTACAACAGACTGTATTTCCTCAAATCATCAAAAACACTGGTCGATCCTT
TTCGCTGACTGAAACTTCTCTAGTATAATCAATCTGCCAACACAAGCACTAATCCATCTTGAGACATTTTATCT
AGCAGCCACGTA
>TCONS_00085141
TATCACTTATCAGGAATTACGTGGCTGCTAGATAAAATGTCTCAAGATGGATTAGTGCTTGTGTTGGCAGATT
GATTATACTAGAGAAGTTTCAGTCAGGTGTTATTGCTTATCATGCAATGGGAAATTAGAAACGGCGGTAACA
ACTTATTTTCGTGCTTCTTCAGCGAAAAGGATCGACCAGTGTTTTTGATGATTTGAGGAAATACAGTCTGTTGT
ACATCTGCTACAATATTTTGATGATCCATCTTCGCACTGGAATGTTGGTCCGTTCTTGATGTCTATAAATGGCTT
CCAACTCAATATTCAACTATAGTGTAATAATCTGGTTGTTAGCCAATGCTACTGGGTTTTCATGGATGCTGCAG
A
>TCONS_00085177
ACTGTCTCTTAAAAGTAAGATCACTGACTATATGTATATGTATCAACATTCTTACCAACTTCACCTTTACTGTG
ACTAACTTCTACTTTCTTTTGTCTCCTTTATTAATTAGGAATTGTATTGGCATTTGAACCCACTTAGCTCCAAAG
TTGTGGGGATTTGAACAATCAGAACATAAATAATAACTTTCATTGGTTGTGGTTAGCATCGTGCTTAACGTATT
TGGACCAACTAAAGGTTTTGTGATTTTGTGATTTTGTAGTCAATGGACAAGCTTGGCTTGTGAGCCCTTGAAAG
TTCTAGCTTCTTCGACTGCCACCTCAGTCTTTGTCCATCTCTTTCACCCGCTTCAGTAGTAGATTGTGTGAAGAC
AGAAACCCAGAAAAAGCTCAAGAAAATCTACAGCTGCTACCACCCCAATGGATTTGTTCAAATTTCTTATCA
GAATTTCTGCTTCTACCA
>TCONS_00085320
GTCAAGTCTGTCTGTCCCAGCTAACAGCAATATCTTTGGAACAGGAGATGACAAAAACTTTTCTGAAAGGCCT
TCGTACCATTCTCTCCAATACTGCTCAGTTTCTTCCAGTCTTGCTCTATGCGTGTAACATTTTTTTGATTCATCAT
ATTTCAATGTGTTGGGGATTGATACACGAGCAGAGTCAATATTTCTCAAAGAGCCTGATTTGACACTCCATTC
AATATCGTCTGAGTCCGGGATCTGAATATCATCTTCTCGATCGAAATAATCTGACCAGTCTAAAGGTGCATAT
TTCTG
>TCONS_00085321
AACAGCAATATCTTTGGAACAGGAGATGACAAAAACTTTTCTGAAAGGCCTTCGTACCATTCTCTCCAATACT
GCTCAGTTTCTTCCAGTCTTGCTCTATGCGTGTAACATTTTTTTGATTCATCATATTTCAATGTGTTGGGGATTG
ATACACGAGCAGAGTCAATATTTCTCAAAGAGCCTGATTTGACACTCCATTCAATCGCTTTTTCAGGGGTAGA
GAAATGCTGCATTCTGTTCGACAGAATCTTCTGCATATGAATCAACGATGCCATTGC
>TCONS_00085342
GAGAAAACGAAATTTTCAAAAGATAAACTAATGAGCTGTATCATCATATATGCTTGCAGGCCAAATATAATT
CATGGTAAACATGATTTAACCATAGAGGACTGCTTAGGTAAGAGGGAACCAAGTGCAAAGTCAGATTAGTG
GAGCATTGGGAGGACAACTGTACATACTGACAACAGACATCACCAATAAACAATTGCCTGTAAGGATTACA
GAGCAACTGGAGTTGCTGTATTCCTAATGATATGGAGGCGTTGATTGAACACACAACTCCGACTCATCTTCTT
CATCATCGTCGTCATCTGCATCTACCCAGTTACTAGGATGGTGTTGAGAAGCATGACCTTTCAAATCTGTCATG
CCTTGAGGTTCTTCCAAATGATTTCTGTCCTCAGAAGGGAAGTCATTATGTATATCAGACATATCTCTTCCAGC
AGCTCCAGCTTCAGAAATGTGTATTGCACCAATTCGGTGAATTTCCCTTAGTTCAATATCATTTTCATTTCTAC
CCCCTCCAGGTTCAGCACCATTACCACCTTTTGTTCTGCTTCCTGAAAGATCGGACCAAATCCTGGTCTGATCA
GATGGAAGCCCAGAATTCCCTTTTGATCTCTCCTGGGGTGGGGCCTGTCTTCCATCATTAGCCTCACCGTGGG
AAGTGCCAGCAACTTGTGGATTTTCAGAGGAGCCAGCAGTATTGAGCTGGGACATAAGCATGGTTGCAAGTA
CCAAGGTGGCATCAAAAGTTGAGATCGAGCCATCATCTAAACCAAATTTTGGCGTCATTAGAA

>TCONS_00085424
CAATACACTCAACACTCTGTGCTCACTGGGGGTGTGTACAGACTCTGGAGGGGCTCCCAAAGCCCAAGCGCT
AAGCACAGACAACTCACGTGCCATCATATCAATACCTGGATCCGCACGGTCAACTCACGTGCTACGCGGACA
ACTCACGCGCTATGGTATCAATACCTGGACCCGCACTGTCAACTCACGTGCTACGCGGACAACTCACGCGCT
ATGGTATTAATA
>TCONS_00085425
GGCCTGGTTGTGAGGATATTAATACCATAGCGCGTGAGTTGTCCGCGTAGCACGTGAGTTGACAGTGCGGGTC
CAGGTATTGATACCATAGCGCGTGAGTTGTCCGCGTAGCACGTGAGTTGACCGTGCGGATCCAGGTATTGATA
TGATGGCACGTGAGTTGTCTGTGCTTAGCGCTTGGGCTTTGGGAGCCCCTCCAGAGTCTGTACACACCCCCAG
TGAGCACAGAGTGTTGAGTGTATTTATACCATAGCGCGTGAGTTGTCCGCGTAGCACGTGAGTTGACCGTGCG
GATCCAG
>TCONS_00085435
ACGTGTACATGTCATGGTATACATGATAGCTCCCACTGCACTAGCGTATGGGATCCTACTCATGCGTTCTCTCT
CTTCAGATGTTTTAGGACAATCCTCCCTACTGAGAGTAATTCCAGTGCCTATCGGTAGATAGCCTCTTTTGGAA
TTATCCATGTTATACCTCTTTAAGATAGTATCAATGTACAAAGACTGGGAAAGTCCAAGCAGCTTCCTAGATC
TATCTCTATAGATCTTTATTCCTAATATATAAGCTGC
>TCONS_00085451
GCAAACCAAGAAAGATCAAACGAAGCTTACGCAGGTCTCGGAACACGGAAATAAGGGCTAGTGTTCAAAAC
GACCTATCGGGTCGTCACACTACGACTTACTGCAGTGAGATGCTGTATTGCACCTTTTGCAGTAGGAAAATTA
CTTGTCCACGTCCTGGACGGATTTGGTTGGAGCAAAGAGGTCTCCAACAAGCGCTTTATTGGCACATAGAAG
CTCGCGTTGTACTGGATCTTCAAGAATTCATTAACTCGGTAAGCATATGAAATCTATTTTTGGGACCTGTGACA
TTCTAATCTACGAAGGCTCACATAAA
>TCONS_00085471
CTTGTTTTGTATTTCCTTCTTTTGTGGGTCAATTATTTTCAATCCTTGTCGAGACTAGTGTGGATGGATATACAT
GTGCAGTGCTATGGAATTTGGTGTTTATCACTTGGATCTTAAACTTTCTCTTTATACTTGCAGGATAGCGAAAT
GCATCGTGGGAAGCCATTGTATCTAAATGAGGAACGCTATGCTGCCTTAACTCATATGGTGGCTTCTCATGGA
CTGGATCGTAGTTCAAAGGTGCTTCGTCAGCCGAATATTGGCACTTTTTTCATGCTTTAGTTCGACTGTTTCGTA
AGGTTAACTAGGTCCCAGGAATTAGTCAAAGATGGTAAAAAGTTATCAGCTGTCTGGTTCTGAGTTTTGACTC
AGACACAGCATGTCCAATGTTCTGCGGTCTGCTACTGTCTAATATACAGATTATCGAGGTTAGTCTATAATTTT
GACGT
>TCONS_00085472
CTTGTTTTGTATTTCCTTCTTTTGTGGGTCAATTATTTTCAATCCTTGTCGAGACTAGTGTGGATGGATATACAT
GTGCAGTGCTATGGAATTTGGTGTTTATCACTTGGATCTTAAACTTTCTCTTTATACTTGCAGGATAGCGAAAT
GCATCGTGGGAAGCCATTGTATCTAAATGAGGAACGCTATGCTGCCTTAACTCATATGGTGGCTTCTCATGGA
CTGGATCGTAGTTCAAAGGTGCTTCGTCAGACGAATATTGGCACTTTTTTCATGCTTTAGTTCGACTGTTTCGT
AAGGTCTGGTAAAGGTCGTTTCATGATCGCCATATTCGGTATATTTGTCTTTACTTACTGACAGGTAGTTTATCT
TTATT
>TCONS_00085557
TTGTCAATTTGCTTTGACTGTCGGCTGCAAGAGTATTATTATTTTAGTCGTGATAATGGAGCCTAGTGCAGATA
TTAACCTTGATACAGACATCTGGAGTCTGAATGATCAAGATAGCAATGCAATTATATTGCCAGACAAGAAAA
AGAAGAAGAAGACAGAAAAGGAACAAGTATCGAAAAAGCTTAAAACGAAGAATAACATTAAGTTAAGTCA
ATCTCAGAAAAAAAA

>TCONS_00085558
GAGATTGACTTAACTTAATGTTATTCTTCGTTTTAAGCTTTTTCGATACTTGTTCCTTTTCTGTCTTCTTCTTCTTT
TTCTTGTCTGGCAATATAATTGCATTGCTATCTTGATCATTCAGACTCCAGATGTCTGTATCAAGGTTAATATCT
GCACTAGGCTCCATTATCACGACTAAAATAATAATACTCTTGCAGCCGACAGTCAAAGCAAATTGACAA
>TCONS_00085813
TGTAGCATAGTGTCCAAATCCATGGTAATATTTCCCAGCTTCAAGCCGTGTCTACTCAAGACAATAATAGCCA
TAAGATGCCTAAATACTCATACAACAAGGAGGCCGCCAAGGCCACCGGTAAAAACTAAACCACCACAAAAC
AACAATTACAAAGACAAGAAAGAGACTCTGATGACAGAGGAGAACAGTAACAGAAAGCTCCAAGACAATA
ATTCCATGGTCAAATCAAACTAGGAATCTTGTTTCTTAAAGCTAATAAGTCATTCGTACTTA
>TCONS_00085815
CTTATTAGCTTTAAGAAACAAGATTCCTAGTTTGATTTGACCATGGAATTATTGTCTTGGAGCTTTCTGTTACTG
TTCTCCTCTGTCATCAGAGTCTCTTTCTTGTCTTTGTAATTGTTGTTTTGTGGTGGTTTAGTTTTTACCGGTGGCCT
TGGCGGCCTCCTTGTTGTATGAGTATTTAGGCATCTTATGGCTATTATTGTCTTGAGTAGACACGGCTTGAAGC
TGGGAAATATTACCATGGATTTGGACACTA
>TCONS_00085870
GAAGCATGACTGCTGAGCAAATCTTGATTTTTGTCACTACTCGAGGTTTTATCTACAAATATAATAGACATAG
TTGCAACAATAAAGCTATTCTGTTTAACCAGAAAATAATGAACATTGGGATATACCTCAATTGCTGTAATAAC
AAAACATTGAACGTAACTTCCATTTTCTAATATAACTTGACTGAGCTTTGGAGACTGAACTCTATCATACATG
GACTGTTTCATTTGGGAAACAAGTCAATGAGGTCCAAGCAGGAGAACGAAATGATCTTTGCCTTACCTCCCA
GCATATTGGTAATCTCCCAAGCATTTTGTGACAGCCTCATTGGCCTTCTGAGATTCCCTTTCTCCTCTTTTTTTCT
TCTCCCTTTCCACATATTCCTTCAACAGCTCTTTAGCCTGAACCAGCTTGGAGAGCAAATTGCGATAAACATC
AAGGTCCCTATCCACACTTTTCTTGTTTATATTCAGTGAAGCACAAAGCTGTCATGCCAATTTCACAAGTCAAC
AATATATTCACCTCCAAGTCCGTTGCAGTTTCATGAACATAAACATCTAAAGGGGG
>TCONS_00085964
TTGGACGACAAATTAAGTGAGGCAAAGTATAGGAAAATCAAGTGAGACCATCTGGTGCTGACAAGCAGTGG
ATAAAAGTTTTTTGAGTTAATAAAAATAATAGTTAAAAGAAGTTTTCATGGAAATGTAAGCAAAATCCCACC
TGTCTCTTCGCATGCTTTTGAAGACAGTTCGGGCTTGTTCCACCATTCCTGAGATTGCAAATGCATCTAGCAAG
ATGTTGTAAGATTTCTGTGTTGGCCTGACACCAGCATCAAGCATTTCCTCAAAAACAGCGAGTGCCTCCTCTT
CTCTTCTAGCCTTTCCATATGCACTAATGAGCAGGGCGTAGCTAACGACATCAGGTCGAAGACCAGCTCTTTG
CATCTGATCATAAATGTTCGCGACCTCCTTGTAGTTCGTCTCAAACGACATCAAACTGTTGTAAGTAACCGTA
TTTTGTGGAATTCCTCGCTCGGTCATCAAAGAAAACAGCTGACGAGCTTTCTCATACTTTCCAGACTTCTTATA
CATATAGATCATCATGTGGAACATTTTCTGGTCTG
>TCONS_00085994
TCACAGCCGTTGATCATAATTACAGGTCTAGATGTGTGTGTATATATACACAAACACACACATTTAATCCTGT
ATTACACTAAACCTCTTTGAAAAAGCCTTGCCTCTTTTCTCTTACCAAGATTTAGCTTTTTCTCTGCCAATCCCT
TTTATTGCTGCTTACTAGGCGCTTGTCTAGTCCGTGTGCTTCCTCTTCAGTCGACCATTCTTCAGTAAATGGGCT
GCTTTCATTCTAAGGTTAAAAAGAAGTTACGTGGACACGAAGATCCTGTAGTCCTTGCGTCCCAAACAGCTTT
TAGTGTTAGCGAAGTAGAAGCATTGTTTGAGCTGTTCAAGAGTATCAGTAGTTCGGTAATTGACGATGGGCTA
ATAAGCAAGGTAATTCTGCTAAACTGGTAAATGTTTTCTTGTTTGCTTTTAGTGTAAGGTTGTGATTCCTG
>TCONS_00085995
TCACAGCCGTTGATCATAATTACAGGTCTAGATGTGTGTGTATATATACACAAACACACACATTTAATCCTGT
ATTACACTAAACCTCTTTGAAAAAGCCTTGCCTCTTTTCTCTTACCAAGATTTAGCTTTTTCTCTGCCAATCCCT

TTTATTGTGAAGCTGCTTACTAGGCGCTTGTCTAGTCCGTGTGCTTCCTCTTCAGTCGACCATTCTTCAGTAAAT
GGGCTGCTTTCATTCTAAGGTTAAAAAGAAGTTACGTGGACACGAAGATCCTGTAGTCCTTGCGTCCCAAACA
GCTTTTAGTGTTAGCGAAGTAGAAGCATTGTTTGAGCTGTTCAAGAGTATCAGTAGTTCGGTAATTGACGATG
GGCTAATAAGCAAGGTAATTCTGCTAAACTGGTAAATGTTTTCTTGTTTGCTTTTAGTGTAAGGTTGTGATTCC
TG

>TCONS_00085996

TCACAGCCGTTGATCATAATTACAGGTCTAGATGTGTGTGTATATATACACAAACACACACATTTAATCCTGT
ATTACACTAAACCTCTTTGAAAAAGCCTTGCCTCTTTTCTCTTACCAAGATTTAGCTTTTTCTCTGCCAATCCCT
TTTATTGGTTAAGATGGTTTTGATGATATATTGTAGTGAAGCTGCTTACTAGGCGCTTGTCTAGTCCGTGTGCTT
CCTCTTCAGTCGACCATTCTTCAGTAAATGGGCTGCTTTCATTCTAAGGTTAAAAAGAAGTTACGTGGACACG
AAGATCCTGTAGTCCTTGCGTCCCAAACAGCTTTTAGTGTTAGCGAAGTAGAAGCATTGTTTGAGCTGTTCAA
GAGTATCAGTAGTTCGGTAATTGACGATGGGCTAATAAGCAAGGTAATTCTGCTAAACTGGTAAATGTTTTCT
TGTTTGCTTTTAGTGTAAGGTTGTGATTCCTG

>TCONS_00086022

CAAAGCTAATTGCATAATATGTAATAGGTAATCCGTTGACAGACGTTAAAAGGAGGATATTATACTAACCAA
AGCAGTCAGAGGAAAGTAGTCAAACAGATCTGTGAAAGTCTTCCACACATTGGCATTACCATACTTCCGTAG
GCATTCATCATAAAACCCATAAACCTGAGTAATCTGGCGACTCTCATGATTACCCCTTAGGATTGTAATTCGC
TGGGGATAGCGCACTTTGAGAGCCACCAAAAGCGTTACTGTTTCAACAGAATAGTATCCACGATCCACATAA
TCTCCCATGAATAAATAATTAGTGTCAGGACACTTTCCACCAATTCGAAAAAGCTCAGCAAGATCATGGAAC
TGACCATGAATATCACCACATATAGTCACAGGGCTTTTCACAGGCTGCACGTTGCTCTCTTCCATTAGAATCTC
CTTCGCTTTCTCACATAGTCCTCTTACCTCCTGTTCAGACAAGGGTTTGCACTGCATAAGCTGAGCAATTTGTT
CGTCAAGATTTCCATGAGATGCGCTCGATGGCACCGGATCCATAGCTGATTGATCTTCTTTTCTACTGCTTCTG
GAGGGAAATTTGTGCAGAAACCCTAGCGGAAAAGGGGGATAAAATCAAAC

>TCONS_00086035

GCCCCGATGAGTAGGAGGGCGCGGCGGTCGCTGCAAAACCTTGGGCGTGAGCCTGGGCGGAGCGGCCGTCG
GTGCAGATCTTGGTGGTAGTAGCAAATATTCAAATGAGAACTTTGAAGGCCGAAGAGGGGAAAGGTTCCATG
TGAACGGCACTTGCACATGGGTTAGTCGATCCTAAGGGTCGGGGGAACCCCGACAGATAGCGCGTTTCGCGC
GTACTCCGAAAGGGAATCGGGTTAAAATTCCTGAACCGGGACGTGGCGGTTGACGGCAACGTTAGGAAGTCC
GGAGACGTCAGCGGGGGCCTCGGGAAGAGTTATCTTTTCTGTTTAACAGCCTGCCCACCCTGGAAACGACTC
AGTCGGAG

>TCONS_00086036

CGCTGACGTCTCCGGACTTCCTAACGTTGCCGTCAACCGCCACGTCCCGGTTCAGGAATTTTAACCCGATTCC
CTTTCGGAGTACGCGCGAAACGCGCTATCTGTCGGGGTTCCCCCGACCCTTAGGATCGACTAACCCATGTGCA
AGTGCCGTTCACATGGAACCTTTCCCCTCTTCGGCCTTCAAAGTTCTCATTTGAATATTTGCTACTACCACCAA
GATCTGCACCGACGGCCGCTCCGCCCAGGCTCACGCCCAAGGTTTTGCAGCGACCGCC

>TCONS_00086037

GGACCCCCGTGCCCAGCCCTCAGAGCCAATCCTTTTCCCGAAGTTACGGATCCATTTTGCCGACTTCCCTTGCC
TACATTGTTCCATCGACCAGAGGCTGTTCACCTTGGAGACCTGATGCGGTTATGAGTACGACCGGGCGTGGAC
GGCACTCGGTCCTCCGGATTTTCAAGGGCCGCCGGGGGCGCACCGGACACCACGCGACGTGCGGTGCTCTTC
CAGCCGCTGGACCCTACCTCCGACTGAGTCGTTTCCAGGGTGGGCAGGCTGTTAAACAGAAAAGATAACTCT
TCCCGAGGCCCCCGCGACGTCTCCGGACTTCCTAACGTTGCCGTCAACCGCCACGTCCCGGTTCAGGAATTTT
AACCCGATTCCCTTTCGGAGTACGCGCGAAACGCGCTATCTAGTCGGGGTTCCCCCGACCCTTAGGATCGACT

AACCCATGTGCAAGTGCCGTTCACATGGAACCTTTCCCCTCTTCGGCCTTCAAAGTTCTCATTTGAATATTTGC
TACTACCACCAAGATCTGCACCGACGGCCGCTCCGCCCAGGCTCACGCCCAAGGTTTTGCAGCGACCGCC
>TCONS_00086071
ATACATAAAGGAAAGACGATTAAAGAGGAAAATCCAGTTTCTTTGCCTCCCATTCATCTTTCAAATCCGTAAA
CCCTAATTCTCTTTTGCGACCATGAGCTACTCTAATTACGACTCCAGATATGGCGACTCAGGCTCCTATCGCCA
GCGTCGCAGTGATCTAGTGGGTCCACCTCATATATATTCTCGGCCTATGCCTGGTGGAGCTGCGGCTAGCTAC
GGTCGTGGTGAGCCCCTTCCTTATGGCGGTCCTCAAGCACCGCCTATGGACTCTGGTGCTAGAGGGGGTAGTG
G
>TCONS_00086072
CCACTACCCCCTCTAGCACCAGAGTCCATAGGCGGTGCTTGAGGACCGCCATAAGGAAGGGGCTCACCACG
ACCGTAGCTAGCCGCAGCTCCACCAGGCATAGGCCGAGAATATATATGAGGTGGACCCACTAGATCACTGCG
ACGCTGGCGATAGGAGCCTGAGTCGCCATATCTGGAGTCGTAATTAGAGTAGCTCATGGTCGCAAAAGAGAA
TTAGGGTTTACGGATTTGAAAGATGAATGGGAGGCAAAGAAACTGGATTTTCCTCTTTAATCGTCTTTCCTTTA
TGTAT
>TCONS_00086094
AATGATTACTACAAGTTAGATCAGGAACCAACTGTCTGAGATGAAGAGGAAAGTTTATTCAGTAACCAAACA
AACATAGGAACACAAGAAATCCAACAAGTAAGAACATAAGCTTCAGATTGTTATCTGCCTGTCCTTGGGCAG
GCCGTGCAGCATTATGAACATTCGCCCCACGATATGCAGGAGGATATCCAAATGGGTAATTTGATGTTGCAC
CAAATGCAGTTGGACTGGGAAATCCATGAAACTGAAAACTGAACAACGATGGAAACAACCCGCCAAAACC
AGCAGACATTGTAAAGTTACCAAAACTAGCAGTTGCTCCTGGAAAAAATCCTCCCACGTGACCAAAACCGA
GATTAGGAAAAGCATTTGATACTGGCGGAGGAGCCGTTTCAGGTCTTTGCCCTGCTGGCCTGTTAGGAATTTC
AACCCCAGGAATCGGTTTTGATCTGGGATCAGTTGAAGTCCGCCCTCTTCCATAAAGAGGGACTAACTTCTCC
TCTTGTACAAGGGCCTTACAAACAGGACATTCATGGGATTGTGAATGAAGCCGTAGCCATCTATAGAGACAT
GGCCAACAATAGAGGTGGCCACAAAGTGTCACAATAGGGTCTTGTGCCAATTCAAAGCAGATGTTACATTCA
AAATCACCAGCATCGTTGCTGCTATTTCCTGAGGAAGAAGAACTCTCATATGCCCTGGTAGTTGAGTTCTGCA
TTTCACCCGATATCTCTTATTGGCACACAGATATGCACTTCACCGATTTGGAAAAACGAGAATGGTGATTGCT
TCAAACTGT
>TCONS_00086135
TCAGAGTCTAACATTTACAAAGTCAAAGCATCCATCCACATGACTTTGCTCGTTGGGTCAAGGAACCTATACC
TTTTCCTTCTCTACATTTTCCCCCACCAATATTAACACTTACAGAGACCTCCCACTTAATCAACGAATAACGAA
AACCTTCAATCAACGTCGTCAACTTCATCTTCGAAATGATTCTCCATTTCTGTGCCATTTAGGTTATTATTTACA
AACTTAGACTGTGTCTCCTTCACAGAGCTCTCCATACTATTTACAAAAGCAAGCCGGCTGTCCGCTTTAGGGC
TCTCACTACTATTTACAAAGCCATTAGCTGTTTGACCTGTCATTGGGATACCATCCAAACCAGTTACTTCTTGG
CCACAGTGAACTTCGCTCCTGGGACCATTCATAGAATGACGCGATCCATAGCCACCATTAGCCATAAGATTG
CAGTTTCCAGAGACCTCCAGCCTAGGAAGTGACTGCTGGCTATGGTCACCGTTAACACATGCCCTCCCTTGCT
GGGTCTTGGAAGCTTGAATATAATCAAAAACTGGAATTAGATCTTTTGATCTCGCCTCAACTAAGTGAGTTGG
AATTGCTTCTATCTCAGACTCTCCTCCAAAAGCACAGACATCGCCTACGTTAATCCCATCTGTAACAAATGAT
TGAAAATAGATCTCCGGTTTTGCCCATAATGGAATCTGGGGTTTATGCATTTCAAGCTCCACTTCAGATATATA
CATATGATGTCTTTCATCAGAACTTCTCTTTTCTTCTGTGATTGTGTTCCTAGTCTTGGAATAGAGACCATTTTC
CACCTTTATGCTTTCAGGAAATACTTTACTGCTATCAGAATCTCCAACTTCACCATATATATCAATATTACCAC
CTCGCTCTTTGCGATTTTGTTTCTGGAAAATATTCCACTTCTGGATTGCATCTATGACTAATCTTGTATCAACTT
CAATGCCTGACTCAAATGTTGTAGCCACTGCAGGAATGGTCATAACAGAATCTAGGCCAGAACACATACGTA

AAGCATATTGTATCACACAGCCAGGAGAAGAAAAAACCAGCAAGTGATAATTTGATTTTAGAAAGGCTGCA
TCTGTATATTGGTTATAGTTTTTGTAGTACTGAAACGCTGAAGCAATGGACCCAGATAGAGAGCTAAAGTTTC
CAGTTGCAGCAGCGGCAGCACCACTTAGAGTATTTTTCCAACCATTAGCTCCACTCCTTATTCGGCCAACAGT
AGAAAGTGCCACGGGTGGGCCAGATCCACAAATGCTTTGTTGATTAAGCACTTGAGAATTCATGCTCCTGGG
AGCTGAAGGTTTTGTCATCACCCCAGAGCCATTAGTGCAAGCACTAAAACATGCATCAGCAGTATGAAAATC
AACTGATCCCCCTGAAGGAGATATCGCAAAAAGATGGCTAGTTCCTCTAGAAGAGCTTATCATAATCCACCG
GCTATCACTACTAAAACTTATGTCTTGTATGACCGCGTTAGTCAGTCCACGCTGCAGCGTATAAAGATGGACA
TAAGAAGACCCAGATAGTCCAGGCACTATACGAAATACATTGATATTATGACCTTGAACTGAAGCTGTCACC
AGAAGAGTAGTGCTAGGATCAAAGCACAAAGCTGAAATGGGACTCTTATGTGCCCTAAACTGTGCAACAAG
AGCTTTGCTGACGATGTCCCTGACTATGACCTGCGGACGCACAAAATGAAACA
>TCONS_00086190
CAATTGTTCCTATAAAGTTGCTTAACTCAGACACTCTTTTGCCGGTTGGTCCAACAGCTTGTCCTTTATCAAAT
GTCACTTCCTGTCTTTCCTCAAAGTTTCTTGCATGAATCATCTTGCATTTTGTTTGACCTCGAACTTTCTTCTTAT
CTGATGTCCCACCTGTAGCAGAATCAACGTCCATAGCTTCGTCTCTAACACACTCA
>TCONS_00086229
CCAGGCCGCATTCTCCACTGATTCAGCTGAAGCCGGTGTTAGTTCCCCAATAACTACTACTACTACTACTAAC
ATCTCTATGCTTCCCACAACTTCTACCCTCAACATGGCTTCTCTTAAATCTTGTTACTTCCACATGCCAAGGTTT
ACTTCTGGGCAAGAAGCCATTGAAATGTAGGTGTTAAGAGTTTGTGCAAGGCTAGCTAGGAGGGATTAACTT
AGCTTGGTAAAAGAATCTTTTTTTATTTACTTTTGGTTTCAATTTTCTTTGTCATTCTTGTTTTGAATTAGATTCTC
TT
>TCONS_00086230
CAGGCCGCATTCTCCACTGATTCAGCTGAAGCCGGTGTTAGTTCCCCAATAACTACTACTACTACTACTAACA
TCTCTATGCTTCCCACAACTTCTACCCTCAACATGGCTTCTCTTAAATCTTGTTACTTCCACATGCCAAGCCATT
GAAATGTAGGTGTTAAGAGTTTGTGCAAGGCTAGCTAGGAGGGATTAACTTAGCTTGGTAAAAGAATCTTTTT
TTATTTACTTTTGGTTTCAATTTTCTTTGTCATTCTTGTTTTGAATTAGATTCTCTT
>TCONS_00086231
CAAGCTAAGTTAATCCCTCCTAGCTAGCCTTGCACAAACTCTTAACACCTACATTTCAATGGCTTCTTGCCCAG
AAGTAAACCTTGGCATGTGGAAGTAACAAGATTTAAGAGAAGCCATGTTGAGGGTAGAAGTTGTGGGAAGC
ATAGAGATGTTAGTAGTAGTAGTAGTAGTTATTGGGGAACTAACACCGGCTTCAGCTGAATCAGTGGAGAAT
GCGGCCT
>TCONS_00086238
TGAGCTGTTCGGCCCATCTTATCATAACTCTGCTCACAGTTCATCAGATCACCGTTGAAATATTCCTGAATGAA
GCTGACATGTCCAGACCCAGTATGAAAAAAGTTGAGTTTATCAAATCTGCCGAAGTCCACATATCCCGCGTG
TGATCTAGACTCGACATCTTTGCAAAATCGAACCACCAAGTCAGCTTCCTTCCCCTCATACTCAAATGGATGT
CCAATCCAGCTACTAAAATCGTGAATGTGGCTGGAGTTATCCAGTGCATAGCAACCAGTATTTGGTGCAACA
ACTGAAACAACCCCAATTGCTTGTAACAATACAGCTGCTACTACTGTGATTAGCAAATGATACGCTACATTTT
CAGTAGAGTAGTTTTTTGGGCTCCGCATTTCCTCGAGTACACCGCGG
>TCONS_00086249
CGAAGTTGAACTGCTACATTAATTATATCATCCCAAGTGTCATTGGTTGGACGTAGAGTATCCAAGGAAGACC
AAATCCTCTCTGGATCTCTTTCCTTCTTTACAAAAGTCAGAATTTGCTGAGCAATTGGGCTTAGAACTGGGAAT
ATACCATCAACAAAACCAGATCCATATTTCCATCCTCTACCCCTTAAAGAACCACATCTTTTCCTTGAAACTTT
TTTGTGATCAAAGTGTCTAAATCTGCCGCGTCTATCAATGAAAATGCTATCTTGCTTCCACTTCTCAATGGGAA

CATTATTGGAGTCCTTTGCTTTCCATCTGAATCCACCTTTACTGATTCTTGGTTCAAAAAGGATATGATTTAATG
GACATCTGGAGACCAACATGAATGCAT
>TCONS_00086260
GTGAATTCAACTTTTTGAGAGAGGCTGACGCAATGGATAGAATTCGGCGTTTTCTCTACGAAAATAACAAAA
AGTCCCCAGTTATTGTTCCCCGGGTGCTTCGAGATATTGTCACAAGGAGGGTCCTAATGATGGAATATATTGA
TGGAACCCCGATCCTGAAGCTGGGTGATGAAATGTCAAAGAGAGGCATAAGTCCAGATGGTAAAGTTGCAG
>TCONS_00086310
GAACCAATTGTAGGCATGTGTAAACTCCGAGACATCATTTTCTTTACATCTTCCTGGATTTGGTTTTGAATAAG
CTTCAACTTTGGCCCCAATAGTATCACCATTGCGATCACTTGATAGTCCTGCATACGTTCAGCAATACCTGCAT
GTGTTCCTTGTTCTAACAATATTGTATCAAGAAATAAAGGCTCATCACCATTATGAAAGATGTGATTCGTGCT
CTTATAAAGATCAAAATCCCATTTTTCTCCTCTTTCATGACGGCCACTAGTTATCCAGTCAACAAGAAGCACA
CTTGAGTCTGAGACAACTTTGAACACTTGCCTCTGAGAATATTTTGCTGTGGAGAAACAGGTGACTGGATCTG
GAATTACAGCCAAAAAGGCATTGCTTCC
>TCONS_00086313
GTACTAAGCTTTCACAGTAACATATAGTTTATGCAGGCAGATTAGACACAATAGTATACGAGCATATGTATTT
TGAATAAGTTGATACAACTGACTGAAATAAAGCATCTGCCATGAAGTCATACCTCCAACCAAAAGGTCCAGT
TTTTATCGCCTGCACCACTTAATCTACATATATATGATGGAGGACCATTACCAGTCTTCTCAGGAGCTGGAAG
AATGTTATCTGCAAATGACCAGCTGGATAATGAACCAGTAATATTAAGGACTGCAACCCAGACTTCCTTCAA
GGAACCAAGGGAAAACTCCAAGTAGATCCTGCGGGAACCCCCACTCAAACTCTCTTGTGGCTTGTTAATCGC
CAAATGTGGGAAATGATTGTATTCAACCAAGACATCCGAACTTTGCGCAGGAAACGTCATGCATCTTGAAAA
CAACGAAGATATTGGAAATATCCCCATCCAATCAGCTTGGTGAGACTGTTTGACAGCAGCAAAGGATAATTC
TGTATTAATGTGCAGTGCATTGGCCACTTCAGGGGCATGTTTGAAAACGAAAGGCAGAGAATTGGAGTCAAC
AACAGCAAAATCATAGGTAGTTTCCATAATTTGACTGGCACCTGCACTCCTGATAGTATGCTGGAATATAACT
CTTTTTGGTGCGTCAGTACTGTATGGAAAGAATTGTGATGAAAGGGCCAGAGCGAGAACACTGCTGTGTAGC
AAAAAATGCATTATAGGTGACCTGGCTAACCAGTAGCTCACAACAGGCAAAAGAGGCCCAATGGACCAACT
AGTCACCAGTCCAATTATTGCTGCCACTATAACATCAGGAACAAAATACCCTGCAAAGCAAATGAGCTACCT
ATTTATCATATTGTATAACTAAATGCTCAAATTCAATTGCAATTTCATCAGATATCAAGGTCTTCACAAGTTCA
CATAAAGGAAAGCTGCAACAGTCAG
>TCONS_00086377
ATAAATTTGGATCTTTCTTGTCAACCCAAACATATGTTTCAGCCTGCAATGTCTTGGAACTATCCATTAGGGAA
ACATCAACAGTCGTCCTTTCATACTCAATGTCCTCAAATATGTCCAGGATATTTAATTCAGGAACAGTGATAT
CCAAAAGAACCTTCCCAGTAACTTTCTTGTTTTCTACGGGTAGAATGGCAGGATAAACACGCCCTTTAATGCT
AAACCTGTGGTAGTTGTGGAGAATAGCAGGATTAGAGGGAGGGACGCGTTTGAGGAGTGCATGAACGACGT
CGTCAGCTAATAGGCTGCCATACACGAACACATTGAACACCGACTG
>TCONS_00086384
CATTTTCTCACTTCCTCTCGAACCCTTCTCACTTTCCCACTTTCACTCGTACCAGTAGCTTTTTCCGGCGCCGCC
ACCGTCGACGGCATTATTATTTCAGATCGTTGTTAAAGTTCAGCTTTTGGGTTTCAGCTTCGCATCTCCATTTTC
TTTCGAATGGCGATTGACAAAATCTTCAAAGATGAAGCCACTGAAGAAAAGGGAGAGCGCGCTAGGATGG
>TCONS_00086389
TTGTAATTCATCATCTCCATAATGTATATAGCATATATTTTCACTGTGCTTGTTTCATAATATAATCATAGTTTA
TCGTCTGTGTCTGATCCTTGTGGTTCTATGCCACTATTTACTAATATTACTCTAATATACTGAAAATGTAGATAC
GTATGGACACTCCATCAGGTGTTAAGGATTCCATTCCTGCTTGGATCAAGTTCTCTGTACAGCCTCCTGGTGAA

ATTCCATACAATGGAATATACTATGATCCACCCGAAGAGGAGAAGTATGTATTCCAACATCCACGGCCAAAG
AAACCAAAGTCACTGAGAATATATGAATCTCATATCGGAATGAGTAGTCCGGTGTGCATCCCTCCTCTTTCAT
GTAATTTTATAATTTATGAATCTGATTTTTGTTATTTCATTTCGTGTTGGACCTGTCATGACTACCATATATGTG
AACAT
>TCONS_00086424
TTTCTAACTTTGGGTCTTCTTGAACAGCCACCTCTTTACCTAACTCAGCTTCTTCACCGCCGCCTGCTGCAGCG
GCGCCGCCATCTCCACCACCCTCTTTTTCTCCTTGGTGTCTCCCTCTTCACCTTTTTTGCTTCTTTCTCACCTTC
CTCTGCTTTCTTCTCTTCATTTCCTTCTTTGGGTTTTTCCTCTTCTTTCTTTTCTGGTTCAACTTTTACTATAACTGC
CCTTTTCCCTGTTCTTTTGCTTACATAATCCACCAATTGTGTTGCCTCAAATACCCCTTTTACTGTAACTTGTGA
ATTCTTCAAGTCTGGTTCTGCATTCTCCACACCTTTCATTCTTTGTATACGCCTTTTGATTTCTTGAGCACAAGCT
TCACAATGCATGTGAACTTTCAGAACTACAATAATCACTTGGGGCGGCTCCTCTTTTTCTCTTCGGGTTTAAC
GACTTCTTTTTCCTCCGGTTTCTTTGGCTCTTCGGCCGGCGGTTTTGGGATCGGAGAAAGAAGCTCAACTTGAC
GGTGGCTCTTCTTTTGCACTCTCTCTAAAACCTTCAACGGATCTGCTTTTTCCCCTTTTACAACCACCTTATGTG
TCTTACAATCCGTTATAACATCCTCAACTCCTTCGAAGCCTTTAAGGGATTTACGGACTTTACGAGCACAGCCT
TCACAATGCATGTAAACTCTCAACACTATTTCTTGAGGTGGCGGCGGAGGTGCTGGTGTTTCTTCTTTGGTTTC
CTCTGTTTTCTTTTCTTCCTTTTTTTCATTATCACCTTTTGGAGCCTCCTCTTTCTTCTCTTCCTCTGGTTTTTTCAC
TTCTTCTGGTTTCTTCTTCTCTT
>TCONS_00086448
AGTATCAGTGTCTCGATCGCCTCCTAACCCGAGGTGAACGAGACCGGGAAATGGAACGAGAAAGCGAGTGG
GAACTAGAGTAAGAACGCGACGGTGAGTGTGAGCGTGAGTGAGAGCGAGTCCGCGACCATGAGCGAGACCT
GGAACGTGAACGGTATCTACGAGAACTCCTTTCCCGGATAGGACTCCGGCTAAGTCGGTGCCTTCGAGGGGA
ACGGCTGTAACTTCTACTACGGCTTCGGCGACGATACCGACTTACTCGGCTTGTTTCTGCAAGCTTTTCAAGCC
TCTGTTTCTCTTGTTCTCTTTTCTTGGCCATCTCAACAGCTGTATCTTTCTTGATTTGTTTGTTCAATTGTTTGCTC
ATTATCCGCTTAAGTCGCTCCTGAGGTGTTTCCTTCTTTTCACCCTGCTGTTTAGAGAGGATTGAACTGCTAGTT
GTCTTGTTTAACTTTGATAATGCTGATGATGCACTTGCTGGTGTTTTCAACATTTGTGCACTCTTATCCTTATCA
ACAGATACCCCAGATGCAGGATCAACATGAAGGGCCTCAAGAATACGACCAGCTGATGGCCGGTTTAATGG
ATCAGTTCGAGACGGAGGAGACGGTGGTGGAGAATATCCTTCAAATTTAGGCTCACTCTCATCGTCTGCGCC
ACCAAATTCAGTGATATACTCTATTTCGGAGCTCTCTCCTTGCCTTGGTAAATATCATCAGATTTTCCTCCAC
GTTCATAGCGTCTTGAATGAGGAGGCGAGTATGAGAGAGACCTTGACCTGGACCTTCTGGAACGTGAATAAG
CTTCGTATGTTGGACTCCGTCGAGACTCGCGATAGGGATCATGATGGAGAACTCTGCTCCCGGTAATGCGGGC
TGCTTCTCTCTCCCTCTCTCTTTCTAATTGAGAAGCCTTCCTTCTTTCTTTGCGACTAAGTTTCCTTATTGCAGGA
TCTCCCTTGATTACTTCTCTTTGCCTCCTTTCTTCCTCCTTTGCCTTTTTGTCCATGTAGACAAGCCATCCATACC
TTTTCACTCCATAATCTTTGGCAATTGACTCCATCACTTCGTCGTTGCTATCATCACTGTTAAAATCTTCTTCCT
CATCATCATCCTCATCATCATCA
>TCONS_00086449
AGTATCAGTGTCTCGATCGCCTCCTAACCCGAGGTGAACGAGACCGGGAAATGGAACGAGAAAGCGAGTGG
GAACTAGAGTAAGAACGCGACGGTGAGTGTGAGCGTGAGTGAGAGCGAGTCCGCGACCATGAGCGAGACCT
GGAACGTGAACGGTATCTACGAGAACTCCTTTCCCGGATAGGACTCCGGCTAAGTCGGTGCCTTCGAGGGGA
ACGGCTGTAACTTCTACTACGGCTTCGGCGACGATACCGACTTACTCGGCTTGTTTCTGCAAGCTTTTCAAGCC
TCTGTTTCTCTTGTTCTCTTTTCTTGGCCATCTCAACAGCTGTATCTTTCTTGATTTGTTTGTTCAATTGTTTGCTC
ATTATCCGCTTAAGTCGCTCCTGAGGTGTTTCCTTCTTTTCACCCTGCTGTTTAGAGAGGATTGAACTGCTAGTT
GTCTTGTTTAACTTTGATAATGCTGATGATGCACTTGCTGGTGTTTTCAACATTTGTGCACTCTTATCCTTATCA

ACAGATACCCCAGATGCAGGATCAACATGAAGGGCCTCAAGAATACGACCAGCTGATGGCCTGCAGAAGAA
GTGAGATATTAAACAAGAAATGGTTTGCGTTAACAAGGAAAACTGGACAGCGTCAAGTAAGTTCAATTCAGG
GAAAATGAGCTTCAGATCGGAAGCTTGCTCCCTCTTCCCCTTGACAGTTATCTCACAGGTGTAAATTGATATA
TTCTAAACCACATTAAGTTCTTCAGCTAACAAATGGATGATAAGCTGCAGCAGAGAAAAGACTGTCCAATCT
AGCTTTGCAAGAATTAAAGGTCAAGTGCTCCGACCGGTTTAATGGATCAGTTCGAGACGGAGGAGACGGTGG
TGGAGAATATCCTTCAAATTTAGGCTCACTCTCATCGTCTGCGCCACCAAATTCAGTGATATACTCTATTTTCG
GAGCTCTCTCCTTGCCTTGGTAAATATCATCAGATTTTCCTCCACGTTCATAGCGTCTTGAATGAGGAGGCGAG
TATGAGAGAGACCTTGACCTGGACCTTCTGGAACGTGAATAAGCTTCGTATGTTGGACTCCGTCGAGACTCGC
GATAGGGATCATGATGGAGAACTCTGCTCCCGGTAATGCGGGCTGCTTCTCTCTCCCTCTCTCTTTCTAATTGA
GAAGCCTTCCTTCTTTCTTTGCGACTAAGTTTCCTTATTGCAGGATCTCCCTTGATTACTTCTCTTTGCCTCCTTT
CTTCCTCCTTTGCCTTTTTGTCCATGTAGACAAGCCATCCATACCTTTTCACTCCATAATCTTTGGCAATTGACT
CCATCACTTCGTCGTTGCTATCATCACTGTTAAAATCTTCTTCCTCATCATCATCCTCATCATCATCA
>TCONS_00086506
ATTTAACTTGCTTCCAAATCTTACAATCCTCATTAGTTTGAGCTGACTATGTAGTTATGTTACCGTCTGTTCTTT
GAAAGCTCATTATGCAATAACTTGTTTCAGCGTGCAGAAGGACCAGAAGGACAGATTGTCAGCAAATGAACT
CATGAGGCACCCTTTCGTCACCATGTATGATGACCTGGATATTGACCTTGGACCTTACTTCACATCCGCCGGA
CCTCCACTGGCAACACTTACTGAGCTATAATTGGTATGTTTGCCTAAGTCTTTGTTGATTCATTTTCACAGCCTC
GGTAGTTTAC
>TCONS_00086523
AAAGTTTTTGATATTACCTGAAAGAACTTAGCCCTATGCTTTGCCATAAAAATGGAGGGTTCCAATAGAGCAT
AAAGCTTTTCATAGAAGTTGGGGTATTCAAGACCATGTTGTGTCATGAGAACAAACAAGCTGCTAAGAGCCA
TCACACTGACTACCCCACCAATATCATACGACCGTGTTAAAAAATCACACAACATGAGAGGATTAGACAGAT
ACGGAATAACAACTTGATGAAGGTTCACCAGAACCTCTTTGTAAACATCAATTGGAAGTGGTAATCTAAGGA
ATGATATCCAGGCTTTAGTAAATTTCAGTTTCATCTTTTTTGCAATATTGGCTGGAGATACCTTGATATTAGAG
GATTCATCCTTGCATTGCTTTCCGCTATGTCCTTT
>TCONS_00086537
CTGTGACATAGCAACATTGACCTCGGCACTGACATGGCACTGTTGCTCCTGACAAGATATTGGCAATCTCTTT
ACAAGCGTCCTTGCTCAACCAAAAAGCAGCATCAGCTATTTCAGCTAAATTTACATTCTCCAAGTGACAGCA
CTTCCATCCCTACATACTGACCACGTTACTCATCAAAAAGCAGGTTTTGTCCATCTCTGGATTATCAACTGCTC
GGTTGATTTCTAAGTAGGAGTGCGCAAGATAATCTACGAATGTCC
>TCONS_00086538
TGGCACATGAATGACAGGGATTATGGACATTCGTAGATTATCTTGCGCACTCCTACTTAGAAATCAACCGAGC
AGTTGATAATCCAGAGATGGACAAAACCTGCTTTTTGATGAGTAACGTGGTCAGTATGTAGGGATGGAAGTG
CTGTCACTTGGAGAATGTAAATTTAGCTGAAATAGCTGATGCTGCTTTTTGGTTGAGCAAGGACGCTTGTAAA
GAGATTGCCAATATCTTGTCAGGAGCAACAGTGCCATGTCAGTGCCGAGGTCAATGTTGCTATGTCACAGAG
GTGGGTAAGAACAATGTGGTTTAACTTTTCTCTGCTGGATCCGTATAATAGTACTGCAAACATTTATCTAAATT
ATTCTTGTTTGACTGAAGATGTGTGGCAAGACATCTATCTAACATTGTTTAGATTTTAATTGCCGACATTAATT
TGAATTTCTAAGGCATACAAAATTCAAACATGGAAATGATAATCCATGGTTTTTTTAATTGTTAGTAACTCTTG
TTC
>TCONS_00086540
CCATTTCTGTGCCTGTCCATGCCATTCTCCTCCCCAAAGACGGCTTACTTTGGCCTTTACAGCTGGTGGAAAGT
CATAGGTTAACCACTGCGGGACCTCTGCAATTATTTCAGCTGATATTACTCCAGTTTCTTCACGCAATTCCCTA

ATGGCAGCAGATCTTGGGTCCTCTCCATCTTCAATGCCTCCCTGTGGCATTTGCCATGCTCCAGGAACATTCAA
TCTGGAAGCCACGAAAACCAAATGATCGTGGTTGATGAGACAAATACCAACGTTGGGACGATAACCAGAGG
GGAGGCCCTCCATTGAAG
>TCONS_00086656
ATGACGTCCAGCAAACTTGGTATGTCTCCTAACATAAGGTTCATCTCAATTTGTCTCTGCTGCTCCTTGCACGA
TCGAGCAACAATTTCATTCACCGTATCACTTGAATGGAGTTTCTGGAGAAGGGCAGAGAGGTGCCTAGGTTG
CTGATTCACAACTTGAAGTGGCCAACTATCCGGTCCAGCAGTTGGCTGAGAAACACCATCTACAAATTCTGG
GCTTCTATTAATCGCTTCTATAAAATTCTTGAAGTGATCCCCTTCACCTGGTTGCAGACGGGTTAATTCCACAT
CAACCTGGAAATCAAGCCATGATTTGGCCATGGCCCAGCAAGCAGACTGGAACAAGATAAAATTGATTTCA
>TCONS_00086730
GGAAGAGCAACTTCCTTACCGCCAAATGCCTCCTTTGCTTGTGGATGCTTGCACTTGTCAGGGTGAACAAGCA
AAGATAACTTACGATATTGCTTTTTGACTTCATCAATGGATGAATCAAATGACAGGTTAAGGTAGTCAAAAGG
ATTCAACTTGAAGCATGAAAGGATCCTGTTAACTTCGTTATCGCGTTCAACTTCACTGACCTCAGCAAAGAAT
TGCTTGAGAAGAGCATCTTCCACCGCGGCGGTTTTCGCCGGCGAAGGTGCGGTGGTTGTAGCGCTGTTGTCTC
CCATTGAATCGGACTTTCACGCTCCGACTGGAGATCCTAAGATGTTTTACAGTTTG
>TCONS_00086754
ACATGGTTGAAATCCATCCAGTTTCTTGAAAAAAGCTGAGTCTGGATCCTTTGCAGCTGTTACCTATACATTA
GCTCAAGCAGAAAAAGGAATGTGAACATATGACCACAAACTTTAGAGAAACAACTAAAAATTGAAATTACG
CATAGATATAAACTCACCGAGTTTTGGGTTTGATCAATGTGATAAACTGGGAATCCAAGAAAATACATGCCG
GCAGATGTGACTTTACCTGTTTTTGAACTATCTTCCTGTAATGCAAGGCTCAGTCCACCATTTTCTTCTTGGTCA
AAGTACAATAGCTTGAATTTGCTTTTTTCTCGTGAATAAACTCGGCAGACAAGGCCCCCCTTCGCTTCCTTTTC
TGTTATGTTAACAGAGTAGAAGTGAGCAGATTGTTTTTCTACCTTTTTACACAAAGGCTGCCCAAGTGGAAGT
GGTTGTATAGGTACTGAACCATTTAAGGCTTCCTCTAGAACAGTTGCGGAGACTGTGGTTTTTATTGGTACACC
AAGTTTACTAAAAAGTGCAGCAAACATAGTGTTTACAGTTCCCAAACTTGAAAGATCAAGCTCCAATTCTTG
GCTTTCTGACTCAACAGCCTCAAAACCAGCTGAGTCATATTGACGCCTTTTATCCGGATCAGATAAAATGTTA
TATGAAAAGGTGACCTCCTT
>TCONS_00086777
GCTATTAAATTGGGGAGCACAAATTCTGGTTATTGGATTTGCAAGTGGAGAAGTACCTGTCATCCCAGCGAAC
ATTGCTTTGGTGAAGAACTGGACGATTCACGGACTCTATTGGGGAAGCCATAAGATATATCAACCTAATGTGC
TTGGAGATTCTCTGAAAGAGCTCCTATCCTGGTTGTCTAAGGGTTTGATTACAATCAACATTTCTCATACATTC
AGCCT
>TCONS_00086811
CCTTGCTTTAAAAGTTTGTACTTTTCCGTCTACACCTCTCTTTTTCTTATAGATCCACTTGCATCCAATGGGTTTA
ACCCCATCAGCTGGTTCTACAAGATCCCAGACCTGATTAGAGTACATAGACTCCATCTCTGATTTCATAGCAG
CAACCCACTTATCGGCATTCTTATCATGTAGTGCTTGGTCGTAATTGACAGGTTCGGAGGTAGGCTCCTCAGG
GATCCTATCATATGATTCTCCCAAGAGCGTATAACGAACTGGCTGTCTTATTTCTCTCCCACTACGACTACGCA
CTACATCAGTTGCAACTACATCACTTTGATTTTGTGGTTGAACCACAACATCATCAGGAACCTGAGTTTCCAT
GCTATCCACAGGGATATCAACGACTTCTTCCTGTTGTGCAGTCTGCTCAACATGACTCCCACTACTTTGTGGTA
GTATGACTTCGGGCACTTGTTCTTGTGGGACATTAAGTCTATTGACATTTCTCCCACTACTAAAGTGCAATGGT
ATGTCAAAATCGACTTC
>TCONS_00086870

CTCGGCTCTTCAAAGTTTCTCTGTTCTGTACTTTTCTTCCTTGCTGAAGAATCACTCTTTGAGACTTGCCTCTTAG
TGAGATGTATATTGGAAAAAGCAAGCTCGTGCAAGTTTTGATCCTTCACTATGGTGCTTGTAGTTGGTTCATAT
ATAATAGGTAGGTCTTGAATATGAAAATATTAAAAATCAAAAGAGTTCTCCAGAATTGTCTGATGTGCATGA
GGCACTTTGCGCTGTGATGGCTGAGCTTGCAGGGATCCCCACTAGCGATGCTACAGGAACAACTCGTAGAGT
CCAGGGTTCGGATCCCTTCTTGAGAAAGTTAAAAAACTCTGCATGTACTGTGCAGAATGGCGGATAGGCCGT
CCGAGGACATAGCCAATAAATCAGCTTTATGGTCTATTACTGAATGGAATATTCACTACACCCTGCATGCAAA
TTTGTTTAACTTTTGTGAGTGTAAACTTGAACTCCGTAATGCACGACATCTTCCTGACTCTGATCCCACTCAGTT
GAAAAGGATATCTTGTCCAGTAATGGGCTAGAAGCCGATTTATTTACTCGA
>TCONS_00086873
GAGTATTTTGGTCCCTTTTAAGCAAATCAGAGAAATTTTATCTAGAGTCCTCAGCAACATGACACAAGAGACA
CACAAGACCTGTTGCACCAGCAACCTGCTTCCAATTTCGGGTGCCTGCACCAAAAGCAGCCCCTGCTACTAC
ACCAGCAGTCAAAACATTAACCCAATCATTTCGTCTTCTATATCTCTGAAGCCCACAGCGAGTGGAAGAAAA
TATTGCAGCAAAGAGTCCTATAAAAGAAAGATGGATGCTTATCAGATGATAATGACATATCTAAAC
>TCONS_00086968
GAGGAGGGAGTGAGAGCATAGCACAAACCAACTGAACGAATGAATTAGAGAAGAGAAGACAACGTCCGTT
CCTCCAAGCAAAGGAGTCATATCAAATTTAGAGTTCTTCTCGAAACATCTATTCACAGACTTCGTAAGAATAA
GTATTATCTGACCATAGAGGAGCTCTAGTTGTACCCTCAATGACTGATATGACTCTTCTGTACAGCTTATGCAA
ACCAAGTAAATAGGTCCTTTCACAAGAAATACAACCTGGTGTTTCCCGGCTCTGACCAAGTTGACTCGATCAC
CCCTGAAAAAGACACCAATCAGGCAAACTTCAATATCCCGCTATGACTTCGCCATTCTCCACAAATGAAATG
ATGGCCTGCAAAGTGGCAGAGAATCCAGCAAGCTTGTATTCATCTCCATATCTTCAAGTGAAATAAAAAAGA
AAAATAAGAAATCCCAGAGAAAATTATATATGATGAAGAAGAAGTAGTTCAATAGTATATAGTACTCTAATT
AAT
>TCONS_00086969
GAGGAGGGAGTGAGAGCATAGCACAAACCAACTGAACGAATGAATTAGAGAAGAGAAGACAACGTCCGTT
CCTCCAAGCAAAGGAGTCATATCAAATTTAGAGTTCTTCTCGAAACATCTATTCACAGACTTCGTAAGAATAA
GTATTATCTGACCATAGAGGAGCTCTAGTTGTACCCTCAATGACTGATATGACTCTTCTGTACAGCTTATGCAA
ACCAAGTAAATAGGTCCTTTCACAAGAAATACAACCTGGTGTTTCCCGGCTCTGACCAAGTTGACTCGATCAC
CCCCATTCTCCACAAATGAAATGATGGCCTGCAAAGTGGCAGAGAATCCAGCAAGCTTGTATTCATCTCCAT
ATCTTCAAGTGAAATAAAAAAGAAAAATAAGAAATCCCAGAGAAAATTATATATGATGAAGAAGAAGTAGT
TCAATAGTATATAGTACTCTAATTAAT
>TCONS_00087044
CCTGTGTGTCAATGCCCGGAAAAGAAGTTTCATACTAACGGACCTAGAATTATTGACTGTTGAAGGAGGTGT
AAAGCAGAGCCACTGAATTACTAAAGCTTTCTGAAGGCTCTGTAATCGATGCTGCTCTGCAACATCTGTTTCA
TTATCATATTTCCCAACTCTGATTTCTGGAGACCTTGAAAGAACCCTTTCGATGATCTCCTCAAAACTGCCCTT
TGAATCATCTTCTGGAGCAAATGGCAAGTATTCAATAGCAGAAAGGAATATTTTATACCTGACGTGAACGCT
G
>TCONS_00087103
TTCTGTCTGAATATCGTATGCTGGTAATGTCCTTTCATCTTTAGCCAAAATTTGGTTTGCTTCCTATTGCATAAC
GGGGTATATTATTGACCTGAATTCTCTCTCTTTTTTTCTTTTTTCTTTTAAACATTTGCAGCACGATCTGTCCCTG
GTTGTTACACGCTTGCTGTGTCTGAGGCACTTCCGGAAGATTTGAAGAATCTATGTCAAGATGAACGTATCCC
ATATAATCCTCCAAAACGCATCTGAATGGACAAATCAGCAGT
>TCONS_00087105

ACTGCTGATTTGTCCATTCAGATGCGTTTTGGAGGATTATATGGGATACGTTCATCTTGACATAGATTCTTCAA
ATCTTCCGGAAGTGCCTCAGACACAGCAAGCGTGTAACAACCAGGGACAGATCGTGCTGCAAATGTTTAAAA
GAAAAAAGAAAAAAAGAGAGAGAATTCAGGTCAATAATATACCCCGTTATGCAATAGGAAGCAAACCAAA
>TCONS_00087133
CAAGTCTTCAACTCTGAAGGGTTCAACGGAAGAAAAAGGCAAGACGAGGTAAGAGAGGTTGAGACGAGAG
TGATAGAGGTGAGCGAATAGCGCAGCAAAAGCTCCGGAAAATGAAGGATAATTGTAGAGGATGAGGTTTGG
CATTTTGTCTTCCGACGCTTTAGCCAACGCTTTCAATGCTGCTTGTGACCTGAAGCACCGAATTAACGGCGAC
>TCONS_00087175
TGTTAGCGTATAGATTGTTTTAGTTGCGATGAGTTCAGGAAATGTGGGGCGGCTGCCATTGATGAATGTTGTTA
AATTTAAAGGAGTGCCAATTCTGCAGCAATTGCATTTGGAGGAACGGTTGCTCAGGACTTCACCTCAAAACT
GGTGCATTGTAAATGATGGAACCAATGAACCCACAATTGTCATGGGTATTTCAGGAAAACCAGCTGAACTTT
TTGAAATCGGTTCTGTTTTGCAAGACAAGATTCCAGTAGTAAAG
>TCONS_00087177
CCTCCAGTAAACCTCTTTACTACTGGAATCTTGTCCTGAAATACCCATGACAATTGTGGGTTCATTGGTTCCAT
CATTTACAATGCACCAGTTTTGAGGTGAAGTCCTGAGCAACCGTTCCTCCAAATGCAATTGCTGCAGAATTGG
CACTCCTTTAAATTTAACAACATTCATCAATGGCAGCCGCCCCACATTTCCTGAACTCATCGCAACTAAAACA
ATCTATACGCTAACAGCTGAA
>TCONS_00087178
TGCCTCCTCCAGTAAACCTCTTTACTACTGGAATCTTGTCTTGCAAAACAGAACCGATTTCAAAAAGTTCAGC
TGGTTTTCCTGAAATACCCATGACAATTGTGGGTTCATTGGTTCCATCATTTACAATGCACCAGTTTTGAGGTG
AAGTCCTGAGCAACCGTTCCTCCAAATGCAATTGCTGCAGAATTGGCACTCCTTTAAATTTAACAACATTCAT
CAATGGCAGCCGCCCCACATTTCCTGAACTCATCGCAACTAAAACAATCTATACGCTAACAGCTGAA
>TCONS_00087702
GTACGACTAGCCAAACTCTTAGATGACATCATAATTCAGCCTCAACTAGATATTACTATTGGACATTGAAACT
GTTGAGTCCATAAACAAGAGTTTATTAGAAAGACACAAAACAAGTGATAAATCTTGGACGCACCTCTGCATG
GTCCGCGCCTGTACCTAGACCCAATTGGCCAAACTGATTTCCCCCAAAAGTATACACACGGCCATGATCACA
TAAGCACACAGAATGCCATAGTCCACCTGCCACGGCTTCTATTCTAGTGTTCAATAGAGAAGAGACACAAGT
TGGCCTCAGCACGTCGTTTGTATTACCTAGTCCACACTGCCCATACAATCCCCAGCCAAATGTAAGCAGCACC
CCAGCATCTGTGATTACTGCACTATGCCGGCCACCACAAGCAATTCTCT
